# Pharmacokinetic and metabolomic studies with a BIO 300 Oral Powder formulation in nonhuman primates

**DOI:** 10.1038/s41598-022-17807-7

**Published:** 2022-08-05

**Authors:** Yaoxiang Li, Michael Girgis, Meth Jayatilake, Artur A. Serebrenik, Amrita K. Cheema, Michael D. Kaytor, Vijay K. Singh

**Affiliations:** 1 Department of Oncology, Lombardi Comprehensive Cancer Center, Georgetown University Medical Center, Washington, DC USA; 2 Humanetics Corporation, Minneapolis, MN 55435 USA; 3 Department of Biochemistry, Molecular and Cellular Biology, Georgetown University Medical Center, Washington, DC USA; 4 Division of Radioprotectants, Department of Pharmacology and Molecular Therapeutics, F. Edward Hébert School of Medicine “America’s Medical School”, Uniformed Services University of the Health Sciences, 4301 Jones Bridge Road, Bethesda, MD 20814 USA; 5 Armed Forces Radiobiology Research Institute, Uniformed Services University of the Health Sciences, Bethesda, MD USA

**Keywords:** Drug delivery, Metabolomics, Lipidomics

## Abstract

BIO 300, a pharmaceutical formulation of genistein, is being developed as a radiation countermeasure to treat hematopoietic acute radiation syndrome (H-ARS) and the delayed effects of acute radiation exposure (DEARE). Several studies have affirmed its safety and efficacy in alleviating the damaging effects of ionizing radiation. However, dose optimization of any drug has always been an important area of research because unnecessarily high drug doses may result in serious complications. In this study, we assessed the pharmacokinetics (PK) and metabolic profiles of two different doses of a novel solid-dosage formulation of BIO 300 (BIO 300 Oral Powder; 100 mg/kg and 200 mg/kg), when administered orally to nonhuman primates (NHPs). While the T_max_ values of both doses remained the same, the area under the curve at 48 h (AUC_0-48_) was tripled by doubling the dose. Additionally, we monitored serum samples for global metabolomic/lipidomic changes using high resolution mass spectrometry followed by functional pathway analysis prior to and at various time points up to 48 h post drug administration. Interestingly, the metabolomic profiles of sera from NHPs that received the lower dose demonstrated a transient perturbation in numerous metabolites between the 4 and 12 h time points. Eventually, the metabolite abundance reverted to near-normal by 48 h. These study results are consistent with our previous studies focused on the PK and metabolomic analysis for parenteral and oral aqueous nanosuspension formulations of BIO 300. This study affirms that administration of a single dose of up to 200 mg/kg of BIO 300 Oral Powder is safe in NHPs and conferred no metabolomic-mediated safety features.

## Introduction

Nuclear accidents and threats can impose a catastrophic impact worldwide^1,2^. In the past few years, numerous US agencies have received nuclear threats that could potentially jeopardize national security and public health. Acute radiation syndrome (ARS) is a pathophysiological condition that is triggered upon exposure to ionizing radiation^3–5^. ARS encompasses 3 sub-syndromes: hematopoietic (H-ARS), gastrointestinal (GI-ARS), and central nervous system (CNS-ARS). CNS-ARS is the major cause of death within days following exposure to high doses of ionizing radiation^6–10^. Total- or partial-body radiation exposures of ≥ 2 Gray (Gy) are associated with H-ARS and GI-ARS^11^. To date, the United States Food and Drug Administration (US FDA) has approved Neupogen (granulocyte-colony stimulating factor, G-CSF), Neulasta (PEGylated G-CSF), Leukine (granulocyte–macrophage colony-stimulating factor, GM-CSF), and Nplate (romiplostim) for the treatment of H-ARS^12–23^. However, these drugs have safety and therapeutic concerns and require parenteral administration shortly after radiation exposure (24 to 48 h), which is not optimal for administration in austere environments. These agents function as cellular growth factors, and their mechanisms of action only support treatment and not prophylaxis for H-ARS.

Genistein (5,7-dihydroxy-3-(4-hydroxyphenyl)chromen-4-one), one of the most abundant isoflavones, was thoroughly examined for its ability to mitigate radiation-induced cellular damage^24^ and although its mechanism of action as a radioprotector is not fully understood, it has selective agonistic activity for estrogen receptor beta (ERβ)^25,26^. ERβ operates as a negative feedback mechanism by stimulating cell cycle checkpoints and suppressing cell growth^27–30^. Stimulation of ERβ by genistein has been shown to occur at nanomolar concentrations with an IC_50_ of 8.4 nM. The cell proliferation rate is directly linked to the radiosensitivity. Hence, genistein administration reduces the cell growth rate and enhances radio-resistance. Genistein protects against radiation-induced acute myeloid injury when administered prior to irradiation^31^. Furthermore, genistein also arrests hematopoietic stem cells at the G2/M phase and reduces the harmful effects of irradiation^32–34^. Genistein’s antioxidant properties also justify its ability to directly scavenge reactive oxygen species (ROS) that are implicated in the formation of cellular oxidative damage including DNA double strand breaks^35,36^.

As a pharmaceutical formulation of genistein, BIO 300 is a potential radiation countermeasure being developed by Humanetics Corporation and is currently under investigation for the prevention of ARS and the treatment of the delayed effects of acute radiation exposure (DEARE). This agent has also been investigated as a mitigator of lethal radiation-induced pneumonitis/fibrosis in a well-established murine model of whole thorax lung injury. BIO 300 significantly improved survival compared to untreated animals when dosing was initiated 24 h post-exposure (11 Gy, LD_50/180_ or 12.5 Gy, LD_90/180_) and continued once a day for 4–6 weeks^34^. A single dose of BIO 300 (200 mg/kg) administered subcutaneously (*sc*) or intramuscularly (*im*) 24 h prior to total-body irradiation (TBI) significantly improved survival in mice^24^. When administered by *im* injection 24 h prior to lethal TBI, BIO 300 has been shown to attenuate radiation-induced proinflammatory markers (IL-1β, IL-6, and COX-2) in mouse bone marrow and spleen^37^. This suggests that mitigation of the radiation-induced inflammatory response may be a component of the drug’s mechanism of action. In addition, we have reported proteomic alterations suggesting that BIO 300 may act through an anti-inflammatory pathway by hampering the complement system or by thrombospondin 1. Additional findings demonstrated that BIO 300 promotes actin elevation^10^.

Currently, BIO 300 is being investigated in nonhuman primates (NHPs) for its efficacy against radiation-induced H-ARS and DEARE. This agent is being developed for regulatory approval following the US FDA Animal Rule. Multiple aqueous formulations of BIO 300 have been developed for oral and parenteral routes of administration. BIO 300 Injectable Suspension (BIO 300 IS) is administered through parenteral route, while BIO 300 Oral Suspension (BIO 300 OS) is administered orally (*po*). These formulations only differ in their non-active excipients which help facilitate the different routes of administration. BIO 300 OS oral bioavailability has been evaluated in mice^38^ and both BIO 300 OS and BIO 300 IS bioavailability have been evaluated in NHPs^33^. Genistein is extensively metabolized by UDP-glucuronosyltransferases (UGT) and sulfotransferases (SULT) in the gut and liver to produce genistein glucuronide and genistein sulfate metabolites^39^. Given the high presence of these metabolic enzymes in the gut, orally delivered genistein is significantly impacted by first-pass metabolism. The influence of first-pass metabolism was observed in NHPs administered BIO 300 OS by oral gavage and BIO 300 IS by *im* where the relative bioavailability of BIO 300 OS was 2.7% compared to BIO 300 IS^33^. These findings were corroborated in mice where the relative bioavailability of BIO 300 OS given orally was 7% and 9% of BIO 300 IS given *im* or *sc*, respectively^38^.

**Figure 1:**
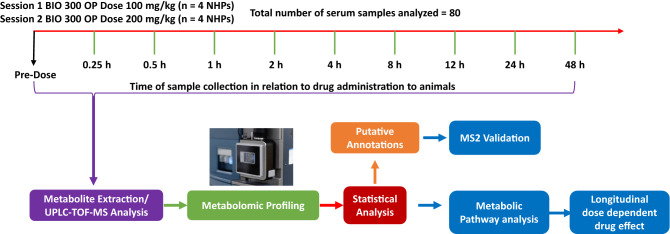
Overall study design schema for metabolic and PK examination following BIO 300 Oral Powder administration to NHPs.

A novel formulation of BIO 300 was recently developed which is more amenable for use in austere environments to protect against radiation injury. This new formulation is an amorphous solid dispersion of synthetic genistein called BIO 300 Oral Powder (BIO 300 OP), which is produced by hot-melt extrusion. BIO 300 OP is a solid dosage, free-flowing dry powder, which is in contrast to previous BIO 300 formulations which are aqueous nanosuspensions. Our initial study using BIO 300 OP formulation has demonstrated that it has efficacy in a murine H-ARS model equivalent to that of BIO 300 OS^24,40^. The purpose of this study was to determine the pharmacokinetics (PK) of a single dose of BIO 300 OP and the associated metabolic changes in an NHP model. Two oral doses of BIO 300 OP, 100 mg/kg and 200 mg/kg, were used and blood was drawn over the course of a 48-h period for PK and metabolomics. PK parameters were examined for the most pharmacologically active form of the drug (genistein-aglycone). Safety was assessed by clinical observations during each blood draw. Serum samples were analysed using global metabolomic and lipidomic profiling (Fig. 1). Our results demonstrate modest changes in metabolomic and lipidomic profiles that were transient in nature and reverted to near normal levels over a 12 h period. These findings support the safety of the drug for medical countermeasure use in the field as a radioprotectant or in the clinic as a treatment.

## Results

In this study, we examined the PK parameters of the BIO 300 OP formulation and changes in the metabolomic profile as part of its safety and toxicity assessment in sera of an NHP model. Global metabolomics approaches were utilized with an ultra-performance liquid chromatography quadrupole time-of-flight mass spectrophotometry (UPLC QTOF-MS) platform. We validated the most significant 100 features that showed transient alterations using tandem mass spectrometry and performed pathway analysis on all detected features.

### BIO 300 OP possessed dose-dependent PK parameters and no treatment-related toxicities were observed

This is the first PK study of BIO 300 OP in a large animal model. A single dose of BIO 300 OP (100 or 200 mg/kg) was administered by oral gavage to NHPs (n = 4/dose). First, animals received 100 mg/kg and then after a washout period of 5 days, the same animals were administered 200 mg/kg. The starting dose of 100 mg/kg was chosen because previous work in NHPs with a related oral genistein formulation, BIO 300 OS, was used to evaluate the PK of a single 100 mg/kg dose^33^. The second dose, 200 mg/kg was chosen in order to investigate a dose–response relationship for PK and metabolic biomarkers. No mortalities occurred while dosing and three animals had minor clinical observations during dosing. This included one female which had a slight decrease in appetite 48 h after the 100 mg/kg dose and a single incidence of liquid feces 4 h after the 200 mg/kg dose. The other female animal also had a slight decrease in appetite 48 h after the 100 mg/kg dose. Neither female animal had decreased appetite during 200 mg/kg dosing. One male animal had a slight decrease in appetite 24 h after the 200 mg/kg dose. None of these observations were directly attributed to BIO 300 OP. There were also no treatment-related effects on body weight at any dose of BIO 300 OP. A single oral gavage administration of BIO 300 OP at a dose of 100 mg/kg or 200 mg/kg was considered well-tolerated.

**Figure 2:**
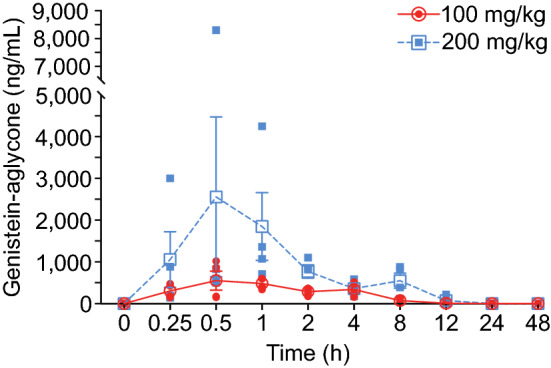
Pharmacokinetic analysis of BIO 300 Oral Powder formulation for two doses (100 mg/kg and 200 mg/kg). Open Symbols are the average of N = 4 NHPs. Closed symbols are the individual NHP genistein-aglycone concentrations for the color-matched dosing groups. Error bars are standard error, and they are not shown when the error bars are smaller than the symbol.

PK parameter evaluation revealed a dose-dependent increase in drug serum level (C_max_ and AUC) by doubling the dose (Table 1). The entire drug serum profile is represented in dose response curves versus the time domain (Fig. 2). Error bars in the 200 mg/kg group between 0.25 and 1 h are largely due to a single male animal which exhibited genistein-aglycone concentrations > 4× higher than the average of the other 3 animals. Additionally, no appreciable sex differences were observed between males and females in the 100 mg/kg group; however, males exhibited a higher C_max_ at 200 mg/kg compared to females, while females had a higher AUC at 200 mg/kg compared to males (Table 1). This is likely due to natural variation between animals that is amplified due to the low number of animals per group (n = 2/sex). We have previously made similar observations with the PK of BIO 300 OS and BIO 300 IS in NHPs^33^. The relative oral bioavailability of 100 mg/kg and 200 mg/kg BIO 300 OP compared to BIO 300 IS administered to NHPs *im* is 3.5% and 10.6%, respectively. This represents a slight improvement compared to 100 mg/kg BIO 300 OS, which had a relative bioavailability of 2.7% compared to BIO 300 IS^33^**.**

**Table 1:** Summary of BIO 300 Oral Powder single dose PK parameters in NHPs.

Dose	Animals	T_max_ (h)	C_max_ (ng/ml)	AUC_0-48_ (ng h/ml)	AUC_0-inf_ (ng h/ml)	T_1/2_ (h)
100 mg/kg	All	1.0 ± 0.71	662.8 ± 329.1	2481 ± 822.2	2505 ± 832.2	1.69 ± 0.17
	Males	0.75 ± 0.35	633.5 ± 326.0	2201 ± 1304	2214 ± 1315	1.56 ± 0.01
	Females	1.25 ± 1.1	692.0 ± 463.9	2761 ± 120.9	2796 ± 94.2	1.83 ± 0.11
200 mg/kg	All	1.13 ± 0.63	2867 ± 3632	7645 ± 3937	7649 ± 3938	1.78 ± 0.27
	Males	0.75 ± 0.35	4504 ± 5369	7026 ± 6672	7030 ± 6673	1.73 ± 0.46
	Females	1.5 ± 0.71	1230 ± 183.8	8268 ± 676.7	8268 ± 678.2	1.84 ± 0.01

Results mean ± SD.

### Administration of BIO 300 leads to transient changes in serum metabolic profiles

We used high resolution mass spectrometry to analyze longitudinally collected serum samples pre and post BIO 300 OP administration. Data deconvolution resulted in the detection of approximately 5000 features. We compared the relative metabolite abundance using statistical analysis to determine temporal patterns over time by comparing baseline samples to post-treatment samples and found modest changes (Supplementary Table 1). Although the higher dose (200 mg/kg) did not show any significant changes at any time point throughout the study, the lower dose (100 mg/kg) exhibited temporal changes at the 4 and 8 h time points which reverted to normal by 12 h.

**Figure 3:**
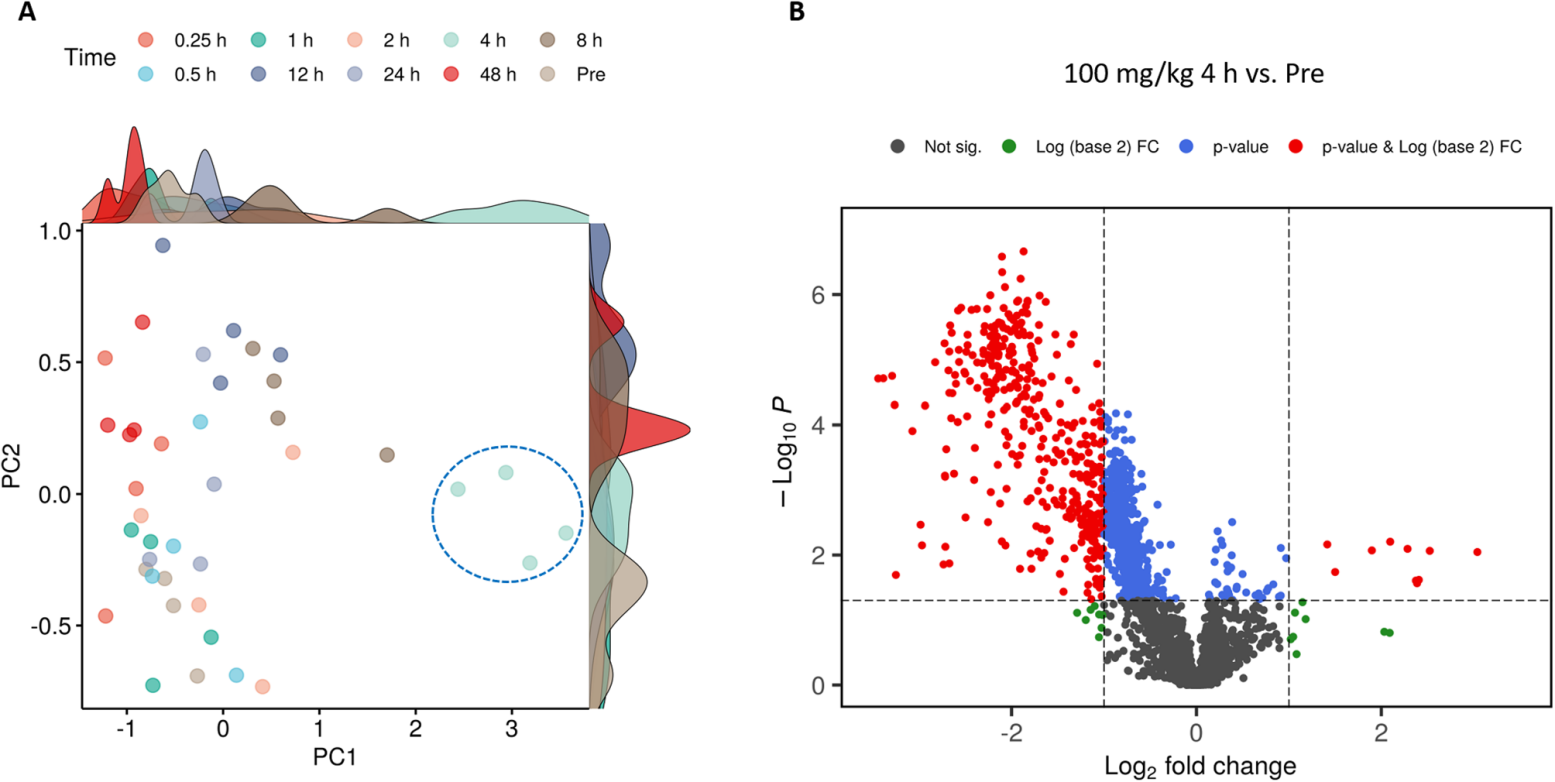
A 2D-PCA plot showing time dependent group separation for the lower dose of BIO 300 Oral Powder (100 mg/kg) (**A**). Volcano plot showing dysregulated metabolites 4 h after BIO 300 Oral Powder administration (**B**).

A principal component analysis (Fig. 3A) revealed minimal metabolic changes across all time points except the 4 h time point. Furthermore, a volcano plot was used to visualize dysregulation of numerous features at the lower dose (Fig. 3B). Statistical analysis performed on all detected features comparing serum level at each time point to the pre-administration time point yielded some significant but transient changes in the metabolomic/lipidomic profile in NHP samples.

**Figure 4:**
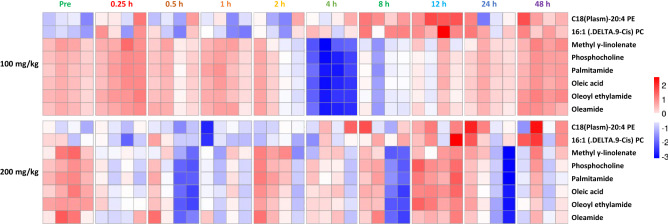
Hierarchical clustering based heatmap visualization of changes in serum abundance of annotated metabolites and lipids over time.

**Figure 5:**
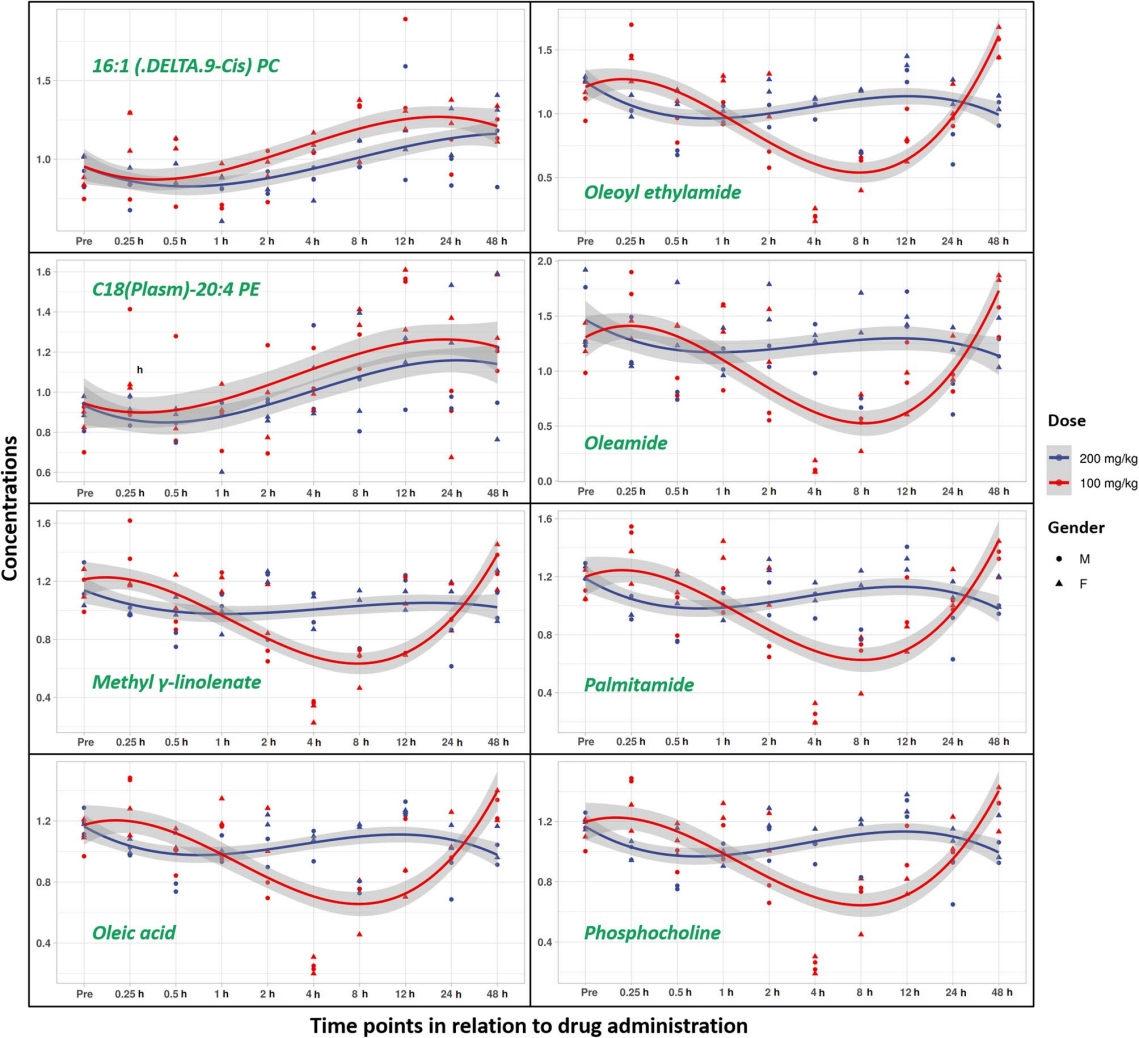
A trend line of the performance of a subset of metabolites/lipids over the study time course showing transient changes that stabilize over time.

The significantly dysregulated features at 4 and 8 h post BIO 300 OP treatment were putatively annotated using a database search and subsequently validated by tandem mass spectrometry and matching MS/MS spectra against NIST spectral database (Supplementary Table 2). These included phosphocholines, fatty acids, and fatty acid amides that were downregulated in the 4–8 h period (Supplementary Table 3, Fig. 4). Furthermore, linear mixed-effect models and longitudinal trend line diagrams of each annotated metabolite/lipid throughout the duration of the study were constructed for better visualization of the gender and time dependent effects (Table 2, Fig. 5). While C18 (Plasm)-20:4 PE and 16:1 (DELTA 9-CIS) PC were slightly upregulated following either dose 4–12 h post drug administration, a total of 6 metabolites followed a unique downregulation pattern during a similar time period post drug administration. Taken together, longitudinal analysis of serum metabolomic profiles revealed a transient downregulation in numerous metabolites/lipids at 4–12 h post drug administration in the serum samples from the NHPs administered the lower dose (100 mg/kg) compared to the higher dose (200 mg/kg).

**Table 2:** Linear mixed effects model showing the abundance of annotated metabolites as the response variable while gender, dose, and time remain the independent variables.

	Linear mixed effect model from time 1 to 48 h				
		Estimate	Std. error	t. value	p. value
C18(Plasm)-20:4 PE	(Intercept)	1.1003	0.0812	13.5518	–
	Dose	− 0.0890	0.0646	− 1.3779	0.1682
	Gender	− 0.0772	0.0924	− 0.8357	0.4033
	Time*	0.0050	0.0021	2.3691	**0.0178**
16:1 (DELTA.9-Cis) PC	(Intercept)	1.0554	0.0580	18.2081	–
	Dose*	− 0.1222	0.0593	− 2.0605	**0.0393**
	Gender	− 0.0483	0.0593	− 0.8144	0.4154
	Time***	0.0061	0.0019	3.2196	**0.0013**
Methyl γ-linolenate	(Intercept)	0.8299	0.0738	11.2397	–
	Dose*	0.1470	0.0683	2.1523	**0.0314**
	Gender	− 0.0620	0.0718	− 0.8641	0.3875
	Time*	0.0058	0.0025	2.3286	**0.0199**
Phosphocholine	(Intercept)	0.8759	0.0706	12.4146	–
	Dose*	0.1693	0.0722	2.3451	**0.0190**
	Gender	− 0.1060	0.0722	− 1.4681	0.1421
	Time*	0.0052	0.0023	2.2487	**0.0245**
Palmitamide	(Intercept)	0.8858	0.0746	11.8819	–
	Dose*	0.1642	0.0763	2.1526	**0.0314**
	Gender	− 0.1167	0.0763	− 1.5296	0.1261
	Time*	0.0053	0.0024	2.1608	**0.0307**
Oleic acid	(Intercept)	0.8840	0.0695	12.7141	–
	Dose*	0.1532	0.0712	2.1524	**0.0314**
	Gender	− 0.1035	0.0712	− 1.4552	0.1456
	Time*	0.0048	0.0023	2.1200	**0.0340**
Oleoyl ethylamide	(Intercept)	0.8499	0.0794	10.6980	–
	Dose*	0.1758	0.0813	2.1619	**0.0306**
	Gender	− 0.1560	0.0813	− 1.9192	0.0550
	Time***	0.0081	0.0026	3.1088	**0.0019**
Oleamide	(Intercept)	0.9410	0.1063	8.8523	–
	Dose*	0.3025	0.1088	2.7805	**0.0054**
	Gender*	− 0.2371	0.1088	− 2.1799	**0.0293**
	Time*	0.0079	0.0035	2.2814	**0.0225**

A comprehensive list of all detected features with test statistics (p-value, FDR-adjusted p-value, fold change and log(2) fold change) for each tested group post drug administration compared to pre-drug administration for both tested doses.

Significant values are given in bold.

*Indicates p < 0.05, ** < 0.01, *** < 0.001.

**Figure 6:**
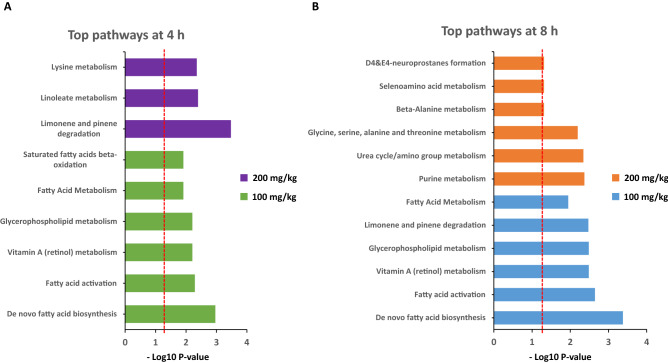
Pathway analysis as computed utilizing all detected features of both tested doses at the 4 h as well as the 8 h time points using Mummichog 2.06 software (**A** and **B**). The dotted lines denote the significance threshold.

For a comprehensive view of the pathways impacted by the drug administration, we utilized Mummichog 2.06 software (Supplementary Table 4A and B), particularly for the data extracted from the samples at the 4 and 8 h time points. Pathway analysis at these two time points of interest also revealed association with de novo fatty acid biosynthesis, activation, and metabolism as well as with the TCA cycle and linoleate metabolism. Surprisingly, the lower dose imposed more prominent pathway changes compared to the higher dose. A diagram presents bar charts with all the pathways altered by both tested doses at the 4 and 8 h time points (Fig. 6).

## Discussion

Over the past several years, we have evaluated various BIO 300 formulations for prophylactic dosing against H-ARS in different animal models. While parental formulations were proven safe and effective, it was essential to develop a formulation that is suitable for oral administration in an austere environment, stable at ambient temperature, and highly effective and safe upon administration. To date, the BIO 300 OP formulation appears to satisfy all these requirements. As part of the development of a medical countermeasure, it is imperative to determine the metabolic consequences of drug administration as a measure of safety and efficacy using an NHP model. Herein, we utilized UPLC QTOF-MS based metabolomics and lipidomic profiling to gain insights into metabolic consequences of BIO 300 OP treatment in NHPs. We found transient alterations in phenylalanine, tyrosine, glycerophosphocholine, and glycerophosphoserine after 4 h of drug treatment that reverted to near pre-treatment levels within 36–48 h after drug administration. In addition, there was an overlap in the metabolite classes including palmitamide and oleamide measured in sera from these data compared to that obtained from NHPs administered an *im* dose of BIO 300 IS and an equivalent dose of BIO 300 OS that we have reported previously^33^. This initial assessment highlighted the importance of using various bioanalytical techniques to interrogate underlying pharmacological actions of the drug and predict drug toxicity. Of note, we did not observe significant changes in the metabolic profile following a single BIO 300 IS *im* dose of 200 mg/kg. In part, this could be due to the small number of animals (2 males and 2 females) or the modulation of regulatory pathways following the higher dose of BIO 300 OP that abrogate the signals observed following the lower BIO 300 OP dose. The intended dosing regimen for BIO 300 OP is 6 days of consecutive prophylactic dosing; thus, it will be important to analyze the metabolic profile following 6 days of consecutive BIO 300 OP administration in future studies.

While PK parameters for a medical countermeasure candidate are very crucial for a better understanding of the mechanisms of absorption, distribution, and elimination of the drug, dose optimization remains a determining factor for the FDA approval process. Analysis of the PK data, obtained from measuring the serum levels of the drug throughout the study for both tested doses, indicate that there was no influence of the dose on the time to maximum plasma concentration (T_max_). Also, the time for half the maximum concentration (T_1/2_) remains the same. Interestingly, there is an apparent shorter T_max_ in males compared to females following either of the BIO 300 OP doses. Although this observation was made in only two animals for each sex, it may be correlated with sex-specific PK parameters. The differential mean gut transit time is shorter in males compared to females which could lead to a faster absorption time in males compared to females^41,42^. If this is the case, the shorter T_max_ in males may constitute a potential safety factor that requires further investigation. One potential implication of this could be that males may require a different dosing regimen (e.g., more frequent dosing) than females. However, one important limitation in our current study was that the PK profile was only examined in two females and two males at each of the BIO 300 OP doses. Confirmatory studies are required to fully investigate potential sex-specific differences in PK.

The average C_max_ value between groups was nearly quadrupled by doubling the BIO 300 OP dose, while the AUC was only tripled by doubling the dose. Of note, this observation was driven by a single animal in the BIO 300 OP 200 mg/kg group. Overall, compared to PK parameters of BIO 300 OS in NHPs, BIO 300 OP demonstrated an enhanced bioavailability. At an equivalent dose (100 mg/kg), BIO 300 OP had a 2× higher C_max_, and 1.3× higher AUC compared to BIO 300 OS^33^. Compared to BIO 300 OS, BIO 300 OP also demonstrated an improved relative oral bioavailability compared to BIO 300 IS administered *im* (3.5% vs 2.7%)^33^. Since genistein is heavily metabolized during first-pass metabolism, the use of older animals may further increase oral bioavailability as older animals are expected to have a reduced first-pass effect^43^. Interestingly, BIO 300 OP appears to be absorbed faster and eliminated more rapidly compared to BIO 300 OS, as evidenced by the 1-h shorter T_max_ and the roughly 1-h shorter half-life (T_1/2_)^33^. This suggests that an amorphous solid dispersion of synthetic genistein has improved dissolution compared to a suspension of synthetic genistein nanoparticles, which may explain the improved bioavailability of BIO 300 OP vs BIO 300 OS.

Both OP doses resulted in very similar metabolomic profiles compared to the pre-dose samples. However, a slight difference between the tested doses was observed in the 4 h and later time points. We found transient downregulation of several metabolites including methyl linolenate, phosphocholine, palmitamide, oleic acid, oleoyl ethylamide, and oleamide. This pattern was comparable to our previous finding in which we reported a transient perturbation around the 4 h time point in the metabolic profiles of C16 sphinganine following the administration of an equivalent BIO 300 OS dose and of glutamate, suberic acid, sphingosine-1-phosphate as well as nonandioic acid following administration of a single BIO 300 IS dose^33^. This could be due to a natural rebound effect caused by the drug’s elimination from the blood. This temporal effect was transient and was not correlated with the identification of any safety-related features.

A few metabolites were modestly upregulated after administration of the BIO 300 OP formulation including C18(Plasm)-20:4 PE and 16:1 (DELTA 9-CIS) PC. Meanwhile, in our earlier PK and metabolomic studies with BIO 300 when administered orally and *im*, we observed a slight upregulation around the same time in taurocholic acid and tauroursodeoxycholic acid which appeared after a single *im* dose of the drug^33^. This could be attributed to transient changes in lipid biosynthesis, activation, and metabolism. These findings were supported by our pathway analysis that we performed on all detected features utilizing Mummichog 2.06v software. Furthermore, statistical analyses built on the annotated metabolites indicate that there is a statistically significant difference between the doses of BIO 300 OP evaluated. Also, time seems to be crucial since most of the transient changes appear to revert to baseline by 48 h post-drug administration, which correlates with the clearance of the drug. Additionally, a 5-day washout period was not expected to impact drug metabolism or elimination because a half-life of < 2 h provides sufficient time for the first dose to be cleared before dosing animals with the second dose (> 50 half-lives). This was supported by BIO 300 OP T_max_ and T_1/2_ at both dose levels which were similar, suggesting there was no impact on drug absorption or elimination. However, we cannot exclude the potential risk that certain metabolic pathways may still be impacted by the first dose of drug (100 mg/kg) and therefore, the impact of the pharmacokinetics and/or pharmacodynamics of the second dose (200 mg/kg). Future studies with longer washout periods will be required to confirm metabolic homeostasis prior to repeat dosing. Alternatively, naïve animals could be used for each cohort.

Assessment of the bioavailability of the two BIO 300 OP doses demonstrates an expected PK pattern with an expected dose dependent increase in the C_max_ and AUC values at the higher dose. Longitudinal metabolomic investigations show transient metabolic changes that occur 4–8 h post drug administration which seem to stabilize over time (by 48 h). Moreover, no changes in the metabolome impacted safety and all noted changes were transient in nature, which reaffirms the safety profile and concurs with previously reported preclinical experimental results. BIO 300 OP is an ideal candidate for further validation assessments as a radiation countermeasure for the prevention of H-ARS.

## Material and methods

### Animals

Four sex matched NHPs (*Macaca mulatta*; Chinese sub-strain) weighing 4.0–8.3 kg and approximately 2–5 years old were used in this PK study. Young adult animals were used for this PK and metabolomics study because they are used in efficacy studies of H-ARS in NHPs exposed to TBI^44–46^. The animal study was conducted at Citoxlab, A Charles River Company (Laval, QC, Canada). All animals underwent health assessments prior to inclusion to the study per the test facility standard operating procedures. The study was performed according to the *Guide for the Care and Use of Laboratory Animals* of the Institute of Laboratory Animal Resources, National Research Council, US National Academy of Sciences^47^. All animal procedures were completed according to a protocol (3019–3873) approved by the Institutional Animal Care and Use Committee of the Citoxlab. This study is reported in accordance with ARRIVE guidelines. Clinical observations for signs of ill health, behaviour changes, etc., were recorded for all animals during both dosing periods. Clinical signs were recorded at least 5 min following each blood collection time point. Additional clinical observations were performed if deemed necessary to properly monitor animals’ health condition. Animal body weights were recorded 24 h prior to study drug dosing.

### Test article, dosing and serum sample collection from NHPs for PK study

BIO 300 OP is an amorphous solid dispersion of genistein (354 mg Active/g) also containing 65% povidone K12 (w/w), which is produced by hot melt extrusion. Following extrusion, the product is milled into a free-flowing dry powder with a median particle size < 150 μm. The bulk and tapped density of BIO 300 OP is 0.58 and 0.82 g/cm^3^, respectively, suggesting good flow (Carr index = 29%). The synthetic genistein (molecular weight = 270.24) used to produce this formulation is crystalline, lipophilic (LogP = 3) and has poor aqueous solubility (3.6 μg/mL in pH 6.5 PBS). Whereas the solubility of the amorphous compound is improved to 20–30 µg/mL in pH 6.5 PBS (unpublished). This improvement in solubility is also evident in 0.5% simulated intestinal fluid, pH 6.5 (14.2 vs > 150 µg/mL). Analytical testing of BIO 300 OP at release included differential scanning calorimetry (T_g_ (°C) = 127.28 ± 0.22 and ΔC_p_ (J/g °C = 0.36 ± 0.02)), water content (0.63 ± 0.0 wt%) and powder diffraction for crystallinity (small inconsequential peak at 18.3° 2-Theta). BIO 300 OP was administered using a gavage tube attached to a syringe, and was delivered as a suspension in vehicle (0.5% (w/v) Methocel A4M and 3% (w/v) polyvinylpyrrolidone K25 in water). The volume of BIO 300 OP administered was calculated and adjusted based on the most recent body weight of each animal. Following dosing, the gavage tube was rinsed with 10 ml water to ensure the entire dose volume was delivered.

A single dose of BIO 300 OP was administered twice by oral gavage to 4 NHPs, first the 100 mg/kg dose and then the 200 mg/kg dose in the same 4 animals. Doses were separated by a 5-day wash-out period. Blood samples were collected from all animals at the following targeted time points: pre-dose, at 0.25, 0.5, 1, 2, 4, 8, 12, 24, and 48 h after dosing. Each blood sample was collected by femoral, cephalic, or saphenous vein into serum-separating tubes and allowed to clot at room temperature. Samples were centrifuged under refrigeration (set to + 4 °C at 1500 g) for 10 min and allowed to separate, and serum was then collected.

### PK analysis

Bioanalysis of NHP serum samples to determine non-glucuronidated genistein (free genistein or genistein aglycone; active drug) was conducted by Inotiv (West Lafayette, IN USA), using a LC–MS/MS technique in accordance with the previously described protocol^33^. Serum samples were analyzed to determine genistein aglycone concentrations. Genistein aglycone is thought to be the more active form of the drug compared to its conjugates and is the most direct indicator of drug efficacy^39^. Data acquisition was performed using Analyst software. Regression and calculation of results and statistics were performed using Watson^®^ LIMS v7.5 (Thermo Fisher Scientific, Waltham, MA, USA). Fourteen of the NHP serum samples were confirmed by re-analysis because they fell outside the expected result range based on the analysis of samples from nearby time points. Repeat values were within 5% of the initial observations. Serum samples were analyzed to determine genistein aglycone concentrations. Genistein aglycone is thought to be the more active form of the drug compared to its conjugates and is the most direct indicator of drug efficacy^39^. Data acquisition was performed using Analyst software. Regression and calculation of results and statistics were performed using Watson^®^ LIMS v7.5 (Thermo Fisher Scientific, Waltham, MA, USA). Fourteen of the NHP serum samples were confirmed by re-analysis because they fell outside the expected result range based on the analysis of samples from nearby time points. Repeat values were within 5% of the initial observations.

PK parameters were estimated using nominal sampling times relative to each dose administered and nominal doses unless otherwise specified. Serum concentration values obtained at the pre-dose time point were used to estimate the concentration at time zero whenever possible. Unquantifiable concentration values (BQL) were assigned a value of zero. The AUC vs. time curves were calculated using the linear trapezoidal method with linear interpolation. The AUC was not calculated for PK profiles with less than 3 quantifiable concentrations of the test item at separate time points. When practicable, the terminal elimination phase of each concentration versus time curve was identified using at least the final three observed concentration values after C_max_, but not including C_max_. The slope of the terminal elimination phase was determined using log linear regression on the unweighted concentration data. Parameters relying on the determination of the terminal elimination phase were not reported if the coefficient of determination was less than 0.800, or if the extrapolation of the AUC to infinity represented more than 20% of the total area.

### Serum Metabolomics Using UPLC QTOF Analysis

Metabolite extraction was performed by combining 25 µL of serum with 75 µL of an extraction solution made up of 35% water, 25% methanol, 40% isopropanol, 0.1% debrisoquine (1 mg/ml in ddH_2_O) and 0.5% 4-nitrobenzoic acid (1 mg/ml in methanol). The samples were vortexed and kept at 4 °C for 20 min. Next, they were combined with 100 µL of acetonitrile (ACN) and left at − 20 °C for 15 min. Finally, the samples were centrifuged at 15,493×*g* for 20 min at 4 °C and the supernatants were transferred to MS vials for LC–MS analysis. A quality control sample (QC) was also prepared by pooling an aliquot of each of the prepared samples and was run periodically throughout the acquisition sequence.

The sample extracts were acquired on an Acquity UPLC coupled to a Xevo G2 QTOF-MS (Waters Corporation, Milford, MA). A volume of 2 μL of each sample was injected onto either an Acquity UPLC BEH C18, 130 Å, 1.7 µm, 2.1 mm × 50 mm column maintained at 40 °C for the metabolomics acquisition or a CSH C18, 130 Å, 1.7 µm, 2.1 mm × 100 mm column maintained at 65 °C for the lipidomics acquisition. The LC solvents used were 100% water with 0.1% formic acid (A), 100% ACN with 0.1% formic acid (B), 100% isopropanol with 0.1% formic acid and 10 mM ammonium formate (C). The metabolomics gradient with a flow rate of 0.5 m/min was set as follows: initial–98% A, 2% B; 0.5 min–98% A, 2% B; 4.0 min–40% A, 60% B; 8.0 min–2% A, 98% B; 9.0 min–2% B, 98% D; 9.5 min–11.2% B, 88.2% C; 11.0 min–11% B, 88.2% C; 11.5 min–50% A, 50% B; 12.0 min–98% A, 2% B, 13.0 min–98% A, 2% B. The lipidomics solvents were the same as previously mentioned with the addition of 10 mM ammonium formate. The gradient had a flow rate of 0.45 m/min and was run set as follows: initial–30% A, 34% B, 36% C; 0.5 min–30% A, 34% B, 36% C; 8.0 min–10% B, 90% C; 8.5 min–10% B, 90% C; 9.0 min–30% A, 34% B, 36% C; and 11.0 min–30% A, 34% B, 36% C.

The column eluent was introduced into the QTOF MS operating in either positive or negative by electrospray ionization. Positive mode had a capillary voltage of 3.00 kV and a sampling cone voltage of 30 V. Negative mode had a capillary voltage of 2.00 kV and had a sampling cone voltage of 30 V. The desolvation gas flow was set to 1000 L/h and the desolvation temperature was set to 500 °C^48^. The cone gas flow was 25 L/h, and the source temperature was 120 °C. The data was acquired in the sensitivity MS mode with a scan time of 0.300 s and an interscan time of 0.014 s. Accurate mass was maintained by infusing leucine enkephalin (556.2771 m/z) in 50% aqueous acetonitrile (2.0 ng/ml) at a rate of 20 µL/min via the Lockspray interface every 10 s. Data was acquired in Centroid mode with a 50.0 to 1200.0 m/z mass range for ToF MS scanning^48^. The pooled QC was injected every 10 samples to monitor any shifts in retention time and intensities.

### Data processing and statistical analysis

Data were log transformed and scaled following feature detection; statistical comparisons were performed to follow longitudinal changes in metabolomics profiles and their possible impact on the phenotype. Untargeted metabolomics raw data files were first converted to the NetCDF file format using the Databridge tool in MassLynx (Waters Corporation, Milford, MA). All parameters for peak picking were optimized by IPO^49^ (Isotopologue Parameter Optimization) R package then processed by XCMS^50^ package. Data was normalized based on the internal standard and QC-RLSC (QC robust LOESS signal correction^51^). The level of differential intensities for each metabolite was calculated using paired t-test, comparing each time point after drug administration to pre-administration for each dose (effect of drug) constrained by false discovery rate (FDR)-adjusted p value < 0.05. The identity of the most significant features was further validated using tandem mass spectrometry by fragmentation pattern matching against National Institute of Standards and Technology (NIST) database. Pathway analysis was performed using the Mummichog software v2.06. To examine the statistical significance of the drastic change in the MS/MS validated metabolites over 48 h across gender dosage and time, linear mixed effects models were fitted into a log2 transformed metabolite intensities as the response variable, while the gender (categorical variable: female and male), dose (categorical variable: 100 mg/kg and 200 mg/kg), and time were considered independent variables. All statistical analyses were performed using recruiting SAS software (version 9.4; SAS Institute Inc., Cary; North Carolina, USA) and R (version 4.0.2).

## Supplementary Information

Supplementary Tables.

## Data Availability

All data generated or analyzed during this study are included in this article (and its Supplementary files).

## References

[1] Hasegawa A (2015). Health effects of radiation and other health problems in the aftermath of nuclear accidents, with an emphasis on Fukushima. Lancet.

[2] Marzaleh MA (2020). Design and validation of a hospital emergency department preparedness questionnaire for radiation accidents, nuclear accidents, and nuclear terrorism in Iran. Am. J. Disaster Med..

[3] MacVittie TJ, Farese AM, Jackson WE (2020). A systematic review of the hematopoietic acute radiation syndrome (H-ARS) in canines and non-human primates: Acute mixed neutron/gamma vs. reference quality radiations. Health Phys..

[4] Singh VK, Olabisi AO (2017). Nonhuman primates as models for the discovery and development of radiation countermeasures. Expert Opin. Drug Discov..

[5] Singh VK, Seed TM (2017). A review of radiation countermeasures focusing on injury-specific medicinals and regulatory approval status: Part I. Radiation sub-syndromes, animal models and FDA-approved countermeasures. Int. J. Radiat. Biol..

[6] Singh VK, Newman VL, Berg AN, MacVittie TJ (2015). Animal models for acute radiation syndrome drug discovery. Expert Opin. Drug Discov..

[7] Girgis M (2021). Short-term metabolic disruptions in urine of mouse models following exposure to low doses of oxygen ion radiation. J. Environ. Sci. Health C Toxicol. Carcinog..

[8] Li Y (2021). Analysis of the metabolomic profile in serum of irradiated nonhuman primates treated with Ex-Rad, a radiation countermeasure. Sci. Rep..

[9] Dissmore T (2021). Longitudinal metabolic alterations in plasma of rats exposed to low doses of high linear energy transfer radiation. J. Environ. Sci. Health C Toxicol. Carcinog..

[10] Girgis M (2020). Comparative proteomic analysis of serum from nonhuman primates administered BIO 300: A promising radiation countermeasure. Sci. Rep..

[11] Hall EJ, Giaccia AJ (2012). Radiobiology for the Radiobiologist.

[12] Farese AM, MacVittie TJ (2015). Filgrastim for the treatment of hematopoietic acute radiation syndrome. Drugs Today.

[13] Hankey KG (2015). Pegfilgrastim improves survival of lethally irradiated nonhuman primates. Radiat. Res..

[14] Singh VK, Seed TM (2018). An update on sargramostim for treatment of acute radiation syndrome. Drugs Today.

[15] Zhong Y (2021). Efficacy of delayed administration of sargramostim up to 120 hours post exposure in a nonhuman primate total body radiation model. Int. J. Radiat. Biol..

[16] Clayton NP (2021). Sargramostim (rhu GM-CSF) improves survival of non-human primates with severe bone marrow suppression after acute, high-dose, whole-body irradiation. Radiat. Res..

[17] U.S. Food and Drug Administration. *Animal Rule approvals*, https://www.fda.gov/drugs/nda-and-bla-approvals/animal-rule-approvals (2021).

[18] Wong K (2020). Pharmacodynamics of romiplostim alone and in combination with pegfilgrastim on acute radiation-induced thrombocytopenia and neutropenia in non-human primates. Int. J. Radiat. Biol..

[19] Gale RP, Armitage JO (2020). Use of molecularly-cloned haematopoietic growth factors in persons exposed to acute high-dose, high-dose rate whole-body ionizing radiations. Blood Rev..

[20] Wong, K. *et al.* in *Annual Conference of Radiation Research Society* (Virtual, 2020).

[21] Farese AM (2013). Filgrastim improves survival in lethally irradiated nonhuman primates. Radiat. Res..

[22] Singh VK, Seed TM (2021). Radiation countermeasures for hematopoietic acute radiation syndrome: Growth factors, cytokines and beyond. Int. J. Radiat. Biol..

[23] Singh, V. K. & Seed, T. M. An update on romiplostim for treatment of acute radiation syndrome. *Drugs Today* **58**, 133–145. 10.1358/dot.2022.58.3.3367994 (2022) (**in press**). 10.1358/dot.2022.58.3.3367994 35274632

[24] Singh VK, Seed TM (2020). BIO 300: A promising radiation countermeasure under advanced development for acute radiation syndrome and the delayed effects of acute radiation exposure. Expert Opin. Investig. Drugs.

[25] Landauer MR, Harvey AJ, Kaytor MD, Day RM (2019). Mechanism and therapeutic window of a genistein nanosuspension to protect against hematopoietic-acute radiation syndrome. J. Radiat. Res..

[26] Landauer, M. *Herbal Radiomodulators: Applications in Medicine, Homeland Defense and Space* (ed Arora, R.) 163–173 (CABI Publishing, 2008).

[27] Haddad YH, Said RS, Kamel R, Morsy EME, El-Demerdash E (2020). Phytoestrogen genistein hinders ovarian oxidative damage and apoptotic cell death-induced by ionizing radiation: Co-operative role of ER-beta, TGF-beta, and FOXL-2. Sci. Rep..

[28] Prossnitz ER, Barton M (2011). The G-protein-coupled estrogen receptor GPER in health and disease. Nat. Rev. Endocrinol..

[29] Xu L, Liu JT, Li K, Wang SY, Xu S (2019). Genistein inhibits Ang II-induced CRP and MMP-9 generations via the ER-p38/ERK1/2-PPARgamma-NF-kappaB signaling pathway in rat vascular smooth muscle cells. Life Sci..

[30] Li XL (2019). Differential effects of genistein and 8-prenylgenistein on reproductive tissues in immature female mice. Pharm. Biol..

[31] Day RM (2013). Enhanced hematopoietic protection from radiation by the combination of genistein and captopril. Int. Immunopharmacol..

[32] Jackson IL (2019). BIO 300, a nanosuspension of genistein, mitigates radiation-induced erectile dysfunction and sensitizes human prostate cancer xenografts to radiation therapy. Int. J. Radiat. Oncol. Biol. Phys..

[33] Cheema AK (2019). Pharmacokinetic and metabolomic studies with BIO 300, a nanosuspension of genistein, in a nonhuman primate model. Int. J. Mol. Sci..

[34] Jackson IL (2017). BIO 300, a nanosuspension of genistein, mitigates pneumonitis/fibrosis following high-dose radiation exposure in the C57L/J murine model. Br. J. Pharmacol..

[35] Park C (2019). Induction of G2/M cell cycle arrest and apoptosis by genistein in human bladder cancer T24 cells through inhibition of the ROS-dependent PI3k/Akt signal transduction pathway. Antioxidants.

[36] Zhang Q, Bao J, Yang J (2019). Genistein-triggered anticancer activity against liver cancer cell line HepG2 involves ROS generation, mitochondrial apoptosis, G2/M cell cycle arrest and inhibition of cell migration. Arch. Med. Sci..

[37] Ha CT, Li XH, Fu D, Xiao M, Landauer MR (2013). Genistein nanoparticles protect mouse hematopoietic system and prevent proinflammatory factors after gamma irradiation. Radiat. Res..

[38] Salem, A. M. *et al.* Interspecies comparison and irradiation effect on pharmacokinetics of BIO 300, a nanosuspension of genistein, following different routes of administration in mice and non-human primates. *Radiat. Res.* **197**, 447–458. 10.1080/14656566.2020.1841748 (2022) (**in Press**). 10.1667/RADE-21-00114.1 35119453

[39] Yang Z, Kulkarni K, Zhu W, Hu M (2012). Bioavailability and pharmacokinetics of genistein: Mechanistic studies on its ADME. Anticancer Agents Med. Chem..

[40] Singh VK (2022). A novel oral formulation of BIO 300 confers prophylactic radioprotection from acute radiation syndrome in mice. Int. J. Radiat. Biol..

[41] Soldin OP, Mattison DR (2009). Sex differences in pharmacokinetics and pharmacodynamics. Clin. Pharmacokinet..

[42] Caballeria J, Baraona E, Rodamilans M, Lieber CS (1989). Effects of cimetidine on gastric alcohol dehydrogenase activity and blood ethanol levels. Gastroenterology.

[43] Mangoni AA, Jackson SH (2004). Age-related changes in pharmacokinetics and pharmacodynamics: Basic principles and practical applications. Br. J. Clin. Pharmacol..

[44] Li Y (2021). Transcriptome of rhesus macaque (*Macaca mulatta*) exposed to total-body irradiation. Sci. Rep..

[45] Singh VK (2016). Radioprotective efficacy of gamma-tocotrienol in nonhuman primates. Radiat. Res..

[46] Beach T (2020). Total body irradiation models in NHPs—Consideration of animal sex and provision of supportive care to advance model development. Int. J. Radiat. Biol..

[47] National Research Council of the National Academy of Sciences (2011). Guide for the care and use of laboratory animals.

[48] Cheema AK (2021). Microbiome study in irradiated mice treated with BIO 300, a promising radiation countermeasure. Anim. Microbiome.

[49] Libiseller G (2015). IPO: A tool for automated optimization of XCMS parameters. BMC Bioinform..

[50] Smith CA, Want EJ, O'Maille G, Abagyan R, Siuzdak G (2006). XCMS: Processing mass spectrometry data for metabolite profiling using nonlinear peak alignment, matching, and identification. Anal. Chem..

[51] Dunn WB (2011). Procedures for large-scale metabolic profiling of serum and plasma using gas chromatography and liquid chromatography coupled to mass spectrometry. Nat. Protoc..

