# Supplementary material for: Pharmacokinetic and metabolomic studies with a BIO 300 Oral Powder formulation in nonhuman primates
Source: Sci Rep. 2022 Aug 5;12:13475. doi: 10.1038/s41598-022-17807-7 (PMC9356050; doi:10.1038/s41598-022-17807-7)
Supplement: Supplementary file 1 — Supplementary Tables. [file 41598_2022_17807_MOESM1_ESM.pdf]

Supplementary Table 1. A comprehensive list of all detected features with test statistics (p- value, FDR-adjusted p-value, fold change and log(2) fold change) for each tested group post drug administration compared to the pre-drug administration for both tested doses.

100 mg/kg

			0.25 h vs. Pre				0.5 h vs. Pre				1 h vs. Pre				2 h vs. Pre				4 h vs. Pre				8 h vs. Pre				12 h vs. Pre				24 h vs. Pre				48 h vs. Pre			
m/z	RT	Mode	p-value	FDR	Fold Change	Log2 (FC)	p-value	FDR	Fold Change	Log2 (FC)	p-value	FDR	Fold Change	Log2 (FC)	p-value	FDR	Fold Change	Log2 (FC)	p-value	FDR	Fold Change	Log2 (FC)	p-value	FDR	Fold Change	Log2 (FC)	p-value	FDR	Fold Change	Log2 (FC)	p-value	FDR	Fold Change	Log2 (FC)	p-value	FDR	Fold Change	Log2 (FC)
462.9774	12.01	olomics Ne	0.001022	0.99918	1.3411	0.42343	0.69403	0.94239	0.96992	-0.04406	0.023267	0.99995	1.1285	0.17445	0.53166	0.99985	0.98221	-0.02589	0.55346	0.99927	1.0986	0.13568	0.82509	0.96067	1.0284	0.040404	0.00531	0.2539	1.1823	0.24154	0.41879	0.99512	0.95535	-0.0659	0.44706	0.99941	1.1222	0.16632
866.7725	10.11	domics Pos	0.001721	0.94464	1.2692	0.34386	0.65088	0.99994	1.0949	0.13085	0.66465	0.99996	1.0644	0.09002	0.059415	0.42032	1.1284	0.17434	0.15134	0.23855	0.86518	-0.20893	0.97245	0.98473	1.0039	0.00564	0.44344	0.80121	1.1899	0.25081	0.23819	0.94365	1.1161	0.15849	0.063977	0.16231	1.3868	0.47171
97.1002	2.76	domics Pos	0.002073	0.94464	1.3256	0.40667	0.425	0.99994	0.90736	-0.14025	0.055712	0.99996	0.87306	-0.19585	0.00036	0.164	0.58125	-0.78276	7.98E-06	0.000198	0.27043	-1.8867	2.99E-06	0.011414	0.45888	-1.1238	0.005221	0.3088	0.56251	-0.83005	0.00102	0.41831	0.60753	-0.71898	0.058094	0.15539	1.2474	0.3189
360.9796	4.36	olomics Ne	0.002764	0.99918	1.1146	0.15647	0.33075	0.74862	0.96038	-0.05833	0.059247	0.99995	1.0692	0.096595	0.38843	0.99985	1.0459	0.064776	0.45822	0.99927	1.0381	0.054008	0.10852	0.57882	1.0862	0.11932	0.007665	0.2539	1.1068	0.14633	0.13751	0.99512	1.0978	0.13468	0.2247	0.99941	1.0613	0.085769
448.9751	4.99	olomics Ne	0.002828	0.99918	1.0726	0.10105	0.2353	0.6568	0.9473	-0.07811	0.73355	0.99995	0.99056	-0.01369	0.73865	0.99985	1.0257	0.03654	0.68444	0.99927	1.0205	0.029242	0.077243	0.53721	1.1012	0.1391	0.026668	0.33786	1.0864	0.11953	0.1885	0.99512	1.0713	0.099342	0.58926	0.99941	1.0214	0.030575
285.2985	2.67	domics Pos	0.002833	0.94464	1.3687	0.45284	0.20498	0.99994	0.85149	-0.23193	0.87368	0.99996	1.0167	0.023908	0.10985	0.53208	0.7078	-0.49858	1.04E-06	0.000144	0.10509	-3.2502	0.003268	0.064159	0.43749	-1.1927	0.041887	0.38314	0.70737	-0.49947	0.44907	0.96776	0.92384	-0.11428	0.000484	0.07528	1.6579	0.7294
215.6598	7.02	olomics Po	0.002907	0.99951	1.318	0.39834	0.99443	0.99998	1.0203	0.02898	0.24695	0.99941	1.0885	0.12237	0.33944	0.83291	0.85203	-0.23102	0.000153	0.002261	0.24438	-2.0328	0.006126	0.065713	0.59236	-0.75546	0.029953	0.34945	0.79023	-0.33966	0.37066	0.99956	0.96004	-0.05883	0.003488	0.2688	1.3643	0.4482
327.3329	7.91	olomics Po	0.003341	0.99951	0.67619	-0.5645	0.007872	0.99998	0.66224	-0.59457	0.086096	0.99941	0.76073	-0.39455	0.033307	0.25203	0.7845	-0.35015	0.10389	0.29508	0.87065	-0.19984	0.000234	0.038868	0.53045	-0.91471	0.048738	0.44698	0.615	-0.70135	0.00174	0.47452	0.56925	-0.81288	0.023326	0.2688	0.67671	-0.5634
306.277	2.67	domics Pos	0.003739	0.94464	1.3473	0.43005	0.41991	0.99994	0.88666	-0.17355	0.52711	0.99996	1.0685	0.095616	0.069625	0.44997	0.68469	-0.54647	6.02E-07	0.000144	0.11476	-3.1233	0.009196	0.086818	0.47	-1.0893	0.027372	0.34512	0.65688	-0.60629	0.31888	0.9524	0.85631	-0.22379	0.001469	0.07528	1.5648	0.64601
110.1062	1.66	domics Pos	0.003913	0.94464	1.2975	0.37575	0.91397	0.99994	1.0884	0.12216	0.86333	0.99996	1.0492	0.069305	0.42808	0.86136	0.90826	-0.13882	8E-06	0.000198	0.25468	-1.9732	0.025913	0.14226	0.73364	-0.44685	0.06552	0.46042	0.727	-0.45998	0.68753	0.97988	1.033	0.046786	0.000416	0.07528	1.641	0.71453
284.2954	2.67	domics Pos	0.004234	0.94464	1.3613	0.44494	0.18689	0.99994	0.84355	-0.24546	0.80124	0.99996	1.0229	0.032658	0.11259	0.53238	0.71907	-0.47579	8.44E-07	0.000144	0.11537	-3.1157	0.003117	0.063546	0.45755	-1.128	0.050873	0.41162	0.7256	-0.46275	0.51825	0.96776	0.93482	-0.09725	0.00094	0.07528	1.6332	0.70769
725.8278	12.05	olomics Po	0.005129	0.99951	0.79683	-0.32765	0.19702	0.99998	0.90362	-0.14621	0.1565	0.99941	0.87392	-0.19443	0.15182	0.54782	0.85802	-0.22091	0.64279	0.98568	1.0374	0.05294	0.68879	0.90962	1.0181	0.025861	0.2451	0.85167	0.87945	-0.18533	0.59496	0.99956	0.96812	-0.04675	0.8606	0.94487	0.98977	-0.01484
874.8013	10.12	domics Pos	0.005246	0.94464	1.2527	0.32506	0.17824	0.99994	1.1088	0.14894	0.47008	0.99996	1.0451	0.06365	0.3765	0.82708	1.0615	0.086053	0.10525	0.1807	0.89899	-0.15362	0.91881	0.95748	1.0034	0.004883	0.38909	0.7665	1.0632	0.088398	0.67138	0.97988	1.0634	0.088675	0.009163	0.092545	1.2993	0.37778
308.2954	2.32	domics Pos	0.006058	0.94464	1.3732	0.45758	0.32515	0.99994	0.86679	-0.20625	0.95469	0.99996	1.0165	0.023645	0.16556	0.59723	0.77488	-0.36796	8.94E-07	0.000144	0.13575	-2.881	0.010136	0.08787	0.528	-0.92139	0.12567	0.58627	0.7669	-0.3829	0.62314	0.97813	0.95509	-0.06629	0.007008	0.091944	1.4781	0.56375
890.5138	4.79	domics Pos	0.006236	0.94464	1.3679	0.45194	0.097805	0.99994	1.1735	0.23087	0.13446	0.99996	1.1394	0.18823	0.54196	0.90473	1.1224	0.1666	0.35385	0.44706	0.91721	-0.12467	0.92002	0.95771	1.0189	0.027055	0.084741	0.50896	1.2789	0.35491	0.77904	0.97988	1.0151	0.02168	0.000475	0.07528	1.5485	0.63092
285.299	6.91	olomics Po	0.006285	0.99951	1.3383	0.42044	0.23769	0.99998	0.87089	-0.19943	0.69715	0.99941	1.0364	0.051554	0.17948	0.60401	0.77717	-0.36371	5.63E-06	0.000265	0.15128	-2.7247	0.00603	0.065713	0.50559	-0.98396	0.033832	0.36264	0.73346	-0.44722	0.3655	0.99956	0.92386	-0.11426	0.003303	0.2688	1.4106	0.49632
1161.739	10.03	domics Pos	0.006594	0.94464	1.1557	0.20872	0.28241	0.99994	1.1107	0.15147	0.93549	0.99996	1.0165	0.023551	0.34768	0.80096	0.93066	-0.10368	0.01625	0.044353	0.79797	-0.3256	0.48149	0.70127	0.94138	-0.08714	0.90448	0.97782	1.0414	0.058569	0.75639	0.97988	0.9798	-0.02944	0.00072	0.07528	1.3954	0.48063
242.2479	5.83	olomics Po	0.007535	0.99951	1.3031	0.3819	0.40054	0.99998	0.91729	-0.12455	0.67206	0.99941	1.0588	0.082365	0.13768	0.52064	0.82117	-0.28424	9.63E-06	0.000313	0.29627	-1.755	0.033887	0.14271	0.63347	-0.65865	0.12288	0.65834	0.81163	-0.30111	0.77766	0.99956	0.98576	-0.02069	0.005774	0.2688	1.2171	0.28347
293.2844	2.76	domics Pos	0.007648	0.94464	1.3801	0.46482	0.26951	0.99994	0.85986	-0.21783	0.75862	0.99996	1.0301	0.042771	0.10446	0.52894	0.71068	-0.49272	1.62E-06	0.000144	0.12669	-2.9807	0.003149	0.063857	0.47039	-1.0881	0.088754	0.51701	0.73424	-0.44568	0.47941	0.96776	0.91356	-0.13042	0.002322	0.082646	1.5864	0.66572
596.7619	0.3	olomics Po	0.007714	0.99951	1.1934	0.25512	0.03293	0.99998	1.2408	0.31128	0.41628	0.99941	1.1252	0.17024	0.29334	0.77976	1.096	0.13227	0.00703	0.037008	1.2155	0.2816	0.025264	0.11873	1.1675	0.22341	0.14795	0.72068	1.2533	0.32572	0.32163	0.99956	1.1485	0.19972	0.23299	0.4991	1.0618	0.086469
312.3265	3.43	domics Pos	0.007754	0.94464	1.5586	0.64024	0.42914	0.99994	0.87761	-0.18835	0.79022	0.99996	1.0304	0.043242	0.10464	0.52894	0.64092	-0.64179	3.97E-05	0.000549	0.209	-2.2585	0.003325	0.064352	0.39938	-1.3242	0.021005	0.33809	0.59815	-0.74142	0.55122	0.96776	0.91392	-0.12986	0.006913	0.091944	2.0332	1.0237
276.2761	7.02	olomics Po	0.007879	0.99951	1.3854	0.47033	0.86631	0.99998	0.99174	-0.01197	0.89499	0.99941	1.0276	0.039227	0.14487	0.53687	0.74048	-0.43346	2.35E-05	0.00053	0.16472	-2.6019	0.016063	0.093702	0.54282	-0.88145	0.038879	0.39092	0.72007	-0.47379	0.39324	0.99956	0.91991	-0.12043	0.003139	0.2688	1.4571	0.54313
268.2638	2.76	domics Pos	0.008362	0.94464	1.3557	0.43904	0.73504	0.99994	0.96224	-0.05553	0.55038	0.99996	0.93622	-0.09507	0.10216	0.52628	0.74868	-0.41758	5.3E-06	0.000159	0.24529	-2.0275	0.001076	0.043236	0.4459	-1.1652	0.16527	0.63453	0.82777	-0.27269	0.25984	0.94365	0.87593	-0.19111	0.002201	0.082646	1.4896	0.57494
97.1011	8.37	olomics Po	0.008737	0.99951	1.2299	0.29849	0.54103	0.99998	1.0499	0.070278	0.088865	0.99941	1.2014	0.26467	0.64003	0.99905	1.0881	0.12177	0.092034	0.26818	1.1545	0.20722	0.62208	0.87981	0.96836	-0.04639	0.19227	0.77069	1.1511	0.20307	0.34743	0.99956	0.91807	-0.12333	0.45537	0.71854	0.91335	-0.13075
202.6516	2.67	domics Pos	0.008749	0.94464	1.287	0.36404	0.28299	0.99994	0.88332	-0.17899	0.19921	0.99996	1.1462	0.19685	0.15602	0.58496	0.78798	-0.34376	1.67E-05	0.00033	0.14201	-2.8159	0.004404	0.071311	0.46102	-1.1171	0.081746	0.49998	0.7591	-0.39765	0.73523	0.97988	1.0325	0.04616	0.000878	0.07528	1.7817	0.83325
295.2832	7.02	olomics Po	0.008915	0.99951	1.399	0.48441	0.29676	0.99998	0.93376	-0.09887	0.26363	0.99941	1.0966	0.13306	0.46014	0.93772	0.89933	-0.15308	1.29E-06	0.000231	0.28185	-1.827	0.00102	0.043099	0.57999	-0.78591	0.019228	0.30136	0.73387	-0.4464	0.31128	0.99956	0.91956	-0.12099	0.000461	0.2688	1.396	0.48134
500.9063	10.02	domics Pos	0.009	0.94464	1.1362	0.18417	0.46633	0.99994	0.94275	-0.08506	0.44896	0.99996	0.9531	-0.0693	0.52632	0.89938	0.95907	-0.06029	0.00166	0.00786	0.7814	-0.35586	0.31555	0.59475	0.91381	-0.13004	0.14965	0.61652	1.0875	0.12099	0.44484	0.96776	1.051	0.071794	0.096099	0.1974	1.1849	0.24481
311.3143	2.76	domics Pos	0.009033	0.94464	1.3583	0.44179	0.30007	0.99994	0.87298	-0.19598	0.88925	0.99996	1.0117	0.016812	0.11184	0.53238	0.72504	-0.46387	3.36E-06	0.000144	0.13561	-2.8825	0.002776	0.061655	0.47646	-1.0696	0.080893	0.4991	0.73587	-0.44248	0.42115	0.96776	0.90657	-0.14151	0.002457	0.082646	1.5514	0.63359
123.1165	2.76	domics Pos	0.009046	0.94464	1.3655	0.44938	0.75901	0.99994	1.0342	0.048568	0.70265	0.99996	1.0573	0.080342	0.056169	0.40873	0.6961	-0.52264	5.74E-06	0.000163	0.29471	-1.7626	0.002112	0.060099	0.54357	-0.87945	0.36399	0.75925	0.86466	-0.20979	0.57129	0.9703	0.93912	-0.09061	0.019291	0.10555	1.5228	0.60669
274.9841	12	olomics Ne	0.009183	0.99918	1.2932	0.37097	0.072736	0.50118	1.1353	0.1831	0.4547	0.99995	1.1414	0.19078	0.023426	0.99985	1.1552	0.20811	0.00486	0.54393	1.2972	0.37545	0.008296	0.36417	1.2118	0.27711	0.718	0.91175	1.047	0.066262	0.018807	0.99512	1.2653	0.33948	0.18232	0.99941	1.1639	0.21896
480.9747	5.16	olomics Ne	0.009252	0.99918	1.107	0.14667	0.12537	0.54655	0.951	-0.07248	0.1447	0.99995	1.0531	0.074642	0.64379	0.99985	1.0281	0.039915	0.43707	0.99927	1.0441	0.062254	0.10676	0.57882	1.1511	0.203	0.033875	0.3559	1.0821	0.11379	0.42463	0.99512	1.0552	0.077507	0.13516	0.99941	1.0536	0.075289
192.1833	6.38	olomics Po	0.009437	0.99951	1.1708	0.22746	0.10422	0.99998	0.86734	-0.20534	0.75165	0.99941	0.97811	-0.03193	0.15066	0.5457	0.80232	-0.31776	1.87E-05	0.000463	0.23398	-2.0955	0.0041	0.054724	0.55257	-0.85576	0.019352	0.30136	0.7227	-0.46854	0.12342	0.8972	0.84287	-0.24662	0.32279	0.60059	1.0949	0.13085
294.2877	2.76	domics Pos	0.00984	0.94464	1.3616	0.44534	0.26273	0.99994	0.86248	-0.21343	0.7754	0.99996	1.0246	0.034992	0.052122	0.39122	0.68659	-0.54248	1.33E-06	0.000144	0.1151	-3.119	0.004776	0.07169	0.43304	-1.2074	0.089034	0.51701	0.73079	-0.45247	0.3702	0.96776	0.88121	-0.18244	0.001767	0.077423	1.5456	0.6282
920.5121	0.55	domics Pos	0.009867	0.94464	1.5121	0.59659	0.40952	0.99994	0.9111	-0.13432	0.63179	0.99996	1.0545	0.076574	0.019627	0.25861	0.57968	-0.78668	0.001514	0.007372	0.48524	-1.0432	0.000659	0.03855	0.5887	-0.76441	0.35957	0.75623	0.86838	-0.20359	0.69096	0.97988	0.97984	-0.02939	0.005616	0.08815	2.319	1.2135
217.1963	1.23	domics Pos	0.010092	0.94464	1.3189	0.39929	0.30469	0.99994	0.88056	-0.18351	0.27249	0.99996	1.1544	0.20713	0.11334	0.53238	0.81658	-0.29234	3.4E-05	0.000495	0.32978	-1.6004	0.024701	0.13871	0.69395	-0.5271	0.35193	0.75095	0.89191	-0.16503	0.4638	0.96776	0.91804	-0.12337	0.014739	0.098791	1.3117	0.39145
241.2211	6.39	olomics Po	0.010222	0.99951	1.2484	0.32007	0.52159	0.99998	0.95011	-0.07383	0.59221	0.99941	1.0335	0.047499	0.30144	0.79062	0.88276	-0.1799	0.000106	0.001675	0.48892	-1.0323	0.021808	0.10951	0.7967	-0.32789	0.28385	0.90284	0.88766	-0.17192	0.53292	0.99956	0.95099	-0.07249	0.11964	0.36992	1.1763	0.2343
286.3016	2.66	domics Pos	0.010391	0.94464	1.3959	0.48117	0.2032	0.99994	0.83484	-0.26043	0.81547	0.99996	1.0191	0.027286	0.1461	0.57298	0.73253	-0.44904	1.98E-06	0.000144	0.11397	-3.1333	0.005825	0.076369	0.44043	-1.183	0.07016	0.47049	0.69302	-0.52904	0.42091	0.96776	0.90497	-0.14405	0.003373	0.085302	1.7068	0.77133
512.9775	5.19	olomics Ne	0.010464	0.99918	1.1195	0.16279	0.66595	0.92948	0.97766	-0.03259	0.20523	0.99995	1.0614	0.085992	0.41052	0.99985	1.0638	0.089266	0.48148	0.99927	1.0395	0.05588	0.063149	0.51001	1.1308	0.17736	0.007018	0.2539	1.1436	0.19357	0.73572	0.99512	1.0279	0.039758	0.42461	0.99941	1.0462	0.06509
125.0966	1.66	domics Pos	0.010492	0.94464	1.396	0.48125	0.5779	0.99994	0.94575	-0.08046	0.16433	0.99996	1.133	0.18011	0.14347	0.57187	0.78379	-0.35147	8.62E-06	0.000205	0.27402	-1.8676	0.008274	0.084185	0.55574	-0.84752	0.317	0.72948	0.87062	-0.19989	0.99792	0.99949	1.0055	0.007958	0.005615	0.08815	1.4752	0.56089
565.2819	2.53	olomics Po	0.010525	0.99951	1.7814	0.83297	0.61976	0.99998	0.94645	-0.0794	0.78254	0.99941	0.96923	-0.04509	0.036335	0.25997	0.66439	-0.58989	0.002325	0.017262	0.45101	-1.1488	0.11509	0.32925	0.69153	-0.53215	0.80186	0.99391	1.1691	0.22543	0.89905	0.99956	1.0859	0.11888	0.008506	0.2688	2.3351	1.2235
390.1796	2.05	domics Pos	0.010756	0.94464	1.4083	0.49396	0.79379	0.99994	1.0326	0.046333	0.51354	0.99996	1.0897	0.12391	0.076807	0.46503	0.78266	-0.35353	3.23E-05	0.000487	0.33846	-1.5629	0.025409	0.14021	0.62123	-0.6868	0.17431	0.64606	0.86025	-0.21718	0.74651	0.97988	0.96545	-0.05072	0.005208	0.088007	1.5526	0.63467
404.858	0.3	olomics Po	0.010764	0.99951	0.79006	-0.33996	0.40183	0.99998	0.94201	-0.08619	0.96238	0.99941	1.0219	0.031279	0.30678	0.79641	0.90319	-0.14689	0.12091	0.33616	0.86267	-0.21312	0.06363	0.21882	0.8319	-0.26551	0.005429	0.2079	0.74437	-0.42591	0.70755	0.99956	0.9759	-0.0352	0.19626	0.45998	0.90645	-0.1417
169.1586	2.05	domics Pos	0.010831	0.94464	1.4938	0.57899	0.59603	0.99994	0.93387	-0.0987	0.65626	0.99996	1.0654	0.091418	0.15038	0.57859	0.76033	-0.39529	0.00016	0.001427	0.25289	-1.9834	0.005214	0.074135	0.59726	-0.74357	0.83059	0.96528	0.99227	-0.01119	0.83291	0.97988	1.0323	0.045818	0.007469	0.091944	1.6256	0.70094
1180.773	10.02	domics Pos	0.010913	0.94464	1.0901	0.12447	0.65405	0.99994	0.97768	-0.03256	0.63642	0.99996	0.96775	-0.04729	0.2496	0.70866	0.93614	-0.0952	0.006721	0.022759	0.85131	-0.23224	0.20414	0.49389	0.92218	-0.11687	0.99607	0.99769	1.0116	0.016707	0.80814	0.97988	1.0286	0.04067	0.001433	0.07528	1.2715	0.34657
136.1206	2.05	domics Pos	0.011011	0.94464	1.3845	0.46937	0.78719	0.99994	1.0362	0.051298	0.1938	0.99996	1.0951	0.131	0.41475	0.85524	0.89486	-0.16026	7.14E-06	0.000186	0.21288	-2.2319	0.001325	0.046324	0.60583	-0.72302	0.10934	0.55771	0.84048	-0.25071	0.89104	0.98023	0.98983	-0.01475	0.000929	0.07528	1.5304	0.61392
152.889	0.34	olomics Ne	0.011115	0.99918	0.93778	-0.09268	0.044914	0.47152	0.90052	-0.15117	0.46982	0.99995	0.97586	-0.03525	0.39952	0.99985	1.0343	0.048649	0.68285	0.99927	1.0234	0.033352	0.8821	0.9778	1.0132	0.018858	0.98044	0.99049	0.99997	-4.1E-05	0.11958	0.99512	0.93541	-0.09633	0.2273	0.99941	0.94166	-0.08672
313.3296	3.43	domics Pos	0.011163	0.94464	1.5225	0.60644	0.34005	0.99994	0.84236	-0.24749	0.78685	0.99996	1.045	0.063518	0.064971	0.43615	0.67	-0.57776	4.2E-05	0.000559	0.18856	-2.4069	0.000226	0.026314	0.40794	-1.2936	0.031395	0.35841	0.60346	-0.72867	0.29613	0.9483	0.82431	-0.27873	0.008643	0.092495	2.044	1.0314
752.9739	12.01	olomics Ne	0.01137	0.99918	1.1954	0.2575	0.302	0.73566	0.90845	-0.13852	0.83239	0.99995	0.98739	-0.0183	0.77552	0.99985	0.98315	-0.02451	0.79967	0.99927	1.0429	0.060571	0.77703	0.95082	1.0549	0.07715	0.11999	0.5007	1.088	0.12172	0.95333	0.99512	1.0034	0.004866	0.10171	0.99941	1.1644	0.21953
184.1699	2.76	domics Pos	0.011469	0.94464	1.3311	0.41265	0.24455	0.99994	0.84562	-0.24193	0.41926	0.99996	0.92562	-0.11151	0.052928	0.39494	0.67255	-0.5723	6.34E-06	0.000174	0.15544	-2.6856	0.001125	0.04331	0.46912	-1.092	0.10101	0.54271	0.7339	-0.44635	0.53146	0.96776	0.92811	-0.10764	0.00469	0.085788	1.4942	0.57935
312.3174	2.76	domics Pos	0.011517	0.94464	1.4198	0.50567	0.31337	0.99994	0.85366	-0.22827	0.72574	0.99996	1.032	0.045392	0.08665	0.49062	0.68601	-0.5437	1.13E-05	0.000249	0.12629	-2.9852	0.003899	0.068644	0.47252	-1.0816	0.13251	0.59878	0.74326	-0.42806	0.57584	0.9703	0.9207	-0.1192	0.003516	0.085302	1.6165	0.69286
150.1366	6	olomics Po	0.011668	0.99951	1.2138	0.27951	0.27936	0.99998	0.91767	-0.12395	0.14289	0.99941	1.1572	0.21063	0.40421	0.89711	0.89075	-0.16691	4.73E-05	0.000892	0.35897	-1.4781	0.000399	0.039572	0.62508	-0.67789	0.2915	0.90579	0.88258	-0.1802	0.50694	0.99956	0.94479	-0.08193	0.26234	0.53246	1.1327	0.17977
291.1747	5.16	olomics Ne	0.011737	0.99918	1.1886	0.24926	0.46503	0.82998	0.93832	-0.09185	0.42302	0.99995	1.0576	0.080787	0.72345	0.99985	0.97562	-0.0356	0.2257	0.99927	1.1098	0.15035	0.75485	0.94707	0.97653	-0.03427	0.91352	0.97375	1.013	0.018637	0.83895	0.99512	1.0145	0.020797	0.3487	0.99941	0.92561	-0.11153
630.7971	10.13	domics Pos	0.011806	0.94464	1.153	0.20534	0.55549	0.99994	0.97712	-0.03339	0.53626	0.99996	1.0535	0.075227	0.15347	0.58077	1.1262	0.17142	0.000397	0.00275	0.79139	-0.33754	0.44398	0.67607	1.0937	0.12916	0.10322	0.54665	1.2146	0.28053	0.042209	0.64896	1.1432	0.19306	0.003311	0.085302	1.3557	0.43904
111.0909	1.66	domics Pos	0.011819	0.94464	1.3602	0.44381	0.71679	0.99994	0.96811	-0.04675	0.30903	0.99996	1.1162	0.1586	0.097798	0.51762	0.77924	-0.35985	8.46E-06	0.000205	0.25972	-1.945	0.007099	0.081828	0.61688	-0.69694	0.72289	0.9233	0.96907	-0.04533	0.77517	0.97988	1.0435	0.061392	0.001565	0.07528	1.4297	0.51574
310.3111	2.76	domics Pos	0.011823	0.94464	1.339	0.42115	0.26868	0.99994	0.86292	-0.21271	0.99396	0.99996	0.99584	-0.00602	0.10134	0.52336	0.71495	-0.48408	3.49E-06	0.000144	0.14062	-2.8301	0.002924	0.062584	0.48051	-1.0573	0.074479	0.48057	0.73018	-0.45367	0.41996	0.96776	0.90482	-0.1443	0.002505	0.082646	1.5476	0.63001
518.9731	11.99	olomics Ne	0.011957	0.99918	1.1284	0.17432	0.94046	0.9934	0.99885	-0.00166	0.18973	0.99995	1.1323	0.17928	0.53871	0.99985	1.0411	0.058153	0.30092	0.99927	1.1022	0.1404	0.12279	0.58865	1.1355	0.18327	0.003356	0.22316	1.1909	0.25203	0.50295	0.99512	1.0594	0.083315	0.96108	0.99945	1.0123	0.017604
836.7804	10.11	domics Pos	0.012034	0.94464	0.86066	-0.21649	0.005566	0.99994	0.80352	-0.31559	0.25897	0.99996	0.8816	-0.1818	0.22613	0.68159	0.90215	-0.14856	0.000101	0.001031	0.68621	-0.54327	0.21323	0.50494	0.81336	-0.29804	0.002385	0.26175	0.84143	-0.24909	0.30622	0.9524	0.93533	-0.09645	0.6056	0.69784	1.0781	0.10851
304.2616	2.05	domics Pos	0.012363	0.94464	1.252	0.32424	0.30851	0.99994	0.90946	-0.13692	0.98121	0.99996	1.0052	0.00749	0.09569	0.51543	0.77305	-0.37137	3.21E-07	0.000144	0.18218	-2.4565	0.009807	0.087714	0.59083	-0.75919	0.16704	0.6382	0.81092	-0.30236	0.63347	0.97988	0.96452	-0.05212	0.000734	0.07528	1.4028	0.48831
195.1747	2.05	domics Pos	0.012659	0.94464	1.3276	0.40881	0.66475	0.99994	0.95864	-0.06094	0.38138	0.99996	1.1002	0.13773	0.13168	0.55569	0.77894	-0.36041	1.6E-06	0.000144	0.17265	-2.534	0.006821	0.080671	0.56167	-0.8322	0.26521	0.69979	0.85428	-0.22721	0.70289	0.97988	0.96423	-0.05256	0.001505	0.07528	1.4834	0.56892
142.1226	2.76	domics Pos	0.012909	0.94464	1.3601	0.44373	0.22813	0.99994	0.83977	-0.25193	0.89559	0.99996	1.0112	0.016022	0.081905	0.48419	0.70636	-0.50153	2.13E-05	0.000378	0.18956	-2.3993	0.001334	0.046324	0.49146	-1.0249	0.047611	0.39725	0.7118	-0.49045	0.23286	0.94365	0.85011	-0.23427	0.00408	0.085451	1.6221	0.69785
456.9788	4.84	olomics Ne	0.013296	0.99918	1.1004	0.13797	0.26845	0.69571	0.94169	-0.08667	0.11992	0.99995	1.069	0.096252	0.16661	0.99985	1.0812	0.11266	0.21062	0.99927	1.0642	0.089811	0.054257	0.48752	1.1502	0.20184	0.004642	0.2381	1.1887	0.24934	0.081876	0.99512	1.0894	0.12356	0.44224	0.99941	1.0391	0.055385
456.8687	10.15	domics Pos	0.013299	0.94464	1.2793	0.35532	0.38121	0.99994	1.0704	0.098188	0.15469	0.99996	1.1726	0.22967	0.26609	0.72731	1.1341	0.18159	0.24564	0.33998	0.85794	-0.22105	0.18543	0.4723	1.1368	0.18498	0.052608	0.4119	1.2875	0.3646	0.060419	0.72218	1.2093	0.27413	0.046136	0.14289	1.4227	0.50866
317.9035	10.11	domics Pos	0.013585	0.94464	1.1915	0.25284	0.70763	0.99994	1.0164	0.023404	0.21742	0.99996	1.1208	0.16458	0.50076	0.89009	1.0409	0.057862	0.000173	0.001528	0.81449	-0.29604	0.48306	0.70274	0.96303	-0.05435	0.000432	0.17164	1.1915	0.25277	0.076241	0.78569	1.1789	0.2374	0.030432	0.11983	1.2514	0.32352
275.2738	2.76	domics Pos	0.01362	0.94464	1.355	0.43825	0.16622	0.99994	0.82926	-0.27011	0.80334	0.99996	0.97033	-0.04345	0.074411	0.4621	0.70039	-0.51376	1.6E-06	0.000144	0.12291	-3.0243	0.005957	0.077041	0.43706	-1.1941	0.052164	0.41166	0.69371	-0.52761	0.2759	0.94365	0.8723	-0.19711	0.00247	0.082646	1.5491	0.63146
240.2327	2.75	domics Pos	0.01376	0.94464	1.4295	0.51554	0.54901	0.99994	0.9081	-0.13907	0.92604	0.99996	1.0037	0.005371	0.15277	0.58077	0.71644	-0.48108	6.8E-06	0.000183	0.16074	-2.6372	0.006415	0.079158	0.52054	-0.94191	0.11105	0.56029	0.74686	-0.42109	0.48187	0.96776	0.90041	-0.15134	0.01177	0.096308	1.5206	0.60463
147.1172	1.67	domics Pos	0.014135	0.94464	1.2723	0.34745	0.30352	0.99994	0.88674	-0.17341	0.46776	0.99996	1.0922	0.12723	0.30531	0.76288	0.86377	-0.21128	4.28E-06	0.000147	0.21657	-2.2071	0.000741	0.04036	0.55591	-0.84707	0.31792	0.72948	0.86911	-0.20238	0.36855	0.96776	0.91112	-0.13429	0.004902	0.087539	1.3916	0.47675
661.4807	5.2	domics Pos	0.014365	0.94464	1.3041	0.38305	0.084927	0.99994	1.2559	0.32873	0.52274	0.99996	1.1896	0.25048	0.36423	0.82032	0.92577	-0.11128	0.028344	0.068058	0.70807	-0.49804	0.058373	0.24091	0.68065	-0.55502	0.23647	0.67743	0.77924	-0.35986	0.008109	0.52955	0.48014	-1.0585	0.043025	0.13814	0.50948	-0.97291
175.1487	1.67	domics Pos	0.014376	0.94464	1.2949	0.37288	0.59127	0.99994	0.94656	-0.07924	0.63576	0.99996	1.0695	0.096897	0.14499	0.57187	0.77604	-0.3658	2.9E-07	0.000144	0.16696	-2.5824	0.003298	0.064159	0.53431	-0.90426	0.16403	0.63418	0.80363	-0.3154	0.64733	0.97988	0.95606	-0.06483	0.001207	0.07528	1.4739	0.55966
150.1365	1.66	domics Pos	0.014982	0.94464	1.3893	0.47433	0.72672	0.99994	1.0434	0.061309	0.038349	0.99996	1.3043	0.38331	0.18645	0.62584	0.82973	-0.26928	0.0002	0.001698	0.233	-2.1016	0.002658	0.061655	0.56462	-0.82465	0.97256	0.99324	1.0314	0.044637	0.46595	0.96776	1.0706	0.098355	0.001946	0.079269	1.524	0.60789
390.1997	4.11	olomics Po	0.015014	0.99951	1.375	0.45941	0.007673	0.99998	0.64613	-0.63011	0.58491	0.99941	0.94003	-0.08922	0.003969	0.17363	0.47856	-1.0632	0.001038	0.009561	0.30467	-1.7147	0.000545	0.039708	0.40718	-1.2963	0.052807	0.45951	0.61406	-0.70356	0.2928	0.99956	0.87186	-0.19783	0.31649	0.59341	1.2186	0.28526
286.2999	6.9	olomics Po	0.015053	0.99951	1.3164	0.39655	0.2273	0.99998	0.84901	-0.23615	0.80626	0.99941	0.9783	-0.03165	0.20054	0.63556	0.77698	-0.36405	1.1E-05	0.000339	0.14097	-2.8265	0.009275	0.077091	0.49985	-1.0004	0.023515	0.32255	0.66223	-0.59459	0.39687	0.99956	0.91555	-0.12729	0.007877	0.2688	1.4136	0.49933
249.2493	6.38	olomics Po	0.015126	0.99951	1.3173	0.39754	0.862	0.99998	0.98648	-0.01964	0.41612	0.99941	1.1022	0.14032	0.24316	0.70595	0.82681	-0.27437	2.72E-05	0.000587	0.26203	-1.9322	0.005624	0.064425	0.58546	-0.77235	0.20553	0.79634	0.84949	-0.23532	0.9195	0.99956	0.99397	-0.00873	0.087048	0.33918	1.1872	0.24759
788.4335	0.56	domics Pos	0.015426	0.94464	1.4333	0.51934	0.33575	0.99994	0.93205	-0.10152	0.70921	0.99996	0.97953	-0.02984	0.004228	0.17581	0.55071	-0.86064	8.43E-05	0.000913	0.42706	-1.2275	0.000123	0.023299	0.53137	-0.91222	0.41248	0.78028	0.88763	-0.17197	0.57819	0.9703	0.94429	-0.08271	0.00907	0.092545	2.1135	1.0796
98.104	2.05	domics Pos	0.015547	0.94464	1.3509	0.43392	0.078923	0.99994	1.2209	0.2879	0.26911	0.99996	1.2467	0.31817	0.71251	0.96346	0.97804	-0.03204	0.000151	0.00138	0.3242	-1.6251	0.11896	0.37395	0.83033	-0.26824	0.93052	0.98468	1.0622	0.087027	0.027155	0.57206	1.3145	0.39451	0.002088	0.07995	1.6963	0.76239
179.1801	2.05	domics Pos	0.015597	0.94464	1.3691	0.45325	0.35442	0.99994	0.87658	-0.19004	0.48802	0.99996	1.0959	0.13218	0.41578	0.85524	0.88514	-0.17603	3.6E-06	0.000144	0.17121	-2.5462	0.031692	0.16072	0.63064	-0.66511	0.5209	0.83957	0.91996	-0.12036	0.47251	0.96776	1.0643	0.089858	0.000589	0.07528	1.6415	0.71501
256.2642	1.91	domics Pos	0.01567	0.94464	1.2664	0.34072	0.2454	0.99994	0.88336	-0.17893	0.37315	0.99996	1.08	0.11103	0.11994	0.54112	0.76709	-0.38253	8.96E-06	0.000208	0.16471	-2.602	0.00463	0.071311	0.52477	-0.93024	0.15529	0.62672	0.82216	-0.2825	0.61169	0.97371	0.95684	-0.06365	0.000881	0.07528	1.4596	0.54557
301.2528	6.38	olomics Po	0.015753	0.99951	1.2467	0.31811	0.45456	0.99998	0.94223	-0.08585	0.60025	0.99941	1.0748	0.10409	0.49272	0.96763	0.92733	-0.10884	1.13E-05	0.000343	0.20117	-2.3135	0.03056	0.13407	0.61929	-0.69131	0.14847	0.72068	0.858	-0.22094	0.65728	0.99956	0.97762	-0.03266	0.011795	0.2688	1.2572	0.33017
95.0483	0.84	domics Pos	0.016137	0.94464	0.69181	-0.53155	0.027216	0.99994	0.55859	-0.84013	0.19942	0.99996	0.77409	-0.36943	0.11379	0.53238	0.80049	-0.32105	0.010009	0.030703	0.64904	-0.62361	0.016732	0.10983	0.57234	-0.80504	0.054012	0.41438	0.6874	-0.54077	0.013135	0.52955	0.68348	-0.54903	0.061059	0.15806	0.77424	-0.36914
284.2959	6.91	olomics Po	0.016505	0.99951	1.3112	0.39086	0.19698	0.99998	0.86771	-0.20471	0.75538	0.99941	1.0316	0.044875	0.16383	0.57278	0.77441	-0.36883	2.98E-06	0.000231	0.15776	-2.6642	0.005028	0.061419	0.50755	-0.97839	0.022997	0.32178	0.71778	-0.47838	0.30781	0.99956	0.91958	-0.12095	0.003032	0.2688	1.4037	0.48925
191.18	2.05	domics Pos	0.016577	0.94464	1.2803	0.35649	0.42634	0.99994	0.9355	-0.09619	0.55892	0.99996	1.0381	0.053945	0.12322	0.54505	0.76893	-0.37908	4.57E-06	0.000149	0.16591	-2.5915	0.006231	0.078151	0.52141	-0.93951	0.11571	0.5679	0.80247	-0.31748	0.55423	0.96798	0.95154	-0.07166	0.000445	0.07528	1.401	0.48642
264.8659	0.3	olomics Po	0.016659	0.99951	0.80756	-0.30835	0.86991	0.99998	1.0224	0.031978	0.20368	0.99941	0.89647	-0.15768	0.010806	0.19754	0.71156	-0.49095	0.41753	0.81857	0.94253	-0.08539	0.1974	0.47102	0.88016	-0.18417	0.015914	0.29145	0.70648	-0.50128	0.14805	0.93461	0.85479	-0.22636	0.13674	0.39122	0.88774	-0.17179
193.1587	1.66	domics Pos	0.01671	0.94464	1.355	0.4383	0.54234	0.99994	0.93252	-0.10079	0.28834	0.99996	1.145	0.19529	0.22215	0.67657	0.81286	-0.29893	1.29E-05	0.000277	0.20547	-2.283	0.01737	0.11287	0.6081	-0.71763	0.28753	0.71484	0.8485	-0.23701	0.98137	0.99461	0.99676	-0.00468	0.00384	0.085302	1.4591	0.54504
177.1644	2.05	domics Pos	0.017016	0.94464	1.3429	0.42538	0.61388	0.99994	0.94793	-0.07715	0.41016	0.99996	1.0782	0.10861	0.24738	0.70693	0.82154	-0.28359	1.08E-06	0.000144	0.18568	-2.4291	0.008946	0.08583	0.58489	-0.77377	0.3048	0.7283	0.86748	-0.20511	0.81102	0.97988	1.0275	0.039109	0.001435	0.07528	1.516	0.60022
193.1594	5.99	olomics Po	0.017027	0.99951	1.2252	0.29302	0.40093	0.99998	0.92103	-0.11867	0.37049	0.99941	1.0966	0.133	0.098987	0.41966	0.75221	-0.41079	1.61E-06	0.000231	0.23562	-2.0855	0.008617	0.075866	0.61009	-0.71291	0.13662	0.68968	0.82718	-0.27373	0.38697	0.99956	0.92647	-0.11019	0.031116	0.2688	1.1777	0.23602
227.2206	6.38	olomics Po	0.017103	0.99951	1.2927	0.37043	0.95566	0.99998	0.99796	-0.00294	0.32006	0.99941	1.1036	0.14223	0.30232	0.79161	0.8586	-0.21994	1.7E-05	0.000443	0.23044	-2.1176	0.021663	0.10951	0.60011	-0.7367	0.18159	0.75808	0.84023	-0.25115	0.8033	0.99956	0.97449	-0.03728	0.082323	0.33228	1.2228	0.29019
134.9491	12.59	olomics Ne	0.017247	0.99918	1.0997	0.13715	0.21185	0.63372	0.93744	-0.0932	0.58442	0.99995	1.0245	0.03485	0.86685	0.99985	1.0121	0.01735	0.58214	0.99927	1.0383	0.054256	0.047108	0.48405	1.1485	0.19973	0.10918	0.47523	1.0892	0.12333	0.66007	0.99512	1.0314	0.04454	0.29201	0.99941	1.0367	0.052033
168.1394	1.66	domics Pos	0.018015	0.94464	1.2591	0.33244	0.22019	0.99994	0.8884	-0.17072	0.73483	0.99996	1.0436	0.06159	0.12641	0.55024	0.74544	-0.42384	3.52E-06	0.000144	0.20285	-2.3015	0.002189	0.060468	0.54971	-0.86325	0.15086	0.61987	0.77693	-0.36414	0.32329	0.9545	0.9183	-0.12297	0.000902	0.07528	1.4585	0.54449
832.4593	0.54	domics Pos	0.018291	0.94464	1.4344	0.52042	0.33731	0.99994	0.93017	-0.10444	0.87408	0.99996	0.99293	-0.01024	0.005469	0.17822	0.53044	-0.91475	0.000476	0.003154	0.42513	-1.234	6.83E-05	0.019289	0.52537	-0.9286	0.20879	0.65208	0.78794	-0.34384	0.45339	0.96776	0.90746	-0.1401	0.005334	0.088007	2.0594	1.0422
293.2848	7.02	olomics Po	0.018666	0.99951	1.2808	0.35702	0.27602	0.99998	0.8802	-0.18409	0.97828	0.99941	1.0088	0.0126	0.18677	0.61863	0.7839	-0.35126	7.08E-06	0.000278	0.16829	-2.571	0.002613	0.046505	0.51342	-0.96178	0.036747	0.38426	0.72229	-0.46935	0.24135	0.99956	0.90158	-0.14948	0.002702	0.2688	1.3247	0.40571

207.1753	5.99	olomics Pc	0.018751	0.99951	1.2843	0.36093	0.5813	0.99998	0.93892	-0.09093	0.9294	0.99941	1.0152	0.021725	0.15351	0.54951	0.78123	-0.35619	1.1E-05	0.000339	0.25388	-1.9778	0.004964	0.061314	0.56888	-0.8138	0.089551	0.60275	0.77615	-0.36559	0.6895	0.99956	0.95979	-0.05921	0.0476	0.28861	1.2124	0.27785
457.3499	6.56	olomics Pc	0.018861	0.99951	0.86745	-0.20515	0.21921	0.99998	0.9375	-0.09311	0.010206	0.99941	0.89175	-0.16529	0.8923	0.99905	1.0158	0.022657	0.57843	0.95445	1.0216	0.030846	0.41669	0.72477	1.1219	0.16595	0.55304	0.99391	1.0908	0.12543	0.16271	0.9444	0.90245	-0.14808	0.72271	0.87727	0.98011	-0.02898
258.9981	10.2	lomics Pos	0.018968	0.94464	0.87426	-0.19387	0.008588	0.99994	0.8072	-0.309	0.16087	0.99996	0.91188	-0.13309	0.26821	0.72894	0.92787	-0.108	0.000312	0.002286	0.70463	-0.50507	0.19578	0.4835	0.84528	-0.24251	0.18678	0.64793	0.92093	-0.11884	0.2751	0.94365	0.9356	-0.09604	0.35444	0.46363	1.0927	0.12785
476.9013	10.13	lomics Pos	0.018981	0.94464	1.2894	0.36668	0.41027	0.99994	1.0496	0.069878	0.24032	0.99996	1.126	0.17126	0.17547	0.60979	1.1515	0.20353	0.035567	0.080629	0.86018	-0.21728	0.26489	0.55194	1.1677	0.22365	0.041876	0.38314	1.2114	0.27664	0.14073	0.89982	1.1862	0.2464	0.024117	0.11079	1.3214	0.40211
651.8512	0.51	lomics Pos	0.019186	0.94464	1.1953	0.25742	0.78182	0.99994	1.0263	0.03741	0.14262	0.99996	1.1177	0.16058	0.83035	0.98621	0.99242	-0.01098	0.022714	0.057509	0.87806	-0.1876	0.8943	0.94574	1.0078	0.011253	0.23528	0.67693	1.1569	0.21022	0.13335	0.88792	1.1163	0.15866	0.000711	0.07528	1.5666	0.64767
564.5545	6.37	olomics Pc	0.019419	0.99951	1.4222	0.50809	0.55076	0.99998	0.92841	-0.10717	0.43227	0.99941	1.1914	0.25268	0.26821	0.74055	0.80143	-0.31934	1.78E-05	0.000456	0.10206	-3.2926	0.015995	0.093702	0.46209	-1.1138	0.17351	0.74875	0.7874	-0.34482	0.18742	0.98726	0.84451	-0.24382	0.01157	0.2688	1.3999	0.48535
352.975	12	olomics Ne	0.019709	0.99918	1.2416	0.31226	0.73864	0.95512	0.98654	-0.01955	0.1579	0.99995	1.0717	0.099871	0.39463	0.99985	1.0854	0.11826	0.078421	0.99927	1.0763	0.10612	0.24994	0.68677	1.1026	0.14097	0.26163	0.64083	1.1629	0.21773	0.32858	0.99512	1.1737	0.23101	0.46632	0.99941	1.0696	0.097094
67.0556	6.39	olomics Pc	0.019961	0.99951	1.311	0.39064	0.79174	0.99998	0.97251	-0.04022	0.98797	0.99941	0.99374	-0.00906	0.16937	0.58307	0.84144	-0.24906	3.87E-05	0.000758	0.30628	-1.7071	0.003593	0.051446	0.65611	-0.60799	0.015632	0.29145	0.71368	-0.48665	0.19294	0.99297	0.87036	-0.20032	0.95871	0.98209	1.0012	0.00179
237.2202	2.76	lomics Pos	0.02009	0.94464	1.491	0.5763	0.091557	0.99994	0.84039	-0.25086	0.14935	0.99996	1.0584	0.081872	0.002325	0.17581	0.78866	-0.34253	0.000228	0.001839	0.38321	-1.3838	0.026108	0.14262	0.70741	-0.49938	0.12475	0.58373	0.83586	-0.25866	0.46426	0.96776	0.93868	-0.09129	0.007288	0.091944	1.5997	0.67784
291.9625	10.13	lomics Pos	0.020248	0.94464	0.87053	-0.20004	0.016828	0.99994	0.75325	-0.4088	0.043924	0.99996	0.86039	-0.21694	0.004951	0.17581	0.77155	-0.37416	3.69E-05	0.000527	0.64379	-0.63534	0.10159	0.33938	0.82006	-0.28619	0.01999	0.33809	0.85024	-0.23405	0.057604	0.71686	0.84258	-0.24711	0.32587	0.43613	1.055	0.077282
623.5285	2.05	lomics Pos	0.020523	0.94464	1.375	0.4594	0.33801	0.99994	0.87479	-0.19298	0.78845	0.99996	0.97506	-0.03644	0.10231	0.52628	0.67302	-0.57127	4.15E-06	0.000147	0.10217	-3.2909	0.0024	0.061655	0.41205	-1.2791	0.09255	0.52195	0.69824	-0.51821	0.62464	0.97813	0.94774	-0.07743	0.003339	0.085302	1.5524	0.63451
137.1331	2.05	lomics Pos	0.020532	0.94464	1.2843	0.36102	0.50631	0.99994	0.9314	-0.10253	0.70697	0.99996	1.0327	0.046445	0.13406	0.55796	0.78567	-0.34801	2.23E-06	0.000144	0.16136	-2.6317	0.002795	0.061655	0.53948	-0.89035	0.312	0.72948	0.85932	-0.21873	0.72124	0.97988	0.96306	-0.0543	0.006501	0.090084	1.4385	0.52459
275.2742	7.02	olomics Pc	0.020605	0.99951	1.2985	0.3769	0.34466	0.99998	0.89271	-0.16374	0.76994	0.99941	1.0314	0.044643	0.22703	0.67593	0.79499	-0.331	7.52E-06	0.000283	0.15677	-2.6733	0.004337	0.055537	0.52165	-0.93884	0.021704	0.31651	0.70957	-0.49498	0.27417	0.99956	0.90048	-0.15123	0.005994	0.2688	1.3841	0.46898
382.2028	2.05	lomics Pos	0.020679	0.94464	1.31	0.38956	0.50662	0.99994	0.95174	-0.07136	0.71167	0.99996	0.97137	-0.04191	0.29714	0.75547	0.84396	-0.24476	0.000961	0.005421	0.41406	-1.2721	0.00837	0.084185	0.65525	-0.60989	0.74706	0.93404	0.97341	-0.03888	0.8751	0.98023	0.99007	-0.0144	0.001822	0.077476	1.3802	0.4649
215.6598	2.76	lomics Pos	0.021055	0.94464	1.3291	0.41047	0.36695	0.99994	0.90284	-0.14746	0.60813	0.99996	1.0619	0.086681	0.22445	0.68031	0.81352	-0.29775	2.87E-05	0.000459	0.15645	-2.6763	0.011846	0.092554	0.54603	-0.87296	0.24287	0.67954	0.85615	-0.22407	0.42193	0.96776	1.0575	0.080706	0.000692	0.07528	1.6482	0.72092
150.137	6.38	olomics Pc	0.021337	0.99951	1.2399	0.3102	0.32397	0.99998	0.89325	-0.16286	0.39638	0.99941	1.0724	0.10084	0.12374	0.48485	0.8046	-0.31366	4.4E-05	0.000845	0.26055	-1.9404	0.004172	0.054724	0.57099	-0.80847	0.044867	0.4245	0.76972	-0.3776	0.082485	0.78717	0.86204	-0.21418	0.7845	0.90661	1.0216	0.03081
210.1858	1.67	lomics Pos	0.02138	0.94464	1.3351	0.41698	0.62455	0.99994	0.94935	-0.07499	0.17866	0.99996	1.1459	0.19648	0.20432	0.6514	0.81213	-0.30021	3.11E-06	0.000144	0.16879	-2.5667	0.01674	0.10983	0.56717	-0.81816	0.25704	0.6916	0.83267	-0.26418	0.86694	0.98023	1.018	0.025698	0.001829	0.077476	1.4552	0.54117
205.1956	2.05	lomics Pos	0.021578	0.94464	1.3234	0.40422	0.40717	0.99994	0.90509	-0.14386	0.94697	0.99996	1.0122	0.017475	0.13602	0.55962	0.77716	-0.36372	1.31E-05	0.000277	0.15711	-2.6701	0.014134	0.10055	0.54113	-0.88594	0.29884	0.72384	0.85953	-0.21838	0.62668	0.97813	0.94766	-0.07755	0.026591	0.11491	1.2989	0.37732
276.2769	2.76	lomics Pos	0.021669	0.94464	1.3809	0.46563	0.34437	0.99994	0.85779	-0.22131	0.99225	0.99996	0.99805	-0.00282	0.13159	0.55569	0.737	-0.44027	2.67E-05	0.000439	0.13214	-2.9198	0.005552	0.075454	0.44121	-1.1804	0.069974	0.47049	0.73911	-0.43613	0.3175	0.9524	0.86943	-0.20185	0.013917	0.098791	1.5618	0.64319
167.0139	12.3	olomics Pc	0.021755	0.99951	0.87728	-0.1889	0.68883	0.99998	0.97842	-0.03148	0.019736	0.99941	0.87486	-0.19288	0.74028	0.99905	0.98249	-0.02548	0.69316	0.99688	0.97327	-0.03908	0.65072	0.8918	1.0483	0.068107	0.936	0.9969	1.0003	0.000488	0.20691	0.99956	0.94757	-0.0777	0.005805	0.2688	0.84449	-0.24385
626.528	2.05	lomics Pos	0.021799	0.94464	1.4097	0.49536	0.62138	0.99994	0.92479	-0.1128	0.1078	0.99996	1.2448	0.31587	0.19152	0.63355	0.76399	-0.38838	0.000112	0.001117	0.15762	-2.6655	0.014596	0.10138	0.54106	-0.88614	0.56113	0.855	0.90745	-0.14011	0.51963	0.96776	1.1293	0.17543	0.004053	0.085451	1.6825	0.75059
83.0864	2.05	lomics Pos	0.021994	0.94464	1.2877	0.36477	0.65646	0.99994	0.96188	-0.05607	0.29127	0.99996	1.1142	0.15607	0.1151	0.53523	0.79724	-0.32692	7.88E-07	0.000144	0.17996	-2.4743	0.012722	0.095104	0.56028	-0.83577	0.31695	0.72948	0.86323	-0.21218	0.8127	0.97988	1.0256	0.036408	0.001106	0.07528	1.3968	0.4821
384.2042	6.38	olomics Pc	0.022031	0.99951	1.2617	0.33532	0.55841	0.99998	0.95818	-0.06163	0.38071	0.99941	1.0937	0.12928	0.82431	0.99905	0.98654	-0.01956	5.78E-06	0.000265	0.38975	-1.3594	0.10949	0.31845	0.78113	-0.35637	0.50927	0.99391	0.94684	-0.0788	0.78976	0.99956	1.0321	0.045629	0.037927	0.28045	1.2246	0.29229
310.3116	7.02	olomics Pc	0.022092	0.99951	1.3032	0.38201	0.34396	0.99998	0.89896	-0.15367	0.88755	0.99941	1.0187	0.026743	0.20484	0.64204	0.79689	-0.32755	6E-06	0.000265	0.17972	-2.4761	0.003911	0.053802	0.53343	-0.90662	0.032258	0.35567	0.72439	-0.46516	0.35339	0.99956	0.91532	-0.12765	0.003992	0.2688	1.3713	0.45558
1045.511	6	lomics Pos	0.02211	0.94464	0.75305	-0.40918	0.064069	0.99994	0.79402	-0.33275	0.16726	0.99996	0.8577	-0.22146	0.10476	0.52894	0.81241	-0.29972	0.009751	0.030216	0.62984	-0.66695	0.17971	0.4675	0.81728	-0.29109	0.88306	0.97532	1.0139	0.01985	0.33839	0.96524	0.889	-0.16974	0.095463	0.19697	1.1541	0.20673
226.2167	2.76	lomics Pos	0.022254	0.94464	1.33	0.41139	0.16457	0.99994	0.8115	-0.30134	0.99763	0.99996	0.99504	-0.00717	0.064278	0.43456	0.71238	-0.48927	8.97E-06	0.000208	0.16656	-2.5859	0.002488	0.061655	0.4826	-1.0511	0.10207	0.54475	0.72451	-0.46493	0.49077	0.96776	0.90153	-0.14955	0.007399	0.091944	1.4989	0.58393
161.1335	6	olomics Pc	0.022279	0.99951	1.1885	0.24916	0.15398	0.99998	0.87117	-0.19898	0.96654	0.99941	1.0087	0.012525	0.061173	0.32568	0.74488	-0.42491	2.61E-07	0.000231	0.23255	-2.1044	0.003864	0.053776	0.55975	-0.83713	0.12635	0.66668	0.78256	-0.35373	0.17677	0.96992	0.84854	-0.23695	0.70249	0.86484	1.0278	0.039505
133.1016	1.67	lomics Pos	0.022372	0.94464	1.2331	0.30233	0.37373	0.99994	0.91454	-0.12888	0.56761	0.99996	1.0761	0.10579	0.13905	0.56536	0.79688	-0.32757	1.05E-06	0.000144	0.21671	-2.2061	0.011448	0.091708	0.58123	-0.78282	0.11845	0.57241	0.76643	-0.38377	0.19708	0.92582	0.88542	-0.17557	0.004014	0.085451	1.4182	0.50406
423.8845	10.08	lomics Pos	0.023058	0.94464	0.86351	-0.21172	0.036145	0.99994	0.82087	-0.28478	0.045506	0.99996	0.85523	-0.22562	0.030181	0.30328	0.82783	-0.2726	0.000409	0.002807	0.61746	-0.69558	0.049638	0.21687	0.7712	-0.37483	0.21369	0.65483	0.90127	-0.14996	0.050795	0.68198	0.82615	-0.27553	0.40075	0.50851	1.0778	0.10802
257.2673	1.91	lomics Pos	0.023134	0.94464	1.253	0.32543	0.24841	0.99994	0.88642	-0.17394	0.39424	0.99996	1.0791	0.10983	0.11204	0.53238	0.75674	-0.40213	7.89E-06	0.000198	0.15591	-2.6813	0.005813	0.076369	0.52162	-0.93892	0.15399	0.62466	0.81662	-0.29226	0.58353	0.97073	0.9539	-0.06809	0.000719	0.07528	1.4576	0.54363
785.9561	3.27	olomics Pc	0.023158	0.99951	1.4498	0.53588	0.47054	0.99998	0.92414	-0.11381	0.50903	0.99941	1.0854	0.1182	0.009291	0.19702	0.62029	-0.68898	0.012252	0.056841	0.57549	-0.79714	0.005521	0.06394	0.69391	-0.52718	0.81143	0.99391	0.99923	-0.00111	0.98085	0.99956	1.0488	0.068728	0.002614	0.2688	1.9297	0.94839
258.2707	6.2	olomics Pc	0.023266	0.99951	1.3308	0.41227	0.70074	0.99998	0.96613	-0.04971	0.4426	0.99941	1.1078	0.14774	0.25099	0.71739	0.83423	-0.26149	8.57E-06	0.000292	0.18665	-2.4216	0.023679	0.11397	0.59182	-0.75677	0.15829	0.73598	0.8198	-0.28666	0.66121	0.99956	0.96819	-0.04664	0.031125	0.2688	1.193	0.25459
311.3148	7.02	olomics Pc	0.023417	0.99951	1.2897	0.36699	0.29864	0.99998	0.88997	-0.16817	0.82148	0.99941	1.0285	0.04053	0.21254	0.65307	0.80238	-0.31764	7.59E-06	0.000283	0.17807	-2.4895	0.004001	0.054326	0.52651	-0.92546	0.043133	0.41783	0.73556	-0.44309	0.34571	0.99956	0.91464	-0.12873	0.003852	0.2688	1.3682	0.45229
876.9744	12.03	olomics Ne	0.023737	0.99918	1.3594	0.44298	0.53102	0.86583	1.0624	0.08731	0.70196	0.99995	1.1138	0.15555	0.084188	0.99985	1.2575	0.33054	0.020546	0.91567	1.4602	0.54619	0.4862	0.84895	1.0951	0.13104	0.77479	0.93803	1.057	0.079924	0.97453	0.99512	0.9896	-0.01508	0.86083	0.99941	1.0163	0.023264
873.0087	3.36	olomics Pc	0.023843	0.99951	1.8222	0.8657	0.9436	0.99998	0.99579	-0.00609	0.83885	0.99941	0.97734	-0.03306	0.023693	0.22481	0.68361	-0.54875	0.008363	0.042191	0.62542	-0.67711	0.003048	0.049681	0.66485	-0.5889	0.5515	0.99391	1.123	0.16739	0.69747	0.99956	0.97853	-0.03131	0.014599	0.2688	2.1232	1.0863
109.1019	2.05	lomics Pos	0.024012	0.94464	1.2993	0.37777	0.35974	0.99994	0.91933	-0.12135	0.2865	0.99996	1.1027	0.14099	0.23721	0.69576	0.81976	-0.28673	4.54E-07	0.000144	0.17579	-2.508	0.004247	0.071311	0.55344	-0.85349	0.23497	0.67693	0.85523	-0.22561	0.73947	0.97988	0.97147	-0.04175	0.001092	0.07528	1.4809	0.56643
523.6365	3.27	olomics Pc	0.024144	0.99951	1.6414	0.71489	0.56119	0.99998	0.93521	-0.09663	0.52619	0.99941	1.0884	0.12215	0.037591	0.2649	0.68945	-0.53649	0.024537	0.098779	0.64267	-0.63786	0.0199	0.1043	0.64307	-0.63696	0.63617	0.99391	0.94739	-0.07797	0.99194	0.99956	1.0768	0.10681	0.016517	0.2688	1.8043	0.85147
228.2326	1.34	lomics Pos	0.02421	0.94464	1.275	0.3505	0.42307	0.99994	0.92241	-0.11652	0.3708	0.99996	1.0961	0.13241	0.10939	0.53208	0.77615	-0.36558	1.5E-06	0.000144	0.20523	-2.2847	0.002103	0.060099	0.55498	-0.84949	0.12075	0.57768	0.80821	-0.30721	0.65471	0.97988	0.95893	-0.0605	0.008716	0.092495	1.3634	0.4472
294.2882	7.02	olomics Pc	0.024704	0.99951	1.2833	0.35981	0.38847	0.99998	0.89431	-0.16115	0.88107	0.99941	1.0222	0.0317	0.2448	0.70876	0.80255	-0.31734	3.28E-05	0.000672	0.16032	-2.641	0.004242	0.05479	0.49935	-1.0019	0.043776	0.42041	0.71401	-0.48599	0.4471	0.99956	0.91692	-0.12513	0.000937	0.2688	1.3539	0.4371
192.6464	6	olomics Pc	0.025098	0.99951	1.3219	0.40258	0.38287	0.99998	0.91364	-0.1303	0.74099	0.99941	1.0494	0.06954	0.43332	0.91715	0.90495	-0.1441	1E-04	0.001599	0.38046	-1.3942	0.016237	0.094365	0.69275	-0.52959	0.68873	0.99391	0.95934	-0.05989	0.94829	0.99956	1.0038	0.005531	0.57118	0.7927	1.0592	0.082934
637.475	2.05	lomics Pos	0.025122	0.94464	1.3501	0.43311	0.33566	0.99994	0.87232	-0.19708	0.33255	0.99996	1.1131	0.15464	0.12805	0.55024	0.72655	-0.46087	4.09E-06	0.000146	0.11584	-3.1098	0.017263	0.11252	0.52054	-0.94193	0.19106	0.64891	0.75482	-0.40579	0.73259	0.97988	0.96257	-0.05503	0.010709	0.094875	1.4316	0.51765
368.2439	2.68	lomics Pos	0.025258	0.94464	1.3569	0.44032	0.15894	0.99994	0.83946	-0.25247	0.79817	0.99996	0.97019	-0.04367	0.008592	0.19971	0.66329	-0.5923	9.25E-05	0.000974	0.26648	-1.9079	0.001192	0.043885	0.51154	-0.96709	0.13747	0.60294	0.76398	-0.38839	0.38807	0.96776	0.88599	-0.17465	0.060033	0.15754	1.3407	0.42303
121.0329	4.38	olomics Pc	0.025647	0.99951	0.77323	-0.37103	0.041919	0.99998	0.75866	-0.39847	0.31556	0.99941	0.88411	-0.1777	0.23575	0.69534	0.8722	-0.19727	0.059844	0.19177	0.82684	-0.27431	0.014591	0.091119	0.67361	-0.57001	0.15016	0.72477	0.79686	-0.3276	0.022145	0.51556	0.735	-0.44417	0.009765	0.2688	0.69721	-0.52033
301.2534	2.06	lomics Pos	0.025731	0.94464	1.3122	0.392	0.47051	0.99994	0.92222	-0.11681	0.29496	0.99996	1.1057	0.14494	0.22246	0.67695	0.8146	-0.29584	4.61E-06	0.000149	0.11854	-3.0765	0.014581	0.10138	0.55455	-0.85061	0.34253	0.74562	0.86663	-0.20652	0.7195	0.97988	1.0364	0.051597	0.003483	0.085302	1.4867	0.5721
470.9714	5.34	olomics Ne	0.026138	0.99918	1.0808	0.11215	0.21406	0.63496	0.95445	-0.06726	0.61436	0.99995	1.0151	0.021673	0.80935	0.99985	1.0317	0.044957	0.8608	0.99927	0.99367	-0.00916	0.43791	0.82254	1.0606	0.084832	0.027911	0.33786	1.1374	0.18569	0.9956	0.99885	1.0067	0.009572	0.45703	0.99941	1.0294	0.041833
332.2928	2.75	lomics Pos	0.026145	0.94464	1.36	0.4436	0.29654	0.99994	0.83597	-0.25848	0.64102	0.99996	0.92966	-0.10522	0.067079	0.43947	0.63556	-0.6539	3.06E-06	0.000144	0.10287	-3.2811	0.00048	0.033979	0.36433	-1.4567	0.051463	0.41162	0.63091	-0.66449	0.14057	0.89982	0.71584	-0.4823	0.058735	0.15622	1.3259	0.40693
170.1535	2.76	lomics Pos	0.026206	0.94464	1.2608	0.33438	0.090617	0.99994	0.79061	-0.33895	0.45657	0.99996	0.92042	-0.11963	0.037945	0.34165	0.69174	-0.5317	5.12E-06	0.000159	0.15496	-2.69	0.001728	0.05445	0.47926	-1.0611	0.11983	0.5769	0.7667	-0.38327	0.90079	0.98023	0.99156	-0.01223	0.004636	0.085788	1.4907	0.57594
240.2327	2.05	lomics Pos	0.026466	0.94464	1.2871	0.36416	0.54104	0.99994	0.94059	-0.08836	0.61789	0.99996	1.0521	0.073271	0.13645	0.56004	0.77146	-0.37433	1.04E-06	0.000144	0.17137	-2.5448	0.010353	0.08787	0.55221	-0.85672	0.19809	0.64891	0.82592	-0.27592	0.68155	0.97988	0.96074	-0.05778	0.002746	0.083394	1.4239	0.50983
109.1024	6.01	olomics Pc	0.026768	0.99951	1.1826	0.24192	0.77109	0.99998	0.97768	-0.03257	0.9222	0.99941	0.99363	-0.00922	0.050619	0.29486	0.73734	-0.4396	2.56E-05	0.000568	0.26085	-1.9387	0.002195	0.045856	0.57065	-0.80933	0.059491	0.49576	0.75615	-0.40325	0.1896	0.98781	0.8415	-0.24896	0.56196	0.79019	1.0472	0.066593
366.2321	2.06	lomics Pos	0.026964	0.94464	1.2463	0.31768	0.66741	0.99994	0.97529	-0.0361	0.15018	0.99996	1.1134	0.15493	0.31775	0.78167	0.89872	-0.15405	7.15E-06	0.000186	0.2716	-1.8804	0.016511	0.10925	0.7119	-0.49024	0.73554	0.92926	0.97592	-0.03517	0.11965	0.88537	1.1407	0.18986	0.000179	0.07528	1.473	0.55877
743.4086	3.22	olomics Pc	0.027178	0.99951	1.487	0.5724	0.098918	0.99998	0.85398	-0.22772	0.54557	0.99941	1.0617	0.086327	0.00711	0.1948	0.61898	-0.69204	0.000663	0.006805	0.57484	-0.79876	0.009992	0.079508	0.70826	-0.49764	0.79525	0.99391	1.0838	0.1161	0.99272	0.99956	1.0587	0.082295	0.020457	0.2688	1.7974	0.84595
202.6521	6.9	olomics Pc	0.027225	0.99951	1.3497	0.43268	0.21547	0.99998	0.89938	-0.153	0.19422	0.99941	1.1193	0.16262	0.32948	0.82464	0.85812	-0.22075	1.59E-06	0.000231	0.17121	-2.5462	0.005309	0.062631	0.52302	-0.93505	0.056867	0.47768	0.79986	-0.32218	0.25346	0.99956	1.0985	0.13554	0.000947	0.2688	1.5356	0.61884
212.2013	7.02	olomics Pc	0.027231	0.99951	1.3633	0.44711	0.61444	0.99998	0.92488	-0.11267	0.74888	0.99941	1.0404	0.057128	0.31947	0.81415	0.81418	-0.29659	2.13E-05	0.0005	0.18296	-2.4504	0.009034	0.076393	0.50954	-0.97273	0.046142	0.43129	0.70747	-0.49926	0.63383	0.99956	0.9314	-0.10253	0.02015	0.2688	1.4275	0.51348
760.8226	10.05	lomics Pos	0.027269	0.94464	1.0644	0.090057	0.26754	0.99994	0.96745	-0.04775	0.19667	0.99996	0.94568	-0.08058	0.034337	0.32488	0.92056	-0.11942	0.000277	0.002091	0.79028	-0.33957	0.27519	0.56257	0.8947	-0.16052	0.98929	0.99646	1.0041	0.005853	0.86063	0.98023	0.99394	-0.00877	0.000734	0.091944	1.1729	0.23004
550.9771	5.25	olomics Ne	0.027366	0.99918	1.0962	0.13245	0.33253	0.74862	0.95737	-0.06285	0.055524	0.99995	1.1092	0.1495	0.31004	0.99985	1.0702	0.097869	0.1706	0.99927	1.0821	0.11378	0.032333	0.47167	1.1548	0.20761	0.03559	0.35618	1.1236	0.16808	0.016753	0.99512	1.1142	0.15607	0.38582	0.99941	1.0608	0.085164
787.43	0.56	lomics Pos	0.027533	0.94464	1.4115	0.4972	0.15117	0.99994	0.90711	-0.14064	0.58868	0.99996	0.97595	-0.03513	0.002921	0.17581	0.55458	-0.85053	4.23E-05	0.000562	0.40311	-1.3108	0.000196	0.026314	0.56121	-0.8334	0.29938	0.72384	0.85241	-0.23038	0.6232	0.97813	0.96368	-0.05338	0.01065	0.094875	2.149	1.1036
919.507	0.55	lomics Pos	0.027643	0.94464	1.4404	0.52644	0.32847	0.99994	0.93013	-0.10449	0.67291	0.99996	0.96929	-0.045	0.003375	0.17581	0.54039	-0.88793	0.000676	0.004089	0.40428	-1.3066	0.000871	0.042199	0.532	-0.91051	0.47601	0.80869	0.91359	-0.13039	0.54777	0.96776	0.94125	-0.08736	0.030281	0.11973	2.1698	1.1176
89.0238	11.7	olomics Ne	0.027656	0.99918	1.2804	0.35654	0.33317	0.74862	1.1149	0.15686	0.33917	0.99995	1.1235	0.16798	0.23411	0.99985	1.123	0.16738	0.44874	0.99927	1.1192	0.16241	0.09824	0.55533	1.1926	0.25412	0.74702	0.92498	1.0134	0.019234	0.055842	0.99512	1.2679	0.34246	0.14261	0.99941	1.3028	0.38159
658.9729	12.01	olomics Ne	0.028147	0.99918	1.2675	0.34197	0.46197	0.82998	0.94929	-0.07508	0.1815	0.99995	1.1153	0.15739	0.74405	0.99985	1.0386	0.054674	0.19968	0.99927	1.2092	0.27402	0.29135	0.71628	1.0992	0.13639	0.028459	0.34013	1.2105	0.27556	0.293	0.99512	1.2181	0.28465	0.59847	0.99941	1.0374	0.053028
1118.743	10.07	lomics Pos	0.02822	0.94464	0.85379	-0.22804	0.004795	0.99994	0.82723	-0.27364	0.035021	0.99996	0.84201	-0.24809	0.4974	0.89009	0.93708	-0.09375	0.005966	0.020793	0.7813	-0.35605	0.576	0.76847	0.94482	-0.08189	0.60268	0.8673	0.96923	-0.04509	0.18773	0.92582	0.89764	-0.15579	0.011274	0.094982	1.1703	0.22691
837.6166	5.34	lomics Pos	0.028222	0.94464	1.0986	0.13562	0.90617	0.99994	1.0073	0.010486	0.77155	0.99996	0.9915	-0.01231	0.11837	0.53907	0.94473	-0.08203	0.000645	0.003961	0.77472	-0.36826	0.088236	0.31297	0.84809	-0.23771	0.3931	0.76945	0.93331	-0.09957	0.73474	0.97988	0.97904	-0.03056	0.043307	0.13853	1.2471	0.31858
809.4463	3.3	olomics Pc	0.028245	0.99951	1.5677	0.64863	0.092264	0.99998	0.83341	-0.2629	0.8989	0.99941	0.98939	-0.01539	0.000631	0.12797	0.56265	-0.8297	0.002639	0.018589	0.55416	-0.85162	0.001192	0.044437	0.59736	-0.74333	0.59442	0.99391	0.94478	-0.08196	0.47693	0.99956	0.91011	-0.13588	0.02468	0.2688	1.7611	0.81649
297.2972	2.41	lomics Pos	0.028492	0.94464	1.2042	0.26812	0.33777	0.99994	0.92586	-0.11113	0.25309	0.99996	1.1083	0.14832	0.15685	0.58521	0.81121	-0.30186	6.12E-05	0.00074	0.18626	-2.4246	0.00204	0.059378	0.4954	-1.0133	0.095765	0.52998	0.76559	-0.38535	0.2847	0.94397	0.89343	-0.16257	0.0041	0.085451	1.4852	0.57065
242.2481	1.58	lomics Pos	0.028581	0.94464	1.2667	0.34111	0.41226	0.99994	0.9116	-0.13352	0.49799	0.99996	1.0926	0.12783	0.13591	0.55962	0.76612	-0.38436	0.000161	0.001434	0.19684	-2.3449	0.003007	0.063179	0.54304	-0.88088	0.21833	0.6607	0.83547	-0.25934	0.86697	0.98023	0.98819	-0.01714	0.006453	0.090084	1.4013	0.48673
149.1331	2.05	lomics Pos	0.028609	0.94464	1.2396	0.30986	0.33673	0.99994	0.90301	-0.14719	0.62721	0.99996	1.0475	0.066966	0.11277	0.53238	0.77072	-0.37573	4.47E-06	0.000149	0.17433	-2.5201	0.00596	0.077041	0.55374	-0.85273	0.19183	0.64891	0.8206	-0.28525	0.63373	0.97988	0.95573	-0.06532	0.002766	0.083394	1.3903	0.47542
202.6475	2.07	lomics Pos	0.029171	0.94464	1.2848	0.36159	0.019576	0.99994	0.90285	-0.14744	0.26086	0.99996	1.1016	0.1396	0.17656	0.60979	0.81056	-0.303	2.93E-05	0.000466	0.29844	-1.7445	0.084927	0.30406	0.76994	-0.37718	0.77978	0.94691	0.98329	-0.02431	0.92222	0.9846	1.0242	0.034493	0.005707	0.088649	1.431	0.51697
171.1561	1.66	lomics Pos	0.029327	0.94464	1.2936	0.37138	0.70381	0.99994	0.95787	-0.06209	0.38789	0.99996	1.1055	0.14466	0.18329	0.62384	0.86411	-0.21072	4.56E-06	0.000149	0.26875	-1.8956	0.001007	0.043236	0.62996	-0.66666	0.80467	0.95494	0.97422	-0.03769	0.78161	0.97988	0.97138	-0.04189	0.007885	0.092077	1.3812	0.46597
763.9427	3.25	olomics Pc	0.029357	0.99951	1.5807	0.66057	0.15841	0.99998	0.85583	-0.2246	0.39125	0.99941	1.0994	0.13666	0.001049	0.12936	0.67578	-0.56538	0.000353	0.004417	0.57722	-0.79281	0.006069	0.065713	0.72536	-0.46324	0.77931	0.99391	1.005	0.007256	0.86638	0.99956	1.0562	0.078934	0.048866	0.289	1.8762	0.90785
142.1233	1.66	lomics Pos	0.029367	0.94464	1.2806	0.35681	0.36524	0.99994	0.88797	-0.17141	0.73863	0.99996	1.0494	0.069514	0.15199	0.58011	0.7713	-0.37463	5.44E-06	0.000159	0.17506	-2.5141	0.014015	0.099884	0.55615	-0.84645	0.14129	0.60462	0.78133	-0.356	0.38053	0.96776	0.90165	-0.14936	0.005104	0.088007	1.4033	0.48886
147.118	6	olomics Pc	0.029566	0.99951	1.1842	0.24389	0.35185	0.99998	0.92463	-0.11305	0.93422	0.99941	0.99619	-0.00551	0.061038	0.32568	0.75813	-0.39948	2.27E-06	0.000231	0.24826	-2.0101	0.001416	0.045425	0.54394	-0.87848	0.097158	0.60726	0.81368	-0.29747	0.28403	0.99956	0.88717	-0.17272	0.18286	0.44651	1.0961	0.13237
655.9785	12.03	olomics Ne	0.029694	0.99918	1.3283	0.40955	0.17994	0.60009	0.84349	-0.24556	0.089512	0.99995	1.3255	0.40653	0.73325	0.99985	1.0974	0.13406	0.80127	0.99927	0.98463	-0.02235	0.96903	0.98999	1.0082	0.01176	0.37431	0.72793	1.1649	0.22017	0.5945	0.99512	0.93357	-0.09917	0.209	0.99941	1.2652	0.33935
256.2648	6.2	olomics Pc	0.029912	0.99951	1.2545	0.32715	0.51122	0.99998	0.94056	-0.0884	0.47875	0.99941	1.0907	0.12524	0.18988	0.6228	0.81742	-0.29084	2.42E-05	0.000541	0.21828	-2.1957	0.013251	0.085844	0.58485	-0.77385	0.11788	0.65162	0.81431	-0.29634	0.54859	0.99956	0.95874	-0.0608	0.01908	0.2688	1.2009	0.26413
589.3124	0.56	lomics Pos	0.030119	0.94464	1.4449	0.53099	0.04227	0.99994	0.86754	-0.20501	0.99657	0.99996	1.0011	0.001609	0.000338	0.164	0.5322	-0.90996	1.22E-05	0.000266	0.42043	-1.25	0.000328	0.028292	0.556	-0.84685	0.62881	0.88019	0.95601	-0.0649	0.65123	0.97988	0.98678	-0.0192	0.01667	0.099614	2.1679	1.1163
265.9313	2.67	olomics Ne	0.030287	0.99918	1.0541	0.076	0.30367	0.73566	0.93272	-0.10048	0.8245	0.99995	1.0112	0.016079	0.37903	0.99985	0.96905	-0.04535	0.48831	0.99927	0.97251	-0.04022	0.075568	0.52855	1.0737	0.10255	0.46153	0.78972	1.0626	0.087566	0.71439	0.99512	1.0157	0.022407	0.012611	0.99941	1.152	0.20409
873.8253	10.02	lomics Pos	0.030351	0.94464	1.2219	0.28914	0.2007	0.99994	1.1107	0.15143	0.9319	0.99996	1.0011	0.001626	0.67942	0.95843	0.96698	-0.04844	0.57979	0.64116	0.94075	-0.08812	0.69584	0.84391	0.96197	-0.05593	0.17777	0.64616	1.0982	0.13515	0.056397	0.70737	1.1965	0.25883	0.039173	0.13265	1.2778	0.35361
258.2703	1.91	lomics Pos	0.030403	0.94464	1.2292	0.29769	0.21056	0.99994	0.86766	-0.2048	0.63692	0.99996	1.0382	0.054151	0.081289	0.4813	0.71393	-0.48615	2.91E-06	0.000144	0.1486	-2.7505	0.003097	0.063487	0.49024	-1.0284	0.11567	0.5679	0.79206	-0.33632	0.2653	0.94365	0.91404	-0.12967	0.001025	0.07528	1.4059	0.49147
381.2035	2.05	lomics Pos	0.030577	0.94464	1.3633	0.44708	0.85525	0.99994	0.98126	-0.02729	0.92029	0.99996	1.0299	0.042476	0.41503	0.85524	0.90085	-0.15063	2.84E-05	0.000458	0.3123	-1.679	0.050393	0.21985	0.7254	-0.46315	0.76956	0.94358	0.97223	-0.04063	0.16764	0.91806	1.2894	0.36665	0.009272	0.092545	1.3759	0.46043
1048.751	10.07	lomics Pos	0.030608	0.94464	1.1007	0.13842	0.72916	0.99994	0.98437	-0.02273	0.73291	0.99996	0.98194	-0.02629	0.099887	0.51973	0.87573	-0.19144	0.00493	0.018075	0.79908	-0.3236	0.25427	0.54102	0.91263	-0.1319	0.67589	0.90042	1.0468	0.065987	0.68098	0.97988	1.0185	0.026384	0.026874	0.11548	1.1791	0.23767
181.1304	2.07	lomics Pos	0.031082	0.94464	1.128	0.17371	0.57448	0.99994	0.96565	-0.05043	0.70569	0.99996	1.0172	0.024586	0.15348	0.58077	0.8389	-0.25343	1.78E-06	0.000144	0.25517	-1.9705	0.006691	0.080671	0.66826	-0.58153	0.17299	0.64606	0.89854	-0.15434	0.9542	0.98838	1.0072	0.010335	0.000213	0.07528	1.3562	0.43952
481.2684	3.21	olomics Pc	0.031112	0.99951	1.3954	0.48072	0.037376	0.99998	0.85488	-0.22621	0.1371	0.99941	0.91599	-0.12659	0.001068	0.12936	0.61998	-0.68971	0.000827	0.008117	0.56274	-0.82946	0.000628	0.039708	0.64768	-0.62664	0.75112	0.99391	0.99671	-0.00476	0.57889	0.99956	0.9446	-0.08222	0.013696	0.2688	1.8489	0.88664
142.124	7.02	olomics Pc	0.031234	0.99951	1.2458	0.3171	0.34008	0.99998	0.89467	-0.16057	0.76684	0.99941	1.0599	0.083936	0.30358	0.79293	0.82185	-0.28305	1.54E-05	0.000416	0.17542	-2.5111	0.014741	0.091119	0.52337	-0.9341	0.046062	0.43129	0.72417	-0.4656	0.43098	0.99956	0.89832	-0.15469	0.019087	0.2688	1.3443	0.42691
655.3515	0.56	lomics Pos	0.03165	0.94464	1.4685	0.55435	0.15286	0.99994	0.89406	-0.16155	0.79434	0.99996	1.0151	0.021552	0.004199	0.17581	0.57266	-0.80424	0.000106	0.001074	0.4204	-1.2502	0.000757	0.04065	0.56296	-0.8289	0.37102	0.76056	0.8738	-0.19462	0.68901	0.97988	0.98338	-0.02418	0.010513	0.094875	2.2079	1.1427
265.2532	2.05	lomics Pos	0.031915	0.94464	1.2502	0.32218	0.28832	0.99994	0.8986	-0.15424	0.66372	0.99996	1.0382	0.054067	0.15179	0.57995	0.79049	-0.33919	2.92E-06	0.000144	0.17989	-2.4748	0.00605	0.077417	0.56634	-0.82027	0.1775	0.64616	0.82484	-0.27781	0.75044	0.97988	0.97233	-0.04049	0.002304	0.082646	1.4091	0.4948
312.3178	7.02	olomics Pc	0.031956	0.99951	1.3126	0.3924	0.32977	0.99998	0.88891	-0.16989	0.95509	0.99941	1.0073	0.010506	0.21589	0.65907	0.78786	-0.34398	3.25E-05	0.000669	0.15679	-2.6731	0.00588	0.065453	0.53182	-0.911	0.056385	0.47617	0.71804	-0.47786	0.26439	0.99956	0.89532	-0.15953	0.007664	0.2688	1.3609	0.44459
285.2889	2.05	lomics Pos	0.031977	0.94464	1.2315	0.30039	0.52577	0.99994	0.93938	-0.09022	0.99996	0.99996	1.0058	0.008317	0.10587	0.52975	0.7609	-0.39422	5.5E-06	0.000159	0.16653	-2.5861	0.006749	0.080671	0.55005	-0.86238	0.16486	0.63453	0.82235	-0.28217	0.51104	0.97988	0.93595	-0.09549	0.005463	0.08815	1.4098	0.49552
765.4211	3.25	olomics Pc	0.03198	0.99951	1.4781	0.56376	0.017407	0.99998	0.82949	-0.2697	0.87059	0.99941	1.0202	0.028905	0.007411	0.1948	0.63441	-0.6565	0.00047	0.00542	0.52658	-0.92527	0.000703	0.041543	0.6295	-0.66772	0.73395	0.99391	0.99956	-0.00063	0.71134	0.99956	0.9798	-0.02944	0.043023	0.28452	1.8133	0.85861
83.0864	2.76	lomics Pos	0.032045	0.94464	1.4369	0.52292	0.70636	0.99994	0.95276	-0.06982	0.51334	0.99996	1.0687	0.095903	0.27221	0.7325	0.79554	-0.32999	1.76E-05	0.000338	0.23746	-2.0742	0.001689	0.053682	0.5462	-0.8725	0.75293	0.93668	0.96496	-0.05146	0.42284	0.96776	0.88241	-0.18048	0.010463	0.094875	1.5438	0.62651
139.1124	2.05	lomics Pos	0.032058	0.94464	1.2953	0.37324	0.33086	0.99994	0.89799	-0.15523	0.28802	0.99996	1.1042	0.14302	0.17441	0.60919	0.79948	-0.32287	2.9E-06	0.000144	0.18616	-2.4254	0.017465	0.11287	0.57765	-0.79173	0.3774	0.76056	0.88416	-0.17763	0.99292	0.99721	0.99659	-0.00493	0.002666	0.082646	1.4583	0.54429
143.1255	1.66	lomics Pos	0.032151	0.94464	1.2656	0.33983	0.9857	0.99994	1.0012	0.001745	0.9742	0.99996	1.0039	0.005622	0.039329	0.34395	0.67899	-0.55854	2.83E-05	0.000458	0.23042	-2.1177	0.00194	0.057382	0.60238	-0.73125	0.39472	0.76955	0.87882	-0.18636	0.10156	0.8541	0.83195	-0.26544	0.041212	0.13535	1.3519	0.43498
198.1863	5.99	olomics Pc	0.032248	0.99951	1.2124	0.27789	0.35983	0.99998	0.92117	-0.11846	0.48181	0.99941	1.0677	0.094497	0.16544	0.57458	0.80854	-0.30662	4.38E-06	0.000232	0.21314	-2.2301	0.014194	0.08942	0.59363	-0.75235	0.23324	0.83874	0.84759	-0.23857	0.4206	0.99956	0.93459	-0.09759	0.012757	0.2688	1.1658	0.22132
69.0706	2.05	lomics Pos	0.032559	0.94464	1.3327	0.41439	0.15691	0.99994	0.87467	-0.19319	0.84605	0.99996	0.98916	-0.01573	0.12029	0.54117	0.74765	-0.41956	7.85E-07	0.000144	0.15402	-2.6988	0.013244	0.097489	0.5514	-0.85883	0.14305	0.60716	0.778	-0.36215	0.3241	0.95459	0.89962	-0.15261	0.000539	0.07528	1.4113	0.49706
270.2796	2.27	lomics Pos	0.032562	0.94464	1.2825	0.35898	0.2623	0.99994	0.87569	-0.19151	0.54491	0.99996	1.0721	0.10049	0.069326	0.44879	0.71654	-0.48087	2.63E-06	0.000144	0.13869	-2.8501	0.01381	0.099356	0.49843	-1.0045	0.12153	0.57924	0.75973	-0.39644	0.62938	0.97988	0.94837	-0.07648	0.003801	0.085302	1.4622	0.54818
343.9833	4.35	olomics Ne	0.032722	0.99918	1.0505	0.071112	0.1197	0.5367	0.90015	-0.15177	0.75928	0.99995	0.99386	-0.00889	0.93236	0.99985	1.0015	0.002194	0.45882	0.99927	0.97006	-0.04386	0.53624	0.87267	1.0437	0.061673	0.14922	0.53641	1.0591	0.082859	0.68926	0.99512	1.0275	0.039093	0.20279	0.99941	1.0624	0.087388
439.2494	3.04	olomics Pc	0.032984	0.99951	1.6451	0.71814	0.42573	0.99998	0.91457	-0.12883	0.44183	0.99941	1.0854	0.11824	0.011636	0.19869	0.70822	-0.49773	0.005945	0.033321	0.61393	-0.70386	0.091611	0.28126	0.81253	-0.29951	0.81588	0.99391	1.1015	0.13948	0.74861	0.99956	1.1968	0.25914	0.025306	0.2688	2.2231	1.1526
570.3135	2.65	olomics Pc	0.033139	0.99951	1.6004	0.67846	0.19744	0.99998	0.90286	-0.14743	0.69616	0.99941	0.97118	-0.0422	0.13203	0.5064	0.73085	-0.45235	0.050231	0.16959	0.64418	-0.63447	0.001239	0.04486	0.64744	-0.62718	0.72286	0.99391	1.004	0.005697	0.39177	0.99956	1.3217	0.40242	0.012422	0.2688	2.4176	1.2736
219.211	2.07	lomics Pos	0.033196	0.94464	1.1471	0.19796	0.45331	0.99994	0.93779	-0.09267	0.78517	0.99996	0.98626	-0.01996	0.001306	0.17581	0.62949	-0.66775	0.000115	0.001135	0.27969	-1.8381	0.000349	0.028292	0.42644	-1.2296	0.044907	0.39004	0.69014	-0.53503	0.26306	0.94365	0.84356	-0.24544	0.14403	0.24827	1.2254	0.29322
348.2372	1.66	lomics Pos	0.033223	0.94464	1.2534	0.32586	0.32096	0.99994	0.91508	-0.12803	0.99418	0.99996	1.0056	0.00809	0.32768	0.78974	0.90234	-0.14825	5.36E-06	0.000159	0.24299	-2.041	0.003221	0.064159	0.53965	-0.8899	0.24761	0.68479	0.87516	-0.19239	0.49155	0.96776	0.93086	-0.10337	0.01405	0.098791	1.3929	0.47811
240.2332	6.38	olomics Pc	0.033315	0.99951	1.2697	0.34447	0.50506	0.99998	0.93744	-0.0932	0.59809	0.99941	1.0633	0.088541	0.24354	0.70641	0.84066	-0.2504	1.8E-05	0.000457	0.20368	-2.2956	0.011709	0.083709	0.59977	-0.73752	0.25014	0.85803	0.85518	-0.2257	0.51857	0.99956	0.94825	-0.07665	0.08511	0.33554	1.1822	0.24143
466.9757	5.15	olomics Ne	0.033444	0.99918	1.0621	0.086965	0.18888	0.60867	0.93906	-0.09071	0.78041	0.99995	1.0127	0.018252	0.82343	0.99985	1.014	0.020054	0.30698	0.99927	1.0498	0.070136	0.48255	0.8481	1.044	0.062087	0.018591	0.29108	1.0778	0.10806	0.40579	0.99512	1.0427	0.060293	0.99257	0.99945	1.0016	0.002255
268.264	1.72	lomics Pos	0.033782	0.94464	1.3369	0.41886	0.31832	0.99994	0.8744	-0.19364	0.30055	0.99996	1.1054	0.14463	0.14958	0.57784	0.76703	-0.38264	5.11E-06	0.000159	0.17986	-2.475	0.012348	0.093415	0.61113	-0.71045	0.35506	0.75255	0.85273	-0.22984	0.81324	0.97988	0.96887	-0.04563	0.010533	0.094875	1.4159	0.50172
249.2547	11.77	olomics Pc	0.034181	0.99951	0.73671	-0.44084	0.51335	0.99998	0.9092	-0.13734	0.24544	0.99941	0.85811	-0.22076	0.46308	0.94005	0.90428	-0.14516	0.74618	0.99688	1.0679	0.094719	0.14411	0.38437	0.79634	-0.32855	0.1713	0.74875	0.82497	-0.27758	0.85403	0.99956	0.96959	-0.04455	0.10992	0.36416	0.8036	-0.31545
444.7141	0.32	olomics Pc	0.034233	0.99951	0.79711	-0.32716	0.052583	0.99998	0.81552	-0.29421	0.036141	0.99941	0.87082	-0.19956	0.10753	0.44163	0.86411	-0.21071	0.1856	0.46846	0.90632	-0.14191	0.009897	0.079508	0.83395	-0.26196	0.23972	0.84407	0.95875	-0.06078	0.030475	0.58535	0.86125	-0.2155	0.001123	0.2688	0.86712	-0.2057

612.8802	10.12	domics Pos	0.034822	0.94464	1.1855	0.2455	0.1951	0.99994	1.0949	0.13077	0.26916	0.99996	1.1142	0.15607	0.74441	0.97302	1.0391	0.055325	0.008958	0.028206	0.79177	-0.33684	0.46586	0.6901	1.0699	0.097411	0.15722	0.62971	1.2028	0.26641	0.17092	0.91806	1.1615	0.21602	0.070755	0.17195	1.3168	0.39701
247.2426	2.05	domics Pos	0.035189	0.94464	1.241	0.31148	0.29452	0.99994	0.89693	-0.15693	0.58604	0.99996	1.0465	0.065514	0.15418	0.58092	0.79522	-0.33057	3.93E-06	0.000145	0.17812	-2.4891	0.007101	0.081828	0.56377	-0.82681	0.20527	0.65061	0.83248	-0.26451	0.78644	0.97988	0.97557	-0.03568	0.002905	0.085194	1.3875	0.47252
167.1436	1.66	domics Pos	0.035295	0.94464	1.2106	0.2757	0.46378	0.99994	0.9295	-0.10548	0.49032	0.99996	1.0824	0.11418	0.10571	0.52975	0.76482	-0.38681	1.11E-06	0.000144	0.18548	-2.4306	0.003265	0.064159	0.54446	-0.8771	0.28196	0.7098	0.85379	-0.22804	0.76532	0.97988	0.97817	-0.03184	0.004747	0.085788	1.4158	0.50162
123.1175	2.05	domics Pos	0.035412	0.94464	1.266	0.3403	0.37496	0.99994	0.91025	-0.13567	0.58152	0.99996	1.0651	0.091055	0.21023	0.65763	0.80857	-0.30655	3.63E-06	0.000144	0.1755	-2.5104	0.009837	0.087714	0.56269	-0.82959	0.15626	0.6285	0.80306	-0.31642	0.73972	0.97988	0.9677	-0.04738	0.008645	0.092495	1.3775	0.46206
198.185	7.02	bolomics Po	0.035498	0.99951	1.3033	0.38218	0.51442	0.99998	0.9281	-0.10764	0.47118	0.99941	1.0728	0.10135	0.19096	0.62312	0.77842	-0.36138	1.66E-06	0.000231	0.19264	-2.376	0.000425	0.039572	0.52846	-0.92013	0.015762	0.29145	0.67883	-0.55887	0.35162	0.99956	0.93313	-0.09985	0.001679	0.2688	1.3722	0.45652
710.9085	3.19	bolomics Po	0.035555	0.99951	1.3659	0.44983	0.36143	0.99998	0.91953	-0.12103	0.82959	0.99941	1.0239	0.034062	0.009239	0.19702	0.70004	-0.51449	0.007006	0.037008	0.61031	-0.71238	0.007403	0.071093	0.66435	-0.58997	0.40286	0.97985	0.90327	-0.14677	0.66648	0.99956	0.97689	-0.03373	0.045681	0.28476	1.7786	0.83071
460.7876	9.92	domics Pos	0.035609	0.94464	1.1583	0.21198	0.47629	0.99994	0.94849	-0.07629	0.71713	0.99996	1.0309	0.04388	0.99028	0.9969	1.0149	0.02138	0.024091	0.059921	0.79651	-0.32824	0.027115	0.14544	0.82458	-0.27826	0.52866	0.84096	0.94829	-0.0766	0.7273	0.97988	1.0464	0.065424	0.058967	0.15646	1.2089	0.27373
128.1077	2.05	domics Pos	0.03571	0.94464	1.2589	0.33212	0.18087	0.99994	0.88024	-0.18403	0.52755	0.99996	1.057	0.079953	0.10917	0.53208	0.7786	-0.36105	4.07E-06	0.000146	0.1666	-2.5855	0.007294	0.082061	0.54511	-0.87537	0.19348	0.64891	0.83564	-0.25904	0.74781	0.97988	0.97386	-0.03822	0.001312	0.07528	1.413	0.49876
810.4471	0.54	domics Pos	0.035721	0.94464	1.3962	0.48155	0.045447	0.99994	0.84287	-0.24662	0.47801	0.99996	0.9526	-0.07006	0.001673	0.17581	0.53494	-0.90254	8.38E-05	0.00091	0.3753	-1.4139	8.57E-06	0.01634	0.52014	-0.94304	0.18966	0.64891	0.79638	-0.32848	0.4545	0.96776	0.90787	-0.13944	0.018962	0.10449	2.03	1.0215
248.2459	2.05	domics Pos	0.035753	0.94464	1.2526	0.32493	0.33292	0.99994	0.90602	-0.14238	0.62327	0.99996	1.0414	0.058505	0.12006	0.54112	0.77108	-0.37504	2.89E-06	0.000144	0.16575	-2.5929	0.007242	0.082061	0.55903	-0.839	0.23353	0.6761	0.84266	-0.24697	0.86077	0.98023	0.98512	-0.02163	0.002097	0.07995	1.4152	0.501
181.1597	5.99	bolomics Po	0.035958	0.99951	1.2473	0.31883	0.44143	0.99998	0.92747	-0.10862	0.33185	0.99941	1.1159	0.15816	0.12459	0.4869	0.77649	-0.36496	2.74E-05	0.000587	0.24736	-2.0153	0.015854	0.093702	0.60432	-0.72662	0.2815	0.89933	0.84661	-0.24024	0.29511	0.99956	0.89185	-0.16513	0.018916	0.2688	1.2622	0.33598
261.2218	1.4	domics Pos	0.036155	0.94464	1.2251	0.29292	0.40604	0.99994	0.91918	-0.12158	0.70685	0.99996	1.0468	0.066009	0.193	0.63549	0.78754	-0.34458	8.85E-06	0.000208	0.20229	-2.3055	0.008948	0.08583	0.56653	-0.81978	0.23641	0.67743	0.85	-0.23447	0.5786	0.9703	0.95065	-0.07301	0.00241	0.082646	1.3805	0.46514
166.1672	2.05	domics Pos	0.036471	0.94464	1.3578	0.44132	0.57639	0.99994	0.91291	-0.13145	0.56416	0.99996	1.0693	0.096681	0.44678	0.86921	0.87917	-0.18579	2.8E-05	0.000457	0.19254	-2.3768	0.005572	0.075454	0.52711	-0.92382	0.37196	0.76056	0.84868	-0.23671	0.86929	0.98023	1.0127	0.018158	0.028164	0.11704	1.4571	0.54314
212.2014	2.05	domics Pos	0.036629	0.94464	1.2543	0.32684	0.363	0.99994	0.91329	-0.13086	0.47699	0.99996	1.0619	0.086705	0.16081	0.59245	0.78632	-0.34682	2.9E-06	0.000144	0.16426	-2.606	0.008685	0.085129	0.54686	-0.87076	0.18332	0.64793	0.82612	-0.27558	0.85398	0.97988	0.98392	-0.02339	0.003714	0.085302	1.3973	0.48266
664.7482	9.92	domics Pos	0.037407	0.94464	1.2264	0.29448	0.66985	0.99994	0.95849	-0.06117	0.9512	0.99996	1.0057	0.008135	0.14962	0.57784	0.88705	-0.17291	0.11248	0.19028	0.80811	-0.30737	0.71732	0.85308	0.96429	-0.05247	0.66457	0.897	0.96201	-0.05588	0.82387	0.97988	1.0333	0.04721	0.046911	0.14344	1.2343	0.30368
136.1208	1.66	domics Pos	0.037476	0.94464	1.2934	0.37113	0.55731	0.99994	0.9374	-0.09327	0.55864	0.99996	1.0515	0.072448	0.030537	0.30481	0.72494	-0.46407	0.000309	0.00227	0.26724	-1.9038	0.008249	0.084185	0.58154	-0.78205	0.24317	0.67954	0.86201	-0.21423	0.49772	0.96776	0.9197	-0.12076	0.006038	0.08977	1.4175	0.50337
155.144	2.05	domics Pos	0.037563	0.94464	1.3896	0.4747	0.71043	0.99994	1.05	0.070373	0.26526	0.99996	1.1965	0.25886	0.38952	0.83582	0.83667	-0.25726	7.95E-05	0.000883	0.21538	-2.215	0.049188	0.21583	0.60834	-0.71705	0.54999	0.85184	0.88994	-0.16822	0.81031	0.97988	1.014	0.020026	0.009754	0.094875	1.5786	0.65865
751.9325	3.24	bolomics Po	0.037729	0.99951	1.4048	0.49036	0.038952	0.99998	0.82715	-0.27378	0.17803	0.99941	0.86146	-0.21515	0.003855	0.17363	0.64672	-0.62878	8.27E-05	0.001393	0.51554	-0.95583	0.000948	0.042705	0.6968	-0.52118	0.832	0.9951	1.0063	0.009067	0.94367	0.99956	1.0171	0.02449	0.050197	0.29228	1.9339	0.95148
1046.757	10.08	domics Pos	0.037958	0.94464	1.2083	0.27301	0.067872	0.99994	1.1001	0.13762	0.34931	0.99996	1.1452	0.19565	0.57551	0.91702	1.067	0.093519	0.013838	0.039319	0.83449	-0.26103	0.90941	0.95263	1.0536	0.075328	0.027425	0.34512	1.1605	0.2148	0.78842	0.97988	0.98565	-0.02085	0.012886	0.098791	1.3551	0.43835
226.2154	7.02	bolomics Po	0.038203	0.99951	1.3304	0.41191	0.20219	0.99998	0.84032	-0.25099	0.56664	0.99941	1.06	0.084078	0.2597	0.7335	0.80907	-0.30566	4.15E-06	0.000231	0.17975	-2.4759	0.012912	0.085844	0.53905	-0.8915	0.094107	0.60726	0.78361	-0.35179	0.60329	0.99956	0.94297	-0.08471	0.025335	0.2688	1.3681	0.45215
279.9733	2.64	bolomics Po	0.03837	0.99951	1.3535	0.4367	0.55908	0.99998	1.0829	0.11492	0.55607	0.99941	1.0658	0.091898	0.58166	0.99905	1.0499	0.070276	0.29276	0.65824	1.2077	0.27221	0.97057	0.99144	0.99343	-0.00951	0.47567	0.99391	0.91564	-0.12714	0.90486	0.99956	1.0432	0.061041	0.9989	0.99922	0.99465	-0.00774
266.2565	2.05	domics Pos	0.038417	0.94464	1.2431	0.314	0.24236	0.99994	0.88711	-0.17282	0.6698	0.99996	1.0346	0.049026	0.1195	0.54112	0.76966	-0.37771	2.43E-06	0.000144	0.16328	-2.6146	0.006297	0.078617	0.55062	-0.86087	0.19449	0.64891	0.82349	-0.28018	0.55421	0.96798	0.94654	-0.07926	0.002993	0.085302	1.3731	0.45749
576.6473	3.34	bolomics Po	0.038668	0.99951	1.3463	0.42903	0.023541	0.99998	0.80521	-0.31256	0.50339	0.99941	0.93111	-0.10298	0.000232	0.077904	0.53225	-0.90983	0.000278	0.003655	0.52222	-0.93728	9.77E-05	0.034543	0.5858	-0.77153	0.36019	0.96545	0.86085	-0.21617	0.20575	0.99956	0.82508	-0.27739	0.019144	0.2688	1.7649	0.81955
582.5255	2.05	domics Pos	0.038955	0.94464	1.5621	0.64348	0.61487	0.99994	0.90749	-0.14004	0.26337	0.99996	1.2115	0.27681	0.19837	0.64373	0.68959	-0.53618	2.35E-06	0.000144	0.022236	-5.491	0.012661	0.095104	0.31279	-1.6767	0.22392	0.66652	0.72352	-0.46689	0.92484	0.98531	0.99682	-0.0046	0.007324	0.091944	1.7465	0.8045
183.1705	2.05	domics Pos	0.038973	0.94464	1.4958	0.58089	0.6964	0.99994	0.91483	-0.12842	0.096046	0.99996	1.4037	0.48924	0.65723	0.95652	1.108	0.14802	0.000246	0.001942	0.27982	-1.8374	0.12145	0.37845	0.71896	-0.47602	0.80434	0.95494	0.95603	-0.06488	0.35607	0.96776	1.2212	0.28826	0.010286	0.094875	1.766	0.82051
573.3389	3.34	bolomics Po	0.039036	0.99951	1.3726	0.45687	0.13738	0.99998	0.89986	-0.15222	0.85183	0.99941	1.0204	0.029134	0.002534	0.15651	0.62303	-0.68262	0.0037	0.023837	0.52245	-0.93664	0.00101	0.043099	0.62471	-0.67873	0.74549	0.99391	0.99528	-0.00682	0.28256	0.99956	0.85445	-0.22694	0.034207	0.27077	1.8261	0.86879
170.1545	1.66	domics Pos	0.039059	0.94464	1.2852	0.36195	0.32372	0.99994	0.89017	-0.16785	0.53659	0.99996	1.0762	0.10598	0.1627	0.5931	0.78091	-0.35677	2.49E-06	0.000144	0.16082	-2.6364	0.009406	0.08684	0.56913	-0.81318	0.24272	0.67954	0.81065	-0.30285	0.66966	0.97988	0.95478	-0.06676	0.006912	0.091944	1.3941	0.47936
164.1518	1.66	domics Pos	0.039144	0.94464	1.3154	0.3955	0.70126	0.99994	0.95721	-0.0631	0.68102	0.99996	1.0499	0.07027	0.2843	0.75005	0.82168	-0.28335	2.05E-05	0.000373	0.19052	-2.392	0.013312	0.097715	0.54696	-0.8705	0.11702	0.56981	0.79419	-0.33245	0.34796	0.96563	0.90335	-0.14665	0.028078	0.11688	1.3942	0.4794
278.2483	1.4	domics Pos	0.03916	0.94464	1.3129	0.39275	0.28999	0.99994	0.8907	-0.16699	0.51341	0.99996	1.0949	0.13079	0.076975	0.46503	0.74637	-0.42203	1.25E-05	0.00027	0.18236	-2.4552	0.004574	0.071311	0.52765	-0.92233	0.19323	0.64891	0.80757	-0.30835	0.74868	0.97988	0.9711	-0.04231	0.004518	0.085788	1.3749	0.45936
608.848	3.05	bolomics Po	0.039171	0.99951	1.2744	0.34981	0.10558	0.99998	0.8489	-0.23634	0.94909	0.99941	1.0105	0.015058	0.002327	0.15466	0.64511	-0.63238	0.007772	0.040004	0.56482	-0.82414	0.000533	0.039708	0.61105	-0.71064	0.58285	0.99391	0.94474	-0.08201	0.35723	0.99956	0.87065	-0.19984	0.012108	0.2688	1.8479	0.88588
257.2679	6.2	bolomics Po	0.039178	0.99951	1.2431	0.31392	0.41959	0.99998	0.92299	-0.11561	0.51995	0.99941	1.0804	0.1116	0.19286	0.62695	0.81719	-0.29125	3.36E-05	0.000679	0.21486	-2.2186	0.013223	0.085844	0.58048	-0.78468	0.12945	0.6766	0.81495	-0.29522	0.568	0.99956	0.96001	-0.05888	0.023751	0.2688	1.1895	0.25033
181.1592	1.66	domics Pos	0.039223	0.94464	1.3367	0.41873	0.66806	0.99994	0.94392	-0.08327	0.53634	0.99996	1.1064	0.14585	0.22192	0.67657	0.81966	-0.28691	5.29E-06	0.000159	0.20089	-2.3155	0.021319	0.12682	0.58893	-0.76384	0.48023	0.8131	0.89112	-0.1663	0.66648	0.97988	0.94598	-0.08011	0.011561	0.096218	1.4882	0.57361
469.9798	4.96	olomics Ne	0.039296	0.99918	1.0775	0.10773	0.52405	0.86583	0.96218	-0.05563	0.79536	0.99995	0.98671	-0.0193	0.95965	0.99985	1.0056	0.008059	0.20423	0.99927	1.0534	0.075064	0.19739	0.65203	1.0993	0.13662	0.065503	0.40302	1.1355	0.18331	0.50857	0.99512	1.0373	0.052828	0.80683	0.99941	0.98822	-0.01709
184.1702	2.05	domics Pos	0.039436	0.94464	1.259	0.33229	0.27843	0.99994	0.89683	-0.1571	0.74178	0.99996	1.0282	0.040078	0.09599	0.51551	0.74991	-0.41521	3.04E-06	0.000144	0.16116	-2.6334	0.005295	0.074232	0.54869	-0.86594	0.20043	0.64891	0.82738	-0.27339	0.81492	0.97988	0.98071	-0.02811	0.001895	0.078631	1.4049	0.49042
488.5993	3.21	bolomics Po	0.039778	0.99951	1.4412	0.52732	0.59149	0.99998	0.95695	-0.06348	0.95233	0.99941	1.0473	0.066681	0.013261	0.20085	0.68633	-0.54302	0.051815	0.17345	0.73202	-0.45004	0.033577	0.14225	0.74718	-0.42048	0.91153	0.9951	1.0923	0.12738	0.98415	0.99956	1.0362	0.051315	0.0109	0.2688	1.9043	0.92927
156.1389	1.66	domics Pos	0.039805	0.94464	1.3267	0.4079	0.3603	0.99994	0.89055	-0.16723	0.51765	0.99996	1.08	0.11106	0.14647	0.57298	0.77407	-0.36946	1.67E-05	0.00033	0.1659	-2.5916	0.008332	0.084185	0.55514	-0.84907	0.2482	0.68479	0.83092	-0.26722	0.61347	0.97371	0.93766	-0.09286	0.009566	0.094007	1.4126	0.49831
142.1234	2.05	domics Pos	0.039847	0.94464	1.2648	0.33888	0.34974	0.99994	0.90369	-0.1461	0.58326	0.99996	1.0432	0.061032	0.12579	0.54879	0.76595	-0.38468	6E-06	0.000168	0.1673	-2.5795	0.005165	0.074042	0.55451	-0.85071	0.19984	0.64891	0.81178	-0.30084	0.80893	0.97988	0.97603	-0.035	0.00387	0.085302	1.4069	0.49256
226.217	2.05	domics Pos	0.040071	0.94464	1.2311	0.29999	0.40134	0.99994	0.92163	-0.11774	0.66396	0.99996	1.0371	0.052579	0.11406	0.53299	0.76281	-0.3906	2.94E-06	0.000144	0.16304	-2.6167	0.007393	0.082061	0.54589	-0.87331	0.19616	0.64891	0.8302	-0.26847	0.6804	0.97988	0.96293	-0.05449	0.003321	0.085302	1.3786	0.46316
198.1858	1.66	domics Pos	0.040095	0.94464	1.2999	0.37843	0.38626	0.99994	0.8873	-0.1725	0.75818	0.99996	1.0412	0.058285	0.14725	0.57394	0.76687	-0.38294	1.86E-06	0.000144	0.1526	-2.7122	0.008614	0.084941	0.54902	-0.86507	0.16292	0.63418	0.79808	-0.32539	0.5296	0.96776	0.92551	-0.11168	0.011008	0.094875	1.3725	0.45677
156.1377	2.76	domics Pos	0.040112	0.94464	1.3905	0.4756	0.34558	0.99994	0.83213	-0.26512	0.9523	0.99996	0.98948	-0.01525	0.1775	0.61058	0.74233	-0.42987	4.1E-05	0.000553	0.19499	-2.3585	0.004605	0.071311	0.48697	-1.0381	0.23621	0.67743	0.78633	-0.34679	0.78213	0.97988	0.94137	-0.08716	0.014264	0.098791	1.571	0.65165
766.4201	0.55	domics Pos	0.040439	0.94464	1.3785	0.46306	0.055308	0.99994	0.87693	-0.18946	0.82854	0.99996	0.98753	-0.01811	0.003008	0.17581	0.59573	-0.74727	6.99E-05	0.00082	0.42289	-1.2417	0.000877	0.042199	0.57946	-0.78723	0.33688	0.74249	0.84927	-0.2357	0.50772	0.96776	0.92298	-0.11563	0.008591	0.092495	2.1627	1.1128
448.8462	10.14	domics Pos	0.040565	0.94464	1.1878	0.24828	0.36682	0.99994	1.0493	0.069394	0.28905	0.99996	1.1746	0.23218	0.60151	0.93327	1.0947	0.13059	0.038059	0.085263	0.80347	-0.31569	0.78292	0.88816	0.98999	-0.01452	0.07048	0.47065	1.21	0.27496	0.01841	0.52955	1.1255	0.17054	0.004437	0.085451	1.3514	0.43444
875.4816	0.54	domics Pos	0.040578	0.94464	1.4016	0.48711	0.095765	0.99994	0.89172	-0.16533	0.74976	0.99996	1.0223	0.031771	0.003118	0.17581	0.5599	-0.83676	4.05E-05	0.000552	0.39839	-1.3278	0.000219	0.026314	0.49212	-1.0229	0.20915	0.65208	0.79962	-0.32261	0.5688	0.9703	0.94622	-0.07976	0.008528	0.092495	2.1788	1.1236
576.6443	0.53	domics Pos	0.041142	0.94464	1.462	0.54797	0.087024	0.99994	0.89353	-0.16241	0.83565	0.99996	0.99004	-0.01444	0.00142	0.17581	0.57222	-0.80535	0.0001	0.001029	0.38379	-1.3816	0.000572	0.038292	0.58123	-0.78282	0.44949	0.80156	0.90727	-0.14039	0.68157	0.97988	0.99387	-0.00888	0.013967	0.098791	2.2736	1.185
203.6416	2.06	domics Pos	0.041265	0.94464	1.1365	0.18461	0.36877	0.99994	0.91899	-0.12187	0.51564	0.99996	0.9496	-0.07462	0.13509	0.55953	0.86209	-0.21408	2.03E-05	0.000372	0.26982	-1.8899	0.018637	0.11669	0.73709	-0.44009	0.38391	0.76442	0.92345	-0.11489	0.85986	0.98023	1.0452	0.063752	0.001065	0.07528	1.3638	0.44767
340.2142	1.91	domics Pos	0.041295	0.94464	1.3371	0.41907	0.70529	0.99994	1.0596	0.083492	0.27718	0.99996	1.1521	0.20427	0.1963	0.64175	0.81273	-0.29915	0.000213	0.001753	0.28065	-1.8332	0.005606	0.07553	0.60719	-0.71979	0.96045	0.99032	0.98828	-0.01702	0.94769	0.98838	1.0253	0.036094	0.070459	0.17145	1.2936	0.37138
517.942	0.53	domics Pos	0.041473	0.94464	1.4247	0.51063	0.037387	0.99994	0.83977	-0.25193	0.77803	0.99996	0.98124	-0.02732	0.00338	0.17581	0.62095	-0.68744	0.000254	0.00197	0.43577	-1.1984	0.00111	0.043236	0.58332	-0.77764	0.63398	0.8822	0.95698	-0.06343	0.51054	0.96776	0.92549	-0.11172	0.014345	0.098791	2.3078	1.2065
262.2533	1.67	domics Pos	0.041492	0.94464	1.3586	0.44208	0.60993	0.99994	0.95122	-0.07215	0.39539	0.99996	1.1125	0.15378	0.2944	0.75244	0.86177	-0.21463	3E-06	0.000144	0.19307	-2.3728	0.002797	0.061655	0.60446	-0.72629	0.61056	0.87064	0.94942	-0.07489	0.77875	0.97988	0.97543	-0.0359	0.002603	0.082646	1.5555	0.63734
170.1551	6.38	bolomics Po	0.041638	0.99951	1.2356	0.30525	0.33898	0.99998	0.92251	-0.11637	0.48955	0.99941	1.069	0.096265	0.26402	0.73853	0.85188	-0.23128	6.26E-06	0.000265	0.22024	-2.1829	0.012139	0.083858	0.61166	-0.7092	0.17637	0.74875	0.84542	-0.24225	0.62281	0.99956	0.96263	-0.05494	0.039545	0.28339	1.184	0.24369
135.1175	1.67	domics Pos	0.041882	0.94464	1.2905	0.36795	0.18034	0.99994	0.83092	-0.26722	0.58439	0.99996	1.0534	0.075003	0.16181	0.59299	0.77317	-0.37114	7.06E-06	0.000186	0.18514	-2.4333	0.022667	0.13196	0.59104	-0.75868	0.28977	0.71515	0.83152	-0.26618	0.81746	0.97988	0.97207	-0.04086	0.007312	0.091944	1.4024	0.48793
367.2314	2.06	domics Pos	0.041936	0.94464	1.1573	0.21081	0.44666	0.99994	0.94633	-0.07958	0.8427	0.99996	0.98454	-0.02248	0.25423	0.71116	0.85259	-0.23007	0.000227	0.001838	0.44243	-1.1765	0.003088	0.063487	0.61901	-0.69196	0.18785	0.64793	0.85957	-0.21832	0.5585	0.96925	0.94879	-0.07583	0.022223	0.1085	1.2144	0.28023
625.4456	3.84	domics Pos	0.041972	0.94464	1.2345	0.30388	0.63097	0.99994	0.95177	-0.07132	0.40372	0.99996	1.1525	0.20481	0.9726	0.99502	1.015	0.021466	0.085914	0.156	0.79419	-0.33245	0.16562	0.45081	1.1924	0.25386	0.30723	0.72831	1.1722	0.22924	0.83672	0.97988	1.1234	0.16784	0.69034	0.769	1.1778	0.23615
762.5988	10.24	bolomics Po	0.042056	0.99951	0.80387	-0.31497	0.1765	0.99998	0.85598	-0.22434	0.12804	0.99941	0.85079	-0.23313	0.065933	0.33591	0.83863	-0.25389	0.59037	0.96255	0.9414	-0.08713	0.69653	0.91033	1.0532	0.074835	0.94903	0.9974	1.0133	0.019069	0.66079	0.99956	0.95779	-0.06222	0.47025	0.73122	0.93324	-0.09968
338.1823	6.47	bolomics Po	0.042288	0.99951	1.2006	0.26374	0.85533	0.99998	1.0245	0.034914	0.86142	0.99941	1.0091	0.013105	0.34321	0.83613	0.8933	-0.16279	4.69E-05	0.00089	0.48221	-1.0523	0.011013	0.083281	0.65701	-0.60601	0.11379	0.64238	0.78382	-0.35141	0.2698	0.99956	0.84608	-0.24114	0.38276	0.65669	0.89341	-0.16261
444.9765	12.02	olomics Ne	0.042293	0.99918	0.82603	-0.27573	0.8334	0.98836	0.98456	-0.02244	0.77761	0.99995	0.97888	-0.03079	0.55224	0.99985	0.92953	-0.10543	0.42502	0.99927	0.93337	-0.09948	0.54611	0.87267	0.93515	-0.09673	0.80443	0.95074	0.98099	-0.02769	0.68018	0.99512	0.96266	-0.0549	0.59127	0.99941	0.95633	-0.06442
287.2733	4.73	domics Pos	0.042815	0.94464	1.1538	0.20643	0.62269	0.99994	0.96166	-0.05641	0.236	0.99996	1.1456	0.1961	0.22777	0.68384	1.1608	0.21509	0.15986	0.2483	0.84644	-0.24052	0.074199	0.28088	1.178	0.23631	0.078903	0.49533	1.2181	0.28461	0.1925	0.92582	1.1809	0.23995	0.015838	0.099299	1.3729	0.45726
149.1331	1.66	domics Pos	0.042947	0.94464	1.2677	0.3422	0.48832	0.99994	0.91773	-0.12386	0.41362	0.99996	1.081	0.11239	0.17646	0.60979	0.7781	-0.36197	2.14E-06	0.000144	0.17392	-2.5235	0.004	0.069859	0.54437	-0.87733	0.25307	0.69047	0.83487	-0.26038	0.87402	0.98023	0.98599	-0.02035	0.007697	0.091944	1.4131	0.49891
721.3915	0.56	domics Pos	0.042979	0.94464	1.3832	0.46799	0.078472	0.99994	0.85847	-0.22016	0.83044	0.99996	0.98713	-0.01869	0.010295	0.21333	0.56481	-0.82416	9.54E-05	0.000988	0.40685	-1.2974	0.00047	0.033979	0.53127	-0.91248	0.27258	0.70555	0.83291	-0.26376	0.48417	0.96776	0.91535	-0.1276	0.009861	0.094875	2.1043	1.0733
705.3253	1.84	domics Pos	0.043032	0.94464	1.546	0.62855	0.69119	0.99994	1.0412	0.058237	0.66566	0.99996	0.95644	-0.06425	0.11097	0.53238	1.2251	0.29285	0.96214	0.96772	1.0094	0.013442	0.52942	0.7362	1.0748	0.10406	0.30728	0.72831	1.2487	0.32042	0.003753	0.52955	1.4115	0.49721	0.004151	0.085451	1.4328	0.51879
245.2269	1.66	domics Pos	0.043166	0.94464	1.2812	0.35749	0.40267	0.99994	0.9047	-0.1445	0.57959	0.99996	1.062	0.086716	0.14225	0.57114	0.77476	-0.36817	3.21E-06	0.000144	0.18966	-2.3985	0.009737	0.087714	0.56866	-0.81435	0.27868	0.70945	0.84188	-0.24831	0.70532	0.97988	0.95642	-0.06428	0.008036	0.092077	1.3882	0.47324
1070.52	6.12	domics Pos	0.043401	0.94464	1.2736	0.34895	0.43725	0.99994	1.097	0.13351	0.66502	0.99996	1.0665	0.092873	0.87033	0.9874	0.98778	-0.01775	0.021521	0.055332	0.76598	-0.38461	0.062376	0.24983	0.77435	-0.36894	0.10808	0.5577	0.8139	-0.29708	0.32015	0.9524	0.88688	-0.17319	0.37887	0.48756	1.1441	0.19417
165.991	10.2	domics Pos	0.043433	0.94464	1.1484	0.19961	0.81689	0.99994	0.98291	-0.02487	0.84345	0.99996	0.98526	-0.02143	0.49355	0.88874	1.0534	0.075034	0.080787	0.14946	0.85691	-0.22278	0.66598	0.82608	1.0641	0.089589	0.033597	0.36346	1.1858	0.24581	0.31838	0.9524	1.0925	0.12768	0.007365	0.091944	1.3215	0.40222
197.0223	12.14	bolomics Po	0.043462	0.99951	0.82393	-0.27941	0.5596	0.99998	0.93793	-0.09245	0.37143	0.99941	0.91029	-0.1356	0.72545	0.99905	0.96279	-0.05471	0.79206	0.99688	0.97289	-0.03966	0.73845	0.926	0.96767	-0.04741	0.59812	0.99391	0.95223	-0.07061	0.097842	0.82188	0.8205	-0.28543	0.66484	0.84814	0.95519	-0.06614
294.9398	0.5	domics Pos	0.043803	0.94464	1.0505	0.071121	0.28927	0.99994	0.95328	-0.06903	0.067227	0.99996	0.94238	-0.08562	0.046021	0.3709	0.90223	-0.14843	2.93E-06	0.000144	0.76879	-0.37934	0.288	0.57347	0.90688	-0.14102	0.55936	0.85477	0.96534	-0.0509	0.25025	0.94365	0.95985	-0.05912	0.020937	0.10765	1.1843	0.24407
156.139	2.05	domics Pos	0.043851	0.94464	1.2222	0.28944	0.21289	0.99994	0.87799	-0.18773	0.78193	0.99996	1.0265	0.037735	0.12885	0.55024	0.76484	-0.38677	2.5E-06	0.000144	0.1624	-2.6224	0.006393	0.079158	0.5603	-0.83574	0.11635	0.56866	0.79457	-0.33175	0.64974	0.97988	0.95715	-0.06318	0.003722	0.085302	1.3896	0.4747
559.5196	1.67	domics Pos	0.043953	0.94464	1.5177	0.6019	0.52121	0.99994	0.87334	-0.19539	0.30254	0.99996	1.1823	0.24165	0.24657	0.70693	0.71793	-0.47808	9.33E-06	0.000213	0.087106	-3.5211	0.009148	0.086817	0.3728	-1.4235	0.46898	0.80647	0.85924	-0.21887	0.301	0.95107	0.79708	-0.32721	0.048192	0.1448	1.5619	0.64334
142.1239	5.99	bolomics Po	0.044847	0.99951	1.1966	0.259	0.53494	0.99998	0.94033	-0.08876	0.83328	0.99941	1.0341	0.04841	0.09301	0.41034	0.77772	-0.36268	4.42E-06	0.000232	0.22442	-2.1558	0.012567	0.085318	0.61258	-0.70704	0.10414	0.61787	0.80291	-0.3167	0.45789	0.99956	0.93782	-0.09262	0.07076	0.31396	1.1494	0.20092
754.9349	3.23	bolomics Po	0.04486	0.99951	1.4685	0.55431	0.028776	0.99998	0.81914	-0.28782	0.69582	0.99941	0.97257	-0.04013	0.00052	0.12797	0.69213	-0.53089	0.001355	0.011665	0.59437	-0.75056	3.59E-05	0.034543	0.67047	-0.57676	0.17883	0.75255	0.80941	-0.30505	0.68597	0.99956	0.97065	-0.04298	0.087678	0.34013	1.5054	0.59016
278.246	1.91	domics Pos	0.044911	0.94464	1.4268	0.51282	0.9291	0.99994	0.97749	-0.03285	0.24639	0.99996	1.1709	0.22761	0.21956	0.67258	0.77091	-0.37537	7.65E-05	0.000864	0.18537	-2.4315	0.032597	0.16205	0.52081	-0.94117	0.2814	0.7098	0.81124	-0.30181	0.93746	0.98616	0.98454	-0.02248	0.014642	0.098791	1.5423	0.62511
145.12	2.04	domics Pos	0.04502	0.94464	1.3475	0.43028	0.12682	0.99994	0.78144	-0.3558	0.86927	0.99996	0.98437	-0.02273	0.10731	0.53121	0.80623	-0.31073	0.000212	0.001753	0.40475	-1.3049	0.056126	0.23466	0.66617	-0.58605	0.33568	0.74176	0.87257	-0.19666	0.36937	0.96776	0.886	-0.17463	0.044613	0.14047	1.3419	0.42432
677.3652	0.56	domics Pos	0.045058	0.94464	1.4322	0.51825	0.10234	0.99994	0.8943	-0.16117	0.95993	0.99996	0.99872	-0.00185	0.002079	0.17581	0.55852	-0.84032	5.29E-05	0.000675	0.39708	-1.3325	0.000151	0.023299	0.54321	-0.88042	0.32193	0.7311	0.85594	-0.22442	0.53326	0.96776	0.93274	-0.10045	0.0138	0.098791	2.0843	1.0596
619.8666	3.08	bolomics Po	0.045346	0.99951	1.616	0.69241	0.051814	0.99998	0.8268	-0.27438	0.69312	0.99941	0.96282	-0.05466	0.008322	0.19702	0.65243	-0.6161	0.000979	0.009122	0.58947	-0.76251	0.001027	0.043099	0.57968	-0.78666	0.59887	0.99391	0.93777	-0.0927	0.34517	0.99956	0.86021	-0.21724	0.057819	0.30327	1.7475	0.80527
256.9867	11.96	olomics Ne	0.045523	0.99918	0.78552	-0.34828	0.39112	0.79189	0.88516	-0.17598	0.11716	0.99995	0.83415	-0.26162	0.32158	0.99985	0.86778	-0.20461	0.084147	0.99927	0.8361	-0.25824	0.14897	0.6202	0.83298	-0.26365	0.073051	0.42218	0.80614	-0.3109	0.1345	0.99512	0.84483	-0.24327	0.23009	0.99941	0.89311	-0.16309
141.1284	2.05	domics Pos	0.045574	0.94464	1.249	0.32076	0.9706	0.99994	1.0133	0.019065	0.081599	0.99996	1.1565	0.20973	0.47749	0.88482	0.91759	-0.12408	1.31E-06	0.000144	0.23415	-2.0945	0.013326	0.097715	0.5953	-0.7483	0.49856	0.82652	0.91864	-0.12243	0.72113	0.97988	1.0478	0.067303	0.003134	0.085302	1.4754	0.56109
123.1175	1.66	domics Pos	0.045606	0.94464	1.2502	0.32216	0.4931	0.99994	0.91869	-0.12235	0.81579	0.99996	1.0262	0.037244	0.13605	0.55962	0.75581	-0.40391	1.31E-05	0.000277	0.18803	-2.411	0.006504	0.079718	0.54396	-0.87843	0.31585	0.72948	0.85407	-0.22757	0.64419	0.97988	0.94083	-0.088	0.022814	0.10945	1.3723	0.45662
81.0707	2.05	domics Pos	0.045619	0.94464	1.2759	0.35154	0.69331	0.99994	0.96024	-0.05853	0.41916	0.99996	1.0712	0.099214	0.21169	0.65978	0.80487	-0.31317	1.74E-05	0.000337	0.1705	-2.5522	0.008848	0.08583	0.55705	-0.84412	0.2106	0.65208	0.84094	-0.24992	0.6134	0.97371	0.94742	-0.07792	0.006566	0.090084	1.4354	0.52142
177.86	10.78	olomics Ne	0.045773	0.99918	0.91818	-0.12315	0.067513	0.50118	0.90784	-0.13949	0.29168	0.99995	0.94761	-0.07764	0.42896	0.99985	0.95733	-0.06291	0.76385	0.99927	0.98686	-0.01909	0.98998	0.99682	1.0028	0.00407	0.48168	0.80454	1.0537	0.075408	0.20214	0.99512	0.92985	-0.10493	0.22046	0.99941	0.93705	-0.0938
591.3195	0.53	domics Pos	0.046332	0.94464	1.3883	0.47335	0.059097	0.99994	0.84808	-0.23772	0.46431	0.99996	0.9509	-0.07264	0.004562	0.17581	0.55964	-0.83743	0.000108	0.001089	0.41024	-1.2854	0.000149	0.023299	0.54169	-0.88447	0.49005	0.82099	0.91722	-0.12466	0.37593	0.96776	0.87733	-0.1888	0.01372	0.098791	2.2439	1.166
184.1701	1.66	domics Pos	0.046637	0.94464	1.303	0.38186	0.43271	0.99994	0.89855	-0.15433	0.41901	0.99996	1.0987	0.1358	0.11871	0.53907	0.73616	-0.44191	2.72E-06	0.000144	0.15191	-2.7188	0.011923	0.092554	0.53558	-0.90083	0.23765	0.67844	0.81917	-0.28776	0.63269	0.97988	0.93973	-0.08968	0.012756	0.098791	1.3835	0.46828
777.545	4.94	domics Pos	0.046823	0.94464	1.1111	0.15193	0.80119	0.99994	0.98834	-0.01692	0.79223	0.99996	0.98515	-0.02159	0.9917	0.99719	1.0027	0.003833	0.034424	0.078787	0.86645	-0.20682	0.68013	0.83467	0.97099	-0.04247	0.14412	0.60716	1.097	0.13362	0.019572	0.52955	1.1483	0.19944	0.000522	0.07528	1.2798	0.35595
935.8058	10.02	domics Pos	0.046871	0.94464	1.1391	0.18795	0.479	0.99994	1.0497	0.070027	0.54399	0.99996	1.052	0.073082	0.54053	0.90385	1.0484	0.068236	0.083053	0.15253	0.85844	-0.22021	0.90491	0.95053	1.0056	0.00807	0.081718	0.49998	1.111	0.15181	0.73329	0.97988	1.049	0.068972	0.091174	0.19165	1.1957	0.25785
184.1708	6.38	bolomics Po	0.047302	0.99951	1.2074	0.27185	0.3274	0.99998	0.92377	-0.11439	0.66093	0.99941	1.0433	0.061119	0.18864	0.6204	0.82616	-0.2755	8.65E-06	0.000292	0.21876	-2.1926	0.012272	0.084558	0.61786	-0.69465	0.088658	0.60275	0.80879	-0.30616	0.35573	0.99956	0.93262	-0.10064	0.05603	0.29896	1.1626	0.2173
406.2182	0.58	domics Pos	0.047385	0.94464	1.5695	0.65026	0.04265	0.99994	0.81932	-0.28751	0.58374	0.99996	1.0432	0.061069	0.00331	0.17581	0.59116	-0.75839	0.00119	0.006234	0.43733	-1.1932	0.000243	0.026314	0.57688	-0.79367	0.84998	0.9681	1.1206	0.16431	0.86367	0.98023	1.0953	0.13129	0.016857	0.099807	2.8943	1.5332
95.0883	4.73	domics Pos	0.047627	0.94464	1.2153	0.28127	0.68608	0.99994	1.032	0.045494	0.11236	0.99996	1.2023	0.26576	0.9292	0.9874	0.99023	-0.01416	0.27823	0.37303	0.87117	-0.19897	0.94633	0.9713	0.99227	-0.01119	0.98722	0.99505	0.99835	-0.00239	0.5658	0.9703	1.102	0.14016	0.18909	0.29671	1.2513	0.32342
135.1175	2.05	domics Pos	0.047778	0.94464	1.2323	0.30131	0.3224	0.99994	0.90146	-0.14966	0.47323	0.99996	1.0586	0.082161	0.10138	0.52336	0.75406	-0.40725	2.32E-06	0.000144	0.17107	-2.5473	0.00572	0.076133	0.54313	-0.88064	0.17239	0.64606	0.82004	-0.28623	0.65469	0.97988	0.95571	-0.06536	0.002536	0.082646	1.431	0.51705
699.3779	0.56	domics Pos	0.047926	0.94464	1.4431	0.52913	0.13774	0.99994	0.90576	-0.1428	0.96964	0.99996	0.99855	-0.00209	0.004641	0.17581	0.57431	-0.80009	7.65E-05	0.000864	0.40793	-1.2936	0.000935	0.043236	0.54745	-0.86919	0.42353	0.78739	0.89199	-0.16489	0.56557	0.9703	0.94356	-0.08381	0.012035	0.098057	2.1489	1.1036
654.3381	0.58	domics Pos	0.048024	0.94464	1.5422	0.62502	0.50661	0.99994	0.94683	-0.07883	0.51418	0.99996	0.94287	-0.08488	0.010614	0.21362	0.59271	-0.75461	3.77E-05	0.00053	0.44136	-1.18	0.000896	0.042199	0.54876	-0.86575	0.63963	0.88465	0.95996	-0.05895	0.971	0.99181	1.0569	0.079802	0.012987	0.098791	2.4714	1.3053
503.2665	0.53	domics Pos	0.048026	0.94464	1.3894	0.47443	0.039465	0.99994	0.8249	-0.27771	0.54502	0.99996	0.95389	-0.0681	0.001446	0.17581	0.54855	-0.86631	0.000101	0.001031	0.4215	-1.2464	4.38E-05	0.019289	0.52407	-0.93218	0.4951	0.82378	0.91821	-0.1231	0.46718	0.96776	0.91276	-0.13169	0.021189	0.10765	2.1588	1.1102
1005.546	0.57	domics Pos	0.048112	0.94464	1.5245	0.60836	0.065829	0.99994	0.87293	-0.19606	0.30701	0.99996	1.1333	0.18048	0.037988	0.34165	0.6625	-0.594	0.000857	0.004958	0.49164	-1.0243	0.002818	0.061757	0.58161	-0.78186	0.84795	0.96798	1.016	0.022842	0.65884	0.97988	1.1114	0.15241	0.010181	0.094875	2.6478	1.4048
736.5769	2.05	domics Pos	0.048134	0.94464	1.2922	0.3698	0.31648	0.99994	0.86825	-0.20382	0.90696	0.99996	1.0026	0.003721	0.052533	0.39276	0.73451	-0.44514	2.18E-05	0.000383	0.1122	-3.1559	0.008664	0.085129	0.45828	-1.1257	0.228	0.67233	0.79516	-0.33068	0.91999	0.98344	1.0003	0.00039	0.000586	0.07528	1.6648	0.73531
187.6283	1.47	domics Pos	0.048243	0.94464	1.3802	0.4649	0.87795	0.99994	1.0191	0.027298	0.98922	0.99996	1.0234	0.033371	0.91715	0.9874	1.0115	0.01652	0.001033	0.0056	0.23903	-2.0647	0.12346	0.38281	0.71557	-0.48283	0.78422	0.94942	0.99126	-0.01266	0.49641	0.96776	1.1038	0.14251	0.011162	0.094875	1.4626	0.54857
163.1492	6	bolomics Po	0.048587	0.99951	1.2436	0.31448	0.42574	0.99998	0.91922	-0.12152	0.57483	0.99941	1.0731	0.10184	0.093265	0.41089	0.76591	-0.38476	7.81E-06	0.000286	0.25223	-1.9872	0.015244	0.092479	0.57528	-0.79767	0.15311	0.72748	0.80075	-0.32058	0.65697	0.99956	0.9598	-0.05919	0.21315	0.4802	1.1504	0.20214
698.7846	10.12	domics Pos	0.048613	0.94464	1.1923	0.25375	0.31619	0.99994	1.066	0.092228	0.62628	0.99996	1.0362	0.051353	0.22915	0.68626	1.0878	0.12148	0.17677	0.26843	0.88449	-0.17709	0.84992	0.92502	1.0224	0.031965	0.034582	0.36356	1.1942	0.2561	0.093475	0.83733	1.1973	0.25984	0.01408	0.098791	1.3857	0.47057
163.1487	1.66	domics Pos	0.048932	0.94464	1.285	0.3618	0.48985	0.99994	0.9127	-0.13178	0.77025	0.99996	1.0268	0.03821	0.14612	0.57298	0.76032	-0.39532	4.79E-06	0.000152	0.15782	-2.6636	0.01308	0.096469	0.51657	-0.95298	0.36257	0.75814	0.86771	-0.20471	0.5872	0.97073	0.93374	-0.0989	0.011965	0.097695	1.375	0.45948
200.6362	1.67	domics Pos	0.049093	0.94464	1.2394	0.30961	0.22474	0.99994	0.89322	-0.16291	0.61678	0.99996	1.0382	0.05405	0.21433	0.66281	0.83633	-0.25786	1.42E-05	0.000291	0.19785	-2.3376	0.002841	0.061884	0.59147	-0.75761	0.55192	0.85184	0.92962	-0.10529	0.67931	0.97988	0.96428	-0.05248	0.023756	0.11051	1.3845	0.46936
114.0919	2.05	domics Pos	0.049262	0.94464	1.2203	0.28726	0.22265	0.99994	0.88219	-0.18083	0.81419	0.99996	1.0221	0.031574	0.1062	0.53006	0.74945	-0.4161	1.14E-06	0.000144	0.15844	-2.658	0.01084	0.090053	0.54149	-0.88498	0.13979	0.60432	0.80088	-0.32035	0.57493	0.9703	0.94959	-0.07463	0.002096	0.07995	1.3853	0.47023
526.2909	0.58	domics Pos	0.049334	0.94464	1.7025	0.76764	0.57365	0.99994	0.92477	-0.11283	0.57998	0.99996	1.0761	0.10586	0.029888	0.30229	0.62539	-0.67717	0.000629	0.003898	0.42149	-1.2464	0.007272	0.082061	0.62991	-0.66678	0.69354	0.9106	0.96164	-0.05643	0.98292	0.99507	1.1909	0.25206	0.007388	0.091944	2.6174	1.3881
636.9719	12	olomics Ne	0.049401	0.99918	1.2082	0.27281	0.66499	0.92948	0.96057	-0.05804	0.008276	0.99995	1.3386	0.42073	0.9882	0.99985	1.0205	0.029222	0.037884	0.99927	1.2167	0.28297	0.26431	0.69165	1.0493	0.069392	0.05394	0.39335	1.1411	0.19037	0.50021	0.99512	1.0736	0.10244	0.40431	0.99941	1.1012	0.13907
128.1077	1.67	domics Pos	0.049445	0.94464	1.2328	0.30198	0.1855	0.99994	0.84635	-0.24067	0.95815	0.99996	0.99792	-0.00301	0.086724	0.49062	0.72745	-0.45909	1.24E-06	0.000144	0.17963	-2.4769	0.007931	0.084185	0.53907	-0.89145	0.12746	0.58744	0.758	-0.39973	0.44138	0.96776	0.90971	-0.13652	0.016477	0.099614	1.3336	0.41537
809.4438	0.54	domics Pos	0.049535	0.94464	1.3589	0.44246	0.12906	0.99994	0.88256	-0.18023	0.75119	0.99996	0.98011	-0.02899	0.004169	0.17581	0.55621	-0.8463	4.76E-05	0.000616	0.39182	-1.3517	7.17E-05	0.019289	0.5353	-0.90159	0.26136	0.69644	0.82245	-0.28199	0.49498	0.96776	0.91688	-0.12519	0.011326	0.095127	2.0756	1.0535
743.4041	0.56	domics Pos	0.049583	0.94464	1.3967	0.48207	0.16431	0.99994	0.89007	-0.168	0.91426	0.99996	0.99367	-0.00916	0.004995	0.17581	0.5806	-0.78437	6.44E-05	0.000767	0.40684	-1.2975	0.000334	0.028292	0.55793	-0.84185	0.30148	0.72409	0.845	-0.24297	0.54588	0.96776	0.93647	-0.0947	0.016806	0.099659	2.0834	1.0589
81.0712	6.03	bolomics Po	0.049583	0.99951	1.1652	0.22055	0.59896	0.99998	0.96194	-0.05598	0.23607	0.99941	0.91443	-0.12906	0.036742	0.26185	0.68898	-0.53747	1.88E-06	0.000231	0.27118	-1.8827	0.000148	0.036471	0.559	-0.83909	0.012279	0.26861	0.7163	-0.48135	0.081461	0.78717	0.84311	-0.24621	0.69871	0.86413	0.97459	-0.03714

700.5723	10.97	olomics Po	0.049709	0.99951	0.70519	-0.50392	0.29341	0.99998	0.82931	-0.27002	0.36061	0.99941	0.82514	-0.27729	0.33547	0.82883	0.80893	-0.30591	0.79421	0.99688	0.95472	-0.06686	0.21571	0.49634	0.79265	-0.33524	0.029789	0.34883	0.59255	-0.75499	0.10961	0.86561	0.67107	-0.57545	0.033565	0.2688	0.63093	-0.66444
647.5033	2.05	lomics Pos	0.049726	0.94464	1.3942	0.47941	0.64673	0.99994	0.93252	-0.1008	0.51448	0.99996	1.1288	0.17479	0.34069	0.79697	0.83501	-0.26014	1.57E-06	0.000144	0.088083	-3.505	0.020438	0.12316	0.51074	-0.96934	0.38577	0.76565	0.8493	-0.23565	0.8124	0.97988	1.0465	0.065572	0.003902	0.085302	1.6203	0.69625
458.8546	10.14	lomics Pos	0.049757	0.94464	1.2791	0.35509	0.7737	0.99994	0.95857	-0.06105	0.2212	0.99996	1.1484	0.19966	0.75074	0.97511	1.0408	0.057698	0.2448	0.33897	0.85986	-0.21782	0.33023	0.60476	1.1589	0.21277	0.1612	0.63418	1.2224	0.28974	0.243	0.94365	1.193	0.25465	0.026159	0.11364	1.35	0.43297
518.9475	0.54	lomics Pos	0.049977	0.94464	1.3935	0.47869	0.31764	0.99994	0.88835	-0.1708	0.88604	0.99996	1.0353	0.04998	0.00378	0.17581	0.67037	-0.57696	0.039813	0.088003	0.68885	-0.53774	0.12686	0.38734	0.77321	-0.37107	0.52235	0.83957	1.1349	0.18255	0.6391	0.97988	0.95421	-0.06763	0.013887	0.098791	2.2592	1.1758
283.283	2.05	lomics Pos	0.04998	0.94464	1.2274	0.2956	0.24361	0.99994	0.8856	-0.17527	0.67089	0.99996	1.0384	0.054411	0.12531	0.54773	0.78327	-0.35242	3.83E-06	0.000145	0.17922	-2.4802	0.005669	0.076114	0.56219	-0.83086	0.20262	0.64891	0.83174	-0.26579	0.71762	0.97988	0.96576	-0.05027	0.003695	0.085302	1.3941	0.47938
191.1805	6.38	olomics Po	0.050156	0.99951	1.2015	0.26485	0.43471	0.99998	0.93027	-0.10428	0.67428	0.99941	1.0479	0.067517	0.15599	0.55586	0.81319	-0.29834	7.63E-06	0.000283	0.22106	-2.1775	0.007169	0.070405	0.59542	-0.74803	0.07637	0.56471	0.78898	-0.34193	0.27332	0.99956	0.90917	-0.13737	0.14851	0.40154	1.1449	0.19523
282.2801	2.05	lomics Pos	0.050178	0.94464	1.2464	0.3178	0.25803	0.99994	0.8816	-0.18181	0.71771	0.99996	1.0308	0.043797	0.12613	0.54962	0.76791	-0.38098	3.63E-06	0.000144	0.17006	-2.5559	0.006225	0.078151	0.55193	-0.85745	0.20847	0.65208	0.8263	-0.27525	0.64858	0.97988	0.95312	-0.06927	0.004947	0.087539	1.3896	0.47462
393.2122	2.86	olomics Po	0.050244	0.99951	1.4112	0.49688	0.34659	0.99998	0.877	-0.18936	0.58843	0.99941	0.93504	-0.09691	0.039706	0.27429	0.615	-0.70133	0.004509	0.027471	0.51702	-0.95171	0.05243	0.19379	0.68218	-0.55177	0.56524	0.99391	0.9338	-0.09882	0.77904	0.99956	0.99643	-0.00517	0.016847	0.2688	2.0218	1.0157
131.9312	12.29	olomics Po	0.050571	0.99951	0.82707	-0.27392	0.30301	0.99998	0.9085	-0.13844	0.20121	0.99941	0.88509	-0.1761	0.12117	0.47951	0.85801	-0.22094	0.34098	0.72281	0.92746	-0.10864	0.26314	0.55407	0.87335	-0.19536	0.772	0.99391	0.97368	-0.03848	0.042532	0.64572	0.82753	-0.27312	0.53932	0.77644	0.94304	-0.08461
263.2375	1.66	lomics Pos	0.050632	0.94464	1.2537	0.3262	0.31927	0.99994	0.89088	-0.1667	0.614	0.99996	1.055	0.077277	0.12866	0.55024	0.77281	-0.37182	4.26E-06	0.000147	0.18765	-2.4138	0.00818	0.084185	0.56159	-0.83241	0.23298	0.6761	0.82828	-0.2718	0.65847	0.97988	0.95041	-0.07337	0.007683	0.091944	1.3816	0.46631
674.7755	9.93	lomics Pos	0.050701	0.94464	1.1299	0.17614	0.10022	0.99994	0.84327	-0.24594	0.060112	0.99996	0.8607	-0.21641	0.52271	0.89938	0.94507	-0.0815	0.000328	0.002367	0.70493	-0.50444	0.11927	0.37461	0.82867	-0.27113	0.77115	0.94358	0.98125	-0.02731	0.20588	0.92582	0.89075	-0.16691	0.016597	0.099614	1.171	0.22777
434.9792	4.82	olomics Ne	0.050753	0.99918	1.0904	0.12486	0.082989	0.50591	0.93385	-0.09874	0.44414	0.99995	1.0288	0.041022	0.73984	0.99985	1.0201	0.028668	0.23363	0.99927	0.95925	-0.06003	0.13226	0.60525	1.0971	0.13372	0.044944	0.38689	1.1077	0.14756	0.82029	0.99512	1.0104	0.014993	0.37921	0.99941	1.0418	0.059081
647.5021	6.38	olomics Po	0.050876	0.99951	1.3022	0.38093	0.81775	0.99998	1.025	0.035655	0.32797	0.99941	1.1313	0.178	0.30606	0.79639	0.8643	-0.2104	2.29E-05	0.00052	0.25659	-1.9625	0.016436	0.094477	0.6258	-0.67624	0.45848	0.99391	0.89731	-0.15632	0.6957	0.99956	0.95534	-0.06591	0.043293	0.28452	1.2714	0.34639
163.1487	2.05	lomics Pos	0.051115	0.94464	1.2266	0.29461	0.345	0.99994	0.91178	-0.13324	0.70714	0.99996	1.0296	0.042082	0.11998	0.54112	0.77304	-0.37138	4.09E-06	0.000146	0.16479	-2.6013	0.002622	0.061655	0.53165	-0.91146	0.17652	0.64616	0.82057	-0.2853	0.31598	0.9524	0.91514	-0.12793	0.001533	0.07528	1.4136	0.49938
672.9761	12.01	olomics Ne	0.05133	0.99918	1.2401	0.31047	0.63226	0.91109	0.9492	-0.07522	0.25869	0.99995	1.1363	0.1843	0.64337	0.99985	0.95774	-0.0623	0.69944	0.99927	1.0792	0.1099	0.35789	0.76486	1.0837	0.11592	0.034694	0.3559	1.2152	0.28123	0.26259	0.99512	1.2186	0.28528	0.98146	0.99945	1.0163	0.023377
110.1045	2.05	lomics Pos	0.051448	0.94464	1.3693	0.4534	0.7041	0.99994	1.0469	0.066063	0.87118	0.99996	0.97	-0.04394	0.16114	0.59245	0.72621	-0.46155	1.65E-05	0.00033	0.22465	-2.1543	0.052588	0.22542	0.69269	-0.52972	0.47265	0.808	0.8826	-0.18017	0.47955	0.96776	0.88459	-0.17693	0.079752	0.18143	1.4907	0.57594
366.9806	4.65	olomics Ne	0.051453	0.99918	1.0805	0.11174	0.59034	0.89276	0.97298	-0.03952	0.5168	0.99995	1.0149	0.021379	0.30397	0.99985	1.0664	0.092751	0.6341	0.99927	0.98261	-0.02531	0.10875	0.57882	1.1207	0.16442	0.018168	0.29108	1.1246	0.16939	0.43887	0.99512	1.0651	0.090978	0.12512	0.99941	1.0622	0.087002
680.5182	2.74	lomics Pos	0.051571	0.94464	1.4677	0.55353	0.72007	0.99994	0.9579	-0.06205	0.5034	0.99996	0.88748	-0.17222	0.15956	0.5895	0.72183	-0.47027	4.76E-05	0.000616	0.19309	-2.3726	0.003907	0.068644	0.43387	-1.2047	0.025076	0.33809	0.53509	-0.90214	0.16831	0.91806	0.70779	-0.4986	0.42188	0.52933	1.2161	0.28222
784.9528	3.27	olomics Po	0.051775	0.99951	1.413	0.49877	0.25532	0.99998	0.9137	-0.1302	0.65029	0.99941	1.0615	0.08617	0.004312	0.18109	0.63018	-0.66617	0.000601	0.00636	0.55511	-0.84914	0.000196	0.036471	0.59909	-0.73915	0.67722	0.99391	0.96271	-0.05483	0.73338	0.99956	0.98112	-0.02749	0.030371	0.2688	1.7859	0.83667
738.449	3.22	olomics Po	0.051794	0.99951	1.4684	0.55428	0.086985	0.99998	0.84536	-0.24237	0.89313	0.99941	0.98988	-0.01467	0.002081	0.14926	0.64078	-0.6421	0.000584	0.006262	0.60783	-0.71825	0.009879	0.079508	0.64518	-0.63222	0.71647	0.99391	0.99429	-0.00827	0.63305	0.99956	0.95819	-0.06161	0.020216	0.2688	1.7884	0.83869
149.1336	6	olomics Po	0.052145	0.99951	1.2382	0.3082	0.44447	0.99998	0.91069	-0.13497	0.83048	0.99941	1.0366	0.051908	0.20421	0.64188	0.83507	-0.26002	4.8E-06	0.000244	0.23702	-2.0769	0.011417	0.083709	0.6126	-0.70698	0.14848	0.72068	0.80757	-0.30834	0.80144	0.99956	0.97464	-0.03706	0.35173	0.62856	1.0709	0.098879
730.943	3.2	olomics Po	0.052296	0.99951	1.4739	0.5596	0.29883	0.99998	0.8692	-0.20224	0.88371	0.99941	0.99765	-0.0034	0.036306	0.25997	0.73527	-0.44365	0.008427	0.042335	0.71683	-0.48029	0.002836	0.048023	0.68863	-0.53821	0.86608	0.9951	1.1112	0.15205	0.95097	0.99956	1.0263	0.037461	0.013668	0.2688	1.9169	0.93879
95.0869	6.01	olomics Po	0.052399	0.99951	1.2227	0.29009	0.33434	0.99998	0.90272	-0.14765	0.46086	0.99941	1.0552	0.077585	0.06446	0.33313	0.73746	-0.43936	6.25E-06	0.000265	0.28886	-1.7916	0.001778	0.045425	0.58921	-0.76314	0.047758	0.44092	0.72463	-0.46468	0.14838	0.93461	0.83903	-0.2532	0.83754	0.93529	1.0105	0.015064
150.1364	2.05	lomics Pos	0.052562	0.94464	1.3092	0.38874	0.22601	0.99994	0.88422	-0.17752	0.57266	0.99996	1.0582	0.081602	0.19502	0.64049	0.79629	-0.32863	2E-06	0.000144	0.16598	-2.5909	0.00702	0.081828	0.53573	-0.90042	0.14528	0.60716	0.79529	-0.33045	0.92179	0.9846	1.0286	0.040625	0.005719	0.088649	1.4014	0.48686
776.9498	3.26	olomics Po	0.052751	0.99951	1.5313	0.6148	0.12276	0.99998	0.81203	-0.30039	0.7784	0.99941	1.0207	0.029516	0.010502	0.19754	0.65103	-0.6192	0.005982	0.033413	0.58239	-0.77993	0.022638	0.1111	0.69106	-0.53311	0.50036	0.99391	0.89592	-0.15856	0.6215	0.99956	1.1177	0.16052	0.019898	0.2688	1.733	0.79328
166.1637	5.99	olomics Po	0.052761	0.99951	1.2354	0.30494	0.90054	0.99998	0.9931	-0.00998	0.71786	0.99941	0.96208	-0.05578	0.21026	0.65124	0.83626	-0.25797	0.00011	0.001719	0.30769	-1.7004	0.026859	0.12339	0.63014	-0.66625	0.49257	0.99391	0.91663	-0.12559	0.95633	0.99956	0.99558	-0.00639	0.44373	0.71169	1.0588	0.082369
179.1788	1.67	lomics Pos	0.052919	0.94464	1.3201	0.40069	0.45586	0.99994	0.90102	-0.15037	0.48621	0.99996	1.0777	0.10791	0.15545	0.58395	0.75076	-0.41359	8.59E-06	0.000205	0.1957	-2.3533	0.014873	0.10274	0.57273	-0.80407	0.39106	0.76822	0.87057	-0.19996	0.73297	0.97988	0.95269	-0.06992	0.012551	0.098791	1.422	0.50797
421.8891	10.07	lomics Pos	0.052933	0.94464	1.1019	0.14005	0.54048	0.99994	0.97065	-0.04298	0.97885	0.99996	1.0004	0.000531	0.2941	0.75244	0.92285	-0.11584	0.002456	0.010511	0.77537	-0.36705	0.57895	0.77062	0.94887	-0.07572	0.61531	0.87186	1.0475	0.066974	0.19692	0.92582	1.1142	0.156	0.074929	0.17705	1.2577	0.33079
532.6174	0.53	lomics Pos	0.052965	0.94464	1.4003	0.48576	0.036466	0.99994	0.83407	-0.26176	0.59026	0.99996	0.96576	-0.05026	0.00403	0.17581	0.57879	-0.78888	0.000129	0.001227	0.38989	-1.3589	0.001192	0.043885	0.57231	-0.80514	0.57419	0.85958	0.93786	-0.09255	0.60484	0.97229	0.95946	-0.05971	0.020825	0.10765	2.2477	1.1684
685.9013	3.16	olomics Po	0.053046	0.99951	1.6576	0.72905	0.7999	0.99998	0.98427	-0.02288	0.69931	0.99941	1.0211	0.030065	0.008122	0.19613	0.7141	-0.48581	0.001071	0.009752	0.61542	-0.70036	0.008181	0.074912	0.76321	-0.38985	0.75117	0.99391	1.1423	0.19195	0.67748	0.99956	0.97046	-0.04326	0.04188	0.28426	1.8493	0.88701
296.2582	1.01	lomics Pos	0.053054	0.94464	1.3551	0.43844	0.53923	0.99994	0.9086	-0.13828	0.14542	0.99996	0.78439	-0.35035	0.13494	0.55953	0.7582	-0.39936	3.33E-05	0.000489	0.30959	-1.6916	0.006482	0.079718	0.57828	-0.79017	0.44437	0.80121	0.87756	-0.18843	0.45765	0.96776	1.0971	0.13365	0.019498	0.10555	1.3572	0.44069
199.1894	5.99	olomics Po	0.053083	0.99951	1.2954	0.37343	0.4128	0.99998	0.91428	-0.1293	0.47778	0.99941	1.1339	0.18135	0.27628	0.74895	0.89358	-0.16233	2.94E-06	0.000231	0.30693	-1.704	0.007395	0.071093	0.57532	-0.79757	0.26247	0.87213	0.86177	-0.21463	0.76103	0.99956	0.97641	-0.03444	0.032252	0.2688	1.2521	0.3243
291.2686	2.32	lomics Pos	0.05312	0.94464	1.3184	0.39883	0.39113	0.99994	0.88002	-0.18439	0.14935	0.99996	1.1777	0.23598	0.10969	0.53208	0.7282	-0.4576	3.12E-05	0.000482	0.18785	-2.4124	0.018867	0.11778	0.56878	-0.81406	0.15927	0.63227	0.76015	-0.39564	0.55591	0.96798	1.0972	0.13386	0.008216	0.092094	1.538	0.62104
650.0178	0.53	lomics Pos	0.053173	0.94464	1.501	0.58596	0.40589	0.99994	0.86847	-0.20346	0.16911	0.99996	1.24	0.31036	0.057327	0.41399	0.67215	-0.57314	0.0026	0.010996	0.41167	-1.2804	0.027151	0.14544	0.66373	-0.59134	0.95816	0.98985	1.0346	0.049072	0.88722	0.98023	1.0207	0.02956	0.025157	0.11206	2.3178	1.2127
285.2892	6.38	olomics Po	0.05344	0.99951	1.2608	0.33433	0.54034	0.99998	0.92464	-0.11303	0.42388	0.99941	1.0835	0.11567	0.26827	0.74055	0.8305	-0.26795	4.06E-05	0.00079	0.21052	-2.248	0.054273	0.19718	0.65999	-0.59947	0.35114	0.95959	0.86861	-0.20322	0.98783	0.99956	0.99678	-0.00465	0.14787	0.40018	1.1558	0.20884
281.2673	1.66	lomics Pos	0.053522	0.94464	1.2807	0.35695	0.38638	0.99994	0.90102	-0.15038	0.56214	0.99996	1.0747	0.10398	0.13681	0.56033	0.77898	-0.36035	5.47E-06	0.000159	0.17354	-2.5267	0.01004	0.08787	0.55822	-0.84109	0.23476	0.67693	0.8244	-0.27858	0.86511	0.98023	0.97739	-0.033	0.008857	0.092495	1.4051	0.49069
156.1395	6.38	olomics Po	0.053533	0.99951	1.2158	0.28193	0.39134	0.99998	0.92591	-0.11105	0.56005	0.99941	1.0554	0.077833	0.20746	0.64511	0.83112	-0.26687	8.25E-06	0.000292	0.20263	-2.3031	0.031391	0.13639	0.62743	-0.67247	0.14825	0.72068	0.83278	-0.26399	0.59125	0.99956	0.9557	-0.06537	0.06719	0.30938	1.1574	0.21084
610.3131	0.58	lomics Pos	0.053568	0.94464	1.6076	0.68495	0.13695	0.99994	0.83994	-0.25164	0.91443	0.99996	1.002	0.00289	0.005198	0.17581	0.56353	-0.82743	0.000202	0.001711	0.39844	-1.3276	0.0024	0.061655	0.58857	-0.76471	0.89508	0.97532	1.0253	0.035999	0.87009	0.98023	1.074	0.10303	0.010618	0.094875	2.5449	1.3476
171.1573	2.05	lomics Pos	0.054173	0.94464	1.2939	0.37173	0.822	0.99994	0.97201	-0.04096	0.13712	0.99996	1.1876	0.24802	0.33335	0.79293	0.84231	-0.24758	1.05E-05	0.000234	0.19868	-2.3315	0.022607	0.1318	0.64294	-0.63724	0.58803	0.86163	0.92271	-0.11605	0.47301	0.96776	1.0757	0.10526	0.014343	0.098791	1.4784	0.56408
545.8022	2.95	olomics Po	0.054222	0.99951	1.486	0.57143	0.7317	0.99998	0.95528	-0.066	0.72588	0.99941	1.0606	0.08487	0.1307	0.50419	0.72954	-0.45495	0.004784	0.02865	0.59411	-0.75121	0.070274	0.23583	0.73173	-0.45062	0.9202	0.9951	1.0847	0.11732	0.91118	0.99956	1.0283	0.040321	0.032012	0.2688	1.7753	0.82807
400.8291	9.92	lomics Pos	0.054288	0.94464	1.1488	0.20007	0.40681	0.99994	0.96636	-0.04936	0.59528	0.99996	0.96827	-0.04653	0.40121	0.84516	0.96736	-0.04788	0.000968	0.005435	0.73265	-0.44881	0.17156	0.45871	0.87709	-0.1892	0.46475	0.80647	1.0446	0.062964	0.75781	0.97988	0.98326	-0.02436	0.000703	0.07528	1.2166	0.2829
679.3712	0.54	lomics Pos	0.054409	0.94464	1.4882	0.57358	0.028114	0.99994	0.76797	-0.38087	0.59748	0.99996	1.0275	0.039198	0.000142	0.164	0.58442	-0.77492	0.000979	0.005467	0.4638	-1.1084	7.22E-05	0.019289	0.55307	-0.85448	0.59478	0.8647	0.94442	-0.0825	0.36291	0.96776	0.87419	-0.19399	0.023496	0.11051	2.1533	1.1066
1158.719	10.05	lomics Pos	0.054496	0.94464	1.1276	0.1733	0.71697	0.99994	0.97614	-0.03484	0.80534	0.99996	0.98457	-0.02243	0.34022	0.79697	0.93699	-0.0939	0.021054	0.054463	0.82222	-0.28241	0.46174	0.68833	0.9308	-0.10345	0.34411	0.74613	1.0898	0.12408	0.72844	0.97988	0.97836	-0.03156	0.036045	0.12905	1.2228	0.29013
288.1611	1.3	lomics Pos	0.054749	0.94464	1.1891	0.24985	0.78545	0.99994	0.97308	-0.03937	0.67542	0.99996	1.04	0.056647	0.89764	0.9874	1.0151	0.02158	0.12294	0.20367	0.80384	-0.31502	0.31896	0.5964	1.1308	0.17738	0.025565	0.34083	1.2805	0.35673	0.19867	0.92582	1.1263	0.17159	0.008781	0.092495	1.5611	0.6426
449.9787	4.98	olomics Ne	0.054876	0.99918	1.1317	0.17845	0.34996	0.76325	0.93077	-0.1035	0.31203	0.99995	1.0866	0.11984	0.29255	0.99985	1.1191	0.16231	0.53875	0.99927	1.0534	0.075096	0.046547	0.48405	1.2234	0.29087	0.38379	0.73607	1.0604	0.084629	0.10699	0.99512	1.1255	0.17058	0.11942	0.99941	1.1126	0.15392
1172.733	10.07	lomics Pos	0.055008	0.94464	1.1146	0.15647	0.34713	0.99994	1.0676	0.0944	0.75195	0.99996	0.97882	-0.03089	0.70303	0.96147	0.9807	-0.02812	0.00019	0.001629	0.73627	-0.44169	0.32559	0.60075	0.9286	-0.10688	0.39143	0.76855	1.1168	0.15939	0.71333	0.97988	1.0301	0.042836	0.31486	0.42524	1.1065	0.14602
657.3666	0.58	lomics Pos	0.055265	0.94464	1.5132	0.59762	0.239	0.99994	0.90735	-0.14027	0.66467	0.99996	1.033	0.046822	0.008347	0.19971	0.59452	-0.75021	5.95E-05	0.000728	0.40128	-1.3173	0.000242	0.026314	0.55368	-0.85287	0.79419	0.95091	1.0127	0.018169	0.95911	0.98838	1.1115	0.15244	0.011093	0.094875	2.5375	1.3434
827.0024	3.32	olomics Po	0.055344	0.99951	1.4574	0.5434	0.24765	0.99998	0.8356	-0.25912	0.54892	0.99941	1.0606	0.084876	0.016639	0.20717	0.63416	-0.65709	0.011776	0.055363	0.55541	-0.84838	0.014826	0.091119	0.66392	-0.59092	0.3428	0.95245	0.8238	-0.27964	0.50831	0.99956	0.8925	-0.16407	0.053853	0.2951	1.7618	0.81705
124.1214	2.05	lomics Pos	0.055576	0.94464	1.1781	0.23651	0.19934	0.99994	0.86256	-0.21331	0.36591	0.99996	1.0503	0.070843	0.058934	0.41916	0.74176	-0.43097	0.000113	0.00112	0.196	-2.3511	0.009846	0.087714	0.58594	-0.77117	0.030532	0.35576	0.68174	-0.5527	0.19912	0.92582	0.87588	-0.19119	0.037134	0.13049	1.4002	0.48563
510.6201	3.26	olomics Po	0.055645	0.99951	1.4359	0.52193	0.14448	0.99998	0.8654	-0.20856	0.81972	0.99941	0.98426	-0.02288	0.006322	0.1948	0.64735	-0.62737	0.001925	0.015084	0.60375	-0.72798	0.010071	0.079508	0.63285	-0.66007	0.53007	0.99391	0.91046	-0.13534	0.5929	0.99956	0.93465	-0.09751	0.020838	0.2688	1.8623	0.89706
831.4561	0.55	lomics Pos	0.055912	0.94464	1.376	0.46047	0.31953	0.99994	0.92239	-0.11656	0.43363	0.99996	0.95454	-0.06712	0.002926	0.17581	0.53137	-0.9122	4.93E-05	0.000636	0.36789	-1.4427	1.63E-05	0.019289	0.50684	-0.9804	0.22103	0.66412	0.82309	-0.28087	0.48093	0.96776	0.91532	-0.12765	0.01388	0.098791	2.0631	1.0448
135.0053	0.61	lomics Neg	0.056285	0.99977	1.3663	0.4503	0.60135	0.99904	1.1751	0.23276	0.093965	0.99994	1.295	0.37297	0.040354	0.58182	1.7058	0.77041	0.77221	0.99989	1.0427	0.060378	0.65092	0.99653	1.0794	0.1102	0.73015	0.80449	1.0386	0.054654	0.42293	0.70353	0.88188	-0.18134	0.22765	0.44041	0.85144	-0.23203
747.4271	0.57	lomics Pos	0.056399	0.94464	1.537	0.62011	0.6014	0.99994	0.90079	-0.15074	0.58585	0.99996	1.0701	0.097717	0.084411	0.48766	0.66982	-0.57815	0.007475	0.024633	0.51336	-0.96197	0.0324	0.16174	0.64629	-0.62975	0.39344	0.76955	1.2687	0.34334	0.77362	0.97988	1.2168	0.28307	0.012529	0.098791	2.3228	1.2159
412.857	0.29	olomics Po	0.056618	0.99951	1.1391	0.18789	0.072187	0.99998	1.1942	0.256	0.64558	0.99941	1.0231	0.032907	0.029011	0.24112	1.1519	0.20405	0.005967	0.033387	1.2033	0.26696	0.53174	0.81946	1.0487	0.068587	0.29744	0.91497	1.1468	0.19763	0.014696	0.47452	1.1269	0.17236	0.07575	0.32186	1.1133	0.15479
212.2013	2.76	lomics Pos	0.056646	0.94464	1.3536	0.43681	0.60188	0.99994	0.92663	-0.10994	0.6849	0.99996	1.0567	0.079554	0.14877	0.57647	0.72939	-0.45523	2.13E-05	0.000378	0.16176	-2.6281	0.001597	0.052056	0.46345	-1.1095	0.19311	0.64891	0.79529	-0.33044	0.37602	0.96776	0.87792	-0.18783	0.00373	0.085302	1.6708	0.74057
161.133	1.67	lomics Pos	0.056755	0.94464	1.296	0.3741	0.49075	0.99994	0.9225	-0.11638	0.27791	0.99996	1.1188	0.16197	0.15594	0.58496	0.79111	-0.33805	2.83E-06	0.000144	0.18428	-2.44	0.002742	0.061655	0.549	-0.86512	0.17632	0.64616	0.81035	-0.30339	0.5275	0.96776	0.92463	-0.11306	0.005927	0.089674	1.4356	0.52169
403.7559	5.47	lomics Pos	0.056986	0.94464	0.84259	-0.24709	0.62233	0.99994	0.95774	-0.0623	0.73849	0.99996	0.9727	-0.03993	0.18555	0.62584	0.7981	-0.32536	0.001009	0.005533	0.64756	-0.62691	0.009714	0.087714	0.7153	-0.48338	0.09753	0.53334	0.8394	-0.25256	0.83515	0.97988	0.99515	-0.00701	0.63585	0.72633	1.1223	0.16641
237.2218	1.47	lomics Pos	0.05699	0.94464	1.2608	0.33435	0.40515	0.99994	0.91224	-0.13251	0.74046	0.99996	1.029	0.041191	0.12933	0.55039	0.7557	-0.40411	2.14E-06	0.000144	0.18932	-2.4011	0.003265	0.064159	0.55744	-0.8431	0.19691	0.64891	0.81268	-0.29924	0.53948	0.96776	0.93786	-0.09255	0.006691	0.090221	1.3727	0.457
149.1337	6.38	olomics Po	0.057218	0.99951	1.1821	0.24134	0.50137	0.99998	0.94069	-0.08821	0.94345	0.99941	0.99265	-0.01065	0.11551	0.46351	0.79217	-0.33611	2.91E-06	0.000231	0.26334	-1.925	0.002485	0.046224	0.56769	-0.81683	0.033895	0.36264	0.74438	-0.42589	0.12765	0.90554	0.851	-0.23277	0.72133	0.87685	1.0366	0.051909
107.0868	6	olomics Po	0.057564	0.99951	1.3263	0.40739	0.72681	0.99998	0.94951	-0.07474	0.90774	0.99941	1.0182	0.026015	0.1005	0.4238	0.76101	-0.394	0.000164	0.0024	0.31687	-1.6581	0.098162	0.29561	0.72816	-0.45768	0.46946	0.99391	0.87258	-0.19664	0.98265	0.99956	0.99299	-0.01015	0.38194	0.65621	1.1222	0.16639
738.9508	3.22	olomics Po	0.057587	0.99951	1.3982	0.48361	0.31428	0.99998	0.89956	-0.15271	0.15355	0.99941	0.86102	-0.21588	0.00857	0.19702	0.56206	-0.8312	0.000611	0.006394	0.54723	-0.86979	0.001794	0.045425	0.59509	-0.74882	0.6514	0.99391	0.94991	-0.07414	0.66039	0.99956	0.95092	-0.0726	0.052797	0.2951	1.5866	0.66594
198.1864	6.38	olomics Po	0.057631	0.99951	1.232	0.30104	0.53867	0.99998	0.9503	-0.07355	0.5468	0.99941	1.0659	0.092008	0.30693	0.79641	0.86242	-0.21354	1.5E-05	0.00041	0.22797	-2.1331	0.017691	0.097937	0.61386	-0.70402	0.16694	0.7434	0.82439	-0.27861	0.63092	0.99956	0.96402	-0.05287	0.048074	0.28861	1.1931	0.25469
785.4541	3.27	olomics Po	0.057653	0.99951	1.4488	0.53481	0.04614	0.99998	0.82186	-0.28304	0.50709	0.99941	0.94813	-0.07685	0.006382	0.1948	0.62443	-0.67939	0.000651	0.006703	0.57026	-0.81031	0.000311	0.039399	0.62509	-0.67787	0.71641	0.99391	0.98505	-0.02174	0.67758	0.99956	0.96825	-0.04655	0.073115	0.31731	1.7125	0.77609
424.2002	2.05	lomics Pos	0.057687	0.94464	1.2735	0.34879	0.73917	0.99994	1.0572	0.080303	0.51097	0.99996	1.1011	0.13892	0.76701	0.97526	0.95781	-0.06219	0.000127	0.001209	0.41209	-1.279	0.080558	0.29367	0.79276	-0.33505	0.63769	0.88419	1.119	0.16219	0.45443	0.96776	1.0704	0.098217	0.004567	0.085788	1.5555	0.63734
678.3681	0.56	lomics Pos	0.0577	0.94464	1.4688	0.55468	0.12052	0.99994	0.86131	-0.21539	0.96234	0.99996	0.99229	-0.01116	0.00281	0.17581	0.56069	-0.83473	0.000211	0.00175	0.38767	-1.3671	0.000993	0.043236	0.56581	-0.82162	0.37673	0.76056	0.86912	-0.20238	0.56112	0.9703	0.94231	-0.08573	0.025332	0.11206	2.1738	1.1202
722.5249	2.05	lomics Pos	0.057834	0.94464	1.3943	0.47957	0.42812	0.99994	0.90215	-0.14857	0.24237	0.99996	1.1426	0.19235	0.1994	0.64478	0.75667	-0.40227	2.49E-05	0.000421	0.10143	-3.3014	0.046781	0.20863	0.51377	-0.96082	0.40177	0.77439	0.86216	-0.21397	0.38896	0.96776	1.1044	0.14329	0.000392	0.07528	1.916	0.93806
1185.256	4.33	lomics Pos	0.057992	0.94464	1.3397	0.42186	0.016845	0.99994	0.70597	-0.50233	0.39636	0.99996	1.0844	0.11692	0.018703	0.25561	0.60592	-0.72281	0.022942	0.057932	0.51617	-0.95408	0.13628	0.40368	0.73165	-0.45078	0.33792	0.74343	0.85246	-0.23029	0.80898	0.97988	1.0681	0.095095	0.13758	0.24298	1.5462	0.62868
470.7557	3.02	olomics Po	0.058185	0.99951	1.3449	0.42749	0.16809	0.99998	0.86663	-0.20652	0.93991	0.99941	0.99368	-0.00915	0.002145	0.14926	0.60629	-0.72193	0.054226	0.17849	0.63528	-0.65454	0.007342	0.070977	0.66851	-0.58097	0.89469	0.9951	1.061	0.085467	0.4457	0.99956	0.89578	-0.15878	0.009047	0.2688	2.0269	1.0193
613.3408	2.72	olomics Po	0.058298	0.99951	1.5846	0.66408	0.9691	0.99998	0.99778	-0.0032	0.90407	0.99941	1.0121	0.017394	0.069735	0.3437	0.66427	-0.59016	0.012549	0.057709	0.59778	-0.74231	0.006193	0.065804	0.6385	-0.64724	0.7665	0.99391	1.0292	0.041521	0.92642	0.99956	1.1493	0.2007	0.015798	0.2688	2.473	1.3063
121.1025	6.38	olomics Po	0.058354	0.99951	1.1548	0.20767	0.24392	0.99998	0.88831	-0.17087	0.55284	0.99941	0.96105	-0.05732	0.092364	0.40863	0.77438	-0.36889	2.7E-06	0.000231	0.26888	-1.895	0.001148	0.044437	0.55	-0.8625	0.01723	0.29966	0.72087	-0.47218	0.059305	0.71507	0.8173	-0.29107	0.81919	0.9256	0.98501	-0.02179
362.1922	0.58	lomics Pos	0.058623	0.94464	1.3992	0.4846	0.050224	0.99994	0.75977	-0.39637	0.47248	0.99996	0.90608	-0.14229	0.002593	0.17581	0.53446	-0.90384	0.000607	0.003818	0.39411	-1.3433	7.35E-05	0.019289	0.50086	-0.99752	0.55831	0.85427	0.93589	-0.09559	0.65443	0.97988	0.99944	-0.00081	0.033647	0.12517	2.4245	1.2777
381.2046	6.38	olomics Po	0.058685	0.99951	1.3526	0.43577	0.64009	0.99998	1.0394	0.055683	0.13237	0.99941	1.1244	0.1691	0.93337	0.99905	1.017	0.024355	0.00015	0.002227	0.45893	-1.1237	0.80521	0.94012	0.97834	-0.03159	0.53499	0.99391	1.0949	0.13081	0.031616	0.58535	1.306	0.38517	0.022461	0.2688	1.3401	0.4223
165.1643	2.05	lomics Pos	0.058954	0.94464	1.2149	0.28089	0.32475	0.99994	0.89143	-0.16581	0.7692	0.99996	1.0314	0.044551	0.16791	0.60254	0.77221	-0.37293	1.58E-06	0.000144	0.15704	-2.6708	0.003423	0.065592	0.52468	-0.93048	0.13916	0.60403	0.79584	-0.32945	0.87755	0.98023	0.98614	-0.02014	0.00774	0.091944	1.357	0.44044
354.1871	1.65	lomics Pos	0.059001	0.94464	1.3303	0.4117	0.92849	0.99994	1.0316	0.044821	0.25088	0.99996	1.1428	0.19259	0.84447	0.98707	0.98869	-0.01641	1.43E-05	0.000291	0.37133	-1.4292	0.031701	0.16072	0.74466	-0.42536	0.16572	0.63508	0.85937	-0.21864	0.34446	0.96524	1.1216	0.16562	0.002661	0.082646	1.5256	0.60939
707.9128	3.19	olomics Po	0.059158	0.99951	1.3378	0.41983	0.12622	0.99998	0.83457	-0.2609	0.95238	0.99941	1.0045	0.006495	0.024128	0.22735	0.61545	-0.70028	0.0017	0.013697	0.58069	-0.78416	0.002181	0.045856	0.60857	-0.71649	0.59148	0.99391	0.94194	-0.08629	0.52647	0.99956	0.92082	-0.11902	0.0195	0.2688	1.8464	0.8847
113.0966	2.05	lomics Pos	0.059195	0.94464	1.3773	0.4618	0.32699	0.99994	0.8191	-0.28788	0.40999	0.99996	1.0947	0.13052	0.17703	0.61055	0.75273	-0.40979	0.000124	0.001188	0.24361	-2.0374	0.016166	0.10794	0.5311	-0.91295	0.4156	0.78082	0.85014	-0.23423	0.95825	0.98838	1.0083	0.011991	0.063321	0.16148	1.4137	0.49949
247.2432	6.38	olomics Po	0.059254	0.99951	1.2171	0.28344	0.30338	0.99998	0.91776	-0.1238	0.55565	0.99941	1.0591	0.082905	0.26924	0.74055	0.86196	-0.21432	6.09E-06	0.000265	0.22656	-2.142	0.014451	0.090678	0.63335	-0.65893	0.16022	0.73694	0.83659	-0.25741	0.58773	0.99956	0.95892	-0.06052	0.031665	0.2688	1.1764	0.23438
853.47	0.54	lomics Pos	0.059268	0.94464	1.3981	0.48342	0.3379	0.99994	0.9281	-0.10764	0.63638	0.99996	0.96932	-0.04496	0.0037	0.17581	0.5252	-0.92907	3.26E-05	0.000488	0.39741	-1.3313	0.000127	0.023299	0.5286	-0.91974	0.30919	0.72874	0.85757	-0.22167	0.49537	0.96776	0.91966	-0.12082	0.009169	0.092545	2.0601	1.0427
302.246	1.66	lomics Pos	0.059276	0.94464	1.2491	0.3209	0.18695	0.99994	0.85701	-0.22261	0.81396	0.99996	1.0336	0.047645	0.12255	0.54337	0.77532	-0.36714	4.1E-06	0.000146	0.1782	-2.4884	0.011247	0.091488	0.54722	-0.8698	0.12639	0.58627	0.78251	-0.35382	0.75036	0.97988	0.96311	-0.05423	0.01739	0.10077	1.3327	0.41431
238.2173	1.66	lomics Pos	0.059561	0.94464	1.3464	0.42907	0.50649	0.99994	0.88971	-0.16859	0.36504	0.99996	1.1354	0.18315	0.14534	0.57193	0.75108	-0.41296	2.92E-05	0.000466	0.1937	-2.3681	0.019802	0.12081	0.59364	-0.75235	0.38338	0.76429	0.84804	-0.2378	0.40652	0.96776	0.87644	-0.19028	0.020579	0.10749	1.6155	0.69198
228.2331	5.49	olomics Po	0.060035	0.99951	1.2372	0.30706	0.30593	0.99998	0.90512	-0.14381	0.61083	0.99941	1.0658	0.091878	0.1966	0.63016	0.83018	-0.26851	1.62E-05	0.000428	0.28976	-1.7871	0.010037	0.079508	0.6134	-0.70509	0.14606	0.71777	0.82345	-0.28024	0.28215	0.99956	0.9108	-0.1348	0.030661	0.2688	1.1822	0.24152
299.3152	7.12	olomics Po	0.060225	0.99951	0.82698	-0.27407	0.069217	0.99998	0.76768	-0.38142	0.1101	0.99941	0.78719	-0.34521	0.050657	0.29486	0.83818	-0.25467	0.86096	0.99688	1.0166	0.023774	0.006603	0.067315	0.61993	-0.68983	0.11251	0.64238	0.72693	-0.46012	0.007843	0.47452	0.72601	-0.46194	0.06289	0.30404	0.79021	-0.3397
276.2268	1.46	lomics Pos	0.060227	0.94464	1.2707	0.34563	0.13912	0.99994	0.83376	-0.2623	0.72145	0.99996	1.051	0.071742	0.076249	0.4637	0.75629	-0.40299	1.4E-05	0.000288	0.21652	-2.2074	0.004593	0.071311	0.56034	-0.83562	0.1961	0.64891	0.81218	-0.30012	0.96704	0.99017	0.99545	-0.00658	0.010975	0.094875	1.4188	0.50465
264.2409	1.66	lomics Pos	0.06023	0.94464	1.2586	0.33182	0.28902	0.99994	0.86856	-0.2033	0.66605	0.99996	1.0523	0.073492	0.12768	0.55024	0.75929	-0.39728	8.18E-06	0.0002	0.17766	-2.4928	0.012068	0.093	0.54775	-0.86842	0.25982	0.69475	0.82997	-0.26888	0.63132	0.97988	0.93782	-0.09263	0.014681	0.098791	1.3747	0.45911
533.6212	0.53	lomics Pos	0.060313	0.94464	1.474	0.55978	0.59941	0.99994	0.95619	-0.06463	0.84303	0.99996	1.035	0.049688	0.006927	0.19397	0.61203	-0.70832	0.000321	0.002336	0.43833	-1.1899	0.008111	0.084185	0.58089	-0.78367	0.81442	0.95934	1.0128	0.018405	0.74547	0.97988	1.0068	0.009803	0.013183	0.098791	2.381	1.2516
394.2541	2.77	lomics Pos	0.060327	0.94464	1.1787	0.23722	0.091257	0.99994	0.80068	-0.3207	0.034935	0.99996	0.82412	-0.27908	0.017731	0.24947	0.58482	-0.77394	3.43E-06	0.000144	0.26799	-1.8997	0.000356	0.028292	0.41566	-1.2665	0.00647	0.3088	0.60339	-0.72883	0.014759	0.52955	0.66871	-0.58055	0.6736	0.75698	1.0461	0.065052
653.3357	0.58	lomics Pos	0.060455	0.94464	1.5458	0.62832	0.5538	0.99994	0.95102	-0.07246	0.3973	0.99996	1.0638	0.089193	0.013655	0.22898	0.60962	-0.71402	5.8E-05	0.000716	0.42286	-1.2418	0.000177	0.024979	0.61214	-0.70807	0.84212	0.96746	1.0192	0.027488	0.93579	0.98616	1.1563	0.20946	0.008853	0.092495	2.5795	1.3671
681.1623	1.29	lomics Pos	0.060582	0.94464	1.1252	0.17014	0.43313	0.99994	1.0787	0.10924	0.1992	0.99996	1.147	0.19783	0.15386	0.58092	0.88758	-0.17205	0.034701	0.079184	0.74948	-0.41604	0.16022	0.44206	0.85134	-0.23219	0.4489	0.80156	1.0701	0.097752	0.78476	0.97988	1.0543	0.07625	0.024436	0.11132	1.2637	0.33762
198.1856	2.76	lomics Pos	0.060616	0.94464	1.3391	0.42131	0.49978	0.99994	0.88332	-0.17899	0.99952	0.99996	0.98534	-0.0213	0.1335	0.55754	0.70829	-0.49759	1.78E-05	0.000338	0.15204	-2.7174	0.004586	0.071311	0.48891	-1.0324	0.15303	0.62341	0.72548	-0.46299	0.55007	0.96776	0.89466	-0.16058	0.024812	0.1117	1.4896	0.57492
224.2014	1.66	lomics Pos	0.06086	0.94464	1.2981	0.37646	0.50281	0.99994	0.91971	-0.12075	0.35825	0.99996	1.1132	0.15474	0.13254	0.55697	0.74206	-0.4304	5.48E-06	0.000159	0.16402	-2.6081	0.007983	0.084185	0.53021	-0.91536	0.37065	0.76056	0.86141	-0.21522	0.65217	0.97988	0.94356	-0.08381	0.011582	0.096218	1.4133	0.4991
921.5206	0.57	lomics Pos	0.061032	0.94464	1.4889	0.57427	0.7422	0.99994	0.96242	-0.05526	0.22181	0.99996	1.1357	0.18361	0.007975	0.19917	0.58318	-0.77799	9.2E-05	0.000972	0.45121	-1.1481	0.002762	0.061655	0.59268	-0.75467	0.90295	0.97756	1.0801	0.11113	0.91612	0.98252	1.0636	0.08898	0.011735	0.096289	2.7598	1.4646
941.5301	4.85	lomics Pos	0.06125	0.94464	0.8366	-0.2574	0.51846	0.99994	0.92152	-0.11792	0.51918	0.99996	0.95447	-0.06723	0.82024	0.98382	0.98297	-0.02479	0.011014	0.033083	0.71786	-0.47823	0.87604	0.93567	0.99486	-0.00743	0.73079	0.92698	0.97152	-0.04169	0.94977	0.98838	1.0081	0.011602	0.71206	0.78595	0.97194	-0.04106
951.5376	10.38	olomics Ne	0.06135	0.99918	1.1404	0.18951	0.003187	0.34605	0.78422	-0.35067	0.059364	0.99995	1.0845	0.11699	0.80432	0.99985	1.0352	0.049876	0.33756	0.99927	0.93102	-0.10312	0.022935	0.43126	1.2737	0.34901	0.96023	0.98872	1.0052	0.007523	0.33384	0.99512	1.044	0.062162	0.66884	0.99941	0.97811	-0.03193
254.2483	1.47	lomics Pos	0.061496	0.94464	1.2548	0.32743	0.40812	0.99994	0.90966	-0.1366	0.51188	0.99996	1.0708	0.09865	0.14417	0.57187	0.78281	-0.35326	8.61E-06	0.000205	0.20215	-2.3065	0.00885	0.08583	0.58724	-0.76797	0.31057	0.72948	0.85273	-0.22984	0.72411	0.97988	0.9598	-0.05919	0.009921	0.094875	1.3718	0.4561
919.546	8.55	olomics Ne	0.061695	0.99918	1.157	0.21043	0.68057	0.93851	0.97223	-0.04064	0.77713	0.99995	1.0296	0.042088	0.32984	0.99985	1.1785	0.2369	0.38564	0.99927	1.072	0.10031	0.072266	0.51869	1.1716	0.22851	0.22966	0.61014	1.138	0.18648	0.04943	0.99512	1.1843	0.24398	0.11721	0.99941	1.1258	0.17099
280.2641	1.66	lomics Pos	0.061755	0.94464	1.2523	0.32462	0.27562	0.99994	0.8854	-0.1756	0.60654	0.99996	1.0583	0.081757	0.1095	0.53208	0.77314	-0.37121	3.33E-06	0.000144	0.18629	-2.4244	0.009294	0.086818	0.56262	-0.82978	0.19925	0.64891	0.82015	-0.28605	0.79112	0.97988	0.97082	-0.04272	0.006154	0.090084	1.3862	0.47112
693.8393	10.05	lomics Pos	0.061795	0.94464	1.1359	0.18382	0.76942	0.99994	1.0239	0.034026	0.31066	0.99996	0.93985	-0.0895	0.059596	0.42081	0.8913	-0.16602	0.039072	0.086869	0.87234	-0.19703	0.18104	0.46866	0.9055	-0.14322	0.27793	0.7084	1.0938	0.12932	0.81685	0.97988	1.013	0.018665	0.013038	0.098791	1.2219	0.28918
565.5573	6.37	olomics Po	0.061843	0.99951	1.3261	0.40722	0.51771	0.99998	0.90634	-0.14187	0.4934	0.99941	1.1625	0.21717	0.23674	0.69626	0.75786	-0.39999	5.08E-05	0.000947	0.1307	-2.9356	0.007174	0.070405	0.47856	-1.0632	0.50727	0.99391	0.8902	-0.1678	0.38067	0.99956	0.87086	-0.19948	0.024927	0.2688	1.4096	0.49529
142.1239	6.38	olomics Po	0.061845	0.99951	1.2125	0.27794	0.35797	0.99998	0.91596	-0.12664	0.69984	0.99941	1.0386	0.054624	0.20228	0.64038	0.82651	-0.2749	8.92E-06	0.000293	0.21502	-2.2174	0.026051	0.12085	0.61187	-0.70869	0.11426	0.64271	0.81745	-0.2908	0.48085	0.99956	0.94771	-0.07748	0.078928	0.32476	1.1285	0.17445
97.1019	2.05	lomics Pos	0.061947	0.94464	1.2825	0.35891	0.60315	0.99994	0.9454	-0.081	0.49129	0.99996	1.0841	0.11655	0.29411	0.75244	0.8449	-0.24315	4.31E-07	0.000144	0.17179	-2.5413	0.009888	0.08787	0.58053	-0.78455	0.25358	0.69047	0.84972	-0.23493	0.82184	0.97988	1.0203	0.028977	0.001585	0.07528	1.5495	0.63179
207.6707	2.76	lomics Pos	0.062285	0.94464	1.3313	0.4128	0.64865	0.99994	0.93602	-0.09539	0.99742	0.99996	1.026	0.037031	0.44373	0.86677	0.87439	-0.19365	0.000253	0.00197	0.24546	-2.0264	0.067969	0.26499	0.76678	-0.38312	0.52111	0.83957	1.1072	0.14685	0.51042	0.96776	1.0789	0.10958	0.000622	0.07528	1.8908	0.91902
518.9732	5.2	olomics Ne	0.0627	0.99918	1.0524	0.073717	0.087582	0.50704	0.92135	-0.11818	0.043372	0.99995	1.06	0.084018	0.92767	0.99985	1.0058	0.008313	0.39463	0.99927	1.032	0.045421	0.071286	0.51869	1.0914	0.12615	0.048554	0.39082	1.0922	0.12724	0.80565	0.99512	1.0136	0.019557	0.7051	0.99941	1.0216	0.030809

786.4191	0.58	domics Pos	0.062721	0.94464	1.4071	0.49271	0.057571	0.99994	0.91275	-0.1317	0.78573	0.99996	1.019	0.027171	0.003663	0.17581	0.63349	-0.65861	3.34E-05	0.000489	0.46819	-1.0948	2.24E-05	0.019289	0.65241	-0.61615	0.57884	0.86163	0.95005	-0.07393	0.87912	0.98023	1.0641	0.089672	0.014367	0.098791	2.4427	1.2885
107.9675	9.82	domics Pos	0.062853	0.94464	1.1461	0.19675	0.75805	0.99994	1.0334	0.047388	0.79937	0.99996	1.0115	0.016485	0.50526	0.89133	0.97971	-0.02958	0.000147	0.001354	0.77347	-0.37057	0.44513	0.67654	0.93125	-0.10276	0.027388	0.34512	1.1016	0.1396	0.63453	0.97988	0.97388	-0.03819	0.001598	0.07528	1.2177	0.28415
634.3507	3.08	oolomics Po	0.062984	0.99951	1.4269	0.51285	0.27112	0.99998	0.94624	-0.07972	0.55252	0.99941	0.95302	-0.06943	0.013388	0.20085	0.65474	-0.61101	0.002373	0.017364	0.61537	-0.70047	0.000224	0.038868	0.57617	-0.79543	0.56019	0.99391	0.93981	-0.08955	0.50561	0.99956	0.9235	-0.11481	0.002494	0.2688	1.9893	0.99227
393.296	7.64	oolomics Po	0.062988	0.99951	0.84823	-0.23748	0.13022	0.99998	0.88314	-0.17928	0.036888	0.99941	0.87067	-0.19981	0.13302	0.50789	0.86533	-0.20867	0.21317	0.51907	0.91385	-0.12997	0.36447	0.67617	0.94581	-0.08038	0.27346	0.89369	0.89838	-0.1546	0.29327	0.99956	0.89375	-0.16206	0.14443	0.3963	0.8606	-0.21658
895.105	4.71	domics Pos	0.063347	0.94464	1.2897	0.36708	0.29078	0.99994	0.92099	-0.11874	0.72297	0.99996	1.058	0.081311	0.045133	0.36714	0.776	-0.36587	0.000398	0.002754	0.5128	-0.96353	0.019737	0.1206	0.63553	-0.65397	0.89216	0.97532	1.0041	0.00597	0.84363	0.97988	1.0961	0.13236	0.008163	0.092094	1.9484	0.96229
97.1005	1.66	domics Pos	0.063602	0.94464	1.2103	0.27542	0.44673	0.99994	0.92911	-0.10608	0.84663	0.99996	1.0091	0.013036	0.1131	0.53238	0.79213	-0.33619	4.59E-06	0.000149	0.24177	-2.0483	0.018873	0.11778	0.62686	-0.67379	0.12538	0.58587	0.79978	-0.32232	0.75142	0.97988	0.96989	-0.04411	0.003654	0.085302	1.5093	0.59385
398.9777	4.71	olomics Ne	0.063729	0.99918	1.0623	0.087196	0.25278	0.67504	0.93348	-0.0993	0.35687	0.99995	1.0401	0.056705	0.81372	0.99985	1.0229	0.032732	0.97737	0.99927	1.0005	0.000781	0.003848	0.36417	1.1383	0.18688	0.013459	0.2716	1.1174	0.16013	0.21503	0.99512	1.0805	0.11169	0.75201	0.99941	1.0146	0.020874
1024.75	10.05	domics Pos	0.063753	0.94464	1.1322	0.17916	0.30518	0.99994	0.9474	-0.07795	0.5912	0.99996	0.96463	-0.05195	0.15288	0.58077	0.92086	-0.11895	0.001145	0.006059	0.77405	-0.36949	0.21221	0.50393	0.89906	-0.15352	0.09025	0.51907	1.1898	0.25073	0.88411	0.98023	1.0302	0.042965	0.016208	0.099614	1.2418	0.31248
321.9819	11.99	olomics Ne	0.063786	0.99918	0.77233	-0.37271	0.038138	0.45581	0.7556	-0.40431	0.21727	0.99995	0.77621	-0.36548	0.78379	0.99985	0.97509	-0.03639	0.82529	0.99927	1.1023	0.1405	0.19585	0.65203	1.1534	0.20595	0.59625	0.86189	0.93648	-0.09468	0.20621	0.99512	0.83085	-0.26734	0.37318	0.99941	0.88626	-0.17421
563.5512	6.38	oolomics Po	0.063867	0.99951	1.3032	0.38203	0.5999	0.99998	0.93202	-0.10156	0.68285	0.99941	1.1047	0.14369	0.25143	0.71739	0.78313	-0.35267	1.94E-05	0.000473	0.095564	-3.3874	0.010304	0.080315	0.44268	-1.1757	0.14652	0.7187	0.76888	-0.37916	0.17811	0.96992	0.82638	-0.27512	0.039206	0.28339	1.3511	0.43412
470.9733	12	olomics Ne	0.063877	0.99918	1.2259	0.2939	0.81724	0.9788	0.98784	-0.01765	0.87135	0.99995	1.0437	0.061772	0.069813	0.99985	1.1478	0.1989	0.76807	0.99927	1.0442	0.062398	0.021183	0.43126	1.1804	0.23928	0.17891	0.55116	1.1377	0.18617	0.33927	0.99512	1.1693	0.22561	0.74957	0.99941	1.0187	0.026679
692.4061	2.74	oolomics Po	0.064029	0.99951	1.4929	0.57813	0.24574	0.99998	0.87808	-0.18758	0.91389	0.99941	0.9978	-0.00318	0.025503	0.23218	0.61968	-0.69041	0.001442	0.012324	0.54047	-0.88773	0.002222	0.045856	0.6481	-0.62572	0.98354	0.99744	1.1389	0.18761	0.8913	0.99956	1.1814	0.24044	0.040179	0.28339	2.4038	1.2653
382.2	2.81	oolomics Po	0.064045	0.99951	1.4389	0.52501	0.046506	0.99998	0.79274	-0.33507	0.96922	0.99941	0.99787	-0.00308	0.007108	0.1948	0.5891	-0.76342	0.000815	0.008025	0.46122	-1.1165	0.015722	0.093567	0.62822	-0.67065	0.89526	0.9951	1.0347	0.049271	0.89409	0.99956	1.0539	0.075702	0.021565	0.2688	2.3234	1.2162
765.4175	0.55	domics Pos	0.064184	0.94464	1.3973	0.48267	0.16277	0.99994	0.88737	-0.17239	0.76748	0.99996	0.97691	-0.0337	0.004737	0.17581	0.577	-0.79336	9.16E-05	0.000972	0.39318	-1.3468	0.000404	0.031428	0.54861	-0.86614	0.33146	0.73996	0.84924	-0.23575	0.53801	0.96776	0.92958	-0.10535	0.017324	0.10064	2.076	1.0538
1190.724	10.06	domics Pos	0.064224	0.94464	1.1739	0.23136	0.47754	0.99994	0.94547	-0.0809	0.36845	0.99996	1.0819	0.1136	0.49219	0.88874	0.94943	-0.07486	0.014163	0.039973	0.79894	-0.32384	0.47299	0.69553	0.93033	-0.10418	0.1611	0.63418	1.132	0.1789	0.73646	0.97988	1.0605	0.084722	0.1287	0.23369	1.1811	0.2401
209.1909	6.38	oolomics Po	0.064226	0.99951	1.2447	0.31583	0.40361	0.99998	0.90036	-0.15143	0.88632	0.99941	1.0091	0.013135	0.10002	0.42289	0.74986	-0.41531	1.83E-05	0.000458	0.22569	-2.1476	0.009046	0.076393	0.54951	-0.86379	0.11791	0.65162	0.79335	-0.33398	0.42624	0.99956	0.91116	-0.13422	0.096096	0.34969	1.1901	0.25106
109.1019	1.67	domics Pos	0.064326	0.94464	1.2294	0.2979	0.23221	0.99994	0.86415	-0.21064	0.5687	0.99996	1.0637	0.089119	0.10256	0.52635	0.73436	-0.44544	3.39E-06	0.000144	0.17851	-2.4859	0.007998	0.084185	0.53448	-0.90381	0.32134	0.73067	0.84361	-0.24535	0.4446	0.96776	0.91331	-0.13082	0.011094	0.094875	1.3904	0.47549
223.2055	6.38	oolomics Po	0.064337	0.99951	1.2473	0.31886	0.15873	0.99998	0.87457	-0.19335	0.96617	0.99941	1.016	0.02289	0.25261	0.72012	0.82873	-0.27103	1.67E-06	0.000231	0.20811	-2.2646	0.005801	0.064803	0.59261	-0.75484	0.025777	0.33277	0.78932	-0.34132	0.20768	0.99956	0.90003	-0.15195	0.18074	0.44542	1.1271	0.17259
170.1546	2.05	domics Pos	0.064906	0.94464	1.2326	0.30168	0.20146	0.99994	0.88302	-0.17948	0.59201	0.99996	1.0388	0.054919	0.13753	0.56146	0.77322	-0.37105	2.64E-06	0.000144	0.155	-2.6896	0.004475	0.071311	0.53162	-0.91154	0.16367	0.63418	0.81822	-0.28945	0.54788	0.96776	0.9463	-0.07962	0.003177	0.085302	1.3585	0.44199
246.2304	1.66	domics Pos	0.06505	0.94464	1.2598	0.33315	0.40902	0.99994	0.89692	-0.15695	0.62785	0.99996	1.0669	0.093423	0.17016	0.60402	0.77228	-0.3728	7.41E-06	0.000191	0.17658	-2.5016	0.007677	0.082461	0.53264	-0.90876	0.16633	0.63634	0.77604	-0.3658	0.68515	0.97988	0.94761	-0.07764	0.016673	0.099614	1.3902	0.47525
1139.765	10.02	domics Pos	0.065139	0.94464	1.1031	0.14157	0.18996	0.99994	0.93494	-0.09705	0.12972	0.99996	0.90263	-0.14779	0.11849	0.53907	0.9068	-0.14114	0.000841	0.004885	0.78039	-0.35772	0.072406	0.27658	0.83779	-0.25534	0.9858	0.99467	1.0041	0.005963	0.17309	0.91806	0.94923	-0.07516	0.31732	0.42784	1.1241	0.16882
499.9059	10.02	domics Pos	0.065156	0.94464	1.1464	0.19706	0.44662	0.99994	0.93615	-0.09519	0.635	0.99996	1.0416	0.058796	0.4268	0.86135	0.93405	-0.09843	0.009193	0.028921	0.78817	-0.34342	0.97463	0.9856	1.0056	0.008016	0.58384	0.86163	1.0427	0.06032	0.97372	0.99223	1.0002	0.000266	0.057615	0.15492	1.2305	0.29927
165.1638	6	oolomics Po	0.065256	0.99951	1.1695	0.22586	0.42144	0.99998	0.91734	-0.12447	0.97407	0.99941	1.0073	0.010471	0.083057	0.38083	0.73138	-0.4513	8.91E-06	0.000293	0.22613	-2.1448	0.003957	0.053954	0.55615	-0.84645	0.053755	0.46139	0.75315	-0.40899	0.45415	0.99956	0.92113	-0.11852	0.25417	0.52466	1.0952	0.13119
457.9822	12.02	olomics Ne	0.065259	0.99918	1.2163	0.28253	0.98954	0.99664	1.0198	0.028239	0.072661	0.99995	1.2021	0.26551	0.058171	0.99985	1.2175	0.28395	0.036529	0.99927	1.3801	0.46476	0.090801	0.55533	1.2965	0.37458	0.24358	0.62946	1.2078	0.27234	0.67587	0.99512	0.97423	-0.03767	0.008499	0.99941	1.525	0.60886
264.2414	5.99	oolomics Po	0.065815	0.99951	1.2036	0.26732	0.43309	0.99998	0.92605	-0.11084	0.73739	0.99941	1.0451	0.063629	0.15042	0.5457	0.79789	-0.32574	6.93E-06	0.000278	0.2185	-2.1943	0.012747	0.085685	0.59925	-0.73877	0.1079	0.62943	0.79434	-0.33218	0.2919	0.99956	0.91787	-0.12364	0.044231	0.28476	1.1763	0.2343
677.3704	3.14	oolomics Po	0.065994	0.99951	1.3857	0.47061	0.091177	0.99998	0.84467	-0.24355	0.80401	0.99941	1.0203	0.028956	0.009507	0.19702	0.58844	-0.76503	6.63E-05	0.00118	0.54685	-0.87078	0.003316	0.049867	0.64391	-0.63508	0.85869	0.9951	1.0808	0.11216	0.69414	0.99956	0.97448	-0.0373	0.023214	0.2688	1.7771	0.82952
406.2259	2.96	oolomics Po	0.06607	0.99951	1.3829	0.46772	0.16332	0.99998	0.8574	-0.22196	0.94589	0.99941	0.99794	-0.00297	0.0228	0.22443	0.6788	-0.55893	0.012528	0.057694	0.63252	-0.66082	0.012703	0.085685	0.6738	-0.56962	0.79148	0.99391	1.0271	0.038576	0.99663	0.99956	1.0888	0.12279	0.011588	0.2688	2.049	1.0349
153.1286	6.38	oolomics Po	0.066105	0.99951	1.1764	0.23435	0.3964	0.99998	0.93489	-0.09713	0.58144	0.99941	0.95689	-0.06357	0.12637	0.49162	0.8058	-0.3115	1.96E-06	0.000231	0.28104	-1.8312	0.002352	0.04601	0.56673	-0.81926	0.023087	0.32178	0.72266	-0.46861	0.076186	0.77466	0.81178	-0.30085	0.97511	0.98829	1.0056	0.00808
226.2176	6.38	oolomics Po	0.066231	0.99951	1.2263	0.29431	0.35164	0.99998	0.9166	-0.12564	0.59938	0.99941	1.0512	0.072048	0.25581	0.72727	0.8437	-0.2452	8.45E-06	0.000292	0.20916	-2.2573	0.022358	0.1103	0.60701	-0.7202	0.14152	0.70661	0.81198	-0.30049	0.54224	0.99956	0.94896	-0.07558	0.088981	0.3414	1.1478	0.19887
210.1862	5.99	oolomics Po	0.066339	0.99951	1.2361	0.30577	0.31294	0.99998	0.90577	-0.14279	0.57157	0.99941	1.0629	0.088026	0.15599	0.55586	0.79304	-0.33453	7.68E-07	0.000231	0.23787	-2.0717	0.010718	0.08175	0.5844	-0.77496	0.13402	0.68208	0.77862	-0.36101	0.32442	0.99956	0.90839	-0.13861	0.15292	0.40935	1.1155	0.15764
156.1394	5.99	oolomics Po	0.066539	0.99951	1.1915	0.25283	0.3069	0.99998	0.89966	-0.15254	0.61519	0.99941	1.059	0.08264	0.13397	0.51028	0.78786	-0.344	1.28E-05	0.000369	0.22558	-2.1483	0.015754	0.093567	0.5933	-0.75316	0.16898	0.74367	0.79166	-0.33705	0.6754	0.99956	0.96206	-0.05581	0.10376	0.35814	1.137	0.18529
284.2862	2.05	domics Pos	0.066558	0.94924	1.2362	0.30591	0.31459	0.99994	0.89653	-0.15758	0.60212	0.99996	1.0485	0.068282	0.13247	0.55697	0.78361	-0.35179	2.8E-06	0.000144	0.17268	-2.5338	0.007623	0.082308	0.56151	-0.83262	0.22099	0.66412	0.83275	-0.26404	0.6913	0.97988	0.96023	-0.05854	0.004572	0.085788	1.3842	0.46906
248.2465	6.38	oolomics Po	0.066687	0.99951	1.1995	0.26244	0.35908	0.99998	0.92198	-0.11719	0.52422	0.99941	1.0667	0.093099	0.22364	0.67157	0.8427	-0.2469	4.26E-06	0.000231	0.21475	-2.2193	0.017554	0.097645	0.62927	-0.66824	0.17705	0.75029	0.84166	-0.24869	0.54492	0.99956	0.95222	-0.07063	0.062831	0.30404	1.1742	0.23171
371.2008	2.8	oolomics Po	0.066714	0.99951	1.6128	0.68955	0.3768	0.99998	0.86117	-0.21562	0.39243	0.99941	1.0975	0.13425	0.11154	0.4528	0.725	-0.46394	0.003471	0.022587	0.53417	-0.90463	0.06034	0.21211	0.68766	-0.54023	0.73217	0.99391	1.1205	0.16408	0.98265	0.99956	1.0978	0.13463	0.036195	0.27637	2.28	1.189
741.3872	0.58	domics Pos	0.066924	0.94924	1.4982	0.58319	0.077516	0.99994	0.87096	-0.19932	0.42967	0.99996	1.0489	0.068945	0.003807	0.17581	0.59248	-0.75517	7.97E-05	0.000883	0.42384	-1.2384	0.000269	0.026314	0.57358	-0.80193	0.76871	0.94339	0.99892	-0.00156	0.9022	0.98023	1.1477	0.1987	0.010999	0.094875	2.4987	1.3212
622.3479	3.08	oolomics Po	0.067064	0.99951	1.5737	0.65412	0.56783	0.99998	0.95312	-0.06927	0.96259	0.99941	0.99874	-0.00181	0.007668	0.1948	0.60578	-0.72313	0.004875	0.028888	0.62128	-0.68668	0.00135	0.045425	0.61873	-0.69262	0.80096	0.99391	0.98972	-0.01491	0.69852	0.99956	0.97168	-0.04145	0.004258	0.2688	2.0218	1.0156
95.0863	2.05	domics Pos	0.067095	0.94924	1.2747	0.35017	0.34695	0.99994	0.88486	-0.17647	0.43821	0.99996	1.085	0.11774	0.1597	0.5895	0.76337	-0.38954	4.22E-06	0.000147	0.15679	-2.6731	0.01264	0.095104	0.55987	-0.83683	0.25522	0.69047	0.83815	-0.25473	0.98437	0.99507	0.99959	-0.00059	0.003844	0.085302	1.4457	0.5318
244.2137	4.73	domics Pos	0.067205	0.94924	0.80274	-0.317	0.33647	0.99994	0.89804	-0.15516	0.12402	0.99996	0.81197	-0.3005	0.60409	0.93327	0.93308	-0.09993	0.011608	0.034397	0.66798	-0.58213	0.28918	0.57347	0.83897	-0.25332	0.088517	0.51701	0.7833	-0.35237	0.69583	0.97988	0.95176	-0.07133	0.60414	0.69657	1.0951	0.13103
443.9783	4.99	olomics Ne	0.067447	0.99918	1.0703	0.097958	0.25577	0.67565	0.94607	-0.07999	0.14379	0.99995	1.0795	0.11038	0.84495	0.99985	0.99217	-0.01135	0.46308	0.99927	1.021	0.029988	0.29214	0.71639	1.0926	0.12771	0.030695	0.34429	1.1113	0.15224	0.84731	0.99512	1.0117	0.016817	0.88904	0.99941	1.0051	0.007322
210.1928	6.38	oolomics Po	0.067775	0.99951	1.1858	0.24581	0.43214	0.99998	0.93136	-0.10258	0.98455	0.99941	1.006	0.008586	0.1041	0.4326	0.78705	-0.34548	3.52E-05	0.000701	0.30765	-1.7006	0.005123	0.061592	0.62	-0.68966	0.0969	0.60726	0.78943	-0.34111	0.091924	0.80182	0.88047	-0.18365	0.060441	0.30404	1.1256	0.17068
267.2602	6.38	oolomics Po	0.067856	0.99951	1.293	0.37072	0.63701	0.99998	0.94185	-0.08644	0.3454	0.99941	1.1034	0.14197	0.37556	0.87115	0.86244	-0.21351	2.23E-05	0.000513	0.2114	-2.2419	0.040473	0.16158	0.64774	-0.62652	0.37522	0.97118	0.87698	-0.18939	0.81587	0.99956	0.97201	-0.04095	0.065237	0.30704	1.2106	0.27578
137.1336	6.39	oolomics Po	0.067881	0.99951	1.1882	0.24873	0.60713	0.99998	0.94801	-0.07703	0.6575	0.99941	0.96007	-0.05879	0.15268	0.54839	0.80465	-0.31357	3.71E-06	0.000231	0.24856	-2.0083	0.003075	0.049681	0.60445	-0.72631	0.034933	0.37176	0.75414	-0.4071	0.12786	0.90554	0.8324	-0.26466	0.93842	0.97398	1.0163	0.02338
261.1089	3.59	oolomics Po	0.067883	0.99951	0.73113	-0.4518	0.44085	0.99998	0.89483	-0.16031	0.17968	0.99941	0.7722	-0.37294	0.40356	0.89682	0.88357	-0.17859	0.81108	0.99688	1.1212	0.1651	0.7016	0.9111	0.97796	-0.03216	0.046999	0.43801	0.70369	-0.50698	0.12751	0.90554	0.74169	-0.43111	0.8103	0.92196	1.3182	0.39857
492.2636	3.05	oolomics Po	0.067929	0.99951	1.3579	0.44137	0.062349	0.99998	0.83614	-0.25818	0.85632	0.99941	1.0316	0.044892	0.015865	0.20428	0.69803	-0.51863	0.000468	0.00542	0.55881	-0.83957	0.00011	0.034696	0.59474	-0.74966	0.67043	0.99391	1.0068	0.009721	0.50941	0.99956	0.92192	-0.11728	0.059563	0.30404	2.0085	1.0061
330.905	10.15	domics Pos	0.067941	0.94924	1.2697	0.34452	0.66545	0.99994	1.0214	0.030532	0.63735	0.99996	1.036	0.051069	0.19274	0.63549	0.91417	-0.12947	0.034191	0.078347	0.85509	-0.22585	0.65098	0.81894	0.96578	-0.05024	0.069239	0.46982	1.1204	0.16397	0.65866	0.97988	0.9642	-0.05259	0.36053	0.46966	1.2231	0.29049
332.2929	7.02	oolomics Po	0.067961	0.99951	1.2528	0.32517	0.28756	0.99998	0.8686	-0.20323	0.79288	0.99941	0.97606	-0.03495	0.040538	0.27667	0.66232	-0.59439	3.87E-06	0.000231	0.15958	-2.6477	0.003027	0.049681	0.47702	-1.0679	0.010531	0.24769	0.63122	-0.6638	0.10253	0.84495	0.81028	-0.30351	0.07919	0.3248	1.1876	0.24811
609.8811	10.05	domics Pos	0.068055	0.94924	1.1844	0.2441	0.33763	0.99994	1.0807	0.11199	0.96105	0.99996	1.0204	0.029119	0.53379	0.90063	1.0592	0.083036	0.096096	0.16925	0.85065	-0.23337	0.088025	0.31251	0.83585	-0.25868	0.028145	0.34956	1.2211	0.2882	0.48671	0.96776	1.1215	0.16538	0.037985	0.13096	1.4212	0.50711
884.5958	4.65	domics Pos	0.068198	0.94924	1.3699	0.45407	0.8667	0.99994	1.0152	0.021771	0.41539	0.99996	1.0973	0.13396	0.33755	0.7957	0.90976	-0.13644	0.000304	0.00224	0.60548	-0.72384	0.073737	0.28032	0.80452	-0.3138	0.58622	0.86163	0.95137	-0.07192	0.81652	0.97988	0.98496	-0.02186	0.063011	0.16114	1.5558	0.63764
151.1464	6.39	oolomics Po	0.068266	0.99951	1.1542	0.20689	0.14611	0.99998	0.87613	-0.19078	0.76363	0.99941	0.97742	-0.03295	0.074659	0.35544	0.77072	-0.37572	1.51E-06	0.000231	0.27847	-1.8444	0.001488	0.045425	0.59413	-0.75115	0.023756	0.32255	0.71352	-0.48697	0.11164	0.87331	0.82663	-0.27469	0.8069	0.92059	0.98025	-0.02878
93.0712	6.01	oolomics Po	0.068318	0.99951	1.1989	0.26166	0.34881	0.99998	0.92237	-0.11658	0.17981	0.99941	0.90176	-0.14918	0.004866	0.18788	0.73727	-0.43973	0.000102	0.001615	0.30615	-1.7077	0.002524	0.046224	0.6027	-0.73048	0.17259	0.74875	0.79205	-0.33633	0.29382	0.99956	0.90516	-0.14375	0.92796	0.96918	0.99741	-0.00374
245.2274	5.99	oolomics Po	0.068404	0.99951	1.1966	0.25897	0.36497	0.99998	0.91532	-0.12766	0.77093	0.99941	1.04	0.056632	0.15112	0.5459	0.80465	-0.31356	1.53E-05	0.000414	0.25069	-1.996	0.014157	0.089365	0.60758	-0.71886	0.13267	0.68208	0.80597	-0.3112	0.4623	0.99956	0.93809	-0.0922	0.065621	0.30735	1.1544	0.2071
740.9264	3.22	oolomics Po	0.068407	0.99951	1.4539	0.53998	0.090841	0.99998	0.90023	-0.15163	0.57798	0.99941	1.0686	0.095717	0.013674	0.20085	0.65144	-0.6183	0.001131	0.010206	0.60687	-0.72055	0.004187	0.054724	0.7088	-0.49654	0.57038	0.99391	0.93977	-0.08962	0.89301	0.99956	1.0259	0.036881	0.013413	0.2688	1.7281	0.78917
286.8436	0.31	olomics Ne	0.068574	0.99918	0.83524	-0.25973	0.005936	0.34605	0.84836	-0.23725	0.25626	0.99995	0.92559	-0.11155	0.66163	0.99985	0.97611	-0.03489	0.90626	0.99927	0.99483	-0.00747	0.15515	0.62813	0.92369	-0.11452	0.28131	0.66213	0.9007	-0.15088	0.32831	0.99512	0.94517	-0.08136	0.20056	0.99941	0.90765	-0.13979
254.2482	2.76	domics Pos	0.069029	0.94924	1.3872	0.4722	0.62299	0.99994	0.88866	-0.17029	0.8085	0.99996	1.021	0.029986	0.25754	0.71521	0.76415	-0.38808	0.000256	0.001986	0.25496	-1.9717	0.021234	0.12671	0.55149	-0.85859	0.35883	0.75581	0.81277	-0.29908	0.83174	0.97988	0.93535	-0.09642	0.037951	0.13096	1.5147	0.59902
675.3887	3.14	oolomics Po	0.069397	0.99951	1.4324	0.51846	0.089666	0.99998	0.85584	-0.22458	0.84248	0.99941	0.9855	-0.02108	0.002512	0.15651	0.63692	-0.65081	0.00045	0.005268	0.53307	-0.90761	0.001948	0.045425	0.63637	-0.65207	0.59305	0.99391	0.94254	-0.08537	0.60738	0.99956	0.95646	-0.06422	0.020392	0.2688	1.8304	0.87217
284.2867	6.38	oolomics Po	0.069461	0.99951	1.1961	0.25833	0.33285	0.99998	0.92799	-0.10782	0.72198	0.99941	1.0394	0.055688	0.21099	0.65159	0.84077	-0.25022	1.25E-05	0.000364	0.2236	-2.161	0.014767	0.091119	0.62165	-0.68583	0.11858	0.65209	0.81551	-0.29422	0.54802	0.99956	0.95529	-0.06599	0.053643	0.2951	1.1619	0.21647
547.2928	0.53	domics Pos	0.069515	0.94924	1.4018	0.48723	0.016796	0.99994	0.80592	-0.3113	0.47209	0.99996	0.94787	-0.07723	0.0006	0.17581	0.56147	-0.83273	7.06E-05	0.000824	0.38892	-1.3625	0.000485	0.033979	0.53617	-0.89924	0.41682	0.78082	0.88543	-0.17555	0.52169	0.96776	0.92908	-0.10613	0.025201	0.11206	2.1996	1.1372
415.2261	2.9	oolomics Po	0.069524	0.99951	1.4698	0.55557	0.43568	0.99998	0.90253	-0.14796	0.49085	0.99941	1.0765	0.10636	0.056072	0.30959	0.69873	-0.5172	0.007002	0.037008	0.54238	-0.88263	0.061953	0.21506	0.70163	-0.51121	0.99901	0.99906	1.0804	0.11158	0.72275	0.99956	1.0392	0.055426	0.018841	0.2688	2.1132	1.0794
712.4923	2.05	domics Pos	0.069638	0.94924	1.4772	0.56291	0.62711	0.99994	0.91276	-0.13169	0.48044	0.99996	1.1234	0.16792	0.26709	0.72822	0.79296	-0.33469	8.26E-05	0.000908	0.18115	-2.4648	0.033643	0.16489	0.55394	-0.85219	0.29204	0.71842	0.7996	-0.32266	0.5273	0.96776	1.0978	0.13457	0.00727	0.091944	1.6417	0.71521
67.055	1.67	domics Pos	0.069686	0.94924	1.4441	0.53013	0.825	0.99994	0.93	-0.1047	0.39698	0.99996	1.1361	0.18403	0.64779	0.95407	0.89338	-0.16265	0.000251	0.001959	0.19768	-2.3388	0.047351	0.21043	0.5995	-0.73816	0.66628	0.8978	0.90768	-0.13975	0.83015	0.97988	1.0265	0.0377	0.038572	0.13216	1.5421	0.62492
962.1519	9.45	oolomics Po	0.069767	0.99951	1.4204	0.5063	0.70407	0.99998	0.95898	-0.06043	0.72631	0.99941	1.0913	0.1261	0.012021	0.20055	0.72411	-0.46573	0.013036	0.059342	0.58464	-0.77439	0.053793	0.1968	0.74848	-0.41797	0.72883	0.99391	0.97384	-0.03824	0.66957	0.99956	0.96689	-0.04857	0.072388	0.3167	1.7571	0.81317
187.0817	10.16	domics Pos	0.070085	0.94924	1.2618	0.33548	0.28952	0.99994	1.083	0.11509	0.42663	0.99996	1.0658	0.091928	0.45431	0.87408	1.0433	0.061127	0.030113	0.071053	0.83917	-0.25297	0.56763	0.76437	1.1162	0.15858	0.10239	0.54475	1.1526	0.20493	0.007764	0.52955	1.3057	0.38488	0.006037	0.08977	1.3616	0.44535
330.9796	12	olomics Ne	0.07022	0.99918	1.0709	0.098847	0.041445	0.46524	0.92609	-0.11078	0.65104	0.99995	1.029	0.041238	0.69702	0.99985	0.97594	-0.03514	0.3079	0.99927	1.0579	0.081207	0.64802	0.92388	1.0513	0.072123	0.006196	0.2539	1.0769	0.10682	0.33701	0.99512	1.0549	0.077139	0.40404	0.99941	1.0408	0.057637
611.3244	0.56	domics Pos	0.070312	0.94924	1.4345	0.52051	0.095368	0.99994	0.85576	-0.22472	0.85279	0.99996	0.98752	-0.01812	0.008769	0.19971	0.57321	-0.80285	0.000121	0.001169	0.41069	-1.2839	0.000805	0.040694	0.55768	-0.8425	0.41297	0.78028	0.88441	-0.17721	0.60233	0.97073	0.95736	-0.06286	0.011213	0.094875	2.1759	1.1216
462.2021	2.3	domics Pos	0.070351	0.94924	1.1142	0.15605	0.40085	0.99994	0.95273	-0.06986	0.30071	0.99996	0.92231	-0.11667	0.64905	0.95432	1.0216	0.030764	0.000406	0.002795	0.77662	-0.36473	0.27643	0.56257	0.88919	-0.16944	0.29721	0.72275	0.86829	-0.20375	0.29115	0.94825	0.89578	-0.15878	0.57766	0.67173	1.0499	0.070289
722.3965	0.56	domics Pos	0.070379	0.94924	1.4579	0.54394	0.27724	0.99994	0.8967	-0.1573	0.71648	0.99996	0.96532	-0.05092	0.01484	0.2319	0.57161	-0.80689	0.000146	0.001349	0.40859	-1.2913	0.000584	0.038423	0.52454	-0.93087	0.2848	0.71383	0.83693	-0.25682	0.51544	0.96776	0.919	-0.12186	0.016708	0.099614	2.0625	1.0444
790.4471	0.58	domics Pos	0.070418	0.94924	1.4838	0.56931	0.15169	0.99994	0.83615	-0.25816	0.79244	0.99996	0.97119	-0.04218	0.010108	0.21094	0.61991	-0.68987	0.000543	0.003506	0.46145	-1.1157	0.001857	0.057105	0.57623	-0.79529	0.58654	0.86163	0.93452	-0.09771	0.90723	0.98236	1.0851	0.1178	0.013742	0.098791	2.6269	1.3934
1016.797	10.02	domics Pos	0.070468	0.94924	1.1511	0.20297	0.65417	0.99994	1.0343	0.048605	0.58452	0.99996	0.95332	-0.06896	0.67197	0.95717	0.96191	-0.05603	0.028252	0.067922	0.79603	-0.3291	0.79495	0.89629	0.98219	-0.02593	0.49797	0.8259	1.0614	0.085919	0.20503	0.92582	1.1103	0.1509	0.003095	0.085302	1.2981	0.37643
134.0196	0.31	oolomics Po	0.07089	0.99951	1.3411	0.42338	0.081678	0.99998	1.3742	0.45863	0.25082	0.99941	1.3029	0.38176	0.01491	0.20331	1.3541	0.43736	0.007798	0.040034	1.8826	0.9127	0.0005	0.039708	1.7947	0.84373	0.001754	0.16989	1.9676	0.97641	0.078124	0.77824	1.2647	0.33884	0.030269	0.2688	1.2002	0.26322
700.3812	0.56	domics Pos	0.070984	0.94924	1.4343	0.52031	0.10976	0.99994	0.88919	-0.16944	0.86313	0.99996	1.0135	0.019341	0.010124	0.21094	0.60659	-0.72121	7.41E-05	0.000851	0.41579	-1.2661	6.8E-05	0.019289	0.5764	-0.79486	0.34133	0.74472	0.86669	-0.20641	0.5921	0.97073	0.9586	-0.06101	0.01103	0.094875	2.1333	1.0931
451.8806	6.37	oolomics Po	0.071066	0.99951	1.5767	0.65693	0.64535	0.99998	0.88918	-0.16946	0.53341	0.99941	1.1179	0.16078	0.54787	0.99905	0.88655	-0.17372	0.000626	0.006511	0.15188	-2.719	0.056659	0.20281	0.47738	-1.0668	0.70074	0.99391	0.91723	-0.12464	0.96661	0.99956	0.99784	-0.00313	0.026783	0.2688	1.5832	0.66289
787.4334	3.27	oolomics Po	0.07108	0.99951	1.5201	0.60417	0.043916	0.99998	0.79895	-0.32383	0.53278	0.99941	1.0612	0.085674	0.01076	0.19754	0.64379	-0.63534	0.004125	0.025526	0.53399	-0.90511	0.005073	0.061592	0.63698	-0.65068	0.72138	0.99391	0.97356	-0.03866	0.5459	0.99956	0.92244	-0.11648	0.020226	0.2688	1.8664	0.90025
612.3271	0.56	domics Pos	0.071222	0.94924	1.4334	0.51942	0.052023	0.99994	0.83686	-0.25694	0.46921	0.99996	0.93282	-0.10033	0.011774	0.21807	0.6205	-0.68849	0.000148	0.00136	0.43124	-1.2134	0.000981	0.043236	0.52791	-0.92165	0.55497	0.85279	0.9256	-0.11154	0.3969	0.96776	0.87246	-0.19683	0.016939	0.099827	2.1436	1.1
268.2643	6.03	oolomics Po	0.071253	0.99951	1.2492	0.32104	0.76147	0.99998	0.96885	-0.04565	0.33907	0.99941	1.1307	0.17721	0.45011	0.92792	0.89306	-0.16316	1.6E-05	0.000426	0.2578	-1.9557	0.021868	0.10951	0.66451	-0.58964	0.21687	0.8123	0.86206	-0.21413	0.95335	0.99956	1.0069	0.009884	0.052859	0.2951	1.2328	0.30196
128.1072	2.76	domics Pos	0.071609	0.94924	1.2589	0.33218	0.29191	0.99994	0.87544	-0.19192	0.98283	0.99996	1.0021	0.003058	0.11104	0.53238	0.75748	-0.40073	9.33E-06	0.000213	0.26635	-1.9086	0.001941	0.057382	0.56413	-0.82589	0.06527	0.46042	0.70261	-0.5092	0.45351	0.96776	0.90479	-0.14434	0.008204	0.092094	1.6007	0.67868
444.5655	0.54	domics Pos	0.071692	0.94924	1.4547	0.54073	0.085096	0.99994	0.81369	-0.29744	0.8668	0.99996	0.98622	-0.02002	0.001123	0.17581	0.61758	-0.69529	0.000712	0.00428	0.42972	-1.2185	0.001111	0.043236	0.59591	-0.74683	0.51507	0.83572	0.91684	-0.12526	0.51169	0.96776	0.91973	-0.12072	0.007624	0.091944	2.2881	1.1941
243.2111	1.39	domics Pos	0.07171	0.94924	1.253	0.32534	0.34369	0.99994	0.88157	-0.18185	0.21713	0.99996	1.19	0.25091	0.13396	0.55796	0.76104	-0.39395	1.44E-05	0.000291	0.21911	-2.1903	0.011519	0.09183	0.59447	-0.75033	0.93074	0.98468	0.98311	-0.02458	0.22296	0.94047	0.84323	-0.24601	0.044653	0.14048	1.3109	0.39055
313.3171	2.75	domics Pos	0.071782	0.94924	1.2931	0.37085	0.15536	0.99994	0.77196	-0.37341	0.81373	0.99996	0.95937	-0.05985	0.095705	0.51543	0.6778	-0.56107	4.26E-05	0.000564	0.15902	-2.6527	0.002033	0.059378	0.41995	-1.2517	0.020716	0.33809	0.62145	-0.68628	0.23266	0.94365	0.82977	-0.26921	0.043464	0.1388	1.3558	0.43912
611.334	3.05	oolomics Po	0.072004	0.99951	1.4777	0.56331	0.3971	0.99998	0.89819	-0.15491	0.54348	0.99941	1.0584	0.081912	0.009237	0.19702	0.62273	-0.68331	0.010453	0.050043	0.56715	-0.81821	0.001731	0.045425	0.56899	-0.81353	0.9923	0.99906	1.0658	0.091874	0.65118	0.99956	0.947	-0.07856	0.021271	0.2688	1.8942	0.92158
360.1768	0.58	domics Pos	0.072115	0.94924	1.6199	0.69592	0.20574	0.99994	0.87381	-0.19461	0.62242	0.99996	1.0671	0.093694	0.0504	0.38141	0.64609	-0.6302	0.001364	0.006879	0.44122	-1.1804	0.002395	0.061655	0.5639	-0.82648	0.97543	0.99341	1.0749	0.10425	0.83559	0.97988	1.2158	0.28186	0.015431	0.09885	2.9615	1.5663
565.2801	0.59	domics Pos	0.072195	0.94924	1.5707	0.65139	0.71258	0.99994	0.93933	-0.0903	0.40811	0.99996	1.1226	0.16686	0.046875	0.3738	0.65429	-0.612	0.001275	0.006577	0.45889	-1.1238	0.007533	0.082061	0.62086	-0.68765	0.90933	0.97939	1.0534	0.075119	0.84261	0.97988	1.1879	0.24837	0.023746	0.11051	2.4217	1.276
968.5804	9.94	olomics Ne	0.072212	0.99918	1.1209	0.16469	0.44685	0.82137	0.9639	-0.05304	0.83904	0.99995	1.0356	0.05052	0.43401	0.99985	1.0698	0.097274	0.31255	0.99927	1.0405	0.057272	0.026661	0.45583	1.1953	0.25741	0.33746	0.71058	0.93245	-0.1009	0.086105	0.99512	1.0679	0.094794	0.087352	0.99941	1.2289	0.29736
829.4803	3.32	oolomics Po	0.072278	0.99951	1.4838	0.56928	0.12834	0.99998	0.92101	-0.11871	0.87581	0.99941	0.9975	-0.00361	0.000109	0.077904	0.62285	-0.68304	0.000554	0.006124	0.57963	-0.78679	0.001846	0.045425	0.59022	-0.76067	0.53293	0.99391	0.92997	-0.10474	0.27478	0.99956	0.8264	-0.27509	0.028053	0.2688	1.8061	0.85288
135.1181	6.38	oolomics Po	0.072374	0.99951	1.1856	0.24561	0.35159	0.99998	0.92114	-0.11851	0.85412	0.99941	1.0131	0.01878	0.13974	0.52592	0.79791	-0.3257	0.000002	0.000231	0.24426	-2.0335	0.005976	0.065713	0.58324	-0.77785	0.049823	0.45099	0.75915	-0.39755	0.25415	0.99956	0.89234	-0.16433	0.71058	0.86888	1.0293	0.041646
937.6575	9.17	oolomics Po	0.072396	0.99951	1.4056	0.49122	0.8086	0.99998	1.0375	0.053121	0.21426	0.99941	1.1716	0.22845	0.18702	0.61868	0.86029	-0.2171	0.026187	0.10389	0.74763	-0.4196	0.087058	0.27045	0.69926	-0.5161	0.9276	0.9951	1.0673	0.093952	0.56804	0.99956	0.93399	-0.09852	0.067242	0.30938	1.5573	0.63903
652.8741	3.11	oolomics Po	0.072555	0.99951	1.3654	0.44927	0.12432	0.99998	0.90831	-0.13875	0.35691	0.99941	0.95623	-0.06457	0.000606	0.12797	0.66437	-0.58994	0.00115	0.010346	0.5886	-0.76464	0.001035	0.043099	0.59971	-0.73767	0.54999	0.99391	0.93965	-0.08981	0.6254	0.99956	0.95889	-0.06056	0.010741	0.2688	1.8776	0.90891
263.238	5.99	oolomics Po	0.072778	0.99951	1.1904	0.25145	0.41485	0.99998	0.92445	-0.11333	0.72459	0.99941	1.0501	0.070533	0.16426	0.57301	0.80986	-0.30425	8.7E-06	0.000292	0.24557	-2.0258	0.013348	0.085847	0.62609	-0.67556	0.12858	0.67606	0.81388	-0.29711	0.29392	0.99956	0.91658	-0.12567	0.068114	0.31036	1.1504	0.20211
807.9698	3.29	oolomics Po	0.07278	0.99951	1.4096	0.49526	0.04042	0.99998	0.82584	-0.27607	0.58735	0.99941	1.0372	0.052648	0.001178	0.13475	0.57777	-0.79143	0.006581	0.035557	0.59721	-0.7437	0.000305	0.039399	0.58297	-0.7785	0.48853	0.99391	0.90552	-0.14319	0.53397	0.99956	0.93247	-0.10087	0.037827	0.28036	1.8334	0.87455
752.5575	9.29	oolomics Po	0.072845	0.99951	1.3002	0.37877	0.46837	0.99998	1.124	0.16869	0.61655	0.99941	1.0591	0.082892	0.59982	0.99905	1.1041	0.14289	0.054628	0.17943	1.2341	0.3035	0.001728	0.045425	1.5358	0.61895	0.000281	0.16989	1.8005	0.84837	0.41358	0.99956	1.1803	0.23914	0.005015	0.2688	1.5406	0.62346
139.113	5.99	oolomics Po	0.072856	0.99951	1.1602	0.21443	0.68	0.99998	0.96688	-0.04859	0.6007	0.99941	0.94272	-0.08509	0.16751	0.57919	0.74053	-0.43336	3.16E-06	0.000231	0.23457	-2.0919	0.012045	0.083709	0.54827	-0.86703	0.055103	0.47167	0.76846	-0.37996	0.13647	0.92085	0.85097	-0.23282	0.2009	0.46635	1.1111	0.15199
656.3545	0.56	domics Pos	0.072873	0.94938	1.403	0.48849	0.049118	0.99994	0.84662	-0.24022	0.91167	0.99996	1.0099	0.014192	0.000893	0.17581	0.52336	-0.93414	9.14E-05	0.000972	0.43318	-1.2069	0.000244	0.026314	0.53352	-0.90638	0.29578	0.72019	0.86296	-0.21264	0.46781	0.96776	0.90767	-0.13976	0.017114	0.10008	2.1231	1.0862
168.074	0.84	domics Pos	0.073043	0.94938	0.86018	-0.21728	0.068898	0.99994	0.74708	-0.42067	0.13128	0.99996	0.85949	-0.21844	0.10143	0.52336	0.86677	-0.20628	0.002065	0.009273	0.69914	-0.51634	0.035199	0.16986	0.6773	-0.56214	0.15545	0.62672	0.79703	-0.32729	0.010308	0.52955	0.76168	-0.39274	0.3494	0.4594	0.91197	-0.13294
499.9054	0.5	domics Pos	0.073075	0.94938	1.1264	0.17171	0.65547	0.99994	1.0266	0.037919	0.74644	0.99996	0.98053	-0.02837	0.32768	0.78974	0.94381	-0.08343	0.008487	0.027035	0.80634	-0.31054	0.40113	0.64773	0.91772	-0.12387	0.37081	0.76056	1.0789	0.10957	0.95967	0.98838	0.99781	-0.00317	0.041152	0.13527	1.25	0.32196
612.5422	5.66	domics Pos	0.07322	0.94938	0.90931	-0.13716	0.63401	0.99994	0.96102	-0.05737	0.79488	0.99996	0.98384	-0.02351	0.073243	0.45875	0.83815	-0.25471	0.023029	0.058037	0.82269	-0.28158	0.5355	0.74088	1.0885	0.12228	0.027205	0.34512	1.3356	0.41745	0.068914	0.76386	1.1552	0.20817	0.000832	0.07528	1.3387	0.42085
829.9846	3.32	oolomics Po	0.073319	0.99951	1.6398	0.71352	0.26423	0.99998	0.92916	-0.106	0.50004	0.99941	1.0868	0.12012	0.004891	0.18788	0.64203	-0.63929	0.003425	0.022474	0.59526	-0.74841	0.002116	0.045856	0.65277	-0.61536	0.60159	0.99391	0.95033	-0.07349	0.80893	0.99956	0.99079	-0.01335	0.024782	0.2688	1.788	0.83832
272.8613	0.3	oolomics Po	0.073513	0.99951	1.1397	0.18868	0.30027	0.99998	1.064	0.089519	0.6987	0.99941	1.0356	0.050471	0.066008	0.33591	1.1871	0.2474	0.02798	0.10894	1.1691	0.22536	0.079999	0.25583	1.2603	0.33375	0.30022	0.92082	1.1629	0.21774	0.070389	0.76039	1.1425	0.19219	0.86905	0.94847	1.0349	0.049531
121.1019	2.05	domics Pos	0.073765	0.94938	1.2365	0.30631	0.46064	0.99994	0.91942	-0.12121	0.7378	0.99996	1.0347	0.049245	0.17569	0.60979	0.78459	-0.34999	2.02E-06	0.000144	0.16967	-2.5592	0.008241	0.084185	0.5465	-0.87171	0.28028	0.70964	0.84708	-0.23943	0.83364	0.97988	0.97686	-0.03378	0.004716	0.085788	1.4142	0.49999
612.3396	3.06	oolomics Po	0.074442	0.99951	1.459	0.54497	0.65854	0.99998	0.92419	-0.11373	0.6818	0.99941	1.0442	0.062443	0.034685	0.25527	0.67025	-0.57723	0.037455	0.13526	0.65143	-0.61832	0.025403	0.11908	0.61244	-0.70737	0.96615	0.9974	1.0836	0.11581	0.27488	0.99956	0.81899	-0.28809	0.013146	0.2688	2.0304	1.0217
129.1178	2.05	domics Pos	0.074458	0.94938	1.2037	0.26748	0.34573	0.99994	0.87563	-0.1916	0.59186	0.99996	0.94513	-0.08141	0.07531	0.46303	0.73523	-0.44374	3.78E-05	0.000531	0.1918	-2.3824	0.020477	0.12316	0.56628	-0.82041	0.091485	0.52136	0.75343	-0.40845	0.25043	0.94365	0.89716	-0.15657	0.012534	0.098791	1.3268	0.4079
333.2944	2.75	domics Pos	0.074601	0.94938	1.3737	0.4581	0.2529	0.99994	0.76403	-0.3883	0.95277	0.99996	0.9635	-0.05365	0.098739	0.51862	0.62951	-0.6677	2.57E-05	0.000431	0.13573	-2.8812	0.00329	0.064159	0.36414	-1.4574	0.045618	0.39255	0.59026	-0.76057	0.10308	0.85577	0.64565	-0.63117	0.23058	0.33769	1.2371	0.30695
850.9917	3.33	oolomics Po	0.074606	0.99951	1.2683	0.34291	0.87345	0.99998	0.98281	-0.02502	0.81024	0.99941	0.97131	-0.042	0.001616	0.14411	0.59458	-0.75007	0.002513	0.01796	0.5686	-0.81452	0.010899	0.08293	0.54112	-0.88598	0.40925	0.98164	0.87386	-0.19452	0.15936	0.93927	0.80691	-0.30952	0.014548	0.2688	1.8725	0.90498
679.5175	2.74	domics Pos	0.074707	0.94938	1.3209	0.40148	0.25343	0.99994	0.78169	-0.35532	0.604	0.99996	0.90307	-0.14709	0.033375	0.31895	0.54382	-0.87881	2.61E-05	0.000436	0.11667	-3.0995	0.000312	0.028292	0.2358	-2.0844	0.051579	0.41162	0.48168	-1.0538	0.043488	0.65325	0.54775	-0.8684	0.50254	0.60409	0.86995	-0.201

115.1027	2.06	domics Pos	0.074773	0.94938	1.2564	0.3293	0.49595	0.99994	0.90263	-0.1478	0.7545	0.99996	1.0447	0.063097	0.29405	0.75244	0.81146	-0.30141	3.2E-05	0.000486	0.21843	-2.1947	0.10051	0.33827	0.70486	-0.5046	0.47576	0.80869	0.88417	-0.17761	0.68733	0.97988	1.0516	0.072572	0.010262	0.094875	1.5054	0.5901
752.957	3.23	bolomics Pc	0.074811	0.99951	1.5191	0.60317	0.34025	0.99998	0.84801	-0.23784	0.3301	0.99941	0.90967	-0.13659	0.00218	0.14926	0.63201	-0.66198	0.002333	0.017262	0.56286	-0.82915	0.02215	0.11005	0.71715	-0.47966	0.4006	0.97985	0.87023	-0.20052	0.62859	0.99956	0.94227	-0.08579	0.098771	0.35294	1.6006	0.67862
574.608	0.34	olomics Ne	0.074951	0.99918	1.143	0.1928	0.011214	0.37315	0.90673	-0.14126	0.34418	0.99995	1.0328	0.046543	0.13359	0.99985	1.1993	0.26219	0.64466	0.99927	1.0381	0.053927	0.000101	0.061927	1.3269	0.40811	0.35862	0.71602	1.0719	0.10019	0.46269	0.99512	1.0433	0.061123	0.87565	0.99941	0.99682	-0.0046
153.128	2.05	domics Pos	0.075106	0.94938	1.2195	0.28627	0.49028	0.99994	0.92482	-0.11275	0.92185	0.99996	1.0077	0.01108	0.10444	0.52894	0.74705	-0.42072	8.79E-07	0.000144	0.16611	-2.5898	0.011074	0.091199	0.5587	-0.83987	0.16482	0.63453	0.79617	-0.32885	0.79522	0.97988	0.9729	-0.03964	0.003421	0.085302	1.4204	0.50626
511.7922	3.08	bolomics Pc	0.075341	0.99951	1.4944	0.57953	0.36897	0.99998	0.86214	-0.214	0.5752	0.99941	1.069	0.096257	0.052739	0.29933	0.73249	-0.44912	0.019538	0.082278	0.66134	-0.59654	0.11141	0.32028	0.7234	-0.46713	0.85507	0.9951	1.146	0.19659	0.92927	0.99956	1.1032	0.1417	0.011423	0.2688	2.3238	1.2165
278.2452	6.2	bolomics Pc	0.075414	0.99951	1.2094	0.27434	0.67441	0.99998	0.94781	-0.07733	0.90957	0.99941	1.0522	0.073353	0.22963	0.67984	0.79196	-0.3365	0.000127	0.001919	0.23811	-2.0703	0.021342	0.10914	0.54904	-0.86502	0.3613	0.96545	0.87588	-0.19119	0.24063	0.99956	0.86354	-0.21167	0.34314	0.61835	1.1758	0.2336
124.1218	1.66	domics Pos	0.075504	0.94938	1.2512	0.32331	0.052003	0.99994	0.83252	-0.26444	0.46422	0.99996	1.0991	0.13627	0.018973	0.25595	0.68225	-0.55163	3.67E-05	0.000527	0.21168	-2.24	0.01754	0.11316	0.54375	-0.87898	0.13045	0.59354	0.77669	-0.3646	0.8887	0.98023	0.98401	-0.02326	0.051085	0.14779	1.3212	0.40185
340.3577	8.4	bolomics Pc	0.075534	0.99951	0.83757	-0.25571	0.14595	0.99998	0.76876	-0.3794	0.099678	0.99941	0.79529	-0.33046	0.16008	0.5653	0.87618	-0.1907	0.22491	0.54095	0.86587	-0.20778	0.01705	0.096516	0.61446	-0.70261	0.065497	0.51838	0.71756	-0.47884	0.012187	0.47452	0.67534	-0.56632	0.049931	0.29131	0.7868	-0.34593
374.7365	0.64	domics Pos	0.075572	0.94938	1.1685	0.22468	0.42817	0.99994	1.114	0.15573	0.46673	0.99996	1.1039	0.14256	0.98594	0.99652	0.9988	-0.00174	0.039475	0.087459	0.81448	-0.29605	0.84286	0.92105	0.98057	-0.02831	0.64157	0.8857	1.0804	0.11156	0.078934	0.79555	1.2067	0.27106	0.033626	0.12517	1.2976	0.37587
389.1943	4.11	bolomics Pc	0.075708	0.99951	1.2863	0.36327	0.008837	0.99998	0.62728	-0.67281	0.24005	0.99941	0.86307	-0.21246	0.00336	0.17363	0.38697	-1.3697	0.00041	0.004897	0.22539	-2.1495	1.93E-05	0.034543	0.28412	-1.8154	0.025081	0.32816	0.5124	-0.96467	0.17104	0.95869	0.79006	-0.33996	0.86653	0.9482	1.0521	0.073206
208.1779	1.66	domics Pos	0.076112	0.94938	1.3089	0.38833	0.62866	0.99994	0.92923	-0.10589	0.92766	0.99996	1.0153	0.02186	0.31863	0.78283	0.84506	-0.24288	3.14E-05	0.000482	0.25244	-1.986	0.012021	0.092972	0.65856	-0.60262	0.44995	0.80156	0.87965	-0.185	0.96977	0.99108	1.0228	0.032457	0.15298	0.25867	1.3578	0.44132
547.3322	2.65	bolomics Pc	0.076188	0.99951	1.2979	0.37622	0.19891	0.99998	0.84374	-0.24513	0.29316	0.99941	0.91332	-0.13081	0.003749	0.17363	0.58494	-0.77363	9.11E-05	0.001485	0.46428	-1.1069	0.002081	0.045856	0.59838	-0.74087	0.47504	0.99391	0.91188	-0.13309	0.97478	0.99956	1.0595	0.08344	0.01372	0.2688	2.0017	1.0013
223.2058	2.05	domics Pos	0.076255	0.94938	1.2838	0.36043	0.34427	0.99994	0.87665	-0.18993	0.50983	0.99996	1.0664	0.092696	0.2058	0.6523	0.76041	-0.39516	1.31E-05	0.000277	0.16804	-2.5731	0.011371	0.091661	0.52683	-0.92459	0.20421	0.64899	0.81122	-0.30184	0.50059	0.96776	0.92361	-0.11465	0.024787	0.1117	1.3438	0.42632
754.4239	3.25	bolomics Pc	0.076286	0.99951	1.3901	0.47514	0.2677	0.99998	0.91229	-0.13243	0.85396	0.99941	0.99543	-0.00661	0.060031	0.32244	0.67658	-0.56366	0.012352	0.057052	0.64312	-0.63684	0.060764	0.21244	0.65241	-0.61616	0.54046	0.99391	0.92977	-0.10506	0.79287	0.99956	1.0108	0.015456	0.017955	0.2688	1.7819	0.83344
897.9975	0.54	domics Pos	0.076298	0.94938	1.4151	0.50087	0.40361	0.99994	0.93712	-0.09369	0.68336	0.99996	0.97135	-0.04194	0.008274	0.19971	0.613	-0.70604	0.000354	0.002507	0.42052	-1.2498	0.003049	0.063179	0.56403	-0.82615	0.19415	0.64891	0.80831	-0.30701	0.50801	0.96776	0.91862	-0.12246	0.020082	0.10652	2.1335	1.0932
518.7497	9.92	domics Pos	0.076551	0.94938	1.2172	0.28354	0.79019	0.99994	1.0499	0.070221	0.51055	0.99996	1.0861	0.1191	0.50068	0.89009	1.0883	0.12213	0.040411	0.088759	0.79676	-0.32779	0.40975	0.65377	0.95121	-0.07217	0.39857	0.77224	1.1461	0.19677	0.12878	0.88537	1.2158	0.28192	0.22657	0.33368	1.123	0.1674
283.2837	6.38	bolomics Pc	0.076755	0.99951	1.2112	0.2765	0.34799	0.99998	0.92364	-0.11459	0.70105	0.99941	1.0393	0.055678	0.21989	0.66625	0.83833	-0.25441	6.36E-06	0.000265	0.22955	-2.1231	0.016402	0.094477	0.62606	-0.67563	0.11984	0.65311	0.81858	-0.28881	0.43296	0.99956	0.9411	-0.08758	0.048314	0.28861	1.1618	0.21635
143.1298	2.05	domics Pos	0.076927	0.94938	1.1902	0.25125	0.091942	0.99994	0.81719	-0.29126	0.6962	0.99996	0.96483	-0.05165	0.12449	0.54705	0.7853	-0.34868	6.08E-06	0.000169	0.19151	-2.3845	0.010228	0.08787	0.58813	-0.7658	0.27872	0.70945	0.8432	-0.24606	0.85953	0.98023	0.98136	-0.02715	0.010682	0.094875	1.3673	0.45137
195.1484	2.76	domics Pos	0.076937	0.94938	1.1888	0.24957	0.32483	0.99994	0.8954	-0.1594	0.7599	0.99996	0.96574	-0.05029	0.14638	0.57298	0.75824	-0.39927	1.06E-06	0.000144	0.19525	-2.3566	0.003741	0.067932	0.50267	-0.99233	0.19631	0.64891	0.81521	-0.29476	0.49055	0.96776	0.92176	-0.11753	0.006696	0.090221	1.4845	0.56997
249.2497	2.05	domics Pos	0.077459	0.95275	1.1896	0.25052	0.17571	0.99994	0.88792	-0.1715	0.70231	0.99996	0.96373	-0.05331	0.1954	0.6412	0.78858	-0.34266	6.51E-07	0.000144	0.16587	-2.5919	0.002628	0.061655	0.598	-0.74177	0.13486	0.60006	0.82516	-0.27726	0.71649	0.97988	0.96921	-0.04513	0.00461	0.085788	1.4112	0.49695
153.1285	5.99	bolomics Pc	0.077481	0.99951	1.2897	0.36706	0.86996	0.99998	0.98701	-0.01887	0.19578	0.99941	1.1281	0.17388	0.18842	0.6204	0.81789	-0.29002	1.32E-05	0.000379	0.27085	-1.8844	0.004232	0.05479	0.60896	-0.71558	0.35205	0.96013	0.87965	-0.18501	0.54332	0.99956	0.94201	-0.08619	0.048377	0.28861	1.2368	0.30658
177.1649	6.38	bolomics Pc	0.077943	0.99951	1.1713	0.22812	0.39633	0.99998	0.93047	-0.10397	0.95311	0.99941	0.99618	-0.00552	0.14815	0.54391	0.81223	-0.30005	3.24E-06	0.000231	0.23513	-2.0884	0.006086	0.065713	0.57871	-0.78908	0.033005	0.35974	0.75555	-0.40439	0.20688	0.99956	0.88211	-0.18097	0.43152	0.69935	1.0732	0.10186
451.3063	8.12	bolomics Pc	0.077969	0.99951	0.74888	-0.41719	0.15182	0.99998	0.80028	-0.32142	0.034449	0.99941	0.72142	-0.47108	0.19983	0.63506	0.80752	-0.30843	0.15182	0.40425	0.79817	-0.32524	0.13784	0.37527	0.75281	-0.40964	0.73746	0.99391	0.99148	-0.01234	0.11501	0.88816	0.75872	-0.39836	0.75734	0.89519	1.0635	0.088812
806.9656	3.3	bolomics Pc	0.078012	0.99951	1.4739	0.55962	0.31376	0.99998	0.88092	-0.18292	0.87672	0.99941	0.98577	-0.02067	0.002163	0.14926	0.6056	-0.72357	0.005125	0.029954	0.53543	-0.90122	0.002104	0.045856	0.63135	-0.66349	0.65649	0.99391	0.96291	-0.05453	0.64563	0.99956	0.95306	-0.06936	0.04435	0.28476	1.7603	0.81581
198.1858	2.05	domics Pos	0.078066	0.95356	1.2303	0.29902	0.35479	0.99994	0.90018	-0.15171	0.67591	0.99996	1.0459	0.064775	0.14425	0.57187	0.77712	-0.3638	6.63E-06	0.00018	0.16665	-2.5851	0.009449	0.08703	0.55645	-0.84569	0.22654	0.66988	0.82689	-0.27424	0.76987	0.97988	0.96775	-0.04729	0.006374	0.090084	1.3886	0.47358
265.2538	6.38	bolomics Pc	0.078199	0.99951	1.1983	0.26103	0.36429	0.99998	0.9269	-0.10952	0.59828	0.99941	1.0515	0.072381	0.26446	0.73853	0.85562	-0.22496	6.23E-06	0.000265	0.23415	-2.0945	0.014839	0.091119	0.62385	-0.68074	0.14458	0.71385	0.82841	-0.27158	0.56727	0.99956	0.95652	-0.06414	0.051013	0.29427	1.1672	0.22303
151.1474	1.67	domics Pos	0.078233	0.95356	1.283	0.35953	0.46861	0.99994	0.90089	-0.15057	0.62758	0.99996	1.0526	0.073889	0.15016	0.57834	0.73188	-0.45033	4.5E-06	0.000149	0.18059	-2.4692	0.009368	0.086818	0.54875	-0.86577	0.35286	0.75095	0.85945	-0.21851	0.59978	0.97073	0.9276	-0.10842	0.02866	0.11732	1.3427	0.42513
613.3407	0.58	domics Pos	0.078459	0.95356	1.5184	0.60257	0.24149	0.99994	0.89233	-0.16435	0.89069	0.99996	1.0092	0.013227	0.007804	0.19917	0.57815	-0.79047	0.000211	0.00175	0.39895	-1.3257	0.000728	0.040248	0.57931	-0.78759	0.65837	0.89305	0.9589	-0.06055	0.89088	0.98023	1.0687	0.095894	0.020148	0.10655	2.4234	1.277
296.2951	2.41	domics Pos	0.07954	0.95356	1.2268	0.29488	0.16002	0.99994	0.81725	-0.29115	0.89778	0.99996	1.0156	0.022399	0.080909	0.4813	0.70236	-0.50972	1.85E-06	0.000144	0.1405	-2.8314	0.004307	0.071311	0.45954	-1.1217	0.066114	0.46086	0.70852	-0.49711	0.20155	0.92582	0.85385	-0.22794	0.019654	0.10555	1.3517	0.43479
350.2507	2.06	domics Pos	0.079675	0.95356	1.2123	0.27769	0.47841	0.99994	0.95606	-0.06483	0.71903	0.99996	1.0158	0.022657	0.30461	0.76213	0.89417	-0.16137	7.11E-06	0.000186	0.28589	-1.8065	0.003358	0.064673	0.63811	-0.64813	0.61743	0.87206	0.95438	-0.06736	0.49873	0.96776	1.0806	0.11181	0.022366	0.10864	1.3208	0.4014
952.7124	5.23	domics Pos	0.079714	0.95356	1.2051	0.26916	0.76909	0.99994	1.0691	0.096399	0.48151	0.99996	0.94678	-0.0789	0.28563	0.7511	0.89863	-0.15419	0.033467	0.077106	0.80453	-0.31377	0.38345	0.63866	0.88509	-0.17611	0.24612	0.68401	0.88465	-0.17683	0.75044	0.97988	0.97938	-0.03006	0.039031	0.13241	1.4026	0.48808
340.3582	8.13	bolomics Pc	0.079992	0.99951	0.86008	-0.21746	0.070355	0.99998	0.79571	-0.32969	0.083497	0.99941	0.7942	-0.33243	0.034637	0.25527	0.83863	-0.2539	0.96668	0.9989	0.99234	-0.0111	0.009575	0.078676	0.66515	-0.58826	0.050498	0.45418	0.75067	-0.41375	0.010424	0.47452	0.77686	-0.36428	0.067832	0.31012	0.8326	-0.2643
930.7883	10.03	domics Pos	0.079998	0.95356	1.0729	0.1015	0.63115	0.99994	0.97143	-0.04182	0.58886	0.99996	0.95962	-0.05947	0.1983	0.64373	0.90063	-0.15099	0.006306	0.021701	0.75054	-0.41401	0.077795	0.28838	0.82652	-0.27489	0.86039	0.97339	1.0113	0.016215	0.72847	0.97988	1.0346	0.049051	0.046169	0.14289	1.2223	0.28964
220.2145	1.46	domics Pos	0.080228	0.95356	1.2221	0.28931	0.90212	0.99994	1.0191	0.027312	0.37695	0.99996	1.1383	0.18688	0.11625	0.53727	0.8113	-0.30169	3E-05	0.000473	0.25516	-1.9705	0.002311	0.061655	0.52629	-0.92607	0.30094	0.72409	0.842	-0.24811	0.1346	0.89103	0.86158	-0.21495	0.007731	0.091944	1.3614	0.44513
630.3033	1.84	domics Pos	0.080248	0.95356	1.2999	0.37838	0.67044	0.99994	0.95351	-0.06869	0.21497	0.99996	1.1454	0.1959	0.2955	0.75244	1.1537	0.20622	0.74254	0.78108	0.9649	-0.05155	0.075746	0.28455	1.2726	0.34777	0.001782	0.23318	1.5186	0.60277	0.018219	0.52955	1.3986	0.48397	0.001792	0.077476	1.8729	0.90527
633.3378	0.56	domics Pos	0.080388	0.95356	1.3785	0.46307	0.032347	0.99994	0.87043	-0.20019	0.55124	0.99996	0.97315	-0.03926	0.001648	0.17581	0.54212	-0.88331	4.1E-05	0.000553	0.41094	-1.283	0.000127	0.023299	0.5187	-0.94704	0.30896	0.72874	0.84983	-0.23476	0.43024	0.96776	0.89907	-0.1535	0.015395	0.09885	2.0487	1.0347
282.2808	6.38	bolomics Pc	0.080413	0.99951	1.2077	0.2722	0.29404	0.99998	0.90976	-0.13644	0.73768	0.99941	1.0353	0.050031	0.21332	0.65365	0.83159	-0.26606	6.79E-06	0.000278	0.21983	-2.1856	0.016993	0.096516	0.6173	-0.69595	0.11919	0.65209	0.80768	-0.30814	0.39079	0.99956	0.93224	-0.10123	0.063264	0.30404	1.1501	0.20176
237.2186	5.68	bolomics Pc	0.080646	0.99951	1.2167	0.283	0.61872	0.99998	0.95097	-0.07253	0.59881	0.99941	1.0838	0.11615	0.22039	0.66625	0.81116	-0.30195	0.00021	0.002902	0.27075	-1.885	0.014541	0.091061	0.63197	-0.66208	0.11361	0.64238	0.83899	-0.25327	0.27354	0.99956	0.90957	-0.13674	0.15655	0.41371	1.185	0.24491
93.0702	2.05	domics Pos	0.081055	0.95356	1.1589	0.21272	0.51293	0.99994	0.92458	-0.11313	0.53844	0.99996	1.1084	0.14852	0.098012	0.51762	0.73467	-0.44483	2.65E-05	0.000439	0.22637	-2.1433	0.000955	0.043236	0.53727	-0.89627	0.21894	0.66097	0.83649	-0.25758	0.95405	0.98838	0.99387	-0.00887	0.001515	0.07528	1.5546	0.63658
434.7577	2.96	bolomics Pc	0.081256	0.99951	1.4277	0.51372	0.58985	0.99998	0.93992	-0.08939	0.88686	0.99941	1.0345	0.04892	0.061758	0.3264	0.70687	-0.50048	0.0099	0.048052	0.63597	-0.65296	0.008866	0.075866	0.73133	-0.45141	0.93732	0.9969	1.0756	0.10513	0.90403	0.99956	1.0378	0.053473	0.040329	0.28339	2.3684	1.2439
1012.728	0.54	domics Pos	0.081457	0.95356	1.2397	0.30998	0.61083	0.99994	1.042	0.059409	0.61179	0.99996	1.0382	0.054022	0.96946	0.99502	0.99987	-0.00019	0.022875	0.057802	0.72995	-0.45413	0.96839	0.98309	1.0048	0.006927	0.3794	0.76056	1.1109	0.15178	0.756	0.97988	1.0457	0.064492	0.08146	0.18304	1.4166	0.50242
697.3631	2.74	bolomics Pc	0.081768	0.99951	1.5576	0.63933	0.34829	0.99998	0.91798	-0.12347	0.98591	0.99941	1.0015	0.002176	0.011313	0.19869	0.61318	-0.70562	0.000714	0.007163	0.50569	-0.98366	0.002435	0.046224	0.56403	-0.82615	0.65542	0.99391	0.97507	-0.03642	0.7761	0.99956	1.0684	0.095413	0.026105	0.2688	2.2084	1.143
175.1492	6	bolomics Pc	0.082207	0.99951	1.1957	0.25787	0.58348	0.99998	0.95672	-0.06384	0.87551	0.99941	1.0163	0.023382	0.09472	0.41348	0.74606	-0.42264	8.19E-06	0.000292	0.22683	-2.1403	0.003609	0.051446	0.56825	-0.81539	0.11649	0.64715	0.78543	-0.34845	0.58346	0.99956	0.94949	-0.07477	0.22966	0.49625	1.0944	0.13014
185.1736	2.05	domics Pos	0.082534	0.95356	1.25	0.32193	0.5042	0.99994	0.91757	-0.1241	0.70653	0.99996	1.0387	0.054728	0.17196	0.60551	0.77825	-0.3617	3.65E-06	0.000144	0.14978	-2.7391	0.028337	0.14826	0.56013	-0.83616	0.17592	0.64616	0.79902	-0.3237	0.66135	0.97988	0.95237	-0.07041	0.013922	0.098791	1.4047	0.49024
193.6557	6.38	bolomics Pc	0.082721	0.99951	1.0928	0.12804	0.053522	0.99998	0.95724	-0.06305	0.94444	0.99941	1.0019	0.002762	0.82514	0.99905	0.99499	-0.00725	4.12E-06	0.000231	0.39836	-1.3278	0.055419	0.19951	0.80101	-0.32011	0.90811	0.9951	1.0003	0.000374	0.26603	0.99956	1.0247	0.035168	0.031389	0.2688	1.143	0.19282
604.2896	1.11	domics Pos	0.082734	0.95356	1.1834	0.24292	0.25789	0.99994	1.106	0.14538	0.13647	0.99996	1.1055	0.14468	0.74768	0.97379	0.97791	-0.03222	0.22018	0.31459	0.88057	-0.1835	0.011393	0.091661	1.3148	0.39482	0.002807	0.26175	1.4798	0.56542	0.000313	0.38756	1.422	0.50797	4.74E-05	0.07528	1.7828	0.83414
303.2453	1.69	domics Pos	0.082843	0.95356	1.21	0.27496	0.098695	0.99994	0.81713	-0.29136	0.25551	0.99996	0.88668	-0.17352	0.007498	0.19917	0.6499	-0.62172	5.96E-07	0.000144	0.1718	-2.5412	0.001488	0.050649	0.44035	-1.1833	0.017896	0.33809	0.6532	-0.61441	0.028695	0.59789	0.75474	-0.40594	0.43257	0.53867	1.0623	0.087166
246.2308	5.99	bolomics Pc	0.082911	0.99951	1.2171	0.28339	0.47744	0.99998	0.92662	-0.10996	0.76285	0.99941	1.0458	0.06455	0.20638	0.6443	0.8224	-0.28209	2.99E-06	0.000231	0.22148	-2.1747	0.012999	0.085844	0.59316	-0.7535	0.15956	0.73694	0.80051	-0.32101	0.37454	0.99956	0.91871	-0.12232	0.13229	0.385	1.1494	0.20084
262.858	12.72	olomics Ne	0.083115	0.99918	1.057	0.079997	0.20944	0.63192	0.94208	-0.08607	0.10907	0.99995	1.0671	0.093675	0.23728	0.99985	1.0914	0.12615	0.70363	0.99927	1.0251	0.035728	0.016829	0.40708	1.2	0.26306	0.027568	0.33786	1.1306	0.17704	0.1398	0.99512	1.0976	0.13439	0.051788	0.99941	1.0685	0.095533
404.9771	4.79	olomics Ne	0.08329	0.99918	1.0871	0.12044	0.4138	0.80087	0.9579	-0.06205	0.54252	0.99995	1.0202	0.028795	0.56506	0.99985	1.029	0.041273	0.78539	0.99927	1.0149	0.021355	0.10008	0.55533	1.0963	0.13268	0.003339	0.22316	1.1339	0.18129	0.22422	0.99512	1.0577	0.080982	0.30657	0.99941	1.053	0.074505
693.4069	2.74	bolomics Pc	0.083388	0.99951	1.4585	0.54445	0.93474	0.99998	1.0075	0.010761	0.81848	0.99941	0.97882	-0.03088	0.016944	0.20931	0.64717	-0.62778	0.012845	0.058725	0.63643	-0.65193	0.011797	0.083709	0.56922	-0.81293	0.68891	0.99391	1.1817	0.2409	0.97197	0.99956	1.1298	0.17608	0.017055	0.2688	2.4148	1.2719
590.3293	3.03	bolomics Pc	0.083593	0.99951	1.3753	0.45973	0.96577	0.99998	1.0072	0.010385	0.78451	0.99941	1.0271	0.038589	0.005504	0.1948	0.68814	-0.53923	0.000465	0.00541	0.61542	-0.70036	0.022376	0.1103	0.76555	-0.38543	0.64219	0.99391	0.96109	-0.05726	0.74688	0.99956	0.97712	-0.03339	0.00464	0.2688	1.8956	0.92265
560.3276	2.53	bolomics Pc	0.083611	0.99951	1.5925	0.67127	0.074289	0.99998	0.87895	-0.18615	0.77905	0.99941	1.0446	0.063012	0.009153	0.19702	0.61867	-0.69276	5.34E-05	0.000983	0.4576	-1.1278	0.002783	0.04765	0.64341	-0.63619	0.58976	0.99391	1.2508	0.32289	0.90263	0.99956	1.1095	0.14997	0.015763	0.2688	2.1513	1.1052
698.3651	0.58	domics Pos	0.083625	0.95356	1.4198	0.50571	0.18315	0.99994	0.85672	-0.22311	0.97994	0.99996	0.99736	-0.00381	0.010463	0.21362	0.55694	-0.8444	0.000565	0.003621	0.42527	-1.2336	0.001154	0.043885	0.5578	-0.84218	0.56886	0.85801	0.92674	-0.10976	0.77083	0.97988	1.0252	0.035964	0.013322	0.098791	2.4502	1.2929
521.2576	0.59	domics Pos	0.083656	0.95356	1.5224	0.60631	0.26721	0.99994	0.87493	-0.19276	0.86953	0.99996	0.98364	-0.0238	0.006529	0.1915	0.61471	-0.70202	0.000144	0.00133	0.37198	-1.4267	0.000259	0.026314	0.55532	-0.8486	0.6744	0.89976	0.98009	-0.02902	0.72363	0.97988	1.0398	0.056331	0.020382	0.10677	2.4668	1.3026
203.18	1.66	domics Pos	0.083796	0.95356	1.2383	0.30841	0.14953	0.99994	0.85795	-0.22104	0.96708	0.99996	1.0069	0.009984	0.039929	0.34602	0.76253	-0.39113	2.46E-06	0.000144	0.20671	-2.2743	0.000987	0.043236	0.51594	-0.95472	0.20907	0.65208	0.81526	-0.29467	0.31189	0.9524	0.89745	-0.1561	0.006359	0.090084	1.3838	0.46863
739.4556	3.22	bolomics Pc	0.083806	0.99951	1.436	0.5221	0.78204	0.99998	0.94954	-0.0747	0.56674	0.99941	1.0597	0.08366	0.03595	0.25923	0.64773	-0.62654	0.015074	0.066691	0.56534	-0.82281	0.030897	0.13499	0.67328	-0.57072	0.85231	0.9951	1.0272	0.038758	0.83386	0.99956	0.98217	-0.02596	0.016527	0.2688	1.8014	0.84913
1134.842	9.63	bolomics Pc	0.083923	0.99951	1.5758	0.65609	0.4855	0.99998	1.1251	0.17003	0.039265	0.99941	1.5287	0.6123	0.50909	0.98198	0.8777	-0.18819	0.25456	0.59753	0.77947	-0.35943	0.37415	0.68521	0.83441	-0.26117	0.69577	0.99391	1.0833	0.1154	0.054213	0.69529	1.5003	0.58528	0.094247	0.34866	2.0667	1.0473
697.362	0.58	domics Pos	0.084153	0.95356	1.492	0.57726	0.17714	0.99994	0.88573	-0.17506	0.92298	0.99996	1.0082	0.011795	0.005851	0.18075	0.5968	-0.74468	4.03E-05	0.000551	0.42934	-1.2198	0.000107	0.023299	0.56448	-0.82501	0.77769	0.94624	1.0017	0.002461	0.8993	0.98023	1.0923	0.12733	0.00866	0.092495	2.436	1.2845
201.644	2.06	domics Pos	0.084261	0.95356	1.1472	0.19812	0.22087	0.99994	0.92455	-0.11318	0.5696	0.99996	1.0275	0.039125	0.16895	0.6035	0.86702	-0.20587	1.83E-05	0.000345	0.28158	-1.8284	0.009143	0.086817	0.68788	-0.53977	0.54851	0.85184	0.94336	-0.08412	0.95796	0.98838	0.99755	-0.00354	0.007547	0.091944	1.3138	0.39373
240.2331	7.02	bolomics Pc	0.084374	0.99951	1.1867	0.247	0.27587	0.99998	0.85411	-0.22751	0.54262	0.99941	0.92282	-0.11588	0.19606	0.62955	0.76381	-0.38871	1.47E-05	0.000409	0.1558	-2.6823	0.005739	0.064803	0.50839	-0.97598	0.025106	0.32816	0.67313	-0.57104	0.099943	0.83286	0.8127	-0.2992	0.06449	0.3064	1.215	0.28097
795.5975	5.19	domics Pos	0.084394	0.95356	1.0993	0.13661	0.7565	0.99994	0.9905	-0.01377	0.84971	0.99996	0.99215	-0.01137	0.086033	0.49062	0.92506	-0.11239	0.001004	0.005526	0.78033	-0.35784	0.14765	0.42234	0.91874	-0.12228	0.23667	0.6775	1.0635	0.08876	0.30223	0.95107	0.96045	-0.05822	0.40683	0.51434	1.0385	0.054527
441.8896	6.37	bolomics Pc	0.084493	0.99951	1.3288	0.41012	0.25853	0.99998	0.83932	-0.2527	0.80587	0.99941	1.0242	0.034436	0.17512	0.59506	0.74363	-0.42734	4.03E-06	0.000231	0.087291	-3.518	0.014613	0.091119	0.49575	-1.0123	0.1105	0.64221	0.69538	-0.52412	0.3571	0.99956	0.89144	-0.16578	0.18833	0.45388	1.1821	0.2414
744.4076	0.56	domics Pos	0.084614	0.95356	1.4109	0.49666	0.19883	0.99994	0.91253	-0.13206	0.5508	0.99996	0.95901	-0.06039	0.004334	0.17581	0.54867	-0.866	0.000266	0.00203	0.40336	-1.3099	0.000132	0.023299	0.50451	-0.98705	0.28215	0.7098	0.81707	-0.29147	0.47726	0.96776	0.91129	-0.13402	0.010358	0.094875	2.1696	1.1175
434.6952	0.63	domics Pos	0.084635	0.95356	1.2161	0.28226	0.26	0.99994	1.1151	0.15718	0.44885	0.99996	1.0588	0.082447	0.19197	0.63374	0.88564	-0.17521	0.053371	0.10965	0.83359	-0.26259	0.50199	0.71797	1.0388	0.054951	0.74606	0.93393	1.0618	0.086518	0.043928	0.65325	1.172	0.22902	0.003637	0.085302	1.3886	0.47363
178.1669	2.05	domics Pos	0.084689	0.95356	1.2355	0.30505	0.19692	0.99994	0.83597	-0.25848	0.49387	0.99996	1.0787	0.10932	0.20075	0.64674	0.77316	-0.37117	7.96E-06	0.000198	0.20251	-2.304	0.011029	0.091128	0.51669	-0.95263	0.20007	0.64891	0.80103	-0.32006	0.77761	0.97988	0.95994	-0.05898	0.037024	0.13049	1.379	0.46363
492.8026	0.33	olomics Ne	0.084707	0.99918	1.1012	0.13903	0.005468	0.34605	0.79159	-0.33718	0.71235	0.99995	1.0299	0.042504	0.99962	0.99985	1.0168	0.024047	0.1327	0.99927	1.1302	0.17663	0.048727	0.48405	1.1906	0.25174	0.8637	0.95689	0.992	-0.01159	0.1711	0.99512	0.91831	-0.12294	0.79831	0.99941	0.98865	-0.01647
121.1018	1.67	domics Pos	0.084866	0.95356	1.1928	0.2543	0.21749	0.99994	0.8866	-0.17365	0.57297	0.99996	1.0488	0.068731	0.090762	0.50222	0.7528	-0.40967	2.45E-06	0.000144	0.18841	-2.4081	0.002346	0.061655	0.54614	-0.87267	0.21381	0.65483	0.81045	-0.3032	0.74111	0.97988	0.97115	-0.04223	0.005176	0.088007	1.3288	0.41014
829.4396	0.58	domics Pos	0.084879	0.95356	1.4348	0.52086	0.010716	0.99994	0.81758	-0.29057	0.46839	0.99996	0.95946	-0.0597	0.004651	0.17581	0.58331	-0.77767	4.01E-05	0.000551	0.4203	-1.2505	0.00024	0.026314	0.57405	-0.80074	0.53083	0.84189	0.92964	-0.10526	0.87068	0.98023	1.0765	0.10636	0.012985	0.098791	2.3447	1.2294
350.9875	4	olomics Ne	0.085312	0.99918	1.0697	0.097215	0.1043	0.52192	0.91309	-0.13117	0.76812	0.99995	0.99194	-0.01168	0.75073	0.99985	1.0264	0.03756	0.60094	0.99927	1.0269	0.038356	0.18991	0.65203	1.0737	0.10254	0.024255	0.33176	1.0785	0.10898	0.37892	0.99512	1.0411	0.058129	0.25113	0.99941	1.0444	0.062736
384.2054	0.58	domics Pos	0.085424	0.95356	1.5083	0.59289	0.047485	0.99994	0.78404	-0.351	0.6708	0.99996	0.9563	-0.06446	0.009009	0.19971	0.58714	-0.76823	0.002992	0.012257	0.43476	-1.2017	0.002559	0.061655	0.58745	-0.76747	0.86836	0.97532	1.048	0.067602	0.98468	0.99507	1.1201	0.16366	0.021124	0.10765	2.7756	1.4728
212.202	6.38	bolomics Pc	0.085546	0.99951	1.2174	0.28376	0.33366	0.99998	0.90705	-0.14075	0.62815	0.99941	1.057	0.07996	0.18544	0.61618	0.81451	-0.296	5.86E-06	0.000265	0.21664	-2.2066	0.015037	0.091796	0.6044	-0.72642	0.11924	0.65209	0.81476	-0.29555	0.63084	0.99956	0.95652	-0.06414	0.067539	0.30978	1.1701	0.22657
525.2885	0.58	domics Pos	0.086319	0.95356	1.4902	0.57552	0.061876	0.99994	0.8509	-0.23295	0.95228	0.99996	0.9981	-0.00275	0.008478	0.19971	0.59358	-0.75248	2.01E-05	0.000372	0.39078	-1.3556	0.000811	0.040694	0.55497	-0.84951	0.6045	0.86762	0.95473	-0.06683	0.84798	0.97988	1.07	0.097632	0.015963	0.099614	2.4568	1.2968
558.8568	0.54	domics Pos	0.086337	0.95356	1.1235	0.16795	0.96396	0.99994	0.995	-0.00724	0.68013	0.99996	0.96701	-0.0484	0.083989	0.48766	0.87292	-0.19608	0.031928	0.074324	0.78303	-0.35286	0.22519	0.51305	0.87646	-0.19024	0.47065	0.80647	0.93395	-0.09858	0.68672	0.97988	0.96543	-0.05076	0.059045	0.15646	1.1629	0.21777
459.2403	0.54	domics Pos	0.086348	0.95356	1.4349	0.52092	0.020804	0.99994	0.75899	-0.39785	0.51555	0.99996	0.93001	-0.10468	0.002341	0.17581	0.5765	-0.79461	0.0003	0.00222	0.42435	-1.2367	0.002704	0.061655	0.55527	-0.84874	0.42532	0.78878	0.89085	-0.16675	0.51253	0.96776	0.92051	-0.1195	0.045033	0.14081	2.1968	1.1354
168.1398	5.99	bolomics Pc	0.08668	0.99951	1.2599	0.33336	0.56723	0.99998	0.92515	-0.11223	0.3593	0.99941	1.1193	0.16257	0.24945	0.71498	0.80703	-0.30931	0.000336	0.00423	0.3077	-1.7004	0.011239	0.083534	0.5458	-0.87355	0.25933	0.86879	0.82415	-0.27903	0.9143	0.99956	0.98579	-0.02065	0.16526	0.42565	1.2089	0.27366
983.7817	10.07	domics Pos	0.086693	0.95356	0.85756	-0.22168	0.046787	0.99994	0.84783	-0.23815	0.42516	0.99996	0.92624	-0.11054	0.26715	0.72822	0.93015	-0.10446	0.00153	0.007423	0.73004	-0.45395	0.16178	0.44508	0.79754	-0.32637	0.94146	0.98682	1.0174	0.024928	0.60856	0.97371	0.95403	-0.0679	0.77048	0.8292	1.0283	0.040202
268.262	7.02	bolomics Pc	0.08676	0.99951	1.207	0.27144	0.29029	0.99998	0.8682	-0.20389	0.73123	0.99941	0.96201	-0.05588	0.12063	0.47798	0.72016	-0.47361	3.77E-06	0.000231	0.20326	-2.2986	0.004182	0.054724	0.50473	-0.9864	0.095487	0.60726	0.76361	-0.38909	0.15993	0.93927	0.84461	-0.24365	0.1222	0.37203	1.2006	0.26381
355.3721	8.54	bolomics Pc	0.087008	0.99951	0.84469	-0.24351	0.038202	0.99998	0.76211	-0.39192	0.06853	0.99941	0.80429	-0.31422	0.14648	0.54094	0.87956	-0.18515	0.9212	0.99688	0.99015	-0.01428	0.017304	0.097219	0.76126	-0.39355	0.2337	0.83874	0.82169	-0.28333	0.029748	0.58535	0.72995	-0.45414	0.018326	0.2688	0.80292	-0.31668
481.2621	0.59	domics Pos	0.087511	0.95356	1.4902	0.57553	0.20734	0.99994	0.84385	-0.24495	0.54992	0.99996	1.0458	0.064623	0.011305	0.21625	0.58985	-0.76159	0.000199	0.001693	0.423	-1.2413	0.011703	0.092481	0.60998	-0.71316	0.75407	0.93687	0.99386	-0.00889	0.80455	0.97988	1.0527	0.074066	0.012395	0.098791	2.4652	1.3017
163.1485	2.77	domics Pos	0.087645	0.95356	1.1742	0.23172	0.51403	0.99994	0.92214	-0.11694	0.12945	0.99996	0.90163	-0.1494	0.014233	0.22898	0.63662	-0.6515	1.31E-06	0.000144	0.20996	-2.2518	0.000153	0.023299	0.41504	-1.2687	0.005368	0.3088	0.63754	-0.64941	0.097225	0.84751	0.71947	-0.47499	0.26344	0.37334	1.1266	0.17201
280.2645	5.99	bolomics Pc	0.087752	0.99951	1.2122	0.2776	0.41812	0.99998	0.92231	-0.11668	0.62189	0.99941	1.0577	0.080991	0.1922	0.62544	0.83742	-0.25599	7.24E-06	0.000278	0.24636	-2.0211	0.013272	0.085844	0.63552	-0.65398	0.10009	0.61338	0.78838	-0.34304	0.25878	0.99956	0.92615	-0.11069	0.016967	0.2688	1.1783	0.23668
258.981	12.01	olomics Ne	0.087795	0.99918	1.1079	0.14789	0.56957	0.88137	1.0427	0.060339	0.080198	0.99995	1.1518	0.20393	0.88269	0.99985	0.99477	-0.00756	0.29818	0.99927	1.1027	0.141	0.25979	0.69038	1.1363	0.18435	0.029375	0.34346	1.1284	0.17424	0.40292	0.99512	1.0525	0.073767	0.14614	0.99941	1.0854	0.11821
222.1506	1.01	domics Pos	0.088108	0.95356	1.1786	0.23712	0.12217	0.99994	0.8067	-0.30989	0.96954	0.99996	1.0083	0.011957	0.52644	0.89938	0.94809	-0.0769	0.00047	0.003133	0.62331	-0.68198	0.04056	0.18724	0.7231	-0.46773	0.38903	0.7665	0.87139	-0.1986	0.47647	0.96776	0.91061	-0.13509	0.3252	0.43557	1.1295	0.17567
254.2488	5.68	bolomics Pc	0.088228	0.99951	1.2042	0.26804	0.51067	0.99998	0.93663	-0.09444	0.4888	0.99941	1.0692	0.096597	0.17897	0.6038	0.81146	-0.30141	1.94E-05	0.000473	0.26531	-1.9142	0.011943	0.083709	0.62418	-0.67997	0.21532	0.81115	0.83926	-0.25282	0.46536	0.99956	0.93087	-0.10335	0.092273	0.34594	1.145	0.19532
455.3059	2.06	domics Pos	0.088321	0.95356	1.2273	0.29545	0.65029	0.99994	0.97028	-0.04352	0.71188	0.99996	1.0504	0.070995	0.25214	0.70924	0.85689	-0.22282	0.000115	0.001134	0.22565	-2.1478	0.019537	0.12004	0.63302	-0.65967	0.58649	0.86163	0.94556	-0.08076	0.83842	0.97988	1.0494	0.069621	0.001203	0.07528	1.4276	0.51356
254.2479	7.02	bolomics Pc	0.088892	0.99951	1.2199	0.28676	0.30366	0.99998	0.84774	-0.23831	0.74925	0.99941	0.96264	-0.05493	0.11936	0.47414	0.72309	-0.46776	1.09E-05	0.000339	0.16868	-2.5677	0.001697	0.045425	0.49257	-1.0216	0.026466	0.33481	0.67944	-0.55758	0.10488	0.84495	0.78666	-0.34618	0.040644	0.28339	1.2813	0.35763
625.5259	2.05	domics Pos	0.088952	0.95356	1.3833	0.46809	0.26729	0.99994	0.83888	-0.25347	0.42454	0.99996	1.1213	0.16518	0.12179	0.54337	0.69173	-0.53173	0.000211	0.00175	0.11685	-3.0973	0.00317	0.063952	0.41007	-1.2861	0.18455	0.64793	0.72234	-0.46925	0.60285	0.97073	0.91702	-0.12498	0.006592	0.090084	1.5577	0.6394
306.2593	2.17	domics Pos	0.089022	0.95356	1.195	0.25702	0.93805	0.99994	1.0281	0.04	0.079955	0.99996	0.90489	-0.14419	0.037628	0.34165	0.76007	-0.3958	1.43E-06	0.000144	0.18136	-2.4631	0.002628	0.061655	0.57046	-0.8098	0.13335	0.59973	0.80163	-0.31899	0.21841	0.94047	0.82883	-0.27085	0.12306	0.22745	1.2263	0.29432
104.1078	0.54	domics Pos	0.089431	0.95356	1.1197	0.16313	0.76788	0.99994	0.98562	-0.0209	0.18011	0.99996	0.92751	-0.10856	0.45124	0.87374	0.93765	-0.09287	6.42E-05	0.000767	0.48102	-1.0558	0.4439	0.67607	0.91575	-0.12698	0.023813	0.33809	1.2937	0.37146	0.68854	0.97988	0.97087	-0.04265	0.002876	0.085002	1.522	0.60596
619.3135	0.54	domics Pos	0.08946	0.95356	1.6484	0.7211	0.56118	0.99994	0.90969	-0.13655	0.98476	0.99996	0.99516	-0.007	0.058281	0.41721	0.62085	-0.68768	0.00248	0.010589	0.44501	-1.1681	0.014373	0.10138	0.64264	-0.63791	0.25705	0.6916	0.80575	-0.3116	0.71363	0.97988	0.9697	-0.04439	0.010529	0.094875	2.2876	1.1939
1184.257	4.33	domics Pos	0.089594	0.95356	1.3228	0.40359	0.62215	0.99994	1.0346	0.049043	0.17068	0.99996	1.1414	0.19077	0.031256	0.30765	0.67851	-0.55955	0.00759	0.024862	0.51346	-0.96169	0.016505	0.10925	0.68903	-0.53736	0.57708	0.86151	1.1158	0.1581	0.80481	0.97988	1.1062	0.14561	0.020094	0.10652	1.8343	0.87521
111.1125	2.05	domics Pos	0.090047	0.95356	1.1932	0.25488	0.18571	0.99994	0.87336	-0.19535	0.98576	0.99996	0.99978	-0.00032	0.090311	0.50095	0.75967	-0.39655	1.19E-05	0.000262	0.17673	-2.5004	0.004379	0.071311	0.51419	-0.95962	0.16541	0.63453	0.80851	-0.30667	0.12855	0.88537	0.88281	-0.17982	0.006208	0.090084	1.332	0.41358
456.7703	3	bolomics Pc	0.090073	0.99951	1.5935	0.67224	0.94141	0.99998	0.99263	-0.01067	0.94835	0.99941	0.98133	-0.0272	0.097951	0.41865	0.71262	-0.48879	0.026978	0.10595	0.63832	-0.64766	0.052575	0.19379	0.65048	-0.62042	0.88785	0.9951	1.1659	0.22147	0.98416	0.99956	1.0242	0.034507	0.027727	0.2688	2.2052	1.1409
882.9741	12.02	olomics Ne	0.090163	0.99918	1.2503	0.3223	0.81211	0.9788	0.9833	-0.0243	0.144	0.99995	1.221	0.28801	0.041897	0.99985	1.2974	0.37559	0.15587	0.99927	0.85647	-0.22353	0.053076	0.48752	1.3737	0.45809	0.032622	0.3492	1.3579	0.44133	0.26497	0.99512	1.1566	0.20993	0.58523	0.99941	1.085	0.11766
742.3918	0.58	domics Pos	0.090547	0.95356	1.507	0.59168	0.2855	0.99994	0.90718	-0.14054	0.48989	0.99996	1.053	0.07457	0.012568	0.22648	0.61949	-0.69084	0.000249	0.001943	0.42363	-1.2391	0.000278	0.026512	0.58676	-0.76915	0.71938	0.92157	0.98801	-0.01741	0.95547	0.98838	1.1542	0.2069	0.023011	0.10989	2.4947	1.3189
85.1011	2.05	domics Pos	0.090567	0.95356	1.2021	0.26562	0.19804	0.99994	0.87424	-0.19389	0.92712	0.99996	1.0071	0.010215	0.17603	0.60979	0.86844	-0.20351	5.72E-06	0.000163	0.20082	-2.316	0.002651	0.061655	0.52454	-0.93088	0.33415	0.74176	0.88786	-0.1716	0.3088	0.9524	0.87774	-0.18814	0.000491	0.07528	1.5367	0.61987
215.1141	0.61	domics Pos	0.090809	0.95356	0.64242	-0.63842	0.42982	0.99994	0.86235	-0.21365	0.25552	0.99996	0.79248	-0.33556	0.21542	0.66508	0.74903	-0.4169	0.010318	0.0315	0.76034	-0.39529	0.17304	0.45921	0.73544	-0.44333	0.11569	0.5679	0.67928	-0.55792	0.3067	0.9524	0.79334	-0.33399	0.86332	0.90039	1.0269	0.038269
741.4282	3.22	bolomics Pc	0.090811	0.99951	1.3572	0.44058	0.053506	0.99998	0.85816	-0.22069	0.45475	0.99941	0.93272	-0.10049	0.001024	0.12936	0.58145	-0.78228	0.002905	0.020066	0.58622	-0.77049	0.001178	0.044437	0.65941	-0.60076	0.45258	0.99391	0.90333	-0.14668	0.66184	0.99956	0.96458	-0.05203	0.026336	0.2688	1.6314	0.70615
302.2441	6.01	bolomics Pc	0.090938	0.99951	1.1915	0.25273	0.76051	0.99998	0.97708	-0.03346	0.90137	0.99941	0.99891	-0.00158	0.12242	0.48261	0.81503	-0.29507	0.000106	0.001675	0.27403	-1.8676	0.005223	0.062556	0.61548	-0.70022	0.012521	0.27021	0.69014	-0.53505	0.051719	0.68405	0.83349	-0.26276	0.36463	0.64385	1.075	0.10435
561.9682	0.53	domics Pos	0.091157	0.95356	1.4339	0.51994	0.1157	0.99994	0.85776	-0.22135	0.93151	0.99996	0.9934	-0.00955	0.004852	0.17581	0.58856	-0.76474	0.000329	0.00237	0.41453	-1.2705	0.000674	0.03855	0.56451	-0.82492	0.59617	0.86481	0.94547	-0.08089	0.53207	0.96776	0.92914	-0.10603	0.018373	0.10363	2.2391	1.1629
1049.695	4.51	domics Pos	0.091204	0.95356	1.5906	0.66959	0.49556	0.99994	0.94132	-0.08724	0.015797	0.99996	1.307	0.38627	0.13734	0.56129	0.83394	-0.26199	0.011359	0.033838	0.55513	-0.84909	0.039299	0.18274	0.76857	-0.37975	0.77567	0.94551	1.1264	0.17177	0.53632	0.96776	1.2082	0.27282	0.022835	0.10945	2.4768	1.3085

852.8659	12.01	olomics Po	0.091318	0.99951	0.83813	-0.25475	0.020363	0.99998	0.78664	-0.34623	0.099381	0.99941	0.91068	-0.13498	0.061207	0.32568	0.91423	-0.12938	0.046418	0.15893	0.85576	-0.22472	0.17717	0.44118	0.89045	-0.16739	0.015023	0.28681	0.8651	-0.20906	0.1505	0.93461	0.9148	-0.12847	0.005043	0.2688	0.85047	-0.23367
948.8116	10.02	omics Pos	0.091391	0.95356	0.85291	-0.22954	0.40599	0.99994	0.91544	-0.12746	0.39869	0.99996	0.93867	-0.09131	0.10655	0.53093	0.85962	-0.21824	0.007937	0.025807	0.74696	-0.42089	0.16715	0.45275	0.81956	-0.28709	0.92849	0.98398	0.9893	-0.01552	0.72356	0.97988	0.9714	-0.04187	0.24554	0.35397	1.1079	0.14785
165.1642	1.67	omics Pos	0.091467	0.95356	1.2569	0.32986	0.44548	0.99994	0.90505	-0.14393	0.62047	0.99996	1.0622	0.087044	0.13515	0.55953	0.76603	-0.38452	6.72E-06	0.000182	0.18795	-2.4116	0.010058	0.08787	0.54719	-0.86988	0.18575	0.64793	0.79175	-0.33688	0.47608	0.96776	0.91183	-0.13317	0.014192	0.098791	1.3812	0.4659
188.0777	10.16	omics Pos	0.091476	0.95356	1.1741	0.23155	0.50259	0.99994	1.0354	0.050161	0.38309	0.99996	1.0647	0.090442	0.67289	0.95737	1.0229	0.032618	0.012047	0.035362	0.82857	-0.2713	0.45779	0.68696	1.1144	0.15624	0.38081	0.76056	1.0848	0.11746	0.011836	0.52955	1.1905	0.25161	0.016577	0.099614	1.3062	0.38536
836.7715	0.56	omics Pos	0.091601	0.95356	1.1477	0.19871	0.069422	0.99994	0.84044	-0.25079	0.4777	0.99996	0.93965	-0.0898	0.87718	0.9874	0.98534	-0.0213	0.016171	0.044263	0.72465	-0.46465	0.35256	0.61857	0.9088	-0.13796	0.71063	0.91681	0.96561	-0.05049	0.54442	0.96776	0.94287	-0.08487	0.072128	0.17377	1.2112	0.27645
798.9625	3.28	olomics Po	0.091629	0.99951	1.3736	0.458	0.66125	0.99998	0.96297	-0.05443	0.65017	0.99941	0.95657	-0.06405	0.00097	0.12936	0.52143	-0.93946	0.003039	0.020811	0.50666	-0.98092	0.000189	0.036471	0.52943	-0.9175	0.96228	0.9974	1.0727	0.10125	0.22818	0.99956	0.8029	-0.3167	0.025895	0.2688	1.633	0.70749
266.2571	6.38	olomics Po	0.091657	0.99951	1.1898	0.25071	0.2845	0.99998	0.91061	-0.13509	0.60267	0.99941	1.0428	0.060419	0.216	0.65907	0.8392	-0.25291	1.04E-05	0.000332	0.21368	-2.2265	0.018536	0.10085	0.6243	-0.67969	0.11345	0.64238	0.8148	-0.29548	0.54801	0.99956	0.95364	-0.06848	0.041241	0.28426	1.1643	0.21946
152.1503	2.05	omics Pos	0.091815	0.95356	1.2739	0.34928	0.50225	0.99994	0.9181	-0.12327	0.23239	0.99996	1.0959	0.13211	0.19597	0.64175	0.78376	-0.35152	3.02E-06	0.000144	0.19862	-2.3319	0.005	0.073217	0.52777	-0.92201	0.3058	0.7283	0.84335	-0.2458	0.83054	0.97988	0.98002	-0.02912	0.001994	0.07995	1.5037	0.5885
967.7827	10.05	omics Pos	0.091874	0.95356	1.1077	0.14755	0.92226	0.99994	1.0112	0.016128	0.8146	0.99996	1.026	0.037031	0.57914	0.9205	0.97145	-0.04179	0.00162	0.007748	0.81731	-0.29105	0.25717	0.54373	1.067	0.093494	0.87753	0.97532	1.0225	0.032036	0.24038	0.94365	1.0762	0.106	0.050222	0.14663	1.2624	0.33622
746.5981	5.55	omics Pos	0.092	0.95356	1.0911	0.12572	0.44264	0.99994	1.0489	0.068831	0.94682	0.99996	1.0045	0.006516	0.12872	0.55024	0.92415	-0.1138	0.010397	0.031613	0.80284	-0.31682	0.97803	0.98735	1.0029	0.004143	0.1982	0.64891	1.1275	0.17311	0.33714	0.96524	1.0948	0.13062	0.088797	0.18968	1.2241	0.29177
219.2112	5.68	olomics Po	0.092008	0.99951	1.237	0.30683	0.43835	0.99998	0.92618	-0.11063	0.67559	0.99941	1.0587	0.082355	0.24552	0.71017	0.86999	-0.20094	5.81E-05	0.001064	0.27643	-1.855	0.001712	0.045425	0.55314	-0.85427	0.11829	0.65209	0.77458	-0.36851	0.29025	0.99956	0.89723	-0.15645	0.07612	0.32186	1.2011	0.26432
423.2109	2.05	omics Pos	0.092064	0.95356	1.3008	0.37942	0.81973	0.99994	1.0124	0.017777	0.31327	0.99996	1.1511	0.20301	0.43451	0.86496	0.87415	-0.19404	0.000122	0.001178	0.28177	-1.8274	0.01616	0.10794	0.64126	-0.64101	0.78228	0.94754	0.96344	-0.05374	0.58717	0.97073	1.1374	0.18576	0.024204	0.11096	1.4338	0.51986
1038.779	10.02	omics Pos	0.092108	0.95356	1.1034	0.14195	0.1653	0.99994	0.92667	-0.10988	0.75369	0.99996	1.0424	0.059894	0.36905	0.82243	0.90485	-0.14425	0.009769	0.030235	0.83494	-0.26026	0.67976	0.83448	0.96734	-0.04791	0.94053	0.98682	1.0188	0.02692	0.35023	0.96776	1.0466	0.065712	0.076151	0.17781	1.2005	0.26362
355.3759	4.65	omics Pos	0.092153	0.95356	0.86414	-0.21066	0.083017	0.99994	0.76976	-0.37752	0.11418	0.99996	0.83193	-0.26546	0.026617	0.28915	0.81894	-0.28817	0.002055	0.009241	0.67138	-0.5748	0.008621	0.084941	0.59136	-0.75789	0.059928	0.43943	0.68254	-0.55101	0.000855	0.41831	0.6484	-0.62505	0.16661	0.27406	0.89926	-0.15319
352.8983	10.07	omics Pos	0.092565	0.95356	1.0911	0.1258	0.38649	0.99994	0.95961	-0.05948	0.72935	0.99996	0.98291	-0.02488	0.32378	0.7879	0.94946	-0.07482	0.001966	0.008967	0.79039	-0.33937	0.41196	0.65457	0.93461	-0.09757	0.81483	0.95952	1.0113	0.016189	0.92437	0.98531	1.0079	0.01139	0.016628	0.099614	1.2136	0.27929
532.6223	3.28	olomics Po	0.092598	0.99951	1.3545	0.43776	0.31814	0.99998	0.91449	-0.12897	0.91382	0.99941	1.0032	0.004594	0.00427	0.18109	0.6485	-0.62482	0.001632	0.013356	0.56066	-0.83481	0.00056	0.039708	0.63138	-0.66342	0.29074	0.90579	0.82451	-0.2784	0.63104	0.99956	0.95063	-0.07305	0.072316	0.3167	1.6662	0.7366
736.432	2.8	olomics Po	0.092611	0.99951	1.4386	0.52469	0.30387	0.99998	0.89631	-0.15793	0.66808	0.99941	1.0405	0.057275	0.007209	0.1948	0.56682	-0.81904	0.012446	0.057402	0.55201	-0.85722	0.015425	0.092755	0.64525	-0.63208	0.92931	0.9951	1.0537	0.075524	0.86088	0.99956	1.0596	0.083485	0.016105	0.2688	2.2884	1.1943
503.3061	2.57	olomics Po	0.092936	0.99951	1.5011	0.58602	0.15073	0.99998	0.86862	-0.20321	0.48372	0.99941	1.0613	0.085801	0.049676	0.29469	0.70307	-0.50826	0.002163	0.016415	0.53711	-0.89671	0.14648	0.38873	0.80651	-0.31024	0.69534	0.99391	1.1399	0.18896	0.74691	0.99956	1.1507	0.20253	0.063353	0.30404	2.0861	1.0608
767.8469	6.2	omics Pos	0.092952	0.95356	1.5074	0.59209	0.31145	0.99994	0.86549	-0.20841	0.89734	0.99996	1.0103	0.014837	0.17051	0.60402	0.72496	-0.46402	0.004085	0.015513	0.52934	-0.91773	0.043429	0.1969	0.67907	-0.55837	0.93021	0.98468	0.99711	-0.00417	0.60262	0.97073	1.2312	0.30005	0.046125	0.14289	2.1908	1.1315
128.1082	5.99	olomics Po	0.093005	0.99951	1.1694	0.22579	0.42732	0.99998	0.92239	-0.11655	0.82585	0.99941	1.0277	0.039437	0.12649	0.49162	0.75193	-0.41134	1.02E-06	0.000231	0.21313	-2.2302	0.012555	0.085318	0.5557	-0.84762	0.20822	0.80086	0.81119	-0.30189	0.40898	0.99956	0.91828	-0.12299	0.15041	0.40449	1.1402	0.18925
164.1519	2.05	omics Pos	0.09303	0.95356	1.2411	0.31166	0.40763	0.99994	0.89492	-0.16017	0.41352	0.99996	1.0748	0.10409	0.20499	0.65191	0.8015	-0.31922	2.16E-06	0.000144	0.17876	-2.4839	0.020147	0.12174	0.56386	-0.8266	0.2543	0.69047	0.84022	-0.25116	0.5756	0.9703	0.93723	-0.09353	0.018677	0.10427	1.3455	0.42812
934.7048	0.56	omics Pos	0.093289	0.95365	0.85247	-0.23029	0.071211	0.99994	0.77593	-0.36601	0.061789	0.99996	0.79872	-0.32424	0.007891	0.19917	0.66869	-0.58059	0.001211	0.00631	0.64875	-0.62426	0.010187	0.08787	0.68961	-0.53614	0.011244	0.3324	0.72696	-0.46006	0.049156	0.6775	0.79155	-0.33725	0.36243	0.47117	0.89987	-0.15222
626.9725	12.01	olomics Ne	0.093309	0.99918	1.288	0.36512	0.85806	0.98874	1.0129	0.018521	0.79393	0.99995	1.0339	0.048136	0.69868	0.99985	1.0281	0.039936	0.89822	0.99927	0.99329	-0.00972	0.13694	0.61523	1.1864	0.24663	0.019375	0.29445	1.3116	0.39132	0.36849	0.99512	1.12	0.16351	0.38859	0.99941	1.1101	0.15068
255.2517	5.68	olomics Po	0.093569	0.99951	1.224	0.29162	0.2774	0.99998	0.87825	-0.1873	0.38799	0.99941	1.107	0.14662	0.2826	0.76084	0.84947	-0.23537	1.44E-05	0.000408	0.2397	-2.0607	0.021845	0.10951	0.61683	-0.69707	0.14293	0.70688	0.83278	-0.264	0.6787	0.99956	0.95895	-0.06047	0.057178	0.30239	1.1888	0.2495
655.3581	3.11	olomics Po	0.093864	0.99951	1.4006	0.48607	0.27772	0.99998	0.89388	-0.16185	0.77553	0.99941	1.0258	0.036803	0.023054	0.22443	0.64016	-0.64349	0.001318	0.011562	0.55554	-0.84805	0.00174	0.045425	0.63526	-0.65458	0.60934	0.99391	0.94664	-0.07911	0.7253	0.99956	0.97732	-0.0331	0.028927	0.2688	1.9185	0.94001
181.633	6.39	olomics Po	0.093934	0.99951	1.2191	0.28585	0.51679	0.99998	1.0827	0.11461	0.017777	0.99941	1.2256	0.2935	0.22317	0.67079	1.1743	0.23181	0.000438	0.005149	0.553	-0.85464	0.15032	0.39492	0.88735	-0.17242	0.15366	0.72748	1.1708	0.22749	0.13756	0.92391	1.1514	0.20339	0.013994	0.2688	1.3117	0.39149
193.1222	0.84	omics Pos	0.094222	0.95932	0.87999	-0.18443	0.039091	0.99994	0.78624	-0.34695	0.14122	0.99996	0.82687	-0.27428	0.30306	0.76085	0.87384	-0.19456	0.000101	0.001032	0.58552	-0.77222	0.00953	0.087209	0.66426	-0.59018	0.16379	0.63418	0.78093	-0.35673	0.14222	0.902	0.86144	-0.21517	0.35918	0.46822	0.90869	-0.13814
426.2245	2.91	olomics Po	0.09436	0.99951	1.5212	0.60518	0.24068	0.99998	0.85332	-0.22885	0.58583	0.99941	1.0437	0.061756	0.017815	0.20936	0.64303	-0.63705	0.010169	0.048904	0.63604	-0.65281	0.054715	0.1981	0.74777	-0.41934	0.97281	0.9974	1.0679	0.094729	0.97027	0.99956	1.1068	0.14644	0.025895	0.2688	2.1321	1.0923
131.9219	10.64	olomics Ne	0.094644	0.99918	1.0511	0.071934	0.49978	0.85536	0.96584	-0.05015	0.91313	0.99995	1.0096	0.013844	0.5865	0.99985	1.0284	0.040426	0.6565	0.99927	1.0256	0.036411	0.006151	0.36417	1.0954	0.13143	0.031604	0.34429	1.1613	0.21569	0.93253	0.99512	1.0128	0.018391	0.26874	0.99941	0.95292	-0.06957
224.1626	8.37	olomics Po	0.095097	0.99951	1.1026	0.14087	0.48654	0.99998	1.0425	0.060017	0.5591	0.99941	1.0367	0.051933	0.2036	0.64188	1.1053	0.14438	0.045647	0.15715	1.1062	0.14561	0.20759	0.48509	1.0978	0.13457	0.3053	0.92336	1.0677	0.094454	0.64339	0.99956	0.96344	-0.05374	0.24519	0.51325	0.9116	-0.13352
417.2389	3	olomics Po	0.095359	0.99951	1.6016	0.6795	0.32922	0.99998	0.85891	-0.21942	0.92406	0.99941	0.98187	-0.02639	0.003734	0.17363	0.63914	-0.6458	0.003565	0.023109	0.65467	-0.61116	0.009081	0.076393	0.65404	-0.61255	0.93096	0.9951	1.0932	0.1285	0.95539	0.99956	1.1812	0.2403	0.039829	0.28339	2.1253	1.0877
404.2142	2.87	olomics Po	0.095452	0.99951	1.4543	0.54032	0.02488	0.99998	0.79225	-0.33597	0.16373	0.99941	1.112	0.15316	0.042326	0.2802	0.68231	-0.5515	0.006176	0.033951	0.56719	-0.8181	0.003198	0.049867	0.67351	-0.57023	0.93908	0.9969	1.0548	0.076912	0.85966	0.99956	1.078	0.1084	0.017062	0.2688	2.2428	1.1653
1066.764	10.03	omics Pos	0.095603	0.96694	0.91657	-0.12569	0.23731	0.99994	0.91661	-0.12562	0.25993	0.99996	0.90825	-0.13885	0.022471	0.26776	0.89534	-0.15948	0.006631	0.022574	0.77475	-0.3682	0.062851	0.25094	0.78216	-0.35446	0.11016	0.5591	0.89323	-0.16289	0.023312	0.54215	0.87034	-0.20034	0.051872	0.14847	1.1356	0.18345
525.6303	3.28	olomics Po	0.095607	0.99951	1.4191	0.505	0.10401	0.99998	0.88113	-0.18257	0.51076	0.99941	1.0781	0.10852	0.003131	0.17363	0.59755	-0.74288	0.001242	0.011022	0.54854	-0.86632	0.005336	0.062645	0.67268	-0.572	0.9921	0.99906	1.0366	0.051914	0.9395	0.99956	1.0851	0.1178	0.023971	0.2688	1.7187	0.78134
798.6289	10.3	olomics Ne	0.095637	0.99918	0.89469	-0.16054	0.005134	0.34605	0.83907	-0.25313	0.02922	0.99995	0.89994	-0.1521	0.11904	0.99985	0.91923	-0.1215	0.39549	0.99927	0.96427	-0.05249	0.54631	0.87267	1.0416	0.058804	0.80169	0.95074	1.0184	0.02628	0.18629	0.99512	0.90084	-0.15066	0.28421	0.99941	0.91435	-0.12918
789.444	0.58	omics Pos	0.095817	0.96694	1.4829	0.5684	0.26383	0.99994	0.89563	-0.15902	0.77863	0.99996	0.97772	-0.03251	0.015641	0.24	0.57709	-0.79312	0.000185	0.001596	0.40895	-1.29	0.000342	0.028292	0.53114	-0.91284	0.59483	0.8647	0.94446	-0.08245	0.79476	0.97988	1.0279	0.039744	0.016117	0.099614	2.446	1.2904
785.4138	0.58	omics Pos	0.096028	0.96694	1.4421	0.52819	0.22032	0.99994	0.91164	-0.13346	0.55976	0.99996	1.0461	0.064953	0.005182	0.17581	0.57922	-0.78783	0.000121	0.00117	0.43695	-1.1944	0.000427	0.032599	0.5836	-0.77695	0.78968	0.95091	1.0038	0.005414	0.96284	0.9885	1.1108	0.15154	0.012103	0.0984	2.3964	1.2609
1059.782	10.03	omics Pos	0.096533	0.96694	1.1109	0.15177	0.85815	0.99994	0.99073	-0.01344	0.49575	0.99996	0.94452	-0.08235	0.89232	0.9874	0.99156	-0.01223	0.00581	0.020494	0.75446	-0.40648	0.56966	0.76437	0.94537	-0.08105	0.63312	0.8822	1.0438	0.061795	0.53991	0.96776	0.95035	-0.07347	0.037681	0.13071	1.2157	0.28179
473.9152	0.54	omics Pos	0.096655	0.96694	1.3589	0.44241	0.033426	0.99994	0.85196	-0.23114	0.66715	0.99996	0.97457	-0.03716	0.001963	0.17581	0.63657	-0.65162	2.13E-05	0.000378	0.42854	-1.2225	0.000356	0.028292	0.59232	-0.75554	0.55376	0.85214	0.93686	-0.09409	0.53822	0.96776	0.93465	-0.0975	0.023628	0.11051	2.2136	1.1464
205.1954	6.38	olomics Po	0.096682	0.99951	1.2214	0.28855	0.67988	0.99998	0.95648	-0.06419	0.58774	0.99941	1.0546	0.076662	0.11006	0.44968	0.77916	-0.36001	2.62E-05	0.000576	0.24699	-2.0175	0.005581	0.064158	0.56977	-0.81155	0.07799	0.57132	0.76586	-0.38485	0.2852	0.99956	0.88732	-0.17248	0.38142	0.65621	1.0856	0.11853
177.1644	1.66	omics Pos	0.096992	0.96694	1.2723	0.34744	0.38809	0.99994	0.86226	-0.21381	0.34829	0.99996	1.1399	0.18888	0.24469	0.70602	0.79293	-0.33474	5.87E-05	0.000722	0.1975	-2.3401	0.01811	0.11549	0.55843	-0.84055	0.37844	0.76056	0.8304	-0.26812	0.92838	0.98573	1.0035	0.004986	0.033408	0.12517	1.4417	0.52781
1080.733	10.07	omics Pos	0.097027	0.96694	1.1196	0.16294	0.32315	0.99994	0.9068	-0.14114	0.35085	0.99996	0.95639	-0.06433	0.86602	0.9874	1.0218	0.031152	0.00187	0.008611	0.77861	-0.36104	0.36442	0.62554	0.91047	-0.13532	0.99026	0.99702	1.0077	0.011094	0.4847	0.96776	0.95121	-0.07217	0.013045	0.098791	1.1732	0.23051
721.3964	3.2	olomics Po	0.097064	0.99951	1.3611	0.44476	0.18678	0.99998	0.85731	-0.22211	0.4616	0.99941	1.0547	0.076815	0.020856	0.22258	0.6281	-0.67094	0.00038	0.004633	0.53972	-0.8897	0.001199	0.044437	0.62295	-0.68281	0.96615	0.9974	1.0349	0.04947	0.9301	0.99956	1.0419	0.059246	0.050542	0.2932	1.7581	0.81406
492.2546	0.57	omics Pos	0.097681	0.96704	1.5236	0.60746	0.084902	0.99994	0.79931	-0.32318	0.99879	0.99996	1.006	0.00868	0.022387	0.26776	0.64506	-0.63248	0.005292	0.019034	0.46003	-1.1202	0.003043	0.063179	0.55147	-0.85865	0.82994	0.96527	1.023	0.032839	0.90948	0.98239	1.1427	0.19251	0.025693	0.113	2.7841	1.4772
345.901	12.08	olomics Po	0.097702	0.99951	1.118	0.16098	0.050794	0.99998	1.2271	0.29524	0.83546	0.99941	1.015	0.021534	0.39619	0.89016	1.0962	0.13252	0.004304	0.026377	1.1721	0.22904	0.24819	0.53954	1.2055	0.26969	0.35389	0.96247	1.0738	0.10268	0.015064	0.47452	1.212	0.27744	0.23001	0.49625	1.2223	0.28954
1183.291	5.98	omics Pos	0.098444	0.96704	1.7124	0.77604	0.59629	0.99994	0.92351	-0.1148	0.074203	0.99996	1.2616	0.33525	0.074057	0.46065	0.71269	-0.48866	0.009885	0.030429	0.55612	-0.84653	0.080561	0.29367	0.71284	-0.48835	0.64403	0.88775	1.1185	0.16158	0.4163	0.96776	1.3368	0.41873	0.13393	0.23874	2.2078	1.1426
162.1363	1.67	omics Pos	0.098592	0.96704	1.2863	0.36319	0.33935	0.99994	0.85011	-0.23429	0.62473	0.99996	1.0589	0.082618	0.34295	0.79881	0.81196	-0.30052	6.86E-05	0.000807	0.28173	-1.8276	0.014392	0.10138	0.54997	-0.86258	0.46621	0.80647	0.87029	-0.20044	0.2429	0.94365	0.79577	-0.32958	0.061129	0.15809	1.3874	0.47239
114.0921	1.66	omics Pos	0.098898	0.96704	1.3116	0.39133	0.63424	0.99994	0.90703	-0.14077	0.48337	0.99996	1.1145	0.15635	0.38945	0.83582	0.82151	-0.28366	5.78E-05	0.000716	0.19934	-2.3267	0.075172	0.28295	0.63122	-0.6638	0.4564	0.80379	0.86156	-0.21497	0.80789	0.97988	1.0329	0.046691	0.048303	0.14485	1.4057	0.49134
907.829	10.02	omics Pos	0.098937	0.96704	1.0995	0.1368	0.59082	0.99994	1.0349	0.049434	0.84238	0.99996	0.98896	-0.01602	0.48325	0.88725	1.0451	0.063703	0.031041	0.072748	0.89042	-0.16744	0.39599	0.64416	0.92575	-0.11131	0.32589	0.73527	1.105	0.14399	0.77874	0.97988	1.0297	0.042245	0.001655	0.07528	1.2894	0.36666
569.3138	0.58	omics Pos	0.099017	0.96704	1.4278	0.51381	0.11787	0.99994	0.85878	-0.21964	0.6287	0.99996	0.96238	-0.05532	0.010802	0.21362	0.58001	-0.78586	2.35E-05	0.000406	0.36944	-1.4366	0.000269	0.026314	0.55995	-0.83662	0.57951	0.86163	0.94112	-0.08756	0.74643	0.97988	1.0409	0.057896	0.016228	0.099614	2.3592	1.2383
1141.237	4.38	omics Pos	0.099164	0.96704	1.3594	0.44301	0.65085	0.99994	0.90369	-0.1461	0.47648	0.99996	1.0987	0.13573	0.22065	0.67468	0.78113	-0.35637	0.016081	0.044112	0.52241	-0.93675	0.14591	0.42117	0.73422	-0.44571	0.94584	0.98829	0.9928	-0.01042	0.49158	0.96776	1.1725	0.22958	0.061411	0.15854	1.8618	0.89667
760.9639	3.25	olomics Po	0.099265	0.99951	1.3304	0.41187	0.051453	0.99998	0.82302	-0.281	0.63791	0.99941	0.95796	-0.06196	0.000677	0.12797	0.59007	-0.76105	0.000583	0.006262	0.54423	-0.8777	0.01705	0.096516	0.63051	-0.6654	0.41906	0.98828	0.87348	-0.19516	0.71216	0.99956	0.97277	-0.03984	0.11364	0.36506	1.6984	0.76416
81.0713	6.39	olomics Po	0.099314	0.99951	1.1816	0.24072	0.61977	0.99998	0.95981	-0.05918	0.46529	0.99941	0.93973	-0.08969	0.10665	0.43932	0.80043	-0.32115	7.98E-06	0.000289	0.292	-1.776	0.002751	0.047591	0.56576	-0.82173	0.020161	0.30386	0.70809	-0.498	0.028373	0.57661	0.81954	-0.28711	0.69973	0.86437	0.96968	-0.04443
1046.216	8.85	olomics Po	0.099334	0.99951	1.4228	0.50876	0.98675	0.99998	1.0224	0.031963	0.029593	0.99941	1.2883	0.36547	0.10123	0.42517	0.85119	-0.23245	0.036019	0.13224	0.72733	-0.45931	0.009535	0.078629	0.72607	-0.46182	0.43467	0.99391	1.1559	0.20897	0.95826	0.99956	1.0718	0.10004	0.084429	0.33453	1.8804	0.91105
699.8856	3.16	olomics Po	0.099821	0.99951	1.3842	0.46905	0.22168	0.99998	0.88516	-0.17599	0.90446	0.99941	1.0101	0.01453	0.01441	0.20085	0.59899	-0.7394	0.003756	0.024093	0.59789	-0.74206	0.007079	0.070405	0.63368	-0.65817	0.81562	0.99391	0.99709	-0.00421	0.59958	0.99956	0.94199	-0.08622	0.007807	0.2688	1.8822	0.91244
710.4004	3.2	olomics Po	0.099868	0.99951	1.4369	0.52299	0.31043	0.99998	0.8684	-0.20357	0.55031	0.99941	0.91701	-0.12499	0.029569	0.24447	0.63585	-0.65325	0.003861	0.024408	0.54901	-0.86508	0.039363	0.15897	0.68117	-0.55392	0.7629	0.99391	1.1094	0.14976	0.9901	0.99956	1.0609	0.085269	0.062737	0.30404	1.8386	0.87862
122.1056	6.38	olomics Po	0.099963	0.99951	1.2041	0.26801	0.92217	0.99998	0.99516	-0.007	0.96764	0.99941	0.99519	-0.00696	0.050798	0.29486	0.72265	-0.46862	5.1E-06	0.000251	0.30555	-1.7105	0.015868	0.093702	0.58112	-0.78309	0.047767	0.44092	0.79951	-0.32281	0.39666	0.99956	0.8886	-0.17039	0.76641	0.90005	0.96642	-0.04927
437.2394	2.96	olomics Po	0.10015	0.99951	1.4073	0.49294	0.62116	0.99998	0.93225	-0.10121	0.5544	0.99941	1.0798	0.11071	0.043853	0.28307	0.64544	-0.63164	0.007058	0.037055	0.60366	-0.7282	0.052599	0.19379	0.68471	-0.54643	0.96356	0.9974	1.1181	0.16109	0.98231	0.99956	1.1126	0.15393	0.015909	0.2688	2.093	1.0656
426.728	2.91	olomics Po	0.10028	0.99951	1.6271	0.70231	0.5305	0.99998	0.90399	-0.14563	0.36747	0.99941	1.1022	0.14044	0.04367	0.2827	0.67489	-0.56727	0.023599	0.095671	0.62275	-0.68328	0.021516	0.10949	0.63139	-0.66339	0.92588	0.9951	1.0596	0.083585	0.88012	0.99956	1.0804	0.11155	0.015332	0.2688	2.057	1.0405
545.6431	3.3	olomics Po	0.10029	0.99951	1.3233	0.40411	0.050867	0.99998	0.80403	-0.31468	0.93348	0.99941	1.0036	0.005187	0.002126	0.14926	0.54273	-0.88169	0.002845	0.01974	0.56193	-0.83154	0.003111	0.049681	0.53512	-0.90208	0.36295	0.96545	0.85508	-0.22588	0.573	0.99956	0.9313	-0.10268	0.031323	0.2688	1.7316	0.79211
436.973	11.99	olomics Ne	0.10054	0.99918	0.87447	-0.19351	0.04658	0.47152	0.83391	-0.26203	0.16007	0.99995	0.88612	-0.17443	0.33293	0.99985	0.94262	-0.08525	0.85444	0.99927	0.99323	-0.0098	0.73044	0.94555	0.98029	-0.02871	0.81184	0.95121	0.98867	-0.01644	0.034461	0.99512	0.86401	-0.21088	0.002503	0.99941	0.78729	-0.34504
212.2013	1.66	omics Pos	0.1007	0.97296	1.2435	0.31435	0.5384	0.99994	0.90777	-0.13961	0.66195	0.99996	1.078	0.10835	0.26963	0.73017	0.81691	-0.29175	2.12E-05	0.000378	0.19623	-2.3494	0.014555	0.10138	0.54662	-0.87138	0.25891	0.69327	0.82507	-0.27742	0.55186	0.96776	0.91736	-0.12444	0.014688	0.098791	1.4376	0.52369
737.4356	2.8	olomics Po	0.10093	0.99951	1.5265	0.61024	0.45276	0.99998	0.8757	-0.1915	0.69557	0.99941	1.0418	0.059135	0.028474	0.24036	0.67442	-0.56828	0.018429	0.07874	0.66142	-0.59635	0.023561	0.11383	0.64847	-0.62489	0.80409	0.99391	1.0834	0.11556	0.71164	0.99956	0.98439	-0.02271	0.047005	0.2875	2.3335	1.2225
719.4148	3.2	olomics Po	0.10103	0.99951	1.3251	0.40614	0.018801	0.99998	0.78991	-0.34024	0.45725	0.99941	0.92234	-0.11663	0.011465	0.19869	0.6108	-0.71123	0.000854	0.008333	0.55243	-0.85615	0.003358	0.049867	0.62053	-0.68842	0.47702	0.99391	0.90269	-0.1477	0.51716	0.99956	0.92114	-0.1185	0.060093	0.30404	1.5582	0.63992
763.8236	10.05	omics Pos	0.10118	0.97296	1.1474	0.19835	0.27215	0.99994	0.93076	-0.10352	0.64148	0.99996	1.0383	0.054174	0.56568	0.91164	0.96156	-0.05655	0.001086	0.005858	0.7268	-0.46037	0.71775	0.85308	0.97127	-0.04205	0.83554	0.96659	1.0234	0.033423	0.39298	0.96776	1.1142	0.15604	0.76316	0.82318	1.0366	0.051806
67.0551	2.05	omics Pos	0.1012	0.97296	1.1962	0.25847	0.15799	0.99994	0.79308	-0.33447	0.79991	0.99996	0.96306	-0.05431	0.021221	0.26474	0.66345	-0.59193	5.85E-06	0.000165	0.16564	-2.5939	0.02669	0.1447	0.5782	-0.79035	0.20584	0.65133	0.75989	-0.39614	0.54026	0.96776	0.91732	-0.1245	0.097526	0.19865	1.2957	0.37376
454.9747	5.08	olomics Ne	0.10139	0.99918	1.0601	0.084142	0.31516	0.74753	0.94864	-0.07607	0.79682	0.99995	1.0057	0.008249	0.76636	0.99985	1.0253	0.036029	0.20558	0.99927	1.0816	0.11322	0.046435	0.48405	1.138	0.18653	0.088329	0.44746	1.0844	0.11693	0.6506	0.99512	1.025	0.035621	0.085672	0.99941	1.0786	0.10918
886.7701	10.07	omics Pos	0.10149	0.97296	1.0652	0.091117	0.21175	0.99994	0.89984	-0.15226	0.22856	0.99996	0.94234	-0.08567	0.097127	0.51652	0.90208	-0.14868	0.004095	0.015536	0.78005	-0.35836	0.4616	0.68833	0.94232	-0.08572	0.62453	0.87671	0.96861	-0.04601	0.68331	0.97988	0.98264	-0.02527	0.058223	0.15546	1.1226	0.16687
752.5573	9.35	olomics Po	0.10174	0.99951	1.328	0.40929	0.38094	0.99998	1.1337	0.18106	0.81595	0.99941	0.98671	-0.0193	0.33063	0.82484	1.1059	0.14526	0.017932	0.077165	1.2631	0.33692	0.003154	0.049867	1.5244	0.60825	0.002582	0.20152	1.7294	0.79026	0.014217	0.47452	1.4027	0.48817	0.000512	0.2688	1.4639	0.54985
344.8758	10.14	omics Pos	0.1021	0.97296	1.2522	0.32447	0.94058	0.99994	0.99954	-0.00066	0.01538	0.99996	1.3594	0.44292	0.16525	0.59723	1.1592	0.21314	0.17546	0.26729	0.86406	-0.21079	0.75819	0.87447	0.96986	-0.04415	0.024974	0.33809	1.3506	0.43356	0.35594	0.96776	1.1551	0.20803	0.000887	0.07528	1.7781	0.8303
95.0869	6.38	olomics Po	0.1023	0.99951	1.1256	0.17073	0.34664	0.99998	0.91944	-0.12117	0.59984	0.99941	0.97054	-0.04314	0.072446	0.35072	0.76839	-0.38009	4.06E-06	0.000231	0.26231	-1.9306	0.001504	0.045425	0.56559	-0.82216	0.00771	0.23352	0.68534	-0.54511	0.027124	0.5548	0.80622	-0.31075	0.87286	0.95061	0.99318	-0.00987
745.4183	0.58	omics Pos	0.10238	0.97296	1.4789	0.56451	0.2631	0.99994	0.90089	-0.15057	0.83555	0.99996	1.0204	0.029093	0.011212	0.21625	0.61458	-0.70232	0.000141	0.001314	0.41582	-1.266	0.000143	0.023299	0.57505	-0.79825	0.77898	0.94624	0.99646	-0.00512	0.89298	0.98023	1.0714	0.099489	0.007566	0.091944	2.4722	1.3058
448.2292	0.58	omics Pos	0.10245	0.97296	1.5174	0.6016	0.1113	0.99994	0.84615	-0.24102	0.77426	0.99996	1.0394	0.055796	0.008055	0.19917	0.59854	-0.74047	0.002278	0.009951	0.44745	-1.1602	0.002062	0.05956	0.62505	-0.67796	0.89603	0.97532	1.0518	0.07286	0.84761	0.97988	1.1002	0.13775	0.029813	0.11881	2.5805	1.3677
157.1424	2.05	omics Pos	0.10277	0.97296	1.2891	0.36636	0.78874	0.99994	0.96186	-0.05611	0.25197	0.99996	1.1369	0.18509	0.19049	0.63224	0.77123	-0.37476	8.71E-05	0.00094	0.16632	-2.588	0.012518	0.094516	0.5551	-0.84919	0.60096	0.86607	0.92585	-0.11115	0.80687	0.97988	1.0837	0.11593	0.008962	0.092545	1.5464	0.62891
426.2157	0.58	omics Pos	0.10292	0.97296	1.5912	0.67008	0.16984	0.99994	0.80717	-0.30906	0.96904	0.99996	0.98887	-0.01615	0.013884	0.22898	0.60567	-0.7234	0.000308	0.002266	0.42007	-1.2513	0.002856	0.061884	0.55494	-0.8496	0.95764	0.98985	1.069	0.096206	0.90102	0.98023	1.0965	0.13295	0.021207	0.10765	2.6595	1.4111
209.1912	2.05	omics Pos	0.10314	0.97296	1.2544	0.32704	0.37869	0.99994	0.88377	-0.17826	0.98826	0.99996	0.99553	-0.00647	0.19476	0.64018	0.78531	-0.34867	2.26E-05	0.000393	0.20406	-2.2929	0.008272	0.084185	0.55106	-0.85972	0.29317	0.7186	0.82843	-0.27155	0.81688	0.97988	0.96631	-0.04944	0.014846	0.098791	1.4252	0.51117
506.6428	0.34	olomics Ne	0.10395	0.99918	0.83567	-0.25899	0.12865	0.54655	0.86915	-0.20232	0.15828	0.99995	0.93561	-0.09602	0.19178	0.99985	0.9056	-0.14306	0.88684	0.99927	0.99799	-0.00291	0.47851	0.8481	0.94158	-0.08685	0.38632	0.73607	0.95281	-0.06974	0.001136	0.69914	0.79526	-0.3305	0.28266	0.99941	0.91975	-0.12069
574.9728	12	olomics Ne	0.10398	0.99918	1.1141	0.15588	0.009435	0.34605	0.85733	-0.22208	0.028571	0.99995	0.86411	-0.21072	0.51098	0.99985	0.9404	-0.08866	0.72959	0.99927	0.97674	-0.03396	0.70552	0.94209	0.9751	-0.03638	0.5731	0.85384	1.0819	0.11361	0.028752	0.99512	0.8888	-0.17007	0.050303	0.99941	0.84332	-0.24585
415.7301	2.9	olomics Po	0.104	0.99951	1.6331	0.70764	0.42958	0.99998	0.9117	-0.13337	0.44504	0.99941	1.114	0.1557	0.053191	0.29934	0.72349	-0.46695	0.022567	0.092545	0.60148	-0.73341	0.11017	0.31906	0.80749	-0.30849	0.94452	0.9974	1.1719	0.22888	0.73478	0.99956	1.2033	0.26694	0.01615	0.2688	2.2	1.1375
179.1744	6	olomics Po	0.10417	0.99951	1.183	0.2424	0.50262	0.99998	0.92982	-0.10498	0.90951	0.99941	1.0229	0.032601	0.07027	0.34478	0.74149	-0.43149	2.86E-06	0.000231	0.26319	-1.9258	0.0019	0.045425	0.58593	-0.7712	0.099818	0.61338	0.77531	-0.36716	0.45674	0.99956	0.92257	-0.11627	0.38601	0.65797	1.0781	0.1085
442.3905	2.05	omics Pos	0.10423	0.97296	1.3125	0.39233	0.67792	0.99994	0.93573	-0.09584	0.48508	0.99996	1.1166	0.15916	0.16422	0.596	0.72613	-0.46171	1.34E-06	0.000144	0.0626	-3.9977	0.02257	0.13179	0.45571	-1.1338	0.39837	0.77223	0.8447	-0.24348	0.96577	0.98939	1.0028	0.004008	0.00595	0.089674	1.6147	0.69129
746.422	0.58	omics Pos	0.10427	0.97296	1.4587	0.54469	0.23718	0.99994	0.8794	-0.18541	0.72101	0.99996	1.035	0.04969	0.014751	0.2319	0.57674	-0.79401	7.27E-05	0.000843	0.42959	-1.219	0.001045	0.043236	0.55686	-0.84462	0.63091	0.88216	0.96111	-0.05722	0.88829	0.98023	1.0558	0.078316	0.017101	0.10008	2.4655	1.3019
109.1025	6.38	olomics Po	0.10437	0.99951	1.142	0.19153	0.18023	0.99998	0.88822	-0.17102	0.40699	0.99941	0.94548	-0.08089	0.12714	0.49321	0.78828	-0.34322	1.25E-06	0.000231	0.28178	-1.8274	0.001357	0.045425	0.57615	-0.79549	0.027942	0.33959	0.73211	-0.44987	0.032327	0.58535	0.78422	-0.35067	0.50689	0.75745	0.94153	-0.08692
196.1703	1.67	omics Pos	0.10443	0.97296	1.2033	0.26703	0.20564	0.99994	0.8424	-0.24743	0.7075	0.99996	1.0521	0.073238	0.090097	0.50079	0.73792	-0.43846	3.47E-06	0.000144	0.18505	-2.434	0.006815	0.080671	0.5135	-0.96155	0.25048	0.68761	0.83142	-0.26636	0.39843	0.96776	0.90373	-0.14603	0.024011	0.11071	1.3396	0.42182
372.7394	0.64	omics Pos	0.10497	0.97296	1.1769	0.23504	0.17219	0.99994	1.1272	0.17278	0.36009	0.99996	1.0415	0.058686	0.31622	0.77841	1.037	0.052395	0.012835	0.037019	0.88325	-0.17911	0.30385	0.58337	0.93311	-0.09988	0.092963	0.52281	1.0596	0.083557	0.074174	0.77699	1.0882	0.12192	0.021639	0.1083	1.2355	0.30506
128.1083	6.38	olomics Po	0.10497	0.99951	1.1849	0.24474	0.34868	0.99998	0.90845	-0.13853	0.84938	0.99941	1.0311	0.044198	0.21327	0.65365	0.80954	-0.30483	2.23E-05	0.000513	0.20801	-2.2653	0.013076	0.085844	0.58507	-0.77331	0.11981	0.65311	0.79946	-0.3229	0.39379	0.99956	0.92488	-0.11267	0.13654	0.3911	1.1411	0.19045
648.3803	2.69	olomics Po	0.10537	0.99951	1.4096	0.49528	0.72246	0.99998	0.95161	-0.07157	0.74308	0.99941	1.0505	0.071098	0.022827	0.22443	0.582	-0.78091	0.004726	0.028467	0.54431	-0.87749	0.006167	0.065804	0.62664	-0.67428	0.85273	0.9951	1.1642	0.21939	0.86653	0.99956	1.1802	0.23906	0.02633	0.2688	2.1241	1.0869
433.2346	6.04	olomics Ne	0.1054	0.99918	1.2312	0.30009	0.62828	0.91093	0.95763	-0.06245	0.76396	0.99995	0.9743	-0.03757	0.16013	0.99985	0.84735	-0.23897	0.000116	0.023712	0.38661	-1.3711	0.25403	0.69038	0.85442	-0.22699	0.064133	0.40279	1.2636	0.33754	0.97844	0.99512	0.99407	-0.00858	0.013221	0.99941	1.4158	0.50163
404.9152	10.03	omics Pos	0.10561	0.97296	1.148	0.19914	0.60517	0.99994	1.0356	0.050455	0.47558	0.99996	1.0371	0.052508	0.94811	0.99318	1.0023	0.003245	0.047142	0.099696	0.87393	-0.19441	0.7212	0.85455	0.97043	-0.04331	0.76364	0.94202	1.0179	0.025618	0.25945	0.94365	1.0878	0.12147	0.009933	0.094875	1.2885	0.36568
492.9731	5.28	olomics Ne	0.10567	0.99918	1.0644	0.090053	0.33203	0.74862	0.95609	-0.06479	0.20357	0.99995	1.0572	0.08028	0.53905	0.99985	1.0471	0.066426	0.2624	0.99927	1.0422	0.059603	0.039397	0.48405	1.1171	0.15978	0.016336	0.29108	1.134	0.18148	0.55829	0.99512	1.0226	0.032225	0.82067	0.99941	1.0097	0.013975
313.7784	2.06	omics Pos	0.10572	0.97296	1.3286	0.4099	0.33531	0.99994	0.89521	-0.1597	0.41613	0.99996	1.1366	0.18477	0.50547	0.89133	0.9022	-0.14848	5.68E-05	0.000707	0.14017	-2.8348	0.061846	0.24902	0.63882	-0.64652	0.96382	0.99138	1.0291	0.041388	0.45379	0.96776	1.1455	0.19599	0.005559	0.08815	1.571	0.65168
653.3759	3.11	olomics Po	0.10572	0.99951	1.3859	0.47082	0.068233	0.99998	0.83926	-0.25281	0.29012	0.99941	0.93037	-0.10412	0.000239	0.077904	0.57735	-0.79249	0.000381	0.004633	0.54771	-0.86852	0.003459	0.050446	0.55866	-0.83996	0.60824	0.99391	0.94864	-0.07607	0.51014	0.99956	0.92116	-0.11847	0.025174	0.2688	1.6587	0.7301
128.1076	7.02	olomics Po	0.10575	0.99951	1.2397	0.31	0.16143	0.99998	0.81652	-0.29244	0.7768	0.99941	0.96348	-0.05367	0.22497	0.67297	0.81193	-0.30057	1.25E-05	0.000364	0.21659	-2.207	0.01118	0.083534	0.57456	-0.79947	0.10256	0.61787	0.72721	-0.45956	0.69147	0.99956	0.9467	-0.07901	0.034209	0.27077	1.3219	0.40256
296.2955	6.74	olomics Po	0.10584	0.99951	1.3126	0.39246	0.18098	0.99998	0.81288	-0.29889	0.85473	0.99941	1.0261	0.037145	0.26334	0.73802	0.81372	-0.2974	1.76E-06	0.000231	0.16741	-2.5786	0.01543	0.092755	0.56004	-0.8364	0.15324	0.72748	0.77104	-0.37512	0.35926	0.99956	0.89526	-0.15962	0.039694	0.28339	1.2194	0.28614
318.2402	1.01	omics Pos	0.10593	0.97296	1.385	0.46993	0.95843	0.99994	0.98087	-0.02786	0.52654	0.99996	1.097	0.13357	0.24116	0.70182	0.80851	-0.30667	3.68E-05	0.000527	0.25243	-1.986	0.015815	0.10692	0.62215	-0.68466	0.39096	0.76822	0.85595	-0.2244	0.85348	0.97988	0.96164	-0.05642	0.025007	0.11203	1.4155	0.50133
918.4955	0.57	omics Pos	0.10601	0.97296	1.4217	0.50762	0.020444	0.99994	0.79029	-0.33955	0.24555	0.99996	0.91109	-0.13433	0.014515	0.2306	0.57534	-0.79751	0.000322	0.002341	0.42388	-1.2383	4.07E-05	0.019289	0.5334	-0.90672	0.37516	0.76056	0.87115	-0.199	0.8005	0.97988	1.0386	0.054627	0.018065	0.10311	2.2025	1.1392
801.7046	7.7	omics Pos	0.10619	0.97296	1.2596	0.33297	0.55019	0.99994	1.2151	0.28105	0.66568	0.99996	1.0942	0.12989	0.82011	0.98382	0.99015	-0.01429	0.73317	0.77277	1.1572	0.21067	0.013566	0.098526	1.3672	0.45127	0.11129	0.56029	1.3841	0.469	0.079946	0.79727	1.3891	0.47419	0.007645	0.091944	1.5626	0.64392
274.8821	9.92	omics Pos	0.10623	0.97296	1.1735	0.23084	0.89766	0.99994	1.0487	0.068533	0.92795	0.99996	1.0111	0.015942	0.79391	0.97671	0.98513	-0.02162	0.002121	0.009461	0.80356	-0.31552	0.50421	0.71874	0.9441	-0.08298	0.30511	0.7283	1.0982	0.13512	0.66941	0.97988	1.0259	0.036886	0.004425	0.085451	1.3787	0.46326
936.6543	9.18	olomics Po	0.1064	0.99951	1.4534	0.53939	0.86338	0.99998	0.9871	-0.01873	0.51469	0.99941	1.1132	0.15478	0.48425	0.96238	0.87829	-0.18723	0.02967	0.11425	0.62739	-0.67257	0.1102	0.31906	0.72343	-0.46708	0.72792	0.99391	1.0905	0.12494	0.88913	0.99956	1.0205	0.029209	0.20394	0.46828	1.5752	0.65557
364.2167	1.66	omics Pos	0.10663	0.97296	1.2961	0.37423	0.2216	0.99994	0.83787	-0.2552	0.52386	0.99996	1.0831	0.11518	0.17027	0.60402	0.7827	-0.35346	9.24E-06	0.000213	0.20391	-2.294	0.028847	0.14965	0.5888	-0.76414	0.56603	0.85656	0.91438	-0.12913	0.81724	0.97988	1.0565	0.079242	0.050499	0.14715	1.3196	0.40012
881.7964	0.52	omics Pos	0.10672	0.97296	1.1266	0.17192	0.2288	0.99994	0.89482	-0.16034	0.81361	0.99996	1.0191	0.027247	0.72425	0.96859	0.98085	-0.0279	0.000365	0.002578	0.74454	-0.42557	0.3907	0.64146	0.91118	-0.1342	0.44975	0.80156	1.0432	0.061055	0.27832	0.94365	0.94783	-0.0773	0.098649	0.20008	1.2552	0.32787
674.887	3.14	olomics Po	0.10677	0.99951	1.3682	0.45224	0.13295	0.99998	0.87174	-0.19803	0.99814	0.99941	1.0006	0.000794	0.021987	0.2227	0.67408	-0.569	0.001592	0.013194	0.56656	-0.8197	0.010395	0.080593	0.62793	-0.67133	0.96621	0.9974	1.0402	0.056907	0.72684	0.99956	0.97451	-0.03726	0.010557	0.2688	1.7921	0.84167

247.9878	12.01	olomics Ne	0.10688	0.99918	0.77561	-0.36659	0.005186	0.34605	0.69953	-0.51554	0.19324	0.99995	0.85911	-0.21909	0.60225	0.99985	0.94208	-0.08609	0.087948	0.99927	0.84424	-0.24428	0.11309	0.58058	0.83229	-0.26483	0.24336	0.62946	0.84065	-0.25042	0.6334	0.99512	0.94559	-0.08072	0.24839	0.99941	0.86647	-0.20679
1142.829	9.7	olomics Po	0.10732	0.99951	1.7743	0.82727	0.76077	0.99998	1.0654	0.091441	0.13813	0.99941	1.1926	0.25413	0.009607	0.19702	0.65962	-0.60028	0.008445	0.042358	0.654	-0.61264	0.039619	0.15926	0.74314	-0.42829	0.94605	0.9974	1.0606	0.084929	0.71346	0.99956	1.3665	0.45049	0.036577	0.27732	2.0959	1.0676
156.133	7.02	olomics Po	0.10733	0.99951	1.3499	0.4329	0.63265	0.99998	0.92054	-0.11945	0.72377	0.99941	1.0649	0.090747	0.25766	0.72978	0.78844	-0.34293	0.000708	0.007129	0.31309	-1.6754	0.006109	0.065713	0.5022	-0.99366	0.099926	0.61338	0.74613	-0.4225	0.88667	0.99956	0.96663	-0.04896	0.024621	0.2688	1.3992	0.4846
632.5216	2.05	domics Pos	0.10792	0.97296	1.1809	0.23985	0.79739	0.99994	0.96486	-0.05161	0.74883	0.99996	0.95787	-0.0621	0.23801	0.69648	0.8066	-0.31007	9.7E-05	0.001	0.24592	-2.0237	0.0011	0.043236	0.46503	-1.1046	0.35414	0.75239	0.84849	-0.23703	0.73777	0.97988	1.0483	0.068048	0.026954	0.11548	1.4284	0.51438
125.0967	2.05	domics Pos	0.10794	0.97296	1.236	0.30571	0.27461	0.99994	0.87531	-0.19214	0.75219	0.99996	1.0343	0.048683	0.1673	0.60157	0.7754	-0.36699	3.48E-06	0.000144	0.17055	-2.5517	0.006132	0.077936	0.55228	-0.85652	0.18033	0.64793	0.80687	-0.30959	0.68601	0.97988	0.95713	-0.06321	0.00619	0.090084	1.3952	0.48045
281.2677	5.99	olomics Po	0.10824	0.99951	1.2285	0.29689	0.42027	0.99998	0.91908	-0.12173	0.71448	0.99941	1.0508	0.071548	0.18753	0.6189	0.83029	-0.26831	2.93E-05	0.000615	0.23658	-2.0796	0.01159	0.083709	0.61633	-0.69822	0.094761	0.60726	0.7736	-0.37034	0.25937	0.99956	0.91504	-0.12809	0.033569	0.2688	1.1845	0.24427
270.978	10.09	domics Pos	0.10842	0.97501	1.1999	0.26287	0.42097	0.99994	1.0758	0.10541	0.7086	0.99996	1.0363	0.051499	0.28104	0.74609	1.0645	0.090164	0.01335	0.038188	0.79186	-0.33669	0.45141	0.68194	1.046	0.064878	0.037851	0.37121	1.2384	0.30845	0.39122	0.96776	1.0817	0.1133	0.013923	0.098791	1.346	0.4287
667.5742	2.05	domics Pos	0.10868	0.97505	1.3178	0.39815	0.30667	0.99994	0.85551	-0.22514	0.49445	0.99996	1.0986	0.13566	0.1169	0.53727	0.72204	-0.46985	5.36E-05	0.000677	0.13207	-2.9206	0.0116	0.091956	0.48347	-1.0485	0.25093	0.68824	0.80898	-0.30583	0.68369	0.97988	0.94151	-0.08696	0.004561	0.085788	1.5803	0.66022
1185.856	5.33	domics Pos	0.10951	0.98022	1.6361	0.71028	0.2646	0.99994	0.87785	-0.18796	0.28239	0.99996	1.1209	0.16472	0.014225	0.22898	0.70841	-0.49734	0.003302	0.013169	0.53595	-0.89984	0.067364	0.26399	0.68526	-0.54528	0.41812	0.78128	1.2079	0.27246	0.53615	0.96776	1.2574	0.33041	0.007887	0.092077	2.2414	1.1644
722.4014	3.2	olomics Po	0.10959	0.99951	1.3402	0.42249	0.18295	0.99998	0.85426	-0.22724	0.76358	0.99941	0.95907	-0.0603	0.03181	0.24911	0.58271	-0.77916	0.000541	0.006024	0.46775	-1.0962	0.014835	0.091119	0.60433	-0.72659	0.91103	0.9951	1.0126	0.018117	0.60337	0.99956	0.93205	-0.10153	0.05473	0.29746	1.5302	0.61373
750.5423	5.18	domics Pos	0.10992	0.98115	1.072	0.10032	0.57353	0.99994	1.0545	0.076541	0.77712	0.99996	1.0409	0.057839	0.1531	0.58077	0.92936	-0.10569	0.005754	0.020334	0.79084	-0.33854	0.70392	0.84729	0.97637	-0.0345	0.1858	0.64793	1.1994	0.26227	0.14102	0.89982	1.1212	0.1651	0.70239	0.77787	1.0323	0.045918
179.1063	4.02	olomics Po	0.11005	0.99951	0.86533	-0.20867	0.0978	0.99998	0.83942	-0.25253	0.42454	0.99941	0.9105	-0.13527	0.7526	0.99905	0.97127	-0.04206	0.32041	0.69367	0.91816	-0.12319	0.042941	0.16845	0.77819	-0.36181	0.16092	0.73694	0.81265	-0.2993	0.061033	0.71737	0.84547	-0.24217	0.052874	0.2951	0.83742	-0.25598
636.4081	2.73	olomics Po	0.11011	0.99951	1.3517	0.43476	0.41112	0.99998	0.88655	-0.17373	0.93099	0.99941	0.99316	-0.00991	0.015194	0.20428	0.58688	-0.76886	0.00135	0.011665	0.51858	-0.94736	0.017758	0.098138	0.6016	-0.73313	0.49249	0.99391	0.91372	-0.13018	0.99041	0.99956	1.0665	0.09288	0.062826	0.30404	2.089	1.0628
880.823	10.02	domics Pos	0.11013	0.98115	1.1826	0.24198	0.49078	0.99994	0.93769	-0.09282	0.76467	0.99996	0.97383	-0.03826	0.55617	0.90877	1.062	0.086811	0.034788	0.079271	0.78734	-0.34494	0.51819	0.72722	0.92502	-0.11245	0.9421	0.9869	1.0079	0.01136	0.50196	0.96776	1.0702	0.097819	0.21653	0.32497	1.1799	0.23871
448.2373	2.96	olomics Po	0.11031	0.99951	1.4803	0.56587	0.84306	0.99998	0.97706	-0.03348	0.83857	0.99941	1.0204	0.029199	0.045018	0.28536	0.66599	-0.58643	0.008413	0.042333	0.61005	-0.713	0.042094	0.16637	0.71598	-0.48201	0.99225	0.99906	1.0916	0.12647	0.9412	0.99956	1.2188	0.28541	0.004497	0.2688	2.3065	1.2057
762.9396	3.25	olomics Po	0.11043	0.99951	1.3987	0.48413	0.48564	0.99998	0.88551	-0.17541	0.82897	0.99941	1.0293	0.041701	0.037329	0.26415	0.60642	-0.72161	0.010109	0.048765	0.55841	-0.84061	0.030185	0.1328	0.6347	-0.65584	0.7397	0.99391	0.94952	-0.07473	0.73179	0.99956	0.94792	-0.07717	0.050542	0.2932	1.6683	0.73839
609.3097	0.58	domics Pos	0.11054	0.98203	1.5401	0.62302	0.40468	0.99994	0.92628	-0.11048	0.40749	0.99996	1.0737	0.10259	0.01829	0.25335	0.60767	-0.71864	0.000157	0.001416	0.42316	-1.2407	0.000675	0.03855	0.59535	-0.7482	0.88029	0.97532	1.032	0.04551	0.86697	0.98023	1.1773	0.23547	0.014912	0.09885	2.5475	1.3491
554.6471	3.32	olomics Po	0.11056	0.99951	1.3588	0.44229	0.15759	0.99998	0.88759	-0.17203	0.74776	0.99941	0.97326	-0.0391	0.002056	0.14926	0.56713	-0.81825	0.002729	0.0191	0.52202	-0.93783	0.006404	0.066819	0.61227	-0.70776	0.4633	0.99391	0.90544	-0.1433	0.45713	0.99956	0.9026	-0.14785	0.001919	0.2688	1.9356	0.95276
259.9629	0.58	domics Pos	0.11075	0.98203	1.3098	0.38936	0.66009	0.99994	1.1324	0.17933	0.50406	0.99996	1.1195	0.16287	0.88419	0.9874	1.0056	0.008046	0.90257	0.91725	0.97955	-0.0298	0.032075	0.16113	1.3635	0.44728	0.010741	0.3324	1.5076	0.5923	0.3024	0.95107	1.1263	0.17155	0.009009	0.092545	1.5747	0.65512
114.0926	6.38	olomics Po	0.11086	0.99951	1.1673	0.22318	0.2518	0.99998	0.90412	-0.14541	0.77157	0.99941	1.0429	0.06065	0.22835	0.6786	0.83368	-0.26244	5.62E-06	0.000265	0.21749	-2.201	0.018975	0.10165	0.60321	-0.72928	0.11625	0.64715	0.80005	-0.32184	0.38592	0.99956	0.93533	-0.09646	0.061784	0.30404	1.1611	0.21555
163.1493	6.38	olomics Po	0.11158	0.99951	1.1612	0.21566	0.35979	0.99998	0.91065	-0.13502	0.55292	0.99941	0.95354	-0.06863	0.091637	0.4063	0.78154	-0.35561	1.23E-06	0.000231	0.26274	-1.9283	0.005294	0.062631	0.55993	-0.83668	0.024681	0.32665	0.73227	-0.44955	0.073737	0.76911	0.82086	-0.28479	0.61649	0.81955	0.9602	-0.0586
1044.798	10.02	domics Pos	0.11162	0.98517	1.1147	0.15671	0.89396	0.99994	0.99643	-0.00516	0.98155	0.99996	1.0036	0.005169	0.11997	0.54112	0.92188	-0.11736	0.039677	0.087753	0.85662	-0.22326	0.99336	0.99597	1.0015	0.002163	0.20973	0.65208	1.0465	0.065526	0.17622	0.91806	1.1087	0.14886	0.002954	0.085302	1.2782	0.35406
179.093	0.3	olomics Po	0.11173	0.99951	1.1948	0.25682	0.95136	0.99998	1.01	0.014375	0.4502	0.99941	1.0795	0.11037	0.61694	0.99905	1.0919	0.12685	0.72512	0.99688	0.952	-0.07097	0.24462	0.53548	1.1498	0.20137	0.008579	0.23862	1.7362	0.79595	0.22748	0.99956	1.2248	0.2926	0.11933	0.36992	1.3386	0.42069
578.3219	3.02	olomics Po	0.11175	0.99951	1.4027	0.48824	0.23504	0.99998	0.9132	-0.131	0.52285	0.99941	0.96414	-0.05269	0.000144	0.077904	0.58862	-0.7646	8.56E-05	0.001435	0.48592	-1.0412	8.03E-05	0.034543	0.5928	-0.75438	0.47908	0.99391	0.91658	-0.12566	0.55404	0.99956	0.94088	-0.08792	0.058039	0.30327	1.6522	0.72443
917.731	5.4	domics Pos	0.1125	0.98897	1.1301	0.17647	0.92515	0.99994	0.99201	-0.01157	0.89812	0.99996	1.0135	0.019285	0.49636	0.89009	0.94196	-0.08626	0.046706	0.09895	0.78089	-0.3568	0.16559	0.45081	0.88796	-0.17143	0.18991	0.64891	1.1985	0.26128	0.086218	0.82317	1.2088	0.27363	0.005605	0.08815	1.4145	0.50028
259.2756	1.91	domics Pos	0.11257	0.98897	1.1445	0.19468	0.086527	0.99994	0.71811	-0.47773	0.94032	0.99996	1.0113	0.016145	0.23299	0.69081	0.83997	-0.25159	3.29E-07	0.000144	0.21424	-2.2227	0.007103	0.081828	0.56762	-0.81701	0.18752	0.64793	0.78118	-0.35627	0.44655	0.96776	0.95135	-0.07195	0.007139	0.091944	1.4518	0.53788
286.2016	2.85	olomics Po	0.11295	0.99951	1.4983	0.58336	0.10365	0.99998	1.4692	0.55507	0.045483	0.99941	1.7925	0.84195	0.007271	0.1948	1.7498	0.80722	0.061928	0.19645	1.8647	0.89896	0.45606	0.7581	1.8242	0.86723	0.66276	0.99391	1.9353	0.95258	0.65633	0.99956	1.0396	0.056071	0.19393	0.45848	0.63535	-0.65438
255.2514	1.47	domics Pos	0.11361	0.99546	1.2721	0.34721	0.34459	0.99994	0.85824	-0.22055	0.62706	0.99996	1.0599	0.083912	0.15814	0.58769	0.7751	-0.36755	1.77E-05	0.000338	0.198	-2.3364	0.010185	0.08787	0.5748	-0.79887	0.3168	0.72948	0.83137	-0.26643	0.56084	0.97028	0.91141	-0.13383	0.034513	0.12629	1.3817	0.46647
85.1019	6.38	olomics Po	0.11366	0.99951	1.1934	0.25505	0.78245	0.99998	0.97493	-0.03663	0.40139	0.99941	1.0797	0.11065	0.27524	0.74848	0.85859	-0.21995	0.000363	0.004495	0.37331	-1.4216	0.003954	0.053954	0.65874	-0.60222	0.15148	0.72698	0.81984	-0.28658	0.62036	0.99956	0.95599	-0.06494	0.12224	0.37203	1.2669	0.34128
729.3785	0.54	domics Pos	0.11422	0.99546	1.663	0.73376	0.36757	0.99994	0.90917	-0.13738	0.93554	0.99996	0.99301	-0.01012	0.016302	0.24	0.58885	-0.76402	0.000281	0.002099	0.43199	-1.2109	0.006523	0.079718	0.6326	-0.66064	0.45764	0.80401	0.89174	-0.1653	0.66198	0.97988	0.9744	-0.03742	0.005797	0.08891	2.1639	1.1136
631.5245	2.05	domics Pos	0.11501	0.99546	1.3053	0.38441	0.22864	0.99994	0.82774	-0.27274	0.65305	0.99996	1.0611	0.085563	0.23167	0.68957	0.78701	-0.34555	6.06E-05	0.000736	0.13222	-2.919	0.029232	0.15124	0.58324	-0.77785	0.25774	0.69174	0.79984	-0.32221	0.46863	0.96776	1.0848	0.11745	0.003873	0.085302	1.602	0.6799
151.1479	6	olomics Po	0.11551	0.99951	1.1814	0.24053	0.27896	0.99998	0.89676	-0.1572	0.98111	0.99941	0.9985	-0.00216	0.062931	0.32975	0.75145	-0.41225	1.29E-06	0.000231	0.26054	-1.9404	0.005656	0.064549	0.61499	-0.70135	0.12279	0.65834	0.77439	-0.36886	0.27583	0.99956	0.87726	-0.18892	0.33243	0.60917	1.095	0.13092
1097.213	4.43	domics Pos	0.11569	0.99546	1.5545	0.63643	0.66805	0.99994	1.0371	0.052544	0.28174	0.99996	1.1379	0.18632	0.10621	0.53006	0.72527	-0.46341	0.003029	0.012287	0.55276	-0.85528	0.058757	0.24091	0.71238	-0.48927	0.92482	0.98262	1.0072	0.010308	0.81347	0.97988	1.0016	0.002364	0.015247	0.09885	1.7598	0.81542
871.1176	9.21	olomics Po	0.116	0.99951	1.5354	0.61859	0.95684	0.99998	1.0201	0.028734	0.31195	0.99941	1.1552	0.20813	0.41122	0.90267	0.86368	-0.21144	0.034357	0.12747	0.62137	-0.68649	0.060842	0.21247	0.73797	-0.43836	0.47106	0.99391	1.2053	0.26944	0.99731	0.99956	1.0438	0.061907	0.078642	0.32467	1.79	0.83994
95.0862	1.66	domics Pos	0.11611	0.99546	1.2318	0.30082	0.29805	0.99994	0.85302	-0.22934	0.80447	0.99996	1.0372	0.052733	0.12204	0.54337	0.72032	-0.47328	9.61E-06	0.000218	0.16817	-2.572	0.012195	0.093	0.56894	-0.81365	0.23369	0.6761	0.77762	-0.36286	0.79632	0.97988	0.96195	-0.05596	0.016734	0.099614	1.405	0.49055
765.9228	3.25	olomics Po	0.11624	0.99951	1.5175	0.60169	0.16209	0.99998	0.82924	-0.27014	0.71955	0.99941	1.027	0.03841	0.045438	0.2866	0.70219	-0.51007	0.000182	0.002591	0.49658	-1.0099	0.012858	0.085844	0.64595	-0.63051	0.86455	0.9951	1.0675	0.094258	0.76657	0.99956	1.002	0.002857	0.051725	0.2951	1.7774	0.82974
797.1982	6.13	domics Pos	0.11637	0.99546	1.5203	0.60438	0.73424	0.99994	0.94194	-0.08629	0.9601	0.99996	0.982	-0.02621	0.06019	0.42344	0.6374	-0.64972	0.005156	0.018628	0.51616	-0.95411	0.086437	0.30802	0.65837	-0.60302	0.98401	0.99431	1.0312	0.044327	0.69403	0.97988	1.104	0.14278	0.11919	0.22343	1.788	0.83836
189.1643	1.66	domics Pos	0.11659	0.99546	1.2341	0.30351	0.53971	0.99994	0.90605	-0.14234	0.89553	0.99996	1.0163	0.023293	0.11667	0.53727	0.73123	-0.4516	2.15E-05	0.00038	0.17841	-2.4867	0.018563	0.11661	0.54746	-0.86918	0.24719	0.68479	0.79446	-0.33195	0.59823	0.97073	0.9169	-0.12517	0.024791	0.1117	1.3972	0.48249
173.149	2.05	domics Pos	0.11684	0.99546	1.1745	0.232	0.14905	0.99994	0.8387	-0.25378	0.91624	0.99996	1.0022	0.00311	0.15651	0.58507	0.84263	-0.24704	2.05E-05	0.000373	0.25969	-1.9451	0.019418	0.11985	0.61903	-0.69192	0.54979	0.85184	0.92544	-0.11179	0.56027	0.96972	1.0516	0.072642	0.045555	0.14174	1.2564	0.32927
1137.8	5.41	domics Pos	0.11699	0.99546	1.5552	0.63711	0.87431	0.99994	0.98758	-0.01803	0.28712	0.99996	1.1228	0.16705	0.029556	0.30146	0.66246	-0.5941	0.001525	0.007407	0.54415	-0.87792	0.054706	0.231	0.68476	-0.54632	0.8368	0.96659	1.073	0.10159	0.93249	0.98602	1.1304	0.17685	0.032627	0.1233	2.1072	1.0753
985.1691	9.43	olomics Po	0.11723	0.99951	1.4466	0.53266	0.32223	0.99998	0.90723	-0.14045	0.18102	0.99941	1.1568	0.21008	0.015526	0.20428	0.71074	-0.49261	0.006575	0.035557	0.70326	-0.50786	0.27057	0.56295	0.91854	-0.12259	0.77332	0.99391	0.99506	-0.00715	0.93812	0.99956	1.0422	0.059581	0.061246	0.30404	1.6911	0.75795
216.9234	10.1	domics Pos	0.11728	0.99546	1.0445	0.062812	0.19717	0.99994	0.94444	-0.08247	0.10662	0.99996	0.94704	-0.07851	0.11127	0.53238	0.93367	-0.09901	8.23E-05	0.000907	0.78311	-0.35271	0.27127	0.55798	0.91385	-0.12998	0.53322	0.84189	1.0284	0.040452	0.86076	0.98023	0.99397	-0.00872	0.005234	0.088007	1.2035	0.26725
444.3407	1.64	domics Pos	0.11742	0.99546	1.217	0.28332	0.31409	0.99994	0.83616	-0.25815	0.81931	0.99996	1.0243	0.034614	0.9653	0.99452	0.99913	-0.00125	0.00716	0.02376	0.58948	-0.76248	0.37112	0.62976	0.86845	-0.20349	0.99803	0.99882	0.99008	-0.01439	0.87498	0.98023	1.0241	0.0344	0.04765	0.14454	1.355	0.43829
194.6546	6.38	olomics Po	0.11771	0.99951	1.1983	0.26094	0.39789	0.99998	1.0951	0.13111	0.65323	0.99941	1.1001	0.13763	0.064853	0.33326	1.2269	0.29497	0.006836	0.036638	0.55111	-0.8596	0.75935	0.93212	1.027	0.038457	0.2476	0.85612	1.1263	0.17163	0.22893	0.99956	1.1344	0.1819	0.012154	0.2688	1.3218	0.40251
400.3111	1.67	domics Pos	0.11815	0.99546	1.1747	0.23234	0.58142	0.99994	0.93391	-0.09864	0.72877	0.99996	1.0317	0.044976	0.43964	0.86677	0.90153	-0.14956	0.000732	0.00439	0.57253	-0.80458	0.004638	0.071311	0.64492	-0.63281	0.27005	0.70383	0.81611	-0.29317	0.69837	0.97988	0.95496	-0.06649	0.27682	0.38543	1.2442	0.31527
299.2403	6	olomics Po	0.11851	0.99951	1.2616	0.33524	0.1827	0.99998	0.80548	-0.31209	0.58329	0.99941	1.1173	0.16008	0.11803	0.47062	0.73987	-0.43466	3.63E-06	0.000231	0.21981	-2.1857	0.032056	0.1379	0.52287	-0.93549	0.17191	0.74875	0.77241	-0.37257	0.59251	0.99956	0.93156	-0.10227	0.12566	0.3747	1.2257	0.29358
161.1336	4.73	domics Pos	0.11875	0.99546	1.0752	0.10461	0.16451	0.99994	0.9506	-0.07308	0.72962	0.99996	0.98286	-0.02495	0.65226	0.95465	0.97271	-0.03991	0.000813	0.004784	0.79247	-0.33557	0.79737	0.89629	0.98703	-0.01883	0.64956	0.89057	0.97742	-0.03294	0.55627	0.96798	0.96946	-0.04475	0.038944	0.13241	1.248	0.3196
545.2864	0.56	domics Pos	0.11881	0.99546	1.375	0.45942	0.10491	0.99994	0.86913	-0.20235	0.73155	0.99996	0.97843	-0.03146	0.012606	0.22648	0.53291	-0.90804	3.29E-05	0.000489	0.41952	-1.2532	0.000476	0.033979	0.50697	-0.98003	0.28938	0.71484	0.8454	-0.2423	0.40223	0.96776	0.88188	-0.18135	0.028701	0.11732	1.9345	0.95198
961.5182	0.57	domics Pos	0.11901	0.99546	1.485	0.57043	0.097215	0.99994	0.83168	-0.26589	0.41601	0.99996	1.0809	0.11229	0.013175	0.22835	0.62011	-0.68941	0.000109	0.0011	0.43006	-1.2174	0.001883	0.057238	0.6237	-0.68107	0.94002	0.98682	1.0471	0.066457	0.77771	0.97988	1.2072	0.27171	0.022563	0.10932	2.6858	1.4253
418.9771	12	olomics Ne	0.11927	0.99918	1.0995	0.13679	0.737	0.95512	0.97848	-0.03139	0.83838	0.99995	1.0183	0.026138	0.7366	0.99985	1.0535	0.075175	0.011868	0.76891	1.1647	0.22	0.24251	0.68153	1.1482	0.19934	0.57395	0.85384	1.0743	0.10336	0.95615	0.99512	1.0002	0.000322	0.034099	0.99941	1.1629	0.21777
299.2377	1.66	domics Pos	0.11956	0.99546	1.3916	0.47673	0.28736	0.99994	0.76155	-0.393	0.51339	0.99996	1.1734	0.23071	0.1834	0.62384	0.63868	-0.64684	1.88E-05	0.000351	0.095478	-3.3887	0.017457	0.11287	0.36897	-1.4384	0.11273	0.56484	0.60881	-0.71594	0.73787	0.97988	0.90477	-0.14437	0.05543	0.15256	1.5379	0.62099
974.8129	10.02	domics Pos	0.11959	0.99546	1.0528	0.074296	0.65117	0.99994	0.98144	-0.02703	0.71538	0.99996	0.98657	-0.0195	0.2054	0.65222	0.94312	-0.08449	0.000246	0.001942	0.77506	-0.36761	0.60811	0.78723	0.96405	-0.05282	0.34056	0.74459	1.0654	0.091336	0.55228	0.96776	1.0218	0.031154	0.005693	0.088649	1.2241	0.2917
213.2031	2.05	domics Pos	0.11975	0.99546	1.2185	0.28515	0.21989	0.99994	0.84852	-0.23698	0.65746	0.99996	1.0407	0.057546	0.11354	0.53238	0.7256	-0.46276	3.15E-05	0.000482	0.22405	-2.1581	0.007523	0.082061	0.55515	-0.84905	0.20112	0.64891	0.82274	-0.2815	0.75842	0.97988	0.95858	-0.06103	0.011033	0.094875	1.392	0.47719
963.5319	0.56	domics Pos	0.12021	0.99564	1.2324	0.3015	0.10726	0.99994	0.79329	-0.33408	0.50793	0.99996	0.94343	-0.08401	0.011781	0.21807	0.56984	-0.81138	0.000247	0.001942	0.38646	-1.3716	0.000677	0.03855	0.52292	-0.93534	0.33709	0.74254	0.8789	-0.18623	0.4563	0.96776	0.89892	-0.15373	0.038707	0.13216	2.0264	1.0189
654.3391	2.68	olomics Po	0.12038	0.99951	1.4922	0.57741	0.60438	0.99998	0.91002	-0.13604	0.84769	0.99941	0.96094	-0.05748	0.085451	0.38954	0.69065	-0.53398	0.003145	0.021166	0.48451	-1.0454	0.054177	0.19706	0.67885	-0.55884	0.86308	0.9951	0.99497	-0.00728	0.87725	0.99956	1.1619	0.21647	0.031428	0.2688	2.1077	1.0757
741.9297	3.22	olomics Po	0.12067	0.99951	1.3633	0.44707	0.099237	0.99998	0.8812	-0.18246	0.84504	0.99941	0.99503	-0.00719	0.040516	0.27667	0.70583	-0.50261	0.002994	0.020592	0.6778	-0.56107	0.094949	0.28786	0.8388	-0.2536	0.66557	0.99391	0.97541	-0.03592	0.87606	0.99956	1.0064	0.009205	0.015974	0.2688	1.7856	0.83643
609.3492	3.05	olomics Po	0.12087	0.99951	1.3152	0.39529	0.028447	0.99998	0.8306	-0.26777	0.96042	0.99941	1.0113	0.016267	0.018129	0.20936	0.66399	-0.59076	0.000414	0.004925	0.56782	-0.8165	0.000766	0.041543	0.57568	-0.79665	0.19392	0.7752	0.83342	-0.26289	0.31389	0.99956	0.83858	-0.25398	0.020606	0.2688	1.7369	0.79647
804.8419	10.02	domics Pos	0.12133	0.99564	1.132	0.17892	0.079339	0.99994	1.118	0.16097	0.44416	0.99996	1.0741	0.10316	0.93466	0.98937	1.0057	0.008157	0.020922	0.054231	0.79579	-0.32955	0.37629	0.63347	0.93861	-0.0914	0.37358	0.76056	1.1354	0.1832	0.84495	0.97988	1.0284	0.040366	0.00163	0.07528	1.2624	0.33615
298.3106	7.46	olomics Po	0.12164	0.99951	0.87974	-0.18485	0.02536	0.99998	0.80522	-0.31255	0.10442	0.99941	0.84829	-0.23738	0.11123	0.4527	0.90182	-0.1491	0.27809	0.63672	0.91592	-0.12671	0.003905	0.053802	0.68823	-0.53904	0.050782	0.45418	0.71601	-0.48196	0.005762	0.47452	0.74251	-0.42951	0.005304	0.2688	0.78891	-0.34207
830.4431	0.58	domics Pos	0.12168	0.99564	1.4706	0.55643	0.13763	0.99994	0.8847	-0.17674	0.97672	0.99996	1.0041	0.005924	0.003301	0.17581	0.5664	-0.82009	0.000246	0.001942	0.44274	-1.1755	0.000262	0.026314	0.52432	-0.93148	0.58161	0.86163	0.94	-0.08926	0.79549	0.97988	1.0369	0.052284	0.01648	0.099614	2.3558	1.2362
567.8785	0.5	domics Pos	0.12173	0.99564	1.065	0.090802	0.14274	0.99994	0.91661	-0.12561	0.16488	0.99996	0.93231	-0.10111	0.000376	0.164	0.80636	-0.3105	2.13E-05	0.000378	0.66484	-0.58893	0.084351	0.30314	0.79474	-0.33144	0.31331	0.72948	0.90787	-0.13945	0.13849	0.89982	0.86038	-0.21695	0.040875	0.13494	1.2119	0.27723
701.3926	0.58	domics Pos	0.12175	0.99564	1.4542	0.54024	0.13435	0.99994	0.86866	-0.20313	0.97993	0.99996	1.0069	0.009943	0.012762	0.22648	0.59586	-0.74697	0.00012	0.001169	0.41231	-1.2782	7.59E-05	0.019289	0.55487	-0.84978	0.66662	0.8978	0.97581	-0.03533	0.8538	0.97988	1.0611	0.085506	0.014943	0.09885	2.4752	1.3076
878.7395	0.54	domics Pos	0.1218	0.99564	1.1868	0.24713	0.75229	0.99994	0.97257	-0.04012	0.51777	0.99996	0.94037	-0.0887	0.25074	0.70866	0.90361	-0.14623	0.009944	0.030554	0.71886	-0.47621	0.10274	0.34081	0.82312	-0.28083	0.2937	0.7186	0.92163	-0.11775	0.63423	0.97988	0.94931	-0.07505	0.15412	0.25979	1.2296	0.29817
678.376	3.14	olomics Po	0.12194	0.99951	1.4146	0.50043	0.86947	0.99998	0.98412	-0.0231	0.80667	0.99941	0.97287	-0.03968	0.017541	0.20936	0.63061	-0.66518	0.001905	0.015005	0.59059	-0.75977	0.008314	0.075694	0.59092	-0.75896	0.90686	0.9951	1.0514	0.072349	0.69779	0.99956	0.96684	-0.04865	0.019329	0.2688	1.8806	0.91117
450.8789	6.37	olomics Po	0.12207	0.99951	1.3766	0.46112	0.69789	0.99998	0.9158	-0.12689	0.78729	0.99941	1.0679	0.094795	0.36656	0.86198	0.79031	-0.33952	4.96E-05	0.000929	0.10403	-3.265	0.053607	0.19635	0.49224	-1.0226	0.32641	0.9393	0.78306	-0.35281	0.85144	0.99956	0.95142	-0.07185	0.11086	0.36452	1.3105	0.39009
986.6422	9.43	olomics Po	0.12227	0.99951	1.3347	0.41652	0.24105	0.99998	0.87408	-0.19416	0.27178	0.99941	1.1667	0.22238	0.0192	0.21472	0.72307	-0.46779	0.005622	0.032022	0.57237	-0.80498	0.032394	0.13902	0.67535	-0.5663	0.58044	0.99391	0.93539	-0.09637	0.52922	0.99956	0.92438	-0.11343	0.10924	0.36376	1.5343	0.61761
398.2461	3.52	domics Pos	0.12235	0.99564	0.87554	-0.19175	0.066957	0.99994	0.86971	-0.2014	0.000593	0.75409	0.67035	-0.57701	0.000323	0.164	0.58307	-0.77827	0.000475	0.003154	0.4498	-1.1526	0.00573	0.076133	0.4758	-1.0716	0.004987	0.3088	0.56652	-0.8198	0.001149	0.41831	0.50625	-0.98207	0.11682	0.22117	0.78865	-0.34255
199.1888	1.66	domics Pos	0.12246	0.99564	1.2425	0.31329	0.33769	0.99994	0.85061	-0.23343	0.34876	0.99996	1.1291	0.17523	0.23883	0.69783	0.77286	-0.37172	6.18E-06	0.000171	0.16078	-2.6369	0.008432	0.084385	0.52641	-0.92575	0.51016	0.83279	0.88091	-0.18293	0.9308	0.98602	0.97872	-0.03103	0.040532	0.13427	1.4112	0.49688
591.3598	2.72	olomics Po	0.12269	0.99951	1.4833	0.56883	0.47322	0.99998	0.89395	-0.16174	0.43326	0.99941	0.88533	-0.17572	0.022478	0.22443	0.57465	-0.79925	0.005788	0.032557	0.57051	-0.80967	0.001586	0.045425	0.50066	-0.99811	0.80187	0.99391	1.0008	0.001172	0.72799	0.99956	0.9746	-0.03713	0.029304	0.2688	2.2802	1.1892
528.7735	0.56	domics Pos	0.12291	0.99714	1.2106	0.27566	0.17991	0.99994	1.2171	0.28341	0.58436	0.99996	1.0448	0.063161	0.018861	0.25593	0.73981	-0.43477	0.22037	0.31459	0.8313	-0.26656	0.076709	0.28715	0.83499	-0.26018	0.55651	0.85357	0.9235	-0.11482	0.8134	0.97988	0.9786	-0.03122	0.19487	0.30304	1.1649	0.22024
271.2704	6.81	olomics Po	0.12299	0.99951	0.85652	-0.22344	0.85852	0.99998	0.99924	-0.00109	0.082065	0.99941	0.74481	-0.42506	0.079045	0.36897	0.72726	-0.45946	0.018284	0.07836	0.7999	-0.3221	0.007262	0.070826	0.63834	-0.6476	0.022225	0.31703	0.66831	-0.58142	0.005928	0.47452	0.67609	-0.56472	0.04856	0.28861	0.69646	-0.52189
213.2047	6.38	olomics Po	0.12318	0.99951	1.261	0.33451	0.65505	0.99998	0.94355	-0.08382	0.75887	0.99941	1.06	0.084076	0.24647	0.71226	0.81782	-0.29015	5.85E-05	0.001066	0.28704	-1.8007	0.026625	0.12262	0.61161	-0.70931	0.22909	0.83662	0.81525	-0.29469	0.71185	0.99956	0.95423	-0.06759	0.1048	0.35957	1.2362	0.30592
107.0857	2.05	domics Pos	0.12352	0.99987	1.1838	0.24342	0.5415	0.99994	0.92579	-0.11125	0.64556	0.99996	1.0462	0.065169	0.26281	0.72224	0.83095	-0.26717	8.11E-06	0.000199	0.19429	-2.3637	0.01045	0.08835	0.57472	-0.79908	0.19328	0.64891	0.81805	-0.28975	0.42785	0.96776	0.91376	-0.13011	0.008568	0.092495	1.3977	0.48304
833.4695	0.57	domics Pos	0.12378	0.99987	1.3639	0.44777	0.041557	0.99994	0.87792	-0.18784	0.70978	0.99996	0.9778	-0.0324	0.003607	0.17581	0.56655	-0.81972	0.000428	0.00291	0.40347	-1.3095	9.89E-05	0.023299	0.58615	-0.77065	0.52643	0.83957	0.92843	-0.10713	0.74635	0.97988	1.0185	0.026501	0.017261	0.10048	2.4084	1.2681
1029.625	1.3	domics Pos	0.12445	0.99987	1.2747	0.3502	0.67318	0.99994	1.1072	0.14697	0.55847	0.99996	1.1581	0.21181	0.92359	0.9874	1.0213	0.030343	0.15546	0.24372	0.67604	-0.56483	0.38011	0.63792	1.1931	0.25469	0.037549	0.37121	1.7416	0.80042	0.079075	0.79555	1.3837	0.46849	0.006682	0.090221	2.449	1.2922
428.2311	0.58	domics Pos	0.12452	0.99987	1.5272	0.61087	0.017817	0.99994	0.76096	-0.3941	0.39957	0.99996	0.93311	-0.09988	0.000914	0.17581	0.56487	-0.82401	0.001829	0.008483	0.4254	-1.2331	0.004457	0.071311	0.57847	-0.78969	0.789	0.95091	1.035	0.049563	0.71612	0.97988	1.0179	0.025666	0.040231	0.13378	2.6089	1.3834
624.938	9.58	olomics Po	0.12457	0.99951	1.4598	0.54577	0.55265	0.99998	0.93303	-0.10001	0.21666	0.99941	1.1217	0.16569	0.010612	0.19754	0.70146	-0.51158	0.008085	0.04121	0.6139	-0.70393	0.043347	0.16962	0.63546	-0.65412	0.92147	0.9951	1.0337	0.047857	0.9337	0.99956	1.1002	0.13778	0.070936	0.31396	1.7464	0.80442
469.4038	9.75	domics Pos	0.12492	0.99987	1.2336	0.30286	0.6504	0.99994	0.96069	-0.05786	0.70469	0.99996	1.0427	0.060295	0.76347	0.97526	0.97899	-0.03063	0.049766	0.10392	0.82352	-0.28012	0.21922	0.50937	0.86546	-0.20845	0.87141	0.97532	0.99084	-0.01327	0.72491	0.97988	1.0411	0.058138	0.17029	0.27795	1.1821	0.24136
382.4046	5.07	domics Pos	0.12493	0.99987	0.89789	-0.1554	0.18815	0.99994	0.81597	-0.29341	0.005021	0.99996	0.67469	-0.5677	0.002494	0.17581	0.72447	-0.46501	0.002441	0.010471	0.73366	-0.44682	0.000172	0.024979	0.54814	-0.86739	0.024776	0.33809	0.70574	-0.50278	0.00012	0.37028	0.61453	-0.70245	0.82448	0.87121	0.9863	-0.0199
562.9745	12.01	olomics Ne	0.12512	0.99918	1.1823	0.24164	0.95787	0.99363	0.99593	-0.00589	0.97675	0.99995	0.9952	-0.00695	0.45001	0.99985	1.0912	0.12587	0.038087	0.99927	1.2915	0.36907	0.29822	0.71853	1.1358	0.18367	0.15107	0.53641	1.268	0.34257	0.31615	0.99512	1.0914	0.12622	0.60085	0.99941	1.0384	0.054361
196.1708	5.99	olomics Po	0.12535	0.99951	1.199	0.26181	0.75943	0.99998	0.97151	-0.04171	0.84949	0.99941	1.02	0.028616	0.15084	0.5457	0.77022	-0.37666	2E-05	0.00048	0.22293	-2.1653	0.010195	0.080256	0.60316	-0.72939	0.15943	0.73694	0.79884	-0.32402	0.29856	0.99956	0.88492	-0.17638	0.063604	0.30416	1.202	0.2655
123.0825	0.84	domics Pos	0.12576	0.99987	0.8665	-0.20674	0.067031	0.99994	0.74417	-0.4263	0.3841	0.99996	0.89797	-0.15526	0.14681	0.57298	0.87921	-0.18573	0.012516	0.036354	0.64562	-0.63124	0.022539	0.13179	0.72885	-0.4563	0.18775	0.64793	0.79594	-0.32928	0.16961	0.91806	0.87746	-0.18859	0.52439	0.62357	0.94464	-0.08216
983.6592	4.6	domics Pos	0.12587	0.99987	1.3519	0.43494	0.23947	0.99994	0.86782	-0.20453	0.49416	0.99996	1.1161	0.15848	0.003502	0.17581	0.56755	-0.81719	0.003238	0.013011	0.58183	-0.78132	0.009575	0.087345	0.6214	-0.68641	0.56164	0.855	0.93244	-0.10092	0.81087	0.97988	1.0165	0.023583	0.11872	0.22318	1.9301	0.9487
166.1659	1.66	domics Pos	0.12591	0.99987	1.2969	0.3751	0.15618	0.99994	0.86566	-0.20812	0.91629	0.99996	0.98665	-0.0194	0.10667	0.53093	0.74596	-0.42283	2.71E-06	0.000144	0.20367	-2.2957	0.005163	0.074042	0.55399	-0.85207	0.30094	0.72409	0.81754	-0.29064	0.099468	0.85039	0.86175	-0.21466	0.020038	0.10652	1.4148	0.50061
692.9727	12	olomics Ne	0.12591	0.99918	1.1609	0.21529	0.30951	0.73839	0.93773	-0.09275	0.56899	0.99995	1.1102	0.15085	0.33513	0.99985	1.1922	0.25362	0.10342	0.99927	1.1359	0.18388	0.038464	0.48405	1.1765	0.23445	0.42972	0.76794	0.95424	-0.06757	0.84148	0.99512	1.0616	0.086181	0.26847	0.99941	0.92634	-0.11038
574.992	3.34	olomics Po	0.12596	0.99951	1.3806	0.46525	0.32615	0.99998	0.88841	-0.17071	0.96625	0.99941	1.0114	0.016343	0.027878	0.24036	0.6597	-0.60012	0.001272	0.011249	0.53277	-0.90843	0.001923	0.045425	0.60827	-0.71723	0.49629	0.99391	0.89233	-0.16435	0.82237	0.99956	0.98014	-0.02894	0.045535	0.28476	1.722	0.78407
589.6681	3.37	olomics Po	0.12646	0.99951	1.3301	0.41154	0.57318	0.99998	0.94155	-0.08689	0.47478	0.99941	1.0503	0.070738	0.0114	0.19869	0.60076	-0.73514	0.024806	0.099287	0.58415	-0.77558	0.002254	0.045856	0.55868	-0.83991	0.68482	0.99391	0.98135	-0.02717	0.42692	0.99956	0.88813	-0.17116	0.022607	0.2688	1.9105	0.93398
207.2098	2.05	domics Pos	0.12652	0.99987	1.28	0.35609	0.55385	0.99994	0.89777	-0.15558	0.61399	0.99996	1.0673	0.094014	0.20351	0.64992	0.76118	-0.3937	0.000326	0.002357	0.2554	-1.9692	0.074254	0.28088	0.6979	-0.51891	0.71689	0.92042	0.94936	-0.07498	0.60995	0.97371	1.063	0.08818	0.006466	0.090084	2.0557	1.0396
404.7176	2.87	olomics Po	0.12653	0.99951	1.4733	0.55908	0.39733	0.99998	0.90416	-0.14535	0.60091	0.99941	1.0746	0.10385	0.18234	0.60975	0.77688	-0.36424	0.011482	0.054208	0.58146	-0.78225	0.063039	0.21742	0.7643	-0.38779	0.91359	0.9951	1.1173	0.16002	0.95391	0.99956	1.1079	0.14779	0.033659	0.2688	2.1864	1.1286
373.2117	2.89	olomics Po	0.12658	0.99951	1.4407	0.52676	0.21574	0.99998	0.85769	-0.22148	0.2274	0.99941	1.1011	0.13888	0.001602	0.14411	0.62463	-0.67892	0.00698	0.037008	0.58641	-0.77003	0.053068	0.19483	0.71657	-0.48082	0.76713	0.99391	0.99816	-0.00265	0.75733	0.99956	1.0101	0.014521	0.026628	0.2688	2.0105	1.0075
139.113	6.38	olomics Po	0.12668	0.99951	1.1198	0.16324	0.3069	0.99998	0.90251	-0.14799	0.74338	0.99941	0.97768	-0.03257	0.095719	0.41474	0.77098	-0.37523	3.24E-06	0.000231	0.25683	-1.9611	0.00187	0.045425	0.55704	-0.84416	0.023685	0.32255	0.71979	-0.47436	0.13367	0.91719	0.84448	-0.24386	0.98741	0.9934	1.0027	0.003928
995.7967	10.02	domics Pos	0.1271	0.99987	0.86042	-0.21689	0.11897	0.99994	0.83904	-0.25319	0.23137	0.99996	0.86164	-0.21484	0.46193	0.87979	0.9233	-0.11513	0.003997	0.015288	0.67451	-0.56809	0.15771	0.43895	0.82004	-0.28623	0.88237	0.97532	0.98592	-0.02045	0.59404	0.97073	0.94473	-0.08202	0.4182	0.52558	1.0914	0.12616
737.5803	2.05	domics Pos	0.12746	0.99987	1.4095	0.49514	0.892	0.99994	1.013	0.018656	0.17829	0.99996	1.1846	0.24437	0.28781	0.75229	0.80144	-0.31934	6.65E-05	0.000785	0.10927	-3.194	0.00622	0.078151	0.48622	-1.0403	0.55079	0.85184	0.90772	-0.13969	0.72201	0.97988	1.1561	0.2093	0.004972	0.087539	1.6372	0.71123
221.1542	5.04	olomics Po	0.12775	0.99951	0.90617	-0.14215	0.067184	0.99998	0.83434	-0.26129	0.46668	0.99941	0.92847	-0.10707	0.8868	0.99905	0.98925	-0.01559	0.24063	0.5682	0.88729	-0.17252	0.018925	0.10165	0.7053	-0.50369	0.12952	0.6766	0.79829	-0.32502	0.11823	0.89567	0.87882	-0.18635	0.07378	0.31793	0.87832	-0.18718
394.9779	4.47	olomics Ne	0.1281	0.99918	1.049	0.069041	0.01914	0.39934	0.90778	-0.13958	0.84177	0.99995	0.99378	-0.009	0.89485	0.99985	1.0065	0.009357	0.93659	0.99927	1.0074	0.010653	0.20655	0.65531	1.0711	0.09914	0.39736	0.74225	1.0477	0.067185	0.73039	0.99512	1.021	0.030006	0.44039	0.99941	0.97877	-0.03097
883.5349	10.48	olomics Ne	0.12821	0.99918	1.2753	0.35083	0.014495	0.37315	0.75914	-0.39756	0.051459	0.99995	1.2126	0.27814	0.32039	0.99985	1.1001	0.13764	0.82179	0.99927	0.97818	-0.03183	0.023122	0.43126	1.2809	0.35715	0.31964	0.69152	1.132	0.17893	0.075099	0.99512	1.2231	0.29057	0.84631	0.99941	1.0316	0.04492
1030.669	9.39	olomics Po	0.12842	0.99951	1.377	0.46148	0.59652	0.99998	0.94146	-0.08703	0.32127	0.99941	1.123	0.16732	0.060614	0.32444	0.73312	-0.44789	0.004862	0.028888	0.64476	-0.63317	0.06011	0.2118	0.69861	-0.51744	0.62131	0.99391	0.94789	-0.07721	0.64992	0.99956	0.96186	-0.0561	0.095056	0.34969	1.6844	0.75223
207.1749	1.66	domics Pos	0.12858	0.99987	1.2315	0.30043	0.33795	0.99994	0.86124	-0.21551	0.58066	0.99996	1.0698	0.097369	0.12438	0.54705	0.71636	-0.48124	8.96E-05	0.000962	0.1697	-2.559	0.005353	0.074454	0.51711	-0.95147	0.28239	0.7098	0.83791	-0.25513	0.56406	0.9703	0.91601	-0.12657	0.024251	0.11101	1.3937	0.47892
382.1893	0.58	domics Pos	0.12883	0.99987	1.461	0.54694	0.03656	0.99994	0.76272	-0.39077	0.79457	0.99996	0.9676	-0.04752	0.017985	0.25028	0.5908	-0.75925	0.001034	0.0056	0.40675	-1.2978	0.001248	0.044487	0.54372	-0.87906	0.66267	0.89623	0.97382	-0.03827	0.73358	0.97988	1.0509	0.071618	0.03428	0.12617	2.6896	1.4274
869.6078	10.25	olomics Ne	0.1289	0.99918	1.0744	0.1035	0.12871	0.54655	0.91369	-0.13022	0.51951	0.99995	1.0436	0.061531	0.92278	0.99985	1.0189	0.026976	0.80802	0.99927	1.0199	0.028478	0.049065	0.48405	1.1546	0.20736	0.90395	0.97375	0.99355	-0.00934	0.53238	0.99512	0.96879	-0.04574	0.148	0.99941	0.91761	-0.12404
168.99	9.88	domics Pos	0.12914	0.99987	1.1963	0.25862	0.5733	0.99994	1.0711	0.099135	0.34426	0.99996	1.0791	0.10989	0.10996	0.53208	0.88446	-0.17713	0.020037	0.052436	0.77872	-0.36083	0.79625	0.89629	0.97845	-0.03143	0.54356	0.84873	1.071	0.098895	0.40412	0.96776	1.0672	0.093784	0.016264	0.099614	1.2491	0.32088
589.3232	3.03	olomics Po	0.12945	0.99951	1.4511	0.53713	0.31301	0.99998	0.86358	-0.21159	0.77736	0.99941	1.0292	0.041558	0.021269	0.22258	0.64593	-0.63056	0.001709	0.013731	0.53186	-0.91087	0.009079	0.076393	0.65848	-0.6028	0.65746	0.99391	0.96197	-0.05594	0.85956	0.99956	0.99634	-0.00529	0.026004	0.2688	2.04	1.0286

530.9668	3.28	olomics Pc	0.12966	0.99951	1.3935	0.4787	0.10769	0.99998	0.82331	-0.2805	0.78093	0.99941	0.96889	-0.0456	0.015748	0.20428	0.71773	-0.47849	0.005784	0.032557	0.61258	-0.70704	0.002132	0.045856	0.63228	-0.66137	0.24757	0.85612	0.84062	-0.25048	0.95811	0.99956	1.015	0.021542	0.027224	0.2688	1.8045	0.85161
167.1444	5.99	olomics Pc	0.13005	0.99951	1.1558	0.20883	0.65124	0.99998	0.95621	-0.0646	0.87522	0.99941	0.99069	-0.01349	0.055407	0.30728	0.71066	-0.49277	2.68E-06	0.000231	0.22718	-2.1381	0.002911	0.048778	0.59078	-0.75932	0.064881	0.51696	0.7605	-0.39497	0.40389	0.99956	0.92432	-0.11353	0.10977	0.36416	1.1645	0.21969
297.2954	6.74	olomics Pc	0.13029	0.99951	1.2273	0.29548	0.19207	0.99998	0.78599	-0.34741	0.85938	0.99941	0.96489	-0.05156	0.5123	0.98198	0.89148	-0.16573	0.000289	0.003742	0.35853	-1.4798	0.006845	0.06889	0.56723	-0.81798	0.23116	0.83662	0.78958	-0.34083	0.94797	0.99956	0.99271	-0.01055	0.099043	0.35294	1.2638	0.33773
650.8481	10.09	lomics Pos	0.13037	0.99987	1.0887	0.12263	0.16601	0.99994	0.93609	-0.09528	0.71288	0.99996	0.9784	-0.03151	0.25282	0.71031	0.90991	-0.1362	0.010814	0.032597	0.81309	-0.29851	0.35698	0.62123	0.91266	-0.13185	0.23296	0.6761	1.0832	0.11524	0.47368	0.96776	1.0545	0.076568	0.15612	0.26236	1.1708	0.2275
452.9913	5.07	olomics Ne	0.13067	0.99918	1.038	0.053762	0.016996	0.37363	0.90728	-0.14039	0.51735	0.99995	1.0152	0.021772	0.83035	0.99985	1.0135	0.019309	0.7461	0.99927	1.0197	0.028081	0.11294	0.58058	1.1181	0.16111	0.020575	0.30247	1.1116	0.15267	0.88433	0.99512	0.99507	-0.00713	0.1723	0.99941	1.0434	0.061252
278.2469	1.01	lomics Pos	0.13109	0.99987	1.2777	0.35356	0.9475	0.99994	1.009	0.012981	0.91565	0.99996	0.97073	-0.04286	0.12269	0.54337	0.75278	-0.40971	2.55E-05	0.00043	0.22836	-2.1306	0.034173	0.1662	0.6488	-0.62414	0.89085	0.97532	0.97986	-0.02936	0.27318	0.94365	0.8343	-0.26136	0.17152	0.27913	1.262	0.33574
270.2796	2.48	lomics Pos	0.13116	0.99987	0.77221	-0.37293	0.073954	0.99994	0.81318	-0.29835	0.063325	0.99996	0.73808	-0.43816	0.8844	0.9874	1.0222	0.031611	0.044101	0.094896	0.82747	-0.27323	0.025255	0.13997	0.6571	-0.60582	0.095042	0.5275	0.70807	-0.49803	0.07294	0.77255	0.73447	-0.44523	0.18793	0.2955	0.82791	-0.27246
610.3153	2.61	olomics Pc	0.13124	0.99951	1.3594	0.44293	0.23697	0.99998	0.8386	-0.25394	0.31924	0.99941	0.82933	-0.26999	0.019352	0.2154	0.60267	-0.73056	0.00652	0.03547	0.54582	-0.87349	0.012294	0.084558	0.54476	-0.8763	0.83429	0.9951	1.0218	0.03105	0.89188	0.99956	1.1698	0.22624	0.023037	0.2688	2.1224	1.0857
958.6667	9.15	olomics Pc	0.13146	0.99951	1.2959	0.37392	0.72053	0.99998	0.95549	-0.06569	0.30888	0.99941	1.1488	0.20012	0.39027	0.88761	0.86454	-0.21	0.022851	0.09336	0.68569	-0.54438	0.18174	0.44831	0.75259	-0.41007	0.68985	0.99391	0.94584	-0.08033	0.63963	0.99956	0.94543	-0.08096	0.20372	0.46828	1.4804	0.56596
969.7784	0.53	lomics Pos	0.13168	0.99987	0.90856	-0.13834	0.043996	0.99994	0.80535	-0.31232	0.1383	0.99996	0.87347	-0.19518	0.44145	0.86677	0.9404	-0.08865	0.000218	0.001787	0.66584	-0.58676	0.16301	0.44781	0.84259	-0.24709	0.23102	0.67447	0.89541	-0.15937	0.27974	0.94365	0.86442	-0.21019	0.36066	0.46967	1.1377	0.18616
152.0378	10.16	lomics Pos	0.13176	0.99987	0.87767	-0.18825	0.2012	0.99994	0.84271	-0.2469	0.10545	0.99996	0.86758	-0.20493	0.00574	0.18075	0.77256	-0.37228	0.004057	0.015446	0.70033	-0.51389	0.052805	0.22547	0.75274	-0.40978	0.21868	0.66097	0.85221	-0.23071	0.010612	0.52955	0.80243	-0.31755	0.7226	0.79334	0.97596	-0.0351
917.4914	0.57	lomics Pos	0.13213	0.99987	1.3876	0.47259	0.12209	0.99994	0.82475	-0.27797	0.91817	0.99996	1.0059	0.008474	0.004813	0.17581	0.53581	-0.90019	0.000229	0.001839	0.41564	-1.2666	6.95E-05	0.019289	0.51202	-0.96574	0.44659	0.80121	0.90066	-0.15095	0.94237	0.98689	1.1035	0.14212	0.016655	0.099614	2.4855	1.3136
227.22	2.05	lomics Pos	0.13214	0.99987	1.2376	0.3075	0.49084	0.99994	0.91852	-0.12261	0.80188	0.99996	1.0246	0.035127	0.18442	0.62505	0.77486	-0.368	3.92E-06	0.000145	0.18919	-2.4021	0.008455	0.08439	0.56124	-0.83331	0.29784	0.72322	0.84023	-0.25115	0.75845	0.97988	0.96369	-0.05336	0.019627	0.10555	1.4758	0.56147
914.6414	9.19	olomics Pc	0.13239	0.99951	1.406	0.49159	0.83972	0.99998	0.97019	-0.04365	0.25105	0.99941	1.1882	0.24874	0.44043	0.92243	0.87673	-0.18979	0.026315	0.10401	0.66226	-0.59452	0.16817	0.42548	0.80113	-0.31989	0.96234	0.9974	1.0273	0.038877	0.95033	0.99956	1.0449	0.063427	0.18933	0.45476	1.5903	0.66933
937.5003	8.12	olomics Ne	0.13264	0.99918	1.1749	0.23258	0.67504	0.93744	0.94545	-0.08092	0.51294	0.99995	1.1194	0.1627	0.96044	0.99985	0.98833	-0.01694	0.84115	0.99927	0.97344	-0.03884	0.46573	0.83451	1.1449	0.19516	0.34439	0.71371	1.1035	0.14213	0.049472	0.99512	1.2674	0.34189	0.896	0.99941	1.0433	0.061116
340.1786	0.58	lomics Pos	0.13302	0.99987	1.4236	0.50954	0.010855	0.99994	0.75306	-0.40916	0.31966	0.99996	0.91276	-0.13169	0.00225	0.17581	0.5773	-0.7926	0.000381	0.002662	0.37278	-1.4236	4.68E-05	0.019289	0.53769	-0.89516	0.72533	0.92498	1.0083	0.011864	0.69537	0.97988	1.0184	0.026371	0.052531	0.14914	2.3942	1.2596
940.5106	5.22	lomics Pos	0.13316	0.99987	0.89633	-0.1579	0.11269	0.99994	0.9338	-0.09882	0.065972	0.99996	0.87249	-0.19678	0.61801	0.93921	0.95917	-0.06014	0.000634	0.003921	0.70858	-0.497	0.36025	0.6221	0.88081	-0.1831	0.61238	0.87064	0.9555	-0.06567	0.44471	0.96776	1.0876	0.1212	0.049977	0.14647	1.1807	0.23962
199.1883	2.05	lomics Pos	0.13317	0.99987	1.1673	0.22314	0.12292	0.99994	0.82521	-0.27717	0.71029	0.99996	0.96947	-0.04473	0.079539	0.47611	0.7556	-0.40431	2.52E-06	0.000144	0.17031	-2.5537	0.002783	0.061655	0.47677	-1.0686	0.12186	0.58007	0.79902	-0.32369	0.56763	0.9703	0.95505	-0.06636	0.000982	0.07528	1.3398	0.42206
404.2026	0.58	lomics Pos	0.13343	0.99987	1.5979	0.67619	0.27713	0.99994	0.84332	-0.24585	0.93987	0.99996	0.99116	-0.01281	0.01756	0.24798	0.56966	-0.81181	0.000558	0.003587	0.41601	-1.2653	0.008576	0.084941	0.58188	-0.7812	0.99504	0.99769	1.0605	0.0847	0.90215	0.98023	1.1415	0.19093	0.021865	0.10841	2.6376	1.3992
658.3679	2.78	olomics Pc	0.13347	0.99951	1.4634	0.54934	0.1906	0.99998	0.86635	-0.20698	0.90989	0.99941	1.0039	0.005685	0.016171	0.20428	0.60181	-0.73263	0.005726	0.032383	0.5734	-0.80237	0.015575	0.093261	0.62951	-0.66769	0.6043	0.99391	0.95782	-0.06218	0.62635	0.99956	0.97027	-0.04354	0.042014	0.28426	2.4211	1.2757
802.8101	10.07	lomics Pos	0.13358	0.99987	1.0996	0.13696	0.27264	0.99994	0.90814	-0.13902	0.67286	0.99996	0.96784	-0.04716	0.30618	0.76456	0.91858	-0.12252	0.013824	0.039306	0.77503	-0.36767	0.35504	0.61963	0.90402	-0.14557	0.89192	0.97532	1.0301	0.042846	0.99377	0.99743	0.99984	-0.00024	0.066582	0.16636	1.1929	0.25445
169.0492	10.17	lomics Pos	0.13361	0.99987	1.1018	0.13981	0.98634	0.99994	0.99994	-8.4E-05	0.91998	0.99996	0.9981	-0.00274	0.007415	0.19909	0.80223	-0.31791	0.001203	0.006279	0.76844	-0.37999	0.29968	0.58008	0.92883	-0.10652	0.62234	0.87467	0.98261	-0.02531	0.67104	0.97988	1.0407	0.057538	0.012408	0.098791	1.1481	0.19921
670.3258	0.57	lomics Pos	0.13387	0.99987	1.5896	0.66868	0.11665	0.99994	0.85766	-0.22152	0.91247	0.99996	1.0233	0.033288	0.008882	0.19971	0.73638	-0.44148	7.59E-06	0.000194	0.48236	-1.0518	0.004982	0.073217	0.72644	-0.46108	0.64697	0.88911	1.0945	0.13021	0.75167	0.97988	1.1668	0.22251	0.004944	0.087539	2.4498	1.2927
167.1436	2.05	lomics Pos	0.13388	0.99987	1.2119	0.27724	0.35265	0.99994	0.88724	-0.1726	0.65848	0.99996	1.0473	0.066639	0.15776	0.58685	0.79015	-0.3398	1.2E-05	0.000262	0.15688	-2.6723	0.00953	0.087209	0.54286	-0.88134	0.17654	0.64616	0.8025	-0.31742	0.57209	0.9703	0.93846	-0.09164	0.007702	0.091944	1.3914	0.47653
1072.776	10.02	lomics Pos	0.13413	0.99987	1.0905	0.12505	0.41821	0.99994	0.97604	-0.03499	0.97946	0.99996	1.005	0.007236	0.015183	0.2363	0.85438	-0.22705	2.44E-05	0.000417	0.76497	-0.38653	0.067786	0.26455	0.81581	-0.29369	0.30124	0.72409	1.1055	0.14469	0.88235	0.98023	0.99608	-0.00567	0.20951	0.31814	1.1918	0.25311
210.16	2.06	lomics Pos	0.13417	0.99987	1.2741	0.34949	0.3112	0.99994	0.88414	-0.17765	0.60351	0.99996	0.93191	-0.10173	0.17447	0.60919	0.84826	-0.23742	1.76E-05	0.000338	0.2882	-1.7949	0.12469	0.38426	0.7678	-0.3812	0.13905	0.60403	0.77812	-0.36193	0.58033	0.97073	0.93819	-0.09205	0.060486	0.15775	1.2787	0.35466
318.1713	2.73	olomics Pc	0.13436	0.99951	1.4275	0.51347	0.40284	0.99998	0.90719	-0.14052	0.55866	0.99941	1.0597	0.083606	0.028272	0.24036	0.71405	-0.4859	0.002372	0.017364	0.58759	-0.76711	0.011747	0.083709	0.62924	-0.66832	0.77863	0.99391	0.98322	-0.02441	0.92981	0.99956	1.1017	0.13979	0.0344	0.2709	2.2735	1.1849
136.1212	6.38	olomics Pc	0.13438	0.99951	1.1793	0.2379	0.078876	0.99998	0.87685	-0.1896	0.86251	0.99941	0.99204	-0.01153	0.11184	0.45341	0.78055	-0.35744	5.3E-05	0.000981	0.24123	-2.0515	0.008059	0.074445	0.61633	-0.69823	0.042284	0.41237	0.72559	-0.46278	0.068893	0.75699	0.85188	-0.23128	0.7444	0.89227	1.0417	0.058935
137.1335	6.04	olomics Pc	0.13478	0.99951	1.1449	0.19527	0.55773	0.99998	0.94127	-0.08732	0.56332	0.99941	0.94408	-0.08302	0.053305	0.29934	0.74695	-0.42092	2.14E-05	0.0005	0.29915	-1.7411	0.003198	0.049867	0.56924	-0.81288	0.018634	0.30136	0.71261	-0.48882	0.085284	0.79247	0.79215	-0.33616	0.72848	0.88156	1.0525	0.073781
170.155	5.99	olomics Pc	0.13519	0.99951	1.1619	0.21652	0.42663	0.99998	0.91643	-0.12591	0.83881	0.99941	1.0382	0.054107	0.17015	0.5845	0.8118	-0.3008	9.76E-06	0.000314	0.21053	-2.2479	0.012568	0.085318	0.57892	-0.78856	0.16908	0.74367	0.8092	-0.30544	0.29129	0.99956	0.90321	-0.14686	0.12907	0.38068	1.1312	0.17789
863.6938	6.41	lomics Pos	0.13539	0.99987	0.81273	-0.29915	0.08086	0.99994	0.80394	-0.31483	0.079619	0.99996	0.8063	-0.31061	0.26448	0.725	0.84917	-0.23588	0.035489	0.080498	0.73429	-0.44558	0.40584	0.65019	0.89542	-0.15936	0.56148	0.855	1.0784	0.10886	0.81694	0.97988	0.99747	-0.00366	0.65032	0.738	0.94403	-0.08309
493.9773	12.01	olomics Ne	0.13563	0.99918	0.90195	-0.14889	0.029312	0.41811	0.79995	-0.32202	0.062017	0.99995	0.81783	-0.29012	0.042839	0.99985	0.78932	-0.34133	0.47877	0.99927	1.0894	0.12358	0.19142	0.65203	0.827	-0.27405	0.76634	0.93262	0.98065	-0.0282	0.23655	0.99512	0.85464	-0.22662	0.30182	0.99941	0.91527	-0.12774
630.8609	3.08	olomics Pc	0.13593	0.99951	1.3608	0.44441	0.56746	0.99998	0.96181	-0.05618	0.5296	0.99941	0.96372	-0.05332	0.000247	0.077904	0.55469	-0.85025	0.000118	0.001839	0.57771	-0.79158	0.000134	0.036471	0.60693	-0.7204	0.48215	0.99391	0.91368	-0.13024	0.39708	0.99956	0.88365	-0.17846	0.008169	0.2688	1.8171	0.86165
96.0897	2.05	lomics Pos	0.13601	0.99987	1.1207	0.16445	0.39024	0.99994	0.9092	-0.13732	0.44474	0.99996	0.93967	-0.08977	0.027086	0.29092	0.6603	-0.59881	4.5E-05	0.00059	0.17614	-2.5052	0.059407	0.24149	0.58466	-0.77433	0.16977	0.64246	0.72475	-0.46444	0.32046	0.9524	0.88979	-0.16847	0.007504	0.091944	1.3977	0.48303
700.3886	3.16	olomics Pc	0.13654	0.99951	1.3724	0.45668	0.6453	0.99998	0.92119	-0.11843	0.78121	0.99941	1.0293	0.041708	0.034194	0.25403	0.61647	-0.69789	0.00982	0.047734	0.5697	-0.81173	0.010326	0.080315	0.5847	-0.77423	0.89416	0.9951	0.98429	-0.02285	0.85854	0.99956	0.99983	-0.00025	0.025165	0.2688	1.8082	0.85453
170.1545	7.02	olomics Pc	0.13654	0.99951	1.2695	0.34422	0.34825	0.99998	0.86754	-0.20499	0.61742	0.99941	1.0797	0.11069	0.37974	0.87698	0.85302	-0.22934	1.11E-05	0.000339	0.19313	-2.3723	0.005575	0.064158	0.55106	-0.85972	0.12236	0.65834	0.75967	-0.39655	0.29392	0.99956	0.86806	-0.20413	0.032914	0.2688	1.3778	0.46242
605.9953	0.53	lomics Pos	0.13668	0.99987	1.4346	0.52062	0.078047	0.99994	0.84062	-0.25047	0.84098	0.99996	0.98175	-0.02658	0.005114	0.17581	0.59731	-0.74346	5.54E-05	0.000698	0.37322	-1.4219	0.001198	0.043885	0.58218	-0.78046	0.89452	0.97532	1.0265	0.03774	0.63865	0.97988	0.96493	-0.0515	0.031672	0.12169	2.1999	1.1375
804.9899	3.29	olomics Pc	0.13683	0.99951	1.4085	0.49411	0.4862	0.99998	0.88039	-0.18379	0.75586	0.99941	0.93898	-0.09083	0.028135	0.24036	0.61597	-0.69907	0.013073	0.059423	0.51463	-0.9584	0.015953	0.093702	0.60094	-0.73472	0.96262	0.9974	1.0552	0.077544	0.52705	0.99956	0.89037	-0.16752	0.031908	0.2688	1.6998	0.7654
577.6495	0.53	lomics Pos	0.13713	0.99987	1.3323	0.41392	0.022143	0.99994	0.81937	-0.28742	0.96725	0.99996	1.0026	0.003677	0.005276	0.17581	0.62242	-0.68405	0.001299	0.006668	0.3802	-1.3952	0.004508	0.071311	0.58816	-0.76571	0.37854	0.76056	0.85532	-0.22547	0.8995	0.98023	1.0334	0.047429	0.041416	0.13573	2.1343	1.0937
468.39	5.85	olomics Pc	0.13741	0.99951	0.8408	-0.25017	0.24812	0.99998	0.88942	-0.16906	0.49572	0.99941	0.93699	-0.09389	0.1502	0.5457	0.90114	-0.15018	0.29184	0.65664	1.0962	0.13255	0.8619	0.95795	1.0287	0.040819	0.007895	0.23684	0.72959	-0.45485	0.029322	0.58535	0.80344	-0.31573	0.4813	0.7392	0.93483	-0.09723
689.9984	9.53	olomics Pc	0.13742	0.99951	1.4576	0.54359	0.53975	0.99998	0.91098	-0.13451	0.41251	0.99941	1.1102	0.15087	0.023943	0.22649	0.6648	-0.589	0.01185	0.055443	0.59551	-0.74781	0.026903	0.12339	0.70589	-0.50249	0.71898	0.99391	1.1278	0.17346	0.97228	0.99956	1.083	0.11506	0.092626	0.34627	1.7811	0.83278
955.5469	12.07	olomics Ne	0.13764	0.99918	1.1744	0.23194	0.023656	0.40327	0.795	-0.33097	0.12312	0.99995	1.2606	0.33406	0.034923	0.99985	1.182	0.24121	0.61368	0.99927	1.0299	0.042491	0.017109	0.40708	1.3807	0.46544	0.032196	0.34766	1.3016	0.38027	0.013262	0.99512	1.3085	0.38792	0.24055	0.99941	1.1203	0.16394
646.4559	9.56	olomics Pc	0.13807	0.99951	1.5263	0.61007	0.96434	0.99998	1.0028	0.004052	0.23755	0.99941	1.203	0.26663	0.091318	0.40629	0.72651	-0.46095	0.026893	0.10576	0.64364	-0.63568	0.15561	0.40477	0.76277	-0.39068	0.57026	0.99391	1.1877	0.24816	0.90325	0.99956	1.1102	0.15086	0.058158	0.30327	1.9284	0.9474
646.9511	9.55	olomics Pc	0.13812	0.99951	1.5203	0.60433	0.97791	0.99998	1.0011	0.001642	0.30238	0.99941	1.1555	0.20855	0.12391	0.48485	0.75068	-0.41374	0.035816	0.13192	0.64898	-0.62375	0.13279	0.36405	0.76255	-0.39109	0.5565	0.99391	1.1953	0.25738	0.9636	0.99956	1.0722	0.10055	0.037498	0.2799	2.0259	1.0185
368.3889	4.97	lomics Pos	0.13814	0.99987	0.86271	-0.21305	0.088415	0.99994	0.73448	-0.44521	0.079331	0.99996	0.83461	-0.26083	0.027876	0.29642	0.81499	-0.29514	0.016265	0.044363	0.80628	-0.31066	0.014359	0.10138	0.59362	-0.75238	0.073297	0.48057	0.69419	-0.5266	0.00231	0.48691	0.71646	-0.48103	0.48421	0.58631	0.91347	-0.13057
478.9769	5.2	olomics Ne	0.13855	0.99918	1.0667	0.093168	0.16497	0.5938	0.94149	-0.08699	0.20117	0.99995	1.0494	0.069541	0.43989	0.99985	1.0572	0.080272	0.34082	0.99927	1.0339	0.048107	0.020582	0.43126	1.1697	0.22612	0.003399	0.22316	1.1295	0.17567	0.2044	0.99512	1.0856	0.11852	0.41749	0.99941	1.0324	0.046029
820.7846	0.54	lomics Pos	0.13865	0.99987	1.075	0.10437	0.493	0.99994	0.94313	-0.08448	0.42489	0.99996	0.94658	-0.07921	0.037966	0.34165	0.87205	-0.19752	0.005189	0.018719	0.77426	-0.36911	0.27457	0.56196	0.88191	-0.1813	0.36058	0.7571	0.91972	-0.12073	0.2897	0.94825	0.89627	-0.158	0.015736	0.099085	1.2488	0.32055
157.142	1.66	lomics Pos	0.13881	0.99987	1.2629	0.33677	0.27244	0.99994	0.84812	-0.23767	0.49958	0.99996	1.1271	0.17266	0.075815	0.4637	0.68866	-0.53814	0.000159	0.001422	0.21091	-2.2453	0.014568	0.10138	0.56867	-0.81434	0.41275	0.78028	0.86967	-0.20147	0.345	0.96524	0.87403	-0.19425	0.086453	0.18719	1.2339	0.30325
1068.765	10.03	lomics Pos	0.13888	0.99987	1.1148	0.15672	0.24487	0.99994	0.88288	-0.17972	0.87696	0.99996	0.98939	-0.01539	0.13339	0.55754	0.8925	-0.16407	0.015323	0.04243	0.80022	-0.32153	0.26018	0.54699	0.87877	-0.18644	0.60688	0.86762	1.0342	0.048495	0.7617	0.97988	1.0254	0.036122	0.074267	0.17622	1.1421	0.19172
269.2591	6.03	olomics Pc	0.13922	0.99951	1.2866	0.3636	0.82163	0.99998	0.98437	-0.02273	0.099857	0.99941	1.1496	0.20115	0.68201	0.99905	0.9599	-0.05905	2.93E-05	0.000615	0.40539	-1.3026	0.10262	0.30554	0.72492	-0.4641	0.75176	0.99391	0.97396	-0.03806	0.62607	0.99956	1.0467	0.065784	0.004109	0.2688	1.3556	0.4389
787.9358	3.27	olomics Pc	0.13937	0.99951	1.3643	0.44819	0.5304	0.99998	0.9166	-0.12564	0.92798	0.99941	0.98676	-0.01923	0.009047	0.19702	0.56471	-0.82443	0.003211	0.021563	0.51041	-0.97028	0.004029	0.054345	0.60709	-0.72002	0.59894	0.99391	0.92851	-0.10701	0.31653	0.99956	0.83016	-0.26854	0.032434	0.2688	1.6483	0.72101
969.7983	10.02	lomics Pos	0.13939	0.99987	1.1428	0.19256	0.93486	0.99994	1.0032	0.004573	0.7581	0.99996	0.9755	-0.03578	0.40088	0.84516	0.9452	-0.08132	0.005944	0.020754	0.78035	-0.35781	0.42248	0.65844	0.91095	-0.13456	0.25469	0.69047	1.1108	0.15165	0.8983	0.98023	1.0145	0.020727	0.075515	0.17753	1.1506	0.20241
196.1779	2.05	lomics Pos	0.13955	0.99987	1.1972	0.25962	0.67707	0.99994	0.94779	-0.07736	0.99833	0.99996	0.99017	-0.01425	0.06822	0.44466	0.70905	-0.49603	2.37E-05	0.000407	0.21865	-2.1933	0.013019	0.096206	0.57053	-0.80963	0.13278	0.59878	0.78321	-0.35252	0.55564	0.96798	0.92882	-0.10654	0.008591	0.092495	1.4011	0.48657
437.3462	7.63	olomics Pc	0.13987	0.99951	0.88906	-0.16965	0.938	0.99998	1.0195	0.027876	0.17988	0.99941	0.85809	-0.22081	0.041307	0.27803	0.82619	-0.27545	0.70569	0.99688	1.0951	0.13107	0.74307	0.92766	0.98097	-0.02772	0.43054	0.99185	0.94821	-0.07672	0.45899	0.99956	0.95802	-0.06187	0.33843	0.61506	0.93124	-0.10277
180.1233	6	olomics Pc	0.14031	0.99951	1.1965	0.25882	0.2972	0.99998	0.87582	-0.19129	0.68206	0.99941	1.0348	0.049364	0.34008	0.83302	0.86402	-0.21086	4.02E-06	0.000231	0.27262	-1.875	0.011569	0.083709	0.61068	-0.71152	0.065154	0.51696	0.72664	-0.4607	0.6743	0.99956	0.95973	-0.05929	0.29185	0.56749	1.1133	0.15487
453.8785	12.06	olomics Pc	0.14048	0.99951	0.85153	-0.23188	0.73977	0.99998	0.97433	-0.03752	0.21639	0.99941	0.90811	-0.13907	0.41943	0.90805	0.90779	-0.13957	0.20102	0.4965	0.90783	-0.1395	0.98022	0.99289	0.99947	-0.00077	0.28479	0.9034	0.91457	-0.12884	0.040147	0.6387	0.86259	-0.21326	0.28951	0.56573	0.87348	-0.19516
429.891	0.54	lomics Pos	0.14067	0.99987	1.3071	0.38639	0.11258	0.99994	0.85405	-0.22761	0.52808	0.99996	0.92352	-0.11479	0.003643	0.17581	0.64151	-0.64045	0.000344	0.002463	0.4799	-1.0592	0.012251	0.093161	0.63266	-0.6605	0.52952	0.84162	0.91611	-0.12641	0.82803	0.97988	1.012	0.017142	0.034261	0.12617	2.1151	1.0807
380.2523	8.37	olomics Pc	0.14112	0.99951	1.0853	0.11811	0.64235	0.99998	1.0537	0.075417	0.90869	0.99941	1.012	0.017253	0.95198	0.99905	1.0042	0.006056	0.040694	0.14354	1.0983	0.13529	0.91527	0.97439	1.0261	0.037191	0.42962	0.99185	1.0554	0.077762	0.50973	0.99956	0.9606	-0.058	0.34281	0.61812	0.92812	-0.10761
839.8425	10.02	lomics Pos	0.14115	0.99987	1.0391	0.055281	0.052514	0.99994	0.87744	-0.18862	0.2373	0.99996	0.94129	-0.08728	0.067002	0.43947	0.93756	-0.09302	4.08E-05	0.000553	0.77618	-0.36553	0.1834	0.47109	0.91868	-0.12236	0.65196	0.89057	1.0385	0.054527	0.61289	0.97371	0.97749	-0.03285	0.007914	0.092077	1.182	0.24128
676.214	1.09	lomics Pos	0.1416	0.99987	1.3364	0.41831	0.93369	0.99994	1.0059	0.008461	0.19364	0.99996	1.2764	0.35209	0.51541	0.89797	1.0922	0.12724	0.66037	0.7113	0.95412	-0.06776	0.043024	0.19553	1.4868	0.5722	0.006685	0.3088	1.4998	0.5848	0.278	0.94365	1.2057	0.2699	0.006589	0.090084	1.3528	0.43591
834.4755	0.58	lomics Pos	0.1418	0.99987	1.3372	0.41923	0.13093	0.99994	0.83914	-0.25302	0.75208	0.99996	0.96188	-0.05607	0.019043	0.25595	0.59109	-0.75855	0.000174	0.001531	0.45405	-1.1391	0.001094	0.043236	0.556	-0.84684	0.69192	0.90976	0.96118	-0.05713	0.82198	0.97988	1.0283	0.040326	0.01535	0.09885	2.3294	1.22
940.1348	9.17	olomics Pc	0.14208	0.99951	1.427	0.51298	0.89275	0.99998	0.96954	-0.04463	0.432	0.99941	1.1152	0.15729	0.53975	0.99905	0.88559	-0.17529	0.048215	0.16384	0.66363	-0.59156	0.30881	0.61035	0.79936	-0.32308	0.76959	0.99391	0.958	-0.0619	0.68519	0.99956	0.94182	-0.08647	0.23307	0.4991	1.5163	0.60057
692.4764	9.53	olomics Pc	0.1424	0.99951	1.5907	0.66969	0.93942	0.99998	1.0067	0.009666	0.31085	0.99941	1.1595	0.21351	0.046492	0.28829	0.70214	-0.51018	0.064677	0.20252	0.68188	-0.55242	0.29355	0.59048	0.81623	-0.29295	0.51435	0.99391	1.2426	0.31337	0.78373	0.99956	1.1771	0.23527	0.042873	0.28452	2.0283	1.0203
936.1483	8.91	olomics Pc	0.14248	0.99951	1.4818	0.56732	0.80515	0.99998	0.9883	-0.01698	0.15856	0.99941	1.2372	0.30703	0.35611	0.84597	0.84741	-0.23887	0.045405	0.15665	0.67783	-0.56101	0.49928	0.79391	0.90356	-0.14631	0.40065	0.97985	1.2361	0.30579	0.38274	0.99956	1.2137	0.27947	0.04217	0.28426	1.8274	0.86977
474.3466	5.86	olomics Pc	0.14293	0.99951	0.84008	-0.25141	0.59956	0.99998	0.9376	-0.09295	0.034061	0.99941	0.78403	-0.35103	0.45446	0.93443	0.90746	-0.14009	0.80978	0.99688	0.97379	-0.03831	0.9356	0.98056	1.0687	0.095826	0.41694	0.98685	1.1574	0.21086	0.080232	0.78488	0.77655	-0.36484	0.3657	0.64464	0.89959	-0.15266
355.2828	6.16	olomics Pc	0.14321	0.99951	1.0811	0.11255	0.38173	0.99998	1.0675	0.094263	0.16072	0.99941	0.91383	-0.13	0.57746	0.99905	0.96919	-0.04515	0.014251	0.063856	1.2596	0.33299	0.98372	0.99406	1.0018	0.002557	0.34577	0.95369	1.0533	0.07492	0.3806	0.99956	0.962	-0.05589	0.83616	0.93529	1.0156	0.022297
526.9744	12.01	olomics Ne	0.14359	0.99918	0.81528	-0.29463	0.09739	0.51286	0.82919	-0.27023	0.46451	0.99995	0.91694	-0.12511	0.040623	0.99985	0.76539	-0.38572	0.43526	0.99927	0.91403	-0.12968	0.016943	0.40708	0.78389	-0.35128	0.60925	0.86704	0.95677	-0.06375	0.005233	0.99512	0.72477	-0.4644	0.081156	0.99941	0.79624	-0.32873
897.5578	4.77	lomics Pos	0.14386	0.99987	1.2648	0.33892	0.88955	0.99994	0.98791	-0.01754	0.45425	0.99996	1.0678	0.094595	0.56633	0.9122	0.94453	-0.08233	0.011464	0.034072	0.77155	-0.37417	0.32735	0.60154	0.88231	-0.18064	0.46428	0.80647	1.0775	0.10775	0.29052	0.94825	1.1334	0.1807	0.011079	0.094875	1.4013	0.48681
764.9749	12.01	olomics Ne	0.14388	0.99918	1.1195	0.16284	0.46824	0.82998	0.92876	-0.10662	0.25553	0.99995	1.1887	0.24936	0.062257	0.99985	1.152	0.20413	0.086817	0.99927	1.159	0.21287	0.13201	0.60525	1.1837	0.24328	0.002201	0.22316	1.291	0.36849	0.071478	0.99512	1.1396	0.18851	0.68563	0.99941	1.1029	0.14128
139.1123	1.66	lomics Pos	0.14404	0.99987	1.2225	0.2898	0.26693	0.99994	0.83008	-0.26867	0.68721	0.99996	1.0369	0.052313	0.14079	0.56868	0.73971	-0.43497	3.47E-05	0.000502	0.17884	-2.4832	0.00931	0.086818	0.54633	-0.87216	0.49158	0.82138	0.88379	-0.17822	0.52607	0.96776	0.89885	-0.15384	0.028713	0.11732	1.3911	0.47621
822.8511	10.01	lomics Pos	0.14417	0.99987	1.0829	0.11488	0.002859	0.99994	0.87866	-0.18663	0.79476	0.99996	0.9871	-0.01873	0.53303	0.90063	0.97825	-0.03173	9.43E-05	0.000982	0.72245	-0.46903	0.24347	0.53144	0.92973	-0.10511	0.61698	0.87206	1.0516	0.072553	0.414	0.96776	0.96749	-0.04769	0.12246	0.22701	1.2218	0.289
316.0281	5.32	olomics Pc	0.1442	0.99951	1.2117	0.277	0.33235	0.99998	1.1887	0.24935	0.79129	0.99941	1.0702	0.09791	0.35568	0.84597	1.1446	0.1948	0.85605	0.99688	0.99176	-0.01194	0.10929	0.31845	1.2277	0.29597	0.038968	0.39092	1.3251	0.40612	0.38318	0.99956	1.1268	0.17217	0.09395	0.34817	1.2113	0.2765
125.0973	6.38	olomics Pc	0.14423	0.99951	1.1357	0.18354	0.31507	0.99998	0.88423	-0.1775	0.46179	0.99941	0.94631	-0.07961	0.073253	0.35124	0.72335	-0.46724	4.23E-06	0.000231	0.28663	-1.8027	0.000736	0.041543	0.55381	-0.85254	0.022787	0.32044	0.6793	-0.55787	0.11666	0.89567	0.80302	-0.31649	0.53322	0.77332	0.95044	-0.07333
538.3082	1.29	lomics Pos	0.14426	0.99987	1.2497	0.32159	0.51659	0.99994	1.0798	0.11071	0.61524	0.99996	1.0545	0.076562	0.46949	0.88476	1.0785	0.10904	0.30633	0.39988	0.85824	-0.22054	0.4811	0.70118	1.0764	0.10619	0.020544	0.33809	1.3338	0.41559	0.072673	0.77255	1.2862	0.36311	0.0036	0.085302	1.5768	0.65698
132.139	9.96	lomics Pos	0.14461	0.99987	1.0862	0.11933	0.42033	0.99994	0.95605	-0.06484	0.97306	0.99996	0.99758	-0.0035	0.72711	0.96881	0.98097	-0.02773	0.003593	0.014055	0.82159	-0.28351	0.36764	0.6273	0.92549	-0.1117	0.12473	0.58373	1.0979	0.13471	0.33918	0.96524	1.0522	0.073467	0.014135	0.098791	1.2351	0.30467
138.9761	10.19	lomics Pos	0.14462	0.99987	0.77721	-0.36362	0.026086	0.99994	0.73354	-0.44706	0.13626	0.99996	0.78994	-0.34019	0.36952	0.823	1.1089	0.14908	0.010179	0.0311	0.58085	-0.78377	0.50759	0.7211	0.8945	-0.16085	0.52521	0.83957	0.91065	-0.13503	0.9603	0.98838	0.986	-0.02034	0.91826	0.93854	0.97952	-0.02986
188.6371	6.2	olomics Pc	0.14464	0.99951	1.118	0.1609	0.45461	0.99998	0.91096	-0.13454	0.57505	0.99941	0.94743	-0.0779	0.068349	0.34279	0.78994	-0.34018	3.39E-05	0.000681	0.21227	-2.236	0.020352	0.10614	0.63038	-0.6657	0.069318	0.53288	0.73708	-0.4401	0.62112	0.99956	0.96052	-0.05811	0.67669	0.85331	1.0354	0.05012
914.4731	8.57	olomics Pc	0.14509	0.99951	0.8654	-0.20855	0.033617	0.99998	0.8417	-0.24862	0.024185	0.99941	0.73467	-0.44483	0.071193	0.34699	0.85125	-0.23235	0.65596	0.99209	0.96431	-0.05243	0.11704	0.33273	0.83826	-0.25454	0.22349	0.82725	0.86594	-0.20766	0.095328	0.81634	0.81595	-0.29345	0.26819	0.54015	0.88045	-0.18369
390.7739	4.95	lomics Pos	0.1457	0.99987	1.1399	0.18888	0.56099	0.99994	0.95308	-0.06933	0.87272	0.99996	0.99116	-0.01281	0.44099	0.86677	0.94221	-0.08588	0.009445	0.029511	0.79186	-0.33668	0.53169	0.73721	0.93671	-0.09433	0.92819	0.98393	0.99484	-0.00746	0.72494	0.97988	1.0407	0.057591	0.004292	0.085451	1.3442	0.4268
762.8208	10.05	lomics Pos	0.14606	0.99987	1.0652	0.091191	0.54268	0.99994	0.97118	-0.04219	0.92088	0.99996	0.99604	-0.00573	0.035104	0.32905	0.88983	-0.16841	0.000248	0.001942	0.80822	-0.30718	0.38061	0.6382	0.92987	-0.1049	0.25767	0.69174	1.0953	0.13126	0.83829	0.97988	1.0159	0.022824	0.035883	0.12859	1.2503	0.32222
1140.233	4.38	lomics Pos	0.14614	0.99987	1.3405	0.42281	0.21398	0.99994	0.89674	-0.15724	0.7863	0.99996	1.0729	0.10149	0.010727	0.21362	0.62032	-0.68892	0.000699	0.004224	0.50982	-0.97194	0.006309	0.078617	0.6449	-0.63286	0.40262	0.77496	0.89007	-0.16802	0.70252	0.97988	0.97273	-0.03989	0.02283	0.10945	1.6539	0.72584
901.8122	10.02	lomics Pos	0.14621	0.99987	1.0964	0.13271	0.071614	0.99994	0.88522	-0.1759	0.50976	0.99996	0.95077	-0.07283	0.31822	0.78233	0.91497	-0.1282	0.003684	0.014365	0.71913	-0.47567	0.29205	0.57461	0.90249	-0.14802	0.89905	0.97738	0.9919	-0.01173	0.93495	0.98616	1.0101	0.014522	0.030408	0.11983	1.1943	0.25617
410.9752	10.67	olomics Ne	0.14633	0.99918	1.0492	0.069322	0.84831	0.98874	1.0105	0.015029	0.28299	0.99995	1.0657	0.091813	0.44844	0.99985	1.0507	0.071352	0.36638	0.99927	1.0583	0.081728	0.081838	0.54163	1.1456	0.19616	0.039656	0.364	1.0743	0.10345	0.83336	0.99512	0.99087	-0.01323	0.96402	0.99945	1.0018	0.002556
1026.174	8.86	olomics Pc	0.14675	0.99951	1.3563	0.43966	0.42372	0.99998	0.89392	-0.16179	0.22613	0.99941	1.1538	0.20635	0.087806	0.39517	0.78481	-0.34959	0.079423	0.23941	0.71343	-0.48716	0.017576	0.097645	0.71002	-0.49406	0.76647	0.99391	1.1034	0.14199	0.65847	0.99956	1.1232	0.16768	0.09701	0.3499	1.7822	0.83363
773.4043	0.54	lomics Pos	0.14694	0.99987	1.6764	0.74536	0.65256	0.99994	0.92651	-0.11012	0.65156	0.99996	1.0379	0.05364	0.039265	0.34395	0.59601	-0.7466	0.000967	0.005435	0.42476	-1.2353	0.013384	0.097766	0.61323	-0.70551	0.8733	0.97532	0.9868	-0.01917	0.73785	0.97988	0.98195	-0.02628	0.01089	0.094875	2.1422	1.0991
294.194	0.58	lomics Pos	0.14702	0.99987	0.77538	-0.36703	0.33732	0.99994	0.84609	-0.24112	0.34469	0.99996	0.854	-0.22769	0.3031	0.76085	0.82731	-0.2735	0.10821	0.18469	0.77985	-0.35873	0.80443	0.90041	1.0399	0.056469	0.036784	0.36759	1.4227	0.50867	0.89193	0.98023	1.0522	0.073358	0.71216	0.78595	1.0491	0.069207
126.1021	2.05	lomics Pos	0.14723	0.99987	1.2114	0.27663	0.90518	0.99994	1.0105	0.015039	0.59395	0.99996	1.0829	0.11491	0.16798	0.60254	0.7683	-0.38026	2.35E-05	0.000406	0.24168	-2.0488	0.024214	0.13719	0.59569	-0.74737	0.41638	0.78082	0.86431	-0.21037	0.88188	0.98023	1.0053	0.007594	0.012308	0.098791	1.4737	0.55946
608.3849	2.72	olomics Pc	0.14756	0.99951	1.3308	0.41226	0.51446	0.99998	0.90372	-0.14606	0.43443	0.99941	0.88561	-0.17526	0.017876	0.20936	0.62796	-0.67126	0.003229	0.021563	0.478	-1.0649	0.005001	0.061419	0.61631	-0.69828	0.50489	0.99391	0.9023	-0.14833	0.74697	0.99956	1.0141	0.020201	0.057566	0.30327	2.0567	1.0403
569.3145	2.65	olomics Pc	0.14761	0.99951	1.4567	0.54265	0.27001	0.99998	0.86301	-0.21256	0.81974	0.99941	0.9769	-0.03371	0.026435	0.23724	0.60196	-0.73225	0.003231	0.021563	0.53109	-0.91297	0.018946	0.10165	0.68375	-0.54847	0.67583	0.99391	0.97144	-0.0418	0.98869	0.99956	1.0966	0.13301	0.045692	0.28476	1.9887	0.9918
964.7845	10.03	lomics Pos	0.14765	0.99987	1.1107	0.15152	0.1896	0.99994	0.94526	-0.08121	0.94658	0.99996	0.99752	-0.00358	0.48493	0.88725	0.97207	-0.04086	0.003574	0.014007	0.78679	-0.34595	0.4271	0.66255	0.92857	-0.10692	0.31694	0.72948	1.0611	0.08557	0.15919	0.91806	1.0805	0.1117	0.012947	0.098791	1.2363	0.30607
1018.771	10.05	lomics Pos	0.14787	0.99987	1.151	0.20291	0.78985	0.99994	0.98137	-0.02713	0.39089	0.99996	1.1597	0.21381	0.25044	0.70866	1.0947	0.13056	0.004236	0.015992	0.7623	-0.39156	0.094077	0.32411	0.86142	-0.21521	0.32179	0.7311	1.161	0.21536	0.52071	0.96776	1.0495	0.069756	0.028302	0.1173	1.292	0.36966
489.7788	3.04	olomics Pc	0.14799	0.99951	1.4929	0.57815	0.14128	0.99998	0.8725	-0.19678	0.95577	0.99941	0.99509	-0.0071	8.04E-05	0.077904	0.57914	-0.78802	0.002505	0.01796	0.54838	-0.86675	0.001303	0.045425	0.62082	-0.68775	0.96943	0.9974	1.1171	0.15976	0.78236	0.99956	1.0345	0.048886	0.021875	0.2688	1.9362	0.95322
658.3697	0.58	lomics Pos	0.14805	0.99987	1.4404	0.52646	0.21732	0.99994	0.86734	-0.20533	0.93208	0.99996	0.987	-0.01888	0.008345	0.19971	0.57006	-0.81082	0.000603	0.003798	0.40913	-1.2894	0.00106	0.043236	0.56025	-0.83586	0.58989	0.86163	0.9361	-0.09527	0.9366	0.98616	1.0968	0.13334	0.018923	0.10449	2.6081	1.383
1118.26	6.23	lomics Pos	0.14819	0.99987	1.5269	0.61058	0.74237	0.99994	0.94771	-0.07748	0.40053	0.99996	1.1453	0.19567	0.030865	0.3066	0.66029	-0.59882	0.003477	0.013708	0.47117	-1.0857	0.01337	0.097766	0.64024	-0.64331	0.81363	0.9593	0.99385	-0.0089	0.90176	0.98023	1.0991	0.13632	0.061153	0.15809	2.1514	1.1053
959.1688	9.14	olomics Pc	0.14836	0.99951	1.3593	0.44286	0.51596	0.99998	0.91086	-0.1347	0.35501	0.99941	1.0969	0.13348	0.22007	0.66625	0.79882	-0.32406	0.041172	0.14474	0.67451	-0.56809	0.10619	0.31348	0.71362	-0.48678	0.56998	0.99391	0.91753	-0.12418	0.62131	0.99956	0.94977	-0.07434	0.27358	0.54681	1.4221	0.50802
834.9734	12.02	olomics Ne	0.1484	0.99918	0.92769	-0.10828	0.099155	0.51286	1.1261	0.17135	0.22648	0.99995	1.093	0.12823	0.56182	0.99985	1.107	0.14668	0.17996	0.99927	1.1552	0.20813	0.006423	0.36417	1.1959	0.25805	0.35558	0.71602	1.1639	0.21894	0.47491	0.99512	1.0757	0.10533	0.32198	0.99941	1.1519	0.20405
1141.828	5.41	lomics Pos	0.14854	0.99987	1.5635	0.6448	0.6675	0.99994	0.93581	-0.09571	0.68111	0.99996	1.0705	0.098334	0.05447	0.40237	0.65564	-0.60903	0.00253	0.010754	0.49888	-1.0032	0.058648	0.24091	0.69236	-0.53041	0.91154	0.97962	1.0561	0.078737	0.97796	0.9929	1.1061	0.14553	0.064081	0.16235	2.1141	1.08
342.9798	12	olomics Ne	0.14868	0.99918	1.0976	0.13435	0.023175	0.40327	0.90248	-0.14803	0.014274	0.99995	1.1087	0.14893	0.63677	0.99985	1.0325	0.046182	0.73166	0.99927	1.0276	0.039318	0.134	0.6057	1.1266	0.17201	0.063977	0.40279	1.1021	0.14022	0.45337	0.99512	1.0534	0.075112	0.61163	0.99941	1.0272	0.038782
718.9136	3.2	olomics Pc	0.14874	0.99951	1.3489	0.43175	0.045939	0.99998	0.79951	-0.3228	0.82653	0.99941	1.0201	0.028743	0.01416	0.20085	0.60229	-0.73146	0.000148	0.00221	0.54234	-0.88273	0.001874	0.045425	0.63069	-0.665	0.42581	0.99185	0.89039	-0.1675	0.57997	0.99956	0.9378	-0.09264	0.044819	0.28476	1.6094	0.68657
545.2981	2.95	olomics Pc	0.14936	0.99951	1.3477	0.43047	0.21359	0.99998	0.80465	-0.31356	0.25258	0.99941	0.85972	-0.21806	0.030632	0.24688	0.63046	-0.66552	0.003225	0.021563	0.54967	-0.86336	0.015406	0.092755	0.63906	-0.64598	0.60376	0.99391	0.92402	-0.114	0.7853	0.99956	0.9764	-0.03445	0.045923	0.28476	1.6924	0.75905
1006.68	9.41	olomics Pc	0.14949	0.99951	1.3052	0.38426	0.48397	0.99998	0.91978	-0.12064	0.33921	0.99941	1.1277	0.17337	0.023694	0.22481	0.75388	-0.40759	0.004931	0.028978	0.55987	-0.83683	0.027366	0.12457	0.62593	-0.67592	0.74889	0.99391	0.99125	-0.01268	0.86529	0.99956	1.0992	0.1364	0.048993	0.289	1.8084	0.85475
362.9272	10.03	lomics Pos	0.14955	0.99987	1.0443	0.0626	0.25274	0.99994	0.94505	-0.08153	0.26734	0.99996	0.95518	-0.06616	0.2545	0.71116	0.9479	-0.07719	0.000289	0.002152	0.77825	-0.36169	0.36204	0.62379	0.92355	-0.11474	0.91722	0.98047	1.0081	0.011654	0.72162	0.97988	0.98592	-0.02046	0.011496	0.095915	1.194	0.25585

644.3607	3.11	oolomics Pc	0.14966	0.99951	1.3694	0.45354	0.17014	0.99998	0.81747	-0.29077	0.8104	0.99941	1.0433	0.061196	0.068649	0.34325	0.69595	-0.52295	0.004723	0.028467	0.53671	-0.8978	0.02212	0.11005	0.61278	-0.70657	0.7595	0.99391	0.96647	-0.04921	0.83232	0.99956	0.99289	-0.0103	0.023266	0.2688	1.9939	0.99556
918.1229	9.19	oolomics Pc	0.14969	0.99951	1.4255	0.51145	0.53199	0.99998	0.91861	-0.12247	0.13806	0.99941	1.196	0.25818	0.3278	0.82368	0.85698	-0.22266	0.017433	0.075316	0.69087	-0.53352	0.2572	0.54743	0.82997	-0.26887	0.70206	0.99391	1.1025	0.14082	0.90615	0.99956	1.0098	0.01404	0.064939	0.30704	1.6478	0.72057
1155.637	0.57	domics Pos	0.14983	0.99987	1.3247	0.40561	0.62478	0.99994	0.96002	-0.05887	0.13787	0.99996	1.0998	0.13727	0.003466	0.17581	0.73701	-0.44024	2.02E-05	0.000372	0.50797	-0.97719	0.010303	0.08787	0.68688	-0.54188	0.8133	0.95921	1.0662	0.092541	0.82291	0.97988	1.0023	0.003261	0.004994	0.087539	1.8211	0.86479
792.7066	0.56	domics Pos	0.15046	0.99987	1.2768	0.35258	0.029485	0.99994	1.3722	0.4565	0.27474	0.99996	1.1481	0.19924	0.65377	0.95548	1.0629	0.087948	0.66878	0.71945	0.95729	-0.06298	0.42493	0.66036	0.91999	-0.12031	0.81577	0.95968	0.97959	-0.02975	0.29036	0.94825	1.1198	0.16322	0.009592	0.094024	1.2895	0.36677
716.4361	3.2	oolomics Pc	0.1507	0.99951	1.3493	0.43221	0.014208	0.99998	0.71527	-0.48344	0.84825	0.99941	0.9795	-0.02988	0.003963	0.17363	0.63223	-0.66149	0.001539	0.012837	0.52887	-0.91902	0.007702	0.072634	0.63682	-0.65103	0.60046	0.99391	0.93677	-0.09423	0.62137	0.99956	0.9448	-0.08192	0.040754	0.28339	1.6313	0.70599
352.3241	6.05	oolomics Pc	0.15093	0.99951	0.88706	-0.17289	0.29359	0.99998	0.87552	-0.1918	0.015144	0.99941	0.8366	-0.25739	0.6568	0.99905	1.1151	0.15713	0.42162	0.82247	1.2259	0.29379	0.15649	0.40671	0.9149	-0.12832	0.3144	0.9323	0.92788	-0.10799	0.002238	0.47452	0.79262	-0.33529	0.60835	0.81633	0.95268	-0.06994
1050.773	9.73	oolomics Pc	0.151	0.99951	1.4878	0.57316	0.82893	0.99998	0.96013	-0.0587	0.54477	0.99941	1.0646	0.090254	0.040816	0.27667	0.68675	-0.54213	0.016027	0.070021	0.61982	-0.69008	0.048346	0.18257	0.70593	-0.50239	0.96128	0.9974	1.02	0.028588	0.85616	0.99956	1.1966	0.25898	0.069565	0.3118	1.8683	0.90171
154.1238	1.67	domics Pos	0.15104	0.99987	1.1796	0.23835	0.16048	0.99994	0.84958	-0.23517	0.40365	0.99996	1.0835	0.11569	0.12248	0.54337	0.75008	-0.41488	5.24E-06	0.000159	0.18132	-2.4634	0.007126	0.081839	0.55107	-0.8597	0.23993	0.67869	0.81294	-0.29877	0.53473	0.96776	0.92665	-0.10991	0.042216	0.1368	1.3063	0.38545
458.3489	6.56	oolomics Pc	0.15174	0.99951	0.89461	-0.16067	0.057987	0.99998	0.89357	-0.16236	0.044686	0.99941	0.8031	-0.31634	0.65103	0.99905	0.96495	-0.05148	0.27268	0.62927	1.0493	0.069418	0.16269	0.41732	0.92467	-0.113	0.53278	0.99391	0.93866	-0.09133	0.92431	0.99956	0.99857	-0.00207	0.61105	0.81633	0.97003	-0.0439
368.9794	4.26	olomics Ne	0.15222	0.99918	1.0422	0.059625	0.047275	0.47152	0.91038	-0.13545	0.35588	0.99995	1.0428	0.060445	0.57682	0.99985	1.0318	0.045119	0.7465	0.99927	0.98857	-0.01658	0.15892	0.62813	1.0762	0.10596	0.14147	0.52742	1.0837	0.11593	0.15392	0.99512	1.0667	0.093092	0.28717	0.99941	1.0359	0.050834
314.7801	2.06	domics Pos	0.15243	0.99987	1.2792	0.35527	0.76548	0.99994	1.0232	0.033039	0.38454	0.99996	1.2275	0.29568	0.97493	0.99556	0.98803	-0.01738	4.44E-06	0.000149	0.12381	-3.0139	0.043857	0.19861	0.67903	-0.55846	0.96809	0.99304	0.99724	-0.00399	0.47868	0.96776	1.102	0.14014	0.007205	0.091944	1.5178	0.60203
382.7048	2.81	oolomics Pc	0.15308	0.99951	1.4291	0.51509	0.49745	0.99998	0.87025	-0.2005	0.64898	0.99941	1.0489	0.068938	0.13214	0.5064	0.66163	-0.59591	0.024554	0.098779	0.62125	-0.68674	0.11032	0.3191	0.68753	-0.5405	0.61244	0.99391	1.1741	0.23156	0.9047	0.99956	1.1285	0.17443	0.017881	0.2688	2.2517	1.171
845.4147	0.57	domics Pos	0.15338	0.99987	1.6374	0.71141	0.15603	0.99994	0.8527	-0.22989	0.95482	0.99996	1.0094	0.013462	0.029569	0.30146	0.65126	-0.61869	3.24E-06	0.000144	0.41002	-1.2862	0.001897	0.057238	0.68451	-0.54686	0.86774	0.97532	1.0613	0.085842	0.93726	0.98616	1.1498	0.2014	0.015073	0.09885	2.378	1.2498
219.2109	1.46	domics Pos	0.15343	0.99987	1.238	0.30796	0.53403	0.99994	0.91094	-0.13457	0.73912	0.99996	1.0433	0.061161	0.18357	0.62386	0.78418	-0.35075	4.78E-06	0.000152	0.19742	-2.3407	0.004223	0.071311	0.52636	-0.92588	0.20211	0.64891	0.79314	-0.33436	0.49006	0.96776	0.90625	-0.14201	0.026058	0.11342	1.3703	0.45454
114.0925	5.99	oolomics Pc	0.15376	0.99951	1.1629	0.21773	0.2338	0.99998	0.87702	-0.18932	0.56984	0.99941	1.0695	0.096975	0.14509	0.53705	0.80857	-0.30656	6.39E-06	0.000265	0.21346	-2.2279	0.016413	0.094477	0.59212	-0.75604	0.32652	0.9393	0.86479	-0.20958	0.51271	0.99956	0.94395	-0.08321	0.16079	0.41855	1.1289	0.17492
688.3875	3.16	oolomics Pc	0.15377	0.99951	1.394	0.47922	0.47666	0.99998	0.93125	-0.10275	0.53333	0.99941	0.94457	-0.08227	0.014259	0.20085	0.69017	-0.53498	0.000597	0.00635	0.53828	-0.89358	0.026161	0.12119	0.72826	-0.45747	0.77217	0.99391	0.98425	-0.0229	0.81878	0.99956	1.0258	0.036728	0.046653	0.28647	1.8114	0.85708
189.0975	10.16	domics Pos	0.15386	0.99987	1.0386	0.054577	0.079412	0.99994	0.91475	-0.12855	0.3598	0.99996	0.95709	-0.06327	0.30776	0.76699	0.94343	-0.08401	7.57E-05	0.000859	0.73343	-0.44727	0.35702	0.62123	0.91513	-0.12796	0.82942	0.96527	1.0138	0.019751	0.86154	0.98023	0.99293	-0.01023	0.015555	0.09885	1.1927	0.25427
198.086	0.6	domics Pos	0.1541	0.99987	0.85787	-0.22117	0.066237	0.99994	0.81536	-0.29448	0.63512	0.99996	0.9486	-0.07613	0.64831	0.95407	1.0414	0.058494	0.22697	0.32119	1.1629	0.21776	0.21035	0.50277	1.3128	0.39264	0.010143	0.3324	1.7974	0.84593	0.55948	0.96925	1.0696	0.097072	0.017474	0.1011	1.3278	0.40901
723.7925	4.46	domics Pos	0.15433	0.99987	1.4709	0.55673	0.46612	0.99994	0.87588	-0.19119	0.76643	0.99996	1.0433	0.061107	0.12999	0.55135	0.7044	-0.50554	0.004425	0.016574	0.44914	-1.1548	0.040892	0.18831	0.64556	-0.63138	0.89142	0.97532	1.0055	0.00786	0.94395	0.98743	1.1066	0.14613	0.083231	0.18494	1.8795	0.91038
267.2597	2.05	domics Pos	0.15452	0.99987	1.2036	0.26735	0.35682	0.99994	0.87171	-0.19808	0.53138	0.99996	1.0674	0.094075	0.15093	0.57977	0.73616	-0.4419	3.85E-06	0.000145	0.14972	-2.7396	0.007264	0.082061	0.52879	-0.91923	0.20841	0.65208	0.79904	-0.32367	0.85805	0.98023	0.97167	-0.04146	0.015161	0.09885	1.4256	0.51162
1074.234	6.3	domics Pos	0.15503	0.99987	1.521	0.60504	0.82701	0.99994	0.9467	-0.07901	0.43238	0.99996	1.1305	0.17694	0.10057	0.52103	0.67774	-0.56119	0.00987	0.030423	0.53209	-0.91026	0.078411	0.28999	0.65918	-0.60126	0.77131	0.94358	0.9528	-0.06975	0.53399	0.96776	1.2101	0.27513	0.048112	0.14468	2.1906	1.1313
742.3909	2.8	oolomics Pc	0.1551	0.99951	1.5441	0.6268	0.19991	0.99998	0.85401	-0.22767	0.040172	0.99941	1.1876	0.24802	0.040671	0.27667	0.67019	-0.57736	9.13E-05	0.001485	0.51449	-0.95878	0.012056	0.083709	0.61747	-0.69557	0.78638	0.99391	1.0174	0.024897	0.85876	0.99956	1.0707	0.098527	0.0235	0.2688	2.4512	1.2935
1094.775	10.02	domics Pos	0.15515	0.99987	0.90387	-0.14581	0.37912	0.99994	0.90292	-0.14734	0.036023	0.99996	0.8127	-0.29921	0.1972	0.64214	0.88356	-0.17859	0.0034	0.013495	0.68965	-0.53606	0.15886	0.44042	0.82147	-0.28372	0.45421	0.80208	0.90625	-0.14203	0.034785	0.64014	0.80957	-0.30477	0.20427	0.31267	1.1445	0.19466
861.4578	0.54	domics Pos	0.15535	0.99987	1.7274	0.78861	0.20733	0.99994	0.8667	-0.20639	0.40326	0.99996	1.07	0.097559	0.046921	0.3738	0.67996	-0.55647	0.000933	0.005291	0.45421	-1.1386	0.00753	0.082061	0.65686	-0.60634	0.7592	0.94108	0.97908	-0.0305	0.66693	0.97988	0.98652	-0.01958	0.002259	0.082646	2.3491	1.2321
221.8968	12.28	olomics Ne	0.1556	0.99918	1.0897	0.12395	0.56811	0.88137	0.96304	-0.05433	0.3626	0.99995	1.0417	0.05896	0.625	0.99985	0.97448	-0.0373	0.40053	0.99927	1.0612	0.085719	0.096491	0.55533	1.1229	0.16727	0.014886	0.27907	1.1402	0.18935	0.16198	0.99512	1.0787	0.10931	0.26899	0.99941	1.0644	0.090102
368.8605	0.32	oolomics Pc	0.15564	0.99951	1.2598	0.33323	0.62388	0.99998	1.2409	0.31134	0.4578	0.99941	1.1647	0.21994	0.55561	0.99905	1.0919	0.12688	0.48051	0.88102	1.088	0.12166	0.026518	0.1223	1.3531	0.43622	0.37535	0.97118	1.1034	0.14198	0.018976	0.51556	1.1733	0.23052	0.059367	0.30404	1.2806	0.35686
271.2843	6.55	oolomics Pc	0.15606	0.99951	1.1781	0.23642	0.10276	0.99998	0.79355	-0.3336	0.14802	0.99941	1.2384	0.30845	0.27012	0.74055	0.81475	-0.29556	2.9E-05	0.000615	0.22212	-2.1706	0.0265	0.1223	0.61248	-0.70727	0.2316	0.83662	0.8284	-0.2716	0.52767	0.99956	1.1872	0.2476	0.005049	0.2688	1.4241	0.51003
153.128	1.66	domics Pos	0.15635	0.99987	1.225	0.29274	0.62302	0.99994	0.91214	-0.13268	0.50207	0.99996	1.1012	0.13905	0.16216	0.59299	0.74724	-0.42036	3.89E-05	0.000541	0.18577	-2.4284	0.023874	0.13546	0.56367	-0.82708	0.53277	0.84189	0.88864	-0.17032	0.94128	0.98646	0.97997	-0.02919	0.047059	0.14355	1.3868	0.47173
252.2324	1.23	domics Pos	0.15654	0.99987	1.1659	0.22143	0.96373	0.99994	1.0064	0.009194	0.45351	0.99996	1.0956	0.13174	0.077169	0.46503	0.76284	-0.39054	1.35E-05	0.000283	0.24194	-2.0473	0.009201	0.086818	0.6004	-0.736	0.27646	0.70707	0.84114	-0.24958	0.52021	0.96776	0.9222	-0.11684	0.046407	0.14295	1.2881	0.3652
511.2902	3.25	oolomics Pc	0.15674	0.99951	1.341	0.4233	0.25158	0.99998	0.83759	-0.25568	0.81759	0.99941	0.95098	-0.07252	0.048403	0.29042	0.6835	-0.54898	0.033999	0.12689	0.66613	-0.58612	0.013354	0.085847	0.58963	-0.76212	0.38724	0.97696	0.83976	-0.25194	0.61131	0.99956	0.91556	-0.12728	0.26337	0.5342	1.5011	0.58598
218.9206	10.1	domics Pos	0.15714	0.99987	1.049	0.069069	0.17178	0.99994	0.93712	-0.0937	0.19841	0.99996	0.94099	-0.08775	0.19008	0.63224	0.92329	-0.11514	0.000313	0.002293	0.77038	-0.37635	0.25259	0.53916	0.91254	-0.13203	0.82084	0.96018	1.0134	0.019181	0.59753	0.97073	0.97842	-0.03148	0.015531	0.09885	1.1719	0.22888
631.8661	3.08	oolomics Pc	0.15731	0.99951	1.3619	0.44567	0.19935	0.99998	0.79589	-0.32936	0.67896	0.99941	0.93142	-0.1025	0.033127	0.25203	0.66596	-0.58649	0.004037	0.025181	0.52104	-0.94054	0.008911	0.075866	0.61037	-0.71226	0.4098	0.98164	0.84302	-0.24636	0.4529	0.99956	0.86817	-0.20395	0.14994	0.4037	1.597	0.67535
696.9002	3.16	oolomics Pc	0.15757	0.99951	1.351	0.43407	0.18697	0.99998	0.88354	-0.17863	0.81696	0.99941	1.0174	0.024896	0.020507	0.22205	0.62783	-0.67155	0.000268	0.00356	0.56389	-0.82651	0.002523	0.046224	0.64869	-0.6244	0.78867	0.99391	0.99471	-0.00765	0.83169	0.99956	0.99862	-0.00199	0.00927	0.2688	1.7568	0.81295
873.0929	9.21	oolomics Pc	0.15777	0.99951	1.4152	0.50101	0.94783	0.99998	0.97755	-0.03277	0.37413	0.99941	1.1294	0.17562	0.21422	0.65513	0.77703	-0.36395	0.045773	0.15724	0.63919	-0.64568	0.14639	0.38873	0.73415	-0.44585	0.9652	0.9974	1.026	0.036978	0.66675	0.99956	0.92581	-0.11122	0.11531	0.36506	1.6641	0.7347
564.9728	12.01	olomics Ne	0.15779	0.99918	0.88271	-0.17999	0.69263	0.94213	1.0839	0.11617	0.41314	0.99995	0.92449	-0.11327	0.92341	0.99985	1.0336	0.047668	0.038783	0.99927	0.82326	-0.28057	0.98292	0.99648	1.0032	0.004618	0.87806	0.95909	0.9968	-0.00463	0.9178	0.99512	0.99813	-0.0027	0.80686	0.99941	0.98539	-0.02123
763.4413	3.25	oolomics Pc	0.15796	0.99951	1.3426	0.42503	0.1288	0.99998	0.86294	-0.21267	0.61144	0.99941	0.94784	-0.07729	0.017514	0.20936	0.64966	-0.62225	0.003346	0.022235	0.55904	-0.83899	0.00197	0.045425	0.6315	-0.66315	0.59057	0.99391	0.93341	-0.09941	0.65417	0.99956	0.95314	-0.06924	0.069139	0.3118	1.6047	0.68233
1063.781	10.02	domics Pos	0.158	0.99987	0.91889	-0.12203	0.78015	0.99994	1.0236	0.033647	0.80458	0.99996	0.98924	-0.01561	0.87606	0.9874	0.99833	-0.00241	0.001373	0.006888	0.71678	-0.4804	0.002749	0.061655	0.87962	-0.18505	0.13561	0.60006	1.1364	0.18441	0.42742	0.96776	1.0403	0.056962	0.26991	0.37962	1.1325	0.1795
468.9755	4.96	olomics Ne	0.1587	0.99918	1.0675	0.094281	0.49628	0.85324	0.97251	-0.04021	0.057015	0.99995	1.0798	0.11071	0.42181	0.99985	1.0623	0.087187	0.10064	0.99927	1.0782	0.1086	0.074334	0.52589	1.13	0.1763	0.00619	0.2539	1.1224	0.16663	0.085887	0.99512	1.1071	0.14676	0.094771	0.99941	1.0754	0.10484
1009.749	5.61	domics Pos	0.15911	0.99987	1.6081	0.68533	0.43018	0.99994	0.88653	-0.17376	0.22244	0.99996	1.1595	0.21346	0.033618	0.32047	0.659	-0.60164	0.005496	0.019605	0.5102	-0.97087	0.037989	0.17861	0.70193	-0.51059	0.92597	0.98294	1.0486	0.068454	0.66454	0.97988	1.2175	0.28389	0.035761	0.12841	2.323	1.216
552.9877	3.32	oolomics Pc	0.15913	0.99951	1.4329	0.51889	0.62641	0.99998	0.92791	-0.10795	0.31919	0.99941	1.1442	0.19439	0.026122	0.23577	0.65401	-0.61261	0.027454	0.10716	0.61531	-0.70062	0.011064	0.083281	0.60083	-0.73498	0.69007	0.99391	1.1041	0.14289	0.93633	0.99956	1.0681	0.095091	0.010284	0.2688	2.0822	1.0581
842.9735	12.02	olomics Ne	0.15946	0.99918	0.85142	-0.23206	0.1784	0.60009	0.86329	-0.21208	0.36576	0.99995	0.91246	-0.13217	0.4944	0.99985	0.92084	-0.11898	0.24641	0.99927	0.89187	-0.16509	0.41481	0.81052	0.9161	-0.12642	0.96823	0.98872	1.0032	0.00461	0.007358	0.99512	0.77286	-0.37172	0.012631	0.99941	0.7595	-0.39687
685.3512	3.13	oolomics Pc	0.15959	0.99951	1.2514	0.32358	0.6248	0.99998	0.92875	-0.10664	0.28025	0.99941	0.83148	-0.26625	0.034116	0.25403	0.59456	-0.75012	0.022593	0.092545	0.62849	-0.67004	0.008352	0.075815	0.5842	-0.77546	0.95997	0.9974	1.0206	0.029443	0.32658	0.99956	0.82993	-0.26893	0.04566	0.28476	1.6271	0.70235
151.1487	2.05	domics Pos	0.15963	0.99987	1.1621	0.21669	0.16946	0.99994	0.84533	-0.24241	0.71167	0.99996	1.0392	0.055478	0.13325	0.55754	0.76083	-0.39434	2.25E-06	0.000144	0.16205	-2.6255	0.007439	0.082061	0.56866	-0.81435	0.21214	0.65409	0.79857	-0.32452	0.63196	0.97988	0.94414	-0.08293	0.009535	0.094007	1.3914	0.47659
668.4511	5.17	domics Pos	0.15986	0.99987	1.4285	0.51451	0.35694	0.99994	0.89639	-0.1578	0.53384	0.99996	1.0491	0.069205	0.036432	0.33474	0.64522	-0.63214	0.002246	0.009853	0.5111	-0.96833	0.049177	0.21583	0.74036	-0.43369	0.69423	0.9106	0.95836	-0.06136	0.8634	0.98023	1.0941	0.12981	0.038632	0.13216	2.0516	1.0367
332.98	12.01	olomics Ne	0.15995	0.99918	1.1351	0.18283	0.084824	0.50591	0.8222	-0.28243	0.79814	0.99995	1.0189	0.027046	0.69326	0.99985	0.97528	-0.03612	0.50064	0.99927	1.0597	0.083606	0.44641	0.82254	1.1029	0.14124	0.008112	0.2539	1.2421	0.3128	0.79435	0.99512	1.0208	0.029696	0.92589	0.99941	1.0492	0.069235
227.9556	10.03	domics Pos	0.15998	0.99987	1.0442	0.06246	0.16154	0.99994	0.92869	-0.10673	0.30112	0.99996	0.95264	-0.07	0.17202	0.60551	0.92195	-0.11724	0.000112	0.001118	0.7662	-0.38421	0.2231	0.51214	0.89943	-0.15292	0.99297	0.99769	1.0025	0.003601	0.86401	0.98023	0.99307	-0.01003	0.001954	0.079269	1.1961	0.2584
197.1749	1.66	domics Pos	0.1605	0.99987	1.1736	0.23089	0.18699	0.99994	0.81011	-0.30382	0.44522	0.99996	0.92237	-0.11658	0.12716	0.55024	0.77496	-0.36781	6.64E-05	0.000785	0.25561	-1.968	0.002455	0.061655	0.53029	-0.91516	0.034586	0.36356	0.68148	-0.55327	0.30629	0.9524	0.88109	-0.18264	0.057776	0.15492	1.2845	0.36124
563.5512	2.05	domics Pos	0.1606	0.99987	1.306	0.38518	0.25578	0.99994	0.77452	-0.36862	0.79927	0.99996	1.0257	0.03667	0.17144	0.60526	0.6569	-0.60626	1.38E-05	0.000287	0.049169	-4.3461	0.022767	0.13213	0.36917	-1.4376	0.15261	0.62234	0.67057	-0.57655	0.51451	0.96776	0.85879	-0.21963	0.064319	0.16253	1.387	0.47201
271.2811	2.26	domics Pos	0.16081	0.99987	1.1835	0.24302	0.70262	0.99994	0.96346	-0.05371	0.27522	0.99996	0.88243	-0.18045	0.033046	0.31659	0.58519	-0.77302	2.98E-06	0.000144	0.20158	-2.3106	0.009305	0.086818	0.48354	-1.0483	0.034958	0.36369	0.67735	-0.56203	0.38887	0.96776	0.93509	-0.09683	0.003828	0.085302	1.5194	0.60346
252.2328	5.39	oolomics Pc	0.16094	0.99951	1.3068	0.38604	0.68985	0.99998	0.95328	-0.06902	0.15006	0.99941	1.1812	0.24027	0.036101	0.25963	0.7918	-0.33679	4.67E-05	0.00089	0.25853	-1.9516	0.085905	0.26926	0.73196	-0.45016	0.91799	0.9951	0.99552	-0.00647	0.27846	0.99956	1.1515	0.20348	0.13701	0.39163	1.2111	0.27634
380.773	9.91	domics Pos	0.16182	0.99987	0.82592	-0.27593	0.88466	0.99994	0.99114	-0.01284	0.098895	0.99996	0.78355	-0.3519	0.065104	0.43627	0.71826	-0.47743	0.036224	0.081778	0.66992	-0.57795	0.25288	0.53928	0.79075	-0.33871	0.68434	0.90509	0.96382	-0.05317	0.76579	0.97988	0.96496	-0.05146	0.93703	0.95353	0.98695	-0.01896
564.3588	2.65	oolomics Pc	0.16187	0.99951	1.3999	0.48534	0.17585	0.99998	0.83187	-0.26557	0.5347	0.99941	0.92234	-0.11662	0.06406	0.33189	0.68076	-0.55477	0.000605	0.006376	0.45405	-1.1391	0.002524	0.046224	0.58471	-0.7742	0.54943	0.99391	0.94195	-0.08628	0.9373	0.99956	1.0461	0.065031	0.026912	0.2688	2.0196	1.0141
547.2974	3.3	oolomics Pc	0.16207	0.99951	1.3117	0.39149	0.58574	0.99998	0.94229	-0.08576	0.81217	0.99941	0.97302	-0.03946	0.020513	0.22205	0.65196	-0.61715	0.003403	0.022456	0.56304	-0.82869	0.025935	0.12085	0.68221	-0.55172	0.9387	0.9969	1.0211	0.030183	0.6308	0.99956	0.95226	-0.07058	0.01646	0.2688	1.7553	0.81174
173.1403	6.36	oolomics Pc	0.16242	0.99951	1.1613	0.21578	0.29603	0.99998	0.8728	-0.19627	0.29946	0.99941	0.90841	-0.13858	0.21973	0.66625	0.8621	-0.21408	0.000178	0.002551	0.49243	-1.022	0.00916	0.076536	0.65632	-0.60754	0.41978	0.98828	0.89043	-0.16742	0.82762	0.99956	0.97729	-0.03315	0.59883	0.81062	0.9478	-0.07734
633.3459	3.08	oolomics Pc	0.16242	0.99951	1.3587	0.44227	0.10985	0.99998	0.83672	-0.25718	0.9834	0.99941	1.0049	0.007081	0.002897	0.17097	0.57919	-0.78789	0.00025	0.003376	0.54399	-0.87835	0.001171	0.044437	0.58624	-0.77044	0.66426	0.99391	0.96399	-0.05291	0.55638	0.99956	0.93156	-0.10228	0.019108	0.2688	1.8173	0.86177
653.3398	2.68	oolomics Pc	0.16261	0.99951	1.3853	0.47018	0.058948	0.99998	0.807	-0.30936	0.82382	0.99941	0.97368	-0.03848	0.004449	0.18199	0.54689	-0.87068	0.00031	0.003947	0.42132	-1.247	0.002432	0.046224	0.61746	-0.69558	0.51827	0.99391	0.91723	-0.12465	0.91949	0.99956	1.1433	0.19325	0.02067	0.2688	2.1048	1.0737
697.402	3.16	oolomics Pc	0.16319	0.99951	1.4153	0.50108	0.11453	0.99998	0.79577	-0.32958	0.45331	0.99941	0.89522	-0.15968	0.035192	0.25661	0.66712	-0.58398	0.006567	0.035557	0.54204	-0.88353	0.012065	0.083709	0.64272	-0.63775	0.92401	0.9951	1.0071	0.01023	0.9248	0.99956	1.0117	0.016714	0.045842	0.28476	1.7665	0.82092
901.4794	4.1	domics Pos	0.16328	0.99987	0.83247	-0.26452	0.43996	0.99994	0.92919	-0.10595	0.85223	0.99996	1.0167	0.023883	0.66091	0.95717	0.95223	-0.07062	0.041064	0.089847	0.78027	-0.35796	0.37567	0.63269	0.8648	-0.20956	0.1689	0.64019	1.13	0.17635	0.79751	0.97988	1.0196	0.028071	0.21771	0.32555	1.3288	0.41009
564.5544	2.05	domics Pos	0.16336	0.99987	1.3193	0.3998	0.3016	0.99994	0.77999	-0.35847	0.7916	0.99996	1.0266	0.037868	0.17061	0.60402	0.64822	-0.62545	1.74E-05	0.000337	0.04662	-4.4229	0.023432	0.13415	0.34629	-1.53	0.16639	0.63634	0.6755	-0.56597	0.52572	0.96776	0.85526	-0.22557	0.091525	0.19187	1.3616	0.44526
638.4775	2.05	domics Pos	0.1634	0.99987	1.2043	0.26819	0.20915	0.99994	0.90459	-0.14466	0.55753	0.99996	1.0555	0.077913	0.13859	0.56517	0.68778	-0.53997	1.73E-05	0.000337	0.16569	-2.5934	0.01742	0.11287	0.52734	-0.92319	0.48351	0.81576	0.90067	-0.15093	0.61248	0.97371	0.9432	-0.08437	0.14096	0.24586	1.2911	0.36855
313.3292	3.64	domics Pos	0.16344	0.99987	0.83396	-0.26196	0.039506	0.99994	0.74593	-0.4229	0.076751	0.99996	0.78625	-0.34694	0.028655	0.29796	0.7432	-0.42818	0.008154	0.026325	0.73623	-0.44177	0.003623	0.067492	0.59683	-0.74461	0.03482	0.36369	0.74127	-0.43194	0.020777	0.52955	0.76765	-0.38148	0.051399	0.14811	0.7605	-0.39497
382.405	8.88	oolomics Pc	0.16401	0.99951	0.85818	-0.22064	0.035192	0.99998	0.75243	-0.41038	0.023758	0.99941	0.74216	-0.4302	0.19521	0.62875	0.86456	-0.20997	0.95402	0.9989	0.99631	-0.00533	0.00677	0.068355	0.58776	-0.7667	0.11374	0.64238	0.74027	-0.43387	0.006534	0.47452	0.66198	-0.59513	0.035897	0.27579	0.75733	-0.40101
258.2466	6.82	oolomics Pc	0.16403	0.99951	0.86061	-0.21657	0.47463	0.99998	0.88942	-0.16906	0.01409	0.99941	0.64278	-0.63761	0.025287	0.23088	0.51926	-0.94548	0.000437	0.005149	0.48386	-1.0473	0.000728	0.041543	0.41193	-1.2795	0.01664	0.29901	0.47784	-1.0654	0.02037	0.51556	0.46934	-1.0913	0.013727	0.2688	0.48635	-1.0399
1124.731	10.07	domics Pos	0.16465	0.99987	0.84215	-0.24785	0.1636	0.99994	0.85481	-0.22633	0.35126	0.99996	0.90924	-0.13727	0.38143	0.82927	0.89903	-0.15356	0.004994	0.018222	0.64974	-0.62206	0.36428	0.62554	0.89863	-0.15421	0.15956	0.63227	0.85925	-0.21884	0.46894	0.96776	0.90323	-0.14684	0.54844	0.64384	1.0674	0.094057
241.2348	2.05	domics Pos	0.16502	0.99987	1.1708	0.22747	0.46694	0.99994	0.90128	-0.14995	0.65267	0.99996	0.94696	-0.07862	0.23665	0.69488	0.84357	-0.24541	1.07E-05	0.00024	0.24482	-2.0302	0.000809	0.040694	0.58552	-0.77222	0.17626	0.64616	0.79211	-0.33623	0.41674	0.96776	0.89517	-0.15976	0.021505	0.1083	1.3619	0.44565
544.8943	12.05	oolomics Pc	0.1651	0.99951	0.93231	-0.10111	0.60603	0.99998	1.0519	0.072929	0.076447	0.99941	0.86037	-0.21698	0.96923	0.99905	1.0288	0.040929	0.9842	0.9989	1.0252	0.035838	0.56061	0.84212	1.0851	0.11787	0.1128	0.64238	0.91672	-0.12545	0.006151	0.47452	0.82944	-0.2698	0.14259	0.39464	0.93125	-0.10275
611.8364	3.05	oolomics Pc	0.16529	0.99951	1.3568	0.44026	0.69891	0.99998	0.95729	-0.06297	0.92607	0.99941	1.0245	0.034988	0.003467	0.17363	0.58754	-0.76723	0.006022	0.033508	0.5702	-0.81046	0.002236	0.045856	0.57441	-0.79986	0.45959	0.99391	0.8893	-0.16925	0.27425	0.99956	0.81381	-0.29723	0.007859	0.2688	1.7858	0.83657
1096.768	9.39	oolomics Pc	0.16551	0.99951	1.3983	0.48367	0.44354	0.99998	0.86411	-0.21071	0.3532	0.99941	1.1381	0.1866	0.02877	0.24036	0.65657	-0.60697	0.064041	0.20093	0.68332	-0.54937	0.29045	0.58611	0.82502	-0.2775	0.93792	0.9969	1.0393	0.055558	0.5646	0.99956	1.2336	0.3029	0.099759	0.35294	1.8062	0.85297
699.3832	3.16	oolomics Pc	0.16552	0.99951	1.353	0.43621	0.26741	0.99998	0.9015	-0.1496	0.71193	0.99941	1.0234	0.033325	0.006874	0.1948	0.64455	-0.63364	0.000609	0.006393	0.55149	-0.8586	0.011977	0.083709	0.71125	-0.49157	0.93961	0.9969	1.0636	0.089005	0.64859	0.99956	0.9683	-0.04647	0.028908	0.2688	1.7721	0.82542
437.7442	2.96	oolomics Pc	0.1656	0.99951	1.3905	0.47563	0.20675	0.99998	0.84218	-0.2478	0.49117	0.99941	0.94439	-0.08254	0.008558	0.19702	0.60057	-0.73561	6.88E-05	0.001205	0.59792	-0.74197	0.011871	0.083709	0.63925	-0.64555	0.87937	0.9951	1.1161	0.15842	0.7781	0.99956	1.0508	0.071429	0.039349	0.28339	2.1135	1.0796
396.2532	2.05	domics Pos	0.16561	0.99987	1.1726	0.22969	0.19974	0.99994	0.92326	-0.11519	0.77396	0.99996	1.0334	0.047353	0.32695	0.78974	0.89568	-0.15895	7.94E-05	0.000883	0.23791	-2.0715	0.021646	0.12836	0.69803	-0.51863	0.83347	0.96621	1.0002	0.000301	0.55114	0.96776	1.0536	0.075348	0.000439	0.07528	1.5725	0.65309
410.9768	12.01	olomics Ne	0.16575	0.99918	1.1093	0.1497	0.082905	0.50591	0.91622	-0.12623	0.18838	0.99995	1.0457	0.064453	0.077167	0.99985	1.0768	0.1068	0.16023	0.99927	1.0639	0.089329	0.080388	0.53781	1.1053	0.14442	0.084111	0.43873	1.1221	0.16623	0.035154	0.99512	1.1645	0.21968	0.91641	0.99941	0.9982	-0.0026
350.2558	6.39	oolomics Pc	0.16612	0.99951	1.177	0.23511	0.2901	0.99998	0.88031	-0.18392	0.39303	0.99941	0.89881	-0.15391	0.070094	0.34446	0.77376	-0.37004	0.000514	0.005826	0.49508	-1.0143	0.024195	0.1153	0.73505	-0.44408	0.20329	0.7948	0.84459	-0.24369	0.5088	0.99956	0.91998	-0.12033	0.16684	0.42787	0.83991	-0.2517
984.1658	9.43	oolomics Pc	0.16644	0.99951	1.5819	0.66168	0.17172	0.99998	0.84278	-0.24677	0.28825	0.99941	1.0963	0.13265	0.068438	0.34279	0.74177	-0.43095	0.021358	0.088524	0.6577	-0.6045	0.22079	0.50324	0.87762	-0.18832	0.91563	0.9951	1.0368	0.052127	0.92934	0.99956	1.1344	0.18194	0.0716	0.31588	1.8342	0.87519
961.14	8.89	oolomics Pc	0.16706	0.99951	1.4978	0.5828	0.76377	0.99998	0.98449	-0.02255	0.42208	0.99941	1.1997	0.26264	0.048522	0.29058	0.74442	-0.42581	0.086868	0.25669	0.78504	-0.34916	0.25538	0.54514	0.85626	-0.22388	0.3444	0.95246	1.2627	0.33649	0.52279	0.99956	1.1361	0.18406	0.039557	0.28339	1.983	0.98772
536.9715	12	olomics Ne	0.16721	0.99918	1.2151	0.28111	0.30669	0.73566	1.1706	0.22728	0.68371	0.99995	1.0535	0.07516	0.98332	0.99985	1.0068	0.00983	0.008447	0.69321	1.3186	0.39897	0.5898	0.89518	1.0552	0.077526	0.094726	0.46226	1.161	0.21537	0.75586	0.99512	0.97435	-0.03749	0.51017	0.99941	1.0777	0.10797
70.0666	0.59	domics Pos	0.16724	0.99987	0.84491	-0.24314	0.023994	0.99994	0.77088	-0.37543	0.92474	0.99996	1.0202	0.028857	0.20222	0.64732	1.3405	0.42278	0.54162	0.60947	1.1716	0.22852	0.01135	0.091661	1.5394	0.62233	0.000211	0.15466	1.9338	0.9514	0.15358	0.91321	1.4863	0.57168	0.015247	0.09885	1.5121	0.59653
180.1227	1.67	domics Pos	0.16725	0.99987	1.1786	0.23712	0.27259	0.99994	0.87255	-0.19669	0.76326	0.99996	1.0394	0.055811	0.15914	0.58914	0.78114	-0.35634	5.51E-06	0.000159	0.1872	-2.4173	0.019017	0.1181	0.5709	-0.8087	0.24979	0.68621	0.83336	-0.26298	0.97167	0.9921	0.99271	-0.01055	0.01484	0.098791	1.3416	0.42397
477.897	10.14	domics Pos	0.16749	0.99987	1.2007	0.26389	0.72136	0.99994	0.97288	-0.03967	0.94245	0.99996	1.0023	0.003301	0.77249	0.97526	0.97702	-0.03355	0.043883	0.094588	0.80886	-0.30604	0.68194	0.83502	0.96916	-0.04519	0.096707	0.53287	1.1332	0.18035	0.2551	0.94365	1.1534	0.20584	0.2395	0.34753	1.3011	0.37977
508.9616	3.25	oolomics Pc	0.1677	0.99951	1.3131	0.39297	0.48491	0.99998	0.92024	-0.11991	0.87984	0.99941	1.0167	0.023901	0.030356	0.24688	0.66748	-0.58321	0.012277	0.056871	0.62713	-0.67317	0.008481	0.075866	0.61912	-0.69171	0.90049	0.9951	1.008	0.011461	0.43651	0.99956	0.87899	-0.18609	0.009845	0.2688	1.8676	0.90115
1194.881	9.54	oolomics Pc	0.16795	0.99951	0.86107	-0.2158	0.42614	0.99998	0.90187	-0.14901	0.1377	0.99941	0.84518	-0.24267	0.072646	0.35072	0.76491	-0.38664	0.03413	0.12723	0.77852	-0.3612	0.81931	0.94504	0.9922	-0.0113	0.57467	0.99391	0.94231	-0.08572	0.10168	0.84285	0.82379	-0.27966	0.95062	0.97899	0.99901	-0.00143
338.1678	2.69	oolomics Pc	0.16798	0.99951	1.3873	0.47224	0.28976	0.99998	0.86118	-0.21562	0.72964	0.99941	0.95682	-0.06368	0.073855	0.35356	0.67129	-0.575	0.007087	0.03713	0.59808	-0.74159	0.010413	0.080593	0.59745	-0.7431	0.6617	0.99391	0.97332	-0.03902	0.93143	0.99956	1.0932	0.12857	0.028754	0.2688	2.0146	1.0105
1095.2	4.18	domics Pos	0.16804	0.99987	1.4652	0.55112	0.86216	0.99994	0.98438	-0.02271	0.29004	0.99996	1.2798	0.35588	0.53175	0.90063	0.8183	-0.28929	0.040216	0.088434	0.50386	-0.9889	0.23185	0.51881	0.70554	-0.50319	0.68974	0.90885	1.1439	0.19394	0.59829	0.97073	1.1779	0.23623	0.14041	0.24559	2.1486	1.1034
592.3222	0.53	domics Pos	0.16808	0.99987	1.8811	0.9116	0.425	0.99994	0.91635	-0.12603	0.37423	0.99996	0.92154	-0.11787	0.019137	0.25595	0.55482	-0.8499	0.000807	0.004757	0.41797	-1.2585	0.000717	0.040218	0.52192	-0.93811	0.49346	0.82295	0.91461	-0.12876	0.82329	0.97988	1.0127	0.018254	0.017991	0.10296	1.9694	0.97779
423.7389	2.9	oolomics Pc	0.16832	0.99951	1.293	0.37072	0.19836	0.99998	0.80607	-0.31102	0.34665	0.99941	0.88907	-0.16964	0.013978	0.20085	0.56341	-0.82775	0.00666	0.0358	0.57365	-0.80177	0.021037	0.10792	0.6381	-0.64815	0.74337	0.99391	1.0142	0.020373	0.54667	0.99956	0.94661	-0.07915	0.073435	0.31731	1.8824	0.91256
470.2516	3.01	oolomics Pc	0.16848	0.99951	1.4088	0.49442	0.014295	0.99998	0.75933	-0.39719	0.49948	0.99941	1.0553	0.077602	0.053589	0.29934	0.709	-0.49613	0.00714	0.037238	0.59393	-0.75163	0.002506	0.046224	0.65965	-0.60022	0.83035	0.9951	1.2026	0.26618	0.99803	0.99956	1.0696	0.097129	0.030753	0.2688	2.3027	1.2033
431.9813	4.91	olomics Ne	0.16872	0.99918	1.0704	0.098114	0.032842	0.44426	0.88502	-0.17622	0.39602	0.99995	1.0551	0.07733	0.30523	0.99985	1.054	0.075928	0.49271	0.99927	1.0401	0.056757	0.4511	0.82254	1.069	0.096257	0.052709	0.39335	1.1047	0.14369	0.51154	0.99512	1.0417	0.058986	0.22267	0.99941	1.0575	0.080664
941.575	10.49	olomics Ne	0.16897	0.99918	1.1809	0.23984	0.16995	0.59971	0.89017	-0.16785	0.21818	0.99995	1.0711	0.099073	0.020477	0.99985	1.1653	0.22075	0.55081	0.99927	1.0396	0.056062	0.011881	0.40245	1.2707	0.34567	0.038433	0.35842	1.2743	0.34966	0.080266	0.99512	1.1083	0.14841	0.94409	0.99941	1.0004	0.000554
666.8799	3.13	oolomics Pc	0.1691	0.99951	1.3294	0.41081	0.023517	0.99998	0.85664	-0.22325	0.56811	0.99941	1.0416	0.058837	0.012364	0.20085	0.64488	-0.6329	0.000523	0.0059	0.53009	-0.9157	0.001778	0.045425	0.62318	-0.68228	0.94804	0.9974	1.0599	0.083953	0.77442	0.99956	1.0112	0.015999	0.02868	0.2688	1.9107	0.93407
500.7972	3.08	oolomics Pc	0.16941	0.99951	1.5046	0.58938	0.75023	0.99998	1.0663	0.092613	0.81954	0.99941	1.0264	0.037604	0.001693	0.14411	0.65039	-0.62062	0.093183	0.27078	0.73787	-0.43857	0.027191	0.12413	0.69008	-0.53517	0.33308	0.94694	1.2129	0.27844	0.78661	0.99956	1.0034	0.004966	0.000726	0.2688	2.3328	1.2221
795.4174	0.54	domics Pos	0.17052	0.99987	1.5416	0.62438	0.29931	0.99994	0.85901	-0.21926	0.83967	0.99996	1.0161	0.023024	0.029405	0.30146	0.60283	-0.73017	0.00097	0.005441	0.39452	-1.3418	0.010632	0.089124	0.60111	-0.7343	0.46538	0.80647	0.88047	-0.18365	0.76474	0.97988	0.98149	-0.02695	0.009478	0.093865	2.1456	1.1014
261.2222	5.65	oolomics Pc	0.1707	0.99951	1.1636	0.2186	0.2389	0.99998	0.88439	-0.17724	0.97069	0.99941	1.0031	0.004397	0.064754	0.33326	0.74111	-0.43224	6.77E-05	0.001198	0.28525	-1.8097	0.002591	0.046402	0.56152	-0.83259	0.16166	0.73694	0.80242	-0.31757	0.12857	0.90554	0.85621	-0.22396	0.14044	0.39464	1.1422	0.19186
895.106	9.2	oolomics Pc	0.17094	0.99951	1.4042	0.48979	0.9753	0.99998	0.99043	-0.01387	0.37032	0.99941	1.1192	0.16242	0.23721	0.69639	0.79759	-0.32628	0.028504	0.11044	0.63446	-0.65639	0.090071	0.27816	0.7055	-0.50328	0.84259	0.9951	1.0681	0.095095	0.60089	0.99956	0.91573	-0.12701	0.11108	0.36485	1.5744	0.65478
828.573	8.83	olomics Ne	0.1712	0.99918	1.0633	0.088608	0.10618	0.52291	0.92059	-0.11936	0.82857	0.99995	0.99132	-0.01257	0.98338	0.99985	1.0149	0.021373	0.68559	0.99927	1.0356	0.050411	0.006238	0.36417	1.2079	0.27251	0.30436	0.6812	1.1109	0.15171	0.9005	0.99512	0.99821	-0.00259	0.2009	0.99941	0.95496	-0.06649
359.9315	12.1	oolomics Pc	0.17148	0.99951	0.85346	-0.2286	0.73571	0.99998	0.97721	-0.03326	0.25981	0.99941	0.93797	-0.09239	0.96145	0.99905	1.0064	0.009228	0.34695	0.73229	0.93319	-0.09975	0.62661	0.87981	0.96254	-0.05508	0.017683	0.30109	0.80056	-0.32092	0.72378	0.99956	0.9714	-0.04186	0.42817	0.69774	0.93096	-0.10321
385.3314	8.12	oolomics Pc	0.17158	0.99951	0.90841	-0.13858	0.91358	0.99998	1.0072	0.010413	0.56942	0.99941	1.0479	0.067462	0.3662	0.86198	0.90321	-0.14687	0.99858	0.9989	1.0022	0.003127	0.47629	0.77695	0.94075	-0.08812	0.20517	0.7959	0.8868	-0.17332	0.041784	0.64572	0.86557	-0.20828	0.43814	0.7074	0.94588	-0.08026
830.0707	9.24	oolomics Pc	0.1717	0.99951	1.4224	0.50831	0.37135	0.99998	0.87195	-0.19768	0.71174	0.99941	1.0391	0.055346	0.045854	0.2866	0.69811	-0.51848	0.005767	0.032556	0.58613	-0.7707	0.061532	0.21393	0.7294	-0.45522	0.79534	0.99391	1.0468	0.065989	0.60998	0.99956	0.94122	-0.08739	0.070954	0.31396	1.5808	0.6607
1023.753	10.07	domics Pos	0.17178	0.99987	1.088	0.12172	0.32916	0.99994	1.0962	0.13254	0.82901	0.99996	0.98253	-0.02543	0.3328	0.79293	0.91376	-0.13012	0.007712	0.025198	0.75344	-0.40843	0.26314	0.54979	0.90242	-0.14813	0.97536	0.99341	1.0076	0.010864	0.29061	0.94825	1.0964	0.13277	0.080689	0.18227	1.2296	0.2982
126.0902	11.83	oolomics Pc	0.17222	0.99951	1.1255	0.17056	0.12607	0.99998	1.1498	0.20135	0.21718	0.99941	1.1108	0.15166	0.72867	0.99905	1.0449	0.063382	0.040988	0.14426	1.2132	0.27884	0.23318	0.52056	1.1356	0.18341	0.18813	0.76465	1.161	0.21539	0.42835	0.99956	1.0827	0.11467	0.067277	0.30938	1.1648	0.22013
666.4747	9.52	oolomics Pc	0.17239	0.99951	1.454	0.54	0.56923	0.99998	0.91831	-0.12294	0.282	0.99941	1.1513	0.20322	0.02325	0.22477	0.66065	-0.59804	0.018348	0.078529	0.61262	-0.70693	0.086625	0.26979	0.756	-0.40354	0.84135	0.9951	1.0996	0.13703	0.70176	0.99956	1.2145	0.28039	0.090933	0.34353	1.8243	0.86736
609.3179	2.61	oolomics Pc	0.17253	0.99951	1.4733	0.55901	0.11339	0.99998	0.81018	-0.30369	0.96469	0.99941	0.98519	-0.02153	0.011069	0.19869	0.59004	-0.7611	0.002513	0.01796	0.4663	-1.1007	0.015765	0.093567	0.61345	-0.70499	0.90897	0.9951	1.0499	0.07031	0.77827	0.99956	1.0477	0.067215	0.065239	0.30704	2.0982	1.0692
442.9745	4.99	olomics Ne	0.17286	0.99918	1.0352	0.049877	0.10865	0.52291	0.92512	-0.11229	0.30333	0.99995	1.0388	0.05497	0.7426	0.99985	1.0268	0.038171	0.67765	0.99927	1.0189	0.027078	0.40653	0.80456	1.0552	0.077491	0.038251	0.35842	1.0859	0.11893	0.84874	0.99512	1.0116	0.016638	0.84375	0.99941	1.0135	0.019376

633.8477	3.08	oolomics Po	0.17318	0.99951	1.3567	0.44012	0.071952	0.99998	0.82603	-0.27573	0.76835	0.99941	0.97202	-0.04094	0.009271	0.19702	0.59918	-0.73893	0.000756	0.007509
123.0136	2.64	olomics Ne	0.17374	0.99918	1.0546	0.076744	0.79736	0.97675	0.98776	-0.01777	0.51897	0.99995	0.96187	-0.05609	0.50461	0.99985	1.0476	0.067065	0.007433	0.69321
596.3036	1.84	domics Pos	0.17381	0.99987	1.2481	0.31976	0.89479	0.99994	0.96342	-0.05376	0.61634	0.99996	1.1671	0.22288	0.99779	0.99936	0.99834	-0.0024	0.45956	0.54058
749.3118	2.44	olomics Ne	0.17388	0.99918	0.67781	-0.56105	0.92355	0.9934	0.98616	-0.0201	0.21187	0.99995	1.3149	0.39493	0.49695	0.99985	1.2736	0.34896	0.069061	0.99927
363.2174	3.19	oolomics Po	0.17398	0.99951	1.3528	0.43594	0.20245	0.99998	1.4357	0.52171	0.085845	0.99941	1.5786	0.65863	0.51136	0.98198	1.0994	0.13665	0.71583	0.99688
1008.667	9.42	oolomics Po	0.17415	0.99951	1.4342	0.5202	0.99422	0.99998	0.9976	-0.00347	0.86796	0.99941	1.0007	0.000973	0.030769	0.24688	0.63549	-0.65406	0.020629	0.086183
466.5929	3.17	oolomics Po	0.17459	0.99951	1.2701	0.34496	0.24483	0.99998	0.87349	-0.19514	0.56928	0.99941	0.93256	-0.10074	0.015445	0.20428	0.60166	-0.73299	0.001481	0.012568
151.1139	4.37	oolomics Po	0.17463	0.99951	0.83871	-0.25375	0.45425	0.99998	0.88207	-0.18103	0.78952	0.99941	0.98069	-0.02813	0.99111	0.99905	1.0138	0.019835	0.28403	0.64553
917.1189	9.19	oolomics Po	0.17482	0.99951	1.3991	0.48449	0.56933	0.99998	0.89288	-0.16347	0.53755	0.99941	1.0813	0.11279	0.39392	0.88909	0.83664	-0.25732	0.033317	0.12494
193.655	2.06	domics Pos	0.17508	0.99987	1.1521	0.20423	0.36235	0.99994	0.91127	-0.13405	0.50122	0.99996	1.0666	0.092979	0.26956	0.73017	0.8623	-0.21374	4.58E-05	0.000596
667.2984	1.83	domics Pos	0.17522	0.99987	1.1164	0.1588	0.85963	0.99994	0.98812	-0.01724	0.93027	0.99996	0.99846	-0.00222	0.1286	0.55024	0.90323	-0.14684	0.30052	0.39404
486.972	5.18	olomics Ne	0.1753	0.99918	1.0566	0.079444	0.49036	0.85323	0.96736	-0.04787	0.15291	0.99995	1.0535	0.075188	0.43626	0.99985	1.0478	0.067426	0.41627	0.99927
414.8677	10.17	domics Pos	0.17541	0.99987	1.2118	0.27714	0.42355	0.99994	1.0809	0.11225	0.36329	0.99996	1.1031	0.14161	0.77056	0.97526	0.97655	-0.03423	0.015925	0.04378
594.9732	12.01	olomics Ne	0.1755	0.99918	1.1848	0.24462	0.98949	0.99664	0.99904	-0.00138	0.8221	0.99995	1.0486	0.068507	0.70317	0.99985	1.0422	0.059599	0.29531	0.99927
743.9106	3.22	oolomics Po	0.17561	0.99951	1.3337	0.41544	0.030729	0.99998	0.75822	-0.39931	0.94046	0.99941	1.0019	0.002675	0.017574	0.20936	0.57776	-0.79146	0.000869	0.008425
471.9787	12.01	olomics Ne	0.17588	0.99918	1.2173	0.28365	0.80003	0.97675	1.03	0.04259	0.12359	0.99995	1.1882	0.24883	0.2391	0.99985	1.1714	0.22822	0.72765	0.99927
285.2504	4.73	domics Pos	0.17661	0.99987	0.87909	-0.18592	0.20641	0.99994	0.83035	-0.2682	0.26104	0.99996	0.87247	-0.19683	0.24974	0.70866	0.87699	-0.18937	0.004192	0.015842
278.052	5.24	oolomics Po	0.17687	0.99951	0.87505	-0.19257	0.63354	0.99998	0.95622	-0.06458	0.031406	0.99941	0.86387	-0.21111	0.92085	0.99905	0.99148	-0.01235	0.26263	0.611
362.2044	2.86	oolomics Po	0.17744	0.99951	1.3581	0.44157	0.51465	0.99998	0.88958	-0.1688	0.80263	0.99941	1.011	0.015766	0.022819	0.22443	0.6187	-0.69269	0.010827	0.051673
906.8258	10.02	domics Pos	0.17789	0.99987	1.0629	0.08807	0.46095	0.99994	0.96645	-0.04923	0.74359	0.99996	0.9862	-0.02005	0.37763	0.82724	0.96243	-0.05524	0.001396	0.006975
975.1487	9.12	oolomics Po	0.17825	0.99951	1.2347	0.30417	0.17964	0.99998	0.79793	-0.32567	0.84023	0.99941	1.0391	0.05527	0.09849	0.41868	0.70474	-0.50483	0.014887	0.066048
808.577	4.76	domics Pos	0.17827	0.99987	1.0726	0.10118	0.60332	0.99994	1.0285	0.040496	0.82363	0.99996	1.0086	0.012401	0.38728	0.83523	0.95579	-0.06523	0.005776	0.020391
623.9593	9.62	oolomics Po	0.17843	0.99951	1.4668	0.55269	0.80086	0.99998	0.96511	-0.05123	0.33622	0.99941	1.1517	0.2038	0.044354	0.28455	0.74212	-0.43027	0.030633	0.1171
129.0365	10.16	domics Pos	0.17846	0.99987	1.1061	0.14554	0.34628	0.99994	0.94125	-0.08735	0.92093	0.99996	1.0138	0.019812	0.64065	0.95076	1.0338	0.047977	0.002911	0.011987
472.9768	12.02	olomics Ne	0.17929	0.99918	0.89602	-0.15839	0.002122	0.34605	0.7168	-0.48035	0.69409	0.99995	0.9727	-0.03993	0.1534	0.99985	0.91261	-0.13193	0.37362	0.99927
795.6397	5.85	domics Pos	0.17991	0.99987	0.83596	-0.2585	0.15016	0.99994	0.72375	-0.46645	0.10753	0.99996	0.78726	-0.34508	0.0146	0.231	0.66496	-0.58867	0.25828	0.35337
634.8766	10.02	domics Pos	0.18041	0.99987	1.0419	0.059214	0.26243	0.99994	0.95009	-0.07387	0.25293	0.99996	0.96267	-0.05489	0.10486	0.52894	0.93519	-0.09667	9.31E-05	0.000975
709.9991	9.46	oolomics Po	0.18071	0.99951	1.3883	0.47327	0.49709	0.99998	0.91451	-0.12892	0.37332	0.99941	1.101	0.13886	0.032655	0.2515	0.70706	-0.50009	0.007037	0.037008
565.5576	2.05	domics Pos	0.18072	0.99987	1.2799	0.35606	0.18026	0.99994	0.73522	-0.44376	0.84563	0.99996	0.9979	-0.00303	0.14423	0.57187	0.61391	-0.7039	3.47E-05	0.000502
181.1593	2.82	domics Pos	0.1808	0.99987	1.1772	0.23539	0.37597	0.99994	0.88781	-0.17168	0.068593	0.99996	0.79414	-0.33254	0.013682	0.22898	0.55119	-0.85939	3.24E-05	0.000487
476.975	12.01	olomics Ne	0.18089	0.99918	1.1446	0.1949	0.52965	0.86583	1.1204	0.16398	0.42887	0.99995	1.0461	0.064996	0.41735	0.99985	0.93148	-0.1024	0.4465	0.99927
643.4969	9.59	oolomics Po	0.18138	0.99951	1.458	0.54394	0.81559	0.99998	0.9704	-0.04335	0.099173	0.99941	1.2473	0.31885	0.2116	0.65263	0.84102	-0.2498	0.19321	0.48303
159.9687	10.03	domics Pos	0.18157	0.99987	1.0589	0.08261	0.75248	0.99994	0.98245	-0.02555	0.50097	0.99996	0.95644	-0.06425	0.71356	0.96346	1.0145	0.020756	0.014094	0.039843
443.865	10.14	domics Pos	0.18233	0.99987	0.87431	-0.19378	0.017896	0.99994	0.7242	-0.46555	0.27995	0.99996	0.89101	-0.16648	0.029272	0.30146	0.77463	-0.36842	0.006009	0.020905
734.4854	9.43	oolomics Po	0.18278	0.99951	1.5345	0.61774	0.63333	0.99998	0.93057	-0.10381	0.5289	0.99941	1.0776	0.10786	0.17145	0.58702	0.77569	-0.36644	0.009743	0.047435
692.8352	10.05	domics Pos	0.1831	0.99987	1.0649	0.090733	0.44723	0.99994	0.95456	-0.06709	0.31307	0.99996	0.94708	-0.07844	0.19913	0.64478	0.92434	-0.1135	0.001148	0.006061
768.7605	0.56	domics Pos	0.18346	0.99987	1.1005	0.13816	0.27922	0.99994	0.90101	-0.15038	0.30379	0.99996	0.89384	-0.16192	0.49544	0.88983	0.94916	-0.07528	0.000598	0.003777
1042.8	10.02	domics Pos	0.18402	0.99987	1.0598	0.083835	0.60529	0.99994	0.97366	-0.0385	0.43194	0.99996	0.96156	-0.05655	0.26738	0.72822	0.93873	-0.09121	0.001077	0.005826
703.8679	10.02	domics Pos	0.18446	0.99987	1.0434	0.061304	0.23884	0.99994	0.9165	-0.12579	0.13243	0.99996	0.92628	-0.11048	0.10491	0.52894	0.92623	-0.11056	0.000347	0.00247
318.1672	0.58	domics Pos	0.18447	0.99987	1.4183	0.50412	0.24201	0.99994	0.84516	-0.2427	0.67294	0.99996	0.96107	-0.05728	0.041664	0.35471	0.60038	-0.73604	0.000539	0.003482
1111.49	6.01	domics Pos	0.18451	0.99987	1.2273	0.29547	0.89787	0.99994	0.97325	-0.03912	0.91095	0.99996	1.0759	0.10553	0.73963	0.97237	1.0443	0.062531	0.82678	0.85387
1135.788	5.16	domics Pos	0.18455	0.99987	1.3774	0.46198	0.48247	0.99994	0.86239	-0.21359	0.71597	0.99996	1.04	0.056638	0.3016	0.76019	0.80542	-0.31219	0.014933	0.041713
567.8136	2.98	oolomics Po	0.18476	0.99951	1.3814	0.46608	0.067218	0.99998	0.80297	-0.31658	0.12522	0.99941	0.87274	-0.19638	0.019223	0.21472	0.67989	-0.55662	0.005072	0.02975
638.3316	1.82	domics Pos	0.18525	0.99987	1.1758	0.23366	0.98848	0.99994	0.999	-0.00145	0.67885	0.99996	1.0587	0.082252	0.44625	0.86909	1.064	0.089535	0.37318	0.46566
1183.247	4.08	domics Pos	0.1854	0.99987	1.4398	0.52586	0.55255	0.99994	0.93283	-0.10032	0.1434	0.99996	1.3374	0.41943	0.16904	0.6035	0.8365	-0.25756	0.001368	0.006881
807.4672	3.3	oolomics Po	0.18549	0.99951	1.3337	0.4154	0.31566	0.99998	0.83126	-0.26663	0.70138	0.99941	0.92285	-0.11583	0.012385	0.20085	0.53965	-0.88992	0.002514	0.01796
722.7867	4.21	domics Pos	0.18551	0.99987	1.4036	0.48909	0.39308	0.99994	0.83111	-0.26689	0.77559	0.99996	1.0601	0.084178	0.044071	0.36496	0.66933	-0.57921	0.002143	0.009534
428.2387	3	oolomics Po	0.18578	0.99951	1.3811	0.4658	0.012254	0.99998	0.76825	-0.38036	0.56274	0.99941	0.93432	-0.09802	0.008699	0.19702	0.64835	-0.62516	0.001889	0.014951
168.0736	4.38	oolomics Po	0.18596	0.99951	0.86855	-0.20331	0.060546	0.99998	0.80734	-0.30875	0.2456	0.99941	0.86437	-0.21029	0.37828	0.87552	0.91803	-0.12339	0.51981	0.91793
1030.874	11.98	olomics Ne	0.18613	0.99918	1.0633	0.088489	0.84434	0.98874	0.98925	-0.0156	0.77276	0.99995	0.98192	-0.02633	0.95789	0.99985	0.99772	-0.00329	0.77597	0.99927
481.2606	2.5	oolomics Po	0.18618	0.99951	1.4475	0.53352	0.097276	0.99998	0.82651	-0.27489	0.94424	0.99941	1.0044	0.006379	0.005027	0.18937	0.58649	-0.76982	0.007277	0.037886
1176.866	9.6	oolomics Po	0.18632	0.99951	1.5744	0.65484	0.99809	0.99998	1.0005	0.000716	0.15917	0.99941	1.2197	0.28649	0.10904	0.44634	0.77212	-0.3731	0.041932	0.14691
433.9028	6.37	oolomics Po	0.18641	0.99951	1.3786	0.46318	0.45701	0.99998	0.88647	-0.17385	0.96362	0.99941	1.0229	0.032714	0.48839	0.96551	0.89663	-0.15741	3.16E-05	0.000656
135.1179	6	oolomics Po	0.18652	0.99951	1.1482	0.19937	0.25138	0.99998	0.89358	-0.16233	0.69285	0.99941	0.96396	-0.05295	0.078058	0.3659	0.73925	-0.43586	3.4E-06	0.000231
102.9426	12.1	olomics Ne	0.18742	0.99918	0.82323	-0.28063	0.016997	0.37363	0.62227	-0.68438	0.35909	0.99995	0.88955	-0.16885	0.32615	0.99985	0.83995	-0.25163	0.49437	0.99927
648.9536	9.59	oolomics Po	0.18757	0.99951	1.3645	0.44839	0.63461	0.99998	0.92842	-0.10715	0.64124	0.99941	1.0537	0.075494	0.022894	0.22443	0.63862	-0.64697	0.006252	0.034132
1077.789	10.02	domics Pos	0.18769	0.99987	0.86239	-0.21359	0.58136	0.99994	0.92981	-0.10499	0.15925	0.99996	0.84411	-0.2445	0.20689	0.65238	0.85834	-0.22037	0.024394	0.060555
719.9168	3.2	oolomics Po	0.1878	0.99951	1.3326	0.4142	0.028997	0.99998	0.82873	-0.27103	0.75134	0.99941	1.0259	0.036894	0.034635	0.25527	0.68024	-0.55588	0.000535	0.006001
1195.732	0.52	domics Pos	0.18826	0.99987	1.0959	0.13209	0.2998	0.99994	0.9498	-0.07431	0.2104	0.99996	1.0729	0.10153	0.9417	0.99181	1.0188	0.026816	0.000378	0.002653
961.6466	9.15	oolomics Po	0.18832	0.99951	1.3251	0.4061	0.69305	0.99998	0.91797	-0.12349	0.41617	0.99941	1.1258	0.17101	0.25661	0.72821	0.77424	-0.36915	0.039814	0.14139
410.8583	0.56	domics Pos	0.18862	0.99987	1.0675	0.094213	0.50529	0.99994	0.9742	-0.03771	0.32484	0.99996	0.96195	-0.05597	0.021355	0.26504	0.88887	-0.16996	0.000596	0.003768
137.1329	1.66	domics Pos	0.18865	0.99987	1.2043	0.26821	0.45048	0.99994	0.86718	-0.2056	0.5863	0.99996	1.0831	0.11513	0.21995	0.67308	0.75909	-0.39767	3.29E-05	0.000489
1124.815	12	oolomics Po	0.18895	0.99951	1.0544	0.076407	0.035266	0.99998	0.87384	-0.19456	0.69167	0.99941	0.98641	-0.01975	0.73478	0.99905	0.9894	-0.01538	0.69008	0.99688
663.8913	3.13	oolomics Po	0.18904	0.99951	1.4012	0.48667	0.022916	0.99998	0.7361	-0.44202	0.37727	0.99941	0.92663	-0.10993	0.003235	0.17363	0.59419	-0.751	0.000374	0.004605
1163.251	4.35	domics Pos	0.18949	0.99987	1.153	0.20536	0.9058	0.99994	0.99359	-0.00927	0.65698	0.99996	0.95553	-0.06563	0.068787	0.44682	0.70367	-0.50702	0.000581	0.00369
567.3082	2.98	oolomics Po	0.18959	0.99951	1.376	0.46049	0.04094	0.99998	0.80632	-0.31057	0.90388	0.99941	0.99334	-0.00964	0.006392	0.1948	0.61325	-0.70546	0.003723	0.02393
738.3223	2.44	olomics Ne	0.18993	0.99918	0.69287	-0.52934	0.94374	0.9934	1.0746	0.10385	0.31166	0.99995	1.409	0.49472	0.3344	0.99985	1.4292	0.51525	0.044448	0.99927
898.5752	4.75	domics Pos	0.19036	0.99987	1.251	0.32307	0.83783	0.99994	0.97976	-0.0295	0.21726	0.99996	1.161	0.21538	0.098292	0.51838	0.81351	-0.29776	0.00251	0.010684
178.1676	6.38	oolomics Po	0.1905	0.99951	1.1254	0.17038	0.41977	0.99998	0.91137	-0.13389	0.32047	0.99941	1.1189	0.16202	0.13539	0.51446	0.83884	-0.25353	2.1E-06	0.000231
901.5699	5.31	domics Pos	0.19054	0.99987	0.83799	-0.25499	0.056767	0.99994	0.7742	-0.36922	0.27709	0.99996	0.82728	-0.27356	0.2704	0.73104	0.89123	-0.16613	0.007737	0.025257
1003.726	9.43	oolomics Po	0.19087	0.99951	1.6037	0.68144	0.71308	0.99998	0.95256	-0.07012	0.35699	0.99941	1.1697	0.22612	0.052411	0.29933	0.75672	-0.40217	0.012295	0.056873
545.4029	6.56	oolomics Po	0.19096	0.99951	0.88125	-0.18238	0.36753	0.99998	0.90215	-0.14856	0.18263	0.99941	0.91274	-0.13173	0.19822	0.63454	0.85348	-0.22858	0.27875	0.63693
550.6287	10.06	domics Pos	0.19099	0.99987	1.0817	0.11336	0.25069	0.99994	0.95052	-0.07321	0.67861	0.99996	0.97423	-0.03767	0.8604	0.9874	1.0107	0.015318	0.000908	0.00519
962.6138	8.89	oolomics Po	0.19105	0.99951	1.3746	0.45896	0.59435	0.99998	0.94954	-0.0747	0.61454	0.99941	1.0702	0.097849	0.004781	0.18788	0.74445	-0.42575	0.036321	0.13246
358.1594	2.18	domics Pos	0.19125	0.99987	0.77987	-0.3587	0.16753	0.99994	0.83452	-0.26098	0.013359	0.99996	0.68176	-0.55266	0.082331	0.48446	0.76026	-0.39544	0.000204	0.001718
941.8098	10.02	domics Pos	0.19132	0.99987	1.1807	0.23969	0.36518	0.99994	0.91085	-0.13472	0.59011	0.99996	0.94401	-0.08313	0.263	0.72224	0.89304	-0.1632	0.034965	0.079543
167.0714	4.38	oolomics Po	0.19134	0.99951	0.87285	-0.1962	0.10544	0.99998	0.81965	-0.28692	0.32161	0.99941	0.88207	-0.18103	0.46882	0.94814	0.92687	-0.10956	0.32878	0.70742
286.9773	3.65	olomics Ne	0.19153	0.99918	1.0598	0.083809	0.072318	0.50118	0.91498	-0.12818	0.62311	0.99995	1.021	0.030027	0.23988	0.99985	1.0399	0.056496	0.86402	0.99927
321.7678	6.38	oolomics Po	0.19154	0.99951	1.2014	0.26468	0.80411	0.99998	0.95051	-0.07323	0.59865	0.99941	1.1156	0.15785	0.87987	0.99905	1.0543	0.076341	9.82E-05	0.001578
684.5801	4.84	domics Pos	0.19187	0.99987	1.1024	0.14062	0.11736	0.99994	0.87836	-0.18711	0.57078	0.99996	0.95703	-0.06337	0.79121	0.97671	0.98219	-0.02592	0.18597	0.27764
354.98	4.46	olomics Ne	0.19204	0.99918	1.0397	0.056162	0.037236	0.45581	0.89633	-0.1579	0.36988	0.99995	1.0288	0.040967	0.71951	0.99985	1.0191	0.027362	0.8126	0.99927
984.6639	9.11	oolomics Po	0.19221	0.99951	1.2018	0.26523	0.51151	0.99998	0.90069	-0.1509	0.28311	0.99941	1.1885	0.24908	0.23812	0.69774	0.81522	-0.29475	0.021014	0.087559
669.3128	0.58	domics Pos	0.19228	0.99987	1.5815	0.66128	0.35179	0.99994	0.86576	-0.20796	0.47429	0.99996	1.0752	0.1046	0.048394	0.3779	0.68419	-0.54754	0.000562	0.003609
1102.806	11.95	oolomics Po	0.19247	0.99951	0.87513	-0.19242	0.819	0.99998	0.97755	-0.03276	0.21262	0.99941	0.87941	-0.18539	0.56898	0.99905	0.94772	-0.07747	0.59203	0.96412
581.966	0.53	domics Pos	0.19284	0.99987	1.4627	0.54866	0.006716	0.99994	0.78298	-0.35296	0.25089	0.99996	0.89433	-0.16112	0.008697	0.19971	0.63281	-0.66015	0.000918	0.005233
272.9451	10.14	domics Pos	0.19313	0.99987	1.0595	0.083411	0.31676	0.99994	0.95495	-0.06651	0.71719	0.99996	0.9857	-0.02078	0.070683	0.45373	0.91732	-0.12451	0.000171	0.001518
641.3277	0.54	domics Pos	0.1935	0.99987	1.4314	0.51743	0.48576	0.99994	0.88079	-0.18314	0.4617	0.99996	0.88365	-0.17844	0.019244	0.25595	0.5485	-0.86642	0.002484	0.010594
517.9782	12.01	olomics Ne	0.19365	0.99918	1.2165	0.28273	0.84461	0.98874	0.99754	-0.00355	0.21332	0.99995	1.1741	0.23152	0.716	0.99985	0.97322	-0.03917	0.9724	0.99927
1006.177	9.42	oolomics Po	0.19376	0.99951	1.3375	0.41952	0.23716	0.99998	0.83289	-0.2638	0.92019	0.99941	1.0104	0.014983	0.028189	0.24036	0.72993	-0.45417	0.018643	0.079465
200.953	10.03	domics Pos	0.1939	0.99987	1.0601	0.084184	0.96229	0.99994	1.0067	0.009573	0.87981	0.99996	0.99213	-0.01139	0.30506	0.76274	0.92255	-0.11631	0.008363	0.026819
785.4165	2.86	oolomics Po	0.19398	0.99951	1.3981	0.48351	0.058986	0.99998	0.82805	-0.27221	0.25727	0.99941	1.0858	0.1188	0.007756	0.19545	0.60132	-0.73379	0.000619	0.006454
566.5606	2.05	domics Pos	0.19409	0.99987	1.2624	0.33612	0.1736	0.99994	0.74597	-0.42281	0.54657	0.99996	1.0886	0.12246	0.20736	0.65238	0.70902	-0.49611	0.000507	0.003333
381.9792	11.98	olomics Ne	0.19443	0.99918	1.1255	0.17058	0.048111	0.47152	0.88787	-0.17158	0.60431	0.99995	1.0617	0.08636	0.59863	0.99985	0.96636	-0.04937	0.99995	0.99995
433.9008	2.05	domics Pos	0.19444	0.99987	1.4017	0.48718	0.89282	0.99994	0.99356	-0.00932	0.31607	0.99996	1.2036	0.26737	0.76539	0.97526	0.92623	-0.11056	3.33E-05	0.000489
959.6698	9.14	oolomics Po	0.19473	0.99951	1.2111	0.27628	0.64838	0.99998	0.94846	-0.07635	0.74945	0.99941	1.0528	0.074182	0.3061	0.79639	0.82428	-0.2788	0.000718	0.007184
342.8707	0.56	domics Pos	0.19512	0.99987	1.0928	0.12808	0.63593	0.99994	1.0216	0.030882	0.87488	0.99996	0.98869	-0.01642	0.2329	0.69081	0.93197	-0.10165	0.018929	0.050018
458.2384	5.98	olomics Ne	0.19533	0.99918	1.2158	0.28192	0.3261	0.74862	0.8633	-0.21206	0.64152	0.99995	0.92781	-0.1081	0.20587	0.99985	0.7837	-0.35164	4.61E-05	0.01419
697.9038	3.16	oolomics Po	0.19563	0.99951	1.3232	0.404	0.039331	0.99998	0.82541	-0.27682	0.77453	0.99941	1.0146	0.020853	0.013686	0.20085	0.58894	-0.76381	0.003622	0.023382
862.4595	0.54	domics Pos	0.19598	0.99987	1.5777	0.65782	0.2081	0.99994	0.81844	-0.28906	0.341	0.99996	0.87824	-0.1873	0.037481	0.34165	0.54689	-0.87069	0.000729	0.004376
329.0405	0.6	domics Pos	0.19648	0.99987	0.70774	-0.49871	0.054485	0.99994	0.5836	-0.77695	0.039547	0.99996	0.57721	-0.79283	0.07339	0.45875	0.59716	-0.74381	0.02718	0.065766
354.8956	10.07	domics Pos	0.19651	0.99987	1.0478	0.06739	0.8631	0.99994	0.99291	-0.01026	0.29523	0.99996	0.96328	-0.05398	0.062378	0.43187	0.93599	-0.09543	0.000319	0.002326
1139.225	4.12	domics Pos	0.19659	0.99987	1.44	0.5261	0.74225	0.99994	0.94383	-0.0834	0.49243	0.99996	1.0886	0.12241	0.013941	0.22898	0.60159	-0.73315	0.00936	0.029351

633.8477	0.51068	-0.96951	0.000885	0.042705	0.63689	-0.65089	0.65369	0.99391	0.95326	-0.06906	0.68257	0.99956	0.97082	-0.04272	0.029074	0.2688	1.7453	0.8035
123.0136	1.1878	0.24829	0.6456	0.92263	1.0334	0.047447	0.83112	0.95121	1.0137	0.01969	0.50812	0.99512	1.035	0.049653	0.67917	0.99941	1.0188	0.026888
596.3036	0.84802	-0.23783	0.32126	0.5977	1.1536	0.20614	0.040871	0.38103	1.4837	0.56919	0.16081	0.91806	1.241	0.31147	0.005521	0.08815	1.9116	0.93481
749.3118	1.5064	0.59115	0.1534	0.62737	1.4709	0.55667	0.1678	0.5427	1.4631	0.54906	0.047471	0.99512	1.6619	0.73284	0.60338	0.99941	0.87933	-0.18552
363.2174	0.84411	-0.24449	0.21238	0.49226	0.62669	-0.67417	0.043143	0.41783	0.4655	-1.1032	0.28567	0.99956	1.2248	0.29256	0.79229	0.91318	0.97493	-0.03663
1008.667	0.63176	-0.66254	0.22672	0.51305	0.80416	-0.31444	0.98196	0.99744	1.0368	0.052189	0.94054	0.99956	1.1233	0.16776	0.15833	0.41597	1.7949	0.84389
466.5929	0.55012	-0.86218	0.012007	0.083709	0.66431	-0.59006	0.84199	0.9951	0.98738	-0.01833	0.42276	0.99956	0.87853	-0.18684	0.066131	0.30735	1.6176	0.69384
151.1139	0.86834	-0.20366	0.012595	0.085318	0.64449	-0.63376	0.063419	0.51223	0.69348	-0.52808	0.00332	0.47452	0.60727	-0.71959	0.008009	0.2688	0.59242	-0.75531
917.1189	0.61891	-0.6922	0.10848	0.31749	0.70493	-0.50445	0.97854	0.99744	1.0305	0.043366	0.54432	0.99956	0.88589	-0.17481	0.21042	0.47719	1.5522	0.63432
193.655	0.28106	-1.831	0.02593	0.14226	0.70159	-0.5113	0.46159	0.80647	0.91305	-0.13123	0.44638	0.96776	1.0785	0.10903	0.015471	0.09885	1.3292	0.41053
667.2984	0.85683	-0.22292	0.090471	0.31714	1.135	0.18266	0.094187	0.52505	1.1527	0.20502	0.041305	0.64308	1.2318	0.30074	0.010139	0.094875	1.5422	0.62499
486.972	1.0442	0.062403	0.069785	0.51869	1.1016	0.13956	0.006361	0.2539	1.1265	0.17179	0.50193	0.99512	1.0405	0.057255	0.20049	0.99941	1.0699	0.097421
414.8677	0.78943	-0.34112	0.94693	0.97163	1.0145	0.020817	0.17079	0.6437	1.1285	0.17444	0.83142	0.97988	1.0521	0.073302	0.076588	0.17807	1.2164	0.28267
594.9732	1.1406	0.18973	0.56065	0.87267	1.1543	0.20699	0.0022	0.22316	1.332	0.41356	0.82018	0.99512	1.0134	0.019252	0.24169	0.99941	1.1273	0.17291
743.9106	0.52769	-0.92224	0.036847	0.1516	0.65105	-0.61915	0.64163	0.99391	0.93686	-0.09409	0.39374	0.99956	0.85992	-0.21772	0.040702	0.28339	1.603	0.68079
471.9787	1.0884	0.12226	0.013077	0.40245	1.3324	0.41401	0.078862	0.43379	1.2921	0.36974	0.52341	0.99512	1.0754	0.10483	0.81588	0.99941	1.0795	0.11036
285.2504	0.55094	-0.86003	0.23679	0.52493	0.88311	-0.17933	0.17305	0.64606	0.86625	-0.20715	0.21879	0.94047	0.8485	-0.23702	0.45989	0.56429	1.1233	0.16772
278.052	0.9036	-0.14624	0.7009	0.91082	1.0464	0.065475	0.18333	0.76087	0.9143	-0.12925	0.77825	0.99956	1.0422	0.059631	0.18694	0.45229	0.83617	-0.25814
362.2044	0.56729	-0.81785	0.062138	0.21533	0.66137	-0.59646	0.92792	0.9951	1.0365	0.051701	0.74512	0.99956	0.98891	-0.01608	0.016784	0.2688	2.1245	1.0871
906.8258	0.79759	-0.32627	0.45001	0.68091	0.93758	-0.09299	0.46139	0.80647	1.0474	0.066874	0.77686	0.97988	1.0126	0.018118	0.006518	0.090084	1.2442	0.31521
975.1487	0.61321	-0.70554	0.14363	0.38429	0.72189	-0.47015	0.42192	0.99035	0.85359	-0.22838	0.28078	0.99956	0.79705	-0.32726	0.46531	0.72741	1.2518	0.32399
808.577	0.77287	-0.3717	0.081409	0.29563	0.81662	-0.29226	0.16191	0.63418	0.865	-0.20923	0.52902	0.96776	0.93499	-0.09698	0.16733	0.2745	1.2116	0.27689
623.9593	0.66829	-0.58146	0.044409	0.1721	0.74631	-0.42215	0.89942	0.9951	1.0683	0.095361	0.87486	0.99956	1.1475	0.19845	0.04832	0.28861	1.9601	0.97095
129.0365	0.73537	-0.44345	0.70443	0.84729	0.96774	-0.04731	0.060508	0.43946	1.1478	0.19886	0.52349	0.96776	1.0552	0.077548	0.070043	0.17098	1.2388	0.30898
472.9768	0.90959	-0.13672	0.56287	0.87267	0.95391	-0.06807	0.15485	0.53641	0.8737	-0.1948	0.16136	0.99512	0.84256	-0.24714	0.2248	0.99941	0.83029	-0.26831
795.6397	0.82765	-0.27291	0.032945	0.16294	0.52936	-0.91767	0.090249	0.51907	0.7741	-0.36941	0.069175	0.76454	0.60509	-0.72478	0.11488	0.21936	0.77322	-0.37106
634.8766	0.76029	-0.39538	0.2631	0.54979	0.9055	-0.14322	0.86318	0.97404	1.0142	0.020298	0.64322	0.97988	0.97912	-0.03044	0.029955	0.1191	1.1878	0.24835
709.9991	0.60716	-0.71986	0.012581	0.085318	0.6934	-0.52825	0.71136	0.99391	0.96101	-0.05738	0.90704	0.99956	1.1075	0.14731	0.083155	0.33309	1.7306	0.79124
565.5576	0.043497	-4.5229	0.01389	0.099586	0.35004	-1.5144	0.23325	0.6761	0.72925	-0.45551	0.4718	0.96776	0.84185	-0.24837	0.12778	0.23279	1.2994	0.3779
181.1593	0.23309	-2.101	0.001476	0.050649	0.38056	-1.3938	0.004894	0.3088	0.52099	-0.94068	0.037706	0.64014	0.59958	-0.73798	0.87527	0.90954	0.99141	-0.01245
476.975	1.0843	0.11681	0.34762	0.75622	1.0942	0.12983	0.011318	0.2716	1.263	0.33681	0.37733	0.99512	0.91136	-0.13391	0.14265	0.99941	1.1702	0.22677
643.4969	0.76896	-0.37903	0.036627	0.15121	0.75675	-0.40211	0.62975	0.99391	1.095	0.13099	0.4491	0.99956	1.2973	0.37546	0.04951	0.28988	1.8651	0.89924
159.9687	0.87192	-0.19773	0.88322	0.93965	1.0515	0.072463	0.86244	0.97404	1.0228	0.032474	0.36425	0.96776	1.0842	0.11662	0.020745	0.10765	1.2757	0.35124
443.865	0.62265	-0.68351	0.032911	0.16294	0.71762	-0.47871	0.32136	0.73067	0.89627	-0.15799	0.27585	0.94365	0.88486	-0.17647	0.22296	0.33092	1.1298	0.17601
734.4854	0.62016	-0.68929	0.029929	0.13218	0.69965	-0.5153	0.68145	0.99391	1.1267	0.17213	0.51049	0.99956	1.1976	0.26021	0.10017	0.35294	1.7675	0.82169
692.8352	0.7789	-0.36049	0.39418	0.64359	0.91226	-0.13248	0.76245	0.94146	1.0157	0.022524	0.75229	0.97988	0.98308	-0.02463	0.016338	0.099614	1.2191	0.28584
768.7605	0.79211	-0.33622	0.4584	0.68734	0.92719	-0.10906	0.33879	0.74343	0.94803	-0.077	0.34461	0.96524	0.95412	-0.06776	0.030215	0.11973	1.2506	0.32261
1042.8	0.76832	-0.38021	0.28907	0.57347	0.91328	-0.13087	0.68288	0.90442	1.0302	0.042949	0.81858	0.97988	1.014	0.020039	0.014089	0.098791	1.2237	0.2912
703.8679	0.79634	-0.32855	0.2248	0.51305	0.86376	-0.21129	0.88286	0.97532	1.019	0.027214	0.38671	0.96776	0.96335	-0.05387	0.074993	0.17705	1.1754	0.23314
318.1672	0.40469	-1.3051	0.003279	0.064159	0.52988	-0.91626	0.56454	0.8563	0.94196	-0.08627	0.80278	0.97988	1.0266	0.037867	0.05934	0.15691	2.5364	1.3428
1111.49	0.96428	-0.05248	0.8476	0.9234	1.0657	0.0918	0.12094	0.57789	1.3531	0.43626	0.022918	0.54215	1.4532	0.53921	0.033528	0.12517	1.5368	0.61989
1135.788	0.52651	-0.92547	0.14901	0.42497	0.71576	-0.48245	0.97804	0.99412	1.0321	0.045526	0.72241	0.97988	1.0666	0.093006	0.1215	0.22569	1.9158	0.93793
567.8136	0.63421	-0.65697	0.000974	0.043099	0.61806	-0.69419	0.54154	0.99391	0.92386	-0.11425	0.4812	0.99956	0.9049	-0.14417	0.033707	0.2688	1.86	0.89534
638.3316	0.88619	-0.17431	0.31696	0.59623	1.0966	0.13298	0.028589	0.35164	1.2969	0.37501	0.15409	0.91321	1.1734	0.23064	0.00209	0.07995	1.6006	0.67862
1183.247	0.52713	-0.92378	0.10763	0.35015	0.79478	-0.33137	0.97408	0.99324	1.0629	0.087975	0.84907	0.97988	1.2029	0.2665	0.039473	0.13319	2.1028	1.0723
807.4672	0.49133	-1.0252	0.012924	0.085844	0.56342	-0.82772	0.56919	0.99391	0.89267	-0.1638	0.51005	0.99956	0.87766	-0.18827	0.055414	0.29791	1.6512	0.72348
722.7867	0.44662	-1.1629	0.065164	0.25748	0.63293	-0.65987	0.17934	0.64777	0.74517	-0.42435	0.99311	0.99721	1.016	0.022917	0.18734	0.29469	1.6529	0.72498
428.2387	0.61207	-0.70822	0.00166	0.045425	0.62751	-0.6723	0.49694	0.99391	0.90679	-0.14116	0.94894	0.99956	1.0459	0.064794	0.018784	0.2688	2.0417	1.0298
168.0736	0.93637	-0.09484	0.013469	0.086062	0.7212	-0.47152	0.16235	0.73696	0.80301	-0.31651	0.030613	0.58535	0.78075	-0.35707	0.026249	0.2688	0.75979	-0.39634
1030.874	0.98329	-0.02432	0.048542	0.48405	1.1353	0.1831	0.42725	0.76668	1.0483	0.068059	0.89807	0.99512	1.0068	0.009774	0.8009	0.99941	1.0259	0.036884
481.2606	0.64831	-0.62524	0.093416	0.28463	0.72687	-0.46023	0.94187	0.9974	1.058	0.081386	0.63482	0.99956	0.96819	-0.04664	0.043215	0.28452	2.1696	1.1174
1176.866	0.67346	-0.57035	0.086665	0.26979	0.75791	-0.3999	0.96841	0.9974	1.0293	0.041682	0.71132	0.99956	1.1956	0.25771	0.041632	0.28426	1.8164	0.86105
433.9028	0.20734	-2.2699	0.005363	0.062645	0.54257	-0.88213	0.17516	0.74875	0.79733	-0.32674	0.33804	0.99956	1.1283	0.17418	0.12616	0.37562	1.1947	0.25668
135.1179	0.24187	-2.0477	0.001548	0.045425	0.55722	-0.84369	0.089424	0.60275	0.7767	-0.36457	0.24075	0.99956	0.86356	-0.21164	0.49821	0.75091	1.0649	0.090708
102.9426	0.91938	-0.12126	0.006426	0.36417	1.2212	0.28825	0.059625	0.40009	0.72154	-0.47084	0.071508	0.99512	1.1467	0.19746	0.31786	0.99941	0.89353	-0.16241
648.9536	0.58365	-0.77682	0.077338	0.24986	0.71805	-0.47785	0.85317	0.9951	1.0766	0.10643	0.88089	0.99956	1.0597	0.083634	0.07414	0.31866	1.6901	0.75711
1077.789	0.67467	-0.56774	0.23748	0.52553	0.83768	-0.25554	0.51318	0.83443	0.91602	-0.12655	0.34737	0.96563	0.89861	-0.15424	0.48279	0.58515	1.0607	0.08498
719.9168	0.50712	-0.97959	0.001228	0.04486	0.60521	-0.7245	0.77657	0.99391	0.98853	-0.01665	0.69919	0.99956	0.97403	-0.03796	0.099873	0.35294	1.511	0.59551
1195.732	0.72383	-0.46627	0.37966	0.63745	1.1206	0.16433	0.18408	0.64793	1.1803	0.23919	0.51734	0.96776	1.0564	0.079124	0.039144	0.13265	1.2075	0.27205
961.6466	0.64319	-0.63668	0.29461	0.59212	0.77839	-0.36143	0.85505	0.9951	0.96695	-0.04848	0.80088	0.99956	0.97942	-0.03001	0.30677	0.58424	1.4002	0.4856
410.8583	0.74586	-0.42303	0.014464	0.10138	0.85002	-0.23444	0.021347	0.33809	0.88223	-0.18077	0.34206	0.96524	0.95841	-0.06129	0.068858	0.17049	1.1591	0.21296
137.1329	0.19612	-2.3502	0.014449	0.10138	0.53617	-0.89924	0.36503	0.76025	0.83573	-0.2589	0.72834	0.97988	0.93066	-0.10367	0.046886	0.14344	1.3743	0.4587
1124.815	0.97447	-0.03731	0.07399	0.24382	0.83353	-0.2627	0.43075	0.99185	1.0575	0.080603	0.96916	0.99956	1.0059	0.008452	0.91432	0.96475	0.99981	-0.00028
663.8913	0.57463	-0.79929	0.002938	0.048961	0.57864	-0.78926	0.49644	0.99391	0.91594	-0.12668	0.52409	0.99956	0.92798	-0.10784	0.014946	0.2688	1.8404	0.88001
1163.251	0.51148	-0.96726	0.019974	0.12102	0.72988	-0.45426	0.95991	0.99032	1.0137	0.019695	0.89425	0.98023	1.0618	0.086467	0.003915	0.085302	1.7484	0.80607
567.3082	0.56194	-0.83152	0.002546	0.046354	0.57856	-0.78946	0.55705	0.99391	0.93662	-0.09447	0.39975	0.99956	0.88645	-0.17389	0.036681	0.27732	1.7343	0.79438
738.3223	1.6218	0.69756	0.15218	0.62446	1.5538	0.63578	0.13403	0.52398	1.5284	0.61203	0.084573	0.99512	1.5925	0.67133	0.26458	0.99941	0.7293	-0.45542
898.5752	0.60935	-0.71467	0.089305	0.31581	0.80357	-0.3155	0.63185	0.8822	1.0792	0.10998	0.45406	0.96776	1.1951	0.25712	0.02718	0.11579	1.7622	0.81742
178.1676	0.26009	-1.9429	0.001899	0.045425	0.55564	-0.84778	0.037919	0.38531	0.7419	-0.4307	0.17675	0.96992	0.87213	-0.19738	0.31041	0.58762	1.1124	0.1537
901.5699	0.72868	-0.45665	0.44169	0.67474	0.88876	-0.17013	0.96227	0.99112	1.0037	0.005272	0.097615	0.84751	0.80577	-0.31156	0.16533	0.27278	0.79237	-0.33575
1003.726	0.64658	-0.62909	0.048859	0.18366	0.72359	-0.46676	0.90905	0.9951	1.134	0.18142	0.82073	0.99956	1.0551	0.077401	0.15592	0.41308	1.7948	0.8438
545.4029	0.90756	-0.13993	0.23212	0.51893	0.92114	-0.11852	0.87568	0.9951	1.0516	0.07257	0.031776	0.58535	0.88646	-0.17387	0.22401	0.49242	0.92022	-0.11995
550.6287	0.71959	-0.47475	0.46528	0.68992	0.94384	-0.08338	0.40684	0.77758	1.0646	0.090277	0.98664	0.99552	1.0022	0.003235	0.056076	0.15328	1.1883	0.24892
962.6138	0.66761	-0.58292	0.073397	0.2427	0.84745	-0.2388	0.69743	0.99391	1.1443	0.19443	0.58495	0.99956	1.0802	0.11128	0.11146	0.36506	1.7739	0.82695
358.1594	0.1741	-2.522	0.009231	0.086818	0.54005	-0.88883	0.054262	0.41523	0.67116	-0.57528	0.041328	0.64308	0.62849	-0.67003	0.20714	0.31606	0.75668	-0.40224
941.8098	0.73937	-0.43563	0.71876	0.85308	0.9574	-0.06281	0.86141	0.97389	1.0156	0.022338	0.5991	0.97073	1.0898	0.12401	0.08743	0.18803	1.2597	0.33313
167.0714	0.90284	-0.14746	0.024958	0.11787	0.73123	-0.45161	0.19508	0.77669	0.82069	-0.28508	0.044311	0.65531	0.79893	-0.32386	0.031949	0.2688	0.77362	-0.3703
286.9773	0.99235	-0.01108	0.16513	0.63921	1.095	0.13087	0.024802	0.33228	1.0916	0.12644	0.5972	0.99512	1.0195	0.027916	0.62333	0.99941	1.0323	0.045927
321.7678	0.21045	-2.2484	0.079449	0.25459	0.66718	-0.58385	0.66674	0.99391	0.92686	-0.10958	0.6461	0.99956	1.0541	0.075947	0.09919	0.35294	1.2859	0.36276
684.5801	0.89534	-0.15949	0.044634	0.20046	1.1738	0.2312	0.086054	0.51087	1.1593	0.21324	0.11834	0.88537	1.2281	0.29643	0.092626	0.19279	1.3374	0.41945
354.98	1.0075	0.010795	0.13346	0.6057	1.0985	0.13548	0.063087	0.40269	1.0646	0.090289	0.38013	0.99512	1.0424	0.059884	0.38679	0.99941	1.0252	0.035838
984.6639	0.69268	-0.52974	0.24544	0.53588	0.77827	-0.36166	0.74507	0.99391	0.95976	-0.05926	0.63521	0.99956	0.95209	-0.07083	0.31329	0.59141	1.3532	0.43641
669.3128	0.44996	-1.1521	0.018007	0.1154	0.6489	-0.62392	0.89557	0.97532	1.0494	0.06951	0.64143	0.97988	1.2079	0.27245	0.020113	0.10652	2.4571	1.2969
1102.806	0.95364	-0.06849	0.41305	0.72073	0.91611	-0.1264	0.36442	0.96545	0.92901	-0.10623	0.044856	0.65719	0.77096	-0.37527	0.32637	0.60309	0.89752	-0.15599
581.966	0.41756	-1.2599	0.002938	0.062584	0.64282	-0.63751	0.58978	0.86163	0.94648	-0.07936	0.51357	0.96776	0.9291	-0.10609	0.025143	0.11206	1.9976	0.99824
272.9451	0.79947	-0.32289	0.2431	0.53136	0.91061	-0.1351	0.70438	0.91485	1.017	0.024258	0.72613	0.97988	1.022	0.031335	0.015158	0.09885	1.1977	0.26024
641.3277	0.4336	-1.2056	0.004049	0.069859	0.52347	-0.93383	0.30708	0.72831	0.80739	-0.30867	0.5945	0.97073	0.91961	-0.1209	0.014519	0.098791	1.9915	0.99385
517.9782	1.0029	0.004243	0.68452	0.93502	1.0967	0.13322	0.003713	0.22316	1.4715	0.55731	0.55889	0.99512	1.1771	0.23522	0.72616	0.99941	1.0864	0.11951
1006.177	0.61881	-0.69243	0.016777	0.09591	0.63322	-0.65921	0.45274	0.99391	0.88055	-0.18353	0.81233	0.99956	0.98619	-0.02006	0.070823	0.31396	1.5744	0.65476
200.953	0.83415	-0.26162	0.2225	0.51214	0.88529	-0.17578	0.76808	0.94339	0.9833	-0.02429	0.26304	0.94365	1.0575	0.080678	0.018241	0.1032	1.2729	0.34811
785.4165	0.58286	-0.77879	0.010281	0.080315	0.69416	-0.52666	0.94927	0.9974	1.1239	0.16856	0.95946	0.99956	1.1419	0.19149	0.026026	0.2688	2.5941	1.3752
566.5606	0.12916	-2.9527	0.01834	0.11616	0.45418	-1.1387	0.34151	0.74472	0.7977	-0.32608	0.51858	0.96776	0.86985	-0.20115	0.14391	0.24827	1.2648	0.33887
381.9792	1.0021	0.003032	0.69532	0.9375	1.0209	0.029867	0.65752	0.87948	1.0428	0.060445	0.51881	0.99512	0.95534	-0.06591	0.71911	0.99941	0.9748	-0.03682
433.9008	0.094127	-3.4092	0.082602	0.29844	0.56562	-0.82209	0.77133	0.94358	0.94098	-0.08776	0.41407	0.96776	1.1167	0.15923	0.017141	0.10009	1.7796	0.83152
959.6698	0.61001	-0.71311	0.028896	0.12915	0.69045	-0.53439	0.62005	0.99391	0.93857	-0.09146	0.47951	0.99956	0.89527	-0.1596	0.61635	0.81955	1.2952	0.37315
342.8707	0.81972	-0.28679	0.10709	0.34962	0.90286	-0.14743	0.48136	0.81375	0.95927	-0.06	0.84099	0.97988	1.0142	0.020286	0.043707	0.13906	1.2171	0.28346
458.2384	0.22418	-2.1573	0.050333	0.48406	0.61504	-0.70126	0.44727	0.78006	0.87853	-0.18684	0.73155	0.99512	0.94857	-0.07618	0.039595	0.99941	1.4213	0.5072
697.9038	0.59747	-0.74306	0.000267	0.039399	0.58918	-0.76322	0.46764	0.99391	0.92207	-0.11704	0.53258	0.99956	0.93117	-0.10288	0.054425	0.29746	1.7262	0.78757
862.4595	0.35078	-1.5113	0.013791	0.099356	0.57017	-0.81053	0.70366	0.91478	0.9431	-0.08451	0.42024	0.96776	0.86414	-0.21066	0.044533	0.14047	1.861	0.89609
329.0405	0.49244	-1.022	0.006412	0.079158	0.46201	-1.114	0.10601	0.55295	0.63252	-0.66082	0.041265	0.64308	0.53775	-0.89498	0.18076	0.28803	0.6865	-0.54267
354.8956	0.79135	-0.3376	0.13422	0.40045	0.88527	-0.1758	0.75468	0.93732	1.0199	0.028422	0.80015	0.97988	0.98917	-0.0157	0.016169	0.099614	1.1975	0.26
1139.225	0.54477	-0.87629	0.059012	0.24091	0.70656	-0.50113	0.6387	0.88463	0.92132	-0.11822	0.72412	0.97988	1.2064	0.27069	0.11544	0.2202	1.7655	0.82007

807.9708	12.04	olomics Ne	0.19677	0.99918	1.165	0.22033	0.95846	0.99363	1.0483	0.067987	0.76339	0.99995	1.0341	0.048341	0.8553	0.99985	1.0292	0.041562	0.6001	0.99927	0.93392	-0.09863	0.046005	0.48405	1.4777	0.56337	0.99563	0.99691	1.0043	0.006208	0.41772	0.99512	1.0868	0.12003	0.55789	0.99941	1.0855	0.11834
794.5931	5.19	domics Pos	0.19685	0.99987	1.0617	0.086345	0.69815	0.99994	0.98292	-0.02486	0.48463	0.99996	0.96436	-0.05236	0.17569	0.60979	0.9171	-0.12485	0.004815	0.017721	0.7842	-0.35071	0.097712	0.33147	0.90306	-0.1471	0.40599	0.77758	1.0552	0.07746	0.49152	0.96776	0.96661	-0.04899	0.46284	0.566	1.0391	0.055358
1037.786	10.02	domics Pos	0.19694	0.99987	1.1557	0.20881	0.97732	0.99994	0.99743	-0.00371	0.47638	0.99996	1.0685	0.095604	0.6777	0.95843	0.96534	-0.05089	0.011353	0.033838	0.77649	-0.36496	0.53074	0.7367	0.94879	-0.07584	0.18604	0.64793	1.1513	0.20322	0.44705	0.96776	1.051	0.071779	0.19237	0.30063	1.1751	0.23278
839.8576	0.5	domics Pos	0.19701	0.99987	1.154	0.20666	0.29993	0.99994	0.95237	-0.07041	0.13438	0.99996	0.94156	-0.08687	0.017205	0.24478	0.83195	-0.26543	9.18E-05	0.000972	0.67748	-0.56175	0.091729	0.31942	0.84732	-0.23902	0.26137	0.69644	0.95351	-0.06868	0.71673	0.97988	0.97606	-0.03496	0.00983	0.094875	1.3701	0.45432
656.5471	4.84	domics Pos	0.19705	0.99987	0.90027	-0.15157	0.23619	0.99994	0.87084	-0.19952	0.038341	0.99996	0.81712	-0.29137	0.04979	0.3797	0.86925	-0.20215	0.13509	0.21826	0.84325	-0.24597	0.076889	0.28743	1.1884	0.24905	0.010426	0.3324	1.236	0.30569	0.50564	0.96776	1.1004	0.13797	0.23427	0.34159	1.0946	0.13046
83.0868	6.99	olomics Pc	0.19708	0.99951	1.2465	0.31789	0.74761	0.99998	0.94172	-0.08664	0.2754	0.99941	0.83603	-0.25838	0.074812	0.35544	0.61824	-0.69375	0.003453	0.022567	0.42259	-1.2427	0.00104	0.043099	0.43588	-1.198	0.003075	0.20152	0.49817	-1.0053	0.075269	0.77396	0.65863	-0.60246	0.32336	0.60109	0.84018	-0.25123
663.339	0.54	domics Pos	0.1971	0.99987	1.4774	0.56302	0.18746	0.99994	0.81587	-0.2936	0.38505	0.99996	0.89282	-0.16356	0.020553	0.26036	0.547	-0.87039	0.001693	0.007969	0.42733	-1.2266	0.000314	0.028292	0.51586	-0.95496	0.2094	0.65208	0.78127	-0.3561	0.38376	0.96776	0.87639	-0.19036	0.005005	0.087539	1.9981	0.99866
734.9951	9.44	olomics Pc	0.19721	0.99951	1.3386	0.42073	0.38054	0.99998	0.88271	-0.17999	0.52249	0.99941	1.0634	0.088657	0.013682	0.20085	0.65602	-0.60818	0.01118	0.053119	0.61763	-0.69518	0.033345	0.1422	0.68403	-0.54788	0.90082	0.9951	1.0039	0.005559	0.76286	0.99956	0.98694	-0.01897	0.082815	0.33309	1.6861	0.75367
696.4372	2.84	olomics Pc	0.19754	0.99951	1.4646	0.55056	0.26872	0.99998	0.86956	-0.20164	0.65395	0.99941	0.93986	-0.08948	0.027839	0.24036	0.59323	-0.75334	0.004673	0.028254	0.52877	-0.9193	0.011078	0.083281	0.60931	-0.71475	0.73428	0.99391	0.9862	-0.02005	0.96325	0.99956	1.1187	0.16178	0.028538	0.2688	2.4264	1.2788
721.898	3.2	olomics Pc	0.19824	0.99951	1.3863	0.47119	0.042292	0.99998	0.81034	-0.3034	0.39083	0.99941	0.92495	-0.11256	0.00621	0.1948	0.57713	-0.79302	0.004829	0.028808	0.50086	-0.99752	0.001976	0.045425	0.60283	-0.73018	0.59994	0.99391	0.94213	-0.086	0.75224	0.99956	0.9947	-0.00766	0.055516	0.29791	1.6471	0.71991
741.3432	2.43	olomics Pc	0.1983	0.99951	0.69426	-0.52645	0.92274	0.99998	1.1071	0.14678	0.29004	0.99941	1.3783	0.46287	0.33382	0.82797	1.4654	0.55129	0.082331	0.24562	1.5388	0.62179	0.17514	0.43751	1.4522	0.53826	0.15404	0.72748	1.509	0.59361	0.039982	0.6387	1.6447	0.71782	0.3097	0.58762	0.75609	-0.40338
1136.871	9.7	olomics Pc	0.19908	0.99951	1.5492	0.63155	0.94986	0.99998	0.96855	-0.0461	0.82849	0.99941	1.0037	0.00526	0.15753	0.55851	0.72416	-0.46562	0.022315	0.091765	0.55436	-0.85112	0.16908	0.42628	0.73597	-0.44229	0.76158	0.99391	0.93917	-0.09054	0.68387	0.99956	1.2385	0.30857	0.055054	0.29746	1.945	0.95975
324.771	0.34	olomics Ne	0.1992	0.99918	0.92961	-0.1053	0.00097	0.34605	0.87759	-0.18838	0.88513	0.99995	1.0179	0.025578	0.37535	0.99985	0.94197	-0.08624	0.97026	0.99927	1.005	0.007138	0.049501	0.48405	0.93919	-0.09052	0.58127	0.85694	0.9843	-0.02283	0.033922	0.99512	0.9319	-0.10175	0.4534	0.99941	0.97385	-0.03823
416.868	10.16	domics Pos	0.19934	0.99987	0.87071	-0.19974	0.069533	0.99994	0.80436	-0.31409	0.049961	0.99996	0.8211	-0.28437	0.10436	0.52894	0.81684	-0.29188	0.006181	0.021367	0.71905	-0.47584	0.31232	0.59101	0.87113	-0.19904	0.23993	0.67869	0.89355	-0.16239	0.1287	0.88537	0.85601	-0.2243	0.42481	0.53174	1.0803	0.11148
694.4232	3.16	olomics Pc	0.19941	0.99951	1.4121	0.49783	0.14946	0.99998	0.84802	-0.23782	0.99251	0.99941	0.99881	-0.00171	0.012011	0.20055	0.56549	-0.82242	0.000252	0.003388	0.55064	-0.86082	0.00975	0.079214	0.58359	-0.77698	0.68678	0.99391	0.9568	-0.06372	0.66586	0.99956	0.9592	-0.0601	0.029418	0.2688	1.6764	0.74537
1002.692	9.09	olomics Pc	0.19943	0.99951	1.2413	0.31187	0.65852	0.99998	0.9381	-0.09219	0.97107	0.99941	1.0062	0.008909	0.31235	0.80657	0.82778	-0.27268	0.014974	0.066338	0.6098	-0.7136	0.084552	0.26634	0.73986	-0.43468	0.66327	0.99391	1.0939	0.12951	0.87002	0.99956	1.0156	0.022275	0.54113	0.7778	1.2443	0.31529
600.3354	3.05	olomics Pc	0.19954	0.99951	1.3712	0.45544	0.26494	0.99998	0.87979	-0.18476	0.75099	0.99941	0.96998	-0.04398	0.009911	0.19702	0.63416	-0.65709	0.000565	0.006154	0.58559	-0.77204	0.022757	0.11149	0.64331	-0.63642	0.77244	0.99391	1.0081	0.0116	0.93484	0.99956	1.0379	0.053679	0.035011	0.272	1.9146	0.93706
182.1579	2.05	domics Pos	0.19963	0.99987	1.2114	0.27664	0.097714	0.99994	0.91889	-0.12204	0.18201	0.99996	1.0924	0.12756	0.096703	0.51634	0.8132	-0.29833	8.64E-08	0.000144	0.20972	-2.2535	0.006031	0.077417	0.57382	-0.80133	0.66357	0.89692	0.96338	-0.05383	0.91755	0.98252	1.0019	0.002724	0.002256	0.082646	1.4696	0.55538
81.0707	1.66	domics Pos	0.20012	0.99987	1.1965	0.25879	0.2917	0.99994	0.84736	-0.23895	0.96124	0.99996	1.0018	0.002571	0.19037	0.63224	0.76551	-0.3855	1.29E-05	0.000277	0.17993	-2.4745	0.004407	0.071311	0.50104	-0.99701	0.18258	0.64793	0.75573	-0.40406	0.52131	0.96776	0.89626	-0.15801	0.043727	0.13906	1.3655	0.4494
757.3644	0.57	domics Pos	0.20012	0.99987	1.6544	0.72634	0.47997	0.99994	0.90946	-0.13692	0.98763	0.99996	0.99388	-0.00886	0.054662	0.40237	0.64697	-0.62823	0.00095	0.005375	0.49036	-1.0281	0.015228	0.10398	0.68079	-0.55472	0.61344	0.87102	1.1286	0.17455	0.8254	0.97988	1.1741	0.23159	0.012254	0.098791	2.4669	1.3027
212.2018	5.98	olomics Pc	0.20026	0.99951	1.1564	0.20963	0.31716	0.99998	0.87642	-0.19031	0.35682	0.99941	1.1164	0.15887	0.38945	0.88728	0.87385	-0.19454	4.64E-06	0.00024	0.22153	-2.1744	0.033858	0.14271	0.64993	-0.62164	0.21002	0.80197	0.83696	-0.25677	0.85602	0.99956	0.98505	-0.02174	0.035501	0.27409	1.2165	0.28271
514.2761	3.09	olomics Pc	0.20027	0.99951	1.4592	0.54516	0.41481	0.99998	0.90848	-0.13848	0.91824	0.99941	1.0079	0.011388	0.008958	0.19702	0.6565	-0.60714	0.000705	0.007113	0.58737	-0.76765	0.046104	0.17603	0.69953	-0.51555	0.76888	0.99391	0.97425	-0.03763	0.67289	0.99956	0.98474	-0.02219	0.032745	0.2688	1.9928	0.99477
446.8883	10.1	domics Pos	0.20082	0.99987	1.0963	0.13259	0.85902	0.99994	0.98462	-0.02236	0.80476	0.99996	0.97847	-0.0314	0.26868	0.72969	0.90109	-0.15025	0.009442	0.029511	0.78361	-0.3518	0.78294	0.88816	0.97755	-0.03276	0.49077	0.82123	1.0616	0.086281	0.92692	0.98573	0.9906	-0.01362	0.076341	0.17807	1.1826	0.24201
1027.183	9.07	olomics Pc	0.20106	0.99951	1.2648	0.33892	0.61787	0.99998	0.91258	-0.13198	0.62497	0.99941	1.0634	0.088677	0.31066	0.80356	0.81347	-0.29784	0.034293	0.12747	0.65041	-0.62058	0.25098	0.54095	0.79213	-0.33619	0.58433	0.99391	1.1028	0.14114	0.83844	0.99956	0.97843	-0.03146	0.6073	0.81633	1.2076	0.27215
438.8541	10.13	domics Pos	0.20125	0.99987	1.1095	0.14985	0.76823	0.99994	0.97837	-0.03155	0.12576	0.99996	0.89454	-0.16078	0.47829	0.88482	1.0774	0.10751	0.013436	0.038404	0.79147	-0.33739	0.34304	0.61179	0.90793	-0.13935	0.26625	0.70012	1.1038	0.14243	0.70808	0.97988	1.0515	0.072478	0.092453	0.19265	1.2718	0.34683
420.8858	10.06	domics Pos	0.20162	0.99987	1.0212	0.030286	0.14437	0.99994	0.92907	-0.10615	0.078052	0.99996	0.93794	-0.09244	0.054648	0.40237	0.91274	-0.13173	3.88E-06	0.000145	0.76346	-0.38937	0.23278	0.5203	0.90929	-0.13719	0.85218	0.96938	0.99364	-0.00921	0.37053	0.96776	0.96183	-0.05614	0.002471	0.082646	1.1515	0.20351
99.9699	10.18	domics Pos	0.20174	0.99987	1.109	0.14929	0.070387	0.99994	0.90935	-0.13709	0.88185	0.99996	1.04	0.056621	0.99445	0.99837	1.0016	0.002295	0.000192	0.001647	0.76487	-0.38672	0.83731	0.91918	1.0388	0.054915	0.1745	0.64606	1.1103	0.15089	0.50899	0.96776	1.0475	0.066889	0.000795	0.07528	1.3417	0.42408
845.6011	5.16	domics Pos	0.20207	0.99987	1.1116	0.1526	0.82356	0.99994	0.97492	-0.03664	0.86637	0.99996	1.0059	0.008422	0.85615	0.9874	0.98053	-0.02837	0.037849	0.084944	0.79204	-0.33635	0.23352	0.52074	0.88937	-0.16915	0.5468	0.8511	0.9421	-0.08605	0.78651	0.97988	0.97024	-0.04359	0.59983	0.69203	1.0846	0.11721
907.5409	10.28	olomics Ne	0.20249	0.99918	1.1455	0.19593	0.00568	0.34605	0.77798	-0.36219	0.71165	0.99995	1.0462	0.065111	0.69162	0.99985	1.0599	0.083958	0.27955	0.99927	0.94554	-0.0808	0.48178	0.8481	1.0777	0.10797	0.28291	0.66462	0.89067	-0.16704	0.10087	0.99512	1.1163	0.15872	0.37924	0.99941	1.0495	0.069651
537.9397	0.54	domics Pos	0.20255	0.99987	1.7632	0.81821	0.24743	0.99994	0.8637	-0.2114	0.75883	0.99996	1.0292	0.041472	0.032401	0.3137	0.65761	-0.60469	0.000481	0.00318	0.47159	-1.0844	0.01897	0.118	0.67552	-0.56594	0.89458	0.97532	1.0458	0.064567	0.70487	0.97988	0.99785	-0.0031	0.010599	0.094875	2.3878	1.2557
884.5409	10.48	olomics Ne	0.20282	0.99918	1.1878	0.24823	0.005031	0.34605	0.75936	-0.39715	0.040063	0.99995	1.1722	0.22926	0.17679	0.99985	1.1128	0.15417	0.31427	0.99927	0.93686	-0.0941	0.036494	0.48305	1.1927	0.2542	0.29773	0.67372	1.1364	0.18448	0.047849	0.99512	1.1688	0.22503	0.94108	0.99941	1.0198	0.028298
873.5964	9.21	olomics Pc	0.20356	0.99951	1.3343	0.41609	0.98371	0.99998	0.99649	-0.00507	0.43751	0.99941	1.1192	0.16242	0.16795	0.58009	0.77096	-0.37528	0.023385	0.094928	0.67285	-0.57164	0.097944	0.29524	0.7409	-0.43264	0.86749	0.9951	1.0696	0.097055	0.54372	0.99956	0.91205	-0.13282	0.08373	0.33342	1.5965	0.6749
307.9042	10.16	domics Pos	0.20375	0.99987	1.1129	0.15429	0.83668	0.99994	1.0427	0.060337	0.4344	0.99996	1.0541	0.075992	0.61708	0.93856	1.0307	0.043579	0.04604	0.097855	0.79928	-0.32323	0.8695	0.93287	0.98911	-0.01579	0.13357	0.59988	1.1107	0.15151	0.8434	0.97988	1.0087	0.012439	0.04809	0.14468	1.2254	0.29319
830.5582	4.77	domics Pos	0.20402	0.99987	1.0618	0.086447	0.30969	0.99994	0.94501	-0.08159	0.88782	0.99996	0.99312	-0.00995	0.47092	0.88476	0.95314	-0.06924	0.001113	0.005954	0.76938	-0.37823	0.35249	0.61857	0.91639	-0.12597	0.70793	0.91516	0.97912	-0.03044	0.83854	0.97988	0.99064	-0.01357	0.003164	0.085302	1.1791	0.23771
363.931	0.5	domics Pos	0.20403	0.99987	1.0655	0.091593	0.91903	0.99994	0.99453	-0.00791	0.32567	0.99996	0.93701	-0.09387	0.12463	0.54705	0.89201	-0.16487	0.002765	0.011497	0.75536	-0.40476	0.27398	0.56105	0.8715	-0.19842	0.60569	0.86762	0.96319	-0.0541	0.69903	0.97988	0.97474	-0.03691	0.049912	0.14647	1.2145	0.28041
167.0134	10.24	domics Pos	0.20448	0.99987	1.129	0.17506	0.49032	0.99994	0.94861	-0.07611	0.47625	0.99996	0.95475	-0.0668	0.74718	0.97368	0.97736	-0.03304	0.006411	0.021942	0.77042	-0.37629	0.48174	0.70136	0.93297	-0.10009	0.58694	0.86163	1.0379	0.053615	0.84927	0.97988	1.0156	0.022335	0.046282	0.14289	1.1741	0.23159
986.1506	4.58	domics Pos	0.20467	0.99987	1.5327	0.61604	0.23234	0.99994	0.83746	-0.2559	0.2905	0.99996	1.1114	0.15234	0.034691	0.32742	0.65815	-0.60351	0.002303	0.010045	0.45155	-1.1471	0.011839	0.092554	0.59546	-0.74791	0.95049	0.98957	1.0198	0.028221	0.91423	0.98248	1.1314	0.17812	0.11935	0.22353	2.0324	1.0232
69.0711	6.99	olomics Pc	0.20468	0.99951	1.1782	0.23654	0.73729	0.99998	1.0259	0.036935	0.70364	0.99941	0.95124	-0.07212	0.22898	0.67919	0.77092	-0.37535	0.000748	0.007459	0.44344	-1.1732	0.004291	0.05517	0.55756	-0.84279	0.00337	0.20152	0.59211	-0.75606	0.083607	0.79241	0.70405	-0.50624	0.71074	0.86888	0.95393	-0.06805
100.9583	10.18	domics Pos	0.20474	0.99987	0.93006	-0.1046	0.041212	0.99994	0.84972	-0.23494	0.20549	0.99996	0.87226	-0.19717	0.10491	0.52894	0.8794	-0.18541	0.00034	0.002443	0.67264	-0.5721	0.093149	0.32266	0.80143	-0.31936	0.84796	0.96798	0.99025	-0.01414	0.01062	0.52955	0.84905	-0.23608	0.046775	0.14344	1.1534	0.20587
1139.812	9.64	olomics Pc	0.2051	0.99951	1.4243	0.51022	0.65939	0.99998	0.94261	-0.08526	0.24217	0.99941	1.1808	0.23979	0.062507	0.32853	0.70604	-0.50218	0.012153	0.056545	0.68972	-0.53591	0.059677	0.21074	0.74796	-0.41897	0.96324	0.9974	1.0483	0.068007	0.48782	0.99956	1.2249	0.29266	0.090688	0.34353	1.9405	0.95643
668.4683	9.54	olomics Pc	0.20599	0.99951	1.4061	0.49167	0.22276	0.99998	0.87076	-0.19966	0.42099	0.99941	1.0768	0.10677	0.037004	0.26312	0.70344	-0.50749	0.002553	0.018191	0.55865	-0.83998	0.045107	0.17328	0.72245	-0.46903	0.89829	0.9951	1.0146	0.020951	0.81547	0.99956	1.1405	0.18969	0.09377	0.34791	1.7658	0.82029
168.051	10.16	domics Pos	0.20617	0.99987	1.1749	0.23255	0.75059	0.99994	1.0152	0.021731	0.92158	0.99996	1.0106	0.015225	0.2137	0.66269	0.93095	-0.10323	0.003883	0.014971	0.80909	-0.30562	0.92387	0.96091	1.0038	0.005528	0.94518	0.98814	1.0025	0.003588	0.052513	0.68583	1.1581	0.21176	0.00772	0.091944	1.245	0.31612
441.2542	2.06	domics Pos	0.20639	0.99987	1.1353	0.18308	0.06597	0.99994	0.84388	-0.24488	0.5976	0.99996	1.0368	0.052169	0.15745	0.58646	0.85944	-0.21853	4.91E-07	0.000144	0.22	-2.1844	0.01037	0.08787	0.66271	-0.59354	0.54948	0.85184	0.94151	-0.08695	0.7863	0.97988	1.0235	0.033532	0.002648	0.082646	1.4598	0.54582
983.1526	8.87	olomics Pc	0.20644	0.99951	1.3749	0.45935	0.56042	0.99998	0.92917	-0.10599	0.64875	0.99941	1.0611	0.085528	0.028107	0.24036	0.69272	-0.52965	0.036557	0.13313	0.65024	-0.62095	0.024572	0.11657	0.74235	-0.42983	0.74978	0.99391	1.1045	0.14345	0.66231	0.99956	0.97157	-0.04162	0.032916	0.2688	1.8769	0.90836
919.5972	9.19	olomics Pc	0.20668	0.99951	1.3786	0.46322	0.90262	0.99998	1.0008	0.001108	0.30199	0.99941	1.16	0.21409	0.36806	0.86198	0.81318	-0.29835	0.046064	0.15806	0.62902	-0.66882	0.1406	0.37951	0.70995	-0.4942	0.94573	0.9974	1.0068	0.009762	0.51985	0.99956	0.86893	-0.20269	0.14407	0.39579	1.5774	0.65757
176.0679	0.34	olomics Pc	0.2072	0.99951	0.82515	-0.27728	0.68825	0.99998	0.93625	-0.09504	0.71286	0.99941	1.0417	0.058936	0.044887	0.28536	1.5426	0.6254	0.07421	0.22651	1.3362	0.41813	0.35639	0.66664	1.128	0.17372	0.032293	0.35567	1.4166	0.50246	0.34585	0.99956	0.83874	-0.25371	0.037733	0.28036	0.69705	-0.52066
287.3019	2.66	domics Pos	0.20723	0.99987	1.1666	0.22225	0.099587	0.99994	0.74922	-0.41654	0.41251	0.99996	0.92751	-0.10857	0.00695	0.19397	0.62334	-0.68191	1.64E-06	0.000144	0.11828	-3.0797	0.004658	0.071326	0.45185	-1.1461	0.001403	0.23318	0.55857	-0.84019	0.073297	0.77419	0.8608	-0.21625	0.000514	0.07528	1.5026	0.58746
1157.284	9	olomics Pc	0.20735	0.99951	1.3448	0.42736	0.59069	0.99998	0.94492	-0.08174	0.57192	0.99941	1.1107	0.15145	0.020243	0.22065	0.77377	-0.37002	0.033306	0.12494	0.64955	-0.62249	0.16068	0.41352	0.84568	-0.24181	0.41274	0.98164	1.2215	0.28864	0.24301	0.99956	1.2804	0.35657	0.017591	0.2688	1.889	0.91764
691.4716	9.48	olomics Pc	0.20798	0.99951	1.3206	0.40124	0.3356	0.99998	0.94315	-0.08444	0.16122	0.99941	1.1322	0.17916	0.025063	0.23087	0.66144	-0.59633	0.000567	0.006154	0.66177	-0.59559	0.000949	0.042705	0.73198	-0.45012	0.55333	0.99391	0.94736	-0.07802	0.77401	0.99956	1.1643	0.2195	0.090154	0.34256	1.6276	0.70272
882.7585	7.89	domics Pos	0.20829	0.99987	0.64966	-0.62223	0.3641	0.99994	0.73944	-0.4355	0.17569	0.99996	0.62212	-0.68474	0.1289	0.55024	0.58562	-0.77197	0.074778	0.14136	0.47633	-1.07	0.032258	0.16163	0.4702	-1.0887	0.44171	0.80121	0.77852	-0.36119	0.42588	0.96776	0.7623	-0.39156	0.063696	0.16191	2.4588	1.298
436.9738	4.88	olomics Ne	0.20847	0.99918	1.0512	0.072029	0.10652	0.52291	0.91414	-0.12952	0.59451	0.99995	1.0185	0.026423	0.67284	0.99985	0.97657	-0.0342	0.85746	0.99927	1.0104	0.014936	0.36893	0.775	1.0551	0.077382	0.028869	0.34171	1.1002	0.13774	0.56283	0.99512	1.03	0.042625	0.53094	0.99941	1.0324	0.045956
582.5261	6.37	olomics Pc	0.20899	0.99951	1.2856	0.36245	0.37256	0.99998	0.83275	-0.26404	0.77942	0.99941	1.0849	0.11761	0.20593	0.64353	0.72237	-0.4692	1.95E-05	0.000473	0.091917	-3.4435	0.019312	0.10212	0.36609	-1.4497	0.0913	0.60607	0.61542	-0.70035	0.43857	0.99956	0.85913	-0.21905	0.13114	0.38344	1.2664	0.3407
1014.717	0.54	domics Pos	0.20924	0.99987	1.072	0.10029	0.76909	0.99994	0.98158	-0.02682	0.85089	0.99996	1.016	0.022907	0.082132	0.48443	0.89724	-0.15643	0.008289	0.026671	0.69591	-0.52302	0.052335	0.22542	0.83399	-0.2619	0.09694	0.53334	0.83052	-0.26791	0.024871	0.56108	0.83671	-0.25721	0.018422	0.10376	1.1827	0.24203
665.9766	9.52	olomics Pc	0.20963	0.99951	1.4106	0.49627	0.12742	0.99998	0.82309	-0.28088	0.38457	0.99941	1.1096	0.15003	0.017629	0.20936	0.6487	-0.62437	0.001842	0.014654	0.61063	-0.71163	0.01023	0.080256	0.69666	-0.52147	0.85767	0.9951	1.0779	0.10822	0.84429	0.99956	1.1545	0.20729	0.11064	0.36452	1.7217	0.78381
318.2411	5.03	olomics Pc	0.20976	0.99951	1.2703	0.34515	0.54723	0.99998	0.90663	-0.14141	0.72125	0.99941	1.055	0.077286	0.2141	0.65513	0.79089	-0.33845	7.44E-05	0.00128	0.29933	-1.7402	0.001777	0.045425	0.50005	-0.99986	0.018741	0.30136	0.59519	-0.74857	0.52735	0.99956	0.89775	-0.15562	0.66531	0.84814	1.0437	0.061776
828.8102	10.03	domics Pos	0.21122	0.99987	1.0558	0.078387	0.35371	0.99994	0.94364	-0.08369	0.51085	0.99996	0.95776	-0.06226	0.19876	0.64445	0.93549	-0.09621	0.001022	0.005575	0.77465	-0.36837	0.23973	0.52848	0.89289	-0.16344	0.72661	0.92597	0.97969	-0.0296	0.13715	0.89702	0.92935	-0.1057	0.015847	0.099299	1.2196	0.28641
189.1649	5.99	olomics Pc	0.21124	0.99951	1.1382	0.18671	0.51415	0.99998	0.92238	-0.11657	0.65579	0.99941	1.0559	0.078423	0.13615	0.51671	0.76616	-0.38427	1.46E-05	0.000409	0.25162	-1.9907	0.006546	0.067169	0.58172	-0.78161	0.16874	0.74367	0.80573	-0.31163	0.55447	0.99956	0.93477	-0.09731	0.14748	0.40018	1.1566	0.20987
1138.804	5.41	domics Pos	0.21141	0.99987	1.2778	0.35366	0.30565	0.99994	0.85545	-0.22525	0.56634	0.99996	1.0727	0.10126	0.029851	0.30229	0.67246	-0.57247	0.003594	0.014055	0.54924	-0.86449	0.039357	0.18279	0.67872	-0.55911	0.9719	0.99324	1.0661	0.092321	0.90017	0.98023	1.0837	0.1159	0.079793	0.18143	1.8059	0.85268
622.8531	3.07	olomics Pc	0.21142	0.99951	1.2921	0.36973	0.16632	0.99998	0.85248	-0.23026	0.25979	0.99941	0.86003	-0.21754	0.003432	0.17363	0.59335	-0.75304	0.003311	0.022047	0.55754	-0.84286	0.012968	0.085844	0.65339	-0.61398	0.36402	0.96545	0.85995	-0.21767	0.63286	0.99956	0.95064	-0.07302	0.036213	0.27637	1.6865	0.75405
229.2358	1.34	domics Pos	0.21147	0.99987	1.1887	0.24943	0.37366	0.99994	0.88115	-0.18254	0.58556	0.99996	1.0738	0.10276	0.3013	0.76019	0.81677	-0.292	1.84E-05	0.000345	0.19819	-2.335	0.007531	0.082061	0.57573	-0.79655	0.10347	0.5472	0.72855	-0.4569	0.87166	0.98023	0.97376	-0.03836	0.021647	0.1083	1.4552	0.54125
621.9517	9.57	olomics Pc	0.21155	0.99951	1.317	0.39722	0.085303	0.99998	0.82563	-0.27643	0.13752	0.99941	1.1252	0.17024	0.047237	0.28918	0.7104	-0.49329	0.002335	0.017262	0.64233	-0.63861	0.03946	0.15916	0.61647	-0.6979	0.64765	0.99391	0.95239	-0.07038	0.77127	0.99956	1.119	0.16215	0.32577	0.60292	1.55	0.63226
533.2928	3.28	olomics Pc	0.21169	0.99951	1.2816	0.35791	0.42488	0.99998	0.89082	-0.1668	0.93543	0.99941	0.9822	-0.02591	0.013952	0.20085	0.60082	-0.735	0.017063	0.073832	0.53208	-0.91027	0.003752	0.052849	0.60247	-0.73103	0.49802	0.99391	0.88775	-0.17178	0.58202	0.99956	0.9187	-0.12234	0.017333	0.2688	1.5819	0.66163
709.1169	4.49	domics Pos	0.21184	0.99987	1.378	0.4626	0.32894	0.99994	0.84061	-0.25048	0.59943	0.99996	0.90715	-0.14059	0.072424	0.45752	0.61168	-0.70916	0.001093	0.005877	0.39589	-1.3368	0.047981	0.21249	0.67569	-0.56557	0.89207	0.97532	0.99288	-0.0103	0.81127	0.97988	0.9929	-0.01028	0.070161	0.17116	1.8891	0.91769
264.2728	2.05	domics Pos	0.21216	0.99987	1.2546	0.32725	0.41076	0.99994	1.1485	0.19975	0.25899	0.99996	1.2779	0.35382	0.44255	0.86677	1.1732	0.23046	0.003112	0.012608	0.21513	-2.2167	0.6823	0.83519	0.90038	-0.15139	0.50979	0.83279	1.1711	0.22783	0.041372	0.64308	1.5218	0.60579	0.005228	0.088007	2.0488	1.0348
1005.673	4.56	domics Pos	0.21232	0.99987	1.4348	0.52081	0.42854	0.99994	0.86586	-0.2078	0.75843	0.99996	1.0292	0.041478	0.031085	0.30745	0.59621	-0.74611	0.009371	0.029361	0.48433	-1.0459	0.013947	0.099586	0.57989	-0.78616	0.79449	0.95091	0.95504	-0.06636	0.94957	0.98838	1.0681	0.095107	0.16785	0.27515	1.909	0.93285
979.67	8.88	olomics Pc	0.21247	0.99951	1.3848	0.46966	0.59427	0.99998	0.91772	-0.12387	0.29934	0.99941	1.1929	0.25442	0.068193	0.34279	0.73493	-0.44432	0.03222	0.12199	0.637	-0.65063	0.036611	0.15121	0.6813	-0.55363	0.74692	0.99391	1.0803	0.11137	0.83923	0.99956	1.0843	0.11679	0.042233	0.28426	1.6884	0.75565
270.28	6.55	olomics Pc	0.21267	0.99951	1.1712	0.22803	0.18914	0.99998	0.84841	-0.23717	0.32067	0.99941	0.9205	-0.1195	0.14891	0.54537	0.78603	-0.34734	2.73E-05	0.000587	0.19348	-2.3697	0.013887	0.088017	0.52432	-0.93149	0.044174	0.42295	0.73934	-0.43568	0.28685	0.99956	0.88876	-0.17014	0.014636	0.2688	1.2689	0.34359
315.9797	12	olomics Ne	0.21318	0.99918	1.5116	0.5961	0.18167	0.60009	0.86266	-0.21314	0.55902	0.99995	1.1609	0.21521	0.91348	0.99985	1.0376	0.053219	0.20997	0.99927	1.198	0.26067	0.021744	0.43126	1.2883	0.36547	0.16858	0.5427	1.1805	0.2394	0.87281	0.99512	1.026	0.037048	0.42549	0.99941	1.0793	0.11012
486.9414	3.21	olomics Pc	0.21352	0.99951	1.2593	0.33266	0.26225	0.99998	0.86493	-0.20934	0.67435	0.99941	0.943	-0.08467	0.005325	0.1948	0.63762	-0.64924	0.006853	0.036651	0.58172	-0.78161	0.007542	0.071961	0.57572	-0.79657	0.82974	0.9951	0.9894	-0.01537	0.83299	0.99956	1.0638	0.089247	0.047448	0.28861	1.5144	0.59876
536.9712	5.44	olomics Ne	0.21353	0.99918	1.0665	0.092841	0.2481	0.67419	0.93503	-0.09691	0.54923	0.99995	1.0342	0.048566	0.67406	0.99985	1.022	0.031394	0.53665	0.99927	1.0406	0.057424	0.01744	0.40708	1.1183	0.16132	0.02064	0.30247	1.1045	0.14343	0.16684	0.99512	1.0906	0.1251	0.34435	0.99941	1.0441	0.062299
644.4646	9.54	olomics Pc	0.21357	0.99951	1.3755	0.45996	0.78605	0.99998	1.0314	0.044599	0.49754	0.99941	1.0905	0.12493	0.051382	0.29547	0.70815	-0.49788	0.036332	0.13246	0.71599	-0.48199	0.054051	0.19706	0.76575	-0.38505	0.78839	0.99391	1.1064	0.14586	0.85503	0.99956	1.1027	0.14103	0.054857	0.29746	1.791	0.84079
1133.841	9.63	olomics Pc	0.21359	0.99951	1.4606	0.54654	0.26583	0.99998	0.90554	-0.14315	0.20836	0.99941	1.137	0.18525	0.016277	0.20428	0.69224	-0.53065	0.003065	0.020946	0.59196	-0.75644	0.001853	0.045425	0.56344	-0.82768	0.95586	0.9974	1.031	0.044081	0.80811	0.99956	1.1193	0.16266	0.065666	0.30735	1.7809	0.8326
1181.775	8.99	olomics Pc	0.21415	0.99951	1.3774	0.46193	0.16381	0.99998	0.85114	-0.23253	0.49572	0.99941	1.0981	0.13497	0.10565	0.43684	0.75951	-0.39686	0.035467	0.13082	0.65788	-0.60409	0.14135	0.38061	0.86193	-0.21436	0.54499	0.99391	1.2066	0.27096	0.37129	0.99956	1.196	0.25819	0.021467	0.2688	1.8456	0.88412
395.2296	2.95	olomics Pc	0.21425	0.99951	1.468	0.55384	0.18372	0.99998	0.8523	-0.23057	0.73523	0.99941	0.97205	-0.04089	0.024823	0.23087	0.63113	-0.66398	0.000171	0.002481	0.58426	-0.77532	0.008521	0.075866	0.68729	-0.54101	0.77452	0.99391	1.0086	0.012415	0.946	0.99956	1.1233	0.16779	0.023437	0.2688	2.0216	1.0155
700.8936	3.16	olomics Pc	0.2144	0.99951	1.3704	0.45463	0.88484	0.99998	0.96927	-0.04503	0.73503	0.99941	1.0284	0.040373	0.049213	0.29249	0.73506	-0.44406	0.002572	0.018287	0.51333	-0.96203	0.052575	0.19379	0.69929	-0.51603	0.70121	0.99391	0.9454	-0.081	0.52426	0.99956	0.89345	-0.16255	0.0315	0.2688	1.7528	0.80964
360.1856	2.75	olomics Pc	0.21486	0.99951	1.3432	0.42562	0.2085	0.99998	0.80522	-0.31255	0.84632	0.99941	1.01	0.014363	0.005905	0.1948	0.5745	-0.79962	0.000541	0.006024	0.48791	-1.0353	0.008086	0.074477	0.57661	-0.79434	0.85449	0.9951	1.0482	0.067886	0.73655	0.99956	1.0019	0.002699	0.045059	0.28476	2.0171	1.0122
694.9251	3.16	olomics Pc	0.21507	0.99951	1.3753	0.45971	0.018549	0.99998	0.80408	-0.31458	0.37521	0.99941	0.91606	-0.12648	0.000731	0.12797	0.54968	-0.86334	0.001599	0.013223	0.49056	-1.0275	0.004749	0.059838	0.62242	-0.68404	0.37195	0.97118	0.878	-0.1877	0.62697	0.99956	0.95774	-0.0623	0.033578	0.2688	1.6653	0.73574
707.3652	0.54	domics Pos	0.21511	0.99987	1.5115	0.59597	0.48289	0.99994	0.91343	-0.13063	0.5449	0.99996	0.93596	-0.09548	0.005077	0.17581	0.51789	-0.94928	0.00028	0.002099	0.40153	-1.3164	0.000971	0.043236	0.53099	-0.91323	0.30487	0.7283	0.84489	-0.24316	0.61365	0.97371	0.94647	-0.07937	0.008582	0.092495	1.9547	0.96696
1182.842	9.61	olomics Pc	0.21511	0.99951	1.5357	0.61894	0.92468	0.99998	0.99423	-0.00835	0.33951	0.99941	1.1283	0.1742	0.1091	0.44634	0.72209	-0.46974	0.030448	0.1167	0.63125	-0.66371	0.092094	0.2822	0.73385	-0.44645	0.66614	0.99391	1.1467	0.19747	0.80447	0.99956	1.1939	0.25565	0.071941	0.31665	1.7731	0.82625
921.1073	9.48	olomics Pc	0.2152	0.99951	1.3375	0.41954	0.30033	0.99998	0.88477	-0.17662	0.76064	0.99941	1.0228	0.032588	0.07681	0.36221	0.74206	-0.43039	0.005325	0.030608	0.60888	-0.71578	0.055189	0.19936	0.69566	-0.52355	0.7092	0.99391	1.1353	0.18302	0.94799	0.99956	1.0552	0.077563	0.04333	0.28452	1.69	0.75703
488.8834	0.54	domics Pos	0.21521	0.99987	1.1105	0.15116	0.0932	0.99994	0.90225	-0.14839	0.37128	0.99996	0.9341	-0.09836	0.00326	0.17581	0.7946	-0.3317	0.00028	0.002099	0.67299	-0.57135	0.08391	0.30184	0.78512	-0.34901	0.53118	0.84189	0.94359	-0.08377	0.4887	0.96776	0.93448	-0.09777	0.051982	0.14847	1.4851	0.57057
686.4151	3.15	olomics Pc	0.21593	0.99951	1.3109	0.39055	0.24969	0.99998	0.80784	-0.30785	0.69673	0.99941	1.0289	0.04108	0.032586	0.2515	0.64087	-0.6419	0.012807	0.058636	0.56592	-0.82133	0.021507	0.10949	0.60065	-0.73541	0.76899	0.99391	1.0974	0.1341	0.74023	0.99956	0.96961	-0.04453	0.052757	0.2951	1.7193	0.78183
422.2986	2.05	domics Pos	0.21615	0.99987	1.2634	0.3373	0.71202	0.99994	0.95545	-0.06575	0.97985	0.99996	1.0965	0.13289	0.47528	0.88482	0.87786	-0.18793	0.001625	0.007757	0.28165	-1.828	0.51711	0.72712	0.89984	-0.15226	0.1732	0.64606	1.1736	0.2309	0.307	0.9524	1.1394	0.18831	0.007487	0.091944	1.8827	0.9128
422.3293	2.72	domics Pos	0.21619	0.99987	1.2053	0.2694	0.74567	0.99994	0.97296	-0.03954	0.19251	0.99996	0.86443	-0.21017	0.027032	0.29092	0.73461	-0.44496	0.00387	0.01495	0.56593	-0.8213	0.025385	0.14021	0.54411	-0.87803	0.05095	0.41162	0.61166	-0.70919	0.01228	0.52955	0.55844	-0.84053	0.35614	0.46553	0.87322	-0.19559
238.2176	5.99	olomics Pc	0.21654	0.99951	1.2595	0.3329	0.37064	0.99998	0.89805	-0.15513	0.21534	0.99941	1.1986	0.26129	0.3296	0.82464	0.86916	-0.2023	2.91E-06	0.000231	0.2662	-1.9094	0.026334	0.12181	0.63217	-0.66163	0.38464	0.97696	0.8754	-0.19199	0.82533	0.99956	0.98151	-0.02693	0.042751	0.28452	1.2233	0.29078
575.2926	0.54	domics Pos	0.21659	0.99987	1.9233	0.94359	0.6851	0.99994	0.95597	-0.06496	0.36807	0.99996	0.90352	-0.14638	0.011706	0.21807	0.60424	-0.72681	0.003455	0.013666	0.50401	-0.98846	0.004693	0.071391	0.65577	-0.60873	0.41292	0.78028	0.90757	-0.13992	0.75034	0.97988	1.1635	0.21846	0.001086	0.07528	2.1703	1.1179
201.6445	6.39	olomics Pc	0.21669	0.99951	1.1039	0.14267	0.33044	0.99998	0.93867	-0.09132	0.75945	0.99941	0.97656	-0.03422	0.64882	0.99905	0.96107	-0.05729	0.000109	0.001714	0.39393	-1.344	0.090721	0.27935	0.8044	-0.31401	0.61541	0.99391	0.95853	-0.0611	0.60216	0.99956	1.0244	0.034735	0.037777	0.28036	1.1492	0.20063
620.3755	3.07	olomics Pc	0.21724	0.99951	1.5422	0.62498	0.27523	0.99998	0.91901	-0.12185	0.51282	0.99941	1.0931	0.12837	0.021147	0.22258	0.58087	-0.78371	0.010505	0.050212	0.56183	-0.8318	0.022339	0.1103	0.67327	-0.57075	0.81603	0.99391	1.0006	0.0009	0.82356	0.99956	1.0286	0.040621	0.007217	0.2688	1.8626	0.89735
1098.803	9.74	olomics Pc	0.21738	0.99951	1.5325	0.61593	0.85276	0.99998	1.0055	0.007909	0.49231	0.99941	1.1031	0.14155	0.23789	0.69771	0.74915	-0.41667	0.025679	0.10213	0.58604	-0.77092	0.2309	0.51767	0.73736	-0.43956	0.59145	0.99391	1.1277	0.17333	0.72622	0.99956	1.1933	0.25498	0.076748	0.32186	2.1017	1.0716
659.3701	0.58	domics Pos	0.21746	0.99987	1.3586	0.44216	0.1103	0.99994	0.83121	-0.26672	0.47697	0.99996	0.95502	-0.0664	0.005633	0.18075	0.55601	-0.84681	6.43E-05	0.000767	0.42655	-1.2292	0.000889	0.042199	0.59485	-0.7494	0.72909	0.92637	0.99215	-0.01137	0.80535	0.97988	1.0243	0.034593	0.010023	0.094875	2.4971	1.3202
664.401	3.13	olomics Pc	0.21833	0.99951	1.3236	0.40443	0.078464	0.99998	0.83108	-0.26694	0.33237	0.99941	0.95604	-0.06486	4.04E-05	0.077904	0.56739	-0.81759	0.002121	0.016137	0.59787	-0.7421	0.006375	0.066819	0.64956	-0.62246	0.43136	0.99253	0.90493	-0.14412	0.72152	0.99956	0.99016	-0.01427	0.019882	0.2688	1.741	0.79995
656.364	3.11	olomics Pc	0.21862	0.99951	1.1834	0.24297	0.093095	0.99998	0.84678	-0.23994	0.34914	0.99941	0.90541	-0.14335	0.006975	0.1948	0.57775	-0.79149	0.000966	0.009082	0.53592	-0.8999	0.000335	0.039399	0.58899	-0.76368	0.37603	0.97118	0.89525	-0.15964	0.32915	0.99956	0.86788	-0.20443	0.040731	0.28339	1.5241	0.60794
427.3562	3.52	domics Pos	0.2191	0.99987	0.84212	-0.2479	0.022778	0.99994	0.71292	-0.48819	0.015832	0.99996	0.67704	-0.56269	0.001045	0.17581	0.53631	-0.89886	3.9E-05	0.000541	0.39034	-1.3572	0.006626	0.080462	0.4164	-1.264	0.000964	0.22944	0.49462	-1.0156	0.019152	0.52955	0.54887	-0.86546	0.088767	0.18968	0.65032	-0.62077
143.1265	5.99	olomics Pc	0.21949	0.99951	1.1805	0.23936	0.79038	0.99998	0.97218	-0.04071	0.95116	0.99941	1.0103	0.014747	0.2558	0.72727	0.82391	-0.27944	0.000276	0.003655	0.36275	-1.463	0.002959	0.049058	0.58472	-0.77418	0.16338	0.73838	0.81513	-0.2949	0.94706	0.99956	1.0332	0.047061	0.27591	0.55041	1.1644	0.21963
412.7784	5.41	domics Pos	0.21963	0.99987	1.0808	0.1121	0.0848	0.99994	0.90275	-0.14761	0.40341	0.99996	0.94916	-0.07528	0.43533	0.86544	0.95013	-0.0738	0.002921	0.012017	0.77662	-0.36471	0.36958	0.62855	0.94257	-0.08533	0.47894	0.81159	1.0665	0.092932	0.27927	0.94365	1.0346	0.049095	0.000139	0.07528	1.3268	0.40798
149.133	2.77	domics Pos	0.21974	0.99987	1.1664	0.22202	0.34424	0.99994	0.83063	-0.26772	0.32393	0.99996	0.83641	-0.25771	0.042132	0.357	0.65282	-0.61524	2.96E-05	0.000469	0.19353	-2.3693	0.00272	0.061655	0.42122	-1.2474	0.022766	0.33809	0.58677	-0.76913	0.11085	0.87212	0.72771	-0.45856	0.49524	0.5974	1.128	0.17374
608.3141	1.84	domics Pos	0.21981	0.99987	1.0981	0.135	0.31968	0.99994	0.92061	-0.11934	0.33296	0.99996	1.0729	0.10153	0.61687	0.93856	1.0361	0.05114	0.010607	0.032151	0.74849	-0.41794	0.24318	0.53136	0.93954	-0.08998	0.049451	0.40631	1.242	0.31264	0.12639	0.88537	1.0973	0.13392	0.002792	0.083394	1.3982	0.4836
448.74	2.96	olomics Pc	0.22019	0.99951	1.3879	0.4729	0.086668	0.99998	0.76468	-0.38707	0.59916	0.99941	1.0417	0.058987	0.15626	0.55616	0.74958	-0.41585	0.002213	0.016716	0.57757	-0.79194	0.044601	0.17239	0.70479	-0.50474	0.73255	0.99391	0.97187	-0.04116	0.85494	0.99956	1.0634	0.088652	0.033256	0.2688	2.0853	1.0603
398.891	10.15	domics Pos	0.22028	0.99987	0.90983	-0.13633	0.51737	0.99994	0.95718	-0.06314	0.36735	0.99996	0.93321	-0.09972	0.054801	0.40246	0.8643	-0.21039	0.008361	0.026819	0.80536	-0.31229	0.28894	0.57347	0.87872	-0.18653	0.39184	0.76892	0.94419	-0.08285	0.51654	0.96776	0.95579	-0.06523	0.052984	0.14965	1.1684	0.22457
229.2157	5.48	olomics Pc	0.22114	0.99951	1.1976	0.2602	0.52316	0.99998	0.91835	-0.12289	0.91239	0.99941	0.98295	-0.02481	0.15702	0.55821	0.78627	-0.3469	0.000299	0.003844	0.40346	-1.3095	0.018636	0.10085	0.67094	-0.57574	0.33392	0.94723	0.86976	-0.20131	0.24162	0.99956	0.85752	-0.22176	0.22796	0.496	1.1409	0.19014
299.3142	3.25	domics Pos	0.22117	0.99987	0.88365	-0.17846	0.063179	0.99994	0.73906	-0.43623	0.38825	0.99996	0.90379	-0.14594	0.41387	0.85524	0.92061	-0.11934	0.040288	0.088542	0.77968	-0.35904	0.03174	0.16072	0.70386	-0.50663	0.17025	0.64272	0.74795	-0.41899	0.067666	0.76335	0.8205	-0.28543	0.90014	0.92713	0.98443	-0.02264
875.5718	9.22	olomics Pc	0.22142	0.99951	1.2685	0.34313	0.39504	0.99998	0.90874	-0.13805	0.25802	0.99941	1.1113	0.1522	0.071271	0.34699	0.71868	-0.47657	0.030454	0.1167	0.68578	-0.54419	0.076089	0.24714	0.72697	-0.46003	0.5665	0.99391	0.92275	-0.11599	0.4684	0.99956	0.9076	-0.13987	0.19013	0.45476	1.4964	0.58149

713.3384	0.57	domics Pos	0.22157	0.99987	1.5641	0.64534	0.65534	0.99994	0.92711	-0.10919	0.58513	0.99996	1.0567	0.079556	0.040674	0.34959	0.61714	-0.69633	0.001388	0.006956	0.44318	-1.174	0.023019	0.13299	0.61492	-0.70154	0.91934	0.981	0.99799	-0.00291	0.87862	0.98023	1.1359	0.18378	0.016658	0.099614	2.5232	1.3353
962.6504	9.14	olomics Po	0.22199	0.99951	1.3276	0.40883	0.63499	0.99998	0.89585	-0.15867	0.42811	0.99941	1.1396	0.18855	0.42513	0.9108	0.83122	-0.2667	0.014124	0.063379	0.51577	-0.95521	0.23063	0.51744	0.75037	-0.41432	0.89108	0.9951	1.0386	0.054608	0.66057	0.99956	0.928	-0.10781	0.65199	0.84136	1.2586	0.33179
164.1527	5.99	olomics Po	0.22222	0.99951	1.1257	0.17087	0.41038	0.99998	0.9069	-0.14099	0.66545	0.99941	1.0843	0.11678	0.052892	0.29934	0.75002	-0.41499	1.04E-06	0.000231	0.30843	-1.697	0.028811	0.12915	0.66838	-0.58126	0.15132	0.72698	0.85771	-0.22144	0.051447	0.68379	0.85247	-0.23028	0.076356	0.32186	1.141	0.19024
1048.82	9.78	olomics Po	0.22249	0.99951	1.4839	0.56936	0.34041	0.99998	0.83631	-0.25789	0.67432	0.99941	1.0413	0.058375	0.042778	0.28056	0.63912	-0.64583	0.022139	0.09123	0.5873	-0.76783	0.028028	0.12667	0.60626	-0.72198	0.3939	0.97985	0.83923	-0.25287	0.74486	0.99956	0.98279	-0.02505	0.12372	0.37349	1.9711	0.97902
1002.186	8.87	olomics Po	0.22328	0.99951	1.3297	0.41112	0.5155	0.99998	0.91715	-0.12478	0.81896	0.99941	1.0234	0.033339	0.042574	0.2802	0.72638	-0.4612	0.014412	0.064301	0.62395	-0.68049	0.008712	0.075866	0.62166	-0.6858	0.81389	0.99391	0.97523	-0.03619	0.71352	0.99956	1.1247	0.16956	0.18337	0.44706	1.5758	0.65608
653.8779	3.11	olomics Po	0.22404	0.99951	1.4008	0.48628	0.10653	0.99998	0.80454	-0.31376	0.78943	0.99941	0.95994	-0.05899	0.062577	0.32853	0.64205	-0.63925	0.003881	0.024452	0.53709	-0.89677	0.01005	0.079508	0.61756	-0.69534	0.46683	0.99391	0.88346	-0.17877	0.70382	0.99956	0.96654	-0.0491	0.023684	0.2688	1.6439	0.71713
577.2839	1.28	domics Pos	0.22443	0.99987	1.0984	0.13539	0.52829	0.99994	1.0512	0.072072	0.83732	0.99996	1.0218	0.031102	0.46614	0.88339	0.93481	-0.09726	0.043316	0.093578	0.76256	-0.39107	0.41031	0.65377	0.91983	-0.12057	0.88535	0.97532	1.0127	0.018148	0.49478	0.96776	1.0612	0.085656	0.049964	0.14647	1.2522	0.32451
716.9383	3.2	olomics Po	0.22533	0.99951	1.3437	0.42624	0.038761	0.99998	0.78265	-0.35356	0.57946	0.99941	0.95226	-0.07058	0.00072	0.12797	0.58831	-0.76535	0.001959	0.015227	0.58294	-0.77857	0.001416	0.045425	0.63705	-0.65052	0.84134	0.9951	1.0101	0.014511	0.59809	0.99956	0.94603	-0.08004	0.019622	0.2688	1.5717	0.65236
295.961	2.64	olomics Ne	0.22619	0.99918	1.0399	0.056445	0.035012	0.44974	0.86529	-0.20875	0.92774	0.99995	1.0003	0.000374	0.8731	0.99985	1.0168	0.024099	0.88532	0.99927	1.0142	0.020281	0.20647	0.65531	0.92399	-0.11405	0.79771	0.94878	0.99131	-0.01259	0.76352	0.99512	1.0251	0.035749	0.60061	0.99941	1.0599	0.083951
504.9728	12	olomics Ne	0.22639	0.99918	1.1176	0.16038	0.015192	0.37315	0.78471	-0.34978	0.67663	0.99995	1.0332	0.047068	0.14521	0.99985	0.90602	-0.14238	0.51437	0.99927	1.0675	0.094246	0.14698	0.6202	1.0976	0.13439	0.84423	0.95428	0.98544	-0.02117	0.93209	0.99512	0.99313	-0.00994	0.56689	0.99941	0.94032	-0.08878
964.137	4.62	domics Pos	0.22735	0.99987	1.4894	0.57476	0.10193	0.99994	0.85128	-0.23229	0.45446	0.99996	1.0647	0.090387	0.006126	0.18537	0.58688	-0.76886	0.000237	0.001888	0.39924	-1.3247	0.012891	0.095682	0.62353	-0.68147	0.96796	0.99304	1.0381	0.053914	0.8226	0.97988	1.0608	0.085111	0.0862	0.18686	2.0176	1.0127
467.7655	3	olomics Po	0.22736	0.99951	1.3561	0.43943	0.37676	0.99998	0.87371	-0.19477	0.77922	0.99941	0.95599	-0.06493	0.032108	0.24911	0.64579	-0.63087	0.034546	0.12802	0.64549	-0.63152	0.022365	0.1103	0.66717	-0.58387	0.95647	0.9974	1.133	0.18013	0.9563	0.99956	1.093	0.12826	0.005842	0.2688	2.2451	1.1668
1142.832	5.41	domics Pos	0.22739	0.99987	1.4243	0.51026	0.11102	0.99994	0.82835	-0.27168	0.59328	0.99996	1.0614	0.085993	0.017048	0.24438	0.61819	-0.69388	0.000661	0.004035	0.53325	-0.90712	0.040307	0.18629	0.69895	-0.51675	0.54463	0.84937	0.9174	-0.12438	0.7856	0.97988	1.1674	0.22336	0.046621	0.14319	2.0936	1.066
590.315	0.56	domics Pos	0.22752	0.99987	1.3345	0.41631	0.06744	0.99994	0.80183	-0.31863	0.16848	0.99996	0.89552	-0.15921	0.001939	0.17581	0.49803	-1.0057	0.00013	0.001228	0.39032	-1.3573	5.25E-05	0.019289	0.48702	-1.038	0.21006	0.65208	0.81805	-0.28973	0.36892	0.96776	0.86811	-0.20406	0.045181	0.14109	1.7879	0.83827
670.9671	9.56	olomics Po	0.22756	0.99951	1.3669	0.45091	0.53079	0.99998	0.91958	-0.12096	0.55087	0.99941	1.0894	0.12357	0.028195	0.24036	0.64523	-0.63212	0.015462	0.068025	0.61645	-0.69793	0.10524	0.31146	0.68683	-0.54198	0.88817	0.9951	1.0863	0.11936	0.88668	0.99956	1.0033	0.004798	0.061751	0.30404	1.786	0.83676
1080.711	0.54	domics Pos	0.22816	0.99987	0.89899	-0.15362	0.48566	0.99994	0.90437	-0.14501	0.010601	0.99996	0.76322	-0.38982	0.1423	0.57114	0.86856	-0.20331	0.026103	0.063965	0.69461	-0.52573	0.25254	0.53916	0.85096	-0.23284	0.099294	0.53627	0.81302	-0.29863	0.27983	0.94365	0.87079	-0.1996	0.4057	0.51342	1.1012	0.13901
478.3295	4.83	domics Pos	0.22841	0.99987	1.1059	0.14521	0.92657	0.99994	0.99622	-0.00547	0.83793	0.99996	1.0128	0.018286	0.66409	0.95717	0.96192	-0.05601	0.010823	0.032597	0.78542	-0.34846	0.64801	0.81547	0.95579	-0.06524	0.4182	0.78128	0.9469	-0.07871	0.92653	0.98573	1.006	0.008613	0.052156	0.14863	1.2167	0.28295
1143.831	9.7	olomics Po	0.22872	0.99951	1.5629	0.64425	0.62174	0.99998	0.9314	-0.10253	0.77047	0.99941	0.96013	-0.0587	0.010873	0.19754	0.64366	-0.63562	0.007356	0.038172	0.64397	-0.63493	0.068625	0.23147	0.70275	-0.50891	0.72364	0.99391	0.95858	-0.06103	0.69309	0.99956	1.2938	0.37156	0.16097	0.41855	1.6871	0.75455
1011.774	0.54	domics Pos	0.22919	0.99987	1.1238	0.16843	0.17939	0.99994	1.087	0.12033	0.14595	0.99996	1.1345	0.18206	0.52213	0.89938	1.1043	0.14312	0.026312	0.06423	0.80555	-0.31196	0.95912	0.97873	1.0127	0.018147	0.52992	0.84162	1.0979	0.13471	0.80167	0.97988	0.98875	-0.01632	0.055001	0.15218	1.2928	0.37051
1109.562	5.8	domics Pos	0.22959	0.99987	1.1268	0.17218	0.91699	0.99994	1.0083	0.011987	0.70336	0.99996	0.95591	-0.06506	0.7015	0.96092	0.95459	-0.06704	0.031979	0.074396	0.75536	-0.40476	0.095432	0.32626	0.77557	-0.36668	0.57489	0.85963	0.93332	-0.09955	0.27844	0.94365	0.88301	-0.17951	0.13413	0.23898	1.169	0.22526
489.2709	3.21	olomics Po	0.22983	0.99951	1.3792	0.46387	0.054536	0.99998	0.77231	-0.37276	0.62822	0.99941	0.93781	-0.09263	0.010912	0.19754	0.57835	-0.78998	0.001159	0.010398	0.45302	-1.1423	0.013077	0.085844	0.639	-0.64611	0.57994	0.99391	0.92525	-0.11209	0.85872	0.99956	1.0201	0.028722	0.014024	0.2688	1.6112	0.68818
1179.836	9.31	olomics Po	0.22989	0.99951	1.4349	0.52098	0.68285	0.99998	0.92792	-0.10793	0.4533	0.99941	1.1142	0.15605	0.13019	0.50318	0.72379	-0.46635	0.033459	0.12516	0.64237	-0.63853	0.34185	0.65262	0.84481	-0.2433	0.59396	0.99391	1.1788	0.23737	0.9813	0.99956	1.0535	0.075236	0.17144	0.43307	1.7549	0.81138
828.9786	3.32	olomics Po	0.23001	0.99951	1.3368	0.41882	0.085434	0.99998	0.80163	-0.31898	0.41818	0.99941	0.92351	-0.1148	0.000883	0.12936	0.56041	-0.83544	0.000198	0.002775	0.48495	-1.0441	0.000883	0.042705	0.59397	-0.75153	0.48305	0.99391	0.90132	-0.14989	0.5765	0.99956	0.93249	-0.10084	0.081981	0.33193	1.6736	0.74299
133.9833	10.12	domics Pos	0.23092	0.99987	1.1078	0.14772	0.80963	0.99994	1.0122	0.017536	0.59206	0.99996	1.0234	0.033375	0.81239	0.98246	1.0169	0.024216	0.024867	0.061529	0.80223	-0.31792	0.1607	0.44307	0.94319	-0.08438	0.82458	0.9618	0.993	-0.01013	0.87402	0.98023	1.0064	0.009241	0.016711	0.099614	1.209	0.27383
158.9648	10.03	domics Pos	0.231	0.99987	1.0375	0.053062	0.28737	0.99994	0.94971	-0.07445	0.39127	0.99996	0.96789	-0.04709	0.20715	0.65238	0.93831	-0.09187	0.000129	0.001227	0.77529	-0.3672	0.29138	0.57461	0.91529	-0.1277	0.97409	0.99324	1.0004	0.000506	0.74168	0.97988	0.98644	-0.0197	0.007165	0.091944	1.1887	0.24933
723.8208	6.3	domics Pos	0.23174	0.99987	1.4971	0.58213	0.38662	0.99994	0.88982	-0.16842	0.30063	0.99996	1.174	0.23148	0.082906	0.4849	0.72148	-0.47097	0.00134	0.006787	0.52172	-0.93865	0.073924	0.28047	0.7515	-0.41216	0.78777	0.95056	1.0919	0.1268	0.70408	0.97988	1.2807	0.35691	0.053473	0.15039	2.0938	1.0661
1139.807	9.37	olomics Po	0.23192	0.99951	1.4532	0.53921	0.78191	0.99998	0.96174	-0.05629	0.28855	0.99941	1.1463	0.19695	0.1161	0.46471	0.78908	-0.34175	0.055767	0.18251	0.66951	-0.57882	0.032721	0.14023	0.6613	-0.59663	0.97643	0.9974	1.0468	0.066033	0.95322	0.99956	1.0454	0.064099	0.12269	0.37265	1.9341	0.95169
852.5917	9.24	olomics Po	0.23204	0.99951	1.2894	0.36674	0.39629	0.99998	0.91953	-0.12103	0.87177	0.99941	1.0323	0.045867	0.3171	0.8121	0.84933	-0.23561	0.006605	0.035625	0.63945	-0.64511	0.10042	0.30083	0.76832	-0.38021	0.86846	0.9951	1.0372	0.052679	0.34043	0.99956	0.87338	-0.19531	0.44706	0.71286	1.1979	0.26049
708.4282	3.19	olomics Po	0.23241	0.99951	1.5271	0.61084	0.062447	0.99998	0.80113	-0.31989	0.53149	0.99941	1.0698	0.097317	0.046325	0.28782	0.6485	-0.62483	0.013744	0.062116	0.64122	-0.64111	0.017994	0.099094	0.6331	-0.6595	0.73971	0.99391	0.9701	-0.0438	0.56204	0.99956	0.93436	-0.09795	0.016803	0.2688	1.7475	0.80527
829.0672	9.24	olomics Po	0.23258	0.99951	1.371	0.45522	0.35955	0.99998	0.83487	-0.26037	0.2668	0.99941	1.1646	0.21989	0.11413	0.45974	0.70847	-0.49723	0.039518	0.1405	0.60987	-0.71343	0.16899	0.42628	0.7598	-0.39632	0.92039	0.9951	1.0317	0.044977	0.97652	0.99956	1.0374	0.052966	0.16288	0.42194	1.5771	0.6573
666.8285	10.1	domics Pos	0.23292	0.99987	1.1417	0.19123	0.028842	0.99994	0.84258	-0.24712	0.94754	0.99996	1.0006	0.000808	0.061679	0.42995	0.82239	-0.2821	0.012168	0.035552	0.81265	-0.29929	0.51105	0.72225	0.92648	-0.11017	0.083008	0.50399	1.1426	0.19237	0.91783	0.98252	0.99428	-0.00827	0.2971	0.40722	1.1694	0.22573
594.3503	1.35	domics Pos	0.23293	0.99987	1.7003	0.76582	0.58676	0.99994	1.1332	0.18045	0.5414	0.99996	1.106	0.14531	0.90026	0.9874	0.98808	-0.0173	0.82918	0.85612	0.90728	-0.14038	0.037174	0.17586	1.834	0.87498	0.030598	0.35576	1.7431	0.80169	0.38308	0.96776	1.247	0.31851	0.086344	0.18706	1.6754	0.74453
984.6665	9.43	olomics Po	0.23311	0.99951	1.3685	0.45262	0.23745	0.99998	0.85879	-0.21962	0.85794	0.99941	1.029	0.041207	0.098091	0.41868	0.79514	-0.33072	0.016506	0.071617	0.64827	-0.62534	0.034163	0.1435	0.70787	-0.49844	0.83637	0.9951	1.0006	0.000867	0.90653	0.99956	1.0273	0.038877	0.13261	0.38536	1.7371	0.79666
413.7807	5.41	domics Pos	0.23366	0.99987	1.0998	0.13727	0.68884	0.99994	1.0161	0.022992	0.95199	0.99996	1.0014	0.001972	0.4905	0.88874	1.0482	0.067898	0.03402	0.078096	0.80903	-0.30573	0.19198	0.47845	0.859	-0.21928	0.16802	0.63957	1.1098	0.15029	0.67783	0.97988	1.0323	0.045928	0.028664	0.11732	1.2508	0.32281
675.8908	3.14	olomics Po	0.23382	0.99951	1.3518	0.43487	0.005179	0.99998	0.80118	-0.3198	0.85964	0.99941	1.0119	0.017038	0.021822	0.2227	0.61195	-0.70851	0.000551	0.006116	0.55909	-0.83884	0.004951	0.061314	0.61064	-0.71161	0.97798	0.9974	1.0355	0.050343	0.671	0.99956	0.9931	-0.00999	0.006047	0.2688	1.8157	0.86049
1184.838	9.34	olomics Po	0.23393	0.99951	1.6197	0.69569	0.8918	0.99998	1.0059	0.008463	0.53218	0.99941	1.0916	0.12649	0.18191	0.60975	0.78303	-0.35286	0.034836	0.1288	0.66295	-0.59303	0.13483	0.36836	0.73786	-0.43858	0.77592	0.99391	1.1055	0.14475	0.59601	0.99956	1.301	0.37963	0.059689	0.30404	1.8155	0.86033
543.9859	3.3	olomics Po	0.23434	0.99951	1.315	0.39511	0.76882	0.99998	0.9543	-0.06748	0.93538	0.99941	0.99649	-0.00507	0.022848	0.22443	0.6365	-0.65177	0.005672	0.032194	0.58966	-0.76205	0.022108	0.11005	0.64115	-0.64126	0.84376	0.9951	1.003	0.004252	0.49867	0.99956	0.88667	-0.17353	0.070534	0.31396	1.7694	0.82323
426.8368	10.14	domics Pos	0.23455	0.99987	0.89257	-0.16397	0.10989	0.99994	0.84387	-0.2449	0.087751	0.99996	0.85458	-0.22672	0.72284	0.96819	0.96943	-0.04479	0.034638	0.079134	0.77075	-0.37567	0.47042	0.6939	0.93791	-0.09248	0.69751	0.91186	0.96738	-0.04785	0.07293	0.77255	0.83744	-0.25594	0.023903	0.11061	1.2649	0.33899
769.5634	10.13	olomics Ne	0.23477	0.99918	0.93142	-0.1025	0.041951	0.46524	0.83828	-0.25449	0.40403	0.99995	0.9472	-0.07825	0.4002	0.99985	0.93596	-0.09549	0.44047	0.99927	0.94651	-0.07931	0.73932	0.94555	0.9771	-0.03342	0.32716	0.69919	1.1075	0.14733	0.89135	0.99512	0.9941	-0.00854	0.77802	0.99941	0.9811	-0.02754
747.6069	5.55	domics Pos	0.23501	0.99987	1.0487	0.068591	0.82671	0.99994	1.0135	0.019362	0.99701	0.99996	0.99996	-6.3E-05	0.50638	0.89133	0.95864	-0.06093	0.008903	0.028095	0.78677	-0.34598	0.068571	0.26653	0.89461	-0.16067	0.44847	0.80156	0.95128	-0.07206	0.21849	0.94047	0.93557	-0.09609	0.52583	0.62461	1.0502	0.07069
650.8993	3.11	olomics Po	0.23504	0.99951	1.2777	0.35353	0.032947	0.99998	0.79241	-0.33568	0.37486	0.99941	0.93695	-0.09396	0.001514	0.14411	0.52865	-0.91961	0.001333	0.01166	0.58571	-0.77175	0.002681	0.046658	0.51956	-0.94463	0.13319	0.68208	0.75583	-0.40387	0.45156	0.99956	0.9077	-0.13972	0.075992	0.32186	1.5697	0.65045
457.235	5.98	olomics Ne	0.23506	0.99918	1.2073	0.27182	0.42357	0.80445	0.86109	-0.21576	0.64394	0.99995	0.91513	-0.12796	0.29195	0.99985	0.79958	-0.32268	1.58E-05	0.009725	0.16742	-2.5785	0.044487	0.48405	0.54134	-0.88538	0.38993	0.73607	0.84412	-0.24448	0.9507	0.99512	0.98618	-0.02007	0.019192	0.99941	1.5407	0.62362
1184.298	6.13	domics Pos	0.23509	0.99987	1.3977	0.48301	0.43221	0.99994	0.87378	-0.19466	0.82691	0.99996	1.0193	0.027578	0.077094	0.46503	0.60348	-0.72861	0.00329	0.013152	0.4861	-1.0407	0.044561	0.20046	0.66873	-0.5805	0.7619	0.94108	0.9644	-0.0523	0.84767	0.97988	1.1174	0.16012	0.10403	0.20689	1.9113	0.93452
329.9967	0.58	domics Pos	0.23533	0.99987	1.1385	0.18719	0.40405	0.99994	0.90755	-0.13996	0.59598	0.99996	1.0467	0.065803	0.063568	0.43296	1.47	0.55584	0.93328	0.94268	0.99418	-0.00842	0.021065	0.12649	1.4479	0.53393	0.001822	0.23318	1.752	0.80903	0.090712	0.82748	1.3369	0.41887	0.075878	0.17781	1.3462	0.42894
820.7815	10.08	domics Pos	0.2354	0.99987	1.1124	0.15363	0.35329	0.99994	0.93021	-0.10437	0.45747	0.99996	0.9302	-0.10439	0.77739	0.97526	0.97599	-0.03506	0.008475	0.027035	0.78592	-0.34754	0.42375	0.65977	0.91691	-0.12515	0.46949	0.80647	1.0524	0.073627	0.80669	0.97988	1.0234	0.033348	0.03412	0.12606	1.2121	0.27748
1142.733	10.05	domics Pos	0.23567	0.99987	1.1178	0.16067	0.64129	0.99994	1.0221	0.031572	0.38908	0.99996	0.92198	-0.11719	0.3911	0.83637	0.94476	-0.08198	0.026857	0.065269	0.79213	-0.3362	0.70086	0.84682	0.97176	-0.04133	0.043895	0.38909	1.1375	0.18585	0.49506	0.96776	1.0989	0.1361	0.029587	0.11849	1.1891	0.24984
86.0977	0.44	olomics Po	0.23579	0.99951	0.81131	-0.30167	0.85852	0.99998	0.97693	-0.03368	0.28673	0.99941	1.1648	0.22003	0.025085	0.23087	1.7399	0.79898	0.07841	0.23749	1.444	0.53011	0.16767	0.42493	1.2775	0.35333	0.005065	0.20472	1.8164	0.86111	0.60793	0.99956	0.91931	-0.12138	0.022178	0.2688	0.69243	-0.53026
937.1555	9.17	olomics Po	0.23596	0.99951	1.346	0.42867	0.69417	0.99998	0.92018	-0.12001	0.24671	0.99941	1.2023	0.26585	0.56045	0.99905	0.88382	-0.17818	0.046137	0.15814	0.6258	-0.67622	0.19071	0.46283	0.71549	-0.48299	0.83676	0.9951	1.0482	0.067914	0.77129	0.99956	0.96064	-0.05793	0.1718	0.43363	1.6093	0.68639
298.8635	10.16	domics Pos	0.23603	0.99987	1.1096	0.15004	0.74269	0.99994	0.96979	-0.04426	0.71404	0.99996	0.96746	-0.04772	0.46985	0.88476	0.94498	-0.08164	0.020081	0.052517	0.8098	-0.30436	0.76583	0.87929	0.97273	-0.03989	0.56483	0.85634	1.0426	0.060185	0.87828	0.98023	0.98432	-0.0228	0.067317	0.16764	1.1757	0.23357
326.2157	2.29	domics Pos	0.23658	0.99987	0.86923	-0.20218	0.43896	0.99994	0.89975	-0.15241	0.03072	0.99996	0.66512	-0.58831	0.000811	0.17581	0.53518	-0.9019	8.98E-05	0.000962	0.49306	-1.0202	0.004755	0.071661	0.40806	-1.2931	0.033041	0.36346	0.55542	-0.84836	0.002316	0.48691	0.45606	-1.1327	0.039889	0.13378	0.56118	-0.83348
645.9726	9.59	olomics Po	0.23661	0.99951	1.4706	0.55643	0.59018	0.99998	0.94567	-0.08059	0.30524	0.99941	1.1054	0.14456	0.018876	0.21385	0.68096	-0.55436	0.004451	0.027224	0.63865	-0.64691	0.018043	0.099188	0.70022	-0.51413	0.80197	0.99391	1.0846	0.1171	0.82085	0.99956	1.2168	0.28304	0.099183	0.35294	1.7456	0.80376
1162.245	4.35	domics Pos	0.23688	0.99987	1.2952	0.37322	0.76841	0.99994	0.96943	-0.04479	0.30183	0.99996	1.0993	0.13662	0.011965	0.22039	0.66698	-0.58429	0.002031	0.009163	0.4866	-1.0392	0.002675	0.061655	0.6158	-0.69947	0.93736	0.98682	1.023	0.032811	0.93805	0.98616	1.0331	0.046955	0.019639	0.10555	1.7326	0.79292
1054.779	5.54	domics Pos	0.23694	0.99987	1.4903	0.57562	0.16914	0.99994	0.85462	-0.22664	0.94608	0.99996	0.99966	-0.00049	0.024283	0.27645	0.63269	-0.66043	0.000412	0.002823	0.47408	-1.0768	0.029087	0.15069	0.70369	-0.50699	0.80458	0.95494	0.99819	-0.00261	0.82947	0.97988	1.1383	0.1869	0.058336	0.15555	2.0667	1.0473
199.1736	2.83	domics Pos	0.23701	0.99987	1.2541	0.32664	0.69825	0.99994	0.91814	-0.12321	0.30815	0.99996	0.79218	-0.3361	0.091157	0.50268	0.67589	-0.56515	0.00142	0.007058	0.32023	-1.6428	0.008389	0.084185	0.4301	-1.2173	0.018286	0.33809	0.50106	-0.99695	0.092146	0.83655	0.61429	-0.70302	0.51778	0.61774	0.85676	-0.22304
717.6018	7.09	domics Pos	0.23731	0.99987	0.87656	-0.19007	0.035606	0.99994	0.77198	-0.37336	0.88715	0.99996	1.038	0.05377	0.073538	0.45892	0.77784	-0.36245	0.15517	0.24348	7.1142	2.8307	0.035669	0.17151	10.013	3.3238	0.16807	0.63957	17.443	4.1246	0.10954	0.87013	7.5386	2.9143	0.063917	0.16226	5.9424	2.571
138.0538	0.59	domics Pos	0.23771	0.99987	1.0563	0.079003	0.048065	0.99994	0.83697	-0.25676	0.72944	0.99996	0.97516	-0.03629	0.065959	0.43825	1.2628	0.33667	0.6958	0.74129	0.97122	-0.04213	0.036016	0.17231	1.3946	0.47988	3.14E-05	0.11971	1.8727	0.9051	0.053037	0.68786	1.3239	0.40484	0.000529	0.07528	1.5014	0.58632
985.1375	4.32	domics Pos	0.23912	0.99987	1.4231	0.50901	0.88594	0.99994	0.93755	-0.09303	0.65429	0.99996	1.0789	0.10951	0.24196	0.70266	0.71539	-0.4832	0.025965	0.063669	0.49835	-1.0048	0.20517	0.49558	0.70649	-0.50127	0.89462	0.97532	0.9501	-0.07386	0.8092	0.97988	1.1088	0.14894	0.18226	0.28944	1.8076	0.85406
620.6156	2.75	domics Pos	0.23917	0.99987	1.625	0.70048	0.42449	0.99994	0.69813	-0.51843	0.38817	0.99996	0.68007	-0.55625	0.036121	0.33474	0.31804	-1.6527	0.00018	0.001557	0.056032	-4.1576	0.002238	0.061386	0.16531	-2.5968	0.03305	0.36346	0.3387	-1.5619	0.18083	0.92305	0.56188	-0.83168	0.74976	0.81286	1.0324	0.045993
619.6138	2.75	domics Pos	0.23931	0.99987	1.5911	0.67004	0.4691	0.99994	0.73561	-0.443	0.51165	0.99996	0.75178	-0.41162	0.054885	0.40246	0.3453	-1.5341	0.000198	0.001687	0.026914	-5.2155	0.015193	0.10398	0.20948	-2.2551	0.021156	0.33809	0.29198	-1.776	0.20509	0.92582	0.58493	-0.77366	0.39004	0.49906	1.2719	0.34701
941.6063	8.91	olomics Po	0.23942	0.99951	1.3674	0.45144	0.47247	0.99998	0.92573	-0.11134	0.18147	0.99941	1.1152	0.15736	0.000139	0.077904	0.72602	-0.46191	0.057188	0.18562	0.66725	-0.5837	0.27393	0.56619	0.89332	-0.16276	0.44021	0.99391	1.3596	0.44314	0.72053	0.99956	1.116	0.15833	0.046906	0.28746	1.9613	0.97181
962.1478	9.14	olomics Po	0.23952	0.99951	1.3023	0.38109	0.7721	0.99998	0.92696	-0.10942	0.52371	0.99941	1.1126	0.154	0.47267	0.95078	0.84705	-0.23947	0.042165	0.14709	0.62262	-0.68357	0.27847	0.5722	0.76374	-0.38884	0.95483	0.9974	1.0371	0.052575	0.78103	0.99956	0.95486	-0.06664	0.34509	0.62093	1.3403	0.42255
312.0405	0.56	domics Pos	0.23985	0.99987	0.79121	-0.33787	0.15842	0.99994	0.73632	-0.4416	0.93063	0.99996	1.0092	0.013185	0.13234	0.55697	1.4216	0.50749	0.96026	0.96608	0.98066	-0.02818	0.93352	0.96621	0.98411	-0.02312	0.036402	0.36759	1.4342	0.52027	0.24224	0.94365	0.78198	-0.3548	0.15026	0.25567	0.7467	-0.4214
880.9745	12.02	olomics Ne	0.24024	0.99918	1.2686	0.34324	0.66338	0.92948	0.94447	-0.08243	0.042718	0.99995	1.3341	0.41587	0.99946	0.99985	0.99943	-0.00082	0.83491	0.99927	1.0337	0.047811	0.5873	0.89313	1.0786	0.10914	0.80169	0.95074	0.96589	-0.05006	0.63017	0.99512	1.0616	0.086192	0.55428	0.99941	0.92086	-0.11895
1010.752	5.61	domics Pos	0.24095	0.99987	1.4517	0.53776	0.68062	0.99994	0.91616	-0.12633	0.78824	0.99996	1.0429	0.060619	0.047882	0.37667	0.65159	-0.61797	0.011429	0.034018	0.49884	-1.0033	0.036926	0.17491	0.61404	-0.70359	0.74467	0.93287	0.95984	-0.05913	0.85579	0.98021	1.0489	0.068841	0.023742	0.11051	2.1712	1.1185
730.3525	6.19	domics Pos	0.24099	0.99987	0.85257	-0.2301	0.027302	0.99994	0.71475	-0.48449	0.66179	0.99996	0.94412	-0.08296	0.013695	0.22898	0.70196	-0.51055	0.006974	0.023285	0.57259	-0.80443	0.25725	0.54373	0.84022	-0.25115	0.068457	0.46863	0.76066	-0.39469	0.075241	0.7796	0.79074	-0.33873	0.56294	0.65884	0.91692	-0.12513
578.8262	3.02	olomics Po	0.24142	0.99951	1.2962	0.37427	0.21163	0.99998	0.85689	-0.22281	0.82586	0.99941	0.97044	-0.04328	0.00971	0.19702	0.52029	-0.94261	0.002041	0.015683	0.55479	-0.84998	0.027818	0.12626	0.72175	-0.47043	0.64217	0.99391	0.94375	-0.08352	0.98203	0.99956	1.0715	0.099628	0.1397	0.39361	1.7017	0.76698
472.9782	5.05	olomics Ne	0.24188	0.99918	1.055	0.077202	0.013878	0.37315	0.91645	-0.12588	0.61964	0.99995	1.0314	0.044569	0.67915	0.99985	1.0238	0.03397	0.95324	0.99927	1.0008	0.001133	0.045962	0.48405	1.1601	0.21424	0.013901	0.27595	1.097	0.13355	0.47825	0.99512	1.0459	0.064747	0.59647	0.99941	1.0388	0.054968
481.7709	3.03	olomics Po	0.24196	0.99951	1.4271	0.51305	0.71481	0.99998	0.94494	-0.08171	0.8604	0.99941	1.0134	0.019182	0.056432	0.31037	0.7088	-0.49655	0.019481	0.082193	0.62543	-0.67708	0.033191	0.14206	0.68074	-0.55482	0.78333	0.99391	0.98493	-0.02191	0.89731	0.99956	1.0135	0.019411	0.018269	0.2688	2.4238	1.2772
432.2986	2.06	domics Pos	0.2422	0.99987	1.2515	0.32362	0.91859	0.99994	0.97421	-0.03769	0.30377	0.99996	1.1589	0.21274	0.96998	0.99502	1.0189	0.027066	0.001504	0.007333	0.39769	-1.3303	0.15275	0.43045	0.79833	-0.32494	0.37915	0.76056	1.1359	0.18386	0.45854	0.96776	1.1243	0.16903	0.014921	0.09885	1.4665	0.55241
622.5705	0.32	olomics Po	0.24224	0.99951	1.1579	0.21151	0.604	0.99998	1.0522	0.073362	0.77427	0.99941	1.0262	0.037265	0.16031	0.5655	1.0902	0.12464	0.015996	0.069982	1.2922	0.36982	0.21821	0.49953	1.1336	0.1809	0.088438	0.60275	1.1652	0.22053	0.032404	0.58535	1.1753	0.23304	0.64714	0.83933	0.95766	-0.06242
470.814	9.92	domics Pos	0.24227	0.99987	1.1652	0.22053	0.84487	0.99994	0.98695	-0.01895	0.26427	0.99996	0.91856	-0.12256	0.45748	0.87572	0.93546	-0.09625	0.001413	0.007035	0.74741	-0.42003	0.54469	0.74601	0.93659	-0.09451	0.68039	0.90364	0.96464	-0.05194	0.77212	0.97988	0.98107	-0.02758	0.081339	0.18298	1.191	0.25222
412.746	2.9	olomics Po	0.24229	0.99951	1.3408	0.42312	0.15372	0.99998	0.81843	-0.28906	0.74104	0.99941	0.95516	-0.06619	0.015592	0.20428	0.64582	-0.63081	0.006571	0.035557	0.57499	-0.7984	0.013306	0.085847	0.62579	-0.67626	0.97466	0.9974	1.0415	0.05866	0.84874	0.99956	1.0372	0.052653	0.079526	0.32557	2.0384	1.0275
723.4961	10.67	olomics Ne	0.24239	0.99918	1.1244	0.16915	0.070134	0.50118	0.83672	-0.25719	0.18413	0.99995	1.1379	0.18632	0.45318	0.99985	1.0515	0.072439	0.96963	0.99927	0.99509	-0.00711	0.06543	0.51001	1.1493	0.20079	0.036161	0.35692	1.3799	0.46458	0.2731	0.99512	1.0662	0.092467	0.89715	0.99941	0.9915	-0.01231
643.9647	9.55	olomics Po	0.24327	0.99951	1.4429	0.52894	0.94971	0.99998	0.97136	-0.04192	0.27876	0.99941	1.1838	0.2434	0.063769	0.33109	0.66049	-0.59839	0.021493	0.08885	0.60506	-0.72484	0.13152	0.3624	0.7379	-0.4385	0.65389	0.99391	1.142	0.19156	0.91856	0.99956	1.0723	0.10076	0.10696	0.36162	1.8657	0.89969
631.5229	6.38	olomics Po	0.2434	0.99951	1.176	0.23387	0.24813	0.99998	0.85844	-0.22021	0.55271	0.99941	1.0506	0.07122	0.23695	0.69626	0.862	-0.21423	7.9E-05	0.001338	0.32913	-1.6033	0.086674	0.26979	0.71882	-0.47629	0.1024	0.61787	0.76623	-0.38416	0.97976	0.99956	0.9858	-0.02063	0.17767	0.44124	1.1746	0.2322
506.9759	5.11	olomics Ne	0.24402	0.99918	1.061	0.085363	0.27438	0.7026	0.93234	-0.10107	0.57475	0.99995	1.0261	0.037239	0.67578	0.99985	1.0297	0.042197	0.45993	0.99927	1.0609	0.085323	0.045439	0.48405	1.1465	0.19719	0.1693	0.5427	1.0856	0.11854	0.22817	0.99512	1.0991	0.13626	0.25989	0.99941	1.0672	0.093779
939.6334	9.17	olomics Po	0.24404	0.99951	1.2893	0.36658	0.42535	0.99998	0.84764	-0.23848	0.86933	0.99941	1.0123	0.01763	0.34503	0.83927	0.80947	-0.30495	0.040206	0.14235	0.6391	-0.64588	0.16008	0.41231	0.71228	-0.48949	0.73054	0.99391	0.93722	-0.09355	0.38972	0.99956	0.82362	-0.27994	0.30164	0.57983	1.4278	0.5138
862.5092	4.86	domics Pos	0.24413	0.99987	0.86962	-0.20154	0.026914	0.99994	0.69964	-0.51532	0.75548	0.99996	0.95837	-0.06134	0.95159	0.99328	1.0106	0.015272	0.059934	0.1189	0.71173	-0.49059	0.63578	0.80915	0.93448	-0.09776	0.7759	0.94551	0.96567	-0.0504	0.91567	0.98252	1.0055	0.007866	0.28318	0.39279	1.1463	0.19701
1097.803	5.48	domics Pos	0.24419	0.99987	1.5172	0.60138	0.57697	0.99994	0.90506	-0.14391	0.7477	0.99996	1.0368	0.052122	0.086488	0.49062	0.67018	-0.57737	0.003266	0.013108	0.45773	-1.1274	0.023676	0.13515	0.64513	-0.63233	0.92134	0.98126	1.037	0.052367	0.83266	0.97988	1.1708	0.22747	0.052817	0.1494	2.2201	1.1506
268.6506	1.3	domics Pos	0.24441	0.99987	1.1651	0.22043	0.87838	0.99994	1.0074	0.010697	0.97128	0.99996	1.0054	0.007707	0.85947	0.9874	0.97905	-0.03055	0.026265	0.064222	0.69439	-0.52618	0.94484	0.97055	1.009	0.012946	0.11513	0.5679	1.2886	0.36584	0.43586	0.96776	1.0978	0.13467	0.008878	0.092495	1.5586	0.64021
981.1813	9.11	olomics Po	0.24462	0.99951	1.2429	0.31368	0.4383	0.99998	0.89524	-0.15965	0.15303	0.99941	1.1708	0.22747	0.14098	0.52853	0.75258	-0.41008	0.023154	0.094353	0.67826	-0.56009	0.078921	0.25368	0.71109	-0.49189	0.8979	0.9951	1.0495	0.069648	0.6811	0.99956	0.97876	-0.03098	0.42523	0.69619	1.3873	0.47227
715.517	10.15	olomics Ne	0.24536	0.99918	0.87479	-0.19299	0.066728	0.50118	0.84645	-0.2405	0.92806	0.99995	1.0665	0.092912	0.41435	0.99985	0.92195	-0.11724	0.53367	0.99927	1.1149	0.15696	0.30494	0.72466	1.1672	0.22304	0.02363	0.33055	1.3636	0.44739	0.29413	0.99512	1.2013	0.26465	0.32862	0.99941	1.1481	0.19926
182.1556	5.99	olomics Po	0.24586	0.99951	1.2162	0.28233	0.24722	0.99998	0.84944	-0.23542	0.98746	0.99941	1.0041	0.005855	0.21839	0.66403	0.79381	-0.33313	2.78E-06	0.000231	0.2315	-2.1109	0.022643	0.1111	0.50004	-0.99988	0.18658	0.76329	0.79796	-0.32562	0.54497	0.99956	0.93233	-0.10109	0.23105	0.49677	1.1312	0.17791
195.1752	6.38	olomics Po	0.24619	0.99951	1.1922	0.25366	0.33097	0.99998	0.85093	-0.23288	0.9117	0.99941	0.98125	-0.0273	0.15471	0.55316	0.7167	-0.48055	7.2E-06	0.000278	0.2022	-2.3061	0.003404	0.049867	0.52054	-0.94192	0.060077	0.49674	0.70126	-0.51199	0.23124	0.99956	0.83464	-0.26078	0.96291	0.98224	0.98586	-0.02055
1093.773	5.48	domics Pos	0.24644	0.99987	1.3665	0.45052	0.70982	0.99994	0.92046	-0.11957	0.7204	0.99996	1.0507	0.071356	0.031848	0.31138	0.62703	-0.67338	0.011847	0.03491	0.51426	-0.95942	0.094771	0.32467	0.67494	-0.56718	0.9822	0.99431	1.0259	0.036859	0.81273	0.97988	1.097	0.13359	0.081636	0.18321	2.0337	1.0241
338.1635	0.58	domics Pos	0.2465	0.99987	1.328	0.40926	0.008569	0.99994	0.68593	-0.54387	0.73037	0.99996	0.96191	-0.05602	0.16395	0.59595	0.79507	-0.33085	0.002111	0.009426	0.4671	-1.0982	0.001209	0.043885	0.52028	-0.94264	0.9025	0.97745	1.0848	0.11738	0.50377	0.96776	0.93083	-0.10341	0.067569	0.16764	2.4552	1.2958
1053.777	5.54	domics Pos	0.24663	0.99987	1.4826	0.56812	0.33374	0.99994	0.85439	-0.22704	0.91363	0.99996	1.005	0.007198	0.043871	0.36496	0.66531	-0.5879	0.003468	0.013701	0.48647	-1.0396	0.028286	0.14826	0.62016	-0.68929	0.95789	0.98985	1.019	0.027149	0.78244	0.97988	1.0349	0.049441	0.042018	0.1364	2.2703	1.1829
107.0868	6.38	olomics Po	0.24717	0.99951	1.1261	0.17133	0.55966	0.99998	0.93799	-0.09235	0.53905	0.99941	0.93524	-0.09659	0.096576	0.41572	0.77077	-0.37563	2.48E-06	0.000231	0.25351	-1.9799	0.003099	0.049681	0.56432	-0.82541	0.027064	0.33696	0.68721	-0.54117	0.070524	0.76039	0.79885	-0.324	0.5007	0.75248	0.92433	-0.11351
185.1735	1.66	domics Pos	0.24729	0.99987	1.1826	0.24197	0.15381	0.99994	0.78135	-0.35596	0.7301	0.99996	1.0427	0.060353	0.12358	0.546	0.72269	-0.46856	7.04E-06	0.000186	0.16901	-2.5648	0.004542	0.071311	0.50506	-0.98548	0.12727	0.58744	0.74197	-0.43057	0.44007	0.96776	0.91194	-0.13299	0.054694	0.15191	1.2694	0.34413
796.4207	0.56	domics Pos	0.24732	0.99987	1.37	0.45417	0.091733	0.99994	0.81921	-0.28769	0.13868	0.99996	0.89779	-0.15555	0.00384	0.17581	0.60708	-0.72004	0.000532	0.003458	0.45752	-1.1281	0.01346	0.097963	0.66961	-0.5786	0.38505	0.76547	0.88587	-0.17483	0.5044	0.96776	0.91983	-0.12056	0.010033	0.094875	1.8028	0.85021
891.6183	8.94	olomics Po	0.24745	0.99951	1.4114	0.49711	0.86638	0.99998	0.9923	-0.01115	0.32426	0.99941	1.155	0.20785	0.3244	0.82077	0.89531	-0.15953	0.052907	0.17506	0.73979	-0.4348	0.014891	0.091257	0.75753	-0.40063	0.58151	0.99391	1.0914	0.12614	0.73091	0.99956	1.1167	0.15926	0.10626	0.36133	1.59	0.66901
872.0839	8.95	olomics Po	0.24751	0.99951	1.2888	0.36602	0.61665	0.99998	0.94737	-0.078	0.57522	0.99941	1.045	0.06353	0.050828	0.29486	0.71366	-0.4867	0.029823	0.1147	0.66752	-0.58313	0.097582	0.29498	0.78667	-0.34616	0.80378	0.99391	1.0823	0.11404	0.93984	0.99956	1.075	0.10434	0.15758	0.41468	1.6196	0.69567
677.8731	3.14	olomics Po	0.24766	0.99951	1.2877	0.36474	0.058144	0.99998	0.80575	-0.31159	0.90866	0.99941	1.0127	0.018212	0.001676	0.14411	0.57912	-0.78806	0.00076	0.00753	0.54673	-0.8711	0.001155	0.044437	0.54734	-0.8695	0.51388	0.99391	0.9152	-0.12784	0.21842	0.99956	0.82513	-0.2773	0.019635	0.2688	1.8247	0.86763
185.0779	4.66	domics Pos	0.24823	0.99987	0.89222	-0.16453	0.39989	0.99994	0.90855	-0.13837	0.34433	0.99996	0.87314	-0.19571	0.40388	0.84614	0.9186	-0.12249	0.002335	0.010151	0.76008	-0.39579	0.057495	0.23933	0.83773	-0.25545	0.23905	0.67869	0.90235	-0.14824	0.16726	0.91806	0.88197	-0.18119	0.50179	0.60339	0.94341	-0.08404
384.2147	2.9	olomics Po	0.24825	0.99951	1.4289	0.51487	0.15654	0.99998	0.83438	-0.26122	0.95723	0.99941	1.0131	0.018765	0.10882	0.44633	0.7064	-0.50144	0.012196	0.056663	0.62633	-0.675	0.024109	0.11506	0.74903	-0.41691	0.6848	0.99391	1.1697	0.22611	0.84293	0.99956	1.1804	0.23923	0.021442	0.2688	2.197	1.1356
182.1551	1.67	domics Pos	0.24837	0.99987	1.1585	0.21232	0.15792	0.99994	0.82356	-0.28006	0.97503	0.99996	0.99045	-0.01384	0.10747	0.53121	0.75651	-0.40257	5.12E-06	0.000159	0.14907	-2.7459	0.006026	0.077417	0.52394	-0.93253	0.081464	0.49998	0.73246	-0.44918	0.31819	0.9524	0.87439	-0.19366	0.023496	0.11051	1.3239	0.40478
1026.677	8.86	olomics Po	0.24852	0.99951	1.3035	0.38236	0.44165	0.99998	0.89106	-0.1664	0.2788	0.99941	1.1143	0.15619	0.02883	0.24036	0.68621	-0.54328	0.037277	0.13512	0.67144	-0.57466	0.034167	0.1435	0.69419	-0.52659	0.98261	0.99744	1.03	0.042633	0.94066	0.99956	1.0693	0.096603	0.10295	0.35675	1.6767	0.74565
217.1983	5.37	olomics Po	0.24858	0.99951	1.1014	0.13939	0.072253	0.99998	0.82003	-0.28625	0.36411	0.99941	0.90356	-0.14631	0.013265	0.20085	0.7436	-0.42739	0.00026	0.003484	0.38937	-1.3608	0.015477	0.092864	0.67219	-0.57307	0.92477	0.9951	1.0197	0.028172	0.95042	0.99956	0.99069	-0.0135	0.44798	0.71341	1.0928	0.12806
829.441	2.9	olomics Po	0.24875	0.99951	1.4652	0.5511	0.11024	0.99998	0.79747	-0.32651	0.46486	0.99941	0.91485	-0.12839	0.004408	0.18199	0.4591	-1.1231	0.000178	0.002551	0.50255	-0.99267	0.010527	0.081075	0.57795	-0.79098	0.60096	0.99391	0.94536	-0.08107	0.7178	0.99956	1.0318	0.045142	0.028835	0.2688	2.2301	1.1571
192.1832	2.05	domics Pos	0.2488	0.99987	1.1685	0.22464	0.37995	0.99994	0.87389	-0.19448	0.91849	0.99996	1.0076	0.01098	0.17137	0.60526	0.79647	-0.32832	2.2E-06	0.000144	0.17206	-2.539	0.003662	0.06753	0.51673	-0.95252	0.21237	0.65409	0.83235	-0.26474	0.44439	0.96776	0.91721	-0.12468	0.010056	0.094875	1.3524	0.43551
201.1597	4.73	domics Pos	0.24882	0.99987	0.90782	-0.13952	0.72859	0.99994	1.029	0.041252	0.33506	0.99996	1.0904	0.12491	0.85	0.9874	0.98547	-0.02112	0.000276	0.002086	0.66054	-0.59828	0.71982	0.8537	0.97131	-0.042	0.43739	0.79683	1.0768	0.10669	0.53936	0.96776	1.1128	0.15413	0.041712	0.13617	1.1904	0.25148
316.2482	3.72	olomics Po	0.24902	0.99951	0.77901	-0.36029	0.083356	0.99998	0.71678	-0.48039	0.7686	0.99941	0.94015	-0.08903	0.018344	0.20936	0.58992	-0.76142	0.047792	0.16257	0.45441	-1.1379	0.005929	0.065713	0.20076	-2.3164	0.027427	0.33821	0.3652	-1.4532	0.035223	0.60963	0.41036	-1.285	0.01078	0.2688	0.24118	-2.0518

464.3741	8.13	bolomics Pc	0.24903	0.99951	0.91461	-0.12878	0.95655	0.99998	1.0146	0.0209	0.066436	0.99941	0.89132	-0.16598	0.72661	0.99905	0.9766	-0.03417	0.87626	0.99688	1.0158	0.022566	0.4324	0.73985	0.94564	-0.08064	0.99906	0.99906	1.0078	0.011204	0.031928	0.58535	0.87417	-0.19402	0.17026	0.4319	0.86858	-0.20327
1110.786	10.02	domics Pos	0.24929	0.99987	1.0456	0.064357	0.2561	0.99994	0.95432	-0.06745	0.55025	0.99996	0.9751	-0.03637	0.023502	0.27405	0.90469	-0.14451	5.33E-05	0.000676	0.75257	-0.41011	0.25469	0.54131	0.90834	-0.1387	0.69925	0.9131	1.0199	0.028414	0.95428	0.98838	1.003	0.004327	0.018539	0.10388	1.2122	0.27759
522.2586	0.58	domics Pos	0.25002	0.99987	1.3903	0.47537	0.38191	0.99994	0.82646	-0.27498	0.9405	0.99996	0.97869	-0.03108	0.14346	0.57187	0.64768	-0.62666	0.003131	0.012674	0.43899	-1.1877	0.10183	0.33955	0.65544	-0.60946	0.49285	0.82243	0.86305	-0.21248	0.50035	0.96776	0.86908	-0.20243	0.05014	0.14661	2.0802	1.0567
570.3167	0.58	domics Pos	0.25022	0.99987	1.4585	0.54444	0.12508	0.99994	0.83909	-0.25311	0.91911	0.99996	0.98847	-0.01673	0.006491	0.1915	0.5957	-0.74734	1.94E-05	0.000362	0.36217	-1.4653	0.001051	0.043236	0.55675	-0.84491	0.59585	0.86481	0.93779	-0.09267	0.77077	0.97988	1.0396	0.055961	0.02738	0.11602	2.5307	1.3395
386.2741	1.05	domics Pos	0.25035	0.99987	0.82221	-0.28242	0.38202	0.99994	0.8492	-0.23582	0.9706	0.99996	0.98048	-0.02844	0.13576	0.55962	0.76382	-0.38869	0.044853	0.096002	0.61379	-0.70419	0.5292	0.7362	0.88868	-0.17026	0.36788	0.76056	1.275	0.35051	0.42917	0.96776	0.85309	-0.22923	0.4346	0.54067	1.1126	0.15399
424.3418	0.65	domics Pos	0.25049	0.99987	0.75378	-0.40778	0.19049	0.99994	0.71799	-0.47797	0.24819	0.99996	0.7542	-0.40698	0.035687	0.33351	0.55809	-0.84143	0.010062	0.030792	0.32602	-1.617	0.03795	0.17861	0.404	-1.3076	0.092324	0.52153	0.50865	-0.97526	0.081741	0.80297	0.4934	-1.0192	0.040289	0.13378	0.49413	-1.017
417.2202	0.75	domics Pos	0.25055	0.99987	1.1737	0.23109	0.08945	0.99994	0.72914	-0.45573	0.60405	0.99996	0.92176	-0.11753	0.86499	0.9874	1.0275	0.039122	0.037903	0.084964	0.61511	-0.70108	0.29865	0.57957	0.79741	-0.32661	0.42959	0.79094	0.87936	-0.18547	0.69324	0.97988	0.94228	-0.08577	0.21653	0.32497	1.3542	0.43745
340.1733	6.47	bolomics Pc	0.2506	0.99951	1.1515	0.20355	0.53961	0.99998	0.9256	-0.11155	0.54156	0.99941	0.93519	-0.09667	0.41069	0.90215	0.85868	-0.2198	0.002299	0.01716	0.47984	-1.0594	0.005965	0.065713	0.52971	-0.91674	0.091391	0.60607	0.70758	-0.49903	0.10305	0.84495	0.66177	-0.59561	0.13113	0.38344	0.74532	-0.42408
485.1142	1.44	domics Pos	0.25095	0.99987	1.3286	0.4099	0.51423	0.99994	1.2834	0.36003	0.23873	0.99996	1.2943	0.37221	0.11778	0.53907	0.75436	-0.40667	0.023276	0.058432	0.75965	-0.39659	0.46098	0.68833	1.6433	0.71659	0.46401	0.80647	1.5067	0.5914	0.36308	0.96776	1.2733	0.34855	0.041049	0.13505	1.5108	0.59528
430.9146	10.03	domics Pos	0.25096	0.99987	1.0339	0.048071	0.19598	0.99994	0.94056	-0.08841	0.22838	0.99996	0.95549	-0.06569	0.14776	0.5749	0.93718	-0.09361	5.16E-05	0.00066	0.77011	-0.37685	0.24935	0.53672	0.90556	-0.14312	0.87134	0.97532	1.0114	0.01637	0.49521	0.96776	0.97158	-0.0416	0.008136	0.092094	1.1946	0.25648
1135.814	9.34	bolomics Pc	0.25121	0.99951	1.453	0.539	0.4004	0.99998	0.84979	-0.23483	0.27965	0.99941	1.1602	0.21443	0.0816	0.37744	0.70344	-0.50751	0.075705	0.23041	0.69358	-0.52786	0.04781	0.18101	0.6762	-0.56448	0.90567	0.9951	1.0552	0.077583	0.93072	0.99956	1.1504	0.20211	0.20957	0.4763	1.7788	0.83091
1004.162	8.87	bolomics Pc	0.25126	0.99951	1.3468	0.42957	0.41284	0.99998	0.90803	-0.13918	0.92999	0.99941	1.0204	0.029173	0.038711	0.27038	0.75068	-0.41372	0.008371	0.042191	0.5857	-0.77176	0.004244	0.05479	0.69478	-0.52537	0.94886	0.9974	1.0603	0.084533	0.93499	0.99956	1.0886	0.1225	0.11475	0.36506	1.7054	0.77015
961.1447	9.15	bolomics Pc	0.25161	0.99951	1.2547	0.3273	0.44203	0.99998	0.8768	-0.18968	0.6283	0.99941	1.053	0.074442	0.25456	0.725	0.79345	-0.33378	0.015535	0.068239	0.59513	-0.74871	0.23923	0.5292	0.75914	-0.39756	0.6679	0.99391	0.92871	-0.1067	0.47447	0.99956	0.87443	-0.19359	0.36465	0.64385	1.3578	0.44124
586.8345	3.02	bolomics Pc	0.25176	0.99951	1.345	0.4276	0.098356	0.99998	0.82521	-0.27717	0.34366	0.99941	1.0974	0.13411	0.006195	0.1948	0.62246	-0.68394	0.000921	0.008819	0.55244	-0.85611	0.000506	0.039708	0.58431	-0.77519	0.62548	0.99391	0.94678	-0.0789	0.85988	0.99956	1.0507	0.071416	0.06092	0.30404	1.6393	0.7131
125.9869	10.24	domics Pos	0.25195	0.99987	1.0531	0.074594	0.19949	0.99994	0.93951	-0.09002	0.87556	0.99996	0.99305	-0.01007	0.083764	0.48766	0.91276	-0.13169	0.000391	0.002724	0.75685	-0.40193	0.20125	0.48967	0.89725	-0.15642	0.98193	0.99431	1.0035	0.005015	0.73009	0.97988	0.98085	-0.0279	0.045986	0.14289	1.1596	0.21362
1029.162	4.27	domics Pos	0.252	0.99987	1.3946	0.47989	0.80802	0.99994	1.0079	0.011412	0.2545	0.99996	1.2583	0.33147	0.21676	0.66737	0.69861	-0.51744	0.026395	0.064391	0.49001	-1.0291	0.14202	0.41399	0.66463	-0.58937	0.738	0.93051	1.0914	0.12613	0.79826	0.97988	1.1413	0.19072	0.12823	0.23317	1.8656	0.89964
864.5092	4.59	domics Pos	0.25213	0.99987	1.0679	0.094809	0.89806	0.99994	0.99454	-0.0079	0.65296	0.99996	1.0553	0.07772	0.15099	0.57977	0.89843	-0.15452	0.4989	0.5735	0.934	-0.09851	0.76393	0.87869	1.0448	0.063286	0.006961	0.31228	1.1873	0.24773	0.45484	0.96776	1.0786	0.10921	0.001592	0.07528	1.2691	0.34381
123.1181	6.38	bolomics Pc	0.2522	0.99951	1.0976	0.13434	0.09234	0.99998	0.8713	-0.19876	0.22229	0.99941	0.92167	-0.11768	0.037401	0.26415	0.74521	-0.42427	5.71E-07	0.000231	0.26754	-1.9022	0.002251	0.045856	0.54333	-0.88009	0.008127	0.23862	0.70482	-0.50467	0.024397	0.52244	0.75214	-0.41093	0.47581	0.73724	0.93586	-0.09563
1024.2	8.86	bolomics Pc	0.25242	0.99951	1.2506	0.32266	0.21506	0.99998	0.83499	-0.26017	0.93036	0.99941	1.0129	0.018496	0.016061	0.20428	0.74718	-0.42047	0.005372	0.030825	0.62027	-0.68902	0.002865	0.048262	0.59418	-0.75102	0.50226	0.99391	0.92501	-0.11246	0.89571	0.99956	1.0842	0.11661	0.11937	0.36992	1.6186	0.69477
1138.25	9.01	bolomics Pc	0.25265	0.99951	1.3649	0.44884	0.43821	0.99998	0.89828	-0.15476	0.71211	0.99941	1.0827	0.11458	0.011737	0.19877	0.68536	-0.54506	0.011843	0.055443	0.59187	-0.75664	0.046984	0.1784	0.77875	-0.36076	0.525	0.99391	1.2332	0.30243	0.30947	0.99956	1.1977	0.26025	0.047138	0.28776	1.7086	0.77285
302.018	0.57	domics Pos	0.25268	0.99987	0.81805	-0.28975	0.18354	0.99994	0.77703	-0.36396	0.58743	0.99996	1.1014	0.13928	0.043827	0.36496	1.4939	0.5791	0.85985	0.88182	0.96188	-0.05608	0.57236	0.76608	1.076	0.10566	0.038642	0.37121	1.4545	0.5405	0.55492	0.96798	0.89177	-0.16526	0.40623	0.51374	0.86791	-0.20438
964.4914	8.32	olomics Ne	0.25274	0.99918	1.1254	0.17048	0.74577	0.95729	0.97393	-0.0381	0.48537	0.99995	1.0682	0.095229	0.70429	0.99985	1.0735	0.10231	0.71639	0.99927	1.043	0.060725	0.038899	0.48405	1.2686	0.34325	0.020162	0.30247	1.2925	0.37014	0.18071	0.99512	1.1354	0.18319	0.24275	0.99941	1.1543	0.20699
178.9773	12.76	olomics Ne	0.25288	0.99918	1.0475	0.066896	0.19279	0.61138	0.93559	-0.09606	0.071044	0.99995	1.0644	0.090069	0.90306	0.99985	1.011	0.015744	0.96928	0.99927	1.0052	0.007553	0.093853	0.55533	1.1681	0.22414	0.027721	0.33786	1.1148	0.15673	0.41411	0.99512	1.0379	0.053658	0.98524	0.99945	1.0041	0.00589
394.2649	7.02	bolomics Pc	0.25289	0.99951	1.1878	0.24827	0.087382	0.99998	0.81783	-0.29013	0.33265	0.99941	0.9014	-0.14976	0.093844	0.41171	0.73894	-0.43646	8.42E-06	0.000292	0.35101	-1.5104	0.008732	0.075866	0.60984	-0.7135	0.028781	0.34212	0.73464	-0.44488	0.079587	0.78343	0.79772	-0.32605	0.34202	0.61726	1.0766	0.10642
177.1084	0.58	domics Pos	0.25304	0.99987	1.0305	0.04337	0.51985	0.99994	1.0226	0.032231	0.36421	0.99996	1.0336	0.047616	0.19954	0.64478	1.0608	0.08522	0.11487	0.19349	1.069	0.096324	0.025997	0.14242	1.0892	0.12326	0.005685	0.3088	1.1918	0.25309	0.36866	0.96776	1.0328	0.046523	0.12569	0.23008	1.041	0.057925
1095.779	9.4	bolomics Pc	0.25322	0.99951	1.5468	0.62933	0.73007	0.99998	0.95597	-0.06497	0.10613	0.99941	1.2407	0.31113	0.24162	0.70401	0.78493	-0.34937	0.00799	0.040922	0.6368	-0.65109	0.0408	0.16268	0.72044	-0.47305	0.86422	0.9951	1.103	0.14141	0.6071	0.99956	1.3177	0.39799	0.095297	0.34969	1.8955	0.92255
757.0079	9.41	bolomics Pc	0.25326	0.99951	1.4311	0.51714	0.79419	0.99998	1.0405	0.057261	0.59751	0.99941	1.0759	0.10552	0.13215	0.5064	0.76017	-0.3956	0.018072	0.077663	0.6557	-0.60889	0.16768	0.42493	0.79038	-0.33939	0.89103	0.9951	1.0433	0.061144	0.85288	0.99956	1.0936	0.1291	0.04753	0.28861	1.8001	0.84805
753.5655	9.29	bolomics Pc	0.25353	0.99951	1.1891	0.24981	0.74466	0.99998	1.0419	0.059224	0.62381	0.99941	0.95215	-0.07073	0.35255	0.84525	1.1519	0.20408	0.574	0.95114	1.0646	0.090344	0.16613	0.42318	1.1748	0.23245	0.023498	0.32255	1.3312	0.41268	0.54414	0.99956	1.1544	0.20709	0.03957	0.28339	1.2579	0.33105
944.7324	0.54	domics Pos	0.25357	0.99987	0.88388	-0.17807	0.023834	0.99994	0.83324	-0.26319	0.037886	0.99996	0.74224	-0.43003	0.004703	0.17581	0.76613	-0.38433	0.000466	0.003119	0.61913	-0.69169	0.051726	0.22341	0.7467	-0.4214	0.007411	0.32129	0.77663	-0.36469	0.61312	0.97371	0.957	-0.06342	0.71913	0.79089	1.1347	0.18231
983.1575	9.11	bolomics Pc	0.25357	0.99951	1.3133	0.39325	0.77223	0.99998	0.93568	-0.09591	0.29232	0.99941	1.2076	0.2721	0.46528	0.94313	0.85742	-0.22193	0.038758	0.13882	0.6184	-0.69339	0.29249	0.58947	0.78198	-0.3548	0.6817	0.99391	1.0884	0.12219	0.93626	0.99956	1.0255	0.03639	0.33515	0.61137	1.2916	0.36919
1098.805	5.48	domics Pos	0.25401	0.99987	1.4445	0.53055	0.79627	0.99994	0.9408	-0.08805	0.54801	0.99996	1.0812	0.11265	0.13278	0.55699	0.71693	-0.4801	0.007453	0.024585	0.5095	-0.97286	0.14721	0.42205	0.71839	-0.47715	0.92452	0.98262	1.0338	0.047909	0.73394	0.97988	1.2491	0.32092	0.027211	0.1158	2.2241	1.1532
404.3097	2.06	domics Pos	0.25407	0.99987	1.1777	0.23593	0.42912	0.99994	0.88713	-0.17279	0.47535	0.99996	1.0785	0.10907	0.36828	0.82243	0.86531	-0.20871	0.000616	0.003839	0.30892	-1.6947	0.074744	0.28218	0.70818	-0.49782	0.74196	0.93154	0.94762	-0.07762	0.49832	0.96776	1.0681	0.095024	0.081511	0.18304	1.288	0.36518
963.1557	9.44	bolomics Pc	0.25411	0.99951	1.4093	0.495	0.40014	0.99998	0.91317	-0.13104	0.10265	0.99941	1.0928	0.12808	0.00919	0.19702	0.63299	-0.65975	0.004075	0.02532	0.65349	-0.61375	0.001614	0.045425	0.65843	-0.60291	0.56005	0.99391	0.93706	-0.09378	0.68084	0.99956	0.9721	-0.04082	0.027537	0.2688	1.761	0.8164
152.145	1.66	domics Pos	0.25449	0.99987	1.1858	0.24591	0.25507	0.99994	0.81936	-0.28743	0.81692	0.99996	1.0245	0.034988	0.29148	0.75244	0.83443	-0.26113	0.000213	0.001753	0.2786	-1.8437	0.028583	0.14869	0.61945	-0.69094	0.24759	0.68479	0.79161	-0.33715	0.8189	0.97988	0.95556	-0.06558	0.22001	0.32795	1.1968	0.25915
202.1814	0.34	bolomics Pc	0.25472	0.99951	1.1684	0.22459	0.26554	0.99998	0.83815	-0.25472	0.85496	0.99941	0.96748	-0.0477	0.15006	0.5457	0.74978	-0.41547	0.0097	0.047373	0.56075	-0.83456	0.031364	0.13639	0.62354	-0.68144	0.14072	0.70473	0.72816	-0.45767	0.38115	0.99956	0.87113	-0.19904	0.2619	0.53235	1.1861	0.24628
301.7566	6.38	bolomics Pc	0.25524	0.99951	1.1698	0.2263	0.20822	0.99998	0.86937	-0.20196	0.60089	0.99941	0.93781	-0.09263	0.28686	0.76772	0.85076	-0.23318	1.72E-06	0.000231	0.18523	-2.4326	0.018821	0.10152	0.58701	-0.76856	0.20949	0.80197	0.8171	-0.29141	0.81884	0.99956	0.97394	-0.0381	0.54097	0.7778	1.0622	0.087103
677.5196	2.05	domics Pos	0.25647	0.99987	1.3088	0.38822	0.22973	0.99994	0.81487	-0.29536	0.17824	0.99996	1.2362	0.30594	0.34902	0.80266	0.83641	-0.25771	2.24E-05	0.000392	0.12863	-2.9588	0.07951	0.29123	0.55351	-0.85333	0.2175	0.66052	0.72525	-0.46345	0.5448	0.96776	1.0838	0.11604	0.009733	0.094875	1.7558	0.81214
395.2486	2.05	domics Pos	0.2565	0.99987	1.1986	0.26141	0.97672	0.99994	0.99578	-0.0061	0.1664	0.99996	1.2017	0.26509	0.98454	0.99652	1.0217	0.030985	8.04E-05	0.000888	0.22548	-2.149	0.33343	0.6073	0.8499	-0.23464	0.86904	0.97532	1.0406	0.057456	0.032234	0.62711	1.2971	0.37523	0.000973	0.07528	1.8748	0.90672
942.1241	4.65	domics Pos	0.25681	0.99987	1.5432	0.62588	0.48586	0.99994	0.917	-0.12501	0.29591	0.99996	1.0822	0.11391	0.045254	0.36714	0.66446	-0.58976	0.001319	0.006694	0.44385	-1.1719	0.021268	0.12671	0.68814	-0.53923	0.80093	0.95377	1.1007	0.13836	0.64724	0.97988	1.2447	0.31585	0.040313	0.13378	2.2608	1.1768
1049.746	5.54	domics Pos	0.25713	0.99987	1.3613	0.44501	0.8369	0.99994	0.96624	-0.04955	0.25057	0.99996	1.1941	0.25594	0.1227	0.54337	0.72651	-0.46095	0.01759	0.047232	0.56418	-0.82577	0.17514	0.46151	0.76516	-0.38616	0.6865	0.90732	1.1383	0.18686	0.81789	0.97988	1.0931	0.12838	0.030544	0.11984	2.4971	1.3202
1097.8	9.74	bolomics Pc	0.25737	0.99951	1.3246	0.40559	0.5308	0.99998	0.8734	-0.19529	0.71964	0.99941	1.0422	0.059629	0.060087	0.32244	0.64108	-0.64143	0.040165	0.14235	0.62721	-0.67297	0.042735	0.16794	0.62357	-0.68137	0.696	0.99391	0.93802	-0.09231	0.91335	0.99956	1.0304	0.043173	0.064334	0.30632	1.9251	0.94495
984.1603	9.11	bolomics Pc	0.25806	0.99951	1.3285	0.40981	0.58241	0.99998	0.89758	-0.15588	0.26863	0.99941	1.1628	0.21758	0.31939	0.81415	0.8058	-0.31151	0.036311	0.13246	0.64951	-0.62257	0.26305	0.55407	0.77853	-0.36117	0.92336	0.9951	1.027	0.038431	0.94553	0.99956	1.0521	0.073249	0.40655	0.67967	1.4142	0.5
774.4069	0.54	domics Pos	0.25826	0.99987	1.6073	0.68467	0.53021	0.99994	0.91787	-0.12364	0.94518	0.99996	0.98974	-0.01488	0.03642	0.33474	0.5628	-0.82931	0.001674	0.007899	0.43714	-1.1938	0.003532	0.066675	0.57992	-0.78608	0.43635	0.79604	0.87345	-0.1952	0.76241	0.97988	0.98324	-0.02439	0.009363	0.092975	1.9694	0.97774
773.9438	3.26	bolomics Pc	0.25835	0.99951	1.221	0.2881	0.38423	0.99998	0.85777	-0.22134	0.74332	0.99941	0.94256	-0.08535	0.024746	0.23087	0.62185	-0.68536	0.014439	0.064334	0.58531	-0.77273	0.017376	0.097219	0.6541	-0.61243	0.40604	0.98164	0.85211	-0.23089	0.98362	0.99956	0.99529	-0.00681	0.077603	0.32314	1.688	0.75531
1178.871	9.6	bolomics Pc	0.25846	0.99951	1.4687	0.55456	0.9936	0.99998	0.99762	-0.00344	0.96462	0.99941	0.97979	-0.02946	0.16958	0.58317	0.72804	-0.4579	0.019492	0.082193	0.5841	-0.77571	0.02433	0.11577	0.62553	-0.67685	0.83627	0.9951	0.97297	-0.03954	0.35811	0.99956	0.86515	-0.20897	0.058826	0.30404	1.5386	0.6216
670.4633	9.56	bolomics Pc	0.25849	0.99951	1.3221	0.4028	0.56502	0.99998	0.92827	-0.10738	0.33607	0.99941	1.1097	0.15019	0.01478	0.2033	0.69148	-0.53224	0.002387	0.017364	0.58484	-0.7739	0.078314	0.25224	0.71902	-0.4759	0.98938	0.99889	1.0507	0.071334	0.84302	0.99956	0.99921	-0.00114	0.069129	0.3118	1.7433	0.80178
1129.741	10.05	domics Pos	0.25853	0.99987	1.1601	0.21426	0.97768	0.99994	1.0153	0.021941	0.57706	0.99996	1.0675	0.094207	0.35973	0.81404	0.93793	-0.09245	0.016504	0.044886	0.83429	-0.26138	0.18057	0.46838	0.86531	-0.20872	0.75202	0.93661	1.051	0.07182	0.97649	0.9929	1.005	0.007239	0.000314	0.07528	1.4055	0.49106
853.5592	9.23	bolomics Pc	0.25911	0.99951	1.3553	0.43862	0.77839	0.99998	0.94239	-0.08561	0.47089	0.99941	1.086	0.11908	0.28501	0.76537	0.8012	-0.31977	0.043601	0.15126	0.63816	-0.64801	0.092358	0.28263	0.70833	-0.4975	0.72096	0.99391	0.94104	-0.08767	0.59441	0.99956	0.90961	-0.13668	0.21327	0.4802	1.5113	0.59576
503.2743	3.23	bolomics Pc	0.25924	0.99951	1.2521	0.32439	0.41964	0.99998	0.91368	-0.13023	0.68425	0.99941	0.94981	-0.07429	0.030746	0.24688	0.65024	-0.62095	0.003954	0.02481	0.57426	-0.80023	0.011939	0.083709	0.65486	-0.61073	0.36033	0.96545	0.88262	-0.18013	0.70311	0.99956	0.9622	-0.05559	0.028779	0.2688	1.6624	0.73327
1123.741	10.07	domics Pos	0.25956	0.99987	0.88336	-0.17893	0.020998	0.99994	0.76595	-0.38468	0.14905	0.99996	0.85659	-0.22332	0.27887	0.74464	0.86018	-0.21729	0.002612	0.011031	0.66597	-0.58646	0.46537	0.68992	0.92036	-0.11972	0.31368	0.72948	0.88135	-0.18222	0.53222	0.96776	0.92526	-0.11208	0.1032	0.20592	1.2028	0.26634
291.269	6.63	bolomics Pc	0.25994	0.99951	1.162	0.21659	0.75098	0.99998	0.96197	-0.05594	0.51589	0.99941	1.104	0.14274	0.14953	0.54537	0.77271	-0.37201	3.18E-06	0.000231	0.21477	-2.2191	0.007613	0.072236	0.48898	-1.0322	0.10115	0.61631	0.7533	-0.4087	0.51795	0.99956	0.91453	-0.12889	0.18442	0.44858	1.1337	0.18103
332.2651	6.53	bolomics Pc	0.25996	0.99951	0.80997	-0.30406	0.31538	0.99998	0.8253	-0.27701	0.002955	0.99941	0.60129	-0.73386	0.011649	0.19869	0.46911	-1.092	0.001676	0.013602	0.35183	-1.507	0.002593	0.046402	0.25752	-1.9573	0.006562	0.21955	0.32139	-1.6376	0.008313	0.47452	0.34966	-1.516	0.006447	0.2688	0.35828	-1.4808
1005.672	9.09	bolomics Pc	0.26027	0.99951	1.2444	0.31543	0.5234	0.99998	0.90666	-0.14136	0.53341	0.99941	1.0651	0.091023	0.27987	0.7548	0.80953	-0.30483	0.024187	0.097806	0.62958	-0.66753	0.23727	0.52746	0.78605	-0.3473	0.77499	0.99391	0.96462	-0.05196	0.73454	0.99956	0.98092	-0.0278	0.46421	0.7264	1.2701	0.3449
453.2909	1.65	domics Pos	0.26088	0.99987	1.2258	0.29373	0.61925	0.99994	0.93773	-0.09275	0.55801	0.99996	0.92857	-0.10691	0.095913	0.51551	0.75096	-0.4132	0.000231	0.001848	0.27052	-1.8862	0.005198	0.074135	0.61742	-0.69566	0.46424	0.80647	0.88419	-0.17757	0.10612	0.86278	0.82567	-0.27636	0.21599	0.32475	1.3032	0.3821
1050.203	9.37	bolomics Pc	0.26092	0.99951	1.4327	0.51875	0.68847	0.99998	0.94723	-0.07821	0.9568	0.99941	0.99449	-0.00797	0.097305	0.41658	0.75631	-0.40295	0.028892	0.11167	0.63772	-0.64901	0.012978	0.085844	0.623	-0.6827	0.70245	0.99391	1.0002	0.000312	0.47454	0.99956	0.88973	-0.16856	0.056091	0.29896	1.7383	0.79767
195.1028	4.38	bolomics Pc	0.26093	0.99951	0.8906	-0.16715	0.11253	0.99998	0.82219	-0.28246	0.33455	0.99941	0.88518	-0.17596	0.52897	0.99205	0.93375	-0.09889	0.45963	0.86129	0.92347	-0.11487	0.0283	0.12757	0.74729	-0.42026	0.17588	0.74875	0.80477	-0.31334	0.047994	0.67731	0.80747	-0.30852	0.032348	0.2688	0.77633	-0.36525
386.7259	0.34	olomics Ne	0.26111	0.99918	0.93407	-0.0984	0.032121	0.43934	0.82231	-0.28224	0.71225	0.99995	0.97166	-0.04148	0.068579	0.99985	0.87676	-0.18975	0.30779	0.99927	1.1097	0.15019	0.82507	0.96067	0.99483	-0.00747	0.093477	0.45845	1.1633	0.2182	0.58741	0.99512	0.95946	-0.05971	0.43148	0.99941	0.95729	-0.06298
1185.851	9.65	bolomics Pc	0.2615	0.99951	1.3652	0.4491	0.25637	0.99998	0.81286	-0.29892	0.53145	0.99941	1.085	0.11775	0.031989	0.24911	0.61775	-0.6949	0.00458	0.027853	0.50845	-0.97581	0.015004	0.091774	0.59171	-0.75703	0.6784	0.99391	0.93463	-0.09753	0.96712	0.99956	1.0914	0.12612	0.1926	0.45685	1.6391	0.71286
432.9767	12.01	olomics Ne	0.26178	0.99918	0.93057	-0.10381	0.019678	0.40092	0.79438	-0.33209	0.25814	0.99995	0.87293	-0.19606	0.050927	0.99985	0.81381	-0.29723	0.27864	0.99927	0.89138	-0.16588	0.42148	0.81323	1.119	0.16217	0.62054	0.87098	0.95145	-0.0718	0.86002	0.99512	0.99207	-0.01148	0.43821	0.99941	0.91319	-0.13101
987.1537	4.58	domics Pos	0.26199	0.99987	1.3426	0.42504	0.64134	0.99994	0.89661	-0.15744	0.876	0.99996	1.0061	0.008833	0.032931	0.31629	0.57874	-0.78902	0.003434	0.013599	0.44752	-1.16	0.035567	0.17123	0.60524	-0.72442	0.63949	0.88465	0.89914	-0.15339	0.62062	0.97813	0.91018	-0.13578	0.060725	0.1578	1.9502	0.96364
220.0688	0.58	domics Pos	0.26202	0.99987	0.85505	-0.22592	0.011012	0.99994	0.73627	-0.4417	0.14164	0.99996	0.84409	-0.24452	0.73579	0.97097	1.0347	0.049244	0.23824	0.33259	1.1484	0.19964	0.1723	0.45896	1.3848	0.46966	0.0108	0.3324	1.7962	0.84495	0.59523	0.97073	1.0764	0.10619	0.009229	0.092545	1.3493	0.4322
1053.774	9.78	bolomics Pc	0.26202	0.99951	1.3895	0.47454	0.3029	0.99998	0.84188	-0.24831	0.97522	0.99941	0.98716	-0.01865	0.009635	0.19702	0.6315	-0.66315	0.008211	0.041648	0.57755	-0.79197	0.031198	0.13593	0.66628	-0.58579	0.47283	0.99391	0.88134	-0.18223	0.93732	0.99956	1.1202	0.16374	0.083832	0.33342	1.9151	0.93741
975.149	4.6	domics Pos	0.26345	0.99987	1.4935	0.57868	0.25109	0.99994	0.83312	-0.26341	0.91649	0.99996	1.0145	0.020831	0.063558	0.43296	0.72801	-0.45796	0.004978	0.018199	0.45944	-1.1221	0.024418	0.13732	0.67952	-0.5574	0.5051	0.8305	0.89095	-0.16658	0.81882	0.97988	0.98214	-0.026	0.065627	0.16491	1.8919	0.91981
597.8558	3.05	bolomics Pc	0.26356	0.99951	1.3359	0.41783	0.031151	0.99998	0.7679	-0.38102	0.10894	0.99941	0.88588	-0.17482	0.000863	0.12936	0.57215	-0.80555	0.000121	0.001856	0.55111	-0.85958	0.000949	0.042705	0.52288	-0.93546	0.62944	0.99391	0.95352	-0.06867	0.45771	0.99956	0.90231	-0.1483	0.051983	0.2951	1.6772	0.74606
304.2621	6.39	bolomics Pc	0.26422	0.99951	1.0958	0.13198	0.45413	0.99998	0.93568	-0.09591	0.41611	0.99941	0.93161	-0.1022	0.1131	0.45616	0.82432	-0.27873	4.95E-06	0.000247	0.22176	-2.1729	0.02089	0.10792	0.62845	-0.67012	0.040813	0.40175	0.75179	-0.41159	0.044057	0.65531	0.83673	-0.25716	0.46848	0.72982	0.91866	-0.12239
199.8085	12.27	olomics Ne	0.26435	0.99918	0.97296	-0.03955	0.034929	0.44974	0.90115	-0.15016	0.9955	0.99995	1.0015	0.002175	0.39371	0.99985	0.9642	-0.0526	0.94772	0.99927	1.0103	0.014783	0.14398	0.6202	1.0589	0.082633	0.012403	0.2716	1.1131	0.15464	0.69913	0.99512	0.9849	-0.02195	0.86958	0.99941	0.99554	-0.00645
612.8825	12.05	bolomics Pc	0.26453	0.99951	0.87753	-0.18847	0.831	0.99998	1.0485	0.068267	0.10217	0.99941	0.85144	-0.23202	0.14744	0.54301	0.84087	-0.25004	0.87456	0.99688	1.0318	0.045134	0.69731	0.91033	1.0346	0.049124	0.57508	0.99391	1.1051	0.14421	0.023821	0.51812	0.86155	-0.215	0.76886	0.90022	0.98504	-0.02174
425.2497	7.49	bolomics Pc	0.26459	0.99951	0.88804	-0.17131	0.34249	0.99998	0.87893	-0.18619	0.008818	0.99941	0.76622	-0.38417	0.007583	0.1948	0.61126	-0.71014	0.000974	0.009119	0.6383	-0.6477	0.003383	0.049867	0.51408	-0.95993	0.001357	0.16989	0.56176	-0.83198	0.001142	0.47452	0.52351	-0.9337	0.008754	0.2688	0.57838	-0.78992
664.7373	0.56	domics Pos	0.26464	0.99987	1.1889	0.24962	0.74298	0.99994	1.0321	0.045582	0.40204	0.99996	0.93282	-0.10034	0.063696	0.43296	0.8072	-0.309	0.02698	0.065442	0.73852	-0.43729	0.008133	0.084185	0.79738	-0.32666	0.36119	0.75755	0.92981	-0.105	0.58287	0.97073	1.0451	0.063651	0.04171	0.13617	1.3174	0.39772
274.9792	4.01	olomics Ne	0.26465	0.99918	1.0518	0.07285	0.047007	0.47152	0.90925	-0.13725	0.92687	0.99995	0.99644	-0.00514	0.87396	0.99985	1.0081	0.011617	0.88492	0.99927	0.99422	-0.00837	0.2492	0.68628	1.0639	0.089323	0.21344	0.59066	1.0536	0.075333	0.92341	0.99512	1.0043	0.006143	0.77521	0.99941	0.9892	-0.01566
461.1902	2.3	domics Pos	0.26502	0.99987	0.93779	-0.09267	0.053027	0.99994	0.86087	-0.21614	0.000239	0.45477	0.69241	-0.53029	0.007071	0.19397	0.61958	-0.69063	0.000395	0.002744	0.55588	-0.84716	0.011274	0.091488	0.61276	-0.70661	0.030617	0.35576	0.79142	-0.33748	0.006608	0.52955	0.64583	-0.63078	0.1013	0.20314	0.81975	-0.28675
729.162	1.09	domics Pos	0.2652	0.99987	1.5087	0.59333	0.37813	0.99994	1.1773	0.23546	0.27279	0.99996	1.2522	0.3245	0.37622	0.82708	1.2053	0.26933	0.66888	0.71945	0.8872	-0.17266	0.27292	0.55982	1.4054	0.49102	0.024428	0.33809	1.6201	0.69609	0.16989	0.91806	1.2791	0.35507	0.060201	0.15775	1.4943	0.5795
534.294	2.94	bolomics Pc	0.26547	0.99951	1.203	0.26667	0.41385	0.99998	0.87128	-0.19879	0.48916	0.99941	0.88926	-0.16933	0.028579	0.24036	0.57478	-0.79892	0.00338	0.022416	0.55698	-0.84431	0.073996	0.24382	0.7469	-0.42101	0.64939	0.99391	0.95794	-0.062	0.64825	0.99956	0.92409	-0.11389	0.081534	0.3314	1.4406	0.52667
961.647	4.63	domics Pos	0.26553	0.99987	1.3306	0.41211	0.43075	0.99994	0.8072	-0.30901	0.6525	0.99996	1.0996	0.13694	0.17277	0.60551	0.68427	-0.54737	0.027837	0.067093	0.46287	-1.1113	0.13636	0.40368	0.66926	-0.57936	0.78125	0.94714	0.92993	-0.10481	0.95015	0.98838	1.0314	0.044655	0.10123	0.20314	1.953	0.96567
817.4306	0.54	domics Pos	0.26582	0.99987	1.522	0.60597	0.23693	0.99994	0.86279	-0.21292	0.73176	0.99996	0.96158	-0.05653	0.030877	0.3066	0.56419	-0.82576	0.000519	0.0034	0.36479	-1.4549	0.001071	0.043236	0.53575	-0.90036	0.41734	0.78082	0.89041	-0.16746	0.73863	0.97988	0.99126	-0.01267	0.007997	0.092077	1.9956	0.99681
890.7054	5.4	domics Pos	0.26606	0.99987	0.92012	-0.12011	0.26177	0.99994	0.92298	-0.11563	0.53397	0.99996	0.96318	-0.05413	0.057629	0.4146	0.88532	-0.17573	0.000825	0.00483	0.72587	-0.46222	0.063594	0.25311	0.85926	-0.21884	0.58893	0.86163	1.0583	0.081739	0.15094	0.91321	0.90592	-0.14255	0.12315	0.22751	1.2079	0.27255
1048.69	8.85	bolomics Pc	0.26609	0.99951	1.3053	0.38442	0.47909	0.99998	0.90691	-0.14097	0.96787	0.99941	0.99659	-0.00493	0.010324	0.19754	0.70894	-0.49626	0.014382	0.064259	0.63745	-0.64962	0.007641	0.072276	0.65643	-0.60729	0.76827	0.99391	0.98184	-0.02643	0.70097	0.99956	1.1018	0.13983	0.06003	0.30404	1.7583	0.81416
912.7776	10.08	domics Pos	0.2671	0.99987	1.0767	0.10663	0.23341	0.99994	0.93239	-0.10099	0.74676	0.99996	0.97966	-0.02965	0.11488	0.53485	0.87617	-0.19072	0.005057	0.018398	0.74845	-0.41802	0.46923	0.69321	0.94777	-0.07739	0.56006	0.85488	0.96215	-0.05567	0.49447	0.96776	0.95697	-0.06346	0.10221	0.20447	1.1438	0.19387
438.1973	0.75	domics Pos	0.26715	0.99987	1.1882	0.24876	0.34495	0.99994	0.83719	-0.25636	0.69251	0.99996	0.92964	-0.10526	0.78219	0.97526	1.0636	0.088959	0.031351	0.073294	0.60106	-0.73441	0.40268	0.64785	0.84147	-0.24902	0.66385	0.89693	0.92415	-0.1138	0.66232	0.97988	0.92664	-0.10991	0.18094	0.28819	1.3167	0.39697
228.9566	10.03	domics Pos	0.26719	0.99987	1.0622	0.087096	0.27783	0.99994	0.9129	-0.13148	0.49734	0.99996	0.94699	-0.07859	0.3795	0.8285	0.93928	-0.09038	0.02495	0.061696	0.81456	-0.2959	0.33575	0.60864	0.9116	-0.13352	0.52549	0.83957	0.95437	-0.06738	0.94762	0.98838	1.004	0.005688	0.034776	0.12689	1.1685	0.22472
321.7674	2.06	domics Pos	0.26757	0.99987	1.1773	0.23551	0.56414	0.99994	0.90487	-0.14422	0.60115	0.99996	1.0567	0.079516	0.2631	0.72224	0.77856	-0.36112	0.000237	0.001888	0.14705	-2.7656	0.027353	0.14587	0.56692	-0.8188	0.37732	0.76056	0.8293	-0.27004	0.86152	0.98023	0.96477	-0.05174	0.046642	0.14319	1.3977	0.48308
680.279	5.19	olomics Ne	0.26758	0.99918	1.1266	0.172	0.95893	0.99363	0.99852	-0.00214	0.14109	0.99995	1.1613	0.21575	0.20175	0.99985	1.1618	0.21636	0.30087	0.99927	1.1171	0.15977	0.00671	0.36417	1.3777	0.46224	0.006872	0.2539	1.3757	0.46014	0.068475	0.99512	1.1984	0.26111	0.06236	0.99941	1.1881	0.24865
1023.694	8.86	bolomics Pc	0.2676	0.99951	1.3538	0.43704	0.62109	0.99998	0.95413	-0.06775	0.15181	0.99941	1.1082	0.14823	0.26271	0.73701	0.84863	-0.23679	0.007017	0.037008	0.76009	-0.39577	0.011696	0.083709	0.74537	-0.42397	0.21195	0.80537	1.1903	0.25136	0.43244	0.99956	1.263	0.33685	0.058247	0.30327	1.711	0.77484
516.3165	1.3	domics Pos	0.26768	0.99987	1.2484	0.32006	0.7902	0.99994	1.033	0.046862	0.58686	0.99996	1.1316	0.17833	0.83388	0.98621	1.0448	0.063223	0.53854	0.60676	0.8611	-0.21575	0.35373	0.61898	1.1603	0.21447	0.046088	0.39255	1.5082	0.59281	0.10264	0.85564	1.3669	0.45092	0.02398	0.1107	1.7941	0.84328
446.3585	2.05	domics Pos	0.26802	0.99987	1.301	0.37957	0.96295	0.99994	0.97544	-0.03587	0.31204	0.99996	1.1919	0.25331	0.97812	0.99625	1.0028	0.003981	0.00092	0.005233	0.29033	-1.7842	0.12337	0.38281	0.71509	-0.4838	0.83174	0.96593	1.0363	0.051382	0.59108	0.97073	1.0635	0.088825	0.009914	0.094875	1.6429	0.71621
938.6251	8.91	bolomics Pc	0.26819	0.99951	1.3631	0.4469	0.55148	0.99998	0.93105	-0.10307	0.33868	0.99941	1.1096	0.15005	0.019521	0.21575	0.71108	-0.49193	0.009145	0.045151	0.61098	-0.71079	0.10421	0.30909	0.8069	-0.30954	0.66128	0.99391	1.1343	0.18182	0.76097	0.99956	1.0715	0.099602	0.083359	0.33309	1.7018	0.76705
294.2804	6.21	bolomics Pc	0.26876	0.99951	1.2089	0.27371	0.24268	0.99998	0.80492	-0.31309	0.67979	0.99941	1.0355	0.050281	0.14871	0.54533	0.68873	-0.53798	0.007804	0.040034	0.39942	-1.324	0.004387	0.055952	0.54475	-0.87635	0.19687	0.77907	0.77086	-0.37545	0.71482	0.99956	0.95027	-0.07359	0.91006	0.96262	1.0626	0.087593
637.243	1.26	domics Pos	0.26913	0.99987	1.1313	0.17793	0.4267	0.99994	1.0547	0.076782	0.34777	0.99996	1.1125	0.15381	0.93819	0.99067	0.99609	-0.00565	0.022357	0.056719	0.7797	-0.35901	0.62346	0.79891	1.094	0.1296	0.46602	0.80647	0.94697	-0.07861	0.010047	0.52955	1.1888	0.24952	0.076042	0.17781	1.351	0.43407
180.1867	2.05	domics Pos	0.26916	0.99987	1.163	0.21782	0.10004	0.99994	0.81043	-0.30325	0.56721	0.99996	0.93692	-0.09401	0.018562	0.25459	0.65854	-0.60266	4.53E-05	0.000591	0.19669	-2.346	0.002631	0.061655	0.48958	-1.0304	0.004326	0.3088	0.60111	-0.73429	0.005469	0.52955	0.78508	-0.34909	0.50949	0.6102	1.0378	0.053552
712.4868	9.46	bolomics Pc	0.26922	0.99951	1.3346	0.41645	0.22014	0.99998	0.87392	-0.19443	0.3684	0.99941	1.0684	0.095477	0.013781	0.20085	0.67706	-0.56265	0.000487	0.005589	0.57454	-0.79953	0.010718	0.08175	0.6867	-0.54225	0.76249	0.99391	0.98338	-0.02418	0.99885	0.99956	1.0671	0.093673	0.077083	0.32215	1.7486	0.80624
415.2121	0.75	domics Pos	0.2697	0.99987	1.2026	0.26615	0.23459	0.99994	0.77803	-0.36211	0.75948	0.99996	0.93356	-0.09919	0.66776	0.95717	1.1042	0.14295	0.04012	0.088376	0.61579	-0.69949	0.63042	0.80422	0.90766	-0.13977	0.76141	0.94108	0.93601	-0.0954	0.87487	0.98023	0.96934	-0.04493	0.19767	0.30601	1.37	0.45416
555.316	3.32	bolomics Pc	0.27023	0.99951	1.2207	0.28769	0.33546	0.99998	0.82351	-0.28015	0.71225	0.99941	1.0915	0.12629	0.013969	0.20085	0.57789	-0.79113	0.013424	0.060844	0.56835	-0.81514	0.028905	0.12915	0.6306	-0.66521	0.60847	0.99391	0.90545	-0.1433	0.77502	0.99956	0.96937	-0.04488	0.058975	0.30404	1.848	0.88593
836.6105	5.24	domics Pos	0.27077	0.99987	1.0949	0.1308	0.97573	0.99994	0.9985	-0.00216	0.94871	0.99996	0.9942	-0.00839	0.59359	0.93028	0.95561	-0.0655	0.010525	0.031951	0.76745	-0.38185	0.19063	0.47727	0.84436	-0.24408	0.55345	0.85214	0.93956	-0.08994	0.7084	0.97988	0.96743	-0.04778	0.020915	0.10765	1.2684	0.34303
712.9825	9.46	bolomics Pc	0.27078	0.99951	1.2819	0.35827	0.027549	0.99998	0.81262	-0.29934	0.87832	0.99941	0.98958	-0.01511	0.006467	0.1948	0.6224	-0.68408	0.000458	0.00534	0.58065	-0.78427	0.023645	0.11397	0.68569	-0.54437	0.49574	0.99391	0.91539	-0.12755	0.57957	0.99956	0.9367	-0.09434	0.079075	0.32476	1.6341	0.70847
826.5589	8.59	olomics Ne	0.27082	0.99918	1.0385	0.054525	0.12706	0.54655	0.93148	-0.1024	0.48267	0.99995	0.96471	-0.05183	0.96767	0.99985	1.0037	0.005299	0.13834	0.99927	1.0828	0.11483	8.85E-05	0.061927	1.2949	0.3728	0.001407	0.22316	1.2178	0.28426	0.22568	0.99512	1.0887	0.12264	0.20572	0.99941	1.056	0.078587
387.2743	2.72	domics Pos	0.27108	0.99987	0.92149	-0.11796	0.19247	0.99994	0.8491	-0.236	0.009606	0.99996	0.81707	-0.29146	0.00234	0.17581	0.58628	-0.77034	0.00022	0.001798	0.46644	-1.1002	0.011154	0.091465	0.45503	-1.136	0.091611	0.52136	0.61446	-0.70261	0.036561	0.64014	0.59	-0.76121	0.13351	0.23832	0.76217	-0.39182
762.8971	12.03	bolomics Pc	0.27182	0.99951	0.89605	-0.15835	0.064774	0.99998	0.70853	-0.4971	0.55971	0.99941	[illegible]	-0.29548	0.093395	0.41089	0.82464	-0.27817	0.23807	0.56341	0.86884	-0.20283	0.11759	0.33359	0.80426	-0.31427	0.14059	0.70473	0.82411	-0.2791	0.044638	0.65705	0.76812	-0.3806	0.14779	0.40018	0.78399	-0.35109
663.9997	9.52	bolomics Pc	0.27228	0.99951	1.4032	0.48877	0.99556	0.99998	0.98451	-0.02253	0.5518	0.99941	1.0874	0.1209	0.60964	0.3437	0.73858	-0.43717	0.032878	0.13288	0.67547	-0.56604	0.037728	0.15451	0.67851	-0.55957	0.87278	0.9951	1.0496	0.069814	0.98653	0.99956	1.1031	0.14155	0.075557	0.32186	1.7159	0.77899
421.7943	5.94	bolomics Pc	0.27236	0.99951	1.2288	0.2972	0.8861	0.99998	1.0154	0.022101	0.73466	0.99941	0.96172	-0.05632	0.29829	0.7896	0.83141	-0.26636	0.001831	0.014605	0.3258	-1.6179	0.10166	0.30352	0.72858	-0.45683	0.13405	0.68208	0.82426	-0.27883	0.52773	0.99956	0.91432	-0.12924	0.11933	0.36992	1.2748	0.35025
408.9736	5.1	olomics Ne	0.27237	0.99918	1.1182	0.16115	0.17428	0.60009	0.90676	-0.1412	0.49087	0.99995	1.1068	0.14637	0.89053	0.99985	0.9972	-0.00404	0.30831	0.99927	1.1046	0.14348	0.22499	0.66171	1.1888	0.24956	0.023121	0.32715	1.2265	0.29452	0.22297	0.99512	1.2187	0.28536	0.54961	0.99941	1.0782	0.10856
262.992	3.61	olomics Ne	0.27241	0.99918	0.94802	-0.07702	0.014407	0.37315	0.86774	-0.20467	0.36811	0.99995	0.95621	-0.0646	0.38192	0.99985	1.0584	0.081905	0.42217	0.99927	0.95386	-0.06816	0.30779	0.72837	1.065	0.090885	0.36958	0.721	1.0338	0.047927	0.63114	0.99512	0.97825	-0.03173	0.14059	0.99941	0.92432	-0.11353
1073.188	4.2	domics Pos	0.27265	0.99987	1.4683	0.55418	0.7347	0.99994	0.89059	-0.16717	0.51714	0.99996	1.1266	0.17192	0.44081	0.86677	0.80276	-0.31697	0.042521	0.092279	0.52514	-0.92924	0.11007	0.35477	0.65332	-0.61433	0.93998	0.98682	1.0098	0.014105	0.59452	0.97073	1.1592	0.21317	0.14088	0.24586	1.8134	0.85873

755.0279	9.41	bolomics Po	0.27279	0.99951	1.3087	0.38817	0.27267	0.99998	0.88699	-0.17301	0.07639	0.99941	1.1862	0.24636	0.035729	0.25923	0.71041	-0.49328	0.001915	0.015046	0.62121	-0.68686	0.001939	0.045425	0.61828	-0.69366	0.7999	0.99391	1.0114	0.016406	0.90031	0.99956	1.0994	0.13666	0.1051	0.35957	1.7123	0.77591
626.4394	9.62	bolomics Po	0.27306	0.99951	1.4434	0.52942	0.62382	0.99998	0.90491	-0.14415	0.90528	0.99941	0.99887	-0.00163	0.064636	0.33323	0.69619	-0.52244	0.007023	0.037008	0.55157	-0.85837	0.085053	0.26738	0.70448	-0.50537	0.92162	0.9951	1.0713	0.099323	0.85644	0.99956	1.0434	0.061236	0.062538	0.30404	1.7618	0.81702
526.9759	5.09	olomics Ne	0.27314	0.99918	1.057	0.080041	0.9119	0.9934	1.0084	0.012002	0.44604	0.99995	1.0512	0.071987	0.38618	0.99985	1.0703	0.097969	0.19805	0.99927	1.0723	0.10068	0.06773	0.51869	1.1194	0.16272	0.029575	0.34346	1.1601	0.21422	0.091294	0.99512	1.1086	0.14879	0.18589	0.99941	1.0729	0.10148
123.0812	4.38	bolomics Po	0.27408	0.99951	0.89573	-0.15886	0.15156	0.99998	0.84144	-0.24907	0.39387	0.99941	0.92186	-0.11739	0.87929	0.99905	1.0099	0.014257	0.86095	0.99688	1.0154	0.02202	0.038232	0.1558	0.74418	-0.42627	0.38555	0.97696	0.89591	-0.15858	0.089198	0.80182	0.83319	-0.26329	0.046121	0.28516	0.78645	-0.34656
578.9743	0.52	domics Pos	0.27417	0.99987	1.1588	0.21257	0.13016	0.99994	0.78681	-0.34591	0.77856	0.99996	0.97162	-0.04154	0.014218	0.22898	0.75227	-0.41069	0.006372	0.021828	0.62469	-0.67879	0.033811	0.16507	0.7904	-0.33934	0.73024	0.92659	1.027	0.038441	0.58779	0.97073	0.93407	-0.0984	0.13297	0.23793	1.4403	0.52641
130.0843	0.6	lomics Neg	0.27425	0.99977	0.78794	-0.34384	0.25274	0.99904	0.75735	-0.40096	0.79225	0.99994	1.0523	0.073571	0.04055	0.58182	1.831	0.87261	0.18775	0.99989	1.2927	0.37039	0.42428	0.99653	1.2055	0.26967	0.054093	0.40029	1.4036	0.48915	0.48382	0.72003	0.86404	-0.21083	0.011909	0.38536	0.55048	-0.86125
1053.19	4.49	domics Pos	0.27437	0.99987	1.3722	0.45648	0.20105	0.99994	0.78963	-0.34076	0.91578	0.99996	0.96225	-0.05552	0.13931	0.56536	0.69328	-0.5285	0.002747	0.011458	0.46137	-1.116	0.050545	0.22026	0.68302	-0.55001	0.77897	0.94624	0.94562	-0.08068	0.98575	0.99515	1.0363	0.051484	0.14242	0.24738	1.7921	0.84162
1052.72	9.41	bolomics Po	0.27514	0.99951	1.3704	0.45456	0.58202	0.99998	0.91172	-0.13334	0.61732	0.99941	1.0469	0.066109	0.029767	0.24494	0.62951	-0.6677	0.005094	0.029826	0.5684	-0.81502	0.14318	0.38352	0.71115	-0.49177	0.45939	0.99391	0.88166	-0.1817	0.49571	0.99956	0.89469	-0.16054	0.14175	0.39464	1.643	0.71633
266.8621	0.57	domics Pos	0.27528	0.99987	1.1228	0.16704	0.88623	0.99994	0.99557	-0.0064	0.78767	0.99996	0.98699	-0.01889	0.06622	0.43847	0.87175	-0.19801	0.004316	0.016212	0.80888	-0.30601	0.31389	0.59309	0.93299	-0.10007	0.41523	0.78082	0.91498	-0.1282	0.52423	0.96776	0.96641	-0.04929	0.1942	0.30236	1.1108	0.15158
1051.168	8.85	bolomics Po	0.27566	0.99951	1.367	0.45099	0.93557	0.99998	1.0506	0.071154	0.97311	0.99941	1.0158	0.022594	0.047729	0.28918	0.82111	-0.28435	0.06158	0.19574	0.68809	-0.53933	0.19962	0.47343	0.85857	-0.22	0.75813	0.99391	1.1475	0.19849	0.63152	0.99956	1.1274	0.17301	0.092777	0.34627	1.9104	0.93386
641.88	3.11	bolomics Po	0.27585	0.99951	1.3913	0.47639	0.2398	0.99998	0.85662	-0.22327	0.70686	0.99941	1.0359	0.050883	0.006174	0.1948	0.61235	-0.70757	0.001173	0.010497	0.49535	-1.0135	0.01672	0.095763	0.60059	-0.73556	0.74272	0.99391	0.96923	-0.04508	0.47699	0.99956	0.89622	-0.15808	0.017455	0.2688	1.7963	0.84503
965.7229	5.68	domics Pos	0.27602	0.99987	1.3021	0.38086	0.35057	0.99994	0.8412	-0.24948	0.87738	0.99996	1.0302	0.042903	0.097914	0.51762	0.71746	-0.47904	0.00354	0.013901	0.49603	-1.0115	0.05307	0.22635	0.64908	-0.62352	0.67632	0.90042	0.94637	-0.07953	0.79241	0.97988	0.98238	-0.02565	0.10081	0.20284	2.0089	1.0064
644.8665	3.1	bolomics Po	0.27638	0.99951	1.1815	0.24061	0.16178	0.99998	0.8821	-0.18098	0.12126	0.99941	0.88948	-0.16896	0.003791	0.17363	0.63546	-0.65412	0.002227	0.016741	0.58181	-0.78137	0.000538	0.039708	0.54281	-0.88147	0.51189	0.99391	0.92597	-0.11097	0.32472	0.99956	0.87369	-0.19481	0.041486	0.28426	1.7354	0.79526
1168.745	10.03	domics Pos	0.27679	0.99987	1.0491	0.069182	0.19884	0.99994	0.92871	-0.1067	0.80679	0.99996	0.98685	-0.0191	0.23981	0.70016	0.94015	-0.08904	0.001031	0.0056	0.74161	-0.43126	0.12866	0.39134	0.86906	-0.20247	0.41054	0.78028	1.0629	0.088058	0.18002	0.92305	0.92136	-0.11816	0.030286	0.11973	1.19	0.25102
393.2135	0.6	domics Pos	0.27701	0.99987	1.2611	0.33464	0.07971	0.99994	0.81447	-0.29606	0.64776	0.99996	0.94825	-0.07666	0.19571	0.64166	0.78654	-0.3464	0.000158	0.001419	0.39696	-1.3329	0.11431	0.36444	0.71487	-0.48425	0.55976	0.85477	0.92519	-0.11218	0.73368	0.97988	0.97395	-0.03808	0.035426	0.12781	1.6124	0.68923
123.118	6.04	bolomics Po	0.27727	0.99951	1.0625	0.087511	0.22359	0.99998	0.9033	-0.14672	0.096417	0.99941	0.88948	-0.16897	0.024178	0.22735	0.68828	-0.53894	2.17E-07	0.000231	0.27355	-1.8701	0.00047	0.039708	0.54642	-0.87193	0.006971	0.22149	0.67698	-0.56281	0.020285	0.51556	0.744	-0.42663	0.14696	0.39976	0.89644	-0.15772
372.31	4.62	bolomics Po	0.27746	0.99951	0.76677	-0.38313	0.087684	0.99998	0.64793	-0.62609	0.10401	0.99941	0.64525	-0.63207	0.026287	0.23658	0.56732	-0.81775	0.016113	0.0703	0.47756	-1.0662	0.006481	0.067158	0.38121	-1.3913	0.013467	0.27797	0.4093	-1.2888	0.051965	0.68405	0.56475	-0.82432	0.007998	0.2688	0.43463	-1.2021
134.0304	0.57	domics Pos	0.27783	0.99987	1.1364	0.18448	0.25397	0.99994	1.2083	0.27303	0.1705	0.99996	1.2127	0.27826	0.11648	0.53727	1.2096	0.27448	0.046382	0.098472	1.3155	0.39558	0.002555	0.061655	1.4789	0.56454	0.001225	0.23318	1.7137	0.77715	0.19068	0.92582	1.1566	0.20994	0.023808	0.11051	1.3497	0.43261
336.0078	0.52	domics Pos	0.27809	0.99987	0.91865	-0.12241	0.53882	0.99994	0.94504	-0.08155	0.37605	0.99996	1.1129	0.15428	0.049116	0.37885	1.2867	0.36369	0.96495	0.96978	1.0038	0.005417	0.044096	0.19922	1.2057	0.26993	0.001672	0.23318	1.5075	0.59218	0.95359	0.98838	1.0028	0.004058	0.29757	0.40771	1.1516	0.20362
1164.717	10.06	domics Pos	0.27816	0.99987	0.92448	-0.11328	0.40393	0.99994	0.94764	-0.07758	0.41676	0.99996	0.91208	-0.13277	0.059142	0.41916	0.84732	-0.23902	0.002216	0.009767	0.73227	-0.44954	0.28757	0.57347	0.87567	-0.19154	0.96041	0.99032	0.99809	-0.00275	0.67407	0.97988	0.97351	-0.03873	0.13228	0.23736	1.0972	0.13383
1096.202	9.04	bolomics Po	0.2784	0.99951	1.4833	0.56881	0.30556	0.99998	0.84257	-0.24713	0.95811	0.99941	1.02	0.028616	0.040366	0.27667	0.75591	-0.40371	0.069566	0.21385	0.66518	-0.58819	0.38171	0.69296	0.89105	-0.16642	0.51941	0.99391	1.2496	0.32145	0.52182	0.99956	1.1516	0.2037	0.12036	0.36992	1.5871	0.66642
307.2638	6.69	bolomics Po	0.27925	0.99951	0.77788	-0.36238	0.38346	0.99998	0.82026	-0.28585	0.043406	0.99941	0.5947	-0.74976	0.033837	0.25378	0.50576	-0.98347	0.003997	0.024996	0.32509	-1.6211	0.003607	0.051446	0.25911	-1.9484	0.006223	0.21836	0.31815	-1.6522	0.004964	0.47452	0.33607	-1.5732	0.008245	0.2688	0.37408	-1.4186
306.9182	0.29	olomics Ne	0.2793	0.99918	0.91145	-0.13376	0.02844	0.41187	0.85242	-0.23036	0.64201	0.99995	0.9695	-0.04469	0.86122	0.99985	1.0134	0.019259	0.85124	0.99927	0.98557	-0.02097	0.41882	0.81191	1.0441	0.062292	0.91271	0.97375	1.0164	0.023485	0.85109	0.99512	0.98588	-0.02051	0.60593	0.99941	0.96145	-0.05672
176.9538	10.24	domics Pos	0.27959	0.99987	0.88727	-0.17255	0.12455	0.99994	0.84052	-0.25064	0.44514	0.99996	0.91537	-0.12757	0.083042	0.4849	0.82724	-0.27362	0.009386	0.029383	0.69613	-0.52257	0.08738	0.31051	0.78081	-0.35695	0.4914	0.82138	0.92086	-0.11895	0.46969	0.96776	0.91638	-0.12598	0.37653	0.4857	1.1127	0.15411
1027.684	9.07	bolomics Po	0.27967	0.99951	1.2351	0.30463	0.29364	0.99998	0.84663	-0.24019	0.90163	0.99941	0.98533	-0.02132	0.1048	0.43494	0.73583	-0.44255	0.006189	0.033964	0.59822	-0.74124	0.12325	0.3457	0.74879	-0.41736	0.73741	0.99391	0.9622	-0.0556	0.70276	0.99956	0.96475	-0.05177	0.70644	0.86688	1.1495	0.20097
312.3268	8.31	bolomics Po	0.27971	0.99951	0.88333	-0.17898	0.2683	0.99998	0.86174	-0.21468	0.78501	0.99941	0.97215	-0.04075	0.92873	0.99905	1.0199	0.028482	0.059341	0.19055	0.77534	-0.36709	0.021854	0.10951	0.75487	-0.40569	0.26887	0.88791	0.84955	-0.23522	0.1818	0.97561	0.85958	-0.21829	0.032427	0.2688	0.77982	-0.35879
257.2484	6.83	bolomics Po	0.27992	0.99951	0.86352	-0.2117	0.55617	0.99998	0.92285	-0.11583	0.082661	0.99941	0.67628	-0.56431	0.030914	0.24688	0.52006	-0.94325	0.00346	0.022567	0.52322	-0.9345	0.003609	0.051446	0.35845	-1.4801	0.022343	0.31703	0.50217	-0.99376	0.029854	0.58535	0.49019	-1.0286	0.026268	0.2688	0.48858	-1.0333
883.4698	0.54	domics Pos	0.28013	0.99987	1.462	0.54793	0.28771	0.99994	0.86265	-0.21315	0.84389	0.99996	0.97949	-0.0299	0.019761	0.25893	0.55263	-0.85561	0.000197	0.001677	0.4221	-1.2443	0.008526	0.084881	0.58752	-0.76729	0.41563	0.78082	0.87277	-0.19633	0.50862	0.96776	0.9063	-0.14194	0.014624	0.098791	1.9598	0.97073
812.8376	10.02	domics Pos	0.28057	0.99987	1.075	0.10431	0.52049	0.99994	1.0365	0.051661	0.77459	0.99996	0.97555	-0.03571	0.87889	0.9874	0.98564	-0.02086	0.004309	0.01621	0.7274	-0.45917	0.24084	0.52869	0.90147	-0.14965	0.4129	0.78028	1.1061	0.14544	0.64433	0.97988	0.96191	-0.05603	0.012402	0.098791	1.2708	0.34576
369.3523	4.73	domics Pos	0.28065	0.99987	1.0467	0.065915	0.16676	0.99994	0.94323	-0.08432	0.99157	0.99996	0.99878	-0.00176	0.54457	0.90473	0.96117	-0.05714	0.00038	0.002662	0.77455	-0.36857	0.74573	0.86962	0.98319	-0.02446	0.76834	0.94339	1.0176	0.02523	0.60054	0.97073	1.0446	0.062976	0.045015	0.14081	1.2332	0.3024
851.0799	9.23	bolomics Po	0.28066	0.99951	1.3665	0.45044	0.35017	0.99998	0.83031	-0.26828	0.51446	0.99941	1.1051	0.14423	0.18139	0.60916	0.75467	-0.40609	0.022813	0.093328	0.59487	-0.74936	0.19264	0.46535	0.76516	-0.38616	0.99461	0.99906	1.0255	0.036279	0.89833	0.99956	1.0038	0.005474	0.17092	0.43307	1.6426	0.71598
718.8361	10.09	domics Pos	0.281	0.99987	1.1183	0.16137	0.041616	0.99994	0.85453	-0.2268	0.4689	0.99996	0.95012	-0.07382	0.60301	0.93327	0.95541	-0.06582	0.012182	0.035566	0.77423	-0.36917	0.15471	0.4337	0.87246	-0.19685	0.86356	0.97419	1.0111	0.015895	0.83839	0.97988	1.0142	0.020315	0.30565	0.4152	1.1068	0.14645
1007.151	4.3	domics Pos	0.28121	0.99987	1.4173	0.50317	0.95918	0.99994	0.97284	-0.03973	0.22616	0.99996	1.209	0.27379	0.21471	0.66344	0.72909	-0.45583	0.008391	0.026864	0.51025	-0.97073	0.060986	0.24659	0.70711	-0.49999	0.83861	0.96659	0.97771	-0.03253	0.86768	0.98023	1.1105	0.15121	0.091587	0.19188	1.8499	0.88748
1028.693	9.39	bolomics Po	0.2814	0.99951	1.3108	0.39041	0.29879	0.99998	0.82461	-0.27821	0.70462	0.99941	0.92528	-0.11203	0.11222	0.45435	0.72526	-0.46343	0.021341	0.088524	0.62001	-0.68965	0.025265	0.11873	0.6071	-0.72	0.21891	0.81684	0.74815	-0.4186	0.66088	0.99956	0.92317	-0.11533	0.12064	0.37038	1.4807	0.5663
1181.849	9.61	bolomics Po	0.28154	0.99951	1.4681	0.55395	0.76202	0.99998	0.94698	-0.0786	0.20131	0.99941	1.1754	0.23321	0.089519	0.40055	0.71332	-0.48737	0.017927	0.077165	0.57861	-0.78934	0.055376	0.19951	0.69138	-0.53245	0.99482	0.99906	1.0227	0.032389	0.64331	0.99956	1.2281	0.29647	0.11928	0.36992	1.7648	0.81954
965.133	9.45	bolomics Po	0.2821	0.99951	1.2837	0.36025	0.53628	0.99998	0.92145	-0.11802	0.62577	0.99941	1.0455	0.064151	0.028844	0.24036	0.62194	-0.68514	0.00203	0.015634	0.59931	-0.73862	0.01903	0.10177	0.67703	-0.56272	0.52465	0.99391	0.90583	-0.14269	0.86351	0.99956	1.0275	0.039186	0.042387	0.28452	1.6845	0.7523
398.9766	12	olomics Ne	0.28247	0.99918	1.1055	0.14469	0.39579	0.7961	1.0755	0.10505	0.11887	0.99995	1.1051	0.14419	0.98838	0.99985	0.9975	-0.00362	0.29799	0.99927	1.1057	0.14494	0.51158	0.86267	1.0645	0.090233	0.074366	0.42579	1.2263	0.29432	0.048257	0.99512	1.2522	0.32449	0.12184	0.99941	1.2706	0.34555
732.9217	3.21	bolomics Po	0.28254	0.99951	1.2564	0.32931	0.36389	0.99998	0.86132	-0.21538	0.60514	0.99941	0.92445	-0.11333	0.010763	0.19754	0.66408	-0.59058	0.010026	0.048513	0.59442	-0.75046	0.044115	0.17135	0.70275	-0.50891	0.62079	0.99391	0.92772	-0.10825	0.59761	0.99956	0.92513	-0.11227	0.11975	0.36992	1.4985	0.58354
919.091	8.93	bolomics Po	0.28288	0.99951	1.3699	0.45409	0.60599	0.99998	0.93891	-0.09094	0.40594	0.99941	1.1067	0.14627	0.02101	0.22258	0.76205	-0.39204	0.12531	0.34535	0.71953	-0.47487	0.25154	0.54095	0.87399	-0.1943	0.43621	0.99391	1.2187	0.28533	0.26992	0.99956	1.1509	0.20277	0.097175	0.3499	1.8889	0.91753
753.5566	10.13	olomics Ne	0.28301	0.99918	1.0811	0.11248	0.81777	0.9788	0.97605	-0.03497	0.15233	0.99995	1.1324	0.17944	0.33195	0.99985	1.1303	0.17665	0.55087	0.99927	1.0782	0.10857	0.024259	0.43917	1.2548	0.32751	0.017526	0.29108	1.2826	0.35911	0.37926	0.99512	1.0987	0.13584	0.74021	0.99941	0.96679	-0.04873
1166.746	10.04	domics Pos	0.28335	0.99987	0.90065	-0.15096	0.33411	0.99994	0.9396	-0.08988	0.72065	0.99996	0.97461	-0.03711	0.41847	0.85533	0.92865	-0.10679	0.006519	0.022273	0.7368	-0.44066	0.2313	0.51854	0.86386	-0.21113	0.81622	0.95968	0.98492	-0.02193	0.89494	0.98023	0.99198	-0.01162	0.004248	0.085451	1.2783	0.35423
851.5821	9.23	bolomics Po	0.28339	0.99951	1.284	0.36066	0.73694	0.99998	0.9476	-0.07765	0.48354	0.99941	1.0829	0.11487	0.29572	0.78478	0.82863	-0.2712	0.044915	0.15514	0.71331	-0.48739	0.086411	0.26979	0.74486	-0.42495	0.90674	0.9951	0.98924	-0.01561	0.49187	0.99956	0.88798	-0.1714	0.14358	0.39507	1.4878	0.57318
943.1209	9.46	bolomics Po	0.28375	0.99951	1.2969	0.37507	0.22391	0.99998	0.84469	-0.24351	0.94047	0.99941	1.0055	0.0079	0.045752	0.2866	0.67876	-0.55903	0.000683	0.006965	0.53001	-0.91592	0.034834	0.14572	0.70581	-0.50265	0.73445	0.99391	0.97171	-0.0414	0.52729	0.99956	0.91397	-0.12978	0.066076	0.30735	1.6175	0.69376
980.1732	8.88	bolomics Po	0.28393	0.99951	1.3519	0.43501	0.55897	0.99998	0.92413	-0.11384	0.5985	0.99941	1.0777	0.10794	0.06302	0.32975	0.75221	-0.41079	0.006839	0.036638	0.61702	-0.69662	0.02184	0.10951	0.74239	-0.42976	0.44581	0.99391	1.1526	0.20495	0.81354	0.99956	1.0822	0.11393	0.077445	0.32312	1.762	0.81717
326.3425	7.92	bolomics Po	0.28396	0.99951	0.88102	-0.18275	0.30945	0.99998	0.86227	-0.21379	0.092979	0.99941	0.80417	-0.31442	0.77341	0.99905	0.97706	-0.03349	0.59353	0.96422	0.9432	-0.08437	0.008417	0.075866	0.7107	-0.49269	0.10766	0.62943	0.82132	-0.28399	0.053254	0.69529	0.76675	-0.38317	0.001499	0.2688	0.64282	-0.6375
657.3671	2.78	bolomics Po	0.2843	0.99951	1.3971	0.48239	0.061986	0.99998	0.78243	-0.35397	0.62148	0.99941	1.0442	0.062343	0.025087	0.23087	0.61974	-0.69027	0.002778	0.019316	0.54381	-0.87882	0.000597	0.039708	0.56737	-0.81763	0.54528	0.99391	0.93381	-0.0988	0.92027	0.99956	1.0876	0.12113	0.023763	0.2688	2.5343	1.3416
500.9795	5.05	olomics Ne	0.28439	0.99918	1.0394	0.055741	0.45708	0.82821	0.97249	-0.04025	0.38357	0.99995	1.0395	0.055842	0.7302	0.99985	1.0206	0.029415	0.36621	0.99927	1.0471	0.066449	0.079047	0.53781	1.11	0.15056	0.012695	0.2716	1.108	0.14791	0.14436	0.99512	1.0524	0.073723	0.3322	0.99941	1.0478	0.067297
889.5385	5.57	domics Pos	0.28466	0.99987	0.9112	-0.13415	0.84653	0.99994	1.0077	0.011028	0.49333	0.99996	0.92527	-0.11205	0.42365	0.86016	0.94245	-0.08551	0.007876	0.025666	0.73741	-0.43946	0.19216	0.47857	0.8733	-0.19545	0.2778	0.7084	0.88631	-0.17411	0.9641	0.98873	1.0043	0.006247	0.80604	0.85611	1.0421	0.059467
149.0607	4.37	bolomics Po	0.28486	0.99951	0.89428	-0.1612	0.13271	0.99998	0.83936	-0.25265	0.43093	0.99941	0.89931	-0.1531	0.60979	0.99905	0.94401	-0.08313	0.45581	0.85802	0.92557	-0.11158	0.020949	0.10792	0.71284	-0.48836	0.21605	0.81115	0.8177	-0.29036	0.057684	0.70429	0.77296	-0.37153	0.043167	0.28452	0.76728	-0.38218
575.8419	3.02	bolomics Po	0.28488	0.99951	1.1861	0.24624	0.17627	0.99998	0.91599	-0.12659	0.4261	0.99941	0.93148	-0.10241	0.003627	0.17363	0.59048	-0.76004	0.008209	0.041648	0.55821	-0.84111	0.001998	0.0456	0.56031	-0.83571	0.49766	0.99391	0.90709	-0.14069	0.66057	0.99956	0.97478	-0.03686	0.020226	0.2688	1.6404	0.71408
86.0969	0.59	domics Pos	0.28489	0.99987	0.78625	-0.34693	0.17192	0.99994	0.73245	-0.44919	0.9417	0.99996	0.96933	-0.04494	0.12688	0.55024	1.4504	0.5365	0.90317	0.91741	0.96915	-0.04522	0.797	0.89629	1.0255	0.036335	0.03118	0.35811	1.5674	0.64841	0.28528	0.94508	0.77558	-0.36665	0.24797	0.35679	0.77993	-0.35858
502.2869	10.15	olomics Ne	0.28502	0.99918	1.1315	0.17823	0.74165	0.95662	1.0375	0.053052	0.40548	0.99995	1.104	0.1427	0.94621	0.99985	0.99302	-0.0101	0.082768	0.99927	1.2701	0.34498	0.007658	0.36417	1.4494	0.53547	0.003443	0.22316	1.3999	0.48531	0.12853	0.99512	1.1741	0.23161	0.18503	0.99941	1.1074	0.14716
614.8205	0.54	domics Pos	0.28503	0.99987	1.066	0.092161	0.63949	0.99994	0.97019	-0.04367	0.37076	0.99996	0.9523	-0.07052	0.019669	0.25861	0.86216	-0.21398	0.001483	0.007308	0.74442	-0.42581	0.11054	0.35568	0.83483	-0.26044	0.051265	0.41162	0.85912	-0.21906	0.24445	0.94365	0.90282	-0.14749	0.030258	0.11973	1.2101	0.27513
677.4668	9.47	bolomics Po	0.2851	0.99951	1.2694	0.3442	0.088599	0.99998	0.79964	-0.32258	0.97923	0.99941	0.99341	-0.00954	0.018034	0.20936	0.69181	-0.53156	0.004232	0.026087	0.63355	-0.65846	0.074954	0.24467	0.79545	-0.33016	0.58826	0.99391	0.93187	-0.1018	0.97949	0.99956	1.0224	0.031911	0.11302	0.36506	1.5492	0.63152
604.7936	0.56	domics Pos	0.28511	0.99987	1.0897	0.1239	0.82677	0.99994	1.018	0.025774	0.39471	0.99996	0.93077	-0.1035	0.062749	0.43188	0.85552	-0.22513	0.002335	0.010151	0.70547	-0.50335	0.077643	0.28838	0.86072	-0.21638	0.038823	0.37121	0.82349	-0.28018	0.79449	0.97988	0.9783	-0.03166	0.18821	0.29581	1.1565	0.20973
666.3744	3.14	bolomics Po	0.28513	0.99951	1.2532	0.32564	0.67445	0.99998	0.95839	-0.06132	0.3579	0.99941	1.0833	0.11542	0.0277	0.24036	0.62771	-0.67182	0.003441	0.022533	0.63919	-0.64568	0.000681	0.041543	0.60103	-0.7345	0.68832	0.99391	0.97574	-0.03543	0.8229	0.99956	1.0044	0.006342	0.026555	0.2688	1.8141	0.85924
959.7761	10.05	domics Pos	0.28547	0.99987	0.89917	-0.15334	0.13063	0.99994	0.86689	-0.20608	0.84879	0.99996	0.97751	-0.03282	0.39891	0.84363	0.90849	-0.13846	0.016625	0.04511	0.74664	-0.42151	0.16561	0.45081	0.84801	-0.23784	0.40634	0.77758	0.90565	-0.14298	0.92729	0.98573	1.0016	0.00228	0.4382	0.54372	1.094	0.12963
1047.22	9.06	bolomics Po	0.28613	0.99951	1.2524	0.32473	0.50351	0.99998	0.9193	-0.12139	0.6936	0.99941	1.0655	0.091571	0.047519	0.28918	0.73532	-0.44356	0.054858	0.18	0.72849	-0.45703	0.019888	0.1043	0.75906	-0.39772	0.95939	0.9974	1.0752	0.10461	0.63092	0.99956	1.1528	0.2051	0.22419	0.49246	1.3922	0.47733
1177.867	9.6	bolomics Po	0.28621	0.99951	1.4945	0.57964	0.8678	0.99998	1.0195	0.027805	0.35527	0.99941	1.1187	0.16184	0.11545	0.46351	0.75261	-0.41002	0.005314	0.030601	0.64663	-0.629	0.11557	0.32973	0.77772	-0.36268	0.8489	0.9951	1.0807	0.11194	0.48505	0.99956	1.293	0.37075	0.074599	0.31928	1.7811	0.83273
550.2981	1.11	domics Pos	0.28624	0.99987	1.2183	0.28487	0.44231	0.99994	1.1422	0.19182	0.5237	0.99996	1.1204	0.16402	0.38337	0.83056	1.123	0.16732	0.54957	0.61578	0.91268	-0.13182	0.039694	0.1839	1.6146	0.69116	0.01973	0.33809	2.2303	1.1573	0.049732	0.6775	1.4995	0.58448	0.000835	0.07528	2.4432	1.2888
370.3556	4.73	domics Pos	0.28648	0.99987	1.0402	0.05689	0.13591	0.99994	0.9378	-0.09265	0.9707	0.99996	0.99827	-0.0025	0.41812	0.85533	0.94823	-0.07669	0.000176	0.001538	0.77422	-0.36919	0.58026	0.77145	0.97094	-0.04255	0.55355	0.85214	1.0316	0.044814	0.5265	0.96776	1.0503	0.070748	0.059093	0.15647	1.2399	0.31019
556.3086	2.98	bolomics Po	0.28666	0.99951	1.2085	0.27317	0.10167	0.99998	0.78094	-0.35672	0.19069	0.99941	0.87268	-0.19648	0.003391	0.17363	0.59559	-0.74762	0.001652	0.013442	0.57702	-0.79331	0.000373	0.039572	0.61793	-0.69448	0.56988	0.99391	0.93834	-0.09182	0.34129	0.99956	0.85809	-0.2208	0.066127	0.30735	1.5472	0.6297
777.3631	2.4	bolomics Po	0.2872	0.99951	0.58733	-0.76776	0.65551	0.99998	1.5223	0.60627	0.26788	0.99941	1.5946	0.67324	0.24417	0.70757	3.33	1.7355	0.008629	0.043006	5.751	2.5238	0.035288	0.14703	3.3397	1.7397	0.052073	0.45947	2.8577	1.5148	0.2825	0.99956	2.2879	1.194	0.23808	0.50476	0.50016	-0.99952
611.4161	10.05	bolomics Po	0.28727	0.99951	1.1504	0.20214	0.76743	0.99998	1.0595	0.0834	0.63199	0.99941	0.93481	-0.09725	0.95642	0.99905	0.99327	-0.00974	0.26756	0.61971	1.1506	0.20236	0.88294	0.96462	1.0081	0.011608	0.97666	0.9974	0.98512	-0.02163	0.015477	0.47796	0.63406	-0.65731	0.13237	0.385	0.81266	-0.29928
596.7633	0.56	domics Pos	0.28749	0.99987	1.1401	0.18917	0.35304	0.99994	1.0746	0.10381	0.75703	0.99996	1.0317	0.045091	0.36684	0.82143	0.91764	-0.124	0.009637	0.029948	0.74129	-0.43189	0.10369	0.34299	0.86844	-0.2035	0.18341	0.64793	0.87177	-0.19799	0.45199	0.96776	1.08	0.11107	0.03851	0.13216	1.1922	0.25367
374.2178	2.04	domics Pos	0.28752	0.99987	1.1841	0.24379	0.69499	0.99994	0.96824	-0.04657	0.050423	0.99996	0.73042	-0.4532	0.027036	0.29092	0.54804	-0.86764	7.04E-05	0.000823	0.45551	-1.1345	0.002917	0.062584	0.38309	-1.3842	0.057482	0.42807	0.58576	-0.77163	0.021249	0.52955	0.48693	-1.0382	0.010767	0.094875	0.63483	-0.65557
870.7937	10.06	domics Pos	0.28765	0.99987	0.92872	-0.10668	0.24138	0.99994	0.88814	-0.17115	0.046906	0.99996	0.84754	-0.23865	0.30657	0.76501	0.91247	-0.13216	0.002712	0.011352	0.70007	-0.51443	0.6246	0.79912	0.95051	-0.07323	0.42598	0.78909	0.92078	-0.11907	0.27729	0.94365	0.89583	-0.1587	0.508	0.60874	1.0623	0.08714
1160.264	9	bolomics Po	0.28799	0.99951	1.4666	0.55247	0.87648	0.99998	1.0173	0.024707	0.40396	0.99941	1.1306	0.17704	0.033448	0.25203	0.72643	-0.46111	0.040219	0.14235	0.64914	-0.62339	0.28053	0.57396	0.85024	-0.23405	0.38785	0.97696	1.2604	0.33392	0.49429	0.99956	1.2651	0.33926	0.010774	0.2688	1.7778	0.8301
918.6265	9.19	bolomics Po	0.28858	0.99951	1.3096	0.38915	0.40249	0.99998	0.85493	-0.22611	0.38668	0.99941	1.1056	0.14488	0.34962	0.84327	0.84948	-0.23535	0.030078	0.11554	0.62194	-0.68515	0.21697	0.49779	0.78123	-0.35618	0.90314	0.9951	1.0698	0.097387	0.83881	0.99956	0.9787	-0.03106	0.11264	0.36506	1.6483	0.72097
730.0363	9.43	bolomics Po	0.28898	0.99951	1.3296	0.41101	0.20384	0.99998	0.82817	-0.27201	0.31263	0.99941	1.1345	0.18205	0.007477	0.1948	0.58299	-0.77846	0.034286	0.12747	0.61398	-0.70375	0.00778	0.072933	0.63415	-0.65711	0.55511	0.99391	0.92478	-0.11282	0.93036	0.99956	1.0965	0.13294	0.13612	0.39051	1.7421	0.80083
972.5879	5.78	domics Pos	0.28899	0.99987	1.0868	0.1201	0.54	0.99994	0.93473	-0.09737	0.32024	0.99996	0.89244	-0.16418	0.40784	0.84932	0.89049	-0.16732	0.006064	0.021038	0.67102	-0.57557	0.019424	0.11985	0.68461	-0.54666	0.094025	0.52505	0.76197	-0.39219	0.025996	0.56113	0.75427	-0.40684	0.098186	0.19935	1.2009	0.26409
135.9274	10.24	domics Pos	0.28906	0.99987	0.88174	-0.18158	0.033903	0.99994	0.7557	-0.40411	0.24821	0.99996	0.8676	-0.2049	0.14469	0.57187	0.86807	-0.20411	0.000987	0.005488	0.58698	-0.76862	0.39987	0.64773	0.88366	-0.17844	0.31862	0.72948	0.87215	-0.19735	0.33644	0.96524	0.87902	-0.18603	0.71711	0.78936	1.0359	0.050899
709.5289	9.5	bolomics Po	0.28959	0.99951	1.362	0.44576	0.041604	0.99998	0.80809	-0.30742	0.053635	0.99941	1.1313	0.17796	0.000156	0.077904	0.68294	-0.55017	0.003139	0.021166	0.67342	-0.57042	0.01635	0.094477	0.62527	-0.67744	0.8382	0.9951	1.0085	0.012277	0.77252	0.99956	1.0273	0.038789	0.028111	0.2688	1.764	0.81888
667.9853	9.56	bolomics Po	0.28981	0.99951	1.3439	0.42642	0.48159	0.99998	0.89603	-0.15838	0.34018	0.99941	1.131	0.17754	0.066261	0.33665	0.70846	-0.49724	0.019474	0.082193	0.59806	-0.74164	0.050514	0.18898	0.70073	-0.51307	0.93232	0.9955	1.0577	0.080891	0.97361	0.99956	1.0425	0.060058	0.072113	0.3167	1.7557	0.81205
1091.512	12.05	olomics Ne	0.28999	0.99918	1.1198	0.16324	0.069574	0.50118	0.83493	-0.26028	0.12682	0.99995	1.2464	0.31777	0.14759	0.99985	1.131	0.1776	0.15282	0.99927	1.1252	0.17014	0.064388	0.51001	1.2166	0.28291	0.1734	0.54872	1.199	0.26187	0.010001	0.99512	1.2616	0.33523	0.44701	0.99941	1.0806	0.11181
150.1372	6.97	bolomics Po	0.29041	0.99951	1.1529	0.20529	0.7799	0.99998	0.95925	-0.06003	0.21814	0.99941	0.82093	-0.28466	0.037766	0.26495	0.6245	-0.67923	0.000435	0.005149	0.46412	-1.1074	0.003399	0.049867	0.46402	-1.1077	0.022448	0.31709	0.61691	-0.69688	0.023525	0.51812	0.6031	-0.72953	0.068377	0.31036	0.68034	-0.55567
310.9067	10.14	domics Pos	0.29057	0.99987	1.1232	0.16764	0.6364	0.99994	1.0551	0.077448	0.8251	0.99996	1.0146	0.020926	0.25059	0.70866	0.93839	-0.09174	0.015027	0.041854	0.83019	-0.26849	0.47085	0.69396	0.92727	-0.10895	0.80978	0.95737	0.98425	-0.0229	0.31821	0.9524	0.95193	-0.07107	0.010283	0.094875	1.22	0.28684
695.4281	3.16	bolomics Po	0.29089	0.99951	1.4101	0.49575	0.27424	0.99998	0.84382	-0.24499	0.98485	0.99941	0.99922	-0.00112	0.000395	0.11324	0.54687	-0.87074	0.000948	0.008967	0.49093	-1.0264	0.009119	0.076393	0.60276	-0.73034	0.58151	0.99391	0.93766	-0.09286	0.86154	0.99956	1.098	0.13493	0.11362	0.36506	1.5072	0.59186
651.2843	1.04	domics Pos	0.29168	0.99987	0.82115	-0.28429	0.68537	0.99994	0.97454	-0.0372	0.44609	0.99996	0.947	-0.07856	0.27845	0.74456	0.9023	-0.14831	0.01951	0.051233	0.67189	-0.5737	0.18713	0.47286	1.0719	0.10015	0.18214	0.64793	1.3145	0.39448	0.78758	0.97988	1.0845	0.11701	0.3368	0.44716	1.1395	0.18839
663.3378	3.11	bolomics Po	0.29207	0.99951	1.4057	0.49132	0.22564	0.99998	0.87384	-0.19456	0.68323	0.99941	1.0478	0.067413	0.021551	0.22258	0.65364	-0.61344	0.000999	0.009286	0.55307	-0.85446	0.093391	0.28463	0.75316	-0.40897	0.89597	0.9951	1.1028	0.14114	0.72165	0.99956	0.98723	-0.01854	0.028772	0.2688	1.7501	0.80742
1090.827	9.4	bolomics Po	0.29211	0.99951	1.4035	0.48904	0.46649	0.99998	0.84326	-0.24595	0.56779	0.99941	1.0651	0.091007	0.040757	0.27667	0.62136	-0.68649	0.078714	0.23773	0.66785	-0.58241	0.060762	0.21244	0.65621	-0.60778	0.88716	0.9951	1.0102	0.014691	0.65674	0.99956	1.2081	0.27278	0.18725	0.45234	1.7907	0.84052
712.4858	5.07	domics Pos	0.29239	0.99987	1.2636	0.33752	0.22307	0.99994	0.82204	-0.28272	0.60258	0.99996	1.0525	0.073847	0.022825	0.26923	0.66927	-0.57935	0.004264	0.016081	0.49858	-1.0041	0.01955	0.12004	0.68195	-0.55227	0.99719	0.99846	1.0024	0.003495	0.88305	0.98023	1.0064	0.009269	0.056255	0.15336	1.7456	0.80375
133.1428	11.78	bolomics Po	0.29254	0.99951	1.074	0.10297	0.18241	0.99998	1.1187	0.16179	0.7407	0.99941	1.0191	0.027308	0.50962	0.98198	1.0473	0.066704	0.22875	0.54753	1.1068	0.14638	0.15936	0.41184	1.1327	0.1798	0.048878	0.44698	1.1179	0.1608	0.91584	0.99956	0.99604	-0.00572	0.012142	0.2688	1.1903	0.25132
1138.505	6.12	domics Pos	0.2931	0.99987	1.1071	0.14679	0.6215	0.99994	0.95433	-0.06743	0.20238	0.99996	1.1096	0.15009	0.062695	0.43188	0.87996	-0.18448	0.00954	0.029693	0.72899	-0.45604	0.021235	0.12671	0.73443	-0.4453	0.07657	0.48741	0.86616	-0.2073	0.22346	0.94047	0.84109	-0.24966	0.64741	0.73535	1.0871	0.12048
1049.196	9.06	bolomics Po	0.29313	0.99951	1.2966	0.37469	0.48779	0.99998	0.9063	-0.14194	0.53248	0.99941	1.0767	0.10658	0.031872	0.24911	0.71086	-0.49235	0.032522	0.12269	0.64897	-0.62378	0.12867	0.35759	0.80103	-0.32007	0.69753	0.99391	1.0956	0.13168	0.84664	0.99956	1.0824	0.11421	0.20463	0.46913	1.3618	0.44551
982.6513	8.88	bolomics Po	0.29364	0.99951	1.3177	0.39803	0.39545	0.99998	0.89049	-0.16733	0.59206	0.99941	1.0629	0.088037	0.013683	0.20085	0.68512	-0.54557	0.026434	0.10408	0.64899	-0.62372	0.05155	0.19149	0.76538	-0.38575	0.61382	0.99391	1.1456	0.19607	0.83929	0.99956	1.075	0.10432	0.11445	0.36506	1.6883	0.75553
348.9775	12.01	olomics Ne	0.29382	0.99918	1.1182	0.16115	0.008589	0.34605	0.77387	-0.36984	0.88791	0.99995	0.99064	-0.01357	0.76396	0.99985	1.0582	0.081599	0.42893	0.99927	1.0755	0.10494	0.32609	0.73944	1.1105	0.15115	0.35813	0.71602	1.0808	0.11214	0.76274	0.99512	1.044	0.062181	0.81406	0.99941	1.0233	0.033186
940.6005	8.91	bolomics Po	0.29482	0.99951	1.3398	0.42197	0.63342	0.99998	0.93395	-0.09858	0.24354	0.99941	1.1707	0.22732	0.045857	0.2866	0.75753	-0.40063	0.10738	0.3039	0.69689	-0.521	0.42278	0.73013	0.88976	-0.16851	0.67503	0.99391	1.1986	0.26131	0.47337	0.99956	1.1486	0.19988	0.12445	0.37442	1.6971	0.76309
741.3905	2.8	bolomics Po	0.29493	0.99951	1.353	0.43614	0.055317	0.99998	0.79441	-0.33204	0.44029	0.99941	0.91674	-0.12541	0.011669	0.19869	0.53813	-0.89397	0.000146	0.002189	0.4358	-1.1983	0.008849	0.075866	0.50763	-0.97815	0.53509	0.99391	0.91685	-0.12524	0.48619	0.99956	0.91244	-0.13219	0.043073	0.28452	2.1002	1.0706
1028.689	9.07	bolomics Po	0.29505	0.99951	1.2091	0.27393	0.35483	0.99998	0.8609	-0.21608	0.98108	0.99941	0.99653	-0.00502	0.093598	0.41121	0.70182	-0.51082	0.040374	0.14274	0.60593	-0.72278	0.08424	0.26562	0.68678	-0.54208	0.75807	0.99391	0.9507	-0.07294	0.80722	0.99956	0.97693	-0.03367	0.43137	0.69935	1.2662	0.34047
596.759	9.92	domics Pos	0.29515	0.99987	1.1134	0.15501	0.048248	0.99994	0.88477	-0.17663	0.98316	0.99996	1.0089	0.012835	0.85686	0.9874	0.98922	-0.01564	0.051849	0.10716	0.83845	-0.2542	0.27989	0.56694	0.85052	-0.23359	0.049438	0.40631	1.1967	0.25912	0.50171	0.96776	0.93778	-0.09269	0.034372	0.12626	1.2377	0.3077
664.9746	12.01	olomics Ne	0.29589	0.99918	1.063	0.088196	0.27453	0.7026	1.1042	0.14302	0.39324	0.99995	1.0992	0.13647	0.51981	0.99985	1.0487	0.068591	0.008198	0.69321	1.3	0.37849	0.026148	0.45335	1.3061	0.38528	0.15564	0.53641	1.2112	0.27643	0.23458	0.99512	1.0778	0.10815	0.7856	0.99941	1.0739	0.1029
1106.762	10.02	domics Pos	0.2959	0.99987	1.0986	0.13573	0.47015	0.99994	0.94666	-0.07908	0.31554	0.99996	0.9292	-0.10595	0.028634	0.29796	0.81088	-0.30244	0.096263	0.16946	0.83269	-0.26415	0.1557	0.43524	0.88604	-0.17455	0.64941	0.89057	1.0619	0.086639	0.51098	0.96776	0.95324	-0.06908	0.048608	0.14514	1.1909	0.252
105.0729	6	bolomics Po	0.29591	0.99951	0.87142	-0.19856	0.45013	0.99998	0.89383	-0.16192	0.34555	0.99941	0.87798	-0.18775	0.078424	0.36707	0.69052	-0.53425	0.000278	0.003655	0.35468	-1.4954	0.015934	0.093702	0.66448	-0.58971	0.18624	0.76329	0.81027	-0.30352	0.14496	0.93461	0.78901	-0.34188	0.88187	0.95289	1.0171	0.024474
983.659	9.11	bolomics Po	0.29598	0.99951	1.24	0.31034	0.41206	0.99998	0.85097	-0.23282	0.45784	0.99941	1.1093	0.14964	0.3014	0.79062	0.79663	-0.32803	0.03435	0.12747	0.62517	-0.67769	0.19646	0.47041	0.71921	-0.47551	0.67401	0.99391	0.91705	-0.12493	0.62958	0.99956	0.9135	-0.13053	0.49878	0.75091	1.2932	0.37089
402.8262	9.92	domics Pos	0.29679	0.99987	1.0754	0.10481	0.77186	0.99994	0.97382	-0.03827	0.48232	0.99996	1.0467	0.065872	0.567	0.91222	0.95014	-0.07379	0.019845	0.051971	0.78585	-0.34768	0.86781	0.93184	0.98606	-0.02026	0.73953	0.93154	0.96845	-0.04625	0.51566	0.96776	1.0531	0.074637	0.072657	0.17431	1.2249	0.29261
781.3895	0.56	domics Pos	0.29688	0.99987	1.607	0.68434	0.36989	0.99994	0.83923	-0.25286	0.80961	0.99996	1.0187	0.02675	0.04487	0.36636	0.62469	-0.67879	0.003877	0.014961	0.49485	-1.0149	0.069418	0.26872	0.69215	-0.53085	0.76389	0.94202	1.0285	0.040479	0.95547	0.98838	1.0399	0.056422	0.00918	0.092545	1.7237	0.78549
655.8606	3.11	bolomics Po	0.29719	0.99951	1.2578	0.33091	0.12872	0.99998	0.8492	-0.23583	0.92066	0.99941	1.011	0.015769	0.018654	0.21213	0.65996	-0.59956	0.001213	0.010796	0.55822	-0.84109	0.008825	0.075866	0.63447	-0.65638	0.63606	0.99391	0.9471	-0.07841	0.3568	0.99956	0.85798	-0.22098	0.02741	0.2688	1.7795	0.83144
1051.675	9.06	bolomics Po	0.29722	0.99951	1.3977	0.48303	0.71415	0.99998	0.96508	-0.05128	0.5972	0.99941	1.0777	0.10789	0.17073	0.58585	0.80852	-0.30665	0.037014	0.13432	0.64849	-0.62485	0.52851	0.81736	0.92445	-0.11333	0.67583	0.99391	1.1696	0.22597	0.89202	0.99956	1.0621	0.086905	0.22528	0.49266	1.3674	0.45148
1030.174	4.52	domics Pos	0.29724	0.99987	1.3634	0.44724	0.19309	0.99994	0.81526	-0.29468	0.98458	0.99996	0.99082	-0.0133	0.036309	0.33474	0.59159	-0.75733	0.002736	0.011425	0.41089	-1.2832	0.024934	0.13941	0.57692	-0.79357	0.81965	0.96018	0.97869	-0.03107	0.69474	0.97988	0.98931	-0.0155	0.052616	0.14916	1.9058	0.93043
1051.749	9.43	bolomics Po	0.29761	0.99951	1.4334	0.51947	0.99327	0.99998	0.99169	-0.01204	0.39626	0.99941	1.1192	0.16244	0.10213	0.42724	0.70049	-0.51356	0.042388	0.1477	0.65307	-0.61468	0.1288	0.35759	0.73177	-0.45055	0.98428	0.99744	1.0431	0.060848	0.75235	0.99956	1.2187	0.28533	0.10693	0.36162	1.9449	0.9597
940.1392	9.46	bolomics Po	0.29797	0.99951	1.2527	0.32499	0.39204	0.99998	0.90969	-0.13655	0.58918	0.99941	1.0525	0.07378	0.066493	0.33728	0.70345	-0.50748	0.006089	0.03365	0.62603	-0.67569	0.007181	0.070405	0.54781	-0.86824	0.67956	0.99391	0.9609	-0.05755	0.83158	0.99956	1.0148	0.021235	0.073319	0.31731	1.652	0.7242

1068.73	9.05	olomics Po	0.29829	0.99951	1.2944	0.37224	0.61988	0.99998	0.95125	-0.0721	0.56012	0.99941	1.1154	0.15752	0.12294	0.48347	0.78197	-0.35482	0.01931	0.081974	0.63577	-0.65342	0.063087	0.21742	0.79465	-0.3316	0.3459	0.95369	1.2664	0.34068	0.41605	0.99956	1.1679	0.22397	0.073357	0.31731	1.5282	0.61185
444.3617	2.06	omics Pos	0.29832	0.99987	1.1937	0.25543	0.5845	0.99994	0.89104	-0.16643	0.53465	0.99996	1.0804	0.1116	0.8558	0.9874	0.96297	-0.05444	0.000395	0.002744	0.31988	-1.6444	0.32307	0.59944	0.81349	-0.2978	0.65483	0.89228	0.91308	-0.13119	0.24676	0.94365	1.1852	0.24511	0.052136	0.14863	1.3876	0.4726
313.0468	0.57	omics Pos	0.29863	0.99987	0.7886	-0.34263	0.59255	0.99994	0.92476	-0.11286	0.50422	0.99996	1.1031	0.14162	0.13576	0.55962	1.3128	0.3926	0.007516	0.024685	0.55074	-0.86057	0.41377	0.65507	1.1495	0.20098	0.1225	0.58026	1.3068	0.38604	0.81536	0.97988	1.1251	0.17007	0.46229	0.56587	1.1901	0.25108
1002.533	6.11	omics Pos	0.29904	0.99987	1.1335	0.18078	0.65396	0.99994	1.0485	0.068354	0.60289	0.99996	1.0536	0.075307	0.70703	0.96282	1.0443	0.062528	0.074902	0.14153	0.77796	-0.36223	0.11707	0.37012	0.8139	-0.29707	0.039453	0.37421	0.76639	-0.38386	0.24369	0.94365	0.86097	-0.21596	0.74775	0.81114	1.0531	0.074668
254.986	3.79	olomics Ne	0.29921	0.99918	1.0248	0.03539	0.027191	0.40327	0.87669	-0.18987	0.74387	0.99995	1.0087	0.01244	0.82132	0.99985	0.99111	-0.01288	0.91813	0.99927	0.99814	-0.00269	0.017619	0.40708	1.0771	0.10715	0.36398	0.71602	1.0327	0.046451	0.87756	0.99512	1.012	0.017268	0.63336	0.99941	0.98826	-0.01704
1117.212	4.14	omics Pos	0.29938	0.99987	1.2489	0.32066	0.41379	0.99994	0.89696	-0.15688	0.41415	0.99996	1.1154	0.15761	0.058597	0.41841	0.69073	-0.5338	0.005282	0.019018	0.58072	-0.78408	0.017689	0.11393	0.65563	-0.60905	0.55953	0.85477	0.91663	-0.12559	0.32084	0.95254	1.2021	0.26555	0.076835	0.17826	1.9588	0.96995
176.1528	1.67	omics Pos	0.2994	0.99987	1.1971	0.25951	0.093602	0.99994	0.68972	-0.53592	0.72814	0.99996	0.92542	-0.11181	0.16888	0.6035	0.72883	-0.45634	4.48E-05	0.00059	0.16879	-2.5667	0.038871	0.18097	0.58202	-0.78086	0.049162	0.40631	0.63544	-0.65417	0.51145	0.96776	0.87458	-0.19334	0.31545	0.42578	1.2141	0.27985
382.1961	6.37	olomics Po	0.29967	0.99951	1.1367	0.18481	0.13984	0.99998	0.84386	-0.24492	0.85099	0.99941	1.0186	0.026525	0.133	0.50789	0.79735	-0.32672	0.000677	0.006923	0.43817	-1.1904	0.009349	0.077313	0.67963	-0.55718	0.28792	0.90579	0.87249	-0.19678	0.36832	0.99956	0.91757	-0.12411	0.98542	0.99299	0.99816	-0.00265
1183.846	5.14	omics Pos	0.29972	0.99987	1.3208	0.40138	0.69975	0.99994	1.0514	0.072305	0.020282	0.99996	1.1583	0.21199	0.06238	0.43187	0.82798	-0.27233	0.096792	0.17031	0.73602	-0.44219	0.00934	0.086818	0.8453	-0.24246	0.013513	0.33809	1.1722	0.22924	0.49804	0.96776	1.1294	0.17554	0.094891	0.196	1.6134	0.69011
556.8131	2.97	olomics Po	0.29978	0.99951	1.1975	0.25998	0.3442	0.99998	0.896	-0.15842	0.26646	0.99941	0.92203	-0.11711	0.003883	0.17363	0.53709	-0.89677	0.000169	0.002464	0.61528	-0.70069	0.000419	0.039572	0.65263	-0.61566	0.64371	0.99391	0.96447	-0.0522	0.2189	0.99956	0.81345	-0.29788	0.041119	0.28426	1.6349	0.70918
486.8599	10.19	omics Pos	0.29987	0.99987	1.1318	0.17857	0.72254	0.99994	0.9694	-0.04483	0.71861	0.99996	0.97074	-0.04285	0.18599	0.62584	0.86437	-0.21028	0.001908	0.008766	0.69133	-0.53256	0.77785	0.88482	0.98391	-0.0234	0.57765	0.86151	1.05	0.070369	0.17607	0.91806	0.864	-0.2109	0.025328	0.11206	1.3159	0.39609
736.49	4.38	omics Pos	0.30013	0.99987	1.1423	0.19194	0.4239	0.99994	0.90015	-0.15176	0.93161	0.99996	1.0092	0.013186	0.045158	0.36714	0.71452	-0.48495	0.12605	0.20744	0.65593	-0.60839	0.50185	0.71797	1.3885	0.47357	0.20576	0.65133	2.0032	1.0023	0.068728	0.76386	1.7537	0.81039	0.021114	0.10765	2.3891	1.2565
331.2642	6.54	olomics Po	0.30046	0.99951	0.83706	-0.2566	0.26933	0.99998	0.81011	-0.30381	0.011186	0.99941	0.59564	-0.74749	0.00505	0.18937	0.44269	-1.1756	0.000244	0.003311	0.34791	-1.5232	0.000619	0.039708	0.22638	-2.1432	0.013035	0.27282	0.32612	-1.6165	0.00559	0.47452	0.32624	-1.616	0.004445	0.2688	0.34069	-1.5535
566.8894	10.02	omics Pos	0.30068	0.99987	1.0363	0.051458	0.28199	0.99994	0.93969	-0.08974	0.3042	0.99996	0.95357	-0.06859	0.27058	0.73104	0.94345	-0.08398	0.000401	0.002773	0.76626	-0.38409	0.35969	0.62208	0.91958	-0.12096	0.87254	0.97532	1.0127	0.018225	0.81115	0.97988	0.98863	-0.0165	0.014324	0.098791	1.1982	0.26086
474.9774	5.07	olomics Ne	0.30083	0.99918	1.032	0.045375	0.044332	0.47152	0.91393	-0.12985	0.97951	0.99995	1.0012	0.001741	0.84873	0.99985	1.0142	0.020322	0.8307	0.99927	1.0102	0.014667	0.22435	0.66171	1.0769	0.10689	0.15543	0.53641	1.0616	0.086291	0.98418	0.99512	1.0022	0.003102	0.86385	0.99941	1.0122	0.01754
658.5095	6.21	olomics Po	0.30105	0.99951	0.90227	-0.14837	0.25369	0.99998	0.86223	-0.21385	0.33695	0.99941	0.91004	-0.13599	0.72562	0.99905	0.97105	-0.04238	0.1155	0.32341	0.89887	-0.15381	0.15969	0.41199	0.89171	-0.16535	0.16417	0.73936	0.87707	-0.18923	0.030602	0.58535	0.79943	-0.32296	0.052719	0.2951	0.83083	-0.26737
631.3626	3.08	olomics Po	0.30125	0.99951	1.2504	0.32241	0.028363	0.99998	0.76452	-0.38737	0.44746	0.99941	0.93257	-0.10072	0.005298	0.1948	0.55673	-0.84495	0.002904	0.020066	0.54069	-0.88712	0.003223	0.049867	0.54059	-0.88738	0.37707	0.97118	0.86165	-0.21482	0.2265	0.99956	0.79827	-0.32506	0.11068	0.36452	1.6339	0.70829
219.2117	6.01	olomics Po	0.30138	0.99951	1.2026	0.26621	0.73324	0.99998	1.0562	0.078823	0.3558	0.99941	1.1399	0.18896	0.2717	0.74421	0.8089	-0.30597	0.000216	0.002967	0.31206	-1.6801	0.006593	0.067315	0.55882	-0.83953	0.57294	0.99391	0.9245	-0.11325	0.63262	0.99956	0.93967	-0.08978	0.082147	0.33217	1.1946	0.25647
687.5188	9.53	olomics Po	0.30156	0.99951	1.4066	0.49225	0.78913	0.99998	0.96842	-0.04629	0.18626	0.99941	1.157	0.21035	0.086026	0.39046	0.74316	-0.42826	0.022528	0.092522	0.68985	-0.53565	0.021027	0.10792	0.68783	-0.53987	0.9346	0.99628	1.1016	0.13961	0.96107	0.99956	1.0847	0.11731	0.034898	0.272	1.8296	0.87156
217.1957	1.67	omics Pos	0.3017	0.99987	1.151	0.20286	0.44907	0.99994	0.86067	-0.21647	0.99369	0.99996	0.99364	-0.0092	0.13662	0.56014	0.7368	-0.44066	3.75E-05	0.00053	0.19776	-2.3382	0.011274	0.091488	0.53862	-0.89265	0.24913	0.68563	0.7941	-0.33261	0.45593	0.96776	0.86728	-0.20542	0.10456	0.20695	1.3007	0.37927
1074.197	4.46	omics Pos	0.30287	0.99987	1.2949	0.37283	0.23226	0.99994	0.85955	-0.21834	0.90293	0.99996	0.98132	-0.02721	0.096594	0.51634	0.67677	-0.56326	0.000575	0.003667	0.48983	-1.0297	0.032759	0.16265	0.63522	-0.65466	0.65039	0.89057	0.94025	-0.08888	0.88578	0.98023	1.0152	0.021705	0.040852	0.13494	1.7592	0.8149
687.988	9.48	olomics Po	0.30291	0.99951	1.3102	0.38979	0.27742	0.99998	0.90242	-0.14813	0.22824	0.99941	1.1217	0.16565	0.003457	0.17363	0.64456	-0.63362	0.002611	0.018484	0.55454	-0.85063	0.005282	0.062631	0.63978	-0.64434	0.76874	0.99391	0.98016	-0.02891	0.82744	0.99956	1.0481	0.06772	0.11168	0.36506	1.6131	0.6898
254.8246	0.64	omics Pos	0.30299	0.99987	1.11	0.15055	0.922	0.99994	1.0058	0.008285	0.50632	0.99996	0.9651	-0.05125	0.084882	0.48909	0.89938	-0.15299	0.002772	0.011514	0.79295	-0.3347	0.063101	0.25141	0.88155	-0.18188	0.27358	0.70555	0.93908	-0.09068	0.45752	0.96776	1.0446	0.062966	0.023023	0.10989	1.1696	0.22607
380.8713	10.15	omics Pos	0.30317	0.99987	1.1004	0.13804	0.35436	0.99994	0.9288	-0.10657	0.51902	0.99996	1.0387	0.054719	0.6948	0.96092	1.0331	0.047042	0.020336	0.052999	0.78217	-0.35444	0.77643	0.88442	0.98045	-0.02849	0.047112	0.39577	1.2297	0.29828	0.9821	0.99489	1.0057	0.00815	0.23985	0.34773	1.1178	0.16063
93.0712	6.38	olomics Po	0.30324	0.99951	1.0709	0.098886	0.24812	0.99998	0.88737	-0.17239	0.1689	0.99941	0.90102	-0.15036	0.042579	0.2802	0.76887	-0.37919	6.34E-06	0.000265	0.25056	-1.9968	9.87E-05	0.034543	0.53737	-0.89601	0.033961	0.36264	0.68728	-0.54103	0.009725	0.47452	0.76159	-0.39292	0.19004	0.45476	0.83254	-0.26441
589.8254	3.03	olomics Po	0.30342	0.99951	1.2309	0.29973	0.16149	0.99998	0.83759	-0.25568	0.16479	0.99941	0.84498	-0.243	0.007912	0.19613	0.54729	-0.86964	0.003398	0.022456	0.57984	-0.78628	0.001566	0.045425	0.53249	-0.90918	0.25377	0.86345	0.81139	-0.30154	0.37158	0.99956	0.87492	-0.19277	0.01608	0.2688	1.7549	0.81136
1137.827	9.65	olomics Po	0.30374	0.99951	1.4687	0.5545	0.67939	0.99998	0.9351	-0.09681	0.48292	0.99941	1.075	0.10431	0.050352	0.29486	0.68718	-0.54124	0.011863	0.055443	0.60444	-0.72633	0.058085	0.20651	0.70756	-0.49908	0.94565	0.9974	1.029	0.041289	0.89315	0.99956	1.1352	0.18295	0.1038	0.35814	1.9347	0.95208
1050.701	9.06	olomics Po	0.30392	0.99951	1.2688	0.34352	0.36791	0.99998	0.87693	-0.18947	0.56625	0.99941	1.09	0.12438	0.048719	0.29121	0.73599	-0.44225	0.048291	0.16392	0.66464	-0.58935	0.17792	0.44199	0.81517	-0.29482	0.9092	0.9951	1.055	0.077216	0.97229	0.99956	1.0368	0.052186	0.37251	0.64903	1.2915	0.36906
872.5551	5.22	omics Pos	0.30447	0.99987	1.0971	0.13369	0.81213	0.99994	1.0394	0.055792	0.9751	0.99996	0.9976	-0.00347	0.58251	0.92348	1.0525	0.073793	0.016229	0.044327	0.77716	-0.36371	0.71862	0.85308	0.96481	-0.05168	0.78026	0.94714	1.0339	0.048137	0.82867	0.97988	0.98254	-0.02542	0.26501	0.37523	1.1584	0.21211
1162.244	9	olomics Po	0.30451	0.99951	1.322	0.40268	0.27768	0.99998	0.82694	-0.27414	0.65446	0.99941	0.94551	-0.08084	0.018001	0.20936	0.62402	-0.68035	0.039365	0.14015	0.61474	-0.70194	0.072931	0.24208	0.79528	-0.33047	0.72094	0.99391	1.151	0.20283	0.96241	0.99956	1.0051	0.007399	0.031241	0.2688	1.6788	0.74742
987.1451	9.43	olomics Po	0.30459	0.99951	1.388	0.47303	0.37598	0.99998	0.87563	-0.1916	0.56097	0.99941	0.92979	-0.10503	0.017477	0.20936	0.64356	-0.63586	0.001087	0.009835	0.51237	-0.96473	0.021186	0.10852	0.69469	-0.52557	0.71233	0.99391	0.95137	-0.07192	0.81318	0.99956	1.0225	0.032072	0.052793	0.2951	1.6215	0.69736
832.0469	9.24	olomics Po	0.3047	0.99951	1.351	0.43405	0.63326	0.99998	0.92066	-0.11926	0.55482	0.99941	1.0707	0.098589	0.153	0.54891	0.75249	-0.41025	0.013806	0.062303	0.61276	-0.7066	0.14514	0.38608	0.74797	-0.41894	0.97157	0.9974	1.0347	0.049149	0.75615	0.99956	0.96695	-0.04848	0.17435	0.43668	1.5857	0.66508
98.9193	0.57	omics Pos	0.30475	0.99987	1.173	0.23017	0.3188	0.99994	0.91833	-0.12292	0.93874	0.99996	0.99993	-0.0001	0.56249	0.91079	0.95733	-0.06292	0.008484	0.027035	0.8306	-0.26778	0.94005	0.96771	1.0073	0.010462	0.32533	0.73488	1.0855	0.11836	0.19689	0.92582	1.0892	0.12329	0.041901	0.13621	1.1716	0.22848
148.533	5.33	olomics Po	0.30476	0.99951	1.1655	0.22095	0.56296	0.99998	1.085	0.11774	0.92978	0.99941	1.0402	0.056839	0.74714	0.99905	1.0497	0.07002	0.81372	0.99688	0.97699	-0.03359	0.038673	0.15698	1.225	0.29273	0.33409	0.94723	1.0876	0.12112	0.22713	0.99956	1.14	0.18906	0.015996	0.2688	1.2759	0.3515
809.5863	5.42	omics Pos	0.30493	0.99987	1.0512	0.072102	0.14134	0.99994	0.91493	-0.12827	0.44117	0.99996	0.9533	-0.06899	0.40243	0.8455	0.94522	-0.08128	0.00202	0.009127	0.77837	-0.36148	0.44909	0.68033	0.93505	-0.09688	0.43351	0.79394	1.0589	0.082503	0.88034	0.98023	1.0062	0.00891	0.004333	0.085451	1.2401	0.31044
643.1869	1.29	omics Pos	0.30496	0.99987	0.94611	-0.07993	0.064408	0.99994	0.89112	-0.16631	0.23424	0.99996	0.93026	-0.1043	0.12703	0.55024	0.84829	-0.23737	0.003733	0.01451	0.69821	-0.51826	0.11702	0.37012	0.88284	-0.17977	0.26994	0.70383	0.94487	-0.08181	0.35281	0.96776	0.9208	-0.11904	0.34672	0.45746	1.0903	0.12473
288.2151	3.15	olomics Po	0.30514	0.99951	0.77874	-0.36079	0.17747	0.99998	0.7411	-0.43226	0.6635	0.99941	0.90569	-0.14291	0.045024	0.28536	0.59654	-0.74532	0.028661	0.11091	0.44388	-1.1718	0.004661	0.058968	0.20578	-2.2808	0.027176	0.33703	0.33776	-1.5659	0.05738	0.70429	0.43125	-1.2134	0.011287	0.2688	0.24403	-2.0349
854.0596	9.23	olomics Po	0.30544	0.99951	1.2492	0.32099	0.39232	0.99998	0.87381	-0.19461	0.56469	0.99941	1.0931	0.12846	0.14395	0.53536	0.75299	-0.4093	0.037326	0.13515	0.65796	-0.60393	0.11695	0.33273	0.71732	-0.47931	0.49636	0.99391	0.8817	-0.18164	0.55314	0.99956	0.90371	-0.14608	0.1591	0.41695	1.5019	0.58675
899.8021	10.05	omics Pos	0.30562	0.99987	0.91963	-0.12087	0.092818	0.99994	0.86066	-0.21649	0.50451	0.99996	0.94025	-0.08888	0.056488	0.40948	0.82137	-0.2839	0.010798	0.032572	0.71259	-0.48885	0.10539	0.34613	0.8254	-0.27683	0.41386	0.78037	0.92063	-0.11931	0.32818	0.95887	0.90504	-0.14394	0.13568	0.24074	1.1319	0.17869
1022.745	10.06	omics Pos	0.30563	0.99987	1.1033	0.14176	0.63362	0.99994	1.0291	0.041395	0.8564	0.99996	1.0077	0.011121	0.26737	0.72822	0.92956	-0.10539	0.00738	0.024364	0.79781	-0.32588	0.27292	0.55982	0.88918	-0.16945	0.3807	0.76056	1.0892	0.12325	0.44116	0.96776	0.95227	-0.07055	0.056197	0.15336	1.1625	0.21726
226.9523	10.03	omics Pos	0.30572	0.99987	1.0334	0.047432	0.16661	0.99994	0.93669	-0.09436	0.19918	0.99996	0.94907	-0.07541	0.11456	0.53403	0.92928	-0.10581	7.88E-05	0.000882	0.77336	-0.37079	0.27494	0.56241	0.91078	-0.13483	0.84899	0.96798	1.0126	0.01806	0.67778	0.97988	0.98345	-0.02407	0.009357	0.092975	1.1905	0.25162
945.5869	10.35	olomics Ne	0.30605	0.99918	1.0726	0.10113	0.58382	0.88869	0.95032	-0.07352	0.30861	0.99995	1.1161	0.15841	0.26824	0.99985	1.1151	0.15716	0.30437	0.99927	1.0973	0.13393	0.1626	0.63542	1.1605	0.21472	0.047161	0.38963	1.1849	0.24473	0.18747	0.99512	1.1345	0.18208	0.15134	0.99941	1.1067	0.14621
206.9722	10.15	omics Pos	0.30648	0.99987	1.1024	0.14069	0.3633	0.99994	0.92209	-0.11703	0.79215	0.99996	1.0231	0.032983	0.0637	0.43296	0.78089	-0.35681	0.021797	0.055631	0.78306	-0.3528	0.39286	0.64284	0.89895	-0.15368	0.67151	0.89901	1.0363	0.051387	0.938	0.98616	0.99172	-0.01199	0.24084	0.34891	1.2047	0.26863
627.845	10.06	omics Pos	0.30652	0.99987	1.1338	0.18118	0.44179	0.99994	0.88732	-0.17248	0.77999	0.99996	0.95522	-0.0661	0.50924	0.89356	0.91056	-0.13517	0.011814	0.034838	0.64125	-0.64104	0.10571	0.34687	0.77127	-0.37469	0.79984	0.95306	1.0355	0.050386	0.79477	0.97988	0.96097	-0.05743	0.53548	0.63253	1.0618	0.086526
252.9776	12.01	olomics Ne	0.30668	0.99918	1.0902	0.12457	0.20236	0.6246	0.94272	-0.0851	0.046261	0.99995	1.1732	0.23044	0.58262	0.99985	1.0373	0.052768	0.80145	0.99927	0.98989	-0.01466	0.20406	0.65287	1.1144	0.15623	0.058703	0.40009	1.1284	0.17432	0.36196	0.99512	1.0561	0.078711	0.39184	0.99941	0.95792	-0.06203
483.9343	0.56	omics Pos	0.30692	0.99987	1.1774	0.23562	0.034662	0.99994	0.78823	-0.34332	0.94105	0.99996	1.0133	0.019092	0.4396	0.86677	0.91051	-0.13526	0.11707	0.19603	0.7785	-0.36123	0.018618	0.11669	0.76468	-0.38708	0.9021	0.97745	1.01	0.014321	0.52092	0.96776	0.92295	-0.11567	0.24108	0.34913	1.2911	0.36857
685.257	5.24	olomics Ne	0.30722	0.99918	1.0976	0.13438	0.92154	0.9934	1.0216	0.030791	0.023171	0.99995	1.2648	0.33895	0.49778	0.99985	1.1105	0.15115	0.66823	0.99927	1.1032	0.14165	0.056081	0.49666	1.2815	0.35779	0.00383	0.22316	1.3679	0.452	0.26687	0.99512	1.0936	0.12905	0.043241	0.99941	1.2441	0.31515
607.4266	6.82	olomics Po	0.30738	0.99951	0.88921	-0.1694	0.16489	0.99998	0.82559	-0.27651	0.000407	0.99941	0.61813	-0.69403	0.012464	0.20085	0.51084	-0.96907	0.000488	0.005589	0.46139	-1.1159	0.008829	0.075866	0.34384	-1.5402	0.003172	0.20152	0.38501	-1.377	0.001801	0.47452	0.33337	-1.5848	0.023102	0.2688	0.45358	-1.1406
776.5587	8.85	olomics Po	0.3076	0.99951	1.0814	0.11292	0.97764	0.99998	1.0019	0.002707	0.81663	0.99941	0.98246	-0.02552	0.97196	0.99905	1.02	0.028591	0.75702	0.99688	0.973	-0.03948	0.86413	0.95795	0.98771	-0.01785	0.74643	0.99391	0.97126	-0.04207	0.019007	0.51556	0.755	-0.40546	0.004229	0.2688	0.72219	-0.46954
262.226	1.39	omics Pos	0.308	0.99987	1.211	0.2762	0.52724	0.99994	0.85653	-0.22342	0.56683	0.99996	1.0857	0.11859	0.092087	0.50411	0.63305	-0.65961	0.000466	0.003119	0.26722	-1.9039	0.034797	0.16859	0.55952	-0.83773	0.29762	0.72322	0.78519	-0.34888	0.6101	0.97371	0.87838	-0.18709	0.13584	0.2408	1.2913	0.36888
523.2669	0.54	omics Pos	0.30824	0.99987	1.4281	0.51405	0.089516	0.99994	0.78779	-0.34411	0.53319	0.99996	0.94426	-0.08274	0.009997	0.2106	0.58286	-0.77877	0.000127	0.001209	0.42548	-1.2328	0.007425	0.082061	0.62747	-0.67239	0.33618	0.74225	0.86203	-0.2142	0.46962	0.96776	0.90299	-0.14722	0.019561	0.10555	2.043	1.0307
717.3672	0.57	omics Pos	0.30877	0.99987	1.6533	0.72538	0.16674	0.99994	0.79902	-0.3237	0.81306	0.99996	1.0137	0.019615	0.048083	0.37724	0.63164	-0.66283	3.83E-05	0.000535	0.41711	-1.2615	0.006075	0.077469	0.6162	-0.69852	0.81432	0.95934	0.98581	-0.02062	0.95281	0.98838	1.1405	0.18971	0.015913	0.099468	2.4119	1.2701
1134.748	10.03	omics Pos	0.30892	0.99987	1.1367	0.18487	0.76035	0.99994	0.98023	-0.0288	0.39525	0.99996	0.94558	-0.08073	0.90939	0.9874	1.0204	0.029115	0.022166	0.056459	0.78438	-0.35038	0.56455	0.76254	0.94309	-0.08453	0.2762	0.70707	0.93306	-0.09996	0.70364	0.97988	0.97751	-0.03281	0.143	0.24751	1.1239	0.16847
354.3739	8.54	olomics Po	0.30931	0.99951	0.90844	-0.13854	0.027286	0.99998	0.77239	-0.3726	0.15818	0.99941	0.84849	-0.23704	0.35501	0.84597	0.92132	-0.11823	0.91333	0.99688	0.98753	-0.0181	0.011066	0.083281	0.72673	-0.4605	0.23071	0.83662	0.82338	-0.28036	0.051155	0.68379	0.76159	-0.39292	0.090893	0.34353	0.85447	-0.2269
502.3168	0.75	omics Pos	0.30935	0.99987	1.2076	0.27209	0.49876	0.99994	0.88809	-0.17122	0.51238	0.99996	0.8958	-0.15875	0.6439	0.95178	0.92041	-0.11965	0.022985	0.057986	0.61021	-0.71262	0.30105	0.58086	0.7965	-0.32826	0.30922	0.72874	0.84919	-0.23584	0.58468	0.97073	0.90468	-0.14452	0.22287	0.33092	1.2643	0.33839
938.1233	8.91	olomics Po	0.3097	0.99951	1.3397	0.42187	0.60703	0.99998	0.94399	-0.08316	0.36195	0.99941	1.1138	0.15554	0.039583	0.27403	0.7512	-0.41274	0.038138	0.13698	0.66756	-0.58303	0.061338	0.21373	0.81976	-0.28672	0.54972	0.99391	1.2117	0.27706	0.50257	0.99956	1.1305	0.17698	0.097614	0.35101	1.8229	0.8662
1072.676	0.56	omics Pos	0.30992	0.99987	1.0967	0.1332	0.024461	0.99994	0.87896	-0.18612	0.034589	0.99996	0.84387	-0.24491	0.000147	0.164	0.79973	-0.32241	0.007491	0.024646	0.73612	-0.442	0.18668	0.47264	0.84818	-0.23755	0.015185	0.33809	0.8283	-0.27178	0.004527	0.52955	0.8847	-0.17674	0.1058	0.20817	1.1231	0.16755
751.3302	2.43	olomics Po	0.30996	0.99951	0.74645	-0.42189	0.57149	0.99998	1.394	0.47923	0.12297	0.99941	1.7142	0.77753	0.24908	0.71493	1.7765	0.82901	0.028322	0.11014	1.7789	0.83099	0.19563	0.47006	1.3694	0.45357	0.13393	0.68208	1.573	0.65354	0.060975	0.71737	1.7204	0.78274	0.2273	0.49531	0.68968	-0.536
894.6274	6.06	omics Pos	0.31049	0.99987	0.92248	-0.1164	0.034451	0.99994	0.80355	-0.31554	0.21638	0.99996	0.88797	-0.17142	0.096987	0.5165	0.77474	-0.36821	0.00666	0.022632	0.73049	-0.45307	0.13625	0.40368	0.79217	-0.33612	0.24225	0.67954	0.86531	-0.20871	0.13336	0.88792	0.85324	-0.22897	0.01586	0.099299	0.69833	-0.51802
558.9786	5.13	olomics Ne	0.31083	0.99918	1.0576	0.080784	0.29612	0.73345	0.92661	-0.10996	0.89534	0.99995	1.0082	0.011824	0.55257	0.99985	1.0372	0.052643	0.39092	0.99927	1.0905	0.12502	0.161	0.63118	1.121	0.16481	0.047858	0.39078	1.1333	0.18059	0.39876	0.99512	1.0795	0.11036	0.20515	0.99941	1.0743	0.10341
316.9457	0.28	olomics Ne	0.31101	0.99918	0.94389	-0.08331	0.023244	0.40327	0.85112	-0.23257	0.04915	0.99995	0.91113	-0.13427	0.49933	0.99985	0.96674	-0.04881	0.27635	0.99927	0.94622	-0.07975	0.87888	0.9778	1.0246	0.035058	0.8158	0.95121	1.0275	0.039126	0.31378	0.99512	0.93517	-0.09671	0.49205	0.99941	0.96416	-0.05266
946.7338	0.54	omics Pos	0.31123	0.99987	1.0837	0.11594	0.91973	0.99994	1.003	0.004313	0.95449	0.99996	0.98938	-0.0154	0.25152	0.70882	0.87382	-0.19459	0.019095	0.050341	0.71112	-0.49183	0.22536	0.51305	0.86839	-0.20358	0.32409	0.73383	0.89035	-0.16755	0.92937	0.98573	1.0116	0.016574	0.017019	0.099988	1.3441	0.4266
1014.727	10.09	omics Pos	0.31127	0.99987	0.97441	-0.03739	0.023087	0.99994	0.91108	-0.13435	0.29688	0.99996	0.92343	-0.11492	0.871	0.9874	1.0162	0.023184	0.001176	0.006175	0.75829	-0.39917	0.23642	0.52493	0.87249	-0.1968	0.49705	0.82509	1.0692	0.096543	0.21887	0.94047	0.92809	-0.10766	0.33694	0.44719	1.1381	0.18666
329.2429	3.52	omics Pos	0.31137	0.99987	1.0965	0.13285	0.33332	0.99994	0.9067	-0.14131	0.054779	0.99996	0.83158	-0.26608	0.004165	0.17581	0.62598	-0.6758	0.000616	0.003839	0.54321	-0.88043	0.003818	0.06842	0.48668	-1.039	0.025838	0.34128	0.6256	-0.67668	0.003957	0.52955	0.51785	-0.94939	0.25066	0.35958	0.79726	-0.32688
600.839	3.04	olomics Po	0.31152	0.99951	1.2416	0.31221	0.29152	0.99998	0.85577	-0.22471	0.93563	0.99941	0.98517	-0.02155	0.037707	0.26495	0.67782	-0.56102	0.003791	0.024219	0.54133	-0.88542	0.004853	0.060423	0.62516	-0.67771	0.36152	0.96545	0.82597	-0.27585	0.76583	0.99956	0.97178	-0.0413	0.012319	0.2688	1.8324	0.87371
393.2043	2.29	omics Pos	0.31164	0.99987	1.1131	0.1546	0.46199	0.99994	0.93641	-0.09479	0.036466	0.99996	0.81254	-0.29949	0.008705	0.19971	0.74088	-0.43269	0.002679	0.011239	0.63039	-0.66568	0.010154	0.08787	0.61235	-0.70757	0.010751	0.3324	0.73716	-0.43996	0.003767	0.52955	0.62118	-0.68692	0.32743	0.43746	0.91138	-0.13388
787.6038	5.4	omics Pos	0.31212	0.99987	1.0662	0.092517	0.48147	0.99994	0.95525	-0.06605	0.5083	0.99996	0.95252	-0.07018	0.31582	0.77792	0.91427	-0.12931	0.008109	0.026249	0.7745	-0.36866	0.51806	0.72722	0.95694	-0.0635	0.28895	0.71484	1.1159	0.15815	0.39989	0.96776	1.0688	0.095993	0.005155	0.088007	1.3291	0.41049
216.9252	0.56	omics Pos	0.31255	0.99987	1.057	0.079995	0.57509	0.99994	0.97803	-0.03206	0.46112	0.99996	0.97178	-0.0413	0.028742	0.29796	0.90137	-0.1498	0.00209	0.009353	0.77612	-0.36565	0.025228	0.13997	0.85961	-0.21825	0.069616	0.46982	0.92688	-0.10955	0.38609	0.96776	0.95719	-0.06313	0.083808	0.18556	1.151	0.20291
773.8407	2.4	olomics Ne	0.31282	0.99918	0.49025	-1.0284	0.93025	0.9934	1.1182	0.16111	0.37453	0.99995	1.5503	0.63252	0.26125	0.99985	2.7426	1.4555	0.016396	0.88267	5.9323	2.5686	0.054255	0.48752	3.7016	1.8881	0.087034	0.44404	2.7844	1.4774	0.24047	0.99512	2.246	1.1674	0.31038	0.99941	0.48599	-1.041
676.4011	2.7	olomics Po	0.31295	0.99951	1.1989	0.26172	0.23028	0.99998	0.83209	-0.2652	0.43612	0.99941	0.88916	-0.16948	0.003637	0.17363	0.53397	-0.90517	0.000388	0.004677	0.44133	-1.1801	0.000778	0.041543	0.49023	-1.0285	0.39324	0.97985	0.84505	-0.2429	0.86893	0.99956	1.0076	0.010878	0.073347	0.31731	1.6429	0.71624
80.9488	0.57	omics Pos	0.31306	0.99987	1.075	0.10438	0.59904	0.99994	1.0274	0.038996	0.853	0.99996	0.99163	-0.01212	0.051743	0.38992	0.92162	-0.11776	0.006667	0.022636	0.81262	-0.29935	0.026256	0.14302	0.88328	-0.17906	0.52321	0.83957	0.96826	-0.04654	0.46626	0.96776	1.0267	0.037985	0.025815	0.11314	1.2132	0.27887
224.2019	5.99	olomics Po	0.31319	0.99951	1.1296	0.1758	0.47943	0.99998	0.91957	-0.12097	0.84832	0.99941	1.0175	0.025075	0.11829	0.47107	0.76851	-0.37987	3.1E-05	0.000646	0.21213	-2.237	0.025747	0.12015	0.59854	-0.74049	0.17595	0.74875	0.79406	-0.33268	0.15586	0.93927	0.87289	-0.19613	0.087111	0.33918	1.1782	0.2366
1025.207	9.07	olomics Po	0.31357	0.99951	1.2131	0.27874	0.15071	0.99998	0.86102	-0.21587	0.95495	0.99941	1.0216	0.030894	0.072709	0.35072	0.74608	-0.42259	0.036196	0.13246	0.6567	-0.6067	0.15249	0.39928	0.79621	-0.32878	0.96108	0.9974	1.015	0.021516	0.79877	0.99956	1.0143	0.020462	0.41448	0.68645	1.2029	0.26646
647.4482	9.55	olomics Po	0.31391	0.99951	1.2214	0.28855	0.3107	0.99998	0.83841	-0.25428	0.95753	0.99941	1.0065	0.009367	0.047528	0.28918	0.68919	-0.53703	0.021743	0.089767	0.61244	-0.70735	0.19121	0.46368	0.75961	-0.39667	0.66053	0.99391	1.1521	0.20425	0.8332	0.99956	1.0296	0.042152	0.088812	0.3414	1.6898	0.75689
544.9746	12.01	olomics Ne	0.31515	0.99918	0.87982	-0.18473	0.049132	0.47152	0.82121	-0.28417	0.40486	0.99995	0.89261	-0.1639	0.2315	0.99985	0.8709	-0.19942	0.45456	0.99927	0.92363	-0.11461	0.21573	0.66131	0.84615	-0.24102	0.29685	0.67298	0.87005	-0.20082	0.053985	0.99512	0.77778	-0.36257	0.12693	0.99941	0.80233	-0.31774
988.7801	10.07	omics Pos	0.31526	0.99987	0.9079	-0.1394	0.34678	0.99994	0.92184	-0.11742	0.61121	0.99996	1.0679	0.094746	0.004794	0.17581	0.80279	-0.31691	0.00393	0.015074	0.70447	-0.5054	0.076739	0.28715	0.80751	-0.30844	0.83668	0.96659	0.98928	-0.01555	0.10842	0.86492	0.89484	-0.16029	0.082025	0.18348	1.1681	0.22415
776.3603	2.4	olomics Po	0.31561	0.99951	0.49803	-1.0057	0.93461	0.99998	1.2807	0.35691	0.42763	0.99941	1.5343	0.61759	0.29249	0.77885	2.917	1.5445	0.024763	0.099287	5.1837	2.374	0.099457	0.29894	3.0128	1.5911	0.11149	0.64238	2.4697	1.3043	0.25544	0.99956	2.1797	1.1241	0.26577	0.53631	0.42396	-1.238
340.1872	2.79	olomics Po	0.31588	0.99951	1.2099	0.2749	0.037774	0.99998	0.75799	-0.39974	0.30991	0.99941	0.88739	-0.17236	0.005917	0.1948	0.5439	-0.87858	0.000704	0.007113	0.49634	-1.0106	0.000754	0.041543	0.57143	-0.80734	0.35683	0.96457	0.85162	-0.23172	0.38153	0.99956	0.86299	-0.21258	0.043355	0.28452	2.0257	1.0184
206.918	0.57	omics Pos	0.31589	0.99987	1.0802	0.11126	0.89439	0.99994	0.99537	-0.00669	0.56903	0.99996	1.0343	0.048692	0.16217	0.59299	0.94826	-0.07664	0.002821	0.011679	0.85873	-0.21972	0.40553	0.65019	0.94281	-0.08497	0.49906	0.827	1.0598	0.083807	0.39314	0.96776	1.0722	0.10053	0.041026	0.13505	1.1875	0.24788
97.1023	5.98	olomics Po	0.31602	0.99951	1.1032	0.14168	0.39351	0.99998	0.89883	-0.15388	0.90496	0.99941	1.0085	0.012208	0.085826	0.39012	0.74104	-0.43239	1.82E-05	0.000458	0.33727	-1.568	0.011849	0.083709	0.58293	-0.77861	0.33528	0.9489	0.87695	-0.18943	0.76953	0.99956	0.96759	-0.04754	0.74895	0.89304	1.0181	0.025939
1195.743	10.03	omics Pos	0.31611	0.99987	0.92777	-0.10817	0.11852	0.99994	0.84745	-0.2388	0.12389	0.99996	0.86342	-0.21187	0.54897	0.90734	0.93271	-0.1005	0.015304	0.042409	0.77841	-0.3614	0.303	0.58233	0.88226	-0.18073	0.28013	0.70964	0.91548	-0.1274	0.337	0.96524	0.93116	-0.10291	0.67355	0.75698	1.0515	0.072495
1118.221	4.41	omics Pos	0.31626	0.99987	1.276	0.35166	0.21112	0.99994	0.82302	-0.28101	0.78082	0.99996	1.0375	0.053129	0.065527	0.43825	0.65162	-0.61789	0.000445	0.003004	0.44303	-1.1745	0.0238	0.13544	0.61885	-0.69234	0.37388	0.76056	0.86337	-0.21196	0.69081	0.97988	0.96864	-0.04597	0.0536	0.15039	1.6254	0.7008
135.1176	2.77	omics Pos	0.31671	0.99987	1.0857	0.11867	0.051643	0.99994	0.76068	-0.39464	0.12735	0.99996	0.81822	-0.28943	0.026166	0.2867	0.56726	-0.81792	2.9E-06	0.000144	0.16303	-2.6168	0.000662	0.03855	0.40824	-1.2925	0.017702	0.33809	0.5918	-0.75681	0.044383	0.65325	0.70901	-0.49613	0.91641	0.93756	0.99603	-0.00574
424.9786	4.78	olomics Ne	0.31673	0.99918	1.0211	0.030173	0.003953	0.34605	0.88806	-0.17127	0.19522	0.99995	1.0228	0.032507	0.86785	0.99985	1.0141	0.020201	0.37901	0.99927	0.9601	-0.05875	0.38694	0.78343	1.0641	0.089699	0.13696	0.52448	1.0769	0.10695	0.77762	0.99512	1.0177	0.025243	0.85989	0.99941	0.99467	-0.00772
211.1899	1.66	omics Pos	0.31695	0.99987	1.122	0.16608	0.63277	0.99994	0.92587	-0.11112	0.79232	0.99996	0.95752	-0.06263	0.18731	0.62759	0.75331	-0.40869	3.23E-05	0.000487	0.24609	-2.0228	0.024373	0.13732	0.65085	-0.6196	0.4809	0.81375	0.89289	-0.16345	0.53581	0.96776	0.90314	-0.14697	0.022151	0.10842	1.392	0.47715
456.3087	2.06	omics Pos	0.31699	0.99987	1.0942	0.12984	0.063496	0.99994	0.7809	-0.35678	0.97359	0.99996	0.99744	-0.0037	0.41307	0.85524	0.88755	-0.17211	3.13E-05	0.000482	0.27867	-1.8434	0.006834	0.080671	0.63514	-0.65485	0.49106	0.82123	0.92204	-0.1171	0.71923	0.97988	1.0262	0.037326	0.078856	0.1809	1.281	0.35726
160.6149	2.06	omics Pos	0.3171	0.99987	1.0589	0.082529	0.41986	0.99994	0.91138	-0.13388	0.27086	0.99996	0.91012	-0.13588	0.071566	0.4548	0.76059	-0.3948	2.45E-05	0.000418	0.32861	-1.6056	0.013938	0.099586	0.6649	-0.5888	0.42475	0.78878	0.88216	-0.18089	0.73126	0.97988	0.97211	-0.04081	0.25093	0.35984	1.0632	0.088437
801.9794	12.04	olomics Ne	0.31728	0.99918	0.86005	-0.21751	0.02955	0.41811	0.66938	-0.5791	0.7091	0.99995	0.95174	-0.07137	0.21184	0.99985	0.82339	-0.28036	0.14789	0.99927	0.8046	-0.31366	0.22326	0.66171	0.85579	-0.22468	0.27804	0.65948	0.8698	-0.20124	0.50771	0.99512	0.90573	-0.14284	0.039791	0.99941	0.70999	-0.49413
409.2488	2.72	omics Pos	0.31735	0.99987	1.1086	0.14872	0.19035	0.99994	0.84449	-0.24385	0.10656	0.99996	0.84534	-0.24239	0.006927	0.19397	0.66049	-0.59839	0.000404	0.002785	0.49344	-1.0191	0.004147	0.070915	0.50242	-0.99304	0.085742	0.51084	0.66385	-0.59107	0.031471	0.62711	0.61169	-0.70914	0.27158	0.38093	0.84879	-0.23652
327.2255	2.19	omics Pos	0.31764	0.99987	0.84602	-0.24123	0.88946	0.99994	0.98538	-0.02125	0.029616	0.99996	0.69906	-0.51652	0.010813	0.21362	0.75283	-0.4096	9.86E-07	0.000144	0.19152	-2.3845	0.015244	0.10398	0.6001	-0.73673	0.073168	0.48057	0.76201	-0.39212	0.07218	0.77255	0.70158	-0.51131	0.15501	0.26095	0.73628	-0.44168
423.7664	4.78	omics Pos	0.31876	0.99987	1.106	0.14538	0.33781	0.99994	0.92092	-0.11885	0.92048	0.99996	1.0217	0.030951	0.28968	0.75244	0.89411	-0.16147	0.025422	0.062579	0.711	-0.49208	0.11874	0.37382	0.8022	-0.31796	0.96351	0.99133	1.0219	0.031307	0.22987	0.94286	0.8576	-0.22162	0.55219	0.64781	0.94987	-0.0742
478.7841	3.04	olomics Po	0.31879	0.99951	1.265	0.33919	0.027102	0.99998	0.77433	-0.36898	0.4393	0.99941	0.90998	-0.13609	0.000195	0.077904	0.57472	-0.79908	0.003842	0.024397	0.59348	-0.75274	0.000187	0.036471	0.56044	-0.83536	0.51346	0.99391	0.92346	-0.11488	0.74409	0.99956	0.9873	-0.01844	0.047844	0.28861	1.8768	0.90827
164.0675	0.6	lomics Neg	0.31897	0.99977	1.1317	0.17845	0.60461	0.99904	0.94541	-0.081	0.24808	0.99994	1.1986	0.26138	0.005418	0.58182	1.8806	0.91123	0.033365	0.65839	1.4493	0.53539	0.013416	0.46473	1.4679	0.55371	0.019369	0.30175	1.5104	0.59496	0.91449	0.95313	1.1156	0.15776	0.56627	0.72708	0.93584	-0.09567
1058.769	10.05	omics Pos	0.31899	0.99987	0.94104	-0.08767	0.17894	0.99994	0.90417	-0.14533	0.30991	0.99996	0.92847	-0.10707	0.016126	0.24	0.83321	-0.26324	0.001703	0.008007	0.72327	-0.46739	0.080234	0.29304	0.85346	-0.22861	0.75214	0.93661	1.0284	0.040469	0.44546	0.96776	0.9449	-0.08177	0.022094	0.10842	1.2084	0.27306
831.8065	10.06	omics Pos	0.32008	0.99987	1.0722	0.10054	0.52231	0.99994	0.95349	-0.06871	0.88387	0.99996	0.98813	-0.01723	0.94392	0.99212	1.0088	0.012609	0.024091	0.059921	0.75196	-0.41128	0.74934	0.87057	0.97229	-0.04055	0.7322	0.92699	1.0307	0.043577	0.86663	0.98023	1.0213	0.030351	0.054645	0.15191	1.3712	0.45543
336.9751	4.27	olomics Ne	0.32013	0.99918	1.0256	0.036512	0.026967	0.40327	0.88409	-0.17774	0.69405	0.99995	0.98721	-0.01858	0.79472	0.99985	0.98935	-0.01545	0.5416	0.99927	0.97463	-0.03707	0.27356	0.6999	1.0573	0.080348	0.17548	0.5503	1.05	0.070362	0.38452	0.99512	1.0389	0.055047	0.89766	0.99941	0.99572	-0.00619
257.9485	10.18	omics Pos	0.32021	0.99987	0.90124	-0.15001	0.42408	0.99994	0.92578	-0.11125	0.20448	0.99996	0.88395	-0.17797	0.11658	0.53727	0.87932	-0.18553	0.048231	0.10155	0.80926	-0.30533	0.24485	0.53144	0.84865	-0.23676	0.75176	0.93661	0.97378	-0.03833	0.24249	0.94365	0.90245	-0.14808	0.28572	0.39616	1.1326	0.17965
1140.272	6.2	omics Pos	0.32044	0.99987	1.3103	0.38992	0.3223	0.99994	0.84386	-0.24493	0.97355	0.99996	0.98815	-0.0172	0.048564	0.3779	0.60595	-0.72273	0.007574	0.024833	0.48243	-1.0516	0.023396	0.13415	0.64732	-0.62744	0.5903	0.86173	0.90916	-0.13739	0.95102	0.98838	1.0605	0.084767	0.10428	0.20689	1.9657	0.97501
713.4821	9.46	olomics Po	0.32057	0.99951	1.2626	0.3364	0.17299	0.99998	0.82755	-0.27309	0.76063	0.99941	1.0488	0.068791	0.013145	0.20085	0.67551	-0.56595	0.01142	0.054095	0.6284	-0.67024	0.018644	0.10085	0.69219	-0.53076	0.68967	0.99391	0.96494	-0.05149	0.94654	0.99956	1.0218	0.031129	0.10994	0.36416	1.6912	0.75806
538.8017	0.56	omics Pos	0.32059	0.99987	1.1041	0.14287	0.93665	0.99994	0.99865	-0.00195	0.93708	0.99996	0.99099	-0.01305	0.16932	0.60365	0.8733	-0.19545	0.023257	0.058432	0.75651	-0.40257	0.27647	0.56257	0.88548	-0.17546	0.28864	0.71484	0.88227	-0.18071	0.78747	0.97988	0.97116	-0.04222	0.093642	0.19426	1.2755	0.35102
624.4432	9.6	olomics Po	0.32098	0.99951	1.3202	0.40071	0.31807	0.99998	0.89688	-0.15701	0.90922	0.99941	1.0159	0.022693	0.008156	0.19613	0.64235	-0.63856	0.000338	0.004244	0.58385	-0.77632	0.009785	0.079234	0.65446	-0.61162	0.81845	0.99391	1.0134	0.019238	0.81404	0.99956	1.0413	0.058337	0.12761	0.37779	1.7025	0.76767
841.8162	0.53	omics Pos	0.32132	0.99987	0.94432	-0.08265	0.028046	0.99994	0.84249	-0.24728	0.33453	0.99996	0.957	-0.06341	0.89774	0.9874	1.0161	0.022989	0.006844	0.022971	0.78107	-0.35648	0.25375	0.54024	0.89071	-0.16697	0.89337	0.97532	0.99481	-0.00751	0.52378	0.96776	0.94889	-0.07568	0.011734	0.096289	1.2284	0.29677

896.7973	10.03	domics Pos	0.32146	0.99987	1.0659	0.09205	0.52328	0.99994	0.96517	-0.05115	0.46955	0.99996	0.96346	-0.0537	0.6554	0.95652	0.97241	-0.04036	0.001135	0.006019	0.7582	-0.39935	0.57576	0.76841	0.95966	-0.05941	0.50399	0.82976	1.0458	0.064548	0.87072	0.98023	1.0098	0.014042	0.041323	0.1356	1.1797	0.23847
167.0708	0.84	domics Pos	0.3217	0.99987	0.92039	-0.11968	0.089628	0.99994	0.7529	-0.40948	0.26797	0.99996	0.87326	-0.19551	0.21024	0.65763	0.89743	-0.15612	0.005043	0.018364	0.70387	-0.50662	0.012693	0.095104	0.66361	-0.59159	0.21792	0.66052	0.82237	-0.28214	0.024089	0.55332	0.78319	-0.35257	0.47815	0.58081	0.9268	-0.10968
436.7709	3.04	oolomics Po	0.32174	0.99951	1.3967	0.48199	0.11392	0.99998	0.81924	-0.28764	0.32881	0.99941	0.89766	-0.15575	0.007619	0.1948	0.64894	-0.62385	7.54E-05	0.001291	0.50546	-0.98433	0.016858	0.096027	0.68757	-0.54041	0.87897	0.9951	1.0234	0.033347	0.61408	0.99956	0.9525	-0.0702	0.041979	0.28426	1.7392	0.79845
427.254	7.49	oolomics Po	0.3222	0.99951	0.82394	-0.2794	0.88781	0.99998	0.9865	-0.0196	0.133	0.99941	0.7715	-0.37426	0.044034	0.28334	0.65244	-0.61609	0.096244	0.27712	0.72494	-0.46406	0.017448	0.097447	0.58154	-0.78205	0.049258	0.44845	0.69268	-0.52974	0.008971	0.47452	0.54729	-0.86963	0.17834	0.44185	0.71482	-0.48435
779.5772	5.62	domics Pos	0.32222	0.99987	1.082	0.11375	0.7736	0.99994	0.97406	-0.03791	0.53333	0.99996	0.9435	-0.0839	0.31186	0.77382	0.90886	-0.13787	0.0286	0.068371	0.78729	-0.34502	0.2014	0.48967	0.88601	-0.17461	0.88596	0.97532	1.0207	0.029563	0.40031	0.96776	0.93143	-0.10247	0.35986	0.46895	0.93323	-0.0997
1028.19	9.39	oolomics Po	0.32255	0.99951	1.3582	0.44169	0.032423	0.99998	0.74787	-0.41914	0.88295	0.99941	1.0172	0.02463	0.01068	0.19754	0.62731	-0.67274	0.000641	0.006646	0.53807	-0.89414	0.002417	0.046224	0.70439	-0.50554	0.8244	0.9951	1.0513	0.072166	0.88913	0.99956	1.0517	0.07276	0.13734	0.39223	1.6148	0.69134
363.9877	4.3	olomics Ne	0.32288	0.99918	1.09	0.12427	0.0369	0.45581	0.86989	-0.2011	0.31512	0.99995	0.9531	-0.0693	0.72028	0.99985	0.9808	-0.02796	0.85988	0.99927	1.0187	0.026757	0.52938	0.87267	1.032	0.0454	0.46815	0.7927	1.0328	0.046616	0.22821	0.99512	0.97363	-0.03856	0.18412	0.99941	1.0742	0.10327
154.1242	5.99	oolomics Po	0.323	0.99951	1.1613	0.2157	0.8656	0.99998	0.94455	-0.08231	0.56517	0.99941	1.0915	0.12626	0.24804	0.71367	0.75439	-0.40662	0.000281	0.003669	0.25114	-1.9934	0.038229	0.1558	0.5618	-0.83188	0.30751	0.92549	0.78551	-0.3483	0.88563	0.99956	0.95347	-0.06874	0.49894	0.75091	1.0856	0.11854
202.1807	0.52	domics Pos	0.32303	0.99987	1.1725	0.22953	0.017846	0.99994	0.70711	-0.49999	0.94318	0.99996	0.98018	-0.02888	0.15271	0.58077	0.73707	-0.44012	0.005526	0.019693	0.48891	-1.0324	0.072275	0.27658	0.63976	-0.64439	0.53305	0.84189	0.90173	-0.14923	0.24949	0.94365	0.83044	-0.26804	0.17558	0.28332	1.2515	0.3237
776.8614	2.4	oolomics Po	0.32307	0.99951	0.49706	-1.0085	0.9143	0.99998	1.3854	0.47027	0.34105	0.99941	1.6247	0.70016	0.31815	0.81279	2.966	1.5685	0.024118	0.097651	5.3008	2.4062	0.10863	0.31749	3.0175	1.5934	0.087995	0.60275	2.562	1.3573	0.2339	0.99956	2.3731	1.2468	0.26417	0.53513	0.42143	-1.2466
1017.787	10.03	domics Pos	0.32392	0.99987	1.0748	0.10413	0.14777	0.99994	0.88877	-0.17012	0.44528	0.99996	0.93342	-0.0994	0.21642	0.66737	0.89999	-0.15202	0.001196	0.006255	0.67943	-0.55759	0.17104	0.45832	0.86592	-0.20769	0.87583	0.97532	0.99031	-0.01405	0.74065	0.97988	0.97305	-0.03941	0.2598	0.36936	1.1366	0.18467
366.2325	6.39	oolomics Po	0.32423	0.99951	1.082	0.11368	0.22209	0.99998	0.89299	-0.16329	0.067717	0.99941	0.86249	-0.21342	0.038445	0.26912	0.78817	-0.34342	4.2E-06	0.000231	0.27548	-1.86	0.038441	0.15641	0.67594	-0.56503	0.075766	0.56292	0.76687	-0.38294	0.15832	0.93927	0.90547	-0.14326	0.36507	0.64424	0.92621	-0.11058
450.2501	3.05	oolomics Po	0.32454	0.99951	1.3206	0.40118	0.034485	0.99998	0.8642	-0.21056	0.25767	0.99941	0.89937	-0.15301	0.009648	0.19702	0.65187	-0.61735	0.01007	0.048653	0.66752	-0.58311	0.007301	0.070977	0.70105	-0.5124	0.89006	0.9951	1.0353	0.049981	0.71818	0.99956	1.0138	0.01982	0.020172	0.2688	1.9431	0.95837
1119.261	6.23	domics Pos	0.32503	0.99987	1.3632	0.44699	0.30422	0.99994	0.86612	-0.20736	0.54447	0.99996	1.0843	0.1168	0.029503	0.30146	0.56512	-0.82338	0.001307	0.006671	0.44458	-1.1695	0.008593	0.084941	0.59226	-0.75569	0.56875	0.85801	0.91018	-0.13578	0.96127	0.9885	1.0463	0.065305	0.22381	0.33143	1.7588	0.81456
825.9763	12.05	olomics Ne	0.32505	0.99918	1.154	0.20668	0.43925	0.8168	0.88715	-0.17275	0.96688	0.99995	0.9943	-0.00825	0.79249	0.99985	1.0491	0.069221	0.65786	0.99927	1.1194	0.16266	0.53383	0.87267	1.1552	0.20816	0.014122	0.27595	1.451	0.53705	0.28369	0.99512	1.3892	0.47422	0.99556	0.99945	1.0191	0.027307
860.8595	11.96	olomics Ne	0.32518	0.99918	0.96439	-0.05232	0.043559	0.47152	0.88218	-0.18086	0.34252	0.99995	0.9223	-0.1167	0.17579	0.99985	0.90244	-0.14809	0.50553	0.99927	0.96551	-0.05064	0.26713	0.69376	1.072	0.10027	0.69461	0.90102	0.97765	-0.03261	0.13769	0.99512	0.93774	-0.09274	0.32822	0.99941	0.93946	-0.0901
476.3044	2.5	oolomics Po	0.32567	0.99951	1.3165	0.39667	0.019078	0.99998	0.80043	-0.32115	0.91828	0.99941	0.99543	-0.0066	0.001215	0.13475	0.66038	-0.59864	0.000119	0.00184	0.51892	-0.94641	0.017357	0.097219	0.72677	-0.46042	0.97667	0.9974	1.0483	0.068049	0.79808	0.99956	0.98731	-0.01842	0.11195	0.36506	1.7525	0.80941
101.9405	12.71	olomics Ne	0.32604	0.99918	1.0269	0.038352	0.073481	0.50118	0.9222	-0.11685	0.36502	0.99995	1.0407	0.057501	0.64942	0.99985	1.0263	0.037458	0.75266	0.99927	1.0174	0.024869	0.03259	0.47167	1.1382	0.1868	0.009333	0.25531	1.0912	0.12586	0.10933	0.99512	1.0629	0.087987	0.69589	0.99941	1.0153	0.021839
690.9725	9.49	oolomics Po	0.32615	0.99951	1.2905	0.36793	0.32155	0.99998	0.88602	-0.17459	0.56128	0.99941	1.0566	0.079469	0.030887	0.24688	0.67295	-0.57144	0.003088	0.021006	0.56049	-0.83524	0.016317	0.094477	0.70423	-0.50589	0.79213	0.99391	0.98896	-0.01602	0.90187	0.99956	1.0423	0.059756	0.1144	0.36506	1.748	0.80573
404.7893	5.41	domics Pos	0.32632	0.99987	1.0966	0.13304	0.56376	0.99994	0.93677	-0.09423	0.40806	0.99996	0.91445	-0.12903	0.34449	0.79913	0.88619	-0.17431	0.020762	0.053855	0.76614	-0.38431	0.71193	0.85043	0.95918	-0.06012	0.68283	0.90442	1.0275	0.039203	0.93476	0.98616	0.98481	-0.02209	0.025983	0.11336	1.2509	0.32302
278.8529	12.73	olomics Ne	0.32652	0.99918	1.0645	0.09021	0.14807	0.57863	0.88584	-0.17488	0.5779	0.99995	1.0412	0.058284	0.9141	0.99985	0.98867	-0.01644	0.95841	0.99927	0.99422	-0.00836	0.049939	0.48405	1.194	0.25578	0.23951	0.62339	1.0805	0.11171	0.35713	0.99512	1.0633	0.088579	0.54324	0.99941	0.94639	-0.07949
1060.77	10.07	domics Pos	0.32657	0.99987	0.96476	-0.05176	0.76543	0.99994	0.98361	-0.02384	0.39758	0.99996	0.95473	-0.06684	0.2832	0.74886	0.91185	-0.13313	0.006282	0.021658	0.80923	-0.30537	0.60089	0.78399	0.96368	-0.05337	0.67047	0.89896	1.0488	0.068684	0.17599	0.91806	1.1413	0.19071	0.037285	0.13067	1.2543	0.32693
331.983	4.21	olomics Ne	0.32688	0.99918	1.0445	0.062871	0.016909	0.37363	0.90985	-0.1363	0.53306	0.99995	1.018	0.025744	0.80727	0.99985	1.0049	0.007022	0.18634	0.99927	1.0911	0.12584	0.41753	0.81191	1.0845	0.11704	0.82387	0.95121	1.0127	0.018199	0.88539	0.99512	1.0212	0.030196	0.18838	0.99941	1.0662	0.092414
849.6376	10.36	olomics Ne	0.32696	0.99918	0.95681	-0.0637	0.002525	0.34605	0.84572	-0.24175	0.12842	0.99995	0.90587	-0.14263	0.14966	0.99985	0.91627	-0.12615	0.61162	0.99927	0.97906	-0.03053	0.63469	0.92263	0.97164	-0.04151	0.58735	0.86041	0.97881	-0.0309	0.30755	0.99512	0.92635	-0.11036	0.004398	0.99941	0.8302	-0.26847
960.638	8.89	oolomics Po	0.32723	0.99951	1.309	0.38845	0.5037	0.99998	0.91624	-0.1262	0.37877	0.99941	1.0926	0.12774	0.010422	0.19754	0.68479	-0.54626	0.02501	0.09985	0.64464	-0.63344	0.10413	0.30909	0.82271	-0.28154	0.84298	0.9951	1.1104	0.15113	0.9026	0.99956	1.0091	0.01301	0.10063	0.35342	1.743	0.80156
1052.176	9.06	oolomics Po	0.32741	0.99951	1.3621	0.44582	0.4018	0.99998	0.86884	-0.20284	0.62394	0.99941	1.0571	0.080143	0.17918	0.6038	0.79822	-0.32515	0.049317	0.16686	0.65263	-0.61565	0.47422	0.7756	0.89767	-0.15574	0.87518	0.9951	1.1208	0.16455	0.98879	0.99956	1.0338	0.047903	0.36942	0.64663	1.3527	0.43587
271.2437	4.73	domics Pos	0.32805	0.99987	0.88597	-0.17467	0.25106	0.99994	0.84432	-0.24414	0.96977	0.99996	1.0278	0.039622	0.79342	0.97671	0.96316	-0.05415	0.035769	0.080977	0.74573	-0.42327	0.28969	0.57347	0.85043	-0.23374	0.53309	0.84189	1.0965	0.13297	0.69055	0.97988	1.0489	0.068913	0.51359	0.61418	1.1484	0.19966
567.8929	10.02	domics Pos	0.3281	0.99987	1.0735	0.10238	0.36073	0.99994	0.93595	-0.0955	0.50663	0.99996	0.95025	-0.07362	0.17753	0.61058	0.88219	-0.18084	0.008002	0.025965	0.76558	-0.38538	0.34985	0.61843	0.90233	-0.14828	0.76016	0.94108	1.0188	0.02694	0.39453	0.96776	0.93427	-0.09808	0.053701	0.15056	1.2026	0.26611
888.7705	10.07	domics Pos	0.32834	0.99987	1.0567	0.079524	0.44409	0.99994	1.0639	0.089337	0.74884	0.99996	1.029	0.04119	0.41424	0.85524	0.97106	-0.04238	0.00022	0.001797	0.79456	-0.33177	0.27291	0.55982	0.89799	-0.15522	0.27092	0.70502	1.0781	0.10847	0.63044	0.97988	0.98008	-0.02903	0.001487	0.07528	1.2802	0.35641
668.9628	9.53	oolomics Po	0.32841	0.99951	1.4143	0.50007	0.30518	0.99998	0.87465	-0.19322	0.72012	0.99941	1.0352	0.049893	0.01742	0.20936	0.62175	-0.68559	0.001681	0.013605	0.5126	-0.96408	0.044496	0.17219	0.6473	-0.6275	0.5984	0.99391	0.93185	-0.10184	0.82578	0.99956	1.0243	0.034644	0.15861	0.41601	1.5451	0.62771
963.1264	4.35	domics Pos	0.3285	0.99987	1.3525	0.43568	0.70367	0.99994	0.92452	-0.11322	0.46351	0.99996	1.1096	0.15004	0.12241	0.54337	0.69259	-0.52994	0.003899	0.015015	0.48817	-1.0345	0.033243	0.16398	0.62112	-0.68706	0.73821	0.93051	0.95299	-0.06946	0.72631	0.97988	1.2195	0.28624	0.17177	0.27941	1.7324	0.7928
769.5922	5.17	domics Pos	0.32882	0.99987	1.0955	0.13153	0.88761	0.99994	1.0141	0.020269	0.86113	0.99996	1.0149	0.021372	0.34158	0.7973	0.93337	-0.09948	0.014057	0.039791	0.80452	-0.3138	0.54332	0.74569	0.94612	-0.0799	0.47059	0.80647	1.0965	0.13284	0.95329	0.98838	1.0054	0.007807	0.28861	0.39873	1.0793	0.11005
996.8354	11.96	olomics Ne	0.32912	0.99918	0.94959	-0.07462	0.016066	0.37315	0.87228	-0.19714	0.58379	0.99995	0.96004	-0.05884	0.21286	0.99985	0.92022	-0.11995	0.73805	0.99927	0.98014	-0.02894	0.33809	0.75413	1.0615	0.086128	0.19984	0.57883	0.91677	-0.12536	0.29351	0.99512	0.91079	-0.1348	0.9708	0.99945	1.0027	0.003828
313.3298	7.56	oolomics Po	0.33003	0.99951	1.1373	0.18563	0.18204	0.99998	0.85558	-0.22503	0.32174	0.99941	0.88162	-0.18177	0.20934	0.64967	0.81803	-0.28978	0.001545	0.012837	0.57158	-0.80697	0.00232	0.045971	0.5242	-0.93181	0.017609	0.30109	0.69583	-0.52319	0.010871	0.47452	0.71597	-0.48204	0.25483	0.52539	1.1743	0.23177
190.1673	1.66	domics Pos	0.33006	0.99987	1.1884	0.24899	0.59247	0.99994	0.90196	-0.14887	0.76231	0.99996	0.93853	-0.09153	0.2714	0.73186	0.80632	-0.31057	8.92E-05	0.000961	0.2662	-1.9094	0.02271	0.132	0.5812	-0.78289	0.24475	0.68168	0.78124	-0.35617	0.85253	0.97988	0.95455	-0.06711	0.093622	0.19426	1.2688	0.34343
458.9747	12	olomics Ne	0.33027	0.99918	0.92369	-0.11453	0.004227	0.34605	0.78782	-0.34407	0.29352	0.99995	0.90433	-0.14507	0.00925	0.99985	0.81008	-0.30387	0.41073	0.99927	0.92019	-0.11999	0.43142	0.82084	0.91577	-0.12694	0.001285	0.22316	0.84274	-0.24683	0.030526	0.99512	0.79586	-0.32941	0.29979	0.99941	0.91042	-0.13539
827.5625	8.6	olomics Ne	0.33043	0.99918	1.0446	0.063018	0.1985	0.61863	0.93076	-0.10351	0.64994	0.99995	0.97476	-0.03688	0.97223	0.99985	1.0115	0.016485	0.11268	0.99927	1.1056	0.14479	0.000246	0.10078	1.3307	0.41223	0.002196	0.22316	1.2477	0.31929	0.12528	0.99512	1.1271	0.17263	0.10741	0.99941	1.0815	0.11304
628.3841	3.08	oolomics Po	0.33045	0.99951	1.2568	0.32974	0.009315	0.99998	0.72523	-0.46348	0.021721	0.99941	0.74405	-0.42653	0.000797	0.12936	0.53834	-0.89341	0.001765	0.01411	0.5934	-0.75293	9.78E-05	0.034543	0.53516	-0.90197	0.36355	0.96545	0.87439	-0.19365	0.26983	0.99956	0.82076	-0.28496	0.10532	0.35981	1.5616	0.64298
276.842	0.32	olomics Ne	0.3305	0.99918	0.92549	-0.11171	0.001749	0.34605	0.86476	-0.20964	0.71025	0.99995	0.97764	-0.03263	0.10293	0.99985	1.1036	0.14226	0.22909	0.99927	0.91224	-0.13251	0.18113	0.65203	1.0953	0.13135	0.024833	0.33228	1.076	0.10562	0.07096	0.99512	0.9489	-0.07567	0.78768	0.99941	0.98727	-0.01849
817.7072	6.41	domics Pos	0.33137	0.99987	0.82855	-0.27133	0.031807	0.99994	0.64076	-0.64214	0.12639	0.99996	0.74046	-0.43351	0.21961	0.67258	0.78268	-0.35351	0.062723	0.12322	0.62111	-0.68708	0.25167	0.5388	0.7085	-0.49716	0.83008	0.96527	1.0571	0.080154	0.19499	0.92582	0.68156	-0.55308	0.34963	0.45943	0.8159	-0.29354
243.212	5.65	oolomics Po	0.33137	0.99951	1.1706	0.22721	0.61864	0.99998	1.0803	0.11144	0.43505	0.99941	1.0992	0.13646	0.63045	0.99905	0.93966	-0.08979	7.01E-05	0.00122	0.32274	-1.6316	0.01861	0.10085	0.60085	-0.73491	0.52108	0.99391	0.91334	-0.13078	0.32263	0.99956	1.1035	0.14209	0.13962	0.39361	1.1822	0.24149
962.6518	9.45	oolomics Po	0.33191	0.99951	1.2721	0.34727	0.16831	0.99998	0.83058	-0.26781	0.44485	0.99941	1.0733	0.10205	0.01467	0.20268	0.67517	-0.56669	0.012696	0.058298	0.65249	-0.61598	0.00218	0.045856	0.59269	-0.75465	0.42356	0.99125	0.87984	-0.18468	0.50888	0.99956	0.90556	-0.14312	0.044742	0.28476	1.6758	0.74488
801.5372	4.6	domics Pos	0.33228	0.99987	0.83713	-0.25647	0.68534	0.99994	0.96259	-0.05501	0.89819	0.99996	1.0669	0.093406	0.67026	0.95717	0.96775	-0.0473	0.53756	0.60589	0.91764	-0.124	0.11882	0.37382	1.4909	0.57621	0.016863	0.33809	1.9069	0.93123	0.353	0.96776	1.356	0.43939	0.03535	0.12781	1.9039	0.92898
562.81	10.13	domics Pos	0.33235	0.99987	0.92084	-0.11898	0.10958	0.99994	0.85284	-0.22966	0.1864	0.99996	0.904	-0.14561	0.1639	0.59595	0.88297	-0.17957	0.004654	0.017264	0.74451	-0.42564	0.22942	0.51761	0.89245	-0.16415	0.71075	0.91681	0.97231	-0.04052	0.70878	0.97988	0.97201	-0.04095	0.20249	0.31141	1.1162	0.15865
263.9211	10.17	domics Pos	0.33309	0.99987	0.94639	-0.0795	0.57501	0.99994	0.97326	-0.03911	0.36901	0.99996	0.93177	-0.10195	0.18842	0.63077	0.8964	-0.15779	0.035785	0.080977	0.80579	-0.31153	0.3365	0.60864	0.89045	-0.16739	0.10092	0.54271	0.91187	-0.1331	0.90069	0.98023	1.0131	0.018739	0.001511	0.07528	1.2499	0.32177
1096.703	9.04	oolomics Po	0.33333	0.99951	1.3897	0.47474	0.83267	0.99998	1.0079	0.011373	0.95058	0.99941	1.0188	0.026921	0.015533	0.20428	0.72025	-0.47344	0.052255	0.17428	0.6897	-0.53595	0.15532	0.40477	0.84326	-0.24596	0.41166	0.98164	1.2364	0.30609	0.67043	0.99956	1.0962	0.1325	0.022745	0.2688	1.7108	0.77465
965.7199	9.88	oolomics Po	0.33365	0.99951	1.5854	0.66488	0.98213	0.99998	0.99413	-0.00849	0.3216	0.99941	1.1969	0.25933	0.076703	0.36221	0.67628	-0.5643	0.024838	0.099287	0.58464	-0.77439	0.13782	0.37527	0.72767	-0.45865	0.52555	0.99391	1.2071	0.27155	0.92155	0.99956	1.208	0.2726	0.073038	0.31731	1.8519	0.88898
949.5032	4.77	domics Pos	0.33389	0.99987	0.89854	-0.15435	0.10599	0.99994	0.81591	-0.29351	0.37526	0.99996	0.89688	-0.15701	0.040491	0.34959	0.79111	-0.33804	0.032325	0.07511	0.70462	-0.50509	0.070239	0.27107	0.79844	-0.32474	0.30962	0.72874	0.89639	-0.1578	0.24936	0.94365	0.88273	-0.17996	0.74388	0.80855	1.0482	0.06792
148.1174	4.74	domics Pos	0.33409	0.99987	1.154	0.2066	0.51904	0.99994	0.93823	-0.09199	0.63668	0.99996	0.96503	-0.05136	0.82061	0.98389	0.98108	-0.02755	0.023584	0.058969	0.78133	-0.356	0.99483	0.9964	1.0103	0.014829	0.46885	0.80647	1.0579	0.081194	0.071893	0.77255	0.84182	-0.24841	0.22407	0.33154	1.1378	0.18627
498.902	10.02	domics Pos	0.33421	0.99987	1.0303	0.043026	0.27603	0.99994	0.94751	-0.07779	0.33626	0.99996	0.96116	-0.05716	0.19658	0.64175	0.93506	-0.09687	9.18E-05	0.000972	0.77346	-0.3706	0.3839	0.63866	0.92493	-0.11259	0.87975	0.97532	0.99414	-0.00848	0.74457	0.97988	0.98591	-0.02048	0.010176	0.094875	1.2081	0.27275
334.2884	2.75	domics Pos	0.3348	0.99987	1.2063	0.27055	0.7336	0.99994	0.88123	-0.18241	0.5285	0.99996	0.79166	-0.33705	0.20042	0.64674	0.63949	-0.64501	0.002323	0.010124	0.26192	-1.9328	0.023405	0.13415	0.42262	-1.2426	0.035431	0.36525	0.46413	-1.1074	0.13561	0.89461	0.5874	-0.76758	0.57943	0.67318	1.1151	0.15717
1052.221	6.34	domics Pos	0.33512	0.99987	1.3443	0.42684	0.064491	0.99994	0.8031	-0.31635	0.7963	0.99996	0.96724	-0.04806	0.014406	0.22983	0.64769	-0.62663	0.000585	0.003704	0.4775	-1.0664	0.042225	0.19305	0.62923	-0.66833	0.50494	0.8305	0.90104	-0.15033	0.8653	0.98023	1.1122	0.1534	0.21196	0.32071	1.8878	0.91673
191.0951	10.16	domics Pos	0.33519	0.99987	1.0609	0.08529	0.10053	0.99994	0.90275	-0.14761	0.88145	0.99996	0.99336	-0.00961	0.77664	0.97526	0.98744	-0.01823	0.000995	0.005516	0.78356	-0.35189	0.35285	0.61858	0.91369	-0.13022	0.5698	0.85811	1.0399	0.056493	0.90473	0.98197	0.99607	-0.00569	0.015297	0.09885	1.2511	0.32319
607.9784	12.02	olomics Ne	0.33519	0.99918	1.1063	0.14568	0.78616	0.97655	0.98716	-0.01864	0.58608	0.99995	1.0616	0.086175	0.72147	0.99985	1.0499	0.070263	0.036589	0.99927	1.2416	0.31219	0.27576	0.70135	1.1014	0.13929	0.10925	0.47523	1.1764	0.23441	0.42399	0.99512	1.0891	0.12315	0.6734	0.99941	1.0376	0.053225
114.9845	12.28	olomics Ne	0.33521	0.99918	1.0627	0.087775	0.033326	0.44592	0.89863	-0.1542	0.093243	0.99995	1.0506	0.071186	0.86584	0.99985	1.0076	0.010905	0.83463	0.99927	1.0104	0.014946	0.20902	0.65564	1.1113	0.15221	0.017539	0.29108	1.0822	0.11402	0.95308	0.99512	0.99821	-0.00258	0.58208	0.99941	1.0289	0.041106
121.1023	6	oolomics Po	0.33531	0.99951	1.081	0.11243	0.34451	0.99998	0.91026	-0.13566	0.86913	0.99941	0.9846	-0.0224	0.05013	0.29486	0.73671	-0.44084	3.45E-06	0.000231	0.25477	-1.9728	0.002473	0.046224	0.55568	-0.84766	0.022103	0.31703	0.70812	-0.49793	0.13922	0.92991	0.8348	-0.26049	0.99873	0.99922	0.99977	-0.00033
97.1025	6.39	oolomics Po	0.33557	0.99951	1.076	0.10572	0.12595	0.99998	0.85305	-0.22929	0.48532	0.99941	0.94005	-0.08919	0.069748	0.3437	0.75312	-0.40906	3.7E-06	0.000231	0.27196	-1.8785	0.00177	0.045425	0.53922	-0.89106	0.012052	0.26736	0.66437	-0.58994	0.049305	0.67731	0.7714	-0.37446	0.56999	0.79176	0.94598	-0.08011
1041.57	5.79	domics Pos	0.33608	0.99987	1.1	0.13756	0.54478	0.99994	0.93839	-0.09174	0.67208	0.99996	0.95024	-0.07363	0.26186	0.72093	0.87111	-0.19907	0.032794	0.075922	0.72457	-0.46479	0.0136	0.098585	0.7112	-0.49167	0.18055	0.64793	0.82973	-0.26928	0.025551	0.56113	0.75437	-0.40665	0.060934	0.15795	1.273	0.34825
712.0112	9.5	oolomics Po	0.33628	0.99951	1.2555	0.32832	0.05603	0.99998	0.78398	-0.35111	0.45849	0.99941	1.0579	0.081165	0.001886	0.14854	0.6404	-0.64295	0.001275	0.011249	0.54945	-0.86394	0.008483	0.075866	0.62016	-0.68928	0.7687	0.99391	0.99071	-0.01346	0.90162	0.99956	1.0723	0.10065	0.11359	0.36506	1.6235	0.69911
998.5381	10.19	oolomics Po	0.33641	0.99951	1.2175	0.28389	0.47116	0.99998	1.2413	0.31184	0.79035	0.99941	1.0643	0.089969	0.0096	0.19702	1.331	0.41248	0.037487	0.13526	1.2697	0.34447	0.44016	0.74543	1.0683	0.095368	0.31687	0.93343	1.1383	0.18688	0.4899	0.99956	1.1358	0.18377	0.012177	0.2688	1.4316	0.51761
360.9788	12.02	olomics Ne	0.33645	0.99918	1.071	0.098892	0.92163	0.9934	1.0189	0.027015	0.25121	0.99995	1.1045	0.14345	0.31099	0.99985	1.1058	0.14512	0.017383	0.88267	1.2081	0.27277	0.098883	0.55533	1.1831	0.24262	0.27071	0.65528	1.1097	0.15018	0.11858	0.99512	1.1062	0.14561	0.29732	0.99941	1.0738	0.10279
982.1494	8.88	oolomics Po	0.33674	0.99951	1.32	0.40051	0.28581	0.99998	0.8736	-0.19495	0.16352	0.99941	1.1571	0.21051	0.050742	0.29486	0.74395	-0.42672	0.021218	0.08829	0.64117	-0.64122	0.11109	0.31996	0.84708	-0.23943	0.42308	0.99125	1.1935	0.25521	0.49141	0.99956	1.1698	0.22632	0.11752	0.36944	1.7828	0.83416
82.9461	0.57	domics Pos	0.33681	0.99987	1.0785	0.10907	0.68643	0.99994	1.0279	0.039675	0.53674	0.99996	1.0417	0.05891	0.35863	0.81307	0.93691	-0.09401	0.018906	0.049992	0.82134	-0.28394	0.092327	0.32062	0.89797	-0.15526	0.66774	0.89786	1.027	0.038389	0.51662	0.96776	1.0454	0.064054	0.024212	0.11096	1.2296	0.29819
786.6007	5.4	domics Pos	0.3373	0.99987	1.0651	0.09103	0.4653	0.99994	0.9524	-0.07037	0.5247	0.99996	0.95354	-0.06864	0.32148	0.7853	0.91162	-0.1335	0.009796	0.030269	0.77265	-0.37212	0.4152	0.65507	0.94578	-0.08043	0.28897	0.71484	1.119	0.16225	0.41619	0.96776	1.0674	0.094074	0.005255	0.088007	1.3325	0.41409
894.0979	8.94	oolomics Po	0.33749	0.99951	1.3343	0.41605	0.51483	0.99998	0.91239	-0.13227	0.42041	0.99941	1.1318	0.17859	0.096773	0.41587	0.75099	-0.41314	0.041974	0.14691	0.66614	-0.58611	0.21511	0.49604	0.8296	-0.26951	0.53616	0.99391	1.1982	0.26089	0.74624	0.99956	1.0965	0.13286	0.11456	0.36506	1.7678	0.82197
886.6802	7.32	domics Pos	0.33756	0.99987	1.2181	0.28465	0.55724	0.99994	1.1943	0.25618	0.45127	0.99996	1.2381	0.30808	0.604	0.93327	0.87396	-0.19435	0.10016	0.17454	0.63554	-0.65394	0.14136	0.41327	0.62044	-0.68863	0.033499	0.36346	0.53394	-0.90524	0.10098	0.85376	0.60317	-0.72935	0.34254	0.45257	0.77399	-0.36961
240.9165	12.12	oolomics Po	0.33798	0.99951	1.1125	0.15382	0.34485	0.99998	1.2096	0.27455	0.5654	0.99941	1.051	0.071758	0.18718	0.61868	1.1375	0.18591	0.23981	0.56669	1.1346	0.18217	0.045162	0.17328	1.2324	0.30151	0.096662	0.60726	1.1833	0.2428	0.28615	0.99956	1.142	0.19157	0.33217	0.60905	1.1177	0.16052
644.973	12.01	olomics Ne	0.3394	0.99918	1.1647	0.22001	0.36261	0.76696	0.90967	-0.13659	0.4589	0.99995	0.93195	-0.10167	0.29833	0.99985	0.89604	-0.15837	0.024246	0.93272	1.2649	0.33902	0.95138	0.98491	1.0488	0.068733	0.40377	0.74856	1.0645	0.090199	0.74964	0.99512	0.9699	-0.0441	0.3342	0.99941	0.90227	-0.14837
644.1944	1.29	domics Pos	0.33992	0.99987	0.91335	-0.13075	0.29284	0.99994	0.85517	-0.22571	0.22306	0.99996	0.87281	-0.19626	0.3036	0.76109	0.90035	-0.15144	0.024987	0.061727	0.75717	-0.4013	0.59415	0.77886	0.94797	-0.07709	0.5892	0.86163	0.93667	-0.09439	0.24791	0.94365	0.89441	-0.161	0.15072	0.25605	1.1677	0.22367
514.8745	10.1	domics Pos	0.34032	0.99987	1.0632	0.088439	0.56904	0.99994	1.0445	0.062873	0.7483	0.99996	1.0242	0.034448	0.17206	0.60551	0.87846	-0.18695	0.012105	0.035449	0.77804	-0.36209	0.26877	0.55659	0.88197	-0.18119	0.64845	0.89004	0.96789	-0.04709	0.89956	0.98023	0.99532	-0.00677	0.001373	0.07528	1.3921	0.4773
623.3168	2.99	domics Pos	0.34081	0.99987	0.80855	-0.30658	0.44141	0.99994	0.85338	-0.22874	0.3467	0.99996	0.81524	-0.2947	0.24556	0.70676	0.79486	-0.33123	0.017659	0.047376	0.44635	-1.1637	0.10944	0.35386	0.71574	-0.48249	0.79379	0.95091	0.94247	-0.08548	0.66266	0.97988	1.0758	0.10534	0.11087	0.21459	1.3387	0.42079
1054.778	9.78	oolomics Po	0.34152	0.99951	1.2933	0.37106	0.50833	0.99998	0.87842	-0.18701	0.75386	0.99941	0.93326	-0.09965	0.026973	0.24036	0.61355	-0.70475	0.038795	0.13882	0.6453	-0.63195	0.022982	0.11224	0.58736	-0.76768	0.41341	0.98208	0.84089	-0.25001	0.67479	0.99956	0.96693	-0.04852	0.043971	0.28476	2.0733	1.0519
1145.768	10.02	domics Pos	0.34181	0.99987	0.87144	-0.19852	0.65302	0.99994	0.95247	-0.07026	0.44463	0.99996	0.89089	-0.16668	0.66589	0.95717	0.95122	-0.07216	0.023087	0.058145	0.67098	-0.57566	0.18464	0.47124	0.83694	-0.2568	0.81942	0.96018	0.97273	-0.03989	0.26897	0.94365	0.8539	-0.22785	0.19409	0.30232	1.2139	0.27964
96.9268	10.13	domics Pos	0.342	0.99987	0.89324	-0.16287	0.28322	0.99994	0.85641	-0.22363	0.68985	0.99996	0.94798	-0.07708	0.49896	0.89009	0.91384	-0.12999	0.02673	0.065085	0.73879	-0.43677	0.29661	0.57939	0.84761	-0.23853	0.86641	0.97507	1.0203	0.028926	0.39082	0.96776	0.89109	-0.16635	0.095924	0.19739	1.2099	0.27491
378.2148	4.76	olomics Ne	0.34203	0.99918	1.0863	0.11945	0.98248	0.99664	1.0011	0.00155	0.7481	0.99995	1.0558	0.078336	0.87131	0.99985	1.013	0.018691	5.61E-06	0.006911	0.3822	-1.3876	0.34189	0.75622	0.92913	-0.10605	0.71137	0.91124	1.0715	0.099648	0.58441	0.99512	1.0306	0.043428	0.25159	0.99941	1.0655	0.091551
1009.804	10.02	domics Pos	0.34337	0.99987	0.92412	-0.11385	0.12465	0.99994	0.8798	-0.18475	0.095958	0.99996	0.8561	-0.22415	0.050251	0.38141	0.84431	-0.24415	0.003301	0.013169	0.70265	-0.50912	0.18691	0.47264	0.81715	-0.29132	0.074304	0.48057	0.85425	-0.22728	0.18049	0.92305	0.90067	-0.15092	0.021225	0.10765	1.1906	0.25165
618.2567	1.1	domics Pos	0.34392	0.99987	1.2329	0.3021	0.62631	0.99994	0.90619	-0.14212	0.4592	0.99996	1.1111	0.15204	0.50363	0.89133	1.1113	0.15227	0.75102	0.78823	1.0781	0.10849	0.024263	0.13726	1.5324	0.61579	0.007721	0.32129	1.8673	0.90095	0.14684	0.91175	1.3652	0.44913	0.018881	0.10449	1.6027	0.6805
282.9051	10.23	domics Pos	0.34402	0.99987	1.0297	0.04219	0.036542	0.99994	0.88044	-0.1837	0.99488	0.99996	1.0047	0.006765	0.028729	0.29796	0.89919	-0.15329	0.000446	0.003005	0.78181	-0.35511	0.036752	0.1743	0.82498	-0.27757	0.81863	0.96018	1.0236	0.033702	0.011061	0.52955	0.85104	-0.2327	0.008043	0.092077	1.2203	0.28722
895.5656	5.77	domics Pos	0.3441	0.99987	1.0539	0.075792	0.1996	0.99994	0.90304	-0.14714	0.49477	0.99996	0.93445	-0.09781	0.61959	0.93987	0.9537	-0.06839	0.015739	0.043394	0.71261	-0.48882	0.01842	0.11629	0.80179	-0.31871	0.040003	0.37662	0.85992	-0.21772	0.026228	0.56184	0.84287	-0.24662	0.02113	0.10765	1.2987	0.37708
110.0094	10.16	domics Pos	0.34453	0.99987	1.0549	0.077132	0.12224	0.99994	0.91452	-0.12892	0.48741	0.99996	0.94747	-0.07785	0.47034	0.88476	0.95393	-0.06804	0.002623	0.011052	0.78706	-0.34545	0.35146	0.61843	0.92342	-0.11493	0.29371	0.7186	1.0699	0.097466	0.88038	0.98023	0.99064	-0.01356	0.05707	0.15433	1.2106	0.27573
355.3736	4.32	domics Pos	0.34457	0.99987	0.93716	-0.09363	0.027267	0.99994	0.86532	-0.2087	0.19487	0.99996	0.85757	-0.22168	0.18016	0.6183	0.85026	-0.23402	0.058644	0.11707	0.85146	-0.23199	0.01072	0.089443	0.75612	-0.4033	0.25455	0.69047	0.85379	-0.22805	0.019264	0.52955	0.75219	-0.41083	0.59851	0.69071	0.95549	-0.06568
1067.766	10.02	domics Pos	0.34477	0.99987	0.91432	-0.12923	0.20794	0.99994	0.86637	-0.20695	0.12288	0.99996	0.84481	-0.24331	0.16546	0.59723	0.86554	-0.20833	0.015561	0.042996	0.72076	-0.47241	0.24393	0.53144	0.87455	-0.19339	0.17857	0.6466	0.87074	-0.19969	0.22986	0.94286	0.89276	-0.16365	0.2129	0.32175	1.1393	0.18821
778.5725	5.61	domics Pos	0.345	0.99987	1.0757	0.10529	0.77244	0.99994	0.97326	-0.0391	0.78207	0.99996	0.9744	-0.03741	0.34756	0.80096	0.91253	-0.13205	0.028722	0.068534	0.79375	-0.33324	0.27649	0.56257	0.91379	-0.13006	0.87868	0.97532	1.0275	0.039123	0.52254	0.96776	0.9499	-0.07415	0.98525	0.98836	0.99413	-0.0085
1132.839	9.63	oolomics Po	0.3456	0.99951	1.3297	0.41106	0.35121	0.99998	0.84615	-0.24102	0.53853	0.99941	1.109	0.14924	0.11269	0.45508	0.69738	-0.51999	0.032015	0.12136	0.63332	-0.659	0.040863	0.16273	0.63185	-0.66235	0.96857	0.9974	1.02	0.028499	0.78166	0.99956	1.0704	0.098206	0.17366	0.43569	1.7444	0.80276
1095.689	1.87	domics Pos	0.34563	0.99987	1.1781	0.23645	0.40371	0.99994	0.89498	-0.16007	0.009574	0.99996	1.2259	0.29383	0.035107	0.32905	0.76273	-0.39075	0.17608	0.26781	0.81718	-0.29128	0.47887	0.69988	0.92093	-0.11884	0.20175	0.64891	1.399	0.48442	0.56546	0.9703	1.0744	0.10352	0.016493	0.099614	1.8501	0.88757
1006.749	9.77	oolomics Po	0.3459	0.99951	1.3207	0.40125	0.64659	0.99998	0.94543	-0.08095	0.56993	0.99941	1.0388	0.054856	0.099776	0.42244	0.73079	-0.45248	0.007498	0.038718	0.66971	-0.57839	0.068633	0.23147	0.68918	-0.53705	0.74453	0.99391	0.9643	-0.05244	0.88215	0.99956	1.1311	0.17777	0.090269	0.34259	1.8812	0.91162
161.9255	12.72	olomics Ne	0.34641	0.99918	1.0445	0.062779	0.11272	0.52959	0.88936	-0.16916	0.69852	0.99995	1.0163	0.02336	0.79831	0.99985	0.98396	-0.02333	0.8422	0.99927	1.019	0.027208	0.01624	0.40708	1.1642	0.21932	0.32683	0.69919	1.053	0.074553	0.44466	0.99512	1.0574	0.080454	0.91316	0.99941	0.99331	-0.00969
1104.547	1.29	domics Pos	0.34722	0.99987	1.1903	0.25138	0.51113	0.99994	1.0956	0.13169	0.88559	0.99996	0.96007	-0.05879	0.63333	0.94754	1.0699	0.097466	0.38748	0.47845	0.78301	-0.3529	0.77542	0.88442	1.0425	0.060072	0.025365	0.33935	1.5903	0.66933	0.13679	0.89649	1.2688	0.34341	0.013834	0.098791	1.8129	0.85829
858.7007	0.56	domics Pos	0.34735	0.99987	1.1	0.13756	0.016387	0.99994	1.2127	0.27818	0.71957	0.99996	0.97281	-0.03977	0.024631	0.27787	0.75465	-0.40612	0.13685	0.22045	0.81157	-0.30122	0.28059	0.56697	0.91513	-0.12795	0.23424	0.67693	0.82697	-0.27409	0.39729	0.96776	0.94335	-0.08414	0.037881	0.13096	1.1885	0.24917
368.3404	4.18	domics Pos	0.34751	0.99987	1.0909	0.12551	0.44986	0.99994	1.0705	0.098266	0.97003	0.99996	1.0216	0.030818	0.60819	0.93547	1.0531	0.074621	0.94794	0.95546	1.0023	0.003369	0.001803	0.056351	1.2116	0.27686	0.00283	0.26175	1.4668	0.55262	0.394	0.96776	1.1573	0.21079	6.69E-06	0.025514	1.4487	0.53475
645.3697	3.1	oolomics Po	0.34754	0.99951	1.3771	0.46159	0.059227	0.99998	0.77747	-0.36315	0.64636	0.99941	0.9437	-0.0836	0.047327	0.28918	0.63653	-0.6517	0.001854	0.014707	0.5562	-0.84632	0.006673	0.067807	0.65479	-0.6109	0.32329	0.93772	0.83812	-0.25478	0.64555	0.99956	0.94518	-0.08133	0.092995	0.34667	1.6849	0.75269
728.5563	4.67	domics Pos	0.3478	0.99987	0.94271	-0.08512	0.17012	0.99994	0.91114	-0.13425	0.18275	0.99996	0.91308	-0.13119	0.085191	0.4892	0.8634	-0.21189	0.00454	0.016938	0.74581	-0.42312	0.95094	0.9734	1.07	0.097618	0.48314	0.8155	1.4388	0.52491	0.29413	0.9483	1.4699	0.55573	0.064783	0.16315	1.8057	0.85254
313.1528	2.46	oolomics Po	0.34808	0.99951	1.4239	0.50982	0.2804	0.99998	0.69458	-0.52578	0.80941	0.99941	0.94516	-0.08137	0.096344	0.41572	0.52489	-0.92991	0.006176	0.033951	0.23232	-2.1058	0.020166	0.10552	0.36894	-1.4385	0.033535	0.36264	0.50274	-0.99212	0.40763	0.99956	0.77652	-0.36491	0.63801	0.83498	1.2794	0.35547
629.5496	6.1	domics Pos	0.34864	0.99987	1.1078	0.14769	0.78051	0.99994	1.0404	0.05714	0.47647	0.99996	1.1027	0.14109	0.36805	0.82243	0.90281	-0.14751	0.14879	0.23568	0.80614	-0.3109	0.009771	0.087714	0.71224	-0.48957	0.12074	0.57768	0.77556	-0.36668	0.91446	0.98248	0.98369	-0.02372	0.030742	0.12047	1.4626	0.54855
1140.228	9.01	oolomics Po	0.34883	0.99951	1.4091	0.49481	0.29569	0.99998	0.83815	-0.25471	0.95634	0.99941	1.0264	0.037532	0.02759	0.24036	0.76724	-0.38225	0.069389	0.21373	0.65265	-0.61561	0.50066	0.79489	0.93599	-0.09543	0.4857	0.99391	1.2674	0.3419	0.53545	0.99956	1.1486	0.1999	0.043549	0.28476	1.7374	0.79693
1115.736	9.02	oolomics Po	0.34883	0.99951	1.3058	0.38493	0.38412	0.99998	0.86009	-0.21744	0.98125	0.99941	1.0154	0.022021	0.024867	0.23087	0.67617	-0.56454	0.068042	0.21034	0.65141	-0.61837	0.14704	0.38942	0.79697	-0.32739	0.59409	0.99391	1.204	0.26782	0.70315	0.99956	1.0931	0.12837	0.023784	0.2688	1.7263	0.78764
205.1957	1.66	domics Pos	0.3491	0.99987	1.1483	0.19944	0.5674	0.99994	0.88417	-0.17761	0.89735	0.99996	1.0044	0.006275	0.29854	0.75637	0.76632	-0.38398	0.000205	0.001726	0.26355	-1.9239	0.018422	0.11629	0.53234	-0.90959	0.47343	0.80806	0.85887	-0.21949	0.31644	0.9524	0.82349	-0.28018	0.11562	0.22031	1.4316	0.51765
534.2949	1.29	domics Pos	0.34951	0.99987	1.1105	0.15119	0.90809	0.99994	1.0126	0.018044	0.78212	0.99996	1.0379	0.053695	0.90861	0.9874	0.97235	-0.04046	0.21185	0.30583	0.76169	-0.39273	0.24527	0.53144	1.1494	0.20091	0.031929	0.36126	1.4006	0.48609	0.16498	0.91806	1.2052	0.26923	0.011458	0.095809	1.5968	0.67516
1141.826	9.7	oolomics Po	0.34955	0.99951	1.424	0.50991	0.37305	0.99998	0.86951	-0.20172	0.93099	0.99941	0.99172	-0.012	0.017811	0.20936	0.62673	-0.67409	0.003864	0.024408	0.56642	-0.82004	0.013461	0.086062	0.66863	-0.58072	0.56593	0.99391	0.92428	-0.11359	0.99538	0.99956	1.1427	0.19251	0.083317	0.33309	1.7607	0.81614
761.8259	10.05	domics Pos	0.34987	0.99987	1.0422	0.059631	0.97072	0.99994	1.0073	0.010553	0.90135	0.99996	0.99613	-0.00559	0.57228	0.9155	1.0284	0.040394	0.0003	0.00222	0.78074	-0.35709	0.811	0.90388	0.99075	-0.01341	0.5205	0.83957	1.0352	0.049891	0.66481	0.97988	1.0274	0.039042	0.034576	0.1264	1.2537	0.32622
1154.755	10.03	domics Pos	0.35	0.99987	1.076	0.10567	0.34112	0.99994	0.91163	-0.13348	0.26951	0.99996	0.89516	-0.15978	0.059135	0.41916	0.83749	-0.25586	0.022241	0.056462	0.79473	-0.33146	0.2237	0.5123	0.87091	-0.19941	0.79853	0.95226	0.97628	-0.03464	0.30965	0.9524	0.92429	-0.11358	0.051727	0.14844	1.2136	0.27935
838.6282	5.64	domics Pos	0.35018	0.99987	1.0671	0.093755	0.8013	0.99994	0.98289	-0.0249	0.95849	0.99996	0.99617	-0.00553	0.24671	0.70693	0.92694	-0.10945	0.008391	0.026864	0.74168	-0.43114	0.012106	0.093	0.8299	-0.26898	0.038648	0.37121	0.85429	-0.22721	0.051987	0.68583	0.85384	-0.22797	0.051223	0.14808	1.2499	0.32176

1096.208	4.43	domics Pos	0.35024	0.99987	1.3337	0.4154	0.52542	0.99994	0.8633	-0.21207	0.54727	0.99996	1.0996	0.13704	0.082199	0.48443	0.61208	-0.70822	0.00586	0.020613	0.47266	-1.0811	0.038046	0.17866	0.59707	-0.74402	0.66982	0.89896	0.90471	-0.14447	0.76413	0.97988	0.94353	-0.08385	0.12738	0.23222	1.7113	0.7751
463.3303	8.38	olomics Pc	0.35037	0.99951	0.92689	-0.10952	0.25173	0.99998	0.91051	-0.13526	0.41229	0.99941	0.92369	-0.11451	0.38151	0.87913	0.92242	-0.1165	0.79878	0.99688	0.97995	-0.02923	0.62188	0.87981	0.95124	-0.07212	0.4927	0.99391	0.94304	-0.08461	0.037887	0.62159	0.79687	-0.32759	0.017745	0.2688	0.79149	-0.33736
385.1798	6.33	olomics Pc	0.35039	0.99951	0.85086	-0.233	0.14712	0.99998	0.76185	-0.39242	0.016371	0.99941	0.63392	-0.65763	0.005945	0.1948	0.45694	-1.1299	0.001631	0.013356	0.41035	-1.2851	0.000521	0.039708	0.33666	-1.5707	0.013981	0.28064	0.42652	-1.2293	0.009628	0.47452	0.39126	-1.3538	0.003222	0.2688	0.42071	-1.2491
463.7689	12.74	olomics Ne	0.35102	0.99918	1.0735	0.10228	0.69217	0.94213	0.9755	-0.03578	0.25235	0.99995	1.0532	0.074797	0.35976	0.99985	1.0905	0.12505	0.076155	0.99927	1.0928	0.12798	0.003291	0.36417	1.2742	0.34955	0.49168	0.80993	1.0521	0.073258	0.92924	0.99512	1.0132	0.018936	0.63992	0.99941	1.0213	0.030373
409.1654	0.69	domics Pos	0.35103	0.99987	1.1771	0.23522	0.93339	0.99994	0.98282	-0.025	0.85607	0.99996	0.97181	-0.04125	0.431	0.86253	0.91052	-0.13525	0.006841	0.022971	0.66359	-0.59163	0.30627	0.58599	0.87465	-0.19323	0.69421	0.9106	1.0294	0.04184	0.65182	0.97988	1.0669	0.093426	0.13208	0.23722	1.3829	0.46774
985.1303	8.88	olomics Pc	0.35103	0.99951	1.2251	0.29286	0.48504	0.99998	0.92307	-0.1155	0.85199	0.99941	1.0238	0.033891	0.001387	0.14098	0.75646	-0.40266	0.063936	0.20093	0.68305	-0.54993	0.080675	0.25728	0.81427	-0.29641	0.84384	0.9951	1.043	0.060693	0.97588	0.99956	1.0255	0.036266	0.11396	0.36506	1.7625	0.81765
629.3157	0.58	domics Pos	0.35116	0.99987	1.5961	0.67454	0.18912	0.99994	0.83421	-0.26152	0.51841	0.99996	0.9318	-0.10191	0.047154	0.3738	0.63824	-0.64783	0.001488	0.007308	0.38684	-1.3702	0.001502	0.050677	0.626	-0.67577	0.95364	0.98985	1.0427	0.060334	0.84928	0.97988	1.2215	0.28863	0.013744	0.098791	2.4542	1.2953
941.3224	11.96	olomics Pc	0.35186	0.99951	0.93776	-0.0927	0.047972	0.99998	1.1718	0.22878	0.41804	0.99941	0.93605	-0.09535	0.89533	0.99905	1.0236	0.033643	0.1054	0.29884	1.1716	0.22848	0.073933	0.24382	1.1019	0.13994	0.68829	0.99391	0.96544	-0.05074	0.16587	0.95497	0.88921	-0.1694	0.86643	0.9482	0.99166	-0.01209
702.8639	10.02	domics Pos	0.35194	0.99987	1.0255	0.036391	0.23181	0.99994	0.93866	-0.09132	0.19526	0.99996	0.94913	-0.07532	0.084914	0.48909	0.91774	-0.12384	0.000142	0.001326	0.76644	-0.38375	0.17669	0.46349	0.88965	-0.16869	0.99525	0.99769	1.0024	0.003464	0.41675	0.96776	0.96412	-0.05271	0.012723	0.098791	1.1975	0.26006
232.8979	0.56	domics Pos	0.35215	0.99987	1.1219	0.166	0.11195	0.99994	0.90332	-0.1467	0.35958	0.99996	0.94372	-0.08357	0.45856	0.87644	0.95098	-0.07252	0.000613	0.003839	0.7863	-0.34685	0.29722	0.57957	0.93426	-0.09811	0.68079	0.90385	0.97974	-0.02953	0.86864	0.98023	1.0074	0.010666	0.002608	0.082646	1.1876	0.24809
783.9717	1.29	domics Pos	0.35241	0.99987	1.2028	0.26642	0.77871	0.99994	1.0779	0.10819	0.80051	0.99996	1.0587	0.082258	0.959	0.9937	0.98228	-0.02579	0.4521	0.5347	0.80896	-0.30586	0.26985	0.55719	1.2494	0.32129	0.046079	0.39255	1.5613	0.64277	0.18431	0.92582	1.2695	0.34427	0.021606	0.1083	1.7955	0.84441
368.3891	8.55	olomics Pc	0.35245	0.99951	0.89193	-0.165	0.045446	0.99998	0.77139	-0.37447	0.052998	0.99941	0.78318	-0.35259	0.51179	0.98198	0.9187	-0.12233	0.37003	0.76655	1.0815	0.11301	0.01604	0.093702	0.66603	-0.58633	0.18791	0.76465	0.78015	-0.35817	0.06721	0.7481	0.7206	-0.47273	0.086946	0.33918	0.81296	-0.29874
897.0788	8.94	olomics Pc	0.35256	0.99951	1.2402	0.3106	0.44759	0.99998	0.9065	-0.14161	0.89689	0.99941	1.0279	0.039703	0.04089	0.27667	0.72851	-0.45697	0.055584	0.1822	0.67501	-0.56701	0.15671	0.40694	0.84229	-0.24761	0.69741	0.99391	1.2005	0.26358	0.90017	0.99956	1.0125	0.017883	0.2379	0.50476	1.6794	0.74791
1005.17	9.09	olomics Pc	0.35272	0.99951	1.2037	0.26749	0.36825	0.99998	0.84865	-0.23677	0.88933	0.99941	1.0083	0.011989	0.16256	0.57085	0.75238	-0.41047	0.008979	0.04454	0.59149	-0.75757	0.18688	0.45882	0.75935	-0.39716	0.79823	0.99391	0.96235	-0.05536	0.58786	0.99956	0.91821	-0.1231	0.70826	0.86777	1.1688	0.22501
278.2488	5.65	olomics Pc	0.35278	0.99951	1.1334	0.1806	0.34664	0.99998	0.90202	-0.14877	0.8647	0.99941	1.0169	0.024128	0.16405	0.57291	0.78371	-0.35162	3.05E-06	0.000231	0.20443	-2.2903	0.004176	0.054724	0.57489	-0.79864	0.086025	0.60175	0.7583	-0.39916	0.12306	0.8972	0.82854	-0.27135	0.12828	0.3787	1.1326	0.17963
1139.726	9.01	olomics Pc	0.35305	0.99951	1.4302	0.51624	0.50018	0.99998	0.91261	-0.13192	0.75667	0.99941	1.065	0.090825	0.021927	0.2227	0.69937	-0.51586	0.049188	0.16661	0.65304	-0.61476	0.33334	0.64457	0.85937	-0.21866	0.46899	0.99391	1.3224	0.40319	0.24364	0.99956	1.2544	0.32704	0.053685	0.2951	1.7448	0.80306
639.2676	1.29	domics Pos	0.35334	0.99987	0.87402	-0.19427	0.24471	0.99994	0.85286	-0.22962	0.17889	0.99996	0.81307	-0.29856	0.11641	0.53727	0.79518	-0.33065	0.001918	0.008788	0.56669	-0.81938	0.034914	0.16873	0.72042	-0.47308	0.035294	0.36525	0.70442	-0.50549	0.12854	0.88537	0.81049	-0.30314	0.97369	0.98127	0.99846	-0.00223
337.2036	6.94	olomics Ne	0.35341	0.99918	0.76589	-0.3848	0.17713	0.60009	0.67853	-0.55951	0.18292	0.99995	0.70016	-0.51425	0.071524	0.99985	0.58495	-0.77362	0.10944	0.99927	0.6394	-0.64522	0.051971	0.48752	0.54506	-0.8755	0.050513	0.39335	0.57288	-0.80368	0.040571	0.99512	0.51077	-0.96924	0.040581	0.99941	0.53165	-0.91146
1049.787	9.75	olomics Pc	0.35349	0.99951	1.385	0.46985	0.42457	0.99998	0.85726	-0.22219	0.54993	0.99941	1.0589	0.082613	0.053131	0.29934	0.67551	-0.56596	0.005561	0.031791	0.55461	-0.85046	0.025292	0.11873	0.61809	-0.6941	0.6835	0.99391	0.93654	-0.09459	0.69875	0.99956	1.0023	0.003364	0.10482	0.35957	1.9223	0.94281
521.8567	10.12	domics Pos	0.35383	0.99987	0.85888	-0.21948	0.4578	0.99994	0.86915	-0.20232	0.52294	0.99996	0.88476	-0.17665	0.12073	0.54142	0.75021	-0.41463	0.022703	0.057509	0.64021	-0.64337	0.1843	0.47109	0.75932	-0.39722	0.22852	0.67233	0.81161	-0.30114	0.30669	0.9524	0.81619	-0.29303	0.8179	0.86632	1.0185	0.026477
312.3265	3.64	domics Pos	0.35395	0.99987	0.89089	-0.16669	0.26158	0.99994	0.81838	-0.28915	0.23038	0.99996	0.83436	-0.26126	0.17981	0.61766	0.86234	-0.21367	0.032412	0.075228	0.76549	-0.38555	0.00839	0.084185	0.60863	-0.71637	0.20797	0.65208	0.77594	-0.36598	0.025301	0.56108	0.68561	-0.54454	0.11863	0.22318	0.81326	-0.29821
235.2391	11.77	olomics Pc	0.35444	0.99951	0.92476	-0.11284	0.19941	0.99998	0.89151	-0.16568	0.012253	0.99941	0.85757	-0.22167	0.19481	0.62875	0.92377	-0.1144	0.35139	0.73891	0.95155	-0.07164	0.48069	0.77813	0.94969	-0.07448	0.89747	0.9951	1.0272	0.038715	0.0748	0.77253	0.87859	-0.18674	0.078	0.32372	0.86244	-0.21351
672.9119	3.14	olomics Pc	0.35457	0.99951	1.3804	0.4651	0.23801	0.99998	0.83397	-0.26193	0.77244	0.99941	1.0203	0.028956	0.000731	0.12797	0.60512	-0.7247	0.000362	0.004495	0.54452	-0.87695	0.018665	0.10085	0.72348	-0.46698	0.65275	0.99391	0.9569	-0.06356	0.93195	0.99956	1.0434	0.061286	0.028939	0.2688	1.916	0.93809
218.921	0.56	domics Pos	0.35468	0.99987	1.0555	0.077872	0.68711	0.99994	0.98245	-0.02555	0.37009	0.99996	0.96024	-0.05853	0.011314	0.21625	0.86829	-0.20375	0.001732	0.008125	0.76145	-0.39318	0.016533	0.10925	0.85423	-0.2273	0.074423	0.48057	0.91998	-0.12032	0.23857	0.94365	0.94618	-0.07981	0.069956	0.17098	1.1455	0.19595
1072.212	9.05	olomics Pc	0.35486	0.99951	1.209	0.27383	0.27387	0.99998	0.86505	-0.20915	0.57552	0.99941	0.94414	-0.08293	0.016043	0.20428	0.73864	-0.43707	0.032375	0.12236	0.62198	-0.68505	0.050776	0.18973	0.77486	-0.368	0.86609	0.9951	1.1051	0.1442	0.85035	0.99956	1.0602	0.084319	0.095756	0.34969	1.4398	0.52585
368.2793	4.16	olomics Pc	0.35504	0.99951	0.6951	-0.52472	0.22017	0.99998	0.62217	-0.68462	0.42927	0.99941	0.73037	-0.45331	0.096604	0.41572	0.53115	-0.91282	0.077525	0.23504	0.40862	-1.2912	0.011132	0.083488	0.19765	-2.339	0.056011	0.47429	0.31714	-1.6568	0.038505	0.62521	0.31543	-1.6646	0.012157	0.2688	0.2102	-2.2501
69.0712	6.38	olomics Pc	0.35539	0.99951	1.1279	0.1737	0.14114	0.99998	0.83861	-0.25393	0.45348	0.99941	0.92598	-0.11095	0.16747	0.57919	0.77898	-0.36034	1.22E-05	0.000362	0.2848	-1.812	0.003862	0.053776	0.57601	-0.79584	0.040991	0.40225	0.68753	-0.54049	0.031817	0.58535	0.73606	-0.44211	0.50963	0.7583	0.92472	-0.11291
1116.33	8.4	olomics Pc	0.3555	0.99951	0.89387	-0.16187	0.52557	0.99998	0.94245	-0.08551	0.23007	0.99941	0.87707	-0.18924	0.018294	0.20936	1.1434	0.19339	0.012782	0.058607	1.1457	0.19627	0.23752	0.52754	0.92444	-0.11335	0.36226	0.96545	0.91353	-0.13048	0.39027	0.99956	0.88077	-0.18316	0.21561	0.48305	0.83448	-0.26106
466.8776	10.14	domics Pos	0.35621	0.99987	1.0898	0.12405	0.70182	0.99994	0.96336	-0.05386	0.65279	0.99996	0.95617	-0.06466	0.095241	0.51511	0.85555	-0.22508	0.019565	0.051342	0.73737	-0.43955	0.20854	0.50137	0.87158	-0.19829	0.82108	0.96018	1.0154	0.021997	0.481	0.96776	0.92808	-0.10768	0.11444	0.21881	1.1331	0.18029
814.6848	10.32	olomics Pc	0.35631	0.99951	0.93335	-0.09951	0.12074	0.99998	0.90451	-0.14479	0.10378	0.99941	0.89523	-0.15966	0.18234	0.60975	0.90472	-0.14446	0.7482	0.99688	0.98322	-0.02441	0.14396	0.38429	0.91485	-0.1284	0.10437	0.61787	0.9038	-0.14593	0.042416	0.64572	0.82305	-0.28096	0.014538	0.2688	0.84074	-0.25026
323.1706	2.41	olomics Pc	0.35635	0.99951	0.76619	-0.38423	0.70727	0.99998	0.95381	-0.06822	0.40511	0.99941	1.4082	0.49381	0.16473	0.5734	1.6628	0.73366	0.042148	0.14709	1.8814	0.9118	0.16817	0.42548	1.4528	0.53888	0.096179	0.60726	1.6409	0.71452	0.2129	0.99956	1.2757	0.35129	0.24201	0.50993	0.71041	-0.49328
1031.178	4.52	domics Pos	0.35723	0.99987	1.2707	0.34562	0.37965	0.99994	0.82317	-0.28074	0.82575	0.99996	0.94579	-0.08041	0.047911	0.37667	0.5992	-0.7389	0.006787	0.022921	0.45576	-1.1337	0.045861	0.20548	0.60794	-0.71799	0.58432	0.86163	0.8857	-0.17511	0.6663	0.97988	0.9399	-0.08943	0.12585	0.23026	1.8413	0.88073
1007.649	9.09	olomics Pc	0.35724	0.99951	1.2225	0.28979	0.46233	0.99998	0.85461	-0.22665	0.55251	0.99941	1.0939	0.12949	0.40883	0.90163	0.83512	-0.25995	0.042022	0.14691	0.64036	-0.64305	0.32736	0.63615	0.78717	-0.34526	0.92937	0.9951	0.97703	-0.03353	0.74448	0.99956	0.94684	-0.07881	0.70974	0.86856	1.1554	0.20844
1186.855	9.65	olomics Pc	0.35759	0.99951	1.4151	0.50093	0.25942	0.99998	0.84751	-0.2387	0.52497	0.99941	1.1138	0.15555	0.064512	0.33313	0.69372	-0.52757	0.001352	0.011665	0.55295	-0.85478	0.023099	0.11246	0.66888	-0.58018	0.61281	0.99391	0.93204	-0.10153	0.89199	0.99956	1.1357	0.18352	0.078477	0.32467	1.6617	0.73269
681.2752	5.22	olomics Ne	0.35777	0.99918	1.1059	0.14522	0.93772	0.9934	0.98951	-0.01521	0.48576	0.99995	1.0832	0.11528	0.34984	0.99985	1.1142	0.15596	0.78443	0.99927	1.0326	0.046232	0.11527	0.58117	1.2195	0.28629	0.016825	0.29108	1.3603	0.44393	0.043178	0.99512	1.2656	0.33985	0.35167	0.99941	1.1017	0.13977
385.2043	7.5	olomics Ne	0.35849	0.99918	0.88202	-0.18112	0.18961	0.60941	0.78318	-0.35258	0.03128	0.99995	0.75057	-0.41395	0.006652	0.99985	0.53259	-0.90891	0.009991	0.72349	0.53025	-0.91525	0.000565	0.13901	0.40528	-1.303	0.002482	0.22316	0.53211	-0.91021	0.001014	0.69914	0.48927	-1.0313	0.009818	0.99941	0.51475	-0.95805
318.9816	3.86	olomics Ne	0.35853	0.99918	0.9631	-0.05424	0.000696	0.34605	0.83396	-0.26196	0.2085	0.99995	0.94018	-0.08899	0.99168	0.99985	1.0027	0.003931	0.16929	0.99927	0.95222	-0.07064	0.96855	0.98999	1.0039	0.0056	0.86324	0.95689	1.0092	0.013246	0.16908	0.99512	0.94146	-0.08702	0.23484	0.99941	0.96359	-0.0535
1118.215	9.02	olomics Pc	0.35886	0.99951	1.4555	0.5415	0.72487	0.99998	0.99175	-0.01195	0.67903	0.99941	1.0963	0.13259	0.042841	0.28056	0.78141	-0.35584	0.10173	0.29001	0.66785	-0.58241	0.41511	0.72336	0.93961	-0.08987	0.5959	0.99391	1.2633	0.33721	0.26844	0.99956	1.1954	0.25743	0.059979	0.30404	1.7482	0.80584
359.9806	11.99	olomics Ne	0.35898	0.99918	1.2292	0.2977	0.85709	0.98874	1.039	0.055162	0.46856	0.99995	1.0538	0.075622	0.89069	0.99985	0.99268	-0.0106	0.044436	0.99927	1.2577	0.33074	0.33808	0.75413	1.2446	0.31573	0.66465	0.87948	1.0835	0.11569	0.97436	0.99512	1.0015	0.002094	0.44719	0.99941	1.0843	0.11682
1137.749	9.01	olomics Pc	0.35935	0.99951	1.3424	0.42486	0.14892	0.99998	0.81816	-0.28954	0.8134	0.99941	1.037	0.052397	0.001798	0.14525	0.6953	-0.52428	0.028479	0.11044	0.63691	-0.65083	0.021825	0.10951	0.77349	-0.37055	0.70714	0.99391	1.1851	0.24498	0.3517	0.99956	1.197	0.25941	0.049103	0.28911	1.6273	0.70248
1070.704	8.84	olomics Pc	0.35976	0.99951	1.3036	0.38253	0.56648	0.99998	0.93176	-0.10198	0.28979	0.99941	1.1015	0.13949	0.11265	0.45508	0.77258	-0.37225	0.039256	0.14004	0.75352	-0.40828	0.035247	0.14703	0.73648	-0.44129	0.88745	0.9951	1.0108	0.01547	0.73781	0.99956	1.1028	0.14123	0.031117	0.2688	1.8076	0.8541
873.5581	5.22	domics Pos	0.35981	0.99987	1.0802	0.11128	0.61129	0.99994	0.95737	-0.06285	0.65752	0.99996	0.95921	-0.06008	0.44306	0.86677	0.94141	-0.08711	0.000821	0.004815	0.70743	-0.49935	0.41497	0.65507	0.90305	-0.14713	0.75183	0.93661	0.97739	-0.033	0.97712	0.9929	1.0178	0.025499	0.71908	0.79089	1.0389	0.055024
759.6356	5.65	domics Pos	0.35984	0.99987	0.85457	-0.22674	0.063952	0.99994	0.66392	-0.59092	0.54468	0.99996	0.90207	-0.14868	0.38517	0.8321	0.8539	-0.22786	0.20207	0.2958	0.75374	-0.40786	0.35945	0.62208	0.80786	-0.30782	0.96127	0.99063	0.99448	-0.00799	0.029602	0.60685	0.66635	-0.58565	0.27349	0.38241	0.81618	-0.29304
931.7888	10.03	domics Pos	0.36037	0.99987	1.0744	0.10347	0.45013	0.99994	0.92997	-0.10474	0.40373	0.99996	0.91198	-0.13293	0.83549	0.98621	0.98263	-0.02528	0.015188	0.042119	0.78802	-0.34369	0.93132	0.96524	1.0074	0.010585	0.8425	0.9676	0.98456	-0.02245	0.50904	0.96776	1.0552	0.077562	0.014056	0.098791	1.265	0.33915
990.785	10.05	domics Pos	0.36041	0.99987	1.0528	0.074216	0.74743	0.99994	0.98166	-0.02671	0.88585	0.99996	0.9959	-0.00592	0.55895	0.91003	0.96705	-0.04834	0.00204	0.009185	0.87459	-0.19333	0.7506	0.87064	1.0567	0.079522	0.52126	0.83957	1.0628	0.087868	0.080823	0.80254	1.0759	0.1056	0.013707	0.098791	1.2212	0.28829
396.2221	2.28	domics Pos	0.36058	0.99987	0.919	-0.12186	0.16523	0.99994	0.78973	-0.34057	0.020098	0.99996	0.6932	-0.52866	0.001914	0.17581	0.56992	-0.81117	4.5E-05	0.00059	0.45407	-1.139	0.015439	0.10475	0.45749	-1.1282	0.021655	0.33809	0.53637	-0.8987	0.004628	0.52955	0.42903	-1.2208	0.032455	0.12301	0.56142	-0.83285
916.6111	8.93	olomics Pc	0.36091	0.99951	1.3266	0.4077	0.41051	0.99998	0.90783	-0.13951	0.45408	0.99941	1.0824	0.11427	0.046087	0.28747	0.74488	-0.42492	0.057073	0.18562	0.7225	-0.46893	0.078219	0.25219	0.83525	-0.25972	0.59544	0.99391	1.1465	0.1972	0.62262	0.99956	1.1171	0.15972	0.094315	0.34866	1.7546	0.81113
465.3134	5.59	olomics Pc	0.36105	0.99951	0.76662	-0.38341	0.54946	0.99998	0.84627	-0.24081	0.25116	0.99941	0.71365	-0.48672	0.37192	0.86654	0.76897	-0.37901	0.000959	0.009043	0.24072	-2.0546	0.031651	0.13695	0.5123	-0.96495	0.366	0.96767	0.76936	-0.37826	0.46827	0.99956	0.80608	-0.31101	0.80257	0.91913	0.92793	-0.10792
803.6887	6.23	domics Pos	0.36118	0.99987	0.8603	-0.21709	0.043377	0.99994	0.6872	-0.5412	0.22246	0.99996	0.81854	-0.28887	0.12467	0.54705	0.76325	-0.38978	0.093186	0.16553	0.74373	-0.42716	0.22769	0.51532	0.7323	-0.44948	0.90956	0.97939	1.0058	0.008335	0.2419	0.94365	0.76338	-0.38953	0.31126	0.42123	0.8191	-0.28789
688.8949	3.15	olomics Pc	0.36133	0.99951	1.2252	0.29299	0.10557	0.99998	0.79664	-0.32801	0.68274	0.99941	0.94138	-0.08716	0.016001	0.20428	0.57644	-0.79476	0.006079	0.03365	0.58793	-0.76628	0.00309	0.049681	0.58846	-0.76499	0.59284	0.99391	0.91877	-0.12222	0.15818	0.93927	0.78882	-0.34222	0.025718	0.2688	1.5663	0.64736
524.9743	12	olomics Ne	0.3616	0.99918	1.1054	0.14463	0.25444	0.67504	1.0764	0.1062	0.15545	0.99995	1.121	0.16476	0.71937	0.99985	1.0255	0.036362	0.19785	0.99927	1.0808	0.11208	0.48777	0.84928	1.0577	0.080869	0.012895	0.2716	1.2531	0.32549	0.18057	0.99512	1.134	0.1814	0.31279	0.99941	1.134	0.18138
766.9298	3.25	olomics Pc	0.36163	0.99951	1.2617	0.33536	0.01261	0.99998	0.78749	-0.34467	0.22866	0.99941	0.87844	-0.18698	0.007419	0.1948	0.64611	-0.63015	0.00049	0.005589	0.48243	-1.0516	0.000319	0.039399	0.63092	-0.66446	0.81207	0.99391	0.98912	-0.01578	0.11256	0.87368	0.82711	-0.27385	0.26563	0.53631	1.4003	0.48577
786.9739	12.01	olomics Ne	0.36168	0.99918	1.2145	0.28034	0.53243	0.86583	1.0549	0.077075	0.77308	0.99995	1.0491	0.069166	0.36876	0.99985	1.0979	0.13472	0.68932	0.99927	0.9622	-0.05559	0.86533	0.9762	1.0223	0.031749	0.012705	0.2716	1.3921	0.47725	0.72838	0.99512	1.068	0.094872	0.20641	0.99941	1.0974	0.13414
353.9079	0.56	domics Pos	0.36183	0.99987	1.0795	0.11039	0.25168	0.99994	0.86864	-0.20316	0.33942	0.99996	0.91107	-0.13436	0.83856	0.98655	1.0078	0.011207	0.000476	0.003154	0.64923	-0.6232	0.6586	0.82147	0.95936	-0.05985	0.48589	0.81703	0.93615	-0.09519	0.83703	0.97988	0.98502	-0.02178	0.043011	0.13814	1.3313	0.41289
1078.76	10.09	domics Pos	0.36207	0.99987	1.0653	0.091262	0.078382	0.99994	0.86043	-0.21688	0.77409	0.99996	0.97841	-0.03148	0.2944	0.75244	0.91367	-0.13026	0.004795	0.017665	0.7196	-0.47473	0.58015	0.77145	0.94476	-0.08198	0.8997	0.97738	1.0059	0.0085	0.37915	0.96776	0.92345	-0.1149	0.17795	0.28538	1.188	0.24852
516.2729	10.18	olomics Ne	0.36222	0.99918	1.0889	0.12292	0.55819	0.88137	0.93711	-0.09371	0.78217	0.99995	0.96654	-0.0491	0.78872	0.99985	0.96737	-0.04787	0.32278	0.99927	1.0953	0.1313	0.030201	0.47167	1.3419	0.42432	0.069832	0.41438	1.2768	0.35252	0.049014	0.99512	1.2201	0.287	0.064701	0.99941	1.2015	0.26487
181.989	10.19	domics Pos	0.36231	0.99987	1.0749	0.10424	0.2814	0.99994	0.89482	-0.16033	0.73789	0.99996	0.96374	-0.05329	0.66879	0.95717	0.95312	-0.06928	0.010573	0.032073	0.72054	-0.47285	0.71019	0.84907	0.95819	-0.06161	0.38918	0.7665	0.91189	-0.13307	0.78566	0.97988	1.0146	0.020861	0.075794	0.17781	1.1824	0.24177
963.1171	8.89	olomics Pc	0.36256	0.99951	1.2938	0.37166	0.6174	0.99998	0.94257	-0.08533	0.99386	0.99941	1.0117	0.016722	0.042411	0.2802	0.79013	-0.33985	0.015759	0.069042	0.64603	-0.63032	0.14049	0.37951	0.8595	-0.21843	0.8013	0.99391	1.1267	0.17214	0.60192	0.99956	1.1517	0.2037	0.089808	0.34256	1.8667	0.90048
342.1837	4.54	domics Pos	0.36259	0.99987	1.1209	0.16462	0.95972	0.99994	1.0195	0.027904	0.54404	0.99996	0.91815	-0.12319	0.88757	0.9874	1.0231	0.032902	0.042061	0.091541	0.66692	-0.58442	0.66902	0.82743	0.94612	-0.07991	0.80618	0.95502	0.96724	-0.04805	0.63142	0.97988	0.93957	-0.08993	0.43101	0.53724	0.90891	-0.13779
889.8008	10.05	domics Pos	0.36286	0.99987	1.0748	0.104	0.7294	0.99994	0.96057	-0.05804	0.5481	0.99996	1.057	0.079952	0.56107	0.91079	0.93001	-0.10468	0.006149	0.021284	0.69901	-0.51661	0.6022	0.78414	0.9345	-0.09774	0.79364	0.95091	1.059	0.082664	0.6501	0.97988	1.0482	0.067897	0.034363	0.12626	1.2823	0.35876
1137.246	9.01	olomics Pc	0.36288	0.99951	1.3209	0.40154	0.036534	0.99998	0.77026	-0.37659	0.98496	0.99941	1.0244	0.034741	0.047821	0.28918	0.7522	-0.41081	0.029007	0.11197	0.63259	-0.66066	0.12434	0.34785	0.8472	-0.23922	0.4812	0.99391	1.2159	0.28199	0.52633	0.99956	1.1987	0.26147	0.024175	0.2688	1.7642	0.81898
858.5896	5.23	domics Pos	0.36361	0.99987	1.0813	0.11279	0.43538	0.99994	0.95163	-0.07153	0.49494	0.99996	0.9522	-0.07067	0.72928	0.96907	0.9746	-0.03712	0.017877	0.047769	0.78189	-0.35497	0.19305	0.47937	0.84743	-0.23884	0.8139	0.95931	0.98378	-0.0236	0.54961	0.96776	0.95719	-0.06313	0.13204	0.23722	1.2069	0.27134
775.8587	2.4	olomics Pc	0.36361	0.99951	0.52521	-0.92903	0.95676	0.99998	1.2228	0.29015	0.41655	0.99941	1.5068	0.59147	0.27657	0.74909	2.9424	1.557	0.027218	0.1065	5.2285	2.3864	0.10047	0.30083	3.1271	1.6448	0.12253	0.65834	2.4457	1.2903	0.25052	0.99956	2.2212	1.1514	0.27125	0.54284	0.41136	-1.2815
367.3363	4.18	domics Pos	0.36368	0.99987	1.1064	0.14591	0.8778	0.99994	1.0144	0.02067	0.90639	0.99996	1.0141	0.02018	0.39417	0.83821	0.92313	-0.1154	0.4126	0.50119	0.91385	-0.12997	0.13096	0.39538	1.1748	0.23238	0.003768	0.30568	1.3715	0.45575	0.95768	0.98838	1.0291	0.041356	0.020312	0.10677	1.348	0.43086
1112.496	6.01	domics Pos	0.3641	0.99987	1.1556	0.20864	0.34212	0.99994	1.1677	0.22372	0.64031	0.99996	0.94417	-0.08288	0.83959	0.98707	0.98379	-0.02358	0.22968	0.32424	1.1358	0.18367	0.1548	0.4337	1.2349	0.30437	0.26852	0.70268	1.4276	0.51364	0.013172	0.52955	1.3082	0.38755	0.002036	0.07995	1.5718	0.65245
354.1956	6.83	olomics Pc	0.36436	0.99951	0.95503	-0.06638	0.84712	0.99998	0.99244	-0.01095	0.02737	0.99941	0.74192	-0.43066	0.15021	0.5457	0.69214	-0.53087	0.000889	0.00859	0.66624	-0.58589	0.00223	0.045856	0.49637	-1.0105	0.006353	0.21836	0.58411	-0.77569	0.010584	0.47452	0.56337	-0.82783	0.034892	0.272	0.57555	-0.797
1100.758	10.03	domics Pos	0.36526	0.99987	1.0337	0.047781	0.51991	0.99994	0.95683	-0.06367	0.36596	0.99996	0.94837	-0.07647	0.37107	0.82414	0.94384	-0.08338	0.004868	0.0179	0.78247	-0.35389	0.15984	0.44196	0.89354	-0.1624	0.81997	0.96018	0.98715	-0.01866	0.81202	0.97988	1.0074	0.010628	0.003153	0.085302	1.2733	0.34855
853.5701	4.79	domics Pos	0.36535	0.99987	1.3419	0.42423	0.12053	0.99994	0.77619	-0.36552	0.81071	0.99996	1.0419	0.059228	0.061827	0.4302	0.67872	-0.5591	0.009469	0.029511	0.50973	-0.9722	0.041932	0.1924	0.70289	-0.50863	0.83548	0.96659	0.99832	-0.00243	0.9158	0.98252	1.0583	0.08177	0.064575	0.16296	2.0176	1.0126
1097.579	6.06	domics Pos	0.36549	0.99987	1.0812	0.11258	0.13125	0.99994	0.80658	-0.3101	0.23023	0.99996	0.8788	-0.18639	0.1199	0.54112	0.83227	-0.26487	0.001974	0.008971	0.55124	-0.85926	0.12146	0.37845	0.77111	-0.37498	0.42896	0.79094	0.91204	-0.13282	0.059447	0.72218	0.79328	-0.33409	0.22361	0.33143	0.88574	-0.17504
1009.806	0.5	domics Pos	0.36621	0.99987	1.0664	0.092713	0.8951	0.99994	0.99069	-0.01349	0.6404	0.99996	0.96895	-0.04551	0.091229	0.50268	0.89213	-0.16467	0.004478	0.016741	0.77059	-0.37596	0.50149	0.71779	0.93025	-0.1043	0.376	0.76056	0.94578	-0.08042	0.77843	0.97988	0.98176	-0.02656	0.14197	0.24696	1.1793	0.23797
779.562	9.14	olomics Ne	0.36622	0.99918	1.1079	0.14789	0.9959	0.99771	1.0145	0.020804	0.85885	0.99995	1.0015	0.002105	0.46028	0.99985	1.0536	0.075271	0.73606	0.99927	1.058	0.081284	0.00765	0.36417	1.3908	0.47594	0.034223	0.3559	1.3738	0.45817	0.074392	0.99512	1.1926	0.25414	0.66613	0.99941	1.0836	0.11588
965.7866	10.03	domics Pos	0.36629	0.99987	0.94566	-0.08061	0.3134	0.99994	0.96005	-0.05882	0.51043	0.99996	0.9523	-0.07052	0.001498	0.17581	0.86966	-0.20148	5.33E-07	0.000144	0.75681	-0.402	0.22196	0.51169	0.87235	-0.19702	0.38988	0.76747	1.0596	0.083529	0.113	0.87929	0.90203	-0.14875	0.012365	0.098791	1.1743	0.23178
943.5904	10.48	olomics Ne	0.36675	0.99918	1.11	0.15056	0.008311	0.34605	0.78853	-0.34275	0.040665	0.99995	1.1545	0.20731	0.15721	0.99985	1.1109	0.15172	0.69534	0.99927	0.97662	-0.03413	0.008961	0.3677	1.2271	0.2952	0.1151	0.49199	1.1832	0.24271	0.11977	0.99512	1.118	0.16095	0.88347	0.99941	1.0324	0.046051
1004.664	8.87	olomics Pc	0.36677	0.99951	1.2632	0.33714	0.65924	0.99998	0.9561	-0.06477	0.35596	0.99941	1.1246	0.16944	0.016037	0.20428	0.74782	-0.41925	0.033263	0.12494	0.67198	-0.5735	0.011979	0.083709	0.73304	-0.44804	0.92807	0.9951	1.0701	0.097782	0.94292	0.99956	1.0679	0.094827	0.099369	0.35294	1.7856	0.83639
1154.751	5.57	domics Pos	0.36718	0.99987	0.91127	-0.13404	0.067877	0.99994	0.80845	-0.30676	0.17488	0.99996	0.84479	-0.24334	0.097561	0.51762	0.83028	-0.26833	0.009202	0.028926	0.71488	-0.48423	0.11221	0.35925	0.75702	-0.40159	0.12131	0.57891	0.83318	-0.26329	0.11641	0.88537	0.80783	-0.30788	0.19665	0.30491	0.85581	-0.22464
794.5676	4.79	domics Pos	0.36739	0.99987	1.0742	0.10321	0.99336	0.99994	1.0018	0.002598	0.96164	0.99996	1.0094	0.013518	0.25298	0.71031	0.89968	-0.15251	0.017749	0.047525	0.71086	-0.49235	0.018271	0.11592	0.82163	-0.28345	0.089366	0.51786	0.83421	-0.26152	0.03949	0.64308	0.79863	-0.3244	0.12529	0.22986	0.8404	-0.25085
520.7453	9.91	domics Pos	0.36756	0.99987	1.1238	0.16837	0.16738	0.99994	1.2001	0.26319	0.90623	0.99996	1.0084	0.012114	0.4013	0.84516	1.0798	0.11071	0.3012	0.39453	0.82747	-0.27323	0.12023	0.37733	0.8206	-0.28524	0.2305	0.67417	1.1428	0.19262	0.037454	0.64014	1.3122	0.39199	0.028028	0.11681	1.4052	0.49075
1173.737	0.53	domics Pos	0.36788	0.99987	0.91499	-0.12818	0.039553	0.99994	0.80583	-0.31146	0.10551	0.99996	0.84745	-0.2388	0.28937	0.75244	0.88575	-0.17503	0.00724	0.023965	0.695	-0.52491	0.11097	0.35635	0.83098	-0.26712	0.29263	0.7186	0.89968	-0.15251	0.56555	0.9703	0.9453	-0.08116	0.31093	0.42101	1.1079	0.14789
942.613	9.18	olomics Pc	0.36826	0.99951	1.2521	0.32432	0.31287	0.99998	0.82066	-0.28514	0.9595	0.99941	0.99156	-0.01223	0.33667	0.82883	0.80266	-0.31714	0.042995	0.14965	0.66727	-0.58366	0.1217	0.34168	0.69543	-0.52401	0.70961	0.99391	0.93739	-0.09328	0.55004	0.99956	0.88241	-0.18048	0.48864	0.7443	1.363	0.44676
770.851	0.5	domics Pos	0.36841	0.99987	1.0239	0.034081	0.33739	0.99994	0.95483	-0.06668	0.13358	0.99996	0.93286	-0.10026	0.032414	0.3137	0.89524	-0.15965	2.64E-05	0.000439	0.74617	-0.42243	0.23032	0.51812	0.88258	-0.18021	0.41344	0.78029	0.94943	-0.07487	0.25836	0.94365	0.94701	-0.07855	0.02311	0.11015	1.1749	0.23256
737.5247	8.39	olomics Ne	0.36868	0.99918	0.94284	-0.08491	0.009839	0.34605	0.84394	-0.24479	0.66836	0.99995	0.97594	-0.03514	0.34708	0.99985	0.94509	-0.08148	0.53411	0.99927	1.0326	0.046235	0.058333	0.50569	1.175	0.23267	0.21533	0.59066	1.0984	0.13537	0.50924	0.99512	1.041	0.058001	0.43087	0.99941	0.95339	-0.06886
925.807	10.05	domics Pos	0.3694	0.99987	1.0773	0.10737	0.27146	0.99994	0.8758	-0.19133	0.11794	0.99996	0.87078	-0.19961	0.25069	0.70866	0.87578	-0.19136	0.008068	0.026137	0.70166	-0.51116	0.62784	0.8019	0.95239	-0.07037	0.55533	0.85279	0.92552	-0.11166	0.61832	0.97751	0.95358	-0.06857	0.79857	0.8503	0.97915	-0.0304
748.528	5.12	domics Pos	0.36949	0.99987	1.0486	0.068486	0.57899	0.99994	0.97318	-0.03923	0.52229	0.99996	0.97209	-0.04083	0.025037	0.27914	0.90681	-0.14112	0.000119	0.00116	0.77364	-0.37027	0.30596	0.58599	0.9202	-0.11999	0.89031	0.97532	1.0329	0.046708	0.72887	0.97988	0.9855	-0.02107	0.012863	0.098791	1.0928	0.12804
946.5725	5.73	domics Pos	0.36957	0.99987	1.0618	0.086513	0.59369	0.99994	0.95408	-0.06782	0.047507	0.99996	0.86461	-0.20987	0.48297	0.88725	0.94037	-0.0887	0.006885	0.023049	0.7637	-0.38893	0.13367	0.40042	0.86071	-0.2164	0.27288	0.70555	0.90077	-0.15077	0.29136	0.94825	0.93985	-0.0895	0.069405	0.17088	1.3745	0.4589
84.9604	10.23	domics Pos	0.36984	0.99987	0.95169	-0.07144	0.41227	0.99994	0.97807	-0.03199	0.21824	0.99996	0.93576	-0.09579	0.081133	0.4813	0.88903	-0.1697	8.31E-05	0.000908	0.7134	-0.48723	0.28202	0.56896	0.90055	-0.15112	0.91081	0.97939	1.0136	0.01949	0.12897	0.88537	0.90328	-0.14676	0.018577	0.10388	1.1943	0.25615
1069.233	9.05	olomics Pc	0.37022	0.99951	1.1425	0.19216	0.26139	0.99998	0.81545	-0.29433	0.64979	0.99941	0.9498	-0.0743	0.013216	0.20085	0.64824	-0.62541	0.02445	0.098614	0.61285	-0.70639	0.061044	0.21294	0.73239	-0.44931	0.93237	0.9955	1.0517	0.072697	0.97824	0.99956	1.0082	0.011824	0.11234	0.36506	1.343	0.42544
617.2597	1.11	domics Pos	0.37032	0.99987	1.1617	0.21621	0.96768	0.99994	1.0135	0.019311	0.34439	0.99996	1.3173	0.39758	0.56191	0.91079	1.1	0.13754	0.67065	0.72062	1.0856	0.11847	0.026396	0.14337	1.6022	0.68005	0.006927	0.31228	1.8138	0.85904	0.044196	0.65325	1.4846	0.57009	0.020197	0.10667	1.7428	0.80145
437.2362	0.59	domics Pos	0.37036	0.99987	1.2392	0.30944	0.046397	0.99994	0.74146	-0.43156	0.64536	0.99996	0.94659	-0.0792	0.019105	0.25595	0.61468	-0.70208	0.000179	0.001557	0.38669	-1.3707	0.023288	0.13414	0.63185	-0.66235	0.30936	0.72874	0.8365	-0.25757	0.69039	0.97988	0.98135	-0.02716	0.041758	0.13621	2.069	1.0489
680.864	10.12	domics Pos	0.3705	0.99987	1.0839	0.11617	0.5199	0.99994	0.95801	-0.06188	0.12842	0.99996	1.1396	0.18849	0.74031	0.97237	1.016	0.022933	0.0057	0.020214	0.80298	-0.31656	0.40235	0.64785	0.93972	-0.0897	0.58542	0.86163	1.0487	0.068653	0.96836	0.99071	0.9993	-0.00101	0.22574	0.33285	1.2363	0.30598
940.6409	9.46	olomics Pc	0.37074	0.99951	1.305	0.38404	0.51447	0.99998	0.92205	-0.11709	0.37892	0.99941	1.1275	0.17308	0.16666	0.57753	0.81929	-0.28755	0.057618	0.18653	0.73208	-0.44992	0.0494	0.18503	0.78145	-0.35578	0.7383	0.99391	0.98012	-0.02896	0.79308	0.99956	1.1129	0.15435	0.10593	0.36112	1.7663	0.82076
177.1642	2.78	domics Pos	0.37083	0.99987	1.1094	0.14977	0.22738	0.99994	0.79161	-0.33713	0.16189	0.99996	0.78845	-0.34292	0.024988	0.27914	0.56238	-0.83037	1.39E-05	0.000288	0.19784	-2.3376	0.000503	0.034246	0.36842	-1.4406	0.015402	0.33809	0.52085	-0.94105	0.088299	0.82455	0.69541	-0.52407	0.91175	0.93505	0.97953	-0.02984
354.3735	8.3	olomics Pc	0.37166	0.99951	0.92621	-0.1106	0.099716	0.99998	0.82043	-0.28555	0.14664	0.99941	0.82476	-0.27796	0.65767	0.99905	0.96579	-0.05022	0.79608	0.99688	1.0122	0.017495	0.010661	0.081707	0.69763	-0.51947	0.12877	0.67606	0.75049	-0.4141	0.014149	0.47452	0.75676	-0.40209	0.061341	0.30404	0.80395	-0.31482
630.5531	6.1	domics Pos	0.37176	0.99987	1.169	0.22531	0.85068	0.99994	1.0388	0.05498	0.57302	0.99996	1.1023	0.14046	0.6527	0.95465	0.93698	-0.09391	0.22738	0.32147	0.80379	-0.31512	0.022113	0.13032	0.68753	-0.5405	0.091211	0.52136	0.77991	-0.35862	0.19296	0.92582	0.8357	-0.25894	0.67187	0.75548	1.0977	0.13443
1119.224	4.4	domics Pos	0.37214	0.99987	1.2022	0.26569	0.095458	0.99994	0.84585	-0.24153	0.8467	0.99996	1.0225	0.032165	0.010929	0.21371	0.58296	-0.77854	0.000778	0.004607	0.58293	-0.7786	0.009381	0.086818	0.58357	-0.77701	0.4083	0.77901	0.89595	-0.1585	0.59309	0.97073	0.94265	-0.08521	0.064236	0.16253	1.5706	0.65127
392.7982	9.92	domics Pos	0.37218	0.99987	1.0755	0.10504	0.13068	0.99994	0.87327	-0.1955	0.10493	0.99996	0.88254	-0.18026	0.2134	0.66262	0.89701	-0.15681	0.013196	0.03786	0.71345	-0.48712	0.42624	0.66174	0.93579	-0.09574	0.4309	0.79133	1.0409	0.057882	0.041008	0.64308	0.85044	-0.23371	0.01301	0.098791	1.3695	0.45363
746.5122	8.74	olomics Ne	0.37287	0.99918	1.1021	0.14029	0.85196	0.98874	1.0427	0.060309	0.62761	0.99995	1.0399	0.056437	0.82737	0.99985	1.0279	0.039725	0.072905	0.99927	1.1606	0.21486	0.10085	0.55533	1.2283	0.29663	0.036243	0.35692	1.2268	0.29495	0.11866	0.99512	1.1303	0.17665	0.10662	0.99941	0.88038	-0.1838
765.0478	4.64	domics Pos	0.3732	0.99987	1.1164	0.15885	0.30291	0.99994	0.8221	-0.28261	0.68277	0.99996	0.93189	-0.10177	0.83666	0.98621	0.9505	-0.07324	0.28404	0.37842	0.78405	-0.35098	0.71884	0.85308	1.0311	0.044196	0.037911	0.37121	1.4398	0.52585	0.68527	0.97988	1.0629	0.088014	0.033284	0.12504	1.4294	0.51537
682.3849	2.28	domics Pos	0.37339	0.99987	0.89496	-0.1601	0.11061	0.99994	0.82711	-0.27385	0.060167	0.99996	0.6665	-0.58531	0.002545	0.17581	0.51567	-0.95547	0.000931	0.005291	0.4684	-1.0942	0.007432	0.082061	0.43501	-1.2009	0.042695	0.38464	0.57572	-0.79656	0.011615	0.52955	0.45628	-1.132	0.056875	0.15414	0.59267	-0.7547
137.9849	2.64	olomics Ne	0.37365	0.99918	0.9432	-0.08437	0.0489	0.47152	0.87543	-0.19194	0.50556	0.99995	1.038	0.05375	0.45292	0.99985	1.0569	0.079867	0.091775	0.99927	1.1239	0.16849	0.17835	0.65203	1.0836	0.11586	0.88912	0.96688	0.99075	-0.01341	0.68595	0.99512	1.0296	0.042103	0.98395	0.99945	1.0017	0.002473
902.7952	10.03	domics Pos	0.37372	0.99987	1.035	0.049662	0.094718	0.99994	0.89666	-0.15737	0.15485	0.99996	0.90546	-0.14327	0.62102	0.94062	0.96679	-0.04873	0.004047	0.015432	0.81323	-0.29826	0.32426	0.60075	0.94076	-0.0881	0.96584	0.99292	1.0103	0.01484	0.59438	0.97073	1.0462	0.065195	0.082187	0.18348	1.1898	0.25077
1153.766	10.03	domics Pos	0.37408	0.99987	1.076	0.10568	0.18336	0.99994	0.88468	-0.17677	0.24104	0.99996	0.90899	-0.13767	0.11323	0.53238	0.88949	-0.16894	0.003183	0.012843	0.74214	-0.43024	0.14267	0.41448	0.87335	-0.19537	0.50352	0.82964	0.95741	-0.06279	0.50393	0.96776	0.94007	-0.08916	0.26672	0.37681	1.0709	0.098758
1028.187	9.07	olomics Pc	0.37417	0.99951	1.2536	0.32606	0.35888	0.99998	0.86166	-0.21482	0.72458	0.99941	1.0271	0.038627	0.13095	0.50428	0.75189	-0.41141	0.027332	0.10682	0.65029	-0.62084	0.26833	0.56005	0.81386	-0.29715	0.64196	0.99391	1.1234	0.16789	0.89137	0.99956	1.0059	0.008513	0.64828	0.83933	1.1827	0.2421

648.4503	9.59	olomics Po	0.37453	0.99951	1.3409	0.42325	0.51178	0.99998	0.90424	-0.14522	0.68617	0.99941	1.0465	0.065526	0.01638	0.20475	0.64429	-0.63423	0.001511	0.012724	0.5074	-0.97881	0.047267	0.17917	0.67677	-0.56326	0.9575	0.9974	1.0303	0.042997	0.83961	0.99956	1.0497	0.069993	0.11068	0.36452	1.6266	0.70182
1095.79	9.68	olomics Po	0.37462	0.99951	1.2642	0.33823	0.47225	0.99998	0.8929	-0.16344	0.95523	0.99941	1.0265	0.037718	0.013647	0.20085	0.59887	-0.73968	0.019408	0.082169	0.62717	-0.67306	0.009202	0.076684	0.52039	-0.94234	0.40766	0.98164	0.84965	-0.23506	0.88429	0.99956	1.0166	0.023779	0.20241	0.46778	1.6524	0.72454
621.4954	2.05	domics Pos	0.3752	0.99987	1.0967	0.13311	0.56012	0.99994	0.91301	-0.13129	0.90856	0.99996	0.99795	-0.00296	0.11226	0.53238	0.69359	-0.52784	3.93E-06	0.000145	0.19533	-2.356	0.004385	0.071311	0.49448	-1.016	0.17296	0.64606	0.78006	-0.35835	0.61629	0.97665	0.93513	-0.09676	0.019045	0.10479	1.3477	0.43052
497.238	0.58	domics Pos	0.37599	0.99987	1.4643	0.55024	0.10185	0.99994	0.74607	-0.42261	0.88867	0.99996	0.96742	-0.04779	0.045875	0.3706	0.59163	-0.75723	0.001181	0.006194	0.4655	-1.1031	0.015016	0.10343	0.64455	-0.63365	0.54549	0.85011	0.89971	-0.15246	0.92908	0.98573	1.038	0.053795	0.032423	0.12301	2.3728	1.2466
90.9775	10.03	domics Pos	0.3763	0.99987	1.0269	0.038333	0.2508	0.99994	0.94763	-0.0776	0.27726	0.99996	0.95714	-0.0632	0.21243	0.66014	0.94076	-0.0881	6.19E-05	0.000745	0.7627	-0.39081	0.36504	0.62558	0.92687	-0.10955	0.76729	0.94339	1.0156	0.022386	0.82055	0.97988	0.99022	-0.01418	0.011038	0.094875	1.1999	0.26293
710.8082	10.12	domics Pos	0.3765	0.99987	1.0976	0.13435	0.14095	0.99994	0.95354	-0.06864	0.54688	0.99996	1.0498	0.070059	0.55242	0.90826	1.0798	0.11073	0.002185	0.009658	0.83148	-0.26625	0.42057	0.65728	0.94165	-0.08673	0.79398	0.95091	0.98686	-0.01909	0.34255	0.96524	1.0834	0.11557	0.000202	0.07528	1.2774	0.35324
154.1209	9.97	domics Pos	0.3771	0.99987	1.2794	0.35546	0.93253	0.99994	1.0116	0.016575	0.52084	0.99996	0.93883	-0.09106	0.18965	0.63211	0.85565	-0.22491	0.016048	0.044055	0.75725	-0.40116	0.028142	0.14808	0.75447	-0.40646	0.95806	0.98985	1.019	0.027204	0.88006	0.98023	0.9955	-0.0065	0.07893	0.1809	1.1633	0.21818
206.1976	2.05	domics Pos	0.37719	0.99987	1.0793	0.11005	0.32805	0.99994	0.87203	-0.19754	0.78112	0.99996	0.97171	-0.0414	0.023886	0.27465	0.75236	-0.41051	8.89E-06	0.000208	0.23478	-2.0906	0.048217	0.21304	0.59546	-0.74792	0.20752	0.65208	0.83247	-0.26454	0.56805	0.9703	0.95058	-0.07312	0.085784	0.18648	1.192	0.25342
645.2995	2.99	domics Pos	0.37748	0.99987	0.83805	-0.25489	0.60837	0.99994	0.91713	-0.12479	0.46732	0.99996	0.89277	-0.16363	0.4575	0.87572	0.88414	-0.17765	0.00707	0.023524	0.40682	-1.2975	0.090746	0.31774	0.74219	-0.43014	0.78493	0.94953	1.0609	0.085346	0.49429	0.96776	1.0766	0.1065	0.050516	0.14715	1.3316	0.41314
195.1239	6.16	olomics Po	0.37813	0.99951	0.93149	-0.10239	0.88415	0.99998	1.0028	0.004104	0.51999	0.99941	0.93784	-0.09259	0.4909	0.96613	1.1167	0.15929	0.003119	0.021166	1.3069	0.38614	0.08267	0.26146	1.2185	0.28513	0.68718	0.99391	1.0582	0.081566	0.78667	0.99956	0.97987	-0.02934	0.54607	0.77975	0.94407	-0.08303
602.4374	9.62	olomics Po	0.37827	0.99951	1.2995	0.378	0.4827	0.99998	0.87622	-0.19064	0.87752	0.99941	1.0214	0.030579	0.053454	0.29934	0.7015	-0.51149	0.02774	0.10814	0.61557	-0.7	0.044744	0.17251	0.65854	-0.60266	0.93042	0.9951	1.0082	0.011813	0.84964	0.99956	0.97708	-0.03345	0.031813	0.2688	1.7626	0.81768
744.9165	3.22	olomics Po	0.37852	0.99951	1.1831	0.24258	0.024236	0.99998	0.83056	-0.26784	0.71639	0.99941	1.0853	0.11815	0.015359	0.20428	0.65298	-0.61489	0.003778	0.024191	0.61916	-0.69161	0.006521	0.067169	0.65641	-0.60733	0.4691	0.99391	0.90665	-0.14138	0.16206	0.9444	0.83261	-0.26429	0.10838	0.36254	1.582	0.66177
428.9785	5	olomics Ne	0.3787	0.99918	1.0275	0.039114	0.045157	0.47152	0.91151	-0.13367	0.53191	0.99995	0.96954	-0.04462	0.555	0.99985	0.96125	-0.05702	0.83758	0.99927	1.0175	0.024992	0.7962	0.95294	0.98914	-0.01576	0.92088	0.9764	1.0068	0.00976	0.29457	0.99512	0.95249	-0.07022	0.50837	0.99941	1.0253	0.036042
234.8629	0.46	olomics Ne	0.37905	0.99918	1.1675	0.22345	0.41083	0.8002	1.2574	0.33043	0.05584	0.99995	1.4142	0.50003	0.047829	0.99985	1.4498	0.53582	0.020207	0.91567	1.6846	0.75237	0.24147	0.68109	1.5114	0.59587	0.018478	0.29108	1.5935	0.67224	0.013722	0.99512	1.6014	0.67931	0.41236	0.99941	1.1822	0.24145
652.4115	2.78	olomics Po	0.3792	0.99951	1.2676	0.34207	0.27481	0.99998	0.89471	-0.1605	0.72259	0.99941	0.96767	-0.04741	0.004705	0.18788	0.51698	-0.95181	0.002379	0.017364	0.49756	-1.0071	0.011712	0.083709	0.59848	-0.74062	0.51984	0.99391	0.92301	-0.11559	0.82023	0.99956	1.0605	0.084736	0.030625	0.2688	2.1359	1.0948
829.5691	9.24	olomics Po	0.37955	0.99951	1.2521	0.32431	0.69959	0.99998	0.9409	-0.08788	0.4124	0.99941	1.1062	0.14555	0.19999	0.63506	0.80813	-0.30735	0.025776	0.10239	0.65679	-0.6065	0.097871	0.29524	0.75123	-0.41267	0.9005	0.9951	0.98439	-0.02269	0.6784	0.99956	0.94318	-0.08439	0.25609	0.52587	1.4387	0.5248
935.6487	8.91	olomics Po	0.37965	0.99951	1.2951	0.37311	0.3733	0.99998	0.90026	-0.15159	0.13454	0.99941	1.1901	0.25105	0.069694	0.3437	0.82126	-0.28409	0.02129	0.088474	0.74884	-0.41728	0.042264	0.16681	0.74503	-0.42462	0.34439	0.95246	1.1743	0.23186	0.69664	0.99956	1.1713	0.22811	0.10461	0.35957	1.5794	0.65939
881.5424	10.38	olomics Ne	0.37988	0.99918	1.0981	0.13507	0.027133	0.40327	0.82529	-0.27702	0.31964	0.99995	1.0629	0.087971	0.62265	0.99985	1.0317	0.044959	0.67944	0.99927	0.97771	-0.03252	0.12065	0.58607	1.1219	0.16589	0.16227	0.54067	1.2867	0.36367	0.27523	0.99512	1.0856	0.1185	0.41088	0.99941	0.93955	-0.08996
1139.746	0.53	domics Pos	0.38002	0.99987	0.96447	-0.05219	0.21222	0.99994	0.89498	-0.16008	0.21628	0.99996	0.91962	-0.12089	0.086982	0.49135	0.88794	-0.17147	0.01486	0.041601	0.80631	-0.31059	0.025782	0.14185	0.85852	-0.22007	0.67906	0.90338	0.97575	-0.03542	0.51806	0.96776	0.94209	-0.08606	0.10804	0.21094	1.2491	0.32093
411.0952	1.07	domics Pos	0.38078	0.99987	1.2138	0.27957	0.46599	0.99994	0.90951	-0.13685	0.87513	0.99996	1.0625	0.087467	0.14826	0.57591	0.7673	-0.38213	0.00182	0.008462	0.63075	-0.66485	0.38828	0.64105	0.88508	-0.17611	0.90045	0.97745	1.0624	0.087319	0.99135	0.99721	1.0219	0.031261	0.03645	0.12924	1.482	0.56753
160.8534	12.29	olomics Ne	0.38081	0.99918	0.96853	-0.04613	0.098598	0.51286	0.92898	-0.10628	0.57012	0.99995	1.0317	0.045087	0.29058	0.99985	0.95482	-0.0667	0.76385	0.99927	0.98256	-0.02538	0.67435	0.92957	1.0241	0.034293	0.017909	0.29108	1.1244	0.1691	0.8101	0.99512	0.98991	-0.01463	0.87819	0.99941	0.99446	-0.00801
1138.817	9.64	olomics Po	0.38083	0.99951	1.4238	0.50976	0.46572	0.99998	0.88708	-0.17286	0.66252	0.99941	1.0843	0.11679	0.047585	0.28918	0.65747	-0.60499	0.013224	0.060021	0.58282	-0.77888	0.034327	0.14398	0.65427	-0.61204	0.83324	0.9951	0.98197	-0.02624	0.86059	0.99956	1.112	0.15311	0.11444	0.36506	1.8119	0.85749
703.3236	5.81	domics Pos	0.38159	0.99987	0.89287	-0.16348	0.63426	0.99994	0.95104	-0.07242	0.27974	0.99996	0.84871	-0.23665	0.018339	0.25335	0.79313	-0.33438	0.054296	0.11079	0.79931	-0.32318	0.054432	0.23061	0.71934	-0.47526	0.4936	0.82295	0.9187	-0.12233	0.36159	0.96776	0.91085	-0.13471	0.36785	0.47659	0.93749	-0.09313
313.3284	7.86	olomics Po	0.38191	0.99951	0.88141	-0.18212	0.10299	0.99998	0.73686	-0.44053	0.12198	0.99941	0.77108	-0.37505	0.4338	0.91715	0.89191	-0.16502	0.81475	0.99688	1.019	0.027125	0.023736	0.11397	0.63536	-0.65435	0.14671	0.7187	0.75986	-0.39619	0.043266	0.6521	0.71268	-0.48867	0.098127	0.35245	0.77156	-0.37414
614.4838	6.21	olomics Po	0.38199	0.99951	0.94988	-0.07418	0.92695	0.99998	1.0164	0.023432	0.030528	0.99941	0.85999	-0.21761	0.52517	0.98929	1.0584	0.081882	0.17809	0.45788	0.92162	-0.11776	0.66494	0.90011	0.97071	-0.04289	0.16969	0.74446	0.95819	-0.06161	0.54063	0.99956	0.94507	-0.0815	0.68737	0.85818	0.98191	-0.02633
117.0742	0.59	domics Pos	0.38216	0.99987	0.86633	-0.20701	0.34634	0.99994	0.84899	-0.23617	0.35293	0.99996	1.1487	0.20005	0.082757	0.4849	1.5544	0.63635	0.19598	0.28941	1.2931	0.37085	0.011422	0.091691	1.6029	0.68066	0.001463	0.23318	2.2857	1.1926	0.055814	0.70726	1.5543	0.63623	0.045575	0.14174	1.5002	0.58515
188.6361	1.91	domics Pos	0.38219	0.99987	1.1302	0.17661	0.13035	0.99994	0.79417	-0.33248	0.95112	0.99996	1.0198	0.028259	0.28475	0.75034	0.81606	-0.29325	2.92E-06	0.000144	0.17239	-2.5362	0.00351	0.06658	0.48772	-1.0359	0.029419	0.35275	0.67751	-0.56169	0.35742	0.96776	0.90621	-0.14208	0.015029	0.09885	1.3289	0.41018
1155.82	9.59	olomics Po	0.3822	0.99951	1.316	0.3961	0.45691	0.99998	0.91069	-0.13497	0.98906	0.99941	0.99656	-0.00498	0.019503	0.21575	0.6154	-0.70041	0.007404	0.038361	0.60211	-0.73189	0.013525	0.086244	0.70829	-0.49758	0.7503	0.99391	0.97273	-0.03989	0.91519	0.99956	1.046	0.064943	0.20747	0.47381	1.3755	0.45996
539.9655	0.52	domics Pos	0.38221	0.99987	1.1213	0.16522	0.72743	0.99994	0.95731	-0.06295	0.26806	0.99996	1.1081	0.14803	0.14431	0.57187	1.2332	0.30237	0.33232	0.42664	0.88463	-0.17685	0.40394	0.64934	1.0972	0.13376	0.008849	0.32443	1.401	0.48646	0.47047	0.96776	1.164	0.21904	0.091336	0.19178	1.4573	0.54334
1030.628	1.3	domics Pos	0.38272	0.99987	1.1132	0.15473	0.86662	0.99994	0.98805	-0.01735	0.5792	0.99996	1.0763	0.10614	0.32518	0.78924	0.88125	-0.18238	0.053836	0.1103	0.65745	-0.60504	0.42873	0.66292	1.1376	0.18601	0.040162	0.37718	1.5868	0.66616	0.43582	0.96776	1.1774	0.23557	0.004107	0.085451	2.2758	1.1864
690.4773	9.5	olomics Po	0.38281	0.99951	1.2563	0.32913	0.49021	0.99998	0.90981	-0.13637	0.9352	0.99941	1.0053	0.007676	0.035034	0.25605	0.67025	-0.57723	0.004507	0.027471	0.61555	-0.70004	0.016842	0.096027	0.65642	-0.60732	0.80613	0.99391	0.9851	-0.02165	0.76736	0.99956	1.0027	0.003819	0.1046	0.35957	1.6538	0.72583
920.8517	12	olomics Po	0.38285	0.99951	0.93961	-0.08986	0.001836	0.99998	0.81403	-0.29685	0.16539	0.99941	0.92833	-0.10729	0.68361	0.99905	0.97999	-0.02917	0.006935	0.036963	0.82503	-0.27749	0.000854	0.042705	0.87415	-0.19404	0.82303	0.9951	0.98999	-0.01451	0.095677	0.81634	0.93893	-0.0909	0.033452	0.2688	0.89192	-0.16501
344.281	4.19	olomics Po	0.38292	0.99951	0.79264	-0.33525	0.04612	0.99998	0.57265	-0.80428	0.3713	0.99941	0.79513	-0.33074	0.01357	0.20085	0.47169	-1.0841	0.043529	0.15118	0.56249	-0.8301	0.007346	0.070977	0.29716	-1.7507	0.032679	0.35743	0.4038	-1.3083	0.019784	0.51556	0.40981	-1.287	0.004832	0.2688	0.26833	-1.8979
1144.777	10.02	domics Pos	0.38345	0.99987	1.0395	0.055943	0.36714	0.99994	0.95171	-0.07141	0.35817	0.99996	0.94087	-0.08794	0.20116	0.64674	0.90855	-0.13835	0.001373	0.006888	0.751	-0.41311	0.64475	0.81443	0.96308	-0.05427	0.76602	0.94339	0.98316	-0.0245	0.59925	0.97073	0.96644	-0.04924	0.00809	0.092077	1.2009	0.2641
1024.705	9.07	olomics Po	0.38403	0.99951	1.2358	0.30549	0.80438	0.99998	0.96494	-0.05149	0.55858	0.99941	1.1227	0.16692	0.19919	0.63506	0.80454	-0.31376	0.025399	0.10115	0.62765	-0.67196	0.20018	0.4741	0.77809	-0.36198	0.20138	0.79125	1.2365	0.30624	0.9721	0.99956	1.0623	0.087242	0.58758	0.80368	1.2164	0.28266
896.5763	8.94	olomics Po	0.38422	0.99951	1.2714	0.34638	0.36836	0.99998	0.88835	-0.17079	0.55117	0.99941	0.94354	-0.08384	0.013917	0.20085	0.70648	-0.50129	0.013859	0.062457	0.6485	-0.62483	0.086352	0.26979	0.82556	-0.27656	0.91496	0.9951	1.0142	0.020314	0.99217	0.99956	1.0172	0.024623	0.23245	0.49844	1.5473	0.62979
119.0864	0.76	domics Pos	0.38541	0.99987	1.195	0.25695	0.28111	0.99994	0.79415	-0.33251	0.95692	0.99996	0.99821	-0.00258	0.66593	0.95717	1.1079	0.14788	0.033657	0.077449	0.62741	-0.67251	0.64763	0.81526	0.92302	-0.11557	0.89274	0.97532	0.96242	-0.05527	0.80911	0.97988	0.9586	-0.061	0.1391	0.24443	1.412	0.49773
1009.748	9.82	olomics Po	0.38559	0.99951	1.2655	0.33972	0.094603	0.99998	0.75958	-0.39673	0.74217	0.99941	1.0273	0.038869	0.001497	0.14411	0.55623	-0.84625	0.008126	0.041353	0.60321	-0.72927	0.014822	0.091119	0.63015	-0.66624	0.58619	0.99391	0.92509	-0.11234	0.58829	0.99956	0.93287	-0.10025	0.11413	0.36506	1.7676	0.82177
1093.81	9.7	olomics Po	0.3857	0.99951	1.3319	0.41352	0.37676	0.99998	0.85437	-0.22707	0.81377	0.99941	1.0334	0.047343	0.041878	0.2802	0.61201	-0.70836	0.005231	0.030289	0.53957	-0.89011	0.030855	0.13499	0.55695	-0.84439	0.44072	0.99391	0.86105	-0.21583	0.98287	0.99956	1.0818	0.11346	0.14259	0.39464	1.726	0.7874
763.6033	5.67	domics Pos	0.38653	0.99987	0.94701	-0.07855	0.11078	0.99994	0.83863	-0.25389	0.16912	0.99996	0.88233	-0.1806	0.085817	0.49062	0.84125	-0.24939	0.006534	0.022283	0.75435	-0.4067	0.9509	0.9734	0.99541	-0.00664	0.13231	0.59878	1.2617	0.3354	0.55188	0.96776	1.0487	0.068649	0.039246	0.13267	1.271	0.34601
941.1037	8.91	olomics Po	0.38696	0.99951	1.3072	0.38647	0.4444	0.99998	0.88238	-0.18053	0.90222	0.99941	1.0253	0.036088	0.030749	0.24688	0.7316	-0.45088	0.12421	0.34377	0.69227	-0.53059	0.71624	0.91513	0.95725	-0.06303	0.5696	0.99391	1.2244	0.29207	0.59075	0.99956	1.132	0.17889	0.10146	0.35354	1.8235	0.86671
1051.175	4.24	domics Pos	0.38708	0.99987	1.2452	0.31638	0.85435	0.99994	0.93504	-0.0969	0.62673	0.99996	1.093	0.12824	0.19721	0.64214	0.72745	-0.45907	0.022821	0.057702	0.51499	-0.95738	0.075947	0.28503	0.63474	-0.65576	0.64125	0.8857	0.89222	-0.16453	0.88151	0.98023	0.98413	-0.02309	0.14777	0.25301	1.8285	0.87063
346.0276	0.57	domics Pos	0.38758	0.99987	0.9373	-0.09341	0.036387	0.99994	0.80301	-0.31652	0.56567	0.99996	0.94894	-0.07561	0.038911	0.34213	1.3437	0.42616	0.90347	0.91743	1.0154	0.021981	0.16896	0.45562	1.1039	0.1426	0.001763	0.23318	1.4289	0.51492	0.46489	0.96776	0.91379	-0.13007	0.9848	0.98817	1.0381	0.053906
678.4691	2.38	domics Pos	0.38803	0.99987	1.2789	0.35486	0.31205	0.99994	1.5048	0.58958	0.67147	0.99996	1.2622	0.33589	0.3338	0.79326	0.78484	-0.34953	0.77473	0.80696	1.306	0.38511	0.26104	0.54699	2.0959	1.0675	0.039491	0.37421	2.1303	1.091	0.88316	0.98023	1.0449	0.063308	0.54679	0.64235	1.3007	0.37924
546.832	10.1	domics Pos	0.3882	0.99987	0.95098	-0.07252	0.53829	0.99994	0.95956	-0.05955	0.35536	0.99996	0.91551	-0.12735	0.066523	0.43847	0.92518	-0.11219	0.001008	0.005533	0.73496	-0.44427	0.15177	0.43005	0.8314	-0.26638	0.37389	0.76056	0.94965	-0.07454	0.092876	0.83714	0.90668	-0.14133	0.11888	0.22318	1.1483	0.19955
1050.199	9.06	olomics Po	0.38899	0.99951	1.2227	0.2901	0.69919	0.99998	0.95017	-0.07374	0.73472	0.99941	1.0627	0.087745	0.15257	0.54839	0.77218	-0.37299	0.030713	0.11716	0.65042	-0.62056	0.12994	0.35968	0.78744	-0.34475	0.9311	0.9951	1.0947	0.13053	0.78378	0.99956	1.0857	0.11868	0.18055	0.44542	1.3673	0.45137
1112.754	9.02	olomics Po	0.38936	0.99951	1.29	0.36737	0.50777	0.99998	0.90187	-0.14901	0.99151	0.99941	1.0405	0.057276	0.009789	0.19702	0.64923	-0.6232	0.013032	0.059342	0.60472	-0.72567	0.16423	0.41957	0.75835	-0.39906	0.54754	0.99391	1.1811	0.24012	0.20272	0.99956	1.2361	0.30575	0.073297	0.31731	1.6188	0.69496
362.9842	4.29	olomics Ne	0.38943	0.99918	0.96226	-0.0555	0.026904	0.40327	0.8963	-0.15795	0.3811	0.99995	0.95114	-0.07227	0.39656	0.99985	0.95762	-0.06248	0.16711	0.99927	0.92966	-0.10523	0.98687	0.99648	1.0075	0.01077	0.9414	0.98374	0.99831	-0.00243	0.48433	0.99512	0.95836	-0.06137	0.21711	0.99941	0.95523	-0.06608
1016.66	5.31	olomics Po	0.38962	0.99951	1.2253	0.29313	0.50895	0.99998	1.2617	0.33541	0.71009	0.99941	1.1637	0.21877	0.80864	0.99905	1.1358	0.18375	0.76701	0.99688	1.1863	0.24643	0.057246	0.20468	1.7131	0.77665	0.010403	0.24769	2.2621	1.1777	0.24762	0.99956	1.4108	0.4965	0.046512	0.28616	1.7975	0.84603
734.8083	10.09	domics Pos	0.38987	0.99987	1.1011	0.13893	0.3226	0.99994	0.92363	-0.11462	0.75171	0.99996	1.0313	0.044424	0.27547	0.74022	1.0763	0.10613	0.026558	0.064749	0.80828	-0.30707	0.71533	0.85236	0.96816	-0.04669	0.38747	0.76619	1.0767	0.10668	0.94724	0.98838	1.0117	0.016791	0.061791	0.15909	1.21	0.27506
714.9954	9.5	olomics Po	0.39003	0.99951	1.2556	0.32832	0.051937	0.99998	0.76159	-0.39291	0.24994	0.99941	1.0762	0.10593	0.008062	0.19613	0.67555	-0.56586	1.16E-05	0.000348	0.47481	-1.0746	0.001797	0.045425	0.58116	-0.783	0.63086	0.99391	0.95906	-0.06031	0.73082	0.99956	1.0105	0.015048	0.12573	0.3747	1.6254	0.70081
718.5727	9.26	olomics Po	0.3906	0.99951	1.0627	0.087745	0.45072	0.99998	1.0481	0.067745	0.87906	0.99941	0.9893	-0.01552	0.40763	0.90108	1.0808	0.11204	0.40517	0.80473	1.0838	0.11615	0.5922	0.86607	1.0511	0.071913	0.3556	0.96419	0.92665	-0.10991	0.012899	0.47452	0.80731	-0.3088	0.080072	0.32714	0.86739	-0.20524
1030.665	9.07	olomics Po	0.39065	0.99951	1.2853	0.36212	0.40218	0.99998	0.84468	-0.24352	0.57048	0.99941	1.0769	0.10694	0.19665	0.63016	0.75242	-0.41039	0.020974	0.087507	0.60405	-0.72727	0.37217	0.68477	0.84889	-0.23635	0.7917	0.99391	1.1035	0.14208	0.92943	0.99956	1.0115	0.016528	0.49347	0.74768	1.2504	0.3224
189.1664	0.3	olomics Po	0.39076	0.99951	1.6208	0.6967	0.8173	0.99998	0.98473	-0.0222	0.62651	0.99941	0.94774	-0.07744	0.40529	0.8978	1.1995	0.26245	0.3121	0.6832	1.1511	0.20306	0.003356	0.049867	1.8771	0.90849	0.001257	0.16989	1.9759	0.98254	0.378	0.99956	1.2209	0.28798	0.03019	0.2688	1.3002	0.37878
539.7727	2.34	olomics Po	0.39094	0.99951	1.3645	0.44835	0.83638	0.99998	0.91826	-0.12302	0.50977	0.99941	0.78853	-0.34277	0.11719	0.46845	0.54333	-0.88011	0.002644	0.018589	0.17702	-2.498	0.20678	0.48391	0.55102	-0.85983	0.53894	0.99391	0.79952	-0.32279	0.37134	0.99956	0.73162	-0.45084	0.28106	0.55612	1.2861	0.36295
191.1799	2.78	domics Pos	0.39107	0.99987	1.1858	0.24582	0.80546	0.99994	0.93651	-0.09464	0.37006	0.99996	0.82227	-0.28232	0.066416	0.43847	0.64166	-0.64013	0.000289	0.002152	0.25447	-1.9745	0.005094	0.073856	0.42577	-1.2319	0.023918	0.33809	0.55368	-0.85289	0.31959	0.9524	0.79146	-0.33741	0.36194	0.47102	1.1582	0.21192
1072.217	9.36	olomics Po	0.39171	0.99951	1.4418	0.52786	0.1858	0.99998	0.83856	-0.25401	0.55803	0.99941	0.94016	-0.08902	0.020593	0.22211	0.6443	-0.6342	0.009675	0.047321	0.56281	-0.82927	0.05147	0.19142	0.68186	-0.55246	0.92879	0.9951	1.0412	0.058306	0.49577	0.99956	0.90442	-0.14494	0.048928	0.289	1.6096	0.68667
1108.558	5.79	domics Pos	0.39209	0.99987	1.0686	0.095732	0.82383	0.99994	0.97997	-0.02919	0.17777	0.99996	0.84802	-0.23784	0.34103	0.79726	0.89406	-0.16156	0.01809	0.04827	0.75271	-0.40984	0.03635	0.17369	0.71384	-0.48633	0.036257	0.36759	0.81493	-0.29526	0.042139	0.64896	0.79945	-0.32292	0.42758	0.53402	1.0807	0.11197
208.8926	0.56	domics Pos	0.39212	0.99987	1.0783	0.10875	0.31837	0.99994	1.0902	0.12464	0.96788	0.99996	0.99838	-0.00234	0.027908	0.29642	0.8805	-0.18361	0.08653	0.15685	0.83329	-0.2631	0.010221	0.08787	0.84109	-0.24967	0.37907	0.76056	0.92728	-0.10892	0.7248	0.97988	0.98008	-0.02903	0.26814	0.37839	1.0966	0.13307
808.5825	5.42	domics Pos	0.39227	0.99987	1.0437	0.061676	0.21202	0.99994	0.92129	-0.11827	0.38842	0.99996	0.94564	-0.08064	0.4114	0.85414	0.94515	-0.08139	0.00259	0.010973	0.77317	-0.37115	0.42238	0.65844	0.92835	-0.10725	0.41228	0.78028	1.0618	0.086477	0.97316	0.99223	1.0012	0.001752	0.004469	0.085635	1.2359	0.30553
582.4124	9.7	olomics Po	0.39237	0.99951	1.424	0.50996	0.97543	0.99998	0.99887	-0.00163	0.62347	0.99941	1.0491	0.069192	0.050796	0.29486	0.73115	-0.45175	0.006032	0.033508	0.56761	-0.81702	0.044419	0.1721	0.7377	-0.43889	0.89319	0.9951	1.0201	0.028751	0.64116	0.99956	1.2695	0.34425	0.21832	0.48624	1.5419	0.6247
460.7788	0.56	domics Pos	0.39346	0.99987	1.1575	0.21107	0.48802	0.99994	1.0787	0.10934	0.97409	0.99996	1.0162	0.023241	0.020906	0.26222	0.79685	-0.32762	0.076289	0.14337	0.80599	-0.31116	0.18957	0.47618	0.85181	-0.2314	0.51539	0.8359	0.92602	-0.11089	0.36105	0.96776	1.1134	0.15493	0.30026	0.41006	1.1792	0.23782
744.5603	9.29	olomics Po	0.39349	0.99951	1.2836	0.36017	0.4863	0.99998	1.3251	0.40615	0.85105	0.99941	0.98237	-0.02566	0.66181	0.99905	1.2785	0.35449	0.043861	0.15199	1.8559	0.89209	0.042559	0.16758	2.0619	1.0439	0.016543	0.29901	2.3742	1.2474	0.18092	0.97561	1.8843	0.91404	0.001214	0.2688	2.0644	1.0457
337.9255	0.33	olomics Po	0.39358	0.99951	0.92248	-0.11641	0.8653	0.99998	1.0017	0.002488	0.48771	0.99941	1.18	0.23883	0.048836	0.29135	1.29	0.3674	0.040813	0.1438	1.2761	0.35169	0.23879	0.5292	1.2541	0.32663	0.004701	0.20152	1.4399	0.52601	0.87105	0.99956	1.0374	0.053011	0.14736	0.40018	0.87097	-0.1993
147.0911	0.6	domics Pos	0.3939	0.99987	0.90243	-0.14811	0.13382	0.99994	0.84967	-0.23503	0.12926	0.99996	1.2327	0.30182	0.081051	0.4813	1.3929	0.47813	0.83199	0.85828	1.0471	0.066344	0.49097	0.70939	1.0675	0.09423	0.013606	0.33809	1.3783	0.46294	0.15347	0.91321	1.208	0.27266	0.060827	0.1578	1.4611	0.54706
437.1941	0.75	domics Pos	0.39431	0.99987	1.1446	0.1949	0.30546	0.99994	0.79352	-0.33366	0.85656	0.99996	0.94633	-0.07958	0.77449	0.97526	1.068	0.094975	0.048559	0.10213	0.61535	-0.70052	0.57054	0.76466	0.87942	-0.18538	0.69758	0.91186	0.90914	-0.13743	0.75553	0.97988	0.93155	-0.10229	0.25358	0.36241	1.2929	0.37062
322.8785	10.15	domics Pos	0.39435	0.99987	0.92585	-0.11115	0.92055	0.99994	0.98712	-0.01871	0.88273	0.99996	0.98515	-0.02159	0.22534	0.6807	0.90019	-0.1517	0.003979	0.015231	0.7102	-0.49371	0.27722	0.56376	0.89367	-0.16218	0.94839	0.98901	1.0033	0.004804	0.469	0.96776	0.92674	-0.10976	0.02205	0.10842	1.244	0.315
818.785	0.54	domics Pos	0.39446	0.99987	1.04	0.056541	0.25219	0.99994	0.92598	-0.11095	0.24324	0.99996	0.87796	-0.18778	0.002625	0.17581	0.81488	-0.29533	0.001818	0.008462	0.69488	-0.52516	0.21419	0.5057	0.85334	-0.2288	0.41591	0.78082	0.93708	-0.09375	0.1746	0.91806	0.88859	-0.17041	0.36193	0.47102	1.1272	0.17272
564.624	0.32	olomics Po	0.39475	0.99951	1.155	0.20795	0.15128	0.99998	1.1554	0.20841	0.27433	0.99941	1.1153	0.15739	0.18355	0.61171	1.1721	0.22912	0.082692	0.24643	1.1915	0.25284	0.020918	0.10792	1.304	0.38297	0.16396	0.73936	1.1555	0.20848	0.19635	0.99341	1.1376	0.18601	0.66884	0.84958	1.0659	0.092051
1181.491	6.28	domics Pos	0.3954	0.99987	1.1794	0.23802	0.79019	0.99994	0.95239	-0.07037	0.73721	0.99996	1.0857	0.11869	0.89801	0.9874	1.026	0.036991	0.75276	0.78941	1.0877	0.12133	0.034841	0.16859	1.533	0.61634	0.015279	0.33809	1.708	0.77235	0.018307	0.52955	1.6636	0.73433	0.023193	0.1104	1.9581	0.96949
860.653	10.34	olomics Ne	0.39578	0.99918	0.94835	-0.07651	0.013997	0.37315	0.8255	-0.27666	0.29557	0.99995	0.9042	-0.14529	0.65679	0.99985	0.96179	-0.0562	0.60826	0.99927	0.95777	-0.06225	0.5489	0.87267	0.9491	-0.07536	0.1561	0.53641	0.86416	-0.21064	0.52543	0.99512	0.95405	-0.06787	0.50509	0.99941	0.9543	-0.06749
1048.186	8.85	olomics Po	0.39585	0.99951	1.2439	0.31484	0.46045	0.99998	0.89637	-0.15784	0.70566	0.99941	1.0416	0.058847	0.034027	0.25399	0.67403	-0.56912	0.03589	0.13192	0.61611	-0.69875	0.057684	0.20572	0.68665	-0.54236	0.9346	0.99628	1.0529	0.074413	0.62208	0.99956	0.94702	-0.07853	0.15538	0.41304	1.6159	0.69232
981.6827	9.11	olomics Po	0.39638	0.99951	1.1949	0.25689	0.38154	0.99998	0.88495	-0.17633	0.49426	0.99941	1.0843	0.11681	0.52088	0.98782	0.90146	-0.14967	0.015277	0.067303	0.69404	-0.5269	0.098341	0.29587	0.71676	-0.48044	0.72544	0.99391	0.95001	-0.07399	0.67993	0.99956	0.95317	-0.0692	0.24144	0.50938	1.3988	0.48421
1027.685	4.54	domics Pos	0.39653	0.99987	1.2747	0.35018	0.091524	0.99994	0.75061	-0.41386	0.652	0.99996	1.0692	0.09656	0.057626	0.4146	0.65236	-0.61626	0.008458	0.027011	0.42789	-1.2247	0.030297	0.15548	0.6339	-0.65768	0.59847	0.86481	0.90793	-0.13935	0.89684	0.98023	1.0514	0.072288	0.14888	0.254	1.8953	0.9224
808.7889	10.1	domics Pos	0.39669	0.99987	1.0627	0.087728	0.88521	0.99994	0.98994	-0.01459	0.6838	0.99996	1.0436	0.061624	0.34973	0.80284	0.93343	-0.09939	0.002166	0.00959	0.7654	-0.3857	0.68158	0.83502	0.96543	-0.05076	0.44699	0.80121	1.048	0.067625	0.8368	0.97988	0.98938	-0.0154	0.056406	0.15341	1.2066	0.27093
699.4808	9.45	olomics Po	0.3974	0.99951	1.2054	0.26953	0.33167	0.99998	0.83782	-0.25529	0.8769	0.99941	1.0207	0.029543	0.067914	0.34279	0.70177	-0.51094	0.004803	0.028711	0.55366	-0.85292	0.075917	0.24704	0.62868	-0.66959	0.81704	0.99391	0.96939	-0.04486	0.52622	0.99956	0.89771	-0.15567	0.02509	0.2688	1.6203	0.69623
842.7936	10.12	domics Pos	0.39841	0.99987	0.91594	-0.12667	0.59923	0.99994	0.93992	-0.08939	0.85979	0.99996	0.98348	-0.02403	0.42785	0.86135	0.90837	-0.13864	0.039317	0.087262	0.71696	-0.48004	0.20949	0.50173	0.84211	-0.24791	0.86555	0.97505	1.0474	0.066772	0.39094	0.96776	0.91142	-0.13381	0.12545	0.22986	1.1852	0.24509
635.8797	0.5	domics Pos	0.39922	0.99987	1.0411	0.058108	0.86612	0.99994	1.0247	0.035243	0.6231	0.99996	0.96874	-0.04582	0.49935	0.89009	0.95335	-0.06893	0.005457	0.01952	0.81191	-0.30061	0.80185	0.89925	0.98427	-0.02288	0.98658	0.99493	1.0061	0.008845	0.48524	0.96776	1.0364	0.051579	0.005379	0.088007	1.2248	0.29259
1159.762	9	olomics Po	0.39931	0.99951	1.3581	0.44156	0.74517	0.99998	0.97077	-0.0428	0.34003	0.99941	1.1212	0.16499	0.096599	0.41572	0.80437	-0.31407	0.026387	0.10405	0.64178	-0.63984	0.10262	0.30554	0.81068	-0.30279	0.30617	0.92378	1.3477	0.4305	0.63117	0.99956	1.1339	0.18129	0.066538	0.30823	1.7267	0.78802
271.8995	12.08	olomics Po	0.39952	0.99951	0.90256	-0.1479	0.13422	0.99998	0.84261	-0.24706	0.37205	0.99941	0.88383	-0.17816	0.71997	0.99905	0.95621	-0.0646	0.73293	0.99688	0.95887	-0.0606	0.35693	0.66686	0.88473	-0.17669	0.70147	0.99391	0.95308	-0.06933	0.040047	0.6387	0.75479	-0.40586	0.15955	0.41696	0.82737	-0.2734
722.5316	0.32	olomics Po	0.39984	0.99951	0.9334	-0.09943	0.94345	0.99998	0.99252	-0.01083	0.29145	0.99941	0.9196	-0.12092	0.031037	0.24688	0.84734	-0.23898	0.55969	0.94138	0.95042	-0.07337	0.033407	0.1422	0.80654	-0.31017	0.1892	0.76502	0.85177	-0.23146	0.64947	0.99956	0.96361	-0.05349	0.68343	0.85735	0.97147	-0.04176
1004.533	5.75	domics Pos	0.39997	0.99987	1.0912	0.12598	0.41894	0.99994	0.9113	-0.13401	0.83307	0.99996	1.0139	0.019957	0.92366	0.9874	1.0028	0.004063	0.006151	0.021284	0.76823	-0.38039	0.32065	0.5977	0.8559	-0.22449	0.82199	0.96036	0.98566	-0.02084	0.70547	0.97988	0.9781	-0.03194	0.051645	0.1484	1.2285	0.29687
373.725	2.89	olomics Po	0.40013	0.99951	1.2485	0.32018	0.47622	0.99998	0.86328	-0.2121	0.91158	0.99941	0.99502	-0.0072	0.16841	0.58103	0.73987	-0.43466	0.027042	0.10595	0.59642	-0.74559	0.11766	0.33359	0.70827	-0.49763	0.66387	0.99391	1.1117	0.15278	0.88657	0.99956	1.0728	0.10139	0.017637	0.2688	1.9464	0.96083
852.0827	9.22	olomics Po	0.40025	0.99951	1.2399	0.31021	0.34435	0.99998	0.83912	-0.25305	0.9135	0.99941	0.99699	-0.00436	0.091709	0.4063	0.67431	-0.56851	0.024314	0.09819	0.57156	-0.80702	0.031583	0.13695	0.62396	-0.68046	0.67459	0.99391	0.92068	-0.11923	0.42908	0.99956	0.84553	-0.24207	0.30613	0.58372	1.4124	0.49812
312.3268	7.56	olomics Po	0.40077	0.99951	1.0717	0.099863	0.082721	0.99998	0.81787	-0.29006	0.28171	0.99941	0.87464	-0.19324	0.098247	0.41868	0.78137	-0.35592	0.000862	0.008381	0.57936	-0.78747	0.000615	0.039708	0.54267	-0.88186	0.00982	0.24769	0.63032	-0.66585	0.011041	0.47452	0.7433	-0.42798	0.57667	0.79636	1.0601	0.084231
788.6069	5.4	domics Pos	0.40131	0.99987	1.0526	0.073973	0.42444	0.99994	0.95195	-0.07105	0.46173	0.99996	0.94981	-0.07428	0.27585	0.74071	0.90676	-0.14121	0.006777	0.022907	0.77008	-0.37692	0.42255	0.65844	0.94448	-0.08241	0.38377	0.76442	1.0958	0.13192	0.46571	0.96776	1.055	0.077246	0.004349	0.085451	1.2817	0.35808
1050.707	9.38	olomics Po	0.40154	0.99951	1.3661	0.45007	0.29151	0.99998	0.81858	-0.2888	0.48862	0.99941	1.0806	0.11184	0.067076	0.33969	0.72841	-0.45717	0.039374	0.14015	0.67312	-0.57107	0.036419	0.15075	0.69685	-0.52108	0.93863	0.9969	1.0592	0.082974	0.46789	0.99956	0.87372	-0.19475	0.06618	0.30735	1.7951	0.84406
1071.711	4.48	domics Pos	0.40205	0.99987	1.3068	0.38601	0.12475	0.99994	0.82664	-0.27466	0.77792	0.99996	0.96997	-0.04398	0.007038	0.19397	0.54826	-0.86706	0.006829	0.022971	0.52115	-0.94023	0.019513	0.12004	0.68131	-0.55363	0.59638	0.86481	0.93425	-0.09812	0.746	0.97988	0.98402	-0.02325	0.049621	0.14647	1.8308	0.87245
195.814	12.28	olomics Ne	0.40233	0.99918	0.97801	-0.03209	0.10158	0.51893	0.92119	-0.11842	0.61006	0.99995	1.0189	0.026984	0.80876	0.99985	0.99239	-0.01102	0.59846	0.99927	1.0341	0.048354	0.050728	0.48407	1.0827	0.11463	0.00902	0.25531	1.1283	0.17409	0.92594	0.99512	0.99845	-0.00224	0.73548	0.99941	1.0139	0.019854
729.925	3.21	olomics Po	0.40261	0.99951	1.2112	0.27644	0.14169	0.99998	0.77819	-0.36181	0.7062	0.99941	0.94535	-0.08108	0.007885	0.19613	0.63623	-0.65237	0.005647	0.03211	0.52479	-0.9302	0.011706	0.083709	0.63901	-0.6461	0.66634	0.99391	0.95774	-0.06229	0.38874	0.99956	0.85421	-0.22733	0.13929	0.39346	1.5677	0.64868
340.3576	4.16	domics Pos	0.40265	0.99987	0.92407	-0.11392	0.26532	0.99994	0.83949	-0.25241	0.15719	0.99996	0.84748	-0.23875	0.078988	0.47505	0.76431	-0.38777	0.002231	0.00981	0.68505	-0.54572	0.024852	0.13915	0.70053	-0.51349	0.081392	0.49998	0.72908	-0.45585	0.00529	0.52955	0.73269	-0.44873	0.6178	0.70997	0.95649	-0.06418
1003.762	0.53	domics Pos	0.40372	0.99987	0.92969	-0.10518	0.48961	0.99994	0.9409	-0.08788	0.36731	0.99996	0.9293	-0.10579	0.88634	0.9874	0.98726	-0.0185	0.04205	0.091541	0.80841	-0.30684	0.22446	0.51305	0.85342	-0.22867	0.77513	0.94518	0.9741	-0.03786	0.38781	0.96776	0.92996	-0.10476	0.29529	0.40589	1.1244	0.1691
312.3263	3.26	domics Pos	0.40455	0.99987	0.89321	-0.16293	0.11541	0.99994	0.70998	-0.49415	0.009394	0.99996	0.64198	-0.6394	0.10708	0.53121	0.78784	-0.34402	0.9589	0.96523	0.98717	-0.01863	0.007642	0.082308	0.63471	-0.65584	0.18331	0.64793	0.76619	-0.38422	0.003772	0.52955	0.57385	-0.80126	0.14273	0.24748	0.84388	-0.24489
1031.172	9.39	olomics Po	0.40474	0.99951	1.2356	0.30525	0.31543	0.99998	0.86291	-0.21271	0.66087	0.99941	1.0645	0.090183	0.047053	0.28918	0.70446	-0.50541	0.007745	0.03993	0.57733	-0.79253	0.021451	0.10949	0.67517	-0.56668	0.52293	0.99391	0.91166	-0.13343	0.57718	0.99956	0.92698	-0.10939	0.11842	0.36992	1.6834	0.75136
696.4357	2.28	domics Pos	0.40503	0.99987	1.1386	0.18721	0.70988	0.99994	0.9682	-0.04662	0.082534	0.99996	0.79922	-0.32334	0.033913	0.32166	0.59695	-0.74431	0.000753	0.004496	0.51557	-0.95576	0.022325	0.13091	0.44151	-1.1795	0.017854	0.33809	0.55659	-0.8453	0.007987	0.52955	0.46594	-1.1018	0.071871	0.17372	0.62078	-0.68784
363.2749	4.96	domics Pos	0.40508	0.99987	0.92992	-0.10482	0.24298	0.99994	0.91966	-0.12082	0.5693	0.99996	0.96078	-0.05772	0.19001	0.63224	0.87619	-0.19069	0.00474	0.017549	0.74597	-0.42281	0.031891	0.16106	0.80226	-0.31786	0.86399	0.97433	0.99212	-0.01141	0.73263	0.97988	0.98392	-0.02339	0.009757	0.094875	1.2459	0.31718
583.3248	7.27	olomics Po	0.40525	0.99951	0.90758	-0.1399	0.10554	0.99998	0.84774	-0.23831	0.76286	0.99941	0.96559	-0.05052	0.2728	0.7466	0.84404	-0.24462	0.000315	0.004004	0.49154	-1.0246	0.1088	0.31763	0.74127	-0.43193	0.56441	0.99391	0.92799	-0.10781	0.70001	0.99956	0.95447	-0.06724	0.98992	0.99365	1.0013	0.001901
1118.717	9.02	olomics Po	0.40552	0.99951	1.386	0.47089	0.41608	0.99998	0.88246	-0.18039	0.71238	0.99941	1.047	0.066311	0.01299	0.20085	0.68436	-0.54717	0.071757	0.21966	0.67557	-0.56583	0.085756	0.26905	0.78504	-0.34917	0.52274	0.99391	1.2375	0.30738	0.44237	0.99956	1.1417	0.19122	0.026529	0.2688	1.6241	0.69968
329.2485	6.32	olomics Po	0.40554	0.99951	0.85201	-0.23106	0.21366	0.99998	0.78686	-0.34583	0.01949	0.99941	0.57719	-0.79289	0.013807	0.20085	0.46927	-1.0915	0.009109	0.045043	0.4714	-1.085	0.001872	0.045425	0.28437	-1.8142	0.01955	0.30136	0.40955	-1.2879	0.004824	0.47452	0.36773	-1.4433	0.00645	0.2688	0.3642	-1.4572
151.146	6.97	olomics Po	0.40564	0.99951	1.1568	0.21014	0.58115	0.99998	0.89578	-0.15879	0.31432	0.99941	0.83778	-0.25536	0.14329	0.53536	0.70541	-0.50347	0.004768	0.028608	0.42706	-1.2275	0.0069	0.069223	0.49242	-1.022	0.004131	0.20152	0.50185	-0.99466	0.070396	0.76039	0.6146	-0.70228	0.15757	0.41468	0.71885	-0.47624
862.8019	10.03	domics Pos	0.40571	0.99987	0.91444	-0.12905	0.42585	0.99994	0.91581	-0.12689	0.1957	0.99996	0.8975	-0.15602	0.12901	0.55024	0.91681	-0.1253	0.002868	0.011847	0.81796	-0.28989	0.56126	0.75997	0.94436	-0.0826	0.052083	0.41166	0.88911	-0.16956	0.18635	0.92582	1.0906	0.12517	0.013048	0.098791	1.134	0.18147
907.609	5.78	domics Pos	0.40581	0.99987	0.93295	-0.10012	0.50744	0.99994	0.9322	-0.10129	0.15431	0.99996	0.85421	-0.22735	0.28609	0.75128	0.86333	-0.21202	0.029047	0.069222	0.6981	-0.51848	0.00384	0.06842	0.65783	-0.60421	0.052467	0.41166	0.76542	-0.38568	0.27086	0.94365	0.88934	-0.16919	0.81384	0.86295	1.0273	0.038905

867.8188	10.02	domics Pos	0.40592	0.99987	0.92792	-0.10792	0.94447	0.99994	1.0007	0.001041	0.071831	0.99996	0.8319	-0.26553	0.57428	0.91693	0.94411	-0.08297	0.017792	0.047609	0.74551	-0.4237	0.21746	0.50811	0.8455	-0.24213	0.53615	0.84392	0.94016	-0.08903	0.74709	0.97988	0.96673	-0.04881	0.031198	0.12145	1.2353	0.30491
964.1247	9.15	olomics Pc	0.40713	0.99951	1.2378	0.30775	0.60572	0.99998	0.86394	-0.211	0.54156	0.99941	1.1071	0.14679	0.26875	0.74055	0.73584	-0.44253	0.037427	0.13526	0.57956	-0.78697	0.3181	0.62354	0.75278	-0.40971	0.81782	0.99391	0.93483	-0.09723	0.47676	0.99956	0.82536	-0.2769	0.44596	0.71286	1.3092	0.38866
869.572	5.72	domics Pos	0.40718	0.99987	0.92421	-0.11371	0.22175	0.99994	0.87991	-0.18457	0.35677	0.99996	0.92106	-0.11864	0.5271	0.89941	0.92814	-0.10759	0.000856	0.004958	0.68075	-0.5548	0.33708	0.60864	0.90372	-0.14605	0.049803	0.40751	0.83732	-0.25615	0.33188	0.96136	1.0629	0.087962	0.057547	0.15492	1.2824	0.3588
318.8267	0.47	olomics Ne	0.40766	0.99918	1.1303	0.17675	0.33439	0.7498	1.1355	0.18329	0.49653	0.99995	1.1195	0.16288	0.090426	0.99985	1.2432	0.31403	0.023059	0.91567	1.6555	0.72726	0.1433	0.6202	1.4934	0.57864	0.052658	0.39335	1.405	0.49059	0.090063	0.99512	1.239	0.30915	0.25672	0.99941	1.2259	0.29384
964.6257	9.14	olomics Pc	0.40768	0.99951	1.2305	0.29929	0.57511	0.99998	0.86475	-0.20964	0.88619	0.99941	0.9488	-0.07583	0.30273	0.79203	0.77641	-0.36511	0.044026	0.1524	0.60914	-0.71515	0.23923	0.5292	0.71923	-0.47548	0.781	0.99391	0.92568	-0.11142	0.56948	0.99956	0.87159	-0.19827	0.45553	0.71854	1.2637	0.33763
1041.632	6.06	domics Pos	0.40906	0.99987	0.88335	-0.17894	0.13169	0.99994	0.747	-0.42082	0.25582	0.99996	0.83054	-0.26789	0.18861	0.63086	0.86973	-0.20136	0.005586	0.019849	0.68131	-0.55361	0.36302	0.6252	0.85267	-0.22994	0.80498	0.955	0.97303	-0.03945	0.14166	0.90033	0.77731	-0.36343	0.2054	0.31406	0.81054	-0.30304
1088.812	9.67	olomics Pc	0.40915	0.99951	1.2983	0.37666	0.35108	0.99998	0.8713	-0.19876	0.39472	0.99941	1.0921	0.12707	0.025213	0.23087	0.59962	-0.73788	0.011795	0.055372	0.60937	-0.71461	0.011485	0.083709	0.60459	-0.72597	0.69542	0.99391	0.94871	-0.07597	0.94407	0.99956	1.0535	0.075169	0.19456	0.45872	1.6037	0.68145
368.1028	0.6	lomics Neg	0.40933	0.99977	1.136	0.18395	0.60938	0.99904	1.2767	0.35242	0.55907	0.99994	1.4202	0.50611	0.59278	0.6924	1.1754	0.23318	0.6224	0.99989	1.127	0.17253	0.66695	0.99653	1.5169	0.60113	0.023086	0.30828	1.9311	0.94944	0.28075	0.64924	1.3016	0.38024	0.091614	0.405	1.5789	0.65891
609.5234	5.18	domics Pos	0.41012	0.99987	0.93614	-0.09521	0.20909	0.99994	0.86797	-0.20429	0.83182	0.99996	1.0548	0.076966	0.028485	0.29796	0.79891	-0.3239	0.003204	0.012914	0.68702	-0.54157	0.37268	0.63038	0.86645	-0.20682	0.39293	0.76945	1.1257	0.1708	0.03687	0.64014	0.7974	-0.32662	0.025983	0.11336	0.77191	-0.37349
332.3284	4.98	olomics Pc	0.41027	0.99951	0.79225	-0.33596	0.08631	0.99998	0.60684	-0.72062	0.98857	0.99941	1.0405	0.057275	0.35464	0.84597	0.79321	-0.33422	0.13403	0.36428	0.65322	-0.61435	0.14711	0.38942	0.65713	-0.60575	0.040358	0.39977	0.56731	-0.8178	0.036754	0.61591	0.51531	-0.95649	0.016063	0.2688	0.45502	-1.136
292.9453	10.18	domics Pos	0.41049	0.99987	1.1046	0.14351	0.62749	0.99994	0.97613	-0.03486	0.41131	0.99996	0.95312	-0.06926	0.038409	0.34165	0.93389	-0.09868	0.014296	0.040289	0.74131	-0.43185	0.99443	0.9964	1.0206	0.029396	0.58165	0.86163	0.96669	-0.04887	0.78031	0.97988	0.98577	-0.02067	0.010037	0.094875	1.1682	0.22431
500.9051	0.51	domics Pos	0.41069	0.99987	1.0791	0.10981	0.68462	0.99994	0.95163	-0.07152	0.44988	0.99996	0.91974	-0.12069	0.64149	0.95138	0.95049	-0.07326	0.045689	0.097374	0.79073	-0.33874	0.63653	0.80957	0.94074	-0.08813	0.71468	0.91919	0.95739	-0.06282	0.66516	0.97988	1.0436	0.061552	0.025219	0.11206	1.2862	0.36312
1004.792	9.82	olomics Pc	0.41079	0.99951	1.3753	0.45973	0.38625	0.99998	0.88887	-0.16996	0.4051	0.99941	1.0937	0.12921	0.017148	0.20936	0.62358	-0.68135	0.014817	0.06583	0.62754	-0.67223	0.029953	0.13218	0.68216	-0.55181	0.80214	0.99391	0.9796	-0.02974	0.80439	0.99956	1.1421	0.19165	0.044215	0.28476	2.0568	1.0404
813.6827	10.32	olomics Pc	0.41103	0.99951	0.93729	-0.09344	0.13231	0.99998	0.89797	-0.15526	0.097744	0.99941	0.89716	-0.15656	0.23344	0.68981	0.91083	-0.13475	0.80038	0.99688	0.98635	-0.01983	0.19556	0.47006	0.91939	-0.12126	0.13169	0.68208	0.90645	-0.1417	0.048688	0.67731	0.82414	-0.27903	0.017847	0.2688	0.83279	-0.26397
1007.143	8.87	olomics Pc	0.41108	0.99951	1.2423	0.31305	0.56492	0.99998	0.93037	-0.10412	0.45092	0.99941	1.0709	0.098792	0.042609	0.2802	0.76837	-0.38013	0.066223	0.20674	0.64165	-0.64014	0.064903	0.22174	0.77627	-0.36537	0.97488	0.9974	1.0399	0.056382	0.84588	0.99956	1.1094	0.1498	0.15082	0.40502	1.8397	0.8795
699.4845	2.17	domics Pos	0.41152	0.99987	1.1488	0.20019	0.97231	0.99994	0.95443	-0.06729	0.74499	0.99996	0.88832	-0.17084	0.118	0.53907	0.62454	-0.67914	6.16E-05	0.000744	0.091322	-3.4529	0.005828	0.076369	0.34822	-1.5219	0.22134	0.66456	0.69222	-0.53069	0.27123	0.94365	0.71248	-0.48908	0.66603	0.7498	1.0792	0.11
911.56	6.11	domics Pos	0.4116	0.99987	1.1068	0.14637	0.7781	0.99994	1.0385	0.054432	0.69104	0.99996	1.0492	0.069241	0.65205	0.95465	0.94673	-0.07898	0.074279	0.14063	0.77079	-0.3756	0.023081	0.13315	0.71734	-0.47927	0.039767	0.37532	0.75393	-0.4075	0.090477	0.82731	0.80538	-0.31226	0.7506	0.81329	1.0668	0.093252
634.2395	1.26	domics Pos	0.41185	0.99987	1.0661	0.092275	0.1199	0.99994	1.1006	0.1383	0.51558	0.99996	1.0383	0.054233	0.36078	0.8151	0.93571	-0.09587	0.014789	0.041494	0.77003	-0.37702	0.17771	0.46538	0.89543	-0.15935	0.58994	0.86163	1.0581	0.081466	0.52615	0.96776	0.95125	-0.0721	0.10418	0.20689	1.173	0.23016
1075.201	4.46	domics Pos	0.41193	0.99987	1.3094	0.38886	0.29362	0.99994	0.84984	-0.23473	0.34114	0.99996	1.1462	0.19685	0.18633	0.62584	0.76852	-0.37985	0.007939	0.025807	0.54787	-0.86808	0.061308	0.24764	0.67667	-0.56348	0.83851	0.96659	0.97864	-0.03114	0.74727	0.97988	1.1002	0.13783	0.048063	0.14468	1.7237	0.78547
895.1015	8.93	olomics Pc	0.41276	0.99951	1.2768	0.35253	0.38185	0.99998	0.87441	-0.19362	0.60243	0.99941	0.93523	-0.0966	0.053596	0.29934	0.6962	-0.52242	0.006646	0.035784	0.62009	-0.68944	0.020453	0.10649	0.64502	-0.63259	0.81558	0.99391	1.1348	0.18244	0.75666	0.99956	1.1134	0.15496	0.16308	0.42212	1.7542	0.81079
906.6212	6.08	domics Pos	0.41284	0.99987	0.90706	-0.14074	0.24395	0.99994	0.90724	-0.14044	0.11377	0.99996	0.84756	-0.23862	0.13433	0.55796	0.85686	-0.22288	0.036568	0.082408	0.75834	-0.39909	0.16868	0.4555	0.87394	-0.19439	0.24158	0.67954	1.0962	0.13248	0.69581	0.97988	0.97	-0.04394	0.2084	0.31696	1.1806	0.23958
95.0868	6.98	olomics Pc	0.41391	0.99951	1.1756	0.23336	0.53185	0.99998	0.88933	-0.16921	0.48286	0.99941	0.88674	-0.17341	0.10036	0.42377	0.66917	-0.57956	0.000376	0.004609	0.34263	-1.5453	0.002198	0.045856	0.45294	-1.1426	0.006038	0.21836	0.53175	-0.91117	0.062631	0.72831	0.61154	-0.70947	0.2169	0.48424	0.79195	-0.33652
1035.766	10.05	domics Pos	0.4148	0.99987	1.1081	0.14813	0.56012	0.99994	0.91888	-0.12205	0.9833	0.99996	0.99376	-0.00902	0.55237	0.90826	0.91725	-0.12461	0.049498	0.10363	0.74147	-0.43153	0.64494	0.81443	0.93385	-0.09874	0.45161	0.80204	1.1017	0.1397	0.60311	0.97073	1.0578	0.081109	0.17275	0.28042	1.2209	0.28794
447.8989	10.08	domics Pos	0.41544	0.99987	1.1019	0.13994	0.31808	0.99994	0.93315	-0.09983	0.35996	0.99996	0.93913	-0.0906	0.066582	0.43847	0.82832	-0.27174	0.01496	0.04176	0.7367	-0.44085	0.5404	0.74414	0.93963	-0.08984	0.038604	0.37121	1.1622	0.21685	0.78619	0.97988	1.0295	0.041953	0.027981	0.11681	1.2499	0.32186
174.9459	10.11	domics Pos	0.4157	0.99987	1.133	0.18018	0.50465	0.99994	0.9425	-0.08543	0.95434	0.99996	1.0175	0.024978	0.12092	0.54142	0.87441	-0.19362	0.00512	0.018521	0.6291	-0.66864	0.23908	0.52848	0.89704	-0.15676	0.15218	0.62225	0.89689	-0.157	0.2092	0.92646	0.8935	-0.16246	0.1103	0.21382	1.1238	0.16833
350.8936	10.22	domics Pos	0.41573	0.99987	1.0496	0.06982	0.060927	0.99994	0.8525	-0.23023	0.2259	0.99996	0.87239	-0.19696	0.13303	0.55741	0.84838	-0.23721	0.003383	0.01345	0.76198	-0.39218	0.12349	0.38281	0.82655	-0.27482	0.88974	0.97532	0.99039	-0.01393	0.27478	0.94365	0.90558	-0.14308	0.86166	0.89915	1.0124	0.017734
458.3048	3.52	domics Pos	0.41599	0.99987	0.88825	-0.17097	0.47449	0.99994	0.88681	-0.1733	0.022938	0.99996	0.72167	-0.47058	0.001375	0.17581	0.49755	-1.0071	0.000257	0.001986	0.42629	-1.2301	0.003739	0.067932	0.3729	-1.4231	0.008679	0.32129	0.49813	-1.0054	0.009113	0.52955	0.4585	-1.125	0.087386	0.18803	0.62749	-0.67234
235.1996	1.22	domics Pos	0.416	0.99987	1.1509	0.20281	0.32637	0.99994	0.86706	-0.20579	0.69624	0.99996	1.0332	0.047123	0.16212	0.59299	0.78247	-0.35388	0.000174	0.001531	0.31165	-1.682	0.008298	0.084185	0.59008	-0.76102	0.258	0.69181	0.81977	-0.28671	0.22133	0.94047	0.81953	-0.28713	0.6655	0.74942	1.0613	0.085866
711.0008	9.45	olomics Pc	0.41752	0.99951	1.2433	0.31417	0.22211	0.99998	0.83512	-0.25995	0.92507	0.99941	0.9788	-0.03091	0.043587	0.2827	0.68587	-0.544	0.009624	0.047149	0.60198	-0.73222	0.005111	0.061592	0.57069	-0.80922	0.54012	0.99391	0.90163	-0.14939	0.55133	0.99956	0.9137	-0.13021	0.099334	0.35294	1.6947	0.76106
1178.773	10.02	domics Pos	0.41801	0.99987	1.0385	0.054538	0.1736	0.99994	0.92271	-0.11606	0.44489	0.99996	0.95771	-0.06234	0.22084	0.67472	0.93166	-0.10212	0.001297	0.006663	0.77689	-0.36423	0.34081	0.61059	0.91745	-0.1243	0.79363	0.95091	1.0214	0.030538	0.80515	0.97988	1.0158	0.022674	0.020889	0.10765	1.217	0.28336
688.9883	9.48	olomics Pc	0.4185	0.99951	1.2316	0.3005	0.059142	0.99998	0.79316	-0.33432	0.71085	0.99941	1.0343	0.048589	0.051185	0.29547	0.64703	-0.62809	0.001991	0.015411	0.55184	-0.85768	0.008001	0.074341	0.63068	-0.66502	0.53598	0.99391	0.9074	-0.1402	0.84769	0.99956	1.0255	0.036388	0.10785	0.36185	1.7124	0.77602
240.9463	10.14	domics Pos	0.41864	0.99987	0.91775	-0.12382	0.51881	0.99994	1.0653	0.091296	0.45679	0.99996	0.94053	-0.08845	0.46658	0.8838	0.94668	-0.07904	0.048142	0.10142	0.78466	-0.34986	0.90323	0.95006	1.0022	0.003183	0.97325	0.99324	1.005	0.007261	0.55672	0.96798	0.9334	-0.09943	0.011118	0.094875	1.2202	0.28707
197.8106	12.27	olomics Ne	0.41865	0.99918	0.98093	-0.02777	0.025916	0.40327	0.91382	-0.13002	0.94991	0.99995	0.99962	-0.00055	0.95219	0.99985	1.0032	0.004597	0.75518	0.99927	1.0234	0.033371	0.13433	0.6057	1.0683	0.095275	0.004	0.22316	1.1487	0.2	0.74443	0.99512	0.987	-0.01888	0.83443	0.99941	1.0092	0.013206
1139.814	5.16	domics Pos	0.41931	0.99987	1.4304	0.51641	0.54248	0.99994	0.90866	-0.13819	0.21215	0.99996	1.1771	0.23527	0.009188	0.2002	0.55329	-0.8539	0.005894	0.020659	0.50968	-0.97234	0.03659	0.17398	0.70304	-0.50832	0.6251	0.87671	1.1853	0.2453	0.77546	0.97988	0.98021	-0.02883	0.047738	0.14458	1.9705	0.97856
1165.761	10.02	domics Pos	0.41942	0.99987	1.0666	0.092998	0.032152	0.99994	0.8335	-0.26274	0.1301	0.99996	0.87234	-0.19704	0.17043	0.60402	0.87441	-0.19362	0.000534	0.003465	0.65911	-0.6014	0.41052	0.65377	0.91806	-0.12334	0.6102	0.87064	1.075	0.10433	0.49386	0.96776	0.94147	-0.08702	0.50013	0.60202	1.0688	0.095964
162.9843	12.14	olomics Pc	0.42046	0.99951	1.063	0.088145	0.96217	0.99998	1.0096	0.013795	0.41879	0.99941	0.93303	-0.10001	0.80843	0.99905	0.98147	-0.02698	0.4136	0.8153	0.93178	-0.10193	0.19868	0.47197	0.87964	-0.18502	0.49962	0.99391	0.94888	-0.07571	0.024878	0.52244	0.78954	-0.34091	0.93501	0.97226	0.9937	-0.00912
669.4621	9.52	olomics Pc	0.42054	0.99951	1.3019	0.38059	0.38748	0.99998	0.87922	-0.18571	0.99418	0.99941	0.99121	-0.01273	0.010561	0.19754	0.6474	-0.62726	0.00056	0.006154	0.53136	-0.91224	0.026025	0.12085	0.68836	-0.53876	0.84467	0.9951	1.0417	0.058889	0.85001	0.99956	1.1821	0.24136	0.13805	0.39227	1.629	0.70394
650.8487	0.5	domics Pos	0.42059	0.99987	1.2112	0.27648	0.76262	0.99994	0.96901	-0.04541	0.64462	0.99996	0.9542	-0.06763	0.53233	0.90063	0.93477	-0.09731	0.026234	0.064203	0.78505	-0.34914	0.97259	0.98473	1.0062	0.008889	0.36105	0.75755	1.0845	0.11701	0.92547	0.9857	1.0147	0.021	0.034208	0.12615	1.2204	0.28733
151.0421	0.31	olomics Pc	0.42143	0.99951	1.1317	0.17852	0.089873	0.99998	1.166	0.22157	0.1066	0.99941	1.1889	0.24963	0.03937	0.27316	1.457	0.54303	0.054213	0.17849	1.3347	0.41648	0.079178	0.25424	1.3842	0.46904	0.095429	0.60726	1.338	0.42003	0.1975	0.99538	1.3462	0.42894	0.069465	0.3118	1.264	0.33802
414.3011	4.19	olomics Pc	0.42164	0.99951	0.93127	-0.10273	0.26087	0.99998	1.1654	0.2208	0.12108	0.99941	2.6707	1.4172	0.052143	0.29864	5.5633	2.476	0.008997	0.04456	8.2177	3.0387	0.011679	0.083709	7.8251	2.9681	0.007273	0.22462	9.9102	3.3089	0.45452	0.99956	1.421	0.50686	0.58696	0.80358	0.7244	-0.46514
226.9524	0.5	domics Pos	0.42195	0.99987	1.0243	0.034621	0.1448	0.99994	0.93166	-0.10212	0.18765	0.99996	0.93699	-0.09389	0.038246	0.34165	0.90249	-0.14802	7.48E-05	0.000852	0.74816	-0.41858	0.24137	0.52923	0.8853	-0.17575	0.49095	0.82123	0.96042	-0.05826	0.23111	0.94365	0.94001	-0.08926	0.037399	0.13071	1.1818	0.24095
385.3456	4	domics Pos	0.42231	0.99987	0.92102	-0.11869	0.009935	0.99994	0.79329	-0.33408	0.47444	0.99996	0.93529	-0.09652	0.085403	0.48968	0.86938	-0.20195	0.001488	0.007308	0.7005	-0.51354	0.018228	0.11584	0.73053	-0.45298	0.13874	0.60403	0.8262	-0.27544	0.11877	0.88537	0.87555	-0.19174	0.99855	0.99881	1.0036	0.005198
909.6469	4.74	domics Pos	0.42262	0.99987	1.112	0.15313	0.94333	0.99994	1.013	0.018649	0.68837	0.99996	1.0401	0.056762	0.67132	0.95717	0.95991	-0.05902	0.003902	0.015015	0.74845	-0.41802	0.079506	0.29123	0.79542	-0.3302	0.32083	0.73067	0.87794	-0.18781	0.81603	0.97988	1.0575	0.080721	0.085076	0.18611	1.2937	0.37155
221.1556	2.19	domics Pos	0.42323	0.99987	0.87802	-0.18768	0.33102	0.99994	0.86854	-0.20334	0.03681	0.99996	0.66405	-0.59064	0.011232	0.21625	0.6677	-0.58273	9.75E-06	0.00022	0.22071	-2.1797	0.00257	0.061655	0.52088	-0.94098	0.01339	0.33809	0.68066	-0.555	0.098686	0.84941	0.67	-0.57778	0.12741	0.23222	0.7246	-0.46474
626.8457	10.05	domics Pos	0.42386	0.99987	1.0463	0.065276	0.2939	0.99994	0.92434	-0.1135	0.40945	0.99996	0.94624	-0.07972	0.2587	0.71687	0.92928	-0.10581	0.004081	0.015513	0.79189	-0.33663	0.35141	0.61843	0.915	-0.12815	0.2718	0.70502	1.0729	0.10147	0.81918	0.97988	0.98654	-0.01955	0.053754	0.1506	1.1691	0.22539
984.6268	8.88	olomics Pc	0.42427	0.99951	1.2963	0.3744	0.89597	0.99998	1.0446	0.0629	0.65007	0.99941	1.0915	0.12629	0.017135	0.20936	0.75971	-0.39648	0.051874	0.17347	0.69819	-0.51831	0.18423	0.45303	0.82487	-0.27776	0.61068	0.99391	1.1328	0.17989	0.63985	0.99956	1.0997	0.13712	0.094415	0.34866	1.783	0.83427
291.0708	0.6	domics Pos	0.42504	0.99987	0.75873	-0.39835	0.76965	0.99994	0.89767	-0.15575	0.8991	0.99996	0.9523	-0.07051	0.63036	0.94708	0.84635	-0.24067	0.007061	0.023513	0.13254	-2.9155	0.94129	0.96872	0.98915	-0.01575	0.88931	0.97532	1.0268	0.038221	0.91434	0.98248	1.0169	0.024238	0.6526	0.73926	1.0956	0.13174
779.0469	4.94	domics Pos	0.42511	0.99987	0.92438	-0.11344	0.46503	0.99994	0.92722	-0.10902	0.038136	0.99996	0.86792	-0.20437	0.15882	0.5885	0.80172	-0.31882	0.002578	0.010933	0.64135	-0.64083	0.48582	0.705	0.95213	-0.07077	0.72789	0.92608	0.97972	-0.02955	0.43031	0.96776	0.94805	-0.07696	0.014195	0.098791	1.2949	0.37286
1189.636	6.78	olomics Pc	0.42523	0.99951	1.2093	0.27414	0.13858	0.99998	0.68377	-0.54841	0.22649	0.99941	0.73234	-0.44942	0.033159	0.25203	0.49375	-1.0182	0.023192	0.094387	0.49102	-1.0261	0.021747	0.10951	0.51645	-0.9533	0.58971	0.99391	0.89499	-0.16006	0.29859	0.99956	0.76097	-0.39409	0.061684	0.30404	1.5297	0.61325
1082.71	10.09	domics Pos	0.4262	0.99987	0.92758	-0.10846	0.15969	0.99994	0.87635	-0.19042	0.28992	0.99996	0.92025	-0.11991	0.099308	0.51973	0.8465	-0.24041	0.006766	0.022893	0.7583	-0.39917	0.31312	0.59223	0.86974	-0.20134	0.8306	0.96528	0.97985	-0.02937	0.44282	0.96776	0.92721	-0.10903	0.28822	0.39833	1.0809	0.1123
377.1706	2.29	domics Pos	0.4269	0.99987	1.0659	0.092092	0.60815	0.99994	1.0705	0.098329	0.17034	0.99996	0.84839	-0.23721	0.020154	0.25894	0.76602	-0.38455	0.00098	0.005467	0.58742	-0.76753	0.063996	0.25399	0.66115	-0.59694	0.0455	0.39255	0.7538	-0.40774	0.050792	0.68198	0.64876	-0.62424	0.32643	0.43673	0.85367	-0.22824
896.797	0.53	domics Pos	0.42757	0.99987	1.0678	0.094585	0.21021	0.99994	0.89731	-0.15632	0.64402	0.99996	0.96322	-0.05407	0.11702	0.53727	0.88831	-0.17087	0.003319	0.013224	0.72874	-0.45652	0.31111	0.58959	0.85985	-0.21785	0.76102	0.94108	0.97586	-0.03525	0.41741	0.96776	0.92459	-0.11312	0.16818	0.27546	1.1988	0.26163
1007.607	6.47	olomics Pc	0.42773	0.99951	1.2513	0.32341	0.55713	0.99998	0.87465	-0.19322	0.52659	0.99941	1.1196	0.16296	0.043268	0.2816	0.64095	-0.64171	0.36753	0.76266	0.81026	-0.30355	0.068506	0.23147	0.6635	-0.59184	0.65042	0.99391	0.90114	-0.15018	0.72097	0.99956	1.0053	0.00759	0.97948	0.98952	0.97181	-0.04126
766.7826	10.12	domics Pos	0.42847	0.99987	1.0989	0.13608	0.30855	0.99994	0.92633	-0.1104	0.68799	0.99996	1.0383	0.054187	0.61917	0.93987	1.0358	0.05072	0.013125	0.037714	0.80707	-0.30924	0.60008	0.78333	1.0448	0.063181	0.030196	0.35536	1.2442	0.31519	0.1479	0.91175	1.1191	0.16238	0.096928	0.19787	1.2557	0.32853
914.1358	8.92	olomics Pc	0.4287	0.99951	1.3335	0.41523	0.46263	0.99998	0.89729	-0.15635	0.21205	0.99941	1.1878	0.24824	0.053594	0.29934	0.75241	-0.4104	0.005686	0.032212	0.56508	-0.82348	0.18862	0.46022	0.8249	-0.27771	0.56346	0.99391	1.1336	0.18092	0.58223	0.99956	1.219	0.2857	0.096858	0.3499	1.6345	0.70887
399.3164	1.67	domics Pos	0.42975	0.99987	1.1011	0.13898	0.38275	0.99994	0.90977	-0.13643	0.62048	0.99996	0.95602	-0.06488	0.15126	0.57987	0.88077	-0.18316	0.00984	0.03038	0.717	-0.47995	0.052658	0.22542	0.6937	-0.52762	0.026335	0.34388	0.71799	-0.47797	0.12799	0.88537	0.78265	-0.35357	0.85657	0.89531	0.99268	-0.0106
424.3423	4.87	olomics Pc	0.43013	0.99951	0.85315	-0.22913	0.50075	0.99998	0.86177	-0.21462	0.33303	0.99941	0.8251	-0.27737	0.07472	0.35544	0.69753	-0.51966	0.043247	0.15036	0.49107	-1.026	0.017798	0.098187	0.41039	-1.2849	0.086248	0.60175	0.51154	-0.96708	0.036759	0.61591	0.50141	-0.99594	0.007373	0.2688	0.4511	-1.1485
671.219	1.83	domics Pos	0.43021	0.99987	0.93807	-0.09223	0.22393	0.99994	0.87781	-0.18803	0.25541	0.99996	0.86575	-0.20797	0.27786	0.74448	0.8904	-0.16747	0.065194	0.12683	0.71487	-0.48426	0.011516	0.09183	0.76386	-0.38862	0.44622	0.80121	0.93959	-0.08989	0.75769	0.97988	0.97455	-0.03719	0.13349	0.23832	1.1068	0.14637
960.1363	8.89	olomics Pc	0.43021	0.99951	1.2662	0.34045	0.30421	0.99998	0.87133	-0.19871	0.91282	0.99941	1.0247	0.035233	0.025797	0.23418	0.75925	-0.39735	0.020066	0.084163	0.61625	-0.69842	0.019184	0.10208	0.74061	-0.43321	0.86638	0.9951	1.0861	0.11912	0.91457	0.99956	1.0396	0.05606	0.13931	0.39346	1.6591	0.73043
262.9157	10.15	domics Pos	0.43037	0.99987	0.94537	-0.08106	0.28314	0.99994	0.91044	-0.13536	0.32441	0.99996	0.94789	-0.0772	0.10783	0.53121	0.88861	-0.17037	0.001967	0.008967	0.73761	-0.43908	0.14671	0.42188	0.86192	-0.21437	0.31829	0.72948	0.93802	-0.09231	0.19183	0.92582	0.92399	-0.11405	0.36256	0.47118	1.0607	0.085083
705.5808	4.66	domics Pos	0.43078	0.99987	1.0569	0.079856	0.30707	0.99994	0.89988	-0.1522	0.83455	0.99996	0.98303	-0.0247	0.57204	0.9155	0.94754	-0.07774	0.031501	0.073553	0.79025	-0.33961	0.29187	0.57461	0.87522	-0.19228	0.93419	0.98559	0.99578	-0.0061	0.1856	0.92582	0.87302	-0.19592	0.95386	0.96602	0.99202	-0.01156
562.3008	5.11	olomics Pc	0.43081	0.99951	0.8706	-0.19992	0.43022	0.99998	0.8773	-0.18885	0.84556	0.99941	0.97529	-0.0361	0.90555	0.99905	1.0377	0.053391	0.43807	0.84503	1.1715	0.2284	0.05962	0.21074	1.3044	0.38342	0.039754	0.39754	1.4095	0.49518	0.74891	0.99956	0.95111	-0.07231	0.91908	0.96508	1.0053	0.007583
606.3701	3.05	olomics Pc	0.43196	0.99951	1.2019	0.26535	0.003433	0.99998	0.67916	-0.55817	0.13471	0.99941	0.82816	-0.27203	0.000935	0.12936	0.57995	-0.78599	0.002207	0.016708	0.49794	-1.006	0.002566	0.046402	0.59472	-0.74972	0.28928	0.90579	0.82737	-0.27339	0.20928	0.99956	0.85302	-0.22935	0.010311	0.2688	1.6788	0.74745
895.7166	7.74	domics Pos	0.4321	0.99987	0.81134	-0.30163	0.35144	0.99994	0.79461	-0.33168	0.21589	0.99996	0.70306	-0.50827	0.2605	0.71926	0.70177	-0.51093	0.02856	0.068355	0.49434	-1.0164	0.17231	0.45896	0.69767	-0.51938	0.5819	0.86163	0.86547	-0.20845	0.23902	0.94365	0.75636	-0.40285	0.17927	0.28645	1.3384	0.42053
291.0694	0.35	olomics Pc	0.43273	0.99951	0.7085	-0.49715	0.90023	0.99998	0.97787	-0.03229	0.97644	0.99941	0.94367	-0.08365	0.7339	0.99905	0.82023	-0.28591	0.013476	0.060992	0.15678	-2.6732	0.70267	0.9111	1.0335	0.047521	0.96217	0.9974	0.9668	-0.04871	0.99576	0.99956	0.98948	-0.01525	0.92285	0.96646	0.86761	-0.20488
558.6139	0.33	olomics Ne	0.43293	0.99918	0.94476	-0.08198	0.19514	0.61289	0.89685	-0.15706	0.62984	0.99995	1.0362	0.051239	0.42767	0.99985	0.94219	-0.08592	0.46484	0.99927	0.9384	-0.09172	0.96908	0.98999	1.021	0.030025	0.54689	0.84682	1.038	0.053835	0.02732	0.99512	0.85259	-0.23008	0.72998	0.99941	0.98019	-0.02887
822.777	0.56	domics Pos	0.43307	0.99987	0.94474	-0.08202	0.26026	0.99994	0.88132	-0.18226	0.50262	0.99996	0.9414	-0.08713	0.051923	0.3905	0.83262	-0.26426	0.008535	0.027164	0.73286	-0.4484	0.49116	0.70939	0.92907	-0.10614	0.16306	0.63418	0.88564	-0.1752	0.20753	0.9264	0.89139	-0.16588	0.15048	0.25581	1.1121	0.15332
694.4419	4.52	domics Pos	0.43343	0.99987	1.3206	0.40119	0.14334	0.99994	0.81558	-0.29409	0.65244	0.99996	1.0423	0.059837	0.01246	0.22648	0.60019	-0.73651	0.002999	0.012257	0.46627	-1.1008	0.006815	0.080671	0.62064	-0.68817	0.46674	0.80647	0.87898	-0.1861	0.83647	0.97988	1.0352	0.049891	0.089664	0.19023	1.7494	0.80685
550.6288	4.33	domics Pos	0.43355	0.99987	0.89048	-0.16735	0.10443	0.99994	0.72952	-0.45498	0.4826	0.99996	0.90356	-0.14631	0.30712	0.76589	0.86458	-0.20992	0.018146	0.048384	0.68606	-0.54359	0.10188	0.33955	0.66437	-0.58995	0.81035	0.9575	1.0838	0.11616	0.37443	0.96776	0.87725	-0.18894	0.94529	0.95963	1.0846	0.1172
604.4343	9.65	olomics Pc	0.43402	0.99951	1.2056	0.26973	0.35766	0.99998	0.87018	-0.20062	0.80947	0.99941	0.96198	-0.05592	0.02155	0.22258	0.65443	-0.61169	0.004627	0.028083	0.62515	-0.67772	0.043421	0.1697	0.70487	-0.50457	0.7845	0.99391	0.98529	-0.02138	0.94098	0.99956	1.0414	0.058512	0.1266	0.37656	1.5895	0.6686
762.5996	5.67	domics Pos	0.43404	0.99987	0.9478	-0.07734	0.13462	0.99994	0.84102	-0.2498	0.3146	0.99996	0.90164	-0.14937	0.1171	0.53727	0.85316	-0.22911	0.01161	0.034397	0.76563	-0.38529	0.92004	0.95771	0.99311	-0.00998	0.16066	0.63417	1.2371	0.30695	0.63422	0.97988	1.0479	0.067435	0.026571	0.11491	1.3271	0.40828
158.9653	0.51	domics Pos	0.43449	0.99987	1.0249	0.035537	0.1359	0.99994	0.93024	-0.10433	0.18483	0.99996	0.93639	-0.09483	0.048751	0.37859	0.9031	-0.14704	8.34E-05	0.000909	0.74629	-0.42219	0.21614	0.50811	0.88143	-0.18208	0.40659	0.77758	0.95312	-0.06927	0.20233	0.92582	0.93729	-0.09344	0.040859	0.13494	1.1808	0.23972
330.2515	6.32	olomics Pc	0.43514	0.99951	0.85593	-0.22443	0.27612	0.99998	0.79829	-0.32502	0.018185	0.99941	0.55861	-0.84007	0.020918	0.22258	0.48003	-1.0588	0.009289	0.045688	0.48183	-1.0534	0.002335	0.045971	0.27575	-1.8586	0.019906	0.30146	0.41194	-1.2795	0.004997	0.47452	0.33823	-1.5639	0.007281	0.2688	0.36334	-1.4606
426.2961	2.28	domics Pos	0.43558	0.99987	0.9045	-0.1448	0.17817	0.99994	0.85156	-0.23182	0.009936	0.99996	0.76602	-0.38455	0.005901	0.18075	0.62204	-0.68493	3.75E-05	0.00053	0.44377	-1.1721	0.030449	0.15584	0.52757	-0.92256	0.007991	0.32129	0.54786	-0.86813	0.012459	0.52955	0.52651	-0.92546	0.013291	0.098791	0.63718	-0.65022
290.9046	10.16	domics Pos	0.43632	0.99987	0.94344	-0.084	0.032195	0.99994	0.83803	-0.25493	0.41667	0.99996	0.93285	-0.10028	0.45469	0.87408	0.95056	-0.07315	0.016333	0.044516	0.77422	-0.36919	0.22151	0.51169	0.87912	-0.18586	0.45226	0.80204	1.0507	0.071417	0.21382	0.93471	0.9118	-0.13322	0.083472	0.18515	1.1634	0.21829
508.2849	5.13	domics Pos	0.43638	0.99987	1.0999	0.13735	0.62846	0.99994	1.0499	0.070191	0.2983	0.99996	1.0919	0.12678	0.83447	0.98621	0.97933	-0.03013	0.031143	0.072941	0.81456	-0.2959	0.85867	0.92844	0.98526	-0.02143	0.11363	0.56565	1.2419	0.3125	0.32586	0.95578	1.0942	0.12981	0.014228	0.098791	1.3787	0.46326
200.6368	6.01	olomics Pc	0.4368	0.99951	1.0873	0.12079	0.12865	0.99998	0.82963	-0.26946	0.97252	0.99941	1.0072	0.010322	0.18941	0.62215	0.81957	-0.28705	1.49E-05	0.00041	0.28464	-1.8128	0.013437	0.086062	0.64849	-0.62484	0.079194	0.57359	0.75395	-0.40745	0.30344	0.99956	0.91537	-0.12757	0.58623	0.80358	1.0455	0.064212
442.6794	0.57	lomics Neg	0.43697	0.99977	0.93783	-0.09259	0.86646	0.99904	0.9991	-0.0013	0.77884	0.99994	0.97961	-0.02971	0.47669	0.63274	1.0834	0.11553	0.19654	0.99989	0.91397	-0.12979	0.45031	0.99653	0.93634	-0.09489	0.004425	0.10075	0.69581	-0.52323	0.056851	0.46971	0.81989	-0.2865	0.055996	0.405	0.72268	-0.46858
531.0101	0.5	domics Pos	0.43714	0.99987	0.885	-0.17626	0.53052	0.99994	0.91078	-0.13482	0.2545	0.99996	1.2589	0.33216	0.086186	0.49062	1.5447	0.62731	0.69001	0.73739	1.0887	0.12262	0.33824	0.60941	1.1778	0.23608	0.033339	0.36346	1.579	0.65897	0.20971	0.92655	1.1957	0.25787	0.13451	0.23955	1.3622	0.44592
112.1101	2.05	domics Pos	0.43755	0.99987	1.1303	0.17675	0.54966	0.99994	0.88817	-0.17109	0.54427	0.99996	1.0732	0.10187	0.48674	0.88725	0.86264	-0.21316	6.03E-05	0.000734	0.23061	-2.1165	0.042316	0.19323	0.58013	-0.78556	0.25533	0.69047	0.783	-0.35292	0.64359	0.97988	1.0442	0.062444	0.094538	0.19546	1.297	0.37519
1049.698	9.06	olomics Pc	0.43755	0.99951	1.2103	0.27534	0.34003	0.99998	0.85442	-0.22698	0.93229	0.99941	0.99049	-0.01379	0.085797	0.39012	0.73965	-0.43509	0.023138	0.094353	0.61123	-0.71022	0.15488	0.40421	0.80595	-0.31124	0.92747	0.9951	1.0557	0.078145	0.98683	0.99956	1.0352	0.049889	0.38506	0.65754	1.2816	0.35799
757.9772	12.04	olomics Ne	0.43818	0.99918	1.1251	0.17003	0.18828	0.60833	1.2006	0.26378	0.55802	0.99995	0.93614	-0.09521	0.8316	0.99985	0.97744	-0.03292	0.36969	0.99927	1.2232	0.29067	0.064575	0.51001	1.3522	0.43527	0.035245	0.35618	1.4029	0.48844	0.079224	0.99512	1.3828	0.46759	0.85963	0.99941	1.0522	0.07339
219.2108	4.73	domics Pos	0.43829	0.99987	1.055	0.077297	0.42708	0.99994	0.92659	-0.10999	0.94694	0.99996	0.99296	-0.01019	0.31945	0.78383	0.88906	-0.16965	0.017194	0.046309	0.75271	-0.40983	0.56278	0.76119	0.95183	-0.07123	0.90187	0.97745	0.99143	-0.01242	0.65983	0.97988	1.0487	0.068569	0.03256	0.12327	1.2732	0.34851
335.1408	6.06	olomics Pc	0.43844	0.99951	1.1005	0.13821	0.9149	0.99998	1.0614	0.086014	0.050593	0.99941	0.80731	-0.30881	0.069498	0.3437	0.74648	-0.42182	0.045748	0.15724	0.77962	-0.35916	0.01811	0.099386	0.72936	-0.4553	0.008823	0.23958	0.71216	-0.48972	0.013922	0.47452	0.65284	-0.6152	0.038325	0.28207	0.66356	-0.59171
623.317	7.27	olomics Pc	0.43846	0.99951	0.92995	-0.10478	0.86223	0.99998	1.0047	0.00671	0.94873	0.99941	0.99344	-0.00949	0.19511	0.62875	0.88153	-0.18192	0.003841	0.024397	0.45662	-1.1309	0.18042	0.44609	0.83242	-0.26462	0.30669	0.92447	0.87266	-0.19651	0.63449	0.99956	0.95776	-0.06226	0.43871	0.70796	0.94727	-0.07816
914.782	10.08	domics Pos	0.43861	0.99987	1.0359	0.05091	0.5255	0.99994	0.95892	-0.06052	0.3724	0.99996	0.92774	-0.10821	0.77524	0.97526	1.0265	0.03768	0.035881	0.081083	0.84766	-0.23845	0.75361	0.87187	0.98547	-0.02111	0.085158	0.50896	1.1505	0.20223	0.13249	0.88792	0.91762	-0.12403	0.005564	0.08815	1.1865	0.24668
298.3106	2.84	domics Pos	0.43909	0.99987	0.93667	-0.0944	0.17355	0.99994	0.82544	-0.27677	0.28008	0.99996	0.85231	-0.23054	0.05831	0.41721	0.8455	-0.24212	0.01664	0.04511	0.79426	-0.33231	0.005291	0.074232	0.63762	-0.64923	0.13532	0.60006	0.75371	-0.40792	0.005225	0.52955	0.75869	-0.39842	0.91673	0.93763	0.99341	-0.00954
1096.246	6.26	domics Pos	0.43931	0.99987	1.235	0.30452	0.19199	0.99994	0.8141	-0.29672	0.94537	0.99996	1.0053	0.007576	0.046107	0.3709	0.6334	-0.65882	0.000465	0.003119	0.38443	-1.3792	0.004339	0.071311	0.47325	-1.0793	0.72353	0.9233	0.95053	-0.0732	0.82464	0.97988	1.0048	0.006884	0.19576	0.30404	1.7576	0.81362
815.6929	10.35	olomics Pc	0.43932	0.99951	0.9306	-0.10377	0.045624	0.99998	0.878	-0.18771	0.049277	0.99941	0.86845	-0.20349	0.2756	0.74848	0.89223	-0.16451	0.49638	0.89759	0.95814	-0.06169	0.21091	0.48995	0.90061	-0.15102	0.088994	0.60275	0.90688	-0.14101	0.06312	0.72831	0.82993	-0.26894	0.007169	0.2688	0.82792	-0.27244
217.8622	12.72	olomics Ne	0.43934	0.99918	1.0271	0.038605	0.007187	0.34605	0.83609	-0.25827	0.23276	0.99995	0.94288	-0.08485	0.71321	0.99985	0.98084	-0.02792	0.38423	0.99927	0.94446	-0.08244	0.060982	0.50987	1.1088	0.14898	0.59306	0.86041	1.0235	0.033462	0.82851	0.99512	1.0118	0.016942	0.32714	0.99941	0.93813	-0.09214
672.41	3.14	olomics Pc	0.43956	0.99951	1.3269	0.40808	0.10277	0.99998	0.74668	-0.42143	0.68757	0.99941	0.93105	-0.10306	0.012033	0.20055	0.56569	-0.82191	0.001305	0.011482	0.47249	-1.0816	0.029903	0.13218	0.55456	-0.85059	0.74635	0.99391	0.94579	-0.0804	0.73394	0.99956	0.97101	-0.04244	0.029658	0.2688	1.6492	0.72179
374.98	4.54	olomics Ne	0.43968	0.99918	1.0247	0.035166	0.035073	0.44974	0.88052	-0.18357	0.83926	0.99995	0.99103	-0.01299	0.538	0.99985	0.97312	-0.03931	0.44806	0.99927	0.96169	-0.05636	0.29758	0.71853	1.0642	0.089758	0.21441	0.59066	1.0462	0.065169	0.82357	0.99512	0.98845	-0.01677	0.81349	0.99941	0.98932	-0.01549
397.234	3.52	domics Pos	0.43997	0.99987	0.86733	-0.20534	0.085371	0.99994	0.75833	-0.3991	0.087318	0.99996	0.72134	-0.47124	0.031305	0.30765	0.60112	-0.73427	0.006843	0.022971	0.5133	-0.96212	0.019939	0.12102	0.46936	-1.0912	0.015091	0.33809	0.56194	-0.83152	0.004122	0.52955	0.49879	-1.0035	0.1729	0.28053	0.71249	-0.48905
445.8678	10.15	domics Pos	0.44034	0.99987	0.912	-0.13289	0.5174	0.99994	0.91689	-0.12518	0.94613	0.99996	1.0126	0.01804	0.88557	0.9874	0.98646	-0.01967	0.021127	0.054616	0.71043	-0.49324	0.11515	0.3659	0.79993	-0.32205	0.60481	0.86762	0.9392	-0.09049	0.80559	0.97988	0.96732	-0.04793	0.42697	0.53379	1.1374	0.18574
185.1006	0.53	domics Pos	0.44047	0.99987	1.2514	0.32357	0.72135	0.99994	1.0664	0.092726	0.37391	0.99996	1.2648	0.3389	0.29371	0.75244	1.2096	0.27457	0.75831	0.79304	0.92226	-0.11676	0.75007	0.87064	1.0878	0.12147	0.214	0.65488	1.4007	0.48618	0.036773	0.64014	1.5251	0.60888	0.44765	0.55233	1.1115	0.15249
853.5281	1.45	domics Pos	0.44058	0.99987	1.2808	0.35705	0.44305	0.99994	0.88995	-0.16821	0.54404	0.99996	1.1043	0.14315	0.050414	0.38141	0.69959	-0.51541	0.016731	0.04531	0.6147	-0.70205	0.14273	0.41448	0.77561	-0.3666	0.35119	0.75061	1.3157	0.39587	0.95631	0.98838	1.1007	0.13845	0.077042	0.17834	1.6944	0.76081
239.2363	6.82	olomics Pc	0.44133	0.99951	0.91412	-0.12954	0.60857	0.99998	0.94227	-0.08578	0.024311	0.99941	0.65715	-0.6057	0.028145	0.24036	0.54299	-0.88101	0.001041	0.009561	0.51189	-0.96608	0.003123	0.049681	0.40321	-1.3104	0.008622	0.23862	0.46858	-1.0936	0.01169	0.47452	0.47267	-1.0811	0.020631	0.2688	0.50727	-0.97918
1165.755	0.52	domics Pos	0.44141	0.99987	0.9106	-0.13511	0.239	0.99994	0.88695	-0.17307	0.34863	0.99996	0.90535	-0.14345	0.70172	0.96092	0.9585	-0.06115	0.035895	0.081083	0.77002	-0.37704	0.23293	0.5203	0.86742	-0.2052	0.39897	0.77238	0.91231	-0.1324	0.47741	0.96776	0.92254	-0.11632	0.3278	0.43764	1.1361	0.18412
606.7913	0.56	domics Pos	0.44148	0.99987	1.0687	0.095924	0.47639	0.99994	0.96587	-0.0501	0.21638	0.99996	0.92938	-0.10566	0.005293	0.17581	0.80896	-0.30586	0.000206	0.001727	0.67209	-0.57327	0.027234	0.14544	0.82885	-0.27083	0.024612	0.33809	0.8094	-0.30508	0.18135	0.92443	0.91743	-0.12433	0.085664	0.18633	1.1342	0.18171
924.7121	7.25	domics Pos	0.44156	0.99987	1.1268	0.17226	0.49481	0.99994	1.1476	0.19856	0.62398	0.99996	1.098	0.13485	0.65656	0.95652	0.92602	-0.11088	0.092618	0.16487	0.70842	-0.49732	0.054203	0.23015	0.51742	-0.9506	0.13338	0.59973	0.59363	-0.75235	0.018362	0.52955	0.34879	-1.5196	0.031722	0.12169	0.40072	-1.3193
525.2798	1.12	domics Pos	0.4418	0.99987	1.1485	0.19972	0.26675	0.99994	1.1286	0.17449	0.097407	0.99996	1.1701	0.2266	0.40246	0.8455	1.0703	0.098019	0.87442	0.89412	0.99198	-0.01161	0.39027	0.64146	1.3242	0.40512	0.020553	0.33809	1.8211	0.86477	0.52307	0.96776	1.2515	0.32365	0.023028	0.10989	1.5282	0.61186
312.3269	7.87	olomics Pc	0.44195	0.99951	0.93032	-0.1042	0.082656	0.99998	0.79786	-0.32579	0.089155	0.99941	0.78204	-0.35468	0.1174	0.4687	0.86344	-0.21183	0.70685	0.99688	0.96336	-0.05386	0.002765	0.047591	0.62002	-0.68961	0.060082	0.49674	0.72419	-0.46556	0.006547	0.47452	0.67976	-0.55689	0.020766	0.2688	0.72928	-0.45545

1028.652	8.86	olomics Pc	0.442	0.99951	1.2641	0.33811	0.90437	0.99998	0.98756	-0.01806	0.5441	0.99941	1.0672	0.09383	0.02748	0.24036	0.66409	-0.59055	0.058374	0.1884	0.60924	-0.71493	0.15302	0.39973	0.78272	-0.35343	0.71128	0.99391	0.94865	-0.07605	0.96559	0.99956	1.0284	0.040365	0.15977	0.41696	1.6289	0.70387
672.5772	7.81	omics Pos	0.44235	0.99987	0.8893	-0.16926	0.065161	0.99994	0.67591	-0.56509	0.33729	0.99996	0.85785	-0.2212	0.044335	0.36496	0.67784	-0.56098	0.30962	0.40292	0.81064	-0.30287	0.72408	0.8569	0.96383	-0.05315	0.28493	0.71383	0.84533	-0.24241	0.097085	0.84751	0.69017	-0.53498	0.36611	0.47482	1.5955	0.67404
1033.774	10.03	omics Pos	0.4425	0.99987	0.95743	-0.06276	0.54661	0.99994	0.95125	-0.07211	0.28303	0.99996	0.93529	-0.09652	0.2123	0.66014	0.90753	-0.13998	0.002872	0.011852	0.77477	-0.36816	0.32751	0.60154	0.90603	-0.14237	0.92587	0.98294	1.0106	0.015245	0.60151	0.97073	0.96486	-0.05161	0.016568	0.099614	1.1935	0.25525
861.6781	10.3	olomics Pc	0.4426	0.99951	0.90887	-0.13785	0.026676	0.99998	0.81151	-0.30132	0.53109	0.99941	0.9377	-0.0928	0.41161	0.90278	0.90509	-0.14387	0.83699	0.99688	0.98464	-0.02233	0.33582	0.64542	1.0718	0.10007	0.091822	0.60726	0.86283	-0.21285	0.43337	0.99956	0.91231	-0.1324	0.018712	0.2688	0.70779	-0.4986
722.5112	8.87	olomics Ne	0.44284	0.99918	1.1018	0.13988	0.92681	0.9934	0.99403	-0.00864	0.64675	0.99995	0.9181	-0.12328	0.55293	0.99985	0.89557	-0.15913	0.57283	0.99927	1.1191	0.16233	0.1145	0.58058	1.3226	0.40338	0.042522	0.37694	1.3763	0.46076	0.19373	0.99512	1.2517	0.32384	0.68754	0.99941	1.042	0.05933
163.1489	6.97	olomics Pc	0.44298	0.99951	1.1615	0.21602	0.58625	0.99998	0.91313	-0.13111	0.20762	0.99941	0.8183	-0.2893	0.035292	0.25674	0.57627	-0.79519	0.002618	0.018492	0.3935	-1.3456	0.001272	0.045425	0.39116	-1.3542	0.008634	0.23862	0.48882	-1.0326	0.037358	0.61903	0.55778	-0.84223	0.10075	0.35342	0.68693	-0.54176
417.7962	5.61	omics Pos	0.44316	0.99987	1.1739	0.23131	0.75305	0.99994	0.98466	-0.0223	0.6431	0.99996	0.95459	-0.06704	0.18164	0.62061	0.84235	-0.24751	0.0071	0.023604	0.72618	-0.46161	0.11007	0.35477	0.82247	-0.28197	0.49282	0.82243	0.92898	-0.10628	0.53693	0.96776	0.92839	-0.1072	0.55567	0.65153	1.0552	0.0775
594.7629	0.56	omics Pos	0.44322	0.99987	1.1028	0.14118	0.1255	0.99994	1.1514	0.20343	0.38952	0.99996	0.95722	-0.06308	0.30283	0.76085	0.92881	-0.10654	0.012423	0.03616	0.75003	-0.41498	0.002702	0.061655	0.85726	-0.22219	0.33356	0.74176	0.88704	-0.17293	0.72265	0.97988	0.97796	-0.03216	0.090413	0.19082	1.1667	0.22247
1005.757	9.8	olomics Pc	0.4433	0.99951	1.3255	0.40655	0.34746	0.99998	0.83453	-0.26096	0.89433	0.99941	0.99498	-0.00726	0.021976	0.2227	0.60916	-0.71511	0.018448	0.07874	0.59951	-0.73813	0.033844	0.14271	0.58936	-0.76278	0.52898	0.99391	0.87554	-0.19176	0.54104	0.99956	0.8811	-0.18262	0.11628	0.36726	1.8148	0.8598
666.9764	9.52	olomics Pc	0.44381	0.99951	1.1977	0.26027	0.12066	0.99998	0.77215	-0.37305	0.70255	0.99941	1.0321	0.045608	0.005587	0.1948	0.66	-0.59946	0.001038	0.009561	0.58749	-0.76737	0.006082	0.065713	0.68369	-0.54858	0.61416	0.99391	0.94506	-0.08152	0.9933	0.99956	1.103	0.14141	0.19872	0.46339	1.6124	0.6892
431.9182	10.02	omics Pos	0.4441	0.99987	1.0295	0.04201	0.060974	0.99994	0.89994	-0.1521	0.59819	0.99996	0.97567	-0.03554	0.2056	0.65222	0.95199	-0.07098	0.000549	0.003537	0.74983	-0.41536	0.11328	0.36206	0.88637	-0.17402	0.87459	0.97532	1.0059	0.008528	0.46668	0.96776	0.95919	-0.06011	0.013679	0.098791	1.1908	0.25193
1041.186	4.5	omics Pos	0.44419	0.99987	1.1914	0.25267	0.087586	0.99994	0.81495	-0.29523	0.69185	0.99996	0.96019	-0.05861	0.044613	0.36583	0.66475	-0.58912	0.000618	0.003843	0.4252	-1.2338	0.001336	0.046324	0.55024	-0.86186	0.59247	0.86323	0.93517	-0.09671	0.57748	0.9703	0.9362	-0.09511	0.031544	0.12169	1.8906	0.91888
1092.845	9.74	olomics Pc	0.44434	0.99951	1.2639	0.33784	0.49842	0.99998	0.84307	-0.24628	0.85258	0.99941	0.9964	-0.00521	0.073176	0.35124	0.6057	-0.72333	0.055795	0.18251	0.584	-0.77597	0.042021	0.16629	0.57189	-0.80619	0.53503	0.99391	0.85218	-0.23077	0.80298	0.99956	0.97443	-0.03737	0.074701	0.31928	1.8055	0.8524
340.3585	5.03	omics Pos	0.44442	0.99987	0.8973	-0.15634	0.2321	0.99994	0.84112	-0.24962	0.56961	0.99996	0.9255	-0.1117	0.17639	0.60979	0.80807	-0.30745	0.006271	0.02164	0.61939	-0.69108	0.003629	0.067492	0.57272	-0.80409	0.3087	0.72874	0.84829	-0.23737	0.088345	0.82455	0.77525	-0.36727	0.29611	0.40672	0.87726	-0.18893
148.038	0.61	omics Pos	0.44558	0.99987	1.2359	0.30551	0.79589	0.99994	0.92624	-0.11054	0.75836	0.99996	0.93121	-0.10282	0.91775	0.9874	1.0469	0.066098	0.042955	0.093008	0.5462	-0.87251	0.46995	0.69377	0.84991	-0.23462	0.98206	0.99431	0.98896	-0.01601	0.55662	0.96798	0.86603	-0.20751	0.074779	0.17703	1.3978	0.4832
962.564	5.79	omics Pos	0.44592	0.99987	0.93747	-0.09315	0.41928	0.99994	0.93415	-0.09827	0.42064	0.99996	0.91076	-0.13486	0.19178	0.63368	0.86803	-0.20418	0.002807	0.011635	0.69306	-0.52894	0.008309	0.084185	0.72627	-0.46143	0.13766	0.60294	0.81314	-0.29842	0.021035	0.52955	0.80608	-0.311	0.137	0.2424	1.1312	0.17787
1095.701	9.04	olomics Pc	0.44616	0.99951	1.3926	0.47779	0.34358	0.99998	0.85046	-0.23368	0.60354	0.99941	0.95186	-0.07118	0.013635	0.20085	0.68263	-0.55082	0.063801	0.20093	0.6397	-0.64454	0.31981	0.62611	0.90828	-0.13878	0.95419	0.9974	1.1208	0.1645	0.97067	0.99956	1.0282	0.04013	0.031863	0.2688	1.6155	0.69194
936.5612	6.34	omics Pos	0.44641	0.99987	1.0974	0.13409	0.72196	0.99994	1.04	0.056619	0.57297	0.99996	0.93351	-0.09926	0.5011	0.89009	0.9142	-0.12942	0.036916	0.083093	0.67099	-0.57564	0.077465	0.28838	0.69815	-0.51838	0.13756	0.60294	0.74307	-0.42844	0.047768	0.67624	0.67188	-0.57372	0.80724	0.8569	0.99474	-0.00761
355.9836	12	olomics Ne	0.44651	0.99918	0.83788	-0.25519	0.046191	0.47152	0.61493	-0.70151	0.047527	0.99995	0.65226	-0.61648	0.078115	0.99985	0.64136	-0.64078	0.062559	0.99927	0.63739	-0.64975	0.84925	0.96837	1.0441	0.062283	0.22998	0.61014	0.78496	-0.34932	0.13745	0.99512	0.70744	-0.49933	0.19682	0.99941	0.76478	-0.38688
559.9782	12.02	olomics Ne	0.44656	0.99918	1.1311	0.17779	0.20406	0.62533	0.87743	-0.18864	0.42682	0.99995	0.91825	-0.12304	0.63539	0.99985	1.1414	0.19084	0.10794	0.99927	1.2266	0.29468	0.036403	0.48305	1.2492	0.32105	0.78236	0.94099	1.0604	0.084583	0.45621	0.99512	0.90691	-0.14097	0.36117	0.99941	0.90838	-0.13864
437.7839	5.24	omics Pos	0.44761	0.99987	0.92333	-0.11508	0.97812	0.99994	1.0041	0.005862	0.47156	0.99996	0.94177	-0.08655	0.006375	0.1899	0.76121	-0.39364	0.023146	0.058254	0.73016	-0.45371	0.22246	0.51214	0.88403	-0.17784	0.56115	0.855	0.93797	-0.09239	0.55136	0.96776	0.93237	-0.10103	0.059543	0.15715	1.2206	0.28756
333.2845	6.92	olomics Pc	0.45011	0.99951	1.122	0.16612	0.47049	0.99998	0.89586	-0.15865	0.27695	0.99941	0.84703	-0.23951	0.007667	0.1948	0.5639	-0.8265	0.002598	0.018433	0.37721	-1.4065	0.001501	0.045425	0.38995	-1.3586	0.005133	0.20472	0.46179	-1.1147	0.056511	0.7036	0.60616	-0.72222	0.083983	0.3336	0.67494	-0.56716
299.311	7.46	olomics Pc	0.45028	0.99951	0.89291	-0.16341	0.2194	0.99998	0.84356	-0.24544	0.049108	0.99941	0.73067	-0.45271	0.19462	0.62875	0.82597	-0.27584	0.34352	0.72673	0.87596	-0.19106	0.005796	0.064803	0.6089	-0.71572	0.13189	0.68208	0.7371	-0.44007	0.009113	0.47452	0.6498	-0.62194	0.034338	0.2709	0.71398	-0.48604
445.368	5.08	omics Pos	0.4509	0.99987	0.91934	-0.12133	0.3708	0.99994	0.94317	-0.08441	0.54518	0.99996	1.0814	0.11295	0.4737	0.88482	0.94758	-0.07769	0.032548	0.07549	0.78252	-0.35379	0.55049	0.75179	0.94897	-0.07556	0.44516	0.80121	0.89884	-0.15387	0.53059	0.96776	1.0931	0.12838	0.14586	0.25053	1.1681	0.22412
164.0323	0.58	omics Pos	0.45101	0.99987	0.90056	-0.1511	0.21363	0.99994	0.83363	-0.26252	0.39513	0.99996	1.1638	0.21885	0.066526	0.43847	1.5214	0.60538	0.30595	0.39951	1.1818	0.241	0.029477	0.1523	1.3737	0.45803	0.001713	0.23318	1.9099	0.93353	0.57093	0.9703	1.1692	0.22555	0.62964	0.721	1.1185	0.16155
893.6737	5.34	omics Pos	0.45103	0.99987	1.0737	0.10256	0.76461	0.99994	0.96735	-0.04788	0.47555	0.99996	0.92583	-0.11118	0.94973	0.99328	0.99251	-0.01085	0.016646	0.04511	0.64923	-0.62321	0.034066	0.16591	0.72821	-0.45757	0.79724	0.95226	1.0225	0.032082	0.78566	0.97988	1.0281	0.039918	0.13139	0.23654	1.2403	0.31069
901.5895	9.97	olomics Ne	0.45119	0.99918	1.0592	0.082991	0.09494	0.51286	0.90969	-0.13655	0.54176	0.99995	0.94812	-0.07685	0.82671	0.99985	1.0532	0.074829	0.69875	0.99927	0.97726	-0.03318	0.035298	0.4828	1.2041	0.26798	0.43877	0.77603	0.91315	-0.13108	0.1191	0.99512	1.1185	0.16154	0.12699	0.99941	1.3647	0.44857
1181.753	10.03	omics Pos	0.45125	0.99987	0.95194	-0.07106	0.88353	0.99994	0.99252	-0.01084	0.10459	0.99996	0.87519	-0.19234	0.51873	0.89932	0.94991	-0.07414	0.028631	0.068402	0.80576	-0.31158	0.13397	0.40042	0.86895	-0.20266	0.1412	0.60462	0.88308	-0.17939	0.66866	0.97988	1.0464	0.065393	0.26857	0.37872	1.1569	0.21032
1026.798	10.02	omics Pos	0.45139	0.99987	0.89735	-0.15625	0.16889	0.99994	0.84984	-0.23474	0.30835	0.99996	0.8839	-0.17805	0.72859	0.96895	0.95521	-0.0661	0.013707	0.039062	0.71206	-0.48994	0.25647	0.54359	0.86038	-0.21696	0.41513	0.78082	0.91332	-0.1308	0.71363	0.97988	0.95374	-0.06833	0.21882	0.32671	1.1234	0.16792
963.6236	9.15	olomics Pc	0.45156	0.99951	1.1967	0.25905	0.25932	0.99998	0.80687	-0.3096	0.98776	0.99941	0.98509	-0.02168	0.2261	0.67509	0.76561	-0.38531	0.020191	0.084577	0.60533	-0.72421	0.24017	0.53089	0.74992	-0.41519	0.58748	0.99391	0.89925	-0.1532	0.47911	0.99956	0.86109	-0.21577	0.64086	0.83691	1.2979	0.37617
1052.185	4.49	omics Pos	0.45187	0.99987	1.2807	0.35693	0.26575	0.99994	0.79858	-0.32449	0.82614	0.99996	0.93719	-0.09359	0.074717	0.46303	0.6015	-0.73336	0.001645	0.007821	0.39222	-1.3503	0.036732	0.1743	0.59232	-0.75555	0.60524	0.86762	0.89056	-0.16722	0.80279	0.97988	0.98079	-0.02798	0.12065	0.22484	1.7083	0.77252
362.254	2.28	omics Pos	0.45245	0.99987	1.1087	0.14887	0.61914	0.99994	0.92038	-0.1197	0.66744	0.99996	0.92977	-0.10506	0.38397	0.83078	0.87227	-0.19715	0.029393	0.069873	0.61985	-0.69001	0.031968	0.16113	0.60788	-0.71814	0.22439	0.66687	0.74463	-0.4254	0.12031	0.88537	0.67504	-0.56696	0.43636	0.54233	0.86339	-0.21192
207.1737	2.28	omics Pos	0.45251	0.99987	0.88369	-0.17838	0.1835	0.99994	0.73315	-0.44782	0.024978	0.99996	0.56821	-0.81551	0.027758	0.29642	0.51033	-0.9705	0.001466	0.00724	0.37281	-1.4235	0.007049	0.081828	0.34152	-1.5499	0.00618	0.3088	0.42246	-1.2431	0.008309	0.52955	0.33473	-1.5789	0.020384	0.10677	0.47047	-1.0878
868.5666	5.72	omics Pos	0.45294	0.99987	0.92511	-0.1123	0.13248	0.99994	0.83735	-0.2561	0.6271	0.99996	0.94805	-0.07697	0.49371	0.88874	0.9236	-0.11466	0.010345	0.031557	0.7309	-0.45225	0.14262	0.41448	0.8315	-0.26622	0.3463	0.74644	0.91347	-0.13057	0.554	0.96798	0.94381	-0.08344	0.12487	0.22947	1.1986	0.26137
772.855	10.02	omics Pos	0.45348	0.99987	1.0463	0.065334	0.072044	0.99994	0.88484	-0.17652	0.29058	0.99996	0.9213	-0.11826	0.27645	0.74181	0.93426	-0.09811	0.008324	0.026762	0.74866	-0.41761	0.1259	0.38618	0.87968	-0.18495	0.61124	0.87064	1.0261	0.037112	0.70563	0.97988	0.97296	-0.03955	0.11446	0.21881	1.1604	0.2146
1061.76	10.05	omics Pos	0.45351	0.99987	1.0559	0.078511	0.67246	0.99994	0.96015	-0.05867	0.7448	0.99996	1.0534	0.075005	0.16221	0.59299	0.88565	-0.1752	0.013805	0.039286	0.73671	-0.44084	0.67367	0.83076	0.95937	-0.05984	0.4094	0.78014	1.0736	0.10243	0.33767	0.96524	1.0883	0.12202	0.011222	0.094875	1.3616	0.44531
548.8295	10.11	omics Pos	0.45386	0.99987	1.0743	0.10335	0.53502	0.99994	0.95315	-0.06923	0.46857	0.99996	1.0624	0.087317	0.29143	0.75244	0.89593	-0.15854	0.008732	0.027608	0.74375	-0.42712	0.33853	0.60941	0.92126	-0.11832	0.61774	0.87206	0.93914	-0.09058	0.32781	0.95887	0.90443	-0.14492	0.13944	0.24468	1.1924	0.25382
944.5808	10.41	olomics Ne	0.45414	0.99918	1.096	0.13223	0.59835	0.8998	0.94836	-0.0765	0.50017	0.99995	1.0719	0.10022	0.33053	0.99985	1.0906	0.12512	0.49959	0.99927	1.0564	0.079118	0.088828	0.55533	1.241	0.31152	0.042563	0.37694	1.2486	0.32026	0.17506	0.99512	1.1208	0.16447	0.56684	0.99941	1.0492	0.069307
788.6156	5.72	omics Pos	0.45538	0.99987	1.0689	0.096146	0.74071	0.99994	0.97073	-0.04286	0.66585	0.99996	0.96127	-0.05699	0.32074	0.78446	0.89607	-0.15831	0.039292	0.087256	0.78798	-0.34376	0.93935	0.9677	0.99801	-0.00287	0.42498	0.78878	1.1347	0.18226	0.62398	0.97813	1.0819	0.11355	0.031702	0.12169	2.0673	1.0478
356.0124	2.63	olomics Ne	0.4563	0.99918	0.95102	-0.07246	0.31066	0.73969	0.9071	-0.14067	0.96735	0.99995	0.99407	-0.00858	0.81403	0.99985	0.98175	-0.02658	0.044962	0.99927	0.85521	-0.22565	0.36556	0.77187	0.93412	-0.09832	0.025743	0.33786	0.81324	-0.29825	0.67045	0.99512	0.96683	-0.04867	0.67805	0.99941	0.96905	-0.04536
209.2017	9.98	omics Pos	0.45705	0.99987	1.0475	0.067009	0.59259	0.99994	0.96015	-0.05866	0.70394	0.99996	0.96807	-0.04681	0.90024	0.9874	0.98869	-0.01641	0.018617	0.049364	0.77156	-0.37415	0.79891	0.89727	0.9783	-0.03165	0.63804	0.88435	1.0475	0.066939	0.42915	0.96776	1.076	0.10572	0.028726	0.11732	1.2961	0.37421
352.8987	0.54	omics Pos	0.45775	0.99987	1.0378	0.053507	0.23491	0.99994	0.93192	-0.10172	0.19219	0.99996	0.93222	-0.10126	0.012745	0.22648	0.86406	-0.21079	0.000658	0.004029	0.73297	-0.44817	0.11427	0.36444	0.85278	-0.22975	0.1457	0.60716	0.90613	-0.14221	0.29497	0.9483	0.93596	-0.09547	0.17003	0.27778	1.156	0.20916
195.1022	0.84	omics Pos	0.45788	0.99987	0.93034	-0.10417	0.11227	0.99994	0.77717	-0.36369	0.3175	0.99996	0.87849	-0.18691	0.29432	0.75244	0.89963	-0.1526	0.00627	0.02164	0.70132	-0.51185	0.011715	0.092481	0.66164	-0.59588	0.20867	0.65208	0.81026	-0.30354	0.047656	0.67624	0.80128	-0.31962	0.41153	0.51891	0.91286	-0.13153
164.1524	6.38	olomics Pc	0.45802	0.99951	1.1303	0.17668	0.3065	0.99998	0.8474	-0.23888	0.88123	0.99941	0.97247	-0.04027	0.16581	0.57522	0.75117	-0.41279	0.000306	0.003915	0.25955	-1.9459	0.005771	0.064803	0.55958	-0.83758	0.081836	0.5819	0.68653	-0.5426	0.20358	0.99956	0.79956	-0.32272	0.62794	0.82935	0.91582	-0.12687
820.5854	5.01	omics Pos	0.45863	0.99987	1.0934	0.12884	0.9308	0.99994	1.0088	0.012687	0.71752	0.99996	1.0414	0.058574	0.85473	0.9874	0.9793	-0.03017	0.045445	0.096968	0.74713	-0.42057	0.035876	0.17185	0.7719	-0.37351	0.030149	0.35536	0.78489	-0.34944	0.088878	0.82455	0.79788	-0.32576	0.57341	0.66802	0.92921	-0.10593
409.2379	7.16	olomics Pc	0.45871	0.99951	0.89611	-0.15825	0.38216	0.99998	0.86019	-0.21727	0.027953	0.99941	0.58133	-0.78257	0.003519	0.17363	0.45011	-1.1517	1.3E-06	0.000231	0.32294	-1.6307	7.38E-05	0.034543	0.23013	-2.1195	0.001639	0.16989	0.35233	-1.505	0.000409	0.47452	0.35694	-1.4862	0.000791	0.2688	0.39769	-1.3303
392.745	2.95	olomics Pc	0.45962	0.99951	1.2117	0.27706	0.057476	0.99998	0.75369	-0.40796	0.11108	0.99941	0.7934	-0.33387	0.007079	0.1948	0.47825	-1.0642	0.001484	0.012568	0.4844	-1.0457	0.010498	0.081051	0.55801	-0.84163	0.37015	0.96894	0.8412	-0.24947	0.41448	0.99956	0.86757	-0.20495	0.04855	0.28861	1.8158	0.8606
373.2122	2.04	omics Pos	0.45972	0.99987	0.88471	-0.17672	0.2431	0.99994	0.82845	-0.27151	0.025816	0.99996	0.66829	-0.58145	0.005303	0.17581	0.48579	-1.0416	0.000639	0.003941	0.38886	-1.3627	0.006592	0.0803	0.3367	-1.5705	0.033839	0.36346	0.45167	-1.1467	0.010024	0.52955	0.41924	-1.2541	0.006029	0.08977	0.48662	-1.0391
838.8385	10.02	omics Pos	0.45973	0.99987	1.0271	0.038635	0.2758	0.99994	0.94697	-0.07861	0.18745	0.99996	0.94923	-0.07518	0.24216	0.70272	0.93687	-0.09408	0.000182	0.001577	0.76858	-0.37974	0.26121	0.54699	0.90243	-0.14812	0.92806	0.98393	1.0062	0.008917	0.74484	0.97988	0.98523	-0.02147	0.010394	0.094875	1.2079	0.27248
462.296	5.23	olomics Pc	0.46053	0.99951	0.77259	-0.37222	0.69858	0.99998	0.88263	-0.18013	0.44305	0.99941	0.76904	-0.37886	0.50473	0.98142	0.82226	-0.28234	0.000124	0.001894	0.11885	-3.0728	0.076104	0.24714	0.53094	-0.91339	0.8205	0.99445	0.89913	-0.1534	0.76779	0.99956	0.93811	-0.09217	0.70925	0.8683	1.1475	0.19853
872.8327	0.5	omics Pos	0.46128	0.99987	0.96301	-0.05437	0.22875	0.99994	0.93924	-0.09043	0.063056	0.99996	0.91246	-0.13216	0.002127	0.17581	0.817	-0.29158	2.67E-05	0.000439	0.7222	-0.46953	0.14166	0.41327	0.82597	-0.27584	0.1761	0.64616	0.88767	-0.17191	0.03356	0.6327	0.88586	-0.17485	0.14004	0.2452	1.1252	0.17024
818.7816	10.07	omics Pos	0.46129	0.99987	1.0296	0.042066	0.82961	0.99994	0.98922	-0.01564	0.63759	0.99996	0.97145	-0.04178	0.17098	0.60478	0.89985	-0.15225	0.001449	0.007174	0.82317	-0.28073	0.16395	0.44908	0.84265	-0.24699	0.87093	0.97532	0.99255	-0.01078	0.37766	0.96776	0.96932	-0.04496	0.003396	0.085302	1.2304	0.29911
456.9775	12	olomics Ne	0.46224	0.99918	1.0704	0.098213	0.20612	0.62805	0.88387	-0.1781	0.71476	0.99995	1.0619	0.086583	0.9377	0.99985	1.0132	0.01892	0.19394	0.99927	1.1771	0.23526	0.56032	0.87267	1.0471	0.066336	0.049063	0.39082	1.2858	0.36267	0.48856	0.99512	1.0981	0.13499	0.098638	0.99941	1.1631	0.21794
1108.615	6.06	omics Pos	0.46225	0.99987	1.0982	0.13508	0.65553	0.99994	0.94831	-0.07656	0.79164	0.99996	0.97195	-0.04105	0.86208	0.9874	0.9776	-0.03268	0.03076	0.072222	0.70704	-0.50013	0.74448	0.86962	0.99289	-0.0103	0.44619	0.80121	0.92474	-0.11287	0.45877	0.96776	0.91551	-0.12734	0.87874	0.91248	0.98176	-0.02656
879.7297	7.57	omics Pos	0.46279	0.99987	0.81658	-0.29234	0.3253	0.99994	0.76874	-0.37942	0.35898	0.99996	0.77287	-0.3717	0.27382	0.7363	0.68594	-0.54384	0.11151	0.18922	0.4879	-1.0353	0.047523	0.21095	0.48444	-1.0456	0.16283	0.63418	0.59407	-0.75129	0.01713	0.52955	0.47048	-1.0878	0.8229	0.87014	0.96407	-0.05279
407.3268	6.84	olomics Pc	0.46298	0.99951	0.92177	-0.11752	0.71486	0.99998	0.96055	-0.05806	0.18405	0.99941	0.86445	-0.21015	0.094197	0.41268	0.80271	-0.31705	0.054533	0.17931	0.8165	-0.29247	0.008731	0.075866	0.73951	-0.43536	0.13312	0.68208	0.81816	-0.28954	0.042166	0.64572	0.74168	-0.43113	0.078807	0.32476	0.76972	-0.3776
837.6745	10.35	olomics Pc	0.46312	0.99951	0.93364	-0.09905	0.54476	0.99998	0.95117	-0.07223	0.061508	0.99941	0.84817	-0.23758	0.91684	0.99905	1.0001	0.000143	0.75136	0.99688	1.0274	0.039005	0.64701	0.88964	1.0364	0.051544	0.93293	0.9955	0.9945	-0.00796	0.02924	0.58535	0.83644	-0.25766	0.006496	0.2688	0.80649	-0.31027
145.9557	10.18	omics Pos	0.46362	0.99987	1.0467	0.065888	0.61603	0.99994	0.96866	-0.04594	0.33774	0.99996	0.95281	-0.06974	0.64079	0.95076	0.9709	-0.04261	0.003474	0.013708	0.8443	-0.24417	0.66084	0.82346	0.96981	-0.04423	0.15187	0.62225	1.1054	0.14454	0.044715	0.65325	1.1577	0.21127	0.007632	0.091944	1.3222	0.40294
552.6123	7.68	olomics Pc	0.46388	0.99951	0.88207	-0.18103	0.204	0.99998	0.79437	-0.33211	0.87809	0.99941	0.98408	-0.02316	0.90259	0.99905	1.02	0.028566	0.034727	0.12854	0.75709	-0.40146	0.32519	0.63387	0.85742	-0.22193	0.6553	0.99391	1.0479	0.067526	0.27377	0.99956	0.87313	-0.19573	0.21579	0.48311	1.2652	0.33931
239.2254	9.91	omics Pos	0.46395	0.99987	1.0238	0.033908	0.32724	0.99994	0.94901	-0.0755	0.50278	0.99996	0.97731	-0.03312	0.29479	0.75244	0.96022	-0.05856	0.003272	0.013118	0.7924	-0.3357	0.39027	0.64146	0.93943	-0.09014	0.6898	0.90885	1.0223	0.031794	0.38503	0.96776	1.0576	0.080735	0.027107	0.11567	1.1644	0.21961
1045.775	10.05	omics Pos	0.46413	0.99987	1.0695	0.097003	0.35192	0.99994	1.0782	0.10864	0.63586	0.99996	1.0328	0.04651	0.35133	0.80554	1.0836	0.11585	0.021812	0.055631	0.72152	-0.47089	0.2633	0.54981	0.86172	-0.21471	0.77419	0.94464	0.96434	-0.05239	0.80958	0.97988	1.0194	0.027715	0.11049	0.21408	1.2896	0.36697
269.6454	1.3	omics Pos	0.46415	0.99987	1.1152	0.1573	0.67715	0.99994	1.0261	0.037181	0.4444	0.99996	0.90814	-0.13901	0.26491	0.7252	0.90722	-0.14047	0.13907	0.22341	0.79925	-0.32329	0.97123	0.98437	1.0007	0.001061	0.027013	0.34512	1.334	0.41576	0.039679	0.64308	1.1395	0.1884	0.002738	0.083394	1.3897	0.47479
1094.797	9.69	olomics Pc	0.46495	0.99951	1.232	0.30103	0.27146	0.99998	0.83799	-0.25499	0.65711	0.99941	1.0445	0.062852	0.028126	0.24036	0.60176	-0.73273	0.014094	0.063332	0.56106	-0.83376	0.017117	0.096631	0.63363	-0.65828	0.35856	0.96545	0.8226	-0.28174	0.84879	0.99956	1.0207	0.029535	0.12211	0.37203	1.7396	0.79873
671.5734	7.81	omics Pos	0.46514	0.99987	0.86774	-0.20466	0.022486	0.99994	0.58549	-0.77227	0.27157	0.99996	0.80627	-0.31066	0.10585	0.52975	0.68704	-0.54153	0.38399	0.47513	0.82576	-0.27621	0.75488	0.87197	0.95764	-0.06244	0.30168	0.72409	0.8243	-0.27875	0.15389	0.91321	0.71597	-0.48204	0.40822	0.51559	1.4995	0.58452
269.9996	0.56	omics Pos	0.46564	0.99987	1.1102	0.15081	0.53313	0.99994	1.2207	0.28771	0.15871	0.99996	1.2979	0.3762	0.40299	0.84551	1.1338	0.18114	0.11108	0.18863	1.3656	0.44957	0.011822	0.092554	1.5392	0.6222	0.028991	0.3527	1.8576	0.89347	0.67785	0.97988	1.0478	0.067426	0.043522	0.13887	1.435	0.52107
1160.725	10.05	omics Pos	0.46565	0.99987	0.94535	-0.08108	0.3333	0.99994	0.91246	-0.13216	0.65587	0.99996	0.96866	-0.04593	0.29282	0.75244	0.91376	-0.13011	0.04542	0.096968	0.84129	-0.24933	0.18866	0.47491	0.85805	-0.22086	0.60541	0.86762	0.95475	-0.0668	0.68469	0.97988	0.97696	-0.03363	0.11131	0.2149	1.1583	0.21205
356.8305	12.74	olomics Ne	0.46589	0.99918	1.064	0.08951	0.00959	0.34605	0.88024	-0.18403	0.95461	0.99995	1.0043	0.006156	0.99077	0.99985	1.0083	0.011901	0.77395	0.99927	0.98353	-0.02396	0.006263	0.36417	1.1439	0.194	0.41138	0.75731	0.96338	-0.05382	0.61768	0.99512	0.96917	-0.04518	0.44011	0.99941	0.96779	-0.04723
184.074	4.66	omics Pos	0.46598	0.99987	1.0516	0.072631	0.47037	0.99994	0.93327	-0.09963	0.89833	0.99996	1.024	0.034147	0.74185	0.97237	0.96714	-0.04821	0.044221	0.0951	0.79752	-0.32641	0.72366	0.85667	0.96293	-0.05449	0.51299	0.83443	1.0543	0.076329	0.27667	0.94365	0.90458	-0.14468	0.70552	0.78023	1.0251	0.035733
764.199	1.03	omics Pos	0.46647	0.99987	0.88068	-0.18332	0.93757	0.99994	0.9971	-0.00419	0.91745	0.99996	0.99342	-0.00952	0.82324	0.98451	1.0162	0.023191	0.039413	0.087373	0.71117	-0.49172	0.16718	0.45275	1.2715	0.34652	0.12872	0.58848	1.3548	0.43807	0.83673	0.97988	1.0258	0.036791	0.25291	0.3618	1.2667	0.34103
1181.272	8.99	olomics Pc	0.46691	0.99951	1.2457	0.31699	0.28736	0.99998	0.83656	-0.25745	0.68682	0.99941	0.96547	-0.0507	0.006983	0.1948	0.67097	-0.57568	0.011496	0.054208	0.64019	-0.64343	0.007175	0.070405	0.6778	-0.56107	0.89796	0.9951	1.1254	0.17042	0.91283	0.99956	1.073	0.10167	0.053246	0.2951	1.655	0.72679
271.9667	0.61	omics Pos	0.46839	0.99987	1.2132	0.27881	0.6289	0.99994	0.89202	-0.16485	0.17787	0.99996	0.7316	-0.45087	0.84704	0.9874	0.97552	-0.03576	0.01621	0.044308	0.47966	-1.0599	0.37347	0.63049	0.80852	-0.30664	0.21373	0.65483	0.73032	-0.45339	0.62637	0.97813	0.89128	-0.16605	0.027385	0.11602	1.6768	0.74573
1192.8	12	olomics Pc	0.46879	0.99951	0.95274	-0.06985	0.22917	0.99998	0.90391	-0.14576	0.018443	0.99941	0.9245	-0.11326	0.64921	0.99905	0.97984	-0.02938	0.66442	0.99521	0.97305	-0.03942	0.27075	0.56295	0.92707	-0.10925	0.45158	0.99391	0.9524	-0.07036	0.031881	0.58535	0.90781	-0.13954	0.011298	0.2688	0.89117	-0.16623
174.0224	0.6	omics Pos	0.46915	0.99987	1.1469	0.1977	0.33126	0.99994	0.90965	-0.13662	0.89705	0.99996	1.0267	0.038072	0.52349	0.89938	0.96328	-0.05397	0.032568	0.075491	0.67839	-0.55982	0.35266	0.61857	0.93461	-0.09757	0.92001	0.981	0.99816	-0.00265	0.44664	0.96776	0.93514	-0.09675	0.008823	0.092495	1.3055	0.38457
688.4877	9.48	olomics Pc	0.46936	0.99951	1.2072	0.27172	0.18555	0.99998	0.85489	-0.22618	0.92509	0.99941	1.0086	0.012414	0.014396	0.20085	0.60967	-0.7139	0.002058	0.015776	0.55986	-0.83687	0.003366	0.049867	0.62305	-0.68259	0.51717	0.99391	0.90609	-0.14228	0.69601	0.99956	0.96704	-0.04836	0.11906	0.36992	1.6055	0.683
393.2324	1.66	omics Pos	0.46941	0.99987	1.0994	0.13671	0.13504	0.99994	0.74249	-0.42955	0.76242	0.99996	0.92453	-0.11321	0.075041	0.46303	0.60708	-0.72004	0.000247	0.001942	0.19922	-2.3275	0.007848	0.083822	0.48303	-1.0498	0.10555	0.55257	0.69753	-0.51967	0.19417	0.92582	0.76035	-0.39526	0.32204	0.43222	1.1453	0.1957
965.1301	9.14	olomics Pc	0.46948	0.99951	1.2238	0.29137	0.36374	0.99998	0.78879	-0.34229	0.89888	0.99941	0.94855	-0.07621	0.69218	0.99905	0.88969	-0.16863	0.047717	0.16251	0.58827	-0.76546	0.22969	0.51644	0.71828	-0.47738	0.77257	0.99391	0.91638	-0.12597	0.45475	0.99956	0.81284	-0.29895	0.51055	0.75895	1.2067	0.27106
354.3735	4.32	omics Pos	0.46961	0.99987	0.87846	-0.18696	0.29623	0.99994	0.82475	-0.27798	0.25196	0.99996	0.8101	-0.30383	0.24739	0.70693	0.82525	-0.27709	0.065167	0.12683	0.70154	-0.51141	0.017405	0.11287	0.58784	-0.76651	0.12926	0.59025	0.69174	-0.5317	0.063615	0.73915	0.72254	-0.46885	0.96915	0.97784	0.976	-0.03504
857.5856	5.23	omics Pos	0.46997	0.99987	1.0693	0.0966	0.72467	0.99994	0.96579	-0.05021	0.72081	0.99996	0.96747	-0.04772	0.56691	0.91222	0.95072	-0.07291	0.012773	0.036897	0.7639	-0.38855	0.35201	0.61857	0.87793	-0.18782	0.56796	0.85768	0.94836	-0.07649	0.61857	0.97751	0.9469	-0.07872	0.37594	0.48523	1.1382	0.18671
944.7531	10.08	omics Pos	0.47081	0.99987	1.1021	0.14028	0.88671	0.99994	1.0088	0.012598	0.18674	0.99996	1.1118	0.1529	0.67575	0.95843	0.96192	-0.05601	0.029228	0.06961	0.81951	-0.28717	0.60123	0.78399	0.95134	-0.07196	0.1844	0.64793	1.1105	0.15127	0.77795	0.97988	1.0454	0.064011	0.014843	0.098791	1.2767	0.3524
428.3728	5.4	olomics Pc	0.47096	0.99951	1.1531	0.20556	0.61475	0.99998	1.094	0.12963	0.84076	0.99941	0.99961	-0.00056	0.91088	0.99905	0.96836	-0.04638	0.031882	0.12104	0.48404	-1.0468	0.13479	0.36836	0.54636	-0.87208	0.082925	0.58568	0.51605	-0.95442	0.18578	0.98353	0.62193	-0.68517	0.14329	0.39507	0.63308	-0.65955
236.8498	0.46	olomics Ne	0.47135	0.99918	1.0966	0.13307	0.61226	0.9021	1.1561	0.20928	0.070269	0.99995	1.3121	0.39188	0.042715	0.99985	1.3806	0.46528	0.016277	0.88267	1.7041	0.769	0.25505	0.69038	1.4973	0.58234	0.027524	0.33786	1.5413	0.62416	0.070507	0.99512	1.4031	0.48866	0.51167	0.99941	1.1689	0.22513
962.7872	10.05	omics Pos	0.47167	0.99987	0.91708	-0.12487	0.19229	0.99994	0.87531	-0.19213	0.36125	0.99996	0.90624	-0.14204	0.12086	0.54142	0.81861	-0.28875	0.04289	0.09292	0.80484	-0.31322	0.3404	0.61059	0.89436	-0.16108	0.72893	0.92637	0.9607	-0.05784	0.61098	0.97371	1.0579	0.081183	0.36473	0.47356	1.1052	0.14427
414.301	0.76	omics Pos	0.47189	0.99987	0.94286	-0.08488	0.37389	0.99994	0.9219	-0.11731	0.17628	0.99996	2.2095	1.1437	0.071519	0.4548	4.6887	2.2292	0.013806	0.039286	5.8052	2.5374	0.01194	0.092554	6.5659	2.715	0.007584	0.32129	9.0105	3.1716	0.41888	0.96776	1.4468	0.53287	0.50528	0.60624	0.83407	-0.26176
227.9556	0.51	omics Pos	0.4723	0.99987	1.0212	0.030321	0.31068	0.99994	0.94739	-0.07797	0.091817	0.99996	0.91655	-0.12571	0.010586	0.21362	0.86395	-0.21097	7.18E-05	0.000834	0.72945	-0.45511	0.2453	0.53144	0.88453	-0.17702	0.22778	0.67223	0.92451	-0.11324	0.11803	0.88537	0.91998	-0.12032	0.067562	0.16764	1.1802	0.23904
409.284	1.89	omics Pos	0.4724	0.99987	1.1786	0.23708	0.67334	0.99994	0.9092	-0.13733	0.65826	0.99996	0.90767	-0.13976	0.15179	0.57995	0.69175	-0.53167	0.000121	0.00117	0.24931	-2.004	0.006747	0.080671	0.52977	-0.91657	0.29334	0.7186	0.81317	-0.29838	0.35355	0.96776	0.81966	-0.28691	0.043585	0.13895	1.4838	0.56932
674.2158	1.11	omics Pos	0.47241	0.99987	1.1244	0.16915	0.65047	0.99994	0.9143	-0.12926	0.35208	0.99996	1.1727	0.22988	0.87459	0.9874	1.045	0.063445	0.64795	0.70126	0.91429	-0.12927	0.055505	0.23283	1.5346	0.61784	0.12402	0.58314	1.4133	0.49909	0.018113	0.52955	1.5444	0.62708	0.11653	0.22095	1.3543	0.43756
570.264	1.3	omics Pos	0.47255	0.99987	0.97058	-0.04308	0.082744	0.99994	0.84483	-0.24326	0.43465	0.99996	0.97636	-0.03451	0.01212	0.22217	0.91952	-0.12104	0.000668	0.004067	0.71986	-0.47422	0.55422	0.75447	0.9554	-0.06582	0.84855	0.96798	0.99455	-0.00789	0.044919	0.65373	0.8436	-0.24537	0.43321	0.53929	1.0939	0.12952
133.9351	12.24	olomics Pc	0.47268	0.99951	0.95209	-0.07083	0.5554	0.99998	0.95558	-0.06555	0.032242	0.99941	0.85213	-0.23086	0.70583	0.99905	0.97024	-0.04358	0.35314	0.74159	0.93062	-0.10374	0.44218	0.74804	0.94045	-0.08857	0.24036	0.84407	0.9326	-0.10066	0.037066	0.61777	0.84439	-0.24402	0.63584	0.83442	0.96585	-0.05013
1040.631	6.06	omics Pos	0.47326	0.99987	0.91243	-0.13221	0.10954	0.99994	0.81916	-0.28778	0.12379	0.99996	0.83655	-0.25748	0.026844	0.29079	0.81774	-0.29028	0.03279	0.075922	0.73056	-0.45293	0.17908	0.46725	0.74214	-0.43025	0.19018	0.64891	0.88584	-0.17487	0.15538	0.91321	0.81068	-0.3028	0.40117	0.50856	0.91025	-0.13567
1074.615	1.29	omics Pos	0.47338	0.99987	1.1701	0.22662	0.97671	0.99994	0.99721	-0.00403	0.74818	0.99996	0.93495	-0.09704	0.92086	0.9874	1.0379	0.053682	0.22515	0.31914	0.71022	-0.49366	0.62943	0.80348	1.0814	0.11288	0.046268	0.39255	1.4778	0.56343	0.42491	0.96776	1.2296	0.29821	0.018893	0.10449	1.8713	0.90407
327.2198	4.86	omics Pos	0.47344	0.99987	0.90845	-0.13852	0.47047	0.99994	0.90158	-0.14948	0.51851	0.99996	0.90407	-0.14549	0.33495	0.79326	0.87776	-0.18811	0.021678	0.055476	0.68964	-0.53608	0.40896	0.65377	0.86062	-0.21654	0.16844	0.64016	0.84825	-0.23743	0.9744	0.99236	0.99019	-0.01423	0.70751	0.78172	1.092	0.12695
966.6694	6.03	omics Pos	0.47412	0.99987	1.0419	0.059193	0.54085	0.99994	0.95487	-0.06662	0.48922	0.99996	0.93487	-0.09716	0.32843	0.78974	0.89017	-0.16784	0.031779	0.074108	0.79456	-0.33177	0.36152	0.62318	0.90364	-0.14618	0.86964	0.97532	1.042	0.059393	0.24733	0.94365	1.0769	0.10688	0.069777	0.17098	1.2467	0.31812
395.2204	2.73	omics Pos	0.4742	0.99987	1.0777	0.10801	0.68257	0.99994	0.94766	-0.07756	0.091688	0.99996	0.80642	-0.3104	0.007008	0.19397	0.59666	-0.74502	0.002443	0.010471	0.48966	-1.0301	0.00837	0.084185	0.46406	-1.1076	0.022887	0.33809	0.56451	-0.82494	0.081286	0.8026	0.63854	-0.64716	0.1428	0.24751	0.75244	-0.41036
412.2473	2.72	omics Pos	0.47428	0.99987	0.93127	-0.10272	0.18687	0.99994	0.86539	-0.20857	0.01449	0.99996	0.72729	-0.4594	0.007357	0.19896	0.53854	-0.89289	0.000132	0.001244	0.4319	-1.2112	0.004958	0.073217	0.37829	-1.4024	0.011703	0.3324	0.49382	-1.0179	0.013315	0.52955	0.46846	-1.094	0.087761	0.18831	0.65612	-0.60797
303.908	10.15	omics Pos	0.47451	0.99987	1.0607	0.084953	0.099277	0.99994	0.86231	-0.21372	0.83556	0.99996	1.0196	0.028068	0.28869	0.75241	0.93045	-0.104	0.008412	0.026909	0.79313	-0.33438	0.31939	0.5964	0.92338	-0.115	0.9251	0.98262	1.0232	0.033072	0.45483	0.96776	0.93984	-0.08951	0.008982	0.092545	1.1836	0.24321
357.6804	1.29	omics Pos	0.47481	0.99987	1.0983	0.13522	0.50777	0.99994	1.2225	0.2898	0.72767	0.99996	1.0629	0.087965	0.89214	0.9874	0.9413	-0.08728	0.37751	0.46979	0.80469	-0.31349	0.74929	0.87057	1.0489	0.068877	0.20368	0.64891	1.2308	0.29954	0.040716	0.64308	1.5145	0.59887	0.048986	0.14579	1.6257	0.70104
787.6612	10.33	olomics Pc	0.47486	0.99951	0.93195	-0.10168	0.15484	0.99998	0.90869	-0.13814	0.22895	0.99941	0.88086	-0.18302	0.35692	0.84724	0.91675	-0.12539	0.65859	0.99209	0.97095	-0.04253	0.1824	0.44922	0.90544	-0.14332	0.025471	0.33018	0.82991	-0.26897	0.070062	0.76039	0.82891	-0.27072	0.11412	0.36506	0.82158	-0.28353
996.6451	3.99	omics Pos	0.47528	0.99987	1.1662	0.22186	0.085368	0.99994	0.79608	-0.32901	0.17574	0.99996	0.77186	-0.37359	0.3544	0.80823	0.8475	-0.23872	0.046203	0.098146	0.64502	-0.63258	0.19097	0.47749	0.79513	-0.33074	0.8809	0.97532	0.98702	-0.01885	0.5831	0.97073	0.92178	-0.11751	0.097116	0.19792	1.3759	0.46042
660.2368	1.11	omics Pos	0.4757	0.99987	1.1423	0.19199	0.97769	0.99994	1.0146	0.020957	0.53223	0.99996	1.1158	0.15808	0.48842	0.88725	1.117	0.15967	0.8837	0.90215	1.0309	0.043894	0.044164	0.19929	1.4206	0.50649	0.051017	0.41162	1.4429	0.52899	0.10567	0.86277	1.4015	0.48701	0.021495	0.1083	1.5516	0.63375
364.2175	6.01	olomics Pc	0.47595	0.99951	1.1522	0.20443	0.2256	0.99998	0.84537	-0.24235	0.82813	0.99941	1.0627	0.087772	0.32227	0.81801	0.84677	-0.23995	2.01E-05	0.00048	0.28123	-1.8302	0.022096	0.11005	0.61286	-0.70638	0.13312	0.68208	0.7516	-0.41197	0.74831	0.99956	0.95291	-0.06959	0.77874	0.90218	1.0232	0.033139
646.197	1.29	omics Pos	0.47671	0.99987	1.0397	0.056178	0.94193	0.99994	1.0077	0.011016	0.54775	0.99996	1.043	0.060776	0.32632	0.78936	0.94344	-0.084	0.037706	0.084672	0.8079	-0.30776	0.31877	0.5964	0.89564	-0.15902	0.86744	0.97532	0.99259	-0.01073	0.5422	0.96776	0.94877	-0.07587	0.34293	0.45293	1.1747	0.2323
398.7627	4.96	omics Pos	0.47698	0.99987	1.0578	0.081037	0.1004	0.99994	0.89387	-0.16186	0.22873	0.99996	0.95831	-0.06143	0.4599	0.87856	0.94761	-0.07763	0.000348	0.00247	0.75191	-0.41138	0.26126	0.54699	0.89578	-0.15878	0.91747	0.98047	1.015	0.02148	0.49675	0.96776	1.036	0.051051	0.060613	0.1578	1.2492	0.321

980.6792	9.11	olomics Po	0.47706	0.99951	1.1399	0.18896	0.57579	0.99998	0.90946	-0.13692	0.77662	0.99941	1.0283	0.040231	0.27763	0.75131	0.80659	-0.3101	0.024809	0.099287	0.69392	-0.52716	0.16878	0.42628	0.75223	-0.41075	0.77291	0.99391	0.95843	-0.06126	0.87663	0.99956	1.0061	0.008815	0.52774	0.76749	1.2285	0.29693
918.5861	8.93	olomics Po	0.47729	0.99951	1.2162	0.28236	0.5056	0.99998	0.91596	-0.12665	0.64119	0.99941	1.0589	0.08263	0.006049	0.1948	0.69388	-0.52724	0.059137	0.19008	0.68943	-0.53652	0.16189	0.41629	0.80477	-0.31335	0.81099	0.99391	1.1113	0.15227	0.81216	0.99956	0.98472	-0.02221	0.088285	0.34081	1.759	0.81476
634.8764	0.5	domics Pos	0.47743	0.99987	1.0247	0.035186	0.32767	0.99994	0.94512	-0.08144	0.22746	0.99996	0.93482	-0.09725	0.083032	0.4849	0.91221	-0.13257	0.000149	0.001364	0.75823	-0.39929	0.24464	0.53144	0.88281	-0.17983	0.58558	0.86163	0.96603	-0.04987	0.26199	0.94365	0.94644	-0.07942	0.029644	0.11849	1.193	0.25456
1180.773	0.5	domics Pos	0.47762	0.99987	1.0367	0.051974	0.43023	0.99994	0.95463	-0.06698	0.2747	0.99996	0.94786	-0.07725	0.45551	0.87441	0.96139	-0.0568	0.000764	0.004537	0.7455	-0.42372	0.43775	0.67081	0.93079	-0.10348	0.98496	0.99461	1.0076	0.010943	0.5668	0.9703	0.96184	-0.05613	0.008931	0.092545	1.2797	0.35586
92.9756	0.51	domics Pos	0.47783	0.99987	1.0869	0.12016	0.88102	0.99994	0.97158	-0.0416	0.90218	0.99996	0.993	-0.01013	0.29458	0.75244	0.86239	-0.21359	0.038691	0.086324	0.72648	-0.46101	0.22629	0.51391	0.83917	-0.25297	0.75957	0.94108	1.0242	0.034471	0.31536	0.9524	0.86969	-0.20143	0.67944	0.76079	1.046	0.064856
332.8399	9.92	domics Pos	0.47889	0.99987	0.94837	-0.07648	0.12127	0.99994	0.86328	-0.2121	0.01412	0.99996	0.82937	-0.26992	0.002779	0.17581	0.78457	-0.35002	0.011534	0.03423	0.71182	-0.49041	0.2265	0.51408	0.83623	-0.25802	0.22603	0.66988	0.88521	-0.1759	0.018147	0.52955	0.78178	-0.35516	0.48344	0.58575	1.1011	0.13889
478.8443	10.11	domics Pos	0.47902	0.99987	0.91675	-0.1254	0.52836	0.99994	0.95928	-0.05998	0.3996	0.99996	1.0702	0.097925	0.34188	0.7973	0.92862	-0.10684	7.44E-05	0.000851	0.73739	-0.4395	0.25691	0.54373	0.82942	-0.26982	0.63236	0.8822	0.95358	-0.06858	0.66087	0.97988	1.0331	0.046953	0.11712	0.22152	1.1955	0.25757
809.4983	1.48	domics Pos	0.47938	0.99987	1.4495	0.53553	0.4562	0.99994	0.91068	-0.13498	0.12564	0.99996	1.2641	0.33808	0.088067	0.49455	0.71225	-0.48954	0.006099	0.021142	0.57945	-0.78725	0.047778	0.21183	0.75939	-0.39709	0.31886	0.72948	1.3166	0.39685	0.50139	0.96776	1.3603	0.44395	0.02849	0.11732	1.8957	0.92276
368.3881	4.66	domics Pos	0.47951	0.99987	1.0507	0.071379	0.3024	0.99994	0.85928	-0.2188	0.39945	0.99996	0.8962	-0.1581	0.23439	0.69282	0.88486	-0.17648	0.12282	0.20362	0.85911	-0.21909	0.002503	0.061655	0.64144	-0.64061	0.069514	0.46982	0.7607	-0.39459	0.046462	0.66351	0.79756	-0.32634	0.46158	0.56545	1.081	0.11239
980.7637	10.07	domics Pos	0.47963	0.99987	1.067	0.09356	0.48333	0.99994	0.94476	-0.08198	0.066098	0.99996	0.87576	-0.19139	0.69614	0.96092	0.96784	-0.04716	0.008449	0.027005	0.81122	-0.30183	0.29821	0.57957	0.92446	-0.11332	0.51012	0.83279	1.0521	0.073329	0.89934	0.98023	0.99221	-0.01128	0.18351	0.2907	1.1328	0.17985
768.5883	5.17	domics Pos	0.48022	0.99987	1.0661	0.092339	0.99035	0.99994	1.0002	0.000349	0.97983	0.99996	1.0027	0.003938	0.2899	0.75244	0.93026	-0.10429	0.011536	0.03423	0.79418	-0.33246	0.59078	0.77705	0.95509	-0.0663	0.56786	0.85768	1.0677	0.094482	0.88147	0.98023	0.98971	-0.01492	0.21359	0.32255	1.0965	0.13295
778.81	0.52	domics Pos	0.48174	0.99987	1.1197	0.16313	0.13156	0.99994	0.89889	-0.15379	0.16835	0.99996	0.9029	-0.14737	0.084132	0.48766	0.87437	-0.19369	0.000984	0.005476	0.69083	-0.53359	0.32634	0.60111	0.92456	-0.11317	0.74778	0.93404	1.0309	0.04387	0.6557	0.97988	1.0738	0.10273	0.001897	0.078631	1.2316	0.30058
373.2092	6.32	olomics Po	0.48185	0.99951	0.84906	-0.23607	0.35044	0.99998	0.79025	-0.33962	0.009599	0.99941	0.53743	-0.89584	0.051849	0.2975	0.50287	-0.99174	0.011662	0.054911	0.44072	-1.1821	0.002498	0.046224	0.30056	-1.7343	0.019613	0.30136	0.44123	-1.1804	0.020103	0.51556	0.37996	-1.3961	0.012484	0.2688	0.43105	-1.2141
526.8482	10.12	domics Pos	0.48301	0.99987	1.0949	0.13073	0.68607	0.99994	1.0394	0.055776	0.24615	0.99996	0.87161	-0.19824	0.14498	0.57187	0.80597	-0.31119	0.003676	0.014345	0.69504	-0.52483	0.17494	0.46151	0.86188	-0.21444	0.72963	0.92644	1.0754	0.10488	0.51309	0.96776	0.92575	-0.11131	0.23953	0.34753	1.1236	0.16818
377.2185	6.96	olomics Po	0.48396	0.99951	0.88133	-0.18225	0.42272	0.99998	0.86684	-0.20617	0.054155	0.99941	0.68641	-0.54286	0.083264	0.38122	0.67809	-0.56045	0.005901	0.033133	0.56849	-0.8148	0.011436	0.083709	0.48084	-1.0564	0.008636	0.23862	0.55101	-0.85985	0.013828	0.47452	0.51973	-0.94418	0.039104	0.28339	0.57983	-0.7863
1016.791	0.5	domics Pos	0.48425	0.99987	1.0634	0.08868	0.11833	0.99994	0.87951	-0.18523	0.005027	0.99996	0.87043	-0.2002	0.080248	0.47885	0.90739	-0.14021	0.001629	0.007764	0.75976	-0.39638	0.53486	0.74053	0.95291	-0.06958	0.27124	0.70502	0.92256	-0.11628	0.26472	0.94365	0.9554	-0.06582	0.00531	0.088007	1.1588	0.21268
1199.657	0.57	domics Pos	0.48427	0.99987	1.1108	0.15164	0.1546	0.99994	0.86433	-0.21034	0.074677	0.99996	0.87807	-0.1876	0.001268	0.17581	0.62332	-0.68196	2.86E-05	0.000459	0.48019	-1.0583	0.000262	0.026314	0.58636	-0.77015	0.66862	0.89786	0.96386	-0.0531	0.65031	0.97988	0.96288	-0.05457	0.009266	0.092545	1.6854	0.75313
431.2766	2.72	domics Pos	0.48485	0.99987	1.0932	0.12862	0.43512	0.99994	0.87406	-0.19419	0.037436	0.99996	0.72779	-0.4584	0.010588	0.21362	0.61876	-0.69254	0.001973	0.008971	0.49604	-1.0115	0.027791	0.14738	0.44519	-1.1675	0.027043	0.34512	0.52211	-0.93759	0.014719	0.52955	0.47074	-1.087	0.096155	0.1974	0.67016	-0.57742
765.4771	1.51	domics Pos	0.4851	0.99987	1.3254	0.40641	0.28815	0.99994	0.82357	-0.28005	0.42479	0.99996	1.1926	0.25417	0.040892	0.34959	0.65759	-0.60475	0.041189	0.090005	0.65271	-0.61549	0.071936	0.27595	0.71248	-0.48909	0.74106	0.93154	1.1345	0.18201	0.8827	0.98023	1.0997	0.13709	0.17019	0.27791	1.6488	0.7214
233.2268	4.73	domics Pos	0.48529	0.99987	1.0827	0.11458	0.45279	0.99994	0.94369	-0.08362	0.67888	0.99996	1.0294	0.041846	0.71307	0.96346	0.96934	-0.04493	0.014733	0.041397	0.79341	-0.33386	0.96771	0.98309	0.99459	-0.00782	0.52207	0.83957	1.045	0.063553	0.89048	0.98023	0.99484	-0.00746	0.18206	0.28944	1.2772	0.35294
120.0753	0.59	domics Pos	0.48592	0.99987	1.0513	0.072155	0.088919	0.99994	0.88343	-0.17881	0.12895	0.99996	1.0966	0.1331	0.014133	0.22898	1.3423	0.42469	0.33477	0.42834	0.92311	-0.11543	0.074967	0.28246	1.1119	0.15298	0.000626	0.17164	1.4589	0.54486	0.81581	0.97988	0.9874	-0.01829	0.13067	0.2358	1.2702	0.34507
540.4469	6.56	olomics Po	0.48595	0.99951	0.94274	-0.08507	0.56869	0.99998	0.95612	-0.06474	0.040842	0.99941	0.85156	-0.23183	0.83562	0.99905	0.99153	-0.01227	0.80992	0.99688	1.0309	0.043839	0.6639	0.89948	1.1728	0.22996	0.2761	0.89515	1.2328	0.30198	0.34608	0.99956	0.92278	-0.11594	0.67219	0.8507	1.0658	0.091904
362.9273	0.5	domics Pos	0.48713	0.99987	1.0217	0.03095	0.18844	0.99994	0.92952	-0.10545	0.22283	0.99996	0.93479	-0.09729	0.053332	0.39717	0.89976	-0.15239	0.000105	0.001067	0.74605	-0.42265	0.2913	0.57461	0.89321	-0.16294	0.54049	0.84601	0.96313	-0.05419	0.22	0.94047	0.94229	-0.08576	0.033538	0.12517	1.187	0.24732
933.541	5.86	domics Pos	0.48776	0.99987	1.0797	0.11064	0.68009	0.99994	1.0577	0.080935	0.75372	0.99996	1.0253	0.036099	0.72869	0.96895	0.97094	-0.04255	0.030338	0.071406	0.83897	-0.25331	0.46146	0.68833	0.9333	-0.09959	0.98084	0.99431	1.0001	0.000173	0.75942	0.97988	1.041	0.057986	0.004386	0.085451	1.3743	0.4587
936.6984	0.56	domics Pos	0.48842	0.99987	1.0685	0.095599	0.76889	0.99994	0.97646	-0.03437	0.14057	0.99996	0.90234	-0.14826	0.048201	0.37739	0.77558	-0.36666	0.33883	0.43198	0.90461	-0.14463	0.1207	0.37754	0.81204	-0.30039	0.031103	0.35811	0.77645	-0.36504	0.25061	0.94365	0.87007	-0.20079	0.022206	0.1085	1.2696	0.34436
1143.747	10.06	domics Pos	0.48847	0.99987	1.0628	0.087937	0.79459	0.99994	0.97606	-0.03496	0.43277	0.99996	0.92785	-0.10804	0.47818	0.88482	0.9205	-0.11951	0.00276	0.011487	0.72191	-0.4701	0.41056	0.65377	0.92519	-0.11218	0.73624	0.92956	0.97131	-0.04199	0.77122	0.97988	1.0378	0.05346	0.7838	0.83916	1.027	0.038431
856.5821	5.23	domics Pos	0.48867	0.99987	1.07	0.097613	0.74294	0.99994	0.96834	-0.04642	0.80424	0.99996	0.97664	-0.0341	0.66388	0.95717	0.9617	-0.05634	0.013272	0.03805	0.76821	-0.38043	0.38028	0.63793	0.88664	-0.17357	0.6449	0.88775	0.95807	-0.0618	0.67236	0.97988	0.95659	-0.06403	0.32383	0.43417	1.1488	0.20013
472.3039	0.77	domics Pos	0.48924	0.99987	0.88974	-0.16855	0.39835	0.99994	0.89932	-0.15309	0.098044	0.99996	2.6768	1.4205	0.048915	0.37885	4.7237	2.2399	0.013076	0.03763	5.7408	2.5212	0.009153	0.086817	7.3595	2.8796	0.006362	0.3088	9.8402	3.2987	0.51713	0.96776	1.512	0.59646	0.4291	0.53539	0.90763	-0.13983
1118.768	0.5	domics Pos	0.49007	0.99987	1.059	0.082666	0.46062	0.99994	0.92617	-0.11065	0.31777	0.99996	0.89917	-0.15333	0.69788	0.96092	0.96895	-0.04551	0.004545	0.016941	0.78898	-0.34194	0.7531	0.87185	0.97493	-0.03663	0.8813	0.97532	0.98925	-0.01559	0.39196	0.96776	0.949	-0.07552	0.013786	0.098791	1.2309	0.29967
887.6119	5.65	domics Pos	0.49023	0.99987	1.0752	0.10456	0.92614	0.99994	0.98427	-0.02287	0.89668	0.99996	1.0235	0.033566	0.89949	0.9874	0.98639	-0.01977	0.17162	0.2628	0.77944	-0.35948	0.049469	0.21656	0.75096	-0.41319	0.13865	0.60403	0.80836	-0.30694	0.62313	0.97813	0.93088	-0.10333	0.37776	0.48666	1.22	0.28684
572.8334	0.54	domics Pos	0.49032	0.99987	0.93771	-0.09279	0.12569	0.99994	0.83527	-0.25968	0.090419	0.99996	0.86712	-0.2057	0.073344	0.45875	0.84183	-0.2484	0.005299	0.019043	0.77649	-0.36496	0.15808	0.43961	0.84709	-0.23941	0.20294	0.64891	0.91437	-0.12915	0.3243	0.95459	0.9024	-0.14816	0.34705	0.45774	1.0881	0.12177
453.172	0.75	domics Pos	0.49064	0.99987	1.153	0.20534	0.17992	0.99994	0.78173	-0.35525	0.84229	0.99996	0.96239	-0.05531	0.86185	0.9874	1.0424	0.059896	0.024444	0.06064	0.62686	-0.67378	0.54193	0.74437	0.89175	-0.16528	0.68346	0.90456	0.93126	-0.10274	0.49127	0.96776	0.88934	-0.1692	0.12427	0.22879	1.3116	0.39128
687.5426	3.91	domics Pos	0.49089	0.99987	0.90437	-0.14501	0.57394	0.99994	0.92581	-0.11122	0.51982	0.99996	0.90792	-0.13937	0.089999	0.50079	0.76368	-0.38896	0.040037	0.088289	0.75478	-0.40587	0.39574	0.64402	0.89462	-0.16066	0.20741	0.65208	0.84439	-0.24402	0.65894	0.97988	0.94122	-0.0874	0.45657	0.56159	0.89943	-0.15292
888.509	4.78	domics Pos	0.49103	0.99987	1.0365	0.051667	0.68054	0.99994	0.96455	-0.05207	0.78408	0.99996	0.98227	-0.0258	0.96058	0.9937	1.0037	0.005306	0.1202	0.20024	0.81309	-0.29852	0.36653	0.62672	0.91579	-0.12692	0.82432	0.9618	0.98847	-0.01673	0.048387	0.67624	1.1281	0.17385	0.10308	0.20579	1.1108	0.15161
832.0176	1.46	domics Pos	0.49125	0.99987	1.2657	0.33994	0.27023	0.99994	0.81219	-0.3001	0.72915	0.99996	1.0734	0.10216	0.043643	0.36496	0.63723	-0.65012	0.047264	0.099844	0.58908	-0.76347	0.076521	0.2869	0.72004	-0.47385	0.76143	0.94108	1.1109	0.15168	0.98726	0.99578	1.0291	0.041332	0.10725	0.20999	1.712	0.77568
341.1714	6.47	olomics Po	0.49146	0.99951	1.0804	0.11153	0.86498	0.99998	1.003	0.004339	0.16748	0.99941	0.85323	-0.22899	0.16358	0.57278	0.7444	-0.42585	0.004161	0.0257	0.53605	-0.89956	0.022014	0.11005	0.5963	-0.74588	0.000123	0.16989	0.4718	-1.0838	0.009848	0.47452	0.55738	-0.84327	0.064377	0.30632	0.6495	-0.6226
220.9351	10.18	domics Pos	0.49218	0.99987	1.031	0.044003	0.12872	0.99994	0.87919	-0.18575	0.36466	0.99996	0.93817	-0.09209	0.13263	0.55697	0.90642	-0.14174	0.001002	0.005526	0.73381	-0.44652	0.27294	0.55982	0.88976	-0.16851	0.69122	0.90976	0.9728	-0.03979	0.059319	0.72218	0.88241	-0.18048	0.08552	0.18633	1.1267	0.17215
279.2497	1.4	domics Pos	0.49245	0.99987	1.0903	0.1247	0.037854	0.99994	0.80961	-0.3047	0.7509	0.99996	1.0636	0.088966	0.084339	0.48766	0.71372	-0.48657	1.42E-06	0.000144	0.22513	-2.1511	0.001027	0.043236	0.5886	-0.76463	0.24708	0.68479	0.83967	-0.25211	0.19053	0.92582	0.87415	-0.19404	0.026611	0.11491	1.2497	0.32158
813.8137	0.54	domics Pos	0.49473	0.99987	1.0735	0.10226	0.93041	0.99994	0.99604	-0.00573	0.14415	0.99996	0.88648	-0.17384	0.37991	0.82872	0.9303	-0.10422	0.001835	0.008483	0.70095	-0.51262	0.36944	0.62855	0.90393	-0.14571	0.58885	0.86163	1.0482	0.067895	0.86689	0.98023	1.0383	0.05427	0.2565	0.36562	1.1516	0.20369
1052.489	5.91	domics Pos	0.49476	0.99987	1.0685	0.095633	0.087411	0.99994	1.1051	0.14422	0.77199	0.99996	1.0315	0.044797	0.28801	0.75229	1.1797	0.23848	0.002034	0.009165	0.80119	-0.31978	0.7717	0.88192	0.98182	-0.02648	0.47066	0.80647	1.1453	0.19566	0.008673	0.52955	1.2415	0.31211	0.21243	0.32116	1.2336	0.30289
171.9864	10.65	olomics Ne	0.49493	0.99918	0.92593	-0.11102	0.11954	0.5367	0.81661	-0.29228	0.82872	0.99995	0.98862	-0.01652	0.056606	0.99985	0.75234	-0.41055	0.18221	0.99927	0.88616	-0.17436	0.16803	0.63987	0.79005	-0.33998	0.046137	0.38697	1.1149	0.15697	0.38923	0.99512	0.91177	-0.13326	0.44331	0.99941	0.91382	-0.13002
273.2573	4.73	domics Pos	0.49542	0.99987	0.94941	-0.0749	0.21609	0.99994	0.906	-0.14241	0.46606	0.99996	0.94409	-0.08301	0.74235	0.97237	0.97729	-0.03314	0.002376	0.010305	0.77349	-0.37055	0.87492	0.93553	0.99546	-0.00656	0.49539	0.82378	0.9574	-0.06281	0.88338	0.98023	0.99956	-0.00064	0.30467	0.41416	1.1335	0.18082
771.8527	0.5	domics Pos	0.4961	0.99987	1.0207	0.029489	0.14934	0.99994	0.9125	-0.1321	0.66089	0.99996	0.97728	-0.03315	0.022993	0.26976	0.9165	-0.1258	6.6E-05	0.000784	0.74402	-0.42658	0.2245	0.51305	0.87365	-0.19487	0.53745	0.84423	0.96813	-0.04673	0.54717	0.96776	0.97071	-0.04289	0.013453	0.098791	1.1981	0.2608
1187.861	9.65	olomics Po	0.49702	0.99951	1.226	0.29396	0.27429	0.99998	0.85672	-0.2231	0.77703	0.99941	0.96296	-0.05446	0.008541	0.19702	0.65272	-0.61546	0.005174	0.030068	0.62569	-0.67648	0.03596	0.14944	0.71931	-0.47531	0.68741	0.99391	0.95719	-0.06312	0.8494	0.99956	1.1112	0.15213	0.13397	0.38786	1.5392	0.62221
157.1427	5.99	olomics Po	0.49897	0.99951	1.049	0.069027	0.16288	0.99998	0.81888	-0.28827	0.49551	0.99941	0.92979	-0.10503	0.10237	0.42769	0.70884	-0.49647	3.69E-05	0.000731	0.27818	-1.8459	0.012785	0.085685	0.61554	-0.70007	0.14183	0.70661	0.80543	-0.31216	0.15964	0.93927	0.84247	-0.2473	0.32681	0.60317	1.0969	0.13339
927.8128	10.02	domics Pos	0.49913	0.99987	1.0986	0.13564	0.87084	0.99994	1.0088	0.012659	0.67605	0.99996	1.0475	0.066883	0.58758	0.92519	0.93232	-0.1011	0.033583	0.077327	0.76254	-0.39111	0.99837	0.99889	0.99866	-0.00193	0.37199	0.76056	1.09	0.12428	0.78571	0.97988	1.0331	0.047032	0.37605	0.48523	1.1076	0.14745
686.438	5.61	domics Pos	0.50005	0.99987	1.1242	0.16889	0.26708	0.99994	0.92023	-0.11994	0.70536	0.99996	0.9654	-0.0508	0.2093	0.65684	0.86967	-0.20146	0.048978	0.10267	0.73542	-0.44336	0.10279	0.34081	0.79777	-0.32595	0.64475	0.88775	1.0775	0.10768	0.10511	0.86277	0.84004	-0.25147	0.26015	0.36972	1.1262	0.17142
707.2088	1.06	domics Pos	0.50038	0.99987	1.1952	0.2573	0.11616	0.99994	1.1994	0.2623	0.021475	0.99996	1.2214	0.28855	0.2323	0.68984	1.1328	0.17985	0.044434	0.095451	1.1667	0.22245	0.037622	0.17738	1.4296	0.51558	0.00063	0.17164	1.6101	0.68716	0.041389	0.64308	1.3321	0.41365	0.002552	0.082646	1.5774	0.65755
975.8153	10.02	domics Pos	0.5004	0.99987	1.016	0.022864	0.25223	0.99994	0.9313	-0.10268	0.061433	0.99996	0.94427	-0.08274	0.3367	0.79511	0.93992	-0.0894	3.33E-06	0.000144	0.76821	-0.38044	0.3868	0.63973	0.93607	-0.09531	0.91449	0.98003	1.0156	0.022264	0.4489	0.96776	0.96088	-0.05757	0.001435	0.07528	1.1942	0.25605
1180.895	9.65	olomics Po	0.50068	0.99951	1.2806	0.35681	0.17818	0.99998	0.80621	-0.31077	0.51676	0.99941	1.0902	0.12455	0.039362	0.27316	0.63956	-0.64485	0.012049	0.056144	0.56726	-0.81791	0.013169	0.085844	0.59494	-0.74918	0.46148	0.99391	0.87836	-0.18711	0.69283	0.99956	1.1484	0.19965	0.17842	0.44185	1.6331	0.70761
83.0864	1.67	domics Pos	0.50092	0.99987	1.0528	0.074185	0.1753	0.99994	0.84342	-0.24568	0.89811	0.99996	1.0024	0.003434	0.039907	0.34602	0.72508	-0.46379	7.68E-06	0.000195	0.2235	-2.1616	0.000658	0.03855	0.51881	-0.94671	0.042404	0.38314	0.76705	-0.38261	0.068738	0.76386	0.85967	-0.21814	0.001389	0.07528	1.3401	0.42229
187.9389	10.47	olomics Ne	0.50148	0.99918	0.94191	-0.08633	0.42036	0.80445	0.95877	-0.06074	0.64891	0.99995	1.0234	0.033351	0.75657	0.99985	1.0137	0.019676	0.37955	0.99927	1.0694	0.096773	0.27982	0.7073	1.0752	0.1046	0.020922	0.30301	1.1481	0.19924	0.94376	0.99512	1.0049	0.00708	0.65151	0.99941	0.98036	-0.02862
589.8492	10.1	domics Pos	0.50157	0.99987	0.94212	-0.08601	0.19997	0.99994	0.85925	-0.21885	0.72693	0.99996	0.96563	-0.05046	0.14138	0.56936	0.83408	-0.26174	0.007524	0.024688	0.71834	-0.47726	0.58445	0.77395	0.94937	-0.07496	0.42918	0.79094	1.0542	0.0762	0.6516	0.97988	0.95555	-0.0656	0.15112	0.25645	1.1409	0.19021
755.5754	5.72	domics Pos	0.50245	0.99987	1.0816	0.11316	0.49005	0.99994	0.90482	-0.1443	0.55127	0.99996	0.9132	-0.131	0.28639	0.75156	0.84117	-0.24952	0.25889	0.35407	0.83105	-0.267	0.55775	0.7572	1.0599	0.083984	0.029994	0.35536	1.3693	0.45339	0.72528	0.97988	1.0538	0.075597	0.07914	0.18102	1.2781	0.35405
993.7796	10.05	domics Pos	0.50296	0.99987	1.0709	0.098862	0.54622	0.99994	0.94297	-0.08472	0.50121	0.99996	0.94927	-0.07511	0.20498	0.65191	0.88455	-0.17699	0.003209	0.012919	0.7339	-0.44634	0.30337	0.58275	0.90044	-0.1513	0.37812	0.76056	0.9056	-0.14305	0.85983	0.98023	0.99157	-0.01221	0.46411	0.56734	1.0849	0.1176
615.7667	2.62	olomics Po	0.50323	0.99951	1.1829	0.24228	0.85382	0.99998	0.90248	-0.14803	0.19015	0.99941	0.64775	-0.62648	0.087844	0.39517	0.45126	-1.148	0.000562	0.006154	0.16238	-2.6225	0.07536	0.24549	0.47014	-1.0889	0.10545	0.62085	0.54625	-0.87237	0.080007	0.78488	0.53283	-0.90825	0.65008	0.83981	1.2501	0.32205
395.7842	5.16	domics Pos	0.50325	0.99987	1.0617	0.086385	0.30254	0.99994	0.91371	-0.1302	0.92443	0.99996	1.0068	0.009789	0.52575	0.89938	0.95424	-0.06758	0.002444	0.010471	0.70668	-0.50086	0.7271	0.85765	0.97355	-0.03867	0.51363	0.83481	0.9255	-0.11169	0.12872	0.88537	1.1508	0.20261	0.14865	0.25383	1.1517	0.2038
387.1672	6.32	olomics Po	0.50355	0.99951	0.89335	-0.1627	0.099289	0.99998	0.76458	-0.38726	0.15018	0.99941	0.74768	-0.4195	0.068872	0.34345	0.60937	-0.71461	0.006062	0.033618	0.458	-1.1266	0.00317	0.049867	0.36841	-1.4406	0.02628	0.3338	0.50136	-0.99609	0.010119	0.47452	0.46616	-1.1011	0.011538	0.2688	0.49036	-1.0281
209.1897	6.97	olomics Po	0.50412	0.99951	1.1179	0.16084	0.81267	0.99998	0.97179	-0.04129	0.048172	0.99941	0.71174	-0.49058	0.030776	0.24688	0.53235	-0.90955	0.001211	0.010796	0.40636	-1.2992	0.001192	0.044437	0.3462	-1.5303	0.00207	0.18112	0.44391	-1.1717	0.040404	0.63934	0.50429	-0.98767	0.019015	0.2688	0.56654	-0.81974
102.9652	0.32	olomics Ne	0.50424	0.99918	0.94288	-0.08486	0.068413	0.50118	0.83996	-0.25161	0.1335	0.99995	0.88395	-0.17796	0.10942	0.99985	0.87629	-0.19052	0.21433	0.99927	0.898	-0.15521	0.012451	0.40245	0.80665	-0.30999	0.14182	0.52742	0.90727	-0.1404	0.036058	0.99512	0.80994	-0.30412	0.019238	0.99941	0.80472	-0.31345
1187.751	0.51	domics Pos	0.5044	0.99987	0.94499	-0.08163	0.33631	0.99994	0.90304	-0.14713	0.090768	0.99996	0.86307	-0.21246	0.061025	0.42617	0.84512	-0.24276	0.00715	0.023749	0.74093	-0.43259	0.099306	0.33539	0.80182	-0.31865	0.26655	0.70012	0.89515	-0.1598	0.13663	0.89649	0.8284	-0.2716	0.97005	0.97826	0.99331	-0.00969
1094.728	9.04	olomics Po	0.50503	0.99951	1.2497	0.32157	0.64791	0.99998	0.96005	-0.05881	0.45053	0.99941	1.1092	0.14951	0.013331	0.20085	0.6888	-0.53784	0.022156	0.09123	0.65805	-0.60373	0.10809	0.31725	0.79375	-0.33325	0.69722	0.99391	1.1626	0.21739	0.56254	0.99956	1.1269	0.17238	0.076838	0.32186	1.5083	0.59295
791.5427	9.11	olomics Ne	0.50507	0.99918	0.96092	-0.05752	0.53206	0.86583	0.93116	-0.1029	0.96158	0.99995	1.0007	0.001069	0.72711	0.99985	0.97801	-0.03209	0.033951	0.99927	0.82863	-0.2712	0.45011	0.82254	0.93999	-0.08929	0.86823	0.95742	1.0403	0.05699	0.51884	0.99512	1.077	0.10703	0.4017	0.99941	1.1588	0.21259
320.0208	0.62	domics Pos	0.50513	0.99987	1.1313	0.17798	0.12845	0.99994	0.87019	-0.2006	0.82521	0.99996	0.97597	-0.03509	0.33304	0.79293	0.89103	-0.16645	0.002465	0.010537	0.59662	-0.74512	0.06565	0.25886	0.78024	-0.35801	0.79493	0.95091	0.97249	-0.04024	0.67369	0.97988	1.0269	0.038278	0.73952	0.80589	1.0401	0.056699
176.1555	4.73	domics Pos	0.50737	0.99987	0.94602	-0.08006	0.36423	0.99994	0.9479	-0.0772	0.87752	0.99996	0.99138	-0.01249	0.9666	0.99502	1.0052	0.007445	0.00372	0.014474	0.79803	-0.32549	0.21982	0.50985	1.0818	0.11349	0.1089	0.55771	1.1034	0.14201	0.47777	0.96776	1.1089	0.14912	0.084627	0.186	1.4195	0.50535
772.5281	8.77	olomics Ne	0.5075	0.99918	1.073	0.10162	0.99003	0.99664	1.0491	0.069192	0.72284	0.99995	1.0668	0.093333	0.61539	0.99985	1.0984	0.13537	0.049512	0.99927	1.1541	0.2068	0.1699	0.63987	1.2452	0.31634	0.00066	0.22316	1.3172	0.39745	0.11992	0.99512	1.1874	0.24777	0.10556	0.99941	0.91025	-0.13566
127.1464	8.13	olomics Po	0.50808	0.99951	0.93959	-0.0899	0.49058	0.99998	0.94274	-0.08507	0.32976	0.99941	0.92554	-0.11163	0.91222	0.99905	1.0099	0.014142	0.87816	0.99688	1.0229	0.03267	0.015773	0.093567	0.81553	-0.2942	0.46686	0.99391	0.93452	-0.0977	0.034024	0.59875	0.81516	-0.29485	0.76675	0.90005	1.0199	0.028496
300.9897	12	olomics Ne	0.50849	0.99918	1.0897	0.12399	0.014047	0.37315	0.88412	-0.17768	0.82499	0.99995	0.98894	-0.01605	0.67651	0.99985	1.0383	0.054182	0.87209	0.99927	1.0474	0.066774	0.64499	0.92263	1.0521	0.073296	0.55895	0.84988	1.027	0.038375	0.83041	0.99512	0.99148	-0.01234	0.74833	0.99941	0.97877	-0.03097
222.8807	9.9	domics Pos	0.50909	0.99987	0.91526	-0.12775	0.51062	0.99994	0.91233	-0.13238	0.66781	0.99996	0.94216	-0.08595	0.92173	0.9874	0.98355	-0.02392	0.043659	0.094266	0.75934	-0.39718	0.703	0.84729	1.0649	0.090666	0.30546	0.7283	1.1301	0.17651	0.57463	0.9703	1.0941	0.12976	0.14398	0.24827	1.2874	0.36443
703.5747	4.66	domics Pos	0.50934	0.99987	1.0489	0.06891	0.24011	0.99994	0.88941	-0.16907	0.74531	0.99996	0.97094	-0.04254	0.52231	0.89938	0.94207	-0.0861	0.026954	0.065442	0.78679	-0.34595	0.28972	0.57347	0.87399	-0.19431	0.96942	0.99319	0.99891	-0.00158	0.15707	0.9146	0.86209	-0.2141	0.97913	0.98379	0.99399	-0.00869
298.3096	7.12	olomics Po	0.50943	0.99951	0.94136	-0.08719	0.15926	0.99998	0.82625	-0.27535	0.11976	0.99941	0.81639	-0.29267	0.30563	0.79639	0.91758	-0.1241	0.56684	0.94863	0.9522	-0.07066	0.003747	0.052849	0.68256	-0.55098	0.12607	0.66647	0.77734	-0.36339	0.020243	0.51556	0.75897	-0.39788	0.18122	0.44585	0.87345	-0.19521
1091.245	9.03	olomics Po	0.51008	0.99951	1.1735	0.23076	0.43061	0.99998	0.8944	-0.161	0.93016	0.99941	1.0386	0.054647	0.016088	0.20428	0.70672	-0.50078	0.046936	0.16036	0.67631	-0.56424	0.068531	0.23147	0.77167	-0.37395	0.51343	0.99391	1.2482	0.31987	0.53297	0.99956	1.2056	0.26973	0.073626	0.3177	1.597	0.67535
784.8802	12.02	olomics Po	0.5102	0.99951	0.95196	-0.07103	0.010881	0.99998	0.71445	-0.48509	0.1532	0.99941	0.91191	-0.13304	0.93366	0.99905	0.99898	-0.00147	0.069686	0.21385	0.86495	-0.2093	0.30422	0.60645	0.90696	-0.14088	0.77136	0.99391	0.98383	-0.02352	0.021102	0.51556	0.80169	-0.31889	0.16104	0.41855	0.88043	-0.18372
313.2711	6.47	olomics Po	0.51088	0.99951	0.89471	-0.1605	0.070695	0.99998	0.71904	-0.47586	0.060874	0.99941	0.73529	-0.44361	0.028568	0.24036	0.68172	-0.55274	0.12454	0.34393	0.71638	-0.48121	0.10988	0.31872	0.70955	-0.49503	0.2583	0.86879	0.80291	-0.31669	0.048791	0.67731	0.7194	-0.47513	0.81597	0.92407	1.0513	0.072234
259.1925	6.4	olomics Po	0.51221	0.99951	0.84979	-0.23483	0.4119	0.99998	0.8203	-0.28577	0.02919	0.99941	0.58519	-0.77303	0.11073	0.45179	0.68135	-0.55353	0.026392	0.10405	0.55912	-0.83876	0.046294	0.17633	0.59039	-0.76025	0.16912	0.74367	0.71647	-0.48103	0.045843	0.66241	0.58722	-0.76802	0.024827	0.2688	0.53273	-0.90852
1058.77	0.5	domics Pos	0.51223	0.99987	1.137	0.18518	0.53937	0.99994	0.93194	-0.1017	0.54784	0.99996	0.94327	-0.08425	0.98871	0.99652	1.0036	0.005189	0.021353	0.055014	0.75984	-0.39623	0.97147	0.98437	1.0047	0.006744	0.2652	0.69979	1.1233	0.16781	0.83812	0.97988	1.023	0.032777	0.040226	0.13378	1.2006	0.26374
329.248	2.04	domics Pos	0.51231	0.99987	0.89058	-0.16719	0.24259	0.99994	0.78378	-0.35147	0.010987	0.99996	0.60783	-0.71826	0.011582	0.21754	0.46737	-1.0974	0.009275	0.029133	0.49138	-1.0251	0.007731	0.082799	0.3044	-1.7159	0.041988	0.38314	0.43986	-1.1849	0.011519	0.52955	0.38115	-1.3916	0.012879	0.098791	0.43024	-1.2168
474.9362	10.07	domics Pos	0.51234	0.99987	1.0538	0.075657	0.69308	0.99994	0.96649	-0.04917	0.394	0.99996	0.95061	-0.07308	0.21721	0.66737	0.8837	-0.17836	0.031235	0.073104	0.793	-0.33461	0.5299	0.73648	0.93708	-0.09376	0.37775	0.76056	0.90063	-0.15099	0.94124	0.98646	1.0149	0.021288	0.076526	0.17807	1.1897	0.25064
675.4707	2.27	domics Pos	0.51245	0.99987	1.1509	0.20274	0.063873	0.99994	0.7713	-0.37464	0.25462	0.99996	0.83411	-0.26169	0.01305	0.22826	0.54258	-0.88209	0.000281	0.002099	0.20975	-2.2533	0.002987	0.063179	0.38444	-1.3792	0.001623	0.23318	0.54425	-0.87765	0.067913	0.76386	0.61476	-0.70191	0.12956	0.23467	0.76625	-0.38412
650.3978	3.11	olomics Po	0.51407	0.99951	1.1776	0.23581	0.035243	0.99998	0.75771	-0.40029	0.44701	0.99941	0.91505	-0.12808	0.011609	0.19869	0.58894	-0.7638	0.002415	0.017484	0.53264	-0.90878	0.004776	0.059942	0.56821	-0.81549	0.33708	0.94973	0.83923	-0.25286	0.4096	0.99956	0.86278	-0.21293	0.10513	0.35957	1.706	0.77058
825.6564	5.97	domics Pos	0.51413	0.99987	1.0783	0.10882	0.59888	0.99994	0.95981	-0.05918	0.49223	0.99996	0.95619	-0.06463	0.11697	0.53727	0.88292	-0.17964	0.071123	0.13602	0.79042	-0.33931	0.025376	0.14021	0.83718	-0.25639	0.60648	0.86762	0.96924	-0.04508	0.21966	0.94047	0.88808	-0.17124	0.1859	0.29339	1.0932	0.12856
846.818	0.5	domics Pos	0.51415	0.99987	1.1237	0.16828	0.82766	0.99994	0.98405	-0.0232	0.48004	0.99996	0.9388	-0.09111	0.36995	0.82347	0.89591	-0.15858	0.016949	0.045801	0.79775	-0.326	0.41039	0.65377	0.91816	-0.12319	0.39277	0.76945	1.0765	0.10634	0.84067	0.97988	0.98438	-0.02272	0.032439	0.12301	1.2117	0.27708
370.976	12	olomics Ne	0.51449	0.99918	0.94705	-0.0785	0.01882	0.39934	0.79661	-0.32805	0.22997	0.99995	0.92172	-0.11759	0.025668	0.99985	0.87833	-0.18716	0.84422	0.99927	1.0367	0.052032	0.95805	0.98716	1.0096	0.013804	0.011275	0.2716	1.0943	0.12998	0.21963	0.99512	0.93027	-0.10428	0.76917	0.99941	0.98218	-0.02595
364.2198	3.19	olomics Po	0.51521	0.99951	1.1972	0.25968	0.36799	0.99998	1.233	0.30215	0.41348	0.99941	1.2362	0.30588	0.56304	0.99905	0.82487	-0.27777	0.43532	0.84074	0.78486	-0.3495	0.054441	0.19735	0.54168	-0.88448	0.011037	0.25477	0.3844	-1.3793	0.67391	0.99956	1.0583	0.081811	0.78458	0.90661	0.88877	-0.17011
969.6936	6.95	domics Pos	0.51522	0.99987	1.3043	0.38323	0.75981	0.99994	1.1469	0.19769	0.6665	0.99996	1.2226	0.29	0.90446	0.9874	1.0461	0.065076	0.48303	0.5605	0.72278	-0.46837	0.24823	0.53505	0.57958	-0.78692	0.14151	0.6049	0.49321	-1.0197	0.045647	0.65733	0.31007	-1.6894	0.009221	0.092545	0.23303	-2.1014
762.9827	6.5	olomics Po	0.5153	0.99951	1.309	0.3885	0.31273	0.99998	0.84602	-0.24124	0.36427	0.99941	1.1445	0.19476	0.048024	0.28924	0.72531	-0.46334	0.37658	0.77319	0.86183	-0.21452	0.33414	0.64542	0.86304	-0.21249	0.60097	0.99391	1.1841	0.24385	0.53587	0.99956	1.2891	0.36635	0.34578	0.62166	1.327	0.40813
908.8216	0.5	domics Pos	0.51591	0.99987	1.054	0.075837	0.48997	0.99994	0.92039	-0.11968	0.81677	0.99996	0.96775	-0.04729	0.68288	0.95954	0.95168	-0.07145	0.037701	0.084672	0.77611	-0.36566	0.35141	0.61843	0.88094	-0.18288	0.55152	0.85184	0.92751	-0.10856	0.81836	0.97988	1.013	0.018704	0.14798	0.25308	1.2287	0.29716
551.3124	5.1	olomics Po	0.5165	0.99951	0.83339	-0.26294	0.73858	0.99998	0.89891	-0.15376	0.98418	0.99941	0.9601	-0.05874	0.65052	0.99905	1.1544	0.20714	0.48033	0.88102	1.1807	0.23965	0.037057	0.15199	1.7173	0.78017	0.026768	0.3363	1.7161	0.77916	0.53487	0.99956	1.0756	0.10514	0.4306	0.69935	1.1222	0.16627
131.0532	0.6	domics Pos	0.51668	0.99987	1.0895	0.12367	0.20975	0.99994	0.8468	-0.2399	0.50704	0.99996	1.1214	0.16533	0.20666	0.65238	1.2173	0.28369	0.67544	0.72446	0.94339	-0.08407	0.60504	0.78562	0.93239	-0.101	0.035835	0.36525	1.4027	0.48818	0.76306	0.97988	1.0575	0.080663	0.082178	0.18348	1.3295	0.41086
336.9279	10.03	domics Pos	0.51669	0.99987	1.0259	0.036901	0.20041	0.99994	0.89155	-0.16561	0.19687	0.99996	0.9335	-0.09929	0.66721	0.95717	0.97098	-0.04249	0.001584	0.007631	0.8053	-0.3124	0.25741	0.54377	0.89248	-0.16411	0.23074	0.67417	0.93149	-0.10239	0.13337	0.88792	0.90052	-0.15117	0.064851	0.16322	1.1871	0.24743
225.1786	6.48	olomics Po	0.51719	0.99951	0.90031	-0.15151	0.61378	0.99998	0.94746	-0.07787	0.09674	0.99941	0.70432	-0.5057	0.048377	0.29042	0.55375	-0.85268	0.00465	0.028167	0.48396	-1.047	0.008686	0.075866	0.43158	-1.2123	0.013501	0.27797	0.4137	-1.2733	0.003411	0.47452	0.39757	-1.3307	0.015265	0.2688	0.39042	-1.3569
232.9022	10.12	domics Pos	0.5173	0.99987	0.9558	-0.06521	0.89755	0.99994	0.99303	-0.0101	0.28713	0.99996	0.92947	-0.10553	0.42673	0.86135	0.94629	-0.07964	0.021663	0.055475	0.81877	-0.28846	0.99659	0.99764	1.0016	0.002246	0.21591	0.65914	1.0653	0.091212	0.27169	0.94365	1.0849	0.11761	0.009803	0.094875	1.2448	0.31587
970.7859	10.03	domics Pos	0.51734	0.99987	1.0679	0.094788	0.35074	0.99994	0.92848	-0.10706	0.26624	0.99996	0.91135	-0.13392	0.82259	0.98421	1.0168	0.024097	0.015012	0.041846	0.78518	-0.34891	0.77954	0.88576	0.9749	-0.03668	0.62133	0.87421	1.0492	0.069313	0.20173	0.92582	0.88786	-0.1716	0.067387	0.16764	1.2358	0.30548
564.5902	0.57	lomics Neg	0.5175	0.99977	1.0388	0.054899	0.6079	0.99904	1.1326	0.17963	0.9371	0.99994	1.0122	0.017441	0.033748	0.58182	1.2647	0.33877	0.25538	0.99989	1.0917	0.12655	0.98313	0.99653	0.99795	-0.00296	0.12243	0.45153	0.83957	-0.25229	0.035849	0.46971	0.81642	-0.29261	0.005796	0.38536	0.72713	-0.45972
737.5522	4.5	domics Pos	0.51796	0.99987	1.1417	0.19124	0.51136	0.99994	1.0926	0.12774	0.051303	0.99996	1.1879	0.24847	0.17759	0.61058	1.1319	0.17874	0.024233	0.060235	0.81167	-0.30103	0.70048	0.84682	1.0445	0.062768	0.1224	0.58026	1.1215	0.1654	0.79438	0.97988	1.063	0.088087	0.013218	0.098791	1.2699	0.34476
467.1625	0.65	domics Pos	0.51822	0.99987	1.0965	0.13293	0.052585	0.99994	0.84517	-0.24268	0.031033	0.99996	0.85545	-0.22524	0.91126	0.9874	1.0144	0.020594	0.007665	0.025065	0.80974	-0.30446	0.05938	0.24149	0.76019	-0.39556	0.016428	0.33809	0.76401	-0.38834	0.022743	0.54215	0.73556	-0.44309	0.21971	0.32775	1.1196	0.16294
520.5091	8.34	olomics Po	0.51903	0.99951	0.94424	-0.08277	0.40609	0.99998	0.93758	-0.09298	0.13918	0.99941	0.82611	-0.27559	0.009945	0.19702	0.72028	-0.47337	0.59255	0.96412	0.93635	-0.09489	0.23457	0.5233	0.83521	-0.2598	0.19214	0.77069	0.81041	-0.30328	0.009224	0.47452	0.71156	-0.49094	0.11523	0.36506	0.73994	-0.43452
861.8082	10.09	domics Pos	0.51986	0.99987	0.90829	-0.13878	0.27209	0.99994	0.84046	-0.25075	0.241	0.99996	0.81917	-0.28777	0.077082	0.46503	0.75639	-0.4028	0.019077	0.050339	0.65066	-0.62003	0.07772	0.28838	0.74615	-0.42247	0.15651	0.62886	0.80858	-0.30654	0.30376	0.9524	0.82629	-0.27529	0.84953	0.88941	0.96192	-0.05601
238.9254	0.3	olomics Ne	0.51987	0.99918	0.96562	-0.05047	0.00968	0.34605	0.85737	-0.22201	0.25889	0.99995	0.9434	-0.08407	0.4133	0.99985	1.0418	0.059018	0.80323	0.99927	1.023	0.032763	0.79586	0.95294	1.014	0.019989	0.94413	0.98493	0.99766	-0.00338	0.87652	0.99512	0.99204	-0.01153	0.44361	0.99941	1.0308	0.043729
390.8035	9.92	domics Pos	0.52026	0.99987	0.93463	-0.09754	0.37976	0.99994	0.91145	-0.13376	0.15263	0.99996	0.87909	-0.18591	0.049495	0.37885	0.82052	-0.28539	0.040445	0.088782	0.78919	-0.34155	0.013627	0.098595	0.77592	-0.36602	0.86304	0.97404	1.0206	0.029456	0.69787	0.97988	1.0239	0.034053	0.97491	0.9816	1.0084	0.012025
1174.851	9.3	olomics Po	0.52068	0.99951	1.1854	0.24538	0.7826	0.99998	0.95879	-0.06071	0.6356	0.99941	1.1041	0.14293	0.39459	0.88909	0.86312	-0.21236	0.036099	0.13238	0.67031	-0.5771	0.40182	0.70949	0.87461	-0.19328	0.97576	0.9974	1.0304	0.043174	0.97795	0.99956	1.0148	0.021188	0.11214	0.36506	1.6807	0.74904
237.2205	2.09	domics Pos	0.5207	0.99987	1.1964	0.25874	0.96177	0.99994	1.0485	0.068346	0.12952	0.99996	0.68234	-0.55144	0.014145	0.22898	0.43491	-1.2012	0.017813	0.04763	0.34797	-1.523	0.005807	0.076369	0.27308	-1.8726	0.015796	0.33809	0.38935	-1.3608	0.031791	0.62711	0.42435	-1.2367	0.13474	0.23974	0.61084	-0.71112

688.9721	12.01	olomics Ne	0.521	0.99918	0.95848	-0.06118	0.012414	0.37315	0.83218	-0.26504	0.4292	0.99995	0.95057	-0.07314	0.77568	0.99985	0.98368	-0.02374	0.62768	0.99927	1.0726	0.10114	0.48158	0.8481	1.1202	0.16378	0.41383	0.7592	1.053	0.074537	0.05861	0.99512	0.88533	-0.17571	0.27036	0.99941	1.0787	0.10926
886.7716	0.54	lomics Pos	0.52157	0.99987	1.0577	0.080889	0.38669	0.99994	0.91703	-0.12496	0.8805	0.99996	0.9889	-0.01611	0.35321	0.80737	0.91781	-0.12373	0.018342	0.048805	0.79844	-0.32474	0.49186	0.70986	0.92275	-0.11599	0.62029	0.87361	0.956	-0.06493	0.64891	0.97988	0.96233	-0.05539	0.022801	0.10945	1.2682	0.3428
141.9844	10.18	lomics Pos	0.52165	0.99987	1.032	0.045445	0.18796	0.99994	0.89534	-0.1595	0.67647	0.99996	0.97311	-0.03933	0.16128	0.59245	0.91746	-0.12429	0.003149	0.012735	0.76159	-0.39292	0.27555	0.56257	0.90132	-0.14989	0.88769	0.97532	1.0153	0.02196	0.43225	0.96776	0.9548	-0.06673	0.030483	0.11983	1.1749	0.23252
133.1422	9.96	lomics Pos	0.52231	0.99987	1.1688	0.22502	0.006667	0.99994	0.82876	-0.27097	0.40994	0.99996	0.92634	-0.11038	0.14847	0.57592	0.88254	-0.18027	0.005068	0.018421	0.71825	-0.47743	0.19183	0.47839	0.86936	-0.20198	0.99281	0.99769	1.0069	0.009925	0.13669	0.89649	0.90246	-0.14807	0.27697	0.38543	1.0942	0.1299
255.2315	2.72	lomics Neg	0.52235	0.99977	0.89626	-0.15801	0.54536	0.99904	0.90172	-0.14925	0.07444	0.99994	0.61786	-0.69464	0.01622	0.58182	0.42903	-1.2209	0.003493	0.10339	0.36571	-1.4512	0.03201	0.63166	0.29075	-1.7822	0.005335	0.11279	0.24971	-2.0017	0.01372	0.46971	0.21606	-2.2105	0.016925	0.38536	0.34252	-1.5457
752.799	0.54	lomics Pos	0.5225	0.99987	1.0271	0.038613	0.13097	0.99994	0.89956	-0.15271	0.50088	0.99996	0.94321	-0.08435	0.000387	0.164	0.80893	-0.30592	0.003925	0.015071	0.76209	-0.39196	0.041016	0.18865	0.7955	-0.33006	0.22243	0.66519	0.89185	-0.16512	0.12586	0.88537	0.87097	-0.1993	0.055216	0.15227	1.2183	0.28487
1071.711	9.05	olomics Po	0.52302	0.99951	1.2071	0.27154	0.31984	0.99998	0.84036	-0.25092	0.90974	0.99941	0.98541	-0.02121	0.035963	0.25923	0.68627	-0.54315	0.038825	0.13882	0.62172	-0.68567	0.06069	0.21244	0.75374	-0.40786	0.79659	0.99391	1.109	0.14923	0.68579	0.99956	1.1298	0.17608	0.11962	0.36992	1.4226	0.50848
957.7843	10.05	lomics Pos	0.52319	0.99987	1.0498	0.070047	0.46451	0.99994	0.93089	-0.10332	7.05E-05	0.26869	0.794	-0.33279	0.28033	0.74609	0.92563	-0.11149	1.65E-05	0.00033	0.79326	-0.33413	0.12504	0.3845	0.89285	-0.16351	0.50357	0.82964	0.9771	-0.03342	0.90155	0.98023	0.99706	-0.00425	0.000264	0.07528	1.2659	0.34021
363.9306	10.02	lomics Pos	0.52417	0.99987	1.0379	0.053733	0.31831	0.99994	0.91797	-0.12349	0.54849	0.99996	0.95457	-0.06707	0.63377	0.94754	0.96145	-0.05671	0.006797	0.022935	0.76229	-0.39159	0.41818	0.65619	0.91618	-0.1263	0.53491	0.84281	1.0535	0.075199	0.59222	0.97073	1.0362	0.051329	0.016604	0.099614	1.2578	0.33088
983.791	0.51	lomics Pos	0.52438	0.99987	1.0741	0.10316	0.65425	0.99994	0.93803	-0.09229	0.75609	0.99996	0.95637	-0.06435	0.79669	0.9771	1.0258	0.036732	0.044866	0.096002	0.76638	-0.38387	0.98322	0.98918	1.0189	0.026973	0.85745	0.97181	0.96966	-0.04445	0.4816	0.96776	0.90759	-0.1399	0.17778	0.2853	1.2155	0.2815
704.5779	4.66	lomics Pos	0.52469	0.99987	1.0452	0.0638	0.24332	0.99994	0.89348	-0.1625	0.73227	0.99996	0.97008	-0.04383	0.49446	0.88894	0.93934	-0.09029	0.02148	0.055265	0.78595	-0.3475	0.29128	0.57461	0.87737	-0.18874	0.96002	0.99032	0.99816	-0.00266	0.15364	0.91321	0.86631	-0.20705	0.92126	0.94125	1.0019	0.002808
847.8037	0.53	lomics Pos	0.52477	0.99987	0.92629	-0.11047	0.22698	0.99994	0.91877	-0.12222	0.13022	0.99996	0.87948	-0.18527	0.079143	0.47523	0.88699	-0.173	0.001102	0.005916	0.72469	-0.46457	0.050877	0.2212	0.79406	-0.33268	0.67867	0.90323	1.033	0.046892	0.06666	0.76335	0.87515	-0.1924	0.066811	0.16672	1.1654	0.22086
571.3556	1.49	lomics Pos	0.52591	0.99987	1.3488	0.43164	0.53185	0.99994	1.2799	0.35598	0.44963	0.99996	1.2318	0.30076	0.41175	0.85418	1.4181	0.50396	0.80063	0.82949	0.9076	-0.13987	0.13453	0.40105	1.4932	0.57845	0.023959	0.33809	2.131	1.0915	0.32101	0.95254	1.3898	0.4749	0.024651	0.1117	2.2596	1.176
361.2013	3.17	olomics Po	0.52657	0.99951	1.1631	0.21793	0.4515	0.99998	1.1779	0.2362	0.36179	0.99941	1.1929	0.25443	0.76464	0.99905	1.0905	0.12492	0.8806	0.99688	1.0367	0.052009	0.012455	0.085318	0.57882	-0.78882	0.000537	0.16989	0.5205	-0.94204	0.46653	0.99956	0.92542	-0.11181	0.2711	0.54284	0.88564	-0.17521
656.899	11.96	olomics Ne	0.52661	0.99918	1.0158	0.02263	0.14204	0.56769	0.92402	-0.11401	0.91441	0.99995	0.99791	-0.00302	0.44672	0.99985	0.96919	-0.04515	0.32095	0.99927	1.045	0.063551	0.035857	0.48305	1.0996	0.13701	0.48528	0.80618	1.0399	0.056485	0.77827	0.99512	0.98801	-0.0174	0.82775	0.99941	1.0129	0.018457
223.202	6.97	olomics Po	0.52705	0.99951	1.0778	0.10808	0.78198	0.99998	0.97545	-0.03586	0.069737	0.99941	0.79609	-0.329	0.011233	0.19869	0.55892	-0.83928	0.000576	0.006226	0.41562	-1.2667	0.000427	0.039572	0.42354	-1.2394	0.006292	0.21836	0.47629	-1.0701	0.00905	0.47452	0.5953	-0.7483	0.10942	0.36397	0.78049	-0.35755
285.9443	0.31	olomics Po	0.52773	0.99951	1.118	0.16095	0.81905	0.99998	1.0679	0.094808	0.32012	0.99941	1.0712	0.099251	0.00695	0.1948	1.2656	0.33982	0.75013	0.99688	0.97726	-0.03319	0.67849	0.9088	0.96735	-0.04789	0.001867	0.16989	1.3738	0.45813	0.48893	0.99956	0.9332	-0.09975	0.55706	0.788	1.1117	0.15273
633.8078	2.37	olomics Po	0.52786	0.99951	0.86152	-0.21505	0.54045	0.99998	0.86685	-0.20614	0.58514	0.99941	0.88472	-0.1767	0.14951	0.54537	0.71137	-0.49132	0.019573	0.082316	0.359	-1.4779	0.15031	0.39492	0.62865	-0.66966	0.50574	0.99391	0.86499	-0.20924	0.52196	0.99956	0.8646	-0.20989	0.38534	0.65754	0.80727	-0.30887
1121.75	10.05	lomics Pos	0.52793	0.99987	1.059	0.08269	0.28114	0.99994	0.92119	-0.11843	0.011132	0.99996	0.81502	-0.29509	0.11079	0.53238	0.83635	-0.25783	0.029763	0.0704	0.77475	-0.36819	0.17369	0.45968	0.87789	-0.18789	0.73767	0.93051	0.97797	-0.03214	0.26378	0.94365	0.90349	-0.14642	0.018348	0.10363	1.1761	0.23406
540.8383	0.53	lomics Pos	0.5282	0.99987	0.96487	-0.0516	0.13352	0.99994	0.96984	-0.04419	0.001804	0.99996	0.88924	-0.16935	0.017192	0.24478	0.87601	-0.19099	7.46E-05	0.000851	0.71451	-0.48497	0.011595	0.091956	0.79362	-0.33348	0.075882	0.48465	0.88898	-0.16977	0.27752	0.94365	0.91539	-0.12754	0.020903	0.10765	1.1881	0.24869
400.8286	0.56	lomics Pos	0.52835	0.99987	1.0615	0.086165	0.2274	0.99994	1.1185	0.16153	0.94712	0.99996	0.99663	-0.00487	0.021828	0.26563	0.8409	-0.25	0.049394	0.10348	0.79527	-0.33048	0.01077	0.089666	0.81891	-0.28823	0.19024	0.64891	0.87284	-0.19621	0.9626	0.9885	0.99751	-0.0036	0.091994	0.19236	1.1455	0.19593
1131.766	0.52	lomics Pos	0.52897	0.99987	0.92093	-0.11884	0.16917	0.99994	0.84769	-0.23839	0.51761	0.99996	0.9272	-0.10904	0.60429	0.93327	0.93772	-0.09277	0.012453	0.036218	0.71598	-0.48201	0.63749	0.81018	0.93931	-0.09033	0.32037	0.73067	0.89098	-0.16653	0.76945	0.97988	0.96237	-0.05534	0.71507	0.7878	1.0666	0.093048
192.0582	0.58	lomics Pos	0.52914	0.99987	1.0547	0.076797	0.77808	0.99994	0.98189	-0.02637	0.30503	0.99996	1.086	0.11905	0.023889	0.27465	1.2794	0.35551	0.91739	0.9286	0.99399	-0.00869	0.036397	0.17369	1.13	0.17631	0.000325	0.15473	1.4667	0.55252	0.32549	0.95542	1.0831	0.11511	0.044273	0.13998	1.2291	0.29763
602.8477	10.14	lomics Pos	0.52925	0.99987	0.95979	-0.0592	0.10922	0.99994	0.87724	-0.18896	0.69117	0.99996	0.97333	-0.03899	0.10963	0.53208	0.91139	-0.13386	0.020721	0.053822	0.77636	-0.36519	0.4155	0.65515	0.90929	-0.13719	0.73575	0.92926	1.03	0.042624	0.41571	0.96776	0.93983	-0.08953	0.61941	0.71139	1.066	0.092176
833.8241	10.02	lomics Pos	0.52931	0.99987	1.0476	0.067103	0.23588	0.99994	0.90516	-0.14376	0.66835	0.99996	0.96257	-0.05504	0.24856	0.70866	0.90592	-0.14254	0.002951	0.012127	0.68317	-0.54969	0.13403	0.40042	0.85129	-0.23228	0.89351	0.97532	1.0172	0.024664	0.86169	0.98023	0.98163	-0.02675	0.062542	0.16026	1.2074	0.27188
296.2564	5.02	olomics Po	0.52947	0.99951	1.0742	0.10327	0.13016	0.99998	0.79375	-0.33325	0.42599	0.99941	0.88071	-0.18326	0.051217	0.29547	0.75287	-0.40952	0.000133	0.002004	0.36336	-1.4605	0.017337	0.097219	0.59265	-0.75474	0.16877	0.74367	0.78193	-0.35489	0.20301	0.99956	0.82829	-0.2718	0.6623	0.84773	0.93762	-0.09293
543.4729	2.27	lomics Pos	0.52973	0.99987	0.88882	-0.17004	0.47203	0.99994	0.86192	-0.21437	0.024659	0.99996	0.46122	-1.1165	0.008997	0.19971	0.34501	-1.5353	0.000143	0.00133	0.25046	-1.9974	0.002698	0.061655	0.22366	-2.1606	0.008673	0.32129	0.24921	-2.0046	0.002414	0.48691	0.22035	-2.1821	0.009543	0.094007	0.33427	-1.5809
906.8256	0.5	lomics Pos	0.53013	0.99987	1.0225	0.032133	0.36622	0.99994	0.95432	-0.06746	0.11882	0.99996	0.93283	-0.10031	0.023914	0.27465	0.87552	-0.19179	0.000143	0.001326	0.73392	-0.4463	0.21049	0.50277	0.87313	-0.19573	0.37882	0.76056	0.94026	-0.08886	0.17336	0.91806	0.93745	-0.09319	0.055007	0.15218	1.1762	0.2341
901.7239	7.45	lomics Pos	0.53069	0.99987	0.83908	-0.25313	0.57283	0.99994	0.85455	-0.22676	0.49974	0.99996	0.83058	-0.26781	0.43008	0.86175	0.79596	-0.32922	0.42726	0.51356	0.81845	-0.28904	0.65454	0.81917	0.90912	-0.13746	0.59727	0.86481	0.87987	-0.18464	0.049646	0.6775	0.56755	-0.81719	0.42207	0.5294	0.7927	-0.33514
276.9499	10.16	lomics Pos	0.53148	0.99987	1.0747	0.10388	0.24878	0.99994	0.89155	-0.16561	0.32846	0.99996	0.89776	-0.15561	0.84509	0.98707	0.98077	-0.02801	0.022788	0.057658	0.79415	-0.33251	0.42439	0.66021	0.90026	-0.15159	0.72244	0.9233	0.96387	-0.0531	0.28022	0.94365	1.1034	0.14192	0.046071	0.14289	1.3044	0.38339
1135.271	9.01	olomics Po	0.5318	0.99951	1.2327	0.30178	0.26024	0.99998	0.82268	-0.2816	0.98964	0.99941	1.0317	0.045049	0.003955	0.17363	0.62528	-0.67743	0.011953	0.055782	0.60888	-0.71576	0.1337	0.36621	0.77908	-0.36015	0.63351	0.99391	1.1567	0.20997	0.22664	0.99956	1.2858	0.36263	0.060658	0.30404	1.6437	0.71699
152.0325	0.6	lomics Pos	0.533	0.99987	0.98395	-0.02334	0.028645	0.99994	0.88105	-0.18271	0.97272	0.99996	1.0138	0.019736	0.74514	0.97302	1.0523	0.073577	0.17911	0.27091	0.89164	-0.16546	0.57523	0.76798	0.96071	-0.05783	0.031876	0.36126	1.2391	0.30932	0.84276	0.97988	0.99267	-0.01061	0.085219	0.18621	1.2218	0.28897
918.5999	4.42	lomics Pos	0.53367	0.99987	1.1622	0.2169	0.47096	0.99994	0.81697	-0.29164	0.8941	0.99996	0.98315	-0.02452	0.1137	0.53238	0.62493	-0.67823	0.005873	0.020635	0.42593	-1.2313	0.054846	0.23111	0.57174	-0.80656	0.4853	0.81703	0.81682	-0.29191	0.61639	0.97665	1.1818	0.24104	0.30264	0.41228	1.5147	0.59901
897.5528	1.4	lomics Pos	0.53391	0.99987	1.2126	0.27806	0.025795	0.99994	0.74255	-0.42944	0.48143	0.99996	1.0865	0.11966	0.016576	0.24309	0.64569	-0.63109	0.001617	0.007748	0.53684	-0.89745	0.010613	0.089124	0.70558	-0.50312	0.69115	0.90976	1.1522	0.2044	0.76069	0.97988	1.1349	0.18256	0.027355	0.11602	1.9695	0.97785
137.0472	0.61	lomics Pos	0.5343	0.99987	1.1846	0.24443	0.66641	0.99994	0.90913	-0.13743	0.70202	0.99996	0.91765	-0.12398	0.31119	0.77301	0.79413	-0.33256	0.003019	0.012283	0.23277	-2.103	0.12655	0.38727	0.66884	-0.58026	0.58476	0.86163	0.8867	-0.17349	0.29689	0.9483	0.76553	-0.38548	0.11382	0.21831	1.2779	0.35383
346.2593	2.84	lomics Pos	0.53473	0.99987	1.1679	0.22386	0.53242	0.99994	0.90736	-0.14026	0.066095	0.99996	0.73076	-0.45253	0.023913	0.27465	0.57302	-0.80333	0.000492	0.003246	0.44581	-1.1655	0.01603	0.1078	0.3986	-1.327	0.022818	0.33809	0.48952	-1.0305	0.038693	0.64308	0.53691	-0.89725	0.22548	0.33285	0.77505	-0.36763
533.8056	3.12	olomics Po	0.53517	0.99951	1.214	0.27976	0.009266	0.99998	0.68898	-0.53747	0.82894	0.99941	0.97947	-0.02993	0.023262	0.22477	0.58296	-0.77854	0.001453	0.01237	0.62408	-0.6802	0.034466	0.14437	0.63793	-0.64853	0.67886	0.99391	0.96224	-0.05553	0.67816	0.99956	0.9697	-0.04439	0.076609	0.32186	1.9145	0.93699
446.689	0.57	lomics Neg	0.53518	0.99977	1.063	0.088088	0.83712	0.99904	1.0378	0.053507	0.84997	0.99994	0.98101	-0.02766	0.15061	0.58182	1.1721	0.22911	0.30886	0.99989	1.1305	0.17699	0.70598	0.99653	1.0495	0.069701	0.049598	0.38213	0.78558	-0.34817	0.43421	0.70353	0.90031	-0.1515	0.04098	0.405	0.74735	-0.42014
931.5275	5.92	lomics Pos	0.53518	0.99987	1.0704	0.098199	0.94165	0.99994	0.99613	-0.00559	0.86968	0.99996	1.017	0.024262	0.75994	0.97526	0.96776	-0.04728	0.018159	0.048385	0.76722	-0.38228	0.12988	0.39426	0.81187	-0.30068	0.14043	0.60462	0.85011	-0.23429	0.46232	0.96776	0.91544	-0.12747	0.089011	0.18993	1.1863	0.24649
841.7072	10.37	olomics Po	0.53533	0.99951	0.93194	-0.10169	0.058867	0.99998	0.83356	-0.26265	0.033986	0.99941	0.85022	-0.23409	0.20386	0.64188	0.85134	-0.23219	0.72109	0.99688	0.97211	-0.04081	0.56292	0.84438	0.94039	-0.08868	0.20509	0.7959	0.83424	-0.26146	0.093325	0.80762	0.78163	-0.35544	0.003265	0.2688	0.76169	-0.39272
1001.53	6.11	lomics Pos	0.53542	0.99987	1.0911	0.12581	0.61155	0.99994	1.0669	0.093439	0.43838	0.99996	1.1062	0.14561	0.93501	0.98937	0.98636	-0.01982	0.05185	0.10716	0.73655	-0.44115	0.032354	0.16174	0.74844	-0.41804	0.041948	0.38314	0.74585	-0.42305	0.2823	0.94365	0.84724	-0.23916	0.78349	0.83916	1.0577	0.080977
821.588	5	lomics Pos	0.53616	0.99987	1.0841	0.11646	0.9605	0.99994	1.0077	0.011027	0.93468	0.99996	0.9932	-0.00984	0.56087	0.91079	0.93787	-0.09253	0.049632	0.10378	0.74589	-0.42297	0.011277	0.091488	0.72517	-0.4636	0.02587	0.34128	0.78482	-0.34956	0.060118	0.72218	0.77435	-0.36895	0.50019	0.60202	0.92015	-0.12006
151.0359	10.17	lomics Pos	0.53639	0.99987	0.94997	-0.07405	0.29385	0.99994	0.89314	-0.16304	0.33194	0.99996	0.92014	-0.12007	0.071173	0.45473	0.85025	-0.23405	0.012691	0.036743	0.76374	-0.38885	0.18991	0.47645	0.85893	-0.21938	0.47081	0.80647	0.93655	-0.09457	0.060033	0.72218	0.85305	-0.22929	0.30077	0.41046	1.0822	0.11393
1049.764	10.07	lomics Pos	0.53705	0.99987	1.0379	0.053666	0.19146	0.99994	0.8978	-0.15554	0.57005	0.99996	0.96715	-0.04819	0.31516	0.77709	0.92368	-0.11453	0.014814	0.041512	0.76437	-0.38765	0.085059	0.30425	0.84343	-0.24566	0.68492	0.90555	1.0366	0.051811	0.91287	0.98248	1.018	0.025759	0.13059	0.23577	1.1377	0.18614
924.569	5.22	lomics Pos	0.5385	0.99987	1.0412	0.058253	0.44053	0.99994	0.91658	-0.12567	0.6648	0.99996	0.96283	-0.05465	0.71832	0.96544	1.0304	0.043247	0.004727	0.017516	0.76196	-0.39221	0.66888	0.82743	0.96006	-0.0588	0.95659	0.98985	0.99864	-0.00196	0.62667	0.97813	0.94991	-0.07413	0.72848	0.79815	1.0541	0.075993
1126.469	5.91	lomics Pos	0.53856	0.99987	0.92936	-0.10569	0.87959	0.99994	0.99227	-0.0112	0.96013	0.99996	1.012	0.01719	0.91381	0.9874	1.0049	0.006992	0.023389	0.058633	0.7891	-0.34172	0.20522	0.49558	0.79909	-0.32357	0.70279	0.91466	0.96158	-0.05652	0.35157	0.96776	0.91036	-0.1355	0.10697	0.20971	1.2145	0.28035
336.2023	2.72	lomics Pos	0.53864	0.99987	0.91075	-0.13488	0.14793	0.99994	0.77612	-0.36565	0.009442	0.99996	0.64669	-0.62884	0.005731	0.18075	0.45474	-1.1369	0.000326	0.002357	0.4092	-1.2891	0.012174	0.093	0.34268	-1.545	0.021369	0.33809	0.45074	-1.1496	0.005704	0.52955	0.41403	-1.2722	0.020863	0.10765	0.53784	-0.89475
921.694	7.1	lomics Pos	0.53922	0.99987	1.0944	0.13008	0.51016	0.99994	1.1345	0.1821	0.43881	0.99996	1.1352	0.18295	0.40553	0.84674	0.87176	-0.198	0.010905	0.032817	0.60415	-0.72703	0.027839	0.14743	0.54645	-0.87185	0.04458	0.38909	0.51847	-0.94766	0.005058	0.52955	0.28724	-1.7997	0.010374	0.094875	0.32039	-1.6421
72.0816	9.96	lomics Pos	0.53965	0.99987	0.85624	-0.22392	0.15412	0.99994	0.72763	-0.45872	0.29753	0.99996	0.78303	-0.35286	0.31528	0.77709	0.79231	-0.33586	0.041559	0.090654	0.59285	-0.75426	0.43976	0.67288	0.82255	-0.28183	0.25107	0.68824	0.76132	-0.39342	0.85038	0.97988	0.97686	-0.03378	0.93699	0.95353	0.94802	-0.07702
520.2799	1.92	lomics Pos	0.5397	0.99987	1.1308	0.17729	0.58274	0.99994	0.90473	-0.14444	0.7886	0.99996	0.96388	-0.05308	0.71228	0.96346	1.0684	0.095388	0.098802	0.17289	0.64877	-0.62423	0.19246	0.47869	1.3387	0.42079	0.033544	0.36346	1.7283	0.78937	0.075806	0.78333	1.3353	0.41712	0.036414	0.12924	1.6909	0.75778
1184.256	8.99	olomics Po	0.53991	0.99951	1.3023	0.38111	0.12206	0.99998	0.82491	-0.27769	0.12134	0.99941	0.84326	-0.24595	0.001283	0.13475	0.65443	-0.6117	0.002383	0.017364	0.55278	-0.85523	0.023491	0.11367	0.73413	-0.44589	0.989	0.99883	1.0864	0.11958	0.91041	0.99956	1.0071	0.010138	0.027693	0.2688	1.5671	0.64813
837.9815	12.04	olomics Ne	0.54033	0.99918	1.0406	0.057387	0.97798	0.99664	0.99876	-0.00179	0.46204	0.99995	1.1475	0.19852	0.14663	0.99985	1.1626	0.21738	0.44438	0.99927	1.1124	0.15368	0.033903	0.47167	1.5246	0.60848	0.03106	0.34429	1.2873	0.36436	0.050651	0.99512	1.3007	0.37933	0.14743	0.99941	1.207	0.27143
1040.574	5.79	lomics Pos	0.54049	0.99987	0.9121	-0.13273	0.31046	0.99994	0.8549	-0.22617	0.2952	0.99996	0.82506	-0.27742	0.49978	0.89009	0.88944	-0.16902	0.015581	0.04302	0.63166	-0.66277	0.016193	0.10794	0.62642	-0.6748	0.057661	0.42807	0.71084	-0.49241	0.040287	0.64308	0.68661	-0.54244	0.91544	0.93727	1.0001	0.00014
173.1349	6.06	olomics Po	0.54074	0.99951	0.91698	-0.12504	0.54491	0.99998	0.92493	-0.11258	0.002093	0.99941	0.64156	-0.64035	0.005405	0.1948	0.43509	-1.2006	0.00134	0.01166	0.36617	-1.4494	0.002827	0.048023	0.39807	-1.3289	0.000599	0.16989	0.35597	-1.4902	0.005774	0.47452	0.38911	-1.3617	0.023183	0.2688	0.43995	-1.1846
633.4509	9.55	olomics Po	0.54074	0.99951	1.1518	0.20393	0.21197	0.99998	0.84435	-0.24409	0.34547	0.99941	1.0816	0.11322	0.021456	0.22258	0.70039	-0.51377	0.033442	0.12516	0.69594	-0.52297	0.032089	0.1379	0.72964	-0.45475	0.36093	0.96545	1.1732	0.23042	0.80796	0.99956	0.98519	-0.02153	0.1239	0.37349	1.4228	0.50873
985.7698	10.06	lomics Pos	0.54084	0.99987	1.0573	0.080389	0.27695	0.99994	0.84491	-0.24312	0.23652	0.99996	0.84434	-0.24411	0.37224	0.82444	0.916	-0.12659	0.020716	0.053822	0.78026	-0.35797	0.3018	0.58179	0.88142	-0.18209	0.94038	0.98682	1.0238	0.033878	0.61512	0.97565	0.95842	-0.06127	0.014232	0.098791	1.3816	0.46629
1105.773	10.02	lomics Pos	0.54161	0.99987	0.96411	-0.05272	0.13872	0.99994	0.91919	-0.12156	0.1288	0.99996	0.90067	-0.15093	0.008798	0.19971	0.8234	-0.28034	0.001917	0.008788	0.7183	-0.47734	0.027176	0.14544	0.78694	-0.34567	0.40425	0.77639	0.93784	-0.09259	0.12624	0.88537	0.90479	-0.14434	0.035463	0.12781	1.1249	0.16982
1111.802	9.63	olomics Po	0.54186	0.99951	1.2403	0.3107	0.11167	0.99998	0.81062	-0.3029	0.86017	0.99941	1.0262	0.037273	0.023673	0.22481	0.64277	-0.63763	0.004872	0.028888	0.59081	-0.75923	0.017565	0.097645	0.55822	-0.84109	0.64598	0.99391	0.94031	-0.08879	0.95972	0.99956	1.0393	0.055616	0.15711	0.4144	1.4302	0.51621
775.5532	5.16	lomics Pos	0.54221	0.99987	1.0693	0.096675	0.57714	0.99994	0.94364	-0.08369	0.65508	0.99996	0.95076	-0.07285	0.25983	0.71791	0.88667	-0.17353	0.006324	0.021724	0.71659	-0.48079	0.27748	0.56398	0.89215	-0.16465	0.63853	0.88463	1.0392	0.055499	0.85232	0.97988	0.97993	-0.02925	0.5663	0.66115	1.067	0.093596
882.5196	5.89	lomics Pos	0.54223	0.99987	0.85765	-0.22155	0.56164	0.99994	0.85925	-0.21885	0.5679	0.99996	0.86832	-0.2037	0.66852	0.95717	0.90318	-0.14691	0.97541	0.97901	0.99828	-0.00248	0.044416	0.20019	1.4869	0.5723	0.036815	0.36759	1.6438	0.71701	0.067524	0.76335	1.5239	0.6078	0.018235	0.1032	2.1686	1.1168
493.9777	5.28	olomics Ne	0.54229	0.99918	0.96143	-0.05675	0.039693	0.46097	0.85118	-0.23246	0.31885	0.99995	0.9396	-0.08988	0.071005	0.99985	0.89921	-0.15328	0.98693	0.99927	1.0092	0.013228	0.71011	0.94255	0.97945	-0.02995	0.83102	0.95121	1.0124	0.017789	0.78639	0.99512	1.0181	0.025933	0.2737	0.99941	0.94001	-0.08926
852.5733	5.67	lomics Pos	0.54232	0.99987	0.9536	-0.06854	0.20365	0.99994	0.88925	-0.16934	0.14576	0.99996	0.85458	-0.22671	0.14364	0.57187	0.87693	-0.18947	0.002751	0.011464	0.67319	-0.57091	0.082942	0.29892	0.81064	-0.30286	0.38475	0.76529	0.92142	-0.11807	0.040622	0.64308	0.82427	-0.27882	0.084779	0.186	1.1964	0.2587
179.0875	0.54	lomics Pos	0.54236	0.99987	1.0736	0.10246	0.26664	0.99994	0.88777	-0.17173	0.048119	0.99996	1.1209	0.16472	0.88694	0.9874	0.99502	-0.00721	0.023278	0.058432	0.68468	-0.54649	0.8579	0.92844	0.99205	-0.01152	0.00849	0.32129	1.55	0.63225	0.9656	0.98939	1.0554	0.077835	0.000212	0.07528	1.6095	0.68663
378.2297	2.28	lomics Pos	0.54355	0.99987	1.1648	0.22013	0.48198	0.99994	0.90132	-0.14989	0.3164	0.99996	0.87883	-0.18635	0.049195	0.37885	0.69249	-0.53013	0.004181	0.015817	0.58873	-0.76432	0.066349	0.26108	0.5961	-0.74637	0.11173	0.56114	0.74363	-0.42735	0.043481	0.65325	0.60887	-0.7158	0.29632	0.40687	0.85269	-0.22991
682.8067	10.09	lomics Pos	0.54433	0.99987	1.0568	0.079638	0.16079	0.99994	0.90918	-0.13736	0.31402	0.99996	0.93523	-0.09661	0.3625	0.81788	0.93936	-0.09026	0.016354	0.044541	0.74073	-0.43297	0.56328	0.76135	0.95093	-0.0726	0.62093	0.87398	1.0431	0.06089	0.7094	0.97988	0.97061	-0.04303	0.11882	0.22318	1.1447	0.19502
616.3422	5.41	olomics Ne	0.54453	0.99918	1.0769	0.10692	0.79859	0.97675	1.0557	0.078247	0.26442	0.99995	1.2149	0.28079	0.66475	0.99985	1.0628	0.087919	0.45866	0.99927	1.1364	0.18452	0.018188	0.40708	1.6812	0.74946	0.007902	0.2539	1.7821	0.8336	0.15719	0.99512	1.2324	0.30145	0.11074	0.99941	1.265	0.33919
598.506	2.72	lomics Pos	0.54533	0.99987	0.86529	-0.20875	0.34704	0.99994	0.79162	-0.33712	0.026538	0.99996	0.57346	-0.80223	0.00159	0.17581	0.40376	-1.3084	0.001368	0.006881	0.27457	-1.8648	0.010119	0.08787	0.28645	-1.8036	0.015164	0.33809	0.35663	-1.4875	0.008479	0.52955	0.31425	-1.67	0.03677	0.13006	0.4459	-1.1652
793.5547	10.19	olomics Ne	0.54569	0.99918	1.0645	0.090243	0.32087	0.74862	0.91534	-0.12762	0.28783	0.99995	1.111	0.15181	0.67346	0.99985	1.0149	0.021319	0.93593	0.99927	0.99991	-0.00013	0.098428	0.55533	1.1147	0.15661	0.37693	0.72956	1.1632	0.21811	0.045869	0.99512	1.0542	0.076122	0.78461	0.99941	1.012	0.017144
835.6037	5.23	lomics Pos	0.54678	0.99987	1.0861	0.11918	0.92814	0.99994	0.98876	-0.01631	0.96311	0.99996	0.99167	-0.01207	0.6027	0.93327	0.93876	-0.09118	0.040585	0.088988	0.75426	-0.40687	0.30213	0.582	0.85888	-0.21947	0.58503	0.86163	0.93268	-0.10054	0.70636	0.97988	0.95507	-0.06632	0.24842	0.3569	1.1924	0.25383
205.1952	4.73	lomics Pos	0.54683	0.99987	1.0409	0.057845	0.602	0.99994	0.95286	-0.06966	0.33703	0.99996	0.90837	-0.13864	0.49663	0.89009	1.0782	0.10857	0.009473	0.029511	0.79581	-0.32951	0.71104	0.84964	0.96828	-0.0465	0.2634	0.69842	1.1427	0.19244	0.33326	0.96267	0.93813	-0.09214	0.17592	0.2836	1.2104	0.27543
838.5099	4.52	lomics Pos	0.54698	0.99987	0.94592	-0.0802	0.25263	0.99994	0.90253	-0.14796	0.2609	0.99996	0.85051	-0.23359	0.071197	0.45473	0.78054	-0.35746	0.001752	0.008195	0.63974	-0.64443	0.19045	0.47713	0.81858	-0.28881	0.27746	0.70815	0.8567	-0.22314	0.86938	0.98023	0.98522	-0.02149	0.02228	0.10856	1.4297	0.51568
947.4885	12.09	olomics Po	0.54715	0.99951	0.92623	-0.11056	0.093232	0.99998	0.7833	-0.35236	0.63306	0.99941	0.93459	-0.09759	0.71889	0.99905	1.0407	0.057594	0.16178	0.42432	0.82753	-0.27312	0.009591	0.078676	0.6845	-0.54688	0.55419	0.99391	0.93076	-0.10352	0.84274	0.99956	0.97559	-0.03565	0.54403	0.77898	0.92951	-0.10546
293.1975	0.58	lomics Pos	0.54769	0.99987	0.89359	-0.16232	0.80337	0.99994	1.0289	0.041117	0.86217	0.99996	0.95728	-0.06298	0.85687	0.9874	0.95854	-0.06109	0.99627	0.9968	0.98859	-0.01656	0.15254	0.43045	1.3241	0.40498	0.00855	0.32129	1.7369	0.79649	0.44981	0.96776	1.1707	0.22743	0.52897	0.62756	1.1171	0.15971
868.7139	0.56	lomics Pos	0.54798	0.99987	1.1033	0.14184	0.72778	0.99994	1.0357	0.0506	0.48311	0.99996	1.0523	0.073553	0.16478	0.59611	0.86184	-0.21451	0.036747	0.082762	0.78091	-0.35677	0.23416	0.52183	0.88206	-0.18106	0.33938	0.74343	0.90173	-0.14924	0.93647	0.98616	0.9881	-0.01727	0.16464	0.27211	1.1595	0.21345
342.8704	10.13	lomics Pos	0.548	0.99987	0.96106	-0.05731	0.11071	0.99994	0.82896	-0.27063	0.35951	0.99996	0.9178	-0.12376	0.69643	0.96092	0.97288	-0.03967	0.000229	0.001839	0.69557	-0.52374	0.70091	0.84682	0.97156	-0.04162	0.41763	0.78099	0.95516	-0.06619	0.29028	0.94825	0.91882	-0.12215	0.73907	0.80563	1.0494	0.069594
1137.323	4.67	lomics Pos	0.54806	0.99987	1.1068	0.14641	0.44502	0.99994	0.91702	-0.12498	0.35973	0.99996	0.87948	-0.18527	0.51698	0.89842	0.9485	-0.07629	0.001968	0.008967	0.68132	-0.5536	0.14381	0.41633	0.84229	-0.24761	0.41095	0.78028	1.0729	0.10154	0.6653	0.97988	0.9617	-0.05634	0.9783	0.98378	1.0019	0.002805
378.9815	12	olomics Ne	0.54835	0.99918	0.95567	-0.06542	0.006765	0.34605	0.81404	-0.29683	0.19061	0.99995	0.92592	-0.11104	0.17643	0.99985	0.86928	-0.20211	0.71207	0.99927	0.9808	-0.02797	0.09539	0.55533	0.88676	-0.17338	0.66316	0.87948	1.0333	0.047277	0.042247	0.99512	0.82721	-0.27368	0.2581	0.99941	0.94484	-0.08186
1115.234	9.02	olomics Po	0.54858	0.99951	1.2565	0.32938	0.31871	0.99998	0.84976	-0.23487	0.84903	0.99941	0.99874	-0.00181	0.014338	0.20085	0.69563	-0.5236	0.057211	0.18562	0.62544	-0.67706	0.09767	0.29498	0.76385	-0.38863	0.73714	0.99391	1.2205	0.28749	0.94477	0.99956	1.0523	0.073598	0.035808	0.27578	1.5695	0.65033
941.7005	7.43	lomics Pos	0.54906	0.99987	0.92498	-0.1125	0.69888	0.99994	0.96468	-0.05188	0.20299	0.99996	0.83296	-0.26368	0.046394	0.37242	0.72581	-0.46233	0.001564	0.00756	0.55952	-0.83774	0.058327	0.24091	0.51699	-0.95178	0.074473	0.48057	0.55762	-0.84265	0.021204	0.52955	0.45537	-1.1349	0.10987	0.21353	0.6217	-0.6857
370.9753	4.75	olomics Ne	0.54909	0.99918	1.0138	0.019782	0.041804	0.46524	0.89435	-0.16109	0.99458	0.99995	1.0026	0.003802	0.7994	0.99985	1.0138	0.019809	0.61537	0.99927	0.98107	-0.02757	0.39381	0.79212	1.0655	0.091542	0.15057	0.53641	1.0732	0.10186	0.92091	0.99512	1.0072	0.010414	0.95466	0.99945	1.006	0.008638
370.2157	2.72	lomics Pos	0.54929	0.99987	0.93801	-0.09233	0.059096	0.99994	0.79779	-0.32591	0.010423	0.99996	0.71895	-0.47604	0.002007	0.17581	0.56616	-0.82073	0.000115	0.001135	0.41978	-1.2523	0.009617	0.087438	0.42021	-1.2508	0.020605	0.33809	0.48265	-1.051	0.007552	0.52955	0.46113	-1.1167	0.071894	0.17372	0.61729	-0.69599
140.9963	10.18	lomics Pos	0.54943	0.99987	1.0232	0.033019	0.30319	0.99994	0.93451	-0.09772	0.27677	0.99996	0.9431	-0.08452	0.16941	0.60365	0.92217	-0.1169	0.000669	0.004067	0.76162	-0.39286	0.20916	0.50159	0.89336	-0.16269	0.73574	0.92926	0.98111	-0.02751	0.49844	0.96776	0.97104	-0.04239	0.014818	0.098791	1.171	0.22776
221.1538	1	lomics Pos	0.54952	0.99987	0.92226	-0.11675	0.23236	0.99994	0.82819	-0.27197	0.66261	0.99996	0.94098	-0.08776	0.92062	0.9874	0.97811	-0.03194	0.00433	0.016248	0.62227	-0.68438	0.064858	0.25654	0.66953	-0.57878	0.63362	0.8822	0.93032	-0.1042	0.16261	0.91806	0.84135	-0.24921	0.65495	0.74148	0.93892	-0.09092
839.6353	5.65	lomics Pos	0.55059	0.99987	1.0592	0.082951	0.84116	0.99994	1.0261	0.037235	0.96809	0.99996	1.0053	0.007659	0.62142	0.94062	0.95579	-0.06524	0.031576	0.073684	0.7845	-0.35015	0.23092	0.51854	0.875	-0.19265	0.34377	0.74613	0.89074	-0.16692	0.27423	0.94365	0.89072	-0.16696	0.042708	0.13766	1.2327	0.30178
946.6971	7.02	lomics Pos	0.5518	0.99987	1.2713	0.34635	0.55606	0.99994	1.268	0.34251	0.54661	0.99996	1.251	0.32312	0.74142	0.97237	1.0553	0.077598	0.46556	0.5457	0.71422	-0.48556	0.13538	0.40206	0.49528	-1.0137	0.070646	0.47093	0.39702	-1.3327	0.014851	0.52955	0.22515	-2.1511	0.005424	0.088007	0.16306	-2.6165
344.2303	6.48	olomics Po	0.55181	0.99951	0.87597	-0.19104	0.76935	0.99998	1.0026	0.003799	0.17761	0.99941	0.72049	-0.47295	0.10195	0.42704	0.63947	-0.64505	0.077205	0.23452	0.63459	-0.6561	0.018258	0.09985	0.47564	-1.0721	0.035267	0.37184	0.53846	-0.89309	0.060193	0.71507	0.55982	-0.83697	0.027819	0.2688	0.50289	-0.9917
909.5453	5.92	lomics Pos	0.55196	0.99987	1.0772	0.10732	0.91565	0.99994	1.0192	0.02748	0.82185	0.99996	1.0262	0.037359	0.76996	0.97526	0.96475	-0.05177	0.030696	0.072115	0.77496	-0.36781	0.093945	0.32411	0.80264	-0.31717	0.17	0.64246	0.84331	-0.24587	0.48985	0.96776	0.91249	-0.13212	0.059612	0.15719	1.2262	0.29416
790.5457	5.45	lomics Pos	0.55286	0.99987	1.0336	0.047623	0.080954	0.99994	0.87515	-0.19239	0.099781	0.99996	0.87759	-0.18838	0.02246	0.26776	0.84417	-0.24439	0.000524	0.003424	0.64718	-0.62776	0.03887	0.18097	0.74589	-0.42297	0.54386	0.84884	0.95235	-0.07044	0.91887	0.98304	1.0045	0.006446	0.92383	0.94312	1.0042	0.006108
204.1194	0.33	olomics Po	0.55293	0.99951	0.84056	-0.25058	0.5282	0.99998	0.83468	-0.2607	0.26302	0.99941	0.72384	-0.46625	0.021436	0.22258	0.48029	-1.058	0.038026	0.13689	0.44164	-1.1791	0.009976	0.079508	0.35718	-1.4853	0.010579	0.24769	0.3635	-1.46	0.094403	0.81248	0.51864	-0.94719	0.0168	0.2688	0.38766	-1.3671
789.619	5.71	lomics Pos	0.55329	0.99987	1.0575	0.080702	0.57322	0.99994	0.95295	-0.06952	0.63557	0.99996	0.96365	-0.05342	0.28824	0.75229	0.90068	-0.15092	0.025244	0.062222	0.77385	-0.36987	0.88057	0.93788	0.99151	-0.0123	0.51504	0.83572	1.1083	0.1483	0.59109	0.97073	1.0984	0.13545	0.029942	0.1191	2.04	1.0286
910.5488	5.92	lomics Pos	0.55354	0.99987	1.0801	0.11123	0.93465	0.99994	1.0144	0.020597	0.87055	0.99996	1.0168	0.024009	0.74265	0.97243	0.95875	-0.06077	0.034322	0.078601	0.76915	-0.37867	0.10224	0.34	0.80039	-0.32123	0.17994	0.64793	0.83527	-0.25968	0.45913	0.96776	0.90417	-0.14533	0.075824	0.17781	1.2188	0.28545
838.8382	0.5	lomics Pos	0.55385	0.99987	1.017	0.024359	0.3306	0.99994	0.9515	-0.07173	0.10194	0.99996	0.92752	-0.10855	0.016703	0.24402	0.8805	-0.1836	3.35E-05	0.00049	0.74007	-0.43428	0.2299	0.51779	0.87597	-0.19105	0.42268	0.78658	0.95188	-0.07115	0.17796	0.92219	0.93931	-0.09033	0.035117	0.12752	1.1849	0.2448
300.2274	1.39	lomics Pos	0.55412	0.99987	1.0642	0.089814	0.1992	0.99994	0.85955	-0.21834	0.75152	0.99996	0.97245	-0.04031	0.046968	0.3738	0.65926	-0.60109	3.13E-05	0.000482	0.21825	-2.1959	0.000799	0.040694	0.4392	-1.187	0.012409	0.3324	0.63804	-0.64829	0.10096	0.85376	0.76853	-0.37982	0.19931	0.3078	1.1973	0.25978
817.6533	6.07	lomics Pos	0.55453	0.99987	0.94961	-0.0746	0.53107	0.99994	0.94142	-0.08709	0.64466	0.99996	0.95514	-0.06622	0.60849	0.93556	0.95316	-0.06921	0.025352	0.062446	0.75801	-0.3997	0.14399	0.41633	0.88304	-0.17945	0.45314	0.80204	1.0805	0.11173	0.42244	0.96776	0.91276	-0.13169	0.20574	0.31442	1.184	0.24363
625.9772	12.02	olomics Ne	0.55479	0.99918	0.88165	-0.18172	0.49829	0.85432	0.85856	-0.22	0.35437	0.99995	0.80554	-0.31197	0.16377	0.99985	0.72234	-0.46925	0.75579	0.99927	0.94371	-0.08358	0.56259	0.87267	0.87915	-0.18582	0.94944	0.98794	0.9833	-0.0243	0.046506	0.99512	0.65893	-0.60181	0.56799	0.99941	0.88145	-0.18205
402.8263	0.56	lomics Pos	0.55485	0.99987	1.049	0.06895	0.67907	0.99994	1.0253	0.036089	0.72961	0.99996	0.97995	-0.02921	0.020627	0.26043	0.85774	-0.22139	0.00571	0.020215	0.78138	-0.35591	0.003684	0.06753	0.85763	-0.22157	0.17693	0.64616	0.90196	-0.14887	0.38181	0.96776	0.96911	-0.04527	0.22074	0.32891	1.1079	0.14777
599.3174	7.14	olomics Ne	0.55537	0.99918	0.88716	-0.17273	0.35552	0.76512	0.83374	-0.26233	0.95546	0.99995	0.96883	-0.04568	0.61088	0.99985	0.90055	-0.15113	0.037298	0.99927	0.53721	-0.89645	0.27405	0.6999	0.79989	-0.32212	0.74256	0.92376	1.0329	0.04671	0.54611	0.99512	1.0921	0.12708	0.44686	0.99941	1.1302	0.1766
442.8706	10.14	lomics Pos	0.55551	0.99987	0.96853	-0.04613	0.20746	0.99994	0.92282	-0.11587	0.10183	0.99996	0.86259	-0.21325	0.02168	0.26563	0.83654	-0.2575	0.004563	0.016992	0.79515	-0.33069	0.12278	0.38218	0.88907	-0.16963	0.74787	0.93404	1.0317	0.044979	0.27588	0.94365	0.94755	-0.07772	0.003885	0.085302	1.1832	0.24273
748.5669	8.39	olomics Ne	0.55596	0.99918	1.0308	0.043792	0.020997	0.40327	0.88651	-0.17379	0.84696	0.99995	1.0137	0.019592	0.53378	0.99985	1.0229	0.032699	0.71757	0.99927	1.0165	0.023643	0.2682	0.69506	1.0667	0.093131	0.50484	0.81449	1.0316	0.044829	0.84476	0.99512	0.99075	-0.0134	0.04542	0.99941	0.87211	-0.19741
74.0979	11.84	olomics Po	0.55616	0.99951	0.92632	-0.11042	0.096156	0.99998	0.82241	-0.28207	0.23453	0.99941	0.88083	-0.18306	0.51966	0.9867	0.92284	-0.11584	0.25813	0.60275	0.83818	-0.25466	0.93619	0.98056	1.0156	0.022315	0.026235	0.3338	0.73102	-0.45203	0.07827	0.77824	0.79124	-0.3378	0.41566	0.68743	0.90938	-0.13704
423.2523	7.49	olomics Po	0.55699	0.99951	0.93106	-0.10306	0.54631	0.99998	0.92337	-0.11502	0.041761	0.99941	0.78117	-0.35629	0.00859	0.19702	0.62028	-0.68901	0.002236	0.016767	0.62675	-0.67403	0.005245	0.062584	0.50764	-0.97813	0.006959	0.22149	0.58551	-0.77223	0.002876	0.47452	0.52168	-0.93877	0.025078	0.2688	0.58998	-0.76127
837.517	4.53	lomics Pos	0.55725	0.99987	1.0623	0.087255	0.30183	0.99994	0.92559	-0.11155	0.27009	0.99996	0.90322	-0.14686	0.030441	0.30465	0.83651	-0.25754	0.002271	0.009929	0.6918	-0.53158	0.020474	0.12316	0.78893	-0.34203	0.90744	0.97907	0.9893	-0.01552	0.88969	0.98023	0.98947	-0.01527	0.27212	0.38104	1.215	0.28094

714.4912	9.5	olomics Pc	0.55725	0.99951	1.1916	0.25289	0.22983	0.99998	0.85954	-0.21837	0.49853	0.99941	1.0737	0.10261	0.002357	0.15466	0.63011	-0.66632	0.00353	0.022927	0.58009	-0.78565	0.054443	0.19735	0.65014	-0.62117	0.81201	0.99391	0.97915	-0.0304	0.88577	0.99956	1.0376	0.053202	0.096398	0.34969	1.5835	0.66308
367.0872	0.59	lomics Neg	0.55756	0.99977	1.1065	0.14602	0.7332	0.99904	1.2541	0.3267	0.58418	0.99994	1.4147	0.50052	0.75792	0.81579	1.125	0.16987	0.79441	0.99989	1.0639	0.089399	0.66432	0.99653	1.5516	0.63379	0.022749	0.30828	2.0991	1.0698	0.24321	0.62019	1.2927	0.37036	0.16445	0.41025	1.5106	0.59516
477.2781	1.34	domics Pos	0.55796	0.99987	1.1444	0.19465	0.81884	0.99994	0.91061	-0.13509	0.84438	0.99996	1.0254	0.036169	0.73162	0.96985	1.0402	0.056903	0.94896	0.95623	0.95082	-0.07275	0.061689	0.24865	1.7462	0.80422	0.012223	0.3324	2.2958	1.199	0.146	0.91175	1.5184	0.60254	0.033078	0.12451	1.9403	0.95629
1133.744	10.07	domics Pos	0.558	0.99987	1.056	0.078631	0.26102	0.99994	0.90009	-0.15185	0.63479	0.99996	1.0537	0.075481	0.33769	0.7957	0.87995	-0.1845	0.011239	0.033632	0.77391	-0.36977	0.30491	0.58482	0.91333	-0.13079	0.31215	0.72948	1.095	0.13094	0.9093	0.98239	0.98991	-0.01463	0.23966	0.34759	1.2955	0.37353
796.5866	5.14	domics Pos	0.55808	0.99987	1.0758	0.10537	0.98739	0.99994	0.9988	-0.00174	0.95478	0.99996	0.99162	-0.01215	0.54437	0.90473	0.93211	-0.10143	0.040205	0.088434	0.7594	-0.39706	0.17084	0.45809	0.84511	-0.24279	0.33506	0.74176	0.90459	-0.14466	0.15715	0.9146	0.8472	-0.23923	0.35595	0.46545	0.88794	-0.17146
815.5426	5.35	domics Pos	0.55822	0.99987	0.8997	-0.15249	0.11884	0.99994	0.73228	-0.44953	0.37231	0.99996	0.84021	-0.25118	0.24928	0.70866	0.77041	-0.37631	0.023778	0.059413	0.57444	-0.79977	0.030295	0.15548	0.66579	-0.58687	0.77144	0.94358	1.0678	0.094643	0.11561	0.88537	1.2323	0.30136	0.18001	0.28731	1.2227	0.29011
1073.464	5.91	domics Pos	0.55857	0.99987	0.95329	-0.06902	0.34953	0.99994	0.90834	-0.1387	0.091725	0.99996	0.92698	-0.10939	0.745	0.97302	1.0538	0.075578	0.015013	0.041846	0.8336	-0.26258	0.94443	0.97039	1.0529	0.074429	0.49532	0.82378	1.0655	0.091556	0.11435	0.88445	0.87587	-0.19121	0.03508	0.12751	1.2238	0.29134
913.6322	8.93	olomics Pc	0.55889	0.99951	1.2637	0.33761	0.24609	0.99998	0.84364	-0.24529	0.6214	0.99941	1.0956	0.13167	0.014275	0.20085	0.71386	-0.48629	0.03673	0.13345	0.69373	-0.52754	0.028395	0.1276	0.68926	-0.53688	0.68705	0.99391	1.0665	0.092834	0.95468	0.99956	1.042	0.059287	0.25968	0.53116	1.4977	0.58277
314.2335	3.49	olomics Pc	0.55924	0.99951	0.89625	-0.15802	0.25009	0.99998	0.79189	-0.33662	0.77684	0.99941	0.94482	-0.08189	0.055739	0.30857	0.71894	-0.47607	0.079671	0.23993	0.50596	-0.98291	0.012488	0.085318	0.23142	-2.1114	0.044953	0.4245	0.36068	-1.4712	0.050477	0.6795	0.42805	-1.2242	0.012038	0.2688	0.2359	-2.0837
780.5513	4.95	domics Pos	0.55947	0.99987	1.0319	0.04531	0.39801	0.99994	0.94367	-0.08364	0.56551	0.99996	0.96822	-0.04659	0.48694	0.88725	0.95667	-0.0639	0.001028	0.005598	0.7699	-0.37725	0.3102	0.58859	0.90738	-0.14022	0.97164	0.99324	1.0021	0.003022	0.49559	0.96776	1.0191	0.027294	0.001008	0.07528	1.27	0.34485
185.9606	0.6	domics Pos	0.56018	0.99987	1.2912	0.36874	0.82816	0.99994	0.96564	-0.05044	0.84055	0.99996	1.0339	0.048028	0.85592	0.9874	0.97755	-0.03275	0.001497	0.00732	0.41109	-1.2825	0.41718	0.65592	0.84852	-0.23698	0.52996	0.84162	1.2311	0.29991	0.77344	0.97988	0.94941	-0.07489	0.43491	0.54087	1.2023	0.26583
1090.743	9.04	olomics Pc	0.56021	0.99951	1.1416	0.1911	0.17287	0.99998	0.83756	-0.25574	0.40464	0.99941	0.91274	-0.13172	0.002432	0.15632	0.6303	-0.6659	0.019272	0.081924	0.62721	-0.67297	0.013208	0.085844	0.73371	-0.44673	0.73721	0.99391	0.99129	-0.01262	0.52934	0.99956	1.1099	0.15043	0.086209	0.33818	1.5305	0.61396
414.8543	0.56	domics Pos	0.56026	0.99987	0.96474	-0.05179	0.060616	0.99994	0.92779	-0.10813	0.083637	0.99996	0.87184	-0.19787	0.008814	0.19971	0.8453	-0.24247	0.066386	0.12875	0.81391	-0.29706	0.002183	0.060468	0.76921	-0.37856	0.015039	0.33809	0.85101	-0.23275	0.033897	0.6327	0.90658	-0.1415	0.096453	0.19752	1.1153	0.15737
121.0853	0.6	olomics Pc	0.56035	0.99951	1.0627	0.087713	0.14549	0.99998	1.1964	0.25866	0.15267	0.99941	1.1683	0.22438	0.033291	0.25203	1.738	0.7974	0.041678	0.1462	1.5567	0.63852	0.06489	0.22174	1.3311	0.41259	0.001145	0.16989	1.8772	0.9086	0.43178	0.99956	1.1092	0.14948	0.91538	0.96475	1.0176	0.025219
587.2792	1.91	domics Pos	0.56038	0.99987	0.93159	-0.10223	0.70868	0.99994	1.0736	0.10248	0.80686	0.99996	0.98657	-0.01951	0.72539	0.96875	0.97239	-0.0404	0.027381	0.066205	0.63804	-0.64829	0.044617	0.20046	0.71387	-0.48626	0.1572	0.62971	0.86522	-0.20885	0.28261	0.94365	0.86022	-0.21722	0.66241	0.74705	1.0857	0.11857
341.3545	8.13	olomics Pc	0.56155	0.99951	0.9631	-0.05425	0.19759	0.99998	0.85548	-0.22519	0.35324	0.99941	0.90615	-0.14218	0.084319	0.38493	0.85054	-0.23355	0.9742	0.9989	1.0021	0.003078	0.01599	0.093702	0.70155	-0.51139	0.11323	0.64238	0.79687	-0.32759	0.017894	0.51556	0.75827	-0.39922	0.1666	0.4277	0.87766	-0.18826
1005.166	8.86	olomics Pc	0.56161	0.99951	1.2204	0.28736	0.87191	0.99998	1.0172	0.024654	0.28856	0.99941	1.1514	0.20335	0.094882	0.41348	0.7629	-0.39043	0.15308	0.4059	0.73924	-0.43589	0.025693	0.12008	0.69987	-0.51485	0.82957	0.9951	1.1044	0.14329	0.83066	0.99956	1.1662	0.22182	0.18772	0.45313	1.7837	0.83489
704.8622	0.5	domics Pos	0.56176	0.99987	1.045	0.063443	0.57469	0.99994	0.95653	-0.06412	0.71634	0.99996	1.0227	0.032315	0.15147	0.57987	0.91904	-0.12179	0.000508	0.003333	0.74814	-0.41861	0.28345	0.57064	0.88122	-0.18243	0.44934	0.80156	1.0549	0.077125	0.90944	0.98239	1.014	0.020069	0.017341	0.10064	1.2739	0.34931
155.9965	10.18	domics Pos	0.56215	0.99987	1.0497	0.069973	0.34141	0.99994	0.93145	-0.10244	0.75738	0.99996	1.0215	0.030665	0.80486	0.97861	0.97809	-0.03197	0.022227	0.056462	0.80009	-0.32177	0.69753	0.84476	0.96185	-0.05612	0.32982	0.73889	1.0872	0.12058	0.29889	0.9483	1.0709	0.098885	0.033938	0.12554	1.2454	0.31662
403.2984	6.49	olomics Pc	0.56252	0.99951	1.0828	0.11471	0.94435	0.99998	0.99207	-0.01149	0.27653	0.99941	0.87785	-0.18796	0.31649	0.81117	0.85984	-0.21787	0.128	0.35152	0.82914	-0.27031	0.33777	0.64718	0.86287	-0.21278	0.040318	0.39977	0.7826	-0.35366	0.13289	0.91598	0.80969	-0.30456	0.44612	0.71286	0.90092	-0.15053
90.9775	0.51	domics Pos	0.56264	0.99987	1.0203	0.029025	0.31419	0.99994	0.95155	-0.07165	0.38497	0.99996	0.95817	-0.06165	0.21394	0.66269	0.93415	-0.09828	0.000373	0.002624	0.76221	-0.39174	0.28299	0.57033	0.89444	-0.16094	0.67047	0.89896	0.9756	-0.03563	0.48318	0.96776	0.96354	-0.05358	0.023374	0.11051	1.2053	0.26945
301.2335	8.37	olomics Pc	0.5631	0.99951	0.94182	-0.08648	0.4757	0.99998	0.93114	-0.10294	0.069763	0.99941	0.81321	-0.2983	0.51306	0.98198	0.93694	-0.09398	0.83083	0.99688	0.97828	-0.03169	0.40609	0.71304	0.90316	-0.14695	0.78783	0.99391	0.97357	-0.03864	0.048865	0.67731	0.76892	-0.37909	0.0463	0.28541	0.81753	-0.29065
908.8266	10.02	domics Pos	0.56321	0.99987	0.95451	-0.06717	0.048055	0.99994	0.84977	-0.23486	0.13209	0.99996	0.88283	-0.17979	0.075411	0.46303	0.86264	-0.21317	0.036392	0.082109	0.81114	-0.30198	0.092127	0.32022	0.84528	-0.2425	0.44768	0.8014	0.9438	-0.08345	0.86503	0.98023	0.9872	-0.01859	0.037688	0.13071	1.175	0.23264
945.693	7.02	domics Pos	0.56336	0.99987	1.2767	0.35236	0.60911	0.99994	1.2342	0.30363	0.51755	0.99996	1.2582	0.33132	0.68382	0.96001	1.1142	0.15597	0.51029	0.58291	0.71864	-0.47665	0.15703	0.438	0.50439	-0.98739	0.11138	0.56029	0.42321	-1.2406	0.019463	0.52955	0.23272	-2.1033	0.005618	0.08815	0.13859	-2.8511
750.9727	12.01	olomics Ne	0.56346	0.99918	0.94029	-0.08882	0.025253	0.40327	0.74839	-0.41813	0.56089	0.99995	0.94366	-0.08366	0.29626	0.99985	0.89998	-0.15203	0.28587	0.99927	0.87272	-0.19642	0.6875	0.93515	1.0879	0.12157	0.13946	0.52448	1.1683	0.22439	0.62286	0.99512	0.95726	-0.06301	0.92925	0.99941	1.0079	0.011383
426.7949	5.77	domics Pos	0.56352	0.99987	0.91082	-0.13475	0.14781	0.99994	0.79237	-0.33575	0.3746	0.99996	0.85991	-0.21775	0.58333	0.92348	0.90943	-0.13697	0.087332	0.15789	0.71464	-0.48471	0.038132	0.17884	0.66757	-0.583	0.14522	0.60716	0.75822	-0.39932	0.087678	0.82455	0.7525	-0.41024	0.18314	0.29024	1.2066	0.27099
549.2947	5.1	olomics Pc	0.5636	0.99951	0.80484	-0.31322	0.58046	0.99998	0.76361	-0.38909	0.9016	0.99941	0.88059	-0.18346	0.80363	0.99905	1.0271	0.03854	0.34231	0.72513	1.2636	0.3375	0.030501	0.134	1.9797	0.98531	0.029213	0.34594	2.163	1.1131	0.34268	0.99956	1.3391	0.42131	0.16335	0.42246	1.4071	0.49272
614.8194	10.1	domics Pos	0.56428	0.99987	1.032	0.045408	0.042821	0.99994	0.93035	-0.10416	0.2274	0.99996	0.93116	-0.10291	0.032871	0.31629	0.88385	-0.17813	0.000111	0.00111	0.71171	-0.49063	0.21233	0.50393	0.90605	-0.14234	0.87357	0.97532	1.0084	0.012026	0.036887	0.64014	0.93377	-0.09886	0.10492	0.20712	1.179	0.23761
460.8781	10.15	domics Pos	0.56448	0.99987	1.0442	0.062407	0.77701	0.99994	0.9836	-0.02386	0.78799	0.99996	0.98266	-0.02524	0.60253	0.93327	0.95127	-0.07207	0.01364	0.038928	0.76219	-0.39178	0.59837	0.78243	0.95294	-0.06954	0.12239	0.58026	1.1525	0.20478	0.28097	0.94365	1.1226	0.16687	0.03491	0.12714	1.3234	0.40422
655.4592	9.5	olomics Pc	0.56469	0.99951	1.1778	0.23612	0.22795	0.99998	0.79704	-0.32727	0.95689	0.99941	0.99302	-0.0101	0.015096	0.20428	0.62781	-0.6716	0.014645	0.06516	0.59136	-0.75789	0.093922	0.28557	0.68473	-0.54638	0.88623	0.9951	1.0441	0.062201	0.60841	0.99956	0.92759	-0.10845	0.083294	0.33309	1.556	0.63788
344.9804	4.14	olomics Ne	0.56502	0.99918	1.0215	0.030649	0.022838	0.40327	0.87102	-0.19922	0.97914	0.99995	0.99971	-0.00042	0.58585	0.99985	1.0457	0.064498	0.89565	0.99927	1.0069	0.009898	0.28862	0.71345	1.065	0.090859	0.52096	0.83286	1.0487	0.068643	0.8557	0.99512	1.0121	0.017287	0.80351	0.99941	0.9882	-0.01713
1116.237	9.02	olomics Pc	0.56507	0.99951	1.1991	0.26198	0.32276	0.99998	0.84833	-0.23729	0.59494	0.99941	0.94631	-0.07962	0.007282	0.1948	0.64788	-0.62621	0.03629	0.13246	0.62177	-0.68555	0.03174	0.13696	0.71874	-0.47645	0.99441	0.99906	1.0583	0.081757	0.93054	0.99956	1.0357	0.050603	0.038232	0.28204	1.5469	0.62942
600.3196	7.14	olomics Ne	0.56519	0.99918	0.91266	-0.13185	0.70804	0.94774	0.93986	-0.08948	0.87406	0.99995	1.0017	0.002448	0.51574	0.99985	0.90574	-0.14284	0.038668	0.99927	0.62856	-0.66988	0.30014	0.71853	0.84712	-0.23936	0.73752	0.92171	0.94477	-0.08196	0.41477	0.99512	1.1016	0.13957	0.27927	0.99941	1.1624	0.21714
190.9281	12.74	olomics Ne	0.5655	0.99918	0.97481	-0.03681	0.040499	0.46524	0.89845	-0.15449	0.64542	0.99995	1.0164	0.023449	0.63059	0.99985	1.0382	0.054036	0.99405	0.99927	1.0008	0.001224	0.31015	0.72837	1.0924	0.12754	0.2729	0.65543	1.0528	0.074273	0.96865	0.99512	1.0058	0.008325	0.68113	0.99941	0.98198	-0.02623
875.0664	8.95	olomics Pc	0.56571	0.99951	1.2066	0.27098	0.19872	0.99998	0.82107	-0.28443	0.8507	0.99941	1.0376	0.053247	0.019745	0.21701	0.68133	-0.55358	0.018963	0.080723	0.58405	-0.77583	0.009708	0.079214	0.83039	-0.26814	0.69168	0.99391	1.1589	0.2127	0.91275	0.99956	1.0164	0.023438	0.24928	0.51729	1.7046	0.76942
356.9087	10.17	domics Pos	0.56577	0.99987	1.0483	0.068018	0.11574	0.99994	0.85741	-0.22194	0.10987	0.99996	0.85927	-0.21882	0.016974	0.24438	0.83857	-0.254	0.002675	0.011235	0.74857	-0.4178	0.28664	0.57283	0.9059	-0.14258	0.88962	0.97532	0.99231	-0.01113	0.45079	0.96776	0.94286	-0.08489	0.13756	0.24298	1.1213	0.16513
785.5881	5.04	domics Pos	0.56622	0.99987	1.0502	0.070657	0.6747	0.99994	0.96258	-0.05503	0.65301	0.99996	0.96008	-0.05877	0.36709	0.82143	0.90547	-0.14327	0.034756	0.079261	0.78302	-0.35288	0.94736	0.97163	0.99914	-0.00124	0.28091	0.7098	1.1088	0.14901	0.82343	0.97988	0.97993	-0.02925	0.067574	0.16764	1.2071	0.2716
767.5416	9.29	olomics Ne	0.56692	0.99918	0.94585	-0.08032	0.073828	0.50118	0.78199	-0.35477	0.29777	0.99995	0.77137	-0.37451	0.43189	0.99985	0.88858	-0.17042	0.028301	0.99927	0.68016	-0.55606	0.97698	0.9943	1.0384	0.054307	0.31241	0.68662	0.77076	-0.37564	0.96452	0.99512	1.0059	0.008458	0.2966	0.99941	0.86138	-0.21527
898.5786	9.49	olomics Ne	0.567	0.99918	1.0698	0.097294	0.16603	0.59413	0.89795	-0.1553	0.23433	0.99995	1.0866	0.11986	0.84461	0.99985	1.0223	0.031793	0.72297	0.99927	1.0323	0.045825	0.040405	0.48405	1.174	0.23146	0.038058	0.35842	1.166	0.22153	0.032552	0.99512	1.1327	0.17978	0.69693	0.99941	1.0343	0.048639
378.2391	4.42	olomics Pc	0.56811	0.99951	1.1636	0.21855	0.96179	0.99998	0.99641	-0.00519	0.91541	0.99941	1.0377	0.053389	0.41265	0.90278	1.1548	0.20769	0.005301	0.030601	0.51264	-0.96399	0.82363	0.94579	0.97989	-0.0293	0.76372	0.99391	1.0509	0.07163	0.7989	0.99956	1.094	0.12964	0.33054	0.60717	1.1311	0.17778
880.5915	5.73	domics Pos	0.56878	0.99987	1.0354	0.050134	0.22724	0.99994	0.93437	-0.09793	0.41079	0.99996	0.95145	-0.07181	0.58398	0.92395	0.95259	-0.07008	0.028307	0.068011	0.75031	-0.41444	0.19322	0.47937	0.89944	-0.1529	0.41154	0.78028	0.93044	-0.10402	0.93857	0.98643	0.99878	-0.00176	0.03762	0.13071	1.4928	0.578
540.8894	10.03	domics Pos	0.56896	0.99987	1.0352	0.049897	0.62574	0.99994	0.98421	-0.02296	0.84578	0.99996	0.99354	-0.00935	0.48668	0.88725	0.96096	-0.05745	0.000934	0.005291	0.79076	-0.33868	0.24063	0.52864	0.87593	-0.19111	0.36567	0.76025	1.0847	0.11732	0.62872	0.97969	0.96879	-0.04575	0.008809	0.092495	1.2439	0.31484
1069.516	6.12	domics Pos	0.56978	0.99987	1.0766	0.10648	0.7897	0.99994	1.0313	0.044515	0.73699	0.99996	1.0394	0.055745	0.80892	0.98074	0.96418	-0.05263	0.14203	0.22706	0.8087	-0.30632	0.049149	0.21583	0.71519	-0.4836	0.089541	0.51809	0.79473	-0.33147	0.19903	0.92582	0.83387	-0.26211	0.56679	0.66152	1.1003	0.13795
1062.752	10.05	domics Pos	0.56992	0.99987	0.94681	-0.07886	0.24032	0.99994	0.89524	-0.15965	0.10254	0.99996	0.86264	-0.21316	0.17511	0.60979	0.88322	-0.17915	0.008755	0.027657	0.77192	-0.37349	0.17918	0.46725	0.83003	-0.26876	0.3947	0.76955	0.9206	-0.11936	0.91407	0.98248	0.99248	-0.01089	0.10435	0.20691	1.1396	0.18859
749.5705	8.4	olomics Ne	0.57009	0.99918	1.0332	0.04711	0.048641	0.47152	0.88609	-0.17447	0.9788	0.99995	1.0056	0.008058	0.78087	0.99985	1.0121	0.017357	0.35637	0.99927	1.0569	0.079843	0.28096	0.70756	1.0847	0.11732	0.14941	0.53641	1.0567	0.079591	0.95261	0.99512	0.99686	-0.00453	0.005599	0.99941	0.8695	-0.20174
1034.771	10.03	domics Pos	0.57125	0.99987	1.0222	0.031666	0.94125	0.99994	1.005	0.007234	0.009868	0.99996	0.89971	-0.15246	0.049387	0.37885	0.89124	-0.16611	0.002629	0.011063	0.74273	-0.42908	0.047021	0.20921	0.85162	-0.23172	0.79452	0.95091	0.98822	-0.01709	0.60769	0.97358	0.98773	-0.01782	0.00918	0.092545	1.2116	0.27696
349.2117	2.18	domics Pos	0.57165	0.99987	0.91614	-0.12636	0.9526	0.99994	0.98334	-0.02424	0.22524	0.99996	0.83083	-0.26738	0.071152	0.45473	0.74898	-0.417	1.6E-05	0.000322	0.14086	-2.8277	0.004505	0.071311	0.52071	-0.94144	0.12064	0.57768	0.78575	-0.34786	0.11898	0.88537	0.6901	-0.53512	0.39259	0.50132	0.85747	-0.22184
468.8182	0.56	domics Pos	0.57189	0.99987	1.0574	0.080499	0.61354	0.99994	1.0339	0.04812	0.59685	0.99996	0.95831	-0.06143	0.10485	0.52894	0.8476	-0.23855	0.011246	0.033632	0.7836	-0.35181	0.026824	0.14508	0.8377	-0.25549	0.020098	0.33809	0.80178	-0.31872	0.9718	0.9921	0.99898	-0.00148	0.1183	0.22312	1.1865	0.24676
349.21	6.42	olomics Pc	0.57241	0.99951	0.90056	-0.1511	0.71002	0.99998	1.0494	0.069502	0.1615	0.99941	0.77652	-0.36491	0.38754	0.88571	0.85464	-0.22661	0.000124	0.001891	0.29039	-1.784	0.005747	0.064803	0.56569	-0.82192	0.15232	0.72698	0.71144	-0.49119	0.17641	0.96992	0.7435	-0.4276	0.22023	0.4882	0.76768	-0.38142
765.5781	5.6	domics Pos	0.57248	0.99987	1.0313	0.044435	0.18221	0.99994	0.93596	-0.09549	0.45706	0.99996	0.94725	-0.07819	0.075304	0.46303	0.89668	-0.15733	0.001165	0.006128	0.72104	-0.47184	0.005515	0.075369	0.79796	-0.32561	0.33983	0.74343	1.0725	0.10092	0.99444	0.99758	1.0034	0.004967	0.021905	0.10841	1.2112	0.27648
266.2769	8.37	olomics Pc	0.57256	0.99951	0.95641	-0.06429	0.47982	0.99998	0.93311	-0.09988	0.31905	0.99941	0.92502	-0.11244	0.17787	0.60312	0.93372	-0.09894	0.91711	0.99688	0.99717	-0.00409	0.2249	0.51041	0.92895	-0.10633	0.21361	0.80949	0.90736	-0.14025	0.005064	0.47452	0.81366	-0.29751	0.008518	0.2688	0.80489	-0.31314
486.2945	1.63	domics Pos	0.57323	0.99987	1.1293	0.1754	0.6701	0.99994	0.92796	-0.10786	0.76187	0.99996	0.97422	-0.03768	0.98352	0.99652	1.0357	0.050642	0.000229	0.001839	0.256	-1.9658	0.14757	0.42234	0.73555	-0.4431	0.85196	0.96938	1.0376	0.053272	0.67567	0.97988	1.1379	0.18638	0.050667	0.14725	1.5819	0.66163
397.3293	7.35	olomics Pc	0.57323	0.99951	0.97586	-0.03525	0.84039	0.99998	0.99711	-0.00418	0.085605	0.99941	0.90583	-0.14269	0.68186	0.99905	1.0412	0.058245	0.983	0.9989	1.0011	0.001531	0.89812	0.96919	0.99832	-0.00242	0.72185	0.99391	1.0384	0.054389	0.03826	0.62445	0.85064	-0.23339	0.7633	0.89917	1.0192	0.027447
1077.34	8.36	olomics Pc	0.57398	0.99951	1.0794	0.11016	0.95207	0.99998	1.0598	0.083738	0.95993	0.99941	1.019	0.027097	0.021688	0.2227	1.2398	0.31012	0.033495	0.12516	1.2573	0.33037	0.79989	0.94009	1.0007	0.001018	0.98223	0.99744	1.0261	0.037124	0.58735	0.99956	0.93979	-0.08959	0.25841	0.52959	0.81903	-0.288
283.2572	3.52	lomics Neg	0.57424	0.99977	0.8895	-0.16893	0.62393	0.99904	0.87383	-0.19458	0.076405	0.99994	0.53727	-0.89629	0.067131	0.58182	0.53341	-0.90669	0.097077	0.99989	0.53656	-0.89819	0.13547	0.99653	0.49873	-1.0037	0.003542	0.0953	0.28689	-1.8014	0.010962	0.46971	0.23129	-2.1122	0.043216	0.405	0.45929	-1.1225
135.118	6.98	olomics Pc	0.57431	0.99951	1.109	0.14923	0.54099	0.99998	0.90552	-0.14318	0.089673	0.99941	0.77626	-0.36539	0.044665	0.28517	0.59154	-0.75744	0.001738	0.013927	0.36707	-1.4459	0.000746	0.041543	0.38117	-1.3915	0.004464	0.20152	0.46268	-1.1119	0.029594	0.58535	0.56298	-0.82884	0.064614	0.30653	0.69261	-0.52988
817.5896	5.2	domics Pos	0.57467	0.99987	1.0593	0.083117	0.73263	0.99994	0.97661	-0.03414	0.90884	0.99996	0.99594	-0.00587	0.67661	0.95843	0.97748	-0.03286	0.01175	0.034676	0.80407	-0.31461	0.74595	0.86962	0.97899	-0.03064	0.17348	0.64606	1.1251	0.17009	0.96273	0.9885	1.0077	0.01104	0.88135	0.91353	1.0241	0.034329
393.719	2.86	olomics Pc	0.57498	0.99951	1.1024	0.14064	0.031879	0.99998	0.78745	-0.34474	0.53413	0.99941	0.92788	-0.10799	0.004255	0.18109	0.56536	-0.82277	0.002	0.015437	0.54043	-0.88782	0.002102	0.045856	0.68099	-0.5543	0.49762	0.99391	0.92087	-0.11893	0.53822	0.99956	0.93682	-0.09415	0.055051	0.29746	1.9616	0.97206
396.224	6.83	olomics Pc	0.57511	0.99951	0.95664	-0.06396	0.50243	0.99998	0.93528	-0.09652	0.005966	0.99941	0.75729	-0.40108	0.020036	0.21915	0.58616	-0.77063	0.000386	0.004677	0.59958	-0.73798	0.004166	0.054724	0.4526	-1.1437	0.00371	0.20152	0.47764	-1.066	0.01051	0.47452	0.49221	-1.0226	0.031333	0.2688	0.58765	-0.76697
351.2179	4.78	domics Pos	0.57526	0.99987	1.0722	0.1006	0.92771	0.99994	0.99089	-0.0132	0.58558	0.99996	1.0603	0.084516	0.83406	0.98621	1.0116	0.016613	0.045487	0.097004	0.68483	-0.54619	0.58817	0.77531	0.89896	-0.15367	0.90664	0.97902	1.0144	0.020636	0.90884	0.98239	0.98379	-0.02358	0.32045	0.43064	1.1568	0.21016
337.1684	2.28	domics Pos	0.57537	0.99987	0.89584	-0.15868	0.33763	0.99994	0.81315	-0.29841	0.033164	0.99996	0.59766	-0.74259	0.014812	0.2319	0.4623	-1.1131	0.001304	0.006671	0.40501	-1.304	0.010286	0.08787	0.34562	-1.5327	0.021514	0.33809	0.44694	-1.1619	0.008553	0.52955	0.38748	-1.3678	0.051913	0.14847	0.53565	-0.90063
650.4724	2.08	domics Pos	0.5755	0.99987	1.1023	0.14056	0.74692	0.99994	0.86188	-0.21444	0.59225	0.99996	0.81385	-0.29717	0.036301	0.33474	0.44658	-1.163	0.00175	0.008195	0.22819	-2.1317	0.00745	0.082061	0.35079	-1.5113	0.059285	0.4364	0.46667	-1.0995	0.092836	0.83714	0.51893	-0.94639	0.40306	0.51041	0.74568	-0.42338
504.3453	4.6	domics Pos	0.57551	0.99987	0.94507	-0.0815	0.25056	0.99994	0.92498	-0.11251	0.055625	0.99996	1.1031	0.14157	0.98181	0.99652	1.017	0.024364	0.01834	0.048805	0.73339	-0.44735	0.15894	0.44042	1.1428	0.19262	0.81691	0.96018	1.0102	0.014659	0.71846	0.97988	0.97514	-0.03632	0.88414	0.9151	1.0631	0.088335
530.29	10.05	olomics Pc	0.57574	0.99951	1.05	0.070368	0.90831	0.99998	0.99875	-0.0018	0.70078	0.99941	0.96717	-0.04816	0.58924	0.99905	0.94258	-0.08532	0.53673	0.92674	0.95219	-0.07067	0.022936	0.11219	1.2218	0.28895	0.004535	0.20152	1.4736	0.55935	0.077167	0.7766	1.1576	0.21117	0.23588	0.50374	1.136	0.18399
235.1675	6.41	olomics Pc	0.57574	0.99951	0.88202	-0.18111	0.34161	0.99998	0.82104	-0.28447	0.13198	0.99941	0.71683	-0.48029	0.18351	0.61171	0.76456	-0.38731	6.89E-05	0.001205	0.21468	-2.2197	0.005705	0.064803	0.47866	-1.0629	0.058644	0.48999	0.60166	-0.73297	0.039847	0.6387	0.5577	-0.84244	0.080556	0.3287	0.60309	-0.72955
840.6387	5.64	domics Pos	0.57623	0.99987	0.92724	-0.10899	0.96749	0.99994	0.98951	-0.01521	0.61305	0.99996	0.92916	-0.106	0.40545	0.84674	0.89383	-0.16193	0.033725	0.077516	0.71515	-0.48369	0.15715	0.438	0.79811	-0.32533	0.2356	0.67693	0.82021	-0.28593	0.33286	0.96224	0.86375	-0.21131	0.15824	0.26486	1.1829	0.24235
535.2988	1.29	domics Pos	0.57698	0.99987	1.0731	0.10185	0.77799	0.99994	1.0459	0.064756	0.88234	0.99996	1.0157	0.022513	0.94565	0.9925	0.9884	-0.01684	0.21327	0.30698	0.77367	-0.37021	0.14974	0.42577	1.1567	0.20995	0.012628	0.3324	1.405	0.49052	0.23936	0.94365	1.1272	0.17278	0.003751	0.085302	1.5391	0.62207
344.8675	9.92	domics Pos	0.57739	0.99987	0.94864	-0.07607	0.24761	0.99994	0.88393	-0.178	0.25216	0.99996	0.89887	-0.15382	0.16109	0.59245	0.86161	-0.21489	0.003796	0.014724	0.70234	-0.50976	0.10142	0.33938	0.81246	-0.29963	0.31506	0.72948	0.90325	-0.1468	0.26053	0.94365	0.88534	-0.1757	0.89509	0.92392	1.0257	0.036648
828.551	4.77	domics Pos	0.57742	0.99987	1.0386	0.054573	0.53116	0.99994	0.94939	-0.07492	0.6611	0.99996	0.97066	-0.04297	0.52196	0.89938	0.95544	-0.06576	0.001786	0.008334	0.75188	-0.41142	0.33646	0.60864	0.8935	-0.16246	0.55813	0.85427	0.95419	-0.06765	0.5521	0.96776	0.9549	-0.06658	0.11855	0.22318	1.1097	0.15014
334.1601	2.28	domics Pos	0.57748	0.99987	0.93839	-0.09174	0.3334	0.99994	0.85421	-0.22734	0.057776	0.99996	0.71976	-0.47441	0.015998	0.24	0.55149	-0.8586	0.00015	0.001372	0.44156	-1.1793	0.015401	0.10468	0.41442	-1.2708	0.02773	0.34781	0.51473	-0.95811	0.012625	0.52955	0.45158	-1.1469	0.05637	0.15341	0.57333	-0.80257
721.4504	1.53	domics Pos	0.57766	0.99987	1.2713	0.34629	0.032831	0.99994	0.68677	-0.54211	0.66295	0.99996	1.0997	0.13717	0.020263	0.25894	0.6643	-0.5901	0.003869	0.01495	0.53071	-0.91399	0.027443	0.14615	0.65688	-0.6063	0.91939	0.981	1.0792	0.10994	0.9404	0.98646	1.0656	0.091628	0.19885	0.30729	1.4934	0.57865
502.9726	12.01	olomics Ne	0.57769	0.99918	1.0638	0.089253	0.73036	0.95443	1.0533	0.074933	0.23696	0.99995	1.1467	0.19748	0.9172	0.99985	1.0027	0.003855	0.50192	0.99927	1.1039	0.14256	0.073633	0.52394	1.163	0.21789	0.038321	0.35842	1.2706	0.34555	0.91959	0.99512	1.0123	0.017696	0.90079	0.99941	1.0046	0.006646
307.2553	6.41	olomics Pc	0.57796	0.99951	0.81441	-0.29617	0.8247	0.99998	0.89422	-0.16129	0.55879	0.99941	0.809	-0.30579	0.43542	0.91929	0.76651	-0.38363	0.00108	0.009801	0.21391	-2.2249	0.10817	0.31725	0.59177	-0.75689	0.25929	0.86879	0.6659	-0.58661	0.23365	0.99956	0.66515	-0.58826	0.1394	0.39346	0.60038	-0.73606
944.9735	12.03	olomics Ne	0.57861	0.99918	0.92518	-0.1122	0.062672	0.50118	0.80373	-0.31522	0.35924	0.99995	0.87362	-0.19493	0.32134	0.99985	0.87238	-0.19697	0.35533	0.99927	1.1015	0.13953	0.77465	0.95062	0.9617	-0.05634	0.82298	0.95121	0.9809	-0.02782	0.045905	0.99512	0.76718	-0.38236	0.82513	0.99941	0.97967	-0.02964
437.2814	1.35	domics Pos	0.5789	0.99987	0.83427	-0.26142	0.88546	0.99994	0.93009	-0.10455	0.69794	0.99996	0.88157	-0.18185	0.72334	0.96819	0.87434	-0.19373	0.57265	0.63512	0.83512	-0.25994	0.23601	0.52474	1.3134	0.39331	0.018557	0.33809	1.9014	0.92703	0.15077	0.91321	1.3727	0.457	0.023838	0.11051	1.8246	0.86762
831.5048	6.49	olomics Pc	0.57913	0.99951	1.1487	0.20006	0.3399	0.99998	0.81759	-0.29056	0.75582	0.99941	0.93362	-0.09909	0.031496	0.24911	0.62076	-0.6879	0.47053	0.86981	0.85675	-0.22305	0.053893	0.1969	0.67597	-0.56498	0.84431	0.9951	0.96807	-0.04682	0.84001	0.99956	1.0181	0.025835	0.94612	0.97875	0.99527	-0.00684
782.4372	0.63	domics Pos	0.58009	0.99987	0.95687	-0.0636	0.37346	0.99994	0.9462	-0.07979	0.71743	0.99996	1.0519	0.07301	0.040825	0.34959	0.80374	-0.31521	0.025153	0.062037	0.71357	-0.48687	0.41807	0.65619	0.91663	-0.12558	0.12792	0.58744	0.87569	-0.19151	0.81199	0.97988	1.0668	0.093273	0.13272	0.23769	1.3253	0.40636
406.3299	6.84	olomics Pc	0.58012	0.99951	1.0592	0.082989	0.63106	0.99998	1.0735	0.10227	0.089635	0.99941	0.86335	-0.21199	0.51044	0.98198	0.92119	-0.11843	0.049726	0.16807	0.82024	-0.28588	0.059807	0.21097	0.82426	-0.27882	0.17174	0.74875	0.89081	-0.1668	0.022169	0.51556	0.76996	-0.37714	0.1817	0.44604	0.84805	-0.23778
214.2528	4.13	olomics Pc	0.58037	0.99951	0.96345	-0.05372	0.731	0.99998	1.0976	0.13437	0.61763	0.99941	0.96757	-0.04756	0.45696	0.9358	0.95633	-0.06442	0.1937	0.48387	1.1868	0.24705	0.99421	0.99706	1.0418	0.05903	0.24644	0.85494	1.0617	0.086336	0.045693	0.66241	0.89416	-0.1614	0.16858	0.42998	1.1068	0.14644
522.5981	10.05	domics Pos	0.58038	0.99987	1.1172	0.1599	0.92097	0.99994	1.007	0.010074	0.23454	0.99996	1.1059	0.14521	0.29318	0.75244	1.1987	0.26142	0.19853	0.29227	0.92308	-0.11547	0.98178	0.98878	1.0094	0.013532	0.018897	0.33809	1.2851	0.36186	0.11959	0.88537	1.1885	0.2492	0.000566	0.07528	1.2854	0.36217
298.3108	3.25	domics Pos	0.58042	0.99987	0.95306	-0.06936	0.060499	0.99994	0.76943	-0.37814	0.20936	0.99996	0.87426	-0.19386	0.092148	0.50411	0.86552	-0.20836	0.004576	0.017023	0.75577	-0.40399	0.007193	0.082061	0.65426	-0.61206	0.14256	0.60601	0.78761	-0.34445	0.018778	0.52955	0.77186	-0.37358	0.68577	0.76614	0.96252	-0.05511
806.5672	5.08	domics Pos	0.58052	0.99987	1.0517	0.072724	0.64482	0.99994	0.95191	-0.0711	0.70847	0.99996	0.96327	-0.05399	0.45191	0.8738	0.92622	-0.11057	0.016471	0.044828	0.78766	-0.34436	0.17	0.45681	0.86757	-0.20495	0.63564	0.88231	0.95668	-0.06389	0.36053	0.96776	0.92368	-0.11454	0.029039	0.11767	1.2022	0.2657
933.5423	6.12	domics Pos	0.58064	0.99987	1.0658	0.091906	0.81818	0.99994	1.034	0.048236	0.80964	0.99996	1.0262	0.037309	0.51224	0.89678	0.93312	-0.09987	0.046844	0.099122	0.74557	-0.42358	0.014611	0.10138	0.72397	-0.46601	0.012234	0.3324	0.72118	-0.47156	0.091148	0.82947	0.82965	-0.26943	0.97778	0.98371	1.0216	0.030839
438.2985	5.45	olomics Pc	0.58092	0.99951	0.88511	-0.17608	0.63863	0.99998	0.90474	-0.14442	0.45699	0.99941	0.84578	-0.24165	0.82056	0.99905	0.96644	-0.04924	0.000226	0.003096	0.18985	-2.3971	0.26744	0.55938	0.76725	-0.38222	0.65517	0.99391	1.1036	0.14223	0.95836	0.99956	1.009	0.01289	0.53867	0.77622	1.1545	0.20726
575.4097	1.53	domics Pos	0.58099	0.99987	0.92113	-0.11852	0.021639	0.99994	0.70812	-0.49793	0.44096	0.99996	0.8921	-0.16473	0.37101	0.82414	0.86154	-0.21501	0.00635	0.021774	0.61948	-0.69086	0.52925	0.7362	0.90407	-0.14549	0.82691	0.96364	0.96709	-0.04828	0.57963	0.9703	0.9201	-0.12014	0.92588	0.94468	1.009	0.012942
1082.545	5.75	domics Pos	0.58119	0.99987	1.0667	0.093206	0.75055	0.99994	0.96362	-0.05347	0.49975	0.99996	0.91405	-0.12965	0.625	0.94343	0.94246	-0.08549	0.0158	0.04353	0.73053	-0.45299	0.30993	0.58859	0.85159	-0.23177	0.80465	0.95494	1.0317	0.044978	0.37173	0.96776	0.91353	-0.13047	0.24418	0.3523	1.2437	0.31465
806.5909	10.12	olomics Ne	0.58124	0.99918	1.039	0.055238	0.15338	0.58235	0.93237	-0.10103	0.32031	0.99995	1.0638	0.089249	0.66541	0.99985	0.98055	-0.02834	0.18865	0.99927	1.064	0.089548	0.032632	0.47167	1.2073	0.27177	0.10706	0.47462	1.1919	0.25324	0.11843	0.99512	1.0857	0.11869	0.53728	0.99941	1.0628	0.087845
383.7901	4.66	domics Pos	0.58128	0.99987	1.1086	0.14871	0.2574	0.99994	0.87322	-0.19559	0.88024	0.99996	1.0318	0.045108	0.95556	0.9937	1.0185	0.026423	0.023575	0.058969	0.76272	-0.39078	0.18433	0.47109	0.81981	-0.28664	0.98293	0.99431	1.0156	0.022355	0.58993	0.97073	0.94505	-0.08154	0.65705	0.7432	0.96978	-0.04428
425.8886	10.14	domics Pos	0.58135	0.99987	1.0333	0.047302	0.015012	0.99994	0.72189	-0.47014	0.2198	0.99996	0.85538	-0.22536	0.33469	0.79326	0.8982	-0.1549	0.000433	0.002937	0.61873	-0.69262	0.021962	0.12963	0.78936	-0.34125	0.42784	0.79094	0.92814	-0.10759	0.063306	0.73915	0.86368	-0.21144	0.45632	0.56145	1.0672	0.09378
1175.597	6.07	domics Pos	0.5814	0.99987	0.95486	-0.06664	0.20723	0.99994	0.83894	-0.25336	0.2242	0.99996	0.85509	-0.22586	0.48558	0.88725	0.94219	-0.08591	0.010367	0.031573	0.75569	-0.40412	0.41944	0.65671	0.87361	-0.19494	0.23049	0.67417	0.87644	-0.19027	0.21177	0.92922	0.88657	-0.1737	0.90052	0.92727	1.011	0.015759
707.4643	4.49	domics Pos	0.5821	0.99987	1.2257	0.29366	0.033958	0.99994	0.73118	-0.45169	0.02767	0.99996	0.91066	-0.13501	0.000278	0.164	0.59876	-0.73996	0.000345	0.002463	0.52266	-0.93606	0.008343	0.084185	0.62875	-0.66944	0.34822	0.74762	0.85725	-0.22221	0.56889	0.9703	0.94513	-0.08142	0.072157	0.17377	1.7307	0.79132
960.5604	6.21	domics Pos	0.58241	0.99987	1.0963	0.1326	0.90473	0.99994	0.98298	-0.02477	0.66178	0.99996	1.0579	0.081181	0.94526	0.99236	0.98721	-0.01858	0.037006	0.083247	0.74293	-0.42869	0.082731	0.29844	0.75438	-0.40664	0.27527	0.70632	0.85505	-0.22593	0.038808	0.64308	0.74899	-0.41698	0.51808	0.6179	1.0678	0.094632
1153.763	0.51	domics Pos	0.58268	0.99987	1.071	0.098961	0.55687	0.99994	0.92546	-0.11176	0.70121	0.99996	1.0353	0.050115	0.88676	0.9874	0.97803	-0.03205	0.026849	0.065269	0.74556	-0.4236	0.93731	0.9677	1.0123	0.01767	0.31905	0.72948	1.109	0.14931	0.59788	0.97073	1.05	0.07033	0.40035	0.50833	1.1194	0.16272
667.527	5.92	domics Pos	0.58295	0.99987	1.062	0.086749	0.96395	0.99994	1.0177	0.025333	0.80387	0.99996	0.97635	-0.03453	0.86986	0.9874	1.0235	0.033473	0.046737	0.09895	0.72941	-0.45519	0.27643	0.56257	0.81342	-0.29793	0.20079	0.64891	0.84547	-0.24217	0.32012	0.9524	0.83353	-0.26269	0.6884	0.76795	1.0738	0.10275
395.7575	5.67	domics Pos	0.583	0.99987	0.94435	-0.0826	0.35295	0.99994	0.90531	-0.14351	0.68408	0.99996	0.96505	-0.05132	0.02554	0.28227	0.83623	-0.25802	0.00166	0.00786	0.67469	-0.5677	0.068787	0.2669	0.78474	-0.34972	0.21966	0.6621	1.2143	0.28016	0.80122	0.97988	1.0307	0.04361	0.021504	0.1083	1.2325	0.3016
914.5362	5.2	domics Pos	0.58351	0.99987	0.97246	-0.04029	0.63672	0.99994	0.96972	-0.04436	0.43533	0.99996	0.93538	-0.09637	0.22406	0.68008	0.89398	-0.16169	0.009456	0.029511	0.79071	-0.33877	0.26138	0.54699	0.84052	-0.25065	0.68889	0.90885	1.043	0.060806	0.10042	0.85376	0.84371	-0.24518	0.79791	0.8503	1.0563	0.078958
366.1013	0.6	lomics Neg	0.58406	0.99977	1.0852	0.11796	0.69804	0.99904	1.2686	0.34327	0.56622	0.99994	1.428	0.51395	0.65098	0.73576	1.1447	0.19493	0.74681	0.99989	1.1131	0.15464	0.58561	0.99653	1.712	0.77564	0.01886	0.30175	2.165	1.1143	0.27833	0.64871	1.27	0.34481	0.15091	0.4061	1.5232	0.60708
1131.771	10.02	domics Pos	0.58409	0.99987	1.0526	0.073987	0.6915	0.99994	0.95776	-0.06227	0.90799	0.99996	1.0125	0.017931	0.89779	0.9874	0.98779	-0.01773	0.025515	0.062725	0.76675	-0.38318	0.82569	0.91363	1.013	0.018613	0.95691	0.98985	1.003	0.004389	0.57351	0.9703	0.93403	-0.09846	0.020851	0.10765	1.3271	0.40827
420.973	4.97	olomics Ne	0.58411	0.99918	1.028	0.039901	0.0498	0.47157	0.89226	-0.16446	0.97401	0.99995	1.003	0.004325	0.92921	0.99985	1.0076	0.010884	0.98357	0.99927	0.99957	-0.00062	0.19615	0.65203	1.0943	0.13004	0.1331	0.52398	1.0928	0.12803	0.65683	0.99512	1.0254	0.036207	0.85593	0.99941	1.0074	0.010682
564.322	1.38	domics Pos	0.58426	0.99987	1.2011	0.26441	0.72395	0.99994	0.90784	-0.1395	0.94961	0.99996	0.9515	-0.07172	0.76386	0.97526	1.0635	0.088784	0.33388	0.42764	0.78833	-0.34313	0.036595	0.17398	1.5365	0.61969	0.17853	0.6466	1.3774	0.46198	0.21047	0.92669	1.3169	0.39719	0.04918	0.14615	1.7185	0.78116
819.6067	5.61	domics Pos	0.58435	0.99987	1.0638	0.0892	0.91862	0.99994	0.98665	-0.01939	0.95434	0.99996	0.99433	-0.0082	0.61589	0.93856	0.9459	-0.08025	0.044474	0.095483	0.77033	-0.37646	0.29295	0.57548	0.8831	-0.17936	0.97059	0.99324	0.9974	-0.00376	0.65792	0.97988	0.95341	-0.06884	0.58004	0.67348	1.0444	0.062619
900.5916	9.94	olomics Ne	0.58438	0.99918	1.0486	0.068496	0.16002	0.5837	0.91238	-0.13229	0.99748	0.99995	1.0078	0.011159	0.87272	0.99985	1.0304	0.043185	0.8962	0.99927	0.99343	-0.0095	0.044022	0.48405	1.2163	0.28244	0.52317	0.83472	0.92707	-0.10925	0.23702	0.99512	1.0961	0.13239	0.086037	0.99941	1.4189	0.50481

155.0141	12.16	olomics Pc	0.58451	0.99951	1.0842	0.11669	0.27704	0.99998	1.159	0.21283	0.18364	0.99941	1.1045	0.1434	0.84843	0.99905	0.98331	-0.02428	0.20958	0.51206	1.0899	0.12418	0.19068	0.46283	1.1107	0.15149	0.010159	0.24769	1.2499	0.32177	0.24184	0.99956	1.1073	0.14702	0.2367	0.50447	1.1516	0.20368
243.9425	9.83	lomics Pos	0.58545	0.99987	1.0179	0.025579	0.082544	0.99994	0.87833	-0.18717	0.00826	0.99996	0.88569	-0.17513	0.28962	0.75244	0.94839	-0.07645	0.015901	0.043744	0.78928	-0.34139	0.45234	0.68195	0.92555	-0.11161	0.85064	0.9685	0.99406	-0.00859	0.085976	0.82317	0.91244	-0.1322	0.063499	0.16163	1.0988	0.13594
430.9147	0.5	lomics Pos	0.58608	0.99987	1.0155	0.022141	0.24053	0.99994	0.93886	-0.09102	0.19229	0.99996	0.93334	-0.09953	0.043491	0.36496	0.89989	-0.15217	5.15E-05	0.00066	0.74218	-0.43015	0.24515	0.53144	0.88362	-0.1785	0.5079	0.8319	0.96076	-0.05775	0.25611	0.94365	0.9456	-0.08071	0.031307	0.12145	1.1852	0.24517
564.3064	0.93	lomics Pos	0.58717	0.99987	0.90201	-0.14878	0.90328	0.99994	1.0111	0.015861	0.66836	0.99996	1.0705	0.09823	0.58807	0.92544	1.0815	0.11301	0.62393	0.67995	0.90513	-0.1438	0.089816	0.31628	1.4069	0.49257	0.022834	0.33809	1.6684	0.73848	0.83225	0.97988	1.0049	0.007049	0.69773	0.77438	1.0837	0.11596
467.3321	6.08	olomics Pc	0.58759	0.99951	0.88381	-0.17819	0.91137	0.99998	1.0164	0.023481	0.22864	0.99941	0.78029	-0.35792	0.88678	0.99905	0.94645	-0.0794	0.004115	0.025519	0.32305	-1.6302	0.68408	0.90883	0.90525	-0.14361	0.19726	0.77954	1.2609	0.33444	0.9473	0.99956	1.026	0.036995	0.19209	0.4564	1.3305	0.41202
432.2791	2.72	lomics Pos	0.58766	0.99987	1.0512	0.071996	0.16959	0.99994	0.81704	-0.29152	0.23553	0.99996	0.82649	-0.27493	0.044133	0.36496	0.66697	-0.58431	0.001834	0.008483	0.51416	-0.95972	0.023347	0.13415	0.50474	-0.98638	0.021004	0.33809	0.61563	-0.69986	0.073892	0.77699	0.62652	-0.67457	0.26191	0.37138	0.7991	-0.32354
205.0753	10.23	lomics Pos	0.58793	0.99987	1.0191	0.027309	0.058618	0.99994	0.92068	-0.11922	0.41086	0.99996	0.94217	-0.08594	0.34668	0.80018	0.94541	-0.08098	0.00021	0.001749	0.74392	-0.42679	0.2997	0.58008	0.89407	-0.16154	0.58895	0.86163	1.0263	0.03746	0.25265	0.94365	0.93278	-0.10039	0.044982	0.14081	1.1689	0.2252
694.833	10.05	lomics Pos	0.58794	0.99987	1.0404	0.057182	0.74198	0.99994	0.96674	-0.0488	0.35298	0.99996	0.91681	-0.1253	0.7981	0.97756	0.97318	-0.03923	0.020749	0.053855	0.783	-0.35291	0.57376	0.76736	0.93826	-0.09194	0.73456	0.92898	1.03	0.042714	0.41752	0.96776	0.92569	-0.1114	0.050846	0.14755	1.2126	0.27809
966.7847	10.03	lomics Pos	0.5881	0.99987	1.0428	0.060455	0.26575	0.99994	0.89753	-0.15597	0.85175	0.99996	0.97943	-0.02999	0.98148	0.99652	0.9965	-0.00506	0.019812	0.05192	0.77871	-0.36085	0.93379	0.96623	0.99303	-0.0101	0.7779	0.94624	1.0245	0.034908	0.46447	0.96776	1.0767	0.10657	0.060395	0.15775	1.2257	0.29361
251.2361	1.72	lomics Pos	0.58892	0.99987	1.0922	0.12729	0.14137	0.99994	0.80438	-0.31405	0.5684	0.99996	0.94064	-0.08828	0.059	0.41916	0.6879	-0.53973	0.000259	0.001997	0.30502	-1.713	0.000671	0.03855	0.47898	-1.062	0.079544	0.4964	0.75262	-0.41	0.19334	0.92582	0.85712	-0.22243	0.02366	0.11051	1.3575	0.4409
941.5682	6.47	olomics Pc	0.58957	0.99951	1.19	0.25095	0.23089	0.99998	0.79639	-0.32845	0.86807	0.99941	1.0259	0.036902	0.030472	0.24688	0.62606	-0.67563	0.11224	0.31539	0.70214	-0.51016	0.023184	0.1127	0.66167	-0.59581	0.58301	0.99391	0.90926	-0.13724	0.69501	0.99956	0.99159	-0.01219	0.69046	0.85933	0.94081	-0.08802
388.7294	0.33	olomics Pc	0.58976	0.99951	1.0603	0.084487	0.74993	0.99998	1.0324	0.04604	0.7407	0.99941	0.97314	-0.03927	0.77266	0.99905	1.0227	0.032427	0.046509	0.15907	1.2365	0.30628	0.31774	0.62331	1.157	0.21045	0.25033	0.85803	1.1073	0.147	0.1774	0.96992	1.085	0.11775	0.77769	0.90218	0.97899	-0.03063
1125.463	5.91	lomics Pos	0.59002	0.99987	0.95798	-0.06193	0.93347	0.99994	1.0115	0.016551	0.12165	0.99996	0.89564	-0.15902	0.31918	0.78367	0.91938	-0.12127	0.008165	0.026338	0.77302	-0.37142	0.096532	0.32835	0.7753	-0.36717	0.05133	0.41162	0.80346	-0.3157	0.14825	0.91175	0.84652	-0.24038	0.69513	0.77298	1.0412	0.058279
598.8291	10.09	lomics Pos	0.5907	0.99987	0.95464	-0.06697	0.19569	0.99994	0.90665	-0.14139	0.59599	0.99996	1.0517	0.072765	0.12776	0.55024	0.9176	-0.12407	0.021148	0.054633	0.8175	-0.29071	0.23631	0.52493	0.86801	-0.20421	0.6719	0.89901	1.0838	0.11613	0.20667	0.92582	0.9246	-0.11309	0.22762	0.33471	1.1429	0.19273
947.5763	5.73	lomics Pos	0.59095	0.99987	1.0516	0.072586	0.49851	0.99994	0.91827	-0.12301	0.84616	0.99996	1.02	0.028609	0.98921	0.99652	0.99859	-0.00203	0.027451	0.066332	0.75136	-0.41243	0.4939	0.71227	0.91813	-0.12323	0.93572	0.98642	0.99797	-0.00293	0.74788	0.97988	1.0244	0.034711	0.021728	0.1083	1.5835	0.66313
1010.784	10.09	lomics Pos	0.59135	0.99987	1.0454	0.064038	0.92267	0.99994	0.99533	-0.00675	0.33782	0.99996	0.92932	-0.10576	0.98913	0.99652	1.0047	0.006761	0.034078	0.078182	0.84067	-0.25039	0.31368	0.593	0.90091	-0.15055	0.67959	0.90338	1.0199	0.028436	0.94678	0.98838	0.99558	-0.00639	0.29313	0.40351	1.1599	0.21397
739.5109	8.73	olomics Ne	0.59161	0.99918	1.0422	0.059638	0.58677	0.88869	0.92125	-0.11833	0.32221	0.99995	0.88349	-0.17872	0.69797	0.99985	0.94651	-0.07931	0.13338	0.99927	1.2597	0.33309	0.033827	0.47167	1.5329	0.61627	0.008197	0.2539	1.5748	0.65516	0.002758	0.99512	1.7723	0.82561	0.013971	0.99941	1.5593	0.64088
461.2783	1.54	lomics Pos	0.59195	0.99987	0.91216	-0.13264	0.50627	0.99994	0.89383	-0.16192	0.4481	0.99996	0.88449	-0.17708	0.60538	0.93327	0.91402	-0.1297	0.001204	0.006279	0.35342	-1.5005	0.2674	0.55532	0.79861	-0.32444	0.51174	0.83388	1.1005	0.13813	0.66186	0.97988	1.0891	0.12314	0.076865	0.17826	1.3952	0.48051
604.7769	2.62	olomics Pc	0.5923	0.99951	1.2771	0.35286	0.50587	0.99998	0.84639	-0.24061	0.17121	0.99941	0.70898	-0.49619	0.079438	0.36907	0.47107	-1.086	0.000602	0.00636	0.15172	-2.7205	0.044826	0.17262	0.43006	-1.2174	0.052699	0.45951	0.52752	-0.9227	0.086605	0.79532	0.58397	-0.77604	0.57438	0.79495	1.2786	0.35452
795.5716	4.79	lomics Pos	0.59251	0.99987	1.0569	0.079884	0.98017	0.99994	1.005	0.007155	0.78756	0.99996	0.96736	-0.04787	0.43569	0.8657	0.8962	-0.1581	0.021597	0.055413	0.69719	-0.52038	0.031865	0.16106	0.74227	-0.42998	0.32725	0.73618	0.87791	-0.18785	0.028933	0.59956	0.71883	-0.47627	0.22845	0.33555	0.84208	-0.24797
911.7087	7.33	lomics Pos	0.59301	0.99987	1.0833	0.11547	0.11086	0.99994	1.1826	0.242	0.93531	0.99996	1.0058	0.008293	0.75358	0.97526	0.96439	-0.05231	0.023881	0.059548	0.70716	-0.4999	0.0243	0.13727	0.565	-0.82369	0.2236	0.66617	0.7423	-0.42992	0.023738	0.54857	0.48284	-1.0504	0.082173	0.18348	0.62292	-0.68287
671.1911	1.11	lomics Pos	0.59303	0.99987	1.1124	0.15362	0.5311	0.99994	1.1151	0.15722	0.39485	0.99996	1.2528	0.32513	0.84293	0.98707	1.0643	0.089897	0.50923	0.58204	0.90073	-0.15084	0.041139	0.18899	1.4057	0.49134	0.01856	0.33809	1.6817	0.74991	0.12961	0.88537	1.2731	0.34838	0.12079	0.22499	1.5415	0.62434
149.1336	6.98	olomics Pc	0.59316	0.99951	1.0822	0.11402	0.49251	0.99998	0.90417	-0.14534	0.1039	0.99941	0.79688	-0.32757	0.016821	0.20861	0.56726	-0.81792	0.000566	0.006154	0.3969	-1.3331	0.002326	0.045971	0.40045	-1.3203	0.00087	0.16989	0.42292	-1.2415	0.022044	0.51556	0.53316	-0.90736	0.062213	0.30404	0.7073	-0.49961
137.1336	6.99	olomics Pc	0.59337	0.99951	1.0729	0.10152	0.92568	0.99998	0.9878	-0.01772	0.30615	0.99941	0.83795	-0.25506	0.042129	0.2802	0.60874	-0.71609	0.005308	0.030601	0.44485	-1.1686	0.001921	0.045425	0.48314	-1.0495	0.013988	0.28064	0.57716	-0.79296	0.056427	0.7036	0.62749	-0.67234	0.49431	0.74788	0.87744	-0.18863
874.5536	5.09	lomics Pos	0.59518	0.99987	1.0398	0.056296	0.4663	0.99994	0.91484	-0.1284	0.91693	0.99996	0.99252	-0.01083	0.7202	0.96729	0.96363	-0.05345	0.029601	0.070149	0.76486	-0.38673	0.65817	0.8212	0.95134	-0.07197	0.86208	0.97404	1.0192	0.027381	0.73805	0.97988	1.0189	0.027046	0.015685	0.099085	1.3942	0.47946
899.6669	6.06	lomics Pos	0.59528	0.99987	1.0399	0.05651	0.98326	0.99994	1.0033	0.004761	0.9915	0.99996	1.0032	0.004601	0.4	0.84499	0.90398	-0.14563	0.037572	0.08447	0.76789	-0.38103	0.39146	0.64213	0.89905	-0.15353	0.94107	0.98682	1.002	0.002924	0.62403	0.97813	0.95608	-0.0648	0.24974	0.35853	1.0996	0.13692
602.5231	5.13	lomics Pos	0.59538	0.99987	1.1668	0.22261	0.80353	0.99994	0.92573	-0.11134	0.88611	0.99996	1.0218	0.031179	0.94147	0.99181	1.0282	0.040085	0.2329	0.32757	1.4861	0.5715	0.018069	0.11549	2.1637	1.1135	0.046431	0.39255	2.161	1.1117	0.31564	0.9524	1.6435	0.71675	0.023551	0.11051	1.8536	0.89035
781.5546	4.95	lomics Pos	0.59542	0.99987	1.027	0.038448	0.39219	0.99994	0.94342	-0.08403	0.50508	0.99996	0.964	-0.0529	0.44437	0.86758	0.95278	-0.06978	0.000978	0.005467	0.76728	-0.38217	0.32456	0.60075	0.90816	-0.13899	0.93757	0.98682	1.0093	0.013354	0.43643	0.96776	1.0219	0.031218	0.000798	0.07528	1.2728	0.34806
553.8283	2.98	olomics Pc	0.59542	0.99951	1.2255	0.29341	0.034253	0.99998	0.78124	-0.35616	0.43036	0.99941	0.91955	-0.121	0.027924	0.24036	0.63738	-0.64978	0.000201	0.002803	0.43946	-1.1862	0.008269	0.075499	0.65353	-0.61368	0.63496	0.99391	0.95011	-0.07383	0.62768	0.99956	0.95203	-0.07092	0.12152	0.37128	1.5976	0.67586
941.8068	0.51	lomics Pos	0.59597	0.99987	0.97715	-0.03335	0.023781	0.99994	0.87898	-0.1861	0.14707	0.99996	0.89813	-0.15501	0.011414	0.21625	0.87803	-0.18766	0.001842	0.008503	0.72459	-0.46477	0.030005	0.15481	0.78822	-0.34334	0.19747	0.64891	0.90933	-0.13712	0.033048	0.6327	0.8039	-0.31492	0.30139	0.41089	1.0555	0.077923
298.9793	12.01	olomics Ne	0.59599	0.99918	1.063	0.088113	0.48407	0.85005	1.0794	0.11027	0.017513	0.99995	1.3259	0.40701	0.46118	0.99985	1.148	0.19906	0.12883	0.99927	1.13	0.17635	0.15569	0.62813	1.275	0.35049	0.12142	0.50496	1.1502	0.20184	0.85994	0.99512	0.99207	-0.01149	0.20543	0.99941	1.0693	0.096726
544.8911	10.12	lomics Pos	0.59635	0.99987	0.96494	-0.05149	0.27386	0.99994	0.89564	-0.15901	0.60077	0.99996	0.96229	-0.05546	0.16739	0.60157	0.88856	-0.17046	0.001146	0.006059	0.75227	-0.41068	0.13702	0.40501	0.84471	-0.24348	0.34885	0.7477	1.062	0.086808	0.50419	0.96776	0.95133	-0.07199	0.078094	0.17992	1.1766	0.23467
903.8246	10.05	lomics Pos	0.59662	0.99987	1.0953	0.13136	0.092118	0.99994	0.87757	-0.18841	0.052815	0.99996	0.81712	-0.29139	0.076034	0.4637	0.79043	-0.33929	0.004031	0.015401	0.69737	-0.51999	0.03133	0.15971	0.79735	-0.32672	0.69448	0.91061	0.96605	-0.04983	0.928	0.98573	1.0072	0.010325	0.42843	0.53491	1.0856	0.11845
747.5135	5.22	lomics Pos	0.59691	0.99987	1.057	0.079946	0.32546	0.99994	0.88184	-0.18142	0.33453	0.99996	0.88142	-0.18209	0.38946	0.83582	0.90343	-0.14652	0.039875	0.08806	0.72675	-0.46048	0.77095	0.88192	1.0481	0.067718	0.037869	0.37121	1.3372	0.41916	0.4351	0.96776	1.1025	0.14083	0.083598	0.18526	1.2601	0.33352
253.241	9.92	lomics Pos	0.59719	0.99987	1.0428	0.060477	0.20234	0.99994	0.87693	-0.18947	0.15516	0.99996	0.91809	-0.12329	0.16459	0.596	0.89386	-0.16188	0.032857	0.075975	0.77746	-0.36315	0.29763	0.57957	0.89392	-0.16177	0.16143	0.63418	0.92491	-0.11262	0.37868	0.96776	1.0703	0.098081	0.056613	0.15364	1.2009	0.2641
494.9747	5.05	olomics Ne	0.59728	0.99918	1.0215	0.03063	0.33599	0.75201	0.94555	-0.08078	0.46451	0.99995	1.0353	0.050094	0.28538	0.99985	1.0674	0.09409	0.53104	0.99927	1.0289	0.041046	0.25732	0.69038	1.0983	0.13525	0.040616	0.36763	1.1252	0.17018	0.49479	0.99512	1.0457	0.064521	0.28051	0.99941	1.0466	0.065712
117.9604	10.19	lomics Pos	0.59784	0.99987	1.037	0.052445	0.34977	0.99994	1.0998	0.13724	0.5033	0.99996	0.931	-0.10314	0.70326	0.96147	1.0492	0.06929	0.037891	0.084964	0.78178	-0.35517	0.93518	0.96635	0.98937	-0.01541	0.19223	0.64891	1.1857	0.24568	0.43907	0.96776	1.0652	0.091148	0.19789	0.30623	1.2368	0.30662
71.0857	2.05	lomics Pos	0.59795	0.99987	0.92697	-0.1094	0.54339	0.99994	0.90528	-0.14356	0.66793	0.99996	0.93798	-0.09237	0.10414	0.52894	0.73387	-0.44641	3.18E-05	0.000484	0.26606	-1.9102	0.010148	0.08787	0.66543	-0.58765	0.40664	0.77758	0.85296	-0.22946	0.33502	0.96443	0.8591	-0.21909	0.054449	0.15191	1.2864	0.36338
672.7805	0.56	lomics Pos	0.59847	0.99987	1.0499	0.070193	0.93829	0.99994	0.99592	-0.00591	0.97808	0.99996	0.98597	-0.02038	0.47845	0.88482	0.90927	-0.13723	0.044866	0.096002	0.76049	-0.39499	0.37515	0.63238	0.88527	-0.17581	0.36219	0.75814	0.88717	-0.17272	0.77254	0.97988	0.95821	-0.06158	0.26924	0.37897	1.1719	0.22883
708.813	10.09	lomics Pos	0.59881	0.99987	0.94799	-0.07705	0.27809	0.99994	0.88824	-0.17098	0.21887	0.99996	0.88444	-0.17716	0.29561	0.75244	0.895	-0.16004	0.017926	0.047865	0.76063	-0.39472	0.98359	0.9893	0.99592	-0.00589	0.9026	0.97745	0.98618	-0.02008	0.10191	0.8541	0.86225	-0.21383	0.17884	0.28604	1.1354	0.18319
738.5057	5.07	lomics Pos	0.59939	0.99987	0.84821	-0.23751	0.2934	0.99994	0.71511	-0.48377	0.41349	0.99996	0.77096	-0.37527	0.39834	0.84289	0.76373	-0.38887	0.64568	0.69903	0.86552	-0.20836	0.23281	0.5203	1.3689	0.45303	0.04477	0.38974	1.9255	0.94521	0.11872	0.88537	1.7213	0.78349	0.02172	0.1083	2.235	1.1603
360.3196	3.49	lomics Pos	0.5996	0.99987	1.1443	0.19444	0.82056	0.99994	1.0764	0.10623	0.066832	0.99996	0.74945	-0.41609	0.049539	0.37885	0.69468	-0.52557	0.28263	0.37694	0.81246	-0.29963	0.025124	0.13997	0.65717	-0.60567	0.3637	0.75906	0.84201	-0.24809	0.011846	0.52955	0.67586	-0.5652	0.028451	0.11732	1.4783	0.56391
248.959	0.28	olomics Ne	0.59967	0.99918	0.96336	-0.05385	0.009054	0.34605	0.82699	-0.27406	0.14704	0.99995	0.92859	-0.10688	0.96609	0.99985	1.001	0.001416	0.45192	0.99927	0.95902	-0.06036	0.87393	0.9778	1.0241	0.034374	0.68456	0.89505	1.0435	0.061457	0.52687	0.99512	0.95641	-0.0643	0.98855	0.99945	0.99889	-0.0016
211.9096	12.72	olomics Ne	0.59967	0.99918	1.068	0.094912	0.1369	0.56119	0.90578	-0.14278	0.69079	0.99995	1.0451	0.063581	0.47479	0.99985	1.0783	0.1087	0.61083	0.99927	1.0716	0.099794	0.046701	0.48405	1.2521	0.3244	0.45743	0.78794	1.0538	0.075633	0.8154	0.99512	1.0293	0.041677	0.14574	0.99941	1.0861	0.11921
999.5144	5.92	lomics Pos	0.59971	0.99987	1.0569	0.079835	0.9995	0.99994	1.0077	0.011131	0.91827	0.99996	0.99008	-0.01438	0.84084	0.98707	0.97823	-0.03175	0.01478	0.041494	0.76974	-0.37755	0.16381	0.44908	0.81415	-0.29663	0.25228	0.69047	0.87531	-0.19213	0.39707	0.96776	0.8983	-0.15473	0.087651	0.18829	1.1874	0.24784
459.2513	5.87	olomics Pc	0.60003	0.99951	1.1359	0.18389	0.65064	0.99998	0.92852	-0.107	0.11027	0.99941	0.72572	-0.46252	0.24793	0.71367	0.77948	-0.35941	1.73E-05	0.000446	0.16366	-2.6112	0.15189	0.39804	0.64538	-0.63177	0.010403	0.24769	0.51739	-0.95068	0.95527	0.99956	1.0177	0.025316	0.096471	0.34969	1.3349	0.4167
462.9788	4.92	olomics Ne	0.60043	0.99918	1.0296	0.042134	0.015948	0.37315	0.90406	-0.1455	0.28423	0.99995	1.0581	0.081424	0.99985	0.99985	1.0048	0.00684	0.36476	0.99927	1.0338	0.048001	0.03355	0.47167	1.1202	0.16371	0.085583	0.43897	1.0562	0.078888	0.64062	0.99512	1.0287	0.040822	0.7212	0.99941	1.0131	0.018748
762.5904	5.27	lomics Pos	0.60108	0.99987	1.0639	0.089398	0.69809	0.99994	0.95722	-0.06309	0.70449	0.99996	0.95811	-0.06174	0.39458	0.83821	0.89637	-0.15784	0.030466	0.071663	0.73715	-0.43996	0.24976	0.53672	0.86893	-0.20269	0.78851	0.95091	0.97233	-0.04048	0.53036	0.96776	1.0861	0.11912	0.052214	0.14869	1.7496	0.80698
924.5705	8.95	olomics Pc	0.60154	0.99951	0.94795	-0.07712	0.63263	0.99998	0.94922	-0.07519	0.97546	0.99941	0.99893	-0.00154	0.51046	0.98198	1.0729	0.10149	0.2935	0.6585	1.1133	0.15479	0.26887	0.56014	1.1693	0.22559	0.04063	0.4012	1.2273	0.29553	0.90745	0.99956	1.0185	0.026399	0.28999	0.56597	1.116	0.15836
725.5325	5.62	lomics Pos	0.60199	0.99987	0.95489	-0.06659	0.025119	0.99994	0.82357	-0.28004	0.38409	0.99996	0.94901	-0.0755	0.15939	0.58948	0.89047	-0.16736	0.055756	0.1129	0.76657	-0.38351	0.81412	0.90634	0.9865	-0.01962	0.3542	0.75239	1.1201	0.16367	0.31477	0.9524	0.93293	-0.10016	0.7663	0.82539	1.0155	0.022192
109.0939	8.37	olomics Pc	0.6023	0.99951	1.0264	0.03757	0.28897	0.99998	0.94472	-0.08204	0.25111	0.99941	1.031	0.043979	0.96509	0.99905	0.99983	-0.00025	0.60353	0.97373	1.0361	0.051132	0.79306	0.93994	0.98849	-0.01671	0.92567	0.9951	1.0041	0.005952	0.00021	0.47452	0.81173	-0.30092	0.044209	0.28476	0.87519	-0.19233
1151.724	10.08	lomics Pos	0.60299	0.99987	0.96008	-0.05878	0.37298	0.99994	0.91971	-0.12075	0.98946	0.99996	0.99695	-0.0044	0.58011	0.9205	1.0579	0.081154	0.033442	0.077094	0.75974	-0.39643	0.93157	0.96524	0.99764	-0.00341	0.67437	0.89976	0.96215	-0.05567	0.89804	0.98023	1.0205	0.02932	0.072688	0.17431	1.3199	0.40042
761.5879	5.27	lomics Pos	0.60304	0.99987	1.0645	0.090113	0.69704	0.99994	0.95682	-0.06368	0.73067	0.99996	0.96171	-0.05633	0.44746	0.86921	0.90782	-0.13953	0.032844	0.075975	0.73909	-0.43617	0.26904	0.55659	0.87402	-0.19425	0.8249	0.96188	0.9783	-0.03166	0.51428	0.96776	1.0907	0.12525	0.049666	0.14647	1.755	0.81146
336.1725	2.28	lomics Pos	0.60309	0.99987	0.94092	-0.08785	0.233	0.99994	0.83259	-0.26431	0.002532	0.99996	0.62676	-0.674	0.020117	0.25894	0.62731	-0.67274	0.000173	0.001528	0.4991	-1.0026	0.008368	0.084185	0.44961	-1.1533	0.016586	0.33809	0.46314	-1.1105	0.014424	0.52955	0.48339	-1.0487	0.073613	0.17569	0.64496	-0.63273
889.7244	7.51	lomics Pos	0.60385	0.99987	0.83554	-0.25922	0.47346	0.99994	0.78366	-0.3517	0.64613	0.99996	0.84107	-0.2497	0.30052	0.75886	0.70575	-0.50278	0.055721	0.11289	0.4722	-1.0825	0.071164	0.27381	0.51681	-0.95231	0.13886	0.60403	0.61354	-0.70476	0.018275	0.52955	0.43981	-1.1851	0.84067	0.88242	0.91315	-0.13108
401.3417	5	olomics Pc	0.6043	0.99951	1.0803	0.11147	0.89591	0.99998	0.94851	-0.07626	0.35481	0.99941	0.81566	-0.29396	0.2424	0.70505	0.7733	-0.3709	0.044275	0.15309	0.56387	-0.82658	0.019128	0.10208	0.47325	-1.0793	0.047797	0.44092	0.49103	-1.0261	0.25289	0.99956	0.73653	-0.44119	0.016713	0.2688	0.51558	-0.95574
330.2515	2.04	lomics Pos	0.60444	0.99987	0.91929	-0.12141	0.37058	0.99994	0.83124	-0.26667	0.05337	0.99996	0.68358	-0.54882	0.009131	0.2002	0.51229	-0.96497	0.008342	0.026795	0.50036	-0.99897	0.005499	0.075369	0.31307	-1.6754	0.024146	0.33809	0.4371	-1.194	0.023021	0.54215	0.44501	-1.1681	0.026567	0.11491	0.49276	-1.0211
572.3712	5.52	olomics Pc	0.60466	0.99951	1.1414	0.19083	0.5742	0.99998	1.1515	0.20354	0.18997	0.99941	1.2986	0.37699	0.41642	0.90805	1.192	0.25336	0.17947	0.45971	1.2459	0.31723	0.009351	0.077313	1.9079	0.93202	0.005134	0.20472	2.0232	1.0167	0.15541	0.93927	1.3097	0.38919	0.0508	0.29361	1.5903	0.66926
760.5849	5.27	lomics Pos	0.60627	0.99987	1.0655	0.091494	0.70882	0.99994	0.95591	-0.06505	0.7246	0.99996	0.95826	-0.06151	0.43486	0.86496	0.90059	-0.15105	0.039398	0.087373	0.73531	-0.44357	0.28502	0.57196	0.87214	-0.19737	0.79483	0.95091	0.97141	-0.04185	0.52388	0.96776	1.0917	0.12651	0.051384	0.14811	1.7791	0.83112
512.9752	12	olomics Ne	0.60692	0.99918	1.0436	0.061628	0.037639	0.45581	0.83388	-0.26209	0.6613	0.99995	1.0688	0.096055	0.97605	0.99985	0.99987	-0.00018	0.53158	0.99927	1.0553	0.077638	0.52267	0.87267	1.0576	0.080786	0.060078	0.40009	1.1627	0.21744	0.61022	0.99512	0.95457	-0.06707	0.85107	0.99941	0.99191	-0.01171
210.0498	0.35	olomics Pc	0.60787	0.99951	1.0581	0.081519	0.64561	0.99998	1.0557	0.078194	0.23193	0.99941	1.1949	0.25683	0.029782	0.24494	1.5471	0.62961	0.1678	0.43685	1.3009	0.37953	0.12884	0.35759	1.2998	0.37827	0.051291	0.45418	1.3741	0.45854	0.84018	0.99956	0.97231	-0.04051	0.81536	0.92407	0.94366	-0.08366
818.6023	5.61	lomics Pos	0.60788	0.99987	1.0548	0.076923	0.69439	0.99994	0.95526	-0.06604	0.93506	0.99996	0.98956	-0.01514	0.63872	0.95076	0.94754	-0.07774	0.044368	0.095364	0.77124	-0.37474	0.34406	0.61218	0.88763	-0.17197	0.88149	0.97532	0.98219	-0.02592	0.73539	0.97988	0.96145	-0.05672	0.59109	0.68423	1.044	0.062066
718.8366	0.5	lomics Pos	0.60794	0.99987	1.1381	0.18661	0.538	0.99994	0.94194	-0.08629	0.65513	0.99996	0.95838	-0.06132	0.94947	0.99328	1.0042	0.006103	0.002153	0.009558	0.74138	-0.43171	0.89894	0.94844	0.99967	-0.00048	0.10853	0.5577	1.1128	0.15424	0.88667	0.98023	1.0184	0.026348	0.023847	0.11051	1.2148	0.2807
360.2216	4.96	lomics Pos	0.6083	0.99987	1.0829	0.11492	0.41387	0.99994	0.92474	-0.11287	0.21088	0.99996	0.8911	-0.16633	0.096358	0.51634	0.85876	-0.21968	0.002126	0.009471	0.67989	-0.55663	0.63698	0.80987	0.94473	-0.08203	0.23043	0.67417	1.1309	0.17748	0.25996	0.94365	0.89136	-0.16593	0.17694	0.28431	1.1884	0.24903
747.5642	8.39	olomics Ne	0.60838	0.99918	1.0255	0.036348	0.02199	0.40327	0.88345	-0.17878	0.90878	0.99995	1.0102	0.01457	0.75397	0.99985	1.0133	0.019088	0.7794	0.99927	1.0139	0.019868	0.45409	0.82254	1.05	0.07036	0.8066	0.95074	1.0144	0.020691	0.6693	0.99512	0.97318	-0.03922	0.046809	0.99941	0.85403	-0.22764
605.312	1.84	lomics Pos	0.60895	0.99987	1.0655	0.09152	0.82203	0.99994	0.97663	-0.03412	0.73194	0.99996	0.95191	-0.0711	0.76972	0.97526	0.95779	-0.06222	0.035368	0.080281	0.67866	-0.55923	0.58735	0.77531	0.93141	-0.10252	0.44535	0.80121	1.0954	0.13148	0.67854	0.97988	1.0594	0.083227	0.062778	0.16076	1.2976	0.37585
611.5333	5.66	lomics Pos	0.60905	0.99987	1.0688	0.096034	0.67963	0.99994	0.948	-0.07704	0.87967	0.99996	0.98251	-0.02546	0.77822	0.97526	1.0293	0.041626	0.070186	0.13462	0.79814	-0.32528	0.033545	0.16483	1.4116	0.49729	0.016513	0.33809	1.481	0.56653	0.023318	0.54215	1.389	0.474	0.028737	0.11732	1.472	0.55781
238.2166	5.67	olomics Pc	0.60905	0.99951	1.0487	0.068605	0.58676	0.99998	0.92399	-0.11405	0.932	0.99941	1.0343	0.048642	0.33647	0.82883	0.86532	-0.20869	0.00093	0.008881	0.33329	-1.5851	0.033379	0.1422	0.60285	-0.73012	0.029768	0.34883	0.70343	-0.50753	0.033504	0.59292	0.7595	-0.39687	0.45635	0.71948	1.0776	0.1078
272.6676	1.38	lomics Pos	0.60933	0.99987	1.1726	0.22972	0.83247	0.99994	1.1091	0.14944	0.8941	0.99996	0.99657	-0.00495	0.75802	0.97526	0.91566	-0.12712	0.83571	0.861	0.93018	-0.10442	0.30896	0.58859	1.2183	0.28487	0.032294	0.36287	1.7449	0.80315	0.47356	0.96776	1.1181	0.16108	0.031191	0.12145	2.2866	1.1932
171.0684	10.16	lomics Pos	0.60954	0.99987	1.0345	0.048913	0.3551	0.99994	0.91248	-0.13214	0.38207	0.99996	0.92346	-0.11487	0.31202	0.77382	0.90904	-0.13758	0.004303	0.01621	0.72113	-0.47167	0.25858	0.54507	0.88596	-0.17469	0.67606	0.90042	0.95939	-0.05982	0.45712	0.96776	0.9304	-0.10408	0.20342	0.31196	1.1275	0.17307
932.531	5.92	lomics Pos	0.60997	0.99987	1.0567	0.07954	0.88007	0.99994	0.9872	-0.01859	0.87463	0.99996	1.0146	0.020937	0.73213	0.96985	0.96466	-0.05191	0.015077	0.041901	0.7677	-0.38139	0.1565	0.43716	0.82896	-0.27062	0.17464	0.64606	0.86088	-0.21611	0.51862	0.96776	0.927	-0.10936	0.077974	0.17976	1.1837	0.24328
361.27	2.72	lomics Pos	0.61026	0.99987	0.96644	-0.04925	0.16939	0.99994	0.8844	-0.17724	0.063013	0.99996	0.84188	-0.24832	0.003793	0.17581	0.63738	-0.64976	0.000226	0.001838	0.51096	-0.96873	0.027661	0.1469	0.52241	-0.93675	0.017988	0.33809	0.59392	-0.75165	0.016792	0.52955	0.58131	-0.78261	0.043658	0.13906	0.65917	-0.60128
1173.841	4.98	lomics Pos	0.61031	0.99987	0.93933	-0.0903	0.084695	0.99994	0.80084	-0.32041	0.080496	0.99996	0.79313	-0.33437	0.222	0.67657	0.83541	-0.25944	0.035372	0.080281	0.71627	-0.48142	0.75985	0.87501	0.96685	-0.04864	0.45452	0.80208	1.1131	0.15462	0.54152	0.96776	0.93447	-0.09778	0.32522	0.43557	1.1144	0.15633
696.9729	12.01	olomics Ne	0.61037	0.99918	1.0427	0.060275	0.80538	0.97773	0.97985	-0.02936	0.14347	0.99995	1.1516	0.2036	0.99305	0.99985	0.99603	-0.00573	0.25161	0.99927	1.1697	0.22616	0.018176	0.40708	1.3231	0.40391	0.35074	0.71526	1.1027	0.14105	0.17166	0.99512	1.1288	0.17479	0.75681	0.99941	1.0453	0.063979
585.2689	3.49	olomics Pc	0.61052	0.99951	0.8879	-0.17153	0.74431	0.99998	1.1764	0.23435	0.97063	0.99941	0.99297	-0.01018	0.20529	0.64242	0.65064	-0.62008	0.016329	0.070946	0.4558	-1.1335	0.25752	0.54765	0.69199	-0.53118	0.45056	0.99391	0.78039	-0.35773	0.33669	0.99956	0.73169	-0.4507	0.3695	0.64663	0.75766	-0.40038
671.9767	12.02	olomics Ne	0.61101	0.99918	0.94994	-0.07409	0.02682	0.40327	0.84226	-0.24767	0.79696	0.99995	0.98105	-0.0276	0.22066	0.99985	0.84654	-0.24035	0.27165	0.99927	1.0959	0.1321	0.38503	0.78301	0.9417	-0.08666	0.48498	0.80618	0.92212	-0.11697	0.77758	0.99512	0.98161	-0.02677	0.67513	0.99941	1.1463	0.19702
942.5762	10.47	olomics Ne	0.61102	0.99918	1.0403	0.057052	0.049411	0.47152	0.85137	-0.23215	0.39254	0.99995	1.0625	0.08745	0.18772	0.99985	1.1138	0.1555	0.40186	0.99927	1.064	0.089474	0.01155	0.40245	1.2499	0.32181	0.15684	0.53641	1.1538	0.20635	0.4223	0.99512	1.0708	0.098665	0.47814	0.99941	0.94984	-0.07425
264.8654	0.57	lomics Pos	0.6111	0.99987	0.95056	-0.07315	0.96218	0.99994	1.0165	0.023604	0.086297	0.99996	0.81521	-0.29476	0.033858	0.32166	0.74344	-0.42771	0.021291	0.054927	0.72721	-0.45955	0.003764	0.068018	0.70446	-0.5054	0.008556	0.32129	0.70084	-0.51284	0.13497	0.89196	0.8617	-0.21473	0.58876	0.68194	0.94838	-0.07647
489.3138	2.2	lomics Pos	0.61111	0.99987	1.1586	0.21233	0.93867	0.99994	1.0131	0.018745	0.88457	0.99996	0.98283	-0.02499	0.80407	0.97859	1.0134	0.019192	0.010618	0.032157	0.412	-1.2793	0.35816	0.62208	0.85068	-0.2333	0.12608	0.58627	1.4439	0.53	0.79429	0.97988	1.0298	0.042331	0.030549	0.11984	1.7084	0.77262
306.8956	10.15	lomics Pos	0.61231	0.99987	1.0237	0.03378	0.64362	0.99994	0.97203	-0.04093	0.52695	0.99996	0.96601	-0.0499	0.41869	0.85533	0.95358	-0.06858	0.001988	0.009026	0.79352	-0.33365	0.5659	0.76382	0.94777	-0.07739	0.80748	0.95502	0.98589	-0.02051	0.72453	0.97988	0.97923	-0.03027	0.005768	0.08891	1.2264	0.29441
166.0874	0.6	olomics Pc	0.61244	0.99951	1.067	0.093544	0.83821	0.99998	1.0261	0.037224	0.25789	0.99941	1.1636	0.21863	0.019772	0.21701	1.6944	0.76081	0.09065	0.26587	1.4261	0.51205	0.061416	0.21377	1.3475	0.43028	0.009352	0.24769	1.621	0.69688	0.59958	0.99956	1.0959	0.13208	0.98721	0.9934	0.9867	-0.01932
898.5457	6.48	olomics Pc	0.6125	0.99951	1.1702	0.2267	0.35826	0.99998	0.83883	-0.25355	0.62797	0.99941	1.0909	0.12558	0.097089	0.41658	0.74247	-0.4296	0.2097	0.51206	0.74161	-0.43126	0.027342	0.12457	0.66549	-0.5875	0.74121	0.99391	0.95072	-0.07291	0.76693	0.99956	1.0075	0.010806	0.99453	0.9958	1.0062	0.00891
398.3227	4.75	olomics Pc	0.61268	0.99951	0.79478	-0.33137	0.4386	0.99998	0.73671	-0.44084	0.2864	0.99941	0.67536	-0.56628	0.094903	0.41348	0.54537	-0.8747	0.036697	0.13345	0.3689	-1.4387	0.019182	0.10208	0.33985	-1.557	0.037551	0.38531	0.36773	-1.4433	0.073036	0.76911	0.44461	-1.1694	0.014022	0.2688	0.33754	-1.5669
804.4312	0.33	olomics Ne	0.61301	0.99918	0.96581	-0.05019	0.33011	0.74862	0.91462	-0.12876	0.40817	0.99995	1.1332	0.18039	0.91609	0.99985	1.0091	0.01303	0.097913	0.99927	1.115	0.15705	0.049686	0.48405	1.1569	0.21023	0.025976	0.33786	1.1818	0.24102	0.39276	0.99512	0.91395	-0.12981	0.94896	0.99941	1.0024	0.003407
465.2195	3.52	lomics Pos	0.61312	0.99987	1.0617	0.086326	0.45614	0.99994	0.96281	-0.05468	0.60599	0.99996	0.95648	-0.0642	0.024273	0.27645	0.79771	-0.32606	0.000417	0.002846	0.615	-0.70133	0.021354	0.12682	0.66025	-0.59892	0.14091	0.60462	0.80339	-0.31583	0.002181	0.48691	0.6935	-0.52802	0.6334	0.72483	0.96038	-0.05832
225.2095	9.91	lomics Pos	0.61325	0.99987	1.0462	0.06519	0.23493	0.99994	0.89146	-0.16576	0.18026	0.99996	0.90877	-0.13802	0.20721	0.65238	0.91642	-0.12592	0.015429	0.042662	0.79087	-0.33848	0.53418	0.73985	0.9418	-0.08652	0.94684	0.98858	0.99355	-0.00933	0.35795	0.96776	1.0808	0.11211	0.14935	0.25457	1.1148	0.15678
519.3279	4.84	olomics Pc	0.61344	0.99951	0.87074	-0.19969	0.88209	0.99998	1.0108	0.015435	0.92166	0.99941	0.94994	-0.07409	0.70735	0.99905	1.1273	0.17281	0.44571	0.85349	1.2732	0.34844	0.071741	0.23888	2.1766	1.1221	0.023699	0.32255	2.7716	1.4707	0.40296	0.99956	1.587	0.6663	0.053686	0.2951	2.2191	1.1499
303.2212	2.28	lomics Pos	0.61362	0.99987	1.0887	0.1226	0.80067	0.99994	0.97001	-0.04392	0.080753	0.99996	0.67863	-0.55931	0.005145	0.17581	0.54588	-0.87334	0.001391	0.006959	0.4529	-1.1427	0.009726	0.087714	0.45033	-1.1509	0.10243	0.54475	0.5793	-0.78763	0.020658	0.52955	0.49438	-1.0163	0.064685	0.16302	0.55432	-0.8512
770.8511	10.02	lomics Pos	0.61439	0.99987	1.0182	0.026042	0.32072	0.99994	0.95359	-0.06855	0.34177	0.99996	0.95668	-0.06389	0.17656	0.60979	0.92951	-0.10545	0.000194	0.00166	0.76009	-0.39576	0.25713	0.54373	0.90512	-0.14383	0.9206	0.98119	1.0063	0.009046	0.52549	0.96776	0.96814	-0.04672	0.011685	0.096289	1.1962	0.25842
214.9181	10.25	lomics Pos	0.61511	0.99987	0.97577	-0.03539	0.058015	0.99994	0.89891	-0.15375	0.074775	0.99996	0.89051	-0.16729	0.30042	0.75886	0.94635	-0.07956	0.000877	0.005044	0.74397	-0.42669	0.18381	0.47109	0.89484	-0.16029	0.67996	0.90338	0.97882	-0.03088	0.47112	0.96776	0.98583	-0.02058	0.10712	0.20988	1.1538	0.20635
403.1691	6.8	olomics Ne	0.6156	0.99918	1.0645	0.090145	0.45562	0.82821	0.87721	-0.189	0.32728	0.99995	0.83868	-0.25381	0.00596	0.99985	0.59143	-0.75772	0.000262	0.040239	0.40889	-1.2902	0.001587	0.32555	0.46956	-1.0906	0.010753	0.2716	0.58043	-0.7848	0.04079	0.99512	0.55631	-0.84604	0.11792	0.99941	0.70127	-0.51195
1071.78	10.02	lomics Pos	0.6157	0.99987	1.0219	0.031191	0.14381	0.99994	0.88385	-0.17813	0.3559	0.99996	0.92739	-0.10876	0.51936	0.89933	0.95813	-0.06171	0.003749	0.014557	0.69434	-0.52628	0.26052	0.54699	0.87082	-0.19956	0.57888	0.86163	0.959	-0.0604	0.11427	0.88445	0.90173	-0.14924	0.13202	0.23722	1.1177	0.16047
399.7676	4.66	lomics Pos	0.61617	0.99987	1.0711	0.099131	0.017492	0.99994	0.78812	-0.34351	0.38152	0.99996	0.91748	-0.12425	0.67334	0.95764	0.96501	-0.05138	0.13118	0.21349	0.76556	-0.38541	0.42752	0.66265	0.87527	-0.1922	0.15366	0.62466	0.83738	-0.25604	0.050471	0.68198	0.85634	-0.22374	0.64609	0.73429	1.0503	0.070769
282.2503	2.85	lomics Neg	0.61622	0.99977	0.9077	-0.13971	0.46682	0.99904	0.85943	-0.21855	0.031219	0.99994	0.51618	-0.95405	0.028513	0.58182	0.38425	-1.3799	0.001821	0.066804	0.29871	-1.7432	0.017272	0.46473	0.24725	-2.016	0.000917	0.093659	0.1645	-2.6038	0.015511	0.46971	0.18979	-2.3975	0.018481	0.39073	0.29058	-1.783
817.5893	8.89	olomics Pc	0.61743	0.99951	1.0493	0.069432	0.32263	0.99998	1.132	0.17891	0.44538	0.99941	0.93344	-0.09937	0.25632	0.72806	1.087	0.12041	0.008246	0.04176	1.1569	0.21027	0.33133	0.64194	1.1406	0.18974	0.090194	0.60449	1.1735	0.23077	0.80094	0.99956	1.0056	0.008015	0.38376	0.65753	0.93197	-0.10165
426.2523	7.49	olomics Pc	0.61765	0.99951	0.94654	-0.07927	0.53629	0.99998	0.93641	-0.09479	0.010561	0.99941	0.70762	-0.49895	0.006431	0.1948	0.59825	-0.74117	0.00921	0.0454	0.63656	-0.65164	0.00035	0.039399	0.47627	-1.0702	0.004885	0.20247	0.59665	-0.74504	0.005985	0.47452	0.53168	-0.91137	0.021743	0.2688	0.61325	-0.70545
971.6394	6.05	lomics Pos	0.61777	0.99987	0.94859	-0.07615	0.15357	0.99994	0.80563	-0.31182	0.098772	0.99996	0.80241	-0.31759	0.32407	0.7879	0.89415	-0.16142	0.022556	0.057185	0.72264	-0.46865	0.32555	0.60075	0.83465	-0.26076	0.5551	0.85279	0.93752	-0.09307	0.23317	0.94365	0.8392	-0.25291	0.56554	0.66046	0.94287	-0.08487
982.7965	0.5	lomics Pos	0.61821	0.99987	1.0803	0.11139	0.44818	0.99994	0.9139	-0.12989	0.86729	0.99996	0.98608	-0.02022	0.7616	0.97526	0.97253	-0.04019	0.026292	0.064222	0.77898	-0.36034	0.79547	0.89629	0.98253	-0.02543	0.97716	0.99392	0.99294	-0.01022	0.40104	0.96776	0.92736	-0.10879	0.039403	0.13308	1.2032	0.26689
823.8171	10.06	lomics Pos	0.61861	0.99987	0.96404	-0.05283	0.37453	0.99994	0.90484	-0.14426	0.33315	0.99996	0.89034	-0.16757	0.45275	0.87408	0.94679	-0.07888	0.13126	0.21353	0.80407	-0.31461	0.20154	0.48967	0.8563	-0.22381	0.020968	0.33809	0.82275	-0.28148	0.42316	0.96776	0.92949	-0.10549	0.015382	0.09885	1.2788	0.35482
1195.322	8.47	olomics Pc	0.61929	0.99951	1.1691	0.2254	0.72831	0.99998	1.1413	0.19066	0.41652	0.99941	1.3121	0.39187	0.42501	0.9108	1.4688	0.55461	0.1326	0.36132	1.5969	0.67523	0.019322	0.10212	2.0749	1.053	0.004804	0.20177	2.1508	1.1049	0.099667	0.83276	1.5118	0.59629	0.040993	0.28426	1.5831	0.66274
304.0131	0.57	lomics Pos	0.61939	0.99987	1.047	0.066268	0.98243	0.99994	0.98636	-0.01981	0.87029	0.99996	1.0111	0.015861	0.086316	0.49062	1.3508	0.43377	0.86835	0.88935	0.95821	-0.06158	0.33339	0.6073	1.142	0.1916	0.034498	0.36356	1.5645	0.64569	0.99081	0.99721	1.0014	0.001973	0.91406	0.93699	0.96619	-0.04961
778.7874	10.11	lomics Pos	0.61941	0.99987	1.0328	0.046526	0.49347	0.99994	0.95083	-0.07275	0.99201	0.99996	1.0077	0.011065	0.44113	0.86677	0.9204	-0.11967	0.023857	0.059548	0.77694	-0.36412	0.77705	0.88442	0.98486	-0.02202	0.1176	0.5712	1.2327	0.30179	0.84939	0.97988	0.98649	-0.01963	0.015538	0.09885	1.2948	0.37278

1117.713	9.02	olomics Pc	0.61962	0.99951	1.2817	0.35808	0.30573	0.99998	0.84863	-0.23679	0.95017	0.99941	1.0238	0.033887	0.023461	0.22481	0.71045	-0.4932	0.12268	0.34019	0.73594	-0.44234	0.25878	0.54892	0.87796	-0.18777	0.77381	0.99391	1.1665	0.22213	0.45847	0.99956	1.2191	0.28582	0.068261	0.31036	1.7285	0.78949
616.8152	0.54	omics Pos	0.61985	0.99987	1.034	0.048168	0.13722	0.99994	0.88179	-0.18149	0.6546	0.99996	0.96261	-0.05498	0.087546	0.49308	0.85105	-0.23269	0.006439	0.022019	0.73694	-0.44037	0.097055	0.32983	0.79034	-0.33945	0.057171	0.42773	0.81551	-0.29422	0.18533	0.92582	0.88711	-0.17282	0.13113	0.23629	1.1978	0.26036
504.9734	5.33	olomics Ne	0.61994	0.99918	1.0122	0.017536	0.15365	0.58235	0.9419	-0.08636	0.95116	0.99995	0.99801	-0.00287	0.85146	0.99985	0.99232	-0.01113	0.77553	0.99927	1.0138	0.019832	0.59187	0.89518	1.0301	0.042756	0.037076	0.35842	1.0965	0.13284	0.91832	0.99512	1.0053	0.007574	0.8424	0.99941	1.0077	0.01106
390.7856	0.57	omics Pos	0.61997	0.99987	1.0992	0.13646	0.23233	0.99994	1.1585	0.21231	0.82133	0.99996	1.0142	0.020403	0.070373	0.45326	0.81021	-0.30364	0.12049	0.20045	0.82969	-0.26936	0.58989	0.77668	0.95281	-0.06974	0.3537	0.75218	0.89218	-0.1646	0.018648	0.52955	1.1689	0.22518	0.78701	0.84152	1.0465	0.065631
169.0601	0.6	omics Pos	0.62014	0.99987	1.0726	0.10112	0.72148	0.99994	0.9658	-0.0502	0.49428	0.99996	1.1496	0.20118	0.14998	0.57825	1.3108	0.39048	0.58393	0.6443	1.0939	0.12947	0.21104	0.50324	1.1104	0.15111	0.019415	0.33809	1.4366	0.52264	0.17499	0.91806	1.2468	0.31828	0.07294	0.17459	1.2989	0.37729
895.5496	4.83	lomics Neg	0.62057	0.99977	1.0833	0.1154	0.91235	0.99904	1.0179	0.025546	0.49601	0.99994	0.9469	-0.07871	0.029996	0.58182	1.2267	0.29474	0.76911	0.99989	1.168	0.22403	0.42539	0.99653	1.2583	0.33148	0.29132	0.54557	0.86874	-0.203	0.093834	0.50803	0.71207	-0.48992	0.90309	0.94457	0.99853	-0.00213
172.0652	10.16	omics Pos	0.62118	0.99987	0.96426	-0.05251	0.22606	0.99994	0.88457	-0.17695	0.23999	0.99996	0.91657	-0.12568	0.15316	0.58077	0.8948	-0.16036	0.002419	0.010424	0.73183	-0.45042	0.07235	0.27658	0.84113	-0.2496	0.43878	0.79822	0.93539	-0.09636	0.093705	0.83733	0.88129	-0.18231	0.2697	0.37948	1.1042	0.143
1014.677	9.05	olomics Pc	0.62178	0.99951	1.1776	0.2358	0.13146	0.99998	0.79949	-0.32286	0.80498	0.99941	0.97576	-0.03541	0.022808	0.22443	0.67002	-0.57772	0.005411	0.030991	0.58995	-0.76134	0.080083	0.25584	0.7157	-0.48258	0.99334	0.99906	1.0334	0.047374	0.85092	0.99956	0.99857	-0.00206	0.47941	0.73879	1.2726	0.34772
1082.696	4.41	omics Pos	0.62274	0.99987	1.1011	0.139	0.13443	0.99994	0.81171	-0.30097	0.69289	0.99996	1.0496	0.069795	0.044411	0.36496	0.66429	-0.59012	0.000116	0.001138	0.43405	-1.2041	0.012315	0.093356	0.59682	-0.74464	0.60583	0.86762	0.93461	-0.09756	0.68552	0.97988	0.97722	-0.03325	0.13022	0.23533	1.4167	0.50253
1056.756	10.07	omics Pos	0.62312	0.99987	1.0472	0.066589	0.99914	0.99994	1.0022	0.003182	0.70989	0.99996	1.0514	0.072262	0.69585	0.96092	1.047	0.066274	0.049654	0.10378	0.75092	-0.41326	0.67794	0.8336	0.943	-0.08467	0.86055	0.97339	0.9711	-0.0423	0.60582	0.97294	1.0739	0.10285	0.12839	0.23335	1.2141	0.27994
458.7881	9.91	omics Pos	0.62318	0.99987	0.95516	-0.06619	0.06744	0.99994	0.82226	-0.28233	0.28116	0.99996	0.8629	-0.21273	0.39538	0.83895	0.93173	-0.10201	0.016191	0.044288	0.73428	-0.44559	0.25959	0.54625	0.84763	-0.23849	0.70278	0.91466	1.0206	0.029348	0.10708	0.86469	0.80829	-0.30705	0.20006	0.30846	1.1077	0.14757
936.7974	10.02	omics Pos	0.62338	0.99987	1.034	0.048198	0.29759	0.99994	0.89129	-0.16603	0.42511	0.99996	0.90674	-0.14123	0.52374	0.89938	0.93022	-0.10436	0.031797	0.074108	0.77803	-0.3621	0.42937	0.66336	0.88509	-0.17611	0.47635	0.80869	0.92066	-0.11926	0.53923	0.96776	0.93364	-0.09906	0.043016	0.13814	1.2693	0.34403
599.4337	2.28	omics Pos	0.62346	0.99987	1.0989	0.13605	0.99687	0.99994	1.0208	0.029756	0.10068	0.99996	0.67062	-0.57644	0.11876	0.53907	0.56693	-0.81875	0.016645	0.04511	0.51738	-0.95072	0.027974	0.14773	0.48815	-1.0346	0.02447	0.33809	0.5248	-0.93015	0.012401	0.52955	0.3783	-1.4024	0.13526	0.24038	0.61569	-0.69972
141.0517	12.4	olomics Pc	0.62383	0.99951	0.96075	-0.05777	0.68617	0.99998	0.97539	-0.03596	0.009922	0.99941	0.7739	-0.36979	0.40193	0.89475	0.93299	-0.10006	0.30827	0.67858	0.94508	-0.08149	0.10865	0.31749	0.85606	-0.22422	0.32558	0.93893	0.90339	-0.14658	0.012936	0.47452	0.77652	-0.3649	0.060099	0.30404	0.84287	-0.24662
646.5053	2.28	omics Pos	0.62384	0.99987	1.2015	0.26482	0.85567	0.99994	1.0577	0.080943	0.13805	0.99996	0.71864	-0.47666	0.022371	0.26776	0.49338	-1.0192	0.000124	0.001186	0.3586	-1.4796	0.00937	0.086818	0.31935	-1.6468	0.02935	0.35275	0.39632	-1.3353	0.033968	0.6327	0.40693	-1.2972	0.013671	0.098791	0.37709	-1.407
828.55	8.58	olomics Pc	0.6239	0.99951	0.97966	-0.02965	0.96915	0.99998	1.014	0.02004	0.53762	0.99941	0.97435	-0.03749	0.64976	0.99905	1.0263	0.037402	0.1824	0.46373	1.0344	0.048782	0.53602	0.82123	1.0503	0.070862	0.67732	0.99391	1.0203	0.029051	0.049958	0.67909	0.91347	-0.13057	0.58239	0.80048	0.9772	-0.03328
386.2694	2.28	omics Pos	0.62479	0.99987	1.0557	0.078243	0.24331	0.99994	0.86388	-0.21109	0.049136	0.99996	0.79948	-0.32287	0.064927	0.43615	0.68348	-0.54902	0.001434	0.007112	0.57256	-0.8045	0.004031	0.069859	0.4602	-1.1197	0.0045	0.3088	0.52745	-0.9229	0.000407	0.38756	0.53779	-0.8949	0.059663	0.15722	0.6825	-0.55111
1150.699	0.54	omics Pos	0.62483	0.99987	1.0334	0.047389	0.15985	0.99994	0.87923	-0.18569	0.06298	0.99996	0.81828	-0.28934	0.090882	0.50222	0.88587	-0.17483	0.006958	0.023272	0.6946	-0.52574	0.004449	0.071311	0.74529	-0.42413	0.029404	0.35275	0.79173	-0.33691	0.27295	0.94365	0.90746	-0.14009	0.44599	0.55088	1.1211	0.16489
863.8043	10.05	omics Pos	0.62611	0.99987	0.94142	-0.08709	0.72879	0.99994	0.95625	-0.06454	0.62939	0.99996	0.94296	-0.08473	0.32418	0.7879	0.87448	-0.1935	0.007915	0.025772	0.69311	-0.52884	0.67655	0.83269	0.94571	-0.08053	0.6335	0.8822	0.93906	-0.09072	0.6545	0.97988	0.9477	-0.07749	0.1869	0.2943	1.2374	0.30728
361.2746	10.09	olomics Pc	0.6265	0.99951	1.0508	0.071455	0.73706	0.99998	0.97616	-0.03482	0.5011	0.99941	0.95926	-0.06	0.58204	0.99905	0.9473	-0.07811	0.93887	0.99688	0.99789	-0.00304	0.063296	0.21791	1.2169	0.28316	0.006447	0.21836	1.4715	0.55728	0.1894	0.98781	1.1736	0.23095	0.044083	0.28476	1.1462	0.19682
140.0691	0.58	omics Pos	0.6269	0.99987	0.92682	-0.10964	0.71359	0.99994	0.94295	-0.08475	0.97733	0.99996	0.99621	-0.00548	0.82409	0.98451	1.0626	0.087599	0.17805	0.27005	1.1744	0.23187	0.02866	0.14888	1.5103	0.59479	0.002026	0.24919	1.8329	0.87416	0.25266	0.94365	1.3028	0.38167	0.27146	0.38093	1.2266	0.29466
179.1425	2.28	omics Pos	0.62718	0.99987	1.1461	0.19674	0.82212	0.99994	1.013	0.018703	0.12773	0.99996	0.69121	-0.53281	0.02086	0.26222	0.50504	-0.98554	0.000571	0.003656	0.34019	-1.5556	0.011536	0.09183	0.34322	-1.5428	0.021283	0.33809	0.47136	-1.0851	0.021233	0.52955	0.41045	-1.2847	0.024297	0.11109	0.46068	-1.1182
829.5544	4.77	omics Pos	0.62822	0.99987	1.0334	0.04743	0.52402	0.99994	0.94628	-0.07967	0.77363	0.99996	0.97883	-0.03087	0.58768	0.92519	0.95963	-0.05946	0.002601	0.010996	0.74896	-0.41705	0.32582	0.60075	0.88979	-0.16846	0.53137	0.84189	0.94828	-0.07661	0.50718	0.96776	0.94771	-0.07749	0.12896	0.23382	1.1083	0.14841
887.6906	7.33	omics Pos	0.62829	0.99987	0.91419	-0.12944	0.82933	0.99994	0.95169	-0.07144	0.67855	0.99996	0.92873	-0.10668	0.14832	0.57591	0.71155	-0.49097	0.019104	0.050341	0.52763	-0.92241	0.01986	0.12097	0.53016	-0.91549	0.002532	0.26175	0.49857	-1.0041	0.001301	0.41831	0.43421	-1.2035	0.14393	0.24827	0.73522	-0.44375
1159.259	9	olomics Pc	0.62847	0.99951	1.1733	0.23057	0.30817	0.99998	0.82847	-0.27148	0.94114	0.99941	1.0197	0.02809	0.008142	0.19613	0.64024	-0.64331	0.061285	0.195	0.63019	-0.66615	0.070526	0.23583	0.73269	-0.44872	0.88204	0.9951	1.1124	0.15368	0.9276	0.99956	1.0299	0.042572	0.040693	0.28339	1.6108	0.68782
328.0387	0.54	omics Pos	0.629	0.99987	0.92167	-0.11768	0.17388	0.99994	0.80689	-0.30955	0.23394	0.99996	1.2145	0.28039	0.11834	0.53907	1.3992	0.48456	0.88798	0.90531	1.0109	0.015677	0.40063	0.64773	1.0973	0.1339	0.033544	0.36346	1.4555	0.54154	0.6172	0.97692	1.0557	0.078248	0.31904	0.42941	1.2272	0.29539
759.5723	4.94	omics Pos	0.62943	0.99987	1.0424	0.059924	0.43101	0.99994	0.94022	-0.08894	0.59623	0.99996	0.95994	-0.05898	0.25338	0.71067	0.90089	-0.15057	0.005457	0.01952	0.74765	-0.41956	0.41506	0.65507	0.9335	-0.09928	0.52563	0.83957	1.0572	0.080259	0.40773	0.96776	1.0491	0.069164	0.014752	0.098791	1.2446	0.31568
119.1266	9.96	omics Pos	0.62966	0.99987	1.0253	0.036043	0.2092	0.99994	0.93321	-0.09973	0.075981	0.99996	0.91103	-0.13443	0.32634	0.78936	0.95028	-0.07357	0.002861	0.011833	0.79277	-0.33503	0.16965	0.45659	0.88801	-0.17135	0.82118	0.96018	0.98959	-0.01509	0.26722	0.94365	0.96195	-0.05596	0.032802	0.12371	1.1711	0.22786
539.2707	2.34	olomics Pc	0.62967	0.99951	1.2002	0.2633	0.66758	0.99998	0.81801	-0.2898	0.31968	0.99941	0.66255	-0.5939	0.13665	0.51735	0.5147	-0.95821	0.003423	0.022474	0.12637	-2.9843	0.16618	0.42318	0.46492	-1.1049	0.39677	0.97985	0.71344	-0.48714	0.33634	0.99956	0.69065	-0.53396	0.47104	0.73148	1.2037	0.26752
1092.737	10.05	omics Pos	0.6298	0.99987	1.0246	0.035061	0.20858	0.99994	0.90736	-0.14026	0.13672	0.99996	0.87706	-0.18925	0.48839	0.88725	0.95052	-0.07321	0.00124	0.006425	0.73289	-0.44833	0.38138	0.63832	0.88941	-0.16908	0.45072	0.80167	0.94896	-0.07558	0.91845	0.98289	0.99864	-0.00196	0.01239	0.098791	1.2719	0.34694
402.7698	4.61	omics Pos	0.63055	0.99987	1.0603	0.084425	0.65727	0.99994	1.0475	0.066993	0.23624	0.99996	1.1714	0.22821	0.7392	0.97225	1.0425	0.059996	0.61158	0.66933	0.90021	-0.15167	0.39719	0.64557	1.2493	0.32107	0.038764	0.37121	1.5325	0.61585	0.52495	0.96776	1.1195	0.16282	0.17417	0.28189	1.4914	0.57663
940.8186	0.5	omics Pos	0.63057	0.99987	0.97422	-0.03769	0.13465	0.99994	0.93168	-0.10209	0.023889	0.99996	0.87802	-0.18767	0.001618	0.17581	0.82514	-0.27729	0.000237	0.001888	0.73078	-0.45249	0.1301	0.39433	0.81707	-0.29147	0.05445	0.41523	0.84449	-0.24385	0.037774	0.64014	0.8855	-0.17544	0.089923	0.19038	1.1464	0.19716
598.2661	0.96	omics Pos	0.63308	0.99987	0.93935	-0.09027	0.46761	0.99994	0.82971	-0.26932	0.74733	0.99996	0.93062	-0.10374	0.57472	0.91693	0.86722	-0.20552	0.32728	0.42159	0.73537	-0.44346	0.15711	0.438	1.7279	0.78898	0.021894	0.33809	2.1569	1.109	0.20958	0.92655	1.4525	0.53857	0.035739	0.12841	2.5489	1.3499
429.2405	10.14	omics Pos	0.63319	0.99987	0.9553	-0.06598	0.82955	0.99994	0.98713	-0.01868	0.93316	0.99996	1.0063	0.008992	0.72341	0.96819	0.97671	-0.03399	0.026979	0.065442	0.83626	-0.25797	0.39209	0.64248	0.87944	-0.18535	0.074422	0.48057	1.176	0.23385	0.32238	0.9529	1.0952	0.13117	0.021576	0.1083	1.2248	0.29249
273.9384	10.15	omics Pos	0.63324	0.99987	1.0249	0.035447	0.43777	0.99994	0.93591	-0.09556	0.44519	0.99996	0.93284	-0.1003	0.060024	0.42305	0.86359	-0.21158	0.005747	0.020326	0.72616	-0.46164	0.29739	0.57957	0.8879	-0.17153	0.35242	0.75095	1.0543	0.076313	0.22139	0.94047	0.91217	-0.13263	0.10996	0.21358	1.1728	0.23
355.2527	2.72	omics Pos	0.63359	0.99987	1.1015	0.13948	0.40841	0.99994	0.84366	-0.24527	0.1282	0.99996	0.73778	-0.43873	0.051143	0.38615	0.6154	-0.70039	0.009888	0.030429	0.47543	-1.0727	0.026054	0.14253	0.44202	-1.1778	0.012722	0.3324	0.50425	-0.98778	0.025952	0.56113	0.52491	-0.92986	0.14388	0.24827	0.66972	-0.57837
625.8519	10.05	omics Pos	0.6336	0.99987	0.9549	-0.06658	0.34337	0.99994	0.91718	-0.12472	0.15514	0.99996	0.87266	-0.1965	0.70014	0.96092	0.96164	-0.05644	0.016523	0.044905	0.76787	-0.38107	0.33867	0.60941	0.88265	-0.18009	0.89467	0.97532	0.99152	-0.01229	0.29112	0.94825	0.90698	-0.14086	0.10825	0.21125	1.1497	0.20131
581.3092	2.4	omics Pos	0.63371	0.99987	1.1527	0.20505	0.92947	0.99994	0.9176	-0.12406	0.83975	0.99996	0.97755	-0.03275	0.71242	0.96346	1.0766	0.10643	0.26442	0.35957	1.3064	0.38555	0.021214	0.12671	2.1722	1.1192	0.025173	0.33809	1.9658	0.97512	0.19446	0.92582	1.7196	0.78211	0.023761	0.11051	2.2755	1.1862
304.219	2.28	omics Pos	0.63372	0.99987	0.94466	-0.08213	0.4189	0.99994	0.87266	-0.19651	0.13108	0.99996	0.74663	-0.42154	0.020257	0.25894	0.57466	-0.79922	0.001656	0.00786	0.51542	-0.95618	0.010312	0.08787	0.49266	-1.0213	0.056032	0.42391	0.63578	-0.65341	0.060372	0.72218	0.57186	-0.80627	0.25591	0.36505	0.78079	-0.35699
171.9851	0.6	omics Pos	0.63414	0.99987	1.1183	0.16126	0.74	0.99994	0.89306	-0.16317	0.57227	0.99996	0.84514	-0.24274	0.5263	0.89938	0.82552	-0.27662	0.002703	0.011325	0.32854	-1.6059	0.24561	0.53164	0.68033	-0.55569	0.88945	0.97532	0.99877	-0.00177	0.19254	0.92582	0.66344	-0.59197	0.88402	0.9151	0.99772	-0.00329
355.0167	2.63	olomics Ne	0.63455	0.99918	1.0128	0.01836	0.024443	0.40327	0.91105	-0.1344	0.61654	0.99995	0.98398	-0.02331	0.1501	0.99985	0.94397	-0.08318	0.01731	0.88267	0.88015	-0.18418	0.11459	0.58058	0.89074	-0.16693	0.013358	0.2716	0.83922	-0.25287	0.12288	0.99512	0.93585	-0.09566	0.52768	0.99941	0.97492	-0.03665
530.973	12	olomics Ne	0.63458	0.99918	0.97168	-0.04144	0.022376	0.40327	0.82066	-0.28515	0.044899	0.99995	0.89465	-0.16061	0.78076	0.99985	0.98295	-0.02481	0.80856	0.99927	1.053	0.074537	0.8416	0.96463	0.98833	-0.01694	0.25273	0.63479	0.9333	-0.09959	0.99668	0.99912	1.0126	0.018003	0.79188	0.99941	0.98628	-0.01993
556.8611	0.54	omics Pos	0.63471	0.99987	0.95699	-0.06343	0.17858	0.99994	0.86339	-0.21191	0.20152	0.99996	0.88463	-0.17686	0.048533	0.3779	0.82645	-0.27501	0.002899	0.01195	0.69242	-0.53028	0.21651	0.50811	0.83062	-0.26774	0.23782	0.67844	0.8553	-0.2255	0.15522	0.91321	0.8586	-0.21995	0.27162	0.38093	1.1478	0.1989
1168.737	0.53	omics Pos	0.63497	0.99987	1.0417	0.058991	0.22236	0.99994	0.88718	-0.1727	0.079221	0.99996	0.87155	-0.19835	0.36895	0.82243	0.9334	-0.09944	0.000868	0.005004	0.79793	-0.32566	0.77136	0.88192	0.98304	-0.02468	0.33198	0.74016	0.90268	-0.14772	0.29801	0.9483	0.93391	-0.09864	0.008341	0.092495	1.2356	0.30521
319.1964	6.48	olomics Pc	0.63497	0.99951	0.93921	-0.09048	0.70395	0.99998	0.97114	-0.04225	0.084303	0.99941	0.67125	-0.57507	0.061595	0.32609	0.68942	-0.53655	0.041584	0.14603	0.70333	-0.50772	0.029962	0.13218	0.61361	-0.70461	0.10682	0.6278	0.63675	-0.6512	0.018473	0.51556	0.47083	-1.0867	0.013777	0.2688	0.41371	-1.2733
760.5747	4.94	omics Pos	0.63555	0.99987	1.0401	0.056774	0.44663	0.99994	0.94716	-0.07832	0.55564	0.99996	0.9593	-0.05994	0.21388	0.66269	0.90216	-0.14854	0.003655	0.01428	0.75257	-0.4101	0.41299	0.65507	0.93473	-0.09738	0.4449	0.80121	1.0671	0.093672	0.36464	0.96776	1.0464	0.065399	0.010763	0.094875	1.2442	0.31523
585.2702	6.98	olomics Pc	0.63581	0.99951	0.93939	-0.0902	0.8582	0.99998	1.0049	0.007042	0.90563	0.99941	1.0068	0.009748	0.92225	0.99905	1.0039	0.005587	0.008323	0.042081	0.69011	-0.5351	0.029834	0.13218	0.67259	-0.5722	0.030587	0.35049	0.71159	-0.49088	0.095431	0.81634	0.71866	-0.47663	0.14627	0.39824	0.75627	-0.40302
242.9373	10.11	omics Pos	0.63584	0.99987	1.0227	0.032371	0.2012	0.99994	0.92877	-0.10661	0.45763	0.99996	0.96161	-0.05647	0.010869	0.21362	0.87797	-0.18775	0.000271	0.002066	0.76755	-0.38167	0.22891	0.51678	0.89185	-0.16513	0.6343	0.8822	1.0304	0.043154	0.84858	0.97988	0.99134	-0.01255	0.046361	0.14295	1.162	0.21656
548.2749	1.07	omics Pos	0.63591	0.99987	0.96342	-0.05376	0.72593	0.99994	0.98137	-0.02713	0.71819	0.99996	1.0217	0.030993	0.19652	0.64175	0.84634	-0.24069	0.04875	0.10236	0.71961	-0.47471	0.9101	0.9531	1.104	0.14279	0.17772	0.64616	1.603	0.68074	0.19639	0.92582	1.3322	0.4138	0.00668	0.090221	1.4064	0.49199
205.1149	0.33	olomics Pc	0.63607	0.99951	0.87226	-0.19717	0.46326	0.99998	0.8221	-0.28262	0.30604	0.99941	0.74386	-0.4269	0.023084	0.22443	0.50254	-0.99268	0.027014	0.10595	0.48599	-1.041	0.012117	0.083858	0.43386	-1.2047	0.021256	0.31584	0.4717	-1.0841	0.37549	0.99956	0.76844	-0.37999	0.015521	0.2688	0.4188	-1.2557
796.5257	4.95	omics Pos	0.63612	0.99987	0.97211	-0.04081	0.27091	0.99994	0.88796	-0.17144	0.50587	0.99996	0.96317	-0.05413	0.69215	0.96092	0.97256	-0.04014	0.002005	0.00907	0.75661	-0.40237	0.40984	0.65377	0.9085	-0.13844	0.98352	0.99431	1.0088	0.012606	0.35924	0.96776	0.95023	-0.07365	0.036945	0.13049	1.2229	0.29026
898.7956	10.03	omics Pos	0.63616	0.99987	1.0241	0.034331	0.19946	0.99994	0.93636	-0.09486	0.021749	0.99996	0.87821	-0.18736	0.03984	0.34602	0.88664	-0.17357	0.001329	0.006736	0.74364	-0.42732	0.26824	0.55648	0.90302	-0.14718	0.80325	0.95444	1.0187	0.026754	0.45945	0.96776	0.95678	-0.06375	0.057744	0.15492	1.1952	0.25728
250.9483	10.16	omics Pos	0.63723	0.99987	1.0421	0.059527	0.91355	0.99994	0.99371	-0.0091	0.33786	0.99996	0.91435	-0.12919	0.88359	0.9874	1.0236	0.033609	0.009935	0.030551	0.76741	-0.38193	0.24029	0.52848	0.88847	-0.1706	0.59742	0.86481	0.96469	-0.05186	0.52528	0.96776	1.0714	0.099552	0.023853	0.11051	1.1807	0.23963
349.1839	0.59	omics Pos	0.63807	0.99987	1.1025	0.14077	0.62948	0.99994	0.88832	-0.17084	0.93694	0.99996	0.95585	-0.06515	0.28063	0.74609	0.7905	-0.33915	0.028886	0.068882	0.60825	-0.71727	0.41351	0.65507	0.8227	-0.28156	0.91074	0.97939	0.9596	-0.05949	0.46844	0.96776	0.84395	-0.24477	0.16107	0.26839	1.4668	0.55263
242.9262	0.51	omics Pos	0.63839	0.99987	1.1004	0.13807	0.39396	0.99994	0.91175	-0.13329	0.57119	0.99996	0.94858	-0.07616	0.90709	0.9874	0.99159	-0.01219	0.012164	0.035552	0.78319	-0.35256	0.98613	0.99055	1.0106	0.015169	0.21912	0.66101	1.1028	0.14113	0.93755	0.98616	0.99293	-0.01024	0.015353	0.09885	1.217	0.28327
1043.802	0.5	omics Pos	0.6387	0.99987	0.97024	-0.04358	0.40539	0.99994	0.95902	-0.06036	0.094626	0.99996	0.8917	-0.16536	0.035122	0.32905	0.85492	-0.22614	0.000642	0.003947	0.72084	-0.47226	0.14928	0.42543	0.86624	-0.20716	0.24349	0.67954	0.91115	-0.13423	0.1014	0.8541	0.89761	-0.15584	0.033944	0.12554	1.1699	0.22635
736.8556	0.51	omics Pos	0.63892	0.99987	1.0266	0.03789	0.39905	0.99994	0.9142	-0.12941	0.032529	0.99996	0.83923	-0.25286	0.008779	0.19971	0.79321	-0.33422	0.001863	0.008589	0.68462	-0.54662	0.13191	0.39728	0.79842	-0.32477	0.88345	0.97532	0.98838	-0.01686	0.38415	0.96776	0.92375	-0.11442	0.54258	0.63873	1.064	0.089533
628.8351	10.15	omics Pos	0.63915	0.99987	1.049	0.069062	0.95436	0.99994	1.0044	0.006396	0.17854	0.99996	0.90697	-0.14087	0.9275	0.9874	1.0059	0.008492	0.0024	0.010374	0.76814	-0.38056	0.30222	0.582	0.90147	-0.14965	0.86586	0.97505	1.0215	0.030628	0.11991	0.88537	0.93194	-0.10169	0.005217	0.088007	1.1759	0.23375
1094.224	9.04	olomics Pc	0.63975	0.99951	1.1568	0.21013	0.21962	0.99998	0.78628	-0.34689	0.84151	0.99941	0.98672	-0.01928	0.0638	0.33109	0.73195	-0.45018	0.031518	0.12005	0.61597	-0.69908	0.048409	0.18257	0.72247	-0.469	0.79754	0.99391	1.146	0.19655	0.80722	0.99956	1.0866	0.11976	0.062488	0.30404	1.5104	0.59493
242.9283	0.28	olomics Pc	0.6406	0.99951	1.1158	0.15803	0.90517	0.99998	0.99012	-0.01432	0.59692	0.99941	0.94837	-0.07648	0.58026	0.99905	1.0472	0.06647	0.27538	0.63347	1.0915	0.12629	0.081962	0.26027	1.1953	0.25743	0.048955	0.44698	1.2236	0.29112	0.29702	0.99956	1.0877	0.12127	0.48336	0.74056	1.0611	0.085592
563.3115	5.1	olomics Pc	0.64063	0.99951	1.233	0.30221	0.50449	0.99998	1.1666	0.22233	0.52175	0.99941	1.1522	0.20435	0.34	0.83302	1.1932	0.2549	0.33243	0.71189	1.3582	0.44171	0.009757	0.079214	1.9664	0.97552	0.008794	0.23958	2.1317	1.092	0.30331	0.99956	1.1937	0.25548	0.092362	0.34594	1.443	0.52906
752.5547	10.11	olomics Ne	0.64087	0.99918	1.0354	0.050136	0.62129	0.90725	0.93296	-0.10011	0.8961	0.99995	1.0325	0.046077	0.82348	0.99985	0.97364	-0.03854	0.56902	0.99927	1.114	0.15575	0.15014	0.6202	1.1763	0.23425	0.034112	0.3559	1.3214	0.40204	0.39648	0.99512	1.133	0.18009	0.87698	0.99941	0.97315	-0.03926
705.1611	1.12	omics Pos	0.64103	0.99987	1.1472	0.19812	0.70117	0.99994	1.1705	0.22716	0.50126	0.99996	1.1513	0.20323	0.70909	0.96346	1.0741	0.10312	0.71856	0.75981	1.0318	0.045149	0.064014	0.25399	1.4607	0.54667	0.023301	0.33809	1.6532	0.72525	0.20687	0.92582	1.2932	0.37096	0.045536	0.14174	1.6949	0.7612
234.8943	10.14	omics Pos	0.64209	0.99987	1.0293	0.041689	0.13182	0.99994	0.9061	-0.14226	0.57906	0.99996	0.95959	-0.05951	0.30279	0.76085	0.91801	-0.12341	0.011685	0.03455	0.77708	-0.36387	0.65356	0.81917	0.96322	-0.05406	0.65166	0.89057	0.96481	-0.05168	0.56432	0.9703	0.95297	-0.0695	0.1829	0.28998	1.1355	0.18335
460.7114	0.35	olomics Ne	0.64279	0.99918	1.0734	0.10213	0.66526	0.92948	1.0279	0.03975	0.15248	0.99995	1.0695	0.096875	0.74969	0.99985	1.0242	0.034494	0.1171	0.99927	1.1348	0.18238	0.007494	0.36417	1.3128	0.39261	0.054831	0.39335	1.2688	0.34343	0.007373	0.99512	1.1887	0.24935	0.43537	0.99941	1.0621	0.086869
1067.605	1.09	omics Pos	0.64331	0.99987	0.91155	-0.1336	0.80993	0.99994	0.96908	-0.04531	0.71429	0.99996	0.91135	-0.13392	0.76103	0.97526	0.91821	-0.1231	0.7274	0.76788	1.1315	0.17822	0.080042	0.29262	1.8133	0.85859	0.005186	0.3088	2.7132	1.44	0.40197	0.96776	1.2446	0.31569	0.25836	0.36786	1.4865	0.57192
514.8728	0.5	omics Pos	0.64334	0.99987	1.1184	0.16146	0.2804	0.99994	0.87175	-0.19802	0.42025	0.99996	0.91126	-0.13406	0.57758	0.91955	0.94271	-0.08511	0.014873	0.041608	0.7188	-0.47634	0.97407	0.9856	1.005	0.007248	0.2553	0.69047	1.1364	0.1845	0.83016	0.97988	1.0249	0.035466	0.011398	0.095518	1.2943	0.37217
464.2125	2.85	omics Pos	0.6442	0.99987	1.0592	0.083026	0.048122	0.99994	0.77242	-0.37254	0.021046	0.99996	0.79367	-0.33338	0.46416	0.8814	0.90503	-0.14397	0.050183	0.10455	0.72988	-0.45427	0.10418	0.34339	0.6851	-0.54562	0.17909	0.64777	0.84408	-0.24455	0.21996	0.94047	0.81389	-0.29709	0.82745	0.87278	1.0716	0.099798
894.5436	8.54	olomics Ne	0.64422	0.99918	1.0467	0.065879	0.24654	0.67145	0.86295	-0.21265	0.98254	0.99995	1.0072	0.010386	0.88555	0.99985	0.99269	-0.01059	0.75357	0.99927	1.0646	0.090343	0.028365	0.47167	1.2973	0.37554	0.051883	0.39335	1.2693	0.34403	0.18133	0.99512	1.242	0.31262	0.11055	0.99941	1.2023	0.26581
91.9811	10.03	omics Pos	0.64438	0.99987	1.0465	0.06557	0.44322	0.99994	0.90963	-0.13664	0.63191	0.99996	0.94957	-0.07465	0.83562	0.98621	0.97427	-0.0376	0.015159	0.042067	0.7339	-0.44634	0.71789	0.85308	0.9574	-0.0628	0.79425	0.95091	0.96817	-0.04667	0.73985	0.97988	0.96116	-0.05715	0.18025	0.28757	1.2369	0.30667
120.0821	0.6	olomics Pc	0.64451	0.99951	1.0616	0.086218	0.81739	0.99998	1.0453	0.063914	0.22091	0.99941	1.1642	0.21934	0.027789	0.24036	1.664	0.73467	0.099193	0.28449	1.3752	0.45968	0.05167	0.19171	1.3468	0.42951	0.004373	0.20152	1.6724	0.74194	0.69936	0.99956	1.0579	0.081197	0.8465	0.94089	1.0319	0.045285
476.9104	12.06	olomics Pc	0.64458	0.99951	1.0534	0.075084	0.92506	0.99998	1.0154	0.022086	0.35608	0.99941	1.193	0.25455	0.96273	0.99905	0.99506	-0.00715	0.015554	0.068239	1.2967	0.37481	0.61498	0.87561	1.0993	0.13656	0.14885	0.72113	1.2685	0.34315	0.45382	0.99956	1.1237	0.1683	0.19042	0.45511	1.2322	0.30129
586.2737	6.98	olomics Pc	0.64463	0.99951	0.93284	-0.10029	0.85899	0.99998	0.98679	-0.01919	0.68475	0.99941	0.93626	-0.09502	0.93146	0.99905	0.97423	-0.03767	0.09371	0.27156	0.77282	-0.3718	0.030011	0.13222	0.65438	-0.61179	0.11701	0.64891	0.74568	-0.42337	0.16788	0.95629	0.75062	-0.41385	0.12113	0.3708	0.74287	-0.42881
725.5565	4.66	omics Pos	0.64465	0.99987	1.0407	0.057524	0.51749	0.99994	0.92525	-0.11208	0.80892	0.99996	0.97519	-0.03624	0.64427	0.95178	0.95322	-0.06912	0.03182	0.074116	0.7663	-0.38401	0.3193	0.5964	0.84996	-0.23453	0.58388	0.86163	0.94356	-0.08382	0.27393	0.94365	0.88375	-0.17829	0.97836	0.98378	0.9958	-0.00607
1024.758	0.53	omics Pos	0.64561	0.99987	1.0683	0.095348	0.43545	0.99994	0.92844	-0.10711	0.19703	0.99996	0.87267	-0.1965	0.28845	0.75229	0.93348	-0.09931	0.005877	0.020635	0.73985	-0.4347	0.31027	0.58859	0.86963	-0.20153	0.95582	0.98985	1.0022	0.003137	0.25961	0.94365	0.89011	-0.16795	0.03524	0.12781	1.2601	0.33355
235.2385	9.97	omics Pos	0.64568	0.99987	0.97275	-0.03987	0.53315	0.99994	1.1528	0.20509	0.96643	0.99996	1.0001	8.01E-05	0.56015	0.91079	0.96104	-0.05734	0.008549	0.027188	0.78248	-0.35387	0.41711	0.65592	0.92277	-0.11595	0.21707	0.66052	1.1123	0.15352	0.85719	0.98023	0.98813	-0.01722	0.046981	0.14352	1.2955	0.37354
948.7123	7.17	omics Pos	0.64585	0.99987	1.1732	0.23046	0.66006	0.99994	1.2096	0.27458	0.64222	0.99996	1.1568	0.21011	0.82212	0.98421	1.033	0.046775	0.50987	0.5826	0.73455	-0.44507	0.27637	0.56257	0.60601	-0.7226	0.28086	0.7098	0.60398	-0.72742	0.041123	0.64308	0.29801	-1.7466	0.028031	0.11681	0.25866	-1.9509
464.2837	1.09	omics Pos	0.64599	0.99987	0.6639	-0.59096	0.58518	0.99994	0.687	-0.54162	0.23789	0.99996	1.464	0.54992	0.12984	0.55135	2.8971	1.5346	0.029954	0.070808	3.6006	1.8482	0.030119	0.15519	4.2228	2.0782	0.00945	0.3324	8.6361	3.1104	0.73644	0.97988	1.0857	0.11864	0.82801	0.87312	0.46853	-1.0938
788.9862	6.5	olomics Pc	0.64614	0.99951	0.9452	-0.08131	0.38702	0.99998	0.87841	-0.18703	0.32299	0.99941	0.87322	-0.19558	0.027677	0.24036	0.69219	-0.53076	0.45473	0.85773	0.89037	-0.16752	0.028879	0.12915	0.67712	-0.56252	0.21159	0.80495	0.79362	-0.33349	0.47616	0.99956	0.89763	-0.1558	0.54125	0.7778	0.91285	-0.13155
905.4811	0.54	omics Pos	0.64645	0.99987	1.1858	0.24586	0.069026	0.99994	0.64349	-0.636	0.56583	0.99996	0.8712	-0.19893	0.01624	0.24	0.49251	-1.0218	0.002544	0.010801	0.33659	-1.5709	0.012745	0.095104	0.50934	-0.97329	0.27384	0.70555	0.74006	-0.43429	0.43094	0.96776	0.82438	-0.27861	0.056475	0.15348	1.8554	0.89174
601.785	2.36	olomics Ne	0.64664	0.99918	1.2624	0.33618	0.97948	0.99664	0.95384	-0.06819	0.7274	0.99995	0.87291	-0.19609	0.38027	0.99985	0.6585	-0.60275	0.011268	0.76891	0.23707	-2.0766	0.24178	0.68109	0.54765	-0.86867	0.46452	0.78972	0.72335	-0.46723	0.74354	0.99512	0.91819	-0.12313	0.61911	0.99941	1.0962	0.13257
1047.722	9.06	olomics Pc	0.64719	0.99951	1.0891	0.12315	0.30962	0.99998	0.87247	-0.19682	0.97177	0.99941	1.0015	0.002139	0.071176	0.34699	0.71034	-0.49342	0.012881	0.058807	0.64016	-0.6435	0.043637	0.17012	0.74173	-0.43103	0.81726	0.99391	0.98859	-0.01655	0.88743	0.99956	1.0482	0.067906	0.4032	0.67918	1.3394	0.4216
156.0567	0.31	olomics Pc	0.64734	0.99951	0.91532	-0.12766	0.85787	0.99998	0.96375	-0.05327	0.86348	0.99941	0.95825	-0.06152	0.45811	0.93704	1.1301	0.17641	0.066866	0.20772	1.3392	0.42141	0.1011	0.30242	1.384	0.46884	0.030195	0.34968	1.4485	0.53458	0.42564	0.99956	1.1679	0.22388	0.60588	0.81615	1.0703	0.098013
197.0378	0.34	omics Pos	0.64772	0.99987	0.95497	-0.06647	0.41391	0.99994	0.90087	-0.1506	0.15244	0.99996	0.85194	-0.23118	0.28821	0.75229	0.85307	-0.22927	0.021767	0.055592	0.71524	-0.4835	0.058815	0.24091	0.82985	-0.26908	0.5595	0.85477	0.92905	-0.10617	0.74373	0.97988	1.0186	0.026592	0.12179	0.22609	1.1592	0.21315
257.1476	0.58	omics Pos	0.64777	0.99987	0.84247	-0.2473	0.65384	0.99994	0.82892	-0.2707	0.81079	0.99996	0.8801	-0.18425	0.91035	0.9874	1.0825	0.11437	0.080614	0.14921	1.5841	0.66368	0.028038	0.14787	2.388	1.2558	0.004175	0.3088	3.2719	1.7101	0.3286	0.95887	1.7946	0.8437	0.34075	0.45075	1.387	0.47198
519.3019	9.6	olomics Pc	0.64811	0.99951	0.90746	-0.1401	0.68531	0.99998	0.92186	-0.11738	0.6912	0.99941	1.1331	0.18029	0.96769	0.99905	0.99609	-0.00566	0.060196	0.1927	1.5626	0.64395	0.009649	0.078946	2.1379	1.0962	0.001839	0.16989	3.0026	1.5862	0.40286	0.99956	1.1105	0.15125	0.72408	0.87792	1.052	0.073164
1113.258	9.02	olomics Pc	0.64812	0.99951	1.1374	0.1857	0.26176	0.99998	0.83246	-0.26454	0.48762	0.99941	0.93613	-0.09522	0.007181	0.1948	0.63958	-0.64481	0.015252	0.067288	0.59255	-0.75499	0.015254	0.092479	0.72702	-0.45994	0.82333	0.9951	1.1149	0.15697	0.83441	0.99956	0.99383	-0.00893	0.045864	0.28476	1.4727	0.55844
420.8861	0.54	omics Pos	0.64932	0.99987	1.021	0.029924	0.14843	0.99994	0.90542	-0.14334	0.087372	0.99996	0.89428	-0.1612	0.023333	0.27291	0.87128	-0.19879	0.001088	0.005858	0.73734	-0.4396	0.13945	0.40965	0.83799	-0.25499	0.17503	0.64606	0.88361	-0.17851	0.15821	0.91678	0.90495	-0.14409	0.13756	0.24298	1.1642	0.21934
683.4912	5.86	omics Pos	0.65001	0.99987	0.93374	-0.09891	0.43399	0.99994	0.88099	-0.18281	0.25146	0.99996	0.81238	-0.29978	0.11125	0.53238	0.77863	-0.36098	0.009857	0.030408	0.62271	-0.68338	0.23952	0.52848	0.79245	-0.33561	0.73212	0.92699	0.95592	-0.06504	0.59932	0.97073	0.92173	-0.11758	0.2437	0.35191	1.3938	0.47904
276.9002	0.57	omics Pos	0.65028	0.99987	1.1199	0.16338	0.92127	0.99994	1.0085	0.01227	0.37401	0.99996	0.88908	-0.16962	0.30411	0.76187	0.85209	-0.23092	0.039897	0.08806	0.72542	-0.46312	0.56894	0.76437	0.9227	-0.11607	0.78591	0.94981	0.97881	-0.0309	0.61108	0.97371	1.0568	0.079747	0.45901	0.56349	1.0797	0.11067
878.5849	5.73	omics Pos	0.65064	0.99987	1.0456	0.064327	0.56294	0.99994	0.95466	-0.06694	0.93799	0.99996	0.99678	-0.00466	0.64771	0.95407	0.96132	-0.05691	0.006717	0.022759	0.76687	-0.38295	0.34797	0.6157	0.92503	-0.11243	0.95811	0.98985	1.0126	0.018127	0.62459	0.97813	1.0292	0.041557	0.037191	0.13049	1.5754	0.65576
1093.723	9.04	olomics Pc	0.65087	0.99951	1.1736	0.23089	0.16702	0.99998	0.81261	-0.29936	0.71586	0.99941	0.96895	-0.04551	0.014	0.20085	0.68572	-0.5443	0.031892	0.12104	0.58929	-0.76295	0.0193	0.10212	0.77158	-0.37411	0.92629	0.9951	1.0947	0.1306	0.69783	0.99956	1.0869	0.12028	0.073025	0.31731	1.4945	0.57967
289.872	12.71	olomics Ne	0.65118	0.99918	1.0225	0.032067	0.041642	0.46524	0.87084	-0.19952	0.58777	0.99995	1.0403	0.05699	0.97853	0.99985	1.0141	0.020176	0.6133	0.99927	0.96365	-0.05342	0.045427	0.48405	1.2072	0.27161	0.5019	0.81279	1.0352	0.04991	0.97692	0.99512	1.007	0.010023	0.49567	0.99941	0.95018	-0.07373
392.2284	2.28	omics Pos	0.65226	0.99987	1.0979	0.13468	0.30969	0.99994	0.88328	-0.17906	0.10111	0.99996	0.78625	-0.34695	0.000778	0.17581	0.62708	-0.67328	0.000263	0.002017	0.51001	-0.97139	0.015935	0.10754	0.53498	-0.90244	0.025027	0.33809	0.58571	-0.77174	0.057717	0.71686	0.62622	-0.67526	0.11063	0.21423	0.67156	-0.57441
785.0438	9.27	olomics Pc	0.65243	0.99951	1.1739	0.23127	0.27659	0.99998	0.88544	-0.17554	0.37922	0.99941	1.0907	0.12519	0.036396	0.25997	0.73129	-0.45148	0.055902	0.18267	0.73258	-0.44894	0.007736	0.072741	0.70723	-0.49975	0.69616	0.99391	1.0718	0.10006	0.85416	0.99956	1.0053	0.007695	0.072241	0.3167	1.5019	0.58677
263.2	4.65	olomics Pc	0.65245	0.99951	0.91365	-0.13028	0.25887	0.99998	0.75532	-0.40483	0.1238	0.99941	0.72326	-0.4674	0.61373	0.99905	0.90508	-0.14389	1.87E-06	0.000231	0.053712	-4.2186	0.077272	0.24986	0.54541	-0.87458	0.40887	0.98164	0.82542	-0.27681	0.9143	0.99956	1.0342	0.048555	0.43182	0.69935	0.83323	-0.26322
712.4926	8.54	olomics Ne	0.65257	0.99918	1.0747	0.10387	0.61282	0.9021	0.85636	-0.22371	0.81803	0.99995	1.019	0.027139	0.68654	0.99985	0.86472	-0.2097	0.94168	0.99927	0.97151	-0.0417	0.083864	0.54462	1.8526	0.88958	0.031357	0.34429	2.1405	1.0979	0.22368	0.99512	1.9873	0.99082	0.048649	0.99941	2.1535	1.1067
947.7085	7.17	omics Pos	0.65284	0.99987	1.1763	0.23432	0.6426	0.99994	1.2383	0.30835	0.66189	0.99996	1.1987	0.26152	0.79192	0.97671	1.0488	0.068734	0.65643	0.70846	0.8136	-0.29761	0.33822	0.60941	0.66472	-0.58917	0.25532	0.69047	0.59967	-0.73775	0.048325	0.67624	0.31807	-1.6526	0.025062	0.11203	0.24607	-2.0229
112.8964	0.57	omics Pos	0.65299	0.99987	1.3051	0.38418	0.41617	0.99994	0.91534	-0.12761	0.84048	0.99996	1.0301	0.042748	0.41737	0.85533	1.1901	0.25112	0.44807	0.53108	0.91741	-0.12437	0.32144	0.5977	1.2624	0.33622	0.16365	0.63418	1.313	0.39289	0.017025	0.52955	1.3168	0.39705	0.037811	0.13095	1.3458	0.42845
538.9757	12	olomics Ne	0.65305	0.99918	0.95723	-0.06306	0.021953	0.40327	0.76697	-0.38276	0.54112	0.99995	0.92794	-0.1079	0.43731	0.99985	0.89979	-0.15234	0.76969	0.99927	1.0344	0.048769	0.72638	0.94555	0.96532	-0.05093	0.35031	0.71526	0.90481	-0.14431	0.095464	0.99512	0.86366	-0.21147	0.093029	0.99941	0.83543	-0.25942
1108.729	10.08	omics Pos	0.65307	0.99987	1.0393	0.055582	0.5209	0.99994	0.96279	-0.0547	0.42332	0.99996	0.95123	-0.07213	0.47612	0.88482	1.0141	0.020142	0.00064	0.003941	0.75558	-0.40435	0.41444	0.65507	0.95472	-0.06685	0.82747	0.96399	1.0211	0.030058	0.37566	0.96776	1.0846	0.11718	0.020885	0.10765	1.2098	0.27478
159.1367	2.05	omics Pos	0.6533	0.99987	1.0435	0.061478	0.3973	0.99994	0.90509	-0.14386	0.25964	0.99996	0.89688	-0.15702	0.12035	0.54117	0.74522	-0.42426	3.14E-05	0.000482	0.24548	-2.0263	0.005463	0.075197	0.57353	-0.80205	0.26042	0.69579	0.87904	-0.18599	0.5469	0.96776	0.94856	-0.07618	0.001254	0.07528	1.4109	0.49657

333.1542	6.06	olomics Po	0.65343	0.99951	0.95141	-0.07186	0.42763	0.99998	0.87723	-0.18897	0.012897	0.99941	0.6095	-0.7143	0.043707	0.2827	0.52864	-0.91966	0.001542	0.012837	0.40178	-1.3155	0.008813	0.075866	0.43127	-1.2133	0.000489	0.16989	0.40689	-1.2973	0.012356	0.47452	0.42882	-1.2216	0.036236	0.27637	0.51525	-0.95665
685.4357	5.6	omics Pos	0.65344	0.99987	1.0157	0.02251	0.38673	0.99994	0.92979	-0.10502	0.23075	0.99996	1.0656	0.091639	0.036249	0.33474	0.83931	-0.25273	0.00444	0.016615	0.74401	-0.4266	0.18807	0.47429	0.84004	-0.25148	0.79197	0.95091	0.98882	-0.01623	0.15727	0.9146	0.88598	-0.17466	0.054666	0.15191	1.0904	0.12484
207.9248	12.7	olomics Ne	0.65358	0.99918	0.977	-0.03357	0.004669	0.34605	0.85769	-0.22147	0.70993	0.99995	0.98394	-0.02335	0.90264	0.99985	0.99656	-0.00497	0.6844	0.99927	0.97979	-0.02945	0.31779	0.73392	1.0578	0.081068	0.096517	0.46226	1.0873	0.1208	0.76666	0.99512	0.98871	-0.01638	0.39474	0.99941	0.9671	-0.04827
175.1484	4.73	omics Pos	0.65406	0.99987	1.0233	0.033184	0.29381	0.99994	0.93087	-0.10335	0.88307	0.99996	0.98886	-0.01616	0.97264	0.99502	0.9978	-0.00318	0.006308	0.021701	0.80247	-0.31748	0.8508	0.92534	0.98657	-0.0195	0.58409	0.86163	1.0287	0.040884	0.97896	0.9929	1.0081	0.011651	0.069866	0.17098	1.1986	0.26132
284.2956	7.77	olomics Po	0.65414	0.99951	0.9408	-0.08803	0.31249	0.99998	0.87971	-0.1849	0.97316	0.99941	0.99983	-0.00024	0.88267	0.99905	0.98095	-0.02775	0.050395	0.16986	0.71857	-0.47679	0.041086	0.16334	0.75277	-0.40971	0.33141	0.94653	0.84684	-0.23984	0.39799	0.99956	0.8952	-0.15972	0.24359	0.51222	0.85973	-0.21805
471.3003	2.72	omics Pos	0.65419	0.99987	0.93183	-0.10186	0.099035	0.99994	0.77357	-0.37039	0.058171	0.99996	0.71526	-0.48345	0.013938	0.22898	0.54044	-0.88781	0.001623	0.007756	0.49596	-1.0117	0.006763	0.080671	0.38637	-1.3719	0.022463	0.33809	0.54395	-0.87845	0.019109	0.52955	0.47703	-1.0679	0.17659	0.28412	0.71747	-0.47901
503.2975	5.08	olomics Po	0.65421	0.99951	0.96127	-0.05698	0.63757	0.99998	1.0504	0.070946	0.97427	0.99941	0.99621	-0.00548	0.64591	0.99905	1.0461	0.065066	0.60073	0.9704	1.1111	0.15204	0.099942	0.30007	1.2683	0.3429	0.036134	0.3794	1.6141	0.69072	0.31248	0.99956	1.1828	0.24223	0.47643	0.73747	1.1254	0.17038
141.0709	0.58	omics Pos	0.65435	0.99987	0.92553	-0.11165	0.81501	0.99994	0.95463	-0.06699	0.77896	0.99996	0.94776	-0.07741	0.74811	0.97379	1.0869	0.12028	0.1935	0.28687	1.1896	0.25053	0.033414	0.16461	1.5418	0.6246	0.002913	0.26175	1.8687	0.90204	0.2776	0.94365	1.3084	0.38777	0.24629	0.35491	1.2294	0.29797
319.1952	5.42	omics Pos	0.65455	0.99987	1.0482	0.067934	0.89354	0.99994	0.99236	-0.01107	0.31952	0.99996	0.90045	-0.15128	0.44371	0.86677	0.93116	-0.1029	0.01414	0.039939	0.77795	-0.36224	0.35858	0.62208	0.91367	-0.13025	0.37777	0.76056	1.0552	0.077572	0.29805	0.9483	1.0725	0.10093	0.007291	0.091944	1.3019	0.38059
675.9777	12.02	olomics Ne	0.65488	0.99918	1.0585	0.08198	0.56155	0.88137	0.92706	-0.10926	0.39274	0.99995	1.1455	0.19604	0.69085	0.99985	1.0812	0.11259	0.28092	0.99927	1.2421	0.31277	0.19888	0.65203	1.1688	0.22507	0.049209	0.39082	1.2452	0.31639	0.47644	0.99512	1.1385	0.18719	0.3623	0.99941	0.9101	-0.13591
830.8728	12.02	olomics Po	0.65591	0.99951	0.96748	-0.0477	0.044911	0.99998	0.77844	-0.36134	0.40802	0.99941	0.92962	-0.10528	0.32735	0.82359	0.91857	-0.12254	0.61035	0.97478	0.95928	-0.05998	0.17241	0.43275	0.90339	-0.14657	0.39626	0.97985	0.90958	-0.13673	0.059763	0.71507	0.87309	-0.19581	0.16198	0.42064	0.87522	-0.19228
463.2027	2.85	omics Pos	0.65632	0.99987	0.95725	-0.06304	0.39394	0.99994	0.89082	-0.1668	0.5881	0.99996	0.95301	-0.06944	0.1054	0.52975	0.85199	-0.23109	0.002511	0.010684	0.61315	-0.70568	0.09633	0.32824	0.69037	-0.53456	0.092232	0.52153	0.80274	-0.31699	0.20369	0.92582	0.80987	-0.30424	0.57541	0.66974	0.93529	-0.09652
191.1794	6.97	olomics Po	0.65638	0.99951	1.0858	0.11876	0.53908	0.99998	0.90989	-0.13624	0.027636	0.99941	0.70215	-0.51015	0.017853	0.20936	0.50922	-0.97363	0.001041	0.009561	0.36739	-1.4446	0.000197	0.036471	0.33961	-1.5581	0.00542	0.2079	0.40915	-1.2893	0.020179	0.51556	0.50985	-0.97187	0.024786	0.2688	0.62647	-0.67467
1071.209	9.05	olomics Po	0.65638	0.99951	1.1708	0.22749	0.27836	0.99998	0.8356	-0.25912	0.72672	0.99941	0.96849	-0.04619	0.028579	0.24036	0.68031	-0.55574	0.058904	0.18953	0.65703	-0.60597	0.038892	0.15763	0.76889	-0.37916	0.96548	0.9974	1.0883	0.12207	0.86459	0.99956	0.99882	-0.00171	0.11528	0.36506	1.4472	0.53327
267.954	10.15	omics Pos	0.65723	0.99987	1.0444	0.062634	0.43237	0.99994	0.90719	-0.14052	0.27816	0.99996	0.87664	-0.18994	0.87261	0.9874	0.97815	-0.03187	0.039207	0.087119	0.76547	-0.38557	0.78736	0.89086	0.97267	-0.03998	0.58216	0.86163	1.0683	0.095294	0.55482	0.96798	1.0627	0.087699	0.59207	0.68494	1.0985	0.13555
1048.731	0.54	omics Pos	0.65738	0.99987	1.0447	0.063145	0.33681	0.99994	0.90066	-0.15094	0.16727	0.99996	0.86364	-0.2115	0.12232	0.54337	0.83162	-0.26601	0.014916	0.041698	0.71089	-0.4923	0.18822	0.47435	0.84671	-0.24005	0.48431	0.81639	0.92082	-0.119	0.65075	0.97988	0.95216	-0.07073	0.32115	0.43132	1.1599	0.21396
632.3032	2.37	olomics Po	0.65761	0.99951	0.8991	-0.15344	0.73755	0.99998	0.91837	-0.12285	0.68849	0.99941	0.90378	-0.14595	0.28083	0.75672	0.75757	-0.40054	0.003606	0.023326	0.30097	-1.7323	0.19435	0.46875	0.63558	-0.65386	0.61605	0.99391	0.8848	-0.17657	0.53001	0.99956	0.84535	-0.24239	0.63316	0.83311	0.87584	-0.19126
484.286	1.12	omics Pos	0.65847	0.99987	0.94288	-0.08485	0.90124	0.99994	0.9793	-0.03018	0.83679	0.99996	1.0196	0.028023	0.9747	0.99556	0.99596	-0.00583	0.41938	0.50701	0.91318	-0.13103	0.56864	0.76437	1.1034	0.1419	0.008992	0.32655	1.563	0.64434	0.77661	0.97988	1.0918	0.12673	0.046254	0.14289	1.3317	0.41329
565.3086	0.93	omics Pos	0.65852	0.99987	0.92482	-0.11275	0.81424	0.99994	1.0162	0.023135	0.37597	0.99996	1.1568	0.21008	0.70811	0.96346	1.0539	0.075714	0.79995	0.82922	0.92434	-0.1135	0.13736	0.4057	1.4043	0.48988	0.043486	0.38832	1.6289	0.70391	0.76965	0.97988	0.91858	-0.12252	0.45484	0.55981	1.1642	0.21932
543.3279	4.84	olomics Po	0.65869	0.99951	0.97428	-0.03759	0.4412	0.99998	1.1069	0.14647	0.21875	0.99941	1.1557	0.20883	0.043209	0.2816	1.2723	0.34743	0.05072	0.17069	1.3008	0.37938	0.000841	0.042705	1.7173	0.78013	0.00126	0.16989	1.7745	0.82745	0.51819	0.99956	0.93792	-0.09246	0.64086	0.83691	0.95361	-0.06853
814.5381	5.35	omics Pos	0.65872	0.99987	0.92602	-0.11089	0.14308	0.99994	0.75835	-0.39907	0.30735	0.99996	0.81231	-0.29989	0.31233	0.77382	0.81327	-0.2982	0.018466	0.049059	0.54562	-0.87403	0.016793	0.10983	0.55569	-0.84766	0.90541	0.97855	1.001	0.001441	0.52151	0.96776	1.1038	0.1425	0.27634	0.38526	1.1529	0.20531
449.3583	3.52	omics Pos	0.65874	0.99987	0.94548	-0.08088	0.2453	0.99994	0.83931	-0.25273	0.031067	0.99996	0.77025	-0.3766	0.001871	0.17581	0.55517	-0.84899	0.001524	0.007407	0.44844	-1.157	0.001199	0.043885	0.41102	-1.2827	0.020219	0.33809	0.56163	-0.83232	0.011353	0.52955	0.51298	-0.96303	0.05977	0.15728	0.62877	-0.6694
237.9739	0.57	omics Pos	0.65882	0.99987	1.1078	0.14773	0.76632	0.99994	0.93288	-0.10024	0.64965	0.99996	0.88425	-0.17748	0.69804	0.96092	0.89857	-0.1543	0.021709	0.055519	0.52314	-0.93474	0.39685	0.64528	0.80991	-0.30416	0.68828	0.90885	1.0357	0.050551	0.62768	0.97904	0.8778	-0.18804	0.18694	0.2943	1.2393	0.30955
1195.826	8.47	olomics Po	0.6589	0.99951	1.1968	0.25924	0.75248	0.99998	1.1932	0.25488	0.9064	0.99941	1.1911	0.25224	0.73957	0.99905	1.2924	0.37008	0.16366	0.42845	1.8191	0.86326	0.036746	0.1515	2.3835	1.2531	0.01663	0.29901	2.4241	1.2774	0.084321	0.79247	1.6856	0.75328	0.051499	0.2951	1.7488	0.80635
573.3739	5.52	olomics Po	0.65896	0.99951	0.88641	-0.17395	0.77021	0.99998	0.93949	-0.09006	0.94705	0.99941	0.97692	-0.03368	0.66677	0.99905	0.88298	-0.17954	0.52925	0.92315	1.146	0.19659	0.11854	0.33418	1.4139	0.49964	0.037015	0.38426	1.6028	0.68055	0.6666	0.99956	1.0674	0.09408	0.26496	0.53583	1.3213	0.40199
1037.645	1.29	omics Pos	0.65971	0.99987	1.1241	0.16872	0.77802	0.99994	1.0729	0.10155	0.4701	0.99996	1.2336	0.30287	0.9816	0.99652	0.9349	-0.09711	0.84187	0.86595	0.90223	-0.14843	0.22882	0.51678	1.4664	0.55225	0.035531	0.36525	2.111	1.0779	0.21032	0.92669	1.576	0.65631	0.033905	0.12554	2.1995	1.1372
1004.788	10.02	omics Pos	0.65989	0.99987	1.026	0.037019	0.87512	0.99994	1.0124	0.017719	0.67539	0.99996	0.96895	-0.0455	0.91742	0.9874	0.99235	-0.01108	0.020978	0.054304	0.80125	-0.31968	0.30629	0.58599	0.91569	-0.12706	0.18614	0.64793	1.084	0.11631	0.27579	0.94365	1.0823	0.11413	0.072313	0.17385	1.2381	0.30813
1126.76	10.05	omics Pos	0.66006	0.99987	0.95491	-0.06656	0.17537	0.99994	0.85329	-0.2289	0.16996	0.99996	0.8737	-0.19478	0.21018	0.65763	0.85914	-0.21903	0.005986	0.020844	0.687	-0.54161	0.095669	0.32628	0.80246	-0.31751	0.36875	0.76056	0.9154	-0.12753	0.50394	0.96776	0.92326	-0.1152	0.79749	0.85011	1.0249	0.035543
280.983	3.81	olomics Ne	0.6601	0.99918	1.0136	0.019493	0.019867	0.40092	0.87072	-0.19971	0.90694	0.99995	1.0049	0.007111	0.39793	0.99985	0.95896	-0.06046	0.368	0.99927	0.96375	-0.05326	0.70351	0.94209	1.0223	0.03178	0.46712	0.79204	1.0282	0.040056	0.61117	0.99512	0.97454	-0.03721	0.51516	0.99941	0.97027	-0.04355
425.3164	2.29	omics Pos	0.66014	0.99987	1.0557	0.078196	0.48127	0.99994	0.91363	-0.13033	0.28738	0.99996	0.85506	-0.2259	0.075752	0.4637	0.72761	-0.45877	0.001346	0.006806	0.69977	-0.51505	0.003867	0.068577	0.63796	-0.64846	0.16026	0.63322	0.75638	-0.40282	0.24172	0.94365	0.81474	-0.29558	0.35251	0.46143	0.89677	-0.15719
1046.52	6.28	omics Pos	0.66041	0.99987	0.89063	-0.16709	0.71819	0.99994	0.92191	-0.1173	0.58854	0.99996	0.87455	-0.19339	0.63871	0.95076	0.8917	-0.16537	0.68309	0.73102	0.90291	-0.14734	0.36769	0.6273	1.2275	0.29574	0.046955	0.39577	1.5988	0.67699	0.11876	0.88537	1.3672	0.45122	0.039829	0.13378	1.9967	0.99761
942.6159	9.46	olomics Po	0.66057	0.99951	1.158	0.21161	0.67979	0.99998	0.92741	-0.10873	0.86615	0.99941	0.97582	-0.03532	0.11531	0.46351	0.73794	-0.43842	0.014278	0.063885	0.63592	-0.65308	0.033365	0.1422	0.6511	-0.61905	0.33992	0.95052	0.82513	-0.27731	0.19679	0.99341	0.77874	-0.36079	0.050749	0.29361	1.5995	0.67759
608.2582	6.97	olomics Po	0.66078	0.99951	0.92721	-0.10904	0.79003	0.99998	1.0185	0.026378	0.21477	0.99941	0.73837	-0.43758	0.081823	0.37784	0.65196	-0.61715	0.010191	0.048934	0.51071	-0.96942	0.000698	0.041543	0.38556	-1.375	0.03241	0.35572	0.48623	-1.0403	0.03552	0.61142	0.47502	-1.0739	0.045421	0.28476	0.53488	-0.9027
344.8693	0.56	omics Pos	0.66189	0.99987	1.0361	0.05119	0.72117	0.99994	1.0267	0.038051	0.12401	0.99996	0.93079	-0.10348	0.014391	0.22983	0.82471	-0.27804	0.001994	0.009042	0.73475	-0.44467	0.007598	0.082308	0.81303	-0.29862	0.007788	0.32129	0.89817	-0.15494	0.013271	0.52955	0.922	-0.11716	0.022845	0.10945	1.1588	0.21266
652.5365	5.35	omics Pos	0.66214	0.99987	1.0367	0.051986	0.71291	0.99994	0.95034	-0.07349	0.90482	0.99996	1.0288	0.040942	0.31275	0.77421	0.8334	-0.26292	0.00322	0.012952	0.61985	-0.69001	0.10394	0.34315	0.76048	-0.39501	0.7105	0.91681	1.0423	0.059786	0.1305	0.88537	1.1853	0.24522	0.026688	0.11512	1.349	0.43187
167.1436	2.82	omics Pos	0.66289	0.99987	1.0606	0.084867	0.66526	0.99994	0.94048	-0.08853	0.12573	0.99996	0.81318	-0.29835	0.049049	0.37885	0.63697	-0.65071	0.000176	0.001538	0.28048	-1.834	0.000824	0.040799	0.37643	-1.4096	0.024996	0.33809	0.56762	-0.817	0.016671	0.52955	0.58892	-0.76386	0.42479	0.53174	0.87713	-0.18914
607.3215	1.06	omics Pos	0.66298	0.99987	1.1551	0.20805	0.046011	0.99994	0.79347	-0.33375	0.3869	0.99996	1.1644	0.21955	0.07278	0.45752	0.69129	-0.53263	0.010705	0.032369	0.53737	-0.89601	0.0512	0.22185	0.71416	-0.48568	0.99944	0.99962	1.0536	0.07526	0.12044	0.88537	0.80225	-0.31787	0.047254	0.14378	1.3408	0.42314
1077.786	0.5	omics Pos	0.66315	0.99987	0.96322	-0.05406	0.55196	0.99994	0.95061	-0.07307	0.69959	0.99996	0.96752	-0.04763	0.098555	0.51862	0.86403	-0.21084	0.006814	0.022971	0.74866	-0.41761	0.21721	0.50811	0.84623	-0.24088	0.33946	0.74343	0.90503	-0.14396	0.3508	0.96776	0.91024	-0.13569	0.05741	0.1547	1.2077	0.27223
1079.76	10.05	omics Pos	0.66378	0.99987	0.97384	-0.03824	0.16047	0.99994	0.93428	-0.09808	0.90107	0.99996	0.99515	-0.00701	0.4134	0.85524	0.91859	-0.12251	0.001587	0.007631	0.72163	-0.47066	0.54649	0.74794	0.94358	-0.08378	0.29151	0.71805	1.11	0.15051	0.75136	0.97988	1.0243	0.034573	0.17528	0.28296	1.2597	0.33307
490.3309	2.72	omics Pos	0.66406	0.99987	1.0573	0.080427	0.43731	0.99994	0.84187	-0.24834	0.072643	0.99996	0.68898	-0.53748	0.039633	0.34581	0.57213	-0.8056	0.008225	0.026489	0.41993	-1.2518	0.010115	0.08787	0.37606	-1.411	0.042348	0.38314	0.54172	-0.88439	0.016373	0.52955	0.45633	-1.1319	0.19749	0.30585	0.68172	-0.55274
424.2554	7.49	olomics Po	0.66461	0.99951	0.93439	-0.0979	0.68983	0.99998	0.93957	-0.08993	0.058164	0.99941	0.76341	-0.38947	0.02771	0.24036	0.60941	-0.71451	0.019336	0.081974	0.63427	-0.65683	0.006133	0.065713	0.46369	-1.1088	0.004637	0.20152	0.52269	-0.93596	0.004164	0.47452	0.47417	-1.0765	0.01872	0.2688	0.55406	-0.85189
626.8461	0.54	omics Pos	0.66516	0.99987	0.97058	-0.04308	0.086399	0.99994	0.86982	-0.20121	0.050451	0.99996	0.83081	-0.26742	0.008149	0.19917	0.79501	-0.33095	0.001908	0.008766	0.67076	-0.57612	0.20535	0.49558	0.84488	-0.24318	0.22294	0.66569	0.90036	-0.15142	0.21231	0.93052	0.87173	-0.19805	0.22868	0.33563	1.2004	0.26352
813.8363	10.05	omics Pos	0.66563	0.99987	1.0302	0.042953	0.22668	0.99994	0.91677	-0.12537	0.19989	0.99996	0.8926	-0.16391	0.12098	0.54142	0.87498	-0.19268	0.001235	0.006406	0.73601	-0.44221	0.49502	0.7126	0.94723	-0.07821	0.63452	0.8822	0.96599	-0.04992	0.074956	0.77877	0.88041	-0.18375	0.26607	0.37616	1.1155	0.15764
418.3318	6.04	omics Pos	0.6661	0.99987	1.0339	0.048085	0.1527	0.99994	0.81404	-0.29682	0.88326	0.99996	0.99301	-0.01012	0.072833	0.45752	0.82503	-0.27748	0.006964	0.023272	0.78466	-0.34987	0.13375	0.40042	0.79099	-0.33827	0.96942	0.99319	1.0091	0.013058	0.33702	0.96524	0.88078	-0.18314	0.68663	0.76688	1.0797	0.11063
658.6103	6.65	omics Pos	0.66629	0.99987	1.0925	0.12768	0.94412	0.99994	1.0006	0.0009	0.46583	0.99996	1.2088	0.27353	0.14141	0.56936	0.76294	-0.39036	0.19993	0.29355	0.797	-0.32735	0.052334	0.22542	0.73425	-0.44566	0.43116	0.79133	0.89833	-0.15469	0.015831	0.52955	0.63744	-0.64964	0.46753	0.57045	0.92284	-0.11584
635.5412	5.56	omics Pos	0.66656	0.99987	1.0433	0.061115	0.92645	0.99994	0.99473	-0.00762	0.30974	0.99996	0.89606	-0.15833	0.53323	0.90063	0.92868	-0.10675	0.04242	0.092215	0.78082	-0.35694	0.73046	0.85884	0.96563	-0.05046	0.4282	0.79094	1.1446	0.19481	0.5919	0.97073	0.94702	-0.07853	0.32893	0.43869	1.1272	0.17275
829.5534	8.58	olomics Po	0.66662	0.99951	0.98289	-0.0249	0.95744	0.99998	1.0152	0.021785	0.61546	0.99941	0.98149	-0.02696	0.61484	0.99905	1.0279	0.03975	0.20104	0.4965	1.0319	0.045318	0.58069	0.85728	1.0455	0.064131	0.67573	0.99391	1.0213	0.030338	0.036278	0.61553	0.91371	-0.13018	0.65515	0.8417	0.98223	-0.02587
956.7619	10.06	omics Pos	0.66701	0.99987	1.0191	0.027268	0.10496	0.99994	0.87353	-0.19507	0.1245	0.99996	0.8991	-0.15345	0.11869	0.53907	0.90522	-0.14365	0.00149	0.007308	0.77158	-0.37411	0.32505	0.60075	0.88595	-0.1747	0.56171	0.855	1.0493	0.069398	0.3275	0.95887	0.91724	-0.12462	0.080249	0.18194	1.1361	0.18403
929.7511	10.46	olomics Po	0.66768	0.99951	0.94888	-0.07571	0.48038	0.99998	0.87337	-0.19534	0.25947	0.99941	0.76531	-0.38588	0.41716	0.90805	0.86563	-0.20817	0.54975	0.93605	0.89261	-0.1639	0.28778	0.58372	0.77073	-0.3757	0.17363	0.74875	0.73028	-0.45348	0.024084	0.51963	0.49246	-1.0219	0.062872	0.30404	0.61078	-0.71128
432.8998	10.02	omics Pos	0.66782	0.99987	1.0319	0.045353	0.23174	0.99994	0.90341	-0.14654	0.20731	0.99996	0.86202	-0.21421	0.086523	0.49062	0.87999	-0.18444	0.005083	0.018453	0.77232	-0.37273	0.15458	0.4337	0.81647	-0.29253	0.41316	0.78028	0.93604	-0.09536	0.19496	0.92582	0.86699	-0.20591	0.15169	0.25717	1.2224	0.28977
940.819	10.02	omics Pos	0.66784	0.99987	0.97878	-0.03094	0.097596	0.99994	0.87018	-0.20062	0.25753	0.99996	0.93771	-0.09279	0.035951	0.33474	0.89461	-0.16067	0.000102	0.001038	0.72218	-0.46957	0.33072	0.60481	0.90987	-0.13628	0.55114	0.85184	0.95765	-0.06244	0.2624	0.94365	0.93654	-0.09458	0.037167	0.13049	1.176	0.23391
169.9873	0.6	omics Pos	0.66784	0.99987	1.0981	0.13501	0.88158	0.99994	0.97966	-0.02965	0.83004	0.99996	1.0476	0.0671	0.70664	0.96282	0.92597	-0.11096	0.001539	0.007456	0.41284	-1.2763	0.19419	0.4805	0.72741	-0.45917	0.78745	0.95047	1.0615	0.086073	0.43673	0.96776	0.84885	-0.23641	0.28814	0.39833	1.1967	0.25906
844.5473	4.73	omics Pos	0.66842	0.99987	1.0299	0.042472	0.52427	0.99994	0.94649	-0.07934	0.35922	0.99996	0.91864	-0.12243	0.49332	0.88874	0.93698	-0.09391	0.038152	0.085322	0.78013	-0.35821	0.6465	0.81508	0.95521	-0.06612	0.54983	0.85184	1.0384	0.0543	0.41417	0.96776	1.0752	0.10455	0.2738	0.38255	1.1249	0.16982
825.5473	8.39	olomics Ne	0.66879	0.99918	1.0322	0.045755	0.42801	0.80889	0.94552	-0.08082	0.85773	0.99995	1.0152	0.021808	0.82745	0.99985	0.98677	-0.01921	0.2523	0.99927	0.93793	-0.09244	0.19578	0.65203	1.0828	0.11472	0.862	0.95689	0.98994	-0.01458	0.009366	0.99512	0.84547	-0.24218	0.012694	0.99941	0.79002	-0.34004
765.9694	6.5	olomics Po	0.66902	0.99951	1.1561	0.20922	0.56576	0.99998	0.91212	-0.13271	0.45771	0.99941	1.0877	0.12129	0.033976	0.25399	0.732	-0.45009	0.62733	0.97826	0.92973	-0.10512	0.14029	0.37931	0.82216	-0.28251	0.80985	0.99391	1.0659	0.09204	0.90253	0.99956	1.0864	0.11954	0.48837	0.74426	1.1628	0.21762
281.2404	2.84	lomics Neg	0.66923	0.99977	0.93433	-0.09799	0.51305	0.99904	0.89761	-0.15583	0.026216	0.99994	0.53262	-0.90882	0.042948	0.58182	0.44361	-1.1726	0.001672	0.066804	0.33575	-1.5745	0.014803	0.46473	0.22941	-2.124	0.002171	0.093659	0.18121	-2.4643	0.011947	0.46971	0.1912	-2.3868	0.01219	0.38536	0.30245	-1.7252
794.8043	10.08	omics Pos	0.67013	0.99987	0.96815	-0.04669	0.94926	0.99994	1.0064	0.009205	0.67088	0.99996	0.96027	-0.05849	0.54604	0.90473	0.94478	-0.08196	0.012149	0.035552	0.74089	-0.43267	0.50999	0.72221	0.93303	-0.1	0.88994	0.97532	0.98907	-0.01586	0.62804	0.97904	1.0701	0.097691	0.095602	0.19715	1.2604	0.3339
627.5351	5.46	omics Pos	0.67136	0.99987	0.95313	-0.06926	0.14905	0.99994	0.78638	-0.3467	0.077453	0.99996	0.79961	-0.32262	0.02633	0.28766	0.73088	-0.4523	0.001922	0.008795	0.59654	-0.74532	0.005714	0.076133	0.66289	-0.59315	0.22638	0.66988	0.84975	-0.23489	0.8819	0.98023	0.99601	-0.00576	0.56345	0.65903	0.92492	-0.1126
1199.754	10.02	omics Pos	0.6716	0.99987	1.038	0.053837	0.84047	0.99994	0.97281	-0.03977	0.99277	0.99996	0.9928	-0.01042	0.97313	0.99502	0.99741	-0.00374	0.044506	0.095499	0.78582	-0.34773	0.92637	0.96224	0.99227	-0.0112	0.58992	0.86163	1.0605	0.084771	0.59159	0.97073	1.0629	0.087948	0.053273	0.15013	1.3587	0.4422
928.5988	9.5	olomics Po	0.67227	0.99951	0.94497	-0.08165	0.10593	0.99998	0.80208	-0.31818	0.44505	0.99941	0.89802	-0.15518	0.95615	0.99905	0.9951	-0.00709	0.16019	0.42121	0.8212	-0.28419	0.24763	0.53907	0.80648	-0.3103	0.18545	0.76289	0.78289	-0.35311	0.046411	0.66755	0.71167	-0.49073	0.095436	0.34969	0.77157	-0.37413
403.7897	8.57	olomics Po	0.67229	0.99951	0.96627	-0.04951	0.91023	0.99998	0.99672	-0.00475	0.22719	0.99941	0.89128	-0.16605	0.026986	0.24036	0.84062	-0.25048	0.18074	0.46212	1.1301	0.17647	0.90491	0.97054	1.0578	0.081134	0.13636	0.68944	0.90104	-0.15033	0.013557	0.47452	0.82495	-0.27763	0.68035	0.85485	0.96557	-0.05055
194.1176	10.12	omics Pos	0.6725	0.99987	1.0378	0.053468	0.76442	0.99994	0.98161	-0.02677	0.67976	0.99996	1.0154	0.022052	0.62101	0.94062	0.96538	-0.05083	0.009628	0.029943	0.75539	-0.4047	0.66922	0.82743	0.96924	-0.04507	0.23575	0.67693	1.0616	0.086203	0.21527	0.93732	1.0906	0.12515	0.050445	0.14715	1.2461	0.31748
921.7957	10.08	omics Pos	0.6739	0.99987	1.0627	0.08769	0.012834	0.99994	0.87931	-0.18556	0.68176	0.99996	1.0274	0.038992	0.8312	0.98621	0.99223	-0.01126	0.12856	0.2103	0.83119	-0.26674	0.66546	0.82571	1.053	0.074449	0.77414	0.94464	1.0698	0.097328	0.32835	0.95887	1.1036	0.14215	0.003682	0.085302	1.2355	0.30511
371.9806	12.01	olomics Ne	0.6741	0.99918	1.0688	0.095935	0.005154	0.34605	0.78464	-0.3499	0.41181	0.99995	1.0873	0.12077	0.55007	0.99985	0.93838	-0.09175	0.36692	0.99927	1.1407	0.18989	0.39592	0.79378	1.153	0.20535	0.096554	0.46226	1.2106	0.27567	0.44293	0.99512	0.9307	-0.10361	0.62162	0.99941	0.95281	-0.06975
162.8561	12.27	olomics Ne	0.67412	0.99918	1.0136	0.019495	0.042654	0.46882	0.90953	-0.13681	0.27453	0.99995	1.0533	0.07491	0.96035	0.99985	1.0014	0.00204	0.89717	0.99927	1.0094	0.013547	0.22176	0.66171	1.0943	0.12996	0.006892	0.2539	1.1207	0.16439	0.81854	0.99512	1.0145	0.020744	0.39717	0.99941	1.0293	0.041709
86.9936	10.16	omics Pos	0.67458	0.99987	1.0416	0.058858	0.6397	0.99994	0.94773	-0.07745	0.85105	0.99996	0.98192	-0.02632	0.67842	0.95843	1.0328	0.046622	0.026833	0.065269	0.7294	-0.45521	0.68556	0.83692	0.9496	-0.0746	0.43786	0.79731	1.0883	0.12209	0.79449	0.97988	1.0293	0.041707	0.036319	0.12924	1.31	0.38957
1067.501	5.92	omics Pos	0.67463	0.99987	1.0521	0.073257	0.97716	0.99994	0.99899	-0.00146	0.81479	0.99996	1.022	0.031354	0.87269	0.9874	0.9779	-0.03225	0.029554	0.070125	0.76063	-0.39473	0.28561	0.57196	0.8405	-0.25069	0.4051	0.77738	0.90092	-0.15053	0.57269	0.9703	0.92842	-0.10716	0.21635	0.32497	1.1454	0.1959
405.2789	0.89	omics Pos	0.6747	0.99987	0.80143	-0.31935	0.56057	0.99994	0.68546	-0.54486	0.50662	0.99996	0.73389	-0.44637	0.78374	0.97526	1.3644	0.44831	0.71458	0.75707	4.0063	2.0023	0.014507	0.10138	11.267	3.494	0.000231	0.15466	16.738	4.065	0.041489	0.64308	4.1903	2.067	0.027696	0.11669	2.7635	1.4665
423.2517	3.52	omics Pos	0.6748	0.99987	0.93484	-0.09721	0.18499	0.99994	0.7498	-0.41542	0.19711	0.99996	0.78598	-0.34744	0.001776	0.17581	0.50074	-0.99787	0.002087	0.009351	0.43515	-1.2004	0.008959	0.08583	0.46332	-1.1099	0.011945	0.3324	0.55436	-0.8511	0.022967	0.54215	0.57414	-0.80053	0.061049	0.15806	0.58324	-0.77784
1169.746	10.02	omics Pos	0.67496	0.99987	0.9727	-0.03994	0.28354	0.99994	0.90486	-0.14424	0.52383	0.99996	0.95892	-0.06051	0.5863	0.92519	0.95039	-0.0734	0.007503	0.024664	0.74389	-0.42684	0.19877	0.48694	0.863	-0.21256	0.8996	0.97738	0.99615	-0.00556	0.98157	0.99461	0.99825	-0.00252	0.25723	0.36653	1.0879	0.12161
793.559	5.34	omics Pos	0.67517	0.99987	1.0281	0.040042	0.17962	0.99994	0.89799	-0.15524	0.25029	0.99996	0.8809	-0.18295	0.060447	0.42447	0.809	-0.3058	0.002425	0.010436	0.62134	-0.68655	0.001527	0.051084	0.65601	-0.60821	0.76456	0.94254	0.9729	-0.03963	0.39173	0.96776	1.0832	0.11533	0.20253	0.31141	1.1695	0.22584
353.2433	2.29	omics Pos	0.67652	0.99987	0.96871	-0.04587	0.29293	0.99994	0.81425	-0.29645	0.021973	0.99996	0.61193	-0.70857	0.00232	0.17581	0.4976	-1.007	0.000537	0.003479	0.31583	-1.6628	0.001243	0.044487	0.29999	-1.737	0.024532	0.33809	0.47887	-1.0623	0.021673	0.53012	0.46371	-1.1087	0.010026	0.094875	0.51893	-0.94639
112.018	10.23	omics Pos	0.67655	0.99987	1.0252	0.035906	0.20406	0.99994	0.90122	-0.15005	0.57997	0.99996	0.95842	-0.06128	0.25732	0.71521	0.91694	-0.12511	0.004311	0.01621	0.73624	-0.44175	0.41138	0.65457	0.92424	-0.11366	0.58126	0.86163	1.0552	0.077454	0.82127	0.97988	0.98013	-0.02895	0.055228	0.15227	1.2318	0.3008
357.1812	6.84	olomics Ne	0.67667	0.99918	1.09	0.12439	0.33715	0.75268	0.84081	-0.25014	0.24067	0.99995	0.84028	-0.25106	0.021352	0.99985	0.63449	-0.65633	0.009354	0.71966	0.65613	-0.60795	0.005313	0.36417	0.52428	-0.93159	0.003525	0.22316	0.51908	-0.94597	0.004576	0.99512	0.52648	-0.92556	0.03738	0.99941	0.569	-0.8135
585.3413	5.72	olomics Po	0.67673	0.99951	0.93911	-0.09063	0.26208	0.99998	0.85208	-0.23094	0.80744	0.99941	1.0531	0.074575	0.032064	0.24911	0.65089	-0.61952	0.026238	0.10396	0.57161	-0.80689	0.029747	0.13216	0.64964	-0.6223	0.9051	0.9951	1.0021	0.003076	0.12048	0.8972	0.70822	-0.49774	0.17802	0.44154	1.1782	0.23653
906.6058	5.78	omics Pos	0.67675	0.99987	1.049	0.069031	0.93157	0.99994	1.0064	0.009275	0.64682	0.99996	0.94275	-0.08505	0.35748	0.81247	0.8831	-0.17935	0.034984	0.079543	0.73995	-0.43449	0.073543	0.28014	0.79857	-0.32452	0.14474	0.60716	0.81614	-0.29312	0.10865	0.86492	0.81314	-0.29842	0.41204	0.51938	1.1168	0.15941
280.0929	0.58	omics Pos	0.67684	0.99987	1.0466	0.065724	0.24896	0.99994	0.81134	-0.30162	0.17583	0.99996	0.83601	-0.25841	0.13972	0.56566	0.80412	-0.31451	0.000647	0.003963	0.40372	-1.3086	0.13256	0.3983	0.74846	-0.418	0.61687	0.87206	1.0737	0.10253	0.51116	0.96776	0.91367	-0.13025	0.006527	0.090084	1.8446	0.88334
1137.503	6.13	omics Pos	0.67691	0.99987	1.0382	0.054022	0.51732	0.99994	1.077	0.10703	0.64686	0.99996	1.0577	0.080904	0.72586	0.96875	0.9423	-0.08575	0.11431	0.19287	0.7707	-0.37577	0.030192	0.15536	0.68108	-0.55411	0.068311	0.46847	0.75767	-0.40036	0.16687	0.91806	0.8086	-0.3065	0.80222	0.85324	1.0349	0.049425
798.5634	5.56	omics Pos	0.67717	0.99987	1.0341	0.048345	0.40297	0.99994	0.93406	-0.09841	0.56468	0.99996	0.95288	-0.06963	0.33521	0.79326	0.92176	-0.11753	0.020604	0.053591	0.77943	-0.3595	0.457	0.68604	0.94444	-0.08248	0.6139	0.87115	1.046	0.064841	0.8135	0.97988	1.0121	0.017353	0.15364	0.25945	1.1081	0.14813
223.1699	6.06	olomics Po	0.67748	0.99951	0.94737	-0.078	0.46612	0.99998	0.86653	-0.20668	0.011716	0.99941	0.48795	-1.0352	0.01339	0.20085	0.38437	-1.3794	0.001444	0.012324	0.3448	-1.5362	0.010042	0.079508	0.37081	-1.4312	0.005606	0.21022	0.35099	-1.5105	0.008134	0.47452	0.27694	-1.8524	0.0238	0.2688	0.36761	-1.4438
213.1905	2.82	omics Pos	0.67775	0.99987	0.91522	-0.1278	0.28648	0.99994	0.80791	-0.30772	0.1105	0.99996	0.73658	-0.44108	0.084276	0.48766	0.6269	-0.67369	0.000577	0.003673	0.29181	-1.7769	0.007925	0.084185	0.39768	-1.3303	0.006265	0.3088	0.42293	-1.2415	0.023192	0.54215	0.54842	-0.86664	0.49443	0.59679	0.86256	-0.2133
802.9734	12.01	olomics Ne	0.67798	0.99918	1.0618	0.0865	0.15767	0.5837	0.87008	-0.20078	0.044966	0.99995	1.2536	0.32608	0.73556	0.99985	0.96794	-0.04702	0.93934	0.99927	1.0061	0.008777	0.97814	0.9943	0.99737	-0.0038	0.25049	0.63479	1.2727	0.3479	0.95859	0.99512	1.0038	0.005518	0.48722	0.99941	0.93539	-0.09637
402.2326	4.75	olomics Po	0.6783	0.99951	0.96396	-0.05296	0.8312	0.99998	1.0273	0.038906	0.9137	0.99941	0.99733	-0.00385	0.62651	0.99905	1.0469	0.066088	0.000847	0.008283	0.50164	-0.99527	0.14199	0.38158	0.83796	-0.25505	0.15348	0.72748	0.87377	-0.19468	0.90954	0.99956	1.0144	0.020562	0.36003	0.63929	1.1441	0.19427
130.0525	0.61	omics Pos	0.67849	0.99987	1.0315	0.044752	0.104	0.99994	0.90065	-0.15096	0.63415	0.99996	1.063	0.088135	0.22466	0.68033	1.1814	0.24049	0.42858	0.51405	0.92014	-0.12007	0.8803	0.93786	1.0219	0.031224	0.013572	0.33809	1.3664	0.45039	0.38939	0.96776	1.1087	0.14888	0.02163	0.1083	1.3794	0.46408
853.5767	4.97	lomics Neg	0.67884	0.99977	1.2216	0.28879	0.88655	0.99904	1.1034	0.14194	0.4299	0.99994	0.91489	-0.12834	0.15949	0.58182	1.4294	0.51536	0.96515	0.99989	1.195	0.25703	0.98393	0.99653	1.1949	0.25695	0.096975	0.4201	0.71852	-0.47689	0.038767	0.46971	0.54036	-0.888	0.19706	0.42853	0.90244	-0.1481
755.4863	4.09	omics Pos	0.67885	0.99987	0.96126	-0.057	0.98037	0.99994	1.0029	0.004202	0.88936	0.99996	1.0176	0.0252	0.29777	0.75592	0.91388	-0.12992	0.024627	0.060976	0.72234	-0.46925	0.28991	0.57347	0.86396	-0.21096	0.73226	0.92699	0.97364	-0.03854	0.83996	0.97988	0.99567	-0.00626	0.13629	0.24137	1.2905	0.36796
200.0955	10.25	omics Pos	0.67912	0.99987	1.0317	0.044958	0.080942	0.99994	0.79585	-0.32944	0.35667	0.99996	0.89415	-0.16141	0.55313	0.9087	0.92799	-0.10782	0.038565	0.086117	0.7186	-0.47674	0.3175	0.5964	0.85209	-0.23092	0.96825	0.99304	0.98753	-0.0181	0.58443	0.97073	1.0581	0.081534	0.59433	0.68734	1.1059	0.14526
854.8116	10.07	omics Pos	0.67926	0.99987	0.94648	-0.07935	0.65189	0.99994	0.93974	-0.08967	0.70634	0.99996	0.94918	-0.07525	0.8162	0.9833	0.9662	-0.0496	0.045408	0.096968	0.76822	-0.3804	0.41955	0.65671	0.892	-0.16489	0.9955	0.99769	0.9874	-0.0183	0.59859	0.97073	0.93448	-0.09776	0.10949	0.213	1.2163	0.28252
844.6171	5.62	omics Pos	0.6794	0.99987	1.0389	0.055108	0.64373	0.99994	0.94514	-0.0814	0.92078	0.99996	0.98237	-0.02567	0.73746	0.97097	0.95742	-0.06277	0.0478	0.10081	0.77567	-0.36648	0.46361	0.68955	0.90976	-0.13644	0.95604	0.98985	0.9867	-0.01932	0.52559	0.96776	0.92919	-0.10596	0.82493	0.87125	0.96936	-0.0449
855.7967	0.52	omics Pos	0.6804	0.99987	1.0997	0.1371	0.32079	0.99994	0.88697	-0.17304	0.25327	0.99996	0.92116	-0.11848	0.41787	0.85533	0.92075	-0.11912	0.04167	0.090845	0.80491	-0.3131	0.61296	0.7912	0.95397	-0.06798	0.098567	0.53521	1.1237	0.1683	0.80109	0.97988	1.0156	0.022373	0.042034	0.1364	1.2016	0.26498
529.3142	1.29	omics Pos	0.68067	0.99987	1.0438	0.061849	0.199	0.99994	0.84468	-0.24352	0.97476	0.99996	1.0141	0.02015	0.80515	0.97865	0.97552	-0.03576	0.009755	0.030216	0.7199	-0.47413	0.10371	0.34299	1.2487	0.32046	0.066556	0.46141	1.5507	0.63295	0.1571	0.9146	1.1406	0.18983	0.013326	0.098791	1.6144	0.691
939.1272	8.9	olomics Po	0.68078	0.99951	1.1931	0.25472	0.48384	0.99998	0.89453	-0.1608	0.77051	0.99941	1.0521	0.073224	0.018994	0.21385	0.71166	-0.49074	0.047247	0.16124	0.6485	-0.62482	0.096529	0.29209	0.79767	-0.32614	0.92215	0.9951	1.0458	0.064641	0.73346	0.99956	0.96158	-0.05653	0.10724	0.36185	1.7232	0.78509
96.0895	6.38	olomics Po	0.68092	0.99951	1.054	0.075819	0.15186	0.99998	0.86197	-0.21429	0.5137	0.99941	0.93491	-0.09709	0.19129	0.62312	0.81356	-0.29768	4.11E-06	0.000231	0.34719	-1.5262	0.008737	0.075866	0.56673	-0.81928	0.076254	0.56471	0.69973	-0.51514	0.16789	0.95629	0.80309	-0.31637	0.9717	0.9861	1.0075	0.010718
713.5445	4.39	omics Pos	0.68145	0.99987	1.0942	0.1299	0.45923	0.99994	0.90476	-0.1444	0.99634	0.99996	1.0041	0.005896	0.78061	0.97526	0.96706	-0.04832	0.048929	0.10262	0.75484	-0.40577	0.61116	0.78997	0.94214	-0.08599	0.83984	0.96659	0.97333	-0.039	0.55016	0.96776	0.91454	-0.12888	0.50489	0.60616	1.1196	0.16301
648.2802	1.03	omics Pos	0.68149	0.99987	0.94584	-0.08033	0.69255	0.99994	1.0525	0.073856	0.29362	0.99996	1.1749	0.23253	0.80071	0.97856	1.0347	0.049278	0.26758	0.36232	0.8151	-0.29496	0.10072	0.33837	1.4393	0.52534	0.030129	0.35536	1.7402	0.79923	0.47477	0.96776	1.1585	0.21228	0.089199	0.19001	1.361	0.44471
763.5932	5.27	omics Pos	0.68159	0.99987	1.0486	0.068426	0.80165	0.99994	0.96891	-0.04557	0.76286	0.99996	0.96214	-0.05569	0.42033	0.8557	0.89253	-0.16403	0.048689	0.10234	0.75454	-0.40634	0.3218	0.5977	0.87684	-0.18961	0.77822	0.94624	0.96682	-0.04868	0.51411	0.96776	1.0963	0.13261	0.055453	0.15256	1.7723	0.82563
964.0826	6.47	olomics Po	0.68164	0.99951	1.1204	0.16403	0.44012	0.99998	0.83036	-0.26819	0.84275	0.99941	1.017	0.024265	0.047602	0.28918	0.64923	-0.62321	0.20112	0.4965	0.70365	-0.50708	0.08568	0.26905	0.67804	-0.56056	0.46025	0.99391	0.83988	-0.25175	0.4643	0.99956	0.8514	-0.23209	0.78547	0.90664	0.94067	-0.08824
1091.503	5.83	omics Pos	0.68179	0.99987	0.95601	-0.06491	0.86642	0.99994	0.98358	-0.02388	0.47402	0.99996	0.92705	-0.10929	0.79544	0.97682	0.97299	-0.03951	0.019356	0.050865	0.77174	-0.37382	0.36503	0.62558	0.89316	-0.16301	0.78555	0.94968	0.97147	-0.04176	0.92432	0.98531	1.0222	0.031665	0.14131	0.24608	1.2903	0.36766
271.6589	1.12	omics Pos	0.68195	0.99987	1.1467	0.19746	0.993	0.99994	0.96977	-0.04428	0.73222	0.99996	1.0892	0.12331	0.43967	0.86677	1.1974	0.25995	0.55039	0.61634	1.1435	0.19343	0.035846	0.17185	1.8897	0.91814	0.019746	0.33809	2.1329	1.0928	0.16167	0.91806	1.4222	0.50808	0.059846	0.15736	1.9371	0.95393
743.4638	1.52	omics Pos	0.68211	0.99987	1.2407	0.31111	0.25014	0.99994	0.80438	-0.31405	0.7083	0.99996	1.0863	0.11942	0.03018	0.30328	0.61434	-0.70289	0.020138	0.052558	0.59657	-0.74524	0.074009	0.28051	0.71415	-0.4857	0.89271	0.97532	1.067	0.093553	0.70469	0.97988	1.2139	0.27962	0.14468	0.24917	1.6595	0.73076
449.3473	8.37	olomics Po	0.68332	0.99951	0.96494	-0.05149	0.82179	0.99998	0.97974	-0.02952	0.15582	0.99941	0.87063	-0.19987	0.41915	0.90805	0.92948	-0.1055	0.97487	0.9989	1.0012	0.001757	0.45288	0.75643	0.92656	-0.11005	0.70976	0.99391	0.96342	-0.05376	0.022423	0.51556	0.79109	-0.33809	0.14274	0.39464	0.83191	-0.26551
629.4147	7.87	olomics Po	0.68333	0.99951	1.0972	0.1338	0.84833	0.99998	1.0567	0.079544	0.29451	0.99941	1.1789	0.2374	0.64596	0.99905	1.1708	0.22749	0.04772	0.16251	1.6038	0.68146	0.25876	0.54892	1.3047	0.38369	0.15231	0.72698	1.3058	0.38491	0.87418	0.99956	1.0894	0.12347	0.48042	0.73883	1.136	0.18391

576.5072	5.6	domics Pos	0.68362	0.99987	1.1049	0.14388	0.72991	0.99994	1.0553	0.077628	0.95135	0.99996	0.98074	-0.02806	0.84393	0.98707	0.91414	-0.12951	0.7001	0.74483	1.0959	0.13211	0.14802	0.42309	1.4809	0.56652	0.046066	0.39255	1.7342	0.79428	0.04423	0.65325	1.7193	0.78186	0.013105	0.098791	2.299	1.201
411.2435	2.72	domics Pos	0.68379	0.99987	0.95797	-0.06195	0.17284	0.99994	0.82156	-0.28356	0.013057	0.99996	0.70963	-0.49485	0.002748	0.17581	0.54985	-0.86289	0.000223	0.001814	0.44479	-1.1688	0.007414	0.082061	0.38308	-1.3843	0.016529	0.33809	0.47011	-1.0889	0.011955	0.52955	0.49661	-1.0098	0.053593	0.15039	0.6103	-0.71241
414.7717	4.78	domics Pos	0.68405	0.99987	0.9089	-0.13781	0.11182	0.99994	0.68279	-0.55049	0.037439	0.99996	0.61704	-0.69657	0.084196	0.48766	0.67496	-0.56712	0.4861	0.56304	0.84508	-0.24285	0.053648	0.2283	0.59492	-0.74923	0.047512	0.39725	0.6234	-0.68176	0.044112	0.65325	0.62099	-0.68737	0.1315	0.23654	0.72055	-0.47282
197.1537	6.48	olomics Pc	0.68407	0.99951	0.936	-0.09541	0.54809	0.99998	0.91332	-0.13081	0.046206	0.99941	0.62617	-0.67536	0.059491	0.32144	0.5567	-0.84503	0.003029	0.020784	0.4489	-1.1555	0.007522	0.071961	0.40948	-1.2881	0.022257	0.31703	0.419	-1.255	0.009578	0.47452	0.37684	-1.408	0.018204	0.2688	0.39379	-1.3445
578.8402	10.14	domics Pos	0.68419	0.99987	1.0531	0.074646	0.7552	0.99994	0.97266	-0.03999	0.28777	0.99996	0.92348	-0.11485	0.93719	0.99037	0.99211	-0.01143	0.014676	0.041269	0.80454	-0.31376	0.69002	0.83974	0.96543	-0.05076	0.75151	0.93661	1.0462	0.065125	0.44993	0.96776	0.94495	-0.08169	0.073629	0.17569	1.1528	0.20517
726.5596	4.66	domics Pos	0.68451	0.99987	1.0352	0.049841	0.46546	0.99994	0.91115	-0.13424	0.78226	0.99996	0.97075	-0.04282	0.62736	0.94476	0.95024	-0.07364	0.039015	0.086844	0.77234	-0.37269	0.31024	0.58859	0.84189	-0.2483	0.54745	0.85166	0.93646	-0.09471	0.25296	0.94365	0.8783	-0.18721	0.82091	0.86852	0.97508	-0.03641
530.9744	5.25	olomics Ne	0.68454	0.99918	1.0286	0.040741	0.075199	0.50118	0.88659	-0.17366	0.41571	0.99995	1.0453	0.063967	0.93439	0.99985	1.0004	0.000567	0.84008	0.99927	0.98867	-0.01644	0.039847	0.48405	1.1378	0.18628	0.69275	0.89955	0.97791	-0.03223	0.8843	0.99512	1.0179	0.025553	0.78451	0.99941	0.9851	-0.02166
414.7582	2.99	olomics Pc	0.68457	0.99951	1.1548	0.20769	0.024189	0.99998	0.73295	-0.44822	0.38612	0.99941	0.92239	-0.11656	0.001241	0.13475	0.51817	-0.94851	0.000265	0.003536	0.446	-1.1649	0.000418	0.039572	0.56621	-0.8206	0.63012	0.99391	0.96256	-0.05505	0.5722	0.99956	0.93136	-0.10259	0.091536	0.34441	1.7836	0.83476
1193.448	5.92	domics Pos	0.68512	0.99987	0.94791	-0.07718	0.28354	0.99994	0.86889	-0.20275	0.34446	0.99996	0.89083	-0.16678	0.10368	0.52894	0.80534	-0.31233	0.008644	0.027398	0.6578	-0.60428	0.051612	0.22338	0.73893	-0.43649	0.16524	0.63453	0.77248	-0.37243	0.42842	0.96776	0.89655	-0.15754	0.2503	0.3592	0.85858	-0.21998
244.079	0.59	domics Pos	0.68522	0.99987	1.133	0.18013	0.95688	0.99994	1.064	0.089518	0.99618	0.99996	1.0206	0.029436	0.76928	0.97526	0.91257	-0.13199	0.009359	0.029351	0.38176	-1.3892	0.3728	0.63038	0.75181	-0.41157	0.64136	0.8857	0.86225	-0.21382	0.84056	0.97988	0.97072	-0.04288	0.53401	0.63137	1.2953	0.37328
287.224	6.41	olomics Pc	0.68528	0.99951	0.90044	-0.1513	0.97761	0.99998	0.97083	-0.04271	0.38912	0.99941	0.80844	-0.30678	0.65064	0.99905	0.88637	-0.17401	7.39E-05	0.001279	0.17918	-2.4805	0.038958	0.15763	0.59361	-0.75242	0.16722	0.7434	0.69546	-0.52397	0.24151	0.99956	0.72698	-0.46001	0.19564	0.45921	0.69197	-0.53121
309.2791	1.45	domics Pos	0.68562	0.99987	1.3561	0.43945	0.18961	0.99994	0.77289	-0.37167	0.64641	0.99996	1.1081	0.14808	0.10025	0.52019	0.69058	-0.53412	0.026283	0.064222	0.61099	-0.71079	0.05601	0.23443	0.70464	-0.50504	0.86317	0.97404	1.0744	0.10359	0.76582	0.97988	1.1847	0.24448	0.21846	0.32641	1.4432	0.52925
385.2785	5.17	domics Pos	0.68588	0.99987	0.97128	-0.04204	0.1694	0.99994	0.91091	-0.13462	0.66213	0.99996	0.97628	-0.03463	0.47581	0.88482	0.94075	-0.08811	0.001275	0.006577	0.72824	-0.45751	0.48808	0.70629	0.91158	-0.13356	0.37902	0.76056	0.94976	-0.07437	0.13993	0.89982	0.89015	-0.16789	0.029501	0.11849	0.84028	-0.25105
557.8642	10.06	domics Pos	0.68614	0.99987	1.0298	0.04232	0.059043	0.99994	0.8419	-0.24829	0.77663	0.99996	0.98219	-0.02593	0.02185	0.26563	0.82535	-0.27693	0.001162	0.006117	0.73797	-0.43837	0.38707	0.63975	0.93499	-0.09697	0.38342	0.76429	1.0595	0.083357	0.214	0.93471	0.92877	-0.10661	0.11169	0.2152	1.155	0.20788
1093.222	9.04	olomics Pc	0.68626	0.99951	1.1786	0.23705	0.1954	0.99998	0.81779	-0.2902	0.7915	0.99941	0.97906	-0.03052	0.009395	0.19702	0.65009	-0.62129	0.038137	0.13698	0.62437	-0.67953	0.045355	0.17359	0.74275	-0.42904	0.85352	0.9951	1.0344	0.048783	0.69955	0.99956	1.1165	0.15898	0.033601	0.2688	1.5342	0.61747
351.2304	2.04	domics Pos	0.68629	0.99987	0.96809	-0.04678	0.46225	0.99994	0.88607	-0.17451	0.020545	0.99996	0.63526	-0.65459	0.011387	0.21625	0.47857	-1.0632	0.017116	0.046219	0.59789	-0.74204	0.00266	0.061655	0.38892	-1.3624	0.057062	0.42773	0.51431	-0.95929	0.006409	0.52955	0.47834	-1.0639	0.005874	0.089361	0.48308	-1.0497
587.5431	9.61	olomics Pc	0.68636	0.99951	1.0771	0.10716	0.73373	0.99998	1.0612	0.085644	0.38007	0.99941	0.92718	-0.10908	0.44038	0.92243	1.3437	0.42624	0.11375	0.31907	1.1687	0.22491	0.028388	0.1276	1.249	0.32078	0.078252	0.57191	1.6991	0.76478	0.395	0.99956	1.4398	0.52585	0.25329	0.52319	1.2831	0.35963
859.5309	5.9	domics Pos	0.68655	0.99987	1.1281	0.17396	0.91652	0.99994	0.92756	-0.10849	0.94308	0.99996	0.98179	-0.02651	0.97227	0.99502	0.98919	-0.01569	0.69257	0.73862	1.068	0.094937	0.022965	0.13288	1.9608	0.97141	0.027327	0.34512	2.0423	1.0302	0.039003	0.64308	1.9398	0.95592	0.019948	0.10636	2.7419	1.4552
536.8029	0.56	domics Pos	0.68676	0.99987	1.0297	0.04229	0.46214	0.99994	0.95964	-0.05944	0.23913	0.99996	0.94885	-0.07574	0.023606	0.27442	0.82794	-0.2724	0.001502	0.007333	0.69724	-0.52027	0.010581	0.089124	0.78021	-0.35806	0.027886	0.34862	0.77909	-0.36013	0.026048	0.56113	0.86681	-0.20622	0.29686	0.4072	1.1599	0.21395
758.5696	4.94	domics Pos	0.6869	0.99987	1.0372	0.052631	0.45769	0.99994	0.94048	-0.08853	0.60927	0.99996	0.95861	-0.06098	0.24748	0.70693	0.89545	-0.15931	0.006864	0.022999	0.7462	-0.42237	0.42562	0.66105	0.93245	-0.1009	0.57635	0.86079	1.0539	0.075766	0.44973	0.96776	1.0466	0.065653	0.016272	0.099614	1.2429	0.31369
206.8949	9.92	domics Pos	0.68692	0.99987	1.0264	0.037651	0.37229	0.99994	0.92787	-0.10801	0.36577	0.99996	0.92976	-0.10507	0.51926	0.89933	0.95623	-0.06457	0.005372	0.019249	0.78137	-0.35593	0.28559	0.57196	0.90685	-0.14106	0.88731	0.97532	0.99235	-0.01108	0.86892	0.98023	1.013	0.018701	0.010303	0.094875	1.2254	0.29322
336.0026	0.29	olomics Pc	0.68696	0.99951	1.0568	0.079709	0.96397	0.99998	0.99175	-0.01195	0.73759	0.99941	0.9582	-0.0616	0.041501	0.27873	1.4154	0.50118	0.033185	0.12489	1.3498	0.43276	0.12404	0.3473	1.1904	0.25144	0.030793	0.35049	1.3497	0.43267	0.76427	0.99956	1.0293	0.041669	0.27075	0.54284	0.87564	-0.19159
110.0719	0.61	domics Pos	0.68714	0.99987	0.96232	-0.05541	0.14699	0.99994	0.86419	-0.21058	0.38957	0.99996	1.0802	0.11134	0.089783	0.5005	1.2457	0.31692	0.28195	0.37682	0.91279	-0.13164	0.065315	0.25781	1.2186	0.28522	0.002526	0.26175	1.451	0.53704	0.35436	0.96776	1.1212	0.16506	0.037673	0.13071	1.2404	0.31085
855.653	5.46	domics Pos	0.68766	0.99987	0.96771	-0.04735	0.72215	0.99994	0.97573	-0.03545	0.45784	0.99996	0.93211	-0.10142	0.08869	0.49659	0.8345	-0.26102	0.003025	0.012283	0.6543	-0.61197	0.046436	0.20733	0.77047	-0.37619	0.40246	0.77496	0.92391	-0.11417	0.70628	0.97988	0.96709	-0.04827	0.84615	0.88661	1.0381	0.053912
472.8734	0.5	domics Pos	0.68827	0.99987	1.0333	0.047285	0.48941	0.99994	0.95272	-0.06987	0.95304	0.99996	0.99787	-0.00308	0.24977	0.70866	0.86945	-0.20183	0.010363	0.031573	0.76689	-0.3829	0.20294	0.49162	0.86556	-0.20829	0.22663	0.66988	0.91732	-0.1245	0.88259	0.98023	1.0055	0.007889	0.031481	0.12162	1.1824	0.24172
749.5308	5.12	domics Pos	0.68885	0.99987	1.0259	0.036932	0.57463	0.99994	0.97204	-0.04092	0.33561	0.99996	0.94516	-0.08137	0.055015	0.40264	0.9084	-0.1386	0.000465	0.003119	0.76388	-0.38857	0.22587	0.51386	0.90408	-0.14548	0.77181	0.94358	1.0516	0.072562	0.75851	0.97988	0.98399	-0.02329	0.14814	0.25308	1.0576	0.080736
725.532	5.22	domics Pos	0.68892	0.99987	1.0306	0.04346	0.42944	0.99994	0.9473	-0.07811	0.44747	0.99996	0.94971	-0.07444	0.082502	0.48472	0.88295	-0.1796	0.008588	0.027289	0.77083	-0.37552	0.26129	0.54699	1.1589	0.21278	0.023197	0.33809	1.4774	0.56308	0.019839	0.52955	1.2014	0.26472	0.004213	0.085451	1.2865	0.36347
381.2132	6.96	olomics Pc	0.6891	0.99951	0.95617	-0.06467	0.4502	0.99998	0.89267	-0.1638	0.041983	0.99941	0.75084	-0.41342	0.072693	0.35072	0.62647	-0.67468	0.001418	0.01217	0.51702	-0.9517	0.011254	0.083534	0.43383	-1.2048	0.001888	0.16989	0.40386	-1.3081	0.009333	0.47452	0.41275	-1.2767	0.023213	0.2688	0.53298	-0.90786
935.5581	6.35	domics Pos	0.68977	0.99987	0.95144	-0.07182	0.27915	0.99994	0.85097	-0.23281	0.30926	0.99996	0.86621	-0.20721	0.41934	0.85533	0.87767	-0.18825	0.012893	0.037132	0.61305	-0.70592	0.016135	0.10794	0.61343	-0.70503	0.15685	0.62953	0.71815	-0.47764	0.037754	0.64014	0.66077	-0.59778	0.70571	0.78023	0.96706	-0.04832
319.227	5.03	olomics Pc	0.69033	0.99951	1.0882	0.12199	0.58754	0.99998	0.94123	-0.08739	0.64712	0.99941	0.94556	-0.08076	0.038929	0.2713	0.7405	-0.43343	0.016667	0.072214	0.63155	-0.66303	0.30763	0.60946	0.88106	-0.18268	0.57755	0.99391	0.92794	-0.10789	0.1468	0.93461	0.85649	-0.22348	0.82004	0.92585	1.0226	0.032302
263.238	6.48	olomics Pc	0.69051	0.99951	0.95132	-0.072	0.58028	0.99998	0.93624	-0.09505	0.099478	0.99941	0.69592	-0.52301	0.042422	0.2802	0.56877	-0.81408	0.004879	0.028888	0.46777	-1.0961	0.013167	0.085844	0.43756	-1.1924	0.014203	0.28317	0.40087	-1.3188	0.012198	0.47452	0.41524	-1.268	0.028191	0.2688	0.40462	-1.3053
421.2895	5.93	olomics Pc	0.69063	0.99951	1.102	0.14012	0.47066	0.99998	0.88624	-0.17423	0.97694	0.99941	1.0016	0.002302	0.3279	0.82368	0.83105	-0.26699	0.000528	0.005945	0.31933	-1.6469	0.048454	0.18257	0.71741	-0.47913	0.56714	0.99391	0.92852	-0.107	0.65235	0.99956	0.94572	-0.08052	0.059067	0.30404	1.2966	0.37471
860.5349	5.9	domics Pos	0.69118	0.99987	1.059	0.082685	0.65143	0.99994	0.83437	-0.26124	0.97366	0.99996	0.94619	-0.0798	0.91227	0.9874	0.95079	-0.0728	0.69278	0.73862	1.0555	0.077892	0.057178	0.23827	1.7059	0.77053	0.033756	0.36346	2.0385	1.0275	0.064823	0.74674	1.8047	0.85174	0.026908	0.11548	2.385	1.254
564.8067	10.14	domics Pos	0.69125	0.99987	1.0221	0.031603	0.83724	0.99994	1.0163	0.023324	0.44612	0.99996	0.94502	-0.08159	0.35116	0.80554	0.91865	-0.12241	0.036522	0.082353	0.84179	-0.24847	0.51484	0.72545	0.93819	-0.09205	0.91661	0.98047	1.0119	0.017057	0.96239	0.9885	0.99484	-0.00746	0.014276	0.098791	1.3923	0.47748
827.6957	6.24	domics Pos	0.69163	0.99987	0.96041	-0.05828	0.10543	0.99994	0.82041	-0.28559	0.13363	0.99996	0.83199	-0.26537	0.067361	0.44056	0.8271	-0.27386	0.033893	0.077851	0.7727	-0.37201	0.091688	0.31942	0.83874	-0.25371	0.33111	0.7398	0.8976	-0.15586	0.21813	0.94047	0.79856	-0.32453	0.39482	0.50349	0.91025	-0.13567
778.5593	9.14	olomics Ne	0.69184	0.99918	1.047	0.066197	0.52134	0.86583	0.92726	-0.10895	0.93812	0.99995	1.038	0.053844	0.45815	0.99985	1.107	0.14665	0.81761	0.99927	1.0769	0.10692	0.034101	0.47167	1.3007	0.37923	0.17833	0.55116	1.3196	0.40014	0.11265	0.99512	1.2331	0.30226	0.67412	0.99941	1.0934	0.12879
708.9706	12.01	olomics Ne	0.69231	0.99918	0.97792	-0.03221	0.34141	0.75652	0.91275	-0.1317	0.10728	0.99995	1.1282	0.17405	0.65533	0.99985	1.1216	0.16556	0.46417	0.99927	1.0529	0.074377	0.26108	0.69038	1.2705	0.34535	0.000538	0.22316	1.2992	0.37764	0.74304	0.99512	1.0982	0.13519	0.28911	0.99941	1.0898	0.124
196.1066	0.84	domics Pos	0.6931	0.99987	1.0399	0.056464	0.30328	0.99994	0.85499	-0.22602	0.69908	0.99996	0.95499	-0.06645	0.80039	0.97856	1.0208	0.029724	0.023549	0.058959	0.72835	-0.4573	0.055277	0.23213	0.77995	-0.35854	0.22842	0.67233	0.82406	-0.27918	0.1698	0.91806	0.8507	-0.23327	0.66724	0.75094	0.9554	-0.06583
447.3419	2.85	domics Pos	0.69357	0.99987	1.0526	0.073923	0.87995	0.99994	0.96305	-0.05432	0.035443	0.99996	0.69004	-0.53525	0.068562	0.44612	0.59538	-0.74812	0.002408	0.010386	0.43732	-1.1932	0.009948	0.08787	0.40326	-1.3102	0.025956	0.34128	0.47842	-1.0636	0.031918	0.62711	0.51981	-0.94394	0.14959	0.25475	0.68036	-0.55563
235.2006	5.37	olomics Pc	0.69374	0.99951	1.0395	0.05584	0.23963	0.99998	0.89076	-0.1669	0.68185	0.99941	1.0374	0.053028	0.079163	0.36897	0.76791	-0.38099	6.29E-05	0.001131	0.48563	-1.0421	0.000915	0.042705	0.64961	-0.62236	0.099271	0.61311	0.78253	-0.35378	0.25567	0.99956	0.9088	-0.13797	0.88167	0.95289	0.98848	-0.01672
201.0326	0.6	lomics Neg	0.69389	0.99977	0.86314	-0.21233	0.91101	0.99904	1.1788	0.23727	0.62811	0.99994	0.87856	-0.18679	0.87317	0.91979	1.3068	0.38606	0.63603	0.99989	2.6775	1.4209	0.012716	0.46473	5.0318	2.3311	0.002449	0.093659	5.1755	2.3717	0.16792	0.59205	2.386	1.2546	0.75258	0.84914	1.207	0.27147
268.2697	7.49	olomics Pc	0.69391	0.99951	0.90175	-0.1492	0.69094	0.99998	0.92553	-0.11165	0.095564	0.99941	0.68692	-0.54179	0.097333	0.41658	0.52874	-0.91937	0.010356	0.049653	0.4881	-1.0348	0.008126	0.074624	0.3666	-1.4477	0.006802	0.22137	0.42757	-1.2258	0.015016	0.47452	0.4152	-1.2681	0.020343	0.2688	0.48607	-1.0407
591.3229	5.08	olomics Pc	0.69426	0.99951	0.97387	-0.03819	0.87659	0.99998	1.0446	0.062928	0.87187	0.99941	1.0222	0.031733	0.56436	0.99905	1.1623	0.21693	0.12065	0.33595	1.167	0.22277	0.003255	0.049867	1.5229	0.60681	0.001133	0.16989	1.7098	0.7738	0.63877	0.99956	1.0549	0.077044	0.99009	0.99365	1.022	0.031368
399.3225	2.72	domics Pos	0.69454	0.99987	0.97415	-0.03778	0.38943	0.99994	0.90711	-0.14064	0.00476	0.99996	0.75173	-0.41171	0.00432	0.17581	0.69618	-0.52247	0.000906	0.005185	0.5481	-0.86748	0.024418	0.13732	0.55309	-0.85442	0.004329	0.3088	0.58369	-0.77672	0.005695	0.52955	0.55397	-0.85211	0.069065	0.17067	0.71876	-0.47642
308.292	6.64	olomics Pc	0.69456	0.99951	1.0675	0.094209	0.49836	0.99998	0.91995	-0.12038	0.50366	0.99941	0.93048	-0.10396	0.14378	0.53536	0.74806	-0.41877	3.72E-05	0.000733	0.26876	-1.8956	0.003093	0.049681	0.46103	-1.1171	0.062844	0.51121	0.72608	-0.4618	0.096622	0.81817	0.80668	-0.30993	0.21972	0.48775	1.1335	0.18081
172.1707	4.16	olomics Pc	0.69479	0.99951	0.93451	-0.09772	0.76946	0.99998	0.94794	-0.07714	0.96617	0.99941	0.99895	-0.00151	0.06514	0.33343	0.71873	-0.47647	0.002684	0.018832	0.53907	-0.89145	0.012725	0.085685	0.62236	-0.68418	0.14199	0.70661	0.80027	-0.32144	0.17311	0.96511	0.80908	-0.30565	0.76192	0.89822	0.94617	-0.07983
754.5697	5.69	domics Pos	0.69487	0.99987	1.0247	0.035152	0.11014	0.99994	0.85845	-0.22019	0.23447	0.99996	0.89131	-0.166	0.17264	0.60551	0.83945	-0.25249	0.19388	0.28732	0.84593	-0.24139	0.9637	0.98146	0.99592	-0.0059	0.009944	0.3324	1.3362	0.41811	0.38766	0.96776	1.0892	0.12333	0.22129	0.32948	1.1701	0.22669
218.1283	0.35	olomics Pc	0.69518	0.99951	1.0551	0.077408	0.3653	0.99998	1.2826	0.35906	0.31113	0.99941	1.2953	0.37329	0.092504	0.40868	1.7304	0.79108	0.006876	0.036709	2.6668	1.4151	0.023081	0.11246	2.8932	1.5327	0.007031	0.22149	2.7103	1.4385	0.082027	0.78717	1.7636	0.81854	0.11856	0.36992	1.8353	0.87605
731.6051	8.94	olomics Pc	0.69545	0.99951	1.093	0.12823	0.82672	0.99998	1.0193	0.027545	0.58197	0.99941	1.0302	0.042859	0.95453	0.99905	1.0242	0.034553	0.086005	0.25438	1.1166	0.15908	0.77788	0.93702	0.97883	-0.03088	0.91631	0.9951	1.0032	0.004647	0.008136	0.47452	0.77376	-0.37004	0.014721	0.2688	0.77706	-0.3639
353.9016	10.09	domics Pos	0.69586	0.99987	0.97316	-0.03925	0.39993	0.99994	0.91314	-0.13109	0.16665	0.99996	0.87712	-0.18915	0.009712	0.20688	0.79426	-0.33232	0.000474	0.003154	0.72002	-0.47389	0.56729	0.76437	0.95974	-0.05929	0.51201	0.83396	1.0446	0.062994	0.85716	0.98023	0.98906	-0.01587	0.040118	0.13378	1.1267	0.17207
368.8777	10.11	domics Pos	0.69598	0.99987	1.0298	0.042351	0.59761	0.99994	0.95201	-0.07095	0.5488	0.99996	0.95418	-0.06767	0.91667	0.9874	1.0229	0.032722	0.10289	0.17816	0.83608	-0.25828	0.93505	0.96635	0.99357	-0.0093	0.025125	0.33809	1.2172	0.28356	0.78015	0.97988	1.0279	0.039735	0.059022	0.15646	1.2212	0.28825
125.8729	12.57	olomics Ne	0.69615	0.99918	1.0143	0.020543	0.045769	0.47152	0.85373	-0.22815	0.57251	0.99995	1.0269	0.03834	0.98435	0.99985	1.0023	0.003378	0.74186	0.99927	1.0166	0.0237	0.15812	0.62813	1.0854	0.11822	0.61904	0.87098	1.0181	0.025818	0.91209	0.99512	1.0097	0.013933	0.35539	0.99941	0.95373	-0.06834
608.3216	1.06	domics Pos	0.69637	0.99987	1.0615	0.08609	0.072572	0.99994	0.79401	-0.33277	0.35425	0.99996	0.90355	-0.14633	0.005192	0.17581	0.67867	-0.55921	0.007018	0.023412	0.55725	-0.8436	0.073834	0.28041	0.75622	-0.40313	0.72275	0.9233	0.96703	-0.04836	0.084623	0.81688	0.82608	-0.27565	0.54209	0.63835	1.1158	0.15807
269.9581	10.1	domics Pos	0.6969	0.99987	1.018	0.025753	0.086558	0.99994	0.90417	-0.14533	0.15696	0.99996	0.93311	-0.09988	0.027291	0.29231	0.88496	-0.17632	0.002186	0.009658	0.74124	-0.43199	0.415	0.65507	0.95645	-0.06424	0.92207	0.98126	0.99502	-0.0072	0.19758	0.92582	0.91132	-0.13397	0.10178	0.20394	1.1681	0.22416
245.2274	6.48	olomics Pc	0.69691	0.99951	0.95193	-0.07107	0.58542	0.99998	0.9363	-0.09495	0.085923	0.99941	0.68932	-0.53676	0.05068	0.29486	0.5738	-0.80137	0.006101	0.033658	0.46595	-1.1017	0.017066	0.096516	0.43089	-1.2146	0.014524	0.28593	0.39173	-1.3521	0.012004	0.47452	0.40348	-1.3094	0.027109	0.2688	0.3942	-1.343
439.8716	10.15	domics Pos	0.69805	0.99987	0.97958	-0.02976	0.16415	0.99994	0.82845	-0.27152	0.052164	0.99996	0.76463	-0.38717	0.002625	0.17581	0.85948	-0.21846	0.039577	0.087636	0.74514	-0.42442	0.027004	0.14542	0.73526	-0.44367	0.000608	0.17164	0.75523	-0.40501	0.013549	0.52955	0.83055	-0.26787	0.18067	0.288	1.129	0.175
356.1464	1.75	domics Pos	0.69814	0.99987	1.1484	0.19961	0.5838	0.99994	0.9083	-0.13876	0.081088	0.99996	0.72515	-0.46365	0.007741	0.19917	0.57465	-0.79924	0.001317	0.006694	0.36139	-1.4684	0.000632	0.03855	0.38593	-1.3736	0.005519	0.3088	0.52564	-0.92785	0.029124	0.60026	0.54638	-0.87203	0.028689	0.11732	0.64267	-0.63784
1018.666	1.3	domics Pos	0.69911	0.99987	1.1075	0.14733	0.95102	0.99994	0.97027	-0.04354	0.92091	0.99996	0.94592	-0.0802	0.88829	0.9874	0.90079	-0.15074	0.57599	0.63807	0.76526	-0.38597	0.2892	0.57347	1.6332	0.70769	0.028533	0.35164	2.3744	1.2476	0.34557	0.96524	1.357	0.44044	0.047287	0.14378	2.9472	1.5593
439.3007	5.45	olomics Pc	0.69993	0.99951	0.88539	-0.17561	0.70971	0.99998	0.88815	-0.17112	0.55608	0.99941	0.82975	-0.26924	0.84876	0.99905	0.93668	-0.09438	0.001614	0.013281	0.22936	-2.1243	0.1053	0.31146	0.6153	-0.70064	0.71624	0.99391	1.0735	0.10228	0.92102	0.99956	0.97607	-0.03495	0.86801	0.94824	1.0168	0.023966
883.6854	5.45	domics Pos	0.69995	0.99987	0.94778	-0.07738	0.74368	0.99994	1.0332	0.047072	0.15189	0.99996	0.82015	-0.28605	0.15353	0.58077	0.78561	-0.34812	0.022206	0.056462	0.58575	-0.77164	0.055118	0.23197	0.65643	-0.6073	0.69619	0.91171	0.94707	-0.07845	0.45186	0.96776	0.8859	-0.17479	0.85388	0.89324	0.97357	-0.03864
1075.195	9.05	olomics Pc	0.70002	0.99951	1.1531	0.20555	0.05036	0.99998	0.74126	-0.43195	0.6136	0.99941	0.9415	-0.08697	0.0235	0.22481	0.6728	-0.57176	0.099257	0.28449	0.74171	-0.43107	0.023277	0.11298	0.71599	-0.482	0.66027	0.99391	0.95483	-0.06668	0.84418	0.99956	1.0005	0.000725	0.22964	0.49625	1.3261	0.40714
1029.774	0.53	domics Pos	0.70011	0.99987	0.95886	-0.06061	0.11939	0.99994	0.83976	-0.25195	0.52304	0.99996	0.93776	-0.09271	0.37492	0.82708	0.92423	-0.11368	0.025545	0.062761	0.76208	-0.39198	0.19743	0.48532	0.83841	-0.25426	0.54687	0.8511	0.93636	-0.09487	0.99249	0.99721	0.99946	-0.00078	0.13833	0.24373	1.1805	0.23935
896.6426	6	domics Pos	0.70029	0.99987	1.0548	0.07701	0.46144	0.99994	0.90958	-0.13673	0.22279	0.99996	0.8108	-0.30259	0.04056	0.34959	0.6623	-0.59445	0.093252	0.16553	0.70839	-0.49737	0.026972	0.14542	0.72343	-0.46708	0.77856	0.94624	0.98414	-0.02306	0.22218	0.94047	0.84614	-0.24104	0.33256	0.44229	0.87945	-0.18533
600.5072	5	domics Pos	0.70044	0.99987	1.0709	0.098882	0.9816	0.99994	1.0122	0.017525	0.92307	0.99996	0.99981	-0.00028	0.3811	0.82927	0.8842	-0.17755	0.040941	0.089717	0.74655	-0.42169	0.37211	0.6301	0.86645	-0.20682	0.94855	0.98901	1.0112	0.016018	0.032236	0.62711	1.4426	0.52864	0.001678	0.07528	1.7752	0.82802
152.9479	10.19	domics Pos	0.70051	0.99987	0.9619	-0.05604	0.46337	0.99994	0.91395	-0.12981	0.79361	0.99996	0.97156	-0.04163	0.42961	0.8617	0.92692	-0.10948	0.025484	0.062691	0.77452	-0.36862	0.7083	0.8485	0.95844	-0.06124	0.54885	0.85184	0.93753	-0.09307	0.30864	0.9524	0.90378	-0.14596	0.087129	0.1878	1.1819	0.24105
1022.749	0.54	domics Pos	0.70055	0.99987	0.95235	-0.07043	0.21867	0.99994	0.86832	-0.2037	0.26043	0.99996	0.83457	-0.2609	0.23353	0.69182	0.87674	-0.18977	0.027174	0.065766	0.7138	-0.48641	0.32181	0.5977	0.83678	-0.25709	0.31745	0.72948	0.84994	-0.23458	0.33018	0.95887	0.88344	-0.1788	0.19802	0.3063	1.1887	0.24944
1148.837	4.94	domics Pos	0.70128	0.99987	0.97053	-0.04316	0.17766	0.99994	0.85008	-0.23433	0.39344	0.99996	0.91983	-0.12056	0.028207	0.29796	0.77424	-0.36914	0.005361	0.019228	0.64652	-0.62924	0.47002	0.69377	0.92797	-0.10786	0.74146	0.93154	0.97679	-0.03388	0.18083	0.92305	0.88286	-0.17974	0.22121	0.32948	1.153	0.20539
391.2637	0.88	domics Pos	0.7014	0.99987	0.85894	-0.21937	0.43502	0.99994	0.69399	-0.52702	0.45611	0.99996	0.74351	-0.42758	0.61323	0.93755	0.9036	-0.14624	0.90748	0.92027	1.2843	0.36101	0.26956	0.5571	2.6034	1.3804	0.003166	0.2624	3.8869	1.9586	0.21591	0.93767	1.6285	0.70356	0.16211	0.26992	1.7011	0.76649
355.3648	8.3	olomics Pc	0.70141	0.99951	0.95132	-0.07199	0.42073	0.99998	0.88459	-0.17691	0.39997	0.99941	0.88766	-0.17192	0.73396	0.99905	0.95657	-0.06406	0.38972	0.78925	1.0772	0.10731	0.031992	0.13786	0.72903	-0.45596	0.24015	0.84407	0.79714	-0.3271	0.045659	0.66241	0.76448	-0.38745	0.35179	0.62856	0.88973	-0.16857
431.9178	0.5	domics Pos	0.70144	0.99987	1.0199	0.028383	0.23297	0.99994	0.90813	-0.13903	0.30176	0.99996	0.9233	-0.11513	0.11567	0.53721	0.87598	-0.19103	0.00125	0.006466	0.72541	-0.46314	0.29358	0.57612	0.88049	-0.18363	0.66018	0.89455	0.96398	-0.05292	0.15436	0.91321	0.89667	-0.15735	0.095322	0.19678	1.1798	0.2385
560.3093	1.37	domics Pos	0.70172	0.99987	1.0686	0.095728	0.9544	0.99994	0.99634	-0.00528	0.96809	0.99996	0.95143	-0.07183	0.95326	0.99365	0.94434	-0.08262	0.81148	0.83943	0.88709	-0.17284	0.1239	0.38348	1.604	0.68166	0.047956	0.39925	1.9748	0.9817	0.36718	0.96776	1.3069	0.3862	0.099696	0.20156	2.1455	1.1013
106.9517	0.51	domics Pos	0.70205	0.99987	1.1006	0.13832	0.41577	0.99994	0.92474	-0.11289	0.61897	0.99996	0.95817	-0.06164	0.98639	0.99652	1.0038	0.005528	0.011942	0.03513	0.80233	-0.31772	0.87675	0.93617	1.0308	0.043719	0.18218	0.64793	1.1114	0.15232	0.62413	0.97813	1.0513	0.072154	0.011284	0.094982	1.2467	0.31817
203.1806	6	olomics Pc	0.70262	0.99951	1.0327	0.046414	0.046927	0.99998	0.81718	-0.29127	0.41577	0.99941	0.94526	-0.08122	0.018155	0.20936	0.74014	-0.43412	3.08E-08	9.71E-05	0.26103	-1.9377	0.009117	0.076393	0.55558	-0.84794	0.020875	0.31165	0.70969	-0.49475	0.12003	0.8972	0.859	-0.21927	0.45518	0.71854	1.0871	0.12044
600.459	2.27	domics Pos	0.70314	0.99987	1.0503	0.07078	0.21263	0.99994	0.79241	-0.33568	0.084182	0.99996	0.75076	-0.41357	0.015916	0.24	0.51351	-0.96152	5.94E-05	0.000728	0.21409	-2.2237	0.011253	0.091488	0.37284	-1.4234	0.031061	0.35811	0.49246	-1.0219	0.034024	0.6327	0.52254	-0.93638	0.078946	0.1809	0.70214	-0.51016
289.9696	0.58	domics Pos	0.70318	0.99987	0.96691	-0.04855	0.28427	0.99994	0.90212	-0.1486	0.32363	0.99996	0.92242	-0.11651	0.5383	0.90305	1.0853	0.11812	0.021763	0.055592	0.79498	-0.33101	0.41185	0.65457	0.91827	-0.12301	0.15963	0.63227	1.1356	0.18343	0.40618	0.96776	1.1464	0.19709	0.19097	0.29911	1.3056	0.3847
344.6134	3.72	domics Pos	0.70333	0.99987	0.96203	-0.05585	0.1368	0.99994	0.77769	-0.36273	0.004729	0.99996	0.64579	-0.63085	0.80347	0.97856	0.96944	-0.04477	0.050763	0.10551	0.69815	-0.51839	0.11876	0.37382	0.77263	-0.37216	0.035921	0.36525	0.65735	-0.60526	0.67269	0.97988	0.94986	-0.07422	0.075521	0.17753	0.75954	-0.39679
197.8079	0.39	olomics Ne	0.70337	0.99918	0.98316	-0.02451	0.012835	0.37315	0.88634	-0.17407	0.92352	0.99995	1.0062	0.00888	0.74952	0.99985	1.011	0.015782	0.41456	0.99927	1.051	0.071795	0.091825	0.55533	1.103	0.14139	0.058844	0.40009	1.0654	0.091347	0.64746	0.99512	0.98284	-0.02497	0.3411	0.99941	0.96626	-0.04951
1141.275	6.2	domics Pos	0.70346	0.99987	1.1056	0.14489	0.22424	0.99994	0.79009	-0.3399	0.70539	0.99996	0.92931	-0.10576	0.044256	0.36496	0.63318	-0.65932	0.011628	0.034422	0.47898	-1.0619	0.021767	0.12888	0.54768	-0.86858	0.57043	0.85811	0.89776	-0.1556	0.67753	0.97988	0.93474	-0.09737	0.14595	0.25057	1.6134	0.69013
867.81	0.53	domics Pos	0.7041	0.99987	0.97129	-0.04203	0.34651	0.99994	0.88827	-0.17094	0.1464	0.99996	0.89115	-0.16626	0.47746	0.88482	0.92765	-0.10835	0.003291	0.013152	0.69888	-0.51689	0.18456	0.47124	0.84676	-0.23997	0.40884	0.77945	0.92564	-0.11148	0.37043	0.96776	0.9496	-0.07461	0.22271	0.33092	1.0936	0.1291
368.2023	2.09	domics Pos	0.70417	0.99987	0.88477	-0.17663	0.42614	0.99994	0.77913	-0.36006	0.11958	0.99996	0.59564	-0.74749	0.044002	0.36496	0.42659	-1.2291	0.017197	0.046309	0.30057	-1.7342	0.008191	0.084185	0.21842	-2.1948	0.025923	0.34128	0.31797	-1.653	0.037351	0.64014	0.3558	-1.4909	0.073754	0.17577	0.47372	-1.0779
1184.722	10.06	domics Pos	0.70484	0.99987	0.97667	-0.03405	0.91769	0.99994	1.0099	0.014203	0.045892	0.99996	0.91171	-0.13335	0.24478	0.70602	0.91954	-0.12102	0.028385	0.06807	0.87916	-0.18581	0.02194	0.12963	0.88009	-0.18428	0.96652	0.99304	1.0017	0.00247	0.85032	0.97988	0.99014	-0.0143	0.00653	0.090084	1.3308	0.41229
440.2783	3.52	domics Pos	0.70514	0.99987	1.0639	0.089421	0.094129	0.99994	0.75105	-0.41302	0.060547	0.99996	0.7463	-0.42218	0.004996	0.17581	0.56721	-0.81804	0.001769	0.008265	0.46901	-1.0923	0.013	0.096206	0.48978	-1.0298	0.012694	0.3324	0.56287	-0.82913	0.003197	0.52955	0.5172	-0.95122	0.16481	0.27227	0.72612	-0.46172
572.3709	1.35	domics Pos	0.70546	0.99987	1.102	0.14011	0.56042	0.99994	1.0871	0.12043	0.45918	0.99996	1.1274	0.17298	0.6504	0.95432	1.0657	0.091844	0.83356	0.85948	0.90968	-0.13657	0.052611	0.22542	1.6059	0.68342	0.024464	0.33809	1.9872	0.99076	0.38295	0.96776	1.1723	0.22935	0.018774	0.10435	2.0825	1.0583
284.7806	0.34	olomics Ne	0.70603	0.99918	1.1348	0.18243	0.20424	0.62533	0.87105	-0.19918	0.61674	0.99995	1.0859	0.1189	0.83227	0.99985	1.029	0.041287	0.063257	0.99927	1.1902	0.25121	0.015191	0.40708	1.2788	0.35476	0.16295	0.54067	1.2341	0.30345	0.33183	0.99512	1.0775	0.10764	0.94677	0.99941	1.0031	0.004394
809.9942	6.49	olomics Pc	0.70622	0.99951	1.1364	0.18449	0.41882	0.99998	0.8733	-0.19545	0.74409	0.99941	1.0525	0.073779	0.013164	0.20085	0.69874	-0.51717	0.68544	0.99688	0.94445	-0.08246	0.10202	0.30432	0.81075	-0.30267	0.99572	0.99906	1.0379	0.053723	0.69062	0.99956	0.95639	-0.06433	0.82402	0.92901	1.0972	0.1338
422.8839	0.54	domics Pos	0.70666	0.99987	1.0165	0.023668	0.4052	0.99994	0.95068	-0.07297	0.26254	0.99996	0.93617	-0.09516	0.046588	0.3732	0.88177	-0.18153	0.001017	0.005561	0.7563	-0.40297	0.18676	0.47264	0.8547	-0.22651	0.28301	0.71041	0.91948	-0.12111	0.48217	0.96776	0.93939	-0.0902	0.164	0.27173	1.1633	0.21823
385.2704	2.28	domics Pos	0.70682	0.99987	0.96551	-0.05063	0.14515	0.99994	0.78414	-0.35082	0.017332	0.99996	0.70217	-0.5101	0.01535	0.23792	0.54025	-0.88831	4.14E-05	0.000555	0.42829	-1.2233	0.010256	0.08787	0.4081	-1.293	0.015444	0.33809	0.55102	-0.85981	0.001039	0.41831	0.41438	-1.271	0.050113	0.14661	0.6216	-0.68595
303.2319	1.1	domics Pos	0.70721	0.99987	1.1452	0.1956	0.88897	0.99994	0.81579	-0.29374	0.63503	0.99996	0.69193	-0.5313	0.72146	0.96819	0.8467	-0.24008	0.001004	0.005526	0.026816	-5.2207	0.18882	0.47491	0.42372	-1.2388	0.38896	0.7665	0.53038	-0.91491	0.70943	0.97988	0.67336	-0.57054	0.71075	0.78486	1.0643	0.089942
836.6681	6.05	domics Pos	0.70779	0.99987	0.96831	-0.04645	0.14265	0.99994	0.81966	-0.2869	0.4116	0.99996	0.91665	-0.12556	0.099422	0.51973	0.82171	-0.2833	0.014096	0.039843	0.72499	-0.46396	0.21387	0.50527	0.80518	-0.31262	0.26606	0.70012	0.90365	-0.14617	0.14705	0.91175	0.84806	-0.23777	0.4506	0.55531	0.93013	-0.10449
864.9743	12.01	olomics Ne	0.70794	0.99918	1.0608	0.085194	0.022391	0.40327	0.75227	-0.41068	0.21618	0.99995	0.8834	-0.17886	0.2937	0.99985	0.869	-0.20256	0.39075	0.99927	0.92692	-0.10948	0.28325	0.70804	1.1632	0.21804	0.20248	0.58373	1.1412	0.19053	0.72905	0.99512	1.037	0.052428	0.7806	0.99941	0.97399	-0.03802
437.9788	4.88	olomics Ne	0.70803	0.99918	0.98003	-0.0291	0.007603	0.34605	0.88143	-0.18208	0.49462	0.99995	0.95879	-0.06071	0.64595	0.99985	0.96764	-0.04745	0.33408	0.99927	0.95403	-0.0679	0.69982	0.94048	1.0323	0.045923	0.34863	0.71526	1.0426	0.060238	0.82832	0.99512	0.98989	-0.01466	0.77339	0.99941	1.0129	0.018457
608.8775	10.03	domics Pos	0.70846	0.99987	0.97493	-0.03663	0.19823	0.99994	0.91389	-0.12991	0.10183	0.99996	0.8861	-0.17447	0.094016	0.51047	0.8791	-0.1859	0.001803	0.008404	0.74747	-0.41992	0.16339	0.44854	0.83469	-0.26068	0.48209	0.81445	0.94879	-0.07584	0.22851	0.94286	0.92052	-0.11948	0.12145	0.22569	1.1098	0.15028
1126.758	0.5	domics Pos	0.70904	0.99987	1.103	0.14142	0.4194	0.99994	0.89785	-0.15545	0.45245	0.99996	0.91539	-0.12753	0.50409	0.89133	0.93128	-0.10272	0.017262	0.046451	0.77678	-0.36442	0.8669	0.93123	0.98897	-0.016	0.39477	0.76955	1.0929	0.12819	0.94845	0.98838	0.99389	-0.00884	0.14506	0.2496	1.1485	0.19976
998.7747	10.03	domics Pos	0.70991	0.99987	1.0272	0.038768	0.26322	0.99994	0.91247	-0.13216	0.1151	0.99996	0.90271	-0.14767	0.15674	0.58521	0.90528	-0.14356	0.018523	0.049151	0.78509	-0.34907	0.069712	0.26931	0.81038	-0.30332	0.34798	0.74762	0.94044	-0.08859	0.14657	0.91175	0.90339	-0.14659	0.16409	0.27173	1.117	0.15959
830.8083	10.03	domics Pos	0.70998	0.99987	1.0295	0.041875	0.030808	0.99994	0.9156	-0.12721	0.12789	0.99996	0.9247	-0.11294	0.43427	0.86496	0.94113	-0.08753	0.000702	0.004234	0.75661	-0.40238	0.13301	0.39936	0.88981	-0.16843	0.84734	0.96798	0.99154	-0.01226	0.93122	0.98602	0.99775	-0.00325	0.03697	0.13049	1.2017	0.26513
741.5572	5.48	domics Pos	0.7106	0.99987	0.96638	-0.04934	0.14819	0.99994	0.84081	-0.25015	0.49282	0.99996	0.92833	-0.10729	0.97012	0.99502	1.0016	0.002247	0.024602	0.060953	0.78916	-0.34161	0.22148	0.51169	0.79916	-0.32345	0.52178	0.83957	0.93248	-0.10085	0.22189	0.94047	0.85444	-0.22694	0.52572	0.62461	0.94704	-0.0785
246.9478	10.15	domics Pos	0.71089	0.99987	0.97689	-0.03373	0.8549	0.99994	0.99206	-0.0115	0.58655	0.99996	0.97073	-0.04286	0.05832	0.41721	0.86496	-0.2093	0.003857	0.014929	0.75732	-0.40102	0.012716	0.095104	0.84717	-0.23927	0.04235	0.38314	1.0972	0.13388	0.72555	0.97988	1.019	0.027187	0.03612	0.12908	1.3051	0.38418
810.973	12.02	olomics Ne	0.71105	0.99918	1.0275	0.039185	0.46647	0.82998	1.0947	0.13049	0.29922	0.99995	1.0975	0.13427	0.76012	0.99985	0.98143	-0.02705	0.11907	0.99927	1.2125	0.27801	0.63944	0.92263	1.1953	0.25743	0.00417	0.22316	1.3552	0.43846	0.65785	0.99512	0.97662	-0.03414	0.813	0.99941	1.0913	0.12599
280.2351	2.27	lomics Neg	0.71152	0.99977	0.9997	-0.00043	0.53712	0.99904	0.95817	-0.06165	0.048411	0.99994	0.57857	-0.78944	0.056956	0.58182	0.49209	-1.023	0.000691	0.040886	0.38228	-1.3873	0.02133	0.48568	0.26518	-1.915	0.002617	0.093659	0.22515	-2.151	0.011946	0.46971	0.1958	-2.3525	0.011252	0.38536	0.25993	-1.9438
1116.74	9.02	olomics Pc	0.71163	0.99951	1.216	0.28213	0.33582	0.99998	0.86498	-0.20926	0.92581	0.99941	1.0043	0.006129	0.002931	0.17097	0.61081	-0.71121	0.060595	0.19378	0.63935	-0.64533	0.023844	0.11432	0.73837	-0.43758	0.8454	0.9951	1.0311	0.044196	0.95242	0.99956	1.0103	0.014807	0.083814	0.33342	1.5207	0.60471
472.3027	6.99	olomics Pc	0.71172	0.99951	1.3584	0.44194	0.9758	0.99998	1.0139	0.019864	0.069199	0.99941	1.4509	0.537	0.48858	0.96551	1.1658	0.22135	0.06522	0.20401	1.5991	0.6773	0.04137	0.16413	1.4158	0.50157	0.05076	0.45418	1.9112	0.93446	0.30575	0.99956	1.3541	0.4373	0.10064	0.35342	1.4591	0.54503
196.1791	6.38	olomics Pc	0.71198	0.99951	1.0402	0.05693	0.48319	0.99998	0.87422	-0.19393	0.93259	0.99941	0.96958	-0.04457	0.30357	0.79293	0.79931	-0.32318	0.000205	0.002846	0.24055	-2.0556	0.11419	0.327	0.70706	-0.5001	0.37329	0.97118	0.82923	-0.27015	0.71823	0.99956	0.92485	-0.11271	0.97063	0.9861	0.98111	-0.02751

364.9316	10.02	domics Pos	0.71205	0.99987	1.0266	0.037849	0.072625	0.99994	1.14	0.18905	0.57715	0.99996	1.0293	0.041682	0.71126	0.96346	0.97345	-0.03882	0.046542	0.098701	0.80173	-0.31882	0.85796	0.92844	1.0211	0.030071	0.3863	0.76579	1.112	0.15314	0.6757	0.97988	1.0391	0.055356	0.033519	0.12517	1.3129	0.39272
628.5386	5.45	domics Pos	0.71267	0.99987	0.9703	-0.04349	0.20932	0.99994	0.87576	-0.19139	0.099961	0.99996	0.89612	-0.15823	0.021246	0.26474	0.79854	-0.32456	0.00262	0.011051	0.68862	-0.53823	0.033747	0.165	0.78152	-0.35565	0.35886	0.75581	1.0743	0.10337	0.71652	0.97988	1.0812	0.11261	0.91007	0.93382	1.0421	0.05952
147.114	6.48	oolomics Pc	0.71293	0.99951	0.94907	-0.07542	0.67995	0.99998	0.96234	-0.05539	0.092874	0.99941	0.69533	-0.52423	0.096236	0.41572	0.619	-0.69198	0.008007	0.040942	0.49585	-1.012	0.008527	0.075866	0.42572	-1.232	0.023461	0.32255	0.43696	-1.1944	0.008663	0.47452	0.39663	-1.3341	0.029996	0.2688	0.45058	-1.1502
175.1197	0.31	oolomics Pc	0.71361	0.99951	0.97476	-0.03689	0.083768	0.99998	0.87624	-0.1906	0.88957	0.99941	1.0128	0.018382	0.92942	0.99905	0.99543	-0.00661	0.15067	0.40222	0.84904	-0.2361	0.91204	0.97434	0.99447	-0.008	0.031253	0.35412	1.4029	0.48838	0.82099	0.99956	0.98786	-0.01763	0.067119	0.30938	1.2279	0.29619
896.5383	4.78	domics Pos	0.71464	0.99987	1.027	0.038459	0.88315	0.99994	0.99453	-0.00791	0.77051	0.99996	0.97816	-0.03185	0.77233	0.97526	0.97838	-0.03153	0.018662	0.049443	0.78484	-0.34954	0.64653	0.81508	0.9546	-0.06703	0.90747	0.97907	0.99335	-0.00963	0.77192	0.97988	0.97939	-0.03005	0.16417	0.27173	1.1361	0.18413
217.1963	6	oolomics Pc	0.71491	0.99951	1.084	0.11641	0.47382	0.99998	0.90999	-0.13608	0.26352	0.99941	0.85435	-0.22709	0.034847	0.25587	0.69472	-0.52549	2.71E-05	0.000587	0.28023	-1.8353	0.000557	0.039708	0.49434	-1.0164	0.037713	0.38531	0.69789	-0.51892	0.013199	0.47452	0.748	-0.41888	0.045695	0.28476	0.81743	-0.29084
317.1792	1.83	domics Pos	0.71492	0.99987	0.96912	-0.04526	0.22034	0.99994	0.80533	-0.31235	0.01667	0.99996	0.69231	-0.5305	0.002186	0.17581	0.39455	-1.3417	0.002727	0.011399	0.38906	-1.3619	0.025249	0.13997	0.37023	-1.4335	0.000226	0.15466	0.30969	-1.6911	0.011951	0.52955	0.36085	-1.4705	0.029709	0.11862	0.44522	-1.1674
352.2398	2.04	domics Pos	0.7151	0.99987	0.97016	-0.04371	0.18463	0.99994	0.87281	-0.19626	0.004074	0.99996	0.69131	-0.53259	0.001735	0.17581	0.55589	-0.84713	0.007379	0.024364	0.59205	-0.75621	0.00278	0.061655	0.41826	-1.2575	0.013944	0.33809	0.58957	-0.76227	0.055225	0.70426	0.63331	-0.65902	0.018551	0.10388	0.63653	-0.65171
180.625	6	oolomics Pc	0.71532	0.99951	1.0759	0.10553	0.22257	0.99998	0.83341	-0.26291	0.53559	0.99941	0.93361	-0.0991	0.33211	0.82503	0.91242	-0.13222	5.89E-05	0.001067	0.23624	-2.0817	0.035914	0.14944	0.63644	-0.6519	0.032037	0.35567	0.67162	-0.57428	0.75902	0.99956	0.97974	-0.02953	0.73252	0.8841	1.0175	0.025015
400.3422	5	oolomics Pc	0.71551	0.99951	1.0409	0.057794	0.98973	0.99998	0.95133	-0.07199	0.51681	0.99941	0.83623	-0.25803	0.38112	0.87913	0.79533	-0.33037	0.023271	0.094586	0.47091	-1.0865	0.023726	0.11397	0.39129	-1.3537	0.06767	0.52762	0.49311	-1.02	0.13681	0.92085	0.61509	-0.70113	0.022575	0.2688	0.46498	-1.1048
730.812	10.12	domics Pos	0.71567	0.99987	1.0401	0.056672	0.83622	0.99994	1.0362	0.051318	0.95457	0.99996	0.99861	-0.00201	0.028443	0.29796	0.87689	-0.18953	0.18181	0.27368	0.88244	-0.18043	0.58267	0.77275	0.958	-0.0619	0.47566	0.80869	1.049	0.069006	0.93456	0.98616	0.99885	-0.00166	0.006051	0.08977	1.2955	0.37347
167.0319	0.56	domics Pos	0.71571	0.99987	0.80166	-0.31893	0.77405	0.99994	0.81778	-0.29022	0.47284	0.99996	0.69013	-0.53507	0.10286	0.52715	0.43299	-1.2076	0.056685	0.11448	0.34557	-1.533	0.026717	0.1447	0.26393	-1.9218	0.01859	0.33809	0.25394	-1.9774	0.097758	0.84751	0.38585	-1.3739	0.051602	0.14839	0.32746	-1.6106
613.4814	5.55	domics Pos	0.71608	0.99987	0.8818	-0.18148	0.53532	0.99994	0.7794	-0.35956	0.50285	0.99996	0.7841	-0.35089	0.20432	0.6514	0.58907	-0.76349	0.039654	0.087753	0.40141	-1.3168	0.38846	0.64105	0.71293	-0.48818	0.87666	0.97532	1.0128	0.01841	0.82462	0.97988	1.0774	0.1076	0.060445	0.15775	2.1096	1.077
340.9294	10.14	domics Pos	0.71622	0.99987	0.96485	-0.05163	0.18533	0.99994	0.87256	-0.19667	0.72596	0.99996	0.96568	-0.05038	0.19815	0.64373	0.88715	-0.17275	0.002163	0.009589	0.68718	-0.54124	0.54068	0.74426	0.94073	-0.08815	0.59832	0.86481	1.045	0.063566	0.62791	0.97904	0.9511	-0.07233	0.4399	0.54494	1.0694	0.096776
382.2062	6.96	oolomics Pc	0.71659	0.99951	0.95902	-0.06036	0.6446	0.99998	0.95098	-0.07251	0.016569	0.99941	0.70317	-0.50806	0.033434	0.25203	0.59071	-0.75947	0.002968	0.020459	0.52823	-0.92077	0.001976	0.045425	0.40917	-1.2892	0.014956	0.28681	0.46507	-1.1045	0.005736	0.47452	0.45224	-1.1449	0.025423	0.2688	0.53219	-0.90999
341.3586	4.17	domics Pos	0.71668	0.99987	1.0387	0.054772	0.95857	0.99994	1.0058	0.008279	0.56068	0.99996	0.92593	-0.11103	0.57497	0.91693	0.92985	-0.10494	0.26286	0.35803	0.87569	-0.19151	0.032423	0.16174	0.69882	-0.51701	0.66283	0.89623	0.94492	-0.08173	0.50954	0.96776	0.92264	-0.11617	0.71642	0.78883	1.1133	0.15491
369.2053	2.72	domics Pos	0.7172	0.99987	0.94105	-0.08765	0.085989	0.99994	0.75475	-0.40592	0.080214	0.99996	0.75513	-0.40521	0.024816	0.27831	0.68545	-0.54488	0.003006	0.012269	0.54549	-0.87438	0.058405	0.24091	0.60923	-0.71495	0.069068	0.46982	0.67722	-0.5623	0.025714	0.56113	0.55601	-0.84681	0.2257	0.33285	0.76473	-0.38697
84.0516	0.6	domics Pos	0.71738	0.99987	0.97969	-0.02961	0.062144	0.99994	0.83468	-0.26071	0.86319	0.99996	0.99464	-0.00776	0.20085	0.64674	1.1777	0.23593	0.44997	0.53291	0.93905	-0.09073	0.34597	0.61311	1.0612	0.085645	0.043882	0.38909	1.2955	0.37355	0.86566	0.98023	1.03	0.042585	0.018478	0.10388	1.3728	0.45717
1014.558	5.74	domics Pos	0.71772	0.99987	1.0276	0.039321	0.44056	0.99994	0.91383	-0.13	0.59557	0.99996	0.9429	-0.08483	0.20112	0.64674	0.86382	-0.2112	0.004127	0.015625	0.69378	-0.52744	0.093116	0.32266	0.78129	-0.35607	0.24789	0.68479	0.88849	-0.17057	0.27915	0.94365	0.91512	-0.12796	0.0423	0.1368	1.2757	0.35125
1071.526	6.1	domics Pos	0.71777	0.99987	0.94623	-0.07973	0.94766	0.99994	1.0055	0.007855	0.69488	0.99996	0.94255	-0.08536	0.12839	0.55024	0.75565	-0.40421	0.70629	0.74975	1.0157	0.022509	0.054281	0.23023	0.66599	-0.58642	0.016106	0.33809	0.60505	-0.72488	0.12646	0.88537	0.76094	-0.39414	0.99033	0.99241	1.0303	0.043116
624.3197	2.99	domics Pos	0.71793	0.99987	0.92408	-0.11391	0.31948	0.99994	0.83058	-0.26781	0.77724	0.99996	0.9364	-0.0948	0.37221	0.82444	0.8322	-0.26499	0.003834	0.014857	0.40957	-1.2878	0.18225	0.47049	0.77074	-0.37568	0.95595	0.98985	0.98423	-0.02294	0.75639	0.97988	1.0412	0.058209	0.057742	0.15492	1.3486	0.4315
118.0472	0.59	lomics Neg	0.71812	0.99977	1.0905	0.12499	0.91799	0.99904	0.99933	-0.00096	0.22943	0.99994	1.3386	0.42068	0.010291	0.58182	2.0183	1.0131	0.011828	0.31827	1.7036	0.76861	0.005401	0.46473	1.8662	0.90013	0.003164	0.093659	2.1474	1.1026	0.3402	0.66332	1.4232	0.50912	0.81307	0.88157	1.0407	0.057523
1159.833	4.95	domics Pos	0.71828	0.99987	1.0292	0.041494	0.40249	0.99994	0.9223	-0.1167	0.34875	0.99996	0.91356	-0.13043	0.40977	0.85195	0.93543	-0.0963	0.004059	0.015446	0.73847	-0.43739	0.35245	0.61857	0.88113	-0.18258	0.60429	0.86762	0.95797	-0.06194	0.59757	0.97073	0.97846	-0.03142	0.019908	0.10636	1.235	0.30457
682.2928	5.21	olomics Ne	0.71869	0.99918	1.0531	0.074633	0.96205	0.99363	1.0108	0.015449	0.24087	0.99995	1.1272	0.17274	0.45835	0.99985	1.0963	0.13258	0.64694	0.99927	1.0554	0.077779	0.012868	0.40245	1.4516	0.53762	0.005779	0.2539	1.4809	0.56649	0.1917	0.99512	1.177	0.23507	0.21812	0.99941	1.1679	0.22393
814.6314	5.78	domics Pos	0.71927	0.99987	1.0371	0.052556	0.72821	0.99994	0.96064	-0.05793	0.64891	0.99996	0.94984	-0.07424	0.29013	0.75244	0.87378	-0.19465	0.038911	0.086671	0.73089	-0.45227	0.072463	0.27658	0.79942	-0.32298	0.2392	0.67869	0.85587	-0.22454	0.20884	0.92646	0.85596	-0.22438	0.069844	0.17098	1.4336	0.51959
877.0353	6.48	oolomics Pc	0.72019	0.99951	1.0984	0.13534	0.18585	0.99998	0.82409	-0.27913	0.82038	0.99941	0.97618	-0.03478	0.012423	0.20085	0.69017	-0.53497	0.10523	0.29862	0.79153	-0.33729	0.029251	0.13051	0.70882	-0.49651	0.45756	0.99391	0.89957	-0.15269	0.37617	0.99956	0.85679	-0.22298	0.35481	0.63216	0.88007	-0.1843
642.3883	3.1	oolomics Pc	0.72069	0.99951	1.0658	0.09188	0.063287	0.99998	0.78167	-0.35538	0.32756	0.99941	0.89773	-0.15564	0.004834	0.18788	0.61862	-0.69288	0.00019	0.002691	0.4653	-1.1038	0.001515	0.045425	0.56101	-0.8339	0.28586	0.90409	0.81536	-0.2945	0.18683	0.98578	0.83203	-0.26529	0.14147	0.39464	1.5315	0.61496
1071.53	6.34	domics Pos	0.7207	0.99987	1.0561	0.07878	0.82736	0.99994	0.97559	-0.03565	0.96029	0.99996	0.99825	-0.00252	0.26991	0.73042	0.84924	-0.23576	0.052498	0.10832	0.73339	-0.44734	0.02723	0.14544	0.67172	-0.57407	0.20369	0.64891	0.80921	-0.30542	0.081919	0.80297	0.76787	-0.38107	0.96188	0.97182	1.0322	0.045738
283.2436	6.3	olomics Ne	0.72093	0.99918	0.97802	-0.03207	0.13417	0.55622	0.75499	-0.40548	0.00244	0.99995	0.67328	-0.57072	0.020884	0.99985	0.49005	-1.029	0.000876	0.11985	0.42991	-1.2179	0.000562	0.13901	0.28681	-1.8018	0.002219	0.22316	0.41898	-1.255	0.015674	0.99512	0.4173	-1.2608	0.007962	0.99941	0.42809	-1.224
539.3137	5.11	oolomics Pc	0.72102	0.99951	0.89923	-0.15323	0.71577	0.99998	0.82539	-0.27685	0.94842	0.99941	0.88971	-0.16859	0.56648	0.99905	1.2624	0.33615	0.30256	0.67211	1.4686	0.55444	0.02184	0.10951	2.6123	1.3853	0.021992	0.31703	2.7094	1.438	0.33105	0.99956	1.6855	0.75315	0.24458	0.51325	1.5381	0.62114
170.0668	10.16	domics Pos	0.72159	0.99987	1.0163	0.023297	0.27521	0.99994	0.9243	-0.11356	0.26388	0.99996	0.94493	-0.08172	0.065973	0.43825	0.89682	-0.1571	0.00063	0.003899	0.75316	-0.40898	0.13838	0.40744	0.86716	-0.20563	0.7965	0.95175	0.98499	-0.02182	0.14073	0.89982	0.91689	-0.12519	0.057934	0.15513	1.1371	0.18535
425.3114	5.72	domics Pos	0.7219	0.99987	1.0266	0.037828	0.11648	0.99994	0.80921	-0.30542	0.50106	0.99996	0.90999	-0.13608	0.37842	0.8273	0.8863	-0.17414	0.033727	0.077516	0.72982	-0.45438	0.351	0.61843	0.86247	-0.21346	0.46872	0.80647	0.90143	-0.14971	0.37451	0.96776	0.854	-0.2277	0.23853	0.34662	0.84082	-0.25012
413.7862	5.72	domics Pos	0.72198	0.99987	0.96504	-0.05134	0.3198	0.99994	0.87582	-0.1913	0.36702	0.99996	0.88029	-0.18395	0.22746	0.68384	0.85177	-0.23146	0.028499	0.068258	0.7277	-0.45858	0.53778	0.74246	0.93323	-0.09969	0.86664	0.97507	0.98441	-0.02267	0.90843	0.98239	0.99297	-0.01018	0.077174	0.17834	1.8853	0.91481
104.1091	9.96	domics Pos	0.72307	0.99987	0.98637	-0.01979	0.010517	0.99994	0.90934	-0.13711	0.048071	0.99996	0.9212	-0.11841	0.092377	0.50463	0.84339	-0.24573	0.000137	0.001285	0.71517	-0.48364	0.17341	0.45968	0.87288	-0.19615	0.85638	0.97126	0.99309	-0.01001	0.48928	0.96776	0.96482	-0.05167	0.021181	0.10765	1.099	0.13618
358.9755	4.71	olomics Ne	0.72322	0.99918	1.0167	0.023926	0.037372	0.45581	0.85447	-0.22691	0.73008	0.99995	0.98104	-0.02761	0.77404	0.99985	0.98114	-0.02748	0.9137	0.99927	1.0061	0.00877	0.55667	0.87267	1.0444	0.062665	0.57953	0.85694	1.0331	0.046976	0.71603	0.99512	0.97911	-0.03045	0.49673	0.99941	0.9619	-0.05604
990.7842	0.5	domics Pos	0.72339	0.99987	1.0738	0.1027	0.32558	0.99994	0.87383	-0.19457	0.75573	0.99996	0.96902	-0.04541	0.95972	0.9937	1.0042	0.006101	0.015869	0.043689	0.77212	-0.37311	0.96647	0.98297	1.0031	0.004476	0.43599	0.79597	1.0896	0.12378	0.62007	0.97813	0.95374	-0.06833	0.044027	0.13978	1.1887	0.2494
510.7606	2.36	oolomics Pc	0.72346	0.99951	1.2368	0.30662	0.84092	0.99998	0.80461	-0.31364	0.54327	0.99941	0.57478	-0.79891	0.29026	0.7742	0.41948	-1.2533	0.020251	0.084717	0.10498	-3.2518	0.16558	0.42233	0.27248	-1.8758	0.38762	0.97696	0.4905	-1.0277	0.47943	0.99956	0.59612	-0.74633	0.65326	0.8417	1.2963	0.37444
391.7759	4.95	domics Pos	0.72359	0.99987	1.0353	0.050056	0.29623	0.99994	0.91058	-0.13515	0.73426	0.99996	0.97494	-0.03661	0.84041	0.98707	0.98997	-0.01455	0.045858	0.097577	0.74145	-0.43157	0.29194	0.57461	0.84803	-0.23781	0.37695	0.76056	0.90267	-0.14773	0.40764	0.96776	0.90622	-0.14207	0.024427	0.11132	1.243	0.31378
642.2469	5.11	oolomics Pc	0.72366	0.99951	0.89429	-0.16119	0.93082	0.99998	0.95784	-0.06214	0.97963	0.99941	0.96358	-0.05352	0.87832	0.99905	1.0788	0.10944	0.37677	0.77319	1.2421	0.31278	0.061993	0.21506	1.4908	0.57613	0.019137	0.30136	1.7529	0.80971	0.40709	0.99956	1.1403	0.18945	0.44178	0.70964	1.1161	0.15846
294.9402	11.98	oolomics Pc	0.72392	0.99951	0.98701	-0.01886	0.15046	0.99998	1.0874	0.12083	0.36472	0.99941	0.9479	-0.07719	0.88865	0.99905	0.9971	-0.0042	0.99088	0.9989	1.0029	0.004112	0.20184	0.47698	1.0667	0.093108	0.32471	0.93893	1.0144	0.020564	0.048749	0.67731	0.95489	-0.0666	0.79176	0.9129	1.0149	0.021368
343.1959	2.18	domics Pos	0.7246	0.99987	0.93878	-0.09115	0.9195	0.99994	0.96245	-0.05522	0.2619	0.99996	0.80278	-0.31692	0.49368	0.88874	0.8793	-0.18557	0.000248	0.001942	0.23901	-2.0648	0.048284	0.21309	0.62058	-0.6883	0.19874	0.64891	0.74841	-0.4181	0.22694	0.94286	0.74553	-0.42367	0.31649	0.42702	0.81692	-0.29173
478.2941	1.16	domics Pos	0.72463	0.99987	0.88709	-0.17284	0.60672	0.99994	0.79947	-0.32289	0.95373	0.99996	0.92352	-0.11479	0.65745	0.95652	1.1954	0.25745	0.18967	0.28239	1.5399	0.62281	0.019995	0.12102	2.3661	1.2425	0.01139	0.3324	2.7916	1.4811	0.28859	0.94825	1.5353	0.6185	0.10288	0.20549	1.9763	0.98283
118.0875	0.33	oolomics Pc	0.72471	0.99951	0.89188	-0.16508	0.8354	0.99998	0.92948	-0.10551	0.84042	0.99941	0.9139	-0.12988	0.72409	0.99905	1.1311	0.1777	0.045519	0.15688	1.6385	0.71241	0.055531	0.19968	1.8029	0.85028	0.015735	0.29145	2.0842	1.0595	0.43892	0.99956	1.4105	0.49617	0.44051	0.70925	1.1878	0.24829
897.5426	6.47	oolomics Pc	0.72472	0.99951	1.1409	0.19018	0.34455	0.99998	0.83203	-0.2653	0.84058	0.99941	0.96346	-0.05371	0.014852	0.20331	0.63301	-0.6597	0.17405	0.4494	0.75405	-0.40726	0.03777	0.15451	0.65817	-0.60347	0.5491	0.99391	0.89476	-0.16043	0.57267	0.99956	0.9377	-0.09281	0.56317	0.79019	0.89923	-0.15323
440.3036	5.46	oolomics Pc	0.72515	0.99951	0.96905	-0.04536	0.94111	0.99998	1.0407	0.057592	0.74783	0.99941	0.97283	-0.03974	0.34925	0.84312	1.1312	0.17783	0.000577	0.006226	0.43933	-1.1866	0.43666	0.7427	0.89685	-0.15706	0.54563	0.99391	1.2503	0.32224	0.94676	0.99956	1.0786	0.10917	0.081017	0.33015	1.2681	0.34271
184.0285	10.23	domics Pos	0.72552	0.99987	0.97543	-0.0359	0.92335	0.99994	0.99988	-0.00017	0.33972	0.99996	0.92877	-0.10661	0.050241	0.38141	0.86622	-0.2072	0.005895	0.020659	0.78814	-0.34347	0.36121	0.62293	0.93222	-0.10126	0.65687	0.89228	0.96748	-0.04769	0.45861	0.96776	1.0361	0.051161	0.001659	0.07528	1.2935	0.37132
954.7613	0.54	domics Pos	0.72614	0.99987	1.0358	0.050762	0.35308	0.99994	0.87417	-0.19402	0.43319	0.99996	0.89632	-0.15792	0.34588	0.79994	0.88153	-0.18192	0.030215	0.071184	0.75649	-0.4026	0.71311	0.85131	0.95557	-0.06556	0.53275	0.84189	0.919	-0.12187	0.24169	0.94365	0.85261	-0.23005	0.1409	0.24586	1.2126	0.27808
306.253	6.41	oolomics Pc	0.72687	0.99951	0.9246	-0.11311	0.72872	0.99998	0.91427	-0.12931	0.42675	0.99941	0.83111	-0.26688	0.47168	0.95078	0.85431	-0.22717	7.88E-05	0.001338	0.15895	-2.6533	0.023975	0.1146	0.58674	-0.7692	0.1261	0.66647	0.66856	-0.58088	0.15226	0.93461	0.6832	-0.54961	0.12023	0.36992	0.64593	-0.63054
196.8659	9.91	domics Pos	0.72816	0.99987	0.97892	-0.03074	0.11297	0.99994	0.89048	-0.16735	0.002026	0.99996	0.77207	-0.37319	0.047884	0.37667	0.83815	-0.25471	0.000526	0.003435	0.74684	-0.42113	0.10481	0.34483	0.86146	-0.21515	0.72726	0.92597	0.98018	-0.02889	0.002399	0.48691	0.84569	-0.2418	0.075682	0.1778	1.15	0.2016
1186.857	5.29	domics Pos	0.72845	0.99987	1.1398	0.18883	0.45345	0.99994	0.87809	-0.18756	0.55319	0.99996	0.90796	-0.13929	0.18126	0.61994	0.7978	-0.32591	0.025064	0.061856	0.65202	-0.61701	0.3908	0.64146	0.84783	-0.23815	0.75512	0.93748	1.1046	0.14354	0.82251	0.97988	1.0112	0.016118	0.042932	0.13814	1.442	0.52804
121.1023	6.98	oolomics Pc	0.72939	0.99951	1.0792	0.10994	0.54797	0.99998	0.91576	-0.12697	0.056083	0.99941	0.77041	-0.37631	0.045365	0.2866	0.61784	-0.69468	0.001046	0.009578	0.39491	-1.3404	6.64E-05	0.034543	0.3637	-1.4592	0.003434	0.20152	0.53349	-0.90647	0.02385	0.51812	0.54662	-0.87139	0.17439	0.43668	0.78864	-0.34256
795.6049	8.87	oolomics Pc	0.72947	0.99951	1.0282	0.040186	0.20501	0.99998	1.0986	0.13564	0.99119	0.99941	1.0043	0.006213	0.61152	0.99905	1.0579	0.081251	0.39605	0.79545	1.0692	0.096563	0.88011	0.96362	0.99061	-0.01361	0.024171	0.32473	1.1449	0.19517	0.36034	0.99956	0.93347	-0.09932	0.10765	0.36185	0.8972	-0.1565
227.2031	6.96	oolomics Pc	0.73062	0.99951	0.9421	-0.08604	0.52643	0.99998	0.88678	-0.17335	0.03755	0.99941	0.6437	-0.63554	0.0302	0.24688	0.50919	-0.97372	0.00492	0.028969	0.43449	-1.2026	0.004149	0.054724	0.3835	-1.3827	0.001768	0.16989	0.37611	-1.4108	0.014983	0.47452	0.43076	-1.2151	0.028741	0.2688	0.51362	-0.96123
1072.532	6.35	domics Pos	0.73093	0.99987	1.0836	0.11585	0.70871	0.99994	0.97729	-0.03315	0.28499	0.99996	0.93644	-0.09475	0.24065	0.70153	0.888	-0.17137	0.005835	0.020563	0.71184	-0.49038	0.008143	0.084185	0.7271	-0.45978	0.028456	0.35164	0.73595	-0.44232	0.56151	0.9703	0.93539	-0.09636	0.92547	0.94454	1.0772	0.10731
300.2301	5.65	oolomics Pc	0.7311	0.99951	1.0653	0.091326	0.64232	0.99998	0.91803	-0.12339	0.58473	0.99941	0.90434	-0.14507	0.32932	0.82464	0.8142	-0.29654	8.94E-05	0.001482	0.27396	-1.868	0.068989	0.23242	0.66018	-0.59907	0.16855	0.74367	0.73422	-0.44572	0.51075	0.99956	0.8843	-0.17739	0.84121	0.93671	1.05	0.070434
799.9786	12.04	olomics Ne	0.73124	0.99918	1.0266	0.037894	0.40971	0.8002	0.8652	-0.2089	0.91312	0.99995	1.0109	0.015594	0.69432	0.99985	1.0531	0.074664	0.57098	0.99927	1.1304	0.17681	0.88121	0.9778	0.97204	-0.04091	0.039919	0.364	1.6003	0.67838	0.66272	0.99512	1.0849	0.11753	0.55198	0.99941	1.0838	0.11612
1072.715	9.05	oolomics Pc	0.73162	0.99951	1.1225	0.16674	0.15151	0.99998	0.80275	-0.31698	0.79041	0.99941	0.9768	-0.03386	0.006335	0.1948	0.67352	-0.57021	0.030633	0.1171	0.6556	-0.60912	0.1094	0.31845	0.7513	-0.41254	0.7831	0.99391	0.98886	-0.01617	0.91667	0.99956	1.0473	0.06665	0.20209	0.46778	1.3319	0.41354
500.2757	5.08	oolomics Pc	0.73185	0.99951	0.90246	-0.14806	0.98242	0.99998	0.99269	-0.01059	0.81988	0.99941	0.96589	-0.05007	0.57818	0.99905	1.2883	0.36542	0.077185	0.23452	2.0935	1.0659	0.01127	0.083534	2.5963	1.3764	0.006621	0.21955	2.896	1.5341	0.297	0.99956	1.5634	0.64471	0.18031	0.44542	1.6902	0.75717
655.5438	4.84	domics Pos	0.73197	0.99987	1.028	0.039843	0.59819	0.99994	0.93105	-0.10307	0.66275	0.99996	0.94524	-0.08124	0.80379	0.97856	0.96806	-0.04684	0.76004	0.79415	0.96771	-0.04735	0.20975	0.50173	1.2101	0.27512	0.007818	0.32129	1.5264	0.61018	0.19937	0.92582	1.2248	0.29252	0.088397	0.18936	1.3829	0.46773
852.6817	6.38	domics Pos	0.73206	0.99987	0.94578	-0.08043	0.59168	0.99994	0.91523	-0.1278	0.61279	0.99996	0.92011	-0.12013	0.45374	0.87408	0.88118	-0.1825	0.041071	0.089847	0.69714	-0.52047	0.19906	0.48694	0.8086	-0.3065	0.33463	0.74176	0.85183	-0.23136	0.5989	0.97073	0.91713	-0.1248	0.67747	0.75932	0.93446	-0.0978
199.1704	6.96	oolomics Pc	0.73237	0.99951	0.95806	-0.06181	0.56229	0.99998	0.91274	-0.13172	0.027789	0.99941	0.64306	-0.63698	0.013071	0.20085	0.48463	-1.045	0.002431	0.017564	0.47053	-1.0876	0.001576	0.045425	0.34704	-1.5268	0.004566	0.20152	0.39717	-1.3322	0.023335	0.51791	0.44008	-1.1842	0.017508	0.2688	0.50069	-0.99802
456.2987	1.35	domics Pos	0.73303	0.99987	1.0946	0.13041	0.84783	0.99994	0.9045	-0.1448	0.92194	0.99996	0.93485	-0.0972	0.84065	0.98707	1.0114	0.016392	0.67672	0.72543	0.84914	-0.23593	0.080033	0.29262	1.6142	0.69078	0.01975	0.33809	2.1568	1.1089	0.079427	0.79699	1.5948	0.67341	0.031888	0.12208	1.91	0.9336
1179.776	10.02	domics Pos	0.73459	0.99987	1.0167	0.023825	0.17277	0.99994	0.89178	-0.16524	0.35246	0.99996	0.94589	-0.08026	0.10813	0.532	0.85423	-0.22731	0.00079	0.004664	0.7275	-0.45898	0.47227	0.69501	0.93063	-0.10372	0.79359	0.95091	1.0239	0.034098	0.98435	0.99507	1.0002	0.000315	0.031147	0.12145	1.2046	0.26859
397.3829	9	oolomics Pc	0.7351	0.99951	1.1342	0.18165	0.9072	0.99998	1.075	0.10438	0.89119	0.99941	1.0467	0.065901	0.49633	0.97169	1.2604	0.33385	0.30352	0.67331	1.4083	0.49395	0.025176	0.11872	1.6966	0.76266	0.080899	0.58181	1.5669	0.64792	0.24675	0.99956	1.342	0.42443	0.30386	0.58116	1.3209	0.40157
467.3329	2.2	domics Pos	0.73511	0.99987	0.91696	-0.12507	0.86844	0.99994	0.93371	-0.09896	0.25389	0.99996	0.76309	-0.39007	0.63443	0.94754	0.87792	-0.18784	0.013305	0.038094	0.33221	-1.5898	0.62144	0.79782	0.87697	-0.1894	0.22073	0.66412	1.3232	0.40402	0.45849	0.96776	1.1692	0.2255	0.051358	0.14811	1.6559	0.72762
326.3419	3.75	domics Pos	0.73526	0.99987	0.97826	-0.03171	0.1005	0.99994	0.77167	-0.37394	0.13959	0.99996	0.81411	-0.2967	0.0372	0.34015	0.81691	-0.29175	0.00238	0.01031	0.79811	-0.32533	0.002318	0.061655	0.61155	-0.70945	0.087797	0.51503	0.72148	-0.47096	0.001317	0.41831	0.68688	-0.54188	0.37199	0.48146	0.91755	-0.12414
761.578	4.94	domics Pos	0.73595	0.99987	1.0299	0.042568	0.53177	0.99994	0.95178	-0.0713	0.62116	0.99996	0.96418	-0.05262	0.20989	0.65763	0.8898	-0.16845	0.003712	0.014457	0.74398	-0.42666	0.39316	0.64284	0.9336	-0.09912	0.62295	0.87521	1.0408	0.05775	0.39739	0.96776	1.045	0.063562	0.013375	0.098791	1.2264	0.29441
161.1322	6.48	oolomics Pc	0.73638	0.99951	0.96134	-0.05688	0.6242	0.99998	0.95459	-0.06705	0.11677	0.99941	0.68261	-0.55086	0.052679	0.29933	0.56049	-0.83525	0.003999	0.024996	0.46379	-1.1084	0.019789	0.10407	0.42257	-1.2427	0.018723	0.30136	0.41387	-1.2727	0.011103	0.47452	0.40465	-1.3052	0.031499	0.2688	0.41431	-1.2712
310.9805	3.99	olomics Ne	0.73643	0.99918	1.0141	0.020218	0.02507	0.40327	0.88475	-0.17666	0.29953	0.99995	1.0381	0.053889	0.85	0.99985	0.99275	-0.01049	0.91515	0.99927	1.0112	0.016072	0.51466	0.86542	1.049	0.068947	0.22357	0.60454	1.0826	0.11456	0.65035	0.99512	0.97759	-0.03271	0.76974	0.99941	0.98545	-0.02115
386.8974	10.16	domics Pos	0.73726	0.99987	1.0318	0.045144	0.093269	0.99994	0.83398	-0.26191	0.78025	0.99996	0.96814	-0.04672	0.84396	0.98707	1.0114	0.016348	0.033343	0.076914	0.75389	-0.40758	0.8451	0.922	0.97762	-0.03265	0.65126	0.89057	1.0393	0.055562	0.95371	0.98838	1.0277	0.039349	0.13929	0.24465	1.1818	0.24096
887.5584	4.82	lomics Neg	0.73729	0.99977	1.5736	0.65409	0.84869	0.99904	1.4237	0.50967	0.76345	0.99994	1.0054	0.007776	0.090693	0.58182	1.8667	0.9005	0.92399	0.99989	2.0443	1.0316	0.65526	0.99653	2.0091	1.0065	0.03239	0.37635	0.58993	-0.76139	0.07178	0.46971	0.52593	-0.92706	0.19653	0.42853	1.8723	0.90478
825.5533	4.76	domics Pos	0.73781	0.99987	1.0639	0.089359	0.98671	0.99994	1.0014	0.001995	0.76029	0.99996	1.0338	0.047952	0.46945	0.88476	0.90845	-0.13853	0.027796	0.067037	0.70323	-0.50794	0.43517	0.6683	0.87379	-0.19465	0.45447	0.80208	0.89468	-0.16056	0.95198	0.98838	0.99033	-0.01402	0.26052	0.36997	1.1631	0.21799
738.8259	10.13	domics Pos	0.73789	0.99987	1.0209	0.029824	0.57565	0.99994	0.94483	-0.08187	0.18364	0.99996	0.85844	-0.22021	0.7157	0.96431	0.95921	-0.06008	0.021462	0.055257	0.7444	-0.42585	0.58976	0.77668	0.93516	-0.09672	0.83757	0.96659	1.0078	0.011168	0.38826	0.96776	0.9092	-0.13733	0.38952	0.49857	1.1208	0.16457
899.0492	6.47	oolomics Pc	0.73809	0.99951	1.1028	0.14115	0.13463	0.99998	0.76059	-0.3948	0.44084	0.99941	0.88968	-0.16864	0.022484	0.22443	0.62749	-0.67234	0.18577	0.46851	0.78128	-0.3561	0.026021	0.12085	0.70075	-0.51302	0.48507	0.99391	0.88232	-0.18063	0.72123	0.99956	0.97934	-0.03012	0.38913	0.66222	0.85395	-0.22777
566.3452	5.43	olomics Ne	0.73839	0.99918	1.0867	0.11992	0.97002	0.99378	1.0098	0.014136	0.76468	0.99995	1.0534	0.075006	0.73156	0.99985	1.0693	0.096611	0.59715	0.99927	1.2067	0.27112	0.066536	0.51513	1.8046	0.85171	0.036586	0.35744	1.8816	0.91195	0.30531	0.99512	1.2973	0.37553	0.14742	0.99941	1.663	0.73379
792.8711	11.96	olomics Ne	0.73849	0.99918	1.0126	0.017997	0.11815	0.5367	0.92821	-0.10748	0.87008	0.99995	0.99479	-0.00753	0.60661	0.99985	0.97449	-0.03728	0.53892	0.99927	1.0279	0.039706	0.029438	0.47167	1.1295	0.17569	0.85905	0.95689	1.0104	0.014984	0.91246	0.99512	1.0127	0.018264	0.46987	0.99941	0.97529	-0.03609
153.128	2.81	domics Pos	0.73882	0.99987	1.0213	0.030367	0.3247	0.99994	0.84871	-0.23666	0.27522	0.99996	0.84297	-0.24644	0.012564	0.22648	0.57583	-0.79629	0.000152	0.00138	0.25232	-1.9867	0.00215	0.060287	0.37837	-1.4021	0.006513	0.3088	0.51764	-0.94998	0.037508	0.64014	0.60492	-0.72518	0.78655	0.84127	1.0695	0.09697
725.5086	10.78	olomics Ne	0.73885	0.99918	1.0378	0.053502	0.36228	0.76696	1.1601	0.21425	0.39557	0.99995	1.1067	0.14629	0.052852	0.99985	1.1953	0.25733	0.92743	0.99927	1.0035	0.004997	0.04528	0.48405	1.325	0.40595	0.046524	0.38697	1.5719	0.65249	0.85482	0.99512	1.0437	0.061696	0.50971	0.99941	0.95935	-0.05988
1133.588	1.29	domics Pos	0.73935	0.99987	0.96087	-0.05758	0.3347	0.99994	0.87149	-0.19844	0.65513	0.99996	0.94311	-0.08451	0.44337	0.86677	0.89908	-0.15347	0.043936	0.094642	0.62324	-0.68214	0.80151	0.89913	1.051	0.071727	0.06588	0.46086	1.2002	0.26333	0.48434	0.96776	1.0881	0.12188	0.038858	0.13241	1.4591	0.54504
1090.732	10.06	domics Pos	0.73941	0.99987	0.97948	-0.02992	0.12103	0.99994	0.90055	-0.15111	0.029193	0.99996	0.84346	-0.24561	0.18086	0.6196	0.90355	-0.14633	0.013909	0.039459	0.73737	-0.43955	0.17015	0.45689	0.87597	-0.19105	0.50976	0.83279	0.94691	-0.0787	0.47307	0.96776	0.9507	-0.07294	0.084732	0.186	1.1891	0.24992
898.0441	6.47	oolomics Pc	0.73944	0.99951	1.1149	0.15688	0.14091	0.99998	0.7876	-0.34446	0.78947	0.99941	0.96616	-0.04967	0.008266	0.19702	0.64136	-0.6408	0.15941	0.41986	0.77514	-0.36747	0.012782	0.085685	0.69132	-0.53257	0.57469	0.99391	0.92077	-0.11908	0.49886	0.99956	0.90525	-0.14362	0.9407	0.97538	1.0053	0.007618
281.2487	6.48	oolomics Pc	0.73948	0.99951	0.9602	-0.05859	0.63635	0.99998	0.95544	-0.06577	0.12345	0.99941	0.70531	-0.50368	0.05446	0.30353	0.57039	-0.80998	0.006247	0.034132	0.46351	-1.1093	0.013027	0.085844	0.43361	-1.2055	0.018043	0.30136	0.3933	-1.3463	0.013045	0.47452	0.41313	-1.2753	0.028464	0.2688	0.39314	-1.3469
171.1397	6.96	oolomics Pc	0.73957	0.99951	0.9651	-0.05125	0.52145	0.99998	0.91261	-0.13193	0.038784	0.99941	0.70744	-0.49933	0.022693	0.22443	0.52496	-0.92973	0.0004	0.004807	0.45378	-1.1399	0.000882	0.042705	0.36919	-1.4375	0.001332	0.16989	0.3984	-1.3277	0.011478	0.47452	0.46763	-1.0966	0.013222	0.2688	0.49433	-1.0165
822.5983	5.16	domics Pos	0.73994	0.99987	1.0426	0.060232	0.5975	0.99994	0.93774	-0.09274	0.74293	0.99996	0.95836	-0.06136	0.60569	0.93327	0.93195	-0.10168	0.032969	0.076187	0.6938	-0.5274	0.058529	0.24091	0.77747	-0.36314	0.14061	0.60462	0.81807	-0.28971	0.089864	0.82491	0.77482	-0.36806	0.72934	0.79815	0.95596	-0.06498
618.4281	6.96	oolomics Pc	0.74004	0.99951	0.97257	-0.04013	0.64017	0.99998	0.96411	-0.05273	0.033648	0.99941	0.66491	-0.58877	0.060665	0.32444	0.50809	-0.97685	0.002362	0.017364	0.43532	-1.1998	0.002256	0.045856	0.31469	-1.668	0.017299	0.29966	0.40975	-1.2872	0.019994	0.51556	0.41362	-1.2736	0.025918	0.2688	0.44802	-1.1584
236.9797	12	olomics Ne	0.74018	0.99918	0.98412	-0.02309	0.003392	0.34605	0.84492	-0.24311	0.79994	0.99995	0.987	-0.01888	0.72168	0.99985	1.0545	0.076538	0.98898	0.99927	1.0048	0.006854	0.76249	0.94811	0.98398	-0.0233	0.60891	0.86704	1.0219	0.031249	0.73015	0.99512	0.98052	-0.02839	0.42442	0.99941	0.95182	-0.07124
852.5723	4.95	lomics Neg	0.74033	0.99977	1.2056	0.26979	0.88235	0.99904	1.1024	0.14059	0.33878	0.99994	0.89613	-0.15822	0.18511	0.58182	1.4094	0.49513	0.95587	0.99989	1.1829	0.24231	0.99316	0.99653	1.1976	0.26014	0.078804	0.41257	0.7	-0.51457	0.044672	0.46971	0.54034	-0.88806	0.30197	0.50215	0.91445	-0.12903
410.7619	4.61	domics Pos	0.74106	0.99987	1.0668	0.093245	0.99296	0.99994	0.99914	-0.00125	0.51424	0.99996	0.89293	-0.16338	0.83616	0.98621	0.97165	-0.04149	0.86912	0.88965	0.98158	-0.02682	0.046113	0.20637	1.4014	0.48691	0.078243	0.49476	1.4844	0.56988	0.77161	0.97988	1.074	0.10304	0.021696	0.1083	1.5775	0.65765
177.0295	0.32	oolomics Pc	0.74134	0.99951	0.9312	-0.10284	0.73894	0.99998	1.0842	0.11667	0.26449	0.99941	1.3049	0.38398	0.081925	0.37784	1.6034	0.68112	0.070504	0.21604	1.6549	0.72676	0.019291	0.10212	1.9534	0.96598	0.000599	0.16989	2.4603	1.2988	0.18481	0.98334	1.4526	0.5386	0.27919	0.55383	1.166	0.22153
926.6353	10.26	olomics Ne	0.74196	0.99918	0.99034	-0.014	0.008418	0.34605	0.86322	-0.2122	0.43069	0.99995	0.91223	-0.13254	0.76865	0.99985	0.98336	-0.02421	0.042392	0.99927	0.86699	-0.20591	0.54302	0.87267	0.96856	-0.04609	0.018564	0.29108	0.7198	-0.47433	0.010461	0.99512	0.84041	-0.25084	0.15046	0.99941	0.92468	-0.11297
363.2735	2.28	domics Pos	0.74215	0.99987	1.0669	0.093381	0.40666	0.99994	0.83687	-0.25692	0.07202	0.99996	0.69041	-0.53446	0.032249	0.31368	0.57182	-0.80637	0.002641	0.011101	0.45087	-1.1492	0.007338	0.082061	0.43765	-1.1922	0.025181	0.33809	0.46205	-1.1139	0.020782	0.52955	0.4437	-1.1724	0.053179	0.14998	0.57719	-0.79288
448.3454	2.85	domics Pos	0.74268	0.99987	1.0501	0.070539	0.55691	0.99994	0.93159	-0.10223	0.1106	0.99996	0.80122	-0.31973	0.013147	0.22835	0.58192	-0.78112	0.002081	0.009337	0.48194	-1.0531	0.006973	0.081806	0.47119	-1.0856	0.024983	0.33809	0.56652	-0.8198	0.03672	0.64014	0.51961	-0.9445	0.15816	0.26485	0.70155	-0.51137
940.524	4.59	domics Pos	0.74307	0.99987	0.97201	-0.04096	0.1332	0.99994	0.88505	-0.17617	0.30435	0.99996	0.87364	-0.19488	0.11315	0.53238	0.85252	-0.23019	0.019726	0.051731	0.70833	-0.49751	0.62623	0.8002	0.95096	-0.07254	0.24362	0.67954	1.1235	0.16803	0.63734	0.97988	0.95828	-0.06148	0.50347	0.60483	1.1036	0.14224
949.8097	10.03	domics Pos	0.74308	0.99987	1.0184	0.02632	0.63478	0.99994	1.0303	0.043106	0.60643	0.99996	0.96601	-0.0499	0.842	0.98707	0.9891	-0.01581	0.005687	0.020189	0.79475	-0.33143	0.80385	0.90041	0.98779	-0.01772	0.55336	0.85214	1.075	0.10429	0.23351	0.94365	0.89632	-0.15791	0.003994	0.085451	1.1965	0.25883
395.221	6.83	oolomics Pc	0.74334	0.99951	0.96162	-0.05646	0.40568	0.99998	0.88663	-0.1736	0.020653	0.99941	0.73701	-0.44025	0.037218	0.26405	0.63649	-0.65178	0.001069	0.009752	0.57496	-0.79846	0.008642	0.075866	0.46613	-1.1012	0.004322	0.20152	0.51046	-0.97013	0.005646	0.47452	0.49007	-1.0289	0.021268	0.2688	0.56886	-0.81385
1032.511	4.77	domics Pos	0.7434	0.99987	0.97654	-0.03424	0.092361	0.99994	0.9043	-0.14512	0.53264	0.99996	1.046	0.064917	0.61268	0.93755	0.9698	-0.04425	0.011452	0.034062	0.71776	-0.47842	0.2028	0.49159	0.8913	-0.16602	0.45258	0.80204	1.1008	0.13858	0.89505	0.98023	0.99995	-6.6E-05	0.28711	0.39751	1.0946	0.13047
645.5026	2.28	domics Pos	0.74373	0.99987	1.1107	0.15144	0.52926	0.99994	0.90476	-0.14439	0.052517	0.99996	0.63403	-0.65737	0.02956	0.30146	0.44217	-1.1773	0.001616	0.007748	0.33302	-1.5863	0.015359	0.10458	0.31893	-1.6487	0.054732	0.41572	0.36291	-1.4623	0.019362	0.52955	0.32674	-1.6138	0.032045	0.12241	0.38933	-1.3609
798.7921	10.11	domics Pos	0.74401	0.99987	1.0188	0.026825	0.39175	0.99994	1.0768	0.10675	0.63483	0.99996	0.96174	-0.05629	0.45115	0.87374	1.1098	0.15032	0.006312	0.021701	0.80411	-0.31454	0.53407	0.73985	0.94725	-0.07818	0.22984	0.67417	1.1245	0.16932	0.011521	0.52955	1.175	0.23271	0.019374	0.10555	1.4073	0.4929
741.5413	8.31	oolomics Pc	0.74421	0.99951	0.9809	-0.02782	0.37166	0.99998	0.95153	-0.07168	0.072259	0.99941	0.90712	-0.14064	0.05258	0.29933	0.88136	-0.18219	0.040485	0.14297	0.77691	-0.36419	0.094291	0.28642	0.71269	-0.48864	0.003983	0.20152	0.67731	-0.56211	0.030833	0.58535	0.68063	-0.55505	0.018189	0.2688	0.64737	-0.62734
518.3248	4.84	oolomics Pc	0.74459	0.99951	0.89274	-0.16369	0.87746	0.99998	0.88529	-0.17578	0.94333	0.99941	0.89419	-0.16135	0.83609	0.99905	1.0359	0.050885	0.65985	0.99213	1.1671	0.22294	0.082967	0.26187	2.1177	1.0825	0.038895	0.39092	2.5836	1.3694	0.38542	0.99956	1.5635	0.64475	0.065307	0.30704	2.1571	1.1091
715.1878	1.03	domics Pos	0.74487	0.99987	0.98829	-0.017	0.86187	0.99994	1.0174	0.024947	0.68844	0.99996	1.0553	0.077683	0.74937	0.97388	1.0422	0.059681	0.016945	0.045801	0.7014	-0.51169	0.17558	0.46179	1.2805	0.35673	0.012728	0.3324	1.6474	0.72019	0.81782	0.97988	1.0604	0.084619	0.13262	0.23764	1.2306	0.29937
446.973	11.99	olomics Ne	0.7455	0.99918	1.0162	0.023158	0.2799	0.70896	0.93688	-0.09406	0.60608	0.99995	1.0356	0.050484	0.097312	0.99985	1.1654	0.22085	0.77865	0.99927	1.0216	0.030783	0.18035	0.65203	1.0832	0.11523	0.003557	0.22316	1.2471	0.31862	0.88457	0.99512	0.99462	-0.00779	0.46156	0.99941	1.0827	0.1147

362.2732	5.01	olomics Po	0.74554	0.99951	1.045	0.06353	0.48971	0.99998	1.067	0.093618	0.7012	0.99941	0.97044	-0.04329	0.75295	0.99905	0.97528	-0.03612	0.035458	0.13082	1.2625	0.33629	0.1291	0.35797	1.2	0.26303	0.096647	0.60726	1.2906	0.36809	0.23078	0.99956	1.1421	0.19174	0.14063	0.39464	1.1768	0.23492
85.0291	0.34	olomics Po	0.74645	0.99951	0.86611	-0.20737	0.63308	0.99998	0.81766	-0.29042	0.42975	0.99941	0.74488	-0.42491	0.083946	0.38379	0.53005	-0.9158	0.083802	0.2488	0.49387	-1.0178	0.089503	0.27668	0.48636	-1.0399	0.043242	0.41783	0.44357	-1.1728	0.17342	0.96515	0.59007	-0.76105	0.041526	0.28426	0.42508	-1.2342
466.2991	1.33	lomics Pos	0.74658	0.99987	0.87634	-0.19043	0.78822	0.99994	0.83905	-0.25317	0.28377	0.99996	1.5863	0.66565	0.10241	0.52628	3.1339	1.648	0.016944	0.045801	3.8432	1.9423	0.016753	0.10983	3.9617	1.9861	0.006103	0.3088	4.7991	2.2628	0.6053	0.97261	1.3093	0.38883	0.46457	0.56751	1.1126	0.15391
837.6158	9.77	olomics Po	0.74678	0.99951	1.0204	0.029171	0.77329	0.99998	1.0213	0.030441	0.23433	0.99941	0.92595	-0.11099	0.86694	0.99905	0.99272	-0.01054	0.72291	0.99688	0.97614	-0.03484	0.01379	0.087577	0.84914	-0.23592	0.027732	0.33945	0.81277	-0.29908	0.005566	0.47452	0.77819	-0.3618	0.37827	0.65368	1.079	0.1097
347.1901	1.75	lomics Pos	0.74689	0.99987	1.1389	0.18762	0.27599	0.99994	0.75114	-0.41285	0.18925	0.99996	0.7182	-0.47754	0.11141	0.53238	0.65771	-0.60448	0.000835	0.004869	0.26755	-1.9021	0.028447	0.14839	0.45671	-1.1307	0.027076	0.34512	0.542	-0.88364	0.040001	0.64308	0.54252	-0.88225	0.11477	0.21925	0.63514	-0.65486
566.3218	5.1	olomics Po	0.74714	0.99951	0.98497	-0.02185	0.93344	0.99998	1.017	0.024253	0.87577	0.99941	1.0632	0.088371	0.43878	0.92082	1.142	0.19158	0.70683	0.99688	1.0919	0.12684	0.088606	0.27471	1.3108	0.39046	0.037876	0.38531	1.3223	0.40305	0.44489	0.99956	1.0756	0.1052	0.9503	0.97899	1.0183	0.026165
538.342	6.97	olomics Po	0.74718	0.99951	1.4527	0.53876	0.90543	0.99998	1.0387	0.054748	0.22382	0.99941	1.5384	0.62146	0.35811	0.8488	1.2929	0.37061	0.18365	0.46578	1.6726	0.74212	0.14373	0.38429	1.4739	0.55959	0.021946	0.31703	2.5014	1.3227	0.019956	0.51556	1.8163	0.86097	0.076409	0.32186	1.7452	0.80339
452.2744	1.33	lomics Neg	0.74727	0.99977	1.0451	0.063629	0.69768	0.99904	0.8707	-0.19974	0.82508	0.99994	0.94115	-0.0875	0.26774	0.58182	1.4958	0.58093	0.66387	0.99989	1.3632	0.44701	0.20611	0.99653	2.1706	1.1181	0.023954	0.30828	1.9006	0.92645	0.88862	0.94289	1.2711	0.34603	0.014895	0.38536	2.1588	1.1102
835.6654	6.05	lomics Pos	0.74786	0.99987	0.97117	-0.0422	0.16126	0.99994	0.83035	-0.26821	0.39736	0.99996	0.90952	-0.13683	0.1099	0.53208	0.83252	-0.26445	0.01461	0.041112	0.72475	-0.46444	0.21483	0.50657	0.80396	-0.31481	0.33404	0.74176	0.91501	-0.12814	0.16659	0.91806	0.85248	-0.23026	0.54018	0.63669	0.94288	-0.08486
466.1582	6.38	olomics Po	0.74786	0.99951	1.0237	0.033814	0.78859	0.99998	0.96818	-0.04666	0.9371	0.99941	0.98478	-0.02213	0.79396	0.99905	0.97256	-0.04014	0.000649	0.0067	0.41969	-1.2526	0.071131	0.23735	0.80736	-0.30872	0.51862	0.99391	0.92454	-0.1132	0.64196	0.99956	0.943	-0.08467	0.45352	0.71788	1.1115	0.15256
380.2567	4.75	olomics Po	0.74787	0.99951	1.0287	0.040885	0.71548	0.99998	1.0376	0.053183	0.75729	0.99941	0.96679	-0.04873	0.96167	0.99905	1.0015	0.002188	3.36E-05	0.000679	0.27504	-1.8623	0.15304	0.39973	0.83502	-0.26012	0.68636	0.99391	1.0267	0.038067	0.61017	0.99956	1.0469	0.066108	0.27712	0.55179	1.1148	0.15684
628.4321	2.28	lomics Pos	0.74873	0.99987	0.94637	-0.07952	0.26571	0.99994	0.81543	-0.29436	0.021753	0.99996	0.65789	-0.60407	0.03222	0.31368	0.61767	-0.6951	0.006033	0.020953	0.53491	-0.90262	0.05279	0.22547	0.54474	-0.87636	0.025041	0.33809	0.44112	-1.1808	0.0145	0.52955	0.46347	-1.1095	0.079055	0.18102	0.60204	-0.73207
1194.743	10.03	lomics Pos	0.74906	0.99987	1.0151	0.021616	0.2449	0.99994	0.88285	-0.17976	0.20031	0.99996	0.8929	-0.16343	0.028756	0.29796	0.81407	-0.29677	0.01079	0.032572	0.73121	-0.45163	0.19732	0.48532	0.83486	-0.2604	0.91738	0.98047	1.0039	0.005671	0.3301	0.95887	0.92482	-0.11275	0.12224	0.22677	1.1235	0.16803
465.3169	1.63	lomics Pos	0.74929	0.99987	0.95471	-0.06687	0.3459	0.99994	0.82987	-0.26904	0.38689	0.99996	0.85246	-0.23029	0.53644	0.90249	0.89655	-0.15754	7.69E-05	0.000865	0.19287	-2.3743	0.059949	0.24292	0.66611	-0.58617	0.91335	0.98003	1.0208	0.029714	0.91023	0.98248	1.0443	0.062556	0.11671	0.22106	1.3478	0.43056
925.5725	5.22	lomics Pos	0.74934	0.99987	1.0158	0.0226	0.66474	0.99994	0.95657	-0.06406	0.74852	0.99996	0.9735	-0.03875	0.78442	0.97526	0.97739	-0.03299	0.010114	0.030927	0.79595	-0.32926	0.67469	0.83174	0.95955	-0.05958	0.67933	0.90338	1.029	0.041287	0.90603	0.98217	1.0191	0.027307	0.67587	0.75819	1.0901	0.12447
606.7889	9.92	lomics Pos	0.74981	0.99987	1.0313	0.044452	0.55599	0.99994	1.058	0.08132	0.9727	0.99996	1.0038	0.005464	0.99432	0.99837	0.99854	-0.00211	0.028435	0.068147	0.73017	-0.4537	0.20688	0.49863	0.86413	-0.21067	0.95022	0.98957	1.0115	0.016519	0.56879	0.9703	1.1363	0.18429	0.37969	0.48828	1.1222	0.16637
815.6353	5.78	lomics Pos	0.74994	0.99987	1.0322	0.045696	0.6634	0.99994	0.94849	-0.07629	0.59976	0.99996	0.93942	-0.09016	0.30279	0.76085	0.87101	-0.19925	0.041062	0.089847	0.72902	-0.45597	0.097456	0.3309	0.80657	-0.31013	0.31241	0.72948	0.87489	-0.19282	0.25106	0.94365	0.86602	-0.20752	0.07967	0.18143	1.428	0.51404
520.9029	0.28	olomics Ne	0.74999	0.99918	0.98136	-0.02715	0.23765	0.66187	0.61717	-0.69626	0.064685	0.99995	0.91311	-0.13113	0.97439	0.99985	1.0003	0.000396	0.023008	0.91567	0.87593	-0.19111	0.41692	0.81191	0.84193	-0.24823	0.62752	0.87286	1.0522	0.07341	0.99862	0.99943	0.999	-0.00144	0.083546	0.99941	0.90242	-0.14813
633.3192	5.09	olomics Ne	0.75039	0.99918	1.0598	0.08378	0.64174	0.91859	0.86975	-0.20132	0.74679	0.99995	1.0455	0.064252	0.61276	0.99985	1.1192	0.16249	0.28245	0.99927	1.2712	0.34619	0.040489	0.48405	1.6711	0.74079	0.049209	0.39082	1.659	0.73028	0.28011	0.99512	1.3124	0.39218	0.32698	0.99941	1.2061	0.27036
1017.682	1.3	lomics Pos	0.75052	0.99987	1.1016	0.13966	0.87745	0.99994	1.0345	0.048942	0.96541	0.99996	0.89182	-0.16518	0.93094	0.98766	0.96304	-0.05434	0.85779	0.88041	0.8548	-0.22634	0.24783	0.53453	1.7163	0.77932	0.044356	0.38909	2.4884	1.3152	0.34427	0.96524	1.3449	0.4275	0.069463	0.17088	3.3456	1.7423
792.5556	5.35	lomics Pos	0.75066	0.99987	0.96609	-0.04978	0.20231	0.99994	0.87903	-0.18602	0.25372	0.99996	0.85532	-0.22546	0.096788	0.51634	0.78416	-0.35079	0.002956	0.012133	0.54318	-0.88049	0.009125	0.086817	0.64924	-0.62318	0.55529	0.85279	0.92704	-0.10929	0.41907	0.96776	1.0866	0.11983	0.22526	0.33278	1.1582	0.21192
275.9264	10.19	lomics Pos	0.75087	0.99987	0.97595	-0.03512	0.051218	0.99994	0.82404	-0.27922	0.24847	0.99996	0.90878	-0.13799	0.30888	0.76826	0.91506	-0.12806	0.001633	0.007774	0.70817	-0.49784	0.25563	0.54302	0.85461	-0.22665	0.97077	0.99324	0.99625	-0.00542	0.081459	0.8026	0.86897	-0.20263	0.17206	0.27977	1.1396	0.18855
154.9057	0.42	olomics Ne	0.75152	0.99918	1.0383	0.054177	0.83805	0.98874	0.98507	-0.02171	0.22409	0.99995	1.1298	0.17608	0.031459	0.99985	1.193	0.25463	0.075885	0.99927	1.3185	0.39893	0.14725	0.6202	1.2765	0.35217	0.027679	0.33786	1.2956	0.37366	0.045924	0.99512	1.2057	0.26983	0.24853	0.99941	1.1091	0.14943
790.5379	4.61	lomics Pos	0.75186	0.99987	1.0696	0.097014	0.63424	0.99994	1.0771	0.1072	0.81094	0.99996	1.0566	0.079484	0.43242	0.86342	0.87796	-0.18777	0.01075	0.03248	0.61173	-0.70904	0.14701	0.42205	0.77318	-0.37112	0.49489	0.82378	0.89251	-0.16407	0.44856	0.96776	0.88048	-0.18364	0.51578	0.61554	1.1117	0.15273
564.3046	1.13	lomics Pos	0.75201	0.99987	0.88408	-0.17775	0.94338	0.99994	1.0305	0.043363	0.75479	0.99996	0.87085	-0.1995	0.57465	0.91693	0.83123	-0.26668	0.89347	0.90994	0.98758	-0.01802	0.12303	0.38264	1.5638	0.64503	0.049936	0.40772	1.9391	0.95536	0.36815	0.96776	1.4224	0.50832	0.050179	0.14661	1.9179	0.9395
229.1933	4.73	lomics Pos	0.75209	0.99987	1.0245	0.034959	0.93417	0.99994	0.99735	-0.00383	0.94423	0.99996	1.0073	0.010543	0.14431	0.57187	0.90035	-0.15145	0.044922	0.096067	0.82745	-0.27325	0.21377	0.50527	0.91448	-0.12898	0.47746	0.80986	0.95911	-0.06023	0.40904	0.96776	0.92111	-0.11856	0.019595	0.10555	1.299	0.37738
749.037	4.65	lomics Pos	0.75226	0.99987	1.036	0.051077	0.091859	0.99994	0.79395	-0.33289	0.20861	0.99996	0.81763	-0.29048	0.038722	0.34213	0.73747	-0.43935	0.004489	0.016765	0.64041	-0.64293	0.054853	0.23111	0.67768	-0.56132	0.20809	0.65208	0.79599	-0.32918	0.23265	0.94365	0.85342	-0.22868	0.1169	0.22121	1.2552	0.32788
836.6137	5.78	lomics Pos	0.7529	0.99987	1.0285	0.040607	0.54855	0.99994	0.9382	-0.09204	0.56858	0.99996	0.94411	-0.08298	0.2951	0.75244	0.88364	-0.17846	0.016163	0.044263	0.72202	-0.46989	0.03062	0.15651	0.79118	-0.33793	0.18412	0.64793	0.84519	-0.24265	0.097799	0.84751	0.8433	-0.24588	0.077847	0.17957	1.3168	0.39708
538.8011	9.92	lomics Pos	0.75318	0.99987	0.96882	-0.0457	0.2042	0.99994	0.86833	-0.20369	0.41836	0.99996	0.92003	-0.12024	0.1645	0.596	0.86608	-0.20744	0.031478	0.073546	0.74478	-0.42511	0.21145	0.50352	0.85568	-0.22486	0.94046	0.98682	0.99148	-0.01235	0.23961	0.94365	0.89341	-0.1626	0.084622	0.186	1.187	0.24735
71.0881	6.38	olomics Po	0.75385	0.99951	1.062	0.086783	0.34422	0.99998	0.88851	-0.17053	0.63769	0.99941	1.0616	0.086249	0.063634	0.33109	0.80038	-0.32125	8.67E-05	0.001446	0.42613	-1.2306	0.006936	0.069358	0.60128	-0.73388	0.011677	0.26088	0.7304	-0.45325	0.008833	0.47452	0.66998	-0.5778	0.8167	0.92407	0.98316	-0.0245
109.1022	6.99	olomics Po	0.75391	0.99951	1.0705	0.098255	0.62059	0.99998	0.93515	-0.09672	0.12508	0.99941	0.7605	-0.39498	0.019009	0.21385	0.61911	-0.69174	0.000328	0.004145	0.32603	-1.6169	0.000265	0.039399	0.42055	-1.2496	0.009539	0.24769	0.55143	-0.85875	0.041941	0.64572	0.62939	-0.66798	0.18181	0.44604	0.80577	-0.31155
498.902	0.5	lomics Pos	0.7544	0.99987	1.0088	0.012601	0.16504	0.99994	0.92895	-0.10633	0.11822	0.99996	0.92042	-0.11964	0.042637	0.36047	0.89047	-0.16737	5.67E-05	0.000707	0.736	-0.44222	0.27896	0.56608	0.88756	-0.17208	0.34579	0.74644	0.94213	-0.086	0.17519	0.91806	0.93632	-0.09493	0.042319	0.1368	1.1674	0.22332
411.2674	2.28	lomics Pos	0.75447	0.99987	1.044	0.062101	0.17192	0.99994	0.88016	-0.18416	0.02443	0.99996	0.84727	-0.2391	0.15305	0.58077	0.78211	-0.35456	3.75E-05	0.00053	0.58156	-0.782	0.056921	0.23746	0.6169	-0.69689	0.033777	0.36346	0.66902	-0.57987	0.001011	0.41831	0.59917	-0.73897	0.29011	0.40022	0.87056	-0.19998
418.8781	10.2	lomics Pos	0.75453	0.99987	0.9775	-0.03283	0.051524	0.99994	0.79702	-0.32731	0.03848	0.99996	0.78361	-0.35179	0.038703	0.34213	0.85583	-0.22461	0.021646	0.055467	0.73645	-0.44134	0.050651	0.22047	0.74905	-0.41687	0.1636	0.63418	0.87368	-0.19483	0.017737	0.52955	0.83576	-0.25884	0.57638	0.67066	1.0323	0.045833
405.791	5.42	lomics Pos	0.75474	0.99987	1.0719	0.10015	0.21586	0.99994	0.88794	-0.17147	0.094812	0.99996	0.87945	-0.18533	0.032833	0.31629	0.75321	-0.40888	0.000584	0.003704	0.65268	-0.61555	0.11374	0.36322	0.8454	-0.2423	0.59304	0.86374	1.1348	0.18242	0.99006	0.99712	1.0079	0.011357	0.32559	0.4359	1.2428	0.3136
924.5976	5.51	lomics Neg	0.75483	0.99977	1.1692	0.22557	0.98296	0.99904	1.0717	0.099909	0.46209	0.99994	0.91563	-0.12717	0.13751	0.58182	1.3494	0.43236	0.98826	0.99989	1.2108	0.27593	0.88508	0.99653	1.1937	0.2554	0.094114	0.41579	0.73788	-0.43854	0.04383	0.46971	0.57416	-0.80048	0.99914	0.99914	1.0305	0.043335
410.1669	4.11	olomics Po	0.75515	0.99951	0.97689	-0.03373	0.92473	0.99998	1.0101	0.014508	0.46752	0.99941	0.93105	-0.10306	0.6765	0.99905	0.96285	-0.05461	0.98825	0.9989	1.0065	0.009353	0.022508	0.11078	0.8405	-0.25069	0.89273	0.9951	0.98857	-0.01658	0.81391	0.99956	0.98106	-0.02758	0.26728	0.53867	0.90047	-0.15124
857.5202	5.6	lomics Pos	0.7553	0.99987	0.94252	-0.08541	0.80936	0.99994	0.93332	-0.09955	0.5006	0.99996	0.83965	-0.25213	0.66864	0.95717	0.91318	-0.13102	0.76582	0.79914	0.9297	-0.10517	0.22263	0.51214	1.2796	0.35566	0.077162	0.48955	1.5534	0.63543	0.045684	0.65733	1.4999	0.58487	0.007654	0.091944	2.0507	1.0361
1064.81	10.02	lomics Neg	0.75578	0.99977	0.96544	-0.05075	0.38901	0.99904	0.91638	-0.12598	0.16554	0.99994	0.86323	-0.21218	0.59508	0.6924	1.0475	0.066971	0.74058	0.99989	1.0239	0.034092	0.71374	0.99653	0.95941	-0.05978	0.039415	0.37635	0.76473	-0.38697	0.1326	0.56884	0.8326	-0.26431	0.1409	0.4061	0.835	-0.26016
396.2325	2.85	lomics Pos	0.75599	0.99987	0.95554	-0.06562	0.23533	0.99994	0.82362	-0.27995	0.027468	0.99996	0.69156	-0.53208	0.016224	0.24	0.51475	-0.95807	0.000609	0.003827	0.40557	-1.302	0.008881	0.08583	0.36508	-1.4537	0.015667	0.33809	0.4205	-1.2498	0.016644	0.52955	0.44536	-1.1669	0.051597	0.14839	0.57721	-0.79283
867.6486	5.15	lomics Pos	0.75677	0.99987	1.0539	0.075693	0.42362	0.99994	0.8832	-0.1792	0.44618	0.99996	0.89413	-0.16144	0.18933	0.63211	0.8152	-0.29477	0.022142	0.056436	0.66718	-0.58385	0.28907	0.57347	0.83837	-0.25434	0.44386	0.80121	1.1335	0.1808	0.8913	0.98023	1.0068	0.009833	0.48946	0.59173	1.1276	0.17326
1029.788	10.02	lomics Pos	0.75703	0.99987	1.0483	0.067986	0.62298	0.99994	0.95476	-0.0668	0.27084	0.99996	0.91609	-0.12643	0.10394	0.52894	0.87245	-0.19686	0.00635	0.021774	0.76067	-0.39465	0.56561	0.76369	0.95388	-0.06812	0.58562	0.86163	1.0225	0.032134	0.49051	0.96776	0.95473	-0.06683	0.072198	0.17377	1.2422	0.31293
840.7022	6.09	lomics Pos	0.75748	0.99987	0.97047	-0.04324	0.43145	0.99994	0.89085	-0.16675	0.33199	0.99996	0.89741	-0.15617	0.24318	0.70354	0.83415	-0.26162	0.048264	0.10156	0.72313	-0.46767	0.054603	0.231	0.71607	-0.48183	0.3881	0.7665	0.89671	-0.15729	0.13011	0.88537	0.75553	-0.40443	0.11428	0.21876	0.8031	-0.31635
914.5801	6.34	lomics Pos	0.75776	0.99987	1.051	0.071826	0.43758	0.99994	0.91219	-0.1326	0.34826	0.99996	0.89025	-0.16772	0.36063	0.8151	0.87812	-0.18751	0.01426	0.040218	0.70151	-0.51146	0.048874	0.21519	0.70819	-0.49779	0.05721	0.42773	0.73043	-0.45318	0.04068	0.64308	0.67392	-0.56936	0.81792	0.86632	1.0175	0.024963
490.8708	10.05	lomics Pos	0.75875	0.99987	1.0161	0.023059	0.75731	0.99994	0.9814	-0.02709	0.16332	0.99996	0.92793	-0.10792	0.41269	0.85521	0.93677	-0.09423	0.000434	0.00294	0.77238	-0.37263	0.21238	0.50393	0.88193	-0.18126	0.34882	0.7477	0.95229	-0.07053	0.66181	0.97988	1.0252	0.035933	0.062427	0.16008	1.1568	0.21019
464.3103	6.09	olomics Ne	0.76	0.99918	0.91908	-0.12174	0.73701	0.95512	0.88123	-0.1824	0.32464	0.99995	0.74516	-0.42437	0.76901	0.99985	0.89379	-0.162	0.021422	0.91567	0.38885	-1.3627	0.75257	0.94707	0.89181	-0.16518	0.20712	0.58992	1.2824	0.35887	0.58432	0.99512	1.0828	0.11477	0.48387	0.99941	1.207	0.27143
597.5017	2.72	lomics Pos	0.76003	0.99987	0.94984	-0.07425	0.21058	0.99994	0.70836	-0.49745	0.042236	0.99996	0.64061	-0.64249	0.002114	0.17581	0.39089	-1.3552	0.000997	0.00552	0.28146	-1.829	0.005901	0.076797	0.23228	-2.1061	0.022058	0.33809	0.33156	-1.5927	0.010517	0.52955	0.29914	-1.7411	0.043266	0.13852	0.41938	-1.2537
319.195	2.28	lomics Pos	0.76009	0.99987	0.97145	-0.04178	0.38481	0.99994	0.873	-0.19595	0.040825	0.99996	0.72093	-0.47208	0.024288	0.27645	0.54386	-0.8787	0.000416	0.00284	0.44763	-1.1596	0.01688	0.11021	0.41472	-1.2698	0.03406	0.36356	0.49484	-1.015	0.020015	0.52955	0.43939	-1.1864	0.048472	0.14485	0.54518	-0.87519
961.5026	8.24	olomics Ne	0.76011	0.99918	1.0497	0.07	0.8239	0.98182	0.97152	-0.04168	0.37405	0.99995	1.0831	0.11513	0.73879	0.99985	0.95992	-0.05901	0.96798	0.99927	1.013	0.018638	0.042312	0.48405	1.2372	0.30709	0.18488	0.56334	1.1523	0.20457	0.81279	0.99512	1.0212	0.030222	0.86296	0.99941	0.97688	-0.03375
935.5577	5.93	lomics Pos	0.76044	0.99987	1.1145	0.15643	0.035286	0.99994	1.2766	0.35235	0.081371	0.99996	1.2307	0.29951	0.29113	0.75244	1.0989	0.13604	0.29798	0.39246	0.80168	-0.3189	0.22772	0.51532	0.85041	-0.23377	0.27657	0.70707	1.1163	0.15877	0.82092	0.97988	1.0329	0.046733	0.015726	0.099085	1.4209	0.50685
606.2915	1.04	lomics Pos	0.76065	0.99987	1.0611	0.085599	0.066638	0.99994	1.2037	0.26754	0.38866	0.99996	1.1789	0.23739	0.70747	0.96308	1.0278	0.039541	0.30507	0.39877	0.90197	-0.14885	0.005147	0.074042	1.6627	0.73351	0.000319	0.15473	2.1688	1.1169	0.35517	0.96776	1.1533	0.2058	0.001554	0.07528	1.8264	0.86897
313.2742	1.32	lomics Pos	0.76069	0.99987	1.073	0.10171	0.94757	0.99994	0.96724	-0.04805	0.90077	0.99996	0.96997	-0.04399	0.87041	0.9874	0.98616	-0.02011	0.65935	0.7108	0.79916	-0.32344	0.12061	0.37754	1.6088	0.68595	0.023102	0.33809	2.2228	1.1524	0.12421	0.88537	1.5737	0.65417	0.046186	0.14289	1.9984	0.99883
138.0527	0.33	olomics Po	0.76084	0.99951	0.9259	-0.11107	0.65288	0.99998	1.1101	0.15063	0.44549	0.99941	1.1987	0.26148	0.087643	0.39517	1.5855	0.66493	0.039015	0.13934	1.5947	0.67326	0.016197	0.094307	1.9255	0.94527	0.016736	0.29901	2.1262	1.0883	0.080909	0.78717	1.5757	0.65598	0.093493	0.3477	1.3219	0.40261
789.6101	5.4	lomics Pos	0.76091	0.99987	1.0165	0.023653	0.45697	0.99994	0.95476	-0.0668	0.39757	0.99996	0.95067	-0.07298	0.40898	0.85122	0.928	-0.10781	0.005704	0.020214	0.77444	-0.36877	0.60353	0.78528	0.96787	-0.04711	0.39957	0.77259	1.0952	0.13115	0.23637	0.94365	1.0846	0.1172	0.012193	0.098791	1.2537	0.32623
340.1907	6.96	olomics Po	0.76164	0.99951	0.95876	-0.06076	0.32293	0.99998	0.84623	-0.24088	0.067384	0.99941	0.73927	-0.43583	0.05824	0.3163	0.6267	-0.67416	0.000935	0.008899	0.50268	-0.99229	0.006406	0.066819	0.55039	-0.86149	0.007585	0.23195	0.5324	-0.90942	0.032084	0.58535	0.52685	-0.92454	0.058362	0.30336	0.56162	-0.83234
837.6711	6.05	lomics Pos	0.76191	0.99987	0.97349	-0.03877	0.18936	0.99994	0.82922	-0.27017	0.3006	0.99996	0.88289	-0.17969	0.11342	0.53238	0.82171	-0.2833	0.012335	0.03596	0.72874	-0.45653	0.20352	0.49272	0.79615	-0.32889	0.21057	0.65208	0.8932	-0.16295	0.17118	0.91806	0.8443	-0.24416	0.42491	0.53174	0.91714	-0.12479
127.8702	0.56	olomics Ne	0.76221	0.99918	0.98589	-0.02051	0.008044	0.34605	0.89221	-0.16454	0.96035	0.99995	0.99911	-0.00129	0.64646	0.99985	0.98524	-0.02145	0.79161	0.99927	0.99175	-0.01195	0.047867	0.48405	1.0807	0.11201	0.82494	0.95121	0.99111	-0.01288	0.78678	0.99512	1.0184	0.026328	0.33589	0.99941	0.93306	-0.09996
659.6092	6.65	lomics Pos	0.76243	0.99987	1.0273	0.038867	0.89964	0.99994	1.0011	0.001551	0.64382	0.99996	1.0968	0.13324	0.24736	0.70693	0.82849	-0.27145	0.23635	0.33091	0.84459	-0.24367	0.17306	0.45921	0.83856	-0.25401	0.83881	0.96659	0.99926	-0.00107	0.014682	0.52955	0.78381	-0.35142	0.68907	0.7684	0.97169	-0.04143
992.7582	0.53	lomics Pos	0.76337	0.99987	1.0786	0.10916	0.29981	0.99994	0.90504	-0.14394	0.23203	0.99996	0.8762	-0.19067	0.18963	0.63211	0.86785	-0.20448	0.099171	0.17322	0.83909	-0.2531	0.016097	0.10794	0.81816	-0.28955	0.772	0.94358	0.97816	-0.03186	0.54278	0.96776	0.94822	-0.07671	0.11985	0.22401	1.1752	0.23294
814.6231	5.52	lomics Pos	0.76384	0.99987	1.0381	0.053948	0.78345	0.99994	0.96764	-0.04745	0.79929	0.99996	0.97057	-0.04309	0.41573	0.85524	0.90672	-0.14128	0.038425	0.085882	0.73368	-0.44678	0.09557	0.32626	0.80488	-0.31316	0.091091	0.52136	0.83961	-0.25221	0.094244	0.83765	0.80801	-0.30755	0.8105	0.85989	0.97295	-0.03957
632.8046	2.37	olomics Po	0.7642	0.99951	0.94353	-0.08386	0.77224	0.99998	0.93303	-0.10001	0.7802	0.99941	0.94359	-0.08377	0.29919	0.78998	0.76503	-0.38641	0.003941	0.024781	0.31214	-1.6798	0.20303	0.47799	0.63372	-0.65809	0.67816	0.99391	0.90625	-0.14201	0.61166	0.99956	0.87819	-0.1874	0.75454	0.89479	0.91349	-0.13055
612.3253	1.38	lomics Pos	0.76424	0.99987	1.1183	0.16135	0.93963	0.99994	0.99114	-0.01284	0.54875	0.99996	0.9001	-0.15184	0.78713	0.9754	0.94846	-0.07634	0.40969	0.49861	0.85235	-0.23049	0.64663	0.81508	1.0851	0.11784	0.037875	0.37121	1.4435	0.52955	0.52783	0.96776	1.1127	0.15412	0.028368	0.11732	1.8026	0.85007
376.9841	4.08	olomics Ne	0.7648	0.99918	1.0096	0.013834	0.015414	0.37315	0.87731	-0.18884	0.67975	0.99995	0.98011	-0.02899	0.47212	0.99985	1.0436	0.061559	0.49285	0.99927	0.96713	-0.04822	0.5032	0.85559	1.0298	0.042358	0.30025	0.67818	1.0433	0.061142	0.97315	0.99512	1.0023	0.003379	0.42046	0.99941	1.0515	0.072454
1084.772	0.5	lomics Pos	0.76508	0.99987	1.0249	0.035516	0.1598	0.99994	0.8816	-0.1818	0.27175	0.99996	0.91999	-0.12032	0.40295	0.84551	0.93646	-0.09472	0.001586	0.007631	0.76585	-0.38486	0.80992	0.90326	0.98594	-0.02043	0.61087	0.87064	0.95373	-0.06834	0.57929	0.9703	0.96874	-0.04582	0.084702	0.186	1.153	0.20544
270.2799	6.78	olomics Po	0.76516	0.99951	0.98038	-0.02859	0.058605	0.99998	0.87237	-0.19698	0.76727	0.99941	0.97876	-0.03097	0.90658	0.99905	1.0156	0.022309	0.61459	0.97478	0.96444	-0.05224	0.05144	0.19142	0.77568	-0.36646	0.030677	0.35049	0.821	-0.28454	0.078632	0.7789	0.85734	-0.22206	0.021036	0.2688	0.86623	-0.20717
855.5082	1.83	lomics Pos	0.76575	0.99987	1.1455	0.19597	0.59093	0.99994	0.88665	-0.17357	0.25198	0.99996	0.70926	-0.49562	0.064179	0.43456	0.45418	-1.1387	0.0051	0.018467	0.35105	-1.5103	0.011793	0.092554	0.43202	-1.2108	0.016873	0.33809	0.45069	-1.1498	0.03197	0.62711	0.41463	-1.2701	0.084258	0.186	0.55364	-0.85299
693.9786	12.03	olomics Ne	0.7662	0.99918	1.0902	0.12457	0.062636	0.50118	0.77031	-0.37649	0.59267	0.99995	1.1346	0.18215	0.36913	0.99985	0.91083	-0.13474	0.047738	0.99927	0.73372	-0.4467	0.54171	0.87267	1.1411	0.19045	0.8026	0.95074	1.1211	0.16486	0.7951	0.99512	1.019	0.027085	0.10512	0.99941	0.77041	-0.37629
809.4923	6.49	olomics Po	0.76677	0.99951	1.1244	0.16913	0.34665	0.99998	0.84545	-0.24221	0.88874	0.99941	0.97754	-0.03278	0.029531	0.24447	0.689	-0.53742	0.52853	0.92315	0.8991	-0.15344	0.07191	0.23909	0.7661	-0.3844	0.95713	0.9974	1.0179	0.025562	0.8919	0.99956	1.0377	0.053387	0.67406	0.8517	1.1468	0.19756
1011.78	10.06	lomics Pos	0.76699	0.99987	1.0262	0.037366	0.97443	0.99994	1.0136	0.01951	0.37746	0.99996	0.89723	-0.15645	0.52584	0.89938	0.93537	-0.09639	0.023304	0.05846	0.81039	-0.30331	0.11328	0.36206	0.83821	-0.25462	0.3053	0.7283	1.1747	0.23234	0.21119	0.92881	0.8741	-0.19414	0.084493	0.186	1.2118	0.27714
1116.739	10.07	lomics Pos	0.76727	0.99987	1.0353	0.050005	0.86747	0.99994	0.98832	-0.01695	0.47545	0.99996	1.0418	0.059061	0.43881	0.86677	0.94258	-0.08531	0.029575	0.070131	0.86276	-0.21297	0.62972	0.80359	1.0489	0.068903	0.17481	0.64606	1.0942	0.12989	0.34343	0.96524	1.0603	0.08453	0.026837	0.11548	1.2838	0.36047
246.2308	6.48	olomics Po	0.7673	0.99951	0.9701	-0.04379	0.53191	0.99998	0.91799	-0.12344	0.099805	0.99941	0.6948	-0.52532	0.040593	0.27667	0.56811	-0.81577	0.003135	0.021166	0.46651	-1.1	0.011363	0.083709	0.43441	-1.2029	0.013805	0.28064	0.38706	-1.3694	0.009445	0.47452	0.38389	-1.3812	0.035058	0.272	0.40463	-1.3053
460.2802	1.54	lomics Pos	0.76748	0.99987	0.92854	-0.10696	0.54049	0.99994	0.86118	-0.21561	0.36551	0.99996	0.80564	-0.3118	0.49342	0.88874	0.84267	-0.24696	0.000764	0.004537	0.25622	-1.9646	0.070515	0.27187	0.6223	-0.68432	0.85495	0.97109	1.0585	0.082013	0.78034	0.97988	1.0504	0.070945	0.14336	0.24801	1.4363	0.52235
362.2463	4.74	olomics Po	0.76761	0.99951	1.0322	0.04569	0.69643	0.99998	1.0483	0.06806	0.55313	0.99941	1.0834	0.11562	0.84724	0.99905	1.0492	0.069262	0.001529	0.012837	0.31535	-1.665	0.77066	0.93356	0.95475	-0.0668	0.78616	0.99391	1.0227	0.03243	0.2733	0.99956	1.1301	0.17641	0.24159	0.50938	1.1745	0.23201
548.8303	0.55	lomics Pos	0.76788	0.99987	1.0172	0.024594	0.83013	0.99994	0.97904	-0.03055	0.82632	0.99996	0.97876	-0.03097	0.093811	0.51027	0.86938	-0.20194	0.008139	0.026301	0.73019	-0.45366	0.19645	0.48421	0.8584	-0.22028	0.31889	0.72948	0.90616	-0.14216	0.73229	0.97988	0.96693	-0.04852	0.077229	0.17836	1.2537	0.32621
644.2626	5.46	olomics Po	0.76811	0.99951	1.1613	0.21568	0.68367	0.99998	1.0995	0.13691	0.70504	0.99941	1.1468	0.19766	0.56163	0.99905	1.1427	0.19246	0.28803	0.65146	1.3596	0.44316	0.041121	0.16334	1.7082	0.77244	0.033759	0.36264	1.7872	0.83769	0.3259	0.99956	1.4131	0.49889	0.086502	0.33891	1.7525	0.80945
309.9189	12.14	olomics Po	0.76818	0.99951	0.97812	-0.03191	0.64441	0.99998	1.0472	0.066493	0.64521	0.99941	1.036	0.051045	0.80393	0.99905	1.0281	0.040025	0.5618	0.94362	0.94844	-0.07637	0.027988	0.12667	1.1632	0.21813	0.47507	0.99391	0.92663	-0.10994	0.67533	0.99956	0.97031	-0.04349	0.26082	0.53177	1.0647	0.090478
873.5436	4.85	lomics Pos	0.76825	0.99987	0.98038	-0.02858	0.050939	0.99994	0.83195	-0.26544	0.50679	0.99996	0.93831	-0.09186	0.77127	0.97526	1.0424	0.05984	0.034498	0.078909	0.75659	-0.40242	0.87591	0.93567	1.0314	0.044578	0.98139	0.99431	1.0318	0.045094	0.82038	0.97988	0.98826	-0.01703	0.063579	0.16173	1.2538	0.32633
333.1503	1.83	lomics Pos	0.76877	0.99987	0.9566	-0.06401	0.34468	0.99994	0.78763	-0.34442	0.040992	0.99996	0.52749	-0.92278	0.005276	0.17581	0.32499	-1.6215	0.008008	0.025965	0.35092	-1.5108	0.028448	0.14839	0.3676	-1.4438	0.014765	0.33809	0.3402	-1.5555	0.036359	0.64014	0.37091	-1.4309	0.021231	0.10765	0.3568	-1.4868
308.892	10.15	lomics Pos	0.7688	0.99987	1.008	0.011479	0.75326	0.99994	1.0227	0.032448	0.54862	0.99996	0.97396	-0.03807	0.45526	0.87441	0.94806	-0.07695	0.003571	0.014007	0.81309	-0.29851	0.4755	0.69815	0.94519	-0.08133	0.91458	0.98003	1.0158	0.022645	0.71302	0.97988	0.97441	-0.0374	0.007298	0.091944	1.2166	0.28281
288.9228	0.28	olomics Po	0.76902	0.99951	1.0599	0.083883	0.99578	0.99998	1.0075	0.010821	0.61602	0.99941	1.2813	0.35765	0.32937	0.82464	1.2027	0.26633	0.39492	0.79545	1.202	0.26549	0.085028	0.26738	1.4485	0.53459	0.047871	0.44092	1.3034	0.38226	0.048746	0.67731	1.3421	0.42447	0.28269	0.55724	1.1927	0.25426
182.0807	0.36	olomics Po	0.76902	0.99951	0.90814	-0.13901	0.90831	0.99998	0.98914	-0.01575	0.42999	0.99941	1.2326	0.30165	0.067727	0.34244	1.877	0.90845	0.15277	0.40542	1.4926	0.57779	0.24662	0.53724	1.3081	0.38742	0.028638	0.3417	1.6785	0.74721	0.34231	0.99956	1.3425	0.42488	0.53456	0.77436	1.1784	0.23678
362.2381	2.84	lomics Pos	0.76919	0.99987	1.0977	0.13447	0.85143	0.99994	0.97228	-0.04055	0.082151	0.99996	0.7003	-0.51395	0.01702	0.24438	0.56731	-0.81778	0.001661	0.00786	0.43914	-1.1872	0.004935	0.073217	0.3756	-1.4127	0.051999	0.41166	0.49554	-1.0129	0.067514	0.76335	0.56677	-0.81917	0.063308	0.16148	0.60489	-0.72526
1103.825	4.66	lomics Pos	0.76944	0.99987	1.0777	0.10794	0.30896	0.99994	0.81982	-0.28662	0.46949	0.99996	0.86453	-0.21002	0.2972	0.75547	0.78516	-0.34894	0.043859	0.094588	0.66707	-0.58408	0.039527	0.18336	0.59028	-0.76052	0.20117	0.64891	0.73958	-0.43521	0.53574	0.96776	0.89548	-0.15927	0.35366	0.46276	1.1936	0.25538
969.7131	7.45	lomics Pos	0.77004	0.99987	0.93496	-0.09702	0.73393	0.99994	0.93094	-0.10323	0.15953	0.99996	0.72799	-0.45801	0.2072	0.65238	0.76123	-0.39359	0.31199	0.40545	0.78789	-0.34393	0.65428	0.81917	0.91916	-0.12161	0.98043	0.99431	1.0821	0.11389	0.019643	0.52955	0.5746	-0.79937	0.16073	0.26805	0.72038	-0.47316
587.5488	5.94	lomics Pos	0.77023	0.99987	0.9801	-0.02899	0.35338	0.99994	0.91916	-0.12161	0.22117	0.99996	0.82543	-0.27678	0.46102	0.87938	0.92595	-0.111	0.007038	0.023459	0.7081	-0.49798	0.17659	0.46349	0.78542	-0.34846	0.013283	0.33809	0.74368	-0.42725	0.031886	0.62711	0.84658	-0.24028	0.24319	0.35164	0.93057	-0.10382
579.282	1.28	lomics Pos	0.7708	0.99987	1.0215	0.030734	0.59122	0.99994	0.93506	-0.09688	0.94191	0.99996	0.98838	-0.01687	0.16264	0.5931	0.85488	-0.2262	0.039908	0.08806	0.72459	-0.46476	0.36356	0.62554	0.87416	-0.19403	0.61072	0.87064	1.0608	0.085086	0.99619	0.99881	0.99324	-0.00978	0.039012	0.13241	1.3163	0.39644
343.2247	1.1	lomics Pos	0.77099	0.99987	1.1188	0.1619	0.79265	0.99994	0.8328	-0.26396	0.52616	0.99996	0.65829	-0.6032	0.7971	0.9771	0.87928	-0.18561	0.00128	0.006595	0.033747	-4.8891	0.14402	0.41633	0.35123	-1.5095	0.2334	0.6761	0.41777	-1.2592	0.49276	0.96776	0.56453	-0.82489	0.86513	0.90148	1.0169	0.024244
364.2732	2.28	lomics Pos	0.77117	0.99987	0.98213	-0.02601	0.40226	0.99994	0.84933	-0.2356	0.13152	0.99996	0.81787	-0.29005	0.007947	0.19917	0.54944	-0.86398	9.52E-05	0.000988	0.42319	-1.2406	0.006827	0.080671	0.46249	-1.1125	0.080518	0.4991	0.58328	-0.77775	0.01347	0.52955	0.4541	-1.1389	0.015648	0.099085	0.51117	-0.96812
179.107	0.78	lomics Pos	0.77219	0.99987	0.96515	-0.05117	0.16632	0.99994	0.82576	-0.2762	0.44969	0.99996	0.91229	-0.13244	0.23641	0.69488	0.88643	-0.17392	0.007212	0.023892	0.69843	-0.51782	0.066606	0.26182	0.73229	-0.44951	0.18352	0.64793	0.81752	-0.29068	0.23683	0.94365	0.8834	-0.17885	0.74246	0.8082	0.96144	-0.05673
560.3103	5.45	olomics Po	0.77223	0.99951	1.0764	0.10627	0.84644	0.99998	1.1391	0.1879	0.86495	0.99941	1.0277	0.039468	0.9107	0.99905	0.94805	-0.07697	0.57002	0.94963	1.2867	0.36367	0.075348	0.24549	1.9346	0.95207	0.027803	0.33945	2.2187	1.1497	0.32804	0.99956	1.3913	0.47643	0.084947	0.33532	2.2346	1.16
522.3463	5.1	olomics Po	0.77299	0.99951	0.91807	-0.12333	0.97821	0.99998	0.96794	-0.04702	0.92505	0.99941	0.9709	-0.04261	0.54084	0.99905	1.2416	0.31222	0.19876	0.49338	1.5259	0.60967	0.019593	0.10338	2.2205	1.1509	0.01482	0.28681	2.4369	1.2851	0.26438	0.99956	1.4513	0.53737	0.1825	0.44634	1.454	0.54002
508.2863	5.55	lomics Pos	0.77343	0.99987	1.1022	0.14037	0.46596	0.99994	0.9116	-0.13352	0.96156	0.99996	1.0063	0.009115	0.95917	0.9937	1.0411	0.058073	0.043303	0.093578	0.71684	-0.48028	0.83305	0.91724	1.0167	0.023949	0.77136	0.94358	1.0296	0.042041	0.98099	0.99455	1.0141	0.020237	0.26522	0.37538	1.1576	0.21114
567.3249	5.1	olomics Po	0.77358	0.99951	0.99999	-7.7E-06	0.98316	0.99998	1.0171	0.024497	0.90205	0.99941	1.0533	0.074905	0.56569	0.99905	1.1067	0.14623	0.2947	0.66007	1.1341	0.18151	0.076274	0.24744	1.2016	0.26492	0.03177	0.35567	1.3833	0.46808	0.3273	0.99956	1.0922	0.12726	0.3544	0.63213	1.0984	0.13537
919.5548	6.47	olomics Po	0.77361	0.99951	1.1119	0.15303	0.48155	0.99998	0.85138	-0.23213	0.84959	0.99941	1.0213	0.030361	0.044722	0.28517	0.64552	-0.63147	0.17638	0.45504	0.70874	-0.49666	0.10757	0.31639	0.68949	-0.5364	0.56911	0.99391	0.88478	-0.17662	0.68107	0.99956	0.95645	-0.06424	0.76572	0.90005	0.941	-0.08774
905.6033	5.78	lomics Pos	0.7737	0.99987	1.0287	0.040888	0.64283	0.99994	0.93909	-0.09066	0.6533	0.99996	0.94016	-0.08902	0.45086	0.87374	0.89484	-0.1603	0.023894	0.059548	0.6794	-0.55768	0.048169	0.21304	0.76397	-0.38841	0.11128	0.56029	0.79891	-0.32389	0.099185	0.84987	0.80593	-0.31127	0.16738	0.2745	1.2215	0.28863
856.6074	5.49	lomics Neg	0.77405	0.99977	1.2084	0.27312	0.96963	0.99904	1.1259	0.17112	0.30852	0.99994	0.8396	-0.25223	0.17397	0.58182	1.4777	0.56338	0.90771	0.99989	1.4228	0.50871	0.93921	0.99653	1.3638	0.4476	0.14937	0.45153	0.70723	-0.49975	0.033196	0.46971	0.49702	-1.0086	0.77298	0.85694	1.0942	0.12994
444.8686	10.15	lomics Pos	0.77433	0.99987	0.96947	-0.04473	0.25439	0.99994	0.87878	-0.18643	0.21776	0.99996	0.85144	-0.23202	0.037998	0.34165	0.80682	-0.30969	0.028384	0.06807	0.7366	-0.44104	0.166	0.45116	0.79722	-0.32695	0.18092	0.64793	0.87219	-0.19728	0.44815	0.96776	0.92508	-0.11235	0.14757	0.25278	1.1375	0.18592
913.5671	6.1	lomics Pos	0.77472	0.99987	1.0869	0.12022	0.69608	0.99994	1.0902	0.12458	0.73777	0.99996	1.074	0.10294	0.32208	0.7853	0.89174	-0.1653	0.13472	0.21776	0.77498	-0.36778	0.001906	0.057238	0.69992	-0.51474	0.052394	0.41166	0.7968	-0.32771	0.16108	0.91806	0.82977	-0.26922	0.78343	0.83916	1.0749	0.10423
207.171	6.47	olomics Po	0.77495	0.99951	0.96796	-0.04698	0.66878	0.99998	0.94823	-0.07669	0.077437	0.99941	0.69214	-0.53086	0.065092	0.33343	0.64264	-0.63792	0.008577	0.042817	0.45346	-1.141	0.008039	0.074445	0.46688	-1.0989	0.005478	0.2079	0.44185	-1.1784	0.007357	0.47452	0.41896	-1.2551	0.015767	0.2688	0.4708	-1.0868
642.2563	1.06	lomics Pos	0.77577	0.99987	1.0767	0.10668	0.50041	0.99994	0.86285	-0.21281	0.49868	0.99996	1.1196	0.16295	0.34873	0.80248	1.1685	0.22464	0.44946	0.53257	0.84934	-0.23559	0.089268	0.31581	1.4385	0.52455	0.021074	0.33809	1.7521	0.80907	0.17385	0.91806	1.2926	0.37033	0.093995	0.19482	1.4848	0.57026
768.5528	4.62	lomics Pos	0.77593	0.99987	1.0553	0.077719	0.966	0.99994	1.0099	0.014181	0.99266	0.99996	1.0101	0.014549	0.32864	0.78974	0.86749	-0.20508	0.034611	0.07912	0.69346	-0.52811	0.027641	0.1469	0.75275	-0.40975	0.24084	0.67954	0.8655	-0.20839	0.38941	0.96776	0.90327	-0.14677	0.76018	0.82043	0.9661	-0.04976
1115.825	8.39	olomics Po	0.77625	0.99951	1.0291	0.041448	0.41651	0.99998	1.1456	0.19609	0.32563	0.99941	1.1632	0.21805	0.002621	0.15874	1.3635	0.44735	0.010125	0.048766	1.3023	0.38109	0.25977	0.54993	1.2241	0.2917	0.28811	0.90579	1.0889	0.12284	0.78929	0.99956	0.97933	-0.03013	0.65377	0.8417	0.95449	-0.0672

812.6697	5.71	domics Pos	0.77645	0.99987	1.0418	0.059129	0.27502	0.99994	0.88941	-0.16908	0.28412	0.99996	0.90265	-0.14777	0.32811	0.78974	0.89507	-0.15993	0.013539	0.038669	0.7519	-0.41139	0.22535	0.51305	0.85612	-0.22411	0.76065	0.94108	0.97048	-0.04323	0.21012	0.92669	0.85207	-0.23095	0.39063	0.49949	0.91333	-0.1308
794.424	0.57	lomics Neg	0.77647	0.99977	0.96629	-0.04948	0.62876	0.99904	0.93751	-0.09309	0.18029	0.99994	0.85381	-0.22801	0.40395	0.59487	1.0954	0.13144	0.85214	0.99989	0.97663	-0.03412	0.40256	0.99653	0.90207	-0.14869	0.046154	0.38213	0.72788	-0.45823	0.16293	0.59205	0.79681	-0.32769	0.057083	0.405	0.77613	-0.36564
961.8038	10.02	domics Pos	0.7767	0.99987	1.0171	0.02442	0.7676	0.99994	1.0192	0.027435	0.89888	0.99996	1.0227	0.03236	0.37815	0.82724	0.9056	-0.14305	0.042722	0.092608	0.80662	-0.31003	0.31921	0.5964	0.8751	-0.19248	0.46937	0.80647	1.0664	0.092699	0.95668	0.98838	0.99542	-0.00663	0.052691	0.14926	1.2717	0.34679
1003.792	10.02	domics Pos	0.77677	0.99987	1.0252	0.035973	0.095986	0.99994	0.88279	-0.17986	0.16942	0.99996	0.91781	-0.12374	0.50553	0.89133	0.94718	-0.07829	0.001117	0.005959	0.73187	-0.45035	0.13073	0.3953	0.83391	-0.26204	0.45007	0.80156	0.93666	-0.09441	0.19424	0.92582	0.91575	-0.12697	0.22565	0.33285	1.1694	0.22582
365.2699	10.03	olomics Pc	0.77712	0.99951	0.93366	-0.09904	0.59813	0.99998	0.89538	-0.15942	0.367	0.99941	0.80785	-0.30785	0.7849	0.99905	0.95048	-0.07327	0.90344	0.99688	0.95865	-0.06093	0.21351	0.49379	1.3052	0.38431	0.037674	0.38531	1.6242	0.69971	0.35424	0.99956	1.1624	0.21707	0.40137	0.67719	1.2094	0.27429
731.3549	6.19	domics Pos	0.77728	0.99987	1.0436	0.061635	0.23721	0.99994	0.91239	-0.13228	0.052084	0.99996	0.81018	-0.30369	0.021875	0.26563	0.80949	-0.30491	0.002405	0.010386	0.66858	-0.58082	0.39833	0.64626	0.87548	-0.19186	0.63492	0.8822	0.95769	-0.06237	0.024985	0.56108	0.74773	-0.41942	0.70096	0.77672	0.96803	-0.04688
354.1815	6.27	olomics Pc	0.77734	0.99951	0.94023	-0.08892	0.62698	0.99998	0.88469	-0.17676	0.12663	0.99941	0.6441	-0.63465	0.045703	0.2866	0.43211	-1.2105	0.011449	0.05415	0.37257	-1.4244	0.011378	0.083709	0.27081	-1.8846	0.005997	0.21836	0.27772	-1.8483	0.009319	0.47452	0.28616	-1.8051	0.03949	0.28339	0.41251	-1.2775
709.5783	8.37	olomics Pc	0.77764	0.99951	0.97655	-0.03424	0.16407	0.99998	0.85021	-0.2341	0.14395	0.99941	0.87424	-0.19389	0.93084	0.99905	0.99556	-0.00642	0.63552	0.98315	0.96503	-0.05136	0.82692	0.94811	0.98476	-0.02215	0.46287	0.99391	0.92466	-0.113	0.042638	0.64572	0.81467	-0.2957	0.005787	0.2688	0.74195	-0.43061
390.8709	10.14	domics Pos	0.77786	0.99987	0.97532	-0.03606	0.1618	0.99994	0.85342	-0.22868	0.099341	0.99996	0.79078	-0.33866	0.49938	0.89009	0.94357	-0.08381	0.011959	0.03513	0.71907	-0.47579	0.25604	0.54331	0.87031	-0.2004	0.19003	0.64891	0.83546	-0.25936	0.10781	0.86492	0.8646	-0.2099	0.28795	0.39824	1.1036	0.1422
332.0735	1.32	domics Pos	0.77939	0.99987	1.0408	0.057695	0.41127	0.99994	0.90211	-0.14862	0.30991	0.99996	0.88753	-0.17212	0.041676	0.35471	0.85013	-0.23424	0.18304	0.27489	0.82306	-0.28093	0.25619	0.54331	0.88217	-0.18087	0.82022	0.96018	0.98547	-0.02112	0.19297	0.92582	0.86257	-0.21328	0.17483	0.28235	1.1533	0.20578
824.544	8.39	olomics Ne	0.77973	0.99918	1.0228	0.03248	0.42873	0.80889	0.94832	-0.07655	0.8959	0.99995	1.0101	0.014512	0.6032	0.99985	0.97013	-0.04375	0.23263	0.99927	0.93755	-0.09303	0.36307	0.76927	1.0563	0.079076	0.96936	0.98872	0.99734	-0.00384	0.01492	0.99512	0.86624	-0.20717	0.019969	0.99941	0.80887	-0.30601
152.1459	6.38	olomics Pc	0.7803	0.99951	1.0379	0.05373	0.78519	0.99998	0.97771	-0.03252	0.79499	0.99941	0.97873	-0.03102	0.098838	0.41959	0.79242	-0.33567	9.34E-05	0.001508	0.40074	-1.3193	0.003758	0.052849	0.63465	-0.65598	0.003617	0.20152	0.65984	-0.59981	0.12754	0.90554	0.84422	-0.2443	0.93968	0.97465	1.0105	0.015074
709.252	5.08	olomics Ne	0.78083	0.99918	0.95913	-0.0602	0.811	0.9788	0.97384	-0.03824	0.56988	0.99995	1.0851	0.11781	0.53748	0.99985	1.1642	0.2194	0.41114	0.99927	1.2336	0.30285	0.014052	0.40708	1.6913	0.75813	0.013265	0.2716	1.6774	0.74621	0.20743	0.99512	1.3	0.37848	0.14271	0.99941	1.2528	0.32519
875.5298	6.48	olomics Pc	0.78093	0.99951	1.1019	0.14003	0.5056	0.99998	0.89419	-0.16134	0.99192	0.99941	1.0077	0.011126	0.040836	0.27667	0.68803	-0.53946	0.2249	0.54095	0.80069	-0.32068	0.053946	0.1969	0.73415	-0.44584	0.72159	0.99391	0.951	-0.07249	0.60782	0.99956	0.94138	-0.08716	0.81201	0.92261	1.0581	0.081472
702.8637	0.5	domics Pos	0.78124	0.99987	1.0078	0.011199	0.24802	0.99994	0.94122	-0.0874	0.17232	0.99996	0.93473	-0.09738	0.043661	0.36496	0.89391	-0.1618	5.69E-05	0.000707	0.74801	-0.41887	0.25015	0.53676	0.88545	-0.17551	0.54298	0.84817	0.96319	-0.05411	0.29474	0.9483	0.94851	-0.07627	0.033608	0.12517	1.1909	0.25204
768.551	5.81	domics Pos	0.78149	0.99987	0.90468	-0.14452	0.33385	0.99994	0.74424	-0.42616	0.31194	0.99996	0.72185	-0.47024	0.7279	0.96881	0.9259	-0.11106	0.414	0.50246	1.632	0.70668	0.003026	0.063179	3.2321	1.6925	0.006333	0.3088	3.9438	1.9796	0.054567	0.70055	2.7567	1.463	0.022139	0.10842	2.9142	1.5431
571.4241	10.05	olomics Pc	0.78203	0.99951	0.97292	-0.03961	0.40535	0.99998	0.90646	-0.14169	0.31894	0.99941	0.88384	-0.17815	0.33679	0.82883	0.87376	-0.1947	0.83739	0.99688	0.98019	-0.02887	0.038482	0.15641	0.7683	-0.38025	0.18396	0.76147	0.79081	-0.33859	0.013606	0.47452	0.6666	-0.58511	0.007947	0.2688	0.68257	-0.55094
464.3138	5.59	olomics Pc	0.78222	0.99951	0.94484	-0.08186	0.70104	0.99998	0.90815	-0.139	0.27024	0.99941	0.73194	-0.4502	0.49444	0.9698	0.82536	-0.27691	0.000236	0.003225	0.1532	-2.7065	0.086536	0.26979	0.5876	-0.7671	0.78113	0.99391	1.0398	0.056345	0.93869	0.99956	0.98804	-0.01736	0.51588	0.76223	1.1691	0.22537
105.9553	8.95	olomics Pc	0.78262	0.99951	1.1083	0.14831	0.44221	0.99998	1.1781	0.23652	0.71776	0.99941	0.95633	-0.06443	0.34145	0.83506	1.1832	0.24267	0.54332	0.93217	1.1022	0.1404	0.10846	0.31749	1.1669	0.22262	0.041171	0.40276	1.2651	0.33921	0.36587	0.99956	1.119	0.16219	0.52265	0.76572	1.0594	0.083258
995.782	0.53	domics Pos	0.78286	0.99987	0.9627	-0.05485	0.25243	0.99994	0.86943	-0.20186	0.68519	0.99996	0.9468	-0.07887	0.34616	0.79994	0.89071	-0.16698	0.030659	0.072074	0.73791	-0.43849	0.35956	0.62208	0.87006	-0.20081	0.17149	0.64488	0.80476	-0.31337	0.44147	0.96776	0.90869	-0.13814	0.46984	0.57275	1.1206	0.16422
104.9575	0.31	olomics Ne	0.78354	0.99918	1.0239	0.034013	0.012235	0.37315	0.80518	-0.31262	0.44685	0.99995	0.93034	-0.10416	0.26376	0.99985	1.0697	0.097157	0.21844	0.99927	0.89819	-0.15491	0.68524	0.93502	1.0483	0.068004	0.81674	0.95121	0.98281	-0.02502	0.46422	0.99512	1.0634	0.088711	0.95912	0.99945	1.0063	0.009132
616.8167	10.1	domics Pos	0.78428	0.99987	1.0106	0.015233	0.60153	0.99994	1.0288	0.040964	0.097231	0.99996	0.86709	-0.20575	0.1122	0.53238	0.93084	-0.10339	0.001403	0.007003	0.76561	-0.38533	0.045628	0.20468	0.82132	-0.28399	0.6744	0.89976	0.97399	-0.03802	0.044553	0.65325	0.88987	-0.16833	0.050628	0.14725	1.1483	0.19946
301.2081	2.72	domics Pos	0.78434	0.99987	1.0791	0.10989	0.41553	0.99994	0.8998	-0.15232	0.058955	0.99996	0.79514	-0.33072	0.013644	0.22898	0.71786	-0.47822	0.000981	0.005467	0.55306	-0.85448	0.018458	0.11633	0.49894	-1.0031	0.097363	0.53334	0.68556	-0.54465	0.042556	0.65167	0.56206	-0.8312	0.089455	0.19023	0.70463	-0.50507
488.3118	2.2	domics Pos	0.78474	0.99987	0.92679	-0.10968	0.76491	0.99994	1.006	0.008673	0.38256	0.99996	0.80384	-0.31503	0.52591	0.89938	0.84266	-0.24698	0.009712	0.030156	0.30076	-1.7333	0.59716	0.78166	0.87152	-0.19839	0.62023	0.87361	1.1551	0.20796	0.74827	0.97988	1.0722	0.10051	0.054931	0.15218	1.4953	0.58039
1178.772	0.5	domics Pos	0.78519	0.99987	1.0097	0.013981	0.49187	0.99994	0.97034	-0.04343	0.10623	0.99996	0.93369	-0.09898	0.012771	0.22648	0.87596	-0.19106	0.000153	0.001385	0.73901	-0.43633	0.19807	0.4857	0.86789	-0.20442	0.33951	0.74343	0.93308	-0.09992	0.11796	0.88537	0.93752	-0.09308	0.048408	0.14485	1.1734	0.23074
920.5588	4.96	lomics Neg	0.78577	0.99977	1.0816	0.11317	0.82829	0.99904	0.99981	-0.00027	0.18793	0.99994	0.88174	-0.18158	0.028248	0.58182	1.2964	0.37448	0.84831	0.99989	1.0312	0.044365	0.87568	0.99653	1.0458	0.064665	0.04237	0.38004	0.71732	-0.47932	0.02316	0.46971	0.58645	-0.76992	0.020095	0.39655	0.83349	-0.26277
332.1455	6.06	olomics Pc	0.78578	0.99951	0.99015	-0.01428	0.4927	0.99998	0.9033	-0.14673	0.054954	0.99941	0.67638	-0.5641	0.047242	0.28918	0.48626	-1.0402	0.003867	0.024408	0.44362	-1.1726	0.029473	0.13131	0.45764	-1.1277	0.003333	0.20152	0.4253	-1.2335	0.023347	0.51791	0.40705	-1.2967	0.017738	0.2688	0.39522	-1.3393
814.6863	6.04	domics Pos	0.78634	0.99987	0.98091	-0.0278	0.12801	0.99994	0.82906	-0.27046	0.25771	0.99996	0.88988	-0.16831	0.10939	0.53208	0.839	-0.25325	0.015072	0.041901	0.74892	-0.41711	0.12997	0.39426	0.79729	-0.32683	0.3623	0.75814	0.93014	-0.10448	0.10858	0.86492	0.82801	-0.27228	0.75155	0.81341	0.97548	-0.03581
189.6379	2.07	domics Pos	0.78647	0.99987	1.0478	0.067415	0.39831	0.99994	0.85191	-0.23123	0.74666	0.99996	0.94419	-0.08285	0.22911	0.68626	0.81924	-0.28764	0.000259	0.001998	0.30483	-1.7139	0.019923	0.12102	0.63496	-0.65525	0.52652	0.83957	0.895	-0.16004	0.77556	0.97988	0.95112	-0.0723	0.02864	0.11732	1.433	0.51903
339.2899	1.39	domics Pos	0.7866	0.99987	1.0511	0.071939	0.84229	0.99994	0.95133	-0.07198	0.77717	0.99996	0.9083	-0.13875	0.67289	0.95737	1.1664	0.2221	0.25652	0.35172	1.7907	0.84056	0.00347	0.066156	2.7925	1.4816	0.001776	0.23318	3.3951	1.7634	0.13343	0.88792	1.9264	0.94593	0.031769	0.12174	2.1859	1.1282
851.5623	4.75	lomics Neg	0.78676	0.99977	1.1592	0.21318	0.96166	0.99904	1.053	0.074497	0.49549	0.99994	0.90251	-0.14798	0.26283	0.58182	1.3566	0.43999	0.87658	0.99989	1.138	0.18652	0.96846	0.99653	1.1857	0.24576	0.14479	0.45153	0.69426	-0.52645	0.048559	0.46971	0.53277	-0.90842	0.91219	0.95073	0.97686	-0.03378
668.5311	5.92	domics Pos	0.78685	0.99987	1.026	0.037096	0.9567	0.99994	1.0106	0.015157	0.50665	0.99996	0.92907	-0.10614	0.38979	0.83592	0.91151	-0.13368	0.02154	0.055346	0.78974	-0.34055	0.15288	0.43045	0.75772	-0.40026	0.06894	0.46982	0.79931	-0.32317	0.2053	0.92582	0.77027	-0.37657	0.89148	0.92168	1.0178	0.025446
200.0393	0.3	olomics Pc	0.78699	0.99951	0.96331	-0.05393	0.83501	0.99998	0.97045	-0.04327	0.3871	0.99941	1.1281	0.1739	0.095378	0.41383	1.4197	0.50555	0.019668	0.082605	1.4128	0.4986	0.014149	0.089365	1.4122	0.49796	0.024802	0.32689	1.4944	0.57952	0.12881	0.90554	1.2566	0.32952	0.41573	0.68743	1.0889	0.12284
190.9587	8.95	olomics Pc	0.78711	0.99951	0.96613	-0.04972	0.69533	0.99998	0.9397	-0.08973	0.40849	0.99941	0.88365	-0.17845	0.26445	0.73853	1.1526	0.20487	0.79372	0.99688	1.0406	0.057369	0.03965	0.15926	1.3101	0.38965	0.23949	0.84407	1.1362	0.18425	0.98907	0.99956	0.98458	-0.02242	0.74828	0.89304	1.0208	0.029671
503.2966	10.09	olomics Pc	0.78765	0.99951	1.0156	0.022331	0.94097	0.99998	0.98415	-0.02306	0.11244	0.99941	0.81032	-0.30344	0.97324	0.99905	1.0008	0.001219	0.72388	0.99688	1.0284	0.040393	0.091974	0.2821	1.2024	0.26594	0.037863	0.38531	1.4262	0.51219	0.088484	0.80182	1.2497	0.32153	0.10507	0.35957	1.1979	0.26052
241.2227	9.91	domics Pos	0.78858	0.99987	1.0121	0.017332	0.15936	0.99994	0.88194	-0.18124	0.12696	0.99996	0.87732	-0.18882	0.42661	0.86135	0.96063	-0.05795	0.002234	0.009816	0.7805	-0.35753	0.48746	0.70592	0.93816	-0.09209	0.71652	0.92042	0.97785	-0.03231	0.52651	0.96776	1.0465	0.065552	0.16845	0.27555	1.0987	0.13584
694.1975	1.24	domics Pos	0.7888	0.99987	1.0308	0.043798	0.94468	0.99994	1.0025	0.00358	0.36959	0.99996	0.93823	-0.09199	0.20736	0.65238	0.92513	-0.11227	0.003915	0.01505	0.76541	-0.38569	0.1147	0.36476	0.81429	-0.29638	0.18664	0.64793	0.83227	-0.26488	0.33549	0.96474	0.92169	-0.11764	0.44679	0.55169	1.0438	0.061839
1186.758	0.5	domics Pos	0.78899	0.99987	0.98383	-0.02352	0.22972	0.99994	0.88863	-0.17035	0.21876	0.99996	0.90105	-0.15032	0.055587	0.40604	0.86752	-0.20503	0.000467	0.003121	0.70399	-0.50636	0.34381	0.61218	0.89249	-0.1641	0.71376	0.91852	0.97468	-0.037	0.1595	0.91806	0.92749	-0.1086	0.025692	0.113	1.1014	0.1393
963.619	8.89	olomics Pc	0.78921	0.99951	1.1658	0.22137	0.66695	0.99998	0.96494	-0.05149	0.82427	0.99941	1.0317	0.044975	0.020915	0.22258	0.71853	-0.47689	0.063924	0.20093	0.68577	-0.5442	0.38543	0.69536	0.91855	-0.12257	0.73614	0.99391	1.2075	0.27207	0.94929	0.99956	1.0446	0.062915	0.096235	0.34969	1.9351	0.9524
109.1019	2.76	domics Pos	0.78937	0.99987	1.0378	0.053467	0.58765	0.99994	0.90774	-0.13964	0.43131	0.99996	0.86732	-0.20536	0.073685	0.45909	0.71152	-0.49102	0.000153	0.001388	0.29291	-1.7714	0.005012	0.073217	0.50834	-0.97612	0.02669	0.34512	0.62426	-0.67977	0.049751	0.6775	0.68687	-0.5419	0.51854	0.61826	1.1793	0.23793
337.2748	5	olomics Pc	0.78939	0.99951	0.90909	-0.13751	0.91768	0.99998	0.95173	-0.07137	0.88909	0.99941	0.93432	-0.09801	0.67865	0.99905	1.2574	0.3304	0.12458	0.34393	1.7769	0.82936	0.018663	0.10085	2.5147	1.3304	0.012781	0.27021	2.7444	1.4565	0.15093	0.93461	1.5737	0.65412	0.17144	0.43307	1.5563	0.63811
1039.627	6.06	domics Pos	0.78941	0.99987	0.97092	-0.04257	0.15619	0.99994	0.79136	-0.33759	0.19466	0.99996	0.86303	-0.21252	0.25717	0.71521	0.86018	-0.21729	0.029975	0.070815	0.72326	-0.46741	0.33342	0.6073	0.83961	-0.25221	0.793	0.95091	0.97209	-0.04085	0.29563	0.9483	0.85721	-0.22228	0.6519	0.73891	0.95426	-0.06755
1120.613	1.11	domics Pos	0.78978	0.99987	0.80586	-0.3114	0.69981	0.99994	0.95081	-0.07278	0.81896	0.99996	0.68245	-0.5512	0.95682	0.9937	0.89444	-0.16094	0.56573	0.62958	1.4116	0.49737	0.059295	0.24149	2.6138	1.3862	0.020276	0.33809	3.6647	1.8737	0.26886	0.94365	1.792	0.84155	0.11127	0.2149	1.9705	0.97856
175.1439	6.48	olomics Pc	0.79007	0.99951	0.99097	-0.01309	0.61612	0.99998	0.95859	-0.06102	0.096696	0.99941	0.73474	-0.44469	0.053595	0.29934	0.60763	-0.71873	0.005183	0.030068	0.51072	-0.9694	0.014706	0.091119	0.48702	-1.0379	0.010515	0.24769	0.41422	-1.2715	0.005455	0.47452	0.41766	-1.2596	0.023671	0.2688	0.42844	-1.2228
1159.719	10.06	domics Pos	0.79026	0.99987	1.0345	0.048919	0.64654	0.99994	0.95456	-0.06709	0.7589	0.99996	1.0374	0.052928	0.7431	0.97243	0.97155	-0.04164	0.013864	0.039363	0.77198	-0.37337	0.98907	0.99297	1.0086	0.012338	0.45077	0.80167	1.0538	0.075548	0.93536	0.98616	0.99324	-0.00979	0.001436	0.07528	1.3897	0.47477
807.5707	5.08	domics Pos	0.7906	0.99987	1.0261	0.037225	0.61779	0.99994	0.94445	-0.08245	0.60499	0.99996	0.9451	-0.08145	0.34786	0.80096	0.90488	-0.1442	0.009463	0.029511	0.7447	-0.42526	0.13131	0.3961	0.84404	-0.24462	0.4659	0.80647	0.93137	-0.10257	0.31712	0.9524	0.90994	-0.13616	0.0468	0.14344	1.1881	0.24869
352.1798	6.27	olomics Pc	0.79068	0.99951	0.9352	-0.09665	0.51347	0.99998	0.83779	-0.25535	0.12396	0.99941	0.60046	-0.73587	0.041093	0.27718	0.4172	-1.2612	0.008841	0.043926	0.30477	-1.7142	0.003875	0.053776	0.19437	-2.3631	0.007256	0.22462	0.24799	-2.0116	0.024832	0.52244	0.30987	-1.6903	0.045034	0.28476	0.43235	-1.2097
1156.825	4.94	domics Pos	0.79071	0.99987	1.0184	0.026257	0.23921	0.99994	0.83823	-0.25459	0.81662	0.99996	0.97657	-0.0342	0.48046	0.88482	0.91158	-0.13356	0.021599	0.055413	0.71491	-0.48417	0.44669	0.67776	0.89035	-0.16756	0.65416	0.89227	0.94313	-0.08447	0.8231	0.97988	1.0107	0.015304	0.13228	0.23736	1.1867	0.2469
727.1656	1.09	domics Pos	0.79095	0.99987	1.0449	0.063351	0.52331	0.99994	0.90997	-0.13611	0.9961	0.99996	1.008	0.011514	0.83572	0.98621	0.978	-0.0321	0.083083	0.15253	0.79966	-0.32254	0.25078	0.5375	1.1938	0.25556	0.037552	0.37121	1.2286	0.29696	0.43199	0.96776	1.0647	0.090505	0.11006	0.21367	1.3254	0.40644
607.3238	5.72	olomics Pc	0.79121	0.99951	1.0493	0.069496	0.17558	0.99998	0.85192	-0.23122	0.48415	0.99941	1.0864	0.11953	0.031034	0.24688	0.71586	-0.48224	0.052073	0.17394	0.66096	-0.59737	0.011204	0.083534	0.71709	-0.47977	0.66327	0.99391	0.95399	-0.06796	0.1857	0.98353	0.8309	-0.26725	0.42908	0.69887	1.1	0.13754
395.2206	2.29	domics Pos	0.79142	0.99987	0.9679	-0.04706	0.41831	0.99994	0.87523	-0.19226	0.061243	0.99996	0.73345	-0.44722	0.025134	0.27941	0.58104	-0.78329	0.000706	0.004254	0.47494	-1.0742	0.025537	0.14071	0.46212	-1.1137	0.032357	0.36287	0.49883	-1.0034	0.009337	0.52955	0.4385	-1.1894	0.054901	0.15218	0.53607	-0.8995
887.0508	1.43	domics Pos	0.79219	0.99987	1.1492	0.20062	0.15551	0.99994	0.83852	-0.25408	0.92179	0.99996	1.027	0.038503	0.040828	0.34959	0.64883	-0.6241	0.009792	0.030269	0.66095	-0.59738	0.04063	0.18733	0.70575	-0.50277	0.76648	0.94339	1.0289	0.04117	0.53803	0.96776	1.1469	0.19774	0.42741	0.53398	1.365	0.44885
583.2554	6.98	olomics Pc	0.79235	0.99951	1.0069	0.009948	0.8994	0.99998	1.034	0.048198	0.83465	0.99941	0.96206	-0.0558	0.45621	0.93558	0.88241	-0.18048	0.005614	0.032022	0.57287	-0.80371	0.017308	0.097219	0.54509	-0.87544	0.019078	0.30136	0.58451	-0.77469	0.085834	0.79289	0.61141	-0.7098	0.068925	0.3118	0.63898	-0.64615
942.5715	6.47	olomics Pc	0.79239	0.99951	1.1106	0.15137	0.3183	0.99998	0.83499	-0.26017	0.86803	0.99941	1.0295	0.04193	0.031657	0.24911	0.66373	-0.59133	0.091879	0.26799	0.68649	-0.5427	0.036865	0.1516	0.65408	-0.61246	0.36684	0.96767	0.83795	-0.25506	0.95233	0.99956	1.0517	0.072666	0.75736	0.89519	0.96084	-0.05764
799.6668	5.91	domics Pos	0.79244	0.99987	1.0393	0.05557	0.33155	0.99994	0.89747	-0.15606	0.46235	0.99996	0.92876	-0.10662	0.23057	0.68793	0.87152	-0.1984	0.038915	0.086671	0.77829	-0.36162	0.10213	0.34	0.81005	-0.30391	0.53396	0.84191	0.92868	-0.10675	0.22917	0.94286	0.85164	-0.23169	0.77724	0.83452	1.0341	0.048347
294.8118	0.44	olomics Ne	0.793	0.99918	1.0258	0.036761	0.92238	0.9934	1.0513	0.072243	0.27851	0.99995	1.117	0.15967	0.1248	0.99985	1.3034	0.38228	0.046291	0.99927	1.4368	0.52284	0.36304	0.76927	1.3691	0.45325	0.26364	0.64137	1.2895	0.36684	0.1235	0.99512	1.1815	0.24063	0.58809	0.99941	1.1197	0.16312
615.5788	6.3	domics Pos	0.79307	0.99987	0.9812	-0.02738	0.92505	0.99994	0.9925	-0.01086	0.45378	0.99996	1.0634	0.088671	0.74506	0.97302	0.97538	-0.03596	0.034873	0.079385	0.80818	-0.30725	0.5105	0.72221	0.92343	-0.11492	0.60709	0.86762	1.086	0.11903	0.89851	0.98023	0.99314	-0.00993	0.4546	0.55969	1.1762	0.23408
745.5635	9.29	olomics Pc	0.7931	0.99951	1.2002	0.26331	0.80008	0.99998	0.97788	-0.03227	0.064956	0.99941	0.79533	-0.33038	0.9722	0.99905	1.1238	0.16834	0.032396	0.12236	1.712	0.77567	0.043633	0.17012	2.1534	1.1066	0.069478	0.53288	2.1477	1.1028	0.29989	0.99956	1.8527	0.88961	0.009337	0.2688	2.0656	1.0466
1177.348	5.14	domics Pos	0.79333	0.99987	1.0534	0.074986	0.34526	0.99994	0.90074	-0.15081	0.37847	0.99996	0.93024	-0.10432	0.24696	0.70693	0.88068	-0.18331	0.004776	0.017627	0.68137	-0.55349	0.090081	0.31628	0.87515	-0.1924	0.52668	0.83957	1.0339	0.048066	0.40188	0.96776	1.0722	0.10056	0.014554	0.098791	1.1849	0.24472
566.8893	0.5	domics Pos	0.79471	0.99987	1.0067	0.009648	0.20424	0.99994	0.93121	-0.10283	0.14409	0.99996	0.93193	-0.10171	0.030224	0.30328	0.88754	-0.17212	4.15E-05	0.000555	0.74702	-0.42077	0.22101	0.51169	0.87963	-0.18503	0.55798	0.85427	0.9641	-0.05275	0.087451	0.82455	0.92947	-0.10552	0.023841	0.11051	1.1895	0.2504
504.3067	1.92	domics Pos	0.79493	0.99987	1.0616	0.086198	0.99437	0.99994	0.9889	-0.01611	0.73837	0.99996	0.8963	-0.15795	0.95451	0.9937	0.94998	-0.07402	0.18658	0.27834	0.6583	-0.60318	0.17236	0.45896	1.3701	0.4543	0.035812	0.36525	1.8334	0.8745	0.18456	0.92582	1.3254	0.40638	0.027506	0.11627	1.8102	0.85618
361.1697	6.41	olomics Pc	0.79501	0.99951	0.94031	-0.0888	0.75777	0.99998	0.92181	-0.11745	0.39327	0.99941	0.82421	-0.27892	0.43867	0.92082	0.83828	-0.2545	9.03E-05	0.001485	0.16697	-2.5823	0.017619	0.097714	0.54066	-0.88721	0.095293	0.60726	0.67615	-0.56458	0.17903	0.97066	0.70018	-0.5142	0.22539	0.49266	0.72006	-0.4738
336.9026	0.49	domics Pos	0.7951	0.99987	1.036	0.051039	0.93268	0.99994	0.99226	-0.01121	0.75032	0.99996	1.0187	0.026664	0.38941	0.83582	0.92303	-0.11555	0.010001	0.030703	0.79444	-0.33199	0.60788	0.78723	0.94594	-0.08018	0.3444	0.74613	1.0826	0.11447	0.85374	0.97988	0.98406	-0.02318	0.21076	0.31953	1.1986	0.26133
354.3736	4.65	domics Pos	0.79519	0.99987	0.97222	-0.04064	0.057299	0.99994	0.76304	-0.39016	0.21928	0.99996	0.85543	-0.22528	0.076212	0.4637	0.8196	-0.28701	0.012614	0.036574	0.74072	-0.433	0.004133	0.070915	0.60448	-0.72623	0.097776	0.53334	0.72913	-0.45576	0.023119	0.54215	0.7469	-0.42102	0.96604	0.97525	1.0012	0.001791
455.2705	2.85	domics Pos	0.79528	0.99987	1.0255	0.036335	0.11465	0.99994	0.84543	-0.24224	0.041933	0.99996	0.80961	-0.3047	0.006793	0.19397	0.57772	-0.79156	0.000531	0.003454	0.52299	-0.93515	0.026229	0.14302	0.50888	-0.9746	0.008284	0.32129	0.61624	-0.69843	0.06066	0.7228	0.65333	-0.61411	0.15304	0.25867	0.77203	-0.37327
631.4652	2.85	domics Pos	0.79535	0.99987	1.0476	0.067061	0.63202	0.99994	0.9278	-0.10812	0.22204	0.99996	0.78063	-0.35728	0.021409	0.26504	0.54431	-0.8775	0.00664	0.022585	0.42014	-1.2511	0.012078	0.093	0.43647	-1.196	0.034428	0.36356	0.47484	-1.0745	0.12189	0.88537	0.59389	-0.75173	0.12542	0.22986	0.6503	-0.62081
195.1721	6.97	olomics Pc	0.79542	0.99951	1.052	0.073114	0.70366	0.99998	0.95064	-0.07303	0.068461	0.99941	0.72972	-0.45459	0.026022	0.23554	0.51441	-0.959	0.001901	0.015005	0.43468	-1.202	0.002635	0.046623	0.41717	-1.2613	0.00439	0.20152	0.46536	-1.1036	0.01888	0.51556	0.4786	-1.0631	0.037313	0.27985	0.57712	-0.79306
1179.773	0.5	domics Pos	0.79632	0.99987	1.0157	0.022536	0.23893	0.99994	0.92701	-0.10935	0.41865	0.99996	0.95177	-0.07132	0.17277	0.60551	0.89804	-0.15515	0.003434	0.013599	0.72039	-0.47315	0.34605	0.61311	0.89998	-0.15204	0.4857	0.81703	0.93507	-0.09686	0.58803	0.97073	0.96559	-0.05052	0.073279	0.17507	1.1902	0.25126
381.2715	2.84	domics Pos	0.79645	0.99987	1.0325	0.04612	0.60922	0.99994	0.9211	-0.11858	0.19084	0.99996	0.80351	-0.31562	0.013859	0.22898	0.59265	-0.75476	0.010037	0.030741	0.50279	-0.99198	0.006871	0.080856	0.4421	-1.1776	0.036725	0.36759	0.65419	-0.61222	0.042831	0.65325	0.56915	-0.81312	0.072204	0.17377	0.68541	-0.54496
184.0741	5.38	domics Pos	0.79666	0.99987	1.0095	0.013608	0.43669	0.99994	0.94744	-0.07789	0.15871	0.99996	0.89575	-0.15884	0.28805	0.75229	0.89472	-0.16049	0.003014	0.012278	0.7732	-0.37108	0.729	0.85825	0.97246	-0.04029	0.87461	0.97532	0.99261	-0.0107	0.44934	0.96776	1.0643	0.089884	0.004257	0.085451	1.293	0.37077
320.2002	2.28	domics Pos	0.79675	0.99987	0.95352	-0.06867	0.4936	0.99994	0.87436	-0.19371	0.26648	0.99996	0.76833	-0.38021	0.020348	0.25894	0.51933	-0.94527	0.001836	0.008483	0.41175	-1.2802	0.007531	0.082061	0.38663	-1.371	0.018963	0.33809	0.46988	-1.0896	0.020636	0.52955	0.42355	-1.2394	0.022101	0.10842	0.52741	-0.923
1152.756	0.5	domics Pos	0.79678	0.99987	1.0289	0.041135	0.25986	0.99994	0.90142	-0.14973	0.27392	0.99996	0.93339	-0.09946	0.092082	0.50411	0.86294	-0.21267	0.001317	0.006694	0.75954	-0.3968	0.28136	0.56793	0.89337	-0.16267	0.52193	0.83957	0.9476	-0.07766	0.17265	0.91806	0.91303	-0.13127	0.032102	0.12241	1.164	0.21911
943.7043	7.45	domics Pos	0.79694	0.99987	1.0417	0.058933	0.80796	0.99994	0.92885	-0.10648	0.39038	0.99996	0.82031	-0.28575	0.31228	0.77382	0.78027	-0.35795	0.081726	0.15069	0.652	-0.61705	0.07915	0.29123	0.61844	-0.69331	0.20824	0.65208	0.69649	-0.52182	0.033952	0.6327	0.52919	-0.91813	0.51481	0.61473	0.86702	-0.20586
492.3456	2.28	domics Pos	0.79697	0.99987	1.0363	0.051486	0.8223	0.99994	1.008	0.011461	0.89547	0.99996	1.0274	0.039006	0.13175	0.55569	0.72354	-0.46686	0.008129	0.026288	0.42444	-1.2364	0.03407	0.16591	0.64393	-0.63503	0.59653	0.86481	1.0691	0.096464	0.51931	0.96776	0.86889	-0.20276	0.7003	0.77623	1.0435	0.061378
559.4238	8.37	olomics Pc	0.79724	0.99951	1.0194	0.027694	0.6312	0.99998	1.0462	0.065122	0.75028	0.99941	0.9766	-0.03417	0.25987	0.7335	0.89603	-0.15837	0.30903	0.67979	0.94028	-0.08884	0.19693	0.47068	1.0822	0.1139	0.78067	0.99391	1.0309	0.043933	0.008306	0.47452	0.75558	-0.40434	0.67971	0.85438	1.1388	0.18747
832.0067	6.49	olomics Pc	0.79791	0.99951	1.1021	0.14021	0.315	0.99998	0.85879	-0.21962	0.58494	0.99941	1.0699	0.097526	0.016228	0.20428	0.72261	-0.4687	0.48241	0.88343	0.90802	-0.13921	0.06789	0.23044	0.80146	-0.3193	0.96366	0.9974	1.0563	0.079022	0.8809	0.99956	1.0623	0.087186	0.59809	0.81031	1.1168	0.15933
1135.489	5.92	domics Pos	0.79794	0.99987	1.04	0.056644	0.54429	0.99994	0.92919	-0.10595	0.60472	0.99996	0.93877	-0.09115	0.89079	0.9874	0.9835	-0.024	0.022229	0.056462	0.73464	-0.44489	0.27939	0.56636	0.81561	-0.29405	0.52423	0.83957	0.92197	-0.1172	0.31566	0.9524	0.89102	-0.16647	0.29847	0.40864	1.1496	0.20111
328.2285	2.04	lomics Neg	0.79809	0.99977	1.0734	0.10225	0.351	0.99904	0.75989	-0.39614	0.078373	0.99994	0.60598	-0.72265	0.091889	0.58182	0.55832	-0.84083	0.013892	0.33903	0.36328	-1.4608	0.01786	0.46473	0.27932	-1.84	0.004213	0.10075	0.31293	-1.6761	0.021839	0.46971	0.28673	-1.8023	0.011375	0.38536	0.41242	-1.2778
746.8568	10.05	domics Pos	0.79825	0.99987	0.97957	-0.02978	0.8438	0.99994	1.0181	0.025857	0.81572	0.99996	0.98097	-0.02772	0.44697	0.86921	0.93919	-0.09052	0.046471	0.098605	0.805	-0.31293	0.84836	0.92397	0.99541	-0.00664	0.19151	0.64891	1.1095	0.14986	0.098105	0.84824	0.86132	-0.21538	0.1336	0.23838	1.1562	0.20939
657.9165	11.98	olomics Ne	0.79852	0.99918	1.0112	0.016102	0.0039	0.34605	0.84144	-0.24906	0.79479	0.99995	0.98939	-0.01539	0.12822	0.99985	0.92426	-0.11363	0.4404	0.99927	0.9379	-0.0925	0.42535	0.81813	1.0466	0.065766	0.079991	0.43379	1.0536	0.075284	0.61108	0.99512	0.97641	-0.03444	0.90371	0.99941	0.99742	-0.00372
118.0873	0.59	domics Pos	0.79872	0.99987	0.95432	-0.06746	0.52645	0.99994	0.89863	-0.15421	0.75023	0.99996	0.9441	-0.08299	0.65185	0.95465	1.0994	0.13666	0.17836	0.2702	1.235	0.30454	0.03152	0.16025	1.6056	0.68312	0.00081	0.20579	2.1612	1.1118	0.4015	0.96776	1.3269	0.40809	0.1989	0.30729	1.2722	0.34736
769.5555	4.62	domics Pos	0.79903	0.99987	1.0477	0.06719	0.8753	0.99994	0.98535	-0.02129	0.82962	0.99996	0.98153	-0.0269	0.15394	0.58092	0.8253	-0.27701	0.03014	0.071073	0.69144	-0.53232	0.032948	0.16294	0.7572	-0.40125	0.25151	0.68894	0.86931	-0.20206	0.27862	0.94365	0.86502	-0.20919	0.86007	0.89799	1.0311	0.044177
337.2739	1.14	domics Pos	0.7996	0.99987	0.91778	-0.12378	0.75197	0.99994	0.9184	-0.1228	0.87796	0.99996	0.9922	-0.0113	0.69482	0.96092	1.2226	0.28995	0.18519	0.27692	1.7565	0.81271	0.005344	0.074454	2.9423	1.5569	0.002278	0.26175	3.4879	1.8023	0.15543	0.91321	1.8665	0.90037	0.049498	0.14647	1.9878	0.99115
724.8857	11.96	olomics Ne	0.79997	0.99918	1.0121	0.017381	0.15117	0.58235	0.92565	-0.11146	0.66347	0.99995	0.97908	-0.0305	0.46246	0.99985	0.95599	-0.06493	0.94896	0.99927	1.0041	0.005946	0.038444	0.48405	1.0892	0.12327	0.29432	0.67198	1.045	0.0635	0.81441	0.99512	1.0109	0.015699	0.92251	0.99941	0.99771	-0.00331
673.2249	1.83	domics Pos	0.80044	0.99987	1.0374	0.053006	0.56967	0.99994	0.94639	-0.07949	0.37199	0.99996	0.90111	-0.15022	0.60578	0.93327	0.95858	-0.06103	0.041433	0.090431	0.7359	-0.44242	0.082695	0.29844	0.82099	-0.28456	0.74365	0.93244	0.9708	-0.04275	0.1829	0.92582	0.86071	-0.2164	0.39984	0.50803	1.2251	0.29285
301.2162	1.75	lomics Neg	0.80075	0.99977	0.97921	-0.03031	0.54948	0.99904	0.88569	-0.17513	0.097562	0.99994	0.60582	-0.72304	0.069079	0.58182	0.59124	-0.75818	0.000592	0.040886	0.27605	-1.857	0.00519	0.46473	0.32799	-1.6083	0.002937	0.093659	0.39006	-1.3582	0.018186	0.46971	0.32003	-1.6437	0.025085	0.405	0.39594	-1.3366
346.8362	12.71	olomics Ne	0.80097	0.99918	1.0271	0.038555	0.60909	0.9021	0.9608	-0.0577	0.73278	0.99995	1.0179	0.025565	0.51173	0.99985	1.0384	0.054388	0.31576	0.99927	0.93206	-0.10151	0.01435	0.40708	1.1797	0.23845	0.96133	0.98872	0.99722	-0.00402	0.080521	0.99512	1.0976	0.13438	0.4844	0.99941	0.95071	-0.07292
194.0288	0.58	domics Pos	0.80113	0.99987	0.93704	-0.09381	0.97379	0.99994	0.982	-0.02621	0.28618	0.99996	1.3174	0.39774	0.0772	0.46503	1.858	0.89372	0.16123	0.24991	1.497	0.58207	0.081025	0.2948	1.7082	0.77247	0.010329	0.3324	2.3483	1.2316	0.22909	0.94286	1.5596	0.6412	0.39772	0.50601	1.1672	0.22308
460.9758	5.02	olomics Ne	0.80137	0.99918	1.0096	0.013733	0.025709	0.40327	0.88616	-0.17436	0.50771	0.99995	0.97014	-0.04373	0.71677	0.99985	1.0318	0.0451	0.83312	0.99927	1.0145	0.020817	0.49602	0.85509	1.0342	0.048579	0.65719	0.87948	1.0252	0.035963	0.72525	0.99512	0.98544	-0.02116	0.61818	0.99941	1.0145	0.020821
974.8124	0.5	domics Pos	0.8014	0.99987	1.0064	0.009181	0.30361	0.99994	0.95267	-0.06995	0.052688	0.99996	0.92034	-0.11975	0.013299	0.22898	0.86644	-0.20683	1.72E-05	0.000337	0.73496	-0.44427	0.17492	0.46151	0.86774	-0.20466	0.35204	0.75095	0.9412	-0.08743	0.15984	0.91806	0.93499	-0.09698	0.04001	0.13378	1.1846	0.2444
115.0759	6.96	olomics Pc	0.80142	0.99951	1.0592	0.083006	0.7323	0.99998	0.96287	-0.05458	0.087691	0.99941	0.68995	-0.53544	0.032571	0.2515	0.57368	-0.80168	0.002257	0.016884	0.44558	-1.1662	0.003317	0.049867	0.40911	-1.2894	0.00157	0.16989	0.36995	-1.4346	0.062985	0.72831	0.52953	-0.91722	0.007896	0.2688	0.44904	-1.1551
377.7561	4.1	domics Pos	0.80145	0.99987	0.96806	-0.04683	0.33177	0.99994	0.88822	-0.17101	0.48861	0.99996	0.91497	-0.12821	0.22996	0.68665	0.83084	-0.26736	0.041932	0.091364	0.6971	-0.52055	0.18402	0.47109	0.84139	-0.24916	0.1624	0.63418	0.8486	-0.23684	0.83725	0.97988	0.98244	-0.02556	0.45775	0.56231	1.1962	0.25842
210.0512	0.57	domics Pos	0.8016	0.99987	1.0191	0.02733	0.35567	0.99994	0.90883	-0.13792	0.10519	0.99996	1.2007	0.26388	0.009499	0.20348	1.5534	0.63541	0.29152	0.38629	1.1158	0.15806	0.021221	0.12671	1.24	0.31037	0.000611	0.17164	1.5314	0.61483	0.91784	0.98252	1.0396	0.056069	0.53469	0.63195	1.1004	0.13809
429.2487	2.71	domics Pos	0.80162	0.99987	0.98627	-0.01994	0.025387	0.99994	0.84461	-0.24364	0.011825	0.99996	0.84297	-0.24644	0.006706	0.19397	0.74418	-0.42628	0.000276	0.002086	0.64905	-0.6236	0.089559	0.31615	0.76146	-0.39315	0.01378	0.33809	0.80587	-0.31139	0.034182	0.6327	0.75926	-0.39734	0.64198	0.7312	0.95911	-0.06024
834.6507	5.72	domics Pos	0.80181	0.99987	1.0253	0.036032	0.33962	0.99994	0.88423	-0.17751	0.731	0.99996	0.95994	-0.05898	0.62612	0.94364	0.94527	-0.0812	0.03811	0.085285	0.77318	-0.37113	0.46713	0.69171	0.89934	-0.15306	0.50114	0.82791	0.92824	-0.10743	0.34182	0.96524	0.87942	-0.18538	0.44775	0.55233	0.9079	-0.1394
904.6003	5.78	domics Pos	0.80184	0.99987	1.0254	0.036141	0.50114	0.99994	0.93316	-0.09981	0.56329	0.99996	0.94019	-0.08898	0.45346	0.87408	0.91149	-0.1337	0.012715	0.036784	0.70386	-0.50663	0.023763	0.13544	0.7807	-0.35716	0.14373	0.60716	0.82794	-0.27241	0.070334	0.76834	0.82503	-0.27748	0.10033	0.20231	1.2379	0.30795
569.3433	1.03	domics Pos	0.80193	0.99987	0.97816	-0.03185	0.96842	0.99994	0.99064	-0.01357	0.61388	0.99996	1.0848	0.11739	0.87235	0.9874	1.0101	0.014529	0.36538	0.45799	0.82302	-0.281	0.073723	0.28032	1.4479	0.53398	0.021541	0.33809	1.7945	0.84362	0.68076	0.97988	1.0726	0.10117	0.085629	0.18633	1.3964	0.48167
740.5201	8.88	olomics Ne	0.80206	0.99918	0.92039	-0.11968	0.68869	0.94213	0.89998	-0.15204	0.54258	0.99995	0.83979	-0.25191	0.86645	0.99985	0.97147	-0.04176	0.19895	0.99927	1.6135	0.69015	0.033951	0.47167	2.4253	1.2782	0.046168	0.38697	2.2942	1.198	0.19422	0.99512	1.8654	0.89952	0.08107	0.99941	1.4864	0.57178
158.0063	10.18	domics Pos	0.8021	0.99987	1.0143	0.020531	0.24079	0.99994	0.93936	-0.09025	0.40354	0.99996	0.93278	-0.10039	0.45165	0.87374	1.0347	0.049196	0.003154	0.012739	0.75484	-0.40576	0.52686	0.73453	0.93852	-0.09154	0.79114	0.95091	1.0208	0.029726	0.62286	0.97813	1.03	0.042592	0.024641	0.1117	1.1975	0.26008
379.2652	2.25	domics Pos	0.80309	0.99987	0.96498	-0.05143	0.025413	0.99994	0.72879	-0.45642	0.024935	0.99996	0.65446	-0.61163	0.001562	0.17581	0.54144	-0.88514	0.74191	0.78085	0.97628	-0.03463	0.082234	0.29782	0.5898	-0.76171	0.033693	0.36346	0.63506	-0.65502	0.063777	0.73915	0.63903	-0.64604	0.53882	0.63587	1.2761	0.35173
591.3251	1.03	domics Pos	0.80315	0.99987	0.98251	-0.02546	0.92146	0.99994	0.99943	-0.00083	0.65315	0.99996	1.1011	0.13894	0.82764	0.98621	1.0148	0.021248	0.52363	0.59317	0.8627	-0.21307	0.064733	0.25631	1.4832	0.56869	0.016561	0.33809	1.8776	0.90891	0.43976	0.96776	1.1484	0.19965	0.087042	0.18633	1.4293	0.51534
716.5235	5.06	domics Pos	0.80331	0.99987	0.92829	-0.10735	0.35708	0.99994	0.78004	-0.35837	0.39861	0.99996	0.79618	-0.32884	0.44122	0.86677	0.813	-0.29868	0.63725	0.69148	0.88144	-0.18207	0.12612	0.38627	1.4297	0.51574	0.032723	0.36346	1.9338	0.95147	0.071374	0.77255	1.853	0.88987	0.014539	0.098791	2.2921	1.1966

341.2978	5.78	olomics Po	0.80358	0.99951	0.9614	-0.05679	0.74099	0.99998	0.94342	-0.08403	0.42502	0.99941	0.87326	-0.19551	0.85758	0.99905	0.96708	-0.0483	0.46029	0.86202	0.88072	-0.18324	0.18561	0.45607	1.2874	0.36448	0.027025	0.33696	1.5626	0.64399	0.63195	0.99956	1.0765	0.10638	0.11516	0.36506	1.2486	0.32031
1179.566	1.11	lomics Pos	0.80384	0.99987	1.035	0.049578	0.72949	0.99994	1.1006	0.13823	0.90552	0.99996	0.92914	-0.10603	0.67184	0.95717	1.0397	0.05619	0.48103	0.55869	1.2723	0.34744	0.038462	0.17973	2.4302	1.2811	0.023173	0.33809	3.0038	1.5868	0.26137	0.94365	1.6964	0.7625	0.12255	0.22705	1.9936	0.99535
924.5683	5.58	lomics Pos	0.80393	0.99987	0.97621	-0.03474	0.81768	0.99994	1.145	0.19537	0.53446	0.99996	1.1085	0.14859	0.49267	0.88874	1.1728	0.22992	0.01108	0.033215	0.69495	-0.52502	0.24985	0.53672	0.8018	-0.31868	0.18797	0.64793	0.81235	-0.29982	0.82279	0.97988	0.97799	-0.03211	0.57249	0.66714	1.1429	0.19275
317.9369	12.6	olomics Po	0.80413	0.99951	1.0361	0.05111	0.38217	0.99998	1.1031	0.14159	0.48667	0.99941	1.0998	0.13726	0.92135	0.99905	1.0392	0.055435	0.17345	0.44821	1.2304	0.29909	0.038982	0.15763	1.4574	0.54341	0.18649	0.76329	1.2511	0.32324	0.88121	0.99956	1.0873	0.12078	0.24517	0.51325	1.1398	0.1888
263.2375	2.28	lomics Pos	0.8044	0.99987	0.98578	-0.02066	0.31997	0.99994	0.82231	-0.28224	0.02859	0.99996	0.60815	-0.7175	0.009913	0.20999	0.47062	-1.0874	0.000268	0.00204	0.33126	-1.594	0.007051	0.081828	0.33429	-1.5808	0.011825	0.3324	0.40352	-1.3093	0.010485	0.52955	0.36515	-1.4534	0.031219	0.12145	0.48078	-1.0565
285.2798	7.49	olomics Po	0.80451	0.99951	0.90246	-0.14807	0.74958	0.99998	0.90341	-0.14654	0.079745	0.99941	0.59318	-0.75345	0.042449	0.2802	0.46966	-1.0903	0.015159	0.066974	0.44691	-1.1619	0.007174	0.070405	0.3357	-1.5747	0.012272	0.26861	0.40297	-1.3113	0.011333	0.47452	0.39017	-1.3578	0.033395	0.2688	0.46621	-1.1009
466.3298	6.08	olomics Po	0.8046	0.99951	0.95953	-0.05959	0.99197	0.99998	0.98363	-0.02381	0.34707	0.99941	0.8122	-0.3001	0.94838	0.99905	0.97482	-0.03679	0.006023	0.033508	0.33267	-1.5878	0.75556	0.93161	0.92294	-0.11569	0.3639	0.96545	1.2176	0.284	0.80959	0.99956	1.0511	0.071878	0.21306	0.4802	1.3187	0.39915
833.6491	5.72	lomics Pos	0.80461	0.99987	1.0238	0.033938	0.28934	0.99994	0.87219	-0.19728	0.58832	0.99996	0.93886	-0.09102	0.49353	0.88874	0.92505	-0.11239	0.027535	0.066493	0.75212	-0.41096	0.38262	0.63832	0.87214	-0.19736	0.37877	0.76056	0.90222	-0.14844	0.30022	0.9508	0.86757	-0.20495	0.4448	0.55012	0.90821	-0.13891
371.2235	6.41	olomics Ne	0.80482	0.99918	0.96889	-0.0456	0.46781	0.82998	0.89227	-0.16445	0.21854	0.99995	0.81994	-0.28641	0.21609	0.99985	0.83459	-0.26086	4.18E-05	0.01419	0.22561	-2.1481	0.004361	0.36417	0.57719	-0.79287	0.060343	0.40009	0.72791	-0.45817	0.053436	0.99512	0.67335	-0.57057	0.19423	0.99941	0.77233	-0.37271
202.0322	0.6	lomics Neg	0.80528	0.99977	0.94979	-0.07431	0.78059	0.99904	1.1556	0.20869	0.7153	0.99994	0.9306	-0.10377	0.4682	0.62709	1.4155	0.50134	0.35559	0.99989	2.8994	1.5357	0.015891	0.46473	5.4203	2.4384	0.000286	0.084527	5.9604	2.5754	0.14791	0.59164	2.3232	1.2161	0.70874	0.8292	1.1384	0.18704
1076.793	0.5	lomics Pos	0.80529	0.99987	1.0163	0.023383	0.9199	0.99994	0.99453	-0.00792	0.089209	0.99996	0.91106	-0.13439	0.008101	0.19917	0.86141	-0.21523	0.000263	0.002017	0.73829	-0.43774	0.10529	0.3461	0.82558	-0.27652	0.51048	0.83279	0.94556	-0.08076	0.21382	0.93471	0.93416	-0.09825	0.11024	0.21382	1.1622	0.21691
525.98	12.01	olomics Ne	0.80541	0.99918	1.0312	0.044348	0.086538	0.50704	0.90724	-0.14045	0.083936	0.99995	0.82511	-0.27734	0.018728	0.99985	0.85451	-0.22684	0.61664	0.99927	0.95254	-0.07016	0.74291	0.94671	0.97763	-0.03263	0.85089	0.95689	0.99486	-0.00743	0.3751	0.99512	0.89275	-0.16368	0.39203	0.99941	0.90464	-0.14458
694.833	0.53	lomics Pos	0.80556	0.99987	1.0183	0.026154	0.29963	0.99994	0.91471	-0.12861	0.46579	0.99996	0.93979	-0.0896	0.053789	0.39902	0.83547	-0.25933	0.002359	0.010242	0.68166	-0.55287	0.25247	0.53916	0.85733	-0.22208	0.37127	0.76056	0.88316	-0.17926	0.29207	0.9483	0.90964	-0.13663	0.25488	0.36372	1.159	0.21292
304.2351	2.17	lomics Neg	0.80557	0.99977	0.97123	-0.04212	0.48886	0.99904	0.88158	-0.18183	0.095285	0.99994	0.70788	-0.49842	0.69182	0.76152	0.92447	-0.1133	0.000221	0.032757	0.17228	-2.5371	0.044418	0.82173	0.47218	-1.0826	0.037296	0.37635	0.50634	-0.98181	0.046907	0.46971	0.40908	-1.2896	0.050031	0.405	0.61369	-0.70441
1110.786	0.5	lomics Pos	0.80573	0.99987	0.98919	-0.01568	0.31426	0.99994	0.94615	-0.07987	0.055797	0.99996	0.91874	-0.12227	0.00945	0.20348	0.86123	-0.21554	0.000117	0.001141	0.74611	-0.42255	0.16718	0.45275	0.85018	-0.23416	0.32721	0.73618	0.93083	-0.10341	0.05637	0.70737	0.91717	-0.12474	0.070347	0.17145	1.1568	0.21019
133.101	6.48	olomics Po	0.80744	0.99951	0.97757	-0.03272	0.63483	0.99998	0.95443	-0.06729	0.045263	0.99941	0.7097	-0.49472	0.068907	0.34345	0.59238	-0.75539	0.006953	0.036996	0.52876	-0.91932	0.008872	0.075866	0.45269	-1.1434	0.00455	0.20152	0.45225	-1.1448	0.015255	0.47577	0.47344	-1.0788	0.031941	0.2688	0.49596	-1.0117
305.2511	6.41	olomics Po	0.80744	0.99951	0.95887	-0.0606	0.66203	0.99998	0.92253	-0.11633	0.35024	0.99941	0.84123	-0.24942	0.31997	0.81415	0.85022	-0.23409	1.65E-05	0.000434	0.17665	-2.501	0.021563	0.10951	0.6169	-0.6969	0.12124	0.6564	0.71297	-0.48809	0.15835	0.93927	0.73499	-0.4442	0.12568	0.3747	0.6943	-0.52638
475.9748	12.01	olomics Ne	0.80759	0.99918	1.0113	0.016246	0.45808	0.82821	0.90103	-0.15036	0.80854	0.99995	1.001	0.001395	0.01471	0.99985	0.74868	-0.41757	0.032128	0.99927	0.82344	-0.28026	0.82566	0.96067	1.0427	0.060262	0.34098	0.71097	0.92593	-0.11103	0.71836	0.99512	0.97677	-0.03391	0.57299	0.99941	0.96665	-0.04894
773.6446	5.87	lomics Pos	0.80767	0.99987	0.96811	-0.04676	0.31533	0.99994	0.82615	-0.27552	0.8326	0.99996	0.97236	-0.04044	0.57264	0.9155	0.92614	-0.11069	0.15355	0.24144	0.81609	-0.2932	0.042417	0.19346	0.76887	-0.37918	0.95142	0.98957	0.98553	-0.02102	0.063314	0.73915	0.74023	-0.43395	0.83932	0.88139	1.027	0.038419
651.5352	5.34	lomics Pos	0.80787	0.99987	1.0472	0.06655	0.13268	0.99994	0.86125	-0.21549	0.33123	0.99996	0.88306	-0.17941	0.2412	0.70182	0.84371	-0.24518	0.001108	0.005935	0.62949	-0.66775	0.011119	0.09137	0.73938	-0.43562	0.9156	0.98011	1.0486	0.068436	0.57092	0.9703	1.0839	0.11621	0.26794	0.37825	1.263	0.33681
344.2412	2.28	lomics Pos	0.80853	0.99987	1.0676	0.0944	0.77234	0.99994	0.97114	-0.04226	0.14469	0.99996	0.73191	-0.45026	0.024463	0.27679	0.71847	-0.477	0.000742	0.00444	0.43054	-1.2158	0.03585	0.17185	0.52397	-0.93244	0.056193	0.42428	0.59074	-0.75939	0.18636	0.92582	0.71768	-0.47858	0.048322	0.14485	0.54517	-0.87523
873.049	1.44	lomics Pos	0.8092	0.99987	1.1567	0.20995	0.078658	0.99994	0.79112	-0.33803	0.82286	0.99996	1.0182	0.026047	0.001445	0.17581	0.61947	-0.69089	0.017443	0.046885	0.63737	-0.6498	0.061465	0.24801	0.73391	-0.44633	0.46651	0.80647	1.2665	0.34079	0.90739	0.98236	1.1665	0.22216	0.060819	0.1578	1.487	0.57245
83.0869	5.99	olomics Po	0.80952	0.99951	1.0302	0.042909	0.026252	0.99998	0.8218	-0.28313	0.05803	0.99941	0.85334	-0.22881	0.010893	0.19754	0.66671	-0.58487	4.53E-07	0.000231	0.2329	-2.1022	0.005112	0.061592	0.61798	-0.69437	0.053234	0.46031	0.71863	-0.47668	0.040593	0.63934	0.77895	-0.36039	0.55745	0.78813	0.96303	-0.05435
633.3054	2.37	olomics Po	0.80965	0.99951	0.90202	-0.14876	0.95465	0.99998	0.95268	-0.06994	0.92246	0.99941	0.9407	-0.08819	0.35867	0.84949	0.71112	-0.49184	0.010963	0.052245	0.31242	-1.6784	0.20215	0.47698	0.58574	-0.77166	0.86864	0.9951	0.94142	-0.0871	0.67763	0.99956	0.86355	-0.21165	0.86928	0.94847	0.9066	-0.14146
156.0477	0.58	lomics Pos	0.8099	0.99987	0.98635	-0.01984	0.35008	0.99994	0.92209	-0.11702	0.99969	0.99996	1.0038	0.005439	0.47428	0.88482	1.1018	0.1398	0.18176	0.27368	1.107	0.14661	0.023542	0.13458	1.4163	0.50217	0.000497	0.17164	1.5817	0.6615	0.12774	0.88537	1.2181	0.28468	0.090309	0.19077	1.2149	0.28082
850.5591	4.75	lomics Neg	0.81006	0.99977	1.1367	0.18482	0.95482	0.99904	1.037	0.052409	0.4438	0.99994	0.88743	-0.1723	0.25755	0.58182	1.3237	0.40459	0.87917	0.99989	1.1293	0.17546	0.9129	0.99653	1.1495	0.20097	0.13556	0.45153	0.68188	-0.55241	0.047071	0.46971	0.53994	-0.88913	0.84191	0.89643	0.96204	-0.05584
165.0549	0.59	lomics Pos	0.81032	0.99987	0.95925	-0.06002	0.88731	0.99994	0.9545	-0.06718	0.41449	0.99996	1.2394	0.30961	0.036549	0.33501	1.7891	0.83922	0.50588	0.57918	1.1953	0.25743	0.19042	0.47713	1.2291	0.29756	0.008026	0.32129	1.9317	0.94987	0.40594	0.96776	1.2988	0.37716	0.20446	0.31284	1.4312	0.51721
111.9856	12.56	olomics Po	0.81037	0.99951	1.0895	0.12369	0.64446	0.99998	0.95746	-0.06272	0.62773	0.99941	1.111	0.15179	0.033524	0.25203	1.3184	0.39877	0.31899	0.69298	1.1818	0.24099	0.25544	0.54514	1.2052	0.26931	0.15841	0.73598	1.1753	0.23308	0.97671	0.99956	1.0211	0.030067	0.52751	0.76749	1.047	0.066239
633.3032	1.12	lomics Pos	0.81095	0.99987	0.96186	-0.0561	0.91771	0.99994	0.96494	-0.05149	0.85085	0.99996	1.017	0.02428	0.82538	0.98534	1.0683	0.095375	0.43439	0.51955	0.84925	-0.23573	0.025176	0.13997	1.5194	0.6035	0.017424	0.33809	1.8958	0.92281	0.20425	0.92582	1.2801	0.35627	0.034207	0.12615	1.6191	0.69518
182.9626	10.17	lomics Pos	0.81102	0.99987	0.97009	-0.04382	0.14248	0.99994	0.82174	-0.28325	0.3863	0.99996	0.87459	-0.19333	0.14961	0.57784	0.81918	-0.28775	0.007481	0.024633	0.63567	-0.65364	0.37214	0.6301	1.3619	0.44559	0.72532	0.92498	1.0862	0.11923	0.15305	0.91321	0.81899	-0.28808	0.76763	0.8266	0.95183	-0.07123
264.2419	6.48	olomics Po	0.81122	0.99951	0.98485	-0.02202	0.59127	0.99998	0.94508	-0.08149	0.12115	0.99941	0.71231	-0.48942	0.045585	0.2866	0.56879	-0.81403	0.004256	0.026184	0.43888	-1.1881	0.010584	0.081313	0.44294	-1.1748	0.019712	0.30142	0.39726	-1.3318	0.017233	0.50732	0.42481	-1.2351	0.031243	0.2688	0.41131	-1.2817
936.5629	5.94	lomics Pos	0.81149	0.99987	0.96678	-0.04874	0.91931	0.99994	0.98516	-0.02156	0.75122	0.99996	1.0538	0.075589	0.33536	0.79326	0.88408	-0.17775	0.045712	0.097374	0.75872	-0.39836	0.026393	0.14337	0.68651	-0.54266	0.21779	0.66052	0.84825	-0.23744	0.25484	0.94365	0.8346	-0.26085	0.53374	0.63125	1.1	0.13754
691.5265	12.02	olomics Po	0.81159	0.99951	0.99349	-0.00942	0.018783	0.99998	0.66695	-0.58435	0.42361	0.99941	0.9402	-0.08896	0.44375	0.92368	0.9269	-0.10952	0.001684	0.013605	0.7471	-0.42062	0.024531	0.11655	0.76927	-0.37843	0.94864	0.9974	0.99783	-0.00313	0.12395	0.8972	0.86809	-0.20409	0.091624	0.34441	0.81944	-0.28728
674.7736	0.56	lomics Pos	0.81162	0.99987	0.98588	-0.02051	0.33748	0.99994	0.95289	-0.06962	0.070437	0.99996	0.88524	-0.17586	0.070507	0.45336	0.86224	-0.21385	0.007186	0.023825	0.74401	-0.4266	0.00213	0.060164	0.71552	-0.48293	0.001023	0.22944	0.77015	-0.37679	0.24871	0.94365	0.94015	-0.08904	0.21556	0.32455	1.081	0.11241
542.3246	4.84	olomics Po	0.81173	0.99951	1.0029	0.0042	0.50538	0.99998	1.0904	0.1248	0.22211	0.99941	1.2058	0.26998	0.065205	0.33343	1.3229	0.40374	0.084619	0.25075	1.3404	0.42265	0.007197	0.070405	1.7244	0.78612	0.006407	0.21836	1.7806	0.83236	0.9937	0.99956	0.98842	-0.0168	0.97858	0.98952	0.98259	-0.02534
760.8233	0.53	lomics Pos	0.81218	0.99987	0.98586	-0.02054	0.048377	0.99994	0.84452	-0.2438	0.25011	0.99996	0.91794	-0.12353	0.065966	0.43825	0.88274	-0.17994	0.000913	0.00521	0.7309	-0.45226	0.36904	0.62848	0.88782	-0.17166	0.31228	0.72948	0.89494	-0.16013	0.23848	0.94365	0.91028	-0.13562	0.11322	0.21737	1.2299	0.29851
1050.783	0.5	lomics Pos	0.81228	0.99987	1.0374	0.052968	0.17143	0.99994	0.85528	-0.22553	0.25039	0.99996	0.9423	-0.08575	0.23515	0.69398	0.90014	-0.15178	0.001117	0.005959	0.77995	-0.35855	0.85379	0.92636	0.99331	-0.00968	0.58717	0.86163	1.0229	0.032683	0.58641	0.97073	1.0372	0.05263	0.016306	0.099614	1.1515	0.20357
852.5635	8.96	olomics Po	0.81228	0.99951	1.0482	0.067852	0.28564	0.99998	0.85465	-0.2266	0.82294	0.99941	0.97129	-0.04203	0.068337	0.34279	0.75622	-0.40312	0.029233	0.11271	0.67508	-0.56688	0.039945	0.15988	0.73118	-0.45171	0.86633	0.9951	1.0455	0.064211	0.85725	0.99956	0.98136	-0.02715	0.25581	0.52564	1.4004	0.4858
212.865	0.33	olomics Ne	0.8132	0.99918	0.99333	-0.00965	0.013427	0.37315	0.84759	-0.23856	0.41598	0.99995	0.94899	-0.07553	0.81664	0.99985	1.017	0.024387	0.47437	0.99927	0.96161	-0.05648	0.31975	0.73392	1.0962	0.13256	0.14234	0.52776	1.041	0.058012	0.81221	0.99512	0.99072	-0.01345	0.077831	0.99941	0.89213	-0.16467
522.3461	1.09	lomics Pos	0.81331	0.99987	0.92745	-0.10866	0.99078	0.99994	0.94615	-0.07986	0.8513	0.99996	1.0055	0.007915	0.74359	0.97266	1.0705	0.098259	0.57769	0.63921	1.1785	0.23696	0.026915	0.14537	2.0791	1.056	0.01053	0.3324	2.5832	1.3692	0.24171	0.94365	1.4374	0.52346	0.076714	0.17825	1.704	0.76892
522.3558	1.37	lomics Pos	0.81367	0.99987	1.0572	0.080279	0.93247	0.99994	1.0003	0.000438	0.95362	0.99996	0.98386	-0.02347	0.89894	0.9874	0.99449	-0.00797	0.80008	0.82922	0.89471	-0.1605	0.13889	0.40832	1.4734	0.55914	0.046402	0.39255	1.8009	0.84869	0.43609	0.96776	1.2047	0.2687	0.071124	0.17219	2.1207	1.0846
430.7415	8.57	olomics Po	0.81371	0.99951	0.9863	-0.0199	0.60535	0.99998	0.97089	-0.04262	0.96861	0.99941	1.0077	0.011114	0.35763	0.8483	0.94155	-0.08689	0.40383	0.80346	1.0627	0.087769	0.27811	0.5722	0.93252	-0.1008	0.072916	0.54945	0.88668	-0.17352	0.019766	0.51556	0.84124	-0.24941	0.83146	0.93201	1.0141	0.020233
279.6484	5.11	olomics Po	0.81395	0.99951	0.94217	-0.08594	0.8339	0.99998	0.91758	-0.12409	0.98408	0.99941	0.96311	-0.05423	0.65196	0.99905	1.1432	0.19312	0.23658	0.56155	1.3593	0.44283	0.033873	0.14271	1.7412	0.80005	0.031879	0.35567	1.837	0.87732	0.42254	0.99956	1.2623	0.33607	0.24113	0.50928	1.303	0.38186
137.0471	0.36	olomics Po	0.81474	0.99951	1.0164	0.023422	0.92846	0.99998	0.97827	-0.03169	0.68384	0.99941	0.91036	-0.13549	0.35302	0.84525	0.83215	-0.26509	0.007125	0.03722	0.23878	-2.0663	0.37309	0.68487	0.82233	-0.28221	0.60012	0.99391	0.88984	-0.16839	0.34751	0.99956	0.80269	-0.31708	0.52314	0.76572	1.0974	0.13415
837.6171	5.78	lomics Pos	0.81504	0.99987	1.0246	0.035047	0.60518	0.99994	0.95013	-0.0738	0.62539	0.99996	0.95506	-0.06634	0.31341	0.77421	0.89541	-0.15938	0.012528	0.036354	0.71626	-0.48145	0.022232	0.13062	0.78789	-0.34393	0.18621	0.64793	0.85104	-0.23269	0.12748	0.88537	0.85812	-0.22075	0.085608	0.18633	1.3189	0.39939
289.1496	2.62	olomics Po	0.81544	0.99951	1.0623	0.087214	0.72846	0.99998	0.87774	-0.18814	0.17	0.99941	0.62659	-0.67441	0.12646	0.49162	0.53611	-0.8994	0.009297	0.045688	0.31949	-1.6462	0.04391	0.17076	0.43671	-1.1952	0.098722	0.61095	0.51202	-0.96574	0.089529	0.80182	0.54517	-0.87523	0.8879	0.9536	1.0518	0.072871
882.6135	5.5	lomics Neg	0.81545	0.99977	1.174	0.23145	0.83226	0.99904	1.0269	0.03825	0.29402	0.99994	0.84957	-0.2352	0.12136	0.58182	1.3353	0.41713	0.97107	0.99989	1.243	0.31385	0.94937	0.99653	1.2137	0.27937	0.03647	0.37635	0.70572	-0.50283	0.046758	0.46971	0.55353	-0.85326	0.3448	0.53436	1.1755	0.23323
823.6031	8.88	olomics Po	0.81571	0.99951	1.0373	0.052841	0.97284	0.99998	1.0179	0.025631	0.89719	0.99941	0.99192	-0.01171	0.86067	0.99905	0.99018	-0.01423	0.61035	0.97478	0.94014	-0.08905	0.44544	0.75074	0.89683	-0.15709	0.22979	0.83662	0.85966	-0.21816	0.033252	0.59177	0.7671	-0.38251	0.11506	0.36506	0.82006	-0.28619
732.6078	8.96	olomics Po	0.81632	0.99951	1.0736	0.10246	0.6913	0.99998	1.0328	0.046532	0.53919	0.99941	1.0294	0.041781	0.90485	0.99905	1.0459	0.06481	0.062652	0.19815	1.1254	0.17046	0.47249	0.7756	0.94319	-0.08438	0.38886	0.97696	0.90852	-0.13842	0.030596	0.58535	0.64573	-0.63099	0.001154	0.2688	0.65846	-0.60282
568.34	5.08	olomics Po	0.81652	0.99951	0.98836	-0.0169	0.62434	0.99998	1.0605	0.084779	0.5835	0.99941	1.0873	0.12081	0.17117	0.58669	1.2032	0.26686	0.18199	0.46305	1.1923	0.25371	0.002312	0.045971	1.6899	0.75697	0.004667	0.20152	1.755	0.81148	0.27899	0.99956	1.1246	0.16943	0.35147	0.62856	1.1331	0.18025
903.6528	6.05	lomics Pos	0.81672	0.99987	0.97737	-0.03302	0.20449	0.99994	0.8349	-0.26033	0.31274	0.99996	0.88472	-0.17671	0.21144	0.65976	0.85847	-0.22017	0.023187	0.05832	0.74392	-0.42679	0.26489	0.55194	0.81291	-0.29883	0.47424	0.80866	0.92817	-0.10753	0.18531	0.92582	0.84543	-0.24225	0.61774	0.70997	0.94628	-0.07966
900.712	7.3	lomics Pos	0.81738	0.99987	0.92061	-0.11934	0.65147	0.99994	0.84299	-0.24641	0.51632	0.99996	0.79978	-0.32232	0.32184	0.7853	0.69897	-0.5167	0.2409	0.33519	0.6427	-0.63779	0.51162	0.72252	0.79954	-0.32275	0.68972	0.90885	0.8821	-0.18099	0.035809	0.64014	0.47967	-1.0599	0.45753	0.56222	0.77165	-0.37398
1122.742	10.06	lomics Pos	0.8177	0.99987	1.0206	0.029458	0.45419	0.99994	0.92544	-0.11178	0.56483	0.99996	1.0818	0.11344	0.46231	0.88001	0.95676	-0.06377	0.003934	0.015076	0.79586	-0.32941	0.91598	0.9561	1.0147	0.021011	0.18292	0.64793	1.1079	0.14783	0.33958	0.96524	1.1056	0.1448	0.000257	0.07528	1.2997	0.37817
861.6788	6.09	lomics Pos	0.81778	0.99987	0.98896	-0.01602	0.64668	0.99994	0.94661	-0.07916	0.21585	0.99996	0.84412	-0.24447	0.038294	0.34165	0.77874	-0.36079	0.049871	0.10408	0.73584	-0.44254	0.12456	0.38426	0.73823	-0.43785	0.23257	0.6761	0.86798	-0.20426	0.16903	0.91806	0.82771	-0.2728	0.36476	0.47356	0.88929	-0.16928
707.9762	12.03	olomics Ne	0.81787	0.99918	1.0242	0.034533	0.89335	0.9934	0.9787	-0.03107	0.43021	0.99995	1.0976	0.13433	0.24401	0.99985	0.85158	-0.23178	0.28201	0.99927	1.2945	0.37236	0.11999	0.58607	1.2408	0.31128	0.22747	0.60705	1.2865	0.36348	0.019394	0.99512	1.4158	0.50164	0.99577	0.99945	0.99513	-0.00704
327.2276	2.85	lomics Pos	0.81796	0.99987	0.96944	-0.04477	0.2967	0.99994	0.8424	-0.24742	0.086393	0.99996	0.79134	-0.33762	0.007838	0.19917	0.58723	-0.76799	0.002189	0.00966	0.44754	-1.1599	0.015028	0.10343	0.43568	-1.1987	0.005309	0.3088	0.4395	-1.1861	0.05182	0.68583	0.56739	-0.8176	0.12334	0.22764	0.67028	-0.57717
124.008	0.59	lomics Neg	0.8183	0.99977	1.0464	0.065407	0.83877	0.99904	1.0154	0.022073	0.68332	0.99994	1.0537	0.075415	0.44591	0.60735	1.1311	0.17777	0.018703	0.39544	0.57863	-0.78929	0.51701	0.99653	0.8714	-0.19859	0.99584	0.99584	0.98548	-0.0211	0.39273	0.6961	0.83555	-0.2592	0.72692	0.8405	0.92077	-0.11908
389.199	2.85	lomics Pos	0.8183	0.99987	1.009	0.012878	0.25319	0.99994	0.83298	-0.26364	0.66483	0.99996	0.92145	-0.11802	0.1293	0.55039	0.71222	-0.4896	0.020134	0.052558	0.59761	-0.74272	0.058898	0.24091	0.59487	-0.74935	0.022365	0.33809	0.61993	-0.68983	0.043465	0.65325	0.62877	-0.66939	0.35663	0.46585	0.83986	-0.25178
857.6101	5.49	lomics Neg	0.81842	0.99977	1.2399	0.31027	0.85995	0.99904	1.1822	0.24148	0.44296	0.99994	0.87662	-0.18998	0.14867	0.58182	1.5623	0.64365	0.98046	0.99989	1.5458	0.62836	0.84951	0.99653	1.4072	0.49283	0.20706	0.515	0.73846	-0.4374	0.029416	0.46971	0.49955	-1.0013	0.60105	0.7444	1.1948	0.25676
899.7085	7.3	lomics Pos	0.81856	0.99987	0.92178	-0.11751	0.73018	0.99994	0.88221	-0.18081	0.45232	0.99996	0.77616	-0.36558	0.29243	0.75244	0.68996	-0.53542	0.25276	0.34756	0.65945	-0.60066	0.39465	0.64359	0.75557	-0.40436	0.64455	0.88775	0.87019	-0.20061	0.030909	0.62689	0.48557	-1.0423	0.36493	0.47362	0.72979	-0.45445
590.3437	5.26	olomics Ne	0.81864	0.99918	0.97522	-0.0362	0.85437	0.98874	0.9716	-0.04157	0.57881	0.99995	1.1528	0.20508	0.63752	0.99985	1.1384	0.18705	0.40234	0.99927	1.2455	0.31671	0.012501	0.40245	1.6684	0.73844	0.015341	0.28186	1.6203	0.69624	0.75483	0.99512	1.06	0.084088	0.44331	0.99941	1.1633	0.21817
570.3543	5.22	olomics Po	0.81876	0.99951	0.99678	-0.00466	0.81921	0.99998	1.021	0.029932	0.36095	0.99941	1.0949	0.13082	0.48542	0.96315	1.1397	0.18859	0.048769	0.16536	1.3093	0.38875	0.008888	0.075866	1.6504	0.72284	0.011081	0.25477	1.587	0.6663	0.20323	0.99956	1.1797	0.23845	0.10891	0.36303	1.2317	0.30064
649.4752	2.07	lomics Pos	0.8189	0.99987	1.0412	0.058231	0.3027	0.99994	0.74674	-0.42132	0.10623	0.99996	0.63206	-0.66186	0.021873	0.26563	0.42056	-1.2496	0.000175	0.001533	0.13039	-2.9391	0.000808	0.040694	0.20426	-2.2915	0.011243	0.3324	0.36108	-1.4696	0.032627	0.63152	0.40752	-1.2951	0.19351	0.30167	0.65931	-0.60098
270.6443	1.12	lomics Pos	0.81902	0.99987	0.93057	-0.10382	0.5032	0.99994	0.79445	-0.33197	0.91266	0.99996	1.0224	0.031916	0.68762	0.96092	1.0392	0.055479	0.57023	0.63261	0.81806	-0.28972	0.13216	0.39774	1.4147	0.50047	0.022855	0.33809	1.8597	0.8951	0.11852	0.88537	1.4101	0.49577	0.096508	0.19752	1.7113	0.77511
614.4017	2.28	lomics Pos	0.81939	0.99987	1.0347	0.049188	0.30596	0.99994	0.82848	-0.27147	0.13159	0.99996	0.74106	-0.43234	0.002109	0.17581	0.5601	-0.83623	0.000158	0.001419	0.43059	-1.2156	0.00558	0.075454	0.50114	-0.9967	0.043993	0.38909	0.55254	-0.85586	0.00716	0.52955	0.44308	-1.1744	0.095732	0.19731	0.65907	-0.6015
601.5189	5.13	lomics Pos	0.81979	0.99987	1.0098	0.014015	0.81059	0.99994	0.8635	-0.21173	0.4307	0.99996	0.70683	-0.50057	0.99356	0.99827	0.99618	-0.00552	0.30088	0.39439	1.4621	0.54801	0.03466	0.16814	2.3229	1.2159	0.042357	0.38314	2.4236	1.2771	0.17804	0.92219	1.9408	0.95667	0.056879	0.15414	2.0459	1.0327
584.2619	6.98	olomics Po	0.8211	0.99951	1.0086	0.012385	0.69741	0.99998	0.9609	-0.05754	0.57656	0.99941	0.91757	-0.12411	0.22691	0.67593	0.82595	-0.27588	0.002384	0.017364	0.53562	-0.90071	0.004037	0.054345	0.47681	-1.0685	0.003725	0.20152	0.50586	-0.98318	0.081186	0.78717	0.6021	-0.73192	0.037416	0.2799	0.61895	-0.6921
614.4028	6.48	olomics Po	0.82127	0.99951	0.99481	-0.0075	0.60137	0.99998	0.93828	-0.09191	0.030978	0.99941	0.60395	-0.72749	0.10262	0.42814	0.58279	-0.77895	0.038513	0.13817	0.60533	-0.7242	0.020752	0.10769	0.41243	-1.2778	0.031739	0.35567	0.45406	-1.139	0.011692	0.47452	0.32746	-1.6106	0.005602	0.2688	0.3404	-1.5547
221.1942	6.45	olomics Po	0.82159	0.99951	1.0583	0.081788	0.89172	0.99998	1.0465	0.065544	0.21251	0.99941	0.83635	-0.25782	0.018315	0.20936	0.702	-0.51045	0.000158	0.002322	0.34865	-1.5202	0.005031	0.061419	0.53297	-0.90788	0.019336	0.30136	0.57686	-0.79371	0.035064	0.60963	0.56828	-0.81533	0.054623	0.29746	0.60932	-0.71474
297.2409	5.87	olomics Po	0.82184	0.99951	0.98073	-0.02808	0.65611	0.99998	0.96699	-0.04842	0.085094	0.99941	0.81721	-0.29122	0.16075	0.56641	0.88382	-0.17817	0.61657	0.97478	0.96067	-0.05789	0.11366	0.32578	0.88566	-0.17517	0.67431	0.99391	0.96622	-0.04958	0.018238	0.51556	0.81218	-0.30013	0.091877	0.34495	0.85581	-0.22463
454.2934	5.29	olomics Po	0.82192	0.99951	0.91814	-0.12322	0.87884	0.99998	0.94579	-0.0804	0.88515	0.99941	0.91673	-0.12544	0.66764	0.99905	1.1172	0.15984	0.60999	0.97478	1.0893	0.12339	0.044709	0.17251	1.8343	0.87521	0.017177	0.29966	2.1486	1.1034	0.10315	0.84495	1.5541	0.63605	0.074166	0.31866	1.6291	0.70409
419.2223	2.18	lomics Pos	0.82198	0.99987	0.99016	-0.01427	0.22938	0.99994	0.83534	-0.25956	0.34679	0.99996	0.85808	-0.22081	0.17265	0.60551	0.80325	-0.31608	4.03E-05	0.000551	0.25187	-1.9892	0.024741	0.13873	0.61107	-0.7106	0.1141	0.56725	0.7562	-0.40316	0.32481	0.95466	0.83498	-0.26019	0.59626	0.68916	0.91674	-0.12542
279.2324	1.83	lomics Pos	0.822	0.99987	1.102	0.1401	0.52217	0.99994	0.87773	-0.18815	0.083037	0.99996	0.61095	-0.71088	0.019265	0.25595	0.35742	-1.4843	0.000616	0.003839	0.27906	-1.8414	0.015479	0.10484	0.33345	-1.5844	0.01133	0.3324	0.34381	-1.5403	0.016293	0.52955	0.33624	-1.5724	0.03768	0.13071	0.4352	-1.2002
437.2913	5.29	olomics Po	0.82221	0.99951	0.92956	-0.10538	0.80003	0.99998	0.93316	-0.09981	0.59967	0.99941	0.8691	-0.2024	0.88179	0.99905	0.95867	-0.06089	0.56265	0.94415	1.166	0.22158	0.043752	0.17036	1.7071	0.77155	0.009517	0.24769	2.1664	1.1153	0.118	0.89567	1.4873	0.57265	0.055247	0.29791	1.5005	0.58542
615.2585	5.11	olomics Po	0.82236	0.99951	0.97763	-0.03264	0.66527	0.99998	0.9105	-0.13526	0.92599	0.99941	0.96648	-0.04918	0.93643	0.99905	1.0112	0.016138	0.33135	0.71099	1.3234	0.40429	0.033594	0.14225	1.4799	0.56554	0.008555	0.23862	1.7869	0.83744	0.54917	0.99956	1.1094	0.14973	0.33359	0.60988	1.1397	0.18859
288.2654	6.4	olomics Po	0.82259	0.99951	1.0458	0.06464	0.96811	0.99998	0.99929	-0.00102	0.54479	0.99941	0.94184	-0.08645	0.14795	0.5438	0.86065	-0.2165	4.22E-06	0.000231	0.26397	-1.9215	0.011969	0.083709	0.65621	-0.60776	0.12916	0.6766	0.77962	-0.35916	0.35323	0.99956	0.87797	-0.18775	0.19223	0.4564	0.85439	-0.22703
237.2213	6.27	olomics Po	0.82284	0.99951	0.90035	-0.15144	0.57788	0.99998	0.83133	-0.2665	0.088684	0.99941	0.47158	-1.0844	0.034984	0.25605	0.31456	-1.6686	0.016318	0.070946	0.26636	-1.9086	0.006225	0.065804	0.15531	-2.6868	0.018323	0.30136	0.23885	-2.0658	0.012987	0.47452	0.23569	-2.085	0.061364	0.30404	0.36862	-1.4398
164.0261	0.32	olomics Po	0.823	0.99951	0.93107	-0.10303	0.88441	0.99998	1.0213	0.030421	0.67076	0.99941	1.1451	0.19544	0.19566	0.62892	1.5182	0.60241	0.13668	0.36955	1.5614	0.64285	0.11231	0.3222	1.4481	0.53414	0.02803	0.33959	1.7993	0.84742	0.52982	0.99956	1.1649	0.22024	0.89418	0.95643	0.96491	-0.05153
127.87	12.57	olomics Ne	0.82301	0.99918	1.0128	0.018286	0.015301	0.37315	0.82297	-0.28108	0.82934	0.99995	0.98858	-0.01657	0.75606	0.99985	0.98176	-0.02656	0.41866	0.99927	0.94692	-0.07869	0.098845	0.55533	1.0803	0.11142	0.63664	0.87663	1.0171	0.024418	0.41446	0.99512	0.95031	-0.07353	0.26439	0.99941	0.92929	-0.1058
840.8463	0.5	lomics Pos	0.82326	0.99987	1.023	0.032805	0.2729	0.99994	0.93846	-0.09163	0.63488	0.99996	0.96587	-0.0501	0.008664	0.19971	0.84644	-0.24053	0.001363	0.006879	0.7095	-0.49512	0.085922	0.30647	0.82466	-0.27813	0.83098	0.96543	0.98844	-0.01677	0.20024	0.92582	0.92047	-0.11955	0.091305	0.19178	1.1413	0.19062
397.2163	6.83	olomics Po	0.82337	0.99951	0.97558	-0.03567	0.39075	0.99998	0.88198	-0.18118	0.030016	0.99941	0.70523	-0.50384	0.008844	0.19702	0.58028	-0.78517	0.00059	0.006301	0.53015	-0.91552	0.005389	0.062645	0.44525	-1.1673	0.008294	0.23862	0.50532	-0.98474	0.005753	0.47452	0.46966	-1.0903	0.022501	0.2688	0.54546	-0.87445
283.2646	6.96	olomics Po	0.8241	0.99951	0.97396	-0.03807	0.51762	0.99998	0.90084	-0.15065	0.018609	0.99941	0.66394	-0.59088	0.021304	0.22258	0.49474	-1.0153	0.002444	0.017614	0.45344	-1.141	0.001719	0.045425	0.33931	-1.5593	0.004018	0.20152	0.37416	-1.4183	0.021403	0.51556	0.42728	-1.2267	0.017691	0.2688	0.46315	-1.1104
501.2834	10.11	olomics Po	0.82417	0.99951	0.99726	-0.00396	0.88518	0.99998	1.0118	0.016977	0.088295	0.99941	0.77303	-0.37141	0.64668	0.99905	0.93535	-0.09642	0.78053	0.99688	1.0755	0.10498	0.024684	0.11675	1.5129	0.59733	0.009382	0.24769	1.5585	0.64011	0.48224	0.99956	1.1557	0.20873	0.28143	0.55651	1.1568	0.21011
253.2402	12.26	olomics Po	0.8244	0.99951	1.0152	0.021826	0.97451	0.99998	1.0012	0.001799	0.9353	0.99941	1.0163	0.023301	0.5465	0.99905	1.0378	0.053484	0.96112	0.9989	1.0065	0.009418	0.049103	0.18414	1.1333	0.18052	0.47226	0.99391	1.0352	0.049919	0.50983	0.99956	1.0558	0.078337	0.25486	0.52539	1.0832	0.11524
390.7319	2.85	olomics Po	0.82445	0.99951	1.0545	0.076565	0.17279	0.99998	0.80073	-0.32061	0.84883	0.99941	0.95806	-0.06181	0.015871	0.20428	0.52928	-0.9179	0.017454	0.075316	0.54256	-0.88214	0.046246	0.17633	0.62865	-0.66967	0.71755	0.99391	0.96221	-0.05558	0.59316	0.99956	0.9301	-0.10454	0.048268	0.28861	1.9316	0.9498
1082.698	0.54	lomics Pos	0.82596	0.99987	1.0291	0.041322	0.5891	0.99994	0.9451	-0.08146	0.80669	0.99996	0.97573	-0.03545	0.06425	0.43456	0.85117	-0.23249	0.005018	0.018292	0.71218	-0.48968	0.15158	0.43003	0.83501	-0.26013	0.255	0.69047	0.88774	-0.17179	0.019961	0.52955	0.76152	-0.39304	0.11628	0.2208	1.1672	0.22304
878.6941	6.37	lomics Pos	0.82598	0.99987	1.0608	0.085093	0.80224	0.99994	1.0402	0.056871	0.54785	0.99996	0.94686	-0.07877	0.25175	0.70895	0.87895	-0.18615	0.030225	0.071184	0.76774	-0.3813	0.10392	0.34315	0.81289	-0.29886	0.51389	0.83488	1.078	0.10832	0.74587	0.97988	0.97673	-0.03397	0.56174	0.65784	1.051	0.071727
203.1798	4.73	lomics Pos	0.82645	0.99987	1.028	0.039791	0.56177	0.99994	0.94838	-0.07647	0.92711	0.99996	1.0065	0.009405	0.4983	0.89009	0.94588	-0.08027	0.006858	0.022999	0.79104	-0.33817	0.98409	0.98952	0.99948	-0.00075	0.87951	0.97532	1.0142	0.020281	0.87249	0.98023	1.015	0.021454	0.12121	0.22555	1.2419	0.3126
521.5117	4.56	lomics Pos	0.82677	0.99987	0.97642	-0.03443	0.085855	0.99994	0.72785	-0.45828	0.94688	0.99996	1.0072	0.010379	0.94346	0.99212	0.99925	-0.00108	0.011032	0.033096	0.72599	-0.46198	0.30243	0.58211	0.82605	-0.2757	0.94966	0.98936	1.1232	0.16758	0.63653	0.97988	0.95222	-0.07063	0.34919	0.45928	0.87639	-0.19035
288.64	5.11	olomics Po	0.8269	0.99951	1.1508	0.20264	0.9948	0.99998	1.0501	0.070496	0.56572	0.99941	1.1166	0.15914	0.31588	0.81063	1.3149	0.39496	0.16841	0.43734	1.4535	0.5395	0.027036	0.12378	2.017	1.0122	0.024477	0.32533	1.9565	0.96829	0.31525	0.99956	1.3351	0.41692	0.23335	0.49936	1.3708	0.455
764.5839	5.62	lomics Pos	0.82692	0.99987	0.98841	-0.01682	0.011453	0.99994	0.84361	-0.24535	0.22055	0.99996	0.91429	-0.12927	0.007982	0.19917	0.84501	-0.24295	0.001313	0.006694	0.73544	-0.44333	0.059501	0.24162	0.84035	-0.25093	0.65785	0.89304	1.0616	0.0863	0.39423	0.96776	0.93446	-0.0978	0.083318	0.18503	1.1436	0.19354
669.5595	4.84	lomics Pos	0.82694	0.99987	0.96646	-0.04922	0.43609	0.99994	0.89813	-0.155	0.47898	0.99996	0.90096	-0.15046	0.74615	0.9735	0.95522	-0.0661	0.95937	0.96545	1.0002	0.000228	0.038234	0.17905	1.3143	0.39434	0.19245	0.64891	1.3015	0.38015	0.76354	0.97988	1.0851	0.11776	0.12702	0.23195	1.3205	0.40111
418.2163	2.18	lomics Pos	0.82695	0.99987	0.95026	-0.07361	0.70396	0.99994	0.91152	-0.13365	0.19761	0.99996	0.75963	-0.39662	0.37651	0.82708	0.82471	-0.27804	9.28E-05	0.000975	0.16569	-2.5934	0.012898	0.095682	0.53668	-0.89787	0.1999	0.64891	0.72498	-0.46399	0.15208	0.91321	0.67398	-0.56923	0.3276	0.43752	0.79241	-0.33569
1101.76	10.03	lomics Pos	0.82698	0.99987	1.0153	0.021876	0.8293	0.99994	0.97974	-0.02952	0.79221	0.99996	1.0177	0.025338	0.55631	0.90877	1.0485	0.068292	0.008663	0.027436	0.76322	-0.38983	0.76538	0.87911	0.97226	-0.04058	0.46218	0.80647	1.0869	0.12027	0.87251	0.98023	1.024	0.034191	0.089062	0.18993	1.205	0.26906
991.7887	10.03	lomics Pos	0.8273	0.99987	0.98582	-0.02061	0.1577	0.99994	0.88131	-0.18227	0.36115	0.99996	0.93336	-0.0995	0.24841	0.70866	0.88113	-0.18258	0.003024	0.012283	0.75962	-0.39666	0.027926	0.14769	0.85519	-0.22569	0.6615	0.89571	0.96265	-0.05492	0.631	0.97988	0.96411	-0.05273	0.1867	0.29429	1.1414	0.19085
142.9934	10.18	lomics Pos	0.82751	0.99987	1.0102	0.014601	0.36277	0.99994	0.94611	-0.07992	0.29277	0.99996	0.94049	-0.08851	0.29037	0.75244	0.9346	-0.09759	0.00044	0.002973	0.75319	-0.40891	0.28423	0.57131	0.90933	-0.13712	0.63182	0.8822	0.97281	-0.03977	0.4483	0.96776	0.9647	-0.05185	0.033751	0.12531	1.1877	0.2482
1045.517	6.28	lomics Pos	0.82779	0.99987	1.0338	0.048004	0.82921	0.99994	0.94627	-0.07967	0.79659	0.99996	0.95434	-0.06742	0.74443	0.97302	0.92254	-0.11631	0.88823	0.90532	1.0525	0.073832	0.21684	0.50811	1.4446	0.53072	0.01721	0.33809	1.944	0.959	0.072917	0.77255	1.5434	0.62615	0.035386	0.12781	2.143	1.0996
299.1396	1.91	lomics Pos	0.82808	0.99987	0.98012	-0.02898	0.49446	0.99994	1.1347	0.18237	0.2303	0.99996	1.2088	0.27355	0.66289	0.95717	1.0834	0.11563	0.043958	0.094642	0.6184	-0.69339	0.62407	0.79911	0.88534	-0.17569	0.51767	0.83809	0.85655	-0.22339	0.54437	0.96776	0.86739	-0.20525	0.44773	0.55233	1.1447	0.19501
608.4352	6.82	olomics Po	0.82812	0.99951	1.0485	0.068274	0.57175	0.99998	0.91977	-0.12065	0.056554	0.99941	0.7368	-0.44065	0.006508	0.1948	0.52144	-0.93942	0.000976	0.009119	0.52448	-0.93105	0.001629	0.045425	0.37029	-1.4333	0.001458	0.16989	0.43984	-1.1849	0.007668	0.47452	0.45769	-1.1275	0.063817	0.30458	0.55729	-0.8435
1161.838	4.96	lomics Pos	0.82816	0.99987	0.97925	-0.03025	0.20575	0.99994	0.85838	-0.22031	0.18013	0.99996	0.84167	-0.24867	0.67027	0.95717	0.95373	-0.06835	0.006562	0.02236	0.70759	-0.49901	0.096525	0.32835	0.83855	-0.25403	0.84795	0.96798	0.98286	-0.02494	0.3071	0.9524	0.89914	-0.15338	0.11781	0.22239	1.2045	0.26847
659.2421	1.11	lomics Pos	0.82832	0.99987	1.0479	0.067502	0.70135	0.99994	1.0603	0.084452	0.86929	0.99996	0.96267	-0.05488	0.56799	0.91302	0.88473	-0.1767	0.90321	0.91741	0.99781	-0.00317	0.1175	0.37089	1.2938	0.37166	0.042771	0.38464	1.4544	0.54045	0.17974	0.92305	1.2453	0.31646	0.06132	0.15841	1.4312	0.5172
872.5388	4.85	lomics Pos	0.82858	0.99987	1.0195	0.027889	0.22963	0.99994	0.90029	-0.15155	0.66298	0.99996	0.95621	-0.06461	0.87302	0.9874	0.98529	-0.02139	0.033252	0.07675	0.76706	-0.38258	0.7454	0.86962	0.97044	-0.04329	0.95124	0.98957	1.0125	0.017855	0.58155	0.97073	1.0523	0.073528	0.074921	0.17705	1.1587	0.21253

811.6621	5.71	domics Pos	0.82893	0.99987	1.022	0.031402	0.22925	0.99994	0.89288	-0.16346	0.32775	0.99996	0.91854	-0.12258	0.28389	0.74963	0.89595	-0.1585	0.011957	0.03513	0.75554	-0.40441	0.21509	0.50657	0.85818	-0.22065	0.65548	0.89228	0.95799	-0.06192	0.25061	0.94365	0.87185	-0.19785	0.27673	0.38543	1.1913	0.2525
556.8605	10.05	domics Pos	0.82901	0.99987	1.0096	0.013757	0.31391	0.99994	0.93663	-0.09444	0.2079	0.99996	0.93022	-0.10436	0.18375	0.62391	0.91538	-0.12755	0.001019	0.005564	0.7931	-0.33442	0.28337	0.57064	0.90556	-0.14312	0.96253	0.99112	0.9987	-0.00188	0.57381	0.9703	0.968	-0.04692	0.01667	0.099614	1.1791	0.23769
952.6826	6.28	domics Pos	0.8306	0.99987	0.89693	-0.15694	0.79943	0.99994	0.88324	-0.17912	0.75023	0.99996	0.89099	-0.16652	0.61709	0.93856	0.84552	-0.24208	0.87127	0.89137	1.0565	0.079344	0.12332	0.38281	1.513	0.59744	0.038452	0.37121	1.902	0.9275	0.14706	0.91175	1.4841	0.56955	0.027693	0.11669	2.1939	1.1335
353.8545	12.73	olomics Ne	0.83148	0.99918	1.0136	0.019489	0.043738	0.47152	0.91478	-0.1285	0.96442	0.99995	1.0026	0.003704	0.4215	0.99985	0.97121	-0.04215	0.79492	0.99927	0.98863	-0.0165	0.15001	0.6202	1.1234	0.16786	0.046053	0.38697	1.0897	0.12387	0.56427	0.99512	1.034	0.048235	0.80287	0.99941	1.0275	0.039163
1008.804	0.5	domics Pos	0.83187	0.99987	1.0096	0.013758	0.47096	0.99994	0.96276	-0.05475	0.15975	0.99996	0.93909	-0.09067	0.010794	0.21362	0.87332	-0.19542	0.000347	0.00247	0.7193	-0.47534	0.20219	0.49075	0.86254	-0.21333	0.21047	0.65208	0.89564	-0.15901	0.17092	0.91806	0.9031	-0.14705	0.092455	0.19265	1.1767	0.23479
217.1857	6.46	oolomics Pc	0.83239	0.99951	0.97577	-0.03539	0.97345	0.99998	1.0059	0.008458	0.075783	0.99941	0.69955	-0.5155	0.051369	0.29547	0.71117	-0.49173	0.002316	0.017247	0.44576	-1.1657	0.042758	0.16794	0.59503	-0.74897	0.012595	0.27021	0.54796	-0.86786	0.066374	0.74616	0.63809	-0.64817	0.027067	0.2688	0.54835	-0.86683
1058.481	5.91	domics Pos	0.83269	0.99987	0.98139	-0.0271	0.84325	0.99994	1.0298	0.042297	0.82236	0.99996	0.97997	-0.02919	0.56957	0.91327	0.94487	-0.08181	0.027961	0.067345	0.75482	-0.40579	0.23322	0.52064	0.8372	-0.25636	0.28848	0.71484	0.9083	-0.13875	0.048903	0.6775	0.78905	-0.34181	0.076107	0.17781	1.1997	0.26267
106.9513	10.11	domics Pos	0.83303	0.99987	0.98209	-0.02608	0.37015	0.99994	0.91798	-0.12347	0.63979	0.99996	0.96217	-0.05563	0.11447	0.53403	0.86978	-0.20128	0.003499	0.01377	0.7092	-0.49573	0.29929	0.58008	0.88045	-0.18368	0.82291	0.96103	0.98101	-0.02767	0.34057	0.96524	0.9349	-0.09711	0.11331	0.21744	1.1187	0.1618
257.2275	4.73	domics Pos	0.8331	0.99987	1.011	0.015831	0.07909	0.99994	0.88765	-0.17194	0.31299	0.99996	0.92238	-0.11657	0.51968	0.89938	0.94348	-0.08393	0.001486	0.007308	0.77187	-0.37357	0.2301	0.51793	0.93934	-0.09029	0.71952	0.92157	1.036	0.05096	0.8703	0.98023	0.9946	-0.00781	0.40099	0.50856	1.1493	0.20082
753.5618	5.66	domics Pos	0.83323	0.99987	1.0133	0.01907	0.19364	0.99994	0.91448	-0.12897	0.22562	0.99996	0.91281	-0.13161	0.063145	0.43296	0.8541	-0.22753	0.007983	0.025927	0.78679	-0.34595	0.24226	0.53058	1.1419	0.19142	0.017886	0.33809	1.3856	0.47056	0.03018	0.61539	1.1927	0.25418	0.00842	0.092495	1.3205	0.40105
280.8941	10.17	domics Pos	0.83368	0.99987	1.017	0.024294	0.26217	0.99994	0.88534	-0.17569	0.76026	0.99996	0.9627	-0.05484	0.44303	0.86677	0.91106	-0.13437	0.012105	0.035449	0.69591	-0.52303	0.53749	0.74246	0.92321	-0.11526	0.74194	0.93154	1.0265	0.037785	0.667	0.97988	1.0515	0.072411	0.2305	0.33769	1.16	0.21413
924.4978	4.61	domics Pos	0.83373	0.99987	1.0235	0.033515	0.80007	0.99994	1.0585	0.081974	0.92395	0.99996	1.0045	0.00641	0.58631	0.92519	1.1852	0.24508	0.79601	0.8259	1.0395	0.055824	0.061955	0.24919	1.5586	0.6403	0.034611	0.36356	1.6331	0.70763	0.26583	0.94365	1.53	0.61349	0.018156	0.10317	1.7316	0.79209
619.258	1.12	domics Pos	0.83395	0.99987	1.0741	0.1031	0.88936	0.99994	0.99765	-0.0034	0.8681	0.99996	0.93206	-0.10151	0.71213	0.96346	1.1369	0.18508	0.83687	0.8615	1.0227	0.032449	0.044091	0.19922	1.5403	0.62317	0.047123	0.39577	1.7441	0.80245	0.45733	0.96776	1.2542	0.32682	0.069713	0.17098	1.6251	0.70057
336.1597	6.05	oolomics Pc	0.83417	0.99951	0.98818	-0.01716	0.4383	0.99998	0.90516	-0.14375	0.012968	0.99941	0.76989	-0.37728	0.001777	0.14525	0.58449	-0.77476	0.00051	0.005801	0.56877	-0.81409	7.06E-05	0.034543	0.58153	-0.78207	0.01265	0.27021	0.72086	-0.47222	0.03252	0.58535	0.67348	-0.5703	0.048842	0.289	0.78648	-0.34652
841.8199	10.05	domics Pos	0.83438	0.99987	1.0083	0.011911	0.83453	0.99994	1.0153	0.02184	0.9854	0.99996	1.0114	0.016332	0.28549	0.7511	0.88762	-0.17198	0.028661	0.06843	0.7864	-0.34667	0.5417	0.74435	0.92072	-0.11917	0.49605	0.82381	1.0579	0.08122	0.94144	0.98646	0.98752	-0.01812	0.2442	0.3523	1.1295	0.17574
756.5573	9.06	oolomics Pc	0.83459	0.99951	1.0504	0.070932	0.79279	0.99998	1.0476	0.067032	0.77416	0.99941	0.97742	-0.03295	0.19063	0.62312	1.1221	0.16619	0.45715	0.85819	1.0896	0.12378	0.018883	0.10165	1.443	0.5291	0.03222	0.35567	1.453	0.53906	0.28627	0.99956	1.2111	0.27636	0.89374	0.95643	#N/A	#N/A
198.8649	9.91	domics Pos	0.83491	0.99987	0.98333	-0.02426	0.80815	0.99994	1.0204	0.029123	0.98867	0.99996	0.99796	-0.00294	0.24737	0.70693	0.8872	-0.17267	0.025648	0.062931	0.78757	-0.34453	0.64208	0.81337	0.95023	-0.07365	0.91736	0.98047	1.0237	0.033806	0.86128	0.98023	1.0258	0.036796	0.28594	0.39633	1.0871	0.12046
928.7414	10.45	oolomics Pc	0.83497	0.99951	1.1011	0.13888	0.80942	0.99998	1.009	0.012915	0.21617	0.99941	0.79398	-0.33282	0.5253	0.98929	0.93393	-0.09862	0.43317	0.83762	0.90306	-0.14711	0.10966	0.31867	0.78377	-0.3515	0.19255	0.77069	0.76146	-0.39315	0.036345	0.61553	0.53819	-0.8938	0.093234	0.34715	0.61557	-0.69999
217.989	10.17	olomics Ne	0.8351	0.99918	0.99241	-0.01099	0.15951	0.5837	0.88754	-0.17211	0.96017	0.99995	1.0062	0.008955	0.90036	0.99985	1.0108	0.015463	0.84534	0.99927	0.99242	-0.01097	0.10025	0.55533	1.0889	0.12281	0.079703	0.43379	1.1506	0.20244	0.02969	0.99512	0.93267	-0.10056	0.22849	0.99941	0.95292	-0.06957
380.2539	2.84	domics Pos	0.83512	0.99987	0.97857	-0.03126	0.26629	0.99994	0.83102	-0.26704	0.016515	0.99996	0.70511	-0.50408	0.004253	0.17581	0.52532	-0.92874	0.000381	0.002662	0.38279	-1.3854	0.004742	0.071661	0.38315	-1.384	0.006566	0.3088	0.45161	-1.1469	0.013635	0.52955	0.47507	-1.0738	0.039249	0.13267	0.60333	-0.72898
909.5513	12.14	olomics Ne	0.83574	0.99918	1.0336	0.047699	0.001517	0.34605	0.71994	-0.47405	0.51507	0.99995	1.0506	0.071154	0.79093	0.99985	1.0429	0.060557	0.034601	0.99927	0.89346	-0.16252	0.7188	0.94555	1.0394	0.055721	0.92034	0.9764	1.0061	0.008714	0.19935	0.99512	1.1638	0.21889	0.042735	0.99941	1.0869	0.12024
324.8077	9.91	domics Pos	0.83592	0.99987	1.0081	0.011694	0.064406	0.99994	0.75859	-0.39861	0.037618	0.99996	0.76589	-0.3848	0.016183	0.24	0.82912	-0.27034	0.00158	0.007627	0.65176	-0.61759	0.20533	0.49558	0.82737	-0.27339	0.272	0.70506	0.86977	-0.2013	0.11221	0.87836	0.84204	-0.24805	0.64856	0.73644	1.0765	0.10637
543.3253	5.11	oolomics Pc	0.83606	0.99951	0.95817	-0.06165	0.74368	0.99998	0.86281	-0.21289	0.90885	0.99941	0.957	-0.06341	0.58695	0.99905	1.212	0.27738	0.23658	0.56155	1.4183	0.50414	0.03094	0.13499	1.8476	0.88566	0.02858	0.3417	2.0261	1.0187	0.29837	0.99956	1.3424	0.4248	0.20226	0.46778	1.3543	0.43754
635.4439	9.57	oolomics Pc	0.83608	0.99951	1.0632	0.088427	0.15654	0.99998	0.7841	-0.3509	0.79518	0.99941	0.95518	-0.06616	0.034281	0.25408	0.70938	-0.49538	0.008479	0.042463	0.61059	-0.71173	0.051001	0.19035	0.65768	-0.60454	0.54788	0.99391	0.89811	-0.15504	0.88011	0.99956	0.97602	-0.03501	0.074252	0.31866	1.5623	0.64369
521.3438	5.11	oolomics Pc	0.83625	0.99951	0.94367	-0.08364	0.98358	0.99998	0.97341	-0.03888	0.88869	0.99941	0.99325	-0.00976	0.52182	0.98783	1.2247	0.2924	0.20059	0.4965	1.5124	0.59679	0.020222	0.10564	2.1741	1.1204	0.014813	0.28681	2.3593	1.2383	0.27365	0.99956	1.422	0.50789	0.17776	0.44124	1.4412	0.52724
279.2274	2.27	lomics Neg	0.83652	0.99977	1.0268	0.038132	0.58326	0.99904	0.98747	-0.01819	0.045518	0.99994	0.5931	-0.75366	0.061569	0.58182	0.51278	-0.96358	0.000139	0.032757	0.38831	-1.3647	0.030949	0.63166	0.29788	-1.7472	0.00298	0.093659	0.25505	-1.9712	0.015165	0.46971	0.20817	-2.2642	0.012197	0.38536	0.28336	-1.8193
523.3458	1.11	domics Pos	0.83672	0.99987	1.0696	0.097016	0.88494	0.99994	1.0068	0.009763	0.62279	0.99996	1.0811	0.1125	0.51532	0.89797	1.2117	0.27698	0.33308	0.42704	1.3835	0.46829	0.036586	0.17398	2.2476	1.1684	0.014588	0.33809	2.8694	1.5207	0.22832	0.94286	1.5423	0.6251	0.10378	0.20665	1.8137	0.85897
287.2368	2.18	domics Pos	0.83693	0.99987	1.0103	0.014787	0.89846	0.99994	0.99533	-0.00675	0.17712	0.99996	0.75137	-0.41241	0.47068	0.88476	0.8591	-0.21911	0.000178	0.00155	0.206	-2.2793	0.033753	0.165	0.59136	-0.75789	0.44518	0.80121	0.83803	-0.25492	0.36888	0.96776	0.81047	-0.30317	0.5746	0.66899	0.88131	-0.18227
790.5709	5.17	domics Pos	0.83719	0.99987	1.0213	0.03034	0.79469	0.99994	0.97433	-0.03752	0.86426	0.99996	0.98298	-0.02477	0.43704	0.86659	0.9215	-0.11794	0.023865	0.059548	0.7693	-0.37839	0.47793	0.69927	0.90655	-0.14155	0.83332	0.96621	1.0397	0.056184	0.97375	0.99223	0.99826	-0.00252	0.26032	0.36983	1.1205	0.16409
523.3591	1.37	domics Pos	0.83777	0.99987	1.0457	0.064486	0.93727	0.99994	1.0002	0.000265	0.91577	0.99996	0.99943	-0.00082	0.92368	0.9874	0.9868	-0.01917	0.82015	0.84794	0.90611	-0.14224	0.13339	0.40018	1.4939	0.57912	0.045982	0.39255	1.8185	0.86277	0.43867	0.96776	1.2049	0.26892	0.070421	0.17145	2.1338	1.0934
813.6836	6.04	domics Pos	0.83835	0.99987	0.98555	-0.021	0.11375	0.99994	0.83231	-0.26481	0.2959	0.99996	0.89579	-0.15876	0.10027	0.52019	0.84559	-0.24197	0.01286	0.037064	0.74496	-0.42476	0.15181	0.43005	0.81055	-0.30303	0.39804	0.77223	0.9353	-0.0965	0.12621	0.88537	0.83292	-0.26375	0.80294	0.85376	0.98159	-0.02681
476.2761	1.15	lomics Neg	0.83848	0.99977	0.95565	-0.06545	0.76703	0.99904	0.9327	-0.10051	0.89873	0.99994	1.0713	0.099388	0.28414	0.58182	1.7572	0.81331	0.072424	0.99989	2.4398	1.2867	0.006769	0.46473	3.2452	1.6983	0.01521	0.28138	3.2362	1.6943	0.83936	0.92082	1.7221	0.78417	0.1121	0.405	2.1098	1.0771
542.3221	5.11	oolomics Pc	0.83853	0.99951	0.96107	-0.05729	0.75224	0.99998	0.87538	-0.19203	0.98568	0.99941	0.94625	-0.0797	0.60405	0.99905	1.1917	0.25306	0.21929	0.53053	1.4384	0.52443	0.031618	0.13695	1.818	0.86239	0.029501	0.34805	1.9511	0.96426	0.30554	0.99956	1.333	0.41472	0.20212	0.46778	1.3506	0.4336
452.3134	1.84	domics Pos	0.83857	0.99987	1.0983	0.13526	0.72174	0.99994	0.95161	-0.07156	0.84192	0.99996	0.9705	-0.04321	0.98119	0.99652	0.99643	-0.00517	0.000759	0.00452	0.36961	-1.4359	0.3716	0.63001	0.87242	-0.1969	0.61874	0.87251	1.0404	0.057176	0.8265	0.97988	1.077	0.10708	0.063398	0.16148	1.517	0.60118
579.4317	2.71	domics Pos	0.83857	0.99987	0.98652	-0.01958	0.81071	0.99994	0.99391	-0.00882	0.56709	0.99996	0.92672	-0.10979	0.10501	0.52894	0.75917	-0.39751	0.000672	0.004073	0.57519	-0.7979	0.016443	0.10923	0.56384	-0.82664	0.16856	0.64016	0.76706	-0.38259	0.008476	0.52955	0.67231	-0.5728	0.17044	0.27809	0.81518	-0.29482
866.5417	5.81	domics Pos	0.83864	0.99987	0.96794	-0.04702	0.14015	0.99994	0.83291	-0.26376	0.50953	0.99996	0.91945	-0.12115	0.36697	0.82143	0.88037	-0.18382	0.015716	0.04336	0.68419	-0.54754	0.33656	0.60864	0.86391	-0.21105	0.45479	0.80208	1.1078	0.14768	0.17849	0.92219	1.1731	0.23033	0.72907	0.79815	0.95269	-0.06992
165.1261	2.28	domics Pos	0.83877	0.99987	0.98321	-0.02443	0.3927	0.99994	0.83885	-0.25351	0.035745	0.99996	0.58557	-0.77208	0.012996	0.22826	0.49026	-1.0284	0.000159	0.001423	0.32613	-1.6165	0.007363	0.082061	0.37129	-1.4294	0.009311	0.3318	0.41902	-1.2549	0.003765	0.52955	0.35217	-1.5057	0.048731	0.14531	0.50359	-0.98969
547.359	1.22	domics Pos	0.83879	0.99987	1.1271	0.1726	0.79563	0.99994	1.0759	0.10558	0.58554	0.99996	1.1611	0.2155	0.69301	0.96092	1.0835	0.1157	0.86996	0.89028	0.94637	-0.07952	0.063028	0.25139	1.6145	0.6911	0.024368	0.33809	1.9418	0.95743	0.51682	0.96776	1.1175	0.16031	0.14803	0.25308	1.6023	0.68018
1037.605	5.74	domics Pos	0.83892	0.99987	0.96146	-0.0567	0.43659	0.99994	0.85656	-0.22337	0.39183	0.99996	0.85941	-0.21859	0.57049	0.91398	0.90625	-0.14202	0.04266	0.092527	0.66068	-0.59798	0.10626	0.3484	0.71381	-0.48638	0.26661	0.70012	0.8208	-0.2849	0.19667	0.92582	0.76053	-0.39492	0.20784	0.31649	0.77287	-0.3717
748.5984	5.53	domics Pos	0.83901	0.99987	0.98312	-0.02456	0.29481	0.99994	0.87263	-0.19656	0.38124	0.99996	0.9132	-0.13099	0.10776	0.53121	0.82927	-0.27008	0.012524	0.036354	0.74669	-0.42141	0.074902	0.28246	0.84098	-0.24986	0.66874	0.89786	0.95846	-0.06121	0.25105	0.94365	0.87987	-0.18463	0.63657	0.72694	0.95393	-0.06804
624.8478	0.54	domics Pos	0.84063	0.99987	1.0111	0.01592	0.12261	0.99994	0.85176	-0.23148	0.27245	0.99996	0.88136	-0.1822	0.16449	0.596	0.89566	-0.15898	0.003401	0.013495	0.70821	-0.49774	0.25009	0.53676	0.85078	-0.23314	0.44467	0.80121	0.91888	-0.12206	0.32498	0.95466	0.91882	-0.12215	0.18117	0.28844	1.1867	0.24693
636.8807	0.5	domics Pos	0.84142	0.99987	1.015	0.021536	0.34049	0.99994	0.92222	-0.11682	0.33028	0.99996	0.93031	-0.10421	0.16956	0.60365	0.89967	-0.15254	0.008621	0.027349	0.69884	-0.51697	0.37551	0.63269	0.90415	-0.14537	0.5264	0.83957	0.94766	-0.07756	0.94489	0.98763	0.99129	-0.01262	0.349	0.45926	1.1336	0.18097
557.3847	3.66	domics Pos	0.84168	0.99987	1.0821	0.11389	0.831	0.99994	1.0715	0.099668	0.95148	0.99996	1.0211	0.030082	0.37381	0.82629	1.2166	0.28282	0.36432	0.45749	1.2919	0.36952	0.022165	0.13042	1.7851	0.83598	0.03928	0.37421	1.6774	0.7462	0.40503	0.96776	1.354	0.4372	0.39101	0.49964	1.3726	0.45686
840.842	10.02	domics Pos	0.84208	0.99987	0.98742	-0.01827	0.59334	0.99994	1.0481	0.067823	0.53925	0.99996	0.9475	-0.07781	0.020276	0.25894	0.85105	-0.23269	0.001493	0.007309	0.71054	-0.49302	0.15287	0.43045	0.91359	-0.13038	0.19926	0.64891	0.92137	-0.11814	0.87263	0.98023	1.008	0.011552	0.044715	0.14056	1.2425	0.31327
854.5203	6.48	oolomics Pc	0.84208	0.99951	1.0785	0.10907	0.17179	0.99998	0.78753	-0.3446	0.79344	0.99941	0.97375	-0.03838	0.031803	0.24911	0.73658	-0.44109	0.24224	0.57115	0.85184	-0.23134	0.045151	0.17328	0.74217	-0.43019	0.58316	0.99391	0.92699	-0.10938	0.58418	0.99956	0.93422	-0.09817	0.91542	0.96475	1.0628	0.087829
117.9336	12.69	olomics Ne	0.8422	0.99918	0.9937	-0.00912	0.015964	0.37315	0.90982	-0.13635	0.71403	0.99995	1.0156	0.022282	0.54017	0.99985	0.97405	-0.03793	0.72152	0.99927	0.98498	-0.02184	0.38561	0.78301	1.0682	0.095202	0.01868	0.29108	1.0882	0.12193	0.96372	0.99512	1.0072	0.010408	0.81929	0.99941	0.99419	-0.00841
551.6323	10.06	domics Pos	0.84236	0.99987	0.98416	-0.02303	0.13724	0.99994	0.8917	-0.16537	0.11083	0.99996	0.88684	-0.17326	0.47983	0.88482	0.92858	-0.10691	0.030036	0.070915	0.79909	-0.32358	0.60235	0.78414	0.95066	-0.07301	0.7011	0.91363	1.0377	0.053333	0.78632	0.97988	1.0277	0.039461	0.041834	0.13621	1.1838	0.2434
430.2737	2.72	domics Pos	0.84248	0.99987	0.96511	-0.05124	0.19052	0.99994	0.78037	-0.35776	0.037404	0.99996	0.71771	-0.47852	0.006201	0.18618	0.53227	-0.90976	0.000894	0.005136	0.43598	-1.1977	0.008874	0.08583	0.39598	-1.3365	0.036827	0.36759	0.49275	-1.0211	0.024305	0.55495	0.47911	-1.0616	0.079524	0.18125	0.6274	-0.67254
612.3041	5.14	olomics Ne	0.84274	0.99918	1.0595	0.083316	0.96051	0.99363	0.9904	-0.01392	0.402	0.99995	1.1311	0.17771	0.70466	0.99985	1.0674	0.094038	0.79528	0.99927	1.0671	0.093677	0.058888	0.50693	1.3313	0.41285	0.008287	0.2539	1.5123	0.59679	0.26624	0.99512	1.1308	0.17736	0.19105	0.99941	1.1688	0.22508
329.2448	2.27	lomics Neg	0.8431	0.99977	0.98564	-0.02087	0.40346	0.99904	0.82213	-0.28257	0.010775	0.99994	0.53165	-0.91144	0.02027	0.58182	0.45305	-1.1423	0.002031	0.066804	0.34322	-1.5428	0.010515	0.46473	0.23247	-2.1049	0.001287	0.093659	0.22569	-2.1476	0.009693	0.46971	0.20083	-2.3159	0.004131	0.38536	0.2706	-1.8858
150.0144	0.59	domics Pos	0.84322	0.99987	1.0197	0.028207	0.46005	0.99994	0.9021	-0.14865	0.89218	0.99996	1.0408	0.05769	0.68372	0.96001	1.0778	0.10809	0.20444	0.29826	0.82709	-0.27388	0.85396	0.92636	0.97315	-0.03926	0.032062	0.36169	1.3785	0.46315	0.65337	0.97988	0.94157	-0.08686	0.74342	0.80852	1.0915	0.12626
815.6893	6.04	domics Pos	0.84356	0.99987	0.98229	-0.02578	0.17728	0.99994	0.84342	-0.24567	0.40767	0.99996	0.91421	-0.12941	0.16866	0.6035	0.85756	-0.22169	0.02974	0.0704	0.7783	-0.3616	0.17935	0.46725	0.82595	-0.27587	0.46453	0.80647	0.93767	-0.09285	0.14413	0.90387	0.83547	-0.25934	0.9521	0.96552	0.99548	-0.00654
438.2985	1.54	domics Pos	0.84374	0.99987	0.96962	-0.04451	0.9005	0.99994	0.98044	-0.0285	0.50545	0.99996	0.87433	-0.19375	0.73699	0.97097	0.93488	-0.09715	0.000214	0.001755	0.20234	-2.3051	0.19697	0.48517	0.74607	-0.42262	0.37887	0.76056	1.2312	0.30006	0.82858	0.97988	1.071	0.098983	0.092024	0.19236	1.4528	0.53888
181.9899	12.01	olomics Ne	0.84377	0.99918	1.0342	0.048552	0.35771	0.76581	0.86625	-0.20715	0.28252	0.99995	0.83956	-0.2523	0.17861	0.99985	0.8676	-0.2049	0.033704	0.99927	0.74113	-0.4322	0.12919	0.60012	1.3768	0.46134	0.19639	0.5757	0.87325	-0.19553	0.567	0.99512	1.0654	0.091377	0.51179	0.99941	1.124	0.16862
548.2922	5.09	oolomics Pc	0.84388	0.99951	0.95403	-0.06789	0.91104	0.99998	0.92636	-0.11036	0.98071	0.99941	0.96396	-0.05295	0.66942	0.99905	1.1482	0.19941	0.13102	0.35826	1.4532	0.53923	0.021013	0.10792	1.7976	0.8461	0.015656	0.29145	2.1414	1.0986	0.23905	0.99956	1.3921	0.47722	0.16707	0.42787	1.3641	0.44791
502.2927	10.09	oolomics Pc	0.84389	0.99951	0.97997	-0.02919	0.52188	0.99998	0.93609	-0.09529	0.14778	0.99941	0.86729	-0.20541	0.55591	0.99905	0.93084	-0.1034	0.89532	0.99688	1.0087	0.012514	0.025619	0.11991	1.2646	0.33868	0.028565	0.3417	1.4187	0.50459	0.15754	0.93927	1.1412	0.19057	0.24014	0.50768	1.1024	0.1406
230.986	3.31	olomics Ne	0.84434	0.99918	1.0072	0.010413	0.11087	0.52291	0.92767	-0.10832	0.79378	0.99995	1.0107	0.015363	0.52496	0.99985	1.0272	0.038672	0.74471	0.99927	0.98405	-0.02319	0.048536	0.48405	1.0973	0.13394	0.25825	0.63824	1.055	0.077222	0.62691	0.99512	1.0264	0.037573	0.7887	0.99941	1.0112	0.016074
620.2603	1.12	domics Pos	0.84457	0.99987	1.0667	0.093191	0.96317	0.99994	1.0065	0.009283	0.79036	0.99996	1.05	0.070435	0.94957	0.99328	1.0081	0.011661	0.99479	0.9961	0.99572	-0.0062	0.19806	0.4857	1.2401	0.31046	0.001835	0.23318	1.6826	0.75068	0.24572	0.94365	1.1996	0.2626	0.044588	0.14047	1.4484	0.53451
958.7545	0.56	domics Pos	0.84475	0.99987	1.0411	0.058115	0.14657	0.99994	0.85068	-0.23331	0.31828	0.99996	0.90561	-0.14304	0.28178	0.74614	0.89621	-0.15809	0.020173	0.052612	0.8482	-0.23752	0.068868	0.2669	0.8545	-0.22685	0.31079	0.72948	1.0757	0.10524	0.76955	0.97988	0.9784	-0.03151	0.054477	0.15191	1.1618	0.21631
741.526	5	domics Pos	0.84479	0.99987	1.0211	0.030107	0.27619	0.99994	0.84884	-0.23644	0.21253	0.99996	0.84162	-0.24875	0.14294	0.57187	0.80571	-0.31166	0.029344	0.069835	0.62953	-0.66766	0.30117	0.58086	0.84606	-0.24118	0.38032	0.76056	1.0922	0.12729	0.052485	0.68583	1.3965	0.48177	0.007841	0.092077	1.7141	0.77748
178.0559	0.61	domics Pos	0.84502	0.99987	1.0187	0.026751	0.14043	0.99994	0.83654	-0.25749	0.50093	0.99996	1.0885	0.1224	0.093506	0.50934	1.2498	0.32166	0.63547	0.69013	0.94987	-0.07419	0.13162	0.39673	1.1764	0.2344	0.004684	0.3088	1.4187	0.50461	0.36198	0.96776	1.1139	0.15559	0.11448	0.21881	1.2179	0.28442
1167.514	6.98	oolomics Pc	0.84524	0.99951	1.1118	0.15292	0.70466	0.99998	0.98291	-0.02487	0.85468	0.99941	1.1171	0.15976	0.81377	0.99905	1.0493	0.069477	0.001961	0.015227	0.51884	-0.94664	0.020856	0.10792	0.49944	-1.0016	0.010171	0.24769	0.56524	-0.82306	0.074475	0.7717	0.63732	-0.6499	0.13583	0.39003	0.72863	-0.45674
408.9181	10.14	domics Pos	0.84539	0.99987	0.99041	-0.0139	0.15732	0.99994	0.92427	-0.11362	0.017824	0.99996	0.87491	-0.1928	0.042054	0.357	0.84704	-0.2395	0.000116	0.001136	0.73107	-0.45191	0.034248	0.16635	0.80004	-0.32186	0.51721	0.83777	0.97066	-0.04297	0.18297	0.92582	0.90506	-0.14392	0.54335	0.63944	1.0923	0.12733
360.2198	2.28	domics Pos	0.84589	0.99987	0.98279	-0.02505	0.21763	0.99994	0.79799	-0.32555	0.06941	0.99996	0.74975	-0.41552	0.005215	0.17581	0.58249	-0.7797	0.000426	0.002899	0.42083	-1.2487	0.010166	0.08787	0.42637	-1.2298	0.028064	0.34956	0.50449	-0.98711	0.016921	0.52955	0.44288	-1.175	0.027881	0.11681	0.54751	-0.86905
332.1442	1.83	domics Pos	0.84595	0.99987	0.96957	-0.04458	0.33848	0.99994	0.77899	-0.36033	0.033029	0.99996	0.53963	-0.88997	0.005926	0.18075	0.3269	-1.6131	0.001105	0.005924	0.30656	-1.7057	0.011041	0.091128	0.30505	-1.7129	0.006346	0.3088	0.28983	-1.7867	0.011698	0.52955	0.29045	-1.7836	0.032079	0.12241	0.38661	-1.371
179.1447	6.48	oolomics Pc	0.84605	0.99951	0.99164	-0.01211	0.65538	0.99998	0.95696	-0.06347	0.085514	0.99941	0.71242	-0.48921	0.044458	0.28464	0.5942	-0.75099	0.003832	0.024397	0.49738	-1.0076	0.015409	0.092755	0.487	-1.038	0.018268	0.30136	0.45629	-1.132	0.010484	0.47452	0.45389	-1.1396	0.030073	0.2688	0.48162	-1.054
776.7938	0.54	domics Pos	0.84646	0.99987	1.0501	0.070519	0.63448	0.99994	0.95397	-0.06799	0.4447	0.99996	1.0735	0.10231	0.39779	0.84265	0.91806	-0.12334	0.013308	0.038094	0.73866	-0.43703	0.76544	0.87911	1.0694	0.096736	0.3801	0.76056	1.099	0.13613	0.955	0.98838	1.0003	0.000371	0.074332	0.17626	1.1778	0.23607
416.3168	4.75	oolomics Pc	0.84657	0.99951	0.97631	-0.03459	0.88719	0.99998	1.1312	0.17782	0.45	0.99941	2.035	1.0251	0.054538	0.30353	4.9128	2.2966	0.00804	0.041049	4.87	2.2839	0.10776	0.31665	1.9682	0.97691	0.017025	0.29966	3.7779	1.9176	0.41646	0.99956	1.2357	0.30528	0.66803	0.84953	1.0664	0.092764
820.5671	10.15	olomics Ne	0.84675	0.99918	0.98782	-0.01768	0.54652	0.87145	1.129	0.17504	0.35997	0.99995	1.1723	0.22939	0.355	0.99985	1.0679	0.09484	0.087145	0.99927	1.2017	0.26505	0.065102	0.51001	1.2699	0.34474	0.009123	0.25531	1.6506	0.72301	0.03364	0.99512	1.1668	0.22255	0.055295	0.99941	1.2234	0.29087
421.8996	0.56	domics Pos	0.84696	0.99987	1.011	0.015783	0.47385	0.99994	0.9276	-0.10843	0.64233	0.99996	0.94709	-0.07842	0.43471	0.86496	0.92007	-0.12018	0.010029	0.03074	0.71529	-0.48341	0.27052	0.55756	0.85169	-0.2316	0.93388	0.98559	0.98716	-0.01865	0.29298	0.9483	0.86403	-0.21085	0.087393	0.18803	1.2426	0.31331
522.9756	12.01	olomics Ne	0.84728	0.99918	1.0494	0.069547	0.031365	0.43383	0.83922	-0.25288	0.96422	0.99995	1.0156	0.022322	0.97386	0.99985	1.0087	0.012494	0.94342	0.99927	1.0085	0.012271	0.74621	0.94707	1.073	0.10159	0.16444	0.54112	1.1048	0.14382	0.82859	0.99512	0.98484	-0.02205	0.48305	0.99941	0.93838	-0.09176
866.7231	0.56	domics Pos	0.8477	0.99987	0.99346	-0.00947	0.26248	0.99994	0.90277	-0.14758	0.063823	0.99996	0.9056	-0.14306	0.11249	0.53238	0.7437	-0.42721	0.004045	0.015432	0.64889	-0.62396	0.0197	0.12057	0.78882	-0.34222	0.057817	0.42807	0.81906	-0.28795	0.40083	0.96776	0.95144	-0.07181	0.2578	0.3672	1.0777	0.10789
439.2751	3.52	domics Pos	0.84792	0.99987	0.96605	-0.04984	0.2169	0.99994	0.80983	-0.30432	0.049981	0.99996	0.73001	-0.454	0.005904	0.18075	0.54284	-0.88139	0.000842	0.004885	0.42161	-1.246	0.00984	0.087714	0.38509	-1.3767	0.014418	0.33809	0.5337	-0.90589	0.005953	0.52955	0.48733	-1.037	0.105	0.20712	0.64301	-0.63708
1042.8	0.5	domics Pos	0.84806	0.99987	0.99229	-0.01117	0.25807	0.99994	0.94419	-0.08285	0.10093	0.99996	0.9252	-0.11217	0.015941	0.24	0.86369	-0.21142	0.000206	0.001726	0.73988	-0.43464	0.19159	0.4781	0.86257	-0.21329	0.35897	0.75581	0.9318	-0.10191	0.14293	0.90379	0.93295	-0.10013	0.036188	0.1292	1.1747	0.23225
773.663	7.5	domics Pos	0.84808	0.99987	1.0181	0.025809	0.64792	0.99994	0.92872	-0.10669	0.55851	0.99996	0.9062	-0.1421	0.52004	0.89938	0.90659	-0.14148	0.9992	0.99935	1.0904	0.12489	0.072194	0.27658	1.5601	0.64165	0.018486	0.33809	1.4498	0.53583	0.13913	0.89982	1.927	0.94635	0.074202	0.17622	1.8476	0.88566
711.2526	5.37	olomics Ne	0.84844	0.99918	1.0474	0.066846	0.92037	0.9934	1.0054	0.007807	0.35152	0.99995	1.1892	0.24998	0.46261	0.99985	1.1058	0.14505	0.71391	0.99927	1.0678	0.094656	0.022525	0.43126	1.4471	0.53312	0.047935	0.39078	1.381	0.46569	0.32428	0.99512	1.2072	0.27168	0.099426	0.99941	1.3266	0.40774
368.34	8.19	oolomics Pc	0.84906	0.99951	1.0084	0.012132	0.79751	0.99998	1.043	0.060672	0.85939	0.99941	0.96552	-0.05063	0.8007	0.99905	0.9624	-0.0553	0.6595	0.99209	1.0461	0.065014	0.1665	0.42365	1.2879	0.36508	0.019591	0.30136	1.4575	0.54353	0.65612	0.99956	0.93081	-0.10344	0.66518	0.84814	1.05	0.070416
520.3405	5.11	oolomics Pc	0.84936	0.99951	0.94451	-0.08236	0.98759	0.99998	0.96552	-0.05062	0.88694	0.99941	0.98925	-0.01559	0.52941	0.99205	1.2294	0.29801	0.20068	0.4965	1.5118	0.5963	0.01965	0.10351	2.2069	1.142	0.015176	0.28798	2.4086	1.2682	0.27326	0.99956	1.4364	0.52244	0.18144	0.44585	1.4462	0.53231
392.8936	10.16	domics Pos	0.84981	0.99987	0.98823	-0.01708	0.036716	0.99994	0.88138	-0.18216	0.21611	0.99996	0.91199	-0.13291	0.047105	0.3738	0.77663	-0.3647	0.002254	0.009879	0.70097	-0.51257	0.003617	0.067492	0.74556	-0.42361	0.32765	0.73621	0.91544	-0.12747	0.22178	0.94047	0.90354	-0.14634	0.87543	0.90954	0.99401	-0.00867
716.5233	8.85	oolomics Pc	0.84998	0.99951	0.94305	-0.08459	0.66239	0.99998	0.88218	-0.18086	0.55572	0.99941	0.8276	-0.273	0.71478	0.99905	0.91067	-0.135	0.58698	0.9614	1.1361	0.18414	0.11529	0.32925	1.6747	0.7439	0.037084	0.38426	2.0526	1.0374	0.16083	0.94166	1.7255	0.78697	0.053621	0.2951	1.893	0.92066
743.4544	6.51	oolomics Pc	0.85026	0.99951	1.1194	0.16279	0.21277	0.99998	0.7955	-0.33007	0.86214	0.99941	1.0273	0.038822	0.043141	0.2816	0.75496	-0.40553	0.87667	0.99688	0.98215	-0.02598	0.11604	0.3305	0.81109	-0.30206	0.81115	0.99391	1.1064	0.14583	0.92822	0.99956	1.0419	0.059258	0.52284	0.76572	1.1127	0.15412
360.2195	6.48	oolomics Pc	0.85073	0.99951	1.033	0.046894	0.80438	0.99998	1.0908	0.12539	0.17447	0.99941	0.74285	-0.42885	0.15762	0.55851	0.73114	-0.45177	0.98775	0.9989	0.98932	-0.01549	0.074541	0.24426	0.65059	-0.62018	0.086342	0.60175	0.62882	-0.66928	0.03578	0.61253	0.51971	-0.94422	0.019082	0.2688	0.46734	-1.0975
919.5484	4.75	lomics Neg	0.85081	0.99977	0.98556	-0.02099	0.58841	0.99904	0.92461	-0.11308	0.38924	0.99994	0.89165	-0.16545	0.17806	0.58182	1.2668	0.34121	0.8101	0.99989	1.0216	0.030799	0.87836	0.99653	1.0317	0.044976	0.08063	0.41257	0.67118	-0.57524	0.028116	0.46971	0.59627	-0.74597	0.19721	0.42853	0.85209	-0.23093
321.2127	2.85	domics Pos	0.85102	0.99987	0.9723	-0.04053	0.71195	0.99994	0.95279	-0.06977	0.042572	0.99996	0.72057	-0.47279	0.017961	0.25028	0.51669	-0.95264	0.002001	0.009061	0.37453	-1.4169	0.014624	0.10138	0.37794	-1.4038	0.018538	0.33809	0.44309	-1.1743	0.052551	0.68583	0.4941	-1.0171	0.08544	0.18633	0.63946	-0.64507
546.7994	5.11	oolomics Pc	0.85125	0.99951	0.85678	-0.223	0.76092	0.99998	0.78394	-0.35118	0.85334	0.99941	0.81954	-0.28711	0.67919	0.99905	1.1131	0.15452	0.17916	0.45956	1.699	0.76472	0.036375	0.15075	2.3539	1.235	0.036961	0.38426	2.5177	1.3321	0.3822	0.99956	1.5505	0.63269	0.16968	0.43104	1.5315	0.61497
459.3038	3.52	domics Pos	0.85137	0.99987	0.95165	-0.07149	0.22017	0.99994	0.77181	-0.37368	0.049736	0.99996	0.68776	-0.54003	0.003274	0.17581	0.50645	-0.98151	0.003492	0.013753	0.44449	-1.1698	0.01964	0.1204	0.45733	-1.1287	0.01259	0.3324	0.54641	-0.87195	0.019859	0.52955	0.51914	-0.9458	0.19144	0.29966	0.69613	-0.52257
190.0994	10.16	domics Pos	0.85204	0.99987	1.0135	0.019406	0.13854	0.99994	0.90787	-0.13945	0.17409	0.99996	0.94421	-0.08282	0.22497	0.68033	0.94595	-0.08017	8.31E-05	0.000908	0.70631	-0.50163	0.2946	0.57665	0.90376	-0.14599	0.71262	0.91798	1.0396	0.056017	0.726	0.97988	0.98281	-0.02501	0.022293	0.10856	1.2123	0.27773
1107.613	6.06	domics Pos	0.85218	0.99987	0.97332	-0.03901	0.17681	0.99994	0.79201	-0.33641	0.3137	0.99996	0.8758	-0.19133	0.33219	0.79293	0.86307	-0.21245	0.042469	0.092218	0.71204	-0.48997	0.39393	0.64355	0.85826	-0.22051	0.79867	0.95226	1.0269	0.038281	0.19379	0.92582	0.82862	-0.27121	0.28706	0.39751	0.87567	-0.19154
787.8306	10.06	domics Pos	0.85225	0.99987	0.98641	-0.01974	0.31542	0.99994	0.92715	-0.10912	0.95691	0.99996	1.003	0.00436	0.2499	0.70866	0.90546	-0.14327	0.004652	0.017264	0.74366	-0.42728	0.40122	0.64773	0.89657	-0.15751	0.74071	0.93154	0.97317	-0.03923	0.82147	0.97988	0.98341	-0.02414	0.17798	0.28538	1.1452	0.19566
676.8628	10.02	domics Pos	0.85249	0.99987	0.98226	-0.02582	0.18311	0.99994	0.87604	-0.19094	0.22536	0.99996	0.91274	-0.13173	0.26494	0.7252	0.90463	-0.1446	0.017448	0.046885	0.74037	-0.43368	0.28844	0.57347	0.87248	-0.1968	0.94922	0.98936	0.99581	-0.00605	0.42943	0.96776	0.93392	-0.09864	0.096934	0.19787	1.1552	0.20814
477.2803	10.16	olomics Ne	0.85261	0.99918	1.0297	0.042207	0.77663	0.96992	0.91473	-0.12859	0.95987	0.99995	0.99152	-0.01229	0.84277	0.99985	0.95734	-0.06289	0.81426	0.99927	1.0562	0.078909	0.15911	0.62813	1.4888	0.57416	0.046445	0.38697	1.6396	0.71334	0.2352	0.99512	1.3117	0.39138	0.31903	0.99941	1.2149	0.28079
479.9807	12.01	olomics Ne	0.85284	0.99918	0.9849	-0.02195	0.53625	0.86583	0.92702	-0.10933	0.46396	0.99995	1.0719	0.10021	0.025535	0.99985	0.81181	-0.30079	0.19927	0.99927	0.90356	-0.14631	0.67955	0.93064	1.0665	0.092894	0.53798	0.84256	1.1228	0.16713	0.82177	0.99512	1.0619	0.086605	0.23067	0.99941	1.2086	0.27335
334.2808	6.92	oolomics Pc	0.85288	0.99951	1.0874	0.12094	0.58159	0.99998	0.90633	-0.14189	0.098792	0.99941	0.74191	-0.43068	0.09023	0.40258	0.6584	-0.60296	0.011213	0.053195	0.46205	-1.1139	0.002077	0.045856	0.34797	-1.523	0.035259	0.37184	0.50876	-0.97493	0.008235	0.47452	0.42705	-1.2275	0.004372	0.2688	0.45267	-1.1435
877.5735	5.47	domics Pos	0.85344	0.99987	1.0245	0.034921	0.8121	0.99994	0.98197	-0.02626	0.42316	0.99996	0.89262	-0.16387	0.62706	0.94468	0.95796	-0.06196	0.013117	0.037714	0.76376	-0.38881	0.4315	0.6645	0.90586	-0.14263	0.57621	0.86079	0.95889	-0.06057	0.63708	0.97988	0.96412	-0.05272	0.10068	0.20269	1.1613	0.21572
1008.805	10.02	domics Pos	0.85393	0.99987	0.99073	-0.01344	0.048117	0.99994	0.88074	-0.18322	0.31796	0.99996	0.95323	-0.06911	0.14108	0.56927	0.89822	-0.15486	0.000625	0.003881	0.73504	-0.4441	0.27865	0.56576	0.88061	-0.18343	0.44847	0.80156	1.0372	0.052661	0.40205	0.96776	0.9554	-0.06582	0.078755	0.1809	1.1653	0.22065
177.1632	6.97	oolomics Pc	0.85429	0.99951	1.0247	0.035188	0.59714	0.99998	0.91678	-0.12535	0.038901	0.99941	0.69363	-0.52776	0.014612	0.20268	0.51144	-0.96735	0.001949	0.015227	0.39136	-1.3534	0.000623	0.039708	0.37899	-1.3998	0.006817	0.22137	0.41398	-1.2724	0.032097	0.58535	0.51046	-0.97014	0.062784	0.30404	0.64091	-0.64181
858.6624	10.3	olomics Ne	0.85533	0.99918	0.99529	-0.00681	0.017838	0.38525	0.88253	-0.18029	0.40453	0.99995	0.94069	-0.0882	0.23255	0.99985	0.96263	-0.05495	0.16465	0.99927	0.96783	-0.04718	0.58155	0.88819	0.97604	-0.03498	0.027995	0.33786	0.84729	-0.23906	0.047107	0.99512	0.90391	-0.14575	0.11995	0.99941	0.91867	-0.12239
281.2482	2.28	domics Pos	0.85573	0.99987	1.0632	0.088385	0.50792	0.99994	0.90491	-0.14415	0.039061	0.99996	0.62499	-0.6781	0.022878	0.26923	0.51574	-0.95528	0.000272	0.002067	0.36542	-1.4524	0.012265	0.093161	0.35337	-1.5007	0.009586	0.3324	0.40577	-1.3013	0.009423	0.52955	0.34543	-1.5335	0.045052	0.14081	0.4969	-1.009
720.5694	4.5	domics Pos	0.85578	0.99987	0.99144	-0.0124	0.087739	0.99994	0.84639	-0.24061	0.22431	0.99996	0.89105	-0.16643	0.25979	0.71791	0.89791	-0.15536	0.11469	0.19333	0.78861	-0.34262	0.012165	0.093	0.75495	-0.40555	0.8581	0.97205	0.98869	-0.01641	0.086557	0.82317	0.77059	-0.37596	0.83878	0.88123	0.98371	-0.0237
464.3137	1.92	domics Pos	0.85643	0.99987	0.94294	-0.08476	0.70755	0.99994	0.87797	-0.18775	0.59436	0.99996	0.83899	-0.25328	0.78589	0.97526	0.89952	-0.15277	0.15955	0.248	0.61672	-0.69732	0.26627	0.55389	1.2702	0.34503	0.041558	0.38314	1.7913	0.84103	0.29546	0.9483	1.2357	0.30527	0.0358	0.12841	1.7164	0.77942
907.8266	0.5	domics Pos	0.85664	0.99987	0.99288	-0.01031	0.17729	0.99994	0.93142	-0.1025	0.005205	0.99996	0.86575	-0.20798	0.004333	0.17581	0.852	-0.23108	7.89E-05	0.000882	0.72715	-0.45967	0.11185	0.35869	0.84443	-0.24395	0.40735	0.77818	0.93952	-0.09001	0.45034	0.96776	0.95036	-0.07346	0.10419	0.20689	1.1725	0.22964
565.3153	2.99	domics Pos	0.85679	0.99987	1.0146	0.020842	0.82601	0.99994	0.9727	-0.03994	0.44908	0.99996	0.91334	-0.13078	0.65557	0.95652	0.94663	-0.07913	0.013157	0.037776	0.52642	-0.92572	0.22141	0.51169	0.84476	-0.24338	0.2176	0.66052	1.2123	0.27769	0.18241	0.92582	1.2534	0.32582	0.038483	0.13216	1.5626	0.64396

922.6972	7.1	domics Pos	0.85712	0.99987	1.026	0.036971	0.71286	0.99994	1.0761	0.10581	0.79641	0.99996	1.0503	0.070848	0.74705	0.97368	0.96208	-0.05577	0.004122	0.015622	0.6031	-0.72954	0.016006	0.1078	0.54933	-0.86427	0.049515	0.40631	0.54918	-0.86466	0.002426	0.48691	0.27479	-1.8636	0.011007	0.094875	0.34303	-1.5436
1071.773	0.52	domics Pos	0.85716	0.99987	1.0267	0.038027	0.042811	0.99994	0.81828	-0.28934	0.15289	0.99996	0.87713	-0.18913	0.80947	0.98109	0.98022	-0.02882	0.041282	0.090154	0.74797	-0.41895	0.30511	0.58492	0.90881	-0.13794	0.15533	0.62672	0.82533	-0.27696	0.67955	0.97988	0.95994	-0.05899	0.066364	0.16604	1.1936	0.25535
741.5234	8.91	iolomics Ne	0.85722	0.99918	0.97673	-0.03397	0.4584	0.82821	0.77492	-0.36788	0.28233	0.99995	0.69193	-0.5313	0.82239	0.99985	0.92827	-0.10739	0.29876	0.99927	1.5636	0.64487	0.049385	0.48405	2.3999	1.263	0.092172	0.45775	2.1829	1.1263	0.2576	0.99512	1.616	0.69243	0.20607	0.99941	1.3241	0.40505
277.2175	5.73	bolomics Pc	0.85731	0.99951	1.0676	0.094367	0.52218	0.99998	0.92241	-0.11653	0.02492	0.99941	0.54286	-0.88134	0.013349	0.20085	0.36345	-1.4602	6.32E-05	0.001131	0.31879	-1.6493	0.000151	0.036471	0.25896	-1.9492	0.003112	0.20152	0.31687	-1.658	0.002408	0.47452	0.28894	-1.7912	0.006767	0.2688	0.38193	-1.3886
153.1282	6.97	bolomics Pc	0.85742	0.99951	0.98483	-0.02205	0.52202	0.99998	0.90448	-0.14483	0.019138	0.99941	0.71393	-0.48615	0.007081	0.1948	0.48971	-1.03	0.002219	0.016725	0.40853	-1.2915	0.001802	0.045425	0.36014	-1.4734	0.002616	0.20152	0.36824	-1.4413	0.01503	0.47452	0.45255	-1.1439	0.018743	0.2688	0.53036	-0.91496
1002.755	10.09	domics Pos	0.85745	0.99987	1.0203	0.029035	0.92653	0.99994	1.0071	0.010235	0.98261	0.99996	1.0209	0.029858	0.20668	0.65238	0.8882	-0.17104	0.034802	0.079271	0.80617	-0.31084	0.31006	0.58859	0.88293	-0.17963	0.88006	0.97532	0.99203	-0.01154	0.80466	0.97988	0.98037	-0.0286	0.27904	0.38775	1.1332	0.18035
583.3244	2.99	domics Pos	0.85747	0.99987	1.0198	0.028241	0.83806	0.99994	0.949	-0.07552	0.90014	0.99996	0.9691	-0.04529	0.80322	0.97856	0.94353	-0.08387	0.014817	0.041512	0.45837	-1.1254	0.1036	0.34299	0.74371	-0.42719	0.45724	0.8038	1.1689	0.22515	0.34696	0.96563	1.1593	0.2133	0.048408	0.14485	1.4617	0.54767
851.6593	6.01	domics Pos	0.85757	0.99987	0.98453	-0.0225	0.66345	0.99994	0.95573	-0.06533	0.20089	0.99996	0.88191	-0.1813	0.099869	0.51973	0.81642	-0.29261	0.04119	0.090005	0.77134	-0.37455	0.13474	0.40138	0.82621	-0.27542	0.80309	0.95444	0.9776	-0.03268	0.36403	0.96776	0.90383	-0.14588	0.90543	0.93082	1.0164	0.023426
1010.751	9.82	bolomics Pc	0.85786	0.99951	1.1115	0.15249	0.16434	0.99998	0.77418	-0.36926	0.86403	0.99941	1.0128	0.018301	0.011396	0.19869	0.5819	-0.78115	0.02145	0.088787	0.54505	-0.87555	0.016021	0.093702	0.60982	-0.71355	0.4114	0.98164	0.85098	-0.23281	0.80412	0.99956	1.005	0.007184	0.1994	0.46423	1.6496	0.7221
465.3034	12.15	iolomics Ne	0.85827	0.99918	0.99287	-0.01032	0.18378	0.60009	0.9109	-0.13463	0.28892	0.99995	0.9417	-0.08667	0.99385	0.99985	1.0044	0.006377	0.10059	0.99927	1.2566	0.32958	0.013508	0.40556	1.4695	0.55532	0.015777	0.28562	1.3181	0.39846	0.31521	0.99512	1.1103	0.15093	0.36073	0.99941	1.0967	0.13312
704.8676	10.02	domics Pos	0.85879	0.99987	0.97838	-0.03153	0.78416	0.99994	1.019	0.027194	0.73391	0.99996	0.96574	-0.05029	0.55723	0.90877	0.93784	-0.09259	0.032096	0.074624	0.77732	-0.36342	0.43594	0.66892	0.90993	-0.13617	0.87458	0.97532	0.98044	-0.0285	0.90636	0.98217	1.0172	0.024556	0.023241	0.11048	1.256	0.32888
1041.675	5.1	bolomics Pc	0.8589	0.99951	0.73575	-0.44272	0.98689	0.99998	0.71185	-0.49035	0.82299	0.99941	0.79104	-0.33817	0.46255	0.94005	1.2997	0.37814	0.271	0.62631	1.8628	0.89748	0.027129	0.12403	4.2207	2.0775	0.018393	0.30136	5.3628	2.423	0.26104	0.99956	1.8498	0.8874	0.25548	0.52545	1.5463	0.62881
800.6681	5.91	domics Pos	0.85911	0.99987	1.0395	0.055823	0.44088	0.99994	0.9252	-0.11216	0.61393	0.99996	0.94729	-0.07811	0.26952	0.73017	0.883	-0.17952	0.038749	0.086404	0.77426	-0.36911	0.24251	0.53067	0.86044	-0.21685	0.9279	0.98393	0.99554	-0.00645	0.38137	0.96776	0.88739	-0.17235	0.3305	0.44016	1.131	0.17762
418.1935	2.62	bolomics Pc	0.85934	0.99951	1.0732	0.1019	0.68462	0.99998	0.88354	-0.17864	0.16266	0.99941	0.61816	-0.69395	0.082733	0.38045	0.48377	-1.0476	0.003125	0.021166	0.20949	-2.2551	0.037991	0.15521	0.39574	-1.3374	0.049824	0.45099	0.41613	-1.2649	0.055531	0.7025	0.4803	-1.058	0.88347	0.95289	1.0221	0.031488
576.5975	0.34	iolomics Ne	0.85947	0.99918	1.0784	0.10892	0.53666	0.86583	0.9465	-0.07933	0.42947	0.99995	1.0792	0.10999	4.84E-05	0.059534	1.2667	0.34107	0.000151	0.02658	1.1789	0.23741	0.003245	0.36417	1.2823	0.35876	4.38E-05	0.053877	1.4353	0.52137	0.16835	0.99512	1.0879	0.12155	0.65378	0.99941	1.0457	0.064486
841.7122	6.42	domics Pos	0.85967	0.99987	0.98743	-0.01825	0.10119	0.99994	0.79289	-0.33481	0.33115	0.99996	0.89195	-0.16497	0.13938	0.56536	0.81188	-0.30066	0.012363	0.036012	0.74269	-0.42917	0.16806	0.45447	0.87471	-0.19312	0.33036	0.73968	1.1981	0.2607	0.37017	0.96776	0.85919	-0.21895	0.21688	0.32505	0.88415	-0.17764
383.2048	1.97	domics Pos	0.85974	0.99987	1.1262	0.17143	0.83016	0.99994	1.0334	0.047428	0.11523	0.99996	0.68202	-0.55212	0.006796	0.19397	0.49116	-1.0257	0.09778	0.17149	0.5642	-0.82572	0.010338	0.08787	0.42045	-1.25	0.019917	0.33809	0.58746	-0.76743	0.018658	0.52955	0.50815	-0.97666	0.063307	0.16148	0.64121	-0.64112
607.5085	5.12	domics Pos	0.85997	0.99987	1.0274	0.038993	0.60687	0.99994	0.95762	-0.06248	0.065674	0.99996	0.91723	-0.12464	0.22929	0.68626	0.90879	-0.13798	9.36E-05	0.000978	0.75834	-0.39908	0.23126	0.51854	0.89968	-0.15252	0.87344	0.97532	0.99947	-0.00076	0.77438	0.97988	0.98409	-0.02314	0.16423	0.27173	1.2028	0.26644
614.3332	5.21	iolomics Ne	0.86	0.99918	0.96645	-0.04924	0.90644	0.9934	1.0185	0.02644	0.52069	0.99995	1.0995	0.13686	0.76984	0.99985	1.0457	0.064487	0.51455	0.99927	1.1261	0.1714	0.025698	0.45192	1.6371	0.71117	0.017235	0.29108	1.669	0.73899	0.33064	0.99512	1.1634	0.21834	0.26781	0.99941	1.1572	0.21065
426.3583	5.1	bolomics Pc	0.86012	0.99951	0.85094	-0.23287	0.55306	0.99998	0.74335	-0.42788	0.46773	0.99941	0.71275	-0.48852	0.15335	0.54951	0.5328	-0.90834	0.10013	0.28649	0.43611	-1.1973	0.018536	0.10085	0.25118	-1.9932	0.050934	0.45418	0.34104	-1.552	0.10964	0.86561	0.44114	-1.1807	0.04165	0.28426	0.35509	-1.4938
960.6502	5.92	domics Pos	0.86069	0.99987	1.0403	0.056973	0.74646	0.99994	0.95709	-0.06327	0.55009	0.99996	0.91522	-0.12781	0.43954	0.86677	0.90083	-0.15067	0.029761	0.0704	0.75828	-0.3992	0.01663	0.10971	0.70845	-0.49726	0.12017	0.57711	0.79631	-0.32859	0.7304	0.97988	0.9567	-0.06386	0.059286	0.15688	1.2386	0.30868
279.1464	7.63	bolomics Pc	0.8616	0.99951	0.99687	-0.00452	0.91216	0.99998	1.0134	0.019202	0.018116	0.99941	0.86827	-0.20379	0.86922	0.99905	1.0252	0.035882	0.8106	0.99688	1.0208	0.02976	0.19832	0.47147	1.0936	0.12911	0.67006	0.99391	0.98087	-0.02787	0.59956	0.99956	0.97242	-0.04035	0.027024	0.2688	0.8961	-0.15826
88.1129	9.93	domics Pos	0.86168	0.99987	0.9916	-0.01217	0.6344	0.99994	0.95995	-0.05897	0.64951	0.99996	0.9755	-0.03579	0.36105	0.8151	0.95213	-0.07077	0.00195	0.008913	0.77193	-0.37345	0.26856	0.55653	0.89729	-0.15635	0.74213	0.93154	1.0268	0.038202	0.46902	0.96776	0.96915	-0.0452	0.01915	0.10521	1.1498	0.20144
1088.67	5.1	bolomics Pc	0.8618	0.99951	1.0301	0.042788	0.88734	0.99998	1.0837	0.11602	0.49904	0.99941	1.1789	0.23745	0.3649	0.86036	1.5976	0.67595	0.096958	0.27892	2.2676	1.1811	0.004638	0.058912	4.4979	2.1693	0.002278	0.19394	5.8166	2.5402	0.17004	0.95869	1.5922	0.671	0.13991	0.39385	1.755	0.81144
1040.674	1.11	domics Pos	0.86251	0.99987	0.77851	-0.36121	0.87068	0.99994	0.86095	-0.21599	0.86949	0.99996	0.79108	-0.33811	0.63983	0.95076	1.112	0.15322	0.45918	0.54042	1.6696	0.73948	0.031019	0.15833	4.6414	2.2146	0.015533	0.33809	6.135	2.6171	0.27594	0.94365	2.1142	1.0801	0.11505	0.21955	2.3809	1.2515
596.97	12.01	iolomics Ne	0.86262	0.99918	1.0268	0.03811	0.099112	0.51286	0.87786	-0.18793	0.61953	0.99995	0.96765	-0.04744	0.12652	0.99985	1.1094	0.14977	0.56187	0.99927	1.1206	0.16425	0.72854	0.94555	1.0355	0.050345	0.002528	0.22316	1.239	0.3092	0.5557	0.99512	1.0784	0.10889	0.035577	0.99941	1.1569	0.21027
569.3434	5.08	bolomics Pc	0.86282	0.99951	1.0187	0.026777	0.66417	0.99998	1.0628	0.087871	0.45899	0.99941	1.1206	0.16425	0.2355	0.69525	1.1682	0.22426	0.15216	0.40447	1.216	0.28216	0.00092	0.042705	1.7365	0.79621	0.001597	0.16989	1.8372	0.87749	0.15084	0.93461	1.1481	0.19925	0.23633	0.50436	1.1445	0.19476
649.5289	2.84	domics Pos	0.86288	0.99987	0.97201	-0.04096	0.35284	0.99994	0.80937	-0.30514	0.028653	0.99996	0.57833	-0.79002	0.038941	0.34213	0.38049	-1.3941	0.001303	0.006671	0.27359	-1.8699	0.001576	0.052056	0.20276	-2.3021	0.002725	0.26175	0.24017	-2.0579	0.008905	0.52955	0.28384	-1.8168	0.022339	0.10864	0.37649	-1.4093
325.2122	2.28	domics Pos	0.86319	0.99987	0.99521	-0.00693	0.4996	0.99994	0.92493	-0.11259	0.055638	0.99996	0.76671	-0.38325	0.020373	0.25894	0.61741	-0.6957	1.98E-06	0.000144	0.4938	-1.018	0.014741	0.10201	0.46923	-1.0916	0.006365	0.3088	0.54716	-0.86998	0.010965	0.52955	0.51428	-0.95938	0.060807	0.1578	0.62618	-0.67535
380.2102	6.96	bolomics Pc	0.86319	0.99951	0.98479	-0.02212	0.53944	0.99998	0.91559	-0.12723	0.043466	0.99941	0.729	-0.45602	0.047508	0.28918	0.58768	-0.7669	0.002398	0.017403	0.54953	-0.86372	0.00485	0.060423	0.42222	-1.2439	0.008923	0.24023	0.45812	-1.1262	0.014766	0.47452	0.47227	-1.0823	0.033201	0.2688	0.53499	-0.9024
948.7956	0.5	domics Pos	0.86324	0.99987	1.0186	0.026641	0.18442	0.99994	0.90816	-0.13898	0.28106	0.99996	0.91856	-0.12256	0.44268	0.86677	0.94065	-0.08827	0.01403	0.039745	0.76141	-0.39326	0.60434	0.78562	0.95369	-0.06841	0.57816	0.86163	1.0441	0.062196	0.35601	0.96776	0.94347	-0.08395	0.01319	0.098791	1.1856	0.24564
752.7923	10.08	domics Pos	0.86328	0.99987	1.0072	0.010331	0.37911	0.99994	0.91566	-0.12711	0.30305	0.99996	0.91948	-0.12112	0.3406	0.79697	0.91728	-0.12456	0.002257	0.00988	0.70809	-0.49799	0.22348	0.5122	0.88512	-0.17606	0.58969	0.86163	0.95452	-0.06715	0.44057	0.96776	0.93984	-0.08952	0.039816	0.13378	1.1578	0.21138
286.2809	7.49	bolomics Pc	0.86373	0.99951	0.8564	-0.22364	0.9323	0.99998	0.927	-0.10936	0.15267	0.99941	0.5459	-0.8733	0.049985	0.29486	0.4014	-1.3169	0.069655	0.21385	0.45222	-1.1449	0.023973	0.1146	0.36267	-1.4633	0.055392	0.47286	0.38828	-1.3648	0.051314	0.68379	0.41648	-1.2637	0.045338	0.28476	0.42335	-1.2401
1144.778	0.5	domics Pos	0.8644	0.99987	1.0133	0.019129	0.47752	0.99994	0.96765	-0.04745	0.089721	0.99996	0.92755	-0.1085	0.004886	0.17581	0.83573	-0.2589	0.000992	0.005504	0.72785	-0.45829	0.22352	0.5122	0.86106	-0.21582	0.19184	0.64891	0.89939	-0.15298	0.31364	0.9524	0.92389	-0.1142	0.12119	0.22555	1.1703	0.22684
121.0847	0.59	domics Pos	0.86449	0.99987	1.0277	0.039444	0.44444	0.99994	0.89913	-0.15339	0.13686	0.99996	1.2148	0.28072	0.02263	0.26881	1.3971	0.48239	0.83887	0.86332	0.97167	-0.04146	0.10673	0.34903	1.1933	0.25491	0.014386	0.33809	1.4246	0.51053	0.99151	0.99721	1.0287	0.040843	0.45996	0.56429	1.1021	0.14023
452.2616	5.27	iolomics Ne	0.86455	0.99918	0.935	-0.09697	0.79196	0.97675	0.91524	-0.12777	0.99995	0.99995	0.96774	-0.04731	0.76198	0.99985	1.0512	0.072014	0.60182	0.99927	1.1467	0.19745	0.037818	0.48405	1.8607	0.89585	0.00663	0.2539	2.2633	1.1784	0.08227	0.99512	1.5467	0.62919	0.076982	0.99941	1.6001	0.67812
317.9613	11.98	iolomics Ne	0.86489	0.99918	0.9931	-0.01	0.079698	0.50591	0.91079	-0.13481	0.95143	0.99995	1.0031	0.00444	0.63347	0.99985	0.97381	-0.03828	0.91443	0.99927	0.99631	-0.00534	0.047261	0.48405	1.0864	0.1195	0.79114	0.94658	1.0117	0.016844	0.47302	0.99512	1.0383	0.054166	0.39461	0.99941	0.9653	-0.05095
829.6773	5.72	domics Pos	0.86536	0.99987	1.0604	0.084634	0.53459	0.99994	0.88026	-0.18401	0.22468	0.99996	0.77023	-0.37665	0.35871	0.81307	0.81582	-0.29367	0.24371	0.33767	0.78152	-0.35565	0.27551	0.56257	1.1664	0.22203	0.044593	0.38909	1.5485	0.6309	0.23316	0.94365	1.2364	0.30617	0.10619	0.20848	1.2746	0.3501
798.7259	0.56	domics Pos	0.866	0.99987	0.98909	-0.01583	0.79416	0.99994	1.0164	0.023471	0.28774	0.99996	0.91982	-0.12057	0.090389	0.50095	0.88162	-0.18178	0.010377	0.031577	0.79145	-0.33743	0.012865	0.095682	0.79287	-0.33484	0.016977	0.33809	0.77338	-0.37076	0.027069	0.57206	0.81865	-0.28869	0.20542	0.31406	1.1336	0.18089
241.2192	6.97	bolomics Pc	0.86623	0.99951	1.0537	0.075491	0.29002	0.99998	0.81565	-0.29397	0.020399	0.99941	0.70439	-0.50555	0.017931	0.20936	0.52621	-0.92628	0.000176	0.002542	0.38303	-1.3845	0.0008	0.041994	0.37931	-1.3985	0.00166	0.16989	0.42182	-1.2453	0.025202	0.52574	0.5051	-0.98535	0.028993	0.2688	0.62432	-0.67965
221.2228	9.97	domics Pos	0.86658	0.99987	0.98789	-0.01757	0.038562	0.99994	0.84486	-0.24322	0.050201	0.99996	0.86569	-0.20808	0.022158	0.26776	0.80296	-0.3166	0.001124	0.005983	0.71583	-0.4823	0.14647	0.42188	0.8657	-0.20806	0.79797	0.95226	0.98139	-0.02711	0.50238	0.96776	0.94785	-0.07726	0.34967	0.45943	1.0802	0.11133
187.6277	5.68	bolomics Pc	0.86671	0.99951	0.94764	-0.07758	0.26185	0.99998	0.77553	-0.36674	0.43361	0.99941	0.84709	-0.23941	0.023049	0.22443	0.5442	-0.87779	0.001339	0.01166	0.28847	-1.7935	0.014449	0.090678	0.52625	-0.92617	0.097162	0.60726	0.65812	-0.60358	0.14907	0.93461	0.73404	-0.44606	0.77447	0.90218	0.94115	-0.0875
1196.836	4.86	domics Pos	0.86672	0.99987	0.98304	-0.02468	0.61537	0.99994	0.92623	-0.11055	0.54511	0.99996	0.91515	-0.12792	0.2838	0.74963	0.83666	-0.25729	0.024586	0.060952	0.68882	-0.53781	0.26906	0.55659	0.81844	-0.28905	0.42142	0.78461	0.88902	-0.16972	0.15254	0.91321	0.79338	-0.33391	0.34727	0.45787	0.88283	-0.17979
97.1021	6.98	bolomics Pc	0.86711	0.99951	1.0092	0.013192	0.32078	0.99998	0.85986	-0.21783	0.10382	0.99941	0.81618	-0.29305	0.063372	0.33051	0.62093	-0.6875	9.14E-05	0.001485	0.36136	-1.4685	0.000284	0.039399	0.4168	-1.2626	0.001799	0.16989	0.53145	-0.91199	0.041752	0.64572	0.64068	-0.64233	0.043303	0.28452	0.74472	-0.42524
240.2288	9.91	domics Pos	0.86714	0.99987	1.0055	0.00793	0.52217	0.99994	0.9339	-0.09866	0.14642	0.99996	0.87034	-0.20035	0.043019	0.3621	0.87275	-0.19635	0.008599	0.0273	0.83875	-0.25369	0.37252	0.63038	0.89552	-0.15919	0.7445	0.93287	0.97565	-0.03557	0.97717	0.9929	1.006	0.008608	0.16832	0.27555	1.1115	0.15255
923.7086	7.25	domics Pos	0.86734	0.99987	1.0262	0.037312	0.87493	0.99994	1.0341	0.04838	0.95821	0.99996	0.99278	-0.01046	0.47573	0.88482	0.85838	-0.22031	0.05796	0.1162	0.63106	-0.66416	0.052674	0.22542	0.51828	-0.94818	0.10557	0.55257	0.53201	-0.91048	0.014535	0.52955	0.32224	-1.6338	0.041021	0.13505	0.38278	-1.3854
457.3469	1.97	domics Pos	0.86734	0.99987	0.97987	-0.02934	0.061916	0.99994	0.82155	-0.28359	0.60676	0.99996	0.94262	-0.08525	0.47886	0.88482	0.91631	-0.12609	0.011862	0.034927	0.72065	-0.47263	0.74259	0.86962	1.1155	0.1577	0.58198	0.86163	1.2593	0.33259	0.25028	0.94365	0.86557	-0.20828	0.51493	0.61473	1.2138	0.27955
374.3024	6.05	bolomics Pc	0.86799	0.99951	0.98713	-0.01869	0.77991	0.99998	0.98195	-0.02627	0.5163	0.99941	0.9242	-0.11372	0.31987	0.81415	0.89766	-0.15576	0.55753	0.94128	1.0643	0.089926	0.25243	0.54167	0.8491	-0.23599	0.23455	0.83959	0.85125	-0.23234	0.020578	0.51556	0.77837	-0.36146	0.1674	0.42801	0.8158	-0.29371
961.6267	6.05	domics Pos	0.86809	0.99987	1.0376	0.053224	0.57888	0.99994	0.93843	-0.09168	0.68514	0.99996	0.96664	-0.04896	0.41607	0.85524	1.1028	0.14121	0.022994	0.057986	0.78475	-0.34969	0.1249	0.38437	0.76727	-0.38219	0.34802	0.74762	0.91448	-0.12898	0.55519	0.96798	0.94943	-0.07486	0.13826	0.24373	1.1976	0.2602
973.5904	5.79	domics Pos	0.86906	0.99987	0.96359	-0.05351	0.65637	0.99994	0.92998	-0.10474	0.96567	0.99996	0.98473	-0.0222	0.94097	0.99181	0.98971	-0.01492	0.047522	0.10028	0.70873	-0.49669	0.2097	0.50173	0.81249	-0.29959	0.20445	0.64911	0.81265	-0.2993	0.29545	0.9483	0.85876	-0.21967	0.11918	0.22343	1.2296	0.29822
299.14	6.98	bolomics Pc	0.86943	0.99951	1.0031	0.004513	0.88384	0.99998	0.99074	-0.01342	0.68401	0.99941	1.0454	0.064003	0.93729	0.99905	0.99977	-0.00033	0.031855	0.12104	0.7314	-0.45127	0.06829	0.2313	0.71676	-0.48045	0.10427	0.61787	0.75389	-0.40757	0.18891	0.98781	0.767	-0.3827	0.24703	0.51464	0.8235	-0.28016
860.6096	5.63	domics Pos	0.86962	0.99987	1.0227	0.032395	0.61634	0.99994	0.93683	-0.09415	0.92437	0.99996	0.98453	-0.0225	0.67764	0.95843	0.94948	-0.07479	0.017712	0.047459	0.71467	-0.48465	0.20162	0.48967	0.81474	-0.29558	0.30259	0.72565	0.86338	-0.21194	0.25042	0.94365	0.85153	-0.23187	0.27592	0.38524	1.1178	0.16066
1016.804	11.98	bolomics Pc	0.86966	0.99951	1.0183	0.026186	0.24338	0.99998	1.1491	0.20045	0.85373	0.99941	1.0238	0.03396	0.43367	0.91715	1.0816	0.11313	0.026301	0.10401	1.1503	0.202	0.014811	0.091119	1.2504	0.32244	0.20388	0.79496	1.1684	0.22448	0.83765	0.99956	0.98846	-0.01675	0.39092	0.66422	1.0829	0.1149
439.2943	1.54	domics Pos	0.87001	0.99987	1.1109	0.15173	0.83896	0.99994	0.95506	-0.06634	0.67647	0.99996	0.91508	-0.12803	0.5406	0.90385	0.87324	-0.19555	0.001833	0.008483	0.21786	-2.1985	0.35713	0.62123	0.82286	-0.28129	0.82362	0.96156	1.0816	0.11319	0.85435	0.97988	1.0749	0.10414	0.076148	0.17781	1.4913	0.57658
853.6818	10.28	bolomics Pc	0.87011	0.99951	0.99049	-0.01379	0.77249	0.99998	0.97844	-0.03145	0.39124	0.99941	0.90805	-0.13915	0.70916	0.99905	0.96894	-0.04552	0.69889	0.99688	1.0356	0.050483	0.47019	0.77383	0.94609	-0.07996	0.32052	0.93343	0.93681	-0.09418	0.042482	0.64572	0.81975	-0.28675	0.052374	0.2951	0.81261	-0.29936
1117.839	4.66	domics Pos	0.87016	0.99987	0.988	-0.01742	0.36137	0.99994	0.89608	-0.1583	0.30755	0.99996	0.83434	-0.2613	0.1115	0.53238	0.79096	-0.33832	0.42737	0.51356	0.91634	-0.12604	0.4508	0.68129	1.058	0.081307	0.033155	0.36346	1.1816	0.24069	0.83311	0.97988	0.98363	-0.02381	0.012972	0.098791	1.278	0.35394
150.8934	0.33	iolomics Ne	0.87027	0.99918	1.0068	0.009785	0.006983	0.34605	0.87385	-0.19454	0.88458	0.99995	0.99418	-0.00842	0.30415	0.99985	1.0412	0.058198	0.85197	0.99927	0.99259	-0.01073	0.24049	0.68109	1.052	0.073146	0.69257	0.89955	1.0139	0.019869	0.96645	0.99512	0.99816	-0.00266	0.21068	0.99941	0.92706	-0.10926
147.1177	4.74	domics Pos	0.87046	0.99987	1.0094	0.013526	0.20882	0.99994	0.90175	-0.1492	0.46748	0.99996	0.94499	-0.08163	0.61356	0.93767	0.94783	-0.0773	0.005479	0.01958	0.75917	-0.3975	0.5726	0.76608	0.9492	-0.07522	0.77208	0.94358	0.97527	-0.03613	0.80038	0.97988	1.0198	0.028333	0.15699	0.26358	1.1592	0.2131
744.549	10.11	iolomics Ne	0.87112	0.99918	0.9261	-0.11075	0.55058	0.8734	0.82786	-0.27254	0.69011	0.99995	0.87589	-0.19118	0.87583	0.99985	0.94679	-0.07889	0.26689	0.99927	1.4582	0.54416	0.051121	0.48407	2.2008	1.1381	0.035337	0.35618	2.378	1.2497	0.1805	0.99512	1.7192	0.78171	0.072212	0.99941	1.6812	0.74949
736.4972	8.44	iolomics Ne	0.87188	0.99918	0.96377	-0.05324	0.80338	0.97675	0.9462	-0.07979	0.75091	0.99995	0.93485	-0.0972	0.83481	0.99985	0.97473	-0.03692	0.27159	0.99927	1.2481	0.31978	0.031888	0.47167	1.6774	0.74623	0.043885	0.38044	1.7031	0.76814	0.23669	0.99512	1.4316	0.51766	0.14478	0.99941	1.3862	0.47112
67.0558	6.03	bolomics Pc	0.87219	0.99951	1.0068	0.009815	0.10425	0.99998	0.84297	-0.24645	0.20633	0.99941	0.8419	-0.24827	0.032035	0.24911	0.64945	-0.6227	2.3E-05	0.00052	0.31523	-1.6655	0.001629	0.045425	0.50924	-0.97359	0.018127	0.30136	0.61108	-0.71057	0.011164	0.47452	0.70003	-0.51451	0.069773	0.3118	0.8641	-0.21073
747.5641	4.66	lomics Neg	0.87233	0.99977	1.0219	0.031214	0.53248	0.99904	0.91045	-0.13534	0.33164	0.99994	0.83591	-0.25859	0.31473	0.58182	1.3416	0.42394	0.97385	0.99989	1.214	0.27975	0.98568	0.99653	1.2288	0.29727	0.037569	0.37635	0.68708	-0.54145	0.050795	0.46971	0.55151	-0.85853	0.76297	0.85252	1.0518	0.072798
392.9771	12	iolomics Ne	0.87243	0.99918	1.0108	0.015474	0.047978	0.47152	0.85126	-0.23233	0.54028	0.99995	0.95464	-0.06698	0.49816	0.99985	0.93836	-0.09178	0.79093	0.99927	1.0284	0.040386	0.58768	0.89313	0.95301	-0.06944	0.50111	0.81279	0.96092	-0.05752	0.26995	0.99512	0.91028	-0.13562	0.43341	0.99941	0.94401	-0.08313
821.6482	5.9	domics Pos	0.87247	0.99987	0.98522	-0.02148	0.2177	0.99994	0.82446	-0.27849	0.23033	0.99996	0.853	-0.22938	0.2441	0.70564	0.85461	-0.22666	0.026625	0.064871	0.7265	-0.46096	0.29889	0.57969	0.83905	-0.25317	0.55166	0.85184	0.92052	-0.11948	0.1858	0.92582	0.80412	-0.31452	0.67966	0.76079	0.9433	-0.08421
193.1588	2.28	domics Pos	0.8727	0.99987	0.97624	-0.03469	0.52827	0.99994	0.88624	-0.17423	0.1336	0.99996	0.68842	-0.53864	0.014227	0.22898	0.49573	-1.0124	0.001126	0.005987	0.34068	-1.5535	0.00892	0.08583	0.3858	-1.3741	0.020437	0.33809	0.45613	-1.1325	0.015519	0.52955	0.39979	-1.3227	0.086112	0.18686	0.5461	-0.87277
193.1593	6.48	bolomics Pc	0.87271	0.99951	0.99701	-0.00431	0.80895	0.99998	1.0145	0.020825	0.18152	0.99941	0.73406	-0.44603	0.047021	0.28918	0.56809	-0.81581	0.004273	0.026239	0.46603	-1.1015	0.013553	0.086248	0.44604	-1.1647	0.024226	0.32473	0.42968	-1.2187	0.010137	0.47452	0.40917	-1.2892	0.0364	0.27696	0.44842	-1.1571
505.3105	1.92	domics Pos	0.87293	0.99987	0.94495	-0.0817	0.6982	0.99994	0.90166	-0.14935	0.5719	0.99996	0.86373	-0.21136	0.75167	0.97526	0.91344	-0.13062	0.12657	0.20799	0.62027	-0.68902	0.41767	0.65592	1.1709	0.22768	0.023533	0.33809	1.739	0.79825	0.26677	0.94365	1.2227	0.2901	0.033942	0.12554	1.6182	0.69436
334.1621	6.48	bolomics Pc	0.87315	0.99951	1.0142	0.020285	0.81885	0.99998	0.97411	-0.03784	0.21561	0.99941	0.77613	-0.36563	0.26935	0.74055	0.7874	-0.34483	0.16915	0.43855	0.77729	-0.36347	0.073425	0.2427	0.60512	-0.7247	0.068884	0.53288	0.5855	-0.77226	0.026044	0.53972	0.56139	-0.83293	0.040714	0.28339	0.51846	-0.9477
203.1793	6.48	bolomics Pc	0.87553	0.99951	0.9912	-0.01275	0.48422	0.99998	0.89054	-0.16724	0.054	0.99941	0.73307	-0.44798	0.065315	0.33346	0.69518	-0.52455	0.000364	0.004495	0.40482	-1.3047	0.006454	0.067091	0.51631	-0.9537	0.010206	0.24769	0.4404	-1.1831	0.016528	0.49126	0.53876	-0.89228	0.025957	0.2688	0.44445	-1.1699
302.3068	4.72	bolomics Pc	0.87555	0.99951	1.027	0.038417	0.77008	0.99998	0.90935	-0.1371	0.79615	0.99941	0.96951	-0.04468	0.76698	0.99905	1.2396	0.3099	0.006387	0.034808	0.42204	-1.2446	0.26604	0.55721	0.72708	-0.45981	0.34193	0.95245	0.76929	-0.3784	0.41641	0.99956	0.79582	-0.32948	0.51941	0.76348	1.2012	0.26443
311.2344	2.05	domics Pos	0.87575	0.99987	1.011	0.01572	0.64769	0.99994	0.93009	-0.10455	0.037328	0.99996	0.69916	-0.51631	0.052361	0.39225	0.60972	-0.71378	0.02161	0.055413	0.531	-0.91321	0.0146	0.10138	0.39567	-1.3376	0.085181	0.50896	0.56371	-0.82697	0.013569	0.52955	0.48378	-1.0476	0.018768	0.10435	0.51477	-0.95799
281.2482	6.96	iolomics Ne	0.87596	0.99918	0.97979	-0.02945	0.36098	0.76696	0.80716	-0.30908	0.090086	0.99995	0.60333	-0.72898	0.02831	0.99985	0.42864	-1.2222	0.001418	0.17451	0.37224	-1.4257	0.002087	0.36417	0.25301	-1.9827	0.00759	0.2539	0.31172	-1.6817	0.017441	0.99512	0.34857	-1.5205	0.022102	0.99941	0.37574	-1.4122
1017.782	0.51	domics Pos	0.87632	0.99987	1.0171	0.024391	0.35796	0.99994	0.91247	-0.13215	0.25185	0.99996	0.90021	-0.15166	0.20194	0.64705	0.87558	-0.19169	0.012662	0.036687	0.74711	-0.42061	0.1287	0.39134	0.79263	-0.33528	0.5487	0.85184	0.94418	-0.08287	0.32024	0.9524	0.88655	-0.17373	0.24035	0.34833	1.0953	0.13129
466.3067	12.15	iolomics Ne	0.87665	0.99918	1.0135	0.019283	0.10983	0.52291	0.88133	-0.18225	0.32126	0.99995	0.94234	-0.08568	0.82511	0.99985	0.98715	-0.01866	0.14653	0.99927	1.2454	0.31656	0.012474	0.40245	1.4786	0.56419	0.027662	0.33786	1.2882	0.36531	0.28807	0.99512	1.1147	0.15664	0.30274	0.99941	1.1257	0.17078
894.5721	5.78	domics Pos	0.87687	0.99987	0.99411	-0.00852	0.4862	0.99994	0.96714	-0.0482	0.79493	0.99996	0.98582	-0.02061	0.23555	0.69438	0.93459	-0.0976	0.001004	0.005526	0.75997	-0.39598	0.00049	0.033979	0.73743	-0.43943	0.029408	0.35275	0.85407	-0.22757	0.001961	0.48691	0.87218	-0.19731	0.25389	0.36271	1.132	0.17885
785.6517	5.71	domics Pos	0.87693	0.99987	1.03	0.042671	0.31463	0.99994	0.89445	-0.16093	0.68963	0.99996	0.95862	-0.06098	0.39317	0.83798	0.89238	-0.16427	0.043746	0.094398	0.75444	-0.40652	0.33478	0.60787	0.87612	-0.1908	0.85398	0.97056	0.99272	-0.01054	0.44752	0.96776	0.90115	-0.15017	0.83374	0.87722	1.0256	0.036487
587.5399	5.72	domics Pos	0.87753	0.99987	1.0115	0.016507	0.31015	0.99994	0.78336	-0.35226	0.82243	0.99996	0.98245	-0.02554	0.69957	0.96092	0.94273	-0.08508	0.66768	0.71856	0.90911	-0.13748	0.032586	0.16205	1.5472	0.6297	0.038307	0.37121	1.7543	0.81092	0.085672	0.82284	1.6358	0.71001	0.045298	0.14134	1.5978	0.67604
248.2462	6.96	bolomics Pc	0.87772	0.99951	0.98634	-0.01984	0.42076	0.99998	0.85445	-0.22693	0.021881	0.99941	0.64099	-0.64164	0.017715	0.20936	0.46354	-1.1092	0.002766	0.019277	0.38962	-1.3599	0.002676	0.046658	0.3098	-1.6906	0.00127	0.16989	0.33364	-1.5837	0.020646	0.51556	0.41192	-1.2796	0.015151	0.2688	0.41547	-1.2672
383.983	12.01	iolomics Ne	0.87815	0.99918	1.0048	0.00694	0.35072	0.76325	0.862	-0.21424	0.042466	0.99995	0.754	-0.40736	0.20473	0.99985	0.83416	-0.2616	0.24868	0.99927	0.81198	-0.30049	0.59901	0.90254	0.92507	-0.11237	0.59198	0.86041	0.92206	-0.11707	0.76737	0.99512	0.95924	-0.06004	0.94943	0.99941	1.023	0.032845
353.2596	2.08	domics Pos	0.87832	0.99987	1.0565	0.07924	0.23906	0.99994	0.75878	-0.39824	0.21853	0.99996	0.74377	-0.42707	0.038439	0.34165	0.61909	-0.69177	0.093917	0.16647	0.6523	-0.61638	0.29381	0.57627	0.77594	-0.36599	0.68905	0.90885	0.90782	-0.13953	0.19625	0.92582	0.7627	-0.39082	0.30307	0.41272	1.3854	0.47028
861.5287	5.9	domics Pos	0.8796	0.99987	1.0567	0.079611	0.88736	0.99994	0.95659	-0.06402	0.79438	0.99996	0.99499	-0.00724	0.7714	0.97526	0.93558	-0.09607	0.56953	0.6322	1.1506	0.20233	0.042762	0.19457	1.833	0.87421	0.040592	0.37935	1.8355	0.87617	0.077514	0.79451	1.7726	0.82588	0.003621	0.085302	2.5186	1.3326
368.3897	8.84	bolomics Pc	0.88036	0.99951	0.97315	-0.03927	0.48236	0.99998	0.90342	-0.14653	0.37258	0.99941	0.88593	-0.17474	0.98397	0.99905	0.98947	-0.01528	0.29152	0.6564	1.1171	0.15974	0.04817	0.18215	0.71072	-0.49265	0.36631	0.96767	0.84579	-0.24163	0.054236	0.69529	0.72696	-0.46006	0.4185	0.6896	0.8754	-0.19198
518.3672	3.52	domics Pos	0.88037	0.99987	0.94746	-0.07786	0.73911	0.99994	0.91114	-0.13426	0.081469	0.99996	0.6633	-0.59227	0.017881	0.25028	0.52574	-0.92759	0.005095	0.018467	0.41122	-1.282	0.02704	0.14542	0.44001	-1.1844	0.047355	0.39685	0.57237	-0.80498	0.079071	0.79555	0.59393	-0.75164	0.23333	0.34061	0.71255	-0.48894
824.543	4.53	lomics Neg	0.88106	0.99977	1.2148	0.28074	0.99797	0.99904	1.1171	0.15975	0.45853	0.99994	0.88298	-0.17955	0.2396	0.58182	1.3866	0.47155	0.83221	0.99989	1.1282	0.174	0.95803	0.99653	1.2515	0.32363	0.1192	0.45153	0.68975	-0.53585	0.038341	0.46971	0.46412	-1.1074	0.36756	0.54948	0.84703	-0.23951
914.5764	5.58	domics Pos	0.88144	0.99987	0.98309	-0.0246	0.43822	0.99994	0.87179	-0.19794	0.97685	0.99996	0.98964	-0.01503	0.4123	0.85487	0.86194	-0.21433	0.04469	0.095814	0.64869	-0.6244	0.22677	0.51408	0.77652	-0.3649	0.20306	0.64891	0.81419	-0.29656	0.21153	0.92921	0.7667	-0.38326	0.44868	0.55312	0.86954	-0.20167
184.0741	4.93	domics Pos	0.88153	0.99987	1.0089	0.012853	0.86511	0.99994	0.98812	-0.01725	0.93993	0.99996	0.99406	-0.0086	0.33806	0.7957	0.93077	-0.1035	0.006831	0.022971	0.7783	-0.3616	0.39177	0.64223	0.93422	-0.09817	0.94095	0.98682	0.99482	-0.00749	0.65203	0.97988	1.0271	0.038632	0.069619	0.17093	1.2182	0.28472
328.7632	0.57	lomics Neg	0.88242	0.99977	0.97624	-0.03469	0.88212	0.99904	1.0012	0.001793	0.56572	0.99994	0.93177	-0.10195	0.36537	0.58182	1.1861	0.24619	0.4804	0.99989	1.0665	0.092906	0.85914	0.99653	1.0096	0.013835	0.033521	0.37635	0.74048	-0.43346	0.18235	0.59205	0.78987	-0.34031	0.083517	0.405	0.76384	-0.38865
264.2414	2.28	domics Pos	0.88267	0.99987	0.99309	-0.01	0.38551	0.99994	0.83427	-0.26141	0.066533	0.99996	0.608	-0.71787	0.004241	0.17581	0.42812	-1.2239	7.37E-05	0.000849	0.29415	-1.7654	0.011394	0.091661	0.32516	-1.6208	0.024784	0.33809	0.38329	-1.3835	0.009427	0.52955	0.32942	-1.602	0.017083	0.10008	0.407	-1.2969
256.8216	1	domics Pos	0.88297	0.99987	1.0187	0.026708	0.7608	0.99994	0.93986	-0.08948	0.83061	0.99996	0.96435	-0.05236	0.53843	0.90305	0.8866	-0.17365	0.073043	0.13884	0.71498	-0.48403	0.18307	0.47109	0.73677	-0.44072	0.015575	0.33809	0.62643	-0.67477	0.96386	0.98873	0.98147	-0.02698	0.46019	0.56439	1.108	0.1479
169.1294	6.47	bolomics Pc	0.88307	0.99951	1.0129	0.018471	0.47046	0.99998	0.92774	-0.10821	0.19318	0.99941	0.88043	-0.18372	0.23244	0.68749	0.76622	-0.38417	0.001643	0.01341	0.44628	-1.164	0.007973	0.074341	0.49456	-1.0158	0.000175	0.16989	0.44697	-1.1617	0.018046	0.51556	0.45885	-1.1239	0.044057	0.28476	0.52756	-0.9226
703.8667	0.5	domics Pos	0.88327	0.99987	1.0052	0.00748	0.26939	0.99994	0.91171	-0.13336	0.54715	0.99996	0.95376	-0.0683	0.32039	0.78434	0.90791	-0.13938	0.004951	0.018117	0.75644	-0.40269	0.35879	0.62208	0.88917	-0.16946	0.58342	0.86163	0.95241	-0.07034	0.84866	0.97988	0.98237	-0.02566	0.13084	0.23589	1.213	0.27862
738.5069	8.74	iolomics Ne	0.88359	0.99918	0.99966	-0.00049	0.54187	0.86854	0.8951	-0.15988	0.25032	0.99995	0.83975	-0.25197	0.62797	0.99985	0.92087	-0.11894	0.36169	0.99927	1.1926	0.25406	0.065245	0.51001	1.5335	0.61678	0.011428	0.2716	1.6437	0.71692	0.00689	0.99512	1.7154	0.77858	0.013218	0.99941	1.522	0.60596
1061.652	1.11	domics Pos	0.88386	0.99987	0.72279	-0.46836	0.90196	0.99994	0.88627	-0.17419	0.83962	0.99996	0.8269	-0.27421	0.82417	0.98451	0.84491	-0.24312	0.43789	0.52243	1.4749	0.56062	0.046188	0.20646	3.9326	1.9755	0.022538	0.33809	4.9166	2.2977	0.24987	0.94365	1.9112	0.93445	0.16074	0.26805	2.0586	1.0416
402.3051	2.73	domics Pos	0.88395	0.99987	0.99043	-0.01387	0.27805	0.99994	0.8305	-0.26794	0.37281	0.99996	0.87564	-0.19159	0.018521	0.25459	0.64264	-0.63791	0.000776	0.0046	0.53177	-0.91113	0.005851	0.076405	0.61969	-0.69038	0.013561	0.33809	0.73531	-0.44357	0.002939	0.52955	0.55255	-0.85582	0.33094	0.44044	0.83377	-0.26229
684.8044	10.09	domics Pos	0.88495	0.99987	0.98529	-0.02138	0.22969	0.99994	0.8935	-0.16246	0.52677	0.99996	0.94961	-0.07459	0.75769	0.97526	0.97264	-0.04002	0.001732	0.008125	0.67574	-0.56546	0.2174	0.50811	0.86424	-0.21049	0.88197	0.97532	0.98639	-0.01978	0.87966	0.98023	0.99203	-0.01155	0.76471	0.82415	1.0337	0.047884
416.3151	0.93	domics Pos	0.88509	0.99987	1.0133	0.019102	0.88468	0.99994	1.1375	0.18584	0.34744	0.99996	2.1938	1.1335	0.045519	0.3685	4.8715	2.2844	0.011718	0.034608	4.5203	2.1764	0.032057	0.16113	2.6818	1.4232	0.007084	0.3141	5.0984	2.35	0.14887	0.91277	1.7216	0.78377	0.10773	0.21059	1.8342	0.87516
200.0415	0.59	domics Pos	0.88511	0.99987	1.0067	0.009629	0.44301	0.99994	0.92268	-0.1161	0.42373	0.99996	1.1113	0.15222	0.071553	0.4548	1.3149	0.39493	0.70555	0.74938	1.0356	0.050497	0.032449	0.16174	1.2719	0.34695	0.00587	0.3088	1.4617	0.54762	0.32184	0.9529	1.1486	0.19994	0.057386	0.1547	1.2651	0.33921
660.2536	1.38	domics Pos	0.88554	0.99987	1.0119	0.0171	0.61612	0.99994	0.91922	-0.12151	0.62277	0.99996	1.1772	0.23532	0.58277	0.92348	1.1282	0.17402	0.4573	0.53955	0.881	-0.18279	0.10951	0.35386	1.2083	0.273	0.038379	0.37121	1.4259	0.5119	0.8798	0.98023	1.0525	0.073763	0.055588	0.15271	1.5678	0.64876
186.0491	10.2	domics Pos	0.88569	0.99987	0.99164	-0.01211	0.28804	0.99994	0.92693	-0.10946	0.45763	0.99996	0.94977	-0.07435	0.15132	0.57987	0.91032	-0.13555	0.004985	0.018206	0.79247	-0.33556	0.1341	0.40042	0.86672	-0.20637	0.44736	0.80121	0.96076	-0.05775	0.33959	0.96524	0.93621	-0.09509	0.016362	0.099614	1.2105	0.27558
196.8661	0.99	domics Pos	0.88574	0.99987	0.98139	-0.0271	0.1361	0.99994	0.82364	-0.27992	0.14604	0.99996	0.8223	-0.28226	0.099911	0.51973	0.75301	-0.40926	0.015399	0.04261	0.67208	-0.5733	0.048402	0.21336	0.69723	-0.5203	0.015418	0.33809	0.61408	-0.70351	0.36915	0.96776	0.89925	-0.15321	0.24746	0.35647	0.85011	-0.23428
364.2514	3.52	domics Pos	0.88585	0.99987	0.95792	-0.06202	0.23576	0.99994	0.77652	-0.3649	0.19364	0.99996	0.75598	-0.40357	0.008929	0.19971	0.52677	-0.92475	0.005159	0.018628	0.45451	-1.1376	0.009631	0.087438	0.4274	-1.2263	0.021391	0.33809	0.49196	-1.0234	0.021551	0.53012	0.53178	-0.91111	0.22717	0.33418	0.73254	-0.44901
455.2963	5.28	bolomics Pc	0.88611	0.99951	0.97237	-0.04042	0.80642	0.99998	0.93886	-0.09102	0.94657	0.99941	0.94889	-0.07569	0.58109	0.99905	1.154	0.20665	0.45592	0.85802	1.1896	0.25047	0.031705	0.13696	1.9515	0.96457	0.011347	0.25802	2.331	1.221	0.068971	0.75699	1.6439	0.71712	0.084593	0.33476	1.6082	0.68541

869.6692	5.46	olomics Pos	0.88621	0.99987	1.0244	0.034837	0.35939	0.99994	0.92132	-0.11822	0.33447	0.99996	0.91759	-0.12408	0.008019	0.19917	0.74034	-0.43373	0.008179	0.026364	0.65327	-0.61424	0.011917	0.092554	0.67824	-0.56012	0.29248	0.7186	0.90008	-0.15187	0.77297	0.97988	0.98085	-0.02789	0.5741	0.66862	0.93952	-0.09
603.7925	2.36	olomics Pc	0.8863	0.99951	1.0152	0.021732	0.81103	0.99998	0.82776	-0.27271	0.5582	0.99941	0.69313	-0.5288	0.30642	0.79639	0.52568	-0.92774	0.013971	0.062872	0.15007	-2.7363	0.18716	0.45906	0.39839	-1.3278	0.44901	0.99391	0.64493	-0.63277	0.55065	0.99956	0.74568	-0.42336	0.76826	0.90022	1.038	0.053846
98.9613	10.18	olomics Pos	0.88655	0.99987	0.99315	-0.00992	0.76498	0.99994	0.97934	-0.03012	0.60641	0.99996	1.0625	0.087449	0.63383	0.94754	0.96302	-0.05436	0.004938	0.018086	0.77353	-0.37047	0.41888	0.65671	0.9325	-0.10083	0.4682	0.80647	1.0378	0.053466	0.47901	0.96776	1.0541	0.075972	0.022849	0.10945	1.2665	0.34087
202.1461	6.39	olomics Pc	0.88675	0.99951	1.0092	0.013231	0.43255	0.99998	0.94526	-0.08122	0.58893	0.99941	0.95556	-0.06559	0.36033	0.85278	0.90254	-0.14794	2.1E-05	0.000496	0.38396	-1.381	0.036992	0.15192	0.75484	-0.40576	0.5552	0.99391	0.94438	-0.08256	0.28644	0.99956	0.93364	-0.09906	0.15334	0.41003	1.1043	0.14309
739.5437	5.45	olomics Pos	0.88719	0.99987	0.97562	-0.0356	0.50319	0.99994	0.90915	-0.13741	0.97583	0.99996	1.0002	0.00028	0.40944	0.85171	0.89028	-0.16767	0.048124	0.10142	0.75568	-0.40416	0.9709	0.98433	1.0007	0.000995	0.051437	0.41162	1.3501	0.43304	0.85872	0.98023	1.0213	0.030451	0.075778	0.17781	1.2103	0.27541
352.8533	12.73	olomics Ne	0.88736	0.99918	1.0063	0.008995	0.057868	0.50118	0.87588	-0.19119	0.1152	0.99995	1.0561	0.078807	0.89148	0.99985	1.0113	0.016251	0.57122	0.99927	0.97877	-0.03095	0.017961	0.40708	1.1366	0.18468	0.18647	0.56539	1.0574	0.08055	0.59634	0.99512	1.0399	0.0564	0.49212	0.99941	1.0224	0.032022
159.6093	1.66	olomics Pos	0.88742	0.99987	1.0109	0.015589	0.72168	0.99994	0.95893	-0.0605	0.70251	0.99996	1.0918	0.12674	0.10546	0.52975	0.7433	-0.42798	3.13E-05	0.000482	0.24314	-2.0401	0.011942	0.092554	0.58505	-0.77337	0.070209	0.47049	0.76173	-0.39265	0.77751	0.97988	0.97057	-0.04309	0.059883	0.15736	1.2961	0.37421
601.47	8.37	olomics Pc	0.88752	0.99951	0.99214	-0.01139	0.29856	0.99998	0.91433	-0.12922	0.23045	0.99941	0.93244	-0.10092	0.057286	0.31383	0.88188	-0.18135	0.66864	0.99535	1.0197	0.028135	0.60779	0.87343	0.96936	-0.0449	0.33263	0.94653	0.91491	-0.1283	0.014112	0.47452	0.80381	-0.31507	0.011246	0.2688	0.79138	-0.33756
544.34	5.1	olomics Pc	0.88815	0.99951	1.0056	0.008095	0.72913	0.99998	1.0509	0.071569	0.62481	0.99941	1.0865	0.11971	0.21613	0.65907	1.1928	0.25429	0.18436	0.46608	1.2396	0.30991	0.015067	0.091801	1.518	0.60215	0.011504	0.25884	1.5545	0.63648	0.35411	0.99956	1.0985	0.13553	0.40847	0.68089	1.1308	0.17729
378.9013	10.1	olomics Pos	0.88828	0.99987	0.98775	-0.01779	0.33011	0.99994	0.92917	-0.10598	0.24525	0.99996	0.8977	-0.1557	0.097876	0.51762	0.84011	-0.25135	0.005086	0.018453	0.76609	-0.38442	0.1147	0.36476	0.83408	-0.26175	0.93095	0.98468	1.0091	0.013024	0.43711	0.96776	0.94263	-0.08523	0.37396	0.48304	1.1141	0.15583
558.8584	10.05	olomics Pos	0.88847	0.99987	0.99036	-0.01398	0.99875	0.99994	1.0016	0.002281	0.97636	0.99996	1.0007	0.001017	0.29313	0.75244	0.93939	-0.0902	0.008934	0.028152	0.78745	-0.34474	0.18276	0.47109	0.88975	-0.16853	0.89376	0.97532	0.99249	-0.01087	0.93637	0.98616	1.0027	0.003921	0.014719	0.098791	1.2305	0.29927
596.2657	1.3	olomics Pos	0.88866	0.99987	1.0142	0.020369	0.31738	0.99994	0.88789	-0.17154	0.18421	0.99996	0.84803	-0.23782	0.46182	0.87979	0.90174	-0.14921	0.018812	0.049779	0.69717	-0.52041	0.96871	0.98315	1.0086	0.012397	0.71896	0.92152	1.0255	0.036367	0.82482	0.97988	1.0292	0.041506	0.12057	0.22484	1.2649	0.33897
560.2984	5.11	olomics Pc	0.88881	0.99951	1.0145	0.02072	0.76534	0.99998	1.0352	0.049877	0.66596	0.99941	1.1125	0.15384	0.16103	0.56677	1.4672	0.5531	0.10169	0.29001	1.6436	0.71689	0.013209	0.085844	1.8818	0.91215	0.014469	0.28593	1.9394	0.95562	0.19299	0.99297	1.3075	0.38686	0.11221	0.36506	1.4561	0.5421
148.9339	12.15	olomics Ne	0.88903	0.99918	0.99405	-0.00861	0.021877	0.40327	0.90028	-0.15156	0.46557	0.99995	0.96867	-0.04593	0.17844	0.99985	0.92101	-0.11871	0.92893	0.99927	1.0089	0.012734	0.86408	0.9762	1.015	0.021418	0.58915	0.86041	0.97657	-0.0342	0.28285	0.99512	0.94968	-0.07448	0.69789	0.99941	0.97724	-0.03322
184.9862	10.18	olomics Pos	0.88968	0.99987	0.99157	-0.01221	0.1924	0.99994	0.91324	-0.13093	0.43415	0.99996	0.95609	-0.06479	0.14973	0.57784	0.91185	-0.13314	0.001127	0.005987	0.76858	-0.37974	0.27319	0.56004	0.89859	-0.15426	0.53071	0.84189	0.96199	-0.05591	0.51248	0.96776	0.96717	-0.04816	0.038862	0.13241	1.1466	0.19737
621.2796	5.46	olomics Pc	0.88977	0.99951	1.0198	0.028261	0.81483	0.99998	0.91148	-0.13372	0.92076	0.99941	0.96708	-0.0483	0.60211	0.99905	1.1775	0.23569	0.55376	0.93933	1.1579	0.21149	0.039689	0.15926	1.6579	0.72939	0.090883	0.60607	1.5003	0.58525	0.39301	0.99956	1.2229	0.29028	0.11346	0.36506	1.6308	0.70559
427.3586	5.1	olomics Pc	0.89004	0.99951	0.84814	-0.23763	0.52119	0.99998	0.72078	-0.47237	0.4561	0.99941	0.70108	-0.51235	0.35604	0.84597	0.65665	-0.6068	0.13213	0.36098	0.49061	-1.0274	0.033598	0.14225	0.33302	-1.5863	0.066162	0.52224	0.39654	-1.3344	0.13029	0.90999	0.47673	-1.0688	0.053061	0.2951	0.4017	-1.3158
575.5029	5.06	olomics Pc	0.89036	0.99987	0.94472	-0.08204	0.41289	0.99994	0.81105	-0.30214	0.48388	0.99996	0.83219	-0.26502	0.43344	0.86439	0.81336	-0.29804	0.56735	0.6307	0.86709	-0.20574	0.12428	0.38426	1.4921	0.57734	0.042947	0.38531	2.1215	1.0851	0.088785	0.82455	1.9849	0.98909	0.009025	0.092545	2.6611	1.412
677.2155	1.11	olomics Pos	0.8905	0.99987	1.0735	0.10233	0.78338	0.99994	1.0528	0.074272	0.62328	0.99996	1.0596	0.083495	0.8655	0.9874	1.0037	0.005277	0.9054	0.9189	0.97799	-0.03211	0.067157	0.26356	1.3009	0.37947	0.018215	0.33809	1.5605	0.64197	0.31488	0.9524	1.214	0.27982	0.029408	0.11849	1.4692	0.55505
762.8221	0.54	olomics Pos	0.89051	0.99987	1.0108	0.015473	0.5157	0.99994	0.94204	-0.08614	0.13371	0.99996	0.83888	-0.25346	0.069245	0.44879	0.85846	-0.22017	0.004748	0.017558	0.76032	-0.39532	0.2631	0.54979	0.84079	-0.25018	0.17775	0.64616	0.88942	-0.16907	0.91493	0.98252	0.99396	-0.00874	0.028862	0.11762	1.2342	0.30355
382.2724	4.87	olomics Pc	0.89057	0.99951	1.1408	0.19004	0.84566	0.99998	1.1935	0.25523	0.99578	0.99941	1.1276	0.17322	0.95941	0.99905	1.1116	0.15265	0.009498	0.046604	0.28827	-1.7945	0.47258	0.7756	0.75979	-0.39633	0.78279	0.99391	0.89264	-0.16385	0.68776	0.99956	0.87486	-0.19288	0.78018	0.90352	0.89271	-0.16374
1083.659	5.1	olomics Ne	0.89059	0.99918	1.0208	0.029756	0.98961	0.99664	0.97347	-0.0388	0.75568	0.99995	1.095	0.13096	0.57172	0.99985	1.2064	0.27065	0.25206	0.99927	1.9097	0.93336	0.024104	0.43917	3.4299	1.7782	0.012889	0.2716	3.606	1.8504	0.15212	0.99512	1.5796	0.65916	0.16196	0.99941	1.762	0.81723
261.2214	6.07	olomics Pc	0.89065	0.99951	1.037	0.052393	0.55817	0.99998	0.93403	-0.09845	0.015822	0.99941	0.54129	-0.88553	0.021424	0.22258	0.4097	-1.2874	0.002077	0.015883	0.38626	-1.3724	0.01095	0.083112	0.37718	-1.4067	0.003349	0.20152	0.3271	-1.6122	0.005471	0.47452	0.28393	-1.8164	0.029855	0.2688	0.38562	-1.3748
540.3062	0.95	olomics Pos	0.89107	0.99987	1.0866	0.11984	0.89316	0.99994	1.0316	0.044924	0.65437	0.99996	1.1774	0.23556	0.83518	0.98621	1.0466	0.065664	0.94363	0.95187	0.93486	-0.09718	0.056474	0.23585	2.3515	1.2336	0.019561	0.33809	3.0166	1.5929	0.16673	0.91806	2.0323	1.0231	0.034482	0.12629	3.1893	1.6732
627.4338	2.28	olomics Pos	0.89127	0.99987	1.0444	0.062631	0.47788	0.99994	0.9033	-0.14673	0.039121	0.99996	0.67554	-0.56589	0.025778	0.28326	0.46943	-1.091	0.000527	0.003437	0.42378	-1.2386	0.010043	0.08787	0.40976	-1.2872	0.030696	0.35576	0.50279	-0.99198	0.017072	0.52955	0.40395	-1.3077	0.059039	0.15646	0.57947	-0.78718
768.5554	5.47	olomics Pos	0.89127	0.99987	1.0061	0.008721	0.17462	0.99994	0.90325	-0.1468	0.17436	0.99996	0.8866	-0.17364	0.069922	0.45112	0.84895	-0.23625	0.001159	0.006114	0.66413	-0.59045	0.033621	0.16489	0.79401	-0.33276	0.95373	0.98985	0.99606	-0.00569	0.86297	0.98023	1.0354	0.050216	0.62561	0.71786	1.0679	0.094842
897.693	7.17	olomics Pos	0.89193	0.99987	0.91822	-0.12309	0.83296	0.99994	0.88295	-0.17959	0.53072	0.99996	0.78112	-0.35639	0.18616	0.62584	0.5904	-0.76024	0.15902	0.24749	0.55082	-0.86035	0.55393	0.75434	0.78714	-0.3453	0.56425	0.8563	0.78307	-0.35278	0.025191	0.56108	0.41011	-1.2859	0.47452	0.5777	0.75425	-0.40658
484.7744	0.59	olomics Pos	0.89262	0.99987	0.99192	-0.01171	0.32765	0.99994	0.89241	-0.16422	0.32724	0.99996	0.90954	-0.13679	0.10759	0.53121	0.76505	-0.38637	0.012225	0.035664	0.72099	-0.47194	0.053398	0.22749	0.79327	-0.33412	0.55221	0.85184	0.92633	-0.1104	0.74139	0.97988	1.0518	0.072889	0.43169	0.53792	1.0791	0.10987
462.296	5.44	olomics Pc	0.89264	0.99951	0.9983	-0.00245	0.90631	0.99998	1.0135	0.019349	0.64738	0.99941	1.0972	0.13383	0.11927	0.47414	1.7855	0.83633	0.018258	0.078354	2.8289	1.5002	0.000339	0.039399	4.6013	2.202	0.001831	0.16989	4.2729	2.0952	0.10642	0.85295	2.33	1.2203	0.017849	0.2688	2.5522	1.3517
842.5693	5.01	olomics Pos	0.89271	0.99987	0.96567	-0.0504	0.58245	0.99994	0.90957	-0.13674	0.7061	0.99996	0.93167	-0.10211	0.81963	0.98371	0.95592	-0.06503	0.043004	0.093061	0.70647	-0.5013	0.079236	0.29123	0.70407	-0.5062	0.045731	0.39255	0.71469	-0.48462	0.17109	0.91806	0.78192	-0.35492	0.76513	0.82437	0.94886	-0.07574
808.593	10.2	olomics Ne	0.8929	0.99918	1.005	0.007152	0.025047	0.40327	0.87162	-0.19823	0.97755	0.99995	0.99871	-0.00187	0.69603	0.99985	0.97831	-0.03163	0.97221	0.99927	1.0016	0.00225	0.45751	0.8258	1.098	0.13484	0.14591	0.53372	1.0804	0.11162	0.90412	0.99512	1.0045	0.00649	0.92978	0.99941	1.0061	0.008799
279.2323	6.06	olomics Pc	0.89306	0.99951	1.0178	0.02542	0.5608	0.99998	0.92294	-0.11569	0.025856	0.99941	0.57786	-0.79122	0.022885	0.22443	0.40652	-1.2986	0.001537	0.012837	0.39625	-1.3355	0.010008	0.079508	0.39013	-1.358	0.004489	0.20152	0.33859	-1.5624	0.009547	0.47452	0.30209	-1.727	0.024702	0.2688	0.3767	-1.4085
303.2325	5.24	olomics Pc	0.89374	0.99951	0.93517	-0.0967	0.64399	0.99998	0.71261	-0.48881	0.59319	0.99941	0.70249	-0.50944	0.8555	0.99905	0.86716	-0.20563	0.00094	0.008918	0.030349	-5.0422	0.21886	0.50044	0.47487	-1.0744	0.36024	0.96545	0.52685	-0.92453	0.77379	0.99956	0.69102	-0.53321	0.91707	0.96508	0.89958	-0.15267
829.8127	10.03	olomics Pos	0.89384	0.99987	1.0094	0.013543	0.35454	0.99994	0.94336	-0.08412	0.17423	0.99996	0.91047	-0.13531	0.23161	0.68957	0.93653	-0.0946	0.000112	0.001118	0.74317	-0.42823	0.18523	0.4723	0.87336	-0.19535	0.8029	0.95444	1.0334	0.047389	0.40441	0.96776	0.92831	-0.10731	0.027573	0.11643	1.1621	0.21676
139.9888	10.18	olomics Pos	0.89388	0.99987	1.0039	0.00562	0.077255	0.99994	0.9068	-0.14115	0.30659	0.99996	0.95219	-0.07068	0.088593	0.49659	0.91343	-0.13064	0.000228	0.001839	0.75023	-0.41459	0.24458	0.53144	0.90091	-0.15054	0.95663	0.98985	0.99743	-0.00372	0.50408	0.96776	0.97255	-0.04016	0.021929	0.10841	1.156	0.20917
740.5276	8.51	olomics Pc	0.89408	0.99951	0.99056	-0.01368	0.76828	0.99998	0.88025	-0.18401	0.8623	0.99941	0.87745	-0.1886	0.66323	0.99905	1.3329	0.41456	0.008493	0.042466	3.726	1.8976	0.006707	0.067928	3.7717	1.9152	0.01588	0.29145	3.1933	1.6751	0.31754	0.99956	2.1832	1.1265	0.096145	0.34969	2.182	1.1257
229.1398	0.34	olomics Pc	0.89417	0.99951	0.99489	-0.0074	0.88918	0.99998	1.0244	0.034814	0.72094	0.99941	1.0893	0.12338	0.76555	0.99905	0.96781	-0.0472	0.10372	0.29487	0.71946	-0.47502	0.006037	0.065713	0.58239	-0.77995	0.27877	0.89698	0.8544	-0.22701	0.40314	0.99956	0.88535	-0.17568	0.13524	0.38976	0.76848	-0.37993
122.9735	12.3	olomics Ne	0.89425	0.99918	0.9918	-0.01188	0.006133	0.34605	0.81239	-0.29975	0.94145	0.99995	1.0034	0.004831	0.91303	0.99985	1.013	0.018565	0.71303	0.99927	1.0253	0.036061	0.25564	0.69038	1.1064	0.1459	0.73725	0.92171	1.0181	0.025915	0.73146	0.99512	0.97825	-0.03172	0.627	0.99941	0.96979	-0.04425
822.5748	10.13	olomics Ne	0.8944	0.99918	1.017	0.024297	0.6643	0.92948	0.95525	-0.06604	0.57564	0.99995	1.0792	0.10997	0.61815	0.99985	1.0427	0.06037	0.48563	0.99927	1.098	0.13487	0.18401	0.65203	1.1811	0.24014	0.03962	0.364	1.3521	0.43519	0.28418	0.99512	1.1082	0.14826	0.16459	0.99941	1.2266	0.29472
350.8569	12.73	olomics Ne	0.89462	0.99918	0.99232	-0.01112	0.44602	0.82137	0.9532	-0.06914	0.21423	0.99995	1.0756	0.1052	0.58991	0.99985	1.0291	0.041369	0.61632	0.99927	0.96878	-0.04576	0.02309	0.43126	1.1493	0.20071	0.054715	0.39335	1.1425	0.19219	0.15228	0.99512	1.094	0.12964	0.061472	0.99941	1.1007	0.13845
576.5073	5.06	olomics Pos	0.89463	0.99987	0.95953	-0.0596	0.099351	0.99994	0.67397	-0.56923	0.51709	0.99996	0.85468	-0.22654	0.47883	0.88482	0.84292	-0.24653	0.52283	0.59291	0.85911	-0.21909	0.18663	0.47264	1.3979	0.48323	0.043376	0.38825	1.88	0.91075	0.058424	0.72218	1.966	0.97529	0.012818	0.098791	2.2803	1.1893
211.1682	6.48	olomics Pc	0.89566	0.99951	1.0084	0.012087	0.5823	0.99998	0.9426	-0.08529	0.17992	0.99941	0.73429	-0.44557	0.090175	0.40258	0.63317	-0.65933	0.008717	0.043376	0.49403	-1.0173	0.011301	0.083566	0.43465	-1.2021	0.02839	0.3417	0.44837	-1.1572	0.012987	0.47452	0.42283	-1.2418	0.037147	0.27927	0.42382	-1.2385
425.3455	4.87	olomics Pc	0.89575	0.99951	0.91972	-0.12074	0.76733	0.99998	0.89236	-0.1643	0.64801	0.99941	0.85951	-0.21841	0.47888	0.95654	0.8074	-0.30865	0.058754	0.18924	0.50022	-0.99936	0.039566	0.15926	0.46136	-1.116	0.29959	0.91978	0.7089	-0.49634	0.088432	0.80182	0.54311	-0.8807	0.01796	0.2688	0.45233	-1.1446
436.2829	1.35	olomics Pos	0.89638	0.99987	0.91357	-0.13041	0.87518	0.99994	0.90789	-0.13941	0.8364	0.99996	0.88251	-0.18031	0.99914	0.99936	0.92383	-0.1143	0.65991	0.711	0.8176	-0.29053	0.18039	0.46822	1.4637	0.54962	0.031334	0.35841	2.048	1.0342	0.16082	0.91806	1.4541	0.54011	0.064071	0.16235	1.7713	0.82479
568.34	1.03	olomics Pos	0.89642	0.99987	1.0022	0.003195	0.88175	0.99994	1.013	0.018582	0.62691	0.99996	1.0814	0.11293	0.78581	0.97526	1.0257	0.036641	0.37987	0.47165	0.83187	-0.26557	0.073034	0.27848	1.4474	0.53344	0.02059	0.33809	1.7817	0.83327	0.58734	0.97073	1.0928	0.12797	0.074959	0.17705	1.4107	0.49642
326.342	4	olomics Pos	0.89649	0.99987	0.98684	-0.01911	0.27092	0.99994	0.87712	-0.18915	0.65953	0.99996	0.95354	-0.06864	0.68999	0.96092	0.96001	-0.05888	0.048745	0.10236	0.8058	-0.31151	0.002573	0.061655	0.61581	-0.69945	0.37107	0.76056	0.86799	-0.20426	0.11402	0.88445	0.85147	-0.23198	0.75819	0.81897	0.96938	-0.04487
196.8277	0.42	olomics Ne	0.89684	0.99918	0.99178	-0.01192	0.49501	0.85324	0.93841	-0.09171	0.78157	0.99995	0.98024	-0.02879	0.10277	0.99985	1.143	0.19281	0.48956	0.99927	1.0768	0.10671	0.3522	0.75906	1.1226	0.16691	0.021358	0.30572	1.2129	0.27848	0.69995	0.99512	0.97354	-0.03868	0.37422	0.99941	1.0462	0.065207
472.9027	10.03	olomics Pos	0.89699	0.99987	0.99229	-0.01116	0.42063	0.99994	0.93065	-0.10369	0.86681	0.99996	0.99016	-0.01426	0.5104	0.89396	0.94642	-0.07945	0.000573	0.003658	0.68433	-0.54723	0.37722	0.63447	0.932	-0.10159	0.55321	0.85214	1.0464	0.065374	0.5083	0.96776	0.95441	-0.06731	0.051797	0.14847	1.1777	0.236
460.2825	5.09	olomics Pc	0.89741	0.99951	1.0205	0.029269	0.98587	0.99998	0.94952	-0.07472	0.82398	0.99941	1.0432	0.060953	0.34759	0.8414	1.5402	0.62308	0.053239	0.17579	2.2144	1.1469	0.009875	0.079508	3.1966	1.6766	0.00371	0.20152	3.7323	1.9	0.16367	0.94744	1.8713	0.904	0.12822	0.3787	1.893	0.92066
545.341	1.38	olomics Pos	0.89786	0.99987	1.0295	0.041959	0.93864	0.99994	1.0007	0.000942	0.89204	0.99996	1.0107	0.015346	0.94921	0.99328	0.95918	-0.06013	0.90582	0.919	0.93194	-0.10169	0.15278	0.43045	1.4854	0.57087	0.049551	0.40631	1.8209	0.86468	0.48187	0.96776	1.2042	0.26812	0.08854	0.18956	2.0233	1.0167
715.574	4.5	olomics Pos	0.89787	0.99987	0.99706	-0.00425	0.91035	0.99994	1.0008	0.001203	0.97361	0.99996	1.0014	0.001953	0.27788	0.74448	0.92411	-0.11387	0.049671	0.10378	0.80698	-0.30939	0.34015	0.61059	0.9181	-0.12328	0.63018	0.88147	0.98076	-0.02802	0.296	0.9483	0.88848	-0.17059	0.50133	0.60301	1.0554	0.077752
341.2932	2.98	olomics Pos	0.8981	0.99987	0.95748	-0.06269	0.7314	0.99994	1.1041	0.14293	0.93521	0.99996	0.96957	-0.04459	0.7964	0.9771	0.92844	-0.10712	0.019343	0.050865	0.46414	-1.1074	0.38334	0.63866	0.82655	-0.27483	0.13946	0.60403	1.3237	0.40455	0.12439	0.88537	1.3358	0.4177	0.03897	0.13241	1.5178	0.60196
727.5743	4.36	olomics Pos	0.89895	0.99987	1.0178	0.025505	0.91882	0.99994	1.0074	0.010633	0.95768	0.99996	0.99841	-0.0023	0.45401	0.87408	0.92053	-0.11946	0.040171	0.088434	0.7574	-0.40088	0.70463	0.84729	0.96261	-0.05498	0.71476	0.91919	0.97081	-0.04274	0.43963	0.96776	0.89475	-0.16044	0.049828	0.14647	0.7563	-0.40297
652.9742	12.01	olomics Ne	0.89918	0.99918	0.99044	-0.01386	0.15175	0.58235	0.88958	-0.16881	0.03955	0.99995	0.80574	-0.31162	0.94988	0.99985	1.0058	0.008275	0.58625	0.99927	1.0551	0.077391	0.29673	0.71853	0.91971	-0.12074	0.939	0.98374	1.0084	0.012056	0.087478	0.99512	0.81028	-0.30351	0.30192	0.99941	0.91321	-0.13098
245.2268	2.28	olomics Pos	0.89925	0.99987	1.003	0.004355	0.41255	0.99994	0.8553	-0.22549	0.0622	0.99996	0.64557	-0.63135	0.009151	0.2002	0.44817	-1.1579	0.000183	0.001581	0.33728	-1.568	0.0044	0.071311	0.31222	-1.6794	0.010188	0.3324	0.38515	-1.3765	0.012992	0.52955	0.36574	-1.4511	0.039558	0.13336	0.48357	-1.0482
836.588	10.24	olomics Ne	0.8995	0.99918	1.0032	0.004675	0.028063	0.41126	0.8558	-0.22466	0.9097	0.99995	1.0036	0.005185	0.7767	0.99985	0.98003	-0.0291	0.44477	0.99927	1.0647	0.090405	0.65268	0.92563	1.0498	0.070175	0.86751	0.95742	0.99017	-0.01426	0.92899	0.99512	0.9935	-0.0094	0.65835	0.99941	0.97487	-0.03672
104.1076	0.31	olomics Pc	0.8996	0.99951	1.0233	0.033165	0.7509	0.99998	0.95577	-0.06527	0.19742	0.99941	0.83132	-0.26652	0.51081	0.98198	0.91292	-0.13144	0.000791	0.00781	0.50864	-0.97528	0.28371	0.57807	0.84321	-0.24603	0.43894	0.99391	1.0856	0.11843	0.67873	0.99956	0.94362	-0.08372	0.54306	0.77827	1.1613	0.21571
367.1954	4.77	olomics Pos	0.90024	0.99987	0.98113	-0.02748	0.59009	0.99994	0.92999	-0.10471	0.91486	0.99996	0.98796	-0.01748	0.71328	0.96346	0.95427	-0.06753	0.017143	0.04626	0.71422	-0.48556	0.28045	0.56697	0.82916	-0.27027	0.30004	0.72384	0.87754	-0.18846	0.57879	0.9703	0.92733	-0.10884	0.72186	0.79282	1.0438	0.061903
825.8185	10.03	olomics Pos	0.90031	0.99987	0.97507	-0.03643	0.25926	0.99994	0.87124	-0.19885	0.12485	0.99996	0.82143	-0.28378	0.79202	0.97671	0.96275	-0.05477	0.020102	0.052534	0.66874	-0.58049	0.59671	0.78134	0.92988	-0.10488	0.90981	0.97939	1.0089	0.012768	0.96406	0.98873	1.0015	0.002113	0.5309	0.62935	1.1088	0.149
198.8063	0.39	olomics Ne	0.90133	0.99918	1.0109	0.01559	0.023526	0.40327	0.92964	-0.10526	0.2057	0.99995	1.0701	0.097811	0.11593	0.99985	0.9327	-0.10052	0.33857	0.99927	1.1176	0.1604	0.008117	0.36417	1.0993	0.13653	0.59883	0.8642	1.041	0.057922	0.90346	0.99512	0.99817	-0.00264	0.6503	0.99941	0.97878	-0.03094
942.0692	6.47	olomics Pc	0.90239	0.99951	1.0741	0.10318	0.30614	0.99998	0.79078	-0.33865	0.66592	0.99941	1.059	0.082767	0.04783	0.28918	0.64137	-0.64077	0.21115	0.51521	0.72572	-0.46252	0.094898	0.28786	0.67648	-0.56388	0.72242	0.99391	0.92308	-0.11548	0.59103	0.99956	0.92253	-0.11633	0.97631	0.98887	0.9916	-0.01216
447.3353	8.37	olomics Pc	0.9025	0.99951	0.99236	-0.01107	0.82175	0.99998	0.98567	-0.02083	0.34379	0.99941	0.93848	-0.0916	0.18317	0.61171	0.9185	-0.12265	0.63234	0.98121	1.0252	0.035913	0.55497	0.83805	0.9585	-0.06115	0.83164	0.9951	0.98643	-0.01971	0.04903	0.67731	0.85556	-0.22506	0.061851	0.30404	0.84816	-0.2376
362.1812	6.42	olomics Pc	0.90297	0.99951	0.94378	-0.08348	0.94016	0.99998	0.95306	-0.06936	0.20845	0.99941	0.72996	-0.45411	0.34271	0.83613	0.79846	-0.32472	0.000408	0.00489	0.26172	-1.9339	0.03488	0.14572	0.58091	-0.78362	0.13689	0.68991	0.67577	-0.56539	0.23063	0.99956	0.71449	-0.48502	0.12013	0.36992	0.67909	-0.55832
604.7957	2.36	olomics Pc	0.90319	0.99951	0.97538	-0.03596	0.63996	0.99998	0.81459	-0.29586	0.47464	0.99941	0.71762	-0.47871	0.24733	0.71367	0.56075	-0.83457	0.00747	0.038635	0.1522	-2.716	0.11112	0.31996	0.36876	-1.4393	0.34141	0.95245	0.6311	-0.66406	0.49757	0.99956	0.7845	-0.35015	0.74031	0.88905	1.07	0.09761
299.1999	2.09	olomics Pos	0.90341	0.99987	1.0855	0.11831	0.55815	0.99994	0.91004	-0.136	0.12212	0.99996	0.69698	-0.52081	0.025433	0.28191	0.50196	-0.99435	0.001227	0.006385	0.33223	-1.5897	0.005047	0.073452	0.30441	-1.7159	0.006293	0.3088	0.40989	-1.2867	0.044667	0.65325	0.49777	-1.0065	0.12633	0.23091	0.64265	-0.63788
228.9567	0.51	olomics Pos	0.9046	0.99987	1.004	0.005734	0.23759	0.99994	0.93764	-0.0929	0.076927	0.99996	0.90133	-0.14988	0.010853	0.21362	0.87558	-0.1917	2.77E-06	0.000144	0.74585	-0.42304	0.19626	0.48406	0.8518	-0.23142	0.13496	0.60006	0.91865	-0.12242	0.003214	0.52955	0.86948	-0.20178	0.038506	0.13216	1.1801	0.23887
304.2138	6.41	olomics Ne	0.90468	0.99918	0.97001	-0.04393	0.7941	0.97675	0.93614	-0.09521	0.39834	0.99995	0.84854	-0.23694	0.18358	0.99985	0.76467	-0.3871	0.000108	0.023712	0.1984	-2.3335	0.003736	0.36417	0.48429	-1.0461	0.082192	0.43873	0.65658	-0.60696	0.070418	0.99512	0.6411	-0.64137	0.18135	0.99941	0.72007	-0.4738
753.5602	6.02	olomics Pos	0.90471	0.99987	0.97685	-0.03379	0.60288	0.99994	0.8922	-0.16456	0.67607	0.99996	0.90982	-0.13635	0.29117	0.75244	0.77154	-0.37418	0.74555	0.78357	0.92583	-0.11119	0.56296	0.76119	1.0772	0.1073	0.042008	0.38314	1.5111	0.59563	0.19056	0.92582	1.293	0.37073	0.26423	0.37425	1.233	0.30223
880.8065	0.5	olomics Pos	0.9051	0.99987	0.9924	-0.011	0.41093	0.99994	0.92606	-0.11082	0.2207	0.99996	0.88054	-0.18355	0.12453	0.54705	0.88799	-0.17139	0.005267	0.018983	0.70212	-0.5102	0.26754	0.55532	0.88029	-0.18394	0.606	0.86762	0.95627	-0.06451	0.24192	0.94365	0.90609	-0.14227	0.20746	0.31631	1.1434	0.19332
500.2779	4.81	olomics Pc	0.90525	0.99951	1.001	0.001438	0.88117	0.99998	1.0159	0.022756	0.77738	0.99941	1.0782	0.10864	0.52335	0.98929	1.1226	0.16681	0.037776	0.13615	1.4515	0.53759	0.015603	0.093261	1.9094	0.93311	0.006282	0.21836	1.8689	0.90216	0.28453	0.99956	1.2537	0.32619	0.69398	0.86166	1.1367	0.18491
380.2622	2.73	olomics Pos	0.90564	0.99987	0.99238	-0.01103	0.26697	0.99994	0.84138	-0.24916	0.018105	0.99996	0.71906	-0.47582	0.003679	0.17581	0.53262	-0.90883	0.000297	0.002206	0.38401	-1.3808	0.004336	0.071311	0.37874	-1.4007	0.006722	0.3088	0.45188	-1.146	0.009857	0.52955	0.46313	-1.1105	0.031601	0.12169	0.61248	-0.70726
560.296	1.1	olomics Pos	0.90705	0.99987	1.0584	0.081889	0.64045	0.99994	1.0876	0.12118	0.79006	0.99996	1.016	0.022905	0.47137	0.88476	1.1955	0.25761	0.20674	0.30054	1.6414	0.71496	0.033173	0.16385	2.2104	1.1443	0.012209	0.3324	2.6825	1.4236	0.12596	0.88537	1.6336	0.70806	0.096702	0.19779	1.7957	0.84456
607.2517	6.97	olomics Pc	0.90719	0.99951	1.0924	0.12755	0.93896	0.99998	1.0561	0.078725	0.22569	0.99941	0.80582	-0.31147	0.044075	0.28334	0.65308	-0.61466	0.007292	0.037905	0.47396	-1.0772	0.002674	0.046658	0.39063	-1.3561	0.011386	0.25802	0.45612	-1.1325	0.032922	0.58922	0.47516	-1.0735	0.058172	0.30327	0.54891	-0.86536
151.9225	12.72	olomics Ne	0.90724	0.99918	1.0083	0.011987	0.075319	0.50118	0.8887	-0.17024	0.58415	0.99995	1.035	0.049602	0.82642	0.99985	0.98949	-0.01524	0.87643	0.99927	1.0042	0.006043	0.004899	0.36417	1.1441	0.1942	0.042869	0.37694	1.1093	0.14959	0.6516	0.99512	1.024	0.034196	0.66943	0.99941	0.98346	-0.02407
544.3377	5.45	olomics Pos	0.90727	0.99987	1.0211	0.030146	0.98085	0.99994	1.0197	0.028098	0.80128	0.99996	1.0594	0.083281	0.88497	0.99905	1.0036	0.005235	0.6725	0.99535	1.1392	0.18805	0.065441	0.22274	1.8768	0.9083	0.045509	0.42792	1.969	0.97749	0.39158	0.99956	1.3448	0.42739	0.14215	0.39464	1.8519	0.88902
736.8425	10.03	olomics Pos	0.90731	0.99987	1.0097	0.013893	0.3121	0.99994	0.92898	-0.10628	0.083142	0.99996	0.89759	-0.15587	0.031123	0.30745	0.84515	-0.24272	0.013686	0.03903	0.77424	-0.36915	0.077773	0.28838	0.80048	-0.32107	0.90795	0.97907	1.0189	0.027007	0.25523	0.94365	0.91107	-0.13436	0.079124	0.18102	1.1394	0.18832
303.2283	2.17	lomics Neg	0.90754	0.99977	0.99003	-0.01445	0.6431	0.99904	0.91664	-0.12558	0.18782	0.99994	0.76863	-0.37963	0.39372	0.58563	0.8646	-0.2099	0.000499	0.040886	0.15276	-2.7107	0.051721	0.85314	0.48563	-1.0421	0.022259	0.30828	0.53306	-0.90763	0.07557	0.47593	0.45652	-1.1312	0.082103	0.405	0.64842	-0.625
1121.608	1.11	olomics Pos	0.90758	0.99987	0.75597	-0.4036	0.90919	0.99994	0.86751	-0.20504	0.94224	0.99996	0.82992	-0.26896	0.81947	0.98371	0.8782	-0.18738	0.60489	0.66325	1.171	0.22769	0.057681	0.23979	2.7668	1.4682	0.034583	0.36356	3.2245	1.6891	0.31876	0.9524	1.7021	0.76734	0.20322	0.31196	1.6679	0.73806
818.0111	6.48	olomics Pc	0.90789	0.99951	1.0136	0.019453	0.046911	0.99998	0.69939	-0.51582	0.69389	0.99941	0.94617	-0.07983	0.011394	0.19869	0.65923	-0.60114	0.21369	0.51939	0.78185	-0.35504	0.10947	0.31845	0.77005	-0.37698	0.55091	0.99391	0.9085	-0.13844	0.58973	0.99956	0.92588	-0.11111	0.91947	0.96508	1.0201	0.028707
956.7709	0.53	olomics Pos	0.90796	0.99987	1.021	0.029977	0.12089	0.99994	0.87287	-0.19616	0.28692	0.99996	0.93827	-0.09192	0.026503	0.28873	0.86063	-0.21654	0.004773	0.017627	0.73439	-0.44539	0.45953	0.68833	0.94644	-0.07941	0.85496	0.97109	0.98754	-0.0181	0.21559	0.93732	0.88661	-0.17363	0.18641	0.29407	1.2117	0.27706
1003.544	6.34	olomics Pos	0.90839	0.99987	1.0138	0.019717	0.39388	0.99994	0.93528	-0.09653	0.5621	0.99996	0.9395	-0.09003	0.36088	0.8151	0.9013	-0.14992	0.028575	0.068355	0.72253	-0.46887	0.001856	0.057105	0.64792	-0.62611	0.050749	0.41162	0.72848	-0.45703	0.054026	0.69594	0.69743	-0.51987	0.62683	0.71862	0.95351	-0.06868
1025.531	5.94	olomics Pos	0.9088	0.99987	1.0062	0.00893	0.17595	0.99994	0.84116	-0.24955	0.84834	0.99996	0.97414	-0.0378	0.23641	0.69488	0.83417	-0.26159	0.025623	0.062911	0.69507	-0.52477	0.013938	0.099586	0.7075	-0.4992	0.034267	0.36356	0.73381	-0.44652	0.052701	0.68583	0.75326	-0.40878	0.79398	0.84708	1.057	0.079912
882.8373	10.05	olomics Pos	0.90884	0.99987	1.0119	0.017071	0.60858	0.99994	0.93849	-0.09159	0.64166	0.99996	0.94052	-0.08847	0.075305	0.46303	0.78559	-0.34816	0.012755	0.036871	0.72015	-0.47362	0.15514	0.43422	0.8287	-0.27108	0.59392	0.86436	1.062	0.08682	0.58612	0.97073	0.93398	-0.09854	0.084875	0.18602	1.2103	0.2754
562.3012	1.12	olomics Pos	0.90892	0.99987	0.94041	-0.08864	0.78665	0.99994	0.89049	-0.16732	0.97883	0.99996	0.98875	-0.01632	0.92809	0.9874	0.93907	-0.09069	0.84956	0.87268	0.93358	-0.09915	0.093989	0.32411	1.4519	0.53791	0.040566	0.37935	1.7058	0.77045	0.26848	0.94365	1.2822	0.35866	0.079493	0.18125	1.5594	0.641
356.2882	6.16	olomics Pc	0.90933	0.99951	0.99918	-0.00119	0.19861	0.99998	1.0424	0.059884	0.18582	0.99941	0.86619	-0.20724	0.040929	0.27667	0.95651	-0.06414	0.18117	0.46218	1.0643	0.08986	0.35226	0.66207	0.94475	-0.082	0.62519	0.99391	0.96583	-0.05016	0.13658	0.92085	0.87622	-0.19064	0.53813	0.77615	1.0394	0.055693
615.3384	5.21	olomics Ne	0.91001	0.99918	1.0203	0.029009	0.8507	0.98874	1.0203	0.028997	0.50214	0.99995	1.1342	0.18166	0.52528	0.99985	1.0966	0.13302	0.56071	0.99927	1.0811	0.11251	0.039175	0.48405	1.6548	0.72668	0.023912	0.33074	1.6007	0.67868	0.54241	0.99512	1.093	0.12824	0.26426	0.99941	1.1677	0.22371
1039.67	1.11	olomics Pos	0.91028	0.99987	0.80211	-0.31812	0.90667	0.99994	0.92293	-0.11571	0.8371	0.99996	0.9469	-0.07871	0.66116	0.95717	1.082	0.1137	0.37321	0.46566	1.7229	0.78481	0.028346	0.14826	5.0749	2.3434	0.011058	0.3324	6.6518	2.7337	0.22692	0.94286	2.0775	1.0549	0.10425	0.20689	2.5341	1.3415
523.3481	5.1	olomics Pc	0.9107	0.99951	0.93116	-0.1029	0.89807	0.99998	0.98071	-0.0281	0.86654	0.99941	1.0022	0.003184	0.54778	0.99905	1.2067	0.27109	0.22497	0.54095	1.6316	0.70628	0.02691	0.12339	2.3905	1.2573	0.01739	0.29966	2.5857	1.3706	0.25555	0.99956	1.4614	0.54733	0.15578	0.41304	1.5744	0.65483
464.3138	1.63	olomics Pos	0.91079	0.99987	0.99216	-0.01136	0.52333	0.99994	0.87036	-0.20031	0.46889	0.99996	0.85136	-0.23215	0.67198	0.95717	0.9142	-0.12942	5.34E-05	0.000676	0.13668	-2.8711	0.1042	0.34339	0.65054	-0.62029	0.90875	0.97939	1.0164	0.023473	0.87271	0.98023	0.98625	-0.01998	0.14905	0.25416	1.3677	0.45176
912.4782	4.77	olomics Pos	0.91104	0.99987	1.0027	0.003914	0.48872	0.99994	0.91866	-0.1224	0.67142	0.99996	0.96055	-0.05806	0.61301	0.93755	0.94667	-0.07906	0.011328	0.033824	0.76098	-0.39407	0.25931	0.54596	0.80452	-0.31379	0.72954	0.92644	0.96815	-0.0467	0.010097	0.52955	0.76276	-0.39069	0.68982	0.76864	0.96191	-0.05602
445.3267	2.28	olomics Pos	0.9111	0.99987	1.0309	0.043945	0.41843	0.99994	0.87904	-0.18601	0.074041	0.99996	0.7372	-0.43988	0.017014	0.24438	0.55927	-0.8384	0.000834	0.004869	0.45237	-1.1444	0.015135	0.10398	0.45732	-1.1287	0.02178	0.33809	0.52671	-0.92491	0.007631	0.52955	0.45221	-1.1449	0.043039	0.13814	0.57803	-0.79079
118.1234	9.96	olomics Pos	0.91246	0.99987	1.0052	0.007465	0.00332	0.99994	0.89177	-0.16526	0.068229	0.99996	0.91324	-0.13093	0.013936	0.22898	0.90321	-0.14687	2.73E-05	0.000447	0.72577	-0.46241	0.13403	0.40042	0.86687	-0.20611	0.60276	0.8673	0.97048	-0.04323	0.10277	0.85564	0.92847	-0.10707	0.027831	0.11681	1.1209	0.16472
986.7652	10.09	olomics Pos	0.9126	0.99987	1.0093	0.013294	0.92942	0.99994	1.0037	0.005399	0.46742	0.99996	0.95625	-0.06454	0.016959	0.24438	0.83608	-0.25828	9.6E-05	0.000992	0.81394	-0.297	0.15357	0.43151	0.89201	-0.16487	0.048146	0.39996	1.1537	0.20625	0.14995	0.91321	0.9405	-0.0885	0.006236	0.090084	1.1877	0.24816
575.5031	8.85	olomics Pc	0.91262	0.99951	1.0257	0.036561	0.64509	0.99998	0.88664	-0.17357	0.72395	0.99941	0.90274	-0.14762	0.54674	0.99905	0.84564	-0.24189	0.32841	0.7071	1.205	0.26906	0.55272	0.83665	1.2552	0.32795	0.014993	0.28681	2.1564	1.1086	0.12098	0.8972	1.7959	0.8447	0.056804	0.30174	1.8469	0.88511
489.8766	10.06	olomics Pos	0.91273	0.99987	0.98269	-0.02519	0.27877	0.99994	0.88888	-0.16994	0.49446	0.99996	0.92935	-0.1057	0.18644	0.62584	0.86592	-0.2077	0.03906	0.086869	0.77522	-0.36733	0.055253	0.23213	0.78002	-0.35841	0.87082	0.97532	0.97699	-0.03358	0.47369	0.96776	0.91589	-0.12675	0.41326	0.52074	1.1056	0.14483
918.5751	10.47	olomics Ne	0.91273	0.99918	0.99744	-0.0037	0.071774	0.50118	0.88434	-0.17733	0.94423	0.99995	1.0027	0.003905	0.42282	0.99985	0.96203	-0.05585	0.13692	0.99927	0.89439	-0.16103	0.17777	0.65203	0.94253	-0.0854	0.011646	0.2716	0.93092	-0.10327	0.83401	0.99512	0.99251	-0.01084	0.90984	0.99941	0.99853	-0.00213
391.2105	3.52	olomics Pos	0.91324	0.99987	1.0247	0.035232	0.34039	0.99994	0.87381	-0.1946	0.040522	0.99996	0.81377	-0.29731	0.00487	0.17581	0.69071	-0.53384	0.001288	0.006625	0.54872	-0.86585	0.028507	0.14849	0.55521	-0.8489	0.043836	0.38909	0.73191	-0.45025	0.005452	0.52955	0.63207	-0.66184	0.15036	0.25572	0.79971	-0.32245
630.2803	2.37	olomics Ne	0.9137	0.99918	1.0824	0.11422	0.99609	0.99771	0.99846	-0.00223	0.72956	0.99995	1.114	0.1558	0.51253	0.99985	0.83346	-0.26282	0.015895	0.88267	0.37789	-1.404	0.48035	0.8481	0.81364	-0.29753	0.98267	0.99049	1.0294	0.041774	0.77834	0.99512	1.1151	0.15712	0.88066	0.99941	1.0299	0.042496
868.8142	10.02	olomics Pos	0.91375	0.99987	1.0075	0.010729	0.32937	0.99994	0.92261	-0.11621	0.73599	0.99996	1.036	0.050982	0.90444	0.9874	1.0069	0.009943	0.00761	0.024907	0.75942	-0.39702	0.25268	0.53916	0.85451	-0.22683	0.67611	0.90042	1.0486	0.068457	0.97288	0.99223	0.99996	-6E-05	0.2999	0.40972	1.1048	0.14377
545.3429	1.08	olomics Pos	0.91383	0.99987	1.0179	0.025657	0.76189	0.99994	1.0526	0.07392	0.63796	0.99996	1.1052	0.1443	0.5462	0.90473	1.1048	0.14376	0.70771	0.75084	0.91061	-0.1351	0.098316	0.33293	1.4511	0.53719	0.024496	0.33809	1.7466	0.80459	0.47184	0.96776	1.1137	0.15541	0.087121	0.1878	1.4331	0.51917
725.531	8.87	olomics Pc	0.91396	0.99951	1.0182	0.025972	0.76288	0.99998	0.97512	-0.03635	0.45259	0.99941	0.93992	-0.08939	0.43245	0.91715	0.92778	-0.10815	0.46477	0.86653	0.95021	-0.07368	0.24088	0.53115	1.1749	0.23256	0.013078	0.27282	1.4237	0.50965	0.46596	0.99956	1.0485	0.068259	0.00824	0.2688	1.2396	0.30988
266.257	6.96	olomics Pc	0.91412	0.99951	0.99942	-0.00084	0.4539	0.99998	0.88298	-0.17955	0.013624	0.99941	0.65218	-0.61666	0.010113	0.19754	0.46881	-1.0929	0.005159	0.030068	0.43982	-1.185	0.003316	0.049867	0.34995	-1.5148	0.003107	0.20152	0.35273	-1.5033	0.02468	0.52244	0.41756	-1.2599	0.015919	0.2688	0.46062	-1.1184

450.7171	0.31	olomics Po	0.91423	0.99951	1.0292	0.041455	0.61755	0.99998	0.94845	-0.07636	0.94173	0.99941	0.98987	-0.01469	0.57352	0.99905	0.94434	-0.08262	0.70325	0.99688	1.0407	0.057559	0.048732	0.1834	0.80117	-0.31981	0.42542	0.99185	0.91639	-0.12597	0.45226	0.99956	0.90894	-0.13774	0.68425	0.85771	0.958	-0.0619
448.9753	12	olomics Ne	0.91427	0.99918	1.0194	0.027778	0.014202	0.37315	0.80682	-0.30968	0.13283	0.99995	1.1341	0.18156	0.52029	0.99985	0.93933	-0.0903	0.54934	0.99927	0.95648	-0.06419	0.44216	0.82254	1.0416	0.058819	0.60595	0.86704	1.0549	0.077143	0.11536	0.99512	0.90375	-0.14601	0.60869	0.99941	1.0324	0.04605
852.7978	10.11	lomics Pos	0.91444	0.99987	1.0106	0.015246	0.20037	0.99994	0.8128	-0.29903	0.53806	0.99996	0.90612	-0.14223	0.49124	0.88874	0.89453	-0.1608	0.027182	0.065766	0.67128	-0.57502	0.45814	0.68721	0.88492	-0.17638	0.58337	0.86163	0.91238	-0.1323	0.54553	0.96776	0.90282	-0.14749	0.74706	0.81086	1.0311	0.044121
324.7766	0.57	lomics Neg	0.91445	0.99977	0.9877	-0.01785	0.91203	0.99904	0.98391	-0.0234	0.55542	0.99994	0.93493	-0.09707	0.22202	0.58182	1.1981	0.26075	0.88201	0.99989	1.0098	0.014035	0.99721	0.99721	0.99898	-0.00147	0.016775	0.29209	0.71618	-0.48161	0.16663	0.59205	0.84707	-0.23945	0.06471	0.405	0.75609	-0.40338
279.6479	1.12	lomics Pos	0.91451	0.99987	0.97654	-0.03425	0.97137	0.99994	0.94355	-0.08384	0.81481	0.99996	1.0114	0.0164	0.71356	0.96346	1.0765	0.10631	0.83139	0.85797	1.0264	0.037636	0.05567	0.23326	1.6536	0.7256	0.026285	0.34388	2.0288	1.0206	0.22837	0.94286	1.3358	0.41775	0.066078	0.16569	1.7361	0.79589
649.282	1.03	lomics Pos	0.91456	0.99987	1.061	0.08543	0.50563	0.99994	1.0932	0.1286	0.62076	0.99996	1.1299	0.17615	0.80766	0.97971	1.0555	0.077911	0.24363	0.33767	0.83388	-0.26208	0.032248	0.16163	1.4548	0.54083	0.012616	0.3324	1.7438	0.80223	0.22242	0.94047	1.1557	0.20875	0.071979	0.17377	1.3352	0.41706
1003.672	5.9	olomics Ne	0.91505	0.99918	0.96846	-0.04623	0.96317	0.99363	0.95867	-0.0609	0.83609	0.99995	1.0117	0.016712	0.96645	0.99985	0.93293	-0.10016	0.6822	0.99927	0.77851	-0.36121	0.17796	0.65203	1.6748	0.74397	0.041685	0.37456	2.0855	1.0604	0.32853	0.99512	1.269	0.3437	0.21748	0.99941	1.4306	0.5166
584.3284	2.99	lomics Pos	0.91523	0.99987	1.031	0.044036	0.9198	0.99994	0.97245	-0.04031	0.96952	0.99996	0.98017	-0.0289	0.59786	0.93237	0.92137	-0.11815	0.005583	0.019849	0.45323	-1.1417	0.23963	0.52848	0.83061	-0.26776	0.63532	0.8822	1.0991	0.13637	0.43048	0.96776	1.1489	0.20024	0.13575	0.24075	1.3318	0.41333
856.5082	1.83	lomics Pos	0.9153	0.99987	1.0469	0.066065	0.38237	0.99994	0.8009	-0.32031	0.1996	0.99996	0.71183	-0.4904	0.056313	0.40899	0.50183	-0.99473	0.003344	0.013311	0.35762	-1.4835	0.014518	0.10138	0.44144	-1.1797	0.008493	0.32129	0.45373	-1.1401	0.015136	0.52955	0.40615	-1.2999	0.036631	0.12969	0.51477	-0.958
294.9397	10.03	lomics Pos	0.91546	0.99987	1.0026	0.00369	0.20723	0.99994	0.90544	-0.1433	0.28955	0.99996	0.92595	-0.111	0.3921	0.83664	0.93734	-0.09336	0.001667	0.007875	0.75536	-0.40476	0.50257	0.71852	0.93564	-0.09597	0.58792	0.86163	0.95793	-0.06201	0.66257	0.97988	0.96744	-0.04775	0.055972	0.15312	1.1673	0.22314
1000.518	5.92	lomics Pos	0.91555	0.99987	1.0135	0.01933	0.83165	0.99994	0.97908	-0.0305	0.99205	0.99996	0.99892	-0.00156	0.70199	0.96092	0.95811	-0.06174	0.031251	0.073104	0.78445	-0.35024	0.15472	0.4337	0.79524	-0.33054	0.16796	0.63957	0.86047	-0.2168	0.42504	0.96776	0.89552	-0.15921	0.14726	0.25244	1.1552	0.20813
1062.655	1.11	lomics Pos	0.91573	0.99987	0.8054	-0.31223	0.92669	0.99994	0.78204	-0.35469	0.89184	0.99996	0.77778	-0.36257	0.68835	0.96092	1.0682	0.095225	0.36163	0.45463	1.7296	0.79045	0.03149	0.16025	4.1268	2.045	0.018695	0.33809	5.0388	2.3331	0.35107	0.96776	1.9339	0.95152	0.12433	0.2288	2.1561	1.1084
545.3434	5.1	olomics Po	0.9162	0.99951	1.0188	0.026867	0.66874	0.99998	1.0612	0.085681	0.46552	0.99941	1.1435	0.1935	0.25873	0.73159	1.1993	0.26219	0.17951	0.45971	1.2545	0.32707	0.018202	0.099714	1.588	0.66722	0.017812	0.30136	1.6062	0.68361	0.30272	0.99956	1.1308	0.17729	0.21856	0.48624	1.2121	0.2775
274.8984	0.56	lomics Pos	0.91653	0.99987	1.0091	0.013119	0.65765	0.99994	1.0552	0.077462	0.25911	0.99996	0.93009	-0.10456	0.029734	0.30229	0.87564	-0.19159	0.011356	0.033838	0.7555	-0.40449	0.003823	0.06842	0.78451	-0.35013	0.046402	0.39255	0.85426	-0.22726	0.22949	0.94286	0.91288	-0.13151	0.20307	0.31196	1.0679	0.094825
340.9835	4.37	olomics Ne	0.91662	0.99918	0.99771	-0.0033	0.0377	0.45581	0.9172	-0.12469	0.35858	0.99995	1.0241	0.03432	0.97682	0.99985	1.0056	0.008102	0.8108	0.99927	0.99154	-0.01226	0.43467	0.82254	1.0562	0.078907	0.30575	0.68222	1.0685	0.095582	0.89744	0.99512	1.0138	0.019825	0.81202	0.99941	0.9924	-0.01101
523.3594	5.46	olomics Po	0.91704	0.99951	1.0374	0.052976	0.99391	0.99998	0.99759	-0.00348	0.90186	0.99941	1.015	0.021507	0.84818	0.99905	1.0582	0.081639	0.59776	0.9681	1.1455	0.19596	0.066842	0.22713	1.7481	0.8058	0.040208	0.39977	1.8628	0.89747	0.50566	0.99956	1.2087	0.27351	0.14611	0.39824	1.7512	0.80836
412.8547	0.56	lomics Pos	0.91706	0.99987	1.0074	0.010591	0.16037	0.99994	0.93301	-0.10003	0.24756	0.99996	0.94364	-0.08369	0.038874	0.34213	0.87194	-0.1977	0.001546	0.007482	0.74986	-0.41531	0.016429	0.10923	0.82789	-0.27248	0.012626	0.3324	0.85773	-0.2214	0.3348	0.96443	0.94531	-0.08114	0.12946	0.23462	1.1441	0.19427
874.6645	6.38	lomics Pos	0.91711	0.99987	1.0225	0.032047	0.90064	0.99994	1.0462	0.065091	0.96125	0.99996	1.0771	0.10715	0.3589	0.81307	0.90472	-0.14445	0.19427	0.28756	0.83642	-0.25769	0.018213	0.11584	0.73837	-0.43758	0.79172	0.95091	1.0522	0.073355	0.092713	0.83714	0.85872	-0.21974	0.89684	0.92498	1.0105	0.015061
879.588	5.72	lomics Pos	0.91713	0.99987	1.0105	0.015097	0.71184	0.99994	0.97124	-0.0421	0.61191	0.99996	0.96102	-0.05737	0.44749	0.86921	0.92334	-0.11507	0.013919	0.039459	0.77533	-0.36711	0.359	0.62208	0.9229	-0.11575	0.92287	0.98157	1.0149	0.021332	0.98815	0.99578	1.0032	0.004669	0.01945	0.10555	1.5937	0.67238
575.3996	6.88	olomics Po	0.91761	0.99951	1.1127	0.15408	0.52802	0.99998	0.91018	-0.13578	0.87195	0.99941	0.98368	-0.02374	0.035809	0.25923	0.75485	-0.40574	0.37146	0.76828	1.0975	0.13424	0.45199	0.75639	0.8884	-0.17072	0.15828	0.73598	1.212	0.27738	0.40043	0.99956	1.4149	0.5007	0.54867	0.78204	1.0885	0.1223
876.0316	6.48	olomics Po	0.91817	0.99951	1.0692	0.096586	0.25684	0.99998	0.80979	-0.30438	0.95183	0.99941	1.01	0.014341	0.023611	0.22481	0.66323	-0.59242	0.16376	0.42845	0.79219	-0.33609	0.033484	0.14225	0.70248	-0.50947	0.56307	0.99391	0.9139	-0.1299	0.90144	0.99956	1.0375	0.053143	0.98612	0.99333	1.0172	0.024627
411.7703	5.06	lomics Pos	0.91824	0.99987	1.0159	0.022713	0.14593	0.99994	0.87852	-0.18685	0.40048	0.99996	0.9278	-0.10811	0.49669	0.89009	0.93629	-0.09497	0.047247	0.099844	0.78953	-0.34093	0.21552	0.50726	0.88495	-0.17633	0.60041	0.86607	1.0551	0.077373	0.48224	0.96776	1.0444	0.062644	0.11389	0.21832	1.1915	0.25273
339.1649	2.29	lomics Pos	0.91843	0.99987	1.0028	0.004013	0.27008	0.99994	0.70136	-0.51177	0.36432	0.99996	0.75637	-0.40284	0.018802	0.25593	0.4505	-1.1504	0.010468	0.031804	0.44268	-1.1757	0.063979	0.25399	0.45536	-1.1349	0.090255	0.51907	0.52721	-0.92356	0.045951	0.65869	0.45508	-1.1358	0.12738	0.23222	0.59664	-0.74506
97.0027	11.89	olomics Ne	0.9186	0.99918	0.99365	-0.00919	0.23824	0.66202	0.88709	-0.17285	0.88443	0.99995	1.009	0.012973	0.68198	0.99985	1.0715	0.09958	0.75789	0.99927	1.0148	0.021203	0.02053	0.43126	1.2395	0.30979	0.62192	0.87098	0.97103	-0.04241	0.74572	0.99512	1.0369	0.052254	0.89185	0.99941	0.99061	-0.01362
244.9256	0.51	lomics Pos	0.91872	0.99987	1.0388	0.054922	0.25558	0.99994	0.86965	-0.2015	0.12849	0.99996	0.87916	-0.18581	0.46086	0.87938	0.93637	-0.09485	0.004897	0.017989	0.76725	-0.38224	0.8521	0.92603	0.9918	-0.01188	0.46314	0.80647	1.0543	0.076336	0.57142	0.9703	0.95641	-0.0643	0.019507	0.10555	1.1961	0.25829
344.2275	1.1	lomics Pos	0.91889	0.99987	1.0219	0.031185	0.64591	0.99994	0.75464	-0.40614	0.5269	0.99996	0.70164	-0.51119	0.69254	0.96092	0.84904	-0.2361	0.003278	0.013129	0.086179	-3.5365	0.14267	0.41448	0.39134	-1.3535	0.19949	0.64891	0.41623	-1.2645	0.367	0.96776	0.52296	-0.93523	0.86636	0.90233	0.88705	-0.17291
277.2135	1.83	lomics Neg	0.91897	0.99977	1.1953	0.25739	0.59955	0.99904	1.0493	0.069452	0.12566	0.99994	0.59949	-0.73819	0.029395	0.58182	0.37857	-1.4014	0.000954	0.047058	0.29988	-1.7375	0.016812	0.46473	0.29664	-1.7532	0.001583	0.093659	0.2468	-2.0186	0.01039	0.46971	0.20211	-2.3068	0.013505	0.38536	0.27396	-1.8679
787.4804	6.5	olomics Po	0.91989	0.99951	1.0258	0.036769	0.56376	0.99998	0.93351	-0.09927	0.83794	0.99941	1.0249	0.035484	0.006978	0.1948	0.70088	-0.51276	0.43104	0.83504	0.91215	-0.13266	0.01327	0.085844	0.76141	-0.39326	0.87942	0.9951	1.0142	0.020347	0.53055	0.99956	0.92044	-0.1196	0.53556	0.77436	1.1289	0.17486
226.0456	0.35	olomics Po	0.92046	0.99951	0.97769	-0.03256	0.67965	0.99998	1.1189	0.16207	0.47965	0.99941	1.2061	0.27038	0.063373	0.33051	1.8326	0.87393	0.09902	0.28433	1.5363	0.61948	0.13114	0.36174	1.3708	0.45507	0.033552	0.36264	1.7536	0.81029	0.46322	0.99956	1.2138	0.27954	0.4858	0.74188	1.189	0.24981
732.7343	0.56	lomics Pos	0.92144	0.99987	0.99422	-0.00836	0.55567	0.99994	1.0493	0.06948	0.079055	0.99996	0.84149	-0.24899	0.017464	0.24755	0.73306	-0.448	0.13282	0.2155	0.83945	-0.25249	0.002665	0.061655	0.74189	-0.43072	0.021055	0.33809	0.75124	-0.41266	0.10534	0.86277	0.91781	-0.12374	0.09931	0.2011	1.145	0.19529
585.5155	6.38	olomics Po	0.92159	0.99951	1.005	0.007191	0.34565	0.99998	0.90519	-0.14371	0.025049	0.99941	0.72453	-0.46489	0.21099	0.65159	0.74672	-0.42136	0.000198	0.002775	0.4189	-1.2553	0.042312	0.16681	0.58217	-0.78048	0.00012	0.16989	0.46862	-1.0935	0.12732	0.90554	0.72904	-0.45593	0.32004	0.59795	0.88552	-0.1754
193.1917	4.73	lomics Pos	0.92166	0.99987	0.99439	-0.00811	0.056779	0.99994	0.86101	-0.2159	0.028588	0.99996	0.7945	-0.33188	0.060795	0.42535	0.84346	-0.24562	0.00141	0.007026	0.67577	-0.56539	0.67091	0.8287	0.97034	-0.04343	0.16738	0.63888	0.87954	-0.18518	0.56975	0.9703	0.9556	-0.06552	0.36892	0.47782	1.127	0.17251
216.0152	0.59	lomics Pos	0.92173	0.99987	1.0196	0.028016	0.1006	0.99994	0.8121	-0.30027	0.38858	0.99996	1.1037	0.14234	0.038274	0.34165	1.2858	0.36272	0.38009	0.47177	0.89711	-0.15665	0.08188	0.29706	1.3944	0.47962	0.044415	0.38909	1.6106	0.68763	0.13038	0.88537	1.1755	0.23322	0.056227	0.15336	1.2248	0.29252
267.2684	7.49	olomics Po	0.92178	0.99951	0.93231	-0.10113	0.82643	0.99998	0.92534	-0.11194	0.13634	0.99941	0.64363	-0.63571	0.06217	0.32748	0.49293	-1.0205	0.028502	0.11044	0.47195	-1.0833	0.011513	0.083709	0.35739	-1.4844	0.021645	0.31651	0.42153	-1.2463	0.011192	0.47452	0.38566	-1.3746	0.044617	0.28476	0.4884	-1.0339
1025.749	0.54	lomics Pos	0.92218	0.99987	1.0259	0.036952	0.43252	0.99994	0.86436	-0.2103	0.45666	0.99996	0.87486	-0.19288	0.32883	0.78974	0.83717	-0.25641	0.012804	0.036957	0.6112	-0.71028	0.34556	0.61311	0.84073	-0.25029	0.69372	0.9106	1.0433	0.061099	0.85079	0.97988	1.0456	0.064373	0.2375	0.34551	1.2431	0.31396
157.0319	10.16	lomics Pos	0.92243	0.99987	1.0044	0.006353	0.53356	0.99994	0.94244	-0.08553	0.39403	0.99996	0.93975	-0.08965	0.005443	0.17822	0.8141	-0.29672	0.014449	0.04069	0.80165	-0.31895	0.17581	0.462	0.89787	-0.15541	0.35717	0.75448	0.94075	-0.08812	0.83388	0.97988	1.0138	0.019767	0.008503	0.092495	1.1994	0.26232
327.2315	2.04	lomics Neg	0.9227	0.99977	1.0384	0.054295	0.33942	0.99904	0.76448	-0.38744	0.07325	0.99994	0.60542	-0.72399	0.070074	0.58182	0.53955	-0.89018	0.01489	0.33903	0.37314	-1.4222	0.01884	0.46473	0.27957	-1.8387	0.005745	0.11336	0.31159	-1.6823	0.030983	0.46971	0.28272	-1.8225	0.012623	0.38536	0.43658	-1.1957
833.0122	6.48	olomics Po	0.92288	0.99951	1.0498	0.070145	0.35972	0.99998	0.83875	-0.25368	0.36442	0.99941	1.1311	0.17774	0.042471	0.2802	0.68703	-0.54156	0.62627	0.97793	0.91487	-0.12836	0.36306	0.67471	0.85544	-0.22525	0.85511	0.9951	0.98824	-0.01706	0.5983	0.99956	0.91278	-0.13165	0.52556	0.76733	1.1667	0.22238
1140.757	10.02	lomics Pos	0.9229	0.99987	1.0125	0.017932	0.5914	0.99994	0.9386	-0.09141	0.25304	0.99996	0.88331	-0.179	0.46848	0.88476	0.91841	-0.12279	0.022239	0.056462	0.75255	-0.41014	0.34532	0.61311	0.88713	-0.17278	0.92968	0.98468	1.0072	0.010419	0.68411	0.97988	0.95068	-0.07296	0.17645	0.284	1.1884	0.24899
843.5723	5.01	lomics Pos	0.92305	0.99987	0.98649	-0.01962	0.91189	0.99994	0.98568	-0.02081	0.92867	0.99996	1.0236	0.03372	0.39473	0.83821	0.87906	-0.18596	0.01927	0.05071	0.71802	-0.4779	0.00537	0.074454	0.63191	-0.66221	0.006531	0.3088	0.68385	-0.54824	0.014297	0.52955	0.69812	-0.51845	0.53098	0.62935	0.91427	-0.12931
619.2566	5.11	olomics Po	0.92334	0.99951	1.0686	0.095785	0.67409	0.99998	0.88357	-0.17859	0.73162	0.99941	0.89085	-0.16674	0.6201	0.99905	1.1366	0.18469	0.34082	0.72281	1.3628	0.44653	0.04307	0.16875	1.5754	0.65575	0.017409	0.29966	1.8383	0.8784	0.27944	0.99956	1.3154	0.39547	0.22398	0.49242	1.2947	0.37266
303.2368	6.03	olomics Po	0.92371	0.99951	1.0433	0.061172	0.80123	0.99998	0.97453	-0.03722	0.076776	0.99941	0.75954	-0.3968	0.008993	0.19702	0.60142	-0.73356	0.000292	0.003769	0.26619	-1.9095	0.000497	0.039708	0.39375	-1.3446	0.000333	0.16989	0.45782	-1.1271	0.007433	0.47452	0.46701	-1.0985	0.00679	0.2688	0.52063	-0.94168
194.0232	0.34	olomics Po	0.92371	0.99951	0.91639	-0.12597	0.92068	0.99998	1.0293	0.041728	0.7481	0.99941	1.1198	0.16326	0.20056	0.63556	1.7584	0.81428	0.18334	0.46536	1.749	0.80652	0.23636	0.52581	1.5456	0.62815	0.030821	0.35049	2.1649	1.1143	0.39956	0.99956	1.3547	0.43795	0.88462	0.95289	1.0028	0.003978
500.2757	10.1	olomics Po	0.92386	0.99951	1.0376	0.053304	0.77616	0.99998	0.96518	-0.05114	0.36208	0.99941	0.84193	-0.24822	0.93518	0.99905	1.0323	0.045833	0.57297	0.95042	1.135	0.18273	0.053016	0.19483	1.5338	0.61713	0.016802	0.29901	1.7244	0.78613	0.13394	0.91719	1.3558	0.43919	0.14894	0.40234	1.2773	0.35307
397.755	4.53	lomics Pos	0.92498	0.99987	1.0059	0.008546	0.28263	0.99994	0.872	-0.19761	0.73594	0.99996	0.9646	-0.05199	0.20321	0.64948	0.7934	-0.33388	0.035863	0.081083	0.65655	-0.60703	0.068453	0.26634	0.75006	-0.41492	0.83748	0.96659	0.99966	-0.00049	0.68922	0.97988	1.0613	0.085766	0.003377	0.085302	2.0106	1.0077
559.2991	1.11	lomics Pos	0.92528	0.99987	0.94524	-0.08125	0.9837	0.99994	0.95267	-0.06995	0.93559	0.99996	1.014	0.020038	0.48728	0.88725	1.1822	0.24143	0.40558	0.49519	1.2555	0.32827	0.018112	0.11549	2.3361	1.2241	0.006646	0.3088	2.7163	1.4416	0.18298	0.92582	1.6024	0.6802	0.047505	0.14422	1.994	0.9957
1102.759	10.03	lomics Pos	0.92534	0.99987	1.0083	0.011986	0.53531	0.99994	0.96747	-0.04771	0.43437	0.99996	0.93296	-0.10012	0.002067	0.17581	0.78853	-0.34277	0.000415	0.00284	0.75997	-0.39598	0.069495	0.26875	0.84322	-0.24602	0.58759	0.86163	0.96595	-0.04998	0.6479	0.97988	0.98124	-0.02733	0.12004	0.22427	1.1886	0.24925
558.2953	1.11	lomics Pos	0.92584	0.99987	0.97234	-0.04046	0.93514	0.99994	0.94834	-0.07652	0.61553	0.99996	1.1158	0.15812	0.5174	0.89842	1.1903	0.25136	0.42362	0.51051	1.2658	0.34001	0.019349	0.11985	2.4329	1.2827	0.00847	0.32129	2.9323	1.552	0.17031	0.91806	1.6553	0.72713	0.049937	0.14647	1.9951	0.99647
160.0197	0.56	lomics Pos	0.9262	0.99987	0.98619	-0.02006	0.58638	0.99994	0.925	-0.11247	0.27326	0.99996	1.2122	0.27764	0.054533	0.40237	1.5999	0.67802	0.14481	0.23084	1.2898	0.36719	0.00763	0.082308	1.6444	0.71755	0.001581	0.23318	2.0176	1.0127	0.0827	0.80648	1.5748	0.65516	0.020862	0.10765	1.4861	0.57153
859.6594	10.34	olomics Ne	0.92651	0.99918	0.99649	-0.00508	0.00309	0.34605	0.82922	-0.27017	0.32093	0.99995	0.92509	-0.11233	0.18927	0.99985	0.93117	-0.10288	0.47919	0.99927	0.96785	-0.04714	0.57107	0.87838	0.96628	-0.04949	0.14752	0.53641	0.88422	-0.17753	0.1996	0.99512	0.93249	-0.10084	0.17716	0.99941	0.93512	-0.09677
241.031	0.65	lomics Pos	0.92652	0.99987	0.99787	-0.00308	0.8381	0.99994	1.0266	0.037913	0.38786	0.99996	1.1359	0.18381	0.78674	0.97526	0.94921	-0.0752	0.02944	0.069896	0.69504	-0.52483	0.10719	0.34962	0.7503	-0.41447	0.15527	0.62672	0.78811	-0.34353	0.4129	0.96776	0.86158	-0.21494	0.66898	0.75268	1.1039	0.14261
928.715	5.34	lomics Pos	0.92686	0.99987	0.9904	-0.01392	0.64742	0.99994	0.92768	-0.10829	0.65783	0.99996	0.92956	-0.10538	0.9089	0.9874	1.0306	0.043523	0.029423	0.069896	0.62276	-0.68325	0.27282	0.55982	0.82083	-0.28484	0.33659	0.74249	0.84833	-0.2373	0.54804	0.96776	0.904	-0.14561	0.47681	0.57956	0.88634	-0.17407
897.7995	10.03	lomics Pos	0.92687	0.99987	0.99874	-0.00181	0.15385	0.99994	0.94124	-0.08737	0.30039	0.99996	0.91218	-0.13261	0.005109	0.17581	0.82492	-0.27768	0.002146	0.009537	0.72286	-0.46822	0.03827	0.17905	0.78462	-0.34993	0.25356	0.69047	0.92483	-0.11274	0.017708	0.52955	0.82116	-0.28426	0.028924	0.11762	1.1361	0.18405
866.5701	5.14	lomics Pos	0.92702	0.99987	1.0038	0.005469	0.48046	0.99994	0.90374	-0.14602	0.87604	0.99996	0.97906	-0.03052	0.99907	0.99936	1.0101	0.014512	0.029359	0.069835	0.76542	-0.38568	0.96831	0.98309	1.0084	0.012086	0.91622	0.98047	0.98414	-0.02306	0.60193	0.97073	1.0747	0.1039	0.082338	0.18371	1.1918	0.25309
593.3386	1.13	lomics Pos	0.92714	0.99987	1.0625	0.087486	0.75941	0.99994	1.0544	0.076355	0.41276	0.99996	1.1815	0.24057	0.686	0.96092	1.0655	0.091593	0.70474	0.74895	0.89489	-0.16022	0.13824	0.40744	1.4383	0.52436	0.029804	0.35536	1.867	0.90076	0.40927	0.96776	1.1901	0.2511	0.025662	0.113	1.6508	0.72317
528.8477	10.16	lomics Pos	0.92771	0.99987	1.0174	0.024958	0.26231	0.99994	0.88983	-0.1684	0.40587	0.99996	0.92427	-0.11362	0.71045	0.96346	1.0203	0.029057	0.024315	0.060399	0.73705	-0.44016	0.042492	0.19358	0.85089	-0.23295	0.96279	0.99112	1.0081	0.011618	0.61029	0.97371	0.96613	-0.04971	0.11413	0.21868	1.1259	0.17114
799.5481	5.55	lomics Pos	0.92776	0.99987	1.0107	0.015407	0.43446	0.99994	0.9462	-0.07978	0.80148	0.99996	0.98138	-0.02711	0.32421	0.7879	0.91721	-0.12467	0.043128	0.093278	0.79429	-0.33226	0.18778	0.47387	0.91128	-0.13403	0.36302	0.75846	1.081	0.11233	0.83759	0.97988	0.98561	-0.02091	0.17681	0.28423	1.0987	0.13573
892.5362	8.35	olomics Ne	0.92788	0.99918	0.99929	-0.00102	0.23471	0.65665	0.85224	-0.23067	0.5382	0.99995	0.92965	-0.10524	0.89015	0.99985	0.9968	-0.00462	0.59524	0.99927	0.9375	-0.09311	0.043619	0.48405	1.2577	0.33074	0.33993	0.71058	1.0952	0.1312	0.36282	0.99512	0.89206	-0.16479	0.45942	0.99941	0.92282	-0.11588
111.0206	10.23	lomics Pos	0.9281	0.99987	0.99131	-0.01259	0.17826	0.99994	0.90462	-0.14462	0.68058	0.99996	0.97111	-0.0423	0.096821	0.51634	0.88098	-0.18282	0.001085	0.005858	0.72624	-0.46149	0.49962	0.71565	0.93382	-0.09878	0.39831	0.77223	0.94297	-0.08471	0.86642	0.98023	0.98675	-0.01925	0.077844	0.17957	1.1419	0.19149
476.2751	1.35	lomics Pos	0.92823	0.99987	1.0104	0.014916	0.94543	0.99994	0.94804	-0.07699	0.99337	0.99996	0.98278	-0.02506	0.79649	0.9771	1.0107	0.015376	0.69716	0.74232	0.86717	-0.20561	0.059342	0.24149	1.6542	0.72614	0.015978	0.33809	2.0702	1.0498	0.070728	0.76834	1.6182	0.69439	0.02629	0.11404	1.9235	0.94375
989.7702	10.07	lomics Pos	0.92828	0.99987	1.0064	0.009259	0.093839	0.99994	0.86471	-0.20971	0.73262	0.99996	0.97186	-0.04118	0.47196	0.88482	0.91188	-0.13308	0.004795	0.017665	0.74961	-0.41578	0.64331	0.81402	0.95475	-0.06681	0.67264	0.89929	1.0426	0.060237	0.64275	0.97988	1.0705	0.098257	0.08021	0.18194	1.2425	0.31328
893.534	8.33	olomics Ne	0.92834	0.99918	1.0146	0.020971	0.94935	0.9934	0.9924	-0.011	0.90366	0.99995	1.013	0.018589	0.99514	0.99985	1.0022	0.003113	0.93114	0.99927	1.0093	0.013321	0.042295	0.48405	1.1958	0.25798	0.19687	0.5757	1.1355	0.18332	0.7918	0.99512	1.0192	0.027373	0.77226	0.99941	1.0281	0.040026
208.198	8.37	olomics Po	0.92851	0.99951	1.0076	0.010992	0.95167	0.99998	1.0074	0.010576	0.54085	0.99941	0.93777	-0.0927	0.029959	0.24576	0.87731	-0.18884	0.26194	0.61029	0.93258	-0.10071	0.78082	0.93703	0.9863	-0.01989	0.11594	0.64715	0.87651	-0.19016	0.002408	0.47452	0.75753	-0.40062	0.027445	0.2688	0.80273	-0.31702
529.4389	9.8	lomics Pos	0.92855	0.99987	1.0163	0.023262	0.77111	0.99994	1.013	0.018664	0.25564	0.99996	0.92271	-0.11605	0.16646	0.59991	0.91894	-0.12196	0.001013	0.005552	0.68992	-0.53549	0.33768	0.60908	0.90592	-0.14255	0.29381	0.7186	0.93736	-0.09333	0.61122	0.97371	0.97079	-0.04277	0.84237	0.88337	1.0322	0.04576
167.1429	6.97	olomics Po	0.9287	0.99951	1.0141	0.020253	0.73706	0.99998	0.94276	-0.08503	0.093029	0.99941	0.68977	-0.53582	0.033024	0.25203	0.52498	-0.92967	0.00489	0.0289	0.44702	-1.1616	0.002403	0.046224	0.35535	-1.4927	0.008196	0.23862	0.40141	-1.3169	0.026886	0.55353	0.46603	-1.1015	0.033301	0.2688	0.52732	-0.92324
572.8405	10.1	lomics Pos	0.92871	0.99987	0.99382	-0.00894	0.13272	0.99994	0.86806	-0.20414	0.16286	0.99996	0.90529	-0.14354	0.38924	0.83582	1.1012	0.13902	0.017178	0.046309	0.78004	-0.35837	0.95867	0.97873	1.0082	0.011817	0.61232	0.87064	1.0456	0.064342	0.91002	0.98248	0.99345	-0.00948	0.070612	0.17171	1.279	0.355
115.9644	10.19	lomics Pos	0.92894	0.99987	1.0073	0.010564	0.009438	0.99994	0.82448	-0.27844	0.27841	0.99996	0.92063	-0.11931	0.89549	0.9874	1.0093	0.013295	0.010942	0.032905	0.71709	-0.47977	0.47394	0.69638	0.91991	-0.12043	0.61288	0.87097	1.0436	0.061534	0.28806	0.94825	0.93786	-0.09256	0.080385	0.18201	1.1407	0.18989
914.8063	0.5	lomics Pos	0.92908	0.99987	1.0021	0.003083	0.32448	0.99994	0.88889	-0.16992	0.15599	0.99996	0.87099	-0.19927	0.36517	0.82116	0.91508	-0.12804	0.005319	0.019097	0.75672	-0.40216	0.76034	0.87509	0.97569	-0.0355	0.83546	0.96659	1.0178	0.025493	0.52876	0.96776	0.95047	-0.07329	0.10415	0.20689	1.1725	0.22953
1111.789	10.02	lomics Pos	0.92922	0.99987	0.98723	-0.01854	0.46649	0.99994	0.93511	-0.09679	0.41321	0.99996	0.9126	-0.13194	0.23565	0.69438	0.88465	-0.17682	0.015073	0.041901	0.73603	-0.44217	0.11801	0.37219	0.83302	-0.26358	0.29292	0.7186	0.89261	-0.16389	0.40233	0.96776	0.92192	-0.11728	0.21883	0.32671	1.1244	0.16918
473.2413	0.95	lomics Pos	0.92928	0.99987	0.95323	-0.06911	0.65987	0.99994	0.85909	-0.21911	0.68101	0.99996	1.3329	0.41461	0.46274	0.88001	1.6223	0.69801	0.45919	0.54042	1.4445	0.53061	0.56836	0.76437	1.8188	0.86297	0.032813	0.36346	9.7225	3.2813	0.12209	0.88537	6.8471	2.7755	0.13374	0.23852	2.7278	1.4477
1168.518	6.98	olomics Po	0.92935	0.99951	1.111	0.15188	0.94574	0.99998	1.0972	0.13385	0.86898	0.99941	0.97089	-0.04263	0.38774	0.88571	0.83254	-0.26441	0.025265	0.10074	0.59528	-0.74837	0.023393	0.11336	0.58657	-0.76963	0.067081	0.52433	0.60752	-0.719	0.057656	0.70429	0.57413	-0.80054	0.12471	0.37447	0.65306	-0.61472
1145.767	0.51	lomics Pos	0.9294	0.99987	0.9839	-0.02342	0.35012	0.99994	0.88041	-0.18375	0.70949	0.99996	0.9448	-0.08191	0.22745	0.68384	0.85358	-0.2284	0.032415	0.075228	0.72141	-0.47111	0.098835	0.3341	0.7735	-0.37053	0.41015	0.78028	0.89523	-0.15967	0.43541	0.96776	0.89951	-0.15279	0.48501	0.5871	1.0874	0.12088
504.6321	0.57	lomics Neg	0.9296	0.99977	0.97727	-0.03317	0.5061	0.99904	0.89925	-0.1532	0.20896	0.99994	0.84451	-0.24382	0.82723	0.88079	1.0332	0.047156	0.96736	0.99989	0.98595	-0.02042	0.14401	0.99653	0.80925	-0.30534	0.03359	0.37635	0.67848	-0.55963	0.21368	0.62019	0.78253	-0.35378	0.042179	0.405	0.67558	-0.56581
429.1811	6.96	olomics Ne	0.92961	0.99918	1.0122	0.017503	0.72618	0.95436	0.94678	-0.0789	0.09983	0.99995	0.7367	-0.44084	0.077193	0.99985	0.64538	-0.63177	0.017926	0.88267	0.62002	-0.68961	0.012584	0.40245	0.52578	-0.92747	0.006118	0.2539	0.57908	-0.78818	0.010632	0.99512	0.52762	-0.92242	0.083774	0.99941	0.65362	-0.61347
848.5396	5	lomics Pos	0.92983	0.99987	1.018	0.025801	0.38202	0.99994	0.87885	-0.18631	0.66127	0.99996	0.95108	-0.07237	0.7467	0.97368	0.96743	-0.04777	0.018476	0.049059	0.69345	-0.52814	0.4025	0.64785	0.86636	-0.20696	0.49005	0.82099	0.92016	-0.12004	0.47364	0.96776	1.0689	0.096062	0.027017	0.11562	1.372	0.45625
1061.651	5.1	olomics Po	0.93016	0.99951	0.98784	-0.01765	0.67309	0.99998	0.78122	-0.35619	0.84932	0.99941	0.83361	-0.26256	0.48555	0.96315	1.6172	0.69351	0.18136	0.46218	2.0646	1.0459	0.030925	0.13499	3.1116	1.6377	0.027486	0.33821	3.6713	1.8763	0.21372	0.99956	2.0104	1.0075	0.18902	0.45476	1.7226	0.78458
762.5722	4.94	lomics Pos	0.93058	0.99987	0.99616	-0.00555	0.4488	0.99994	0.95092	-0.0726	0.18718	0.99996	0.9297	-0.10516	0.094116	0.51047	0.87666	-0.18991	0.000834	0.004869	0.753	-0.40927	0.25849	0.54507	0.92817	-0.10754	0.42796	0.79094	1.0623	0.087182	0.087247	0.82455	1.1013	0.13917	0.00095	0.07528	1.2941	0.37197
332.925	10.15	lomics Pos	0.93092	0.99987	0.99825	-0.00253	0.18444	0.99994	0.85882	-0.21957	0.13152	0.99996	0.84595	-0.24135	0.099358	0.51973	0.83345	-0.26283	0.030871	0.072438	0.77077	-0.37564	0.21207	0.50393	0.8392	-0.25291	0.25638	0.69104	0.87272	-0.19641	0.13378	0.88865	0.83942	-0.25254	0.18962	0.29729	1.1428	0.19256
484.3305	1.92	lomics Pos	0.93097	0.99987	1.0347	0.049185	0.98751	0.99994	0.97121	-0.04215	0.7731	0.99996	0.91207	-0.13279	0.77951	0.97526	0.9025	-0.14799	0.28013	0.37492	0.7164	-0.48116	0.1743	0.46089	1.2861	0.36305	0.039551	0.37421	1.7765	0.82908	0.2439	0.94365	1.2835	0.36004	0.028998	0.11767	1.7282	0.78925
904.6553	6.05	lomics Pos	0.93107	0.99987	0.99372	-0.00909	0.20196	0.99994	0.83924	-0.25285	0.49443	0.99996	0.93141	-0.10251	0.25615	0.71396	0.88399	-0.1779	0.018997	0.050164	0.74536	-0.424	0.29353	0.57612	0.83999	-0.25156	0.61233	0.87064	0.95748	-0.06269	0.27615	0.94365	0.88915	-0.1695	0.41406	0.52141	0.92539	-0.11187
197.0383	12.49	olomics Po	0.9317	0.99951	1.0022	0.003132	0.89062	0.99998	0.99547	-0.00655	0.1732	0.99941	0.95708	-0.06329	0.35029	0.84365	1.0751	0.10449	0.35474	0.74429	1.0591	0.082843	0.20508	0.48137	1.1426	0.19231	0.030175	0.34968	1.1248	0.16965	0.70325	0.99956	1.0574	0.08048	0.1422	0.39464	1.077	0.10706
708.2359	1.03	lomics Pos	0.93171	0.99987	1.085	0.11764	0.70584	0.99994	1.0416	0.058788	0.3136	0.99996	1.1991	0.2619	0.69769	0.96092	0.94008	-0.08914	0.29225	0.38666	0.83137	-0.26643	0.1794	0.46725	1.2933	0.37106	0.041481	0.38314	1.5795	0.65944	0.40386	0.96776	1.1109	0.15179	0.025303	0.11206	1.4381	0.52417
600.4665	6.47	olomics Po	0.93171	0.99951	1.0056	0.008045	0.070675	0.99998	0.76664	-0.38338	0.027337	0.99941	0.75575	-0.40402	0.024436	0.22909	0.66381	-0.59116	7.23E-06	0.000278	0.29075	-1.7822	0.00063	0.039708	0.4416	-1.1792	0.000741	0.16989	0.50968	-0.97235	0.01153	0.47452	0.51935	-0.94523	0.031056	0.2688	0.55592	-0.84706
479.3013	1.16	lomics Pos	0.93188	0.99987	1.0138	0.019726	0.82269	0.99994	0.97477	-0.03687	0.94608	0.99996	1.0145	0.020783	0.60409	0.93327	1.1387	0.18735	0.34357	0.43624	1.3221	0.40284	0.010635	0.089124	2.1161	1.0814	0.002952	0.26175	2.4779	1.3091	0.14798	0.91175	1.4614	0.54735	0.089702	0.19023	1.7655	0.82007
784.4358	0.63	lomics Pos	0.93216	0.99987	1.0016	0.002276	0.77376	0.99994	0.97537	-0.03598	0.3784	0.99996	0.92294	-0.11569	0.063066	0.43296	0.81909	-0.28791	0.005911	0.020677	0.7466	-0.42159	0.36943	0.62855	0.91614	-0.12637	0.1015	0.54433	0.85346	-0.2286	0.33483	0.96443	0.9114	-0.13385	0.029499	0.11849	1.2931	0.3708
744.8489	10.02	lomics Pos	0.93262	0.99987	0.98781	-0.01769	0.20002	0.99994	0.84803	-0.23782	0.19421	0.99996	0.84202	-0.24808	0.29853	0.75637	0.9021	-0.14864	0.021247	0.054851	0.76696	-0.38278	0.20145	0.48967	0.83377	-0.26228	0.17529	0.64616	0.8317	-0.26586	0.23035	0.94286	0.88336	-0.17893	0.38062	0.48915	1.0928	0.12803
388.2596	8.37	olomics Po	0.93275	0.99951	1.0229	0.032664	0.19772	0.99998	0.85874	-0.21971	0.13583	0.99941	0.90176	-0.14919	0.22029	0.66625	0.91404	-0.12967	0.42344	0.82489	0.95175	-0.07135	0.61301	0.87426	0.96246	-0.0552	0.72944	0.99391	0.97751	-0.03282	0.011828	0.47452	0.75461	-0.40619	0.17992	0.44485	0.84601	-0.24125
608.3275	5.72	olomics Po	0.93319	0.99951	1.0175	0.02501	0.54931	0.99998	0.94916	-0.07528	0.53804	0.99941	1.0586	0.082112	0.049074	0.29222	0.77422	-0.36918	0.057533	0.18645	0.66948	-0.57889	0.028309	0.12757	0.80093	-0.32026	0.60164	0.99391	0.94768	-0.07753	0.24553	0.99956	0.86886	-0.20281	0.20388	0.46828	1.1296	0.17587
432.919	0.5	lomics Pos	0.93331	0.99987	1.0024	0.003428	0.73095	0.99994	0.98126	-0.02729	0.056818	0.99996	0.90623	-0.14205	0.13093	0.5541	0.91415	-0.12949	0.002382	0.01031	0.75748	-0.40072	0.40564	0.65019	0.91543	-0.12748	0.55166	0.85184	1.0316	0.044953	0.32446	0.95459	0.93457	-0.09762	0.013109	0.098791	1.1882	0.24878
195.8113	0.39	olomics Ne	0.93351	0.99918	0.99918	-0.00119	0.030898	0.43222	0.90493	-0.14412	0.88821	0.99995	0.99622	-0.00546	0.58094	0.99985	1.0176	0.025166	0.33781	0.99927	1.0638	0.08922	0.06546	0.51001	1.1057	0.14502	0.030161	0.34429	1.0748	0.10402	0.82806	0.99512	0.99326	-0.00975	0.46098	0.99941	0.97571	-0.03548
1064.662	1.1	lomics Pos	0.93364	0.99987	1.0032	0.004571	0.75543	0.99994	1.1906	0.25174	0.6545	0.99996	1.1193	0.16264	0.56282	0.91088	1.1584	0.2122	0.35949	0.45284	1.6887	0.75593	0.017924	0.11525	4.3673	2.1267	0.004642	0.3088	5.6374	2.495	0.17206	0.91806	1.8033	0.85065	0.07505	0.17705	2.2058	1.1413
197.1538	2.28	lomics Pos	0.93413	0.99987	1.0168	0.024053	0.64933	0.99994	0.93809	-0.0922	0.03138	0.99996	0.57991	-0.7861	0.003444	0.17581	0.46607	-1.1014	0.000242	0.001922	0.3778	-1.4043	0.001648	0.052793	0.40409	-1.3073	0.038311	0.37121	0.5119	-0.96607	0.026911	0.57206	0.48389	-1.0472	0.019536	0.10555	0.50487	-0.98601
455.297	1.35	lomics Pos	0.93436	0.99987	0.94149	-0.08698	0.88134	0.99994	0.90048	-0.15123	0.97084	0.99996	0.94046	-0.08856	0.83413	0.98621	0.98455	-0.02247	0.88393	0.90215	0.88661	-0.17363	0.099547	0.33561	1.6439	0.71712	0.022142	0.33809	2.231	1.1577	0.093089	0.83714	1.6486	0.72122	0.033609	0.12517	2.0536	1.0381
324.8907	10.17	lomics Pos	0.93518	0.99987	1.0104	0.014873	0.77686	0.99994	0.96036	-0.05835	0.4183	0.99996	0.90626	-0.142	0.54436	0.90473	0.91508	-0.12803	0.007839	0.025567	0.66861	-0.58076	0.64513	0.81443	0.93668	-0.09437	0.97956	0.99431	0.99645	-0.00513	0.66664	0.97988	1.0398	0.056242	0.39742	0.50579	1.1125	0.15382
343.1818	6.43	olomics Po	0.93555	0.99951	1.0219	0.031259	0.76106	0.99998	0.96664	-0.04896	0.27213	0.99941	0.89118	-0.16621	0.21004	0.6512	0.80157	-0.31911	0.000194	0.002737	0.43187	-1.2113	0.008429	0.075866	0.7327	-0.44871	0.11164	0.64238	0.77264	-0.37213	0.11045	0.86978	0.71527	-0.48343	0.029636	0.2688	0.73308	-0.44795
314.2775	1.32	lomics Pos	0.93559	0.99987	0.96976	-0.04431	0.91793	0.99994	0.95338	-0.06888	0.69912	0.99996	1.0703	0.097997	0.6884	0.96092	1.0383	0.054155	0.95779	0.96437	0.96769	-0.04738	0.18607	0.47264	1.4913	0.57657	0.035677	0.36525	2.1508	1.1049	0.15527	0.91321	1.5357	0.6189	0.044158	0.13997	2.0325	1.0233
780.439	0.63	lomics Pos	0.93615	0.99987	0.9941	-0.00854	0.5472	0.99994	0.92949	-0.1055	0.16177	0.99996	0.83681	-0.25703	0.14672	0.57298	0.83001	-0.26881	0.027977	0.067345	0.74124	-0.43198	0.10777	0.35034	0.79123	-0.33783	0.5237	0.83957	0.92324	-0.11523	0.40881	0.96776	0.89335	-0.16271	0.14965	0.25475	1.1963	0.25852
422.8831	10.06	lomics Pos	0.93652	0.99987	1.0014	0.001994	0.31132	0.99994	0.93528	-0.09653	0.085131	0.99996	0.89803	-0.15517	0.4197	0.85533	0.95581	-0.0652	0.001234	0.006406	0.75443	-0.40654	0.50903	0.72221	0.93704	-0.09382	0.84608	0.96798	1.0209	0.029785	0.82122	0.97988	0.99086	-0.01325	0.011183	0.094875	1.1868	0.24707
395.2216	6.32	olomics Ne	0.93653	0.99918	1.0474	0.066866	0.1933	0.61138	0.78074	-0.35708	0.080776	0.99995	0.75471	-0.406	0.018733	0.99985	0.51353	-0.96148	0.008327	0.69321	0.4505	-1.1504	0.002564	0.36417	0.37299	-1.4228	0.008456	0.2539	0.44934	-1.1541	0.010296	0.99512	0.4708	-1.0868	0.011795	0.99941	0.50637	-0.98175
181.1593	6.97	olomics Po	0.93694	0.99951	0.99047	-0.01381	0.55872	0.99998	0.905	-0.14401	0.03426	0.99941	0.6856	-0.54456	0.009898	0.19702	0.46565	-1.1027	0.001963	0.015227	0.41637	-1.2641	0.001858	0.045425	0.33691	-1.5696	0.004631	0.20152	0.38633	-1.3721	0.017771	0.51556	0.45954	-1.1217	0.025606	0.2688	0.52346	-0.93386
454.2933	1.35	lomics Pos	0.93714	0.99987	0.98958	-0.01511	0.97753	0.99994	0.94358	-0.08378	0.88092	0.99996	0.91147	-0.13374	0.88454	0.9874	0.97771	-0.03253	0.63954	0.69356	0.8159	-0.29354	0.10835	0.35192	1.5649	0.64603	0.022563	0.33809	2.122	1.0855	0.11098	0.87212	1.5433	0.62599	0.039613	0.13343	1.9239	0.944
975.8103	0.5	lomics Pos	0.93761	0.99987	1.0007	0.00101	0.93029	0.99994	0.99238	-0.01103	0.28831	0.99996	0.92729	-0.10891	0.031547	0.30922	0.85462	-0.22665	0.004909	0.018016	0.74813	-0.41863	0.19789	0.4857	0.85644	-0.22357	0.52512	0.83957	0.9574	-0.0628	0.34781	0.96563	0.91579	-0.1269	0.13149	0.23654	1.1703	0.22693

162.1377	4.73	domics Pos	0.93764	0.99987	0.99214	-0.01139	0.62369	0.99994	0.9533	-0.069	0.93773	0.99996	0.9955	-0.00651	0.66997	0.95717	0.96333	-0.05389	0.042444	0.092215	0.84852	-0.23697	0.59972	0.78317	1.0691	0.09636	0.34408	0.74613	1.0904	0.12485	0.63472	0.97988	1.0538	0.075613	0.12788	0.23286	1.2486	0.3203
397.236	2.84	domics Pos	0.93794	0.99987	0.99517	-0.00698	0.38502	0.99994	0.87077	-0.19963	0.062956	0.99996	0.72482	-0.4643	0.022701	0.26882	0.56913	-0.81317	0.000816	0.004792	0.40875	-1.2907	0.005245	0.074135	0.35057	-1.5122	0.026505	0.34492	0.4518	-1.1462	0.031464	0.62711	0.48858	-1.0333	0.073909	0.1758	0.60626	-0.722
307.263	2.51	domics Pos	0.93801	0.99987	1.0469	0.066074	0.22014	0.99994	0.83723	-0.25631	0.074539	0.99996	0.69355	-0.52793	0.001058	0.17581	0.50621	-0.9822	0.000134	0.001257	0.25832	-1.9528	0.001597	0.052056	0.29533	-1.7596	0.001689	0.23318	0.28545	-1.8087	0.02531	0.56108	0.42609	-1.2308	0.078859	0.1809	0.58541	-0.77247
776.5597	5.55	domics Pos	0.93842	0.99987	1.0095	0.013573	0.83472	0.99994	0.98337	-0.02419	0.64038	0.99996	0.95636	-0.06438	0.22968	0.68665	0.88936	-0.16916	0.046727	0.09895	0.78744	-0.34476	0.3599	0.62208	0.94252	-0.08541	0.47319	0.808	1.0698	0.097288	0.84135	0.97988	1.0091	0.013069	0.18256	0.2898	1.1064	0.14586
480.3333	5.45	oolomics Pc	0.93859	0.99951	1.0607	0.085001	0.95169	0.99998	1.0333	0.04722	0.83478	0.99941	1.0727	0.10119	0.60187	0.99905	1.2731	0.34835	0.33372	0.71366	1.3499	0.43284	0.047008	0.1784	1.9885	0.9917	0.043426	0.41833	1.9991	0.99939	0.40099	0.99956	1.3158	0.39593	0.1807	0.44542	1.5445	0.62718
604.2942	2.36	oolomics Pc	0.93873	0.99951	1.0305	0.043349	0.7354	0.99998	0.828	-0.27229	0.52039	0.99941	0.70254	-0.50936	0.22896	0.67919	0.50963	-0.97248	0.007096	0.03713	0.12771	-2.9691	0.13192	0.36272	0.366	-1.4501	0.41272	0.98164	0.65276	-0.61538	0.55773	0.99956	0.78302	-0.35288	0.80676	0.92059	1.0534	0.075007
485.9782	12.02	olomics Ne	0.93953	0.99918	0.98775	-0.01778	0.18205	0.60009	0.79895	-0.32382	0.35432	0.99995	0.88023	-0.18404	0.74984	0.99985	0.96199	-0.05591	0.42493	0.99927	0.88395	-0.17797	0.54235	0.87267	0.9115	-0.13369	0.65189	0.87948	0.93091	-0.10328	0.025768	0.99512	0.70411	-0.50613	0.52492	0.99941	0.90246	-0.14806
1012.752	10.07	domics Pos	0.93998	0.99987	0.99787	-0.00308	0.82433	0.99994	0.99059	-0.01364	0.031758	0.99996	0.90474	-0.14442	0.89177	0.9874	0.99456	-0.00787	0.000858	0.004958	0.77771	-0.36269	0.42804	0.66265	0.91866	-0.12239	0.26652	0.70012	1.0352	0.049973	0.76348	0.97988	0.98905	-0.01588	0.022151	0.10842	1.1589	0.21278
620.2897	1.84	domics Pos	0.94006	0.99987	1.0373	0.05289	0.46347	0.99994	1.0828	0.11483	0.77981	0.99996	0.96509	-0.05126	0.99139	0.99715	0.98762	-0.01797	0.85694	0.87978	1.1373	0.18565	0.084678	0.30346	1.4375	0.52356	0.014059	0.33809	1.6496	0.72212	0.039955	0.64308	1.334	0.41578	0.007518	0.091944	1.6772	0.74605
1180.334	4.72	domics Pos	0.94047	0.99987	1.0024	0.003502	0.007807	0.99994	0.78615	-0.34713	0.028672	0.99996	0.87057	-0.19997	0.33291	0.79293	0.89803	-0.15516	0.007314	0.024187	0.71442	-0.48515	0.094315	0.32428	0.77157	-0.37413	0.68103	0.90385	0.9637	-0.05334	0.28124	0.94365	0.89626	-0.158	0.96014	0.97032	1.0117	0.016732
299.3126	2.84	domics Pos	0.94063	0.99987	1.0121	0.017388	0.52259	0.99994	0.93082	-0.10342	0.057526	0.99996	0.80065	-0.32075	0.038092	0.34165	0.82948	-0.26972	0.00669	0.022695	0.69967	-0.51525	0.00445	0.071311	0.59331	-0.75314	0.19529	0.64891	0.80091	-0.32028	0.035978	0.64014	0.77419	-0.36924	0.54541	0.64128	0.93154	-0.1023
220.2139	4.73	domics Pos	0.9408	0.99987	1.0187	0.026709	0.41915	0.99994	0.92092	-0.11885	0.18133	0.99996	1.1226	0.16688	0.52269	0.89938	0.9137	-0.13021	0.005955	0.020776	0.73495	-0.44429	0.5503	0.75179	0.92688	-0.10954	0.65652	0.89228	1.0448	0.063206	0.57007	0.9703	0.93824	-0.09198	0.78188	0.83839	0.97249	-0.04024
129.094	6.96	oolomics Pc	0.94122	0.99951	1.008	0.01154	0.5758	0.99998	0.91299	-0.13133	0.032149	0.99941	0.65051	-0.62036	0.015486	0.20428	0.47342	-1.0788	0.003408	0.022456	0.42764	-1.2255	0.001843	0.045425	0.34914	-1.5181	0.002481	0.20152	0.36322	-1.4611	0.014018	0.47452	0.37937	-1.3983	0.017946	0.2688	0.49633	-1.0106
736.5255	5.52	domics Pos	0.94125	0.99987	1.0903	0.12477	0.46602	0.99994	0.90862	-0.13825	0.35453	0.99996	0.86621	-0.20722	0.5083	0.89275	0.9137	-0.13021	0.11678	0.19565	0.77631	-0.3653	0.043104	0.19566	1.2957	0.37372	0.12621	0.58627	1.4952	0.58035	0.61678	0.97665	1.1764	0.23433	0.082498	0.18374	1.3708	0.45499
422.7626	4.78	domics Pos	0.94127	0.99987	1.0024	0.003489	0.87017	0.99994	0.96567	-0.0504	0.88362	0.99996	1.0026	0.003766	0.87301	0.9874	1.0033	0.004696	0.047414	0.10011	0.70749	-0.49921	0.36847	0.62799	0.83614	-0.25819	0.88982	0.97532	0.97315	-0.03927	0.76809	0.97988	0.94714	-0.07835	0.56408	0.6593	1.0975	0.13423
261.2193	1.83	domics Pos	0.94138	0.99987	1.0334	0.047394	0.47278	0.99994	0.83971	-0.25204	0.125	0.99996	0.6259	-0.67601	0.015802	0.24	0.42524	-1.2336	0.001032	0.0056	0.30938	-1.6925	0.018001	0.1154	0.38045	-1.3942	0.014443	0.33809	0.40472	-1.305	0.039115	0.64308	0.47977	-1.0596	0.11106	0.21467	0.57371	-0.8016
539.3118	1.11	domics Pos	0.94142	0.99987	1.0032	0.004557	0.97972	0.99994	0.89468	-0.16056	0.84967	0.99996	0.9899	-0.01465	0.71635	0.96449	1.1189	0.16203	0.5188	0.58997	1.3082	0.38756	0.037252	0.17601	2.5853	1.3704	0.019551	0.33809	3.3195	1.731	0.30157	0.95107	1.6174	0.69371	0.094019	0.19482	2.2003	1.1377
385.8896	10.11	domics Pos	0.94175	0.99987	1.006	0.008676	0.84554	0.99994	0.98404	-0.0232	0.9826	0.99996	1.0005	0.000757	0.024414	0.27679	0.8399	-0.25171	0.011223	0.033616	0.78867	-0.34251	0.24416	0.53144	0.87348	-0.19515	0.76388	0.94202	1.0267	0.037977	0.89029	0.98023	0.99337	-0.00959	0.26585	0.3761	1.2358	0.30543
632.3168	5.09	olomics Ne	0.94199	0.99918	0.98599	-0.02035	0.80378	0.97675	0.92787	-0.108	0.59955	0.99995	1.0991	0.13636	0.47067	0.99985	1.1705	0.22717	0.18521	0.99927	1.442	0.52811	0.032297	0.47167	1.8261	0.86877	0.014962	0.27907	2.0163	1.0117	0.19067	0.99512	1.3402	0.42245	0.15651	0.99941	1.4085	0.49415
428.2773	2.72	domics Pos	0.9422	0.99987	0.98479	-0.02211	0.35769	0.99994	0.8447	-0.24348	0.030645	0.99996	0.7013	-0.51189	0.056897	0.41167	0.64416	-0.6345	0.000661	0.004035	0.42944	-1.2195	0.004944	0.073217	0.45365	-1.1403	0.035869	0.36525	0.56191	-0.83159	0.014685	0.52955	0.48336	-1.0488	0.14269	0.24748	0.72569	-0.46257
265.2536	6.96	oolomics Pc	0.94308	0.99951	0.998	-0.00289	0.50629	0.99998	0.89053	-0.16726	0.024811	0.99941	0.6631	-0.5927	0.015733	0.20428	0.48646	-1.0396	0.004903	0.028923	0.44932	-1.1542	0.003484	0.050573	0.35293	-1.5026	0.003297	0.20152	0.375	-1.415	0.022336	0.51556	0.42494	-1.2347	0.016853	0.2688	0.45827	-1.1257
1199.854	9.56	oolomics Pc	0.94311	0.99951	1.0279	0.039725	0.21221	0.99998	0.8098	-0.30435	0.6169	0.99941	0.92697	-0.1094	0.025212	0.23087	0.64584	-0.63074	0.052848	0.17505	0.73165	-0.45078	0.056139	0.20141	0.75346	-0.40839	0.68765	0.99391	0.9379	-0.09249	0.49673	0.99956	0.88926	-0.16932	0.18145	0.44585	1.3933	0.47853
1050.764	10.07	domics Pos	0.94346	0.99987	1.0017	0.002474	0.79713	0.99994	0.98855	-0.01662	0.32425	0.99996	0.8948	-0.16037	0.001342	0.17581	0.8411	-0.24965	0.002778	0.011524	0.77797	-0.36221	0.004872	0.072843	0.8282	-0.27194	0.68959	0.90885	0.97176	-0.04133	0.067027	0.76335	0.87324	-0.19556	0.096743	0.19779	1.1211	0.16487
239.15	6.2	oolomics Pc	0.94389	0.99951	0.99432	-0.00821	0.90061	0.99998	0.99053	-0.01373	0.040524	0.99941	0.84668	-0.24012	0.37129	0.86634	1.0745	0.10365	0.23236	0.55324	1.068	0.094972	0.7573	0.93161	1.0204	0.029098	0.34892	0.9578	0.94118	-0.08746	0.6584	0.99956	0.96386	-0.05311	0.16358	0.42247	0.91365	-0.13028
436.283	5.29	oolomics Pc	0.94393	0.99951	0.88883	-0.17003	0.98204	0.99998	0.9474	-0.07796	0.82462	0.99941	0.99144	-0.0124	0.61909	0.99905	1.1283	0.17414	0.62302	0.97726	1.0847	0.11723	0.054907	0.19857	1.9648	0.97438	0.025963	0.3338	2.3804	1.2512	0.13468	0.91827	1.5838	0.66337	0.10645	0.36133	1.6938	0.76027
328.2482	3.7	oolomics Pc	0.9442	0.99951	1.0428	0.060407	0.40577	0.99998	0.81948	-0.28722	0.41562	0.99941	1.23	0.29867	0.6531	0.99905	1.1503	0.20199	0.35694	0.74758	0.77801	-0.36215	0.039837	0.15965	0.4079	-1.2937	0.074146	0.55478	0.46678	-1.0992	0.21321	0.99956	0.64917	-0.62334	0.015528	0.2688	0.39232	-1.3499
600.2806	1.11	domics Pos	0.94453	0.99987	1.0273	0.038801	0.75932	0.99994	1.0611	0.085556	0.90529	0.99996	1.0105	0.015041	0.83611	0.98621	1.0083	0.011892	0.76331	0.79696	1.055	0.077197	0.085647	0.30578	1.426	0.51198	0.037951	0.37121	1.6235	0.69915	0.25048	0.94365	1.3074	0.38667	0.060836	0.1578	1.5555	0.63738
577.5189	5.89	domics Pos	0.94456	0.99987	1.0068	0.009806	0.87043	0.99994	0.96593	-0.05	0.97614	0.99996	0.98258	-0.02535	0.92302	0.9874	0.99787	-0.00307	0.55086	0.61664	1.1077	0.14762	0.009537	0.087209	1.8827	0.91283	0.020464	0.33809	1.9997	0.99981	0.051819	0.68583	1.6974	0.76331	0.008236	0.092094	2.8086	1.4898
369.9835	4.25	olomics Ne	0.94467	0.99918	1.0101	0.014514	0.046302	0.47152	0.88463	-0.17686	0.58325	0.99995	1.0451	0.063599	0.55803	0.99985	1.0499	0.070219	0.60231	0.99927	1.0274	0.039042	0.60491	0.90369	1.0557	0.078228	0.53702	0.84256	1.0677	0.094505	0.68676	0.99512	1.0337	0.047832	0.61221	0.99941	1.0211	0.030178
590.3217	5.08	oolomics Pc	0.94477	0.99951	1.0899	0.12413	0.74343	0.99998	1.0742	0.10331	0.15801	0.99941	1.1791	0.23769	0.31552	0.81063	1.1864	0.24659	0.081589	0.24453	1.267	0.3414	0.016316	0.094477	1.5342	0.61751	0.000958	0.16989	1.7702	0.8239	0.20714	0.99956	1.1764	0.23434	0.51582	0.76223	1.1201	0.16364
810.5978	5.72	domics Pos	0.94499	0.99987	1.0046	0.006605	0.48479	0.99994	0.95226	-0.07057	0.38719	0.99996	0.94518	-0.08134	0.19052	0.63224	0.892	-0.16489	0.006034	0.020953	0.76823	-0.3804	0.3591	0.62208	0.93931	-0.09033	0.96935	0.99319	1.0085	0.012152	0.89855	0.98023	1.0163	0.023379	0.028279	0.1173	1.722	0.78408
282.8013	0.35	olomics Ne	0.94513	0.99918	0.99774	-0.00327	0.62409	0.9081	0.96828	-0.0465	0.67582	0.99995	1.075	0.10428	0.8992	0.99985	1.0404	0.057094	0.25198	0.99927	1.0941	0.12978	0.007837	0.36417	1.2288	0.29725	0.069889	0.41438	1.2037	0.2675	0.35339	0.99512	1.0742	0.10331	0.67694	0.99941	1.0431	0.06093
547.3589	5.31	oolomics Pc	0.94583	0.99951	1.0793	0.11014	0.91716	0.99998	1.032	0.045407	0.62951	0.99941	1.1465	0.19727	0.44887	0.92618	1.2327	0.30183	0.27407	0.63108	1.484	0.56951	0.006534	0.067169	2.0048	1.0034	0.004734	0.20152	2.0214	1.0154	0.54877	0.99956	1.1319	0.1787	0.21963	0.48775	1.3905	0.47557
551.3121	1.1	domics Pos	0.94609	0.99987	1.0457	0.064467	0.92949	0.99994	0.99831	-0.00243	0.38618	0.99996	1.1867	0.24691	0.91992	0.9874	0.97287	-0.03968	0.6812	0.72942	0.8895	-0.16894	0.06841	0.26634	1.502	0.58684	0.031561	0.35923	1.8548	0.89125	0.44742	0.96776	1.1597	0.21371	0.035341	0.12781	1.6159	0.69235
296.0011	0.56	domics Pos	0.94609	0.99987	1.0016	0.00226	0.4634	0.99994	0.87855	-0.18681	0.40573	0.99996	1.1876	0.24805	0.06688	0.43947	1.5975	0.67578	0.204	0.29806	1.2753	0.35081	0.037635	0.17738	1.518	0.60215	0.007672	0.32129	2.005	1.0036	0.11473	0.88537	1.4805	0.5661	0.097828	0.19894	1.4877	0.57313
875.5568	5.09	domics Pos	0.94637	0.99987	0.9962	-0.00549	0.20115	0.99994	0.85853	-0.22006	0.8321	0.99996	0.98742	-0.01827	0.51009	0.89383	0.93145	-0.10245	0.020974	0.054304	0.78987	-0.34032	0.42899	0.66304	0.89819	-0.15491	0.9576	0.98985	1.0062	0.008919	0.39359	0.96776	0.94114	-0.08752	0.007954	0.092077	1.3907	0.47585
456.2867	2.85	domics Pos	0.9467	0.99987	0.9982	-0.0026	0.60348	0.99994	0.91037	-0.13548	0.083018	0.99996	0.74306	-0.42845	0.021718	0.26563	0.55728	-0.84354	0.000896	0.005136	0.45069	-1.1498	0.005395	0.074533	0.42971	-1.2186	0.009293	0.3318	0.44139	-1.1799	0.035708	0.64014	0.51025	-0.97073	0.079395	0.18125	0.65225	-0.6165
804.8404	0.5	domics Pos	0.94698	0.99987	1.0108	0.015492	0.3539	0.99994	0.9533	-0.069	0.010664	0.99996	0.89108	-0.16637	0.012899	0.22771	0.84348	-0.24557	0.000335	0.002409	0.71926	-0.47541	0.10164	0.33938	0.8108	-0.30258	0.085294	0.50896	0.83563	-0.25906	0.10589	0.86277	0.87587	-0.19121	0.10002	0.2019	1.1054	0.1446
544.3389	1.08	domics Pos	0.94786	0.99987	1.0224	0.031928	0.78945	0.99994	1.0395	0.05591	0.61411	0.99996	1.0946	0.13037	0.62587	0.94364	1.0739	0.10282	0.76779	0.80076	0.92901	-0.10624	0.079231	0.29123	1.4619	0.54783	0.02305	0.33809	1.7382	0.79757	0.41434	0.96776	1.1244	0.16913	0.074605	0.1768	1.4465	0.53256
647.5556	6.5	domics Pos	0.9485	0.99987	0.95903	-0.06036	0.63583	0.99994	0.86165	-0.21482	0.60213	0.99996	0.85822	-0.22057	0.30184	0.76019	0.73525	-0.44368	0.43468	0.51957	0.79424	-0.33236	0.26614	0.55389	1.4099	0.49562	0.010644	0.3324	2.3405	1.2268	0.42424	0.96776	1.2147	0.28055	0.44593	0.55088	1.3435	0.42595
213.1864	6.96	oolomics Pc	0.94852	0.99951	0.98781	-0.01769	0.56871	0.99998	0.90972	-0.1365	0.047586	0.99941	0.68317	-0.54968	0.033156	0.25203	0.49092	-1.0265	0.009928	0.048113	0.47321	-1.0794	0.001904	0.045425	0.33954	-1.5583	0.005236	0.20617	0.38506	-1.3768	0.021952	0.51556	0.44865	-1.1563	0.023485	0.2688	0.48619	-1.0404
501.2788	1.15	domics Pos	0.94885	0.99987	0.96664	-0.04895	0.76206	0.99994	0.8868	-0.17333	0.89179	0.99996	0.97655	-0.03424	0.46999	0.88476	1.299	0.37739	0.10376	0.17893	1.945	0.95974	0.00448	0.071311	3.0104	1.5899	0.002603	0.26175	3.4172	1.7728	0.25378	0.94365	1.8703	0.90325	0.059556	0.15715	2.3031	1.2036
185.1546	6.96	oolomics Pc	0.94935	0.99951	1.0232	0.033127	0.76805	0.99998	0.9602	-0.05859	0.03573	0.99941	0.67145	-0.57465	0.03295	0.25203	0.50756	-0.97834	0.002765	0.019277	0.41828	-1.2574	0.005387	0.062645	0.38044	-1.3943	0.003425	0.20152	0.37763	-1.405	0.02277	0.51791	0.44459	-1.1695	0.028754	0.2688	0.48061	-1.0571
1085.647	5.1	oolomics Pc	0.94944	0.99951	1.1617	0.21619	0.67121	0.99998	0.86667	-0.20645	0.88559	0.99941	0.90545	-0.1433	0.13965	0.52592	1.6235	0.69913	0.13042	0.35692	1.722	0.78411	0.024612	0.11658	2.5375	1.3434	0.019409	0.30136	2.5844	1.3699	0.082684	0.78717	1.7512	0.80834	0.21171	0.47874	1.544	0.62663
319.1952	4.96	domics Pos	0.94978	0.99987	0.99459	-0.00783	0.25266	0.99994	0.89848	-0.15445	0.50013	0.99996	0.94036	-0.08872	0.22776	0.68384	0.87544	-0.19192	0.012085	0.035446	0.71145	-0.49116	0.40055	0.64773	0.92818	-0.10753	0.24863	0.68548	1.1032	0.14173	0.63096	0.97988	1.0379	0.053613	0.15516	0.26108	1.2049	0.26894
522.356	5.45	oolomics Pc	0.94982	0.99951	1.0233	0.033245	0.98978	0.99998	0.99242	-0.01098	0.93616	0.99941	0.99902	-0.00142	0.84632	0.99905	1.0496	0.069877	0.61788	0.97478	1.1322	0.17912	0.074732	0.24426	1.7126	0.77616	0.04501	0.4245	1.8227	0.86607	0.50107	0.99956	1.2029	0.26654	0.14154	0.39464	1.7393	0.79854
96.0788	0.58	domics Pos	0.94988	0.99987	1.0079	0.011382	0.79268	0.99994	0.98228	-0.0258	0.84603	0.99996	1.0285	0.040517	0.56651	0.9122	1.1213	0.16516	0.14043	0.22499	1.2237	0.29131	0.030433	0.15584	1.6578	0.72928	0.002818	0.26175	1.9188	0.94023	0.16441	0.91806	1.426	0.51193	0.1053	0.20751	1.297	0.37515
643.5269	12.02	oolomics Pc	0.95018	0.99951	1.0045	0.006479	0.20535	0.99998	0.83011	-0.26862	0.26519	0.99941	0.84423	-0.24429	0.8915	0.99905	1.0127	0.018167	0.082333	0.24562	0.71529	-0.4834	0.001399	0.045425	0.62535	-0.67727	0.064634	0.51678	0.80707	-0.30923	0.10488	0.84495	0.86535	-0.20865	0.47172	0.73199	0.9153	-0.12769
111.0215	8.23	oolomics Pc	0.95054	0.99951	1.0016	0.002251	0.87648	0.99998	1.0122	0.017485	0.63005	0.99941	0.95242	-0.07033	0.72286	0.99905	0.97935	-0.0301	0.72909	0.99688	1.0237	0.033863	0.68628	0.90883	1.018	0.025689	0.020652	0.30978	1.1316	0.17839	0.63906	0.99956	1.0258	0.036737	0.44064	0.70925	1.0659	0.092072
572.3611	1.12	domics Pos	0.95099	0.99987	1.0887	0.12261	0.68173	0.99994	1.0605	0.084739	0.2917	0.99996	1.2322	0.30129	0.89864	0.9874	1.0405	0.057286	0.41246	0.50118	0.81136	-0.30159	0.22186	0.51169	1.5192	0.60335	0.010797	0.3324	2.0264	1.0189	0.75854	0.97988	1.0705	0.098334	0.069376	0.17088	1.4193	0.50518
925.7241	7.43	domics Pos	0.95137	0.99987	0.9334	-0.09943	0.95779	0.99994	0.95949	-0.05966	0.82718	0.99996	0.90931	-0.13716	0.52301	0.89938	0.80311	-0.31634	0.15545	0.24372	0.60559	-0.7236	0.15914	0.44066	0.54919	-0.86462	0.21741	0.66052	0.59273	-0.75455	0.045264	0.65624	0.37177	-1.4275	0.084004	0.18577	0.43747	-1.1927
139.1122	6.97	oolomics Pc	0.9514	0.99951	1.0082	0.011809	0.42176	0.99998	0.87477	-0.19303	0.026805	0.99941	0.69893	-0.51679	0.010307	0.19754	0.46245	-1.1126	0.001345	0.011665	0.40726	-1.296	0.001106	0.044437	0.34799	-1.5229	0.001328	0.16989	0.40852	-1.2915	0.016446	0.49126	0.46135	-1.1161	0.029902	0.2688	0.57433	-0.80004
398.2454	2.84	domics Pos	0.95145	0.99987	1.0212	0.03028	0.27145	0.99994	0.87521	-0.19229	0.020761	0.99996	0.74809	-0.41872	0.015973	0.24	0.60256	-0.73083	0.000316	0.002305	0.42245	-1.2431	0.019383	0.11985	0.44713	-1.1612	0.007848	0.32129	0.45476	-1.1368	0.008206	0.52955	0.50607	-0.9826	0.076506	0.17807	0.66793	-0.58224
558.6266	0.56	lomics Neg	0.95151	0.99977	0.99686	-0.00453	0.60666	0.99904	1.0881	0.12179	0.90508	0.99994	0.9921	-0.01144	0.11842	0.58182	1.1968	0.25917	0.28893	0.99989	1.1189	0.16205	0.82835	0.99653	1.0161	0.022992	0.042158	0.38004	0.73709	-0.44008	0.2652	0.64614	0.84586	-0.24151	0.052187	0.405	0.75217	-0.41087
724.5267	8.87	oolomics Pc	0.95179	0.99951	1.006	0.00869	0.84464	0.99998	0.98113	-0.02748	0.40909	0.99941	0.92545	-0.11177	0.54994	0.99905	0.93927	-0.09039	0.96634	0.9989	0.99008	-0.01438	0.1923	0.46525	1.1974	0.2599	0.026798	0.3363	1.4025	0.48805	0.29281	0.99956	1.0951	0.13111	0.052803	0.2951	1.1955	0.25758
579.2933	1.95	domics Pos	0.95213	0.99987	1.0503	0.07083	0.59752	0.99994	0.89987	-0.15221	0.69337	0.99996	1.049	0.068994	0.77076	0.97526	1.0484	0.068157	0.97272	0.97682	1.0017	0.002466	0.025143	0.13997	1.8958	0.92278	0.065688	0.46042	1.6933	0.75986	0.096256	0.84751	1.3942	0.47945	0.02411	0.11079	1.6445	0.71768
394.2171	2.28	domics Pos	0.95325	0.99987	1.0169	0.024196	0.40424	0.99994	0.87713	-0.18913	0.078898	0.99996	0.76102	-0.39399	0.019384	0.25663	0.58442	-0.77491	0.000366	0.002582	0.47256	-1.0814	0.022848	0.1324	0.47023	-1.0886	0.029045	0.3527	0.53513	-0.90203	0.021688	0.53012	0.48563	-1.0421	0.06991	0.17098	0.59787	-0.74209
1160.835	4.95	domics Pos	0.95353	0.99987	1.0118	0.016878	0.45522	0.99994	0.93098	-0.10318	0.18097	0.99996	0.89749	-0.15604	0.74854	0.97379	0.98087	-0.02787	0.000672	0.004073	0.70389	-0.50657	0.4859	0.705	0.92649	-0.11016	0.60833	0.86907	0.96618	-0.04963	0.40637	0.96776	0.97375	-0.03837	0.016913	0.099826	1.2713	0.34633
804.5496	4.61	domics Pos	0.95362	0.99987	1.006	0.008697	0.74606	0.99994	0.94552	-0.08082	0.83745	0.99996	0.96404	-0.05284	0.7021	0.96092	0.94097	-0.08778	0.68773	0.73536	0.93582	-0.0957	0.074466	0.28141	1.305	0.3841	0.045278	0.39237	1.4242	0.51014	0.40868	0.96776	1.1753	0.23305	0.047355	0.14388	1.4759	0.56159
545.3409	5.45	oolomics Pc	0.95366	0.99951	1.0733	0.10207	0.9843	0.99998	1.0294	0.041773	0.81333	0.99941	1.0533	0.074946	0.75142	0.99905	1.1098	0.15026	0.62225	0.97663	1.1672	0.22309	0.046617	0.17735	2.026	1.0186	0.035296	0.37184	2.12	1.0841	0.38768	0.99956	1.4274	0.51344	0.12754	0.37779	1.9792	0.98495
858.6291	5.72	lomics Neg	0.95384	0.99977	1.157	0.21034	0.96607	0.99904	1.0771	0.10711	0.42181	0.99994	0.84236	-0.24749	0.33848	0.58182	1.3458	0.42849	0.97922	0.99989	1.4929	0.5781	0.91626	0.99653	1.296	0.37402	0.04723	0.38213	0.61726	-0.69605	0.044784	0.46971	0.50015	-0.99958	0.28929	0.49786	1.4153	0.50107
367.1953	5.23	domics Pos	0.95421	0.99987	0.98963	-0.01503	0.58716	0.99994	0.91386	-0.12996	0.72211	0.99996	1.045	0.063564	0.80312	0.97856	0.95442	-0.06731	0.031011	0.072721	0.69466	-0.52562	0.48355	0.70292	0.88679	-0.17334	0.94153	0.98682	0.97702	-0.03354	0.74485	0.97988	0.95105	-0.0724	0.44472	0.55012	1.0961	0.13234
374.8851	10.15	domics Pos	0.9554	0.99987	0.99806	-0.0028	0.36935	0.99994	0.94599	-0.08011	0.58302	0.99996	1.0389	0.055062	0.11169	0.53238	0.87173	-0.19804	0.00351	0.013799	0.78298	-0.35294	0.075462	0.28376	0.87256	-0.19667	0.71541	0.9194	1.0329	0.046717	0.89325	0.98023	0.99283	-0.01039	0.14298	0.24751	1.142	0.19152
328.2304	2.85	domics Pos	0.95556	0.99987	0.99227	-0.0112	0.41499	0.99994	0.89604	-0.15836	0.22062	0.99996	0.82394	-0.27939	0.042806	0.3611	0.56404	-0.82612	0.000208	0.00174	0.41571	-1.2663	0.012137	0.093	0.44822	-1.1577	0.01115	0.3324	0.54387	-0.87868	0.049556	0.6775	0.57937	-0.78744	0.21026	0.31909	0.74937	-0.41625
899.7148	10.42	oolomics Pc	0.95654	0.99951	1.0066	0.009467	0.63016	0.99998	0.91754	-0.12416	0.26229	0.99941	0.77429	-0.36906	0.54269	0.99905	0.8902	-0.16779	0.47488	0.87375	0.86468	-0.20977	0.77454	0.93515	1.1508	0.20266	0.67451	0.99391	0.9337	-0.09897	0.004249	0.47452	0.53043	-0.91476	0.25816	0.52943	0.78471	-0.34977
570.354	1.13	domics Pos	0.95657	0.99987	1.0389	0.055025	0.70041	0.99994	1.0588	0.082375	0.53048	0.99996	1.1069	0.1465	0.68288	0.95954	1.0515	0.072496	0.67327	0.72254	0.8892	-0.16941	0.067743	0.26455	1.5727	0.65321	0.016983	0.33809	1.994	0.99563	0.37047	0.96776	1.1795	0.23813	0.0361	0.12908	1.5566	0.63837
752.5578	5.66	domics Pos	0.9566	0.99987	1.0037	0.005379	0.15575	0.99994	0.90487	-0.14422	0.20405	0.99996	0.89718	-0.15653	0.060673	0.42527	0.84415	-0.24443	0.011689	0.03455	0.78534	-0.3486	0.31209	0.59087	1.113	0.15445	0.023947	0.33809	1.3394	0.42154	0.037051	0.64014	1.1754	0.23311	0.006522	0.090084	1.3212	0.4018
1119.609	1.11	domics Pos	0.95664	0.99987	0.83096	-0.26715	0.96243	0.99994	0.79927	-0.32324	0.74795	0.99996	0.88718	-0.17271	0.79074	0.97671	0.90617	-0.14214	0.47425	0.55264	1.3516	0.43462	0.051035	0.22138	3.0457	1.6068	0.041033	0.38161	3.3055	1.7249	0.31886	0.9524	1.864	0.8984	0.14215	0.24715	2.0703	1.0498
296.8672	10.16	domics Pos	0.95672	0.99987	1.0006	0.000918	0.81371	0.99994	0.97618	-0.03478	0.98252	0.99996	0.99204	-0.01153	0.53482	0.90073	0.94824	-0.07667	0.042418	0.092215	0.80877	-0.30619	0.35591	0.62052	0.89672	-0.15727	0.80321	0.95444	1.0179	0.02558	0.8029	0.97988	0.97554	-0.03572	0.052036	0.14851	1.1963	0.25856
264.9245	10.11	domics Pos	0.95677	0.99987	1.0061	0.008775	0.76838	0.99994	0.98811	-0.01726	0.25737	0.99996	0.93685	-0.09412	0.42547	0.86135	0.94884	-0.07577	0.00029	0.002152	0.73053	-0.45298	0.33037	0.60476	0.88682	-0.17329	0.47051	0.80647	0.97058	-0.04308	0.5217	0.96776	0.95572	-0.06535	0.11425	0.21876	1.1577	0.21123
145.0178	10.23	domics Pos	0.95687	0.99987	0.99999	-2.1E-05	0.080672	0.99994	0.92132	-0.11823	0.83153	0.99996	0.99228	-0.01118	0.18458	0.62505	0.93301	-0.10004	5.64E-05	0.000707	0.79011	-0.33988	0.35419	0.6194	0.92562	-0.11151	0.84206	0.96746	1.0127	0.018167	0.95368	0.98838	1.0055	0.007894	0.004256	0.085451	1.2261	0.29404
560.8642	10.15	domics Pos	0.95794	0.99987	1.0048	0.006945	0.61906	0.99994	0.95453	-0.06714	0.19552	0.99996	0.86999	-0.20092	0.24471	0.70602	0.89696	-0.15689	0.001427	0.007084	0.72235	-0.46923	0.29157	0.57461	0.86566	-0.20813	0.27521	0.70632	0.89584	-0.15869	0.076594	0.78721	0.8439	-0.24485	0.03546	0.12781	1.2123	0.27775
335.1649	2.28	domics Pos	0.95839	0.99987	1.0004	0.000515	0.46139	0.99994	0.89099	-0.16652	0.11243	0.99996	0.74332	-0.42794	0.024786	0.27831	0.60098	-0.73462	0.000275	0.002086	0.45084	-1.1493	0.015207	0.10398	0.46858	-1.0936	0.026713	0.34512	0.52455	-0.93086	0.03339	0.6327	0.51636	-0.95356	0.087177	0.1878	0.63491	-0.65538
304.238	6.03	oolomics Pc	0.95861	0.99951	1.0264	0.037566	0.85617	0.99998	1.069	0.096266	0.28885	0.99941	0.73999	-0.43443	0.072816	0.35072	0.59542	-0.74802	0.001495	0.012627	0.28181	-1.8272	0.003333	0.049867	0.37003	-1.4343	0.009682	0.24769	0.43526	-1.2001	0.034765	0.6084	0.48991	-1.0294	0.034939	0.272	0.49712	-1.0083
421.3265	2.72	domics Pos	0.95871	0.99987	0.99473	-0.00762	0.43647	0.99994	0.88226	-0.18072	0.20168	0.99996	0.79583	-0.32946	0.004991	0.17581	0.59688	-0.7445	0.001491	0.007308	0.46282	-1.1115	0.018925	0.11791	0.42706	-1.2275	0.030667	0.35576	0.56679	-0.81911	0.018866	0.52955	0.54604	-0.87293	0.15763	0.26419	0.69854	-0.51759
339.1649	6.48	oolomics Pc	0.95872	0.99951	1.0142	0.020276	0.59848	0.99998	0.9299	-0.10485	0.089806	0.99941	0.77278	-0.37186	0.077007	0.36243	0.65016	-0.62114	0.002104	0.016046	0.55741	-0.84319	0.011771	0.083709	0.4859	-1.0413	0.018027	0.30136	0.52081	-0.94118	0.006779	0.47452	0.47752	-1.0664	0.031237	0.2688	0.51612	-0.95423
702.3212	5.81	domics Pos	0.95874	0.99987	1.0137	0.019596	0.2976	0.99994	0.84145	-0.24906	0.26482	0.99996	0.82064	-0.28518	0.35242	0.80737	0.86813	-0.20401	0.048839	0.10249	0.72278	-0.46836	0.33351	0.6073	0.82104	-0.28448	0.37339	0.76056	0.84542	-0.24226	0.66008	0.97988	0.92968	-0.10519	0.46422	0.56734	0.88549	-0.17545
964.7856	0.53	domics Pos	0.95905	0.99987	0.99758	-0.0035	0.13277	0.99994	0.79611	-0.32897	0.67253	0.99996	0.94111	-0.08756	0.40071	0.84516	0.89865	-0.15416	0.04604	0.097855	0.71494	-0.4841	0.50327	0.71871	0.90032	-0.15149	0.87804	0.97532	0.97588	-0.03523	0.2735	0.94365	0.85554	-0.22509	0.35182	0.46131	1.1273	0.17289
952.7434	7.46	domics Pos	0.95915	0.99987	0.88248	-0.18037	0.83013	0.99994	0.86211	-0.21406	0.96417	0.99996	0.91079	-0.13481	0.62971	0.94679	0.76497	-0.38653	0.28611	0.38038	0.5973	-0.74347	0.32958	0.60418	0.62044	-0.68864	0.30013	0.72384	0.61478	-0.70185	0.048143	0.67624	0.33189	-1.5912	0.11232	0.21594	0.42111	-1.2477
460.2828	1.16	domics Pos	0.95944	0.99987	0.9974	-0.00375	0.96872	0.99994	0.98299	-0.02475	0.80759	0.99996	1.0675	0.094227	0.54093	0.90385	1.3549	0.4382	0.14639	0.23296	1.9104	0.93391	0.0047	0.071391	3.2492	1.7001	0.004836	0.3088	3.7504	1.9071	0.20889	0.92646	1.7482	0.80584	0.040311	0.13378	2.1395	1.0973
285.9853	0.57	domics Pos	0.95978	0.99987	1.0106	0.015156	0.21652	0.99994	0.85073	-0.23323	0.77816	0.99996	1.0467	0.065896	0.13734	0.56129	1.3925	0.47769	0.56526	0.62948	1.0682	0.095128	0.23184	0.51881	1.1343	0.18177	0.028963	0.3527	1.5209	0.6049	0.52444	0.96776	1.1694	0.22573	0.11975	0.22394	1.3098	0.38935
586.2749	1.91	domics Pos	0.95979	0.99987	1.0275	0.039111	0.34236	0.99994	1.24	0.31039	0.10926	0.99996	1.3318	0.41342	0.48222	0.88699	1.1934	0.25511	0.038575	0.086117	0.6291	-0.66863	0.52325	0.73163	0.86824	-0.20383	0.4702	0.80647	0.84848	-0.23705	0.36735	0.96776	0.80862	-0.30646	0.42871	0.53509	1.153	0.2054
1170.746	10.03	domics Pos	0.9601	0.99987	1.0021	0.003044	0.35342	0.99994	0.92272	-0.11604	0.80786	0.99996	0.97511	-0.03637	0.39195	0.83664	0.92472	-0.11292	0.02763	0.06668	0.79368	-0.33337	0.18993	0.47645	0.86325	-0.21216	0.83347	0.96621	0.97827	-0.03169	0.91094	0.98248	0.98565	-0.02085	0.24335	0.35174	1.1401	0.18915
466.3297	2.2	domics Pos	0.96015	0.99987	0.99769	-0.00333	0.98421	0.99994	0.98089	-0.02784	0.53785	0.99996	0.85875	-0.21968	0.78034	0.97526	0.91753	-0.12417	0.005495	0.019605	0.30154	-1.7296	0.6653	0.82571	0.89419	-0.16135	0.13277	0.59878	1.4253	0.51123	0.43099	0.96776	1.1635	0.21848	0.054221	0.15157	1.6731	0.74256
955.5285	5.83	domics Pos	0.96025	0.99987	0.99741	-0.00374	0.78476	0.99994	0.98217	-0.02595	0.76447	0.99996	0.97977	-0.02948	0.49005	0.88874	0.94536	-0.08107	0.020528	0.053428	0.82625	-0.27536	0.14744	0.42234	0.89755	-0.15593	0.44989	0.80156	0.96247	-0.05519	0.64767	0.97988	0.96449	-0.05216	0.06241	0.16008	1.2438	0.31475
363.1706	6.42	oolomics Pc	0.96122	0.99951	1.0178	0.025452	0.38907	0.99998	0.8885	-0.17056	0.28534	0.99941	0.88816	-0.17111	0.072751	0.35072	0.82963	-0.26945	4.1E-05	0.000792	0.29533	-1.7596	0.015266	0.092479	0.62522	-0.67756	0.09286	0.60726	0.74281	-0.42894	0.016531	0.49126	0.69295	-0.52918	0.12281	0.37265	0.74814	-0.41862
1174.746	10.02	domics Pos	0.96136	0.99987	1.0014	0.002025	0.29897	0.99994	0.9348	-0.09727	0.40524	0.99996	0.92869	-0.10673	0.049579	0.37885	0.82849	-0.27145	0.004344	0.016287	0.68419	-0.54754	0.2699	0.55719	0.887	-0.173	0.83393	0.96621	1.0321	0.045532	0.71878	0.97988	0.97291	-0.03962	0.024724	0.1117	1.202	0.26539
1127.764	10.03	domics Pos	0.96138	0.99987	1.004	0.005784	0.23133	0.99994	0.91378	-0.13008	0.53276	0.99996	0.94905	-0.07544	0.22896	0.68626	0.90959	-0.13671	0.02708	0.065643	0.80907	-0.30566	0.23912	0.52848	0.86646	-0.2068	0.45509	0.80225	0.95116	-0.07224	0.41658	0.96776	0.94047	-0.08854	0.057006	0.15429	1.1529	0.20531
367.3364	8.19	oolomics Pc	0.9614	0.99951	0.99087	-0.01323	0.85274	0.99998	0.97504	-0.03646	0.61077	0.99941	0.94104	-0.08768	0.94794	0.99905	1.0061	0.008753	0.21554	0.52349	1.1463	0.19701	0.082306	0.26092	1.2962	0.37427	0.030541	0.35049	1.4656	0.55152	0.62691	0.99956	1.0785	0.10899	0.50913	0.75792	1.079	0.10972
1177.568	1.11	domics Pos	0.96156	0.99987	0.80549	-0.31207	0.94277	0.99994	0.86496	-0.2093	0.49351	0.99996	1.3139	0.39389	0.71064	0.96346	0.971	-0.04245	0.4611	0.54164	1.2729	0.34817	0.049351	0.21629	2.5436	1.3469	0.034746	0.36369	2.8667	1.5194	0.22152	0.94047	1.738	0.79739	0.12132	0.22566	1.9704	0.97847
341.9307	10.14	domics Pos	0.96161	0.99987	0.99635	-0.00527	0.57251	0.99994	0.96549	-0.05067	0.32063	0.99996	0.92367	-0.11455	0.56337	0.91124	0.95906	-0.06031	0.001307	0.006671	0.73403	-0.44609	0.67553	0.83208	0.96461	-0.05198	0.1562	0.6285	0.86308	-0.21243	0.020245	0.52955	0.8355	-0.2593	0.034976	0.12725	1.1462	0.1969
840.6091	5.59	lomics Neg	0.96166	0.99977	1.0591	0.082873	0.71799	0.99904	0.88462	-0.17687	0.19269	0.99994	0.63971	-0.64451	0.44694	0.60735	1.2033	0.26694	0.90146	0.99989	1.3681	0.45214	0.91473	0.99653	1.1055	0.14476	0.14134	0.45153	0.60271	-0.73047	0.047552	0.46971	0.44792	-1.1587	0.93314	0.96195	0.97331	-0.03902
499.3337	3.52	domics Pos	0.96205	0.99987	0.98509	-0.02168	0.072911	0.99994	0.7311	-0.45185	0.27567	0.99996	0.83163	-0.26598	0.020256	0.25894	0.53758	-0.89546	0.001464	0.007238	0.43706	-1.1941	0.006156	0.077983	0.35973	-1.475	0.005965	0.3088	0.5119	-0.96607	0.00766	0.52955	0.51443	-0.95897	0.071037	0.17215	0.67202	-0.57342
885.6747	7.32	domics Pos	0.96236	0.99987	1.0463	0.065342	0.80728	0.99994	1.0755	0.10499	0.8825	0.99996	1.0298	0.042309	0.48826	0.88725	0.84069	-0.25035	0.10787	0.18419	0.6611	-0.59706	0.035236	0.16986	0.48035	-1.0578	0.010556	0.3324	0.46255	-1.1123	0.0366	0.64014	0.50283	-0.99186	0.33803	0.44816	0.77049	-0.37616
367.1932	6.11	oolomics Pc	0.96266	0.99951	1.0108	0.015464	0.88736	0.99998	1.1191	0.16232	0.15436	0.99941	0.65784	-0.60419	0.079182	0.36897	0.50472	-0.98645	0.38277	0.78047	0.81835	-0.28921	0.045326	0.17359	0.45687	-1.1301	0.071185	0.54032	0.58606	-0.77089	0.023028	0.51791	0.41993	-1.2518	0.065193	0.30704	0.4657	-1.1025
592.3353	1.12	domics Pos	0.96272	0.99987	1.0224	0.031982	0.5424	0.99994	1.1373	0.18559	0.56289	0.99996	1.1178	0.16068	0.6637	0.95717	1.0565	0.07925	0.48846	0.56422	0.83971	-0.25204	0.054669	0.231	1.6031	0.68089	0.017561	0.33809	1.9126	0.93556	0.2636	0.94365	1.2859	0.36274	0.023608	0.11051	1.6141	0.69077
546.3557	5.31	oolomics Pc	0.96282	0.99951	1.0414	0.058541	0.71521	0.99998	1.0923	0.12736	0.64269	0.99941	1.1223	0.16643	0.41904	0.90805	1.2436	0.31452	0.31938	0.69313	1.3642	0.44804	0.011496	0.083709	1.9428	0.95814	0.010373	0.24769	1.9522	0.96512	0.62191	0.99956	1.1052	0.14426	0.29374	0.56941	1.3056	0.38473
812.8295	0.5	domics Pos	0.96323	0.99987	1.003	0.004279	0.051405	0.99994	0.80635	-0.31052	0.48782	0.99996	0.92842	-0.10715	0.23673	0.69488	0.87942	-0.18538	0.017668	0.047376	0.76111	-0.39382	0.4872	0.70581	0.92375	-0.11443	0.59149	0.86213	0.9523	-0.07051	0.97864	0.9929	0.99983	-0.00025	0.17137	0.27913	1.1949	0.25688
1062.655	5.1	oolomics Pc	0.96448	0.99951	0.8694	-0.20191	0.75323	0.99998	0.73265	-0.44881	0.91076	0.99941	0.70759	-0.499	0.61698	0.99905	1.144	0.19409	0.20528	0.50362	1.8563	0.89243	0.065312	0.22265	2.32	1.2141	0.038197	0.38688	3.0305	1.5995	0.30212	0.99956	1.4403	0.5264	0.2184	0.48624	1.3798	0.46442
282.006	0.58	domics Pos	0.96521	0.99987	1.0209	0.029771	0.55326	0.99994	0.91592	-0.12671	0.81482	0.99996	0.99255	-0.01078	0.38032	0.82914	0.87376	-0.19469	0.015967	0.043862	0.52791	-0.92163	0.33891	0.60956	0.85085	-0.23301	0.85678	0.97142	0.99282	-0.0104	0.91314	0.98248	0.99152	-0.01228	0.015352	0.09885	1.4454	0.53145
1141.731	1.88	domics Pos	0.96523	0.99987	1.0826	0.11445	0.17167	0.99994	0.7819	-0.35495	0.73249	0.99996	0.95075	-0.07287	0.047585	0.37644	0.64223	-0.63883	0.17476	0.26644	0.73627	-0.4417	0.38169	0.63832	0.84254	-0.24718	0.69826	0.91211	1.1701	0.22666	0.60619	0.97294	1.114	0.1558	0.27029	0.37997	1.3233	0.40409
960.756	10.08	domics Pos	0.9653	0.99987	0.99925	-0.00108	0.046578	0.99994	0.81617	-0.29306	0.78642	0.99996	1.0233	0.033231	0.16675	0.60038	0.87945	-0.18533	0.002996	0.012257	0.741	-0.43246	0.18684	0.47264	0.82723	-0.27364	0.63594	0.88241	0.96737	-0.04787	0.71899	0.97988	0.97926	-0.03024	0.096412	0.19752	1.1044	0.14332

343.2869	4.66	domics Pos	0.96552	0.99987	1.0073	0.010556	0.61702	0.99994	0.92921	-0.10593	0.98053	0.99996	1.0222	0.031692	0.64563	0.95265	0.946	-0.08008	0.040539	0.088939	0.7673	-0.38214	0.88867	0.94151	0.99189	-0.01175	0.47624	0.80869	0.90244	-0.1481	0.080083	0.79727	0.75892	-0.39799	0.16429	0.27173	0.85551	-0.22515
543.3253	1.11	domics Pos	0.96574	0.99987	0.98258	-0.02535	0.89344	0.99994	1.0011	0.001564	0.87077	0.99996	1.0004	0.000619	0.77151	0.97526	1.032	0.045464	0.69219	0.73862	1.1014	0.13934	0.046916	0.20899	1.816	0.8608	0.021254	0.33809	2.126	1.0881	0.25637	0.94365	1.4244	0.51034	0.082074	0.18348	1.6951	0.76135
323.1981	1.84	domics Pos	0.96579	0.99987	1.0027	0.003919	0.2081	0.99994	0.72218	-0.46956	0.076502	0.99996	0.69012	-0.53508	0.020083	0.25894	0.51108	-0.96838	0.016106	0.044151	0.44457	-1.1695	0.13559	0.40234	0.59545	-0.74795	0.014186	0.33809	0.45133	-1.1478	0.027784	0.58208	0.43557	-1.199	0.072802	0.17448	0.55293	-0.85483
380.8158	0.42	olomics Ne	0.9659	0.99918	1.0138	0.019732	0.3088	0.73812	1.1207	0.16434	0.16781	0.99995	1.1837	0.24334	0.34545	0.99985	1.1135	0.15506	0.047745	0.99927	1.2927	0.37035	0.008579	0.36417	1.3414	0.42372	0.03086	0.34429	1.2174	0.28381	0.16947	0.99512	1.2018	0.26519	0.30142	0.99941	1.1215	0.16537
1171.831	4.91	domics Pos	0.96614	0.99987	0.99349	-0.00942	0.4778	0.99994	0.89813	-0.15501	0.33638	0.99996	0.86918	-0.20228	0.63039	0.94708	0.93812	-0.09215	0.023993	0.059755	0.70978	-0.49456	0.35473	0.61961	0.8401	-0.25137	0.23273	0.6761	0.86562	-0.2082	0.1647	0.91806	0.82893	-0.27068	0.10022	0.20219	1.2064	0.2707
367.2234	6.36	oolomics Pc	0.96638	0.99951	0.98433	-0.02279	0.064186	0.99998	0.74547	-0.42379	0.29088	0.99941	0.86828	-0.20378	0.080654	0.37362	0.77422	-0.36918	0.004736	0.028469	0.4693	-1.0914	0.02233	0.1103	0.63231	-0.6613	0.013889	0.28064	0.68868	-0.5381	0.26654	0.99956	0.87455	-0.19338	0.15167	0.4066	0.82126	-0.28409
948.5838	5.29	lomics Neg	0.96642	0.99977	1.0333	0.04732	0.73263	0.99904	0.97716	-0.03334	0.31786	0.99994	0.91284	-0.13156	0.013646	0.58182	1.2832	0.35978	0.77461	0.99989	1.1029	0.14127	0.99103	0.99653	1.0472	0.066555	0.049191	0.38213	0.77349	-0.37054	0.041611	0.46971	0.67365	-0.56993	0.9705	0.98379	0.99931	-0.001
145.9304	12.7	olomics Ne	0.9665	0.99918	1.0012	0.001692	0.034172	0.44974	0.91105	-0.1344	0.59208	0.99995	1.0202	0.028834	0.86714	0.99985	1.0086	0.012335	0.92459	0.99927	1.0063	0.009022	0.060228	0.50987	1.1097	0.15015	0.054945	0.39335	1.061	0.085481	0.40406	0.99512	1.0389	0.055028	0.79945	0.99941	0.99082	-0.0133
322.2159	2.84	domics Pos	0.96656	0.99987	0.98294	-0.02483	0.52308	0.99994	0.84232	-0.24756	0.2777	0.99996	0.76936	-0.37827	0.025739	0.28326	0.53762	-0.89534	0.018672	0.049443	0.4352	-1.2003	0.011792	0.092554	0.44213	-1.1775	0.044563	0.38909	0.52651	-0.92548	0.012	0.52955	0.48446	-1.0455	0.17427	0.28193	0.66908	-0.57975
327.344	4	domics Pos	0.96694	0.99987	0.97733	-0.03309	0.12836	0.99994	0.67167	-0.57417	0.56741	0.99996	0.90535	-0.14345	0.12157	0.54337	0.77453	-0.3686	0.058033	0.11628	0.72591	-0.46213	0.016766	0.10983	0.57116	-0.80803	0.29551	0.72001	0.80715	-0.30909	0.081138	0.8026	0.72752	-0.45894	0.8804	0.91319	0.97164	-0.04151
154.0604	0.61	lomics Neg	0.96733	0.99977	0.99153	-0.01227	0.55666	0.99904	0.92146	-0.118	0.70687	0.99994	1.0852	0.11799	0.017346	0.58182	1.609	0.68616	0.045821	0.84769	1.4508	0.53681	0.086472	0.99653	1.3593	0.44289	0.055777	0.40268	1.3365	0.41841	0.79749	0.89078	1.113	0.15443	0.58608	0.73202	0.92677	-0.10971
116.0714	0.59	domics Pos	0.96742	0.99987	0.99195	-0.01167	0.62334	0.99994	0.93018	-0.10442	0.329	0.99996	1.2921	0.36968	0.028289	0.29796	1.7724	0.8257	0.087175	0.15768	1.4985	0.58356	0.004046	0.069859	1.8596	0.895	0.000243	0.15466	2.5862	1.3708	0.04041	0.64308	1.8211	0.8648	0.004696	0.085788	1.7593	0.815
1051.771	0.51	domics Pos	0.96756	0.99987	0.99709	-0.0042	0.73063	0.99994	0.97314	-0.03928	0.63068	0.99996	0.95432	-0.06745	0.34007	0.79697	0.93398	-0.09854	0.024995	0.061727	0.83151	-0.2662	0.61058	0.78973	0.95294	-0.06955	0.70492	0.91485	1.0388	0.054967	0.77622	0.97988	0.97961	-0.02972	0.090243	0.19074	1.1728	0.22993
392.2033	1.83	domics Pos	0.96762	0.99987	1.0419	0.059267	0.43474	0.99994	0.85017	-0.23417	0.024471	0.99996	0.57716	-0.79297	0.016055	0.24	0.36927	-1.4373	0.000614	0.003839	0.29735	-1.7498	0.013463	0.097963	0.33436	-1.5805	0.004044	0.3088	0.32708	-1.6123	0.009153	0.52955	0.28455	-1.8132	0.023944	0.11067	0.38475	-1.378
898.6973	7.17	domics Pos	0.96784	0.99987	0.94293	-0.08478	0.83401	0.99994	0.88837	-0.17077	0.68406	0.99996	0.8334	-0.26291	0.26652	0.72795	0.64521	-0.63216	0.23429	0.32892	0.60961	-0.71403	0.71381	0.85171	0.84449	-0.24384	0.56756	0.85768	0.78241	-0.35401	0.048992	0.6775	0.47451	-1.0755	0.54481	0.64082	0.77572	-0.3664
590.3219	1.03	domics Pos	0.96785	0.99987	1.028	0.039844	0.74818	0.99994	1.0478	0.067426	0.50668	0.99996	1.1407	0.18996	0.77734	0.97526	1.0278	0.039512	0.56433	0.62862	0.86901	-0.20256	0.053925	0.22923	1.501	0.58589	0.021496	0.33809	1.8425	0.88165	0.40324	0.96776	1.1627	0.21748	0.080866	0.1825	1.4664	0.55224
1084.663	5.1	olomics Ne	0.968	0.99918	1.0844	0.1169	0.86278	0.98874	0.93403	-0.09847	0.65382	0.99995	1.1377	0.18617	0.41696	0.99985	1.3281	0.40934	0.28831	0.99927	1.8431	0.88215	0.027364	0.46144	3.4075	1.7687	0.009293	0.25531	3.643	1.8651	0.17886	0.99512	1.7647	0.81944	0.17998	0.99941	1.7242	0.78595
930.5549	5.71	domics Pos	0.96815	0.99987	1.001	0.001494	0.3303	0.99994	0.89767	-0.15575	0.21056	0.99996	0.9197	-0.12076	0.78626	0.97526	0.97913	-0.03043	0.049517	0.10363	0.77589	-0.36608	0.42168	0.65842	0.95431	-0.06747	0.38849	0.7665	1.0862	0.11924	0.79957	0.97988	1.0216	0.030812	0.005292	0.088007	1.35	0.43296
331.1926	3.17	olomics Ne	0.96823	0.99918	1.0143	0.020514	0.60574	0.90165	1.0905	0.12501	0.2532	0.99995	1.2177	0.28419	0.88368	0.99985	0.97765	-0.03261	0.021923	0.91567	0.72138	-0.47117	0.021779	0.43126	0.66123	-0.59678	0.002806	0.22316	0.45997	-1.1204	0.37906	0.99512	1.1139	0.15562	0.24211	0.99941	0.83587	-0.25865
787.9821	6.5	oolomics Pc	0.96859	0.99951	1.0358	0.050681	0.30845	0.99998	0.84849	-0.23703	0.76178	0.99941	1.0334	0.04743	0.007062	0.1948	0.6743	-0.56853	0.4173	0.81857	0.88332	-0.17898	0.093431	0.28463	0.80324	-0.3161	0.85132	0.9951	0.99873	-0.00184	0.82719	0.99956	1.0043	0.006136	0.7027	0.86484	1.0928	0.12804
461.2775	1.15	domics Pos	0.96896	0.99987	0.97854	-0.03129	0.92497	0.99994	1.0135	0.019356	0.76404	0.99996	1.0609	0.08529	0.66798	0.95717	1.1772	0.23531	0.17766	0.26956	1.5457	0.62824	0.011017	0.091128	2.4552	1.2958	0.003166	0.2624	2.9246	1.5482	0.26705	0.94365	1.5578	0.63947	0.075251	0.17722	1.9442	0.95914
349.2314	6.95	olomics Ne	0.96916	0.99918	1.0304	0.043224	0.94821	0.9934	1.062	0.086846	0.91158	0.99995	1.0251	0.035773	0.22048	0.99985	0.76438	-0.38763	0.087374	0.99927	0.66799	-0.5821	0.15669	0.62813	0.75986	-0.3962	0.018944	0.2915	0.61046	-0.71204	0.043727	0.99512	0.61099	-0.71079	0.041511	0.99941	0.61602	-0.69895
652.237	1.11	domics Pos	0.97001	0.99987	0.97468	-0.037	0.72626	0.99994	1.0609	0.085277	0.79091	0.99996	1.0857	0.11864	0.97163	0.99502	0.98832	-0.01694	0.37372	0.46614	0.82528	-0.27704	0.41233	0.65482	1.1728	0.22993	0.044368	0.38909	1.4653	0.55117	0.55341	0.96798	1.0689	0.096179	0.043139	0.13822	1.466	0.55193
724.5273	5.22	domics Pos	0.97024	0.99987	0.99723	-0.004	0.097517	0.99994	0.90185	-0.14904	0.1086	0.99996	0.90754	-0.13996	0.053435	0.39717	0.86776	-0.20464	0.002431	0.010449	0.77676	-0.36446	0.30739	0.58721	1.1385	0.18708	0.023627	0.33809	1.4059	0.4915	0.01499	0.52955	1.1533	0.20576	0.004895	0.087539	1.2722	0.34728
239.9668	10.2	domics Pos	0.9709	0.99987	1.0043	0.006228	0.18443	0.99994	0.86399	-0.21091	0.24897	0.99996	0.91231	-0.13241	0.064898	0.43615	0.85863	-0.21989	0.003009	0.012269	0.70871	-0.49674	0.53786	0.74246	0.94859	-0.07614	0.24339	0.67954	0.89651	-0.15761	0.367	0.96776	1.057	0.080042	0.22387	0.33143	1.0894	0.12356
247.243	6.96	oolomics Pc	0.97104	0.99951	1.0042	0.006071	0.47251	0.99998	0.88406	-0.17779	0.022526	0.99941	0.66084	-0.59762	0.014046	0.20085	0.47801	-1.0649	0.003074	0.020956	0.43135	-1.2131	0.003369	0.049867	0.33717	-1.5684	0.003807	0.20152	0.36925	-1.4373	0.020885	0.51556	0.42741	-1.2263	0.0129	0.2688	0.45586	-1.1333
219.2113	6.27	oolomics Pc	0.97126	0.99951	1.0094	0.013524	0.66635	0.99998	0.86571	-0.20805	0.098863	0.99941	0.53405	-0.90495	0.028172	0.24036	0.43346	-1.206	0.016282	0.070939	0.29016	-1.7851	0.007999	0.074341	0.20287	-2.3014	0.008499	0.23862	0.31742	-1.6555	0.022016	0.51556	0.30692	-1.704	0.10674	0.36162	0.53607	-0.89951
586.3133	4.83	olomics Ne	0.97156	0.99918	1.0404	0.057079	0.7161	0.94991	1.0479	0.067461	0.35344	0.99995	1.204	0.26779	0.14182	0.99985	1.3343	0.41605	0.29084	0.99927	1.2494	0.32124	0.011909	0.40245	1.8822	0.91242	0.01491	0.27907	1.8432	0.88225	0.55039	0.99512	1.0834	0.11553	0.82788	0.99941	0.99879	-0.00175
730.5741	6.02	domics Pos	0.97159	0.99987	0.99095	-0.01312	0.2999	0.99994	0.79093	-0.33838	0.50366	0.99996	0.8587	-0.21977	0.39052	0.83637	0.81871	-0.28857	0.28038	0.37512	0.78764	-0.34439	0.22353	0.5122	1.2168	0.2831	0.029977	0.35536	1.6246	0.70005	0.33074	0.95959	1.2415	0.31205	0.256	0.36505	1.3234	0.40426
588.3295	5.08	olomics Ne	0.97237	0.99918	1.0366	0.051923	0.89675	0.9934	1.0141	0.02022	0.41944	0.99995	1.1918	0.25316	0.44984	0.99985	1.1543	0.20702	0.47576	0.99927	1.1422	0.19181	0.04229	0.48405	1.4197	0.50557	0.060532	0.40009	1.4319	0.51794	0.72151	0.99512	1.0571	0.080123	0.54018	0.99941	1.0886	0.12242
772.5247	10.05	oolomics Pc	0.97275	0.99951	1.0111	0.015874	0.83664	0.99998	0.99175	-0.01195	0.4444	0.99941	1.1168	0.15941	0.60307	0.99905	1.1151	0.15722	0.71403	0.99688	0.96406	-0.0528	0.046009	0.17588	1.2251	0.29295	0.003009	0.20152	1.4111	0.49687	0.23388	0.99956	1.2319	0.30092	0.73595	0.88585	0.96726	-0.04802
501.2799	10.15	olomics Ne	0.97289	0.99918	0.99789	-0.00304	0.46632	0.82998	0.9356	-0.09603	0.49006	0.99995	0.93528	-0.09654	0.26516	0.99985	0.87968	-0.18496	0.74261	0.99927	1.0423	0.059774	0.029758	0.47167	1.2061	0.27034	0.031442	0.34429	1.3323	0.41396	0.099093	0.99512	1.1916	0.2529	0.43714	0.99941	1.0907	0.12519
838.528	5.02	domics Pos	0.97473	0.99987	1.0011	0.001603	0.56732	0.99994	0.93777	-0.0927	0.32403	0.99996	0.89054	-0.16725	0.23745	0.69591	0.90108	-0.15028	0.02613	0.06399	0.79544	-0.33017	0.14123	0.41327	0.79913	-0.32349	0.35475	0.75255	0.8873	-0.1725	0.86463	0.98023	0.98744	-0.01824	0.075977	0.17781	1.2943	0.37218
265.2531	2.84	domics Pos	0.97473	0.99987	0.99629	-0.00537	0.60372	0.99994	0.92468	-0.11297	0.045114	0.99996	0.69376	-0.52749	0.00766	0.19917	0.48761	-1.0362	0.000837	0.004875	0.30877	-1.6954	0.004184	0.071223	0.32097	-1.6395	0.003045	0.2624	0.34827	-1.5217	0.019501	0.52955	0.43071	-1.2152	0.06644	0.16612	0.45542	-1.1347
980.7533	0.54	domics Pos	0.97562	0.99987	1.0078	0.011185	0.72387	0.99994	1.0311	0.044191	0.012193	0.99996	0.88043	-0.18372	0.12488	0.54731	0.86793	-0.20435	0.001592	0.007646	0.74982	-0.41539	0.80072	0.89851	0.98682	-0.01915	0.6522	0.89057	0.97375	-0.03838	0.5868	0.97073	0.97163	-0.04152	0.33998	0.45011	1.0861	0.11921
1130.73	10.05	domics Pos	0.97573	0.99987	0.99433	-0.0082	0.2136	0.99994	0.8646	-0.20989	0.99473	0.99996	1.0095	0.013629	0.72881	0.96895	0.96474	-0.05179	0.008677	0.027456	0.71234	-0.48937	0.3604	0.6221	0.87986	-0.18465	0.95542	0.98985	1.0017	0.002503	0.69226	0.97988	0.9567	-0.06386	0.27338	0.3824	1.1308	0.17733
554.2831	1.29	domics Pos	0.97576	0.99987	1.0256	0.036463	0.57372	0.99994	0.92167	-0.11768	0.74479	0.99996	0.95128	-0.07205	0.24235	0.70272	0.84443	-0.24395	0.020349	0.052999	0.66448	-0.58969	0.71602	0.85262	0.94732	-0.07808	0.96787	0.99304	0.99973	-0.00039	0.58541	0.97073	0.92907	-0.10615	0.10572	0.20812	1.2085	0.27326
521.3437	1.12	domics Pos	0.97603	0.99987	0.99639	-0.00522	0.92049	0.99994	0.98852	-0.01666	0.79771	0.99996	1.0323	0.045902	0.65246	0.95465	1.1019	0.13997	0.62254	0.67917	1.14	0.18901	0.028314	0.14826	1.9947	0.99619	0.011433	0.3324	2.4363	1.2847	0.22723	0.94286	1.4278	0.51375	0.071718	0.17352	1.7884	0.83867
112.9855	0.28	olomics Ne	0.97616	0.99918	0.99949	-0.00074	0.006922	0.34605	0.85162	-0.23173	0.1577	0.99995	0.94356	-0.08382	0.51488	0.99985	1.0236	0.033691	0.65844	0.99927	0.97754	-0.03277	0.31064	0.72837	1.0901	0.12447	0.63041	0.87422	1.0373	0.052778	0.74954	0.99512	0.98504	-0.02175	0.70579	0.99941	1.0201	0.028752
292.9829	3.97	olomics Ne	0.97625	0.99918	0.99923	-0.00111	0.039067	0.45802	0.87659	-0.19002	0.53599	0.99995	0.96871	-0.04587	0.61214	0.99985	0.96893	-0.04554	0.45853	0.99927	0.96349	-0.05366	0.71091	0.94255	1.0235	0.033461	0.82987	0.95121	1.0106	0.015229	0.6011	0.99512	1.0247	0.035204	0.76331	0.99941	0.9847	-0.02225
387.1842	2.29	domics Pos	0.97631	0.99987	1.0143	0.020456	0.35472	0.99994	0.91879	-0.12219	0.006949	0.99996	0.7116	-0.49086	0.001137	0.17581	0.67708	-0.56259	0.000754	0.004496	0.51602	-0.95449	0.013782	0.099356	0.59291	-0.75411	0.00657	0.3088	0.66744	-0.58328	0.000194	0.37028	0.56113	-0.83358	0.12152	0.22569	0.77405	-0.3695
769.5586	5.47	domics Pos	0.97722	0.99987	0.99814	-0.00268	0.097856	0.99994	0.88615	-0.17438	0.093188	0.99996	0.88323	-0.17914	0.022304	0.26776	0.81283	-0.29897	0.000356	0.002521	0.63707	-0.65048	0.022351	0.13091	0.76796	-0.38089	0.34383	0.74613	0.92647	-0.11018	0.86749	0.98023	0.99543	-0.00661	0.94121	0.95677	1.0313	0.04446
331.9432	10.14	domics Pos	0.97754	0.99987	0.98685	-0.0191	0.77793	0.99994	0.96244	-0.05523	0.21116	0.99996	0.86353	-0.21168	0.4523	0.87408	0.91459	-0.12881	0.034679	0.07918	0.75077	-0.41356	0.42683	0.66239	0.89664	-0.15741	0.86921	0.97532	0.97401	-0.03799	0.90067	0.98023	1.0222	0.031742	0.17872	0.28597	1.1416	0.1911
717.5268	8.86	oolomics Pc	0.97766	0.99951	0.98074	-0.02806	0.71575	0.99998	0.88058	-0.18347	0.42805	0.99941	0.71999	-0.47396	0.66302	0.99905	0.85543	-0.22528	0.8906	0.99688	1.0708	0.098716	0.093217	0.28463	2.0029	1.0021	0.021393	0.31638	2.8862	1.5291	0.090034	0.80182	2.2945	1.1981	0.021301	0.2688	2.7117	1.4392
571.3587	5.22	oolomics Pc	0.97769	0.99951	1.0234	0.033411	0.94892	0.99998	1.0125	0.017911	0.53896	0.99941	1.0853	0.11814	0.5775	0.99905	1.1343	0.18182	0.19576	0.48823	1.3071	0.38632	0.027968	0.12667	1.772	0.8254	0.02544	0.33018	1.8602	0.89546	0.47702	0.99956	1.129	0.1751	0.43999	0.70925	1.1172	0.1599
304.218	1.79	domics Pos	0.97774	0.99987	1.0294	0.041767	0.69504	0.99994	0.93273	-0.10046	0.40796	0.99996	0.85204	-0.231	0.089122	0.49827	0.70902	-0.49611	0.002227	0.009806	0.43541	-1.1996	0.058899	0.24091	0.68635	-0.54299	0.41698	0.78082	0.86106	-0.21582	0.197	0.92582	0.76725	-0.38223	0.65584	0.74205	0.92133	-0.11822
718.5724	5.45	domics Pos	0.97799	0.99987	0.99588	-0.00596	0.49701	0.99994	0.91879	-0.1222	0.66651	0.99996	0.95182	-0.07124	0.5064	0.89133	0.92902	-0.10621	0.044703	0.095814	0.76912	-0.37872	0.17695	0.46371	0.82545	-0.27674	0.78458	0.94942	0.96941	-0.04482	0.14393	0.90387	0.86813	-0.20401	0.72789	0.79778	1.0445	0.062868
351.97	0.57	domics Pos	0.97818	0.99987	1.0216	0.030836	0.17	0.99994	0.88819	-0.17105	0.90914	0.99996	1.0135	0.019362	0.18083	0.6196	1.1952	0.25729	0.25426	0.34899	0.89053	-0.16726	0.40837	0.65315	1.0707	0.098495	0.000111	0.15466	1.4869	0.57235	0.9541	0.98838	1.009	0.012877	0.10728	0.20999	1.175	0.23264
571.3581	1.13	domics Pos	0.97844	0.99987	1.0284	0.040357	0.88476	0.99994	0.98822	-0.0171	0.43435	0.99996	1.1656	0.22112	0.54564	0.90473	1.0966	0.13306	0.70144	0.74584	0.87263	-0.19656	0.094684	0.32467	1.5876	0.66682	0.035005	0.36369	1.9934	0.9952	0.45381	0.96776	1.1785	0.23698	0.060318	0.15775	1.5097	0.59423
521.512	8.34	oolomics Pc	0.97866	0.99951	1.0005	0.000672	0.63354	0.99998	0.95758	-0.06254	0.13725	0.99941	0.86043	-0.21687	0.046274	0.28782	0.75354	-0.40824	0.37003	0.76655	0.85123	-0.23238	0.23619	0.5258	0.83576	-0.25885	0.27797	0.89698	0.87415	-0.19404	0.088641	0.80182	0.78222	-0.35435	0.090083	0.34256	0.70751	-0.49917
546.3557	1.22	domics Pos	0.97917	0.99987	1.067	0.093515	0.80525	0.99994	1.067	0.0936	0.66187	0.99996	1.1253	0.17029	0.73635	0.97097	1.0639	0.089375	0.83484	0.86057	0.95109	-0.07234	0.071425	0.27446	1.5004	0.58537	0.023087	0.33809	1.8058	0.85261	0.63849	0.97988	1.0734	0.10221	0.14299	0.24751	1.5231	0.60702
287.6324	5.11	oolomics Pc	0.9792	0.99951	1.0295	0.041993	0.73209	0.99998	0.90221	-0.14846	0.84378	0.99941	1.0246	0.035127	0.433	0.91715	1.2841	0.36072	0.15889	0.41882	1.4744	0.5601	0.05222	0.19329	1.67	0.73981	0.026203	0.3338	1.8798	0.91055	0.26527	0.99956	1.3894	0.47449	0.098495	0.35294	1.5333	0.61667
825.5465	4.53	lomics Neg	0.97962	0.99977	1.1936	0.25528	0.90742	0.99904	1.1082	0.14826	0.31983	0.99994	0.87337	-0.19533	0.30454	0.58182	1.3669	0.45092	0.78126	0.99989	1.1341	0.18154	0.90229	0.99653	1.2028	0.26638	0.089011	0.41257	0.65042	-0.62056	0.018755	0.46971	0.40946	-1.2882	0.18614	0.42711	0.80104	-0.32005
216.0345	0.59	lomics Neg	0.97981	0.99977	0.96423	-0.05255	0.60383	0.99904	0.88012	-0.18422	0.64589	0.99994	1.0495	0.06964	0.20201	0.58182	1.3492	0.43214	0.11825	0.99989	1.4816	0.5672	0.073768	0.94937	1.6532	0.72528	0.038725	0.37635	1.6561	0.72776	0.73419	0.84233	1.1501	0.20172	0.52767	0.6911	0.86093	-0.21604
504.3085	5.09	olomics Ne	0.97999	0.99918	1.0169	0.024231	0.98432	0.99664	0.97747	-0.03288	0.6236	0.99995	1.1019	0.13993	0.42461	0.99985	1.2283	0.29671	0.21445	0.99927	1.5293	0.6129	0.024809	0.4426	2.1589	1.1103	0.011916	0.2716	2.3163	1.2118	0.18835	0.99512	1.497	0.58207	0.17068	0.99941	1.4333	0.5193
942.5323	5.11	domics Pos	0.98102	0.99987	1.0059	0.008457	0.5493	0.99994	0.92338	-0.115	0.72106	0.99996	0.95348	-0.06873	0.21941	0.67258	0.84334	-0.24582	0.035275	0.080157	0.74378	-0.42705	0.16097	0.44349	0.78775	-0.34419	0.63294	0.8822	0.93864	-0.09135	0.91387	0.98248	0.98482	-0.02207	0.15395	0.25963	1.2569	0.32988
893.8181	10.02	domics Pos	0.98132	0.99987	0.98941	-0.01537	0.1025	0.99994	0.79278	-0.335	0.49688	0.99996	0.91633	-0.12606	0.97376	0.99515	0.98444	-0.02262	0.040058	0.088289	0.71673	-0.4805	0.96536	0.98263	1.0046	0.00664	0.78906	0.95091	1.0259	0.036823	0.5762	0.9703	0.92499	-0.11248	0.29952	0.4097	1.176	0.23391
938.7769	10.05	domics Pos	0.98212	0.99987	1.0002	0.000273	0.57407	0.99994	0.96492	-0.05151	0.060128	0.99996	0.84819	-0.23753	0.062499	0.43187	0.87548	-0.19186	0.006528	0.022283	0.75329	-0.40873	0.29104	0.57461	0.88766	-0.17191	0.7617	0.94108	1.0397	0.056184	0.27745	0.94365	1.0643	0.089848	0.001461	0.07528	1.2436	0.31448
795.5723	8.6	oolomics Pc	0.98219	0.99951	0.99751	-0.0036	0.95966	0.99998	1.0079	0.011358	0.81338	0.99941	0.97278	-0.03981	0.69536	0.99905	0.95279	-0.06976	0.56391	0.94484	0.92602	-0.11088	0.24275	0.53327	0.84675	-0.24	0.24829	0.85666	0.8457	-0.24178	0.048376	0.67731	0.75187	-0.41144	0.017156	0.2688	0.72829	-0.45742
222.8868	0.31	oolomics Pc	0.98222	0.99951	1.0138	0.019715	0.94612	0.99998	1.0053	0.007678	0.18227	0.99941	1.2719	0.34699	0.93913	0.99905	1.0108	0.01551	0.030721	0.11716	1.3531	0.43623	0.63967	0.88609	1.2103	0.27534	0.023883	0.32288	1.5337	0.61703	0.023042	0.51791	1.3578	0.44128	0.58699	0.80358	1.0741	0.10318
1032.771	10.03	domics Pos	0.98245	0.99987	0.99788	-0.00307	0.37119	0.99994	0.93225	-0.10121	0.27604	0.99996	0.93297	-0.10009	0.36854	0.82243	0.93639	-0.09482	0.001682	0.007928	0.73564	-0.44294	0.3411	0.61059	0.89754	-0.15595	0.52216	0.83957	0.95383	-0.0682	0.81297	0.97988	0.9824	-0.02562	0.076488	0.17807	1.1624	0.21714
1002.689	0.56	domics Pos	0.98247	0.99987	0.99629	-0.00536	0.55073	0.99994	0.91133	-0.13395	0.499	0.99996	0.90756	-0.13993	0.12788	0.55024	0.82542	-0.2768	0.015126	0.042007	0.67774	-0.56119	0.13521	0.40206	0.81673	-0.29207	0.042204	0.38314	0.76541	-0.38569	0.71006	0.97988	0.95307	-0.06935	0.55232	0.64781	1.0958	0.13204
852.5774	9.9	oolomics Pc	0.98249	0.99951	0.99674	-0.00471	0.4584	0.99998	0.90075	-0.1508	0.03385	0.99941	0.76988	-0.37729	0.10581	0.43684	0.85627	-0.22385	0.098064	0.28185	0.87059	-0.19993	0.3126	0.61543	0.90451	-0.14479	0.072604	0.54945	0.81316	-0.29839	0.1963	0.99341	0.82336	-0.2804	0.30381	0.58116	0.87726	-0.18892
520.3403	1.11	domics Pos	0.98257	0.99987	0.99339	-0.00956	0.94212	0.99994	0.98073	-0.02808	0.81404	0.99996	1.0255	0.036355	0.67678	0.95843	1.098	0.13489	0.62655	0.68189	1.144	0.19405	0.028155	0.14808	2.0014	1.001	0.010868	0.3324	2.47	1.3045	0.23273	0.94365	1.4246	0.51059	0.070036	0.17098	1.7905	0.84038
199.805	0.39	olomics Ne	0.98291	0.99918	1.0057	0.008162	0.037842	0.45581	0.89954	-0.15273	0.87626	0.99995	1.0109	0.015698	0.70957	0.99985	1.0158	0.022671	0.34618	0.99927	1.0696	0.097102	0.019164	0.42126	1.1216	0.16558	0.034522	0.3559	1.0908	0.1254	0.99812	0.99943	1.0023	0.00328	0.40101	0.99941	0.96893	-0.04554
103.9206	12.72	olomics Ne	0.98321	0.99918	0.99569	-0.00624	0.039043	0.45802	0.85709	-0.22249	0.68597	0.99995	1.0295	0.042005	0.30654	0.99985	1.0797	0.11057	0.78132	0.99927	0.97763	-0.03263	0.03043	0.47167	1.1927	0.25424	0.19078	0.56864	1.0975	0.13427	0.43113	0.99512	1.0631	0.088271	0.5287	0.99941	0.95843	-0.06126
565.5662	5.93	domics Pos	0.98327	0.99987	0.99646	-0.00512	0.66387	0.99994	1.0453	0.063898	0.99069	0.99996	1.0016	0.002343	0.32955	0.78974	0.88811	-0.17118	0.005927	0.020715	0.696	-0.52283	0.26073	0.54699	0.84765	-0.23846	0.54464	0.84937	0.93655	-0.09457	0.85271	0.97988	1.0178	0.02551	0.46069	0.56482	1.0982	0.13513
750.7941	10.08	domics Pos	0.98352	0.99987	0.99934	-0.00096	0.50216	0.99994	0.95609	-0.06478	0.60008	0.99996	0.96262	-0.05497	0.96752	0.99502	1.0069	0.009962	0.010642	0.032204	0.80073	-0.32061	0.28594	0.57234	0.89626	-0.158	0.77737	0.94624	0.97798	-0.03213	0.82023	0.97988	1.0216	0.030866	0.01389	0.098791	1.2376	0.30751
331.2637	2.28	domics Pos	0.98359	0.99987	1.0307	0.04364	0.25953	0.99994	0.81255	-0.29948	0.021542	0.99996	0.64269	-0.6378	0.000246	0.164	0.40371	-1.3086	0.000163	0.001447	0.25617	-1.9648	0.000611	0.03855	0.20689	-2.2731	0.011026	0.3324	0.33189	-1.5912	0.017765	0.52955	0.36542	-1.4524	0.013836	0.098791	0.42432	-1.2368
408.3306	3.52	domics Pos	0.98392	0.99987	0.99878	-0.00176	0.49968	0.99994	0.86365	-0.21149	0.18614	0.99996	0.75563	-0.40425	0.009278	0.201	0.53942	-0.89052	0.000956	0.005398	0.38379	-1.3816	0.008359	0.084185	0.40815	-1.2928	0.005283	0.3088	0.48252	-1.0513	0.010519	0.52955	0.47284	-1.0806	0.080914	0.1825	0.65175	-0.61762
337.1711	6.48	oolomics Pc	0.98402	0.99951	1.0161	0.022979	0.6868	0.99998	0.96084	-0.05763	0.062626	0.99941	0.76337	-0.38954	0.082395	0.37945	0.68332	-0.54936	0.000921	0.008819	0.58192	-0.78111	0.006207	0.065804	0.52569	-0.92772	0.019861	0.30146	0.54139	-0.88525	0.009278	0.47452	0.50542	-0.98445	0.032132	0.2688	0.52935	-0.91771
370.4065	6.29	domics Pos	0.98412	0.99987	0.97463	-0.03707	0.96925	0.99994	0.99245	-0.01093	0.92978	0.99996	1.0311	0.044216	0.71547	0.96431	0.93309	-0.09991	0.05733	0.11517	0.51288	-0.96332	0.038593	0.18011	0.57372	-0.80159	0.71103	0.91686	0.93754	-0.09305	0.65261	0.97988	0.90338	-0.14659	0.64954	0.73733	0.87737	-0.18873
692.1979	1.26	domics Pos	0.9842	0.99987	0.99405	-0.00861	0.85674	0.99994	0.97286	-0.0397	0.44842	0.99996	0.90333	-0.14668	0.34537	0.79994	0.89002	-0.16808	0.045767	0.097436	0.72607	-0.46182	0.35113	0.61843	0.85677	-0.22302	0.32811	0.7368	0.86669	-0.20642	0.78014	0.97988	1.0233	0.033181	0.79305	0.84679	1.0426	0.060234
1182.822	8.48	oolomics Pc	0.98421	0.99951	1.0433	0.061139	0.83819	0.99998	1.0769	0.10692	0.98436	0.99941	1.0406	0.057456	0.72502	0.99905	1.1304	0.17683	0.55518	0.94111	1.1482	0.19943	0.33136	0.64194	1.2939	0.37169	0.044459	0.4231	1.5565	0.63831	0.043648	0.65472	1.5386	0.62157	0.016894	0.2688	1.7268	0.78809
256.2646	8.37	oolomics Pc	0.98421	0.99951	1.0018	0.002525	0.63254	0.99998	0.96843	-0.04628	0.41664	0.99941	0.94464	-0.08216	0.47454	0.95149	0.96098	-0.05742	0.62327	0.97726	1.0289	0.041164	0.86106	0.95795	0.98996	-0.01456	0.9972	0.99906	1.0041	0.005835	0.016176	0.49126	0.8654	-0.20856	0.032854	0.2688	0.83438	-0.26122
707.1695	3.44	domics Pos	0.9844	0.99987	1.0959	0.13213	0.86585	0.99994	1.0396	0.056012	0.63839	0.99996	0.95429	-0.0675	0.094963	0.51434	0.65449	-0.61156	0.009753	0.030216	0.58494	-0.77365	0.6735	0.83076	2.3048	1.2046	0.72693	0.92597	1.4021	0.48762	0.89206	0.98023	0.99809	-0.00275	0.10781	0.21059	1.5572	0.63898
599.5035	5	domics Pos	0.98446	0.99987	0.98526	-0.02142	0.35642	0.99994	0.83282	-0.26393	0.3887	0.99996	0.85316	-0.22912	0.27963	0.7451	0.80932	-0.30522	0.045087	0.096365	0.64594	-0.63052	0.18127	0.46866	0.73805	-0.43822	0.71049	0.91681	1.0673	0.09398	0.08877	0.82455	1.322	0.40275	0.00823	0.092094	1.7102	0.77416
1004.7	0.54	domics Pos	0.98454	0.99987	1.0007	0.000994	0.50056	0.99994	1.0233	0.033205	0.01134	0.99996	0.84746	-0.23878	0.013569	0.22898	0.81543	-0.29437	0.023531	0.058952	0.76723	-0.38228	0.001635	0.052793	0.73889	-0.43657	0.036811	0.36759	0.77636	-0.36521	0.10734	0.86469	0.86474	-0.20965	0.22364	0.33143	1.1511	0.20307
603.2819	1.11	domics Pos	0.98482	0.99987	1.0434	0.061294	0.64387	0.99994	1.0909	0.1255	0.83907	0.99996	1.0279	0.039727	0.58949	0.92652	1.0676	0.09439	0.5339	0.60265	1.1306	0.1771	0.03351	0.16483	1.5196	0.60373	0.0205	0.33809	1.6264	0.70171	0.1331	0.88792	1.4571	0.54308	0.019968	0.10636	1.6653	0.73574
996.7702	10.05	domics Pos	0.98484	0.99987	1.0063	0.009052	0.40877	0.99994	0.96082	-0.05766	0.11647	0.99996	0.89704	-0.15676	0.08514	0.4892	1.0812	0.11269	0.002108	0.009424	0.72307	-0.46779	0.41524	0.65507	0.92643	-0.11025	0.50642	0.8319	1.0689	0.096157	0.21867	0.94047	1.1001	0.13763	0.084067	0.18577	1.2362	0.30588
440.8711	10.17	domics Pos	0.98487	0.99987	1.0116	0.016639	0.89195	0.99994	0.99025	-0.01414	0.19252	0.99996	0.91979	-0.12063	0.14503	0.57187	0.86709	-0.20575	0.01914	0.050401	0.71546	-0.48306	0.29254	0.57504	0.87365	-0.19487	0.6454	0.8881	0.97084	-0.0427	0.58191	0.97073	0.95459	-0.06705	0.02177	0.10837	1.3093	0.38883
154.0356	0.34	olomics Ne	0.98532	0.99918	1.0022	0.003174	0.46151	0.82998	0.93561	-0.09602	0.083045	0.99995	1.2932	0.3709	0.011134	0.99985	1.4288	0.5148	0.053422	0.99927	1.5002	0.58512	0.008143	0.36417	1.5547	0.63661	0.001735	0.22316	1.6652	0.73566	0.2068	0.99512	1.2463	0.31762	0.28833	0.99941	1.1254	0.17039
116.0719	0.33	oolomics Pc	0.98537	0.99951	1.0062	0.008943	0.996	0.99998	0.99635	-0.00527	0.29861	0.99941	1.3674	0.45146	0.028831	0.24036	2.0021	1.0015	0.011173	0.053119	1.9586	0.96985	0.010242	0.080256	2.1949	1.1341	0.001293	0.16989	2.6125	1.3854	0.049234	0.67731	1.9371	0.95391	0.032835	0.2688	1.5231	0.60703
339.1892	6.96	oolomics Pc	0.9855	0.99951	1.0151	0.02166	0.78757	0.99998	0.97559	-0.03566	0.007888	0.99941	0.71431	-0.48538	0.050251	0.29486	0.59576	-0.74721	0.001615	0.013281	0.53482	-0.90288	0.001844	0.045425	0.46425	-1.107	0.001196	0.16989	0.47845	-1.0636	0.012337	0.47452	0.46473	-1.1055	0.024967	0.2688	0.56369	-0.82703
416.316	4.08	oolomics Pc	0.9857	0.99951	1.0864	0.11959	0.69928	0.99998	1.4032	0.48871	0.3607	0.99941	1.7856	0.83641	0.10578	0.43684	2.8001	1.4855	0.006246	0.034132	4.2752	2.096	0.016687	0.095743	2.7792	1.4747	0.004477	0.20152	2.9137	1.5428	0.36283	0.99956	1.2615	0.33509	0.14906	0.40234	1.5335	0.61686
323.2257	3.52	domics Pos	0.98597	0.99987	0.99203	-0.01154	0.15184	0.99994	0.78744	-0.34475	0.022474	0.99996	0.70308	-0.50824	0.007349	0.19896	0.61705	-0.69653	0.000494	0.003251	0.45256	-1.1438	0.008847	0.08583	0.51718	-0.95126	0.004145	0.3088	0.57057	-0.80954	0.020968	0.52955	0.57296	-0.80349	0.13008	0.23517	0.75738	-0.40091
218.9863	2.94	olomics Ne	0.986	0.99918	0.99991	-0.00012	0.10938	0.52291	0.90103	-0.15035	0.75051	0.99995	1.0193	0.027545	0.38576	0.99985	0.95082	-0.07276	0.69448	0.99927	0.97276	-0.03985	0.030368	0.47167	1.1619	0.21654	0.7709	0.93495	1.0161	0.023032	0.63234	0.99512	1.0285	0.04048	0.74953	0.99941	0.98278	-0.02506
579.288	5.48	oolomics Pc	0.98605	0.99951	1.0197	0.02821	0.61471	0.99998	0.90852	-0.13841	0.76175	0.99941	1.0278	0.039498	0.2931	0.77976	1.2409	0.31138	0.035863	0.13192	1.6934	0.75994	0.006377	0.066819	2.6165	1.3876	0.009677	0.24769	1.9087	0.93262	0.14725	0.93461	1.399	0.48439	0.041957	0.28426	1.5305	0.614
219.992	7.64	oolomics Pc	0.98647	0.99951	1.0178	0.02539	0.86505	0.99998	0.98082	-0.02794	0.19725	0.99941	0.88415	-0.17764	0.42344	0.9108	0.90025	-0.15161	0.64856	0.98974	0.94767	-0.07755	0.58388	0.85985	1.0724	0.10082	0.43517	0.99391	0.90604	-0.14235	0.049454	0.67731	0.78475	-0.3497	0.83122	0.93201	1.0442	0.062371
926.4621	8.22	oolomics Pc	0.98662	0.99951	1.0121	0.017419	0.69894	0.99998	0.96189	-0.05606	0.67037	0.99941	0.95324	-0.06909	0.4463	0.92368	0.88429	-0.17741	0.99652	0.9989	1.0279	0.039646	0.56894	0.84837	0.93802	-0.09232	0.032201	0.35567	0.68183	-0.55252	0.14022	0.92991	0.82444	-0.27852	0.94581	0.97874	1.0303	0.043091
243.2114	4.73	domics Pos	0.98685	0.99987	1.003	0.00428	0.015079	0.99994	0.8549	-0.22617	0.83369	0.99996	1.0142	0.020313	0.21179	0.65978	0.91842	-0.12277	0.008908	0.028095	0.72605	-0.46186	0.68169	0.83502	0.97263	-0.04004	0.51064	0.83279	0.95527	-0.06602	0.78742	0.97988	0.98484	-0.02204	0.29956	0.4097	1.1829	0.24235
740.5224	5	domics Pos	0.98708	0.99987	0.99277	-0.01047	0.2782	0.99994	0.85389	-0.22789	0.19303	0.99996	0.82586	-0.27604	0.1085	0.53208	0.77616	-0.36558	0.028107	0.067616	0.64846	-0.62491	0.20819	0.50083	0.81272	-0.29917	0.27332	0.70555	1.1215	0.16538	0.063644	0.73915	1.3574	0.4409	0.008288	0.092405	1.6862	0.75379
854.0189	6.48	oolomics Pc	0.98726	0.99951	1.0292	0.041593	0.28199	0.99998	0.8357	-0.25894	0.77453	0.99941	0.96437	-0.05234	0.010575	0.19754	0.68025	-0.55586	0.20419	0.50211	0.78602	-0.34737	0.040131	0.16042	0.72221	-0.46951	0.39796	0.97985	0.87489	-0.19283	0.44516	0.99956	0.88361	-0.17852	0.91676	0.96508	1.005	0.007184
692.8354	0.53	domics Pos	0.98733	0.99987	0.99713	-0.00414	0.24512	0.99994	0.89985	-0.15224	0.31477	0.99996	0.91568	-0.12708	0.087411	0.49304	0.86393	-0.21101	0.004624	0.017185	0.7091	-0.49595	0.33386	0.60735	0.86443	-0.21018	0.41694	0.78082	0.91163	-0.13348	0.44466	0.96776	0.9325	-0.10083	0.10934	0.21282	1.2213	0.28845
476.2744	5.28	oolomics Pc	0.98768	0.99951	0.95521	-0.0661	0.80107	0.99998	0.93135	-0.1026	0.92414	0.99941	0.97275	-0.03986	0.66811	0.99905	1.0988	0.13597	0.68449	0.99688	1.0444	0.062622	0.029535	0.1314	1.7968	0.84545	0.012696	0.27021	2.0812	1.0574	0.082716	0.78717	1.6381	0.71203	0.20087	0.46635	1.4036	0.48916
143.1082	6.96	oolomics Pc	0.9877	0.99951	1.0236	0.033699	0.44637	0.99998	0.85734	-0.22207	0.022529	0.99941	0.62068	-0.68809	0.02066	0.22211	0.47601	-1.0709	0.005172	0.030068	0.42605	-1.2309	0.002036	0.045856	0.34081	-1.553	0.00125	0.16989	0.3048	-1.7141	0.014126	0.47452	0.38759	-1.3674	0.01623	0.2688	0.43371	-1.2052
300.1438	1.91	domics Pos	0.98773	0.99987	1.0176	0.025204	0.80252	0.99994	1.026	0.037002	0.34626	0.99996	1.0728	0.10142	0.75569	0.97526	1.0439	0.061987	0.005903	0.020669	0.7483	-0.4183	0.26673	0.55455	0.87836	-0.18712	0.15244	0.62232	0.87019	-0.2006	0.41016	0.96776	0.90206	-0.14871	0.18546	0.29295	1.1317	0.17855
1025.709	9.07	oolomics Pc	0.98819	0.99951	1.0366	0.051854	0.052374	0.99998	0.80276	-0.31697	0.87828	0.99941	0.98605	-0.02027	0.047925	0.2892	0.67822	-0.56017	0.009734	0.047435	0.58494	-0.77364	0.007562	0.071961	0.63748	-0.64954	0.85557	0.9951	0.99694	-0.00443	0.67808	0.99956	0.96212	-0.05572	0.67878	0.85424	1.1768	0.23491
624.848	10.05	domics Pos	0.98861	0.99987	0.99874	-0.00182	0.18356	0.99994	0.92524	-0.1121	0.57361	0.99996	0.97064	-0.04299	0.34444	0.79913	0.9339	-0.09865	0.00078	0.004609	0.77171	-0.37387	0.5749	0.76798	0.95353	-0.06864	0.86971	0.97532	0.99061	-0.01362	0.76823	0.97988	1.0177	0.02531	0.018191	0.1032	1.1938	0.2556
500.2754	1.16	domics Pos	0.98881	0.99987	0.97634	-0.03454	0.98824	0.99994	0.98045	-0.02849	0.82349	0.99996	1.0842	0.11665	0.4976	0.89009	1.3581	0.44162	0.097768	0.17149	1.9121	0.93516	0.00525	0.074135	3.0255	1.5972	0.002287	0.26175	3.5258	1.8179	0.14064	0.89982	1.9467	0.96106	0.038173	0.13148	2.3265	1.2182
375.2097	2.28	domics Pos	0.98915	0.99987	1.0098	0.014079	0.53419	0.99994	0.8712	-0.19893	0.096295	0.99996	0.66123	-0.59678	0.011456	0.21625	0.50933	-0.97334	0.000871	0.005015	0.37279	-1.4236	0.003671	0.06753	0.38193	-1.3886	0.012005	0.3324	0.43128	-1.2133	0.020102	0.52955	0.46381	-1.1084	0.032786	0.12371	0.52981	-0.91644
488.8732	10.06	domics Pos	0.98925	0.99987	1.0021	0.003039	0.088138	0.99994	0.91629	-0.12612	0.047631	0.99996	0.95901	-0.06038	0.17224	0.60551	0.93357	-0.09917	2.32E-07	0.000144	0.74129	-0.43189	0.066193	0.26074	0.87813	-0.1875	0.82159	0.96036	0.99093	-0.01315	0.22504	0.94286	0.96683	-0.04866	0.006301	0.090084	1.1588	0.21257

252.9024	10.15	lomics Pos	0.9899	0.99987	0.99616	-0.00555	0.09829	0.99994	0.85695	-0.22271	0.16494	0.99996	0.90117	-0.15012	0.2873	0.75229	0.91291	-0.13146	0.011019	0.033083	0.74929	-0.41641	0.44028	0.67288	0.91724	-0.12462	0.99459	0.99769	1.003	0.00435	0.89723	0.98023	1.008	0.011507	0.020384	0.10677	1.2525	0.32485
853.517	6.48	olomics Pc	0.9899	0.99951	1.0253	0.036039	0.43787	0.99998	0.87287	-0.19616	0.76363	0.99941	0.95208	-0.07084	0.021967	0.2227	0.70696	-0.5003	0.37843	0.77508	0.84625	-0.24085	0.020751	0.10769	0.69229	-0.53056	0.75025	0.99391	0.95652	-0.06414	0.66142	0.99956	0.95342	-0.06882	0.98103	0.98952	1.0172	0.024615
926.7278	7.43	lomics Pos	0.99012	0.99987	0.96713	-0.04822	0.88455	0.99994	1.0225	0.03204	0.87704	0.99996	0.94286	-0.08489	0.52504	0.89938	0.82017	-0.28601	0.16124	0.24991	0.62196	-0.6851	0.17465	0.46149	0.57156	-0.80702	0.21302	0.65483	0.60622	-0.72208	0.037329	0.64014	0.36132	-1.4687	0.090757	0.19109	0.46348	-1.1094
361.2739	5.01	olomics Pc	0.99027	0.99951	1.0001	0.000144	0.64032	0.99998	0.95543	-0.06578	0.50877	0.99941	0.9438	-0.08345	0.96618	0.99905	1.0053	0.00761	0.10598	0.30021	1.1879	0.2484	0.10345	0.30741	1.1877	0.24822	0.024465	0.32533	1.3936	0.47878	0.072666	0.76911	1.2708	0.34579	0.16054	0.41829	1.2587	0.33196
937.1103	6.46	olomics Pc	0.99042	0.99951	1.0737	0.10252	0.55423	0.99998	0.89796	-0.15528	0.75814	0.99941	1.0395	0.055945	0.013586	0.20085	0.59387	-0.75177	0.073506	0.22458	0.67644	-0.56396	0.11909	0.33524	0.76852	-0.37985	0.72098	0.99391	0.94328	-0.08424	0.88755	0.99956	1.1612	0.21563	0.98276	0.99062	1.0194	0.027651
376.1852	2.28	lomics Pos	0.99048	0.99987	1.0224	0.032004	0.45283	0.99994	0.90766	-0.13977	0.043347	0.99996	0.75214	-0.41093	0.015952	0.24	0.63042	-0.66561	7.32E-05	0.000845	0.5081	-0.97681	0.018544	0.11661	0.52742	-0.92296	0.038845	0.37121	0.55842	-0.84057	0.033963	0.6327	0.54658	-0.87149	0.16371	0.27152	0.76078	-0.39446
398.219	6.83	olomics Pc	0.99083	0.99951	1.0056	0.008042	0.61781	0.99998	0.94007	-0.08916	0.049187	0.99941	0.76218	-0.3918	0.031988	0.24911	0.59589	-0.74689	0.000283	0.003688	0.53576	-0.90034	0.008874	0.075866	0.44225	-1.1771	0.002991	0.20152	0.5133	-0.96213	0.001833	0.47452	0.45676	-1.1305	0.008324	0.2688	0.52191	-0.93814
724.7064	0.56	lomics Pos	0.99147	0.99987	1.0258	0.036783	0.96838	0.99994	1.0147	0.021077	0.33579	0.99996	1.1001	0.13766	0.33447	0.79326	0.89269	-0.16378	0.00557	0.019831	0.7006	-0.51334	0.042066	0.19256	0.82707	-0.27392	0.32487	0.73428	0.85715	-0.22238	0.90433	0.98183	1.0087	0.01252	0.33885	0.44909	1.1953	0.2574
564.3298	5.09	olomics Ne	0.99152	0.99918	0.99873	-0.00183	0.94657	0.9934	0.97149	-0.04173	0.61761	0.99995	1.103	0.14137	0.49388	0.99985	1.1871	0.2475	0.25219	0.99927	1.4675	0.55331	0.021242	0.43126	2.1248	1.0874	0.011292	0.2716	2.2192	1.1501	0.18006	0.99512	1.4507	0.53672	0.15685	0.99941	1.4159	0.50175
375.1988	6.49	olomics Pc	0.99172	0.99951	0.99294	-0.01022	0.7491	0.99998	0.9451	-0.08145	0.17819	0.99941	0.75485	-0.40574	0.14603	0.53989	0.72319	-0.46755	0.10786	0.30498	0.72977	-0.45449	0.041605	0.16485	0.61283	-0.70644	0.093639	0.60726	0.66033	-0.59874	0.021238	0.51556	0.52679	-0.92469	0.10094	0.35349	0.63778	-0.64886
542.3222	1.11	lomics Pos	0.99174	0.99987	0.99946	-0.00078	0.85893	0.99994	1.0151	0.021635	0.80597	0.99996	1.0278	0.039582	0.72674	0.96881	1.0519	0.072979	0.64452	0.69829	1.1244	0.16912	0.041993	0.19245	1.8179	0.8623	0.018318	0.33809	2.1414	1.0986	0.23579	0.94365	1.4283	0.51434	0.068806	0.17047	1.7394	0.79858
171.14	6.49	olomics Pc	0.99181	0.99951	0.99456	-0.00787	0.79021	0.99998	0.97589	-0.0352	0.26142	0.99941	0.88412	-0.17769	0.08023	0.3722	0.7824	-0.35402	0.024606	0.098865	0.77263	-0.37216	0.036039	0.14957	0.74832	-0.41827	0.051329	0.45418	0.7863	-0.34685	0.037535	0.61903	0.7431	-0.42838	0.070965	0.31396	0.75548	-0.40453
1085.65	1.1	lomics Pos	0.99202	0.99987	0.95016	-0.07376	0.97443	0.99994	0.8396	-0.25223	0.91283	0.99996	0.78806	-0.34362	0.83226	0.98621	0.95106	-0.07239	0.67292	0.72237	1.2772	0.353	0.040045	0.18531	2.7706	1.4702	0.015467	0.33809	3.4856	1.8014	0.29342	0.9483	1.4805	0.56605	0.18378	0.29101	1.7349	0.79485
1111.786	0.5	lomics Pos	0.99217	0.99987	1.0013	0.001869	0.24588	0.99994	0.94572	-0.08052	0.22168	0.99996	0.92307	-0.11548	0.008086	0.19917	0.85966	-0.21817	0.000344	0.002463	0.70521	-0.50388	0.13049	0.39488	0.82653	-0.27487	0.46657	0.80647	0.94967	-0.07451	0.15321	0.91321	0.92988	-0.10489	0.11662	0.22101	1.1807	0.2397
528.3075	5.08	olomics Ne	0.99223	0.99918	1.0389	0.055082	0.49005	0.85323	1.0766	0.10646	0.29242	0.99995	1.2228	0.29013	0.46784	0.99985	1.1218	0.16584	0.2139	0.99927	1.2375	0.30743	0.010928	0.40245	1.3986	0.48402	0.043738	0.38044	1.4582	0.5442	0.33617	0.99512	1.1364	0.18451	0.090214	0.99941	1.2153	0.28129
977.686	6.1	lomics Pos	0.99299	0.99987	0.99823	-0.00255	0.72047	0.99994	0.94813	-0.07685	0.68128	0.99996	0.94111	-0.08756	0.67225	0.95717	0.93937	-0.09024	0.086589	0.15685	0.74405	-0.42652	0.010714	0.089443	0.6541	-0.61243	0.080849	0.4991	0.76416	-0.38805	0.062907	0.73915	0.73837	-0.43759	0.75022	0.81313	0.9559	-0.06506
881.513	5.89	lomics Pos	0.99324	0.99987	1.0107	0.015311	0.62614	0.99994	0.84706	-0.23946	0.61313	0.99996	0.84074	-0.25026	0.85259	0.9874	0.95303	-0.06941	0.83524	0.86075	1.0348	0.049342	0.050934	0.2212	1.6302	0.70504	0.028779	0.3527	1.9841	0.98849	0.061526	0.73084	1.6084	0.68559	0.013954	0.098791	2.4839	1.3126
913.5759	6.35	lomics Pos	0.99345	0.99987	#N/A	#N/A	0.62219	0.99994	0.9215	-0.11795	0.35415	0.99996	0.87259	-0.19663	0.25223	0.70924	0.81143	-0.30146	0.038113	0.085285	0.67248	-0.57245	0.077824	0.28838	0.64028	-0.64323	0.11339	0.56565	0.68506	-0.54571	0.078195	0.79555	0.67774	-0.56119	0.6379	0.72785	0.94184	-0.08645
1074.754	10.05	lomics Pos	0.99349	0.99987	0.99669	-0.00479	0.49132	0.99994	1.0786	0.10922	0.97711	0.99996	0.99691	-0.00447	0.23613	0.69488	1.0815	0.11298	0.035747	0.080977	0.84216	-0.24784	0.88737	0.94146	0.98935	-0.01544	0.76973	0.94358	1.0261	0.037155	0.084016	0.81413	1.1576	0.21114	0.013399	0.098791	1.2774	0.35326
137.8993	12.73	olomics Ne	0.99363	0.99918	0.99938	-0.0009	0.016065	0.37315	0.87873	-0.1865	0.63564	0.99995	1.0327	0.046399	0.85172	0.99985	0.99172	-0.012	0.73892	0.99927	0.9816	-0.02679	0.7835	0.95294	1.0227	0.032365	0.35641	0.71602	1.0466	0.065663	0.52779	0.99512	1.0294	0.041862	0.77235	0.99941	1.0093	0.013286
819.0579	4.77	lomics Pos	0.9939	0.99987	0.99616	-0.00555	0.26009	0.99994	0.83199	-0.26536	0.7828	0.99996	0.95463	-0.06699	0.18494	0.6253	0.78066	-0.35723	0.021326	0.05498	0.64931	-0.62303	0.17167	0.45871	0.74583	-0.42308	0.25421	0.69047	0.82983	-0.2691	0.65716	0.97988	0.93324	-0.09968	0.2762	0.38526	1.2084	0.27306
178.0562	0.31	olomics Pc	0.99426	0.99951	0.9915	-0.01231	0.8512	0.99998	0.96018	-0.05862	0.48612	0.99941	1.1554	0.2084	0.10114	0.42517	1.3203	0.40089	0.091184	0.26694	1.3804	0.46505	0.074751	0.24426	1.3352	0.41705	0.010615	0.24769	1.6214	0.69724	0.084218	0.79247	1.286	0.36295	0.94793	0.97899	0.99035	-0.01399
393.2231	6.83	olomics Pc	0.99455	0.99951	1.0164	0.023517	0.96661	0.99998	1.046	0.064888	0.020976	0.99941	0.73409	-0.44598	0.072746	0.35072	0.69761	-0.51951	0.000911	0.008772	0.61156	-0.70944	0.001196	0.044437	0.41564	-1.2666	0.010426	0.24769	0.5562	-0.84632	0.053744	0.69529	0.60741	-0.71927	0.075958	0.32186	0.66488	-0.58883
531.3209	5.11	olomics Pc	0.99484	0.99951	1.0527	0.074143	0.66473	0.99998	0.80677	-0.30978	0.73168	0.99941	1.0315	0.044734	0.51389	0.98281	1.295	0.37298	0.12743	0.35028	1.634	0.70837	0.027766	0.12621	2.0424	1.0302	0.018816	0.30136	2.4663	1.3023	0.39932	0.99956	1.3612	0.44493	0.17613	0.43997	1.5296	0.6132
1063.66	1.11	lomics Pos	0.99488	0.99987	0.84907	-0.23604	0.8754	0.99994	0.89711	-0.15664	0.79748	0.99996	1.0018	0.002579	0.73227	0.96985	0.95777	-0.06225	0.39686	0.48751	1.6665	0.73682	0.02385	0.13546	4.1034	2.0368	0.01173	0.3324	5.3473	2.4188	0.20392	0.92582	1.8748	0.90677	0.13057	0.23577	2.0887	1.0626
386.1892	6.34	olomics Pc	0.9951	0.99951	0.9818	-0.0265	0.30328	0.99998	0.85007	-0.23435	0.23057	0.99941	0.82491	-0.27769	0.10577	0.43684	0.69185	-0.53146	0.004053	0.025232	0.46895	-1.0925	0.002302	0.045971	0.4863	-1.0401	0.05334	0.46031	0.71386	-0.48628	0.083769	0.79241	0.75197	-0.41125	0.068128	0.31036	0.74446	-0.42574
351.2866	2.97	lomics Pos	0.99552	0.99987	1.0083	0.011934	0.32516	0.99994	0.81635	-0.29274	0.061174	0.99996	0.66771	-0.5827	0.005694	0.18075	0.43577	-1.1983	0.000265	0.002027	0.38251	-1.3864	0.049654	0.21687	0.53898	-0.8917	0.032884	0.36346	0.60658	-0.72122	0.000713	0.41831	0.3756	-1.4127	0.053814	0.15065	0.5593	-0.8383
791.5399	5.47	lomics Pos	0.99598	0.99987	0.99584	-0.00601	0.21295	0.99994	0.88863	-0.17035	0.15639	0.99996	0.85968	-0.21812	0.079976	0.47798	0.84056	-0.25057	0.000965	0.005434	0.6302	-0.66611	0.032061	0.16113	0.72663	-0.46071	0.42932	0.79094	0.92223	-0.1168	0.6554	0.97988	0.95031	-0.07353	0.98038	0.98477	1.0124	0.01779
144.1029	0.59	lomics Pos	0.99642	0.99987	0.9779	-0.03223	0.96307	0.99994	0.99392	-0.00879	0.8775	0.99996	1.0243	0.034689	0.91936	0.9874	0.93116	-0.10289	0.75868	0.79321	0.80388	-0.31495	0.36675	0.62673	1.4778	0.56343	0.049058	0.40631	2.4098	1.2689	0.62504	0.97813	1.1545	0.20733	0.30739	0.41711	1.9236	0.94378
489.2307	2.62	olomics Pc	0.99691	0.99951	0.96951	-0.04468	0.73251	0.99998	0.90042	-0.15133	0.1012	0.99941	0.59956	-0.73803	0.055048	0.30582	0.42868	-1.222	0.000702	0.007113	0.1888	-2.4051	0.048951	0.18379	0.41366	-1.2735	0.021516	0.31651	0.4294	-1.2196	0.044172	0.65531	0.5008	-0.99768	0.94364	0.97714	1.0315	0.044764
636.8781	10.02	lomics Pos	0.99692	0.99987	0.99489	-0.00739	0.51193	0.99994	1.0509	0.071595	0.80846	0.99996	1.0288	0.041032	0.41921	0.85533	0.92112	-0.11854	0.034187	0.078347	0.74312	-0.42833	0.57758	0.7694	0.93611	-0.09524	0.18405	0.64793	1.1423	0.19198	0.63771	0.97988	1.0477	0.067293	0.17468	0.28234	1.2679	0.34242
358.9764	11.98	olomics Ne	0.99699	0.99918	0.99849	-0.00218	0.98079	0.99664	0.99838	-0.00234	0.46863	0.99995	1.0549	0.077156	0.92322	0.99985	1.0228	0.032521	0.73322	0.99927	1.0453	0.063868	0.016122	0.40708	1.2114	0.27671	0.68458	0.89505	1.024	0.03424	0.32006	0.99512	1.0904	0.12483	0.67927	0.99941	1.0458	0.064655
565.333	5.09	olomics Ne	0.99711	0.99918	1.0038	0.005431	0.92615	0.9934	0.9665	-0.04916	0.64978	0.99995	1.1003	0.13795	0.49247	0.99985	1.1958	0.25801	0.22551	0.99927	1.4997	0.58464	0.017161	0.40708	2.1867	1.1287	0.009965	0.26668	2.2599	1.1762	0.17524	0.99512	1.467	0.55285	0.13585	0.99941	1.4518	0.53785
166.0858	0.58	lomics Pos	0.99739	0.99987	1.0096	0.013796	0.87915	0.99994	0.9807	-0.02812	0.62145	0.99996	1.156	0.20913	0.47615	0.88482	1.2567	0.3296	0.76451	0.798	0.91891	-0.122	0.28396	0.57131	1.3146	0.39461	0.015503	0.33809	1.9135	0.93618	0.70099	0.97988	1.094	0.12957	0.13152	0.23654	1.5513	0.63349
578.5206	5.89	lomics Pos	0.99744	0.99987	0.97196	-0.04104	0.96255	0.99994	0.95252	-0.07017	0.87384	0.99996	0.94873	-0.07594	0.62774	0.94496	0.85762	-0.22158	0.99522	0.99624	0.96144	-0.05673	0.058161	0.24091	1.527	0.61069	0.023691	0.33809	1.7792	0.83121	0.048417	0.67624	1.6399	0.7136	0.019582	0.10555	2.299	1.201
1196.714	0.53	lomics Pos	0.99749	0.99987	1.016	0.022884	0.95694	0.99994	0.99236	-0.01107	0.75814	0.99996	0.96963	-0.04449	0.60978	0.93636	0.9451	-0.08146	0.018451	0.049059	0.75638	-0.40282	0.96015	0.97889	1.0181	0.025829	0.51006	0.83279	1.066	0.092159	0.2629	0.94365	1.1333	0.18051	0.20086	0.30932	1.1941	0.25595
1044.799	0.5	lomics Pos	0.9975	0.99987	1.0046	0.006599	0.27076	0.99994	0.95397	-0.06799	0.29609	0.99996	0.93961	-0.08986	0.044851	0.36636	0.9098	-0.13638	0.005841	0.020566	0.77774	-0.36264	0.093252	0.32266	0.83214	-0.2651	0.60594	0.86762	0.97528	-0.03611	0.39473	0.96776	0.95322	-0.06912	0.00886	0.092495	1.1476	0.19864
240.1006	5.17	olomics Pc	0.99754	0.99951	1.0296	0.04204	0.94857	0.99998	1.0013	0.001813	0.77981	0.99941	1.0133	0.01902	0.32095	0.81532	1.251	0.32305	0.22953	0.54815	1.3431	0.42555	0.008874	0.075866	1.7878	0.83822	0.006127	0.21836	1.8663	0.9002	0.32992	0.99956	1.3195	0.39996	0.28314	0.55778	1.2781	0.35405
425.3313	4	lomics Pos	0.99826	0.99987	1.0007	0.000983	0.87584	0.99994	1.0119	0.017029	0.98086	0.99996	1.0101	0.014501	0.96323	0.99399	1.0044	0.006281	0.033195	0.076665	0.77108	-0.37504	0.44389	0.67607	0.92973	-0.10512	0.3559	0.75338	1.0746	0.10375	0.44591	0.96776	0.90486	-0.14424	0.21106	0.31986	1.0931	0.12846
558.2942	5.11	olomics Pc	0.99836	0.99951	1.0009	0.001326	0.75772	0.99998	0.85201	-0.23106	0.95606	0.99941	0.88786	-0.17159	0.57669	0.99905	1.2065	0.27087	0.1884	0.47439	1.5408	0.62365	0.093525	0.28464	1.5995	0.67766	0.044312	0.42298	1.9539	0.96638	0.21877	0.99956	1.4153	0.50107	0.1994	0.46423	1.3557	0.43901
562.3119	4.84	olomics Ne	0.99926	0.99977	0.97776	-0.03244	0.84931	0.98874	0.98114	-0.02748	0.6157	0.99995	1.0321	0.04555	0.58232	0.99985	1.1352	0.18292	0.49495	0.99927	1.2389	0.30902	0.055173	0.49216	2.4217	1.276	0.038046	0.35842	2.6434	1.4024	0.26159	0.99512	1.7082	0.77247	0.052266	0.99941	2.1195	1.0837
1173.758	10.02	lomics Pos	0.9995	0.99987	0.99823	-0.00255	0.29081	0.99994	0.88993	-0.16824	0.55015	0.99996	0.95401	-0.06792	0.21066	0.65841	0.91716	-0.12475	0.002998	0.012257	0.74728	-0.42028	0.48991	0.70839	0.92748	-0.10861	0.92793	0.98393	0.99589	-0.00594	0.90523	0.98197	0.99037	-0.01396	0.059448	0.15709	1.1936	0.25536
1000.785	10.03	lomics Pos	0.9996	0.99987	1.0027	0.003821	0.42903	0.99994	0.94373	-0.08356	0.54318	0.99996	0.95796	-0.06197	0.76966	0.97526	0.98322	-0.02441	0.000132	0.001244	0.70408	-0.50619	0.82634	0.91387	0.99152	-0.01229	0.60622	0.86762	1.0647	0.090485	0.55976	0.96928	1.0222	0.031667	0.001474	0.07528	1.249	0.32078
1068.505	5.92	lomics Pos	0.99987	0.99987	1.0009	0.001328	0.60962	0.99994	0.93451	-0.09772	0.6341	0.99996	0.94115	-0.08751	0.66692	0.95717	0.94756	-0.07772	0.011281	0.033711	0.73304	-0.44803	0.10961	0.35389	0.74149	-0.43149	0.10844	0.5577	0.81694	-0.2917	0.14325	0.9038	0.83628	-0.25795	0.77739	0.83452	1.0241	0.03434

200 mg/kg

			0.25 h vs. Pre				0.5 h vs. Pre				1 h vs. Pre				2 h vs. Pre				4 h vs. Pre				8 h vs. Pre				12 h vs. Pre				24 h vs. Pre				48 h vs. Pre			
m/z	*RT*	*Mode*	*p-value*	*FDR*	*Fold Change*	*Log2 (FC)*	*p-value*	*FDR*	*Fold Change*	*Log2 (FC)*	*p-value*	*FDR*	*Fold Change*	*Log2 (FC)*	*p-value*	*FDR*	*Fold Change*	*Log2 (FC)*	*p-value*	*FDR*	*Fold Change*	*Log2 (FC)*	*p-value*	*FDR*	*Fold Change*	*Log2 (FC)*	*p-value*	*FDR*	*Fold Change*	*Log2 (FC)*	*p-value*	*FDR*	*Fold Change*	*Log2 (FC)*	*p-value*	*FDR*	*Fold Change*	*Log2 (FC)*
866.7725	10.11	lomics Pos	0.29794	0.99995	0.86339	-0.21192	0.75682	0.99979	0.96362	-0.05346	0.49856	0.99601	0.91211	-0.13272	0.18122	0.99996	0.82703	-0.27399	0.21625	0.99598	0.8527	-0.22989	0.024616	0.99959	0.73987	-0.43465	0.86187	0.92105	1.0354	0.050145	0.22157	0.99984	0.85679	-0.22299	0.87269	0.99966	0.97231	-0.04051
360.9796	4.36	olomics Ne	0.68747	0.9951	0.9803	-0.02871	0.35921	0.99975	1.0492	0.069298	0.31071	0.99731	0.95991	-0.05903	0.35777	0.99915	1.0597	0.083668	0.416	0.77355	1.0517	0.072699	0.084965	0.52866	1.0938	0.12934	0.001954	0.04149	1.1958	0.25798	0.52131	0.99481	1.0263	0.037496	0.12625	0.91738	0.92186	-0.11739
448.9751	4.99	olomics Ne	0.82116	0.9951	1.01	0.014396	0.56139	0.99975	0.97132	-0.04199	0.073815	0.99731	0.92493	-0.11258	0.71146	0.99915	0.98021	-0.02883	0.16667	0.65299	1.0709	0.098885	0.1126	0.5383	1.0896	0.12379	0.009737	0.060961	1.1754	0.23321	0.95174	0.99481	0.99736	-0.00382	0.20047	0.91738	0.94641	-0.07946
285.2985	2.67	lomics Pos	0.01591	0.68962	0.79449	-0.33191	0.046734	0.99441	0.68518	-0.54544	0.001891	0.13302	0.69669	-0.52142	0.2681	0.99996	0.85376	-0.2281	0.00582	0.46849	0.73765	-0.43899	0.077162	0.99959	0.71095	-0.49217	0.17678	0.38852	1.2276	0.29585	0.12972	0.99984	0.72402	-0.4659	0.024418	0.99966	0.82139	-0.28387
215.6598	7.02	olomics Pc	0.18595	0.99964	0.89441	-0.16099	0.012758	0.88953	0.69648	-0.52184	0.016483	0.63482	0.78875	-0.34235	0.069158	0.99987	0.83508	-0.26002	0.15497	0.95986	0.89006	-0.16802	0.1601	0.99796	0.81446	-0.29609	0.69055	0.99768	1.0486	0.068415	0.16123	0.9981	0.805	-0.31294	0.14225	0.99995	0.83903	-0.25321
306.277	2.67	lomics Pos	0.02883	0.89298	0.76018	-0.39559	0.029919	0.99441	0.60036	-0.7361	0.001446	0.13123	0.67332	-0.57063	0.14605	0.99996	0.79134	-0.33764	0.041687	0.6519	0.78054	-0.35746	0.070568	0.99959	0.65669	-0.60671	0.28233	0.50314	1.1805	0.23938	0.17506	0.99984	0.74327	-0.42805	0.012556	0.99749	0.78827	-0.34324
110.1062	1.66	lomics Pos	0.1114	0.99995	0.82139	-0.28386	0.28654	0.99979	0.82671	-0.27455	0.067834	0.57097	0.70928	-0.49558	0.39508	0.99996	0.85808	-0.22082	0.04848	0.69757	0.69248	-0.53016	0.093562	0.99959	0.71112	-0.49183	0.86145	0.92086	1.0219	0.031211	0.18874	0.99984	0.73246	-0.44918	0.23169	0.99966	0.80312	-0.31632
284.2954	2.67	lomics Pos	0.015276	0.68962	0.80569	-0.3117	0.040187	0.99441	0.67698	-0.56282	0.002508	0.14271	0.7088	-0.49656	0.2262	0.99996	0.84458	-0.2437	0.006983	0.46849	0.74951	-0.41598	0.069667	0.99959	0.70302	-0.50836	0.19067	0.40393	1.2105	0.27566	0.14225	0.99984	0.74203	-0.43044	0.017734	0.99966	0.8188	-0.28843
308.2954	2.32	lomics Pos	0.1337	0.99995	0.87799	-0.18773	0.054758	0.99441	0.79189	-0.33664	0.007449	0.19587	0.74278	-0.429	0.41443	0.99996	0.8926	-0.16391	0.051864	0.70881	0.78609	-0.34723	0.092319	0.99959	0.74306	-0.42846	0.25546	0.47308	1.2216	0.2888	0.092496	0.99984	0.74932	-0.41635	0.32847	0.99966	0.92333	-0.11508
285.299	6.91	olomics Pc	9.88E-05	0.15559	0.7901	-0.3399	0.037781	0.88953	0.69711	-0.52054	0.001005	0.35157	0.78268	-0.35351	0.14639	0.99987	0.89722	-0.15647	0.02926	0.95986	0.85366	-0.22827	0.061464	0.99796	0.73579	-0.44263	0.22359	0.99768	1.0539	0.07576	0.099737	0.9981	0.73641	-0.44142	0.013752	0.99995	0.83824	-0.25457
1161.739	10.03	lomics Pos	0.45146	0.99995	0.9332	-0.09974	0.62626	0.99979	0.94968	-0.07448	0.21166	0.83096	0.84768	-0.23841	0.93635	0.99996	1.0164	0.023406	0.97301	0.99701	1.0054	0.007756	0.71734	0.99959	1.0613	0.085817	0.014077	0.16859	1.3912	0.4763	0.76157	0.99984	0.97608	-0.03493	0.36142	0.99966	1.139	0.18771
242.2479	5.83	olomics Pc	0.019638	0.86976	0.82519	-0.2772	0.05545	0.91931	0.73334	-0.44744	0.073159	0.99882	0.81821	-0.28945	0.057436	0.99987	0.84552	-0.24208	0.15761	0.95986	0.87122	-0.19889	0.15857	0.99796	0.77419	-0.36924	0.8297	0.99768	1.0232	0.033122	0.088128	0.9981	0.76414	-0.38809	0.024494	0.99995	0.80233	-0.31773
293.2844	2.76	lomics Pos	0.026051	0.85508	0.82744	-0.27327	0.026876	0.99441	0.67775	-0.56118	0.00173	0.13302	0.69065	-0.53397	0.22288	0.99996	0.82952	-0.26965	0.006122	0.46849	0.73356	-0.44701	0.074465	0.99959	0.69613	-0.52257	0.29458	0.51572	1.1738	0.23125	0.12022	0.99984	0.73659	-0.44106	0.009933	0.99749	0.79673	-0.32783
312.3265	3.43	lomics Pos	0.10714	0.99995	0.82216	-0.2825	0.052082	0.99441	0.67097	-0.57569	0.014759	0.26926	0.70523	-0.50383	0.1242	0.99996	0.76644	-0.38375	0.008925	0.46849	0.73223	-0.44964	0.088084	0.99959	0.67688	-0.56303	0.23351	0.45419	1.2568	0.32975	0.11448	0.99984	0.71906	-0.47581	0.014047	0.99749	0.75644	-0.40271
276.2761	7.02	olomics Pc	0.005305	0.49147	0.7821	-0.35458	0.038943	0.88953	0.71779	-0.47836	0.004637	0.48835	0.70168	-0.51111	0.090339	0.99987	0.86032	-0.21705	0.015442	0.95986	0.7947	-0.33152	0.057673	0.99796	0.72645	-0.46107	0.73676	0.99768	1.0189	0.027082	0.037125	0.9981	0.69767	-0.51939	0.002718	0.7981	0.76791	-0.38099
268.2638	2.76	lomics Pos	0.26581	0.99995	0.8551	-0.22583	0.12072	0.99979	0.74615	-0.42247	0.019369	0.30022	0.68986	-0.53562	0.4198	0.99996	0.88488	-0.17644	0.062044	0.76594	0.71562	-0.48274	0.18779	0.99959	0.79238	-0.33574	0.45948	0.67219	1.1703	0.2269	0.25543	0.99984	0.78078	-0.35702	0.15149	0.99966	0.80495	-0.31302
202.6516	2.67	lomics Pos	0.053571	0.99995	0.73113	-0.45181	0.19852	0.99979	0.7657	-0.38514	0.033292	0.39059	0.66604	-0.58632	0.41992	0.99996	0.86798	-0.20427	0.036231	0.62141	0.68156	-0.55309	0.34336	0.99959	0.82319	-0.2807	0.12432	0.32476	1.3103	0.38985	0.26995	0.99984	0.75775	-0.40021	0.40228	0.99966	0.86774	-0.20466
295.2832	7.02	olomics Pc	0.02757	0.9464	0.83426	-0.26143	0.015824	0.88953	0.65112	-0.61901	0.002057	0.39871	0.79056	-0.33905	0.021045	0.99987	0.77746	-0.36316	0.015244	0.95986	0.7795	-0.35938	0.12237	0.99796	0.7641	-0.38816	0.39686	0.99768	0.96317	-0.05413	0.089454	0.9981	0.72328	-0.46737	0.13135	0.99995	0.79558	-0.32991
500.9063	10.02	lomics Pos	0.20348	0.99995	1.1467	0.1975	0.39318	0.99979	1.0801	0.11122	0.063901	0.55764	1.119	0.16215	0.3151	0.99996	1.0884	0.1222	0.72407	0.99701	0.98588	-0.02052	0.57207	0.99959	1.0494	0.069533	0.01721	0.17568	1.3091	0.38862	0.45338	0.99984	1.0694	0.09681	0.87387	0.99966	0.99327	-0.00974
311.3143	2.76	lomics Pos	0.033399	0.92284	0.84069	-0.25036	0.037328	0.99441	0.70033	-0.51388	0.001021	0.13107	0.70194	-0.51057	0.25081	0.99996	0.84527	-0.24252	0.010125	0.46849	0.76048	-0.39502	0.072188	0.99959	0.70372	-0.50693	0.28781	0.50853	1.1762	0.2341	0.14194	0.99984	0.75344	-0.40843	0.01327	0.99749	0.8171	-0.29142
480.9747	5.16	olomics Ne	0.88666	0.9951	1.0063	0.009017	0.73969	0.99975	1.019	0.027145	0.23076	0.99731	0.96139	-0.05681	0.41596	0.99915	1.0536	0.075369	0.10486	0.60605	1.102	0.14015	0.043016	0.4903	1.1031	0.14159	0.004056	0.050041	1.2392	0.30937	0.51889	0.99481	1.0247	0.035201	0.47084	0.94605	0.96865	-0.04595
192.1833	6.38	olomics Pc	0.03308	0.99964	0.84957	-0.23519	0.15692	1	0.85932	-0.21873	0.17184	0.99882	0.90402	-0.14557	0.85459	0.99987	0.98593	-0.02044	0.10698	0.95986	0.88346	-0.17877	0.30604	0.99796	0.88197	-0.1812	0.1061	0.90395	1.1565	0.20979	0.22073	0.9981	0.84121	-0.24946	0.27721	0.99995	0.8839	-0.17804
294.2877	2.76	lomics Pos	0.11212	0.99995	0.85709	-0.22248	0.050301	0.99441	0.69881	-0.51702	0.001353	0.13107	0.70684	-0.50054	0.26342	0.99996	0.85137	-0.23214	0.013013	0.48469	0.75306	-0.40915	0.10453	0.99959	0.73588	-0.44246	0.26697	0.48567	1.2	0.26307	0.14988	0.99984	0.75695	-0.40173	0.025975	0.99966	0.81073	-0.30271
217.1963	1.23	lomics Pos	0.53765	0.99995	0.94504	-0.08155	0.33966	0.99979	0.91994	-0.12038	0.12402	0.72566	0.83485	-0.26041	0.56101	0.99996	0.94054	-0.08843	0.027188	0.55437	0.83288	-0.26382	0.50893	0.99959	0.92871	-0.10669	0.026151	0.19102	1.3979	0.48324	0.52216	0.99984	0.93769	-0.09282	0.56529	0.99966	0.95951	-0.05963
241.2211	6.39	olomics Pc	0.001599	0.29623	0.8133	-0.29815	0.00149	0.88953	0.75723	-0.4012	0.009636	0.55189	0.83826	-0.25454	0.55422	0.99987	0.95444	-0.06728	0.097121	0.95986	0.90559	-0.14307	0.090435	0.99796	0.88246	-0.18039	0.097082	0.87638	1.0664	0.092796	0.099983	0.9981	0.84661	-0.24022	0.062097	0.99995	0.88427	-0.17744
286.3016	2.66	lomics Pos	0.036968	0.95889	0.79062	-0.33895	0.063406	0.99979	0.64378	-0.63536	0.008335	0.19873	0.67384	-0.56952	0.11992	0.99996	0.79916	-0.32344	0.024213	0.5516	0.69891	-0.51682	0.060839	0.99959	0.65408	-0.61247	0.42168	0.63923	1.1688	0.22498	0.12974	0.99984	0.72106	-0.4718	0.018272	0.99966	0.77366	-0.37023
512.9775	5.19	olomics Ne	0.11738	0.9951	1.0444	0.062678	0.16419	0.99975	1.0468	0.065966	0.54539	0.99731	0.98086	-0.02788	0.28329	0.99915	1.0453	0.063923	0.03054	0.51873	1.0893	0.12338	0.039407	0.47098	1.1329	0.17997	0.012944	0.062979	1.2272	0.2954	0.1059	0.99481	1.0767	0.10663	0.70566	0.97327	0.98618	-0.02007
125.0966	1.66	lomics Pos	0.10733	0.99995	0.88457	-0.17696	0.03339	0.99441	0.71019	-0.49371	0.009346	0.21077	0.74448	-0.4257	0.39561	0.99996	0.91031	-0.13557	0.017098	0.49216	0.78039	-0.35773	0.030643	0.99959	0.68745	-0.54067	0.64874	0.7959	1.1132	0.15468	0.15898	0.99984	0.80555	-0.31196	0.12032	0.99966	0.8811	-0.18263
169.1586	2.05	lomics Pos	0.10813	0.99995	0.84288	-0.24661	0.24106	0.99979	0.80967	-0.3046	0.022861	0.32238	0.73085	-0.45234	0.47903	0.99996	0.90641	-0.14177	0.14816	0.90678	0.81032	-0.30344	0.30147	0.99959	0.84524	-0.24257	0.072163	0.25957	1.3096	0.38914	0.39338	0.99984	0.87345	-0.1952	0.57295	0.99966	0.93228	-0.10117
136.1206	2.05	lomics Pos	0.003036	0.59581	0.79017	-0.33977	0.059442	0.99979	0.74713	-0.42057	0.005333	0.17089	0.73177	-0.45053	0.27772	0.99996	0.88745	-0.17226	0.027098	0.55437	0.82338	-0.28038	0.1284	0.99959	0.77004	-0.37699	0.10542	0.30498	1.2367	0.3065	0.24447	0.99984	0.80872	-0.30628	0.080038	0.99966	0.88696	-0.17307
152.889	0.34	olomics Ne	0.85104	0.9951	0.98866	-0.01646	0.81799	0.99975	0.98501	-0.02179	0.90398	0.99731	0.99396	-0.00874	0.73808	0.99915	1.0261	0.037128	0.042973	0.55684	1.1557	0.20878	0.41873	0.77401	1.0566	0.079494	0.29157	0.40787	1.101	0.13878	0.34184	0.99481	1.0423	0.059715	0.27117	0.91738	0.93928	-0.09037
313.3296	3.43	lomics Pos	0.1708	0.99995	0.80472	-0.31345	0.050819	0.99441	0.70292	-0.50857	0.005895	0.17561	0.6189	-0.69222	0.24353	0.99996	0.82339	-0.28036	0.015032	0.4899	0.69924	-0.51614	0.033056	0.99959	0.67108	-0.57545	0.50689	0.70564	1.1524	0.20465	0.094336	0.99984	0.66561	-0.58726	0.048683	0.99966	0.75696	-0.40171
752.9739	12.01	olomics Ne	0.74239	0.9951	1.054	0.075869	0.091692	0.99975	1.1416	0.19108	0.6476	0.99731	0.96038	-0.05832	0.31907	0.99915	1.2158	0.28195	0.36167	0.75206	1.0829	0.11496	0.21602	0.6375	1.1471	0.19794	0.041217	0.11376	1.1702	0.22682	0.51406	0.99481	1.0612	0.085698	0.046726	0.91738	1.2507	0.32271
184.1699	2.76	lomics Pos	0.11709	0.99995	0.91698	-0.12504	0.025658	0.99441	0.68346	-0.54908	0.00038	0.13107	0.7219	-0.47014	0.24298	0.99996	0.83763	-0.25561	0.013901	0.4899	0.78075	-0.35708	0.059674	0.99959	0.6571	-0.60581	0.18823	0.40185	1.1636	0.21864	0.22556	0.99984	0.79005	-0.33998	0.002814	0.99749	0.72762	-0.45874
312.3174	2.76	lomics Pos	0.046027	0.99995	0.81348	-0.29782	0.049081	0.99441	0.70628	-0.5017	0.000765	0.13107	0.69388	-0.52723	0.17361	0.99996	0.81742	-0.29086	0.01584	0.49216	0.73888	-0.43658	0.056222	0.99959	0.66815	-0.58176	0.45142	0.66535	1.1335	0.18083	0.093584	0.99984	0.72315	-0.46764	0.009161	0.99749	0.80303	-0.31647
150.1366	6	olomics Pc	0.016059	0.86976	0.81624	-0.29293	0.15508	1	0.83468	-0.26071	0.13692	0.99882	0.88889	-0.16993	0.86648	0.99987	1.0506	0.071152	0.81288	0.99139	0.98226	-0.02583	0.49992	0.99796	0.90831	-0.13874	0.30374	0.99768	1.1272	0.17275	0.52824	0.9981	0.92447	-0.1133	0.89835	0.99995	0.99571	-0.0062
291.1747	5.16	olomics Ne	0.1074	0.9951	0.95477	-0.06677	0.082235	0.99975	1.0813	0.11277	0.35851	0.99731	1.0274	0.039025	0.40355	0.99915	1.0782	0.10868	0.21769	0.70622	1.0862	0.11924	0.35793	0.74235	1.1029	0.14126	0.012627	0.062293	1.2005	0.26359	0.19721	0.99481	1.0687	0.095817	0.34626	0.92398	1.0397	0.056224
630.7971	10.13	lomics Pos	0.96549	0.99995	1.0096	0.013754	0.96438	0.99979	1.0104	0.014912	0.03637	0.41645	0.82769	-0.27285	0.45974	0.99996	1.0585	0.081988	0.19309	0.97027	0.92201	-0.11714	0.8161	0.99959	1.0169	0.024107	0.80165	0.8842	1.0396	0.055979	0.82805	0.99984	1.0234	0.03344	0.58757	0.99966	0.96729	-0.04798
111.0909	1.66	lomics Pos	0.82679	0.99995	0.9879	-0.01757	0.28885	0.99979	0.87941	-0.1854	0.011551	0.23303	0.82085	-0.28481	0.33777	0.99996	0.88696	-0.17305	0.033012	0.59375	0.81749	-0.29073	0.28681	0.99959	0.87684	-0.18961	0.071581	0.25937	1.2647	0.33874	0.10939	0.99984	0.77963	-0.35914	0.94247	0.99966	1.0021	0.002988
310.3111	2.76	lomics Pos	0.034555	0.94348	0.84235	-0.2475	0.034857	0.99441	0.69729	-0.52017	0.001298	0.13107	0.7154	-0.48317	0.23569	0.99996	0.84084	-0.2501	0.009271	0.46849	0.75333	-0.40865	0.080213	0.99959	0.71387	-0.48627	0.24621	0.46637	1.1868	0.24703	0.14366	0.99984	0.75429	-0.40681	0.013947	0.99749	0.81538	-0.29446
304.2616	2.05	lomics Pos	0.032712	0.91043	0.85675	-0.22305	0.081016	0.99979	0.79848	-0.32467	0.008075	0.19873	0.7679	-0.38101	0.37244	0.99996	0.89384	-0.16192	0.02322	0.5516	0.79879	-0.32411	0.1612	0.99959	0.81625	-0.29291	0.096092	0.29197	1.1965	0.25888	0.12443	0.99984	0.75947	-0.39694	0.13766	0.99966	0.8984	-0.15458
195.1747	2.05	lomics Pos	0.022407	0.8137	0.84318	-0.24609	0.087568	0.99979	0.77416	-0.36929	0.000657	0.13107	0.73529	-0.44362	0.3194	0.99996	0.88379	-0.17822	0.005922	0.46849	0.78317	-0.3526	0.11343	0.99959	0.78017	-0.35814	0.075373	0.26318	1.2367	0.30654	0.14051	0.99984	0.77429	-0.36906	0.045504	0.99966	0.88337	-0.17891
142.1226	2.76	lomics Pos	0.23382	0.99995	0.86663	-0.20651	0.016142	0.99441	0.66585	-0.58673	0.005486	0.17146	0.72985	-0.45434	0.2279	0.99996	0.85966	-0.21816	0.06435	0.76594	0.84957	-0.23519	0.17946	0.99959	0.7825	-0.35384	0.10534	0.30498	1.2829	0.35944	0.30305	0.99984	0.83512	-0.25994	0.11118	0.99966	0.8384	-0.25429
456.9788	4.84	olomics Ne	0.66422	0.9951	1.0365	0.051677	0.19342	0.99975	1.0758	0.10547	0.40205	0.99731	0.96064	-0.05793	0.24145	0.99915	1.0669	0.093437	0.37505	0.75206	1.0675	0.094237	0.10782	0.53696	1.0962	0.13253	0.020179	0.075503	1.191	0.25221	0.63178	0.99481	1.0291	0.041399	0.43097	0.94458	0.95568	-0.06541
456.8687	10.15	lomics Pos	0.30349	0.99995	1.1769	0.23498	0.97577	0.99979	1.0001	0.000167	0.79626	0.99601	1.0786	0.10919	0.66063	0.99996	0.95121	-0.07216	0.96103	0.99701	0.99932	-0.00098	0.92113	0.99959	1.017	0.024367	0.046725	0.22017	1.4534	0.53942	0.12064	0.99984	1.1982	0.26082	0.30509	0.99966	1.1133	0.15491
275.2738	2.76	lomics Pos	0.045646	0.99995	0.85605	-0.22423	0.027651	0.99441	0.67444	-0.56825	0.001919	0.13302	0.71978	-0.47437	0.2796	0.99996	0.85437	-0.22707	0.010386	0.46849	0.73339	-0.44734	0.11513	0.99959	0.7286	-0.45681	0.14925	0.35747	1.2004	0.26355	0.1287	0.99984	0.75772	-0.40026	0.020319	0.99966	0.81868	-0.28863
240.2327	2.75	lomics Pos	0.11308	0.99995	0.85023	-0.23408	0.022376	0.99441	0.6652	-0.58813	0.002228	0.13479	0.70067	-0.51319	0.32199	0.99996	0.84988	-0.23466	0.024931	0.5516	0.76897	-0.379	0.10226	0.99959	0.72239	-0.46916	0.24975	0.46958	1.1836	0.24317	0.092106	0.99984	0.76827	-0.38031	0.014388	0.99749	0.77943	-0.35951
147.1172	1.67	lomics Pos	0.082506	0.99995	0.87939	-0.18543	0.039579	0.99441	0.7749	-0.36792	0.00477	0.16683	0.74705	-0.42072	0.51433	0.99996	0.9244	-0.11341	0.00592	0.46849	0.78342	-0.35215	0.17622	0.99959	0.81601	-0.29334	0.076962	0.26509	1.2959	0.37397	0.40127	0.99984	0.87912	-0.18588	0.34434	0.99966	0.92961	-0.1053

175.1487	1.67	domics Pos	0.002296	0.59581	0.75987	-0.39618	0.034758	0.99441	0.74862	-0.41769	0.002202	0.13479	0.71223	-0.48959	0.21969	0.99996	0.84889	-0.23635	0.003029	0.46849	0.77824	-0.36171	0.15944	0.99959	0.76891	-0.37911	0.22972	0.45179	1.1251	0.1701	0.095779	0.99984	0.74859	-0.41775	0.10105	0.99966	0.88065	-0.18336
150.1365	1.66	domics Pos	0.60275	0.99995	0.96351	-0.05362	0.01244	0.99441	0.73434	-0.44547	0.005755	0.17488	0.76376	-0.38881	0.29118	0.99996	0.85985	-0.21785	0.00562	0.46849	0.80299	-0.31654	0.17736	0.99959	0.78755	-0.34457	0.0945	0.29106	1.2189	0.28553	0.40995	0.99984	0.89387	-0.16187	0.3172	0.99966	0.91127	-0.13405
390.1997	4.11	olomics Po	0.038221	0.99964	0.58695	-0.76868	0.01996	0.88953	0.68813	-0.53925	0.090543	0.99882	0.75136	-0.41242	0.033406	0.99987	0.6395	-0.64497	0.000632	0.95986	0.45983	-1.1208	0.071023	0.99796	0.67844	-0.55971	0.58779	0.99768	0.9251	-0.11232	0.11657	0.9981	0.66507	-0.58841	0.04755	0.99995	0.61134	-0.70995
286.2999	6.9	olomics Po	0.007385	0.60235	0.7425	-0.42953	0.046206	0.88953	0.68746	-0.54064	0.00355	0.43007	0.72913	-0.45575	0.3077	0.99987	0.89685	-0.15706	0.060947	0.95986	0.80893	-0.30591	0.12835	0.99796	0.78036	-0.35778	0.63954	0.99768	1.0418	0.059042	0.11304	0.9981	0.71825	-0.47744	0.080972	0.99995	0.82386	-0.27953
249.2493	6.38	olomics Po	0.030607	0.98816	0.83134	-0.2665	0.076913	0.99372	0.76468	-0.38707	0.017592	0.63482	0.87182	-0.19791	0.52739	0.99987	0.94566	-0.0806	0.21723	0.95986	0.8866	-0.17365	0.064203	0.99796	0.78203	-0.35471	0.25693	0.99768	1.0541	0.075944	0.11022	0.9981	0.78856	-0.3427	0.039267	0.99995	0.85926	-0.21883
98.104	2.05	domics Pos	0.14789	0.99995	0.86835	-0.20365	0.079973	0.99979	0.71816	-0.47762	0.007915	0.19873	0.72578	-0.46239	0.008382	0.99996	0.71543	-0.48312	0.31797	0.99701	0.87743	-0.18865	0.14747	0.99959	0.76365	-0.38901	0.347	0.5708	1.2097	0.27469	0.32679	0.99984	0.85074	-0.23321	0.25011	0.99966	0.90404	-0.14554
179.1801	2.05	domics Pos	0.001139	0.59581	0.72428	-0.46538	0.035961	0.99441	0.63132	-0.66356	0.003526	0.15208	0.73846	-0.43741	0.10115	0.99996	0.80606	-0.31104	0.002356	0.46849	0.68277	-0.55052	0.10686	0.99959	0.75068	-0.41373	0.15947	0.36844	1.1232	0.16756	0.11055	0.99984	0.73746	-0.43936	0.083129	0.99966	0.82233	-0.28221
256.2642	1.91	domics Pos	0.012267	0.68962	0.85015	-0.23421	0.063502	0.99979	0.76165	-0.3928	0.002317	0.13479	0.75573	-0.40405	0.34335	0.99996	0.89842	-0.15454	0.010516	0.46849	0.81718	-0.29128	0.1857	0.99959	0.80836	-0.30692	0.069917	0.25604	1.2082	0.27291	0.21689	0.99984	0.81213	-0.30021	0.15869	0.99966	0.90547	-0.14326
301.2528	6.38	olomics Po	0.038621	0.99964	0.84592	-0.2414	0.017857	0.88953	0.77916	-0.36	0.007852	0.5377	0.77245	-0.37249	0.16339	0.99987	0.92629	-0.11047	0.016267	0.95986	0.84671	-0.24005	0.2773	0.99796	0.85003	-0.23442	0.075523	0.83633	1.0985	0.13548	0.054576	0.9981	0.74004	-0.43432	0.083944	0.99995	0.86585	-0.20781
284.2959	6.91	olomics Po	1.51E-05	0.047666	0.78311	-0.35272	0.036072	0.88953	0.68841	-0.53867	0.000455	0.35157	0.76973	-0.37757	0.098966	0.99987	0.8796	-0.18507	0.012928	0.95986	0.83972	-0.25202	0.057426	0.99796	0.73655	-0.44115	0.19357	0.99768	1.0557	0.078185	0.10071	0.9981	0.7377	-0.43889	0.010103	0.99995	0.82429	-0.27877
191.18	2.05	domics Pos	0.042723	0.99995	0.83759	-0.25569	0.081094	0.99979	0.78005	-0.35837	0.005465	0.17146	0.74725	-0.42034	0.33578	0.99996	0.88032	-0.1839	0.062594	0.76594	0.80897	-0.30584	0.15147	0.99959	0.79839	-0.32484	0.093251	0.28973	1.1942	0.25605	0.15008	0.99984	0.76504	-0.38639	0.14754	0.99966	0.88606	-0.17452
193.1587	1.66	domics Pos	0.087189	0.99995	0.87774	-0.18813	0.009424	0.99441	0.6668	-0.58468	0.004043	0.15415	0.69944	-0.51573	0.20537	0.99996	0.83157	-0.26609	0.002962	0.46849	0.75494	-0.40556	0.19888	0.99959	0.80855	-0.3066	0.033933	0.20254	1.2071	0.27152	0.18492	0.99984	0.81402	-0.29687	0.079333	0.99966	0.8666	-0.20656
177.1644	2.05	domics Pos	0.015445	0.68962	0.84894	-0.23627	0.069684	0.99979	0.76854	-0.37981	0.004634	0.16474	0.76471	-0.38702	0.33525	0.99996	0.89032	-0.1676	0.016246	0.49216	0.79633	-0.32856	0.14036	0.99959	0.7837	-0.35163	0.20671	0.4224	1.1689	0.22511	0.14545	0.99984	0.77952	-0.35934	0.067551	0.99966	0.86058	-0.21661
227.2206	6.38	olomics Po	0.028572	0.96775	0.81176	-0.30087	0.029155	0.88953	0.77228	-0.3728	0.076693	0.99882	0.83602	-0.25839	0.492	0.99987	0.93999	-0.08928	0.16881	0.95986	0.89896	-0.15368	0.19098	0.99796	0.81891	-0.28822	0.3118	0.99768	1.0861	0.1191	0.031025	0.9981	0.74441	-0.42583	0.16397	0.99995	0.85103	-0.23272
134.9491	12.59	olomics Ne	0.4948	0.9951	1.0381	0.053927	0.086464	0.99975	1.0877	0.12127	0.7227	0.99731	0.98306	-0.02465	0.1404	0.99915	1.0868	0.1201	0.029672	0.51873	1.1504	0.20216	0.060427	0.51317	1.1204	0.16401	0.00052	0.041313	1.2966	0.37477	0.44237	0.99481	1.0306	0.043433	0.37707	0.92651	0.95562	-0.06549
168.1394	1.66	domics Pos	0.010105	0.68962	0.80418	-0.31442	0.029011	0.99441	0.7282	-0.45759	0.006176	0.17872	0.72778	-0.45843	0.12539	0.99996	0.7808	-0.35698	0.017042	0.49216	0.7791	-0.36012	0.071309	0.99959	0.74842	-0.41809	0.1921	0.40512	1.1274	0.17302	0.10949	0.99984	0.74617	-0.42242	0.10782	0.99966	0.84213	-0.24789
293.2848	7.02	olomics Po	0.000891	0.29623	0.8037	-0.31527	0.030539	0.88953	0.70077	-0.51299	0.000734	0.35157	0.74228	-0.42996	0.077057	0.99987	0.85951	-0.21842	0.016495	0.95986	0.84176	-0.24852	0.054576	0.99796	0.72834	-0.45731	0.29324	0.99768	1.0451	0.063625	0.056882	0.9981	0.71155	-0.49097	0.005741	0.99388	0.80082	-0.32046
207.1753	5.99	olomics Po	0.016547	0.86976	0.85338	-0.22874	0.080863	0.99372	0.77401	-0.36957	0.002405	0.39871	0.82022	-0.28592	0.85831	0.99987	0.99586	-0.00598	0.038314	0.95986	0.90367	-0.14613	0.11573	0.99796	0.80298	-0.31656	0.096448	0.87638	1.1034	0.14199	0.11536	0.9981	0.77589	-0.36607	0.097838	0.99995	0.88063	-0.18339
651.8512	0.51	domics Pos	0.54036	0.99995	1.0698	0.097346	0.27987	0.99979	1.0994	0.13673	0.51902	0.99601	0.93339	-0.09944	0.48936	0.99996	1.1411	0.19043	0.46529	0.99701	0.93241	-0.10096	0.04081	0.99959	1.2408	0.31129	0.074025	0.26183	1.4705	0.55628	0.47269	0.99984	1.0695	0.096967	0.14397	0.99966	1.1646	0.21978
67.0556	6.39	olomics Po	0.19452	0.99964	0.90185	-0.14904	0.024591	0.88953	0.76623	-0.38414	0.58667	0.99882	0.95458	-0.06705	0.76546	0.99987	1.0766	0.10647	0.90327	0.99906	0.98889	-0.01612	0.18779	0.99796	0.80847	-0.30673	0.35149	0.99768	1.0723	0.10071	0.49248	0.9981	0.93121	-0.10282	0.070573	0.99995	0.81756	-0.2906
623.5285	2.05	domics Pos	0.00464	0.60743	0.74524	-0.42422	0.044389	0.99441	0.65729	-0.60539	0.003833	0.15284	0.67478	-0.56751	0.42674	0.99996	0.90781	-0.13954	0.007289	0.46849	0.71815	-0.47764	0.074663	0.99959	0.75351	-0.4083	0.071795	0.2595	1.2595	0.33283	0.13702	0.99984	0.75811	-0.39952	0.02808	0.99966	0.83745	-0.25593
137.1331	2.05	domics Pos	0.010214	0.68962	0.86961	-0.20155	0.042744	0.99441	0.75223	-0.41076	0.002723	0.14832	0.76742	-0.38191	0.21008	0.99996	0.85724	-0.22224	0.023418	0.5516	0.80155	-0.31914	0.057459	0.99959	0.73277	-0.44857	0.07764	0.26646	1.1929	0.25448	0.12625	0.99984	0.76943	-0.37814	0.1311	0.99966	0.90032	-0.15149
275.2742	7.02	olomics Po	0.00328	0.38271	0.85073	-0.23322	0.051424	0.90372	0.71404	-0.48592	0.000691	0.35157	0.77353	-0.37047	0.19284	0.99987	0.8618	-0.21457	0.046373	0.95986	0.8824	-0.18049	0.086028	0.99796	0.73453	-0.44511	0.054474	0.77855	1.1116	0.15268	0.12688	0.9981	0.76189	-0.39235	0.020593	0.99995	0.8325	-0.26448
382.2028	2.05	domics Pos	0.009823	0.68962	0.88532	-0.17573	0.055962	0.99653	0.80889	-0.30599	0.62787	0.99601	1.7926	0.84209	0.52935	0.99996	0.95171	-0.07141	0.012953	0.48469	0.79884	-0.32401	0.046712	0.99959	0.81858	-0.28881	0.05504	0.23421	1.2522	0.32452	0.031591	0.99984	0.86179	-0.21459	0.055965	0.99966	0.90979	-0.13639
215.6598	2.76	domics Pos	0.01732	0.73379	0.79382	-0.33312	0.067764	0.99979	0.71254	-0.48897	0.007935	0.19873	0.70515	-0.504	0.014441	0.99996	0.73865	-0.43704	0.001847	0.46849	0.70838	-0.49741	0.068312	0.99959	0.68696	-0.54171	0.17996	0.39232	1.1428	0.19261	0.0853	0.99984	0.69931	-0.516	0.039927	0.99966	0.85686	-0.22287
150.137	6.38	olomics Po	0.077142	0.99964	0.93057	-0.10382	0.004579	0.88953	0.7811	-0.35643	0.020697	0.65853	0.88391	-0.17803	0.60427	0.99987	0.95689	-0.06357	0.47605	0.95986	0.96434	-0.05239	0.061751	0.99796	0.77699	-0.36404	0.78723	0.99768	1.0161	0.023096	0.031171	0.9981	0.78127	-0.3561	0.002251	0.7981	0.83292	-0.26374
210.1858	1.67	domics Pos	0.010627	0.68962	0.84262	-0.24704	0.02739	0.99441	0.73788	-0.43854	0.001038	0.13107	0.74083	-0.43278	0.18317	0.99996	0.82372	-0.27978	0.0085	0.46849	0.77524	-0.36728	0.096323	0.99959	0.75298	-0.40931	0.16577	0.37512	1.1599	0.21399	0.12699	0.99984	0.76264	-0.39092	0.034451	0.99966	0.8508	-0.2331
205.1956	2.05	domics Pos	0.008891	0.68962	0.82207	-0.28267	0.071877	0.99979	0.75086	-0.41338	0.003428	0.15208	0.77098	-0.37523	0.35857	0.99996	0.8847	-0.17674	0.009392	0.46849	0.81177	-0.30086	0.070599	0.99959	0.73565	-0.4429	0.10193	0.2999	1.2236	0.29114	0.044303	0.99984	0.71905	-0.47584	0.033278	0.99966	0.84777	-0.23826
276.2769	2.76	domics Pos	0.20081	0.99995	0.87076	-0.19965	0.035304	0.99441	0.65353	-0.61368	0.012291	0.23979	0.74852	-0.41788	0.21609	0.99996	0.83114	-0.26684	0.026146	0.5516	0.76355	-0.3892	0.028624	0.99959	0.6133	-0.70535	0.21641	0.43499	1.2281	0.29649	0.1794	0.99984	0.76285	-0.39053	0.14913	0.99966	0.85918	-0.21896
626.528	2.05	domics Pos	0.000995	0.59581	0.7676	-0.38157	0.073805	0.99979	0.69214	-0.53086	0.02022	0.30717	0.78509	-0.34908	0.26444	0.99996	0.85485	-0.22626	0.000853	0.46849	0.76627	-0.38408	0.23261	0.99959	0.7954	-0.33025	0.083867	0.27473	1.3652	0.44912	0.25308	0.99984	0.8003	-0.32138	0.1149	0.99966	0.89925	-0.15321
83.0864	2.05	domics Pos	0.004674	0.60743	0.81522	-0.29474	0.032853	0.99441	0.74282	-0.42891	0.017011	0.28747	0.76339	-0.3895	0.12553	0.99996	0.82931	-0.27002	0.006049	0.46849	0.75903	-0.39778	0.056795	0.99959	0.76472	-0.387	0.076812	0.26508	1.2111	0.2763	0.077908	0.99984	0.72929	-0.45543	0.0352	0.99966	0.84812	-0.23766
384.2042	6.38	olomics Po	0.019235	0.86976	0.7996	-0.32266	0.45271	1	0.92595	-0.11099	0.07961	0.99882	0.82047	-0.28549	0.11983	0.99987	0.89096	-0.16656	0.43489	0.95986	0.92558	-0.11157	0.22428	0.99796	0.83288	-0.26382	0.95277	0.99768	0.99266	-0.01063	0.019258	0.9981	0.79576	-0.3296	0.50734	0.99995	0.9425	-0.08544
310.3116	7.02	olomics Po	0.000977	0.29623	0.81587	-0.29359	0.039732	0.88953	0.71172	-0.49062	0.00023	0.35157	0.77024	-0.37662	0.080822	0.99987	0.86097	-0.21597	0.002707	0.95986	0.83372	-0.26237	0.066358	0.99796	0.73537	-0.44347	0.12395	0.94474	1.0597	0.083643	0.080556	0.9981	0.7396	-0.43518	0.005899	0.99388	0.81553	-0.2942
226.2167	2.76	domics Pos	0.23969	0.99995	0.90443	-0.14493	0.051463	0.99441	0.69208	-0.53099	0.002165	0.13479	0.72921	-0.45559	0.30104	0.99996	0.84697	-0.23962	0.012195	0.48469	0.79808	-0.3254	0.070671	0.99959	0.70815	-0.49788	0.10809	0.30988	1.2756	0.35118	0.12779	0.99984	0.76105	-0.39394	0.016169	0.99966	0.82487	-0.27777
161.1335	6	olomics Po	0.26096	0.99964	0.92652	-0.11011	0.073536	0.99372	0.81847	-0.28901	0.007019	0.51418	0.87703	-0.1893	0.80585	0.99987	1.0432	0.060993	0.30342	0.95986	0.95123	-0.07213	0.15335	0.99796	0.80554	-0.31197	0.56864	0.99768	1.0175	0.025059	0.10245	0.9981	0.82437	-0.27863	0.051693	0.99995	0.87525	-0.19223
133.1016	1.67	domics Pos	0.16306	0.99995	0.88724	-0.1726	0.053753	0.99441	0.72678	-0.4604	0.009397	0.21077	0.72526	-0.46343	0.39176	0.99996	0.89738	-0.1562	0.049862	0.70432	0.77803	-0.3621	0.15892	0.99959	0.77596	-0.36594	0.17074	0.3825	1.2216	0.28875	0.23232	0.99984	0.82067	-0.28512	0.24092	0.99966	0.86912	-0.20237
257.2673	1.91	domics Pos	0.007296	0.68962	0.84238	-0.24746	0.057133	0.99653	0.75298	-0.40932	0.001353	0.13107	0.74871	-0.41753	0.28775	0.99996	0.88356	-0.17859	0.00947	0.46849	0.81437	-0.29624	0.18429	0.99959	0.80329	-0.31601	0.064259	0.2485	1.2124	0.27789	0.22244	0.99984	0.81382	-0.29721	0.13596	0.99966	0.90099	-0.15042
785.9561	3.27	olomics Po	0.8415	0.99964	0.97883	-0.03087	0.43472	1	0.8857	-0.17511	0.045949	0.98463	0.70797	-0.49824	0.95536	0.99987	1.0589	0.082575	0.18487	0.95986	0.73463	-0.44492	0.89862	0.99796	0.97831	-0.03164	0.66557	0.99768	1.259	0.33231	0.59258	0.9981	0.91324	-0.13094	0.97188	0.99995	1.0604	0.084648
258.2707	6.2	olomics Po	0.004613	0.46876	0.81633	-0.29278	0.075742	0.99372	0.73635	-0.44155	0.002121	0.39871	0.8023	-0.31778	0.37759	0.99987	0.91985	-0.12053	0.093232	0.95986	0.87765	-0.18829	0.086789	0.99796	0.77549	-0.36681	0.19481	0.99768	1.058	0.081402	0.062769	0.9981	0.75401	-0.40734	0.019971	0.99995	0.82386	-0.27954
311.3148	7.02	olomics Po	0.001536	0.29623	0.82002	-0.28627	0.042825	0.88953	0.71139	-0.49128	0.000287	0.35157	0.76683	-0.38303	0.095773	0.99987	0.87031	-0.2004	0.003386	0.95986	0.84003	-0.25148	0.076038	0.99796	0.73842	-0.43749	0.12827	0.94474	1.0693	0.096618	0.088197	0.9981	0.74495	-0.42478	0.013845	0.99995	0.82852	-0.27139
109.1019	2.05	domics Pos	0.011353	0.68962	0.80703	-0.30931	0.067816	0.99979	0.73465	-0.44486	0.006064	0.17872	0.73397	-0.4462	0.32485	0.99996	0.8911	-0.16634	0.025691	0.5516	0.78177	-0.35519	0.1268	0.99959	0.76439	-0.38762	0.15836	0.36728	1.1578	0.21135	0.14932	0.99984	0.76596	-0.38465	0.033974	0.99966	0.83213	-0.26512
228.2326	1.34	domics Pos	0.005407	0.66407	0.88846	-0.17061	0.077274	0.99979	0.78525	-0.34877	0.000722	0.13107	0.76152	-0.39305	0.37191	0.99996	0.90505	-0.14392	0.016628	0.49216	0.82246	-0.28198	0.17257	0.99959	0.80078	-0.32052	0.075473	0.26319	1.2533	0.32578	0.26205	0.99984	0.84752	-0.23869	0.16756	0.99966	0.91931	-0.12138
294.2882	7.02	olomics Po	0.001519	0.29623	0.76673	-0.38322	0.029468	0.88953	0.69558	-0.52372	0.000911	0.35157	0.71933	-0.47527	0.15906	0.99987	0.8612	-0.21559	0.005195	0.95986	0.82404	-0.27921	0.079294	0.99796	0.75316	-0.40897	0.95009	0.99768	0.99957	-0.00062	0.065378	0.9981	0.70684	-0.50054	0.002787	0.7981	0.81951	-0.28717
637.475	2.05	domics Pos	0.50987	0.99995	0.92992	-0.10482	0.005587	0.99441	0.63274	-0.66032	0.10129	0.68915	0.77689	-0.36421	0.11239	0.99996	0.75973	-0.39644	0.072565	0.7883	0.7749	-0.36793	0.076781	0.99959	0.68353	-0.54893	0.22079	0.44007	1.2468	0.31828	0.10205	0.99984	0.68424	-0.54742	0.12714	0.99966	0.84828	-0.23739
368.2439	2.68	domics Pos	0.79814	0.99995	0.97804	-0.03203	0.19247	0.99979	0.87045	-0.20017	0.26903	0.88508	0.9145	-0.12894	0.5385	0.99996	0.92592	-0.11103	0.32181	0.99701	0.88849	-0.17057	0.020052	0.99959	0.72866	-0.45669	0.084074	0.27517	1.1905	0.25158	0.39452	0.99984	0.90419	-0.1453	0.24007	0.99966	0.90817	-0.13897
301.2534	2.06	domics Pos	0.010757	0.68962	0.85348	-0.22857	0.042538	0.99441	0.72452	-0.46491	0.010208	0.21867	0.76434	-0.3877	0.28662	0.99996	0.87611	-0.19082	0.060385	0.76594	0.8308	-0.26743	0.14846	0.99959	0.77549	-0.36683	0.068997	0.25493	1.2288	0.2973	0.16849	0.99984	0.79211	-0.33623	0.067263	0.99966	0.86613	-0.20734
470.9714	5.34	olomics Ne	0.77437	0.9951	1.0179	0.025661	0.56209	0.99975	1.0471	0.066416	0.18711	0.99731	0.92989	-0.10487	0.63382	0.99915	1.0276	0.039235	0.42271	0.78132	1.0507	0.071344	0.24315	0.64922	1.0903	0.12468	0.025625	0.086424	1.2176	0.28405	0.50894	0.99481	1.0479	0.067508	0.58307	0.96789	0.96658	-0.04904
332.2928	2.75	domics Pos	0.28816	0.99995	0.89282	-0.16356	0.051444	0.99441	0.66935	-0.57918	0.008933	0.2077	0.71079	-0.4925	0.40933	0.99996	0.86986	-0.20114	0.083741	0.80836	0.75517	-0.40513	0.050944	0.99959	0.65545	-0.60944	0.55603	0.74443	1.108	0.14798	0.1024	0.99984	0.73928	-0.4358	0.009432	0.99749	0.73	-0.45403
170.1535	2.76	domics Pos	0.013413	0.68962	0.82205	-0.2827	0.009215	0.99441	0.61968	-0.6904	0.000848	0.13107	0.67017	-0.57741	0.034159	0.99996	0.74867	-0.41759	0.00387	0.46849	0.64518	-0.63223	0.048815	0.99959	0.64548	-0.63155	0.45994	0.67219	1.0981	0.13497	0.16779	0.99984	0.73511	-0.44398	0.001078	0.99749	0.70796	-0.49826
240.2327	2.05	domics Pos	0.01813	0.74245	0.85172	-0.23155	0.043717	0.99441	0.75811	-0.39953	0.001375	0.13107	0.75656	-0.40247	0.2641	0.99996	0.86242	-0.21353	0.0113	0.47875	0.80747	-0.30852	0.16849	0.99959	0.81193	-0.30057	0.083162	0.27417	1.2253	0.29316	0.2237	0.99984	0.82009	-0.28615	0.11686	0.99966	0.88874	-0.17017
366.2321	2.06	domics Pos	0.10208	0.99995	0.91278	-0.13165	0.46769	0.99979	0.93264	-0.10061	0.025026	0.34014	0.83507	-0.26002	0.77317	0.99996	0.98241	-0.0256	0.031197	0.59375	0.88546	-0.1755	0.29685	0.99959	0.90044	-0.1513	0.028164	0.19668	1.2539	0.32645	0.1622	0.99984	0.81484	-0.29542	0.94818	0.99966	1.0094	0.013505
202.6521	6.9	olomics Po	0.096919	0.99964	0.84229	-0.24761	0.027322	0.88953	0.65653	-0.60707	0.012812	0.60269	0.73558	-0.44305	0.22139	0.99987	0.88011	-0.18424	0.0193	0.95986	0.83014	-0.26858	0.12557	0.99796	0.77234	-0.3727	0.25205	0.99768	0.95649	-0.06418	0.036602	0.9981	0.74285	-0.42886	0.063864	0.99995	0.88481	-0.17656
212.2013	7.02	olomics Po	0.000202	0.21218	0.7793	-0.35976	0.023321	0.88953	0.66516	-0.58822	0.000413	0.35157	0.72876	-0.45649	0.21825	0.99987	0.86045	-0.21684	0.030581	0.95986	0.8288	-0.2709	0.013982	0.99796	0.68887	-0.5377	0.44633	0.99768	1.064	0.089521	0.064602	0.9981	0.68238	-0.55135	0.008136	0.99995	0.81711	-0.2914
760.8226	10.05	domics Pos	0.94687	0.99995	0.99858	-0.00205	0.10505	0.99979	1.0941	0.12975	0.076242	0.60691	0.95113	-0.07229	0.42759	0.99996	1.0782	0.1086	0.67998	0.99701	0.98308	-0.02461	0.88975	0.99959	1.0167	0.023906	0.018807	0.17894	1.2688	0.34342	0.52691	0.99984	1.0519	0.073061	0.084226	0.99966	1.1225	0.16669
550.9771	5.25	olomics Ne	0.82107	0.9951	1.0128	0.018313	0.25029	0.99975	1.0517	0.072737	0.58478	0.99731	0.97654	-0.03425	0.2217	0.99915	1.0814	0.1129	0.11247	0.61361	1.0849	0.11756	0.077099	0.5223	1.0969	0.13341	0.001649	0.041313	1.2332	0.30245	0.98896	0.99785	0.99871	-0.00186	0.55376	0.96586	0.96803	-0.04688
1118.743	10.07	domics Pos	0.41139	0.99995	1.1253	0.17035	0.70623	0.99979	1.0385	0.05454	0.018624	0.30022	1.1777	0.23601	0.98003	0.99996	1.0105	0.015071	0.11241	0.85684	1.1258	0.17089	0.067689	0.99959	1.2075	0.27202	0.017281	0.17568	1.3813	0.46601	0.073507	0.99984	1.1067	0.14632	0.95779	0.99966	1.0152	0.021803
297.2972	2.41	domics Pos	0.14608	0.99995	0.8402	-0.25119	0.085267	0.99979	0.74827	-0.41837	0.040256	0.43361	0.73238	-0.44934	0.14909	0.99996	0.81322	-0.29828	0.024508	0.5516	0.72176	-0.47041	0.14484	0.99959	0.77897	-0.36036	0.61692	0.77895	1.1736	0.231	0.18418	0.99984	0.70468	-0.50497	0.031604	0.99966	0.76423	-0.38791
242.2481	1.58	domics Pos	0.11626	0.99995	0.8613	-0.21541	0.14323	0.99979	0.81244	-0.29967	0.002155	0.13479	0.73699	-0.44029	0.37501	0.99996	0.88959	-0.16879	0.009652	0.46849	0.77984	-0.35874	0.12576	0.99959	0.78542	-0.34847	0.15407	0.36252	1.1832	0.24274	0.25764	0.99984	0.82893	-0.27068	0.16627	0.99966	0.88964	-0.1687
149.1331	2.05	domics Pos	0.007288	0.68962	0.84444	-0.24394	0.039397	0.99441	0.75254	-0.41016	0.006618	0.18153	0.75482	-0.4058	0.26266	0.99996	0.87928	-0.18561	0.009335	0.46849	0.79972	-0.32243	0.13694	0.99959	0.78127	-0.35611	0.12872	0.33008	1.1835	0.24307	0.17685	0.99984	0.79527	-0.33047	0.048226	0.99966	0.87401	-0.19428
202.6475	2.07	domics Pos	0.23415	0.99995	0.89965	-0.15256	0.4634	0.99979	0.90773	-0.13967	0.20986	0.83096	0.82888	-0.27077	0.49936	0.99996	0.92857	-0.10692	0.073697	0.7908	0.84173	-0.24857	0.031063	0.99959	0.78632	-0.34682	0.15029	0.35839	1.1376	0.18594	0.1944	0.99984	0.83621	-0.25807	0.66506	0.99966	0.96	-0.0589
171.1561	1.66	domics Pos	0.29626	0.99995	0.94092	-0.08785	0.005062	0.99441	0.7842	-0.3507	0.006281	0.17872	0.73343	-0.44726	0.37407	0.99996	0.88613	-0.17441	0.027883	0.55491	0.80561	-0.31184	0.22691	0.99959	0.81985	-0.28656	0.14214	0.34996	1.2114	0.2767	0.3876	0.99984	0.8865	-0.17381	0.024776	0.99966	0.83907	-0.25314
763.9427	3.25	olomics Po	0.71266	0.99964	0.96177	-0.05624	0.03743	0.88953	0.73624	-0.44175	0.028514	0.79314	0.68126	-0.55372	0.50717	0.99987	0.90743	-0.14014	0.14132	0.95986	0.71115	-0.49177	0.40633	0.99796	0.86686	-0.20613	0.8431	0.99768	1.2045	0.26841	0.32641	0.9981	0.86227	-0.21378	0.58643	0.99995	0.92863	-0.10682
142.1233	1.66	domics Pos	0.006499	0.68962	0.86754	-0.205	0.021827	0.99441	0.7583	-0.39915	0.006401	0.17975	0.74155	-0.43139	0.26597	0.99996	0.85003	-0.23442	0.032286	0.59375	0.80599	-0.31117	0.10253	0.99959	0.77004	-0.377	0.077985	0.26717	1.209	0.27386	0.24975	0.99984	0.82159	-0.28351	0.047304	0.99966	0.88773	-0.17181
147.118	6	olomics Po	0.13623	0.99964	0.8771	-0.18919	0.15339	1	0.761	-0.39404	0.074613	0.99882	0.83617	-0.25814	0.46943	0.99987	0.91438	-0.12914	0.28561	0.95986	0.895	-0.16004	0.049333	0.99796	0.75227	-0.41069	0.99366	0.99932	0.99767	-0.00336	0.05114	0.9981	0.78375	-0.35153	0.11946	0.99995	0.84874	-0.2366
256.2648	6.2	olomics Po	0.003256	0.38271	0.80273	-0.31701	0.046577	0.88953	0.75718	-0.40129	0.002764	0.41947	0.79941	-0.323	0.43819	0.99987	0.94381	-0.08344	0.023235	0.95986	0.84869	-0.23669	0.096921	0.99796	0.80662	-0.31003	0.31731	0.99768	1.048	0.067647	0.074096	0.9981	0.76352	-0.38926	0.020399	0.99995	0.83858	-0.25398
589.3124	0.56	domics Pos	0.79861	0.99995	1.0832	0.11525	0.13771	0.99979	0.80523	-0.31253	0.04334	0.45105	0.7664	-0.38384	0.6841	0.99996	1.0736	0.10251	0.051392	0.70556	0.65638	-0.60741	0.25999	0.99959	0.83887	-0.25348	0.78813	0.87825	1.337	0.41897	0.7802	0.99984	0.98127	-0.02727	0.81038	0.99966	1.0155	0.022138
258.2703	1.91	domics Pos	0.02421	0.85085	0.84926	-0.23573	0.069431	0.99979	0.74668	-0.42145	0.001828	0.13302	0.7226	-0.46874	0.52553	0.99996	0.92788	-0.10799	0.021368	0.53763	0.80777	-0.30798	0.18179	0.99959	0.7945	-0.33188	0.078251	0.26784	1.2131	0.27869	0.14046	0.99984	0.7643	-0.38779	0.19893	0.99966	0.90793	-0.13935
381.2035	2.05	domics Pos	0.043454	0.99995	0.79525	-0.33052	0.14514	0.99979	0.80081	-0.32047	0.33679	0.92252	0.87855	-0.18681	0.199	0.99996	0.80794	-0.30768	0.017824	0.49608	0.74828	-0.41834	0.018592	0.99959	0.73417	-0.44581	0.9164	0.95497	1.0185	0.026424	0.047699	0.99984	0.68675	-0.54214	0.080853	0.99966	0.80949	-0.30491
1048.751	10.07	domics Pos	0.49119	0.99995	0.96053	-0.05809	0.31326	0.99979	1.0787	0.10934	0.074669	0.59936	0.92546	-0.11175	0.41021	0.99996	1.0567	0.079502	0.5762	0.99701	1.0726	0.10116	0.38524	0.99959	1.039	0.055194	0.019053	0.17894	1.3071	0.38637	0.58613	0.99984	1.0906	0.12515	0.083719	0.99966	1.1183	0.1613
181.1304	2.07	domics Pos	0.085966	0.99995	0.91367	-0.13026	0.28406	0.99979	0.91234	-0.13236	0.014863	0.26986	0.87505	-0.19257	0.80914	0.99996	0.98803	-0.01737	0.095861	0.82209	0.90715	-0.14059	0.36141	0.99959	0.91863	-0.12245	0.023778	0.18728	1.2211	0.28812	0.19068	0.99984	0.86049	-0.21676	0.47192	0.99966	0.96008	-0.05877
142.124	7.02	olomics Po	0.005035	0.49147	0.7767	-0.36457	0.019412	0.88953	0.7381	-0.43811	0.001666	0.39871	0.75683	-0.40196	0.4442	0.99987	0.92261	-0.1162	0.050128	0.95986	0.77349	-0.37055	0.085472	0.99796	0.73619	-0.44185	0.77025	0.99768	1.0458	0.064592	0.019186	0.9981	0.67748	-0.56174	0.00631	0.99388	0.80891	-0.30595
265.2532	2.05	domics Pos	0.002297	0.59581	0.84982	-0.23477	0.051876	0.99441	0.76902	-0.3789	0.002277	0.13479	0.76274	-0.39074	0.32464	0.99996	0.89376	-0.16204	0.00684	0.46849	0.80734	-0.30876	0.11567	0.99959	0.79051	-0.33915	0.096892	0.29321	1.1913	0.25248	0.15543	0.99984	0.79227	-0.33594	0.057427	0.99966	0.88763	-0.17197
312.3178	7.02	olomics Po	0.029999	0.98592	0.84245	-0.24733	0.086454	1	0.72275	-0.46842	0.004806	0.48835	0.81201	-0.30044	0.19283	0.99987	0.88413	-0.17768	0.048636	0.95986	0.87969	-0.18494	0.086428	0.99796	0.74021	-0.434	0.14357	0.95284	1.06	0.08407	0.072022	0.9981	0.74561	-0.4235	0.016129	0.99995	0.81672	-0.29208
285.2889	2.05	domics Pos	0.036523	0.95608	0.8001	-0.32175	0.14778	0.99979	0.8254	-0.27684	0.003651	0.15208	0.71971	-0.47451	0.70959	0.99996	0.96609	-0.04977	0.044316	0.66526	0.82263	-0.28168	0.27166	0.99959	0.84438	-0.24403	0.11715	0.32013	1.2423	0.31305	0.24534	0.99984	0.81368	-0.29746	0.38458	0.99966	0.92644	-0.11023
83.0864	2.76	domics Pos	0.57567	0.99995	0.93201	-0.10158	0.024754	0.99441	0.69835	-0.51799	0.24399	0.85893	0.84128	-0.24933	0.98816	0.99996	1.0692	0.0965	0.28647	0.99701	0.87314	-0.19571	0.040477	0.99959	0.65536	-0.60965	0.83051	0.90194	1.0906	0.12507	0.22419	0.99984	0.85562	-0.22495	0.32546	0.99966	0.90243	-0.14811
139.1124	2.05	domics Pos	0.004779	0.60743	0.86822	-0.20387	0.018327	0.99441	0.71176	-0.49053	0.001581	0.13302	0.76733	-0.38209	0.28674	0.99996	0.88401	-0.17787	0.003737	0.46849	0.77108	-0.37505	0.11252	0.99959	0.76811	-0.38061	0.20712	0.42281	1.1733	0.23058	0.13423	0.99984	0.7804	-0.35772	0.024917	0.99966	0.84574	-0.24172
143.1255	1.66	domics Pos	0.039084	0.96211	0.76528	-0.38593	0.062771	0.99979	0.70451	-0.50531	0.058561	0.53292	0.74047	-0.43349	0.21901	0.99996	0.81181	-0.30079	0.013787	0.4899	0.68289	-0.55027	0.16224	0.99959	0.7314	-0.45127	0.33373	0.55658	1.1482	0.19937	0.21313	0.99984	0.76097	-0.39409	0.18078	0.99966	0.85672	-0.22311
198.1863	5.99	olomics Po	0.007693	0.60235	0.80133	-0.31952	0.12004	1	0.81276	-0.29909	0.004146	0.46641	0.79364	-0.33344	0.47646	0.99987	0.94344	-0.084	0.013401	0.95986	0.8492	-0.23582	0.10128	0.99796	0.7942	-0.33243	0.38507	0.99768	1.0346	0.049033	0.073672	0.9981	0.78696	-0.34563	0.019876	0.99995	0.85107	-0.23265
69.0706	2.05	domics Pos	0.013556	0.68962	0.82037	-0.28565	0.095984	0.99979	0.77246	-0.37246	0.000646	0.13107	0.73743	-0.43941	0.3352	0.99996	0.86913	-0.20235	0.00934	0.46849	0.75659	-0.40242	0.11773	0.99959	0.78121	-0.35621	0.26061	0.47751	1.1415	0.19093	0.11733	0.99984	0.73817	-0.43798	0.036423	0.99966	0.83604	-0.25835
270.2796	2.27	domics Pos	0.18291	0.99995	0.85376	-0.2281	0.17127	0.99979	0.78479	-0.34962	0.020094	0.30717	0.70587	-0.50253	0.34566	0.99996	0.86136	-0.21532	0.058712	0.76299	0.77654	-0.36487	0.13085	0.99959	0.73058	-0.45288	0.17846	0.39041	1.2131	0.27873	0.15122	0.99984	0.72029	-0.47334	0.40497	0.99966	0.90516	-0.14376
343.9833	4.35	olomics Ne	0.4143	0.9951	1.0562	0.078948	0.15764	0.99975	1.1145	0.1564	0.75334	0.99731	1.0182	0.025979	0.10057	0.99915	1.0974	0.13408	0.074674	0.5958	1.1371	0.18535	0.007118	0.45618	1.1963	0.25855	0.004859	0.051563	1.3099	0.38948	0.097675	0.99481	1.1202	0.1637	0.57192	0.96658	1.0396	0.05601
348.2372	1.66	domics Pos	0.66293	0.99995	0.97048	-0.04324	0.28459	0.99979	0.88068	-0.18332	0.021722	0.3198	0.77725	-0.36356	0.40571	0.99996	0.896	-0.15842	0.34261	0.99701	0.92038	-0.1197	0.24531	0.99959	0.87617	-0.19071	0.022891	0.18641	1.2074	0.27187	0.32504	0.99984	0.87872	-0.18652	0.042987	0.99966	0.835	-0.26016
240.2332	6.38	olomics Po	0.020424	0.86976	0.87895	-0.18615	0.072605	0.99372	0.782	-0.35476	0.050088	0.99882	0.86434	-0.21032	0.68157	0.99987	0.97006	-0.04385	0.32486	0.95986	0.95074	-0.07288	0.13018	0.99796	0.81986	-0.28655	0.033668	0.65873	1.116	0.1583	0.14283	0.9981	0.81976	-0.28673	0.11061	0.99995	0.88252	-0.1803
466.9757	5.15	olomics Ne	0.62706	0.9951	1.0268	0.038197	0.39041	0.99975	1.0495	0.069656	0.56313	0.99731	0.97164	-0.04151	0.38491	0.99915	1.06	0.084005	0.18013	0.67053	1.0711	0.099141	0.095723	0.52866	1.1282	0.17396	0.007352	0.057477	1.2315	0.30036	0.64603	0.99481	1.0284	0.040437	0.57284	0.96658	0.97837	-0.03155
268.264	1.72	domics Pos	0.007641	0.68962	0.82937	-0.26992	0.043091	0.99441	0.70987	-0.49437	0.009938	0.2165	0.75192	-0.41135	0.19927	0.99996	0.84108	-0.24968	0.009406	0.46849	0.80978	-0.3044	0.13131	0.99959	0.76883	-0.37926	0.15695	0.36581	1.1144	0.1563	0.13983	0.99984	0.78385	-0.35135	0.036544	0.99966	0.83158	-0.26607
247.2426	2.05	domics Pos	0.003068	0.59581	0.85668	-0.22317	0.05553	0.99504	0.77835	-0.36151	0.002958	0.15208	0.76905	-0.37885	0.36833	0.99996	0.90513	-0.1438	0.008615	0.46849	0.81349	-0.2978	0.13118	0.99959	0.79609	-0.329	0.10316	0.30197	1.1958	0.25802	0.1761	0.99984	0.80189	-0.31852	0.074509	0.99966	0.89993	-0.15212
167.1436	1.66	domics Pos	0.089591	0.99995	0.86113	-0.21571	0.13227	0.99979	0.80577	-0.31156	0.012484	0.24164	0.7532	-0.40889	0.46293	0.99996	0.9112	-0.13416	0.010249	0.46849	0.78131	-0.35604	0.20784	0.99959	0.82543	-0.27678	0.077379	0.26581	1.2526	0.3249	0.19809	0.99984	0.80408	-0.31459	0.22537	0.99966	0.89928	-0.15316
123.1175	2.05	domics Pos	0.012407	0.68962	0.84204	-0.24803	0.061168	0.99979	0.76504	-0.3864	0.007617	0.19873	0.75561	-0.40428	0.31853	0.99996	0.87943	-0.18536	0.017005	0.49216	0.79538	-0.33028	0.093174	0.99959	0.76751	-0.38175	0.17251	0.38396	1.2048	0.26877	0.18665	0.99984	0.79929	-0.3232	0.073335	0.99966	0.8795	-0.18524
198.185	7.02	olomics Po	0.19509	0.99964	0.87992	-0.18455	0.043937	0.88953	0.69186	-0.53145	0.029038	0.79538	0.77916	-0.36	0.30429	0.99987	0.8889	-0.1699	0.17192	0.95986	0.86155	-0.215	0.16584	0.99796	0.76047	-0.39504	0.068821	0.82218	1.2044	0.26832	0.11742	0.9981	0.73489	-0.4444	0.40465	0.99995	0.89849	-0.15443
128.1077	2.05	domics Pos	0.003594	0.59581	0.85752	-0.22176	0.034555	0.99441	0.74819	-0.41853	0.00368	0.15208	0.76783	-0.38114	0.28677	0.99996	0.88448	-0.1771	0.003907	0.46849	0.81036	-0.30336	0.12387	0.99959	0.79446	-0.33195	0.10672	0.30711	1.2017	0.2651	0.1947	0.99984	0.80422	-0.31433	0.072437	0.99966	0.87574	-0.19143
248.2459	2.05	domics Pos	0.016888	0.72353	0.86358	-0.2116	0.063702	0.99979	0.78056	-0.35743	0.008795	0.20702	0.77431	-0.36902	0.28008	0.99996	0.87922	-0.18571	0.024022	0.5516	0.80971	-0.30452	0.14485	0.99959	0.80097	-0.32018	0.11659	0.31965	1.2131	0.27869	0.18263	0.99984	0.80381	-0.31508	0.085688	0.99966	0.89261	-0.1639
261.2218	1.4	domics Pos	0.080272	0.99995	0.85974	-0.21802	0.022467	0.99441	0.75653	-0.40252	0.005779	0.17488	0.71631	-0.48135	0.43362	0.99996	0.90224	-0.14841	0.006339	0.46849	0.75705	-0.40154	0.22171	0.99959	0.81744	-0.29081	0.13728	0.34347	1.1458	0.19637	0.20161	0.99984	0.8063	-0.31061	0.19914	0.99966	0.89544	-0.15933
166.1672	2.05	domics Pos	0.027585	0.86927	0.84694	-0.23967	0.095541	0.99979	0.75508	-0.40531	0.022493	0.32122	0.80287	-0.31676	0.16188	0.99996	0.82105	-0.28446	0.57324	0.99701	0.93857	-0.09147	0.13617	0.99959	0.75575	-0.40402	0.18501	0.39743	1.2187	0.28535	0.16981	0.99984	0.76621	-0.38418	0.035351	0.99966	0.8201	-0.28612
212.2014	2.05	domics Pos	0.013311	0.68962	0.85451	-0.22683	0.059775	0.99979	0.76985	-0.37735	0.003127	0.15208	0.7585	-0.39878	0.31744	0.99996	0.89251	-0.16405	0.017587	0.49574	0.81264	-0.29931	0.15323	0.99959	0.79553	-0.33001	0.12016	0.32219	1.2041	0.268	0.19828	0.99984	0.80566	-0.31176	0.10641	0.99966	0.90296	-0.14726
664.7482	9.92	domics Pos	0.49603	0.99995	0.93935	-0.09027	0.75643	0.99979	1.0215	0.030727	0.86744	0.99702	1.0132	0.018857	0.068028	0.99996	1.1888	0.24948	0.12473	0.86372	1.1324	0.17938	0.20194	0.99959	1.122	0.16604	0.033616	0.20196	1.3079	0.38723	0.86588	0.99984	1.0118	0.016863	0.051503	0.99966	1.2239	0.29146
136.1208	1.66	domics Pos	0.36274	0.99995	0.94694	-0.07865	0.39596	0.99979	0.90358	-0.14627	0.038252	0.424	0.83162	-0.266	0.66021	0.99996	0.96328	-0.05398	0.000488	0.46849	0.78077	-0.35703	0.16381	0.99959	0.82823	-0.2719	0.051918	0.23013	1.335	0.41684	0.90408	0.99984	1.0032	0.004629	0.2758	0.99966	1.0297	0.042286
155.144	2.05	domics Pos	0.071563	0.99995	0.90922	-0.1373	0.01263	0.99441	0.72789	-0.45821	0.003977	0.15412	0.74987	-0.41528	0.50698	0.99996	0.94108	-0.08762	0.060811	0.76594	0.83736	-0.25607	0.18519	0.99959	0.81345	-0.29787	0.11585	0.31895	1.2604	0.33386	0.38426	0.99984	0.86684	-0.20616	0.031295	0.99966	0.85353	-0.22848
226.2154	7.02	olomics Po	0.082696	0.99964	0.8954	-0.1594	0.043938	0.88953	0.69219	-0.53076	0.002797	0.41947	0.80056	-0.32092	0.25708	0.99987	0.87722	-0.18899	0.14954	0.95986	0.86756	-0.20497	0.056876	0.99796	0.70648	-0.50127	0.80715	0.99768	1.0147	0.020998	0.04166	0.9981	0.68849	-0.53849	0.03751	0.99995	0.80948	-0.30494
279.9733	2.64	olomics Po	0.65852	0.99964	0.95498	-0.06645	0.028164	0.88953	1.365	0.44891	0.94643	0.99882	1.0014	0.001949	0.009269	0.99987	1.2746	0.35008	0.6709	0.9703	1.107	0.14672	0.47723	0.99796	1.0933	0.12869	0.71926	0.99768	0.96792	-0.04704	0.086113	0.9981	1.2485	0.3202	0.44477	0.99995	1.072	0.10028
266.2565	2.05	domics Pos	0.007263	0.68962	0.8572	-0.22229	0.071545	0.99979	0.77075	-0.37567	0.001779	0.13302	0.76662	-0.38341	0.30401	0.99996	0.88826	-0.17094	0.014785	0.4899	0.81345	-0.29788	0.12217	0.99959	0.7919	-0.33662	0.11561	0.3189	1.1953	0.25736	0.17108	0.99984	0.80016	-0.32164	0.045105	0.99966	0.88795	-0.17144
582.5255	2.05	domics Pos	0.013121	0.68962	0.72886	-0.45629	0.036108	0.99441	0.56479	-0.82422	0.002018	0.13479	0.58124	-0.7828	0.16654	0.99996	0.76338	-0.38952	0.020565	0.53763	0.69464	-0.52567	0.082428	0.99959	0.60552	-0.72375	0.41953	0.63834	1.1665	0.22222	0.086459	0.99984	0.62569	-0.67647	0.009898	0.99749	0.70535	-0.50358
183.1705	2.05	domics Pos	0.017551	0.73542	0.78685	-0.34584	0.010543	0.99441	0.66528	-0.58798	0.011645	0.2337	0.703	-0.5084	0.19223	0.99996	0.81278	-0.29906	0.043555	0.66031	0.74978	-0.41545	0.30115	0.99959	0.80474	-0.3134	0.10638	0.30683	1.224	0.29155	0.22546	0.99984	0.78643	-0.3466	0.002105	0.99749	0.72115	-0.47162

170.1545	1.66	domics Pos	0.032127	0.91043	0.85906	-0.21917	0.027199	0.99441	0.74713	-0.42058	0.004183	0.15793	0.7619	-0.39232	0.30171	0.99996	0.87723	-0.18898	0.016356	0.49216	0.81349	-0.2978	0.094487	0.99959	0.77472	-0.36825	0.14188	0.3499	1.1747	0.23231	0.24316	0.99984	0.83164	-0.26596	0.073266	0.99966	0.9043	-0.14513
164.1518	1.66	domics Pos	0.022111	0.8137	0.81784	-0.29011	0.063291	0.99979	0.72485	-0.46424	0.00584	0.17535	0.80605	-0.31106	0.47232	0.99996	0.90655	-0.14154	0.036506	0.62141	0.80746	-0.30854	0.11846	0.99959	0.85774	-0.22138	0.050276	0.22687	1.27	0.34486	0.30666	0.99984	0.82511	-0.27734	0.67359	0.99966	0.96995	-0.04402
278.2483	1.4	domics Pos	0.024017	0.85085	0.82766	-0.27289	0.10989	0.99979	0.82514	-0.27729	0.005222	0.16899	0.70835	-0.49746	0.4677	0.99996	0.91621	-0.12624	0.026184	0.5516	0.81187	-0.30067	0.099351	0.99959	0.79315	-0.33434	0.11101	0.3135	1.193	0.25463	0.29197	0.99984	0.83172	-0.26584	0.25048	0.99966	0.91936	-0.12129
257.2679	6.2	bolomics Pc	0.002121	0.33442	0.79874	-0.3242	0.042307	0.88953	0.7535	-0.40832	0.002396	0.39871	0.80296	-0.31661	0.30175	0.99987	0.92694	-0.10946	0.014933	0.95986	0.84302	-0.24637	0.09607	0.99796	0.8054	-0.31222	0.30226	0.99768	1.0491	0.069091	0.08109	0.9981	0.76338	-0.38953	0.017187	0.99995	0.83462	-0.26081
181.1592	1.66	domics Pos	0.036608	0.95608	0.8291	-0.27038	0.007417	0.99441	0.71408	-0.48585	0.015783	0.27907	0.76776	-0.38127	0.17324	0.99996	0.82789	-0.27249	0.041716	0.6519	0.80207	-0.31819	0.16524	0.99959	0.7868	-0.34593	0.07349	0.26156	1.241	0.31147	0.23302	0.99984	0.80622	-0.31075	0.28809	0.99966	0.91351	-0.1305
469.9798	4.96	olomics Ne	0.6799	0.9951	1.0289	0.041116	0.89594	0.99975	1.0098	0.014037	0.60804	0.99731	0.97551	-0.03577	0.45729	0.99915	1.0491	0.069122	0.15036	0.64314	1.0775	0.10767	0.032889	0.4612	1.116	0.15837	0.007701	0.057477	1.2571	0.33011	0.49461	0.99481	1.0447	0.063096	0.92075	0.99833	0.99608	-0.00567
184.1702	2.05	domics Pos	0.004442	0.60743	0.8483	-0.23735	0.070212	0.99979	0.78135	-0.35596	0.001724	0.13302	0.74816	-0.41858	0.38012	0.99996	0.90229	-0.14834	0.010637	0.46849	0.79642	-0.3284	0.14766	0.99959	0.79369	-0.33334	0.11109	0.3135	1.2035	0.26723	0.18191	0.99984	0.79142	-0.33748	0.076261	0.99966	0.88459	-0.17693
488.5993	3.21	bolomics Pc	0.5933	0.99964	0.93479	-0.09728	0.13021	1	0.84002	-0.2515	0.041999	0.93262	0.74783	-0.41922	0.97787	0.99987	1.0782	0.10862	0.11471	0.95986	0.67762	-0.56145	0.57703	0.99796	0.9298	-0.10501	0.65241	0.99768	1.3441	0.42662	0.8942	0.9981	1.0152	0.021764	0.62749	0.99995	0.94671	-0.07901
156.1389	1.66	domics Pos	0.025069	0.85508	0.87159	-0.19828	0.03426	0.99441	0.75292	-0.40942	0.003399	0.15208	0.73373	-0.44668	0.30152	0.99996	0.87242	-0.19691	0.026439	0.55306	0.8068	-0.30972	0.15358	0.99959	0.79537	-0.33029	0.067467	0.25306	1.1833	0.24281	0.19814	0.99984	0.80321	-0.31614	0.09938	0.99966	0.88407	-0.17777
142.1234	2.05	domics Pos	0.003555	0.59581	0.86166	-0.21481	0.046245	0.99441	0.76988	-0.37729	0.002951	0.15208	0.78165	-0.3554	0.38294	0.99996	0.90388	-0.14579	0.009715	0.46849	0.82278	-0.28142	0.12246	0.99959	0.78855	-0.34273	0.14195	0.3499	1.1749	0.23258	0.14202	0.99984	0.7965	-0.32826	0.052097	0.99966	0.88623	-0.17425
226.217	2.05	domics Pos	0.003464	0.59581	0.84496	-0.24305	0.061456	0.99979	0.77321	-0.37107	0.00259	0.14521	0.76167	-0.39276	0.32807	0.99996	0.89549	-0.15925	0.007728	0.46849	0.79589	-0.32936	0.074239	0.99959	0.74524	-0.42422	0.12663	0.32691	1.1957	0.25781	0.1483	0.99984	0.78746	-0.34472	0.013655	0.99749	0.86904	-0.2025
198.1858	1.66	domics Pos	0.015839	0.68962	0.86945	-0.20182	0.031183	0.99441	0.76062	-0.39476	0.001306	0.13107	0.74267	-0.42921	0.31933	0.99996	0.88586	-0.17484	0.008234	0.46849	0.82167	-0.28336	0.15731	0.99959	0.7859	-0.34758	0.1486	0.35725	1.1669	0.22267	0.17615	0.99984	0.80076	-0.32055	0.036428	0.99966	0.87266	-0.1965
156.1377	2.76	domics Pos	0.14125	0.99995	0.84089	-0.25002	0.042048	0.99441	0.67116	-0.57526	0.009772	0.21537	0.75182	-0.41155	0.20307	0.99996	0.81334	-0.29808	0.034863	0.61544	0.72859	-0.45681	0.027534	0.99959	0.66137	-0.59648	0.6949	0.82108	1.0696	0.097014	0.24414	0.99984	0.80311	-0.31633	0.012113	0.99749	0.77397	-0.36964
203.6416	2.06	domics Pos	0.23683	0.99995	0.91238	-0.1323	0.28646	0.99979	0.83839	-0.25431	0.031757	0.38441	0.86215	-0.21399	0.39662	0.99996	0.89317	-0.163	0.030548	0.58579	0.80344	-0.31574	0.2293	0.99959	0.86309	-0.21241	0.31735	0.53843	1.1858	0.24581	0.15665	0.99984	0.83483	-0.26044	0.80951	0.99966	1.0246	0.035059
340.2142	1.91	domics Pos	0.42286	0.99995	0.91714	-0.12478	0.33221	0.99979	0.89519	-0.15974	0.069523	0.58007	0.80472	-0.31344	0.80587	0.99996	0.97454	-0.03721	0.44801	0.99701	0.92049	-0.11953	0.42335	0.99959	0.87315	-0.19571	0.016321	0.17568	1.2979	0.37622	0.41484	0.99984	0.88232	-0.18063	0.44655	0.99966	0.90573	-0.14285
517.942	0.53	domics Pos	0.78107	0.99995	1.0761	0.10575	0.18233	0.99979	0.82784	-0.27257	0.024804	0.34014	0.72694	-0.46009	0.80417	0.99996	1.1152	0.15729	0.088361	0.81976	0.70033	-0.51389	0.4427	0.99959	0.88651	-0.17378	0.57852	0.75676	1.5288	0.61243	0.74999	0.99984	0.98049	-0.02842	0.93307	0.99966	1.0963	0.13258
262.2533	1.67	domics Pos	0.84835	0.99995	1.0275	0.039182	0.092242	0.99979	0.81935	-0.28746	0.18151	0.80578	0.86258	-0.21327	0.608	0.99996	0.94814	-0.07682	0.36561	0.99701	0.94341	-0.08404	0.38232	0.99959	0.88369	-0.17838	0.001437	0.089845	1.3814	0.4661	0.24576	0.99984	0.84821	-0.23751	0.008373	0.99749	0.81692	-0.29174
170.1551	6.38	bolomics Pc	0.002104	0.33442	0.86304	-0.2125	0.037823	0.88953	0.77218	-0.373	0.001988	0.39871	0.84395	-0.24476	0.54595	0.99987	0.95824	-0.06155	0.067746	0.95986	0.88228	-0.18068	0.093474	0.99796	0.80864	-0.30642	0.10428	0.90326	1.0739	0.10292	0.10328	0.9981	0.79369	-0.33334	0.034631	0.99995	0.86096	-0.21599
135.1175	1.67	domics Pos	0.025841	0.85508	0.89116	-0.16625	0.040642	0.99441	0.77657	-0.36481	0.013561	0.25347	0.81565	-0.29398	0.4238	0.99996	0.90936	-0.13708	0.01121	0.47875	0.79532	-0.33038	0.098778	0.99959	0.75983	-0.39625	0.17479	0.38636	1.1659	0.2214	0.10705	0.99984	0.79255	-0.33543	0.076061	0.99966	0.88811	-0.17118
338.1823	6.47	bolomics Pc	0.47695	0.99964	0.96364	-0.05343	0.60201	1	0.96845	-0.04625	0.37542	0.99882	0.94348	-0.08393	0.44945	0.99987	0.90974	-0.13648	0.24932	0.95986	1.0322	0.045693	0.31012	0.99796	0.86006	-0.21749	0.048113	0.76159	1.1608	0.21507	0.33543	0.9981	0.94017	-0.08901	0.14667	0.99995	0.91349	-0.13054
149.1331	1.66	domics Pos	0.012888	0.68962	0.87086	-0.19949	0.051966	0.99441	0.77888	-0.36053	0.001157	0.13107	0.73843	-0.43748	0.3062	0.99996	0.8765	-0.19018	0.022536	0.55084	0.80885	-0.30605	0.1484	0.99959	0.80332	-0.31595	0.13235	0.33553	1.1793	0.23791	0.14062	0.99984	0.78192	-0.35491	0.060119	0.99966	0.90504	-0.14394
705.3253	1.84	domics Pos	0.22813	0.99995	1.2177	0.28418	0.82578	0.99979	0.98964	-0.01502	0.5363	0.99601	0.93869	-0.09127	0.85285	0.99996	0.98723	-0.01855	0.62042	0.99701	1.0852	0.11793	0.34196	0.99959	1.1613	0.21568	0.04974	0.22586	1.4358	0.52187	0.2436	0.99984	0.90834	-0.13869	0.091339	0.99966	1.1671	0.22293
245.2269	1.66	domics Pos	0.013857	0.68962	0.87948	-0.18527	0.030624	0.99441	0.76615	-0.3843	0.001982	0.13479	0.76289	-0.39044	0.35747	0.99996	0.89274	-0.16369	0.002817	0.46849	0.80744	-0.30858	0.14175	0.99959	0.79186	-0.33669	0.071605	0.25937	1.2179	0.2844	0.21342	0.99984	0.82555	-0.27657	0.073139	0.99966	0.8928	-0.1636
165.991	10.2	domics Pos	0.13724	0.99995	1.1867	0.24689	0.044908	0.99441	1.0584	0.081892	0.43304	0.98164	1.0598	0.083827	0.028319	0.99996	1.2549	0.32752	0.03348	0.59655	1.1309	0.17748	0.056379	0.99959	1.1117	0.15274	0.001794	0.099125	1.2912	0.36867	0.51001	0.99984	1.099	0.13623	0.10905	0.99966	1.1748	0.23241
294.9398	0.5	domics Pos	0.99332	0.99995	1.0038	0.005541	0.6782	0.99979	0.97818	-0.03183	0.023569	0.32869	0.93326	-0.09966	0.32343	0.99996	0.94619	-0.0798	0.073307	0.7908	0.92801	-0.1078	0.58242	0.99959	0.97141	-0.04184	0.080941	0.27033	1.1851	0.24507	0.49934	0.99984	0.95959	-0.05951	0.91727	0.99966	1.0116	0.016698
156.139	2.05	domics Pos	0.009068	0.68962	0.87907	-0.18596	0.044372	0.99441	0.76296	-0.39032	0.00654	0.18071	0.78801	-0.34371	0.32807	0.99996	0.90038	-0.15139	0.031905	0.59375	0.82375	-0.27972	0.16621	0.99959	0.81231	-0.2999	0.067433	0.25306	1.245	0.31611	0.18354	0.99984	0.81555	-0.29415	0.14444	0.99966	0.91453	-0.1289
559.5196	1.67	domics Pos	0.099479	0.99995	0.71884	-0.47627	0.094478	0.99979	0.62689	-0.67371	0.004002	0.15412	0.56362	-0.8272	0.41788	0.99996	0.84567	-0.24184	0.010832	0.46936	0.61348	-0.70492	0.084647	0.99959	0.63022	-0.66607	0.72712	0.84098	1.1427	0.19241	0.16408	0.99984	0.68827	-0.53895	0.090277	0.99966	0.77986	-0.35872
142.1239	5.99	bolomics Pc	0.10515	0.99964	0.86309	-0.21242	0.053476	0.91628	0.75542	-0.40465	0.027742	0.78784	0.81919	-0.28773	0.69247	0.99987	0.96802	-0.04689	0.022619	0.95986	0.84813	-0.23763	0.13385	0.99796	0.79221	-0.33605	0.69809	0.99768	1.0287	0.040826	0.12594	0.9981	0.79329	-0.33408	0.045498	0.99995	0.85097	-0.23281
278.246	1.91	domics Pos	0.47256	0.99995	0.913	-0.13132	0.15178	0.99979	0.79183	-0.33674	0.029303	0.36633	0.73288	-0.44836	0.75112	0.99996	0.96184	-0.05613	0.13748	0.88067	0.83887	-0.25348	0.18857	0.99959	0.78909	-0.34175	0.26761	0.48637	1.1676	0.22358	0.17264	0.99984	0.75992	-0.39608	0.49681	0.99966	0.92391	-0.11417
141.1284	2.05	domics Pos	0.19624	0.99995	0.90548	-0.14324	0.15451	0.99979	0.83907	-0.25315	0.002891	0.15208	0.77219	-0.37298	0.49398	0.99996	0.91657	-0.12568	0.044602	0.66693	0.79323	-0.33419	0.0141	0.99959	0.75443	-0.40655	0.21248	0.43003	1.1531	0.2055	0.156	0.99984	0.75409	-0.4072	0.008535	0.99749	0.83635	-0.25782
123.1175	1.66	domics Pos	0.02904	0.89298	0.90143	-0.14971	0.013067	0.99441	0.72551	-0.46293	0.000594	0.13107	0.7529	-0.40948	0.24303	0.99996	0.86826	-0.2038	0.038163	0.63362	0.81148	-0.30136	0.19691	0.99959	0.79433	-0.33218	0.15434	0.36252	1.1698	0.22629	0.2202	0.99984	0.81024	-0.30358	0.01438	0.99749	0.86989	-0.20109
81.0707	2.05	domics Pos	0.010907	0.68962	0.87827	-0.18726	0.16586	0.99979	0.82548	-0.27669	0.007794	0.19873	0.76051	-0.39496	0.34129	0.99996	0.89772	-0.15566	0.007549	0.46849	0.80562	-0.31183	0.15435	0.99959	0.81054	-0.30305	0.08996	0.28541	1.2015	0.26486	0.16949	0.99984	0.79389	-0.333	0.060406	0.99966	0.87765	-0.18829
177.86	10.78	olomics Ne	0.70455	0.9951	1.0196	0.02803	0.47089	0.99975	1.0433	0.061174	0.34782	0.99731	0.93477	-0.09732	0.54767	0.99915	1.0503	0.070776	0.29729	0.7204	1.0827	0.11464	0.25899	0.66013	1.0705	0.098243	0.018507	0.073021	1.26	0.3334	0.89936	0.99481	1.0146	0.020849	0.26326	0.91738	0.92246	-0.11644
184.1701	1.66	domics Pos	0.030555	0.90678	0.87917	-0.18578	0.049527	0.99441	0.76772	-0.38134	0.004813	0.16683	0.77029	-0.37652	0.29686	0.99996	0.87333	-0.19541	0.023748	0.5516	0.82394	-0.27939	0.15375	0.99959	0.80361	-0.31543	0.072134	0.25957	1.24	0.31037	0.22605	0.99984	0.82591	-0.27595	0.29579	0.99966	0.92683	-0.10962
935.8058	10.02	domics Pos	0.79986	0.99995	1.0225	0.032049	0.97976	0.99979	1.0026	0.003731	0.6958	0.99601	0.98654	-0.01955	0.60639	0.99996	0.95655	-0.06409	0.77923	0.99701	0.98485	-0.02203	0.41264	0.99959	0.96145	-0.05672	0.025993	0.19068	1.232	0.30099	0.57574	0.99984	1.0835	0.11572	0.58808	0.99966	0.95674	-0.0638
184.1708	6.38	bolomics Pc	0.002347	0.35208	0.83122	-0.26669	0.033511	0.88953	0.76331	-0.38965	0.001731	0.39871	0.81643	-0.29259	0.43003	0.99987	0.94871	-0.07596	0.032389	0.95986	0.87089	-0.19943	0.1406	0.99796	0.83038	-0.26815	0.041387	0.69717	1.0876	0.12119	0.097103	0.9981	0.79421	-0.3324	0.083651	0.99995	0.87368	-0.19483
95.0883	4.73	domics Pos	0.70105	0.99995	0.95954	-0.05959	0.30844	0.99979	1.077	0.107	0.30112	0.89817	1.0981	0.135	0.90123	0.99996	0.99017	-0.01425	0.74567	0.99701	1.0323	0.045824	0.41235	0.99959	1.0782	0.10859	0.044705	0.21733	1.4035	0.48898	0.43641	0.99984	1.1096	0.14998	0.083066	0.99966	1.199	0.26181
135.1175	2.05	domics Pos	0.02491	0.85508	0.88011	-0.18425	0.064092	0.99979	0.79461	-0.33169	0.003703	0.15208	0.77605	-0.36578	0.29326	0.99996	0.87821	-0.18736	0.016279	0.49216	0.8003	-0.32139	0.12779	0.99959	0.79883	-0.32404	0.059202	0.24015	1.2439	0.31485	0.17341	0.99984	0.79062	-0.33894	0.19358	0.99966	0.91381	-0.13004
736.5769	2.05	domics Pos	0.13587	0.99995	0.72074	-0.47245	0.082417	0.99979	0.66774	-0.58265	0.044207	0.45434	0.63057	-0.66528	0.21898	0.99996	0.77257	-0.37226	0.12253	0.86273	0.70584	-0.50259	0.43174	0.99959	0.83109	-0.26692	0.073613	0.26156	1.3591	0.44267	0.18005	0.99984	0.66722	-0.58377	0.63228	0.99966	0.8953	-0.15955
187.6283	1.47	domics Pos	0.10405	0.99995	0.8621	-0.21407	0.007243	0.99441	0.6515	-0.61817	0.019171	0.30022	0.69058	-0.53412	0.00133	0.99996	0.65107	-0.61912	0.028651	0.56604	0.76404	-0.38827	0.054858	0.99959	0.69866	-0.51734	0.61284	0.77659	1.0978	0.13458	0.24718	0.99984	0.8171	-0.29141	0.10556	0.99966	0.83039	-0.26814
163.1492	6	bolomics Pc	0.045213	0.99964	0.84895	-0.23625	0.088638	1	0.76473	-0.38698	0.048558	0.99882	0.81619	-0.29302	0.30598	0.99987	0.90154	-0.14953	0.054948	0.95986	0.86453	-0.21001	0.1795	0.99796	0.79263	-0.33528	0.35669	0.99768	1.0721	0.10046	0.14497	0.9981	0.80201	-0.31831	0.076287	0.99995	0.84051	-0.25066
163.1487	1.66	domics Pos	0.035607	0.944	0.85894	-0.21938	0.036031	0.99441	0.75356	-0.4082	0.000937	0.13107	0.73301	-0.4481	0.30162	0.99996	0.87411	-0.19411	0.017167	0.49216	0.7843	-0.35052	0.063937	0.99959	0.73938	-0.4356	0.25213	0.47033	1.1461	0.19677	0.080384	0.99984	0.7611	-0.39384	0.066003	0.99966	0.88711	-0.17282
200.6362	1.67	domics Pos	0.12047	0.99995	0.82272	-0.28153	0.052735	0.99441	0.78686	-0.34582	0.011104	0.22917	0.7226	-0.46873	0.10414	0.99996	0.83586	-0.25867	0.022709	0.55152	0.77618	-0.36555	0.14895	0.99959	0.77266	-0.37209	0.21541	0.43393	1.261	0.33457	0.14409	0.99984	0.76806	-0.38071	0.051129	0.99966	0.82344	-0.28026
114.0919	2.05	domics Pos	0.031418	0.91043	0.8605	-0.21676	0.11152	0.99979	0.79378	-0.33319	0.013729	0.25537	0.79022	-0.33967	0.46855	0.99996	0.91682	-0.12529	0.021174	0.53763	0.78637	-0.34671	0.13954	0.99959	0.79887	-0.32397	0.15001	0.35809	1.1623	0.21698	0.13661	0.99984	0.79085	-0.33852	0.086547	0.99966	0.8925	-0.16408
636.9719	12	olomics Ne	0.783	0.9951	0.97988	-0.02932	0.067445	0.99975	1.2045	0.2684	0.63378	0.99731	0.96365	-0.05342	0.92363	0.99915	1.0314	0.044564	0.44015	0.79466	1.0724	0.10081	0.1782	0.60765	1.1721	0.22911	0.019972	0.074983	1.2905	0.36793	0.92769	0.99481	1.0196	0.028028	0.20953	0.91738	0.8943	-0.16117
128.1077	1.67	domics Pos	0.031806	0.91043	0.85135	-0.23218	0.041818	0.99441	0.75955	-0.39678	0.000354	0.13107	0.74749	-0.41988	0.38247	0.99996	0.89842	-0.15454	0.007861	0.46849	0.78852	-0.34279	0.2207	0.99959	0.82567	-0.27636	0.085013	0.27673	1.19	0.25096	0.16604	0.99984	0.81016	-0.30371	0.055096	0.99966	0.89294	-0.16337
647.5033	2.05	domics Pos	0.07288	0.99995	0.88363	-0.17848	0.18294	0.99979	0.80008	-0.32178	0.010951	0.22818	0.66932	-0.57924	0.21416	0.99996	0.83633	-0.25786	0.086141	0.81301	0.8434	-0.24572	0.19345	0.99959	0.785	-0.34924	0.023055	0.18664	1.2845	0.36123	0.18566	0.99984	0.76669	-0.38328	0.081524	0.99966	0.85649	-0.22349
518.9475	0.54	domics Pos	0.50195	0.99995	0.88859	-0.17041	0.2278	0.99979	0.81334	-0.29807	0.022723	0.32209	0.65602	-0.6082	0.47813	0.99996	0.90252	-0.14797	0.065084	0.76594	0.62914	-0.66855	0.72113	0.99959	1.0502	0.070725	0.075202	0.26286	1.8776	0.90892	0.27218	0.99984	0.77111	-0.37499	0.3919	0.99966	0.84464	-0.24358
283.283	2.05	domics Pos	0.003204	0.59581	0.86456	-0.20995	0.046783	0.99441	0.77083	-0.37552	0.003177	0.15208	0.77502	-0.3677	0.35945	0.99996	0.90207	-0.14868	0.007304	0.46849	0.81476	-0.29556	0.12602	0.99959	0.79839	-0.32483	0.09308	0.28973	1.1971	0.25952	0.18393	0.99984	0.80359	-0.31548	0.05897	0.99966	0.89602	-0.1584
191.1805	6.38	bolomics Pc	0.010196	0.68734	0.86429	-0.21042	0.054333	0.91931	0.76216	-0.39183	0.014315	0.60937	0.82918	-0.27025	0.61857	0.99987	0.96513	-0.05121	0.23751	0.95986	0.94787	-0.07724	0.23047	0.99796	0.83913	-0.25303	0.056325	0.77855	1.1304	0.17682	0.18124	0.9981	0.84311	-0.2462	0.22154	0.99995	0.8881	-0.17121
282.2801	2.05	domics Pos	0.006502	0.68962	0.86576	-0.20797	0.039711	0.99441	0.74724	-0.42036	0.003384	0.15208	0.76655	-0.38354	0.31499	0.99996	0.88884	-0.17	0.008316	0.46849	0.79529	-0.33045	0.1245	0.99959	0.78372	-0.3516	0.12482	0.32487	1.1903	0.25128	0.19233	0.99984	0.80371	-0.31525	0.068558	0.99966	0.87958	-0.18511
935.7888	0.53	domics Pos	0.62305	0.99995	1.0613	0.085847	0.36531	0.99979	1.0489	0.068839	0.056538	0.52452	0.87822	-0.18734	0.68171	0.99996	0.96958	-0.04457	0.96583	0.99701	1.0065	0.009403	0.60182	0.99959	0.96066	-0.0579	0.021677	0.18449	1.3442	0.42679	0.81153	0.99984	1.0432	0.061064	0.74398	0.99966	0.97596	-0.0351
263.2375	1.66	domics Pos	0.003846	0.60743	0.86301	-0.21255	0.030075	0.99441	0.76113	-0.39378	0.001855	0.13302	0.76228	-0.3916	0.28438	0.99996	0.87279	-0.19629	0.007866	0.46849	0.81332	-0.2981	0.15976	0.99959	0.79917	-0.32343	0.090481	0.28631	1.1823	0.24155	0.18272	0.99984	0.81226	-0.29998	0.045495	0.99966	0.88502	-0.17622
674.7755	9.93	domics Pos	0.75284	0.99995	1.0307	0.043566	0.50955	0.99979	0.92803	-0.10776	0.025282	0.34064	0.8205	-0.28542	0.36672	0.99996	0.92767	-0.10832	0.80244	0.99701	0.97908	-0.0305	0.39943	0.99959	0.91279	-0.13164	0.024507	0.18846	1.2545	0.3271	0.41338	0.99984	0.90927	-0.13722	0.6732	0.99966	0.95552	-0.06564
434.9792	4.82	olomics Ne	0.63077	0.9951	1.0242	0.034556	0.25771	0.99975	1.0534	0.07504	0.11706	0.99731	0.92829	-0.10735	0.34665	0.99915	1.0313	0.044485	0.15389	0.64314	1.0767	0.10666	0.016304	0.45618	1.1295	0.17571	0.026174	0.086848	1.193	0.25462	0.63674	0.99481	1.0329	0.04664	0.19988	0.91738	0.92306	-0.11551
647.5021	6.38	bolomics Pc	0.54252	0.99964	0.94742	-0.07792	0.32977	1	0.88363	-0.17848	0.026053	0.77259	0.80032	-0.32135	0.96684	0.99987	1.0042	0.006048	0.084361	0.95986	0.86213	-0.21403	0.55166	0.99796	0.93612	-0.09524	0.043342	0.71108	1.1645	0.21971	0.16063	0.9981	0.79426	-0.33231	0.21808	0.99995	0.91184	-0.13314
163.1487	2.05	domics Pos	0.00148	0.59581	0.8382	-0.25464	0.0609	0.99979	0.7764	-0.36513	0.00023	0.13107	0.7466	-0.4216	0.2747	0.99996	0.87259	-0.19662	0.010689	0.46849	0.80251	-0.3174	0.11393	0.99959	0.78436	-0.35042	0.11475	0.31869	1.1829	0.24237	0.16369	0.99984	0.78637	-0.34672	0.025558	0.99966	0.8821	-0.18099
366.9806	4.65	olomics Ne	0.3624	0.9951	1.0657	0.091844	0.94228	0.99975	0.99943	-0.00083	0.90204	0.99731	1.0112	0.01611	0.34219	0.99915	1.0322	0.045765	0.014517	0.50209	1.1338	0.18115	0.10375	0.53216	1.0986	0.13569	0.02928	0.092883	1.2274	0.29557	0.16324	0.99481	1.0768	0.10675	0.36993	0.92651	0.95922	-0.06007
680.5182	2.74	domics Pos	0.81823	0.99995	0.96093	-0.05749	0.030744	0.99441	0.59526	-0.74841	0.2265	0.83605	0.78628	-0.34688	0.22504	0.99996	0.72041	-0.4731	0.3033	0.99701	0.7991	-0.32355	0.029816	0.99959	0.4891	-1.0318	0.48763	0.69172	0.86396	-0.21097	0.32288	0.99984	0.79251	-0.3355	0.2104	0.99966	0.72325	-0.46744
149.1336	6	bolomics Pc	0.12619	0.99964	0.90827	-0.1388	0.044312	0.88953	0.79092	-0.3384	0.1147	0.99882	0.89002	-0.16809	0.89289	0.99987	1.0271	0.038522	0.34709	0.95986	0.94124	-0.08737	0.11268	0.99796	0.79997	-0.32197	0.11561	0.92707	1.1167	0.1593	0.2188	0.9981	0.85314	-0.22914	0.20851	0.99995	0.92339	-0.11498
150.1364	2.05	domics Pos	0.015714	0.68962	0.87845	-0.18698	0.016153	0.99441	0.7266	-0.46076	0.012326	0.23979	0.80752	-0.30843	0.16706	0.99996	0.84203	-0.24806	0.00594	0.46849	0.82271	-0.28155	0.045838	0.99959	0.7404	-0.43363	0.094483	0.29106	1.1573	0.21074	0.14143	0.99984	0.81108	-0.30209	0.06939	0.99966	0.86154	-0.21501
166.1637	5.99	bolomics Pc	0.097491	0.99964	0.79884	-0.32401	0.48105	1	0.9186	-0.12249	0.083366	0.99882	0.8003	-0.32138	0.95351	0.99987	1.0158	0.022681	0.029682	0.95986	0.75411	-0.40715	0.057532	0.99796	0.8098	-0.30437	0.37923	0.99768	0.91616	-0.12632	0.029621	0.9981	0.77252	-0.37236	0.15625	0.99995	0.85144	-0.23202
179.1788	1.67	domics Pos	0.008257	0.68962	0.84287	-0.24662	0.054687	0.99441	0.781	-0.35661	0.001158	0.13107	0.71867	-0.47659	0.47105	0.99996	0.91427	-0.12931	0.007882	0.46849	0.80607	-0.31102	0.061021	0.99959	0.74127	-0.43193	0.22991	0.45179	1.1096	0.15004	0.10559	0.99984	0.78243	-0.35397	0.041793	0.99966	0.86568	-0.20809
421.8891	10.07	domics Pos	0.48605	0.99995	1.0389	0.055046	0.32691	0.99979	0.93751	-0.09309	0.026543	0.34652	0.89117	-0.16623	0.56822	0.99996	1.0641	0.089616	0.18406	0.96469	0.90743	-0.14014	0.17429	0.99959	0.91461	-0.12877	0.38645	0.60866	1.0659	0.092052	0.30511	0.99984	0.9132	-0.13099	0.68023	0.99966	1.0388	0.054961
532.6174	0.53	domics Pos	0.80934	0.99995	1.0575	0.080613	0.15414	0.99979	0.81035	-0.30339	0.023717	0.32884	0.71047	-0.49316	0.72106	0.99996	1.0751	0.10448	0.05883	0.76299	0.65465	-0.61121	0.41783	0.99959	0.87255	-0.19669	0.56878	0.75096	1.5253	0.60912	0.60944	0.99984	0.92852	-0.10699	0.89105	0.99966	1.0689	0.096123
291.2686	2.32	domics Pos	0.21148	0.99995	0.87467	-0.19319	0.047031	0.99441	0.7043	-0.50574	0.10054	0.68915	0.79537	-0.3303	0.55524	0.99996	0.92401	-0.11403	0.019833	0.5336	0.75484	-0.40576	0.60029	0.99959	0.9288	-0.10656	0.24733	0.46664	1.1803	0.23918	0.33905	0.99984	0.83166	-0.26594	0.2386	0.99966	0.87699	-0.18937
285.2892	6.38	bolomics Pc	0.055315	0.99964	0.85476	-0.2264	0.043823	0.88953	0.74301	-0.42854	0.014837	0.60995	0.85461	-0.22667	0.21793	0.99987	0.90273	-0.14764	0.14813	0.95986	0.8959	-0.15859	0.082373	0.99796	0.778	-0.36215	0.52865	0.99768	1.0167	0.023954	0.095398	0.9981	0.80127	-0.31963	0.004807	0.97908	0.87234	-0.19703
281.2673	1.66	domics Pos	0.024323	0.85085	0.87118	-0.19896	0.052332	0.99441	0.77244	-0.37251	0.003558	0.15208	0.75944	-0.39699	0.35402	0.99996	0.887	-0.17299	0.021726	0.54145	0.82541	-0.27682	0.16293	0.99959	0.80279	-0.31691	0.080889	0.27033	1.1911	0.25235	0.19875	0.99984	0.81546	-0.29431	0.069237	0.99966	0.88701	-0.17297
156.1395	6.38	bolomics Pc	0.002123	0.33442	0.84706	-0.23947	0.029249	0.88953	0.76725	-0.38222	0.013393	0.60269	0.82283	-0.28134	0.362	0.99987	0.92558	-0.11156	0.027631	0.95986	0.88611	-0.17445	0.13093	0.99796	0.82025	-0.28587	0.087305	0.85775	1.0681	0.094997	0.057601	0.9981	0.77767	-0.36277	0.036423	0.99995	0.85643	-0.2236
545.8022	2.95	bolomics Pc	0.5744	0.99964	0.92835	-0.10726	0.033881	0.88953	0.7908	-0.33861	0.058371	0.99882	0.69921	-0.51619	0.78811	0.99987	1.0199	0.028381	0.015614	0.95986	0.60291	-0.72999	0.19332	0.99796	0.851	-0.23276	0.97489	0.99768	1.1366	0.18476	0.24979	0.9981	0.83068	-0.26763	0.55453	0.99995	0.92849	-0.10705
400.8291	9.92	domics Pos	0.40597	0.99995	1.0665	0.092871	0.6698	0.99979	1.0225	0.032154	0.43515	0.98164	0.9579	-0.06205	0.5016	0.99996	0.94316	-0.08443	0.3423	0.99701	0.92044	-0.1196	0.56579	0.99959	0.95175	-0.07135	0.01529	0.17097	1.2456	0.3169	0.89863	0.99984	1.0202	0.028789	0.67353	0.99966	1.0327	0.046491
1158.719	10.05	domics Pos	0.26565	0.99995	1.1345	0.18205	0.97045	0.99979	1.0075	0.010807	0.15986	0.78347	0.9036	-0.14624	0.83188	0.99996	0.98779	-0.01772	0.46829	0.99701	0.94607	-0.07998	0.9247	0.99959	1.017	0.02436	0.029023	0.19804	1.2664	0.34077	0.48037	0.99984	1.1159	0.15818	0.49267	0.99966	0.93644	-0.09475
288.1611	1.3	domics Pos	0.44305	0.99995	1.0709	0.098787	0.36536	0.99979	0.8968	-0.15715	0.1807	0.80578	0.85987	-0.21781	0.5751	0.99996	0.94323	-0.08432	0.2942	0.99701	0.8727	-0.19644	0.89609	0.99959	1.029	0.041245	0.046789	0.22017	1.2339	0.30324	0.46388	0.99984	0.93171	-0.10205	0.17406	0.99966	1.1275	0.17312
449.9787	4.98	olomics Ne	0.25736	0.9951	1.1048	0.14382	0.20122	0.99975	1.0632	0.088428	0.67028	0.99731	0.98005	-0.02907	0.20096	0.99915	1.0559	0.078428	0.071072	0.5958	1.177	0.23512	0.056219	0.51062	1.1312	0.17789	0.010411	0.06126	1.2479	0.31951	0.034315	0.99481	1.1267	0.17204	0.67929	0.96812	0.97622	-0.03472
124.1214	2.05	domics Pos	0.059079	0.99995	0.75425	-0.40689	0.13573	0.99979	0.77686	-0.36427	0.008277	0.19873	0.66329	-0.59228	0.30711	0.99996	0.83492	-0.2603	0.087243	0.81905	0.76145	-0.39318	0.1119	0.99959	0.70304	-0.50833	0.30261	0.52448	1.1715	0.22833	0.11052	0.99984	0.70041	-0.51373	0.30412	0.99966	0.87231	-0.19708
135.0053	0.61	lomics Neg	0.1691	0.99486	1.4614	0.5473	0.12514	0.99506	1.4929	0.57809	0.048377	0.6372	1.7419	0.80062	0.036248	0.80547	1.641	0.7146	0.068983	0.39567	1.4197	0.50555	0.038241	0.89898	1.5001	0.58503	0.16562	0.99905	1.2734	0.34871	0.31207	0.96955	1.1772	0.23539	0.32621	0.74722	1.1839	0.24358
412.857	0.29	bolomics Pc	0.6256	0.99964	0.97221	-0.04066	0.60541	1	0.97257	-0.04013	0.52834	0.99882	0.95882	-0.06066	0.4995	0.99987	0.94669	-0.07903	0.01676	0.95986	0.87818	-0.18741	0.50781	0.99796	0.96158	-0.05652	0.15742	0.96247	0.88603	-0.17458	0.18047	0.9981	0.90804	-0.13917	0.23402	0.99995	0.93255	-0.10075
212.2013	2.76	domics Pos	0.83522	0.99995	0.98454	-0.02248	0.025612	0.99441	0.70107	-0.51238	0.019279	0.30022	0.8136	-0.29761	0.40121	0.99996	0.88174	-0.18157	0.016743	0.49216	0.81657	-0.29236	0.18722	0.99959	0.77033	-0.37645	0.074348	0.26191	1.287	0.36402	0.28555	0.99984	0.83183	-0.26565	0.10411	0.99966	0.87829	-0.18722
161.133	1.67	domics Pos	0.001704	0.59581	0.81977	-0.2867	0.079157	0.99979	0.78735	-0.34492	0.010523	0.22168	0.74611	-0.42253	0.40697	0.99996	0.90169	-0.14929	0.013289	0.48721	0.78538	-0.34853	0.06393	0.99959	0.74493	-0.42482	0.20716	0.42281	1.1902	0.25126	0.16682	0.99984	0.81198	-0.30048	0.064436	0.99966	0.87695	-0.18943
237.2218	1.47	domics Pos	0.010946	0.68962	0.8103	-0.30347	0.026499	0.99441	0.77099	-0.37522	0.003709	0.15208	0.7095	-0.49513	0.36855	0.99996	0.88929	-0.16927	0.005295	0.46849	0.78259	-0.35367	0.20825	0.99959	0.81666	-0.29219	0.093187	0.28973	1.1121	0.15323	0.16756	0.99984	0.77983	-0.35876	0.03906	0.99966	0.88443	-0.17717
149.1337	6.38	bolomics Pc	0.021852	0.88137	0.90447	-0.14485	0.012909	0.88953	0.79701	-0.32733	0.017952	0.63482	0.8913	-0.16602	0.95943	0.99987	1.007	0.0101	0.69925	0.97678	0.98143	-0.02705	0.14307	0.99796	0.82827	-0.27183	0.13977	0.95284	1.0787	0.10923	0.19231	0.9981	0.88021	-0.18409	0.15884	0.99995	0.89321	-0.16294
198.1864	6.38	bolomics Pc	0.005287	0.49147	0.85114	-0.23253	0.034057	0.88953	0.77092	-0.37535	0.008323	0.55189	0.83968	-0.2521	0.4626	0.99987	0.95117	-0.07222	0.044907	0.95986	0.88394	-0.17798	0.1914	0.99796	0.83845	-0.25421	0.036599	0.66991	1.1084	0.14852	0.078126	0.9981	0.79108	-0.3381	0.16244	0.99995	0.89062	-0.16711
424.2002	2.05	domics Pos	0.32956	0.99995	1.0928	0.12805	0.43497	0.99979	1.1131	0.15459	0.64552	0.99601	1.061	0.085465	0.54333	0.99996	1.1	0.13748	0.34044	0.99701	1.109	0.14923	0.86202	0.99959	1.0417	0.058897	0.046573	0.22017	1.5664	0.64741	0.50471	0.99984	1.1617	0.21625	0.29896	0.99966	1.1421	0.19173
678.3681	0.56	domics Pos	0.88584	0.99995	1.0461	0.065064	0.13665	0.99979	0.79519	-0.33064	0.045834	0.46561	0.75774	-0.40022	0.64507	0.99996	1.0431	0.060861	0.077175	0.79327	0.6365	-0.65177	0.17914	0.99959	0.78456	-0.35005	0.84113	0.90801	1.3129	0.39275	0.73029	0.99984	0.96559	-0.05052	0.8315	0.99966	1.0409	0.057763
722.5249	2.05	domics Pos	0.09373	0.99995	0.77991	-0.35862	0.066198	0.99979	0.6635	-0.59183	0.025215	0.34064	0.67943	-0.5576	0.12924	0.99996	0.78664	-0.34622	0.038542	0.63362	0.7008	-0.51292	0.10178	0.99959	0.74501	-0.42467	0.079131	0.26837	1.2326	0.30168	0.052579	0.99984	0.67564	-0.56567	0.089033	0.99966	0.83551	-0.25927
1155.744	10.05	domics Pos	0.17751	0.99995	1.1918	0.2532	0.6256	0.99979	1.0487	0.068643	0.81333	0.99702	0.99116	-0.01281	0.57785	0.99996	0.95207	-0.07085	0.89343	0.99701	1.0169	0.024231	0.17894	0.99959	1.1532	0.20563	0.002226	0.10737	1.4127	0.4985	0.99739	0.99984	1.0245	0.034953	0.18221	0.99966	0.94152	-0.08693
121.1025	6.38	bolomics Pc	0.13947	0.99964	0.91228	-0.13245	0.018002	0.88953	0.82618	-0.27546	0.050472	0.99882	0.89651	-0.1576	0.69353	0.99987	0.96829	-0.04648	0.59833	0.95986	0.96422	-0.05257	0.17218	0.99796	0.84364	-0.2453	0.13094	0.94474	1.088	0.12169	0.20099	0.9981	0.87579	-0.19134	0.23469	0.99995	0.89506	-0.15994
165.1643	2.05	domics Pos	0.047147	0.99995	0.83551	-0.25928	0.05421	0.99441	0.74393	-0.42676	0.004666	0.16474	0.72479	-0.46436	0.35208	0.99996	0.88881	-0.17006	0.050534	0.70556	0.82536	-0.2769	0.23553	0.99959	0.81137	-0.30157	0.069308	0.25546	1.242	0.31269	0.29094	0.99984	0.8256	-0.27648	0.15908	0.99966	0.87148	-0.19845
354.1871	1.65	domics Pos	0.20316	0.99995	0.88322	-0.17915	0.003959	0.99441	0.74584	-0.42306	0.016247	0.28182	0.73031	-0.45343	0.62268	0.99996	0.95246	-0.07027	0.14708	0.90678	0.84715	-0.23931	0.33792	0.99959	0.87072	-0.19971	0.23228	0.45379	1.1742	0.23166	0.18185	0.99984	0.82874	-0.27101	0.016546	0.99966	0.84993	-0.23458
113.0966	2.05	domics Pos	0.38997	0.99995	0.89126	-0.16609	0.14524	0.99979	0.81339	-0.29799	0.039379	0.42996	0.81249	-0.29958	0.61571	0.99996	0.9466	-0.07917	0.23746	0.99701	0.91603	-0.12653	0.15279	0.99959	0.82703	-0.27399	0.64433	0.79355	1.0853	0.11807	0.23673	0.99984	0.85139	-0.23212	0.059922	0.99966	0.876	-0.191
247.2432	6.38	bolomics Pc	0.007053	0.60235	0.85633	-0.22377	0.039003	0.88953	0.76995	-0.37716	0.011717	0.59292	0.84439	-0.24402	0.31235	0.99987	0.9238	-0.11435	0.070504	0.95986	0.89199	-0.1649	0.12386	0.99796	0.81294	-0.29878	0.0949	0.87638	1.0693	0.096678	0.087298	0.9981	0.80004	-0.32185	0.044515	0.99995	0.85807	-0.22084
302.246	1.66	domics Pos	0.10689	0.99995	0.88626	-0.1742	0.049462	0.99441	0.7637	-0.38893	0.049173	0.48449	0.81552	-0.29421	0.28382	0.99996	0.85517	-0.22571	0.090305	0.82209	0.85613	-0.2241	0.19087	0.99959	0.79385	-0.33306	0.079342	0.26844	1.1846	0.24435	0.26034	0.99984	0.84169	-0.24863	0.1168	0.99966	0.87789	-0.18789
238.2173	1.66	domics Pos	0.18541	0.99995	0.88412	-0.17769	0.085388	0.99979	0.79632	-0.32859	0.011877	0.23587	0.75509	-0.40528	0.37573	0.99996	0.873	-0.19595	0.16615	0.93861	0.87028	-0.20045	0.28739	0.99959	0.83596	-0.2585	0.03011	0.19804	1.2002	0.26328	0.32777	0.99984	0.85433	-0.22714	0.25524	0.99966	0.88678	-0.17334
392.9772	4.66	olomics Ne	0.49096	0.9951	1.0334	0.047347	0.74482	0.99975	1.0161	0.023084	0.6039	0.99731	0.97904	-0.03055	0.24511	0.99915	1.0345	0.048965	0.050863	0.58152	1.0957	0.13182	0.008439	0.45618	1.1125	0.1538	0.014149	0.06612	1.2224	0.28974	0.39013	0.99481	1.0423	0.05975	0.40835	0.94239	0.96037	-0.05834
228.2331	5.49	bolomics Pc	0.019663	0.86976	0.88087	-0.183	0.097183	1	0.79681	-0.3277	0.00871	0.55189	0.8188	-0.28842	0.59696	0.99987	0.9605	-0.05815	0.006792	0.95986	0.86165	-0.21483	0.3649	0.99796	0.88504	-0.17618	0.35927	0.99768	1.043	0.06078	0.13434	0.9981	0.81328	-0.29818	0.040628	0.99995	0.86851	-0.20338
299.3152	7.12	bolomics Pc	0.28068	0.99964	1.0985	0.13555	0.055324	0.91931	1.1664	0.22206	0.15261	0.99882	1.1583	0.21202	0.05222	0.99987	1.2278	0.29603	0.60931	0.95986	1.036	0.051062	0.044765	0.99796	1.2272	0.29534	0.2698	0.99768	1.0985	0.13558	0.83898	0.9981	1.0245	0.034896	0.19922	0.99995	1.1093	0.14962
276.2268	1.46	domics Pos	0.35208	0.99995	0.94314	-0.08446	0.00821	0.99441	0.75541	-0.40467	0.016495	0.28182	0.76307	-0.39012	0.14801	0.99996	0.81597	-0.29342	0.29253	0.99701	0.87825	-0.18729	0.10986	0.99959	0.76925	-0.37848	0.29028	0.51147	1.1492	0.20065	0.32106	0.99984	0.87182	-0.1979	0.025745	0.99966	0.74756	-0.41973
264.2409	1.66	domics Pos	0.01115	0.68962	0.86196	-0.2143	0.032063	0.99441	0.74982	-0.41538	0.003362	0.15208	0.76446	-0.38749	0.31185	0.99996	0.87407	-0.19418	0.00673	0.46849	0.81021	-0.30363	0.14069	0.99959	0.78172	-0.35528	0.12302	0.32395	1.1826	0.24193	0.1874	0.99984	0.81652	-0.29244	0.079888	0.99966	0.89147	-0.16574
533.6212	0.53	domics Pos	0.92191	0.99995	1.0646	0.090283	0.14641	0.99979	0.83423	-0.26148	0.012595	0.24256	0.65349	-0.61376	0.85577	0.99996	1.1745	0.23208	0.056617	0.74699	0.6113	-0.71005	0.4123	0.99959	0.89771	-0.15568	0.47219	0.68341	1.6473	0.72011	0.62563	0.99984	0.93844	-0.09167	0.96893	0.99966	1.1225	0.16668
681.1623	1.29	domics Pos	0.37419	0.99995	1.1114	0.15232	0.88361	0.99979	0.99176	-0.01194	0.54726	0.99601	1.0473	0.066667	0.15836	0.99996	1.1152	0.15732	0.41666	0.99701	0.94066	-0.08826	0.96611	0.99959	1.0041	0.005919	0.041759	0.21081	1.2786	0.3546	0.22331	0.99984	1.1091	0.14941	0.79537	0.99966	1.0553	0.07759
198.1856	2.76	domics Pos	0.10118	0.99995	0.85135	-0.23218	0.006677	0.99441	0.56816	-0.81564	0.001802	0.13302	0.72386	-0.46621	0.1522	0.99996	0.77824	-0.36172	0.014636	0.4899	0.75518	-0.40512	0.081417	0.99959	0.69423	-0.52651	0.2843	0.50444	1.1493	0.20078	0.2013	0.99984	0.77963	-0.35913	0.008374	0.99749	0.79444	-0.33199
224.2014	1.66	domics Pos	0.1311	0.99995	0.88987	-0.16833	0.12415	0.99979	0.78927	-0.34141	0.020128	0.30717	0.75706	-0.40152	0.35418	0.99996	0.87644	-0.19028	0.073098	0.7908	0.84408	-0.24455	0.22681	0.99959	0.80771	-0.30809	0.088977	0.28388	1.1978	0.2604	0.30257	0.99984	0.84217	-0.24782	0.33618	0.99966	0.92177	-0.11752

941.5301	4.85	domics Pos	0.56154	0.99995	1.0664	0.092686	0.76828	0.99979	0.97847	-0.0314	0.37021	0.93382	1.0451	0.063673	0.4646	0.99996	1.0849	0.11761	0.6441	0.99701	1.0514	0.072282	0.52994	0.99959	1.1306	0.17714	0.003548	0.11893	1.3545	0.43773	0.1243	0.99984	1.1452	0.19554	0.013359	0.99749	1.1706	0.22724
254.2483	1.47	domics Pos	0.021118	0.79726	0.88669	-0.17349	0.036163	0.99441	0.78277	-0.35335	0.00353	0.15208	0.77063	-0.3759	0.31423	0.99996	0.88402	-0.17785	0.01595	0.49216	0.82444	-0.27852	0.11449	0.99959	0.78507	-0.3491	0.10862	0.31085	1.1719	0.22888	0.1786	0.99984	0.80244	-0.31753	0.1079	0.99966	0.8979	-0.15537
280.2641	1.66	domics Pos	0.025387	0.85508	0.87874	-0.18649	0.045787	0.99441	0.76823	-0.38038	0.007378	0.19537	0.78593	-0.34752	0.32432	0.99996	0.87982	-0.18471	0.035346	0.61824	0.8336	-0.26258	0.14886	0.99959	0.79779	-0.32592	0.058813	0.24012	1.1877	0.24814	0.21859	0.99984	0.82841	-0.27158	0.064491	0.99966	0.88807	-0.17125
693.8393	10.05	domics Pos	0.49745	0.99995	1.0445	0.062879	0.037027	0.99441	1.1969	0.25928	0.29305	0.89498	0.92752	-0.10855	0.26112	0.99996	1.1429	0.19271	0.76865	0.99701	0.97448	-0.03729	0.35814	0.99959	1.1101	0.15074	0.066847	0.25287	1.3004	0.37895	0.87658	0.99984	1.0125	0.017862	0.33521	0.99966	1.0634	0.088627
142.1239	6.38	olomics Pc	0.00117	0.29623	0.84671	-0.24005	0.026428	0.88953	0.7491	-0.41677	0.002001	0.39871	0.83499	-0.26017	0.34963	0.99987	0.92774	-0.10821	0.048099	0.95986	0.88207	-0.18104	0.12563	0.99796	0.81944	-0.28729	0.34518	0.99768	1.0315	0.0447	0.066682	0.9981	0.77971	-0.359	0.034379	0.99995	0.85923	-0.21888
97.1019	2.05	domics Pos	0.001126	0.59581	0.81402	-0.29686	0.044805	0.99441	0.77823	-0.36174	0.007069	0.19062	0.743	-0.42856	0.44655	0.99996	0.92301	-0.11558	0.008038	0.46849	0.83487	-0.26038	0.26507	0.99959	0.83856	-0.25401	0.013722	0.1677	1.2327	0.30178	0.19978	0.99984	0.79655	-0.32816	0.037318	0.99966	0.89714	-0.15659
207.6707	2.76	domics Pos	0.083862	0.99995	0.75351	-0.4083	0.026718	0.99441	0.60549	-0.72383	0.003616	0.15208	0.63569	-0.6536	0.040903	0.99996	0.71036	-0.49338	0.065565	0.76687	0.69337	-0.5283	0.22054	0.99959	0.78608	-0.34725	0.10319	0.30197	1.3056	0.38467	0.1294	0.99984	0.67055	-0.57659	0.15242	0.99966	0.83172	-0.26584
1023.525	12.06	olomics Ne	0.3807	0.9951	1.1293	0.17548	0.3384	0.99975	1.1157	0.15801	0.58963	0.99731	1.0715	0.099602	0.068225	0.99915	1.3084	0.38775	0.3513	0.75078	1.1096	0.1501	0.011285	0.45618	1.2837	0.36031	0.008446	0.059313	1.2867	0.36372	0.208	0.99481	1.128	0.17378	0.36715	0.92651	0.91011	-0.13588
518.9732	5.2	olomics Ne	0.35263	0.9951	1.0519	0.07299	0.54958	0.99975	1.0267	0.038057	0.84901	0.99731	0.99378	-0.009	0.59422	0.99915	1.0351	0.049736	0.074606	0.5958	1.0802	0.11134	0.037362	0.4612	1.1161	0.15847	0.001698	0.041313	1.2889	0.3661	0.26162	0.99481	1.0407	0.05752	0.074811	0.91738	0.92273	-0.11602
107.9675	9.82	domics Pos	0.34338	0.99995	1.0849	0.11759	0.17224	0.99979	1.125	0.16991	0.77107	0.99601	1.0251	0.035777	0.91463	0.99996	1.038	0.053846	0.60736	0.99701	0.9596	-0.0595	0.94543	0.99959	0.99702	-0.00431	0.009864	0.15738	1.2903	0.36766	0.99386	0.99984	1.0111	0.015885	0.21293	0.99966	1.1259	0.17105
393.296	7.64	olomics Pc	0.036472	0.99964	0.85741	-0.22195	0.22882	1	1.068	0.094872	0.65507	0.99882	0.97272	-0.03991	0.098264	0.99987	0.94077	-0.08809	0.40354	0.95986	0.95141	-0.07186	0.23059	0.99796	0.94828	-0.07661	0.60694	0.99768	0.9703	-0.04349	0.56704	0.9981	0.95999	-0.0589	0.59763	0.99995	0.97473	-0.03693
97.1005	1.66	domics Pos	0.087831	0.99995	0.81675	-0.29203	0.058156	0.99653	0.74603	-0.42268	0.0269	0.34652	0.65382	-0.61304	0.16783	0.99996	0.82657	-0.2748	0.017682	0.49574	0.73137	-0.45133	0.16255	0.99959	0.76675	-0.38317	0.39087	0.61308	1.0978	0.13455	0.20818	0.99984	0.8019	-0.31851	0.05878	0.99966	0.81068	-0.3028
398.9777	4.71	olomics Ne	0.40228	0.9951	1.0468	0.065984	0.78316	0.99975	1.0191	0.027294	0.65202	0.99731	0.97941	-0.03001	0.69443	0.99915	0.98676	-0.01922	0.018969	0.50209	1.142	0.19152	0.048457	0.49377	1.107	0.14671	0.006824	0.057338	1.2265	0.2945	0.32937	0.99481	1.0247	0.035182	0.32837	0.91738	0.93678	-0.09422
209.1909	6.38	olomics Pc	0.001517	0.29623	0.84482	-0.24328	0.056993	0.92065	0.77569	-0.36644	0.001428	0.39871	0.82747	-0.27322	0.40429	0.99987	0.93133	-0.10264	0.020132	0.95986	0.8505	-0.23361	0.1717	0.99796	0.83421	-0.26151	0.096453	0.87638	1.0527	0.074141	0.080613	0.9981	0.77564	-0.36655	0.14907	0.99995	0.89068	-0.16702
109.1019	1.67	domics Pos	0.071812	0.99995	0.86883	-0.20286	0.003263	0.99441	0.68807	-0.53936	0.008339	0.19873	0.74037	-0.43369	0.20605	0.99996	0.83038	-0.26816	0.004213	0.46849	0.76415	-0.38808	0.11755	0.99959	0.75119	-0.41275	0.17457	0.38619	1.1877	0.24814	0.075411	0.99984	0.74913	-0.41671	0.028408	0.99966	0.81732	-0.29103
223.2055	6.38	olomics Pc	0.049759	0.99964	0.82124	-0.28413	0.049463	0.90372	0.77629	-0.36533	0.022818	0.71165	0.78804	-0.34366	0.53252	0.99987	0.93344	-0.09936	0.17768	0.95986	0.87487	-0.19286	0.14503	0.99796	0.81789	-0.29003	0.66144	0.99768	1.0395	0.05592	0.096042	0.9981	0.77262	-0.37216	0.048378	0.99995	0.80185	-0.3186
170.1546	2.05	domics Pos	0.003495	0.59581	0.85751	-0.22178	0.041328	0.99441	0.76166	-0.39278	0.001515	0.1313	0.77636	-0.36521	0.40199	0.99996	0.90625	-0.14201	0.015681	0.49216	0.81789	-0.29003	0.14341	0.99959	0.79943	-0.32296	0.093316	0.28973	1.2149	0.28078	0.19658	0.99984	0.81232	-0.29988	0.04979	0.99966	0.90524	-0.14363
246.2304	1.66	domics Pos	0.026567	0.85714	0.87823	-0.18733	0.021143	0.99441	0.7526	-0.41005	0.006254	0.17872	0.75156	-0.41204	0.32431	0.99996	0.88347	-0.17875	0.022197	0.5478	0.80771	-0.3081	0.17759	0.99959	0.78988	-0.3403	0.045446	0.21852	1.2479	0.31949	0.16302	0.99984	0.79167	-0.33703	0.12848	0.99966	0.89241	-0.16422
499.9059	10.02	domics Pos	0.20934	0.99995	1.1379	0.18632	0.91489	0.99979	1.0093	0.013379	0.56453	0.99601	1.0414	0.058476	0.83767	0.99996	1.0189	0.027002	0.98389	0.99701	1.0076	0.010877	0.98928	0.99959	1.0027	0.003896	0.045915	0.21994	1.2354	0.30502	0.89415	0.99984	1.0139	0.019938	0.58642	0.99966	1.0771	0.10716
165.1638	6	olomics Pc	0.003942	0.42508	0.84353	-0.24549	0.024241	0.88953	0.78024	-0.35802	0.00558	0.51418	0.83099	-0.26709	0.62	0.99987	0.95978	-0.05922	0.047379	0.95986	0.8647	-0.20972	0.19816	0.99796	0.84087	-0.25005	0.4835	0.99768	1.0517	0.072727	0.14359	0.9981	0.84156	-0.24886	0.10322	0.99995	0.87312	-0.19576
264.2414	5.99	olomics Pc	0.020985	0.86976	0.87042	-0.20022	0.044612	0.88953	0.78981	-0.34042	0.013041	0.60269	0.83742	-0.25598	0.69014	0.99987	0.97125	-0.04208	0.033269	0.95986	0.88788	-0.17156	0.1912	0.99796	0.8254	-0.27684	0.37853	0.99768	1.0461	0.065037	0.1096	0.9981	0.80287	-0.31676	0.088234	0.99995	0.87144	-0.19853
153.1286	6.38	olomics Pc	0.050466	0.99964	0.8836	-0.17853	0.007477	0.88953	0.76207	-0.39201	0.039435	0.90672	0.88289	-0.17969	0.52224	0.99987	0.9373	-0.09342	0.47119	0.95986	0.94416	-0.08289	0.070523	0.99796	0.79095	-0.33834	0.7365	0.99768	1.0173	0.024783	0.083717	0.9981	0.81599	-0.29338	0.097849	0.99995	0.82995	-0.26891
226.2176	6.38	olomics Pc	0.007695	0.60235	0.8409	-0.24999	0.04388	0.88953	0.765	-0.38647	0.011664	0.59292	0.8298	-0.26917	0.3968	0.99987	0.9313	-0.10269	0.059976	0.95986	0.87786	-0.18794	0.11199	0.99796	0.80839	-0.30687	0.31126	0.99768	1.0394	0.055789	0.055442	0.9981	0.77138	-0.37448	0.03411	0.99995	0.85039	-0.23381
210.1862	5.99	olomics Pc	0.020061	0.86976	0.81189	-0.30065	0.15286	1	0.79455	-0.33178	0.057423	0.99882	0.83289	-0.2638	0.68531	0.99987	0.96163	-0.05644	0.042593	0.95986	0.83769	-0.25551	0.093443	0.99796	0.77665	-0.36466	0.5311	0.99768	1.0513	0.072139	0.12978	0.9981	0.79087	-0.33848	0.1481	0.99995	0.84998	-0.2345
156.1394	5.99	olomics Pc	0.089361	0.99964	0.89893	-0.15372	0.06617	0.98785	0.80797	-0.30763	0.039988	0.91278	0.85401	-0.22768	0.68832	0.99987	0.97085	-0.04267	0.26711	0.95986	0.92243	-0.11649	0.20154	0.99796	0.84987	-0.23469	0.26695	0.99768	1.041	0.057991	0.068545	0.9981	0.79778	-0.32593	0.085485	0.99995	0.91021	-0.13573
284.2862	2.05	domics Pos	0.002358	0.59581	0.86763	-0.20484	0.044422	0.99441	0.75761	-0.40047	0.001499	0.1313	0.7632	-0.38987	0.30136	0.99996	0.88986	-0.16835	0.007223	0.46849	0.8091	-0.30561	0.12124	0.99959	0.78909	-0.34175	0.11508	0.31869	1.1836	0.24317	0.15998	0.99984	0.7942	-0.33242	0.036578	0.99966	0.88768	-0.17188
248.2465	6.38	olomics Pc	0.002964	0.38271	0.83816	-0.2547	0.023092	0.88953	0.75764	-0.40042	0.006936	0.51418	0.84679	-0.23993	0.337	0.99987	0.93002	-0.10466	0.04389	0.95986	0.89505	-0.15996	0.11001	0.99796	0.79207	-0.3363	0.27009	0.99768	1.0473	0.066611	0.070933	0.9981	0.7829	-0.35309	0.03377	0.99995	0.85343	-0.22866
1196.714	10.07	domics Pos	0.013768	0.68962	1.1917	0.25297	0.78434	0.99979	1.0346	0.049128	0.44357	0.98465	1.0839	0.11619	0.13631	0.99996	1.0768	0.1068	0.38764	0.99701	0.91217	-0.13262	0.78412	0.99959	1.0275	0.039196	0.022855	0.18641	1.2057	0.26993	0.013268	0.99984	1.1767	0.23476	0.20682	0.99966	1.1448	0.19508
95.0863	2.05	domics Pos	0.000225	0.51602	0.85117	-0.23249	0.024101	0.99441	0.73616	-0.4419	0.003382	0.15208	0.7858	-0.34777	0.32738	0.99996	0.8943	-0.16117	0.015847	0.49216	0.82793	-0.27243	0.11103	0.99959	0.7905	-0.33917	0.077201	0.26567	1.2393	0.30954	0.11645	0.99984	0.75637	-0.40284	0.01044	0.99749	0.88541	-0.17558
390.7631	0.31	olomics Pc	0.75609	0.99964	1.0401	0.056675	0.14849	1	1.1698	0.22624	0.58306	0.99882	1.036	0.050998	0.041797	0.99987	1.1608	0.21507	0.47854	0.95986	1.0817	0.11333	0.36655	0.99796	1.1572	0.21064	0.52774	0.99768	0.92777	-0.10816	0.33307	0.9981	1.0877	0.12127	0.41832	0.99995	1.0705	0.098268
443.9783	4.99	olomics Ne	0.2828	0.9951	1.0904	0.12484	0.81887	0.99975	1.02	0.028621	0.76446	0.99731	0.9751	-0.03638	0.14377	0.99915	1.105	0.14403	0.14651	0.64314	1.1197	0.16316	0.064258	0.517	1.171	0.2278	0.023683	0.082823	1.2888	0.36605	0.35176	0.99481	1.0914	0.12613	0.93038	0.99833	0.99162	-0.01214
210.1928	6.38	olomics Pc	0.027471	0.9464	0.81474	-0.29559	0.012435	0.88953	0.7376	-0.43909	0.11748	0.99882	0.85394	-0.22779	0.37904	0.99987	0.94834	-0.07653	0.20212	0.95986	0.8782	-0.18738	0.22388	0.99796	0.8584	-0.22029	0.46071	0.99768	1.0632	0.088373	0.065994	0.9981	0.7909	-0.33844	0.088185	0.99995	0.86131	-0.2154
267.2602	6.38	olomics Pc	0.26466	0.99964	0.93308	-0.09993	0.026026	0.88953	0.72272	-0.46849	0.049206	0.99882	0.83027	-0.26835	0.53945	0.99987	0.94415	-0.08292	0.19974	0.95986	0.88569	-0.17513	0.13415	0.99796	0.80043	-0.32115	0.51351	0.99768	1.049	0.069029	0.083819	0.9981	0.76206	-0.39202	0.014953	0.99995	0.81091	-0.30238
137.1336	6.39	olomics Pc	0.022647	0.88846	0.92053	-0.11946	0.014805	0.88953	0.79404	-0.33272	0.049334	0.99882	0.93278	-0.1004	0.689	0.99987	0.97235	-0.04045	0.4706	0.95986	0.95613	-0.06471	0.12604	0.99796	0.81125	-0.30178	0.13088	0.94474	1.1047	0.14363	0.10795	0.9981	0.85193	-0.23119	0.3667	0.99995	0.90941	-0.137
330.905	10.15	domics Pos	0.17216	0.99995	1.1397	0.18861	0.36185	0.99979	1.1042	0.14301	0.49269	0.99601	0.9536	-0.06854	0.83874	0.99996	0.99093	-0.01314	0.74752	0.99701	0.97969	-0.0296	0.86713	0.99959	0.98888	-0.01613	0.029052	0.19804	1.2901	0.36751	0.28196	0.99984	1.0899	0.12425	0.081999	0.99966	1.1495	0.20097
332.2929	7.02	olomics Pc	0.13915	0.99964	0.85593	-0.22443	0.044986	0.88953	0.7203	-0.47333	0.015923	0.63482	0.78441	-0.35032	0.05186	0.99987	0.83762	-0.25563	0.24957	0.95986	0.89175	-0.16529	0.030372	0.99796	0.68632	-0.54304	0.73347	0.99768	1.0369	0.052221	0.08505	0.9981	0.71761	-0.47873	0.0729	0.99995	0.82048	-0.28546
609.8811	10.05	domics Pos	0.87536	0.99995	0.98878	-0.01629	0.041701	0.99441	1.2159	0.28206	0.8256	0.99702	0.98206	-0.02611	0.51677	0.99996	1.0655	0.091482	0.60663	0.99701	1.0524	0.073666	0.34494	0.99959	1.119	0.16224	0.13209	0.33512	1.3338	0.41559	0.94505	0.99984	1.0204	0.029144	0.30433	0.99966	1.1086	0.14868
93.0712	6.01	olomics Pc	0.45855	0.99964	0.94005	-0.08919	0.018997	0.88953	0.79063	-0.33893	0.042587	0.93262	0.86364	-0.2115	0.5603	0.99987	1.1136	0.15526	0.090511	0.95986	0.84246	-0.24732	0.10529	0.99796	0.7998	-0.32229	0.93653	0.99768	1.0086	0.012347	0.13171	0.9981	0.80487	-0.31317	0.35739	0.99995	0.91472	-0.1286
245.2274	5.99	olomics Pc	0.01571	0.86976	0.86391	-0.21105	0.046153	0.88953	0.78504	-0.34916	0.004717	0.48835	0.83482	-0.26046	0.61289	0.99987	0.9642	-0.0526	0.039907	0.95986	0.89175	-0.16529	0.17584	0.99796	0.84393	-0.2448	0.33958	0.99768	1.04	0.056594	0.07648	0.9981	0.79861	-0.32443	0.04058	0.99995	0.88593	-0.17473
286.8436	0.31	olomics Ne	0.12634	0.9951	1.126	0.17115	0.16316	0.99975	1.14	0.18904	0.10688	0.99731	1.1103	0.151	0.52453	0.99915	1.0504	0.070969	0.013577	0.50209	1.2447	0.3158	0.016951	0.45618	1.2138	0.27955	0.006744	0.057338	1.257	0.33001	0.17099	0.99481	1.155	0.20787	0.092132	0.91738	1.1101	0.15063
254.2482	2.76	domics Pos	0.49971	0.99995	0.95799	-0.06191	0.023228	0.99441	0.70919	-0.49576	0.000161	0.13107	0.81527	-0.29465	0.44846	0.99996	0.90832	-0.13872	0.039654	0.64262	0.89196	-0.16495	0.092477	0.99959	0.71057	-0.49295	0.2641	0.48137	1.1688	0.22501	0.17679	0.99984	0.8162	-0.293	0.082371	0.99966	0.85407	-0.22757
284.2867	6.38	olomics Pc	0.001397	0.29623	0.83328	-0.26313	0.042427	0.88953	0.77432	-0.369	0.009227	0.55189	0.82704	-0.27397	0.42806	0.99987	0.94216	-0.08596	0.025464	0.95986	0.88105	-0.18271	0.13615	0.99796	0.81643	-0.2926	0.11353	0.9217	1.0695	0.096878	0.079796	0.9981	0.78783	-0.34405	0.059281	0.99995	0.87047	-0.20013
547.2928	0.53	domics Pos	0.76393	0.99995	1.0817	0.11325	0.17252	0.99979	0.83445	-0.26111	0.014099	0.26097	0.70322	-0.50795	0.77821	0.99996	1.1038	0.14246	0.096466	0.82209	0.70383	-0.50671	0.53652	0.99959	0.90825	-0.13883	0.61099	0.77553	1.53	0.61356	0.82297	0.99984	1.0004	0.000632	0.90926	0.99966	1.0678	0.09459
712.4923	2.05	domics Pos	0.20027	0.99995	0.89094	-0.1666	0.32778	0.99979	0.86061	-0.21656	0.21408	0.83418	0.81318	-0.29836	0.23195	0.99996	0.83081	-0.26741	0.37785	0.99701	0.89508	-0.15991	0.36352	0.99959	0.84829	-0.23737	0.047103	0.22017	1.3644	0.44829	0.18532	0.99984	0.77627	-0.36536	0.38089	0.99966	0.92812	-0.10762
330.9796	12	olomics Ne	0.87038	0.9951	1.013	0.018692	0.73405	0.99975	1.0317	0.045068	0.38632	0.99731	0.93878	-0.09114	0.71033	0.99915	1.0241	0.034384	0.24562	0.71555	1.0775	0.10766	0.44051	0.7741	1.0646	0.090289	0.020624	0.076566	1.1838	0.24345	0.92787	0.99481	1.0077	0.011126	0.20727	0.91738	0.91763	-0.12401
1016.797	10.02	domics Pos	0.15351	0.99995	1.2443	0.31532	0.22371	0.99979	1.1753	0.23306	0.70314	0.99601	1.0248	0.035307	0.67	0.99996	1.0543	0.076234	0.6095	0.99701	1.0765	0.10641	0.10556	0.99959	1.1603	0.21446	0.012494	0.16313	1.4893	0.57462	0.43271	0.99984	1.0988	0.13595	0.28937	0.99966	1.1089	0.14916
134.0196	0.31	olomics Pc	0.31473	0.99964	1.2141	0.27993	0.21838	1	1.1178	0.16061	0.66274	0.99882	1.0383	0.054217	0.61508	0.99987	1.0455	0.064258	0.83952	0.99631	1.056	0.078586	0.019408	0.99796	1.5101	0.59461	0.001405	0.27616	1.9938	0.99549	0.15315	0.9981	0.8036	-0.31544	0.027812	0.99995	0.71056	-0.49296
700.3812	0.56	domics Pos	0.87246	0.99995	1.0312	0.044275	0.40896	0.99979	0.85044	-0.23372	0.045995	0.46561	0.65306	-0.61471	0.69791	0.99996	1.0591	0.082771	0.046796	0.68893	0.59179	-0.75684	0.29634	0.99959	0.81046	-0.30319	0.80032	0.88401	1.3085	0.38792	0.65795	0.99984	0.91875	-0.12225	0.77538	0.99966	0.98258	-0.02535
451.8806	6.37	olomics Pc	0.000926	0.29623	0.64615	-0.63006	0.072485	0.99372	0.67603	-0.56483	0.009532	0.55189	0.6231	-0.68246	0.22631	0.99987	0.82365	-0.2799	0.13348	0.95986	0.70589	-0.50248	0.074855	0.99796	0.61893	-0.69214	0.65261	0.99768	0.95574	-0.06532	0.034551	0.9981	0.53929	-0.89086	0.013592	0.99995	0.69963	-0.51534
457.9816	4.85	olomics Ne	0.73442	0.9951	1.0226	0.032175	0.34466	0.99975	1.0331	0.047027	0.90993	0.99731	1.0156	0.022313	0.019555	0.99915	1.0847	0.11735	0.21279	0.70038	1.1253	0.17031	0.19669	0.63384	1.0984	0.13536	1.81E-05	0.011118	1.3548	0.43806	0.16343	0.99481	1.1082	0.14822	0.29331	0.91738	0.91225	-0.1325
128.1072	2.76	domics Pos	0.05712	0.99995	0.8434	-0.24571	0.005438	0.99441	0.69023	-0.53485	0.009993	0.2165	0.70181	-0.51084	0.10609	0.99996	0.80151	-0.31921	0.001271	0.46849	0.76652	-0.3836	0.12753	0.99959	0.7679	-0.38101	0.16552	0.37482	1.1574	0.21093	0.18388	0.99984	0.78967	-0.34067	0.079708	0.99966	0.8495	-0.23531
444.5655	0.54	domics Pos	0.61122	0.99995	0.93817	-0.09208	0.038179	0.99441	0.77606	-0.36576	0.007035	0.19062	0.64594	-0.63053	0.57142	0.99996	1.0146	0.020896	0.027942	0.55491	0.62989	-0.66684	0.18117	0.99959	0.8198	-0.28666	0.75458	0.85759	1.3168	0.39706	0.23829	0.99984	0.79307	-0.33449	0.73645	0.99966	1.0024	0.003414
243.2111	1.39	domics Pos	0.7051	0.99995	0.96806	-0.04683	0.054676	0.99441	0.79337	-0.33393	0.01141	0.23141	0.81874	-0.28853	0.64922	0.99996	0.95349	-0.06872	0.078921	0.80167	0.84861	-0.23682	0.30487	0.99959	0.85231	-0.23055	0.15059	0.35887	1.2173	0.28372	0.38091	0.99984	0.87009	-0.20076	0.11942	0.99966	0.8245	-0.2784
313.3171	2.75	domics Pos	0.18059	0.99995	0.87558	-0.19168	0.039378	0.99441	0.70151	-0.51147	0.10691	0.68915	0.83987	-0.25176	0.24341	0.99996	0.83996	-0.25162	0.39215	0.99701	0.90163	-0.1494	0.045648	0.99959	0.6907	-0.53386	0.20806	0.42333	1.2065	0.27088	0.32994	0.99984	0.83191	-0.2655	0.033934	0.99966	0.77182	-0.37366
968.5804	9.94	olomics Ne	0.8108	0.9951	1.0483	0.067988	0.44885	0.99975	0.94829	-0.0766	0.57706	0.99731	0.94255	-0.08536	0.56497	0.99915	1.0374	0.052949	0.27264	0.71896	1.0741	0.10319	0.3158	0.70329	1.0768	0.10678	0.01533	0.067031	1.2288	0.29722	0.64565	0.99481	0.96341	-0.05378	0.12126	0.91738	0.86134	-0.21535
135.1181	6.38	olomics Pc	0.00784	0.60235	0.91643	-0.1259	0.013443	0.88953	0.78246	-0.3539	0.003105	0.43007	0.89385	-0.16189	0.72722	0.99987	0.97817	-0.03184	0.41033	0.95986	0.96777	-0.04727	0.091487	0.99796	0.81361	-0.29759	0.038883	0.67297	1.0884	0.12227	0.099684	0.9981	0.83881	-0.25358	0.078106	0.99995	0.87504	-0.19257
263.238	5.99	olomics Pc	0.0372	0.99964	0.87102	-0.19922	0.045877	0.88953	0.77987	-0.35869	0.013258	0.60269	0.8355	-0.25929	0.60172	0.99987	0.95844	-0.06124	0.11383	0.95986	0.90416	-0.14535	0.1692	0.99796	0.82798	-0.27233	0.3363	0.99768	1.0435	0.061376	0.093694	0.9981	0.80265	-0.31716	0.064335	0.99995	0.87464	-0.19325
807.9698	3.29	olomics Pc	0.78799	0.99964	1.0927	0.12794	0.37327	1	0.90275	-0.14761	0.037014	0.89885	0.75903	-0.39777	0.95693	0.99987	1.0733	0.10205	0.34134	0.95986	0.83524	-0.25973	0.98794	0.99873	1.0578	0.081112	0.56482	0.99768	1.3896	0.4747	0.83104	0.9981	1.0002	0.000229	0.6089	0.99995	0.95161	-0.07155
752.5575	9.29	olomics Pc	0.66122	0.99964	1.0261	0.037132	0.36753	1	0.94195	-0.08628	0.45663	0.99882	0.92559	-0.11155	0.82279	0.99987	1.0107	0.015374	0.18214	0.95986	1.181	0.23996	0.36061	0.99796	1.155	0.20787	0.037535	0.66991	1.2683	0.34295	0.10324	0.9981	1.2948	0.37268	0.29074	0.99995	1.2528	0.32516
139.113	5.99	olomics Pc	0.020914	0.86976	0.81242	-0.29971	0.095358	1	0.81656	-0.29238	0.061533	0.99882	0.83585	-0.25869	0.84392	0.99987	1.0293	0.041666	0.12586	0.95986	0.88953	-0.16889	0.12687	0.99796	0.79914	-0.32348	0.80029	0.99768	1.0336	0.047681	0.10095	0.9981	0.82939	-0.26988	0.4186	0.99995	0.9317	-0.10206
499.9054	0.5	domics Pos	0.4134	0.99995	1.0579	0.081164	0.26296	0.99979	1.1208	0.16449	0.25947	0.87787	0.92915	-0.10602	0.85866	0.99996	1.0194	0.027743	0.56225	0.99701	0.95697	-0.06345	0.88736	0.99959	0.99254	-0.01081	0.013477	0.16631	1.267	0.34145	0.6865	0.99984	0.96698	-0.04844	0.97726	0.99966	1.0042	0.006056
612.5422	5.66	domics Pos	0.5274	0.99995	1.063	0.088175	0.52379	0.99979	1.0862	0.11926	0.43606	0.98176	0.9161	-0.12643	0.82573	0.99996	0.97929	-0.03019	0.96623	0.99701	1.004	0.005829	0.14207	0.99959	1.1527	0.20506	0.032383	0.20029	1.5772	0.65734	0.22081	0.99984	1.1464	0.19705	0.091794	0.99966	1.2125	0.27798
121.1019	2.05	domics Pos	0.01088	0.68962	0.85501	-0.22598	0.072862	0.99979	0.79593	-0.32929	0.004994	0.16853	0.74631	-0.42215	0.27355	0.99996	0.88068	-0.1833	0.02792	0.55491	0.80305	-0.31643	0.10613	0.99959	0.78579	-0.34779	0.097878	0.29456	1.2012	0.26446	0.19898	0.99984	0.79683	-0.32766	0.15089	0.99966	0.91468	-0.12866
129.1178	2.05	domics Pos	0.020667	0.796	0.79122	-0.33784	0.12698	0.99979	0.79638	-0.32848	0.015606	0.27806	0.76106	-0.39391	0.094645	0.99996	0.74266	-0.42923	0.004304	0.46849	0.73547	-0.44326	0.09361	0.99959	0.74439	-0.42587	0.15257	0.36054	1.1532	0.20561	0.076341	0.99984	0.72082	-0.47229	0.012681	0.99749	0.79887	-0.32397
333.2944	2.75	domics Pos	0.1538	0.99995	0.81167	-0.30103	0.058197	0.99653	0.64903	-0.62364	0.011177	0.22917	0.62819	-0.67074	0.60548	0.99996	0.93928	-0.09038	0.098253	0.82209	0.69628	-0.52226	0.030904	0.99959	0.62199	-0.68504	0.97	0.98396	1.0122	0.017436	0.079109	0.99984	0.70259	-0.50924	0.012662	0.99749	0.66477	-0.58907
153.128	2.05	domics Pos	0.007748	0.68962	0.86049	-0.21677	0.027017	0.99441	0.75724	-0.40118	0.002138	0.13479	0.74693	-0.42096	0.40034	0.99996	0.90149	-0.14962	0.010651	0.46849	0.78323	-0.3525	0.10382	0.99959	0.78597	-0.34745	0.053687	0.23183	1.2337	0.30296	0.17104	0.99984	0.80636	-0.31051	0.056323	0.99966	0.86855	-0.20332
278.2452	6.2	olomics Pc	0.08826	0.99964	0.79774	-0.32601	0.037803	0.88953	0.73088	-0.45229	0.012509	0.60269	0.68965	-0.53607	0.97205	0.99987	1.0325	0.046134	0.24975	0.95986	0.82427	-0.27881	0.10326	0.99796	0.75477	-0.40588	0.54203	0.99768	1.0621	0.086956	0.027735	0.9981	0.64159	-0.64029	0.1229	0.99995	0.82989	-0.26901
124.1218	1.66	domics Pos	0.011987	0.68962	0.79722	-0.32696	0.003602	0.99441	0.7329	-0.44831	0.000327	0.13107	0.68225	-0.55162	0.18552	0.99996	0.78656	-0.34637	0.074097	0.7908	0.78176	-0.35521	0.11331	0.99959	0.77693	-0.36414	0.17542	0.38686	1.1027	0.14099	0.12532	0.99984	0.77534	-0.36709	0.43996	0.99966	0.94137	-0.08716
340.3577	8.4	olomics Pc	0.90337	0.99964	0.98893	-0.01606	0.12305	1	1.1533	0.20583	0.80974	0.99882	1.0406	0.057355	0.026852	0.99987	1.1972	0.25962	0.46116	0.95986	1.0862	0.11929	0.099266	0.99796	1.1452	0.19565	0.9616	0.99768	0.99963	-0.00053	0.84136	0.9981	1.0262	0.037381	0.32336	0.99995	1.0906	0.1251
389.1943	4.11	olomics Pc	0.012023	0.74258	0.60906	-0.71534	0.001504	0.88953	0.63602	-0.65286	0.28326	0.99882	0.87922	-0.1857	0.24405	0.99987	0.80336	-0.31588	0.00071	0.95986	0.52075	-0.94132	0.020639	0.99796	0.68951	-0.53635	0.79489	0.99768	1.051	0.071711	0.1697	0.9981	0.76713	-0.38245	0.00019	0.29995	0.66783	-0.58245
223.2058	2.05	domics Pos	0.015916	0.68962	0.77669	-0.36459	0.067496	0.99979	0.73346	-0.44721	0.01838	0.29983	0.74413	-0.42638	0.48627	0.99996	0.91867	-0.12238	0.019103	0.52048	0.73241	-0.44929	0.040966	0.99959	0.69597	-0.52291	0.28187	0.50294	1.1144	0.1563	0.078109	0.99984	0.71185	-0.49035	0.077502	0.99966	0.8423	-0.2476
897.9975	0.54	domics Pos	0.77344	0.99995	0.96043	-0.05824	0.28867	0.99979	0.85071	-0.23326	0.018855	0.30022	0.65583	-0.6086	0.68074	0.99996	1.0272	0.038784	0.03289	0.59375	0.59546	-0.74791	0.27698	0.99959	0.7884	-0.34301	0.80093	0.8842	1.3456	0.42828	0.6589	0.99984	0.93039	-0.10409	0.85876	0.99966	1.0213	0.030417
518.7497	9.92	domics Pos	0.28921	0.99995	0.87436	-0.19369	0.7048	0.99979	0.95	-0.074	0.013347	0.25194	0.70355	-0.50727	0.061056	0.99996	0.78862	-0.3426	0.036065	0.62141	0.75559	-0.40432	0.53474	0.99959	0.91741	-0.12436	0.94482	0.9741	1.022	0.031353	0.18435	0.99984	0.83238	-0.26469	0.55309	0.99966	0.91396	-0.12979
283.2837	6.38	olomics Pc	0.001346	0.29623	0.86291	-0.21272	0.051553	0.90372	0.78684	-0.34586	0.008732	0.55189	0.84565	-0.24186	0.39452	0.99987	0.94	-0.08927	0.037423	0.95986	0.90237	-0.14821	0.14573	0.99796	0.82639	-0.27511	0.092943	0.87442	1.0655	0.091534	0.087116	0.9981	0.8031	-0.31634	0.053963	0.99995	0.8738	-0.19462
143.1298	2.05	domics Pos	0.002555	0.59581	0.84452	-0.24379	0.035846	0.99441	0.71993	-0.47407	0.00523	0.16899	0.69655	-0.5217	0.19155	0.99996	0.83536	-0.25953	0.003164	0.46849	0.7683	-0.38026	0.085961	0.99959	0.76198	-0.39218	0.20789	0.42321	1.1412	0.19053	0.24357	0.99984	0.82502	-0.2775	0.091443	0.99966	0.84981	-0.23478
195.1484	2.76	domics Pos	0.078155	0.99995	0.81496	-0.29519	0.082026	0.99979	0.73158	-0.4509	0.032382	0.38827	0.78399	-0.35109	0.32681	0.99996	0.88448	-0.17709	0.004811	0.46849	0.80651	-0.31024	0.058395	0.99959	0.71522	-0.48355	0.3165	0.53803	1.1133	0.15484	0.069908	0.99984	0.75721	-0.40123	0.001745	0.99749	0.81058	-0.30298
249.2497	2.05	domics Pos	0.078734	0.99995	0.82179	-0.28315	0.023108	0.99441	0.71801	-0.47793	0.007099	0.19062	0.73093	-0.4522	0.45379	0.99996	0.90205	-0.14873	0.082924	0.80455	0.80719	-0.30901	0.08808	0.99959	0.78839	-0.34302	0.31503	0.53714	1.1148	0.15676	0.16566	0.99984	0.78443	-0.35029	0.035708	0.99966	0.82195	-0.28287
177.1649	6.38	olomics Pc	0.011075	0.71196	0.87337	-0.19533	0.036638	0.88953	0.80351	-0.31561	0.006268	0.51418	0.84559	-0.24198	0.65638	0.99987	0.97084	-0.04269	0.17125	0.95986	0.93283	-0.10031	0.10345	0.99796	0.80903	-0.30574	0.28135	0.99768	1.053	0.074464	0.066916	0.9981	0.8264	-0.27508	0.14534	0.99995	0.88763	-0.17196
198.1858	2.05	domics Pos	0.012296	0.68962	0.87181	-0.19791	0.030096	0.99441	0.74918	-0.41662	0.003904	0.15348	0.77935	-0.35966	0.32555	0.99996	0.89369	-0.16216	0.024322	0.5516	0.81997	-0.28636	0.1583	0.99959	0.8017	-0.31886	0.089953	0.28541	1.2099	0.27484	0.16449	0.99984	0.8018	-0.31868	0.088168	0.99966	0.88367	-0.17842
265.2538	6.38	olomics Pc	0.001556	0.29623	0.84811	-0.23767	0.039386	0.88953	0.77969	-0.35902	0.014019	0.60587	0.83613	-0.25819	0.37139	0.99987	0.93836	-0.09179	0.039171	0.95986	0.89546	-0.1593	0.1164	0.99796	0.81706	-0.29148	0.065715	0.81416	1.0668	0.093341	0.10922	0.9981	0.80927	-0.3053	0.048227	0.99995	0.86353	-0.21168
151.1474	1.67	domics Pos	0.067093	0.99995	0.88329	-0.17905	0.041832	0.99441	0.75501	-0.40544	0.022073	0.32086	0.78439	-0.35036	0.22698	0.99996	0.83757	-0.25572	0.026966	0.55437	0.84152	-0.24894	0.11982	0.99959	0.7588	-0.39822	0.089486	0.28458	1.2521	0.32432	0.19773	0.99984	0.79245	-0.33561	0.33318	0.99966	0.92462	-0.11307
296.2951	2.41	domics Pos	0.049726	0.99995	0.83482	-0.26047	0.023649	0.99441	0.68585	-0.54404	0.001704	0.13302	0.72354	-0.46686	0.23704	0.99996	0.87213	-0.19738	0.009233	0.46849	0.76932	-0.37835	0.097791	0.99959	0.7366	-0.44105	0.18103	0.39332	1.2204	0.28736	0.2225	0.99984	0.7966	-0.32807	0.04736	0.99966	0.86002	-0.21756
350.2507	2.06	domics Pos	0.99932	0.99995	0.99879	-0.00174	0.56221	0.99979	0.95341	-0.06883	0.015809	0.27907	0.82079	-0.28492	0.81944	0.99996	0.98571	-0.02076	0.92568	0.99701	0.99564	-0.0063	0.78719	0.99959	0.98136	-0.02715	0.042108	0.21126	1.2343	0.30371	0.68347	0.99984	0.96272	-0.05481	0.2166	0.99966	1.0779	0.10828
499.9798	12.01	olomics Ne	0.34225	0.9951	1.0791	0.10988	0.18013	0.99975	1.2796	0.35566	0.44822	0.99731	1.1681	0.22421	0.72832	0.99915	1.0536	0.075388	0.94039	0.99084	1.0113	0.016248	0.38501	0.76125	0.92609	-0.11077	0.004002	0.050041	1.3221	0.40282	0.4456	0.99481	1.3167	0.3969	0.88777	0.99423	1.0338	0.047944
340.3582	8.13	olomics Pc	0.1539	0.99964	1.146	0.19661	0.27255	1	1.0854	0.11827	0.3938	0.99882	1.0735	0.10236	0.006579	0.99987	1.2774	0.3532	0.20065	0.95986	1.0861	0.11918	0.11358	0.99796	1.2002	0.26324	0.12677	0.94474	1.1803	0.23914	0.47159	0.9981	1.1218	0.16575	0.13312	0.99995	1.1267	0.17212
334.9153	10.18	domics Pos	0.25665	0.99995	1.0963	0.13267	0.98782	0.99979	1.0107	0.015308	0.37117	0.93383	1.051	0.071722	0.883	0.99996	1.0388	0.054885	0.75249	0.99701	1.0426	0.060214	0.96855	0.99959	1.006	0.008673	0.014939	0.17068	1.2856	0.36248	0.4793	0.99984	1.0941	0.12971	0.32125	0.99966	1.1091	0.14939
220.2145	1.46	domics Pos	0.038517	0.96211	0.82195	-0.28288	0.11509	0.99979	0.83231	-0.2648	0.004281	0.15847	0.66655	-0.58522	0.50119	0.99996	0.92027	-0.11988	0.036358	0.62141	0.83755	-0.25575	0.41266	0.99959	0.91343	-0.13064	0.15329	0.36152	1.2683	0.34293	0.33911	0.99984	0.84856	-0.23691	0.93496	0.99966	0.99347	-0.00945
282.2808	6.38	olomics Pc	0.00156	0.29623	0.86395	-0.21097	0.039464	0.88953	0.77354	-0.37044	0.007524	0.52669	0.84567	-0.24183	0.42684	0.99987	0.94417	-0.08288	0.046033	0.95986	0.89958	-0.15267	0.13247	0.99796	0.81995	-0.28639	0.056871	0.77855	1.0587	0.082351	0.088467	0.9981	0.80182	-0.31865	0.05204	0.99995	0.87052	-0.20005
237.2186	5.68	olomics Pc	0.000509	0.29623	0.78651	-0.34647	0.042904	0.88953	0.7568	-0.40201	0.051527	0.99882	0.85435	-0.2271	0.68396	0.99987	0.97165	-0.04149	0.029579	0.95986	0.86587	-0.20778	0.36476	0.99796	0.87762	-0.18833	0.19714	0.99768	1.0412	0.058258	0.17245	0.9981	0.82795	-0.27239	0.26919	0.99995	0.92348	-0.11484
93.0702	2.05	domics Pos	0.14837	0.99995	0.83206	-0.26524	0.026548	0.99441	0.66738	-0.58342	0.036858	0.41952	0.70372	-0.50694	0.37173	0.99996	0.88811	-0.1712	0.12485	0.86372	0.82505	-0.27745	0.31865	0.99959	0.82273	-0.28151	0.05815	0.23937	1.2593	0.33267	0.28855	0.99984	0.80602	-0.31112	0.33076	0.99966	0.88587	-0.17484
175.1492	6	olomics Pc	0.023198	0.89113	0.90048	-0.15123	0.13422	1	0.85122	-0.2324	0.009114	0.55189	0.82762	-0.27296	0.96733	0.99987	1.0267	0.038073	0.1973	0.95986	0.91801	-0.12342	0.073476	0.99796	0.79091	-0.33841	0.11513	0.92707	1.1273	0.17292	0.22832	0.9981	0.84505	-0.2429	0.27026	0.99995	0.9333	-0.09958
185.1736	2.05	domics Pos	0.007592	0.68962	0.89225	-0.16448	0.14986	0.99979	0.82035	-0.2857	0.035111	0.40571	0.74247	-0.42959	0.51328	0.99996	0.93674	-0.09428	0.041061	0.65175	0.8078	-0.30792	0.030456	0.99959	0.72094	-0.47205	0.18885	0.40253	1.1562	0.20939	0.14069	0.99984	0.80208	-0.31819	0.027694	0.99966	0.86707	-0.20579
193.6557	6.38	olomics Pc	0.044461	0.99964	0.82092	-0.28469	0.066964	0.99187	0.8112	-0.30187	0.04927	0.99882	0.82206	-0.28268	0.41994	0.99987	0.91538	-0.12755	0.083014	0.95986	0.8555	-0.22515	0.2347	0.99796	0.88297	-0.17957	0.47251	0.99768	0.93579	-0.09575	0.053872	0.9981	0.76395	-0.38844	0.21206	0.99995	0.89563	-0.15902
604.2896	1.11	domics Pos	0.73267	0.99995	1.0363	0.051452	0.87371	0.99979	0.98066	-0.02817	0.068904	0.5787	0.76303	-0.39019	0.9405	0.99996	1.0051	0.007409	0.70654	0.99701	0.95787	-0.0621	0.03692	0.99959	1.3261	0.4072	0.009223	0.15224	1.3792	0.46388	0.60078	0.99984	1.0403	0.05705	0.088425	0.99966	1.2436	0.31449
246.2308	5.99	olomics Pc	0.038924	0.99964	0.86794	-0.20433	0.073135	0.99372	0.78348	-0.35203	0.054103	0.99882	0.84473	-0.24344	0.63799	0.99987	0.959	-0.0604	0.21713	0.95986	0.92132	-0.11822	0.24923	0.99796	0.84514	-0.24273	0.33334	0.99768	1.059	0.082667	0.19831	0.9981	0.83453	-0.26097	0.1435	0.99995	0.88771	-0.17184
262.858	12.72	olomics Ne	0.35502	0.9951	1.0671	0.093722	0.57239	0.99975	1.0262	0.03725	0.91785	0.99731	1.0063	0.009042	0.36884	0.99915	1.042	0.059317	0.052521	0.58152	1.0985	0.1355	0.45567	0.78526	1.0544	0.076445	0.000627	0.041313	1.2588	0.3321	0.015312	0.99481	1.1093	0.1497	0.15664	0.91738	0.93113	-0.10295
404.9771	4.79	olomics Ne	0.75024	0.9951	1.0134	0.019207	0.27583	0.99975	1.0556	0.07813	0.38202	0.99731	0.97366	-0.0385	0.3712	0.99915	1.0622	0.087048	0.079289	0.5958	1.0735	0.10232	0.090546	0.52866	1.1	0.13752	0.003897	0.050041	1.2378	0.30783	0.3817	0.99481	1.0373	0.052801	0.32479	0.91738	0.95823	-0.06156
203.18	1.66	domics Pos	0.061625	0.99995	0.82132	-0.28399	0.020428	0.99441	0.72044	-0.47305	0.011179	0.22917	0.68483	-0.54618	0.50773	0.99996	0.91778	-0.12377	0.0476	0.69275	0.81085	-0.3025	0.16948	0.99959	0.76871	-0.37949	0.20834	0.42346	1.1832	0.24264	0.31367	0.99984	0.84676	-0.23998	0.28433	0.99966	0.90009	-0.15186
201.644	2.06	domics Pos	0.002524	0.59581	0.94764	-0.07759	0.082554	0.99979	0.82979	-0.26918	0.00101	0.13107	0.83888	-0.25346	0.17102	0.99996	0.89997	-0.15205	0.003464	0.46849	0.86337	-0.21195	0.17298	0.99959	0.85961	-0.21824	0.02382	0.18728	1.1713	0.2281	0.32797	0.99984	0.89012	-0.16792	0.20033	0.99966	0.93111	-0.10298
240.2331	7.02	olomics Pc	0.018165	0.86976	0.77038	-0.37635	0.033107	0.88953	0.65538	-0.6096	0.003333	0.43007	0.7362	-0.44183	0.054689	0.99987	0.78638	-0.3467	0.012763	0.95986	0.79362	-0.33349	0.07932	0.99796	0.68031	-0.55574	0.42646	0.99768	0.93562	-0.09601	0.037801	0.9981	0.68138	-0.55347	0.030791	0.99995	0.78854	-0.34274
795.5975	5.19	domics Pos	0.74559	0.99995	1.0218	0.031128	0.225	0.99979	1.0594	0.083213	0.40607	0.9581	0.96601	-0.04989	0.83076	0.99996	0.9893	-0.01552	0.696	0.99701	0.98083	-0.02793	0.5386	0.99959	1.0385	0.054437	0.017029	0.17568	1.2569	0.32983	0.68624	0.99984	1.0318	0.045156	0.10416	0.99966	1.101	0.13878
441.8896	6.37	olomics Pc	0.056615	0.99964	0.79361	-0.3335	0.037783	0.88953	0.6414	-0.64071	0.05269	0.99882	0.79689	-0.32755	0.51731	0.99987	0.92629	-0.11046	0.042036	0.95986	0.82206	-0.28269	0.1128	0.99796	0.73886	-0.43662	0.43266	0.99768	1.047	0.066223	0.16285	0.9981	0.74392	-0.42678	0.081828	0.99995	0.78237	-0.35408

434.6952	0.63	domics Pos	0.030415	0.90678	1.1994	0.26229	0.98283	0.99979	1.009	0.012869	0.75048	0.99601	0.97149	-0.04173	0.79275	0.99996	1.0051	0.007289	0.38857	0.99701	0.91759	-0.12408	0.23687	0.99959	0.9059	-0.14258	0.78215	0.87433	1.0787	0.10932	0.41348	0.99984	1.068	0.094971	0.23978	0.99966	1.1546	0.20741
178.1669	2.05	domics Pos	0.32276	0.99995	0.92732	-0.10886	0.075598	0.99979	0.74258	-0.42938	0.029952	0.37201	0.825	-0.27754	0.35023	0.99996	0.87149	-0.19844	0.18027	0.95949	0.82811	-0.2721	0.22906	0.99959	0.81911	-0.28788	0.08225	0.27271	1.3042	0.38313	0.32093	0.99984	0.84869	-0.23668	0.12716	0.99966	0.86964	-0.20152
121.1018	1.67	domics Pos	0.021788	0.8137	0.89186	-0.16511	0.010213	0.99441	0.70599	-0.50228	0.001432	0.13123	0.79765	-0.32617	0.35832	0.99996	0.88503	-0.17621	0.012715	0.48469	0.79092	-0.3384	0.11848	0.99959	0.77474	-0.36822	0.079487	0.26849	1.2349	0.30444	0.11697	0.99984	0.79061	-0.33896	0.04003	0.99966	0.83657	-0.25745
350.9875	4	olomics Ne	0.83726	0.9951	0.98947	-0.01527	0.47544	0.99975	0.95675	-0.06379	0.091336	0.99731	0.88746	-0.17225	0.50799	0.99915	0.9576	-0.0625	0.99534	0.99983	0.99928	-0.00104	0.20388	0.63388	1.0819	0.11363	0.025044	0.086116	1.1724	0.22952	0.80799	0.99481	1.0155	0.022247	0.12078	0.91738	0.9144	-0.12911
212.202	6.38	olomics Pc	0.001068	0.29623	0.8622	-0.21391	0.051905	0.90372	0.78341	-0.35217	0.009301	0.55189	0.83976	-0.25195	0.53011	0.99987	0.95729	-0.06297	0.063381	0.95986	0.89917	-0.15334	0.12612	0.99796	0.8138	-0.29725	0.016323	0.4752	1.0799	0.11089	0.058614	0.9981	0.7872	-0.3452	0.063704	0.99995	0.86306	-0.21247
558.8568	0.54	domics Pos	0.63093	0.99995	0.96618	-0.04963	0.65463	0.99979	1.0792	0.10996	0.042256	0.44593	0.86444	-0.21016	0.93142	0.99996	1.002	0.002818	0.12224	0.86273	0.88561	-0.17525	0.68792	0.99959	0.96939	-0.04485	0.40238	0.62344	1.0843	0.11678	0.33394	0.99984	0.93264	-0.10061	0.80251	0.99966	1.0208	0.02973
168.1398	5.99	olomics Pc	0.008748	0.65613	0.80328	-0.31603	0.042777	0.88953	0.78693	-0.3457	0.008804	0.55189	0.78668	-0.34615	0.4788	0.99987	0.93098	-0.10318	0.10237	0.95986	0.87139	-0.1986	0.111	0.99796	0.74062	-0.43319	0.73136	0.99768	0.97747	-0.03288	0.090149	0.9981	0.74816	-0.41857	0.017591	0.99995	0.83438	-0.26122
163.1485	2.77	domics Pos	0.55123	0.99995	0.91401	-0.12972	0.099397	0.99979	0.75854	-0.39871	0.20494	0.83096	0.81209	-0.30029	0.58629	0.99996	0.92177	-0.11752	0.19989	0.97344	0.82295	-0.28112	0.034113	0.99959	0.62897	-0.66893	0.34976	0.57385	1.1774	0.23559	0.56402	0.99984	0.91614	-0.12635	0.15221	0.99966	0.80803	-0.30752
280.2645	5.99	olomics Pc	0.045395	0.99964	0.87543	-0.19193	0.067524	0.99187	0.78596	-0.34747	0.051722	0.99882	0.86012	-0.2174	0.49692	0.99987	0.94443	-0.08249	0.15716	0.95986	0.904	-0.14561	0.19805	0.99796	0.84657	-0.2403	0.16094	0.9712	1.0708	0.098716	0.09611	0.9981	0.81331	-0.29812	0.083374	0.99995	0.83293	-0.26373
258.981	12.01	olomics Ne	0.6675	0.9951	1.0234	0.033346	0.3977	0.99975	1.0851	0.11786	0.6972	0.99731	0.96886	-0.04564	0.036302	0.99915	1.1616	0.21611	0.52031	0.84983	1.052	0.073107	0.57308	0.82775	1.0639	0.089313	0.097099	0.18548	1.1914	0.25267	0.72296	0.99481	1.0589	0.082547	0.54298	0.96586	0.95448	-0.06722
254.2488	5.68	olomics Pc	0.004048	0.42508	0.85787	-0.22116	0.046992	0.88953	0.78603	-0.34734	0.010733	0.58293	0.82275	-0.28147	0.67237	0.99987	0.97271	-0.03993	0.049201	0.95986	0.88235	-0.18058	0.20373	0.99796	0.8454	-0.2423	0.091831	0.87442	1.0665	0.092907	0.14247	0.9981	0.80725	-0.30891	0.12926	0.99995	0.89604	-0.15836
455.3059	2.06	domics Pos	0.8176	0.99995	0.98682	-0.01914	0.1847	0.99979	0.85413	-0.22747	0.026133	0.34591	0.81473	-0.2956	0.53723	0.99996	0.94672	-0.07899	0.092966	0.82209	0.90737	-0.14024	0.7519	0.99959	0.98147	-0.02698	0.036795	0.20439	1.3359	0.41778	0.68198	0.99984	0.96554	-0.0506	0.93916	0.99966	1.0123	0.017651
589.9215	11.98	olomics Ne	0.68874	0.9951	1.0249	0.035537	0.71353	0.99975	0.97879	-0.03093	0.79921	0.99731	0.98565	-0.02085	0.93148	0.99915	1.0076	0.01096	0.18373	0.67513	1.102	0.14018	0.49317	0.79937	1.0751	0.10445	0.01469	0.066581	1.2538	0.32626	0.92772	0.99481	1.0028	0.004103	0.20704	0.91738	0.9175	-0.12422
219.1647	5.2	olomics Pc	0.23964	0.99964	1.0771	0.1072	0.63646	1	0.97036	-0.04341	0.75741	0.99882	0.98001	-0.02913	0.92535	0.99987	0.99961	-0.00056	0.049589	0.95986	1.1507	0.20247	0.58511	0.99796	0.97098	-0.04249	0.30569	0.99768	1.1009	0.13872	0.68512	0.9981	0.96764	-0.04745	0.67406	0.99995	1.0209	0.029861
254.2479	7.02	olomics Pc	0.055234	0.99964	0.8593	-0.21877	0.041286	0.88953	0.67516	-0.56669	0.010615	0.58293	0.74728	-0.42028	0.40403	0.99987	0.91174	-0.13331	0.091337	0.95986	0.86031	-0.21708	0.17528	0.99796	0.75094	-0.41323	0.21357	0.99768	1.0854	0.11818	0.095137	0.9981	0.7175	-0.47895	0.15891	0.99995	0.85711	-0.22245
625.5259	2.05	domics Pos	0.009143	0.68962	0.8164	-0.29265	0.064472	0.99979	0.70082	-0.51288	0.001251	0.13107	0.71026	-0.49358	0.12324	0.99996	0.82082	-0.28487	0.013558	0.4899	0.804	-0.31474	0.14653	0.99959	0.73127	-0.45152	0.15498	0.36343	1.2685	0.3431	0.25907	0.99984	0.79433	-0.33219	0.093884	0.99966	0.8499	-0.23463
306.2593	2.17	domics Pos	0.31999	0.99995	0.8866	-0.17365	0.2477	0.99979	0.86985	-0.20116	0.033626	0.3933	0.73576	-0.44269	0.49177	0.99996	0.90076	-0.15078	0.059791	0.76594	0.814	-0.29689	0.24997	0.99959	0.85981	-0.2179	0.87117	0.92761	1.0417	0.058889	0.30066	0.99984	0.82987	-0.26905	0.63031	0.99966	0.94295	-0.08475
104.1078	0.54	domics Pos	0.96396	0.99995	1.0076	0.010896	0.32961	0.99979	0.88786	-0.17159	0.12442	0.72566	0.78294	-0.35302	0.49747	0.99996	0.92497	-0.11252	0.51606	0.99701	0.91993	-0.1204	0.95954	0.99959	0.99631	-0.00533	0.012705	0.16313	1.4176	0.5034	0.25476	0.99984	0.86421	-0.21055	0.93457	0.99966	1.0279	0.039761
111.1125	2.05	domics Pos	0.032691	0.91043	0.87109	-0.1991	0.057252	0.99653	0.76553	-0.38546	0.003126	0.15208	0.74029	-0.43383	0.37275	0.99996	0.89969	-0.1525	0.005389	0.46849	0.76687	-0.38294	0.12704	0.99959	0.76613	-0.38434	0.078355	0.26795	1.1842	0.2439	0.21891	0.99984	0.81493	-0.29525	0.039817	0.99966	0.86825	-0.20382
741.4282	3.22	olomics Pc	0.53175	0.99964	0.92451	-0.11323	0.24511	1	0.89709	-0.15667	0.041702	0.93262	0.73411	-0.44594	0.69437	0.99987	0.99628	-0.00537	0.12386	0.95986	0.73169	-0.4507	0.57135	0.99796	0.93453	-0.09769	0.85699	0.99768	1.1985	0.26126	0.5861	0.9981	0.93507	-0.09685	0.60003	0.99995	0.94806	-0.07695
302.2441	6.01	olomics Pc	0.042344	0.99964	0.8676	-0.2049	0.15146	1	0.84559	-0.24196	0.35281	0.99882	0.93921	-0.09048	0.46357	0.99987	0.94367	-0.08364	0.50448	0.95986	0.95692	-0.06353	0.23961	0.99796	0.87522	-0.19229	0.10317	0.90326	1.1001	0.13768	0.087559	0.9981	0.83872	-0.25374	0.047163	0.99995	0.76993	-0.3772
561.9682	0.53	domics Pos	0.84611	0.99995	0.98501	-0.02179	0.23096	0.99979	0.84088	-0.25004	0.0184	0.29983	0.6876	-0.54035	0.72788	0.99996	1.0711	0.099063	0.087904	0.81951	0.68389	-0.54817	0.30889	0.99959	0.84565	-0.24187	0.63682	0.78992	1.4997	0.58464	0.57208	0.99984	0.91364	-0.13031	0.73808	0.99966	1.0029	0.00415
291.9446	12.1	olomics Pc	0.70042	0.99964	0.97219	-0.04068	0.63655	1	0.96066	-0.05791	0.39297	0.99882	0.93857	-0.09147	0.041699	0.99987	0.86729	-0.20541	0.46305	0.95986	0.95526	-0.06604	0.78968	0.99796	1.0116	0.016597	0.17014	0.97894	1.0921	0.12707	0.25112	0.9981	0.92649	-0.11014	0.48092	0.99995	0.95428	-0.06752
852.8659	12.01	olomics Pc	0.83207	0.99964	1.02	0.028589	0.52667	1	1.0749	0.10415	0.86239	0.99882	0.99405	-0.00861	0.27864	0.99987	1.0892	0.12327	0.011168	0.95986	1.1371	0.18538	0.51757	0.99796	0.95711	-0.06325	0.79963	0.99768	0.98793	-0.01751	0.21704	0.9981	1.1252	0.17015	0.13991	0.99995	1.1358	0.18374
165.1642	1.67	domics Pos	0.22086	0.99995	0.88313	-0.1793	0.037604	0.99441	0.74981	-0.4154	0.009628	0.21409	0.75156	-0.41203	0.39775	0.99996	0.88084	-0.18304	0.067441	0.77566	0.8289	-0.27074	0.18046	0.99959	0.80221	-0.31796	0.16182	0.37073	1.1792	0.2378	0.23441	0.99984	0.82071	-0.28506	0.34204	0.99966	0.91112	-0.13428
188.0777	10.16	domics Pos	0.25498	0.99995	1.0867	0.11999	0.84149	0.99979	1.0121	0.017311	0.24289	0.85835	0.90167	-0.14933	0.98368	0.99996	1.0131	0.018846	0.45297	0.99701	0.92822	-0.10747	0.62987	0.99959	0.96715	-0.04819	0.039184	0.20439	1.2514	0.32353	0.5239	0.99984	0.96006	-0.0588	0.47552	0.99966	1.0798	0.11083
266.2571	6.38	olomics Pc	0.002905	0.38271	0.84254	-0.24719	0.041789	0.88953	0.76673	-0.3832	0.02472	0.74312	0.84434	-0.24411	0.33118	0.99987	0.92352	-0.11479	0.024115	0.95986	0.87163	-0.19821	0.12307	0.99796	0.80498	-0.31297	0.15693	0.96247	1.0581	0.081449	0.065057	0.9981	0.78395	-0.35116	0.033081	0.99995	0.85571	-0.22481
152.1503	2.05	domics Pos	0.18311	0.99995	0.92697	-0.10941	0.74854	0.99979	0.97741	-0.03296	0.007275	0.19397	0.78029	-0.35792	0.81762	0.99996	0.99145	-0.01239	0.17961	0.95949	0.86287	-0.21279	0.25258	0.99959	0.86599	-0.20759	0.02763	0.1951	1.3572	0.44067	0.23569	0.99984	0.80865	-0.30641	0.69977	0.99966	0.97171	-0.04141
338.9861	12	olomics Ne	0.66762	0.9951	0.9155	-0.12737	0.40343	0.99975	1.3321	0.41367	0.72665	0.99731	1.041	0.058001	0.24121	0.99915	1.421	0.50695	0.18246	0.67248	1.2063	0.27064	0.22075	0.63777	1.2037	0.2675	0.011047	0.061656	1.7735	0.82661	0.97126	0.99785	1.0004	0.000533	0.73256	0.97489	0.931	-0.10315
352.8983	10.07	domics Pos	0.19576	0.99995	1.0859	0.11893	0.88873	0.99979	0.99525	-0.00688	0.064852	0.55764	0.95155	-0.07164	0.66379	0.99996	0.97628	-0.03463	0.27822	0.99701	0.94821	-0.07673	0.90455	0.99959	0.99675	-0.00469	0.018272	0.17894	1.2187	0.2853	0.90365	0.99984	0.99644	-0.00515	0.56247	0.99966	1.0401	0.056773
532.6223	3.28	olomics Pc	0.86145	0.99964	0.98857	-0.01659	0.2839	1	0.87909	-0.18591	0.042645	0.93262	0.79855	-0.32454	0.66205	0.99987	1.234	0.30337	0.16663	0.95986	0.76469	-0.38704	0.81577	0.99796	1.0864	0.11955	0.34373	0.99768	1.5681	0.64901	0.96587	0.9981	1.0703	0.098063	0.95866	0.99995	1.0384	0.054375
503.3061	2.57	olomics Pc	0.64985	0.99964	1.2254	0.29326	0.04009	0.88953	0.76991	-0.37724	0.3505	0.99882	0.845	-0.24298	0.92006	0.99987	1.338	0.42005	0.26938	0.95986	0.80915	-0.30552	0.96536	0.99796	1.0258	0.036708	0.90685	0.99768	1.3775	0.46205	0.84753	0.9981	0.98658	-0.0195	0.66653	0.99995	1.2886	0.36581
128.1082	5.99	olomics Pc	0.009779	0.68452	0.80894	-0.30589	0.031533	0.88953	0.73749	-0.4393	0.019328	0.63482	0.8216	-0.28349	0.57006	0.99987	0.94547	-0.0809	0.099794	0.95986	0.8561	-0.22415	0.21792	0.99796	0.82157	-0.28354	0.53602	0.99768	1.0342	0.048468	0.1296	0.9981	0.78249	-0.35386	0.19937	0.99995	0.89489	-0.16021
164.1519	2.05	domics Pos	0.035651	0.944	0.8499	-0.23464	0.10722	0.99979	0.7926	-0.33533	0.003848	0.15284	0.75296	-0.40936	0.39839	0.99996	0.8986	-0.15424	0.012003	0.48469	0.82294	-0.28114	0.089767	0.99959	0.75839	-0.39898	0.021078	0.18176	1.3274	0.40866	0.25904	0.99984	0.82554	-0.27659	0.32041	0.99966	0.92227	-0.11674
934.7048	0.56	domics Pos	0.66436	0.99995	0.97178	-0.04129	0.28666	0.99979	0.86478	-0.2096	0.040002	0.43226	0.84339	-0.24573	0.23995	0.99996	0.90029	-0.15154	0.027644	0.55491	0.84482	-0.24328	0.16358	0.99959	0.85054	-0.23354	0.11735	0.32013	0.89859	-0.15426	0.69547	0.99984	0.98237	-0.02566	0.23483	0.99966	0.90701	-0.14081
626.9725	12.01	olomics Ne	0.80725	0.9951	1.0186	0.026536	0.82516	0.99975	0.98046	-0.02847	0.44021	0.99731	0.91857	-0.12254	0.71862	0.99915	0.96687	-0.04861	0.049649	0.58152	0.84045	-0.25077	0.21435	0.6375	0.90331	-0.1467	0.85209	0.89346	0.98298	-0.02477	0.99777	0.99785	1.0016	0.002261	0.22676	0.91738	0.85651	-0.22345
627.9776	12.03	olomics Ne	0.80024	0.9951	1.056	0.078616	0.39481	0.99975	0.87673	-0.18979	0.39758	0.99731	0.86585	-0.20782	0.557	0.99915	1.1604	0.21461	0.40651	0.76847	0.87821	-0.18736	0.96769	0.99269	0.99256	-0.01077	0.59215	0.67307	1.0977	0.13443	0.040938	0.99481	0.7381	-0.43812	0.24937	0.91738	0.81315	-0.29841
181.633	6.39	olomics Pc	0.10034	0.99964	0.89891	-0.15375	0.074514	0.99372	0.81047	-0.30317	0.018696	0.63482	0.82533	-0.27696	0.43078	0.99987	0.93875	-0.09119	0.21208	0.95986	0.8862	-0.1743	0.34022	0.99796	0.91285	-0.13155	0.062449	0.7932	1.1689	0.22516	0.64087	0.9981	0.9582	-0.0616	0.78634	0.99995	0.98189	-0.02637
131.9219	10.64	olomics Ne	0.17037	0.9951	1.1043	0.14307	0.2948	0.99975	1.0845	0.11698	0.53523	0.99731	1.0377	0.053387	0.17073	0.99915	1.0895	0.12371	0.01576	0.50209	1.201	0.26429	0.076435	0.5223	1.1688	0.22507	0.01016	0.061007	1.3703	0.45453	0.13743	0.99481	1.0974	0.13411	0.66513	0.96789	0.97449	-0.03728
1066.764	10.03	domics Pos	0.4817	0.99995	1.0697	0.097166	0.04686	0.99441	1.1173	0.15995	0.75505	0.99601	0.98885	-0.01617	0.68533	0.99996	1.053	0.074473	0.23831	0.99701	0.9494	-0.07491	0.5168	0.99959	1.0899	0.12413	0.009129	0.1521	1.3243	0.40527	0.78893	0.99984	1.0271	0.038622	0.10283	0.99966	1.1471	0.19803
525.6303	3.28	olomics Pc	0.98771	0.99964	1.0231	0.032957	0.31236	1	0.85448	-0.22689	0.028704	0.79314	0.73798	-0.43834	0.80226	0.99987	1.1797	0.23843	0.065047	0.95986	0.69608	-0.52267	0.42517	0.99796	0.87782	-0.188	0.66977	0.99768	1.2667	0.34112	0.60492	0.9981	0.92589	-0.11109	0.81718	0.99995	0.99578	-0.0061
798.6289	10.3	olomics Ne	0.33553	0.9951	1.0785	0.10906	0.50618	0.99975	1.06	0.084076	0.97875	0.99731	0.99956	-0.00064	0.5334	0.99915	0.95374	-0.06834	0.26	0.71555	1.1048	0.14379	0.22519	0.6407	1.0917	0.12658	0.048781	0.12484	1.2606	0.3341	0.21477	0.99481	1.1083	0.14833	0.31272	0.91738	0.93105	-0.10306
1059.782	10.03	domics Pos	0.52516	0.99995	1.0982	0.13516	0.68714	0.99979	1.0301	0.042775	0.76417	0.99601	1.0155	0.022228	0.19353	0.99996	1.1194	0.16277	0.8661	0.99701	1.0243	0.034601	0.17227	0.99959	1.154	0.2067	0.01088	0.1625	1.3726	0.45689	0.14294	0.99984	1.1499	0.20156	0.17734	0.99966	1.1947	0.25665
473.9152	0.54	domics Pos	0.75559	0.99995	1.0791	0.10979	0.18177	0.99979	0.81982	-0.28662	0.031122	0.38157	0.71376	-0.48648	0.7984	0.99996	1.1045	0.14337	0.095315	0.82209	0.70312	-0.50816	0.43422	0.99959	0.86703	-0.20585	0.52442	0.72006	1.4825	0.56803	0.6494	0.99984	0.93689	-0.09404	0.93821	0.99966	1.0892	0.12332
205.1954	6.38	olomics Pc	0.10444	0.99964	0.9162	-0.12627	0.080456	0.99372	0.79211	-0.33623	0.078627	0.99882	0.90446	-0.14487	0.8818	0.99987	1.0287	0.040811	0.37057	0.95986	0.9385	-0.09157	0.29186	0.99796	0.87787	-0.18793	0.033211	0.65634	1.1298	0.17613	0.22236	0.9981	0.87561	-0.19165	0.35678	0.99995	0.90598	-0.14245
177.1644	1.66	domics Pos	0.92484	0.99995	0.98789	-0.01757	0.03636	0.99441	0.72667	-0.46064	0.12725	0.72638	0.83791	-0.25514	0.59475	0.99996	0.93359	-0.09914	0.41741	0.99701	0.91448	-0.12898	0.31923	0.99959	0.84734	-0.23898	0.025204	0.18884	1.3819	0.46662	0.38192	0.99984	0.8642	-0.21056	0.51167	0.99966	0.92812	-0.10761
280.983	12.01	olomics Ne	0.72754	0.9951	1.013	0.01868	0.37272	0.99975	1.0591	0.082801	0.37104	0.99731	0.95804	-0.06184	0.32515	0.99915	1.0409	0.057891	0.38107	0.75206	1.0352	0.049931	0.2208	0.63777	1.0668	0.093259	0.003801	0.050041	1.1975	0.25998	0.99125	0.99785	1.0021	0.002967	0.043165	0.91738	0.91847	-0.1227
1183.291	5.98	domics Pos	0.76724	0.99995	1.0662	0.092468	0.24172	0.99979	0.8344	-0.26118	0.17417	0.7963	0.83473	-0.26062	0.80813	0.99996	1.1757	0.23358	0.043985	0.66291	0.66031	-0.59878	0.075176	0.99959	0.76916	-0.37865	0.6037	0.7709	1.6519	0.7241	0.40188	0.99984	0.89565	-0.159	0.79214	0.99966	1.0727	0.10129
162.1363	1.67	domics Pos	0.52543	0.99995	0.95366	-0.06845	0.009342	0.99441	0.73319	-0.44774	0.016339	0.28182	0.79341	-0.33387	0.35938	0.99996	0.89644	-0.15773	0.51003	0.99701	0.93571	-0.09587	0.40112	0.99959	0.88438	-0.17725	0.007128	0.13972	1.2315	0.30041	0.48152	0.99984	0.90501	-0.14399	0.046326	0.99966	0.87108	-0.19913
114.0921	1.66	domics Pos	0.34937	0.99995	0.94219	-0.08591	0.095032	0.99979	0.77744	-0.36321	0.076893	0.60717	0.84051	-0.25066	0.44946	0.99996	0.89827	-0.15478	0.040073	0.64262	0.81818	-0.28951	0.12742	0.99959	0.7738	-0.36997	0.068493	0.2547	1.2454	0.31656	0.16505	0.99984	0.80627	-0.31066	0.49774	0.99966	0.95564	-0.06547
907.829	10.02	domics Pos	0.51381	0.99995	1.0768	0.10671	0.17208	0.99979	1.071	0.098987	0.20766	0.83096	1.0689	0.096188	0.81185	0.99996	1.0257	0.036601	0.66931	0.99701	0.97286	-0.03969	0.51929	0.99959	1.0585	0.082075	0.039869	0.20711	1.296	0.37403	0.68892	0.99984	1.021	0.029913	0.20403	0.99966	1.104	0.14268
81.0713	6.39	olomics Pc	0.1075	0.99964	0.89235	-0.16432	0.003208	0.88953	0.75896	-0.3979	0.073465	0.99882	0.88949	-0.16894	0.52884	0.99987	0.94154	-0.08691	0.36938	0.95986	0.94194	-0.08629	0.03935	0.99796	0.74963	-0.41576	0.58778	0.99768	1.0334	0.047393	0.085378	0.9981	0.81834	-0.28923	0.081073	0.99995	0.81411	-0.29671
122.1056	6.38	olomics Pc	0.12822	0.99964	0.8805	-0.1836	0.001945	0.88953	0.76175	-0.39261	0.054676	0.99882	0.85922	-0.2189	0.71774	0.99987	0.97416	-0.03777	0.54176	0.95986	0.95208	-0.07085	0.039683	0.99796	0.71885	-0.47623	0.84949	0.99768	1.0148	0.021132	0.063613	0.9981	0.84432	-0.24414	0.10222	0.99995	0.86694	-0.206
212.2013	1.66	domics Pos	0.1157	0.99995	0.86507	-0.20911	0.038516	0.99441	0.71423	-0.48554	0.009127	0.21077	0.74906	-0.41684	0.27411	0.99996	0.85064	-0.23338	0.025907	0.5516	0.8008	-0.32048	0.17458	0.99959	0.79097	-0.33831	0.15177	0.36054	1.2534	0.32583	0.18761	0.99984	0.77854	-0.36116	0.21648	0.99966	0.88824	-0.17097
264.8535	9.91	domics Pos	0.035178	0.944	1.172	0.22903	0.68241	0.99979	0.97056	-0.04312	0.23324	0.84219	0.92206	-0.11707	0.093711	0.99996	1.1309	0.17751	0.71155	0.99701	0.96981	-0.04423	0.12849	0.99959	1.1628	0.21763	0.24392	0.4642	1.2056	0.26976	0.44811	0.99984	1.091	0.12559	0.28486	0.99966	1.088	0.12169
67.0551	2.05	domics Pos	0.044033	0.99995	0.82041	-0.28558	0.025524	0.99441	0.68501	-0.54581	0.022715	0.32209	0.73707	-0.44013	0.27208	0.99996	0.84456	-0.24373	0.050009	0.70432	0.80502	-0.3129	0.085928	0.99959	0.73957	-0.43525	0.56307	0.74704	1.0553	0.077619	0.076751	0.99984	0.7235	-0.46694	0.044451	0.99966	0.80788	-0.30779
454.9747	5.08	olomics Ne	0.30298	0.9951	1.0659	0.092079	0.89417	0.99975	1.0047	0.006815	0.94319	0.99731	0.99737	-0.0038	0.56829	0.99915	1.0282	0.040074	0.10286	0.60605	1.0986	0.13564	0.049175	0.49499	1.1318	0.17866	0.002633	0.048382	1.2773	0.35306	0.15222	0.99481	1.0617	0.086348	0.57194	0.96658	0.971	-0.04246
344.8758	10.14	domics Pos	0.49697	0.99995	1.1157	0.15794	0.78041	0.99979	1.0661	0.092314	0.050038	0.49174	1.2792	0.35521	0.28662	0.99996	1.1632	0.21815	0.10959	0.85684	1.1907	0.25178	0.17682	0.99959	1.1964	0.2587	0.004459	0.12687	1.561	0.64252	0.020277	0.99984	1.3429	0.4253	0.11488	0.99966	1.2358	0.3054
95.0869	6.38	olomics Pc	0.007821	0.60235	0.90941	-0.137	0.014878	0.88953	0.79356	-0.3336	0.006327	0.51418	0.88676	-0.17339	0.81883	0.99987	0.99254	-0.01081	0.54867	0.95986	0.97194	-0.04106	0.059509	0.99796	0.79817	-0.32523	0.34355	0.99768	1.0492	0.069241	0.083674	0.9981	0.82683	-0.27434	0.087839	0.99995	0.84661	-0.24024
157.1424	2.05	domics Pos	0.16466	0.99995	0.82839	-0.27162	0.25223	0.99979	0.82106	-0.28443	0.01643	0.28182	0.72067	-0.4726	0.82055	0.99996	0.98838	-0.01686	0.03241	0.59375	0.73723	-0.43981	0.042191	0.99959	0.77158	-0.37412	0.33163	0.55387	1.1222	0.16631	0.096951	0.99984	0.75545	-0.4046	0.30395	0.99966	0.90804	-0.13918
1020.576	10.22	olomics Pc	0.017088	0.86976	1.2437	0.31464	0.046697	0.88953	1.2286	0.29701	0.36147	0.99882	1.0946	0.13045	0.11567	0.99987	1.1777	0.23596	0.049431	0.95986	1.3204	0.40097	0.057193	0.99796	1.3108	0.3905	0.13399	0.95058	1.2835	0.36012	0.94841	0.9981	1.0211	0.030128	0.14752	0.99995	1.1923	0.25379
209.1912	2.05	domics Pos	0.021058	0.79726	0.83061	-0.26775	0.03838	0.99441	0.71635	-0.48126	0.026485	0.34652	0.80777	-0.30798	0.3607	0.99996	0.87844	-0.18698	0.035961	0.62141	0.81643	-0.29261	0.19655	0.99959	0.80473	-0.31342	0.093386	0.28973	1.2425	0.31326	0.1423	0.99984	0.78906	-0.34179	0.15554	0.99966	0.89136	-0.16592
805.4655	8.14	olomics Ne	0.39221	0.9951	1.1018	0.13984	0.90432	0.99975	1.0057	0.008263	0.65033	0.99731	1.0348	0.049283	0.77588	0.99915	0.97999	-0.02916	0.36219	0.75206	1.1386	0.1873	0.74666	0.91275	1.0431	0.060842	0.012599	0.062293	1.2253	0.29308	0.76793	0.99481	1.0232	0.033104	0.47965	0.94624	0.94602	-0.08006
261.9454	12.46	olomics Pc	0.27848	0.99964	1.1711	0.22787	0.001147	0.88953	1.3181	0.39846	0.16394	0.99882	1.0694	0.09681	0.047456	0.99987	1.1842	0.24388	0.3579	0.95986	1.1459	0.19649	0.097421	0.99796	1.2027	0.26623	0.001213	0.25467	1.3827	0.46746	0.005497	0.9981	1.2557	0.32844	0.28631	0.99995	1.1302	0.17658
574.9728	12	olomics Ne	0.82937	0.9951	1.016	0.022849	0.055243	0.99975	1.2378	0.30778	0.33245	0.99731	0.8922	-0.16457	0.81142	0.99915	1.0256	0.036403	0.35772	0.75206	1.0779	0.1082	0.25848	0.66013	1.1241	0.16874	0.031992	0.097723	1.3501	0.43304	0.71617	0.99481	1.066	0.092163	0.84077	0.98477	0.9793	-0.03018
179.1744	6	olomics Pc	0.20756	0.99964	0.93312	-0.09987	0.027356	0.88953	0.8119	-0.30063	0.063154	0.99882	0.8753	-0.19214	0.73424	0.99987	0.97412	-0.03783	0.36516	0.95986	0.9417	-0.08665	0.26076	0.99796	0.84856	-0.23692	0.34021	0.99768	1.0605	0.084745	0.21407	0.9981	0.87055	-0.2	0.16606	0.99995	0.8724	-0.19693
442.3905	2.05	domics Pos	0.18515	0.99995	0.85531	-0.22548	0.053369	0.99441	0.65847	-0.60281	0.029683	0.36988	0.72136	-0.4712	0.11842	0.99996	0.7979	-0.32572	0.091894	0.82209	0.7825	-0.35383	0.14912	0.99959	0.71741	-0.47914	0.15251	0.36054	1.2666	0.34093	0.20759	0.99984	0.74746	-0.41993	0.077739	0.99966	0.81634	-0.29276
109.1025	6.38	olomics Pc	0.026703	0.9464	0.91305	-0.13123	0.010264	0.88953	0.79854	-0.32457	0.16276	0.99882	0.96599	-0.04992	0.72278	0.99987	0.9765	-0.03431	0.98092	0.99906	1.0025	0.003605	0.14213	0.99796	0.8147	-0.29565	0.01306	0.43304	1.123	0.16742	0.31295	0.9981	0.89953	-0.15275	0.23613	0.99995	0.89329	-0.1628
196.1703	1.67	domics Pos	0.043828	0.99995	0.86125	-0.2155	0.030707	0.99441	0.72071	-0.4725	0.000739	0.13107	0.69631	-0.52219	0.33896	0.99996	0.87936	-0.18548	0.036923	0.62436	0.81418	-0.29659	0.16612	0.99959	0.78227	-0.35426	0.13007	0.33153	1.1771	0.23523	0.15127	0.99984	0.79384	-0.33308	0.29543	0.99966	0.9213	-0.11826
128.1083	6.38	olomics Pc	0.003075	0.38271	0.8361	-0.25826	0.041602	0.88953	0.78682	-0.34589	0.006433	0.51418	0.81842	-0.28909	0.80817	0.99987	0.98765	-0.01793	0.04262	0.95986	0.86929	-0.20209	0.08377	0.99796	0.83073	-0.26755	0.25476	0.99768	1.0465	0.065638	0.085864	0.9981	0.7853	-0.34869	0.064481	0.99995	0.86363	-0.21152
433.2346	6.04	olomics Ne	0.27563	0.9951	0.9001	-0.15184	0.016264	0.99975	0.77005	-0.37698	0.020527	0.99731	0.72874	-0.45652	0.50146	0.99915	0.95456	-0.06709	0.72824	0.92514	0.97564	-0.03559	0.73498	0.91163	1.0824	0.11425	0.004763	0.051484	1.6143	0.69088	0.43724	0.99481	0.90614	-0.14219	0.25152	0.91738	0.86595	-0.20764
920.6562	10.25	olomics Ne	0.74054	0.9951	0.98329	-0.02431	0.46766	0.99975	1.0473	0.066718	0.52891	0.99731	0.94398	-0.08318	0.20481	0.99915	1.0578	0.081087	0.009357	0.50209	1.1851	0.245	0.01699	0.45618	1.1799	0.23868	0.005658	0.053163	1.1951	0.25711	0.56242	0.99481	1.0384	0.054398	0.60921	0.96789	0.96928	-0.04501
404.9152	10.03	domics Pos	0.46767	0.99995	1.0401	0.056746	0.73636	0.99979	1.0392	0.055542	0.10608	0.68915	0.91196	-0.13296	0.46729	0.99996	0.95328	-0.06902	0.68069	0.99701	0.97822	-0.03176	0.56255	0.99959	1.0664	0.092706	0.020488	0.18119	1.2151	0.28103	0.45409	0.99984	0.95398	-0.06797	0.94001	0.99966	1.0052	0.007427
492.9731	5.28	olomics Ne	0.48211	0.9951	1.0458	0.064543	0.45719	0.99975	1.0398	0.05624	0.077115	0.99731	0.94649	-0.07934	0.25048	0.99915	1.0567	0.07953	0.036733	0.53488	1.1151	0.1572	0.17338	0.60765	1.0942	0.12984	0.021867	0.077935	1.2258	0.29377	0.16112	0.99481	1.0543	0.076255	0.58155	0.96789	0.97479	-0.03683
128.1076	7.02	olomics Pc	0.014058	0.83315	0.78854	-0.34275	0.023781	0.88953	0.65755	-0.60483	0.002185	0.39871	0.72047	-0.47299	0.044523	0.99987	0.77826	-0.36168	0.015867	0.95986	0.81516	-0.29485	0.097461	0.99796	0.70017	-0.51422	0.14695	0.95305	0.89353	-0.16241	0.066937	0.9981	0.65448	-0.61157	0.001859	0.7981	0.75747	-0.40074
296.2955	6.74	olomics Pc	0.06326	0.99964	0.83778	-0.25535	0.024516	0.88953	0.68888	-0.53768	0.11061	0.99882	0.84075	-0.25025	0.21376	0.99987	0.86143	-0.21519	0.16196	0.95986	0.86087	-0.21614	0.10837	0.99796	0.7372	-0.43988	0.76884	0.99768	0.97998	-0.02917	0.118	0.9981	0.77081	-0.37556	0.067322	0.99995	0.86612	-0.20737
318.2402	1.01	domics Pos	0.98218	0.99995	0.99355	-0.00933	0.2229	0.99979	0.86455	-0.20998	0.041582	0.44135	0.78777	-0.34416	0.56818	0.99996	0.92483	-0.11273	0.11129	0.85684	0.82127	-0.28407	0.22951	0.99959	0.84264	-0.24702	0.10687	0.30716	1.2303	0.29905	0.47055	0.99984	0.89209	-0.16473	0.80614	0.99966	1.016	0.022857
795.5638	9.06	olomics Ne	0.22009	0.9951	1.1541	0.20672	0.18299	0.99975	0.90006	-0.15191	0.80646	0.99731	1.0393	0.055629	0.82085	0.99915	0.98717	-0.01862	0.030201	0.51873	1.2016	0.26492	0.86056	0.96599	1.0551	0.077334	0.076177	0.16196	1.2768	0.35256	0.51986	0.99481	1.0847	0.11727	0.48686	0.9483	0.91711	-0.12484
364.2167	1.66	domics Pos	0.017774	0.73663	0.85457	-0.22673	0.031166	0.99441	0.72135	-0.47123	0.020168	0.30717	0.75474	-0.40594	0.18484	0.99996	0.83336	-0.26299	0.012635	0.48469	0.80335	-0.31589	0.11753	0.99959	0.7312	-0.45167	0.20222	0.41769	1.1528	0.20511	0.061597	0.99984	0.73998	-0.43445	0.024364	0.99966	0.81002	-0.30397
881.7964	0.52	domics Pos	0.57699	0.99995	1.076	0.10573	0.95112	0.99979	1.0124	0.017795	0.33379	0.92074	0.95008	-0.07387	0.18508	0.99996	0.89547	-0.15928	0.9801	0.99701	1.0108	0.015493	0.71174	0.99959	1.025	0.035676	0.018114	0.17847	1.3043	0.38323	0.46838	0.99984	0.93788	-0.09252	0.75539	0.99966	1.0451	0.063616
247.9878	12.01	olomics Ne	0.16568	0.9951	0.79419	-0.33245	0.54297	0.99975	0.9108	-0.1348	0.019456	0.99731	0.62825	-0.67059	0.6575	0.99915	0.9357	-0.09588	0.36878	0.75206	0.87126	-0.19882	0.30982	0.69825	0.85209	-0.23093	0.45791	0.56482	0.88258	-0.1802	0.25858	0.99481	0.82631	-0.27524	0.12219	0.91738	0.79117	-0.33793
156.133	7.02	olomics Pc	0.020595	0.86976	0.80999	-0.30402	0.065277	0.97915	0.71771	-0.47853	0.003535	0.43007	0.7721	-0.37314	0.26671	0.99987	0.87931	-0.18555	0.16968	0.95986	0.8524	-0.2304	0.16931	0.99796	0.7633	-0.38968	0.87745	0.99768	0.99074	-0.01342	0.10222	0.9981	0.71886	-0.47622	0.029412	0.99995	0.82786	-0.27253
387.9771	4.62	olomics Ne	0.095819	0.9951	1.0803	0.11146	0.20094	0.99975	1.0846	0.11718	0.33353	0.99731	1.0359	0.050919	0.066398	0.99915	1.1073	0.147	0.075624	0.5958	1.123	0.16737	0.044203	0.49377	1.0988	0.13593	0.017153	0.069918	1.2758	0.35135	0.3258	0.99481	1.069	0.096276	0.56575	0.96658	0.97128	-0.04204
907.6757	6.41	domics Pos	0.14316	0.99995	1.4331	0.51918	0.95858	0.99979	0.9485	-0.07628	0.30266	0.89817	1.3543	0.43757	0.62154	0.99996	1.169	0.22528	0.35473	0.99701	1.2449	0.31601	0.40316	0.99959	0.80707	-0.30923	0.021042	0.18176	1.7477	0.80546	0.31362	0.99984	1.2776	0.3534	0.38586	0.99966	1.1472	0.19817
632.5216	2.05	domics Pos	0.031801	0.91043	0.78253	-0.35379	0.002455	0.99441	0.67039	-0.57694	0.022131	0.32086	0.72586	-0.46225	0.21581	0.99996	0.8029	-0.31671	0.017577	0.49574	0.7495	-0.416	0.044393	0.99959	0.71069	-0.4927	0.26313	0.47996	1.1316	0.17835	0.054927	0.99984	0.67569	-0.56557	0.085307	0.99966	0.81193	-0.30058
125.0967	2.05	domics Pos	0.010893	0.68962	0.83982	-0.25185	0.03812	0.99441	0.73674	-0.44077	0.000179	0.13107	0.75361	-0.40811	0.30938	0.99996	0.8791	-0.1859	0.004547	0.46849	0.80309	-0.31636	0.089405	0.99959	0.76124	-0.39358	0.17231	0.38396	1.1833	0.24277	0.15765	0.99984	0.79604	-0.32909	0.039392	0.99966	0.87457	-0.19335
667.5742	2.05	domics Pos	0.008919	0.68962	0.68154	-0.55312	0.074922	0.99979	0.68763	-0.5403	0.016114	0.28182	0.61986	-0.68998	0.15039	0.99996	0.76921	-0.37856	0.014991	0.4899	0.62824	-0.67062	0.030462	0.99959	0.60034	-0.73615	0.64229	0.79267	1.0656	0.091708	0.011928	0.99984	0.51085	-0.96904	0.099418	0.99966	0.76367	-0.38897
750.5423	5.18	domics Pos	0.51873	0.99995	1.0579	0.081225	0.18626	0.99979	1.1003	0.13789	0.60102	0.99601	0.95387	-0.06813	0.48897	0.99996	1.06	0.084033	0.84669	0.99701	1.0159	0.022726	0.087646	0.99959	1.1393	0.18821	0.011384	0.16313	1.5089	0.59348	0.14963	0.99984	1.1348	0.18244	0.01975	0.99966	1.3333	0.41502
378.8149	0.44	olomics Ne	0.69066	0.9951	1.0444	0.062695	0.033408	0.99975	1.2973	0.37551	0.80711	0.99731	0.98176	-0.02655	0.16299	0.99915	1.143	0.19286	0.20088	0.69888	1.2553	0.32805	0.072839	0.5223	1.1566	0.20987	0.29254	0.40876	1.136	0.18396	0.1629	0.99481	1.1573	0.2108	0.32827	0.91738	1.122	0.16612
259.9629	0.58	domics Pos	0.5875	0.99995	1.1047	0.14366	0.73086	0.99979	1.0316	0.044899	0.079716	0.62032	0.73443	-0.44529	0.38851	0.99996	0.85475	-0.22642	0.43334	0.99701	0.85635	-0.22373	0.14996	0.99959	1.2585	0.33165	0.041262	0.20971	1.6599	0.73107	0.20431	0.99984	0.79027	-0.33959	0.4706	0.99966	0.88039	-0.18378
114.0926	6.38	olomics Pc	0.030743	0.98816	0.85378	-0.22807	0.060669	0.93223	0.77028	-0.37655	0.037095	0.89885	0.83835	-0.25437	0.50713	0.99987	0.94474	-0.08201	0.092402	0.95986	0.878	-0.1877	0.24761	0.99796	0.85512	-0.2258	0.27828	0.99768	1.0699	0.097439	0.11705	0.9981	0.80137	-0.31946	0.08013	0.99995	0.85623	-0.22393
163.1493	6.38	olomics Pc	0.026648	0.9464	0.91117	-0.13421	0.017553	0.88953	0.80034	-0.32131	0.011649	0.59292	0.89306	-0.16318	0.54681	0.99987	0.94944	-0.07486	0.29227	0.95986	0.94371	-0.08358	0.078341	0.99796	0.79506	-0.33087	0.45588	0.99768	1.0368	0.052127	0.057004	0.9981	0.84379	-0.24505	0.10837	0.99995	0.86574	-0.208
1044.798	10.02	domics Pos	0.24793	0.99995	0.9403	-0.08881	0.75421	0.99979	1.0208	0.029647	0.014184	0.26127	0.88285	-0.17977	0.67708	0.99996	0.97679	-0.03388	0.075759	0.7908	0.91217	-0.13263	0.39617	0.99959	0.93964	-0.08982	0.083152	0.27417	1.169	0.22525	0.2933	0.99984	0.93608	-0.09529	0.14619	0.99966	0.91303	-0.13127
259.2756	1.91	domics Pos	0.87514	0.99995	1.0137	0.019687	0.27919	0.99979	0.82556	-0.27656	0.34611	0.92979	0.87599	-0.19101	0.7958	0.99996	0.97934	-0.03012	0.66761	0.99701	1.0592	0.082971	0.32211	0.99959	0.8724	-0.19693	0.016644	0.17568	1.3603	0.44389	0.78119	0.99984	0.97471	-0.03695	0.72631	0.99966	0.96421	-0.05258
602.9721	12.01	olomics Ne	0.24704	0.9951	1.157	0.21034	0.086464	0.99975	1.2622	0.33598	0.71494	0.99731	1.0269	0.038252	0.21581	0.99915	1.1866	0.24685	0.80572	0.94834	0.98503	-0.02176	0.011515	0.45618	1.2108	0.276	0.034249	0.10135	1.2354	0.30499	0.48081	0.99481	1.0737	0.10253	0.55634	0.96586	0.95789	-0.06206
255.2514	1.47	domics Pos	0.009448	0.68962	0.91093	-0.13458	0.05086	0.99441	0.78719	-0.34522	0.001162	0.13107	0.78665	-0.34621	0.37046	0.99996	0.89422	-0.1613	0.019872	0.5336	0.81842	-0.28909	0.18201	0.99959	0.82351	-0.28015	0.052681	0.23013	1.2288	0.29726	0.18567	0.99984	0.80438	-0.31405	0.059409	0.99966	0.87957	-0.18512
631.5245	2.05	domics Pos	0.080992	0.99995	0.77305	-0.37136	0.019689	0.99441	0.70602	-0.50222	0.005178	0.16899	0.71116	-0.49175	0.34875	0.99996	0.87335	-0.19537	0.26746	0.99701	0.87209	-0.19744	0.3308	0.99959	0.85456	-0.22675	0.061855	0.24567	1.3165	0.39668	0.11393	0.99984	0.76157	-0.39296	0.30623	0.99966	0.88731	-0.17249
151.1479	6	olomics Pc	0.15694	0.99964	0.9179	-0.12359	0.026101	0.88953	0.74135	-0.43178	0.11219	0.99882	0.88511	-0.17607	0.22175	0.99987	0.8806	-0.18344	0.49638	0.95986	0.95475	-0.06681	0.15891	0.99796	0.75872	-0.39835	0.29455	0.99768	1.0805	0.11169	0.088786	0.9981	0.824	-0.27928	0.10207	0.99995	0.85679	-0.22299
95.0862	1.66	domics Pos	0.011618	0.68962	0.82962	-0.26947	0.019143	0.99441	0.73615	-0.44192	0.011248	0.22935	0.73317	-0.44778	0.2147	0.99996	0.83377	-0.26228	0.009625	0.46849	0.76551	-0.38551	0.1098	0.99959	0.75488	-0.40567	0.14123	0.34951	1.1692	0.22552	0.21305	0.99984	0.8129	-0.29884	0.043617	0.99966	0.873	-0.19594
189.1643	1.66	domics Pos	0.007961	0.68962	0.84277	-0.24679	0.029991	0.99441	0.70114	-0.51222	0.000301	0.13107	0.72404	-0.46586	0.31713	0.99996	0.87064	-0.19985	0.008117	0.46849	0.76936	-0.37827	0.056554	0.99959	0.7346	-0.44497	0.42069	0.63908	1.1	0.13755	0.13881	0.99984	0.77314	-0.3712	0.063891	0.99966	0.88318	-0.17921
173.149	2.05	domics Pos	0.022631	0.81406	0.82657	-0.27479	0.06155	0.99979	0.75823	-0.39928	0.005454	0.17146	0.77749	-0.3631	0.27974	0.99996	0.88825	-0.17096	0.06359	0.76594	0.8432	-0.24606	0.041644	0.99959	0.77141	-0.37443	0.24496	0.46432	1.1398	0.18881	0.25685	0.99984	0.83903	-0.25321	0.13916	0.99966	0.88441	-0.17721
380.9772	4.61	olomics Ne	0.37359	0.9951	1.0377	0.053351	0.74038	0.99975	1.0137	0.019647	0.80746	0.99731	0.98899	-0.01598	0.26901	0.99915	1.0503	0.070837	0.1924	0.69001	1.0917	0.12657	0.037307	0.4612	1.1461	0.19679	0.001684	0.041313	1.2482	0.31987	0.16481	0.99481	1.0694	0.096819	0.51457	0.95528	0.96533	-0.05091

216.9234	10.1	domics Pos	0.97444	0.99995	1.0052	0.007416	0.80845	0.99979	1.0146	0.020869	0.007723	0.19873	0.92618	-0.11064	0.82799	0.99996	0.99108	-0.01292	0.18093	0.95949	0.94585	-0.08031	0.59865	0.99959	0.97213	-0.04078	0.024836	0.18846	1.2308	0.29965	0.70231	0.99984	0.97916	-0.03038	0.61793	0.99966	1.034	0.048266
444.3407	1.64	domics Pos	0.25697	0.99995	1.1876	0.24799	0.78312	0.99979	0.98244	-0.02556	0.69374	0.99601	0.97176	-0.04133	0.82519	0.99996	0.99189	-0.01175	0.49137	0.99701	0.93273	-0.10047	0.76684	0.99959	0.97942	-0.03	0.024861	0.18846	1.3268	0.408	0.38344	0.99984	0.93173	-0.10202	0.91429	0.99966	1.0151	0.021664
545.2864	0.56	domics Pos	0.90138	0.99995	1.0157	0.022429	0.22959	0.99979	0.82138	-0.28388	0.026897	0.34652	0.6711	-0.57541	0.74893	0.99996	1.0693	0.096702	0.092242	0.82209	0.68702	-0.54158	0.23386	0.99959	0.81031	-0.30345	0.7585	0.85999	1.3349	0.41678	0.73155	0.99984	0.97479	-0.03683	0.92785	0.99966	1.1537	0.20629
418.9771	12	olomics Ne	0.38162	0.9951	1.0573	0.080425	0.19264	0.99975	1.0808	0.11209	0.6373	0.99731	1.0263	0.037429	0.63759	0.99915	1.0657	0.091752	0.0103	0.50209	1.1442	0.19433	0.26356	0.66079	1.1017	0.13969	0.000932	0.041313	1.4507	0.53675	0.093926	0.99481	1.1494	0.20088	0.68742	0.97023	1.0414	0.058589
299.2377	1.66	domics Pos	0.015274	0.68962	0.77654	-0.36487	0.021598	0.99441	0.61182	-0.70883	0.022493	0.32122	0.69446	-0.52603	0.20407	0.99996	0.7902	-0.33972	0.021432	0.53763	0.71773	-0.4785	0.12922	0.99959	0.65771	-0.60448	0.0918	0.28857	1.2317	0.30068	0.13809	0.99984	0.69236	-0.5304	0.048795	0.99966	0.78818	-0.3434
974.8129	10.02	domics Pos	0.25484	0.99995	1.0595	0.083374	0.37096	0.99979	1.056	0.078617	0.1632	0.78771	0.96771	-0.04735	0.70919	0.99996	1.034	0.048266	0.77257	0.99701	0.98891	-0.01609	0.83828	0.99959	0.99153	-0.01228	0.048753	0.2237	1.2378	0.30777	0.87751	0.99984	1.0181	0.02594	0.35993	0.99966	1.0771	0.10719
255.945	10.26	domics Pos	0.21305	0.99995	1.1608	0.21516	0.55515	0.99979	1.0702	0.097946	0.65488	0.99601	1.0489	0.068855	0.60435	0.99996	1.0485	0.068344	0.95297	0.99701	1.0062	0.008848	0.8619	0.99959	0.99723	-0.004	0.009135	0.1521	1.2912	0.36874	0.56422	0.99984	1.0751	0.10454	0.7764	0.99966	1.0437	0.06174
213.2031	2.05	domics Pos	0.030916	0.90678	0.78232	-0.35417	0.04519	0.99441	0.75449	-0.40642	0.006488	0.18057	0.7242	-0.46555	0.29136	0.99996	0.85338	-0.22873	0.002852	0.46849	0.72573	-0.4625	0.055253	0.99959	0.75829	-0.39918	0.22001	0.43953	1.1708	0.2275	0.094269	0.99984	0.75867	-0.39846	0.080259	0.99966	0.88134	-0.18223
954.7573	10.06	domics Pos	0.13296	0.99995	1.1573	0.21081	0.89977	0.99979	0.99071	-0.01347	0.97935	0.99702	0.99353	-0.00936	0.63583	0.99996	1.0428	0.060406	0.42057	0.99701	1.1087	0.14892	0.79972	0.99959	0.97719	-0.0333	0.047137	0.22017	1.3087	0.38811	0.34773	0.99984	1.1641	0.21917	0.57672	0.99966	1.0509	0.071618
218.9869	12.02	olomics Ne	0.34589	0.9951	0.95744	-0.06274	0.40976	0.99975	1.0584	0.081913	0.451	0.99731	0.96276	-0.05475	0.37273	0.99915	1.0426	0.060228	0.40055	0.76683	1.0414	0.058456	0.42504	0.77401	1.0542	0.076089	0.012499	0.062293	1.1779	0.23623	0.91346	0.99481	0.9937	-0.00912	0.20983	0.91738	0.9235	-0.11482
804.8419	10.02	domics Pos	0.3925	0.99995	1.0813	0.11282	0.81104	0.99979	0.98727	-0.01849	0.26658	0.88313	0.91025	-0.13566	0.47233	0.99996	1.0548	0.077008	0.33495	0.99701	0.94263	-0.08524	0.91396	0.99959	1.0375	0.053069	0.000137	0.060994	1.3799	0.46459	0.5857	0.99984	0.96043	-0.05825	0.51042	0.99966	1.0952	0.13116
567.8785	0.5	domics Pos	0.86593	0.99995	1.0129	0.018551	0.41118	0.99979	0.95143	-0.07184	0.00493	0.16782	0.84618	-0.24097	0.4865	0.99996	0.93188	-0.10178	0.12727	0.86468	0.88312	-0.17932	0.033527	0.99959	0.90039	-0.15138	0.19864	0.41243	1.2348	0.3043	0.70903	0.99984	0.97382	-0.03827	0.84282	0.99966	1.0441	0.062279
450.8789	6.37	polomics Pc	0.006644	0.59798	0.76008	-0.39577	0.17002	1	0.76573	-0.3851	0.09622	0.99882	0.85478	-0.22638	0.84001	0.99987	1.0499	0.070222	0.092396	0.95986	0.82842	-0.27156	0.17698	0.99796	0.7554	-0.40469	0.051839	0.77855	1.1323	0.17926	0.066804	0.9981	0.69411	-0.52677	0.007289	0.99995	0.7949	-0.33116
199.1888	1.66	domics Pos	0.046219	0.99995	0.85173	-0.23154	0.006338	0.99441	0.67612	-0.56465	0.003097	0.15208	0.70969	-0.49474	0.055402	0.99996	0.7341	-0.44595	0.077028	0.79327	0.77759	-0.36291	0.14527	0.99959	0.74618	-0.4224	0.53714	0.73226	1.0627	0.087667	0.11977	0.99984	0.80472	-0.31345	0.10293	0.99966	0.80906	-0.30569
399.9814	12	olomics Ne	0.10114	0.9951	0.81412	-0.29668	0.60779	0.99975	1.0438	0.061842	0.79066	0.99731	0.96904	-0.04538	0.016527	0.99915	0.72801	-0.45797	0.27662	0.71896	0.87591	-0.19115	0.82251	0.94627	0.97246	-0.04029	0.4139	0.5199	1.1428	0.19255	0.58452	0.99481	0.9308	-0.10345	0.11895	0.91738	0.8044	-0.31401
610.9737	12.01	olomics Ne	0.23812	0.9951	1.0972	0.13385	0.26899	0.99975	1.1572	0.21063	0.29216	0.99731	1.1862	0.24632	0.18501	0.99915	1.1159	0.15825	0.19995	0.69888	1.1424	0.19203	0.37212	0.75412	1.127	0.17246	0.043031	0.11566	1.4516	0.53761	0.43472	0.99481	1.0967	0.13316	0.97051	0.99833	1.0162	0.023204
213.2047	6.38	polomics Pc	0.01903	0.86976	0.8238	-0.27963	0.044252	0.88953	0.72136	-0.47121	0.006104	0.51418	0.81288	-0.29888	0.68262	0.99987	0.96825	-0.04655	0.030379	0.95986	0.83956	-0.25229	0.12237	0.99796	0.81252	-0.29952	0.38757	0.99768	1.0427	0.060382	0.14735	0.9981	0.82918	-0.27025	0.059452	0.99995	0.86605	-0.20748
107.0857	2.05	domics Pos	0.028696	0.89298	0.85575	-0.22475	0.034973	0.99441	0.76174	-0.39263	0.13736	0.74334	0.83803	-0.25492	0.29353	0.99996	0.87296	-0.19601	0.11289	0.85684	0.8301	-0.26865	0.10261	0.99959	0.75441	-0.40659	0.25892	0.47615	1.1819	0.24108	0.15244	0.99984	0.78093	-0.35673	0.04624	0.99966	0.81749	-0.29072
490.9758	5.3	olomics Ne	0.1059	0.9951	1.1616	0.21608	0.001834	0.75238	1.1782	0.23662	0.096311	0.99731	1.1094	0.14981	0.078844	0.99915	1.1091	0.14939	0.003581	0.34325	1.1749	0.2325	0.077844	0.5223	1.1582	0.21185	0.001809	0.04149	1.3399	0.42217	0.022787	0.99481	1.1019	0.13996	0.29266	0.91738	1.0184	0.026364
469.4038	9.75	domics Pos	0.21468	0.99995	1.4238	0.50971	0.36284	0.99979	1.1389	0.18758	0.659	0.99601	1.0372	0.052651	0.21291	0.99996	1.1648	0.22006	0.431	0.99701	1.0977	0.13451	0.21791	0.99959	1.2423	0.313	0.015209	0.17082	1.4638	0.54972	0.52434	0.99984	1.1141	0.15582	0.004043	0.99749	1.5984	0.67665
562.9745	12.01	olomics Ne	0.017912	0.9951	1.1941	0.25587	0.45134	0.99975	1.0951	0.13108	0.53031	0.99731	1.0511	0.071903	0.7712	0.99915	1.0436	0.061564	0.56324	0.86442	1.0686	0.095754	0.36321	0.74467	1.0769	0.10687	0.10077	0.18997	1.1939	0.2557	0.35336	0.99481	1.1402	0.18933	0.025702	0.91738	0.84409	-0.24454
196.1708	5.99	polomics Pc	0.084572	0.99964	0.89443	-0.16096	0.037413	0.88953	0.78969	-0.34064	0.034702	0.89167	0.83214	-0.2651	0.84669	0.99987	0.9899	-0.01465	0.37145	0.95986	0.94183	-0.08646	0.38695	0.99796	0.89622	-0.15807	0.2946	0.99768	1.082	0.11365	0.061846	0.9981	0.7941	-0.33261	0.2041	0.99995	0.90321	-0.14687
166.1659	1.66	domics Pos	0.1683	0.99995	0.9011	-0.15024	0.031185	0.99441	0.70761	-0.49896	0.004907	0.16782	0.72795	-0.45809	0.20897	0.99996	0.87611	-0.19082	0.095592	0.82209	0.79747	-0.3265	0.34548	0.99959	0.85571	-0.2248	0.041896	0.21081	1.2475	0.31903	0.10466	0.99984	0.74115	-0.43217	0.057221	0.99966	0.80929	-0.30527
692.9727	12	olomics Ne	0.18086	0.9951	1.2078	0.27244	0.13718	0.99975	1.1783	0.23675	0.45345	0.99731	0.90935	-0.1371	0.1987	0.99915	1.227	0.29518	0.21718	0.70622	1.1675	0.22342	0.045288	0.49377	1.2497	0.32162	0.24075	0.35408	1.1249	0.16974	0.24363	0.99481	1.1114	0.15239	0.658	0.96789	0.94725	-0.07819
684.9731	12.02	olomics Ne	0.43793	0.9951	1.0755	0.10504	0.4631	0.99975	1.0832	0.11534	0.94162	0.99731	0.97951	-0.02987	0.18836	0.99915	1.2517	0.32387	0.33871	0.74566	1.1076	0.14745	0.17616	0.60765	1.2145	0.28034	0.025956	0.086591	1.4521	0.53812	0.68068	0.99481	0.94539	-0.08102	0.37443	0.92651	1.1533	0.20572
207.2098	2.05	domics Pos	0.012303	0.68962	0.82838	-0.27163	0.02737	0.99441	0.73728	-0.43971	0.001066	0.13107	0.69217	-0.5308	0.42688	0.99996	0.9137	-0.1302	0.12	0.86273	0.84752	-0.23868	0.36873	0.99959	0.91835	-0.12289	0.038165	0.20439	1.308	0.38736	0.13879	0.99984	0.76718	-0.38235	0.5493	0.99966	0.95458	-0.06706
783.9752	12.03	olomics Ne	0.9266	0.9951	1.0313	0.044422	0.18236	0.99975	1.1934	0.25508	0.8315	0.99731	0.99611	-0.00562	0.87308	0.99915	1.01	0.014332	0.38395	0.75206	0.91297	-0.13137	0.093743	0.52866	0.834	-0.26187	0.025837	0.086526	1.3677	0.45172	0.86945	0.99481	1.035	0.049594	0.22069	0.91738	0.90818	-0.13894
139.113	6.38	polomics Pc	0.029731	0.98592	0.88737	-0.17239	0.014567	0.88953	0.79887	-0.32396	0.035395	0.89167	0.88881	-0.17005	0.60133	0.99987	0.95566	-0.06543	0.45977	0.95986	0.95588	-0.0651	0.076186	0.99796	0.78559	-0.34815	0.82156	0.99768	1.0134	0.019189	0.10497	0.9981	0.85089	-0.23296	0.080402	0.99995	0.87007	-0.2008
737.5803	2.05	domics Pos	0.40527	0.99995	0.88215	-0.1809	0.089333	0.99979	0.70711	-0.5	0.10463	0.68915	0.77568	-0.36646	0.82859	0.99996	1.0049	0.007109	0.034363	0.60942	0.78957	-0.34086	0.10807	0.99959	0.7294	-0.45522	0.061439	0.24567	1.2846	0.36134	0.3593	0.99984	0.8414	-0.24914	0.92605	0.99966	1.0228	0.032474
394.9779	4.47	olomics Ne	0.8157	0.9951	1.0104	0.014908	0.22259	0.99975	1.0469	0.0661	0.94447	0.99731	0.99771	-0.00331	0.41368	0.99915	1.0535	0.075215	0.21616	0.70622	1.053	0.074469	0.022956	0.4612	1.1221	0.1662	0.01143	0.062031	1.2245	0.29223	0.27657	0.99481	1.0479	0.067455	0.35974	0.92651	0.95409	-0.06781
207.1749	1.66	domics Pos	0.012505	0.68962	0.8611	-0.21575	0.054654	0.99441	0.7801	-0.35828	0.005701	0.17488	0.76613	-0.38434	0.45297	0.99996	0.92214	-0.11695	0.014665	0.4899	0.85469	-0.22653	0.16059	0.99959	0.82231	-0.28225	0.11909	0.32077	1.2199	0.28675	0.091955	0.99984	0.75389	-0.40758	0.12107	0.99966	0.90309	-0.14705
869.6078	10.25	olomics Ne	0.82425	0.9951	1.0185	0.026495	0.55899	0.99975	1.0395	0.055953	0.34546	0.99731	0.92995	-0.10478	0.74926	0.99915	1.0182	0.026052	0.37773	0.75206	1.0777	0.10795	0.27838	0.6688	1.0852	0.11798	0.016992	0.06956	1.2402	0.31059	0.59386	0.99481	1.0328	0.046511	0.93947	0.99833	0.99117	-0.01279
372.9824	4.52	olomics Ne	0.6991	0.9951	0.98017	-0.02889	0.7765	0.99975	1.0145	0.020767	0.12609	0.99731	0.92256	-0.11628	0.82649	0.99915	1.0145	0.020749	0.57226	0.86442	1.0342	0.048584	0.22796	0.6407	1.0853	0.11808	0.006486	0.057338	1.2521	0.32439	0.43799	0.99481	1.05	0.070335	0.15612	0.91738	0.90343	-0.14652
530.9668	3.28	polomics Pc	0.92139	0.99964	0.99928	-0.00103	0.79004	1	1.0578	0.081122	0.20934	0.99882	0.8149	-0.29531	0.72838	0.99987	1.1592	0.21317	0.041265	0.95986	0.69497	-0.52498	0.68742	0.99796	0.94616	-0.07984	0.81824	0.99768	1.1658	0.22134	0.48993	0.9981	0.90102	-0.15037	0.7215	0.99995	0.98214	-0.02601
352.9762	4.48	olomics Ne	0.89473	0.9951	1.0059	0.008448	0.97293	0.99975	1.0023	0.00328	0.61232	0.99731	0.9794	-0.03003	0.91577	0.99915	1.006	0.008621	0.091118	0.5958	1.078	0.10838	0.087163	0.52866	1.1048	0.14381	0.005992	0.05505	1.2007	0.26387	0.25388	0.99481	1.0427	0.060287	0.27515	0.91738	0.94063	-0.0883
167.1444	5.99	polomics Pc	0.083845	0.99964	0.89483	-0.16031	0.034351	0.88953	0.74231	-0.42991	0.006773	0.51418	0.7776	-0.3629	0.56393	0.99987	0.95006	-0.07392	0.12434	0.95986	0.82459	-0.27825	0.11332	0.99796	0.77686	-0.36428	0.80714	0.99768	0.98508	-0.02168	0.11197	0.9981	0.74734	-0.42016	0.012732	0.99995	0.79708	-0.3272
433.9822	12.01	olomics Ne	0.04366	0.9951	0.76566	-0.38523	0.86327	0.99975	0.98525	-0.02144	0.90397	0.99731	1.0286	0.040628	0.64252	0.99915	0.95663	-0.06397	0.86879	0.96786	1.0137	0.019684	0.50437	0.79937	1.0461	0.065049	0.87958	0.91303	1.0213	0.030342	0.50185	0.99481	0.91625	-0.12619	0.64739	0.96789	0.95252	-0.07018
297.2954	6.74	polomics Pc	0.13864	0.99964	0.9001	-0.15184	0.091302	1	0.74061	-0.43321	0.049804	0.99882	0.81849	-0.28896	0.16765	0.99987	0.88758	-0.17205	0.18765	0.95986	0.90249	-0.14802	0.1334	0.99796	0.78933	-0.34131	0.68514	0.99768	0.96668	-0.04889	0.10922	0.9981	0.76834	-0.38019	0.14969	0.99995	0.86065	-0.21651
650.8481	10.09	domics Pos	0.89439	0.99995	0.99562	-0.00633	0.7483	0.99979	1.0332	0.04705	0.19316	0.82046	0.93175	-0.10198	0.97085	0.99996	0.99946	-0.00078	0.74081	0.99701	0.98003	-0.0291	0.86223	0.99959	0.99108	-0.01293	0.020411	0.18119	1.2557	0.32847	0.48159	0.99984	0.93762	-0.09293	0.87751	0.99966	0.99061	-0.01362
452.9913	5.07	olomics Ne	0.73677	0.9951	1.0136	0.019548	0.73742	0.99975	1.017	0.024384	0.41847	0.99731	0.95985	-0.05912	0.25116	0.99915	1.0687	0.095853	0.038438	0.53488	1.1046	0.14348	0.10854	0.53696	1.1304	0.17683	0.005574	0.052776	1.2711	0.34606	0.30256	0.99481	1.04	0.056652	0.79353	0.97489	0.98629	-0.01992
278.2469	1.01	domics Pos	0.48577	0.99995	0.93807	-0.09223	0.21083	0.99979	0.87634	-0.19043	0.018682	0.30022	0.77114	-0.37493	0.51437	0.99996	0.92279	-0.11592	0.026543	0.55306	0.80924	-0.30536	0.1917	0.99959	0.84318	-0.24609	0.222	0.44156	1.2888	0.36599	0.11392	0.99984	0.79569	-0.32972	0.28015	0.99966	0.89701	-0.15681
969.7784	0.53	domics Pos	0.32445	0.99995	0.94828	-0.07661	0.2425	0.99979	0.9291	-0.10609	0.12572	0.72566	0.86856	-0.20331	0.11648	0.99996	0.88144	-0.18207	0.016803	0.49216	0.88105	-0.1827	0.60496	0.99959	0.96633	-0.04941	0.48311	0.68941	1.0426	0.060119	0.15871	0.99984	0.86094	-0.21601	0.053896	0.99966	0.85205	-0.231
152.0378	10.16	domics Pos	0.44374	0.99995	1.1366	0.18477	0.23598	0.99979	1.1431	0.19291	0.93156	0.99702	1.0025	0.003556	0.047993	0.99996	1.1789	0.23739	0.16165	0.93665	1.096	0.13223	0.9624	0.99959	1.0043	0.006121	0.003593	0.11893	1.3983	0.48367	0.23593	0.99984	1.2222	0.28946	0.19258	0.99966	1.0962	0.13249
227.22	2.05	domics Pos	0.004585	0.60743	0.88183	-0.18143	0.069147	0.99979	0.75892	-0.39798	0.001286	0.13107	0.74541	-0.4239	0.13717	0.99996	0.83033	-0.26824	0.006547	0.46849	0.76384	-0.38865	0.13304	0.99959	0.78283	-0.35322	0.36796	0.59046	1.1594	0.21337	0.19527	0.99984	0.80092	-0.32028	0.058879	0.99966	0.88412	-0.17769
425.9823	4.78	olomics Ne	0.68882	0.9951	1.04	0.056629	0.27907	0.99975	1.0206	0.029423	0.40256	0.99731	1.0282	0.040059	0.002112	0.99915	1.1057	0.14498	0.057411	0.59097	1.1488	0.20012	0.16101	0.59931	1.1158	0.1581	0.001661	0.041313	1.2736	0.34897	0.027942	0.99481	1.1377	0.18613	0.45902	0.94605	0.96497	-0.05145
199.1883	2.05	domics Pos	0.22192	0.99995	0.87395	-0.19437	0.17468	0.99979	0.8001	-0.32176	0.050358	0.4928	0.75269	-0.40988	0.53618	0.99996	0.92491	-0.11262	0.11219	0.85684	0.81689	-0.29178	0.27065	0.99959	0.84436	-0.24407	0.040799	0.20898	1.3221	0.40286	0.15988	0.99984	0.77027	-0.37656	0.80542	0.99966	0.97174	-0.04136
169.0492	10.17	domics Pos	0.55133	0.99995	1.0333	0.047254	0.29892	0.99979	1.0407	0.05753	0.1953	0.82468	0.91608	-0.12646	0.40669	0.99996	1.1022	0.14043	0.10182	0.83496	0.92621	-0.11058	0.48706	0.99959	1.0781	0.10851	0.02038	0.18119	1.3153	0.39535	0.96061	0.99984	1.0011	0.001592	0.70327	0.99966	1.0492	0.069243
670.3258	0.57	domics Pos	0.53286	0.99995	0.88693	-0.17311	0.036132	0.99441	0.66172	-0.59571	0.043837	0.45176	0.65208	-0.61687	0.83467	0.99996	1.1393	0.18817	0.3381	0.99701	0.79288	-0.33483	0.6457	0.99959	0.91408	-0.1296	0.59069	0.76401	1.5727	0.65322	0.77637	0.99984	0.95156	-0.07164	0.90321	0.99966	1.0728	0.10141
167.1436	2.05	domics Pos	0.003152	0.59581	0.81641	-0.29264	0.049605	0.99441	0.75457	-0.40627	0.005087	0.16899	0.71652	-0.48091	0.24372	0.99996	0.863	-0.21257	0.012506	0.48469	0.80186	-0.31859	0.1007	0.99959	0.75354	-0.40825	0.12893	0.33033	1.1841	0.24383	0.15876	0.99984	0.76656	-0.38353	0.080132	0.99966	0.87517	-0.19237
1072.776	10.02	domics Pos	0.74199	0.99995	0.97654	-0.03425	0.74944	0.99979	1.0528	0.074163	0.40044	0.9525	0.94749	-0.07781	0.77546	0.99996	0.98005	-0.02907	0.66572	0.99701	0.97223	-0.04062	0.46417	0.99959	0.94324	-0.08431	0.023356	0.18728	1.3026	0.38135	0.61252	0.99984	0.95646	-0.06422	0.076904	0.99966	1.1922	0.25365
136.1212	6.38	polomics Pc	0.027495	0.9464	0.85468	-0.22655	0.000988	0.88953	0.71157	-0.49093	0.033472	0.89167	0.82642	-0.27506	0.16774	0.99987	0.87577	-0.19137	0.062845	0.95986	0.87105	-0.19917	0.028487	0.99796	0.69576	-0.52334	0.95134	0.99768	0.99357	-0.0093	0.10864	0.9981	0.77837	-0.36147	0.054233	0.99995	0.80244	-0.31754
170.155	5.99	polomics Pc	0.011343	0.71461	0.83665	-0.2573	0.039877	0.88953	0.74401	-0.4266	0.017184	0.63482	0.81068	-0.3028	0.54431	0.99987	0.94367	-0.08365	0.13147	0.95986	0.88458	-0.17693	0.15568	0.99796	0.79992	-0.32208	0.44041	0.99768	1.0431	0.060858	0.10843	0.9981	0.78617	-0.34708	0.028863	0.99995	0.84529	-0.24248
863.6938	6.41	domics Pos	0.068032	0.99995	1.3903	0.47543	0.92024	0.99979	1.0215	0.030628	0.49333	0.99601	1.1342	0.18165	0.30637	0.99996	1.1331	0.18033	0.18519	0.96528	1.2586	0.33185	0.96976	0.99959	1.039	0.055247	0.011955	0.16313	1.4333	0.51932	0.66964	0.99984	1.0664	0.092767	0.82409	0.99966	1.0608	0.085147
96.0897	2.05	domics Pos	0.037531	0.96043	0.72801	-0.45798	0.37582	0.99979	0.86013	-0.21737	0.05813	0.53281	0.72736	-0.45926	0.58891	0.99996	0.91755	-0.12414	0.030573	0.58579	0.65534	-0.60968	0.047277	0.99959	0.72181	-0.4703	0.68488	0.81761	1.057	0.080031	0.14262	0.99984	0.6929	-0.52927	0.38863	0.99966	0.88551	-0.17541
170.1545	7.02	polomics Pc	0.24585	0.99964	0.92725	-0.10897	0.076164	0.99372	0.74398	-0.42667	0.022479	0.70809	0.8239	-0.27946	0.10733	0.99987	0.81353	-0.29773	0.4163	0.95986	0.93124	-0.10278	0.18676	0.99796	0.78043	-0.35765	0.015	0.45844	1.191	0.25213	0.17138	0.9981	0.82188	-0.28299	0.028034	0.99995	0.83506	-0.26005
955.5469	12.07	olomics Ne	0.064895	0.9951	1.2659	0.34019	0.56294	0.99975	1.0989	0.13602	0.52025	0.99731	1.08	0.11099	0.068374	0.99915	1.3577	0.44115	0.062108	0.5958	1.3288	0.41015	0.037466	0.4612	1.3153	0.39544	0.025721	0.086509	1.3545	0.43771	0.035687	0.99481	1.2884	0.36563	0.92239	0.99833	0.98417	-0.02302
478.9769	5.2	olomics Ne	0.25091	0.9951	1.0894	0.12347	0.70991	0.99975	1.0246	0.035051	0.47113	0.99731	1.0351	0.049824	0.46093	0.99915	1.0418	0.05911	0.044592	0.56591	1.0952	0.13116	0.019561	0.45618	1.1448	0.19508	0.002483	0.047017	1.2893	0.36657	0.32539	0.99481	1.053	0.07449	0.71941	0.97327	0.98321	-0.02443
157.142	1.66	domics Pos	0.061291	0.99995	0.7634	-0.38948	0.051807	0.99441	0.79674	-0.32781	0.028451	0.35921	0.7491	-0.41678	0.53525	0.99996	0.92026	-0.11988	0.097908	0.82209	0.75143	-0.41228	0.022553	0.99959	0.75016	-0.41472	0.4405	0.65632	1.1114	0.15233	0.15173	0.99984	0.77875	-0.36077	0.50928	0.99966	0.94649	-0.07934
1068.765	10.03	domics Pos	0.7547	0.99995	0.97427	-0.0376	0.28841	0.99979	1.0951	0.13104	0.71208	0.99601	0.97366	-0.03851	0.503	0.99996	1.1005	0.13821	0.50624	0.99701	1.0955	0.13162	0.37609	0.99959	1.1055	0.14475	0.023641	0.18728	1.303	0.38189	0.92106	0.99984	0.99755	-0.00355	0.58745	0.99966	1.0439	0.061922
269.2591	6.03	polomics Pc	0.10231	0.99964	0.88815	-0.17113	0.64473	1	0.95639	-0.06433	0.26548	0.99882	0.90361	-0.14622	0.65012	0.99987	0.9646	-0.052	0.99822	0.99906	1.0096	0.0138	0.048645	0.99796	0.78057	-0.3574	0.47869	0.99768	1.0458	0.064579	0.2923	0.9981	0.8545	-0.22684	0.31262	0.99995	0.91446	-0.12901
969.7983	10.02	domics Pos	0.67073	0.99995	0.96779	-0.04724	0.97259	0.99979	1.0056	0.008009	0.021929	0.32086	0.87065	-0.19984	0.31856	0.99996	0.92539	-0.11187	0.097901	0.82209	0.84518	-0.24267	0.79886	0.99959	0.98485	-0.02203	0.10327	0.30197	1.2045	0.26849	0.22349	0.99984	0.89089	-0.16667	0.96422	0.99966	1.0039	0.005676
196.1779	2.05	domics Pos	0.17862	0.99995	0.88918	-0.16945	0.32302	0.99979	0.87884	-0.18633	0.008148	0.19873	0.77288	-0.37168	0.74469	0.99996	0.97536	-0.03599	0.61685	0.99701	0.95851	-0.06113	0.43761	0.99959	0.90805	-0.13915	0.005514	0.1319	1.5343	0.6176	0.75774	0.99984	0.99257	-0.01076	0.86108	0.99966	0.99178	-0.01191
437.3462	7.63	polomics Pc	0.092986	0.99964	0.87034	-0.20034	0.04098	0.88953	1.1233	0.16778	0.20989	0.99882	0.93856	-0.09148	0.48483	0.99987	0.94321	-0.08435	0.6588	0.96792	0.96222	-0.05556	0.99473	0.99987	1.0087	0.012454	0.64579	0.99768	1.0492	0.069259	0.063305	0.9981	0.78733	-0.34495	0.30818	0.99995	1.1489	0.20026
453.8785	12.06	polomics Pc	0.8954	0.99964	1.0208	0.02963	0.52558	1	1.039	0.055239	0.33773	0.99882	1.0507	0.071395	0.40822	0.99987	1.0808	0.11212	0.27993	0.95986	1.1021	0.14026	0.54732	0.99796	1.0452	0.063782	0.029899	0.6314	1.186	0.24614	0.66051	0.9981	1.0326	0.046349	0.061704	0.99995	1.1057	0.14499
429.891	0.54	domics Pos	0.90844	0.99995	0.98001	-0.02914	0.17716	0.99979	0.79422	-0.33238	0.022278	0.32122	0.66806	-0.58194	0.61134	0.99996	0.98463	-0.02235	0.03273	0.59375	0.63921	-0.64565	0.22971	0.99959	0.80119	-0.31978	0.53823	0.7333	1.4641	0.55005	0.36444	0.99984	0.83556	-0.25918	0.86645	0.99966	1.0334	0.047344
839.8425	10.02	domics Pos	0.060629	0.99995	1.1416	0.19103	0.53102	0.99979	1.0483	0.068057	0.31193	0.9038	1.031	0.044025	0.12063	0.99996	1.1074	0.14721	0.54977	0.99701	1.0471	0.066446	0.60884	0.99959	1.0334	0.047397	0.012841	0.16313	1.3536	0.43676	0.79473	0.99984	1.0511	0.07187	0.34096	0.99966	1.0759	0.1056
676.214	1.09	domics Pos	0.86322	0.99995	0.98721	-0.01857	0.53039	0.99979	1.0868	0.12009	0.36051	0.93332	0.89759	-0.15588	0.29794	0.99996	1.1546	0.20743	0.50604	0.99701	1.1352	0.18292	0.006141	0.99959	1.3691	0.45324	0.006032	0.13681	1.4284	0.51436	0.78484	0.99984	1.0191	0.027291	0.0326	0.99966	1.3476	0.43044
139.1123	1.66	domics Pos	0.025723	0.85508	0.84966	-0.23505	0.002341	0.99441	0.70474	-0.50484	0.000683	0.13107	0.69135	-0.53252	0.18933	0.99996	0.8374	-0.25601	0.012528	0.48469	0.76318	-0.38991	0.074846	0.99959	0.72698	-0.46001	0.074375	0.26191	1.163	0.2178	0.070085	0.99984	0.74726	-0.42032	0.008441	0.99749	0.82611	-0.27559
556.9743	5.34	olomics Ne	0.40752	0.9951	1.0364	0.05161	0.94463	0.99975	0.9987	-0.00188	0.94781	0.99731	0.99767	-0.00337	0.36012	0.99915	1.0445	0.062761	0.12196	0.62697	1.0833	0.11548	0.23655	0.64348	1.0548	0.076987	0.004901	0.051563	1.2195	0.28631	0.20964	0.99481	1.043	0.060745	0.18751	0.91738	0.93374	-0.09891
316.0281	5.32	polomics Pc	0.030047	0.98592	0.82779	-0.27266	0.31895	1	0.93599	-0.09543	0.095079	0.99882	0.82468	-0.27809	0.49529	0.99987	0.93411	-0.09834	0.36045	0.95986	0.85589	-0.22451	0.38114	0.99796	0.90224	-0.14842	0.45222	0.99768	0.9319	-0.10175	0.019892	0.9981	0.76317	-0.38992	0.48413	0.99995	0.94625	-0.0797
125.0973	6.38	polomics Pc	0.065343	0.99964	0.91238	-0.1323	0.071397	0.99372	0.80928	-0.30529	0.013101	0.60269	0.91175	-0.13329	0.78225	0.99987	0.98732	-0.01841	0.61056	0.95986	0.96306	-0.0543	0.094996	0.99796	0.80639	-0.31045	0.72125	0.99768	1.0261	0.037205	0.07075	0.9981	0.80247	-0.31748	0.067536	0.99995	0.86306	-0.21247
538.3082	1.29	domics Pos	0.39057	0.99995	1.0982	0.1352	0.8916	0.99979	0.98969	-0.01494	0.031326	0.38225	0.80497	-0.31299	0.69889	0.99996	0.96162	-0.05647	0.16468	0.93861	0.85938	-0.21863	0.78631	0.99959	0.97476	-0.03688	0.049767	0.22586	1.2543	0.32686	0.54769	0.99984	0.93482	-0.09724	0.12771	0.99966	1.2019	0.26531
600.9754	12.01	olomics Ne	0.21469	0.9951	1.1784	0.2368	0.072122	0.99975	0.76482	-0.3868	0.36852	0.99731	0.90378	-0.14596	0.80712	0.99915	0.97071	-0.04289	0.30986	0.73412	1.118	0.16091	0.59415	0.83876	1.0679	0.094722	0.026585	0.087502	1.3636	0.44739	0.22481	0.99481	1.2041	0.26795	0.55392	0.96586	0.9154	-0.12752
132.139	9.96	domics Pos	0.77892	0.99995	1.0243	0.034636	0.93219	0.99979	0.99701	-0.00432	0.023342	0.32722	0.89688	-0.15702	0.89099	0.99996	1.0195	0.027794	0.40416	0.99701	0.95431	-0.06747	0.93584	0.99959	1.007	0.010054	0.035911	0.20439	1.1816	0.24077	0.31117	0.99984	0.93641	-0.09479	0.58148	0.99966	1.0354	0.050224
188.6371	6.2	polomics Pc	0.033108	0.99964	0.81995	-0.28639	0.050897	0.90372	0.74333	-0.42793	0.034254	0.89167	0.80609	-0.311	0.78284	0.99987	0.97932	-0.03015	0.36242	0.95986	0.93336	-0.09949	0.060119	0.99796	0.84471	-0.24348	0.31598	0.99768	1.0867	0.11998	0.088934	0.9981	0.81137	-0.30157	0.37444	0.99995	0.89589	-0.15861
390.7739	4.95	domics Pos	0.47876	0.99995	1.0678	0.094612	0.90611	0.99979	0.99655	-0.00499	0.60249	0.99601	0.95135	-0.07195	0.32639	0.99996	1.115	0.15704	0.81382	0.99701	0.98436	-0.02275	0.55482	0.99959	1.0991	0.13633	0.022667	0.18641	1.3636	0.44738	0.2401	0.99984	1.1148	0.15679	0.029907	0.99966	1.1734	0.23073
762.8208	10.05	domics Pos	0.25559	0.99995	1.0977	0.13453	0.14312	0.99979	1.1105	0.15115	0.17042	0.7924	0.95707	-0.0633	0.29988	0.99996	1.0908	0.12538	0.6782	0.99701	1.0125	0.017965	0.76462	0.99959	1.0411	0.058132	0.015924	0.17398	1.3332	0.41494	0.89729	0.99984	1.0222	0.031722	0.32376	0.99966	1.1005	0.13818
901.8122	10.02	domics Pos	0.16362	0.99995	0.91244	-0.1322	0.54389	0.99979	0.93831	-0.09187	0.5256	0.99601	0.9589	-0.06055	0.14485	0.99996	0.88229	-0.18068	0.030316	0.58579	0.82503	-0.27748	0.12717	0.99959	0.85484	-0.22627	0.39551	0.61731	1.0805	0.11176	0.78535	0.99984	0.98049	-0.02842	0.88339	0.99966	1.0154	0.022099
410.9752	10.67	olomics Ne	0.93207	0.9951	0.99029	-0.01407	0.35698	0.99975	1.0606	0.084849	0.30971	0.99731	0.90324	-0.14682	0.42061	0.99915	1.0536	0.075392	0.52049	0.84983	1.0575	0.080711	0.14721	0.57164	1.1111	0.15198	0.040134	0.11162	1.2454	0.31655	0.64676	0.99481	1.0468	0.065979	0.39222	0.92784	0.93024	-0.10433
826.9731	12.02	olomics Ne	0.021873	0.9951	1.3019	0.38059	0.12388	0.99975	1.1968	0.25916	0.54705	0.99731	1.0508	0.071433	0.19001	0.99915	1.1653	0.22072	0.65772	0.89366	0.97847	-0.0314	0.50433	0.79937	1.1127	0.15411	0.18786	0.29309	1.2307	0.29951	0.24338	0.99481	1.1159	0.15821	0.71199	0.97327	1.0673	0.093952
294.194	0.58	domics Pos	0.37768	0.99995	0.89412	-0.16146	0.71373	0.99979	0.95686	-0.06361	0.3579	0.93332	0.89566	-0.15898	0.26568	0.99996	0.84217	-0.24782	0.22665	0.99701	0.85534	-0.22543	0.18673	0.99959	1.2764	0.35205	0.002509	0.10737	1.7589	0.81465	0.24614	0.99984	1.2193	0.28605	0.31904	0.99966	1.2439	0.31486
126.1021	2.05	domics Pos	0.41077	0.99995	0.92809	-0.10766	0.038366	0.99441	0.71906	-0.47582	0.008163	0.19873	0.70624	-0.50177	0.39722	0.99996	0.91842	-0.12277	0.064309	0.76594	0.81357	-0.29765	0.30516	0.99959	0.8389	-0.25343	0.055099	0.23421	1.4165	0.50236	0.24829	0.99984	0.83625	-0.25799	0.59283	0.99966	0.9458	-0.08039
964.7845	10.03	domics Pos	0.30391	0.99995	1.1003	0.13793	0.70223	0.99979	1.0382	0.054066	0.30242	0.89817	0.95316	-0.06921	0.50048	0.99996	1.0524	0.073676	0.91034	0.99701	1.0054	0.007775	0.72544	0.99959	1.0406	0.057378	0.047175	0.22017	1.2294	0.29795	0.69341	0.99984	0.97384	-0.03824	0.40694	0.99966	1.0682	0.095229
1018.771	10.05	domics Pos	0.001823	0.59581	1.2088	0.27361	0.19204	0.99979	1.1072	0.14685	0.34376	0.92896	1.057	0.080028	0.4982	0.99996	1.1033	0.14181	0.098897	0.82449	1.1344	0.18189	0.68705	0.99959	0.97488	-0.0367	0.05318	0.23103	1.4252	0.51116	0.036903	0.99984	1.1394	0.18831	0.045562	0.99966	1.2923	0.36996
834.9734	12.02	olomics Ne	0.048964	0.9951	0.91847	-0.12269	0.074811	0.99975	1.1291	0.1752	0.103	0.99731	0.88299	-0.17952	0.43385	0.99915	1.1391	0.18787	0.89811	0.97408	1.0126	0.018108	0.96322	0.99269	1.0053	0.00764	0.30497	0.42345	1.1165	0.15896	0.002458	0.99481	0.84925	-0.23574	0.42952	0.94458	0.91802	-0.1234
342.9798	12	olomics Ne	0.3438	0.9951	1.08	0.11102	0.44089	0.99975	1.0788	0.10938	0.29645	0.99731	1.0818	0.11345	0.29804	0.99915	1.0953	0.1313	0.22366	0.70622	1.095	0.1309	0.41402	0.77401	1.0997	0.13713	0.04456	0.11766	1.2355	0.30504	0.62418	0.99481	1.0494	0.06956	0.96505	0.99833	0.99008	-0.01438
362.9272	10.03	domics Pos	0.42104	0.99995	1.0497	0.070002	0.94362	0.99979	1.0056	0.008013	0.057749	0.53188	0.94716	-0.07832	0.95741	0.99996	1.0018	0.002602	0.48313	0.99701	0.96641	-0.04929	0.80928	0.99959	0.98992	-0.01462	0.016741	0.17568	1.2517	0.32388	0.95022	0.99984	1.0142	0.02032	0.67743	0.99966	1.0344	0.048801
644.3607	3.11	polomics Pc	0.99644	0.99964	1.0178	0.025432	0.10112	1	0.81249	-0.29958	0.065434	0.99882	0.78265	-0.35355	0.85583	0.99987	1.0798	0.11074	0.046752	0.95986	0.6671	-0.58403	0.32967	0.99796	0.85765	-0.22154	0.75757	0.99768	1.3235	0.40434	0.64007	0.9981	0.93995	-0.08935	0.98502	0.99995	1.0401	0.056762
1155.637	0.57	domics Pos	0.5937	0.99995	0.9083	-0.13876	0.37204	0.99979	0.85975	-0.21802	0.17266	0.7963	0.78675	-0.34603	0.56527	0.99996	0.91767	-0.12395	0.035196	0.61824	0.63611	-0.65265	0.58044	0.99959	0.90075	-0.1508	0.68705	0.81765	1.4124	0.4981	0.72149	0.99984	0.93539	-0.09637	0.7804	0.99966	0.9627	-0.05484
913.589	10.44	olomics Ne	0.40336	0.9951	1.0812	0.1126	0.47718	0.99975	0.92237	-0.11658	0.34125	0.99731	0.89098	-0.16654	0.47952	0.99915	1.0617	0.086336	0.44943	0.80297	1.1335	0.18083	0.32467	0.70479	1.0941	0.12973	0.041682	0.11377	1.3466	0.42931	0.32345	0.99481	1.145	0.1954	0.15649	0.91738	0.8454	-0.24229
154.1238	1.67	domics Pos	0.17553	0.99995	0.84978	-0.23483	0.065604	0.99979	0.7426	-0.42934	0.040578	0.4348	0.73347	-0.44719	0.38057	0.99996	0.86971	-0.20139	0.089686	0.82209	0.80231	-0.31776	0.23647	0.99959	0.82011	-0.2861	0.24684	0.46646	1.2129	0.27841	0.26445	0.99984	0.81571	-0.29387	0.29789	0.99966	0.87754	-0.18846
147.0837	0.32	polomics Pc	0.96742	0.99964	1.0063	0.008996	0.89615	1	0.9997	-0.00044	0.87864	0.99882	1.0331	0.047033	0.18104	0.99987	1.1904	0.25143	0.021184	0.95986	1.2312	0.30009	0.83073	0.99796	1.0276	0.039322	0.003168	0.29567	1.5346	0.61785	0.61213	0.9981	0.94514	-0.08141	0.55979	0.99995	0.9435	-0.08391
368.9794	4.26	olomics Ne	0.44758	0.9951	1.0278	0.03954	0.94716	0.99975	1.0057	0.008245	0.47185	0.99731	0.96176	-0.05626	0.51756	0.99915	1.0367	0.051972	0.068436	0.5958	1.0894	0.12353	0.02669	0.4612	1.125	0.16998	0.01179	0.062031	1.2248	0.29251	0.14693	0.99481	1.0809	0.11218	0.62678	0.96789	0.97732	-0.0331
314.7801	2.06	domics Pos	0.20445	0.99995	0.88157	-0.18185	0.009339	0.99441	0.70093	-0.51266	0.089085	0.65323	0.83211	-0.26516	0.31281	0.99996	0.91629	-0.12612	0.17366	0.95113	0.84784	-0.23814	0.22407	0.99959	0.81873	-0.28854	0.17979	0.39232	1.4241	0.51009	0.23439	0.99984	0.78004	-0.35837	0.39978	0.99966	0.92681	-0.10965
219.2109	1.46	domics Pos	0.052975	0.99995	0.86143	-0.21519	0.015589	0.99441	0.74031	-0.43379	0.016556	0.28182	0.73197	-0.45015	0.2477	0.99996	0.85875	-0.21968	0.042476	0.65306	0.83192	-0.26549	0.15637	0.99959	0.7785	-0.36124	0.11874	0.32077	1.1735	0.23083	0.23962	0.99984	0.81324	-0.29825	0.15355	0.99966	0.86632	-0.20702
114.0925	5.99	polomics Pc	0.0915	0.99964	0.83447	-0.26106	0.01472	0.88953	0.66025	-0.59891	0.024771	0.74312	0.76455	-0.38732	0.62646	0.99987	0.93724	-0.09352	0.081386	0.95986	0.81865	-0.28868	0.066305	0.99796	0.73465	-0.44487	0.79301	0.99768	1.0152	0.02172	0.088218	0.9981	0.75338	-0.40855	0.11989	0.99995	0.8004	-0.32121
189.0975	10.16	domics Pos	0.22506	0.99995	1.089	0.123	0.54509	0.99979	1.028	0.039792	0.5758	0.99601	1.0148	0.021208	0.97008	0.99996	1.003	0.00435	0.97608	0.99701	1.0012	0.001681	0.88233	0.99959	0.99573	-0.00618	0.023613	0.18728	1.2785	0.35442	0.69009	0.99984	1.0374	0.053017	0.41866	0.99966	1.0653	0.091197
198.086	0.6	domics Pos	0.46428	0.99995	0.92094	-0.11882	0.9603	0.99979	1.0161	0.023023	0.13796	0.74403	0.81148	-0.30137	0.57839	0.99996	1.104	0.14277	0.16758	0.94108	1.2842	0.36089	0.29621	0.99959	1.1958	0.25802	0.004142	0.12302	2.009	1.0065	0.66514	0.99984	0.95069	-0.07295	0.69384	0.99966	1.0824	0.11419
267.2597	2.05	domics Pos	0.000271	0.51602	0.822	-0.28279	0.024286	0.99441	0.72582	-0.46231	0.002823	0.1516	0.72196	-0.47001	0.26867	0.99996	0.87028	-0.20045	0.001943	0.46849	0.78906	-0.34179	0.096349	0.99959	0.75817	-0.3994	0.021087	0.18176	1.2306	0.29934	0.21131	0.99984	0.80208	-0.31818	0.10794	0.99966	0.88252	-0.1803
1094.775	10.02	domics Pos	0.85176	0.99995	0.98718	-0.01862	0.57874	0.99979	1.0391	0.055276	0.47227	0.99601	0.95143	-0.07182	0.37868	0.99996	1.0917	0.12661	0.287	0.99701	0.92917	-0.10598	0.90519	0.99959	0.99088	-0.01322	0.030107	0.19804	1.2684	0.34298	0.64018	0.99984	1.1236	0.16813	0.25667	0.99966	1.1123	0.15355
153.128	1.66	domics Pos	0.014739	0.68962	0.82203	-0.28275	0.014389	0.99441	0.69398	-0.52704	0.000247	0.13107	0.69589	-0.52307	0.2651	0.99996	0.83099	-0.2671	0.006994	0.46849	0.7653	-0.3859	0.070375	0.99959	0.73263	-0.44884	0.30778	0.52984	1.1436	0.19361	0.24034	0.99984	0.82607	-0.27567	0.005187	0.99749	0.83729	-0.2562
252.2324	1.23	domics Pos	0.097088	0.99995	0.79929	-0.3232	0.018932	0.99441	0.81593	-0.29349	0.064296	0.55764	0.82516	-0.27725	0.47217	0.99996	0.92517	-0.11221	0.038257	0.63362	0.80234	-0.31771	0.13881	0.99959	0.8607	-0.21642	0.1165	0.31965	1.2293	0.29786	0.3026	0.99984	0.84011	-0.25135	0.33106	0.99966	0.91745	-0.12429

218.9206	10.1	idomics Pos	0.41674	0.99995	1.051	0.071755	0.97233	0.99979	1.0012	0.001688	0.14591	0.76226	0.9456	-0.08069	0.7097	0.99996	0.98378	-0.02359	0.74311	0.99701	0.98442	-0.02266	0.76169	0.99959	0.98532	-0.02133	0.01782	0.17787	1.2688	0.34347	0.66269	0.99984	0.97347	-0.03879	0.71846	0.99966	1.0298	0.042342
631.8661	3.08	oolomics Pos	0.68905	0.99964	1.0727	0.10122	0.10833	1	0.83119	-0.26674	0.038937	0.90186	0.71932	-0.47529	0.69718	0.99987	1.0063	0.009076	0.18539	0.95986	0.8322	-0.265	0.83914	0.99796	0.99474	-0.00761	0.79495	0.99768	1.2465	0.31783	0.97751	0.9981	1.0867	0.11995	0.9064	0.99995	1.0335	0.04753
1063.781	10.02	idomics Pos	0.51301	0.99995	1.1225	0.1667	0.024108	0.99441	1.2463	0.31764	0.11038	0.7009	0.86501	-0.20921	0.81344	0.99986	0.98865	-0.02834	0.042365	0.65306	0.83796	-0.25504	0.84749	0.99959	1.0259	0.036898	0.38404	0.60616	1.1082	0.14825	0.8869	0.99984	1.0139	0.019941	0.42879	0.99966	1.05	0.070378
468.9755	4.96	idomics Pos	0.40017	0.9951	1.0508	0.07145	0.52759	0.99975	1.031	0.044009	0.57815	0.99731	0.97848	-0.03139	0.34155	0.99915	1.0466	0.065707	0.026389	0.50727	1.1157	0.15794	0.071437	0.5223	1.104	0.14279	0.001089	0.041313	1.2746	0.35003	0.20504	0.99481	1.0552	0.077542	0.87268	0.99168	0.9936	-0.00926
151.1487	2.05	idomics Pos	0.10743	0.99995	0.89256	-0.16398	0.080003	0.99979	0.76235	-0.39147	0.026999	0.34662	0.77202	-0.37329	0.36825	0.99996	0.88704	-0.17293	0.082513	0.80455	0.84363	-0.24533	0.15205	0.99959	0.79351	-0.33368	0.08691	0.28013	1.2491	0.32089	0.17591	0.99984	0.79209	-0.33627	0.20888	0.99966	0.89154	-0.16563
227.9556	10.03	idomics Pos	0.34813	0.99995	1.0342	0.048555	0.94796	0.99979	1.0003	0.000493	0.45137	0.99046	0.97948	-0.02992	0.983	0.99996	1.0124	0.017814	0.078516	0.80167	0.93965	-0.08981	0.73905	0.99959	0.98571	-0.02076	0.025281	0.18901	1.2287	0.2971	0.8504	0.99984	0.99313	-0.00995	0.68014	0.99966	1.0381	0.05396
563.5512	2.05	idomics Pos	0.093015	0.99995	0.77323	-0.37103	0.017771	0.99441	0.59782	-0.74223	0.001326	0.13107	0.62789	-0.67141	0.17605	0.99996	0.80696	-0.30944	0.008253	0.46849	0.69	-0.53533	0.049345	0.99959	0.60848	-0.71671	0.79742	0.88304	1.0512	0.072081	0.095169	0.99984	0.67255	-0.57229	0.000962	0.99749	0.72484	-0.46427
252.2328	5.39	oolomics Pos	0.024937	0.92791	0.8207	-0.28507	0.071491	0.99372	0.80667	-0.30994	0.060801	0.99882	0.82384	-0.27956	0.076583	0.99987	0.7349	-0.44439	0.027215	0.95986	0.82034	-0.2857	0.093944	0.99796	0.73138	-0.45131	0.25072	0.99768	0.91483	-0.12843	0.043271	0.9981	0.78226	-0.35427	0.045975	0.99995	0.82673	-0.27451
380.773	9.91	idomics Pos	0.24723	0.99995	0.9059	-0.14257	0.89	0.99979	1.0092	0.013265	0.013413	0.25194	0.82853	-0.27137	0.98052	0.99996	1.0306	0.04354	0.012205	0.48469	0.82654	-0.27485	0.87457	0.99959	0.99805	-0.00282	0.055805	0.23538	0.84198	-0.24815	0.085604	0.99984	0.85079	-0.23312	0.92742	0.99966	1.0255	0.036346
318.9349	10.15	idomics Pos	0.082486	0.99995	1.1699	0.22639	0.69121	0.99979	1.0275	0.039131	0.32554	0.9131	1.085	0.11763	0.68713	0.99996	1.069	0.096226	0.25689	0.99701	1.1097	0.15023	0.14326	0.99959	1.1689	0.22511	0.0061	0.13681	1.6264	0.70168	0.39677	0.99984	1.1206	0.16424	0.1766	0.99966	1.1421	0.19174
564.5544	2.05	idomics Pos	0.051184	0.99995	0.74243	-0.42968	0.011029	0.99441	0.57949	-0.78715	0.003498	0.15208	0.64279	-0.63758	0.20992	0.99996	0.82298	-0.28107	0.014758	0.4899	0.70689	-0.50045	0.057339	0.99959	0.61696	-0.69674	0.6117	0.77593	1.083	0.11498	0.10301	0.99984	0.69032	-0.53466	0.002888	0.99749	0.74019	-0.43404
638.4775	2.05	idomics Pos	0.13534	0.99995	0.75171	-0.41175	0.18798	0.99979	0.78445	-0.35024	0.046379	0.46661	0.67499	-0.56705	0.87908	0.99996	0.99631	-0.00533	0.10658	0.85409	0.71036	-0.49338	0.24737	0.99959	0.77909	-0.36014	0.77096	0.86748	1.046	0.064868	0.10604	0.99984	0.6977	-0.51932	0.51252	0.99966	0.88539	-0.17561
382.405	8.88	idomics Pos	0.10099	0.99964	1.1613	0.21574	0.014199	0.88953	1.2838	0.36037	0.65699	0.99882	1.0877	0.12132	0.044295	0.99987	1.2377	0.30763	0.37353	0.95986	1.1166	0.15913	0.14588	0.99796	1.3467	0.42942	0.17092	0.97894	1.2644	0.33845	0.61516	0.9981	1.1164	0.15881	0.016146	0.99995	1.3335	0.4152
1124.731	10.07	idomics Pos	0.022226	0.8137	1.1164	0.15879	0.96229	0.99979	1.0081	0.011665	0.85706	0.99702	0.99292	-0.01025	0.97814	0.99996	1.0021	0.003071	0.19035	0.97027	0.90853	-0.1384	0.70624	0.99959	0.97871	-0.03105	0.00762	0.14104	1.268	0.3425	0.61516	0.99984	1.0783	0.10871	0.22555	0.99966	1.162	0.21665
241.2348	2.05	idomics Pos	0.10515	0.99995	0.85665	-0.22321	0.14597	0.99979	0.80784	-0.30786	0.03064	0.37688	0.76704	-0.38263	0.42551	0.99996	0.90453	-0.14476	0.040111	0.64262	0.78778	-0.34414	0.19074	0.99959	0.81512	-0.29491	0.19718	0.4113	1.2216	0.28882	0.22884	0.99984	0.7954	-0.33026	0.25285	0.99966	0.89642	-0.15775
396.2532	2.05	idomics Pos	0.22683	0.99995	0.90263	-0.1478	0.21437	0.99979	0.85447	-0.2269	0.019193	0.30022	0.77748	-0.36311	0.61926	0.99996	0.95614	-0.0647	0.006603	0.46849	0.82367	-0.27986	0.19918	0.99959	0.83424	-0.26147	0.052892	0.23049	1.3784	0.46295	0.19017	0.99984	0.82803	-0.27224	0.78084	0.99966	0.98185	-0.02643
410.9768	12.01	olomics Ne	0.2429	0.9951	1.1005	0.13818	0.91705	0.99975	0.99986	-0.0002	0.75857	0.99731	1.0371	0.052576	0.568	0.99915	1.0428	0.060513	0.098082	0.60285	1.1019	0.13995	0.26615	0.66152	1.0946	0.13037	0.004202	0.050041	1.2663	0.34058	0.69052	0.99481	1.0303	0.043036	0.95684	0.99833	0.99923	-0.00111
350.2558	6.39	oolomics Pos	0.3613	0.99964	0.94727	-0.07816	0.45663	1	0.95306	-0.06936	0.018752	0.63482	0.86454	-0.20999	0.38336	0.99987	0.94414	-0.08292	0.62104	0.96686	1.0348	0.049296	0.22622	0.99796	0.8943	-0.16117	0.50295	0.99768	1.0423	0.059829	0.13661	0.9981	0.90317	-0.14693	0.33222	0.99995	0.9336	-0.09912
70.0666	0.59	oolomics Pos	0.69629	0.99995	1.074	0.10306	0.9225	0.99979	0.98881	-0.01624	0.7981	0.99601	1.0656	0.091611	0.34504	0.99996	1.1794	0.23807	0.49635	0.99701	1.357	0.44039	0.12783	0.99959	1.3587	0.44219	0.011388	0.16313	2.586	1.3707	0.98603	0.99984	0.99485	-0.00745	0.97031	0.99966	0.97682	-0.03384
180.1227	1.67	idomics Pos	0.018528	0.74366	0.87648	-0.1902	0.017659	0.99441	0.75256	-0.41013	0.006411	0.17975	0.774	-0.36959	0.22218	0.99996	0.83998	-0.25158	0.003199	0.46849	0.80733	-0.30878	0.11383	0.99959	0.77242	-0.37254	0.10206	0.30004	1.1923	0.25371	0.15254	0.99984	0.8069	-0.30954	0.033655	0.99966	0.86931	-0.20206
592.3222	0.53	idomics Pos	0.71202	0.99995	0.94936	-0.07497	0.061913	0.99979	0.75348	-0.40835	0.021652	0.3198	0.60255	-0.73084	0.93945	0.99996	1.1249	0.16984	0.052716	0.71735	0.65806	-0.6037	0.34652	0.99959	0.82953	-0.26963	0.75012	0.85465	1.438	0.52402	0.44513	0.99984	0.86265	-0.21315	0.83787	0.99966	1.0116	0.016691
431.9813	4.91	idomics Ne	0.73333	0.9951	0.983	-0.02473	0.2007	0.99975	1.0949	0.13077	0.051087	0.99731	0.88994	-0.16821	0.38832	0.99915	1.0489	0.068917	0.30744	0.60605	1.0553	0.077675	0.1102	0.53696	1.1495	0.20099	0.017008	0.06956	1.1657	0.22122	0.69184	0.99481	1.0461	0.065017	0.083822	0.91738	0.91744	-0.12432
666.8799	3.13	idomics Pos	0.76165	0.99964	1.0589	0.082593	0.10102	1	0.80599	-0.31116	0.038057	0.90186	0.75107	-0.41298	0.53634	0.99987	0.9368	-0.09419	0.090301	0.95986	0.74136	-0.43176	0.35356	0.99796	0.88594	-0.17471	0.68722	0.99768	1.2045	0.26839	0.69417	0.9981	0.96138	-0.05683	0.75658	0.99995	1.0099	0.014221
736.481	0.33	idomics Ne	0.82904	0.9951	1.0231	0.032925	0.11013	0.99975	1.1904	0.25144	0.22105	0.99731	1.1808	0.23979	0.17252	0.99915	1.1764	0.23438	0.016178	0.50209	1.3735	0.45786	0.20494	0.63388	1.141	0.19033	0.18683	0.29224	1.2283	0.29667	0.2341	0.99481	1.1927	0.25427	0.27496	0.91738	1.1002	0.13783
261.2222	5.65	idomics Pos	0.022846	0.88846	0.82745	-0.27326	0.037774	0.88953	0.76811	-0.38062	0.074741	0.99882	0.83274	-0.26407	0.61561	0.99987	0.9468	-0.07887	0.089244	0.95986	0.84007	-0.25142	0.083311	0.99796	0.77164	-0.374	0.64909	0.99768	0.96229	-0.05546	0.17396	0.9981	0.79937	-0.32306	0.28692	0.99995	0.89679	-0.15716
359.9315	12.1	oolomics Pos	0.23222	0.99964	1.1345	0.18205	0.55882	1	1.0763	0.10612	0.9739	0.99882	0.99805	-0.00282	0.15612	0.99987	1.0963	0.13268	0.30531	0.95986	1.146	0.19658	0.8875	0.99796	1.0158	0.022684	0.014575	0.45844	1.3464	0.42905	0.45534	0.9981	1.0817	0.11325	0.92937	0.99995	0.99262	-0.01068
385.3314	8.12	idomics Pos	0.60631	0.99964	1.0635	0.088773	0.083697	1	1.1822	0.24142	0.56129	0.99882	1.0852	0.1179	0.04918	0.99987	1.2476	0.31911	0.69567	0.97678	1.0272	0.038705	0.13307	0.99796	1.1355	0.18333	0.1102	0.9111	1.1361	0.18406	0.4006	0.9981	1.0566	0.079435	0.27024	0.99995	1.064	0.089532
442.9745	4.99	idomics Ne	0.49041	0.9951	1.0484	0.068173	0.37396	0.99975	1.0303	0.04309	0.67022	0.99731	0.99811	-0.00272	0.54551	0.99915	1.0316	0.044856	0.02437	0.50209	1.0916	0.12646	0.047858	0.63388	1.1079	0.14785	0.006999	0.057338	1.2694	0.34418	0.14233	0.99481	1.0578	0.072834	0.75156	0.97489	0.99004	-0.01444
356.9812	11.96	idomics Ne	0.47571	0.9951	1.0674	0.094149	0.57391	0.99975	1.0855	0.11841	0.74953	0.99731	1.0236	0.033693	0.2513	0.99915	1.175	0.23272	0.13993	0.64314	1.1549	0.20781	0.1282	0.54233	1.1806	0.23951	0.003551	0.050041	1.3505	0.43344	0.067595	0.99481	1.2242	0.29181	0.97214	0.99833	0.98977	-0.01483
363.2174	3.19	oolomics Pos	0.046659	0.99964	1.4472	0.5333	0.042559	0.88953	1.5255	0.60928	0.061957	0.99882	1.6346	0.70892	0.18909	0.99987	1.3479	0.44459	0.7912	0.99139	0.92132	-0.11822	0.26854	0.99796	0.73482	-0.44454	0.18929	0.99768	0.66901	-0.57991	0.14717	0.9981	1.277	0.35274	0.48802	0.99995	1.1319	0.17873
151.1139	4.37	idomics Pos	0.55938	0.99964	1.0818	0.11344	0.28236	1	1.2501	0.3221	0.2715	0.99882	1.3312	0.4127	0.020116	0.99987	1.4573	0.54334	0.19138	0.99139	1.3711	0.45535	0.013382	0.99796	1.3769	0.46143	0.86858	0.99768	0.99623	-0.00545	0.038359	0.9981	1.4078	0.49343	0.23868	0.99995	1.2072	0.27163
193.655	2.06	idomics Pos	0.45038	0.99995	0.95409	-0.0678	0.081444	0.99979	0.82009	-0.28614	0.018342	0.29983	0.84353	-0.24548	0.21976	0.99996	0.86412	-0.21069	0.21646	0.99598	0.91579	-0.12691	0.12324	0.99959	0.8441	-0.24452	0.099106	0.29579	1.2198	0.2866	0.26401	0.99984	0.86286	-0.21281	0.54407	0.99966	0.95083	-0.07274
486.972	5.18	idomics Ne	0.89415	0.9951	1.0067	0.009609	0.71483	0.99975	1.0164	0.023401	0.41949	0.99731	0.9708	-0.04276	0.54765	0.99915	1.0433	0.061205	0.26744	0.71896	1.0723	0.10072	0.10346	0.53216	1.1058	0.14507	0.013573	0.064762	1.2188	0.28552	0.58484	0.99481	1.0235	0.033575	0.3842	0.92771	0.95535	-0.0659
471.9787	12.01	olomics Ne	0.12975	0.9951	1.3134	0.39329	0.43208	0.99975	1.1029	0.14136	0.93776	0.99731	1.0251	0.035768	0.72569	0.99915	0.96781	-0.0472	0.28169	0.71896	0.89907	-0.1535	0.88265	0.97735	0.99022	-0.01418	0.001941	0.04149	1.3705	0.45474	0.47089	0.99481	1.0679	0.0948	0.62612	0.96789	0.94475	-0.08199
388.1918	4.1	idomics Pos	0.86124	0.99964	0.99474	-0.00761	0.93042	1	1.0168	0.024083	0.20032	0.99882	1.0989	0.13602	0.18351	0.99987	1.1792	0.23781	0.15597	0.95986	1.263	0.33685	0.018387	0.99796	1.2069	0.27135	0.031801	0.64627	1.155	0.15772	0.094844	0.9981	1.2367	0.30645	0.4881	0.99995	1.0331	0.047022
285.2504	4.73	idomics Pos	0.039232	0.96211	0.76343	-0.38944	0.27267	0.99979	0.89098	-0.16653	0.045898	0.46561	0.78711	-0.34537	0.29447	0.99996	0.90314	-0.14698	0.297	0.99701	0.89247	-0.16413	0.50071	0.99959	0.92411	-0.11387	0.16684	0.37644	1.2887	0.36595	0.36527	0.99984	0.89679	-0.15715	0.86908	0.99966	0.98128	-0.02727
1127.753	0.51	idomics Pos	0.74152	0.99995	1.0313	0.0445	0.32544	0.99979	1.07	0.09763	0.65464	0.99601	0.97267	-0.03997	0.42765	0.99996	1.069	0.096286	0.45467	0.99701	1.0683	0.095351	0.15752	0.99959	1.1803	0.2391	0.012525	0.16313	1.4036	0.48917	0.57243	0.99984	1.1107	0.15146	0.39366	0.99966	1.0972	0.13387
278.052	5.24	idomics Pos	0.075773	0.99964	1.1832	0.24272	0.4946	1	1.076	0.10561	0.44834	0.99882	1.0815	0.11302	0.659	0.99987	1.0471	0.066421	0.011351	0.95986	1.2072	0.27171	0.614	0.99796	0.96062	-0.05796	0.21052	0.99768	1.1157	0.15793	0.4532	0.9981	1.0405	0.057327	0.8192	0.99995	1.0343	0.04871
480.843	10.11	idomics Pos	0.68624	0.99995	1.0656	0.091633	0.81553	0.99979	1.067	0.093615	0.95552	0.99702	0.98707	-0.01878	0.49107	0.99996	1.1011	0.13896	0.31528	0.99701	1.1511	0.20307	0.24696	0.99959	1.1719	0.22884	0.037585	0.20439	1.492	0.57726	0.15571	0.99984	1.2393	0.30952	0.12463	0.99966	1.2635	0.3374
906.8258	10.02	idomics Pos	0.33575	0.99995	1.0617	0.086339	0.45511	0.99979	1.0383	0.054236	0.20019	0.82788	0.9549	-0.06658	0.56547	0.99996	1.0412	0.058194	0.96049	0.99701	1.0001	0.000149	0.96773	0.99959	1.0014	0.001975	0.011258	0.16313	1.2951	0.37301	0.78934	0.99984	1.0277	0.039457	0.30459	0.99966	1.0804	0.11152
233.9219	12.08	oolomics Pos	0.74913	0.99964	1.0312	0.04432	0.87175	1	0.99422	-0.00836	0.99758	0.99986	0.99846	-0.00223	0.019829	0.99987	0.86146	-0.21514	0.80918	0.99139	1.0099	0.014152	0.62593	0.99796	0.95854	-0.06112	0.13717	0.66991	1.1565	0.20972	0.69619	0.9981	1.0315	0.044798	0.47814	0.99995	1.0582	0.08163
634.8766	10.02	idomics Pos	0.38012	0.99995	1.0485	0.06835	0.55188	0.99979	1.0312	0.044274	0.015337	0.27456	0.95439	-0.06735	0.88853	0.99996	1.0128	0.018299	0.48138	0.99701	0.96909	-0.0453	0.98796	0.99959	1.0049	0.007035	0.019351	0.17969	1.2775	0.35336	0.67595	0.99984	1.0331	0.047046	0.39968	0.99966	1.0662	0.092499
565.5576	2.05	idomics Pos	0.074101	0.99995	0.72634	-0.46129	0.021909	0.99441	0.57387	-0.80121	0.000644	0.13107	0.57186	-0.80628	0.12305	0.99996	0.77378	-0.37	0.007299	0.46849	0.61248	-0.70726	0.065043	0.99959	0.60547	-0.72386	0.54677	0.73912	1.0922	0.12719	0.13158	0.99984	0.68785	-0.53984	0.002356	0.99749	0.73187	-0.45035
363.9212	0.28	idomics Pos	0.1164	0.99964	0.84092	-0.24996	0.38873	1	0.92641	-0.11028	0.027762	0.78784	0.82736	-0.27341	0.094208	0.99987	0.8738	-0.19462	0.22477	0.95986	0.89199	-0.1649	0.32143	0.99796	0.90597	-0.14247	0.65497	0.99768	1.0389	0.055049	0.003201	0.9981	0.76696	-0.38277	0.36451	0.99995	0.91884	-0.12211
443.865	10.14	idomics Pos	0.71431	0.99995	1.0167	0.023866	0.99752	0.99988	1.0087	0.012544	0.58157	0.99601	0.95644	-0.06425	0.24891	0.99996	1.1671	0.22292	0.71342	0.99701	1.0282	0.040143	0.57636	0.99959	1.0631	0.08824	0.02561	0.18992	1.4067	0.49236	0.67665	0.99984	1.0493	0.069372	0.67701	0.99966	0.9736	-0.0386
692.8352	10.05	idomics Pos	0.61517	0.99995	1.0524	0.073662	0.99513	0.99979	1.0022	0.003236	0.26044	0.87845	0.95179	-0.07129	0.92247	0.99996	1.0132	0.018857	0.80872	0.99701	1.0235	0.033457	0.80093	0.99959	1.0251	0.035754	0.017665	0.17679	1.3211	0.40173	0.75715	0.99984	1.0329	0.046731	0.17014	0.99966	1.1006	0.13825
446.8746	12.09	idomics Pos	0.99988	0.99988	0.99588	-0.00596	0.25935	1	1.0918	0.12675	0.41206	0.99882	1.0664	0.09277	0.52392	0.99987	1.0656	0.091637	0.1302	0.95986	1.1155	0.15764	0.72947	0.99796	0.97361	-0.03858	0.035457	0.66788	1.2336	0.30289	0.33048	0.9981	1.116	0.15831	0.48763	0.99995	1.0372	0.052671
825.6734	6.23	idomics Pos	0.27119	0.99995	1.3797	0.46435	0.62752	0.99979	1.1363	0.1843	0.36356	0.93332	1.3128	0.39266	0.029435	0.99996	1.5341	0.61735	0.40023	0.99701	1.2431	0.31396	0.2624	0.99959	0.85332	-0.22883	0.03269	0.20029	1.8765	0.90801	0.36576	0.99984	1.2321	0.30108	0.033638	0.99966	1.5588	0.64044
1042.8	10.02	idomics Pos	0.41284	0.99995	1.0623	0.087241	0.99972	0.99999	1.0025	0.003567	0.16698	0.7924	0.94889	-0.07569	0.92124	0.99996	0.99863	-0.00197	0.42914	0.99701	0.96542	-0.05077	0.7118	0.99959	0.98322	-0.02441	0.043185	0.21497	1.2299	0.29852	0.95547	0.99984	1.0121	0.01739	0.40219	0.99966	1.0625	0.087518
703.8679	10.02	idomics Pos	0.38338	0.99995	1.0686	0.09573	0.49336	0.99979	1.0302	0.042957	0.56621	0.99601	0.97389	-0.03817	0.90998	0.99996	1.0007	0.000987	0.64967	0.99701	1.0283	0.04023	0.68342	0.99959	1.0288	0.040984	0.032153	0.20029	1.288	0.36515	0.86326	0.99984	0.99159	-0.01218	0.60907	0.99966	1.0422	0.059669
1111.49	6.01	idomics Pos	0.91299	0.99995	0.99487	-0.00742	0.71175	0.99979	1.0289	0.041041	0.22434	0.83453	0.86878	-0.20293	0.7759	0.99996	0.9795	-0.02988	0.56874	0.99701	1.0586	0.082223	0.75086	0.99959	1.0349	0.049492	0.037636	0.20439	1.2873	0.36432	0.14136	0.99984	1.1272	0.1728	0.21797	0.99966	1.2451	0.31629
641.5366	4.84	idomics Pos	0.25503	0.99995	0.86309	-0.21242	0.31064	0.99979	0.90983	-0.13633	0.83608	0.99702	1.0359	0.050861	0.060614	0.99996	0.8593	-0.21876	0.77865	0.99701	0.97922	-0.0303	0.86659	0.99959	1.0076	0.010957	0.049929	0.22586	1.5209	0.60495	0.35811	0.99984	1.1544	0.20708	0.32078	0.99966	1.1169	0.15945
119.1272	11.8	oolomics Pos	0.93538	0.99964	0.98875	-0.01632	0.70219	1	1.0287	0.040818	0.94436	0.99882	0.99062	-0.0136	0.47841	0.99987	0.93835	-0.0918	0.13636	0.95986	1.1546	0.20735	0.58429	0.99796	0.95354	-0.06864	0.023559	0.57962	1.3702	0.45438	0.57496	0.9981	1.1139	0.15559	0.63161	0.99995	1.0663	0.092646
1030.874	11.98	olomics Ne	0.51257	0.9951	1.047	0.066294	0.6026	0.99975	1.0295	0.041901	0.12167	0.99731	1.0745	0.10363	0.27066	0.99915	1.052	0.073068	0.10177	0.60605	1.121	0.16477	0.065892	0.5223	1.1546	0.20735	0.002834	0.050041	1.3207	0.40133	0.28375	0.99481	1.1084	0.14851	0.94116	0.99833	0.99699	-0.00435
433.9028	6.37	oolomics Pos	0.014547	0.68953	0.78172	-0.35528	0.004373	0.88953	0.65396	-0.61274	0.42182	0.99882	0.88307	-0.1794	0.23453	0.99987	0.85975	-0.21801	0.76026	0.99139	0.98415	-0.02305	0.2348	0.99796	0.83816	-0.25471	0.28054	0.99768	1.0786	0.10914	0.049165	0.9981	0.68135	-0.55352	0.58521	0.99995	0.95116	-0.07224
135.1179	6	oolomics Pos	0.018349	0.86976	0.83944	-0.2525	0.045087	0.88953	0.75375	-0.40784	0.012694	0.60269	0.80773	-0.30805	0.52282	0.99987	0.94595	-0.08017	0.036958	0.95986	0.86439	-0.21024	0.12899	0.99796	0.79795	-0.32563	0.85824	0.99768	0.9904	-0.01392	0.094896	0.9981	0.79548	-0.33009	0.033237	0.99995	0.81055	-0.30302
137.1329	1.66	idomics Pos	0.21319	0.99995	0.93134	-0.10263	0.046338	0.99441	0.75699	-0.40165	0.017975	0.29799	0.79472	-0.33147	0.5763	0.99996	0.93901	-0.09078	0.013093	0.46849	0.80837	-0.31766	0.12765	0.99959	0.78711	-0.34537	0.13595	0.34165	1.238	0.30802	0.2188	0.99984	0.83677	-0.25693	0.051024	0.99966	0.90833	-0.13871
663.8913	3.13	idomics Pos	0.90929	0.99964	1.0513	0.072148	0.35044	1	0.90101	-0.15038	0.10824	0.99882	0.7396	-0.43519	0.8091	0.99987	1.0967	0.13312	0.023804	0.95986	0.66843	-0.58114	0.60167	0.99796	0.93179	-0.10193	0.93396	0.99768	1.2079	0.27253	0.56716	0.9981	0.92936	-0.1057	0.83465	0.99995	1.0108	0.015519
438.9802	12.01	olomics Ne	0.55979	0.9951	0.92908	-0.10612	0.88895	0.99975	1.007	0.010006	0.96531	0.99731	1.0132	0.018935	0.20136	0.99915	0.8737	-0.19479	0.81499	0.95275	1.0232	0.033127	0.50637	0.80014	1.0982	0.13518	0.032846	0.099344	1.3895	0.47454	0.68636	0.99481	1.0648	0.090537	0.86472	0.99168	0.97581	-0.03534
178.1676	6.38	idomics Pos	0.43554	0.99964	0.95367	-0.06844	0.11631	1	0.844	-0.24468	0.28186	0.99882	0.91666	-0.12554	0.51655	0.99987	0.94681	-0.07886	0.003563	0.95986	0.91355	-0.13045	0.3827	0.99796	0.89081	-0.16681	0.007646	0.36601	1.0951	0.13107	0.17754	0.9981	0.86917	-0.20229	0.29175	0.99995	0.89316	-0.163
901.5699	5.31	idomics Pos	0.24741	0.99995	1.2375	0.30739	0.28725	0.99979	1.2243	0.29192	0.6177	0.99601	1.098	0.13483	0.47735	0.99996	1.1314	0.17816	0.25824	0.99701	1.2435	0.3144	0.021633	0.99959	1.3399	0.42208	0.001136	0.089845	1.9228	0.94324	0.15541	0.99984	1.4004	0.48587	0.015858	0.99966	1.5718	0.65243
286.9773	3.65	idomics Ne	0.9716	0.9951	0.99724	-0.00399	0.98463	0.99975	1.0042	0.006101	0.27798	0.99731	0.94518	-0.08134	0.50919	0.99915	1.0046	0.06293	0.65646	0.89366	1.025	0.03565	0.29025	0.67814	1.0642	0.089806	0.024798	0.085507	1.164	0.21914	0.71507	0.99481	0.98026	-0.02876	0.14474	0.91738	0.93257	-0.10072
321.7678	6.38	oolomics Pos	0.081984	0.99964	0.81366	-0.2975	0.02676	0.88953	0.66117	-0.5969	0.040898	0.92683	0.76838	-0.38011	0.081446	0.99987	0.8045	-0.31383	0.21307	0.95986	0.85302	-0.22936	0.17698	0.99796	0.76486	-0.38674	0.99411	0.99932	0.99584	-0.00601	0.16505	0.9981	0.80641	-0.31041	0.015429	0.99995	0.71335	-0.48733
684.5801	4.84	idomics Pos	0.65141	0.99995	1.0492	0.069317	0.67113	0.99979	0.938	-0.09234	0.56201	0.99601	0.9094	-0.13701	0.63857	0.99996	0.93236	-0.10104	0.87985	0.99701	1.0083	0.011933	0.25984	0.99959	1.1849	0.24473	0.010814	0.1625	1.4704	0.55619	0.85452	0.99984	1.0145	0.020711	0.32845	0.99966	1.154	0.20666
354.98	4.46	idomics Ne	0.27782	0.9951	1.0691	0.09642	0.46736	0.99975	1.0462	0.065223	0.78449	0.99731	0.98714	-0.01867	0.36139	0.99915	1.0502	0.070618	0.034305	0.52749	1.1095	0.14992	0.046295	0.49377	1.1376	0.18604	0.011195	0.061656	1.2295	0.29802	0.07705	0.99481	1.0862	0.11923	0.71036	0.97327	0.98527	-0.0214
272.9451	10.14	idomics Pos	0.25213	0.99995	1.0623	0.087182	0.30191	0.99979	1.0645	0.090214	0.32822	0.91551	0.97741	-0.03296	0.5246	0.99996	1.0594	0.083214	0.47446	0.99701	0.98141	-0.02708	0.45063	0.99959	1.0599	0.083931	0.005326	0.1319	1.3135	0.39338	0.90885	0.99984	1.002	0.002837	0.40207	0.99966	1.0791	0.10984
346.9538	11.89	olomics Ne	0.45831	0.9951	0.94165	-0.08674	0.008435	0.88953	1.1246	0.16937	0.38747	0.99882	0.92569	-0.1114	0.084444	0.99987	0.99286	-0.01236	0.97078	0.99906	0.96256	-0.05506	0.073643	0.83627	1.2806	0.27339	0.84849	0.9981	1.0133	0.019011	0.36599	0.99995	0.93873	-0.09121
200.953	10.03	idomics Pos	0.36062	0.99995	1.0658	0.086038	0.21667	0.99979	1.0705	0.098343	0.013142	0.2493	0.91956	-0.12098	0.95498	0.99996	1.0069	0.009984	0.59282	0.9977	1.014	0.00203	0.38589	0.99959	1.0744	0.10359	0.004528	0.12695	1.2718	0.34691	0.62425	0.99984	1.0409	0.057881	0.41591	0.99966	1.0657	0.091862
566.5606	2.05	idomics Pos	0.53662	0.99995	0.88838	-0.17076	0.041094	0.99441	0.63899	-0.64614	0.06166	0.5532	0.69467	-0.5256	0.55977	0.99996	0.89459	-0.16071	0.12651	0.86468	0.75246	-0.41032	0.22836	0.99959	0.70691	-0.50039	0.21551	0.43393	1.2592	0.33246	0.17561	0.99984	0.70499	-0.50432	0.16496	0.99966	0.76939	-0.37822
381.9792	11.98	olomics Ne	0.056336	0.9951	1.1448	0.19505	0.34165	0.99975	1.1185	0.16151	0.029953	0.99731	1.1602	0.21438	0.098522	0.99915	1.1431	0.19298	0.052154	0.58152	1.2888	0.36603	0.007169	0.45618	1.3352	0.417	0.014627	0.066581	1.3073	0.3866	0.2659	0.99481	1.0983	0.13529	0.54767	0.96586	1.0315	0.044689
433.9008	2.05	idomics Pos	0.015525	0.68962	0.74749	-0.41987	0.0164	0.99441	0.60817	-0.71745	0.026309	0.34591	0.70956	-0.495	0.075977	0.99996	0.76908	-0.37879	0.024983	0.5516	0.72545	-0.46306	0.10709	0.99959	0.72323	-0.46747	0.36445	0.58727	1.1288	0.17479	0.060454	0.99984	0.74302	-0.42853	0.022413	0.99749	0.76324	-0.38979
342.8707	0.56	idomics Pos	0.61075	0.99995	0.98442	-0.02266	0.17521	0.99979	0.95343	-0.0688	0.18502	0.81072	0.95498	-0.06646	0.55282	0.99996	0.96542	-0.05077	0.015829	0.49216	0.91286	-0.13154	0.13195	0.99959	0.86806	-0.20413	0.57813	0.75676	0.95903	-0.06036	0.5369	0.99984	1.0335	0.04757	0.73762	0.99966	1.0402	0.056881
458.2384	5.98	idomics Ne	0.50772	0.9951	0.923	-0.1156	0.009825	0.99975	0.77148	-0.37429	0.026007	0.99731	0.62686	-0.67378	0.11531	0.99915	0.86997	-0.20096	0.24335	0.71555	0.83717	-0.25641	0.003463	0.45618	0.75151	-0.41213	0.066524	0.14991	1.2308	0.29962	0.2983	0.99481	0.82485	-0.2778	0.45486	0.94605	0.92115	-0.11849
356.0608	0.57	idomics Pos	0.87659	0.99995	1.0015	0.002195	0.80999	0.99979	0.97964	-0.02967	0.26554	0.88313	0.84822	-0.23748	0.67399	0.99996	1.0113	0.013916	0.11782	0.86265	1.1228	0.29016	0.74237	0.99959	1.0376	0.053281	0.001058	0.089845	1.9277	0.94686	0.15367	0.99984	0.83533	-0.25958	0.082398	0.99966	0.86172	-0.21472
196.1002	4.38	idomics Pos	0.12457	0.99964	1.1648	0.22006	0.035051	0.88953	1.1679	0.22392	0.40677	0.99882	1.087	0.12037	0.007583	0.99987	1.3098	0.38932	0.11431	0.95986	1.1342	0.18173	0.076317	0.99796	1.2282	0.29654	0.36871	0.99768	1.0854	0.11816	0.37988	0.9981	1.1072	0.14697	0.095322	0.99995	1.1731	0.23028
724.5159	0.32	idomics Pos	0.87645	0.99964	0.99338	-0.00959	0.58953	1	1.0297	0.042193	0.41389	0.99882	0.93377	-0.09886	0.4531	0.99987	0.9467	-0.07902	0.40329	0.95986	0.92204	-0.11709	0.053124	0.99796	0.89395	-0.16173	0.025996	0.59691	0.79296	-0.33469	0.38388	0.9981	0.94151	-0.08695	0.75215	0.99995	0.98088	-0.02785
604.3101	1.84	idomics Pos	0.32588	0.99995	1.0808	0.11215	0.22616	0.99979	1.0936	0.1291	0.21758	0.83453	0.90577	-0.14278	0.97812	0.99996	1.0131	0.018624	0.95373	0.99701	0.99489	-0.00739	0.68252	0.99959	1.0515	0.072485	0.04629	0.22017	1.2119	0.27727	0.88248	0.99984	1.0116	0.016604	0.21434	0.99966	1.2089	0.27374
354.8956	10.07	idomics Pos	0.76035	0.99995	0.98237	-0.02566	0.71538	0.99979	1.0442	0.062391	0.10718	0.68915	0.94517	-0.08136	0.67882	0.99996	0.97691	-0.0337	0.18531	0.95986	0.92628	-0.11047	0.92092	0.99959	1.0108	0.015559	0.017518	0.17671	1.2616	0.33524	0.52743	0.99984	0.96183	-0.05615	0.1087	0.99966	1.095	0.13098
796.9749	12.02	olomics Ne	0.041847	0.9951	1.1095	0.14992	0.20668	0.99975	1.1627	0.21752	0.23314	0.99731	1.1482	0.19942	0.1789	0.99915	1.3374	0.41945	0.00199	0.24494	1.3975	0.4828	0.060283	0.51317	1.3494	0.43227	0.000169	0.041313	1.5269	0.61058	0.025358	0.99481	1.3452	0.42781	0.48109	0.9463	1.1891	0.24993
807.9708	12.04	olomics Ne	0.31112	0.9951	1.1933	0.25494	0.39367	0.99975	1.167	0.22278	0.29258	0.99731	1.171	0.22778	0.38253	0.99915	1.218	0.28449	0.14504	0.64314	1.2786	0.35458	0.61035	0.84705	1.1645	0.090161	0.021678	0.070884	1.4476	0.53363	0.10377	0.99481	1.4344	0.52047	0.93603	0.99833	1.0179	0.025569
794.5931	5.19	idomics Pos	0.34421	0.99995	1.0618	0.064669	0.49058	0.99979	1.0354	0.050184	0.2056	0.83096	0.95338	-0.06888	0.92613	0.99996	0.99726	-0.00396	0.59224	0.99701	0.97204	-0.04092	0.59336	0.99959	1.0289	0.041104	0.032534	0.20029	1.2303	0.29896	0.47969	0.99984	1.055	0.077259	0.41599	0.99966	1.1134	0.15499
83.0868	6.99	idomics Pos	0.33843	0.99964	1.2199	0.28674	0.6886	1	0.96883	-0.04569	0.65869	0.99882	1.0463	0.065352	0.84277	0.99987	1.0892	0.12325	0.11223	0.95986	1.2395	0.30981	0.31169	0.99796	0.84306	-0.2463	0.016161	0.4752	1.1491	0.20052	0.41047	0.9981	1.1997	0.26272	0.72814	0.99995	0.97521	-0.03621
600.3354	3.35	idomics Pos	0.29854	0.99964	1.1961	0.25833	0.033152	0.88953	0.793	-0.33461	0.20819	0.99882	0.85447	-0.2269	0.94118	0.99987	1.1388	0.18756	0.41667	0.95986	0.87041	-0.20023	0.94029	0.99796	1.0318	0.045146	0.64491	0.99768	1.341	0.42335	0.70607	0.9981	1.0782	0.10862	0.67729	0.99995	0.97856	-0.03127
182.1579	2.05	idomics Pos	0.12613	0.99995	0.87895	-0.18615	0.19873	0.99979	0.8293	-0.27004	0.009335	0.21077	0.73455	-0.44507	0.45607	0.99996	0.9281	-0.10765	0.020375	0.53763	0.84085	-0.25009	0.055886	0.99959	0.81695	-0.29168	0.11767	0.32047	1.1317	0.17848	0.31605	0.99984	0.84637	-0.24063	0.34066	0.99966	0.94613	-0.07989
81.0707	1.66	idomics Pos	0.085426	0.99995	0.88705	-0.17291	0.019341	0.99441	0.76859	-0.37972	0.012164	0.23259	0.73959	-0.4352	0.23858	0.99996	0.85414	-0.22746	0.01911	0.52048	0.79426	-0.33232	0.1375	0.99959	0.78451	-0.35014	0.11885	0.32077	1.2378	0.30776	0.25351	0.99984	0.82997	-0.26886	0.043543	0.99966	0.86252	-0.21338
446.8883	10.1	idomics Pos	0.7313	0.99995	1.0342	0.04851	0.85238	0.99979	0.98913	-0.01577	0.84304	0.99702	0.98855	-0.01662	0.35531	0.99996	1.0811	0.11255	0.72819	0.99701	1.0562	0.078941	0.99665	0.99959	1.001	0.001444	0.014502	0.1696	1.4554	0.54139	0.99257	0.99984	1.0055	0.00795	0.13442	0.99966	1.1595	0.21346
438.8541	10.13	idomics Pos	0.34185	0.99995	1.0955	0.13163	0.67796	0.99979	0.97358	-0.03863	0.38121	0.9382	0.92902	-0.10622	0.28463	0.99996	1.078	0.10838	0.35873	0.99701	0.91519	-0.12786	0.33559	0.99959	1.0759	0.10551	0.038274	0.20439	1.3187	0.3991	0.77605	0.99984	0.97893	-0.03073	0.93348	0.99966	1.0232	0.03303
590.6806	0.55	idomics Neg	0.93875	0.99486	1.0083	0.011931	0.16166	0.99506	1.1674	0.22333	0.37154	0.70401	1.1249	0.1698	0.010197	0.80547	1.409	0.49468	0.45916	0.82276	1.1133	0.15481	0.18759	0.89898	1.1777	0.23595	0.8546	0.99905	1.02	0.02857	0.055682	0.96955	1.2466	0.31796	0.1054	0.74722	1.2707	0.34559
420.8858	10.06	idomics Pos	0.80955	0.99995	1.0178	0.025513	0.88384	0.99979	0.99604	-0.00573	0.008081	0.19873	0.94719	-0.07827	0.80656	0.99996	0.99002	-0.01446	0.97078	0.99906	0.96256	-0.06553	0.65456	0.99959	0.98052	-0.02839	0.020132	0.18119	1.2297	0.29836	0.82667	0.99984	0.99138	-0.01248	0.72566	0.99966	1.028	0.040968
342.9798	4.35	idomics Ne	0.46723	0.9951	1.0343	0.048702	0.57515	0.99975	1.0329	0.046646	0.44301	0.99731	0.97324	-0.03913	0.29422	0.99915	1.0462	0.065094	0.039235	0.53488	1.096	0.13223	0.037309	0.4612	1.1186	0.16172	0.000427	0.050628	1.2263	0.29432	0.29617	0.99481	1.0596	0.083585	0.60244	0.96789	0.97636	-0.03451
537.9397	0.54	idomics Pos	0.97115	0.99995	1.0746	0.10378	0.27051	0.99979	0.85778	-0.22132	0.17164	0.79523	0.81254	-0.29949	0.95065	0.99996	1.1987	0.26147	0.039866	0.62467	0.70417	-0.506	0.2871	0.99959	0.86916	-0.20231	0.54542	0.73873	1.5754	0.65571	0.93976	0.99984	1.0021	0.003091	0.8248	0.99966	1.2704	0.34527
355.9837	4.46	idomics Ne	0.82072	0.9951	1.017	0.024389	0.26146	0.99975	1.0781	0.10848	0.33264	0.99731	1.0543	0.076335	0.27264	0.99915	1.1171	0.15977	0.093146	0.60285	1.0922	0.12724	0.087329	0.52866	1.1403	0.18947	0.049092	0.12538	1.2532	0.32558	0.78517	0.99481	0.98258	-0.02535	0.99442	0.99833	0.99793	-0.00299
830.5582	4.77	idomics Pos	0.55213	0.99995	1.0267	0.037956	0.6053	0.99979	1.0292	0.041502	0.5648	0.99601	0.97128	-0.04205	0.79303	0.99996	0.98537	-0.02126	0.25539	0.99701	0.94733	-0.07806	0.9185	0.99959	1.0164	0.023426	0.041907	0.21081	1.2396	0.30991	0.84078	0.99984	0.99019	-0.01423	0.96276	0.99966	1.0053	0.007561
363.931	0.5	idomics Pos	0.61996	0.99995	1.0501	0.070518	0.54097	0.99979	0.95929	-0.05996	0.16313	0.78771	0.92218	-0.11689	0.3878	0.99996	0.95064	-0.07528	0.70782	0.9977	0.97661	-0.03415	0.95462	0.99959	0.99784	-0.00311	0.029773	0.19804	1.1877	0.24812	0.96267	0.99984	1.0065	0.009352	0.50618	0.99966	1.054	0.075899
69.0711	6.99	idomics Pos	0.79553	0.99964	1.0704	0.098194	0.87793	1	1.0043	0.006197	0.93986	0.99882	1.0108	0.01545	0.93519	0.99987	1.0633	0.088491	0.93574	0.99906	0.99831	-0.00244	0.04571	0.99796	0.75798	-0.39976	0.7987	0.99768	1.0662	0.092505	0.89034	0.9981	1.0432	0.061012	0.77204	0.99995	0.98401	-0.02325
100.9583	10.18	idomics Pos	0.59879	0.99995	0.94019	-0.08897	0.27703	0.99979	0.88526	-0.17583	0.035622	0.41036	0.76704	-0.38262	0.94006	0.99996	1.0014	0.002016	0.24138	0.99701	0.87443	-0.19359	0.59895	0.99959	0.99784	-0.00311	0.34572	0.56968	1.1377	0.18618	0.35421	0.99984	1.0065	0.009352	0.44147	0.99966	0.9121	-0.13274
168.051	10.16	idomics Pos	0.73879	0.99995	0.98935	-0.01544	0.17203	0.99979	0.92145	-0.11802	0.054281	0.51486	0.88502	-0.17622	0.75537	0.99996	0.98226	-0.02582	0.23565	0.99701	0.91352	-0.1305	0.002386	0.99959	0.8869	-0.17316	0.25682	0.47444	1.1211	0.16495	0.10376	0.99984	0.90705	-0.14074	0.66544	0.99966	0.97075	-0.04283
441.2542	2.06	idomics Pos	0.68713	0.99995	0.98091	-0.02781	0.20776	0.99979	0.85538	-0.22536	0.024365	0.33661	0.86088	-0.21611	0.6306	0.99996	0.96601	-0.04989	0.44164	0.99701	0.95994	-0.05898	0.58792	0.99959	0.94838	-0.07646	0.020496	0.18119	1.3998	0.48519	0.3409	0.99984	0.88183	-0.18143	0.54632	0.99966	1.018	0.025705
176.0679	0.34	idomics Pos	0.98941	0.99964	1.0317	0.045013	0.8305	1	1.083	0.11498	0.57156	0.99882	1.1492	0.20061	0.12608	0.99987	1.4401	0.52612	0.019773	0.95986	1.5632	0.64452	0.36773	0.99796	1.1161	0.15849	0.007783	0.36601	1.6595	0.73077	0.58271	0.9981	0.92042	-0.11964	0.13984	0.99995	0.7956	-0.32988
287.3019	2.66	idomics Pos	0.11005	0.99995	0.72574	-0.46248	0.14026	0.99979	0.70097	-0.51257	0.092813	0.66529	0.71765	-0.47864	0.56684	0.99996	0.91053	-0.13523	0.12186	0.86273	0.71764	-0.47866	0.14434	0.99959	0.76127	-0.39352	0.73617	0.84677	1.0272	0.038714	0.044627	0.99984	0.6625	-0.594	0.02841	0.99966	0.73605	-0.44213
407.7609	5.56	idomics Pos	0.20206	0.99995	1.1717	0.22857	0.25592	0.99979	1.1946	0.25657	0.44001	0.98288	1.096	0.1322	0.35717	0.99996	1.2266	0.29468	0.69865	0.99701	1.0389	0.054988	0.14496	0.99959	1.1787	0.23716	0.048351	0.2237	1.3831	0.46787	0.25575	0.99984	1.2764	0.35204	0.022939	0.99966	1.3489	0.43183
782.6192	5.67	idomics Pos	0.53001	0.99995	1.191	0.25221	0.4937	0.99979	1.174	0.23149	0.94427	0.99702	1.0025	0.003601	0.48624	0.99996	1.1665	0.22217	0.94385	0.99701	1.0882	0.082954	0.78297	0.99959	0.93084	-0.1034	0.03222	0.20029	1.5898	0.66887	0.58123	0.99984	1.0872	0.12066	0.06539	0.99966	1.4326	0.51861

436.9738	4.88	olomics Ne	0.61577	0.9951	1.0161	0.023092	0.37265	0.99975	1.0415	0.058676	0.65808	0.99731	0.97831	-0.03164	0.36311	0.99915	1.0487	0.068618	0.22379	0.70622	1.0732	0.10197	0.19947	0.63388	1.0799	0.11095	0.009988	0.060961	1.2416	0.31215	0.50795	0.99481	1.0332	0.04714	0.6234	0.96789	0.97926	-0.03024
582.5261	6.37	olomics Po	0.04031	0.99964	0.71758	-0.47879	0.095931	1	0.62656	-0.67447	0.066871	0.99882	0.7343	-0.44556	0.56658	0.99987	0.914	-0.12973	0.20537	0.95986	0.80458	-0.3137	0.20909	0.99796	0.73289	-0.44833	0.65571	0.99768	1.0526	0.073987	0.10669	0.9981	0.64811	-0.6257	0.18905	0.99995	0.80164	-0.31897
1014.717	0.54	lomics Pos	0.53397	0.99995	0.9652	-0.0511	0.009831	0.99441	0.89988	-0.1522	0.004516	0.16399	0.84018	-0.25123	0.071504	0.99996	0.88175	-0.18157	0.00343	0.46849	0.73961	-0.43517	0.058478	0.99959	0.85813	-0.22073	0.18626	0.39899	0.89085	-0.16675	0.12565	0.99984	0.83237	-0.26471	0.96163	0.99966	1.0059	0.008544
464.9742	5.09	olomics Ne	0.28966	0.9951	1.0411	0.058127	0.22735	0.99975	1.0696	0.097008	0.30234	0.99731	0.96088	-0.05758	0.2519	0.99915	1.0419	0.059233	0.21909	0.70622	1.07	0.097555	0.034433	0.4612	1.1561	0.20927	0.002915	0.050041	1.2482	0.31987	0.32297	0.99481	1.042	0.05934	0.71525	0.97327	0.98025	-0.02878
318.2411	5.03	olomics Po	0.21264	0.99964	0.85473	-0.22646	0.11982	1	0.83012	-0.2686	0.16253	0.99882	0.81514	-0.29488	0.48744	0.99987	0.92337	-0.11501	0.14458	0.95986	0.83326	-0.26316	0.029424	0.99796	0.66673	-0.58483	0.79832	0.99768	1.0136	0.019421	0.34418	0.9981	0.85471	-0.2265	0.16348	0.99995	0.85152	-0.23189
828.8102	10.03	lomics Pos	0.94694	0.99995	1.008	0.011526	0.52729	0.99979	1.0309	0.043843	0.015181	0.27304	0.91676	-0.12538	0.715	0.99996	1.0338	0.048018	0.39127	0.99701	0.95611	-0.06476	0.47145	0.99959	0.95537	-0.06587	0.010224	0.15929	1.2719	0.34696	0.98811	0.99984	1.0045	0.006519	0.54435	0.99966	1.0398	0.056305
189.1649	5.99	olomics Po	0.034167	0.99964	0.81003	-0.30396	0.01872	0.88953	0.72443	-0.46509	0.052047	0.99882	0.82523	-0.27713	0.45456	0.99987	0.9135	-0.13052	0.052125	0.95986	0.83671	-0.2572	0.1424	0.99796	0.75472	-0.40599	0.97398	0.99768	0.9927	-0.01058	0.12993	0.9981	0.76565	-0.38524	0.26842	0.99995	0.88174	-0.18158
229.2358	1.34	lomics Pos	0.52712	0.99995	0.93618	-0.09515	0.038347	0.99441	0.67966	-0.55711	0.085633	0.64149	0.7999	-0.32211	0.29872	0.99996	0.83526	-0.25971	0.1235	0.86372	0.79508	-0.33083	0.29048	0.99959	0.8076	-0.30828	0.18353	0.39604	1.1769	0.23499	0.28773	0.99984	0.81682	-0.29192	0.17367	0.99966	0.85474	-0.22645
533.2928	3.28	olomics Po	0.66908	0.99964	0.95612	-0.06474	0.39638	1	0.91167	-0.13341	0.018336	0.63482	0.72583	-0.4623	0.74032	0.99987	1.2011	0.26434	0.021159	0.95986	0.64868	-0.62441	0.70119	0.99796	1.1153	0.15747	0.68217	0.99768	1.3585	0.44202	0.97882	0.9981	1.0271	0.038589	0.87053	0.99995	1.004	0.00582
709.1169	4.49	lomics Pos	0.95037	0.99995	1.041	0.057942	0.92964	0.99979	0.97381	-0.03829	0.16625	0.7924	0.79482	-0.3313	0.75231	0.99996	1.2408	0.31128	0.026038	0.5516	0.55642	-0.84575	0.22433	0.99959	0.834	-0.26188	0.66964	0.80889	1.3888	0.47379	0.52591	0.99984	0.88796	-0.17143	0.91866	0.99966	1.0768	0.10679
270.28	6.55	olomics Po	0.02191	0.88137	0.79388	-0.33301	0.13543	1	0.77855	-0.36114	0.055728	0.99882	0.80961	-0.3047	0.31467	0.99987	0.89888	-0.1538	0.23255	0.95986	0.90386	-0.14583	0.31649	0.99796	0.84234	-0.24753	0.20733	0.99768	1.1327	0.17973	0.091502	0.9981	0.74104	-0.43238	0.21363	0.99995	0.87817	-0.18742
1112.786	10.02	lomics Pos	0.647	0.99995	1.0549	0.07716	0.03437	0.99441	1.1393	0.1882	0.68981	0.99601	0.97677	-0.03391	0.26941	0.99996	1.121	0.16473	0.75836	0.99701	1.0189	0.027019	0.11794	0.99959	1.2018	0.26514	0.001927	0.10065	1.3723	0.45661	0.43174	0.99984	1.1135	0.15516	0.103	0.99966	1.0982	0.13516
778.5722	9.25	olomics Po	0.29493	0.99964	1.2467	0.31812	0.42566	1	1.1206	0.16431	0.1166	0.99882	1.2586	0.33187	0.17141	0.99987	1.2319	0.30088	0.44654	0.95986	1.1914	0.25269	0.18995	0.99796	1.2363	0.30606	0.02418	0.57962	1.3994	0.48476	0.084743	0.9981	1.2688	0.34349	0.044632	0.99995	1.3961	0.48144
516.4262	9.81	lomics Pos	0.68446	0.99995	1.0678	0.094662	0.86912	0.99979	0.98163	-0.02674	0.45146	0.99046	0.91506	-0.12806	0.59886	0.99996	0.93124	-0.10278	0.64924	0.99701	0.94036	-0.08872	0.38277	0.99959	0.8946	-0.16069	0.009894	0.15738	1.4338	0.51981	0.59556	0.99984	0.93097	-0.1032	0.30199	0.99966	0.86148	-0.21511
536.9712	5.44	olomics Ne	0.066109	0.9951	1.0619	0.086596	0.10773	0.99975	1.0709	0.098767	0.42543	0.99731	0.97485	-0.03675	0.51512	0.99915	1.0488	0.068805	0.020905	0.50209	1.1032	0.14165	0.00887	0.45618	1.1527	0.20497	0.005265	0.052004	1.2221	0.28941	0.45895	0.99481	1.0313	0.044405	0.087311	0.91738	0.92189	-0.11734
453.9301	10.02	lomics Neg	0.92931	0.99486	1.0001	0.000211	0.48227	0.99506	1.0516	0.072609	0.14488	0.6372	1.2049	0.26895	0.18406	0.80547	1.0954	0.13147	0.031259	0.39567	1.1936	0.25528	0.30187	0.89898	1.0635	0.088791	0.50303	0.99905	1.0485	0.068272	0.48818	0.96955	1.0395	0.055932	0.325	0.74722	1.1127	0.15409
195.9133	0.1	olomics Po	0.039035	0.99964	0.84565	-0.24186	0.35734	1	0.76454	-0.38734	0.13684	0.99882	0.86569	-0.20809	0.34758	0.99987	0.90689	-0.141	0.9227	0.99906	0.99467	-0.00771	0.68797	0.99796	0.96504	-0.05134	0.50186	0.99768	0.94104	-0.08768	0.20834	0.9981	0.87956	-0.18514	0.3662	0.99995	0.77107	-0.37507
488.8834	0.54	lomics Pos	0.52218	0.99995	1.0246	0.035029	0.32926	0.99979	0.92482	-0.11275	0.005189	0.16899	0.84043	-0.25079	0.80571	0.99996	1.0006	0.000911	0.029066	0.57128	0.86192	-0.21438	0.16078	0.99959	0.90569	-0.14291	0.34339	0.56705	1.2512	0.32333	0.72109	0.99984	0.97622	-0.03472	0.68212	0.99966	1.0586	0.082205
238.2176	5.99	olomics Po	0.019461	0.86976	0.78924	-0.34147	0.1662	1	0.77635	-0.36522	0.21705	0.99882	0.85836	-0.22035	0.23089	0.99987	0.8901	-0.16796	0.31881	0.95986	0.90971	-0.13653	0.10495	0.99796	0.75604	-0.40346	0.71342	0.99768	1.0209	0.029889	0.14719	0.9981	0.76885	-0.37922	0.050492	0.99995	0.7717	-0.37388
496.9712	12	olomics Ne	0.51641	0.9951	1.0971	0.1337	0.16634	0.99975	1.0865	0.11965	0.75183	0.99731	0.97731	-0.03311	0.82913	0.99915	0.98325	-0.02438	0.06873	0.5958	1.1868	0.24713	0.3131	0.70078	1.1275	0.17307	0.005773	0.053834	1.2829	0.35941	0.71649	0.99481	0.97015	-0.04372	0.5013	0.95528	1.0685	0.095548
379.2531	4.96	lomics Pos	0.46632	0.99995	1.117	0.15966	0.62633	0.99979	1.0833	0.11539	0.5687	0.99601	1.0355	0.050391	0.013279	0.99996	1.1453	0.19575	0.051213	0.70556	1.1077	0.14756	0.86038	0.99959	0.99621	-0.00548	0.004587	0.12765	1.5075	0.59216	0.74649	0.99984	0.98416	-0.02303	0.16241	0.99966	1.1702	0.22673
398.891	10.15	lomics Pos	0.62267	0.99995	0.95628	-0.06449	0.20168	0.99979	0.91148	-0.13372	0.022913	0.32238	0.87127	-0.1988	0.98284	0.99996	1.0064	0.009149	0.56366	0.99701	0.95691	-0.06354	0.663	0.99959	0.9621	-0.05575	0.033056	0.20029	1.1905	0.25162	0.33647	0.99984	0.9367	-0.09435	0.74056	0.99966	1.0341	0.048307
1030.812	10.02	lomics Neg	0.24327	0.99486	1.2187	0.28533	0.44859	0.99506	1.0921	0.12713	0.071787	0.6372	1.3457	0.42834	0.19032	0.80547	1.1802	0.23899	0.045516	0.39567	1.3788	0.46339	0.43322	0.89898	1.0826	0.11455	0.17692	0.99905	1.1871	0.24744	0.32861	0.96955	1.1409	0.19016	0.43316	0.76776	1.1319	0.17868
681.9802	12.03	olomics Ne	0.25416	0.9951	1.1638	0.21884	0.75189	0.99975	1.0423	0.059785	0.96585	0.99731	0.97915	-0.03041	0.27446	0.99915	1.2181	0.28466	0.37715	0.75206	1.1067	0.14632	0.023678	0.4612	1.4776	0.56321	0.045324	0.11846	1.6103	0.6873	0.50481	0.99481	1.1521	0.20423	0.70484	0.97327	1.0371	0.052507
164.1527	5.99	olomics Po	0.32908	0.99964	0.87157	-0.19832	0.046356	0.88953	0.65661	-0.60688	0.58802	0.99882	0.92327	-0.11518	0.98845	0.99987	0.99941	-0.00086	0.4264	0.95986	0.89005	-0.16804	0.12183	0.99796	0.74552	-0.42368	0.68272	0.99768	0.93618	-0.09514	0.16547	0.9981	0.79195	-0.33652	0.30665	0.99995	0.85701	-0.22262
754.8775	11.91	olomics Po	0.23506	0.99964	0.93691	-0.09402	0.30277	1	0.9452	-0.08131	0.11801	0.99882	0.88437	-0.17727	0.036985	0.99987	0.88307	-0.1794	0.11411	0.95986	0.92561	-0.11153	0.008449	0.99128	0.84147	-0.24902	0.5236	0.99768	1.0586	0.082185	0.11445	0.9981	0.89276	-0.16365	0.3497	0.99995	0.92927	-0.10583
577.2839	1.28	lomics Pos	0.076675	0.99995	0.90789	-0.13942	0.99023	0.99979	1.002	0.002875	0.088832	0.65263	0.84173	-0.24858	0.36476	0.99996	0.93036	-0.10414	0.040359	0.64389	0.85242	-0.23036	0.54776	0.99959	0.97504	-0.03647	0.66787	0.80818	1.0461	0.065025	0.20853	0.99984	0.91742	-0.12434	0.51252	0.99966	1.0412	0.058233
504.9728	12	olomics Ne	0.097121	0.9951	1.1299	0.17618	0.04713	0.99975	1.217	0.28334	0.030818	0.99731	1.1507	0.20246	0.25013	0.99915	1.1046	0.14357	0.002387	0.26714	1.3407	0.42298	0.001786	0.45618	1.4342	0.52027	0.025342	0.08627	1.2578	0.33092	0.085122	0.99481	1.2649	0.33903	0.081424	0.91738	1.1365	0.18454
585.2291	1.29	lomics Pos	0.44304	0.99995	0.92116	-0.11847	0.2192	0.99979	0.86508	-0.2091	0.039244	0.42996	0.74887	-0.41722	0.52953	0.99996	0.92002	-0.12026	0.12706	0.86468	0.80376	-0.31516	0.70467	0.99959	0.95894	-0.06049	0.44519	0.66026	1.0732	0.10189	0.17769	0.99984	0.84627	-0.2408	0.88017	0.99966	1.0073	0.010485
498.9742	5.22	olomics Ne	0.46247	0.9951	0.96543	-0.05076	0.16832	0.99975	0.94162	-0.08679	0.091681	0.99731	0.93394	-0.0986	0.87006	0.99915	0.99211	-0.01143	0.7694	0.93601	1.011	0.01585	0.52781	0.80414	1.0283	0.040315	0.010878	0.061656	1.183	0.24241	0.80413	0.99481	0.98887	-0.01614	0.023225	0.91738	0.8957	-0.15892
1080.711	0.54	lomics Pos	0.14685	0.99995	0.91874	-0.12227	0.14457	0.99979	0.86813	-0.20402	0.033024	0.38971	0.84072	-0.2503	0.16756	0.99996	0.90757	-0.13991	0.069014	0.77855	0.86686	-0.20612	0.091121	0.99959	0.88768	-0.17188	0.36456	0.58727	1.07	0.097593	0.22631	0.99984	0.90042	-0.15134	0.43795	0.99966	0.90827	-0.1388
133.9833	10.12	lomics Pos	0.84005	0.99995	1.0139	0.019945	0.72718	0.99979	0.9785	-0.03135	0.25455	0.8744	0.94119	-0.08744	0.50929	0.99996	0.95233	-0.07046	0.15143	0.91944	0.91587	-0.12679	0.32892	0.99959	0.95303	-0.06941	0.031135	0.19986	1.2288	0.29729	0.53841	0.99984	0.96976	-0.0443	0.71658	0.99966	1.0194	0.027694
158.9648	10.03	lomics Pos	0.48427	0.99995	1.04	0.056514	0.83332	0.99979	1.0117	0.016718	0.029092	0.3649	0.94222	-0.08586	0.99717	0.99996	1.0059	0.008485	0.44939	0.99701	0.96321	-0.05409	0.8922	0.99959	0.99608	-0.00566	0.014213	0.16883	1.2584	0.33156	0.88706	0.99984	0.99658	-0.00495	0.49499	0.99966	1.0426	0.06012
331.9544	12.08	olomics Po	0.2055	0.99964	1.0788	0.10948	0.003469	0.88953	1.307	0.38621	0.25181	0.99882	1.0932	0.12853	0.29484	0.99987	1.0951	0.13108	0.096686	0.95986	1.1062	0.14566	0.39903	0.99796	1.0962	0.13245	0.2652	0.99768	1.1567	0.21006	0.16541	0.9981	1.115	0.15699	0.048561	0.99995	1.1783	0.23669
580.9059	12.05	olomics Po	0.43713	0.99964	0.9234	-0.11497	0.65951	1	0.9707	-0.0429	0.049005	0.99882	0.84709	-0.23942	0.49985	0.99987	0.96478	-0.05173	0.50639	0.95986	0.95987	-0.05908	0.48305	0.99796	0.9344	-0.09789	0.54984	0.99768	0.95015	-0.07377	0.91532	0.9981	1.0118	0.016955	0.80532	0.99995	0.98654	-0.01955
177.9651	12.24	olomics Po	0.77638	0.99964	0.97254	-0.04018	0.56708	1	1.0666	0.093083	0.92747	0.99882	1.0166	0.023804	0.81454	0.99987	1.01	0.014417	0.3901	0.95986	1.0688	0.095986	0.32856	0.99796	1.0734	0.10216	0.032949	0.65634	1.2486	0.32027	0.43534	0.9981	1.0752	0.10466	0.2783	0.99995	1.1072	0.14698
747.6069	5.55	lomics Pos	0.38421	0.99995	1.0947	0.13051	0.16536	0.99979	1.1257	0.17079	0.79778	0.99601	0.98768	-0.01788	0.28312	0.99996	1.0903	0.12467	0.88212	0.99701	1.0152	0.021746	0.31556	0.99959	1.0755	0.10495	0.004624	0.12777	1.2124	0.27785	0.22886	0.99984	1.0657	0.091756	0.37272	0.99966	1.0885	0.12234
457.235	5.98	olomics Ne	0.66982	0.9951	0.95245	-0.07028	0.007227	0.99975	0.69702	-0.52072	0.032667	0.99731	0.64527	-0.63203	0.11409	0.99915	0.83734	-0.25611	0.55847	0.8636	0.92622	-0.11057	0.009622	0.45618	0.72795	-0.45809	0.030141	0.093697	1.2944	0.37225	0.23814	0.99481	0.79475	-0.33142	0.30893	0.91738	0.85264	-0.22998
329.9967	0.58	lomics Pos	0.096571	0.99995	0.84966	-0.23505	0.10397	0.99979	0.84357	-0.24541	0.000787	0.13107	0.8115	-0.30133	0.018158	0.99996	0.76566	-0.38522	0.63544	0.99701	0.96007	-0.05879	0.50678	0.99959	1.0524	0.073623	0.000964	0.089845	1.5845	0.66402	0.013847	0.99984	0.74872	-0.41751	0.15504	0.99966	0.86625	-0.20714
820.7815	10.08	lomics Pos	0.4464	0.99995	1.0755	0.105	0.21335	0.99979	1.1046	0.1435	0.1229	0.72442	0.87515	-0.1924	0.58407	0.99996	1.0457	0.064487	0.71069	0.99701	0.9617	-0.05635	0.74598	0.99959	1.0494	0.069611	0.035624	0.20439	1.2627	0.33656	0.93506	0.99984	1.0244	0.03474	0.41446	0.99966	1.1098	0.15034
1142.733	10.05	lomics Pos	0.015166	0.68962	0.88739	-0.17237	0.56312	0.99979	0.95861	-0.06099	0.037383	0.424	0.87104	-0.19919	0.3451	0.99996	0.92556	-0.11161	0.047071	0.69031	0.8311	-0.2669	0.33095	0.99959	0.9559	-0.06506	0.002748	0.11265	1.1615	0.21596	0.001233	0.99984	0.87884	-0.18634	0.21074	0.99966	0.87984	-0.18469
86.0977	0.44	olomics Po	0.71214	0.99964	0.99012	-0.01432	0.97568	1	1.0813	0.11276	0.93814	0.99882	1.102	0.14006	0.18176	0.99987	1.4481	0.53419	0.006002	0.95986	1.7683	0.82234	0.43108	0.99796	1.0859	0.11891	0.000268	0.09364	1.9265	0.94595	0.15329	0.9981	0.86486	-0.20946	0.051929	0.99995	0.76436	-0.38768
298.8635	10.16	lomics Pos	0.1996	0.99995	1.1224	0.16661	0.65841	0.99979	0.95617	-0.06467	0.59289	0.99601	1.0448	0.063158	0.77134	0.99996	1.0148	0.021148	0.54813	0.99701	1.0603	0.08453	0.69519	0.99959	1.0443	0.062595	0.011916	0.16313	1.4073	0.49292	0.48401	0.99984	1.0858	0.11869	0.52301	0.99966	1.0877	0.12126
338.2696	5	olomics Po	0.68118	0.99964	1.0647	0.090466	0.98007	1	1.0182	0.025984	0.30353	0.99882	1.6167	0.69306	0.73011	0.99987	1.1196	0.16295	0.27136	0.95986	1.3331	0.41484	0.036402	0.99796	2.2232	1.1527	0.004052	0.31128	1.9989	0.99918	0.26631	0.9981	1.2987	0.37707	0.54063	0.99995	1.1293	0.17541
304.899	12.17	olomics Po	0.024399	0.92597	0.81535	-0.29451	0.93103	1	1.0042	0.006118	0.41462	0.99882	0.93912	-0.09061	0.63551	0.99987	0.96977	-0.04429	0.13742	0.95986	0.88331	-0.179	0.4982	0.99796	0.92058	-0.11938	0.44339	0.99768	0.94784	-0.07729	0.28279	0.9981	0.91462	-0.12876	0.46063	0.99995	0.9526	-0.07006
138.0538	0.59	lomics Pos	0.93248	0.99995	1.002	0.002826	0.8492	0.99979	1.0201	0.028686	0.68034	0.99601	0.96623	-0.04955	0.06296	0.99996	1.1361	0.18414	0.5181	0.99701	1.2323	0.30131	0.066339	0.99959	1.2905	0.36789	0.005474	0.1319	2.0841	1.0594	0.4663	0.99984	1.0611	0.085522	0.77855	0.99966	1.0367	0.052049
649.9772	12.02	olomics Ne	0.79269	0.9951	1.0225	0.032064	0.021888	0.99975	1.482	0.56752	0.58803	0.99731	0.92508	-0.11236	0.31194	0.99915	1.1383	0.18691	0.42481	0.78402	1.1544	0.20712	0.094876	0.52866	1.3909	0.47604	0.076889	0.16291	1.3359	0.41779	0.53221	0.99481	1.2083	0.27293	0.68069	0.96812	0.94758	-0.07768
620.6156	2.75	lomics Pos	0.49843	0.99995	0.76354	-0.38922	0.075186	0.99979	0.457	-1.1297	0.14857	0.76707	0.56133	-0.83307	0.70379	0.99996	0.99434	-0.00819	0.39861	0.99701	0.70253	-0.50937	0.032321	0.99959	0.32353	-1.628	0.75746	0.85922	0.8325	-0.26447	0.25223	0.99984	0.61091	-0.71098	0.22331	0.99966	0.61253	-0.70715
619.6138	2.75	lomics Pos	0.3658	0.99995	0.68295	-0.55016	0.034538	0.99441	0.37929	-1.3986	0.11092	0.70258	0.52926	-0.91796	0.52323	0.99996	0.87268	-0.19648	0.37547	0.99701	0.68738	-0.54082	0.021637	0.99959	0.28186	-1.8269	0.73093	0.84277	0.83046	-0.26803	0.30366	0.99984	0.64361	-0.63573	0.11652	0.99966	0.5021	-0.99396
312.0405	0.56	lomics Pos	0.97347	0.99995	1.0988	0.13591	0.65933	0.99979	0.94829	-0.0766	0.77369	0.99601	1.0907	0.12522	0.25578	0.99996	1.3035	0.38235	0.012891	0.48469	1.5893	0.66842	0.3861	0.99959	1.0973	0.13394	0.000792	0.089845	2.0478	1.0341	0.33636	0.99984	0.87787	-0.18792	0.11506	0.99966	0.7935	-0.3337
800.9722	12.01	olomics Ne	0.37311	0.9951	1.0544	0.076437	0.99915	0.99975	1.0027	0.003938	0.087581	0.99731	0.85343	-0.22865	0.053292	0.99915	1.1873	0.2477	0.84473	0.96264	1.0268	0.03822	0.9538	0.99141	1.0068	0.009713	0.030521	0.094164	1.1005	0.13818	0.66016	0.99481	0.96487	-0.0516	0.62757	0.96789	0.95795	-0.06198
472.9782	5.05	olomics Ne	0.45438	0.9951	1.0224	0.031954	0.59973	0.99975	1.0197	0.028101	0.4896	0.99731	1.0178	0.025523	0.44048	0.99915	1.0439	0.062029	0.096478	0.60285	1.1301	0.17644	0.013075	0.45618	1.121	0.16482	0.003401	0.050041	1.2426	0.31331	0.066934	0.99481	1.0835	0.11571	0.75779	0.97489	1.0226	0.032255
622.5705	0.32	olomics Po	0.72867	0.99964	1.0397	0.056139	0.051928	0.90372	1.1935	0.25516	0.43795	0.99882	1.1237	0.16828	0.035629	0.99987	1.1908	0.2519	0.22017	0.95986	1.0869	0.12016	0.051975	0.99796	1.1793	0.23793	0.57287	0.99768	1.0684	0.095458	0.16284	0.9981	1.1104	0.15112	0.20158	0.99995	1.1128	0.15424
470.814	9.92	lomics Pos	0.61687	0.99995	0.96208	-0.05577	0.8566	0.99979	0.99035	-0.014	0.20292	0.83096	0.89884	-0.15386	0.6756	0.99996	0.96872	-0.04585	0.94744	0.99701	1.017	0.024338	0.52985	0.99959	1.0462	0.065137	0.02925	0.19804	1.1779	0.23616	0.83752	0.99984	1.0322	0.04568	0.47021	0.99966	1.0289	0.041086
723.4961	10.67	olomics Ne	0.087881	0.9951	1.2526	0.32493	0.82699	0.99975	1.017	0.02433	0.71709	0.99731	1.0282	0.040188	0.49656	0.99915	1.0751	0.10447	0.42922	0.78613	1.08	0.11104	0.18971	0.62443	1.1523	0.20448	0.009122	0.059819	1.3851	0.46994	0.17301	0.99481	1.1709	0.22762	0.61454	0.96789	0.94016	-0.08902
304.9831	12	olomics Ne	0.78456	0.9951	1.0352	0.049937	0.40103	0.99975	1.2382	0.30827	0.66411	0.99731	1.0393	0.055636	0.21084	0.99915	1.2944	0.37233	0.21162	0.70038	1.434	0.52	0.33454	0.71392	1.4143	0.50011	0.04189	0.11408	1.2513	0.32346	0.41694	0.99481	1.2587	0.33191	0.89812	0.99423	0.98432	-0.02279
631.5229	6.38	olomics Po	0.26327	0.99964	0.88704	-0.17293	0.15137	1	0.82695	-0.27412	0.15386	0.99882	0.84664	-0.24018	0.6937	0.99987	0.96579	-0.05022	0.79201	0.99139	0.97853	-0.03132	0.45658	0.99796	0.92179	-0.11749	0.012398	0.43304	1.2419	0.31259	0.063287	0.9981	0.81588	-0.29358	0.57355	0.99995	0.94294	-0.08476
551.633	7.69	olomics Po	0.052097	0.99964	1.9526	0.96541	0.39985	1	1.5816	0.66139	0.3439	0.99882	1.5618	0.64322	0.045333	0.99987	1.9893	0.99223	0.044146	0.95986	2.05	1.0356	0.10487	0.99796	4.3355	2.1162	0.47504	0.99768	1.4166	0.50243	0.026602	0.9981	2.053	1.0377	0.090252	0.99995	1.7617	0.81694
506.9759	5.11	olomics Ne	0.36099	0.9951	1.0386	0.05464	0.71995	0.99975	1.0126	0.018015	0.22573	0.99731	0.97068	-0.04294	0.75599	0.99915	1.0265	0.037744	0.083271	0.5958	1.0713	0.099423	0.029823	0.4612	1.1097	0.15011	0.002812	0.050041	1.2198	0.28659	0.095113	0.99481	1.0462	0.065102	0.78869	0.97489	0.98841	-0.01681
268.6506	1.3	lomics Pos	0.85162	0.99995	1.0565	0.079276	0.87911	0.99979	0.97517	-0.03628	0.24189	0.858	0.85221	-0.23073	0.90826	0.99996	0.99866	-0.00194	0.9276	0.99701	1.0435	0.061475	0.40798	0.99959	1.1222	0.16633	0.045058	0.21733	1.2902	0.36764	0.29751	0.99984	1.1611	0.21554	0.41936	0.99966	1.1271	0.17267
794.561	9.05	olomics Ne	0.10709	0.9951	1.1679	0.22392	0.66638	0.99975	0.97041	-0.04333	0.79958	0.99731	1.0352	0.049966	0.30136	0.99915	1.1139	0.15562	0.020342	0.50209	1.164	0.21908	0.37877	0.75758	1.1804	0.23923	0.015642	0.067031	1.3536	0.43676	0.46267	0.99481	1.1212	0.16499	0.67559	0.96789	0.97162	-0.04154
715.517	10.15	olomics Ne	0.47135	0.9951	1.0795	0.11041	0.2043	0.99975	1.1547	0.20754	0.64495	0.99731	1.2119	0.27723	0.61547	0.99915	1.0772	0.10726	0.38286	0.75206	1.0781	0.10843	0.46901	0.79091	1.0971	0.13364	0.033706	0.10038	1.5016	0.58646	0.079795	0.99481	1.2639	0.33788	0.72115	0.97327	0.95642	-0.06429
182.1556	5.99	olomics Po	0.12513	0.99964	0.84795	-0.23794	0.07912	0.99372	0.78705	-0.34546	0.019347	0.63482	0.79154	-0.33727	0.76455	0.99987	0.97368	-0.03849	0.11132	0.95986	0.87036	-0.20031	0.26158	0.99796	0.82311	-0.28084	0.25722	0.99768	1.0854	0.11825	0.11844	0.9981	0.76335	-0.38958	0.21215	0.99995	0.88342	-0.17883
195.1752	6.38	olomics Po	0.67285	0.99964	0.97113	-0.04227	0.017861	0.88953	0.65021	-0.62101	0.19884	0.99882	0.87331	-0.19543	0.44312	0.99987	0.93298	-0.10009	0.51646	0.95986	0.967	-0.04841	0.055251	0.99796	0.75933	-0.39719	0.75921	0.99768	1.0154	0.022052	0.041973	0.9981	0.84441	-0.24398	0.10964	0.99995	0.80992	-0.30415
107.0868	6.38	olomics Po	0.068094	0.99964	0.85183	-0.23137	0.005862	0.88953	0.75873	-0.39835	0.003443	0.43007	0.84308	-0.24625	0.37344	0.99987	0.91901	-0.12185	0.25368	0.95986	0.91658	-0.12567	0.041912	0.99796	0.74391	-0.4268	0.97279	0.99768	1.0039	0.005629	0.13533	0.9981	0.84368	-0.24523	0.13222	0.99995	0.86045	-0.21684
185.1735	1.66	lomics Pos	0.057941	0.99995	0.82853	-0.27137	0.029089	0.99441	0.76152	-0.39305	0.001894	0.13302	0.71539	-0.4832	0.43875	0.99996	0.91444	-0.12905	0.10489	0.84731	0.84036	-0.25093	0.13445	0.99959	0.78416	-0.35079	0.32099	0.54349	1.1432	0.19309	0.22193	0.99984	0.80305	-0.31644	0.044813	0.99966	0.85858	-0.21998
185.0779	4.66	lomics Pos	0.86929	0.99995	1.0349	0.049423	0.26616	0.99979	1.0884	0.12218	0.69276	0.99601	1.0451	0.063633	0.92289	0.99996	1.0187	0.026673	0.84329	0.99701	0.98267	-0.02521	0.97768	0.99959	0.99769	-0.00334	0.022643	0.18641	1.3811	0.46582	0.51364	0.99984	1.0883	0.12209	0.030205	0.99966	1.2337	0.303
182.1551	1.67	lomics Pos	0.065894	0.99995	0.8968	-0.15714	0.049044	0.99441	0.78662	-0.34625	0.00802	0.19873	0.73872	-0.4369	0.38905	0.99996	0.88988	-0.16831	0.058697	0.76299	0.81228	-0.29995	0.25591	0.99959	0.83036	-0.26819	0.13564	0.34138	1.2143	0.28017	0.32216	0.99984	0.84489	-0.24316	0.19068	0.99966	0.90271	-0.14767
217.1983	5.37	olomics Po	0.22266	0.99964	0.8864	-0.17397	0.7448	1	0.96996	-0.04401	0.11147	0.99882	0.7801	-0.35827	0.55987	0.99987	0.94568	-0.08058	0.055585	0.95986	0.80846	-0.30674	0.054492	0.99796	0.72976	-0.45451	0.47196	0.99768	0.92181	-0.11747	0.025798	0.9981	0.77874	-0.36079	0.62244	0.99995	0.96356	-0.05355
192.1832	2.05	lomics Pos	0.004524	0.60743	0.81849	-0.28897	0.006378	0.99441	0.66179	-0.59556	0.002644	0.14613	0.68412	-0.54767	0.295	0.99996	0.86537	-0.2086	0.076309	0.7908	0.76814	-0.38055	0.056027	0.99959	0.70587	-0.50252	0.7642	0.86398	1.041	0.05795	0.036135	0.99984	0.73279	-0.44854	0.000644	0.99749	0.75797	-0.39978
464.3741	8.13	olomics Po	0.93345	0.99964	1.0066	0.009442	0.40912	1	1.0781	0.10845	0.35645	0.99882	1.0364	0.051522	0.91571	0.99987	1.0108	0.015544	0.88242	0.99904	1.0079	0.011299	0.96074	0.99796	1.0147	0.021037	0.027783	0.60775	1.1218	0.16581	0.85379	0.9981	1.011	0.015728	0.5753	0.99995	1.0234	0.033321
1110.786	10.02	lomics Pos	0.10481	0.99995	1.0923	0.12739	0.32972	0.99979	1.0422	0.059681	0.2583	0.8768	0.9659	-0.05005	0.84469	0.99996	1.0168	0.023973	0.74446	0.99701	0.9872	-0.01858	0.76892	0.99959	1.017	0.02425	0.022431	0.18641	1.2389	0.30902	0.91782	0.99984	0.99857	-0.00206	0.52224	0.99966	1.0592	0.082937
522.9758	5.25	olomics Ne	0.37204	0.9951	1.047	0.066235	0.74369	0.99975	1.0144	0.020563	0.87569	0.99731	1.0096	0.01378	0.46342	0.99915	1.0398	0.056343	0.38966	0.75404	1.0355	0.05037	0.12645	0.54162	1.1085	0.14857	0.008091	0.057907	1.2097	0.27466	0.31934	0.99481	1.0576	0.080853	0.58507	0.96789	1.0347	0.049231
970.6163	10.23	olomics Ne	0.91994	0.9951	0.9934	-0.00955	0.91005	0.99975	1.0043	0.006163	0.45173	0.99731	0.94267	-0.08517	0.81804	0.99915	1.0083	0.01192	0.07639	0.5958	1.1597	0.21372	0.44478	0.77799	1.1064	0.14586	0.015598	0.067031	1.2232	0.29062	0.68227	0.99481	1.0334	0.04736	0.16372	0.91738	0.90822	-0.13888
430.9146	10.03	lomics Pos	0.35133	0.99995	1.0576	0.08084	0.74776	0.99979	1.0178	0.02543	0.072153	0.58912	0.96337	-0.05384	0.89752	0.99996	1.0127	0.018199	0.64105	0.99701	0.98118	-0.02742	0.96102	0.99959	1.0078	0.011171	0.012907	0.16313	1.2668	0.34117	0.89256	0.99984	1.0175	0.024975	0.45719	0.99966	1.0545	0.076523
125.9869	10.24	lomics Pos	0.43641	0.99995	1.0489	0.068908	0.58581	0.99979	1.038	0.05385	0.1074	0.68942	0.95368	-0.06843	0.417	0.99996	1.0839	0.11629	0.37115	0.99701	0.96484	-0.05164	0.97351	0.99959	1.0015	0.002131	0.02845	0.19688	1.2099	0.27487	0.96809	0.99984	1.0184	0.026356	0.88197	0.99966	1.009	0.012898
123.1181	6.38	olomics Po	0.1376	0.99964	0.92745	-0.10866	0.01139	0.88953	0.79534	-0.33036	0.038488	0.90186	0.90625	-0.14202	0.85721	0.99987	0.99226	-0.01121	0.52751	0.95986	0.97132	-0.04198	0.13517	0.99796	0.79083	-0.33856	0.052629	0.77855	1.0867	0.11998	0.23982	0.9981	0.88635	-0.17405	0.24236	0.99995	0.87718	-0.18906
217.1951	4.73	lomics Pos	0.32538	0.99995	1.1111	0.15198	0.21826	0.99979	1.1384	0.18698	0.59508	0.99601	0.95229	-0.07053	0.32317	0.99996	1.1188	0.16195	0.3606	0.99701	1.093	0.12828	0.048971	0.99959	1.3479	0.43071	0.026763	0.19234	1.4207	0.50665	0.069107	0.99984	1.2231	0.29058	0.10504	0.99966	1.2327	0.30186
1138.25	9.01	olomics Po	0.421	0.99964	0.89095	-0.16659	0.2009	1	0.86225	-0.21383	0.091505	0.99882	0.73483	-0.44452	0.5258	0.99987	0.93147	-0.10241	0.036232	0.95986	0.59627	-0.74596	0.064951	0.99796	0.71714	-0.47968	0.85247	0.99768	1.2404	0.31081	0.17225	0.9981	0.85731	-0.22212	0.91124	0.99995	1.1164	0.15888
302.018	0.57	lomics Pos	0.96646	0.99995	1.0562	0.078947	0.57343	0.99979	0.93069	-0.10362	0.94382	0.99702	1.0522	0.073422	0.21759	0.99996	1.2465	0.31783	0.014177	0.4899	1.5665	0.64753	0.80114	0.99959	0.98365	-0.02379	0.000947	0.089845	1.8426	0.88172	0.30038	0.99984	0.89547	-0.15928	0.087068	0.99966	0.76548	-0.38557
178.9773	12.76	olomics Ne	0.33943	0.9951	1.0648	0.09063	0.3318	0.99975	1.0618	0.086478	0.35781	0.99731	0.93957	-0.08993	0.21546	0.99915	1.0767	0.10658	0.13125	0.6311	1.1012	0.13905	0.27044	0.66152	1.0744	0.10356	0.005148	0.052004	1.3114	0.39109	0.31333	0.99481	1.0759	0.10551	0.67697	0.96789	1.0205	0.029247
394.2649	7.02	olomics Po	0.93227	0.99964	1.0085	0.012279	0.060418	0.93223	0.81481	-0.29547	0.02731	0.78784	0.80301	-0.31652	0.03692	0.99987	0.88831	-0.17086	0.62653	0.96686	1.0395	0.055904	0.17145	0.99796	0.83651	-0.25754	0.071411	0.82924	1.3001	0.37864	0.44392	0.9981	0.90803	-0.13919	0.743	0.99995	1.0473	0.066692
177.1084	0.58	lomics Pos	0.501	0.99995	0.98985	-0.01472	0.36209	0.99979	1.033	0.046775	0.89696	0.99702	0.99699	-0.00435	0.29363	0.99996	1.0452	0.063801	0.19624	0.97344	1.0516	0.072533	0.002006	0.99959	1.1269	0.17239	0.012223	0.16313	1.3101	0.38972	0.35593	0.99984	1.023	0.032833	0.056822	0.99966	1.0456	0.064278
404.3097	2.06	lomics Pos	0.51581	0.99995	0.9688	-0.04574	0.027196	0.99441	0.83082	-0.2674	0.058336	0.53292	0.83267	-0.26417	0.12987	0.99996	0.8575	-0.22179	0.002618	0.46849	0.81366	-0.2975	0.21839	0.99959	0.85389	-0.22788	0.12843	0.33008	1.2252	0.29302	0.28924	0.99984	0.88961	-0.16875	0.19307	0.99966	0.87197	-0.19765
384.3704	8.84	olomics Po	0.59428	0.99964	0.9706	-0.04306	0.74917	1	1.0127	0.018226	0.34882	0.99882	0.92507	-0.11236	0.51023	0.99987	0.94945	-0.07484	0.15933	0.95986	1.2385	0.30858	0.17881	0.99796	1.1626	0.21736	0.02261	0.5728	1.3347	0.41653	0.25135	0.9981	1.1464	0.19706	0.80837	0.99995	0.98856	-0.0166
482.808	12.68	olomics Ne	0.51694	0.9951	1.0452	0.063729	0.14455	0.99975	1.1218	0.16576	0.28075	0.99731	0.92398	-0.11407	0.76359	0.99915	1.029	0.041243	0.16567	0.65155	1.0863	0.11947	0.18006	0.61062	1.152	0.20415	0.01539	0.067031	1.2147	0.28058	0.40086	0.99481	1.0565	0.079257	0.77519	0.97489	0.97841	-0.03148
152.145	1.66	lomics Pos	0.9148	0.99995	1.003	0.004293	0.21363	0.99979	0.80493	-0.31306	0.53458	0.99601	0.92939	-0.10564	0.37776	0.99996	0.90182	-0.14909	0.32642	0.99701	0.91252	-0.13207	0.59649	0.99959	0.93761	-0.09294	0.018827	0.17894	1.3586	0.44212	0.44549	0.99984	0.88594	-0.17472	0.72875	0.99966	1.04	0.056517
202.1814	0.34	olomics Po	0.14518	0.99964	1.1165	0.15895	0.83792	1	0.98479	-0.02212	0.9759	0.99882	0.99494	-0.00732	0.95202	0.99987	1.0181	0.025899	0.25189	0.95986	0.82858	-0.2713	0.79849	0.99796	0.97884	-0.03086	0.038068	0.66991	0.76839	-0.38008	0.56953	0.9981	1.1376	0.18596	0.5304	0.99995	1.1206	0.16431
301.7566	6.38	olomics Po	0.019294	0.86976	0.76767	-0.38143	0.10488	1	0.81121	-0.30185	0.005952	0.51418	0.73041	-0.45322	0.22889	0.99987	0.90136	-0.14983	0.16919	0.95986	0.81921	-0.2877	0.092759	0.99796	0.81559	-0.29408	0.77928	0.99768	1.0161	0.022976	0.042212	0.9981	0.7261	-0.46176	0.13205	0.99995	0.87992	-0.18456
262.0501	10.16	lomics Pos	0.45224	0.99995	1.1526	0.20485	0.41194	0.99979	1.0801	0.11117	0.34125	0.92676	0.89953	-0.15275	0.903	0.99996	1.0205	0.029241	0.4527	0.99701	0.92874	-0.10665	0.70378	0.99959	1.035	0.04964	0.12126	0.32366	1.2429	0.3137	0.026468	0.99984	0.78867	-0.3425	0.40638	0.99966	1.1287	0.17464
677.5196	2.05	lomics Pos	0.24805	0.99995	0.82523	-0.27713	0.035763	0.99441	0.57141	-0.8074	0.025646	0.34194	0.62536	-0.67725	0.10232	0.99996	0.66633	-0.58569	0.23629	0.99701	0.82178	-0.28318	0.12563	0.99959	0.72693	-0.46012	0.57854	0.75676	1.0948	0.13066	0.070582	0.99984	0.6716	-0.57433	0.24331	0.99966	0.78639	-0.34668
395.2486	2.05	lomics Pos	0.19191	0.99995	0.88966	-0.16867	0.03735	0.99441	0.79178	-0.33683	0.036858	0.41952	0.8222	-0.28244	0.47353	0.99996	0.92655	-0.11006	0.1213	0.86273	0.83279	-0.26397	0.92649	0.99959	1.0111	0.015984	0.004826	0.12958	1.3953	0.48056	0.085813	0.99984	0.80031	-0.32136	0.43563	0.99966	0.9368	-0.09419
402.8279	0.3	olomics Po	0.014417	0.83315	1.1557	0.20875	0.14299	1	1.2032	0.2669	0.047631	0.99882	1.1336	0.18094	0.36156	0.99987	1.1236	0.16815	0.10802	0.95986	1.1222	0.16637	0.008011	0.99128	1.1401	0.18915	0.58774	0.99768	1.0581	0.081476	0.13171	0.9981	1.1471	0.198	0.10734	0.99995	1.1382	0.18679
1129.741	10.05	lomics Pos	0.23492	0.99995	1.2073	0.27184	0.40591	0.99979	1.1426	0.1923	0.45654	0.99153	1.0617	0.086355	0.52051	0.99996	1.0693	0.096691	0.99111	0.9977	0.99767	-0.00336	0.16839	0.99959	1.2305	0.29927	0.002906	0.11425	1.5964	0.67484	0.83398	0.99984	1.0409	0.057895	0.61522	0.99966	1.0791	0.10982
809.5466	8.51	olomics Ne	0.75727	0.9951	1.0831	0.11514	0.64296	0.99975	1.0456	0.064286	0.35104	0.99731	1.1182	0.16113	0.67451	0.99915	1.1038	0.14249	0.084069	0.5958	1.1471	0.19799	0.36379	0.74467	1.1073	0.14708	0.045011	0.1182	1.2478	0.31943	0.32312	0.99481	1.1918	0.25316	0.6418	0.96789	0.94747	-0.07785
503.2743	3.23	olomics Po	0.79859	0.99964	1.0165	0.02365	0.21942	1	0.87256	-0.19667	0.014592	0.60995	0.6239	-0.68062	0.98103	0.99987	1.0881	0.12187	0.05161	0.95986	0.70939	-0.49536	0.61602	0.99796	0.94002	-0.08923	0.93267	0.99768	1.1969	0.25934	0.81226	0.9981	1.0037	0.005371	0.68414	0.99995	0.97645	-0.03439
915.7864	0.52	lomics Pos	0.25177	0.99995	1.1027	0.14099	0.47427	0.99979	1.0563	0.079039	0.51293	0.99601	1.0279	0.039741	0.69079	0.99996	0.97102	-0.04243	0.66592	0.99701	0.9661	-0.04975	0.41699	0.99959	1.0712	0.099224	0.032084	0.20029	1.1669	0.22266	0.58382	0.99984	1.0522	0.073357	0.19385	0.99966	1.1778	0.23608
572.2681	10.11	olomics Ne	0.45014	0.9951	1.0686	0.095783	0.2104	0.99975	1.1042	0.14305	0.81828	0.99731	1.0283	0.040237	0.81354	0.99915	0.98165	-0.02672	0.3077	0.73124	1.15	0.20167	0.029687	0.4612	1.2966	0.37471	0.03883	0.11014	1.4144	0.50023	0.15611	0.99481	1.2095	0.27438	0.10329	0.91738	1.1981	0.26079
291.269	6.63	olomics Po	0.0105	0.68905	0.80198	-0.31836	0.040196	0.88953	0.69025	-0.53481	0.010966	0.58546	0.77143	-0.37439	0.72458	0.99987	0.97126	-0.04207	0.081432	0.95986	0.81958	-0.28705	0.18959	0.99796	0.85121	-0.23241	0.47683	0.99768	1.058	0.081286	0.055518	0.9981	0.76266	-0.39088	0.067817	0.99995	0.87762	-0.18833
332.2651	6.53	olomics Po	0.88828	0.99964	1.0972	0.13386	0.36641	1	0.85098	-0.23281	0.87471	0.99882	0.97784	-0.03233	0.8545	0.99987	1.0312	0.044364	0.80825	0.99139	1.1865	0.24671	0.021511	0.99796	0.47039	-1.0881	0.08415	0.8482	0.61415	-0.70334	0.93365	0.9981	1.0424	0.05992	0.34373	0.99995	0.80414	-0.31448
453.2909	1.65	lomics Pos	0.15111	0.99995	0.81823	-0.28942	0.39568	0.99979	0.87178	-0.19797	0.02501	0.34014	0.68532	-0.54515	0.66869	0.99996	0.93562	-0.096	0.18458	0.96528	0.81308	-0.29853	0.25433	0.99959	0.79148	-0.33737	0.035665	0.20439	1.4594	0.54541	0.50986	0.99984	0.88975	-0.16853	0.8617	0.99966	1.0244	0.034722
330.9797	4.21	olomics Ne	0.33527	0.9951	1.0321	0.045557	0.23758	0.99975	1.0721	0.10042	0.26186	0.99731	0.96041	-0.05827	0.38045	0.99915	1.0556	0.078059	0.007612	0.49556	1.089	0.123	0.082213	0.52711	1.1242	0.16891	0.000384	0.041313	1.2475	0.31907	0.31127	0.99481	1.0522	0.073476	0.30729	0.91738	0.96607	-0.0498

220.0688	0.58	olomics Pos	0.60951	0.99995	1.0847	0.11725	0.2871	0.99979	0.89635	-0.15786	0.71848	0.99601	0.96637	-0.04936	0.28382	0.99996	1.135	0.18266	0.013667	0.4899	1.5183	0.60247	0.59052	0.99959	1.1677	0.2237	0.000544	0.089845	2.1164	1.0816	0.5392	0.99984	1.0966	0.13308	0.33466	0.99966	1.1522	0.20438
304.2621	6.39	olomics Pc	0.46074	0.99964	0.94051	-0.08848	0.018843	0.88953	0.79268	-0.33519	0.39238	0.99882	0.92733	-0.10885	0.8162	0.99987	0.97912	-0.03044	0.9607	0.99906	1.0033	0.004763	0.1268	0.99796	0.84233	-0.24755	0.32397	0.99768	1.0866	0.11987	0.32109	0.9981	0.90257	-0.14789	0.46104	0.99995	0.90979	-0.13639
199.8085	12.27	olomics Ne	0.22733	0.9951	1.0344	0.048741	0.12822	0.99975	1.0685	0.095545	0.79828	0.99731	0.99039	-0.01393	0.2574	0.99915	1.0643	0.089839	0.052114	0.58152	1.1045	0.14341	0.23504	0.64348	1.0734	0.10219	0.000906	0.041313	1.2759	0.35149	0.33619	0.99481	1.0271	0.038607	0.3223	0.91738	0.9618	-0.05619
274.9792	4.01	olomics Ne	0.9875	0.99691	0.99704	-0.00428	0.88046	0.99975	0.99128	-0.01263	0.3052	0.99731	0.94871	-0.07596	0.92614	0.99915	1.0052	0.007493	0.48303	0.82931	1.0336	0.047728	0.28088	0.6688	1.062	0.086786	0.013809	0.065382	1.1925	0.25396	0.79885	0.99481	1.0135	0.019305	0.25573	0.91738	0.92607	-0.1108
534.294	2.94	olomics Pc	0.83838	0.99964	0.98201	-0.02619	0.059307	0.93188	0.76045	-0.39508	0.026382	0.77259	0.72052	-0.47289	0.34709	0.99987	0.8514	-0.23208	0.056241	0.95986	0.71514	-0.4837	0.75269	0.99796	0.97755	-0.03276	0.58526	0.99768	1.2991	0.37748	0.89606	0.9981	1.0234	0.033326	0.34503	0.99995	0.86026	-0.21715
1135.751	10.02	olomics Pos	0.2667	0.99995	1.125	0.16986	0.90818	0.99979	0.99673	-0.00472	0.45069	0.99046	1.027	0.038395	0.037031	0.99996	1.1564	0.20964	0.6026	0.99701	1.1114	0.15237	0.5055	0.99959	1.1229	0.16718	0.003705	0.12074	1.5037	0.58855	0.40195	0.99984	1.1548	0.20759	0.88722	0.99966	1.0347	0.049164
912.7776	10.08	olomics Pos	0.13961	0.99995	1.1237	0.16821	0.64089	0.99979	0.97444	-0.03736	0.77498	0.99601	1.0161	0.022972	0.23186	0.99996	1.0565	0.079231	0.54953	0.99701	1.0255	0.036361	0.52391	0.99959	1.091	0.12561	0.008326	0.14619	1.2327	0.30188	0.2303	0.99984	1.0882	0.12195	0.13237	0.99966	1.1124	0.15365
228.9566	10.03	olomics Pos	0.86467	0.99995	0.99338	-0.00959	0.47805	0.99979	1.0316	0.044879	0.02684	0.34652	0.90778	-0.13958	0.79915	0.99996	0.98695	-0.01895	0.27378	0.99701	0.93214	-0.10139	0.76293	0.99959	1.0367	0.052006	0.097061	0.29349	1.1846	0.24439	0.57873	0.99984	0.96655	-0.04908	0.68078	0.99966	1.0324	0.04604
321.7674	2.06	olomics Pos	0.077696	0.99995	0.85384	-0.22796	0.002421	0.99441	0.61538	-0.70046	0.012977	0.24793	0.69822	-0.51824	0.0379	0.99996	0.76379	-0.38874	0.10857	0.85684	0.87052	-0.20006	0.066454	0.99959	0.65266	-0.61559	0.24818	0.46731	1.201	0.26422	0.38772	0.99984	0.85822	-0.22058	0.1091	0.99966	0.83008	-0.26867
904.9743	12.02	olomics Ne	0.68563	0.9951	1.0374	0.052997	0.48927	0.99975	1.0832	0.11531	0.17047	0.99731	1.3283	0.4096	0.23172	0.99915	1.2002	0.26327	0.065083	0.5958	1.1718	0.22871	0.04736	0.49377	1.2737	0.34908	0.011219	0.061656	1.4798	0.56543	0.22378	0.99481	1.1879	0.24838	0.9319	0.99833	1.0031	0.004418
446.3585	2.05	olomics Pos	0.11334	0.99995	0.81871	-0.28857	0.18727	0.99979	0.82259	-0.28176	0.062635	0.55542	0.74368	-0.42725	0.68906	0.99996	0.95825	-0.06152	0.037006	0.62436	0.80615	-0.31087	0.60869	0.99959	0.93688	-0.09407	0.003355	0.11893	1.7465	0.8045	0.14481	0.99984	0.77405	-0.3695	0.50152	0.99966	0.93151	-0.10236
538.9773	5.13	olomics Ne	0.79041	0.9951	1.0215	0.03072	0.38382	0.99975	1.031	0.043988	0.7717	0.99731	0.98838	-0.01686	0.2817	0.99915	1.0643	0.089961	0.050369	0.58152	1.1039	0.14259	0.016833	0.45618	1.1911	0.25235	0.000296	0.041313	1.3257	0.4067	0.28331	0.99481	1.065	0.090788	0.85457	0.99149	0.99288	-0.01031
314.9742	12	olomics Ne	0.76325	0.9951	0.97978	-0.02948	0.43123	0.99975	1.0633	0.088567	0.42148	0.99731	0.94227	-0.08578	0.32527	0.99915	1.1222	0.16633	0.2525	0.71555	1.0657	0.091868	0.6088	0.84705	1.0356	0.050457	0.036839	0.10546	1.2424	0.31312	0.61691	0.99481	0.96867	-0.04592	0.32465	0.91738	0.93588	-0.0956
294.2804	6.21	olomics Pc	0.73416	0.99964	0.97284	-0.03973	0.069318	0.9925	0.81299	-0.2987	0.68593	0.99882	0.96461	-0.05198	0.84314	0.99987	0.98992	-0.01462	0.025516	0.95986	0.71561	-0.48276	0.33457	0.99796	0.91578	-0.12692	0.85358	0.99768	0.99015	-0.01428	0.82119	0.9981	0.98834	-0.01692	0.40086	0.99995	0.89041	-0.16746
841.6111	10.2	olomics Ne	0.177	0.9951	1.1956	0.25772	0.21569	0.99975	1.1793	0.23793	0.45564	0.99731	1.129	0.17509	0.5475	0.99915	1.0831	0.11523	0.29201	0.71896	1.1668	0.2226	0.17656	0.60765	1.2434	0.31433	0.029869	0.093322	1.4043	0.48987	0.8524	0.99481	1.0095	0.013613	0.74353	0.97489	1.0331	0.046911
180.1867	2.05	olomics Pos	0.009781	0.68962	0.64571	-0.63104	0.015707	0.99441	0.63873	-0.64673	0.12363	0.72547	0.71801	-0.47792	0.021163	0.99996	0.67379	-0.56962	0.14268	0.90317	0.79418	-0.33246	0.24166	0.99959	0.77538	-0.36702	0.44635	0.66146	1.0786	0.10917	0.32586	0.99984	0.81465	-0.29575	0.039806	0.99966	0.71273	-0.48858
826.5589	8.59	olomics Ne	0.69382	0.9951	0.96954	-0.04462	0.86444	0.99975	0.98681	-0.01916	0.48652	0.99731	0.94596	-0.08016	0.96981	0.99915	0.99583	-0.00603	0.32799	0.74138	1.0927	0.12788	0.084009	0.52866	1.1415	0.19095	0.009068	0.059819	1.2413	0.3118	0.37387	0.99481	1.0813	0.11276	0.8516	0.98898	1.0063	0.009083
421.7943	5.94	olomics Pc	0.12639	0.99964	0.92175	-0.11755	0.16629	1	0.85435	-0.2271	0.19178	0.99882	0.94254	-0.08537	0.86422	0.99987	1.0315	0.044679	0.17742	0.95986	0.85675	-0.22305	0.28408	0.99796	0.86814	-0.20401	0.28714	0.99768	1.0853	0.11807	0.049878	0.9981	0.73909	-0.43617	0.76615	0.99995	0.98213	-0.02602
262.992	3.61	olomics Ne	0.23331	0.9951	1.0382	0.054028	0.30881	0.99975	1.0524	0.073691	0.29213	0.99731	0.95858	-0.06103	0.33019	0.99915	1.075	0.10429	0.023629	0.50209	1.101	0.13882	0.028347	0.4612	1.0982	0.1352	0.015426	0.067031	1.2627	0.33651	0.47667	0.99481	1.032	0.045389	0.47657	0.94605	0.95436	-0.06739
473.9823	12.03	olomics Ne	0.056271	0.9951	1.4722	0.55796	0.046051	0.99975	1.4839	0.5694	0.01053	0.99731	1.4571	0.54308	0.016813	0.99915	1.4764	0.5621	0.097051	0.60285	1.3651	0.44903	0.041113	0.48299	1.4088	0.49448	0.011126	0.061656	1.5622	0.6436	0.35022	0.99481	1.1983	0.26101	0.31417	0.91738	1.1893	0.25008
526.9759	5.09	olomics Ne	0.15944	0.9951	1.0595	0.08333	0.68446	0.99975	0.98494	-0.02189	0.80164	0.99731	0.99006	-0.01442	0.54612	0.99915	1.0255	0.036332	0.031194	0.51891	1.1118	0.15283	0.015913	0.45618	1.1059	0.14523	0.000481	0.041313	1.2416	0.31222	0.14313	0.99481	1.0826	0.1145	0.67084	0.96789	0.9751	-0.03637
578.9743	0.52	olomics Pos	0.051096	0.99995	1.2205	0.28743	0.9072	0.99979	1.0128	0.018371	0.65728	0.99601	0.96942	-0.0448	0.93722	0.99996	1.0031	0.004411	0.2378	0.99701	0.93033	-0.10419	0.63773	0.99959	0.96727	-0.04801	0.047403	0.22069	1.2617	0.33538	0.49219	0.99984	1.0799	0.1109	0.88884	0.99966	1.0264	0.037642
130.0843	0.6	lomics Neg	0.94698	0.99486	1.1034	0.14195	0.80486	0.99506	1.0292	0.041539	0.52567	0.78224	1.3387	0.42088	0.16324	0.80547	1.5626	0.64395	0.015323	0.39567	1.8972	0.92387	0.66139	0.89898	1.1086	0.14872	0.005613	0.2769	1.828	0.87029	0.91464	0.97738	1.0085	0.012259	0.19592	0.74722	0.77027	-0.37656
179.1798	4.73	olomics Pos	0.28058	0.99995	0.87254	-0.1967	0.62735	0.99979	0.97171	-0.04141	0.6266	0.99601	0.954	-0.06794	0.19897	0.99996	0.90528	-0.14356	0.11718	0.86265	0.85061	-0.23343	0.55006	0.99959	0.94485	-0.08184	0.015211	0.17082	1.2025	0.26606	0.72805	0.99984	0.97706	-0.03348	0.56529	0.99966	0.95344	-0.06879
405.7956	5.71	olomics Pos	0.21014	0.99995	1.1451	0.19549	0.046619	0.99441	1.3721	0.45644	0.71865	0.99601	1.0654	0.091457	0.097626	0.99996	1.2885	0.36565	0.33526	0.99701	1.1402	0.18931	0.10794	0.99959	1.2024	0.2659	0.14725	0.3557	1.2376	0.30758	0.17542	0.99984	1.1828	0.24227	0.4941	0.99966	1.1122	0.15341
484.2832	5.66	olomics Pos	0.36508	0.99995	1.0924	0.12745	0.35874	0.99979	1.1019	0.14001	0.40472	0.95675	0.90211	-0.14863	0.87165	0.99996	0.98502	-0.02177	0.23715	0.99701	0.8602	-0.21725	0.90486	0.99959	1.0074	0.010666	0.018745	0.17894	1.3657	0.44967	0.71261	0.99984	1.0311	0.044144	0.10208	0.99966	1.2603	0.3338
307.856	12.74	olomics Ne	0.88094	0.9951	1.0123	0.01767	0.71862	0.99975	1.0156	0.022376	0.47798	0.99731	0.95836	-0.06136	0.79766	0.99915	1.0123	0.017619	0.27142	0.71896	1.0701	0.097691	0.20356	0.63388	1.0798	0.11081	0.00522	0.052004	1.2657	0.33994	0.46776	0.99481	1.0501	0.070492	0.33783	0.92398	0.93314	-0.09984
430.9754	4.92	olomics Ne	0.48805	0.9951	1.047	0.066208	0.24611	0.99975	1.0575	0.080685	0.42706	0.99731	0.96041	-0.05828	0.39984	0.99915	1.0438	0.061871	0.053959	0.58152	1.1243	0.16901	0.057408	0.51062	1.1371	0.18534	0.00996	0.060961	1.274	0.34932	0.33811	0.99481	1.0693	0.096717	0.82688	0.9778	0.9883	-0.01698
314.8852	10.16	olomics Pos	0.37345	0.99995	1.0599	0.083911	0.16368	0.99979	1.1094	0.14973	0.74834	0.99601	1.0175	0.025063	0.51879	0.99996	1.0912	0.12585	0.85444	0.99701	1.0117	0.01676	0.21582	0.99959	1.0899	0.12419	0.047645	0.22128	1.2911	0.36858	0.95474	0.99984	1.0071	0.010149	0.79729	0.99966	1.0185	0.026482
539.3117	1.33	olomics Pos	0.70618	0.99995	0.95052	-0.07321	0.67969	0.99979	0.94537	-0.08105	0.07989	0.62041	0.74276	-0.42903	0.85622	0.99996	0.97899	-0.03064	0.009608	0.46849	0.66498	-0.58862	0.079087	0.99959	0.74868	-0.41759	0.23207	0.45379	0.86418	-0.21059	0.27626	0.99984	0.84534	-0.2424	0.48629	0.99966	0.91663	-0.12558
393.2135	0.6	olomics Pos	0.96547	0.99995	1.03	0.042685	0.066365	0.99979	0.71613	-0.48171	0.27607	0.88589	0.82878	-0.27093	0.91491	0.99996	1.076	0.10569	0.021166	0.53763	0.68424	-0.54742	0.14075	0.99959	0.79324	-0.33417	0.61578	0.77854	1.2578	0.33086	0.41408	0.99984	0.87055	-0.2	0.92585	0.99966	1.0889	0.12289
386.9728	12	olomics Ne	0.57401	0.9951	0.96355	-0.05357	0.14249	0.99975	1.0891	0.12317	0.25278	0.99731	0.91516	-0.1279	0.98478	0.99915	1.0047	0.006805	0.87179	0.96856	0.98859	-0.01656	0.40453	0.76729	1.0966	0.13301	0.04121	0.11376	1.1925	0.254	0.83456	0.99481	1.0191	0.027334	0.035533	0.91738	0.87196	-0.19767
134.0304	0.57	olomics Pos	0.90824	0.99995	1.0084	0.012045	0.49805	0.99979	1.0875	0.12103	0.61381	0.99601	0.95465	-0.06695	0.68313	0.99996	0.96326	-0.05401	0.9922	0.9977	1.0164	0.023536	0.035588	0.99959	1.4284	0.51438	0.003186	0.11681	1.9766	0.98306	0.087755	0.99984	0.77131	-0.37461	0.039969	0.99966	0.82746	-0.27324
336.0078	0.52	olomics Pos	0.91928	0.99995	0.98916	-0.01572	0.41532	0.99979	1.1227	0.16691	0.68763	0.99601	0.95284	-0.06969	0.23933	0.99996	1.231	0.29977	0.247	0.99701	1.1648	0.22008	0.10088	0.99959	1.2555	0.32832	0.002693	0.11265	1.815	0.85999	0.73302	0.99984	0.95998	-0.05892	0.57703	0.99966	1.0486	0.068447
1164.717	10.06	olomics Pos	0.09575	0.99995	1.0998	0.13728	0.1376	0.99979	1.0832	0.11536	0.59197	0.99601	0.97895	-0.03069	0.93976	0.99996	1.0131	0.018805	0.82199	0.99701	0.99044	-0.01387	0.73047	0.99959	1.0379	0.05364	0.034749	0.20384	1.287	0.36402	0.62099	0.99984	1.0491	0.069107	0.11882	0.99966	1.1411	0.1904
812.8376	10.02	olomics Pos	0.97062	0.99995	1.0019	0.002681	0.67617	0.99979	1.0248	0.035398	0.51221	0.99601	0.95121	-0.07216	0.44892	0.99996	0.94941	-0.07489	0.68784	0.99701	0.96696	-0.04848	0.88194	0.99959	1.009	0.01292	0.03715	0.20439	1.289	0.3663	0.96918	0.99984	1.0025	0.00361	0.81928	0.99966	0.98408	-0.02315
765.8317	10.03	olomics Pos	0.040542	0.98463	1.1937	0.25549	0.64267	0.99979	0.97332	-0.03901	0.46391	0.99351	1.0526	0.074006	0.87374	0.99996	1.015	0.021498	0.89356	0.99701	0.99752	-0.00358	0.8032	0.99959	0.98752	-0.01812	0.026983	0.19234	1.2792	0.35523	0.073467	0.99984	1.1495	0.20103	0.90862	0.99966	1.0363	0.051487
753.5566	10.13	olomics Ne	0.65051	0.9951	1.0515	0.072413	0.90768	0.99975	1.028	0.039908	0.3958	0.99731	1.0681	0.095056	0.90522	0.99915	1.0147	0.021049	0.11884	0.6225	1.1273	0.17281	0.14377	0.56991	1.1323	0.17921	0.006642	0.057338	1.2727	0.3479	0.27387	0.99481	1.1565	0.2097	0.26934	0.91738	1.0992	0.1365
1166.746	10.04	olomics Pos	0.059723	0.99995	0.89145	-0.16578	0.38839	0.99979	0.94303	-0.08463	0.003827	0.15284	0.7643	-0.3878	0.27424	0.99996	0.91559	-0.12722	0.026889	0.55437	0.89448	-0.16087	0.30141	0.99959	0.90152	-0.14957	0.029857	0.19804	1.1675	0.22337	0.53741	0.99984	0.94394	-0.08323	0.65081	0.99966	1.0656	0.091683
243.0205	12.14	olomics Pc	0.40727	0.99964	1.041	0.05798	0.53512	1	1.0486	0.068511	0.74841	0.99882	0.98336	-0.02421	0.85122	0.99987	1.0107	0.01534	0.48127	0.95986	1.0246	0.035109	0.66638	0.99796	1.0257	0.036603	0.049194	0.77249	1.1261	0.17128	0.49679	0.9981	1.0332	0.047124	0.28262	0.99995	1.0487	0.068611
566.9734	12	olomics Ne	0.75155	0.9951	1.0178	0.025478	0.5155	0.99975	1.1318	0.17868	0.969	0.99731	1.0029	0.00418	0.23532	0.99915	1.1539	0.20658	0.37329	0.75206	1.0884	0.12219	0.41059	0.77284	1.0744	0.10359	0.041579	0.11377	1.3084	0.38782	0.45939	0.99481	1.0657	0.091817	0.7415	0.97489	1.0275	0.039074
500.9795	5.05	olomics Ne	0.75576	0.9951	1.0165	0.023566	0.12276	0.99975	1.073	0.10169	0.19062	0.99731	0.94969	-0.07446	0.31619	0.99915	1.0661	0.092337	0.11791	0.62181	1.0499	0.070214	0.029427	0.4612	1.1279	0.17369	0.011896	0.062031	1.219	0.28564	0.45551	0.99481	1.0344	0.048858	0.13797	0.91738	0.95109	-0.07235
889.5385	5.57	olomics Pos	0.77513	0.99995	1.0805	0.11164	0.10897	0.99979	1.2192	0.28592	0.82127	0.99702	1.0178	0.025402	0.3831	0.99996	1.1564	0.20958	0.72265	0.99701	0.95566	-0.06543	0.051904	0.99959	1.3694	0.45356	0.024628	0.18846	1.3236	0.40443	0.31134	0.99984	1.1519	0.20404	0.032819	0.99966	1.3474	0.43021
86.0969	0.59	olomics Pos	0.91191	0.99995	1.049	0.068963	0.7584	0.99979	0.99109	-0.01291	0.97431	0.99702	1.0798	0.11071	0.073723	0.99996	1.4128	0.49857	0.006503	0.46849	1.6981	0.76393	0.36684	0.99959	1.1156	0.15781	0.000932	0.089845	2.1933	1.1331	0.19366	0.99984	0.85245	-0.23031	0.023145	0.99966	0.75963	-0.39663
502.2869	10.15	olomics Ne	0.74907	0.9951	0.96224	-0.05554	0.64342	0.99975	1.0443	0.062474	0.29745	0.99731	0.87988	-0.18462	0.60477	0.99915	0.94021	-0.08894	0.97067	0.99403	0.99102	-0.01302	0.070528	0.5223	1.2029	0.26656	0.004401	0.050628	1.4845	0.56994	0.80587	0.99481	1.0547	0.076893	0.68859	0.97023	1.0381	0.053984
614.8205	0.54	olomics Pos	0.85442	0.99995	1.0118	0.016969	0.61983	0.99979	1.0422	0.059619	0.28401	0.89276	0.93188	-0.10179	0.52886	0.99996	0.95767	-0.0624	0.032455	0.59375	0.92473	-0.11289	0.66587	0.99959	0.98534	-0.0213	0.15423	0.36252	1.1	0.13745	0.57402	0.99984	1.0555	0.077991	0.39417	0.99966	1.0509	0.071626
604.7936	0.56	olomics Pos	0.92567	0.99995	0.99503	-0.00718	0.2719	0.99979	0.94087	-0.08794	0.043414	0.45105	0.86536	-0.20862	0.11177	0.99996	0.86955	-0.20167	0.029965	0.58295	0.80428	-0.31422	0.1397	0.99959	0.90164	-0.14938	0.25642	0.47396	0.88696	-0.17306	0.79391	0.99984	0.98267	-0.02522	0.8776	0.99966	0.99728	-0.00394
959.7761	10.05	olomics Pos	0.85937	0.99995	1.029	0.041222	0.024534	0.99441	0.86014	-0.21736	0.18234	0.80658	0.88704	-0.17293	0.90856	0.99996	1.01	0.014366	0.53539	0.99701	0.95152	-0.07169	0.36824	0.99959	0.91556	-0.12727	0.65733	0.80282	1.0981	0.13498	0.84208	0.99984	0.99405	-0.00861	0.36648	0.99966	1.0938	0.12939
522.5942	7.47	olomics Pc	0.051426	0.99964	1.9573	0.96888	0.54931	1	1.5245	0.60832	0.27332	0.99882	1.8957	0.92271	0.07613	0.99987	1.9676	0.97643	0.048535	0.95986	1.9793	0.98502	0.16387	0.99796	4.3363	2.1165	0.75074	0.99768	1.2005	0.26359	0.019828	0.9981	2.4642	1.3011	0.13796	0.99995	1.8472	0.88532
235.9218	10.16	olomics Pos	0.10364	0.99995	1.1494	0.20087	0.29747	0.99979	1.0703	0.098071	0.56942	0.99601	1.0787	0.10926	0.9456	0.99996	1.0012	0.001753	0.11902	0.86273	1.1666	0.22229	0.4382	0.99959	1.0676	0.094424	0.010631	0.16149	1.4788	0.56445	0.55929	0.99984	1.1701	0.22659	0.12385	0.99966	1.1879	0.24839
550.2981	1.11	olomics Pos	0.3768	0.99995	0.91711	-0.12484	0.77081	0.99979	0.97116	-0.04222	0.36131	0.93332	0.88345	-0.17879	0.5489	0.99996	0.92497	-0.11253	0.87302	0.99701	#N/A	#N/A	0.067302	0.99959	1.32	0.40053	0.001072	0.089845	1.5466	0.62908	0.20667	0.99984	1.1126	0.15388	0.61636	0.99966	1.0489	0.068815
149.1331	4.73	olomics Pos	0.36841	0.99995	0.93085	-0.10338	0.3575	0.99979	0.94649	-0.07935	0.62043	0.99601	0.96685	-0.04863	0.11632	0.99996	0.90415	-0.14536	0.59047	0.99701	0.96998	-0.04398	0.95631	0.99959	#N/A	#N/A	0.009526	0.15489	1.2053	0.26934	0.33281	0.99984	0.93004	-0.10463	0.87397	0.99966	0.9931	-0.00998
832.9737	12.01	olomics Ne	0.62336	0.9951	1.091	0.12567	0.75059	0.99975	1.032	0.045376	0.70183	0.99731	1.05	0.070367	0.24516	0.99915	1.0694	0.096862	0.15627	0.64314	1.1063	0.1458	0.38315	0.76125	1.1785	0.23696	0.004823	0.051563	1.3293	0.41071	0.54353	0.99481	1.1442	0.19436	0.41236	0.94239	0.95085	-0.07271
135.9274	10.24	olomics Pos	0.24702	0.99995	1.144	0.19404	0.59499	0.99979	0.94703	-0.07852	0.9248	0.99702	1.0148	0.021235	0.89991	0.99996	0.99931	-0.00099	0.43114	0.99701	0.90875	-0.13804	0.55323	0.99959	0.92994	-0.10478	0.035024	0.20439	1.2914	0.3689	0.48391	0.99984	0.92325	-0.11521	0.92643	0.99966	1.0061	0.008746
1091.512	12.05	olomics Ne	0.13006	0.9951	1.1504	0.20208	0.4133	0.99975	1.1477	0.1987	0.31494	0.99731	1.0918	0.12671	0.042954	0.99915	1.2677	0.3422	0.12653	0.6311	1.1803	0.23909	0.017973	0.45618	1.3416	0.42394	0.041668	0.11377	1.3251	0.40609	0.022272	0.99481	1.2886	0.36579	0.74512	0.97489	0.96288	-0.05457
310.9067	10.14	olomics Pos	0.40135	0.99995	1.0912	0.1259	0.24661	0.99979	1.0946	0.13045	0.5981	0.99601	0.95812	-0.06173	0.17296	0.99996	1.1403	0.18938	0.56488	0.99701	1.0425	0.060048	0.49251	0.99959	1.081	0.11232	0.017074	0.17568	1.3759	0.46042	0.75334	0.99984	1.0452	0.063721	0.38159	0.99966	1.1411	0.19041
695.4281	3.16	olomics Pc	0.74874	0.99964	0.99395	-0.00876	0.37703	1	0.8772	-0.18902	0.13565	0.99882	0.72991	-0.45421	0.62761	0.99987	0.97473	-0.03693	0.048255	0.95986	0.65277	-0.61536	0.39168	0.99796	0.8672	-0.20556	0.60406	0.99768	0.94741	-0.07795	0.25012	0.9981	0.81279	-0.29905	0.56646	0.99995	0.93357	-0.09917
651.2843	1.04	olomics Pos	0.35949	0.99995	1.1713	0.22809	0.52234	0.99979	1.123	0.16736	0.68669	0.99601	1.0464	0.065457	0.28453	0.99996	1.3523	0.43544	0.78718	0.99701	1.0699	0.097435	0.06411	0.99959	1.5857	0.66507	0.022138	0.18641	1.6311	0.70583	0.46137	0.99984	1.1837	0.24327	0.27757	0.99966	1.2288	0.29731
891.7935	10.06	olomics Pos	0.46066	0.99995	0.95569	-0.06539	0.60694	0.99979	0.95424	-0.06758	0.3034	0.89817	0.91251	-0.13209	0.027201	0.99996	0.89449	-0.16086	0.28375	0.99701	0.90239	-0.14817	0.39598	0.99959	0.9297	-0.10516	0.52379	0.71968	1.0857	0.11861	0.48241	0.99984	0.94676	-0.07892	0.73424	0.99966	0.97971	-0.02957
778.9723	12.02	olomics Ne	0.2458	0.9951	1.2031	0.26678	0.087887	0.99975	1.331	0.41252	0.25948	0.99731	1.1177	0.16054	0.60986	0.99915	1.1265	0.17186	0.026041	0.50727	1.4677	0.5536	0.072002	0.5223	1.2707	0.34557	0.042977	0.11566	1.4309	0.51693	0.30307	0.99481	1.1567	0.21	0.78492	0.97489	1.0762	0.10596
419.982	4.64	olomics Ne	0.50972	0.9951	1.0384	0.054368	0.76175	0.99975	1.0169	0.024143	0.96689	0.99731	0.99952	-0.00069	0.17203	0.99915	1.0958	0.13195	0.13726	0.64226	1.1462	0.19685	0.062983	0.51317	1.1576	0.21111	0.00708	0.057338	1.3368	0.41877	0.19134	0.99481	1.0868	0.12005	0.62072	0.96789	0.96352	-0.05362
596.759	9.92	olomics Pos	0.93984	0.99995	1.0006	0.000887	0.112	0.99979	1.1636	0.21857	0.98538	0.99727	0.9978	-0.00318	0.068028	0.99996	1.2147	0.28062	0.98666	0.99701	1.0003	0.00037	0.34944	0.99959	1.1006	0.13826	0.021378	0.18318	1.3387	0.42087	0.58815	0.99984	1.0632	0.088411	0.16008	0.99966	1.1797	0.23847
1106.762	10.02	olomics Pos	0.65444	0.99995	0.96346	-0.0537	0.46325	0.99979	0.94443	-0.08248	0.021288	0.31902	0.87667	-0.1899	0.92064	0.99996	1.0018	0.002607	0.12062	0.86273	0.90975	-0.13645	0.34337	0.99959	0.93499	-0.09698	0.002075	0.10412	1.279	0.35497	0.18728	0.99984	0.92705	-0.10929	0.83643	0.99966	1.0284	0.040392
402.8262	9.92	olomics Pos	0.18786	0.99995	1.1478	0.19883	0.054767	0.99441	1.1467	0.19751	0.15417	0.77443	1.0896	0.1238	0.3256	0.99996	1.1107	0.15147	0.63947	0.99701	1.0695	0.096957	0.50379	0.99959	1.0843	0.11673	0.028869	0.19804	1.3714	0.45569	0.1479	0.99984	1.1246	0.16945	0.29759	0.99966	1.0971	0.13367
781.3895	0.56	olomics Pos	0.541	0.99995	0.91999	-0.12032	0.19176	0.99979	0.80166	-0.31893	0.035068	0.40571	0.63737	-0.6498	0.98176	0.99996	1.1384	0.18699	0.12745	0.86468	0.72024	-0.47346	0.50601	0.99959	0.88409	-0.17773	0.99693	0.99824	1.1346	0.18213	0.43568	0.99984	0.83877	-0.25365	0.76389	0.99966	1.2065	0.27088
254.986	3.79	olomics Ne	0.652	0.9951	1.0178	0.025467	0.64909	0.99975	0.97814	-0.03188	0.18682	0.99731	0.95191	-0.07111	0.95511	0.99915	1.0034	0.004919	0.15118	0.64314	1.0767	0.10667	0.11667	0.54044	1.072	0.10027	0.028618	0.091981	1.1677	0.22362	0.054538	0.99481	1.1021	0.14026	0.20667	0.91738	0.93605	-0.09534
578.257	5.12	olomics Pc	0.32472	0.99964	0.8703	-0.20041	0.794	1	1.0428	0.060409	0.41686	0.99882	0.906	-0.14241	0.73958	0.99987	1.13	0.17636	0.15119	0.95986	1.1511	0.20303	0.035536	0.99796	1.2822	0.35866	0.006684	0.36303	1.464	0.54994	0.73377	0.9981	1.1595	0.21351	0.20989	0.99995	1.156	0.20911
331.2642	6.54	olomics Pc	0.81451	0.99964	0.96377	-0.05325	0.65863	1	0.91237	-0.1323	0.76231	0.99882	0.94155	-0.08689	0.96825	0.99987	1.1193	0.16254	0.96546	0.99906	1.1232	0.16764	0.026982	0.99796	0.54098	-0.88634	0.07732	0.84277	0.63685	-0.65097	0.79746	0.9981	0.96807	-0.04681	0.38147	0.99995	0.813	-0.29867
566.8894	10.02	olomics Pos	0.18706	0.99995	1.0784	0.10886	0.50916	0.99979	1.0339	0.048129	0.10419	0.68915	0.96302	-0.05436	0.92752	0.99996	1.01	0.014365	0.77171	0.99701	0.9869	-0.01903	0.99292	0.99959	1.0058	0.008384	0.018901	0.17894	1.2873	0.36432	0.72505	0.99984	1.0391	0.055366	0.60343	0.99966	1.0449	0.063432
1150.529	5.77	olomics Pos	0.90961	0.99995	1.0296	0.042081	0.26316	0.99979	0.8504	-0.23379	0.73177	0.99601	0.96728	-0.04799	0.63148	0.99996	0.94685	-0.07879	0.59777	0.99701	0.94483	-0.08187	0.852	0.99959	1.0628	0.087886	0.01391	0.16838	1.3618	0.44548	0.8001	0.99984	0.99329	-0.00972	0.90912	0.99966	0.98739	-0.01831
923.8046	10.05	olomics Pos	0.34431	0.99995	1.1312	0.1778	0.66772	0.99979	0.96319	-0.0541	0.16903	0.7924	0.87514	-0.19242	0.28172	0.99996	0.93205	-0.10152	0.52978	0.99701	0.94325	-0.0843	0.85395	0.99959	0.99176	-0.01194	0.000887	0.089845	1.4027	0.48818	0.87622	0.99984	1.0325	0.046133	0.9138	0.99966	1.0107	0.015317
474.9774	5.07	olomics Ne	0.46801	0.9951	1.0387	0.054742	0.79588	0.99975	1.0062	0.008975	0.58	0.99731	0.98005	-0.02907	0.70884	0.99915	1.0149	0.021308	0.032568	0.52432	1.0991	0.13636	0.031255	0.4612	1.0875	0.12105	0.00127	0.041313	1.2404	0.3108	0.50011	0.99481	1.0369	0.052333	0.44769	0.94605	0.97761	-0.03267
219.2117	6.01	olomics Pc	0.034972	0.99964	0.77313	-0.37122	0.046593	0.88953	0.70536	-0.50357	0.29121	0.99882	0.88072	-0.18325	0.78011	0.99987	1.0377	0.053353	0.78236	0.99139	0.96692	-0.04854	0.65014	0.99796	0.94595	-0.08016	0.66174	0.99768	1.0739	0.10285	0.62783	0.9981	0.93883	-0.09107	0.57248	0.99995	0.92785	-0.10804
217.1957	1.67	olomics Pos	0.030333	0.90678	0.84439	-0.24402	0.035038	0.99441	0.69992	-0.51474	0.000768	0.13107	0.71914	-0.47566	0.33268	0.99996	0.87116	-0.19899	0.003814	0.46849	0.76039	-0.39518	0.11833	0.99959	0.76621	-0.38419	0.30334	0.52527	1.1188	0.16196	0.11383	0.99984	0.78781	-0.34408	0.035783	0.99966	0.83479	-0.26051
819.7932	0.54	olomics Pos	0.12207	0.99995	0.88203	-0.1811	0.16082	0.99979	0.89122	-0.16614	0.11756	0.71506	0.85765	-0.22153	0.69707	0.99996	0.97148	-0.04175	0.022268	0.5478	0.77102	-0.37516	0.36194	0.99959	0.91426	-0.12933	0.54362	0.73692	1.0706	0.098355	0.052185	0.99984	0.84317	-0.24611	0.20552	0.99966	0.84953	-0.23525
852.5711	8.67	olomics Ne	0.55612	0.9951	1.0402	0.056897	0.67514	0.99975	1.0203	0.028978	0.28813	0.99731	0.95296	-0.06951	0.52725	0.99915	1.0366	0.05183	0.083136	0.5958	1.0836	0.11579	0.49996	0.79937	1.0662	0.092492	0.013764	0.065382	1.16	0.21415	0.55954	0.99481	0.97554	-0.03573	0.15705	0.91738	0.92256	-0.11628
1162.244	9	olomics Pc	0.47584	0.99964	0.89375	-0.16206	0.61009	1	0.94022	-0.08893	0.26533	0.99882	0.8291	-0.27038	0.84116	0.99987	1.0484	0.068235	0.01414	0.95986	0.6735	-0.57026	0.34525	0.99796	0.86622	-0.2072	0.76394	0.99768	1.2694	0.34418	0.74078	0.9981	0.96134	-0.05688	0.91972	0.99995	1.1382	0.18673
809.5863	5.42	olomics Pos	0.56001	0.99995	1.0493	0.069486	0.95569	0.99979	0.99524	-0.00689	0.44187	0.98414	0.95565	-0.06545	0.74172	0.99996	0.97752	-0.0328	0.58109	0.99701	0.95598	-0.06495	0.94007	0.99959	1.0067	0.009601	0.031045	0.19986	1.2699	0.34468	0.94719	0.99984	1.0061	0.008798	0.45965	0.99966	1.0929	0.12815
643.1869	1.29	olomics Pos	0.20516	0.99995	1.1451	0.19542	0.47526	0.99979	1.0714	0.099438	0.43913	0.98225	1.0388	0.054909	0.75921	0.99996	1.021	0.029946	0.24122	0.99701	0.9147	-0.12863	0.95282	0.99959	1.0111	0.015993	0.028105	0.19663	1.241	0.31151	0.26211	0.99984	1.0937	0.12923	0.20567	0.99966	1.1534	0.2059
899.8021	10.05	olomics Pos	0.029382	0.89627	1.2563	0.32914	0.11074	0.99979	1.1528	0.20514	0.55715	0.99601	0.95677	-0.06375	0.091782	0.99996	1.2353	0.30482	0.20044	0.97344	1.1625	0.21727	0.040602	0.99959	1.304	0.38291	0.004383	0.12565	1.4325	0.51854	0.54495	0.99984	1.0778	0.1081	0.008546	0.99749	1.2744	0.34982
226.9523	10.03	olomics Pos	0.52387	0.99995	1.0302	0.042907	0.76021	0.99979	1.019	0.02709	0.013004	0.24793	0.94941	-0.07489	0.96641	0.99996	1.003	0.004254	0.3213	0.99701	0.96024	-0.05853	0.8489	0.99959	0.993	-0.01014	0.014988	0.17068	1.2425	0.31319	0.86382	0.99984	0.99438	-0.00813	0.59737	0.99966	1.0402	0.05687
945.5869	10.35	olomics Ne	0.48945	0.9951	1.0508	0.07146	0.44744	0.99975	1.0586	0.082213	0.59943	0.99731	0.9595	-0.05965	0.23837	0.99915	1.0911	0.12583	0.3247	0.74138	1.1076	0.14747	0.009777	0.45618	1.2843	0.36095	0.060029	0.14129	1.2071	0.27151	0.53849	0.99481	1.0541	0.076049	0.27234	0.91738	0.91344	-0.13062
206.9722	10.15	olomics Pos	0.29868	0.99995	1.1226	0.16678	0.65837	0.99979	1.0682	0.095245	0.48622	0.99601	0.95067	-0.07299	0.20858	0.99996	1.106	0.14532	0.48992	0.99701	1.0637	0.089085	0.28089	0.99959	1.0511	0.071903	0.000443	0.088851	1.2962	0.37424	0.43164	0.99984	1.0698	0.097355	0.23333	0.99966	1.0763	0.10609
252.9776	12.01	olomics Ne	0.3078	0.9951	1.0947	0.13055	0.019796	0.99975	1.2072	0.27166	0.64781	0.99731	0.97736	-0.03304	0.27538	0.99915	1.0649	0.090726	0.028675	0.51873	1.2039	0.26768	0.50169	0.79937	1.054	0.075817	0.034772	0.10167	1.2606	0.33415	0.041951	0.99481	1.0902	0.12453	0.77153	0.97489	1.0402	0.056863
81.5219	12.37	olomics Pc	0.19218	0.99964	1.1608	0.2151	0.005357	0.88953	1.7644	0.81921	0.30392	0.99882	0.86314	-0.21233	0.33437	0.99987	1.1171	0.15981	0.50999	0.95986	1.1593	0.21328	0.16442	0.99796	1.2858	0.36263	0.34589	0.99768	1.2951	0.37308	0.3264	0.9981	1.3272	0.40837	0.044013	0.99995	1.2843	0.36101
685.257	5.24	olomics Ne	0.86607	0.9951	1.0211	0.03008	0.92585	0.99975	0.99408	-0.00856	0.187	0.99731	0.8612	-0.21559	0.79051	0.99915	0.97737	-0.03303	0.68768	0.9128	1.0564	0.079129	0.18062	0.61082	1.1767	0.23471	0.02538	0.08627	1.2565	0.32943	0.49548	0.99481	1.0483	0.068048	0.24971	0.91738	1.0847	0.11732
776.5587	8.85	olomics Pc	0.45323	0.99964	1.0381	0.053928	0.46553	1	1.0758	0.10538	0.11405	0.99882	1.092	0.12691	0.41056	0.99987	1.0631	0.0883	0.26704	0.95986	1.1246	0.16942	0.002182	0.75294	1.1535	0.20603	0.18093	0.99768	1.1632	0.21811	0.2193	0.9981	1.1475	0.19852	0.006268	0.99388	1.295	0.37298
262.226	1.39	olomics Pos	0.17757	0.99995	0.84342	-0.24568	0.007457	0.99441	0.78053	-0.35748	0.045481	0.46493	0.77389	-0.3698	0.046934	0.99996	0.77724	-0.36357	0.082796	0.80455	0.90459	-0.14466	0.27574	0.99959	0.79519	-0.33064	0.59873	0.76907	1.1175	0.16029	0.52956	0.99984	0.92147	-0.118	0.89178	0.99966	1.001	0.001482
1134.748	10.03	olomics Pos	0.62263	0.99995	1.0663	0.092652	0.78645	0.99979	1.0274	0.03899	0.84315	0.99702	0.98715	-0.01866	0.67883	0.99996	1.094	0.12958	0.87279	0.99701	0.98976	-0.01485	0.090233	0.99959	1.2467	0.31807	0.001831	0.099759	1.3806	0.46529	0.87603	0.99984	1.0554	0.077808	0.22088	0.99966	1.0749	0.10423
894.6274	6.06	olomics Pos	0.43687	0.99995	1.0996	0.13699	0.79868	0.99979	0.97743	-0.03293	0.72579	0.99601	1.0298	0.042304	0.40566	0.99996	0.88981	-0.16843	0.66666	0.99701	1.0978	0.13466	0.68839	0.99959	0.95822	-0.06157	0.02412	0.18774	1.3776	0.4622	0.54947	0.99984	0.93189	-0.10177	0.98332	0.99966	1.0107	0.01539
558.9786	5.13	olomics Ne	0.63325	0.9951	1.0246	0.035012	0.27392	0.99975	1.0506	0.07117	0.45069	0.99731	0.97803	-0.03205	0.38499	0.99915	1.0545	0.076615	0.001883	0.24494	1.1286	0.17449	0.12785	0.54233	1.0943	0.12999	0.00083	0.041313	1.2101	0.27508	0.34144	0.99481	1.0314	0.044538	0.23574	0.91738	0.95807	-0.0618
316.9457	0.28	olomics Ne	0.094498	0.9951	1.1213	0.16511	0.87289	0.99975	0.99531	-0.00678	0.79317	0.99731	1.0194	0.02776	0.64655	0.99915	0.98158	-0.02682	0.039975	0.53488	1.1362	0.18427	0.18851	0.62277	1.0912	0.12591	0.014874	0.066581	1.2402	0.31054	0.8025	0.99481	1.0146	0.020923	0.055921	0.91738	0.93064	-0.10371
554.9723	12.01	olomics Ne	0.008416	0.9951	1.3929	0.47806	0.1369	0.99975	1.1671	0.22292	0.28077	0.99731	1.1051	0.14414	0.018213	0.99915	1.1921	0.2535	0.024472	0.50209	1.3742	0.45858	0.007945	0.45618	1.2882	0.36534	7.9E-05	0.02431	1.5177	0.60186	0.12278	0.99481	1.2997	0.37815	0.44496	0.94601	1.167	0.22279
216.9252	0.56	olomics Pos	0.87267	0.99995	1.0038	0.005512	0.20495	0.99979	0.96098	-0.05742	0.018588	0.30022	0.9256	-0.11154	0.54883	0.99996	0.95975	-0.05927	0.023018	0.5516	0.92	-0.12029	0.06795	0.99959	0.9208	-0.11904	0.75139	0.85549	1.0345	0.048982	0.56364	0.99984	1.0426	0.060184	0.65776	0.99966	1.0351	0.04982
160.97	10.03	olomics Pos	0.22087	0.99995	1.2182	0.28481	0.085165	0.99979	1.2286	0.29704	0.35234	0.93176	1.0832	0.11532	0.13911	0.99996	1.2103	0.27533	0.42896	0.99701	1.0972	0.13376	0.012859	0.99959	1.2077	0.27222	0.012603	0.16313	1.4821	0.56761	0.21849	0.99984	1.2131	0.27875	0.20912	0.99966	1.2547	0.32739
676.4011	2.7	olomics Pc	0.77917	0.99964	1.1843	0.24403	0.55884	1	0.909	-0.13764	0.019873	0.64537	0.6743	-0.56853	0.99898	0.99987	1.3483	0.43115	0.31586	0.95986	0.81248	-0.29959	0.80459	0.99796	0.9626	-0.05499	0.96132	0.99768	1.2348	0.30432	0.28579	0.9981	0.795	-0.33097	0.75106	0.99995	1.217	0.28331
224.2019	5.99	olomics Pc	0.097264	0.99964	0.86782	-0.20453	0.043352	0.88953	0.74762	-0.41963	0.037697	0.90186	0.80563	-0.31181	0.34946	0.99987	0.91475	-0.12855	0.077996	0.95986	0.89245	-0.16415	0.13431	0.99796	0.8026	-0.31724	0.64535	0.99768	1.0238	0.033887	0.072436	0.9981	0.76525	-0.386	0.042567	0.99995	0.83911	-0.25306
254.9376	10.11	olomics Pos	0.10433	0.99995	1.186	0.24608	0.93347	0.99979	0.99098	-0.01307	0.43823	0.98176	1.0983	0.13526	0.65714	0.99996	1.0699	0.097411	0.55657	0.99701	1.1092	0.14951	0.84046	0.99959	0.98074	-0.02806	0.008429	0.14619	1.3785	0.46309	0.52952	0.99984	1.2452	0.31637	0.40066	0.99966	1.1857	0.24575
500.9077	11.96	olomics Pc	0.62526	0.99964	1.0333	0.047205	0.013217	0.88953	1.1395	0.18844	0.66063	0.99882	1.0294	0.041857	0.71005	0.99987	1.0322	0.045753	0.25067	0.95986	1.0913	0.12604	0.008348	0.99128	1.146	0.19658	0.007572	0.36601	1.1892	0.25	0.34639	0.9981	1.0563	0.079041	0.37434	0.99995	1.0396	0.056082
1195.743	10.03	olomics Pos	0.53384	0.99995	1.0862	0.11935	0.45601	0.99979	0.90541	-0.14336	0.96966	0.99702	0.98243	-0.02558	0.69514	0.99996	0.94351	-0.08389	0.91238	0.99701	1.0127	0.018214	0.60954	0.99959	0.92999	-0.10471	0.03136	0.20029	1.3268	0.408	0.69213	0.99984	1.043	0.060777	0.86717	0.99966	1.0218	0.031115
135.1176	2.77	olomics Pos	0.82212	0.99995	1.0539	0.075682	0.13999	0.99979	0.75542	-0.40465	0.18942	0.8198	0.84706	-0.23946	0.44276	0.99996	0.88057	-0.18349	0.23753	0.99701	0.84684	-0.23984	0.037165	0.99959	0.60914	-0.71516	0.57469	0.75461	1.0872	0.12061	0.2636	0.99984	0.84229	-0.24761	0.21105	0.99966	0.84309	-0.24624
424.9786	4.78	olomics Ne	0.26496	0.9951	1.0578	0.08112	0.70385	0.99975	1.021	0.030028	0.90506	0.99731	1.0085	0.01227	0.22201	0.99915	1.0615	0.086048	0.024276	0.50209	1.1377	0.18608	0.029941	0.45618	1.1451	0.19548	0.002469	0.047017	1.2538	0.32625	0.27345	0.99481	1.0579	0.081185	0.93837	0.99833	0.99761	-0.00345

211.1899	1.66	lomics Pos	0.24592	0.99995	0.89227	-0.16445	0.069877	0.99979	0.74385	-0.42691	0.014939	0.26996	0.77503	-0.36767	0.25147	0.99996	0.83904	-0.25319	0.043606	0.66031	0.81939	-0.28737	0.26898	0.99959	0.8493	-0.23565	0.26978	0.48868	1.1711	0.22787	0.32272	0.99984	0.87731	-0.18885	0.41239	0.99966	0.90289	-0.14737
354.9801	11.99	olomics Ne	0.14157	0.9951	1.1952	0.25723	0.20347	0.99975	1.1658	0.22128	0.11448	0.99731	1.1974	0.25985	0.18045	0.99915	1.3339	0.41561	0.074211	0.5958	1.4733	0.55906	0.29037	0.67814	1.1304	0.17683	0.016255	0.068763	1.4118	0.49754	0.17391	0.99481	1.1929	0.25449	0.25751	0.91738	1.153	0.20538
456.3087	2.06	lomics Pos	0.061421	0.99995	0.82515	-0.27726	0.089427	0.99979	0.7607	-0.39461	0.043628	0.45176	0.76252	-0.39116	0.3902	0.99996	0.90961	-0.13668	0.20046	0.97344	0.88759	-0.17203	0.1483	0.99959	0.81952	-0.28714	0.26991	0.48869	1.1771	0.2352	0.18098	0.99984	0.83262	-0.26427	0.086129	0.99966	0.82897	-0.27061
1058.769	10.05	lomics Pos	0.25918	0.99995	1.0659	0.092064	0.35064	0.99979	1.0597	0.083636	0.64431	0.99601	1.0266	0.037913	0.48299	0.99996	1.0703	0.097952	0.5817	0.99701	1.0453	0.063883	0.8386	0.99959	1.0154	0.021986	0.008802	0.15003	1.2866	0.36357	0.39041	0.99984	1.052	0.073113	0.1638	0.99966	1.1279	0.17364
336.9751	4.27	olomics Ne	0.20129	0.9951	1.0491	0.069122	0.8136	0.99975	1.0162	0.023114	0.69952	0.99731	0.98257	-0.02537	0.23955	0.99915	1.0617	0.086366	0.14656	0.64314	1.0647	0.090506	0.11927	0.54162	1.0866	0.11987	0.003571	0.050041	1.2455	0.31675	0.19988	0.99481	1.0614	0.086002	0.32691	0.91738	0.94666	-0.07908
257.9485	10.18	lomics Pos	0.30051	0.99995	1.0497	0.069908	0.33281	0.99979	0.94718	-0.07829	0.71483	0.99601	0.98406	-0.02318	0.89167	0.99996	0.99405	-0.00861	0.95546	0.99701	0.99695	-0.0044	0.40704	0.99959	1.0774	0.1076	0.002037	0.10412	1.3465	0.42926	0.27601	0.99984	1.0644	0.090107	0.21271	0.99966	1.0975	0.13417
841.8162	0.53	lomics Pos	0.48289	0.99995	0.94918	-0.07525	0.8344	0.99979	0.99119	-0.01277	0.032932	0.38971	0.88002	-0.1844	0.79158	0.99996	0.98738	-0.01832	0.031646	0.59375	0.88964	-0.16871	0.64114	0.99959	0.97671	-0.03399	0.043501	0.21586	1.2126	0.27809	0.27555	0.99984	0.93058	-0.1038	0.3058	0.99966	0.90963	-0.13665
896.7973	10.03	lomics Pos	0.81473	0.99995	1.0184	0.02637	0.62657	0.99979	1.0293	0.041664	0.55349	0.99601	0.98259	-0.02534	0.81866	0.99996	1.0285	0.040577	0.90879	0.99701	1.0094	0.01353	0.81935	0.99959	1.0168	0.024089	0.01006	0.15803	1.2626	0.33637	0.63736	0.99984	1.0363	0.051505	0.5304	0.99966	1.0637	0.089071
779.5772	5.62	lomics Pos	0.49322	0.99995	1.0534	0.075006	0.13832	0.99979	1.1213	0.16519	0.73242	0.99601	0.97275	-0.03987	0.39485	0.99996	1.0693	0.0966	0.84484	0.99701	1.0106	0.015175	0.048531	0.99959	1.1558	0.20887	0.03913	0.20439	1.3427	0.42518	0.31872	0.99984	1.0883	0.12213	0.012931	0.99749	1.3098	0.38935
363.9877	4.3	olomics Ne	0.35526	0.9951	1.0423	0.059758	0.44593	0.99975	1.0684	0.095476	0.77692	0.99731	1.0315	0.044738	0.77745	0.99915	0.98689	-0.01904	0.1203	0.62484	1.0956	0.13178	0.018687	0.45618	1.1302	0.17653	0.09071	0.17838	1.2376	0.3076	0.14294	0.99481	1.127	0.17254	0.24883	0.91738	0.93175	-0.10199
154.1242	5.99	olomics Pc	0.064628	0.99964	0.85376	-0.2281	0.056118	0.92065	0.7253	-0.46335	0.038742	0.90186	0.83824	-0.25456	0.81834	0.99987	0.98473	-0.02221	0.67379	0.97186	0.96666	-0.04892	0.19581	0.99796	0.80586	-0.3114	0.2862	0.99768	1.0831	0.11519	0.2372	0.9981	0.85906	-0.21917	0.16539	0.99995	0.88896	-0.16981
366.2325	6.39	olomics Pc	0.94469	0.99964	1.006	0.008605	0.018426	0.88953	0.83561	-0.2591	0.17947	0.99882	0.94334	-0.08416	0.56219	0.99987	0.97602	-0.03502	0.8821	0.99904	0.99445	-0.00802	0.10511	0.99796	0.88921	-0.16941	0.02687	0.60458	1.101	0.13886	0.27248	0.9981	0.91019	-0.13575	0.2892	0.99995	0.95635	-0.06439
825.9763	12.05	olomics Ne	0.034767	0.9951	1.2444	0.31545	0.91184	0.99975	1.133	0.18019	0.18768	0.99731	1.2442	0.31526	0.97653	0.99915	1.0032	0.004551	0.38255	0.75206	1.1921	0.25354	0.51518	0.8008	1.1738	0.23124	0.02025	0.075538	1.4495	0.53553	0.025934	0.99481	1.3567	0.44009	0.3603	0.92651	1.1973	0.2598
860.8595	11.96	olomics Ne	0.69203	0.9951	1.0272	0.038697	0.63011	0.99975	1.0275	0.039085	0.38839	0.99731	1.0339	0.048136	0.45723	0.99915	1.0432	0.060951	0.20461	0.69944	1.064	0.089438	0.2099	0.6375	1.0877	0.12133	0.018963	0.07326	1.2166	0.28283	0.60996	0.99481	1.0262	0.037259	0.79259	0.97489	0.98919	-0.01568
101.9405	12.71	olomics Ne	0.92214	0.9951	1.0059	0.008463	0.51287	0.99975	1.0286	0.040633	0.48967	0.99731	0.969	-0.04543	0.31941	0.99915	1.0473	0.06674	0.13471	0.63916	1.0667	0.093113	0.11014	0.53696	1.1064	0.14588	0.005484	0.052776	1.2604	0.33383	0.70676	0.99481	1.0237	0.033733	0.16112	0.91738	0.93314	-0.09983
705.9793	12.03	olomics Ne	0.15927	0.9951	1.2138	0.27953	0.044897	0.99975	1.464	0.54988	0.12424	0.99731	1.2241	0.29172	0.019221	0.99915	1.6368	0.71087	0.20249	0.69888	1.3226	0.40336	0.034067	0.4612	1.6073	0.6846	0.075004	0.16029	1.3223	0.40304	0.13192	0.99481	1.2352	0.30477	0.12809	0.91738	1.2549	0.3276
278.8529	12.73	olomics Ne	0.52555	0.9951	1.0301	0.042743	0.33942	0.99975	1.0432	0.06098	0.92691	0.99731	0.99607	-0.00568	0.039545	0.99915	1.1147	0.15663	0.40805	0.76847	1.0652	0.09118	0.38875	0.76125	1.04	0.056628	0.008764	0.059819	1.2383	0.30834	0.24889	0.99481	1.0445	0.062838	0.43232	0.94458	0.97045	-0.04328
331.983	4.21	olomics Ne	0.89408	0.9951	1.0051	0.007351	0.3836	0.99975	1.0627	0.087751	0.19873	0.99731	0.94144	-0.08706	0.53976	0.99915	1.0355	0.050286	0.23751	0.7131	1.0453	0.063908	0.41746	0.77401	1.0536	0.07537	0.012494	0.062293	1.1814	0.24045	0.61488	0.99481	1.043	0.060741	0.28382	0.91738	0.95277	-0.0698
567.8929	10.02	lomics Pos	0.44101	0.99995	1.0561	0.078731	0.98903	0.99979	1.0056	0.008071	0.097403	0.68022	0.94517	-0.08136	0.79341	0.99996	1.0391	0.055275	0.4404	0.99701	0.93813	-0.09214	0.88836	0.99959	0.99751	-0.0036	0.014199	0.16883	1.2748	0.3503	0.934	0.99984	1.0029	0.004166	0.34942	0.99966	1.0725	0.10092
507.9791	5.12	olomics Ne	0.38566	0.9951	0.94171	-0.08664	0.31685	0.99975	0.93434	-0.09798	0.10628	0.99731	0.91691	-0.12514	0.50964	0.99915	0.95829	-0.06146	0.50466	0.84755	1.0321	0.045612	0.99171	0.99877	1.0002	0.000341	0.025509	0.08627	1.1532	0.20567	0.70271	0.99481	0.98083	-0.02792	0.053395	0.91738	0.86475	-0.20965
996.8354	11.96	olomics Ne	0.47017	0.9951	1.0311	0.044223	0.494	0.99975	1.0437	0.061708	0.47636	0.99731	0.97605	-0.03498	0.88081	0.99915	0.99381	-0.00895	0.27622	0.71896	1.0425	0.060115	0.40156	0.76531	1.0884	0.12224	0.028121	0.091097	1.2145	0.28031	0.67826	0.99481	1.0184	0.026234	0.41286	0.94239	0.96717	-0.04816
583.5106	5.22	lomics Pos	0.94507	0.99995	1.0006	0.000821	0.80088	0.99979	1.0184	0.026369	0.69316	0.99601	1.0241	0.034297	0.49991	0.99996	0.94038	-0.08868	0.41132	0.99701	1.14	0.18901	0.92625	0.99959	1.0312	0.044382	0.019017	0.17894	1.5105	0.59503	0.52963	0.99984	1.0856	0.11851	0.14008	0.99966	1.1875	0.24789
149.9241	10.65	olomics Ne	0.026328	0.9951	1.1365	0.18466	0.28911	0.99975	1.0508	0.07147	0.48588	0.99731	1.0288	0.041015	0.68098	0.99915	1.0232	0.033123	0.2969	0.7204	1.0466	0.065751	0.10639	0.53519	1.0896	0.12386	0.009756	0.060961	1.1655	0.22097	0.30594	0.99481	1.0766	0.10654	0.76161	0.97489	0.98539	-0.02124
190.1673	1.66	lomics Pos	0.006046	0.68962	0.86349	-0.21175	0.043559	0.99441	0.70372	-0.50693	0.000125	0.13107	0.69198	-0.5312	0.22053	0.99996	0.84947	-0.23537	0.089762	0.82209	0.81967	-0.28688	0.16611	0.99959	0.77118	-0.37485	0.13251	0.33568	1.2117	0.27698	0.19265	0.99984	0.78811	-0.34354	0.002673	0.99749	0.81922	-0.28768
827.5625	8.6	olomics Ne	0.73766	0.9951	0.97191	-0.0411	0.99888	0.99975	0.9996	-0.00057	0.47826	0.99731	0.94097	-0.08778	0.96514	0.99915	0.99987	-0.00019	0.36267	0.75206	1.0908	0.12543	0.080765	0.52604	1.1485	0.19978	0.008813	0.059819	1.249	0.32076	0.32275	0.99481	1.0982	0.13508	0.76576	0.97489	1.0134	0.019152
628.3841	3.08	olomics Pc	0.72754	0.99964	0.9506	-0.07309	0.43338	1	0.88343	-0.17881	0.099697	0.99882	0.7583	-0.39916	0.59393	0.99987	0.94282	-0.08494	0.041168	0.95986	0.65683	-0.60641	0.69113	0.99796	0.93896	-0.09087	0.8178	0.99768	1.253	0.32539	0.41408	0.9981	0.86666	-0.20646	0.54407	0.99995	0.90313	-0.147
817.7072	6.41	lomics Pos	0.17364	0.99995	1.457	0.54302	0.62529	0.99979	0.89556	-0.15913	0.26865	0.88508	1.4394	0.52542	0.84657	0.99996	1.077	0.10705	0.54332	0.99701	1.1559	0.20897	0.49856	0.99959	0.86064	-0.21652	0.012282	0.16313	1.9113	0.93452	0.20165	0.99984	1.3918	0.477	0.10948	0.99966	1.3531	0.43622
609.3089	1.3	lomics Pos	0.044568	0.99995	0.82991	-0.26897	0.23496	0.99979	0.87536	-0.19204	0.039175	0.42996	0.75197	-0.41126	0.34039	0.99996	0.89462	-0.16065	0.095668	0.82209	0.82843	-0.27155	0.24328	0.99959	0.9163	-0.12611	0.11971	0.32144	1.1244	0.16921	0.079897	0.99984	0.85229	-0.23058	0.73623	0.99966	1.0243	0.034671
130.9914	12.11	olomics Ne	0.31108	0.9951	0.95434	-0.06742	0.27436	0.99975	1.0442	0.062424	0.46707	0.99731	0.9585	-0.06116	0.66233	0.99915	0.97665	-0.03409	0.26457	0.71896	1.0654	0.091394	0.48729	0.79937	1.0398	0.056268	0.010387	0.06126	1.1888	0.24952	0.93916	0.99481	0.99795	-0.00296	0.10739	0.91738	0.93594	-0.09552
965.7199	9.88	olomics Pc	0.80684	0.99964	1.0038	0.005441	0.4379	1	0.8876	-0.17202	0.28541	0.99882	0.78155	-0.35559	0.98814	0.99987	1.3672	0.45123	0.045659	0.95986	0.61788	-0.6946	0.38511	0.99796	0.87219	-0.19729	0.92048	0.99768	1.2231	0.29049	0.27245	0.9981	0.80812	-0.30736	0.93633	0.99995	1.1484	0.1996
148.1174	4.74	lomics Pos	0.32377	0.99995	1.1378	0.18618	0.05432	0.99441	1.2063	0.27064	0.13884	0.7467	1.1965	0.25883	0.59909	0.99996	1.1205	0.16416	0.34971	0.99701	1.1085	0.14866	0.35121	0.99959	1.0889	0.12286	0.005109	0.13187	1.3911	0.4762	0.91987	0.99984	1.0069	0.009874	0.054042	0.99966	1.1941	0.25591
498.902	10.02	lomics Pos	0.43451	0.99995	1.0525	0.073862	0.99065	0.99979	1.0027	0.003819	0.026746	0.34652	0.95177	-0.07132	0.98197	0.99996	1.0046	0.006638	0.71341	0.99701	0.98432	-0.02281	0.9468	0.99959	1.0091	0.013131	0.025652	0.18992	1.2571	0.33007	0.97375	0.99984	1.0103	0.014715	0.45536	0.99966	1.0616	0.086245
510.8553	10.14	lomics Pos	0.14855	0.99995	1.1183	0.16133	0.22933	0.99979	0.85745	-0.22187	0.8021	0.99601	0.98388	-0.02345	0.4383	0.99996	0.92705	-0.10928	0.60954	0.99701	0.95774	-0.06229	0.93154	0.99959	1.0162	0.023245	0.032894	0.20029	1.3176	0.39787	0.89663	0.99984	0.99905	-0.00137	0.90017	0.99966	1.0003	0.000475
334.2884	2.75	lomics Pos	0.96051	0.99995	1.0316	0.044948	0.19514	0.99979	0.77497	-0.36779	0.38786	0.94139	0.87242	-0.19691	0.84267	0.99996	1.0346	0.049067	0.59242	0.99701	0.93057	-0.10382	0.013275	0.99959	0.60253	-0.73089	0.83094	0.90216	1.0715	0.099677	0.46846	0.99984	0.9078	-0.13956	0.13849	0.99966	0.82737	-0.27339
550.6283	7.69	olomics Pc	0.048489	0.99964	1.8611	0.89616	0.53963	1	1.3793	0.46389	0.36815	0.99882	1.4968	0.58185	0.062495	0.99987	1.7389	0.79816	0.043372	0.95986	1.8329	0.87415	0.12118	0.99796	3.6915	1.8842	0.6287	0.99768	1.3999	0.48533	0.03132	0.9981	1.9753	0.98206	0.10892	0.99995	1.6183	0.69449
191.0951	10.16	lomics Pos	0.97797	0.99995	1.0024	0.003484	0.78589	0.99979	1.0353	0.050055	0.033115	0.38971	0.9399	-0.08941	0.66598	0.99996	1.0189	0.026994	0.43573	0.99701	0.95957	-0.05953	0.51776	0.99959	0.96554	-0.05059	0.060729	0.24375	1.2202	0.28706	0.76984	0.99984	1.032	0.045438	0.88405	0.99966	1.012	0.017222
114.9845	12.28	olomics Ne	0.95466	0.9951	1.0013	0.001916	0.84883	0.99975	0.98971	-0.01492	0.33797	0.99731	0.95479	-0.06675	0.63177	0.99915	1.0232	0.033122	0.16988	0.6597	1.0832	0.11534	0.2605	0.66013	1.0823	0.11412	0.007881	0.057477	1.2432	0.31409	0.50492	0.99481	0.97119	-0.04217	0.38146	0.92651	0.94855	-0.07621
121.1023	6	olomics Pc	0.010255	0.68734	0.8447	-0.24349	0.010434	0.88953	0.83403	-0.26183	0.017695	0.63482	0.80047	-0.32108	0.9102	0.99987	1.0055	0.007869	0.23425	0.95986	0.91701	-0.12498	0.15923	0.99796	0.83147	-0.26626	0.17282	0.98544	1.0835	0.11563	0.36568	0.9981	0.88928	-0.1693	0.19289	0.99995	0.86796	-0.2043
97.1025	6.39	olomics Pc	0.098979	0.99964	0.89	-0.16812	0.01382	0.88953	0.80621	-0.31078	0.10284	0.99882	0.90786	-0.13946	0.57678	0.99987	0.94685	-0.07879	0.63551	0.96686	0.97206	-0.04088	0.1328	0.99796	0.79557	-0.32994	0.38859	0.99768	1.0529	0.074394	0.15275	0.9981	0.86043	-0.21687	0.13653	0.99995	0.88108	-0.18265
82.9461	0.57	lomics Pos	0.9223	0.99995	0.99621	-0.00549	0.83825	0.99979	0.99068	-0.01351	0.2052	0.83096	0.92742	-0.1087	0.47872	0.99996	0.94423	-0.08279	0.24439	0.99701	0.9483	-0.07659	0.021775	0.99959	0.89001	-0.16811	0.62123	0.78216	1.0638	0.089248	0.13461	0.99984	1.1015	0.13942	0.66907	0.99966	1.0411	0.058071
854.7285	7.73	lomics Pos	0.88201	0.99995	0.96877	-0.04577	0.45788	0.99979	0.82751	-0.27315	0.98871	0.99787	0.96331	-0.05392	0.66596	0.99996	0.88693	-0.1731	0.9945	0.99817	0.97834	-0.0316	0.045872	0.99959	0.54674	-0.87107	0.66186	0.80526	0.92616	-0.11066	0.9439	0.99984	0.9486	-0.07613	0.93557	0.99966	0.94889	-0.07568
1088.77	10.06	lomics Pos	0.43122	0.99995	1.1476	0.19866	0.12514	0.99979	1.0954	0.13142	0.70651	0.99601	1.0338	0.047986	0.53114	0.99996	1.0785	0.10904	0.17577	0.95335	1.1106	0.15134	0.19156	0.99959	1.1956	0.25777	0.004106	0.12302	1.4115	0.49721	0.040256	0.99984	1.2089	0.27372	0.026295	0.99966	1.2007	0.26384
240.9165	12.12	olomics Pc	0.80701	0.99964	1.0082	0.011779	0.7422	1	0.98086	-0.02788	0.31805	0.99882	0.95372	-0.06837	0.013631	0.99987	0.85745	-0.22188	0.17868	0.95986	0.9222	-0.11684	0.28077	0.99796	0.93616	-0.09518	0.41588	0.99768	1.0427	0.060257	0.40096	0.9981	0.93027	-0.10428	0.73936	0.99995	0.98268	-0.02521
644.973	12.01	olomics Ne	0.41042	0.9951	1.1184	0.16147	0.24119	0.99975	1.1874	0.2478	0.51268	0.99731	1.1136	0.15518	0.70398	0.99915	1.0399	0.056387	0.31412	0.74077	1.2225	0.28982	0.45367	0.78329	1.0971	0.13365	0.028598	0.091981	1.5814	0.66123	0.44769	0.99481	1.1865	0.24669	0.6652	0.96789	0.90788	-0.13943
816.9742	12.03	olomics Ne	0.28048	0.9951	1.1727	0.22982	0.030182	0.99975	1.4383	0.52441	0.34914	0.99731	1.2173	0.28365	0.15324	0.99915	1.3127	0.39256	0.36049	0.75206	1.1019	0.14004	0.00733	0.45618	1.4696	0.55541	0.022304	0.078672	1.4206	0.50652	0.34826	0.99481	1.0743	0.10339	0.12183	0.91738	1.2144	0.28027
644.1944	1.29	lomics Pos	0.59143	0.99995	1.1198	0.16321	0.48635	0.99979	0.97701	-0.03356	0.44316	0.98465	0.95304	-0.06939	0.28782	0.99996	1.081	0.11234	0.026141	0.5516	0.86303	-0.21252	0.64954	0.99959	0.95886	-0.06062	0.079934	0.26901	1.2477	0.31927	0.95813	0.99984	1.0082	0.011834	0.31396	0.99966	1.1008	0.13852
514.8745	10.1	lomics Pos	0.71448	0.99995	1.02	0.028513	0.77678	0.99979	1.0161	0.023054	0.70524	0.99601	0.96522	-0.05108	0.66563	0.99996	1.0643	0.089968	0.29524	0.99701	0.90418	-0.14532	0.55261	0.99959	1.0428	0.060423	0.046048	0.22017	1.1991	0.26197	0.78681	0.99984	0.97765	-0.03261	0.2882	0.99966	1.0892	0.12323
905.6688	6.41	lomics Pos	0.25718	0.99995	1.3156	0.39567	0.84738	0.99979	1.0207	0.029499	0.35163	0.93176	1.2757	0.3513	0.41989	0.99996	1.2413	0.31188	0.76604	0.99701	1.0515	0.072464	0.59436	0.99959	0.87545	-0.1919	0.045199	0.2176	1.6635	0.73422	0.53019	0.99984	1.1368	0.18496	0.38426	0.99966	1.2302	0.29888
778.5509	10.23	olomics Ne	0.27648	0.9951	1.1101	0.15069	0.7262	0.99975	0.9561	-0.06477	0.44269	0.99731	1.094	0.12963	0.82955	0.99915	0.9742	-0.03771	0.046334	0.58152	1.2906	0.36808	0.021973	0.4612	1.3675	0.45151	0.036061	0.10421	1.4611	0.54707	0.20073	0.99481	1.2378	0.30777	0.1668	0.91738	1.1977	0.26025
618.2567	1.1	lomics Pos	0.69617	0.99995	0.94147	-0.08701	0.79901	0.99979	1.0264	0.037581	0.24238	0.85804	0.81522	-0.29474	0.42275	0.99996	1.1697	0.22618	0.64804	0.99701	0.92992	-0.10481	0.05178	0.99959	1.2935	0.37126	0.026353	0.19106	1.4774	0.56309	0.88085	0.99984	1.0162	0.023126	0.11466	0.99966	1.2424	0.31308
282.9051	10.23	lomics Pos	0.73713	0.99995	1.0292	0.041578	0.41764	0.99979	1.0682	0.095213	0.92449	0.99702	1.0078	0.011265	0.97682	0.99996	1.0043	0.006135	0.90402	0.99701	1.0034	0.004954	0.77007	0.99959	1.033	0.046827	0.017952	0.17809	1.2864	0.36339	0.63892	0.99984	1.0386	0.054655	0.39363	0.99966	1.1161	0.15848
110.0094	10.16	lomics Pos	0.3345	0.99995	1.0542	0.076142	0.87024	0.99979	0.9947	-0.00767	0.33768	0.92252	0.94828	-0.07661	0.88524	0.99996	1.0116	0.016619	0.80475	0.99701	0.99379	-0.00899	0.94664	0.99959	1.0023	0.003254	0.006618	0.13972	1.2273	0.29548	0.98509	0.99984	1.0065	0.009278	0.48694	0.99966	1.0385	0.054501
1067.766	10.02	lomics Pos	0.037263	0.96003	1.087	0.12031	0.046909	0.99441	1.1175	0.16028	0.74307	0.99601	1.0498	0.070062	0.2849	0.99996	1.0517	0.072685	0.11599	0.8579	1.0669	0.09341	0.11301	0.99959	1.1437	0.19374	0.006092	0.13681	1.3632	0.44695	0.21051	0.99984	1.0917	0.12661	0.065535	0.99966	1.1711	0.22785
778.5725	5.61	lomics Pos	0.64658	0.99995	1.0307	0.043655	0.41225	0.99979	1.0586	0.082089	0.66272	0.99601	0.96659	-0.04902	0.81021	0.99996	1.0156	0.022351	0.78062	0.99701	0.97678	-0.0339	0.21625	0.99959	1.0851	0.11783	0.024685	0.18846	1.3316	0.41311	0.3795	0.99984	1.0846	0.11721	0.020294	0.99966	1.2604	0.33389
888.526	5.08	lomics Pos	0.88202	0.99995	1.1083	0.14831	0.67263	0.99979	0.96437	-0.05234	0.49245	0.99601	0.9156	-0.12721	0.93854	0.99996	1.0855	0.11835	0.93422	0.99701	1.0399	0.056392	0.78231	0.99959	1.087	0.12037	0.038042	0.20439	1.3895	0.47457	0.90501	0.99984	1.0406	0.057461	0.76155	0.99966	1.124	0.16862
450.9817	4.75	olomics Ne	0.6055	0.9951	1.0245	0.034871	0.3399	0.99975	1.0314	0.044594	0.52288	0.99731	0.97049	-0.04321	0.52022	0.99915	1.0401	0.056739	0.078761	0.5958	1.0902	0.12457	0.1001	0.53216	1.1254	0.17048	0.029996	0.093482	1.2198	0.2866	0.21858	0.99481	1.086	0.11902	0.32258	0.91738	0.95826	-0.06151
1113.823	4.65	lomics Pos	0.65446	0.99995	1.085	0.11772	0.14379	0.99979	1.1566	0.20989	0.96729	0.99702	1.0054	0.007813	0.42764	0.99996	1.0711	0.099063	0.9569	0.99701	1.0014	0.001976	0.97062	0.99959	1.001	0.001407	0.022978	0.18641	1.4848	0.57023	0.36232	0.99984	1.1583	0.21201	0.14196	0.99966	1.3768	0.46136
645.3697	3.1	olomics Pc	0.81452	0.99964	1.0584	0.081924	0.161	1	0.87253	-0.19672	0.023117	0.71215	0.79941	-0.323	0.91347	0.99987	1.0875	0.12095	0.044641	0.95986	0.64715	-0.62784	0.52084	0.99796	0.91759	-0.12408	0.8324	0.99768	1.1052	0.1443	0.54457	0.9981	0.93191	-0.10173	0.21867	0.99995	0.79662	-0.32803
728.5563	4.67	lomics Pos	0.92636	0.99995	1.0064	0.009168	0.91196	0.99979	1.015	0.021424	0.79594	0.99601	1.0018	0.00257	0.97284	0.99996	1.0066	0.009506	0.3055	0.99701	0.90466	-0.14455	0.054495	0.99959	0.93477	-0.09731	0.031725	0.20029	1.2157	0.28185	0.58601	0.99984	1.119	0.16218	0.062586	0.99966	1.2556	0.32836
610.3116	1.3	lomics Pos	0.46593	0.99995	0.9343	-0.09804	0.8064	0.99979	1.055	0.077183	0.22164	0.83453	0.87968	-0.18495	0.04691	0.99996	0.83017	-0.26852	0.33479	0.99701	0.89162	-0.16549	0.52317	0.99959	0.93445	-0.09781	0.11813	0.32066	1.1345	0.182	0.089406	0.99984	0.87383	-0.19457	0.97894	0.99966	1.0296	0.042134
604.2945	1.38	lomics Pos	0.96729	0.99995	0.99355	-0.00933	0.73205	0.99979	0.9561	-0.06476	0.13727	0.74334	0.84874	-0.2366	0.34412	0.99996	0.87523	-0.19227	0.21548	0.99598	0.83122	-0.26669	0.55895	0.99959	1.075	0.10439	0.040986	0.20898	1.3596	0.44316	0.62133	0.99984	0.93845	-0.09165	0.8553	0.99966	0.97384	-0.03825
205.1957	1.66	lomics Pos	0.11335	0.99995	0.82582	-0.27611	0.10506	0.99979	0.7771	-0.36383	0.001284	0.13107	0.6779	-0.56086	0.57434	0.99996	0.94744	-0.07789	0.16971	0.94469	0.90201	-0.14878	0.25097	0.99959	0.86701	-0.20588	0.028733	0.19804	1.2815	0.35785	0.34259	0.99984	0.86104	-0.21585	0.48581	0.99966	0.9417	-0.08666
957.7717	0.54	lomics Pos	0.31124	0.99995	1.1228	0.16714	0.46122	0.99979	1.1041	0.14285	0.65328	0.99601	0.98296	-0.0248	0.055244	0.99996	1.1795	0.23813	0.68033	0.99701	0.97718	-0.0333	0.036924	0.99959	1.1505	0.2022	0.03669	0.20439	1.3517	0.43473	0.50543	0.99984	1.0542	0.076214	0.21242	0.99966	1.1558	0.20892
761.8259	10.05	lomics Pos	0.23468	0.99995	1.0912	0.12596	0.2647	0.99979	1.1163	0.15876	0.64225	0.99601	1.0542	0.07621	0.53888	0.99996	1.0523	0.073492	0.80091	0.99701	0.98463	-0.02235	0.63849	0.99959	1.0662	0.092429	0.016835	0.17568	1.2366	0.30634	0.39964	0.99984	1.0866	0.11977	0.24392	0.99966	1.0972	0.1338
1154.755	10.03	lomics Pos	0.6735	0.99995	1.0408	0.05776	0.34962	0.99979	0.96099	-0.0574	0.80124	0.99601	0.98764	-0.01795	0.17039	0.99996	0.89377	-0.16202	0.87258	0.99701	0.99451	-0.00795	0.20113	0.99959	0.93656	-0.09455	0.025782	0.19041	1.3016	0.38028	0.92743	0.99984	1.0035	0.005054	0.4545	0.99966	1.0708	0.098725
1097.777	10.02	lomics Pos	0.20648	0.99995	0.90764	-0.1398	0.56491	0.99979	1.0509	0.071689	0.28892	0.89498	0.93433	-0.09799	0.5681	0.99996	0.96455	-0.05207	0.041986	0.65306	0.88495	-0.17634	0.27268	0.99959	0.91944	-0.12117	0.12275	0.32368	1.129	0.17499	0.63782	0.99984	0.9596	-0.05949	0.50588	0.99966	1.0651	0.090983
463.7689	12.74	olomics Ne	0.56682	0.9951	1.0574	0.080481	0.80825	0.99975	1.018	0.02577	0.29529	0.99731	1.0587	0.08225	0.21306	0.99915	1.0605	0.084761	0.1001	0.60605	1.1269	0.17235	0.42125	0.77401	1.0621	0.086967	0.018944	0.07326	1.2688	0.34342	0.17999	0.99481	1.0787	0.10923	0.23292	0.91738	0.91676	-0.12539
409.1654	0.69	lomics Pos	0.76236	0.99995	1.0452	0.063739	0.12835	0.99979	1.0974	0.13412	0.50602	0.99601	0.97173	-0.04138	0.20943	0.99996	0.93736	-0.09333	0.02413	0.5516	0.84169	-0.24865	0.91118	0.99959	1.0026	0.00378	0.49164	0.69446	1.159	0.21291	0.90696	0.99984	0.99599	-0.0058	0.29245	0.99966	1.1592	0.21315
941.3224	11.96	olomics Pc	0.90723	0.99964	1.0116	0.016652	0.74306	1	1.0398	0.056249	0.67049	0.99882	1.0123	0.017574	0.83534	0.99987	1.036	0.051034	0.66002	0.96836	1.0368	0.052104	0.5204	0.99796	0.95792	-0.06203	0.042553	0.70447	1.131	0.17757	0.61231	0.9981	1.056	0.078586	0.4327	0.99995	1.0718	0.099982
702.8639	10.02	lomics Pos	0.33537	0.99995	1.064	0.08944	0.54772	0.99979	1.0258	0.036763	0.04281	0.44722	0.9668	-0.04872	0.77774	0.99996	1.0244	0.034798	0.79751	0.99701	0.99054	-0.01371	0.63581	0.99959	1.0346	0.049134	0.009111	0.1521	1.2952	0.37313	0.82647	0.99984	1.028	0.039816	0.46678	0.99966	1.0586	0.082164
278.2488	5.65	olomics Pc	0.019369	0.86976	0.89504	-0.15998	0.020149	0.88953	0.75303	-0.40921	0.010731	0.58293	0.81181	-0.30079	0.91648	0.99987	1.0072	0.0103	0.14152	0.95986	0.8937	-0.16213	0.34869	0.99796	0.89013	-0.16791	0.029108	0.62801	1.1296	0.17575	0.27333	0.9981	0.85246	-0.2303	0.058639	0.99995	0.82091	-0.2847
1139.726	9.01	olomics Pc	0.63226	0.99964	0.92117	-0.11847	0.28936	1	0.84766	-0.23844	0.20032	0.99882	0.77753	-0.36302	0.37239	0.99987	0.83444	-0.26111	0.043973	0.95986	0.67696	-0.56286	0.12149	0.99796	0.7639	-0.38855	0.94345	0.99768	1.1198	0.16326	0.4884	0.9981	0.88967	-0.16865	0.88379	0.99995	1.1149	0.15696
218.921	0.56	lomics Pos	0.89713	0.99995	1.0038	0.005405	0.55994	0.99979	0.97935	-0.03011	0.01614	0.28182	0.92053	-0.11946	0.63744	0.99996	0.96922	-0.0451	0.039115	0.63738	0.91905	-0.12178	0.15609	0.99959	0.94801	-0.07703	0.76716	0.865	1.0283	0.040251	0.41232	0.99984	1.0567	0.079546	0.42199	0.99966	1.058	0.081317
839.5879	8.9	olomics Ne	0.84394	0.9951	0.99003	-0.01446	0.32512	0.99975	0.95474	-0.06681	0.43681	0.99731	0.96515	-0.05117	0.51769	0.99915	1.0488	0.068671	0.10363	0.60605	1.1039	0.14256	0.20364	0.63388	1.1229	0.16723	0.005509	0.052776	1.2273	0.29547	0.9439	0.99481	0.99638	-0.00523	0.10356	0.91738	0.91839	-0.12281
69.0712	6.38	olomics Pc	0.17565	0.99964	0.91159	-0.13354	0.009261	0.88953	0.71814	-0.47765	0.14423	0.99882	0.90381	-0.14592	0.9437	0.99987	1.0338	0.048026	0.9955	0.99906	1.004	0.005757	0.075338	0.99796	0.77631	-0.36529	0.39877	0.99768	1.0516	0.072642	0.18174	0.9981	0.86121	-0.21556	0.39992	0.99995	0.91036	-0.13549
412.9817	4.63	olomics Ne	0.61209	0.9951	1.019	0.027206	0.24137	0.99975	1.0759	0.10558	0.45134	0.99731	0.96449	-0.05216	0.50136	0.99915	1.0483	0.068115	0.23683	0.7131	1.0585	0.082049	0.15176	0.58382	1.115	0.15707	0.005859	0.054231	1.2308	0.29962	0.50164	0.99481	1.0415	0.058619	0.49328	0.95203	0.96832	-0.04644
726.5414	5.31	lomics Pos	0.64041	0.99995	1.073	0.10162	0.76935	0.99979	1.0368	0.052172	0.39533	0.9483	0.86966	-0.20147	0.774	0.99996	0.95619	-0.06464	0.47407	0.99701	0.89362	-0.16227	0.22691	0.99959	1.2022	0.26571	0.022477	0.18641	1.6796	0.74808	0.34199	0.99984	1.2208	0.28778	0.1006	0.99966	1.3666	0.45056
405.9808	4.79	olomics Ne	0.1881	0.9951	1.0938	0.12932	0.79092	0.99975	1.0226	0.032285	0.26012	0.99731	0.91134	-0.13394	0.20689	0.99915	1.0729	0.10148	0.057609	0.59097	1.1694	0.22576	0.047491	0.49377	1.1851	0.24498	0.010832	0.061656	1.2487	0.32038	0.54083	0.99481	1.04	0.056587	0.51683	0.95528	1.0482	0.067961
286.9775	12	olomics Ne	0.88165	0.9951	0.99456	-0.00787	0.3585	0.99975	1.1013	0.13916	0.39454	0.99731	1.0448	0.063278	0.1466	0.99915	1.0866	0.11979	0.060681	0.5958	1.0605	0.084812	0.21682	0.6375	1.0818	0.11344	0.020757	0.076566	1.2701	0.34491	0.8652	0.99481	1.0094	0.013496	0.030475	0.91738	0.92338	-0.11501
318.9816	3.86	olomics Ne	0.18393	0.9951	1.0743	0.10338	0.47732	0.99975	1.0509	0.07166	0.5265	0.99731	0.96466	-0.05191	0.85022	0.99915	1.0126	0.018023	0.048646	0.58152	1.0988	0.13588	0.074875	0.5223	1.129	0.17501	0.010823	0.061656	1.2177	0.28416	0.15675	0.99481	1.0789	0.10962	0.72173	0.97327	0.98034	-0.02865
213.0066	10.19	lomics Pos	0.14941	0.99995	1.1967	0.2591	0.89259	0.99979	1.0207	0.029505	0.086746	0.64718	1.1756	0.23339	0.31927	0.99996	1.1089	0.14907	0.2494	0.99701	1.1546	0.20735	0.71218	0.99959	1.0288	0.040971	0.007074	0.13972	1.3831	0.46788	0.31105	0.99984	1.1494	0.20091	0.064088	0.99966	1.2974	0.37564
1137.749	9.01	olomics Pc	0.90631	0.99964	0.98471	-0.02222	0.57103	1	0.91456	-0.12886	0.19744	0.99882	0.77736	-0.36335	0.75601	0.99987	1.0037	0.005319	0.049611	0.95986	0.67615	-0.56459	0.39988	0.99796	0.87374	-0.19472	0.63912	0.99768	1.3851	0.46997	0.72687	0.9981	0.94603	-0.08004	0.98547	0.99995	1.1405	0.1897
759.6356	5.65	lomics Pos	0.41881	0.99995	1.2439	0.31492	0.35326	0.99979	0.84796	-0.23793	0.37942	0.9382	1.197	0.25945	0.75856	0.99996	1.0406	0.057471	0.8135	0.99701	1.1268	0.17221	0.34543	0.99959	0.81322	-0.29828	0.041304	0.20971	1.4096	0.49532	0.55383	0.99984	1.0934	0.12887	0.46319	0.99966	1.1828	0.24217
258.6428	1.3	lomics Pos	0.7033	0.99995	1.0869	0.12018	0.04684	0.99441	0.8088	-0.30615	0.18169	0.80578	0.90354	-0.14635	0.11673	0.99996	0.83783	-0.25527	0.085065	0.8126	0.86811	-0.20405	0.54736	0.99959	0.95802	-0.06187	0.081023	0.27033	1.1911	0.25235	0.39999	0.99984	0.89033	-0.16759	0.78724	0.99966	0.98412	-0.02309
292.983	12.01	olomics Ne	0.70923	0.9951	1.0305	0.043328	0.75539	0.99975	1.0407	0.057615	0.5966	0.99731	0.96081	-0.05768	0.54767	0.99915	1.0519	0.072986	0.35593	0.75206	1.0635	0.088764	0.61806	0.85073	1.0424	0.059868	0.044232	0.11766	1.1659	0.22148	0.84218	0.99481	0.9831	-0.02459	0.3846	0.92771	0.93399	-0.09851
452.9031	0.28	olomics Ne	0.015969	0.9951	1.135	0.18265	0.15159	0.99975	0.57081	-0.80891	0.0433	0.99731	1.0973	0.13401	0.14029	0.99915	0.54729	-0.86962	0.000465	0.20011	1.1771	0.23521	0.4352	0.77401	0.85936	-0.21866	0.47919	0.58346	0.94049	-0.08851	0.12337	0.99481	1.0808	0.11209	0.34677	0.92398	0.75609	-0.40336
803.6887	6.23	lomics Pos	0.27366	0.99995	1.2853	0.36213	0.61246	0.99979	1.0995	0.13679	0.28616	0.8936	1.2254	0.29325	0.73864	0.99996	1.1202	0.16377	0.99156	0.9977	1.0022	0.003134	0.83086	0.99959	0.97555	-0.03572	0.029536	0.19804	1.6848	0.75255	0.64382	0.99984	1.1292	0.1753	0.049956	0.99966	1.2808	0.35709
555.9772	12.02	olomics Ne	0.01565	0.9951	1.4471	0.53312	0.32527	0.99975	1.1737	0.2311	0.11042	0.99731	1.229	0.2975	0.37397	0.99915	1.1672	0.22311	0.01615	0.50209	1.4214	0.50731	0.090392	0.52866	1.3348	0.41668	0.076092	0.16196	1.5411	0.62397	0.083308	0.99481	1.3438	0.42631	0.38488	0.92771	0.87115	-0.19901
524.9743	12	olomics Ne	0.80472	0.9951	1.0167	0.023841	0.63241	0.99975	1.0561	0.078774	0.8785	0.99731	1.0355	0.050361	0.074629	0.99915	1.2058	0.26997	0.26971	0.71896	1.1179	0.16073	0.66321	0.87408	1.0258	0.036797	0.044637	0.11766	1.2785	0.3545	0.98193	0.99785	0.99375	-0.00905	0.23081	0.91738	0.88551	-0.17542
766.9298	3.25	olomics Pc	0.52853	0.99964	0.92579	-0.11125	0.026359	0.88953	0.72978	-0.45447	0.12289	0.99882	0.84359	-0.24538	0.60776	0.99987	0.94512	-0.08143	0.14755	0.95986	0.76357	-0.38917	0.057576	0.99796	0.77996	-0.35852	0.98652	0.99932	1.1396	0.18852	0.53138	0.9981	0.92444	-0.11335	0.65058	0.99995	0.95729	-0.06298
1078.76	10.09	lomics Pos	0.31791	0.99995	1.1165	0.15898	0.7363	0.99979	1.0293	0.04169	0.36329	0.93332	0.91326	-0.1309	0.93581	0.99996	1.0014	0.002032	0.92899	0.99701	1.0014	0.001948	0.79214	0.99959	0.97797	-0.03214	0.019929	0.18098	1.2349	0.30435	0.25133	0.99984	1.174	0.23146	0.76879	0.99966	1.0651	0.090922
516.2729	10.18	olomics Ne	0.94522	0.9951	0.99786	-0.0031	0.40541	0.99975	1.0759	0.1055	0.26358	0.99731	0.91399	-0.12974	0.68547	0.99915	1.0224	0.031936	0.26168	0.71743	1.065	0.090847	0.18375	0.61607	1.1601	0.21421	0.001534	0.041313	1.4191	0.50498	0.27593	0.99481	1.1158	0.15805	0.022339	0.91738	1.1272	0.17276
181.989	10.19	lomics Pos	0.35442	0.99995	1.0733	0.10201	0.47872	0.99979	1.0752	0.10463	0.1763	0.79932	1.1275	0.17317	0.41029	0.99996	1.0759	0.10551	0.24933	0.99701	1.0878	0.12136	0.79039	0.99959	1.0176	0.025114	0.0002	0.063527	1.3324	0.41401	0.14111	0.99984	1.1494	0.20083	0.050631	0.99966	1.0989	0.13608
858.5896	5.23	lomics Pos	0.89723	0.99995	1.0364	0.051569	0.84722	0.99979	1.0272	0.038689	0.58899	0.99601	0.94702	-0.07854	0.94774	0.99996	1.0058	0.008308	0.94149	0.99701	0.99931	-0.001	0.77001	0.99959	0.97205	-0.0409	0.025536	0.18992	1.2176	0.28407	0.91601	0.99984	0.99566	-0.00628	0.6313	0.99966	1.0704	0.098202
367.3363	4.18	lomics Pos	0.45945	0.99995	1.0592	0.083014	0.3481	0.99979	1.0856	0.11846	0.055357	0.51947	0.84442	-0.24396	0.6782	0.99996	0.96323	-0.05405	0.55037	0.99701	1.055	0.077243	0.11378	0.99959	1.169	0.22532	0.036598	0.20439	1.4182	0.50405	0.11425	0.99984	1.1414	0.19078	0.16318	0.99966	1.1349	0.18252
276.8771	9.92	lomics Pos	0.93682	0.99995	1.0041	0.005911	0.055087	0.99504	1.25	0.3219	0.038599	0.4266	0.74466	-0.42534	0.48041	0.99996	1.0982	0.13513	0.48808	0.99701	0.93161	-0.1022	0.79838	0.99959	1.0602	0.084373	0.085225	0.27673	1.3687	0.45281	0.30983	0.99984	1.177	0.23512	0.14221	0.99966	1.4045	0.4901
118.9922	12.03	olomics Ne	0.97684	0.9951	0.99651	-0.00504	0.4236	0.99975	1.0729	0.10155	0.4151	0.99731	0.94953	-0.07471	0.1957	0.99915	1.0699	0.097418	0.70732	0.92089	1.0187	0.026778	0.17521	0.60765	1.1175	0.16021	0.007432	0.057477	1.1976	0.26013	0.95194	0.99481	1.0077	0.011059	0.22752	0.91738	0.93718	-0.09361
1112.496	6.01	lomics Pos	0.6575	0.99995	0.956	-0.06492	0.3454	0.99979	0.89205	-0.16481	0.0202	0.30717	0.7749	-0.36792	0.25945	0.99996	0.87196	-0.19766	0.95566	0.99701	1.0127	0.018159	0.985	0.99959	1.0255	0.036364	0.35742	0.57968	1.1463	0.19693	0.77755	0.99984	1.0362	0.051248	0.61067	0.99966	1.055	0.077309
965.7866	10.03	lomics Pos	0.18046	0.99995	1.0836	0.11588	0.70674	0.99979	1.0255	0.036319	0.97638	0.99702	0.99812	-0.00272	0.45407	0.99996	0.96302	-0.05437	0.92304	0.99701	1.0068	0.009708	0.44087	0.99959	0.94786	-0.07725	0.012845	0.16313	1.2642	0.33817	0.81632	0.99984	1.0297	0.042187	0.25941	0.99966	1.1289	0.17492
493.3291	6.27	olomics Ne	0.56477	0.9951	0.92561	-0.11153	0.5447	0.99975	0.94671	-0.079	0.81424	0.99731	0.97884	-0.03086	0.30463	0.99915	0.88537	-0.17564	0.83298	0.96161	1.0387	0.054771	0.13264	0.55497	1.2454	0.3166	0.043523	0.11647	1.2335	0.30272	0.69946	0.99481	1.0751	0.10444	0.86413	0.99168	1.0399	0.056394
1154.751	5.57	lomics Pos	0.71541	0.99995	1.1472	0.19808	0.48273	0.99979	1.2307	0.29945	0.47052	0.99601	1.1734	0.23075	0.83038	0.99996	1.1075	0.14729	0.53453	0.99701	1.185	0.24491	0.59902	0.99959	1.1226	0.16689	0.036555	0.20439	1.4325	0.51858	0.61347	0.99984	1.1228	0.16705	0.11668	0.99966	1.2261	0.29413
770.851	0.5	lomics Pos	0.67383	0.99995	1.0226	0.032307	0.64862	0.99979	1.0267	0.037989	0.040018	0.43226	0.93483	-0.09722	0.72186	0.99996	0.98186	-0.02641	0.3879	0.99701	0.96835	-0.0464	0.97662	0.99959	1.0026	0.003778	0.036923	0.20439	1.1993	0.26222	0.88544	0.99984	0.9962	-0.0055	0.54703	0.99966	1.0381	0.053998

737.5247	8.39	olomics Ne	0.606	0.9951	1.0682	0.09522	0.97421	0.99975	1.0032	0.004567	0.92091	0.99731	1.005	0.00717	0.47422	0.99915	1.0579	0.081256	0.18476	0.6754	1.1952	0.25725	0.022666	0.4612	1.2034	0.26707	0.003916	0.050041	1.4351	0.52111	0.055364	0.99481	1.1566	0.2099	0.32744	0.91738	0.91802	-0.1234
925.807	10.05	domics Pos	0.44923	0.99995	1.0853	0.11807	0.56782	0.99979	1.1096	0.15004	0.415	0.96712	1.123	0.16736	0.49065	0.99996	1.0666	0.092969	0.18999	0.97027	1.1593	0.21329	0.87994	0.99959	1.021	0.03003	0.039025	0.20439	1.3199	0.40043	0.70693	0.99984	1.0515	0.072419	0.32141	0.99966	1.0875	0.12095
748.528	5.12	domics Pos	0.57603	0.99995	1.0427	0.060311	0.36612	0.99979	1.0595	0.083375	0.38599	0.94042	0.943	-0.08467	0.74198	0.99996	1.0436	0.061627	0.99083	0.9977	0.99837	-0.00236	0.23215	0.99959	1.1107	0.15149	0.008098	0.14438	1.3684	0.45245	0.216	0.99984	1.1352	0.18293	0.009795	0.99749	1.2859	0.36283
84.9604	10.23	domics Pos	0.81824	0.99995	1.0134	0.019265	0.82606	0.99979	1.0129	0.01855	0.2906	0.89498	0.88458	-0.17693	0.57913	0.99996	0.954	-0.06795	0.37463	0.99701	0.93639	-0.09482	0.93458	0.99959	0.99903	-0.0014	0.01174	0.16313	1.3396	0.42177	0.9199	0.99984	0.99538	-0.00668	0.6716	0.99966	1.0239	0.034066
229.8736	12.64	olomics Ne	0.31891	0.9951	1.1067	0.14629	0.26863	0.99975	1.0826	0.11445	0.57612	0.99731	0.9711	-0.04231	0.98264	0.99915	1.0045	0.006522	0.26627	0.71896	1.0966	0.13303	0.1986	0.63388	1.1422	0.19178	0.007802	0.057477	1.2321	0.30116	0.91721	0.99481	1.0096	0.013784	0.34525	0.92398	0.94036	-0.08871
617.2597	1.11	domics Pos	0.74918	0.99995	0.96809	-0.04679	0.3833	0.99979	1.1739	0.23133	0.68847	0.99601	0.95902	-0.06036	0.6585	0.99996	1.1036	0.14223	0.44954	0.99701	1.0808	0.11208	0.005822	0.99959	1.4111	0.49687	0.005484	0.1319	1.7598	0.8154	0.71642	0.99984	1.0319	0.045243	0.037336	0.99966	1.2593	0.33261
104.1081	1.84	domics Pos	0.55906	0.99995	0.94513	-0.08142	0.64692	0.99979	0.96665	-0.04893	0.29378	0.89498	0.87791	-0.18785	0.24032	0.99996	0.8674	-0.20523	0.24218	0.99701	0.8851	-0.17609	0.67842	0.99959	0.96213	-0.0557	0.007961	0.14438	1.2734	0.34873	0.82799	0.99984	0.98807	-0.01732	0.56949	0.99966	1.0821	0.11385
103.0387	0.54	domics Pos	0.33129	0.99995	1.1406	0.18981	0.80697	0.99979	1.0317	0.045016	0.54678	0.99601	1.1135	0.15516	0.71203	0.99996	1.097	0.13362	0.15061	0.9159	1.1814	0.24046	0.77804	0.99959	1.1501	0.2018	0.013717	0.1677	1.9997	0.99981	0.12356	0.99984	1.3608	0.44444	0.91828	0.99966	1.0235	0.033566
354.3735	8.3	olomics Pc	0.25854	0.99964	1.1087	0.14886	0.049797	0.90372	1.1807	0.23959	0.43533	0.99882	1.0753	0.10474	0.008955	0.99987	1.3275	0.4087	0.080634	0.95986	1.1426	0.19233	0.018316	0.99796	1.2795	0.35557	0.17397	0.98544	1.1452	0.19563	0.32017	0.9981	1.1084	0.14844	0.042103	0.99995	1.1812	0.24021
392.7982	9.92	domics Pos	0.92741	0.99995	1.0092	0.013182	0.92205	0.99979	1.0334	0.047332	0.039466	0.42996	0.80166	-0.31893	0.99056	0.99996	1.0025	0.003649	0.66494	0.99701	0.97836	-0.03156	0.41432	0.99959	0.94516	-0.08136	0.030901	0.19986	1.2545	0.32716	0.6936	0.99984	1.0653	0.091285	0.45848	0.99966	1.1648	0.22009
746.5122	8.74	olomics Ne	0.91317	0.9951	0.98392	-0.02339	0.34098	0.99975	1.0988	0.13599	0.83882	0.99731	0.98425	-0.02291	0.41383	0.99915	1.0975	0.13425	0.25518	0.71555	1.1359	0.18388	0.079073	0.52333	1.2478	0.31944	0.026477	0.087443	1.2872	0.36422	0.06358	0.99481	1.2233	0.29078	0.016496	0.91738	1.3186	0.39902
137.9849	2.64	olomics Ne	0.46134	0.9951	1.0467	0.065819	0.98987	0.99975	0.99427	-0.0083	0.77091	0.99731	0.97649	-0.03432	0.96983	0.99915	1.0021	0.00303	0.29261	0.71896	1.0833	0.11542	0.42822	0.77401	1.0595	0.083389	0.021493	0.077635	1.2148	0.28072	0.15306	0.99481	1.1038	0.14243	0.64387	0.96789	1.0325	0.046104
1153.766	10.03	domics Pos	0.66312	0.99995	0.97227	-0.04056	0.96824	0.99979	1.0098	0.014064	0.010472	0.22168	0.82339	-0.28036	0.72244	0.99996	0.97458	-0.03715	0.4371	0.99701	1.0302	0.042989	0.74519	0.99959	0.97988	-0.02933	0.026358	0.19106	1.1601	0.21425	0.028586	0.99984	0.83838	-0.25432	0.8982	0.99966	0.99474	-0.0076
621.4954	2.05	domics Pos	0.99208	0.99995	1.0172	0.02461	0.029546	0.99441	0.73994	-0.43453	0.003168	0.15208	0.74186	-0.43079	0.60404	0.99996	0.94381	-0.08343	0.13378	0.87647	0.88478	-0.1766	0.14443	0.99959	0.74554	-0.42364	0.043621	0.21601	1.2886	0.36576	0.18176	0.99984	0.75901	-0.39781	0.78968	0.99966	1.0583	0.081748
295.647	5.32	olomics Pc	0.10205	0.99964	0.78382	-0.35141	0.3731	1	0.9016	-0.14944	0.023286	0.71215	0.70367	-0.50703	0.70766	0.99987	1.0318	0.045127	0.85782	0.99867	1.0359	0.050909	0.61563	0.99796	1.0721	0.10045	0.80401	0.99768	1.0302	0.042988	0.55839	0.9981	0.92315	-0.11536	0.77142	0.99995	1.0253	0.035992
90.9775	10.03	domics Pos	0.4613	0.99995	1.0511	0.071955	0.92756	0.99979	1.0067	0.009601	0.10202	0.68915	0.96617	-0.04965	0.9393	0.99996	1.0012	0.001769	0.70275	0.99701	0.9839	-0.02342	0.91378	0.99959	1.0117	0.016828	0.020729	0.1817	1.2563	0.32921	0.89408	0.99984	1.0174	0.024946	0.5185	0.99966	1.0457	0.06448
710.8082	10.12	domics Pos	0.41782	0.99995	1.0698	0.097298	0.10662	0.99979	1.0847	0.11733	0.95804	0.99702	0.99989	-0.00015	0.77085	0.99996	0.98157	-0.02683	0.34167	0.99701	0.92956	-0.10538	0.26127	0.99959	1.0971	0.13376	0.019653	0.18098	1.4625	0.54843	0.87516	0.99984	0.99325	-0.00978	0.096022	0.99966	1.1768	0.23484
154.1209	9.97	domics Pos	0.80139	0.99995	0.97607	-0.03494	0.2101	0.99979	1.3953	0.48057	0.20667	0.83096	0.84285	-0.24664	0.71161	0.99996	0.95304	-0.06939	0.012474	0.48469	0.72081	-0.47231	0.79943	0.99959	1.0999	0.13736	0.31705	0.53843	1.1273	0.17284	0.95779	0.99984	1.0405	0.057269	0.92041	0.99966	1.0466	0.065694
206.1976	2.05	domics Pos	0.12518	0.99995	0.86051	-0.21673	0.095583	0.99979	0.766	-0.38458	0.010172	0.21867	0.70671	-0.50081	0.59017	0.99996	0.93712	-0.09369	0.079055	0.80167	0.78599	-0.34743	0.15358	0.99959	0.77721	-0.36363	0.24218	0.46374	1.1801	0.23893	0.10962	0.99984	0.76296	-0.39032	0.15201	0.99966	0.87272	-0.19641
645.2995	2.99	domics Pos	0.71444	0.99995	1.0234	0.033354	0.5427	0.99979	0.93182	-0.10188	0.028694	0.36109	0.82224	-0.28237	0.21799	0.99996	0.88641	-0.17396	0.29422	0.99701	0.90086	-0.15062	0.036965	0.99959	0.78081	-0.35695	0.86676	0.92472	0.9894	-0.01538	0.97227	0.99984	1.0057	0.008187	0.91988	0.99966	1.0144	0.020679
195.1239	6.16	olomics Pc	0.38303	0.99964	1.1932	0.25482	0.024301	0.88953	1.1466	0.1974	0.50287	0.99882	1.06	0.08412	0.82132	0.99987	0.9872	-0.01858	0.86675	0.99904	1.0283	0.040294	0.7842	0.99796	1.028	0.039827	0.91826	0.99768	1.0323	0.045915	0.47147	0.9981	1.1499	0.20155	0.87067	0.99995	1.0157	0.022464
428.9785	5	olomics Ne	0.72817	0.9951	0.98168	-0.02668	0.32298	0.99975	1.0538	0.075634	0.35249	0.99731	0.95438	-0.06736	0.52497	0.99915	1.0325	0.04619	0.23457	0.7131	1.0597	0.083648	0.018921	0.45618	1.1407	0.18996	0.018371	0.072716	1.2491	0.32093	0.89533	0.99481	1.01	0.014416	0.46168	0.94605	0.95657	-0.06406
881.5424	10.38	olomics Ne	0.093489	0.9951	1.2223	0.28963	0.69369	0.99975	1.0515	0.072416	0.3407	0.99731	1.095	0.13089	0.47481	0.99915	1.0693	0.096715	0.12349	0.62697	1.1906	0.25169	0.070488	0.5223	1.2276	0.29587	0.013905	0.065583	1.4281	0.51405	0.18262	0.99481	1.1422	0.19185	0.68106	0.96812	0.94463	-0.08219
160.8534	12.29	olomics Ne	0.61627	0.9951	0.97321	-0.03918	0.19933	0.99975	1.0675	0.094204	0.15186	0.99731	0.90106	-0.15031	0.41632	0.99915	1.0603	0.084439	0.69519	0.91332	1.0307	0.04359	0.19787	0.63388	1.0994	0.13673	0.008986	0.059819	1.2153	0.28136	0.81117	0.99481	0.98545	-0.02115	0.34639	0.92398	0.95119	-0.0722
614.4838	6.21	olomics Pc	0.16184	0.99964	0.91821	-0.1231	0.08157	0.99372	0.88005	-0.18435	0.074469	0.99882	0.88764	-0.17195	0.12438	0.99987	0.94463	-0.08218	0.013193	0.95986	0.8684	-0.20357	0.1666	0.99796	0.93149	-0.10239	0.98128	0.99817	0.99977	-0.00034	0.14629	0.9981	0.91804	-0.12338	0.000404	0.42385	0.83506	-0.26005
117.0742	0.59	domics Pos	0.77713	0.99995	1.0297	0.042277	0.9638	0.99979	0.99422	-0.00836	0.72117	0.99601	0.89783	-0.15548	0.6411	0.99996	1.0679	0.094783	0.61471	0.99701	1.2084	0.27311	0.17482	0.99959	1.3321	0.41368	0.015902	0.17398	2.1915	1.1319	0.83321	0.99984	0.92489	-0.11264	0.98574	0.99966	0.95214	-0.07076
188.6361	1.91	domics Pos	0.052448	0.99995	0.86324	-0.21217	0.056582	0.99653	0.7633	-0.38968	0.008508	0.2015	0.83228	-0.26485	0.6177	0.99996	0.95434	-0.06742	0.023829	0.5516	0.8373	-0.25618	0.33958	0.99959	0.85932	-0.21872	0.02243	0.18641	1.3057	0.38478	0.22129	0.99984	0.79525	-0.33052	0.90727	0.99966	0.99386	-0.00889
539.9655	0.52	domics Pos	0.81645	0.99995	0.94327	-0.08425	0.83245	0.99979	0.94984	-0.07424	0.88996	0.99702	0.95747	-0.06269	0.57681	0.99996	1.098	0.13486	0.71315	0.99701	1.0661	0.092384	0.73769	0.99959	1.0417	0.058922	0.048621	0.2237	1.5487	0.63109	0.99676	0.99984	1.0002	0.000228	0.89599	0.99966	0.95131	-0.07201
1144.777	10.02	domics Pos	0.38379	0.99995	1.0792	0.10991	0.20458	0.99979	1.0421	0.059553	0.13009	0.73262	0.94731	-0.07809	0.90065	0.99996	1.035	0.049573	0.48634	0.99701	0.94813	-0.07685	0.68108	0.99959	1.0325	0.046131	0.043533	0.21586	1.3041	0.38304	0.8408	0.99984	1.0301	0.042851	0.33902	0.99966	1.072	0.1003
277.9254	10.15	domics Pos	0.79819	0.99995	1.0231	0.032901	0.64388	0.99979	0.96193	-0.056	0.76487	0.99601	1.0323	0.045807	0.3812	0.99996	1.0813	0.1128	0.7599	0.99701	1.0419	0.059211	0.3815	0.99959	0.92038	-0.1197	0.017464	0.17663	1.2999	0.37843	0.52867	0.99984	1.0616	0.086188	0.004919	0.99749	1.2956	0.37367
272.8256	0.57	lomics Neg	0.40208	0.99486	1.1013	0.13916	0.49845	0.99506	1.0623	0.087253	0.015545	0.6372	1.2748	0.3503	0.090932	0.80547	1.2042	0.26806	0.035824	0.39567	1.187	0.24734	0.12922	0.89898	1.1302	0.17659	0.51711	0.99905	1.0558	0.078294	0.13946	0.96955	1.1254	0.17044	0.37866	0.74722	1.1079	0.14787
760.8173	0.28	olomics Pc	0.72173	0.99964	0.98155	-0.02687	0.044725	0.88953	1.1074	0.14717	0.061222	0.99882	0.90887	-0.13785	0.36165	0.99987	0.91683	-0.12528	0.015433	0.95986	0.89561	-0.15906	0.73128	0.99796	0.97819	-0.03181	0.94943	0.99768	1.0149	0.021393	0.25028	0.9981	0.96517	-0.05115	0.65794	0.99995	1.0227	0.032438
455.3876	9.75	domics Pos	0.23785	0.99995	1.1006	0.13824	0.23362	0.99979	1.0895	0.12373	0.22087	0.83453	1.0884	0.12215	0.20698	0.99996	0.90989	-0.13623	0.48528	0.99701	1.1031	0.14158	0.45503	0.99959	0.93951	-0.09003	0.007099	0.13972	1.4867	0.57209	0.78057	0.99984	0.98134	-0.02718	0.13905	0.99966	1.1751	0.23282
996.6731	0.56	domics Pos	0.90619	0.99995	0.99571	-0.0062	0.42141	0.99979	0.93661	-0.09448	0.55879	0.99601	0.95686	-0.06362	0.67269	0.99996	0.96421	-0.05258	0.116	0.8579	0.91769	-0.12392	0.02024	0.99959	0.86004	-0.21753	0.47772	0.68525	0.9259	-0.11107	0.9229	0.99984	1.0248	0.035298	0.15606	0.99966	1.1432	0.19303
346.0276	0.57	domics Pos	0.79354	0.99995	0.97076	-0.04281	0.24366	0.99979	0.87357	-0.195	0.83603	0.99702	0.98546	-0.02114	0.33434	0.99996	1.3392	0.42132	0.085245	0.8126	1.4869	0.57231	0.15894	0.99959	1.2041	0.26791	0.003876	0.12235	1.7324	0.79276	0.6402	0.99984	0.94406	-0.08304	0.20672	0.99966	0.84751	-0.23869
678.4691	2.38	domics Pos	0.097559	0.99995	0.40841	-1.2919	0.38554	0.99979	0.61018	-0.71269	0.59764	0.99601	0.70053	-0.51348	0.68596	0.99996	0.76913	-0.37869	0.038719	0.63362	0.30961	-1.6915	0.44197	0.99959	0.64014	-0.64354	0.14641	0.35512	0.47331	-1.0791	0.21116	0.99984	0.50553	-0.98413	0.40222	0.99966	0.61887	-0.69228
443.9784	12.01	olomics Ne	0.14537	0.9951	1.2119	0.27732	0.13173	0.99975	1.2032	0.26686	0.036421	0.99731	1.1441	0.19418	0.58282	0.99915	1.09	0.12431	0.001688	0.24494	1.4019	0.48743	0.037439	0.4612	1.1435	0.1935	0.001439	0.041313	1.5439	0.62654	0.00787	0.99481	1.3218	0.40248	0.86829	0.99168	1.0302	0.042865
546.832	10.1	domics Pos	0.1225	0.99995	1.1443	0.1945	0.9147	0.99979	1.0172	0.024592	0.92336	0.99702	0.99306	-0.01005	0.63646	0.99996	1.0556	0.078085	0.49409	0.99701	1.073	0.10161	0.71183	0.99959	1.0336	0.047676	0.001292	0.089845	1.344	0.42659	0.63789	0.99984	1.0756	0.10513	0.29915	0.99966	1.1117	0.15278
811.6655	10.03	olomics Pc	0.10968	0.99964	1.1987	0.26152	0.021673	0.88953	1.1855	0.24549	0.16538	0.99882	1.119	0.16221	0.032735	0.99987	1.2911	0.36862	0.041155	0.95986	1.2857	0.36259	0.00105	0.47502	1.2126	0.27814	0.0921	0.87442	1.2134	0.27903	0.047333	0.9981	1.1552	0.20814	0.76507	0.99995	1.0831	0.1152
1112.754	9.02	olomics Pc	0.79363	0.99964	0.98979	-0.0148	0.63976	1	0.94625	-0.07971	0.21032	0.99882	0.77879	-0.36069	0.92984	0.99987	1.1267	0.17213	0.004373	0.95986	0.62043	-0.68865	0.34553	0.99796	0.86365	-0.21149	0.72885	0.99768	1.2909	0.36837	0.53763	0.9981	0.941	-0.08774	0.92496	0.99995	1.1978	0.26043
362.9842	4.29	olomics Ne	0.52108	0.9951	1.0425	0.060114	0.44958	0.99975	1.046	0.064824	0.7201	0.99731	0.98046	-0.02847	0.24931	0.99915	1.081	0.11233	0.11153	0.61361	1.109	0.1492	0.054252	0.51062	1.1455	0.19601	0.003079	0.050041	1.3176	0.39796	0.13631	0.99481	1.1058	0.14516	0.99218	0.99833	0.99921	-0.00115
189.1664	0.3	olomics Pc	0.16091	0.99964	1.2292	0.29772	0.34482	1	1.2079	0.27252	0.10015	0.99882	1.3579	0.44136	0.84642	0.99987	1.0277	0.039451	0.29231	0.95986	1.2513	0.32344	0.025049	0.99796	1.7028	0.76788	0.03125	0.64311	1.5891	0.66819	0.14118	0.9981	1.4172	0.50309	0.17563	0.99995	1.3616	0.44533
131.5346	8.29	olomics Pc	0.54795	0.99964	1.2473	0.31886	0.19511	1	1.3437	0.42618	0.59556	0.99882	0.9092	-0.13734	0.7246	0.99987	1.0568	0.079649	0.052987	0.95986	1.3332	0.41493	0.86232	0.99796	1.0667	0.093121	0.029464	0.63137	1.4379	0.52393	0.088624	0.9981	1.2808	0.35708	0.44904	0.99995	1.2117	0.27703
454.975	12	olomics Ne	0.28469	0.9951	1.1667	0.22242	0.040192	0.99975	1.2299	0.29849	0.55792	0.99731	1.0676	0.094434	0.35337	0.99915	1.2164	0.2826	0.085011	0.5958	1.1931	0.25476	0.17469	0.60765	1.2013	0.26463	0.040169	0.11162	1.3443	0.42685	0.25301	0.99481	1.1984	0.26109	0.4559	0.94605	1.0603	0.084436
808.5825	5.42	domics Pos	0.65717	0.99995	1.0386	0.054605	0.87594	0.99979	0.98949	-0.01525	0.40489	0.95675	0.95033	-0.0735	0.80159	0.99996	0.98302	-0.02471	0.62005	0.99701	0.96051	-0.05812	0.9024	0.99959	1.0107	0.015314	0.028434	0.19688	1.2678	0.3423	0.85223	0.99984	1.0155	0.022236	0.47341	0.99966	1.0847	0.11724
853.5765	8.7	olomics Ne	0.78274	0.9951	1.0292	0.041585	0.84893	0.99975	0.99349	-0.00942	0.50951	0.99731	0.96109	-0.05725	0.31116	0.99915	1.0783	0.10882	0.16097	0.64598	1.0699	0.097489	0.21751	0.6375	1.1081	0.14804	0.041586	0.11377	1.1297	0.17589	0.16461	0.99481	0.94295	-0.08475	0.21338	0.91738	0.91587	-0.12678
147.0911	0.6	domics Pos	0.76253	0.99995	0.95274	-0.06984	0.66904	0.99979	1.1054	0.14459	0.57078	0.99601	0.91763	-0.12401	0.89052	0.99996	0.99132	-0.01257	0.68698	0.99701	1.0984	0.13541	0.56972	0.99959	1.1126	0.15391	0.023961	0.18774	1.7233	0.78519	0.58014	0.99984	0.91403	-0.12969	0.99478	0.99966	0.99209	-0.01146
322.8785	10.15	domics Pos	0.8612	0.99995	0.99429	-0.00826	0.74973	0.99979	1.0154	0.022084	0.76209	0.99601	0.98436	-0.02275	0.45811	0.99996	0.93223	-0.10123	0.35848	0.99701	0.96319	-0.05411	0.81514	0.99959	0.98963	-0.01503	0.019025	0.17894	1.3138	0.39377	0.8437	0.99984	0.99273	-0.01052	0.33942	0.99966	1.0378	0.053568
818.785	0.54	domics Pos	0.52891	0.99995	0.96445	-0.05222	0.808	0.99979	0.99159	-0.01218	0.031584	0.38353	0.91208	-0.13277	0.22667	0.99996	0.90231	-0.14831	0.22986	0.99701	0.94465	-0.08215	0.64383	0.99959	0.97346	-0.0388	0.31723	0.53843	1.0507	0.071288	0.38867	0.99984	0.9218	-0.11747	0.79098	0.99966	1.0136	0.019446
240.2345	4.11	olomics Pc	0.53174	0.99964	1.1074	0.14717	0.63338	1	1.1577	0.21121	0.44723	0.99882	1.3364	0.41835	0.3173	0.99987	1.2497	0.32159	0.81362	0.99139	0.93802	-0.09232	0.15474	0.99796	1.6974	0.76335	0.80065	0.99768	0.98016	-0.02891	0.024814	0.9981	2.1958	1.1347	0.83775	0.99995	1.0272	0.038677
674.9719	12	olomics Ne	0.52712	0.9951	1.0603	0.084511	0.084079	0.99975	1.2742	0.34963	0.49303	0.99731	1.0834	0.1156	0.2903	0.99915	1.1337	0.181	0.13072	0.6311	1.222	0.28921	0.15691	0.59434	1.1532	0.20563	0.029351	0.092883	1.4108	0.49651	0.23732	0.99481	1.1594	0.21342	0.75792	0.97489	1.0559	0.078436
593.2617	1.31	domics Pos	0.83715	0.99995	0.98179	-0.02652	0.55753	0.99979	0.94231	-0.08573	0.037548	0.424	0.81595	-0.29344	0.69416	0.99996	0.96283	-0.05465	0.1449	0.90375	0.83525	-0.25972	0.39728	0.99959	1.074	0.10295	0.19133	0.40462	1.2168	0.28304	0.078022	0.99984	0.84745	-0.2388	0.69267	0.99966	1.0562	0.078817
742.5297	4.66	domics Pos	0.5478	0.99995	1.1532	0.20567	0.75302	0.99979	1.0301	0.04274	0.45626	0.99153	1.1983	0.26096	0.79014	0.99996	1.0327	0.046485	0.47753	0.99701	1.1926	0.25415	0.49127	0.99959	1.1038	0.14249	0.040957	0.20898	1.5057	0.5904	0.57548	0.99984	1.0667	0.093114	0.74832	0.99966	1.0868	0.12008
808.7889	10.1	domics Pos	0.54258	0.99995	1.062	0.086725	0.75496	0.99979	1.0231	0.0329	0.62925	0.99601	0.96769	-0.04738	0.54586	0.99996	1.0695	0.096993	0.64945	0.99701	1.0362	0.051262	0.75329	0.99959	0.97612	-0.03487	0.018627	0.17894	1.3782	0.46276	0.59742	0.99984	0.95684	-0.06364	0.7231	0.99966	1.0662	0.092415
842.7936	10.12	domics Pos	0.018909	0.75104	1.1783	0.23669	0.55043	0.99979	1.0455	0.064201	0.064621	0.55764	0.87847	-0.18694	0.46457	0.99996	1.1285	0.17445	0.88061	0.99701	1.0152	0.021784	0.68982	0.99959	0.96984	-0.04418	0.052575	0.23013	1.358	0.44146	0.61223	0.99984	1.0898	0.12406	0.60075	0.99966	1.0474	0.066868
635.8797	0.5	domics Pos	0.2898	0.99995	0.92644	-0.11024	0.347	0.99979	0.9494	-0.07491	0.022364	0.32122	0.84125	-0.24939	0.63466	0.99996	0.96238	-0.05532	0.12271	0.86273	0.89806	-0.15512	0.31586	0.99959	0.92669	-0.10984	0.093216	0.28973	1.1881	0.2486	0.51617	0.99984	0.95067	-0.07299	0.51461	0.99966	0.95963	-0.05946
722.5316	0.32	olomics Pc	0.76916	0.99964	0.98328	-0.02432	0.025455	0.88953	1.1575	0.21102	0.80089	0.99882	1.0298	0.042369	0.067339	0.99987	1.115	0.15703	0.51076	0.95986	0.93641	-0.09478	0.34347	0.99796	1.0682	0.095143	0.31748	0.99768	0.95648	-0.06419	0.086113	0.9981	1.1879	0.24842	0.034036	0.99995	1.1017	0.13967
562.9062	10.03	lomics Neg	0.40118	0.99486	1.086	0.11899	0.82555	0.99506	0.98048	-0.02844	0.079572	0.6372	1.1884	0.24897	0.17	0.80547	1.1471	0.19804	0.045694	0.39567	1.1959	0.25814	0.12516	0.89898	1.1338	0.18116	0.39898	0.99905	1.0567	0.079533	0.18292	0.96955	1.0939	0.12947	0.28282	0.74722	1.1158	0.15805
195.814	12.28	olomics Ne	0.823	0.9951	1.0056	0.008104	0.25222	0.99975	1.0376	0.053187	0.51742	0.99731	0.97943	-0.02999	0.55919	0.99915	1.0279	0.039638	0.11703	0.62181	1.0631	0.088333	0.071815	0.5223	1.0797	0.1106	0.002007	0.041875	1.2388	0.30895	0.25247	0.99481	1.0291	0.041366	0.11541	0.91738	0.94762	-0.07763
1003.762	0.53	domics Pos	0.094513	0.99995	0.92129	-0.11827	0.26079	0.99979	0.90038	-0.1514	0.070542	0.58701	0.91775	-0.12382	0.21728	0.99996	0.90205	-0.14872	0.44366	0.99701	0.95646	-0.06423	0.16422	0.99959	0.90344	-0.1465	0.025017	0.18884	1.1296	0.17582	0.33435	0.99984	0.91667	-0.12552	0.60733	0.99966	0.9676	-0.04751
1180.483	6.02	domics Pos	0.60043	0.99995	0.93357	-0.09917	0.7013	0.99979	0.95243	-0.07031	0.055584	0.51947	0.68976	-0.53584	0.96515	0.99996	0.99174	-0.01197	0.038576	0.63362	0.7305	-0.45305	0.67236	0.99959	1.0643	0.089839	0.0765	0.26501	1.3202	0.40077	0.77054	0.99984	0.96763	-0.04748	0.89409	0.99966	0.99337	-0.0096
312.3263	3.26	domics Pos	0.52423	0.99995	1.1368	0.18502	0.029203	0.99441	1.5227	0.60662	0.66357	0.99601	1.0685	0.095524	0.24884	0.99996	1.2612	0.33484	0.56817	0.99701	0.90803	-0.13918	0.22671	0.99959	1.4509	0.53699	0.22562	0.44643	1.3652	0.44909	0.59708	0.99984	1.1339	0.18128	0.032619	0.99966	1.441	0.52705
645.1856	1.29	domics Pos	0.6974	0.99995	0.97215	-0.04075	0.57141	0.99979	0.96267	-0.05488	0.31602	0.90473	0.91935	-0.12132	0.70167	0.99996	0.97564	-0.03557	0.018157	0.50167	0.86024	-0.21718	0.084421	0.99959	0.84452	-0.2438	0.040208	0.20746	1.2318	0.3008	0.5935	0.99984	0.9632	-0.05409	0.84049	0.99966	1.0424	0.059891
867.8188	10.02	domics Pos	0.90011	0.99995	0.98829	-0.01699	0.40393	0.99979	1.1099	0.15044	0.70872	0.99601	0.96452	-0.05212	0.14914	0.99996	0.86918	-0.20227	0.98516	0.99701	0.99917	-0.00119	0.96215	0.99959	0.99726	-0.00396	0.049685	0.22586	1.2722	0.34727	0.93638	0.99984	1.0131	0.018767	0.49031	0.99966	1.0853	0.11812
1122.313	8.39	olomics Pc	0.70119	0.99964	1.0414	0.058539	0.30938	1	1.1027	0.14105	0.58735	0.99882	0.95447	-0.06723	0.3813	0.99987	1.1318	0.17866	0.056408	0.95986	1.2429	0.31376	0.64548	0.99796	1.112	0.15318	0.011993	0.42929	1.2568	0.32972	0.53697	0.9981	1.0556	0.078011	0.42536	0.99995	1.0758	0.10542
402.7706	4.86	domics Pos	0.52053	0.99995	1.1401	0.18917	0.46095	0.99979	0.94353	-0.08386	0.64625	0.99601	0.96553	-0.05061	0.75787	0.99996	0.97327	-0.03908	0.41173	0.99701	0.94107	-0.08763	0.37634	0.99959	1.245	0.3162	0.00377	0.12181	1.4125	0.49825	0.80286	0.99984	0.99496	-0.00729	0.073571	0.99966	1.2004	0.26349
119.9164	10.64	olomics Ne	0.34725	0.9951	1.088	0.12172	0.3876	0.99975	1.1028	0.14117	0.73408	0.99731	1.0419	0.059163	0.82832	0.99915	0.98678	-0.0192	0.37335	0.75206	1.0598	0.083744	0.52289	0.80259	1.0935	0.12897	0.03601	0.10421	1.3223	0.40307	0.36063	0.99481	1.046	0.064937	0.28022	0.91738	0.92129	-0.11828
332.3284	4.98	olomics Pc	0.047614	0.99964	2.3886	1.2562	0.23761	1	1.6332	0.70772	0.14497	0.99882	6.0248	2.5909	0.16565	0.99987	2.2168	1.1485	0.21622	0.95986	1.2324	0.3015	0.004041	0.97916	1.6147	0.69126	0.67211	0.99768	1.2478	0.31941	0.003391	0.9981	1.8301	0.87188	0.003167	0.80366	1.7222	0.78423
140.0687	0.31	olomics Pc	0.6311	0.99964	0.94702	-0.07854	0.88818	1	1.0349	0.049433	0.51551	0.99882	0.92108	-0.11861	0.62102	0.99987	0.94279	-0.08499	0.85756	0.99867	1.0003	0.00049	0.029707	0.99796	1.4288	0.51482	0.036556	0.66991	1.4065	0.49211	0.81927	0.9981	0.97824	-0.03175	0.14283	0.99995	1.1899	0.25082
173.871	12.71	olomics Ne	0.74209	0.9951	1.0198	0.028253	0.78731	0.99975	1.0188	0.026922	0.46253	0.99731	0.9761	-0.0349	0.75409	0.99915	1.0203	0.028949	0.13191	0.6311	1.0817	0.11329	0.48853	0.79937	1.0443	0.062479	0.007199	0.057477	1.212	0.27743	0.88955	0.99481	0.99605	-0.00572	0.074414	0.91738	0.94189	-0.08637
338.7965	0.36	olomics Ne	0.010822	0.9951	1.2506	0.32262	0.046219	0.99975	1.2548	0.32746	0.50017	0.99731	1.0962	0.13256	0.21526	0.99915	1.1934	0.25511	0.15848	0.64386	1.1751	0.23275	0.10183	0.53216	1.2635	0.33746	0.007687	0.057477	1.2811	0.35741	0.026319	0.99481	1.2162	0.28241	0.73896	0.97489	1.0446	0.062958
787.6674	6.04	domics Pos	0.33328	0.99995	1.2606	0.33416	0.42997	0.99979	0.80919	-0.30545	0.418	0.96903	1.2166	0.28291	0.64449	0.99996	1.041	0.057989	0.8324	0.99701	1.1097	0.15022	0.29257	0.99959	0.74118	-0.4321	0.048151	0.22336	1.6131	0.68985	0.56841	0.99984	1.1115	0.15252	0.35322	0.99966	1.2712	0.34619
449.9791	12	olomics Ne	0.28939	0.9951	1.1967	0.25909	0.016294	0.99975	1.3226	0.40335	0.022215	0.99731	1.4916	0.57691	0.10782	0.99915	1.2368	0.30665	0.024033	0.50209	1.2542	0.32679	0.016486	0.45618	1.6111	0.688	0.001885	0.04149	1.8505	0.88791	0.049248	0.99481	1.3366	0.41853	0.20747	0.91738	1.3038	0.38276
393.9806	12	olomics Ne	0.15415	0.9951	1.1783	0.23674	0.40929	0.99975	1.1157	0.15799	0.8434	0.99731	1.0556	0.078093	0.17391	0.99915	1.3067	0.38598	0.11315	0.61361	1.1447	0.19501	0.12384	0.54162	1.3533	0.43644	0.021338	0.077635	1.3769	0.46141	0.65366	0.99481	1.118	0.16089	0.85745	0.99168	0.99797	-0.00293
687.9768	12.03	olomics Ne	0.94153	0.9951	1.0117	0.016733	0.28672	0.99975	1.222	0.28924	0.39678	0.99731	0.88385	-0.17812	0.50949	0.99915	0.90667	-0.14136	0.81964	0.95457	0.97931	-0.03016	0.72352	0.90976	0.95067	-0.07298	0.56265	0.65342	0.92105	-0.11864	0.010083	0.99481	0.66744	-0.5833	0.92117	0.99833	1.1515	0.2035
854.3746	0.33	olomics Ne	0.023277	0.9951	1.2181	0.28461	0.055414	0.99975	1.1857	0.24579	0.42068	0.99731	1.0799	0.11085	0.19314	0.99915	1.2168	0.28312	0.1372	0.64226	1.1968	0.25923	0.11501	0.5383	1.1699	0.22632	0.29667	0.41358	1.1703	0.2269	0.014771	0.99481	1.3149	0.39496	0.026957	0.91738	1.188	0.24856
95.0868	6.98	olomics Pc	0.65163	0.99964	1.1558	0.20892	0.27319	1	0.92806	-0.10772	0.085199	0.99882	1.1532	0.20563	0.61125	0.99987	0.94987	-0.0742	0.17214	0.95986	1.1778	0.2361	0.04868	0.99796	0.66156	-0.59606	0.88271	0.99768	0.99218	-0.01132	0.89409	0.9981	1.0939	0.12948	0.38727	0.99995	0.90153	-0.14956
1035.766	10.05	domics Pos	0.40595	0.99995	0.92751	-0.10857	0.19622	0.99979	1.1146	0.15658	0.43704	0.98176	0.93074	-0.10355	0.74967	0.99996	1.0277	0.03938	0.03286	0.59375	0.81964	-0.28694	0.58222	0.99959	0.95191	-0.0711	0.43408	0.64959	1.0892	0.12327	0.90304	0.99984	1.01	0.014324	0.8234	0.99966	0.97829	-0.03166
1152.771	10.02	domics Pos	0.001992	0.59581	1.289	0.36622	0.018788	0.99441	1.142	0.19159	0.002056	0.13479	1.154	0.20659	0.30786	0.99996	1.0456	0.064355	0.052889	0.71735	1.1781	0.23641	0.30826	0.99959	1.1006	0.13824	0.002206	0.10737	1.5014	0.58634	0.17601	0.99984	1.1716	0.22847	0.11045	0.99966	1.1958	0.25803
174.9459	10.11	domics Pos	0.087091	0.99995	1.2305	0.29924	0.70327	0.99979	1.106	0.14539	0.36522	0.93332	1.0442	0.062465	0.056486	0.99996	1.0864	0.11959	0.93908	0.99701	1.0166	0.023821	0.41223	0.99959	1.0397	0.056177	0.037291	0.20439	1.2319	0.30089	0.28461	0.99984	1.1036	0.14218	0.18423	0.99966	1.2792	0.35519
307.9909	12.01	olomics Ne	0.9048	0.9951	1.0364	0.051598	0.42357	0.99975	1.1934	0.25512	0.71467	0.99731	1.1072	0.14686	0.77467	0.99915	0.96762	-0.04748	0.52124	0.84986	1.1098	0.1503	0.50406	0.79937	1.1913	0.25249	0.004025	0.050041	1.4442	0.53025	0.62623	0.99481	1.064	0.089444	0.67475	0.96789	0.94849	-0.0763
600.4666	5.87	olomics Pc	0.71193	0.99964	0.96254	-0.05508	0.47246	1	0.93045	-0.10399	0.75679	0.99882	0.96971	-0.04438	0.89715	0.99987	1.0414	0.058476	0.20684	0.95986	0.88695	-0.17308	0.92048	0.99796	1.0326	0.046238	0.26402	0.99768	0.89599	-0.15845	0.040671	0.9981	0.8223	-0.28226	0.1882	0.99995	0.81434	-0.29629
1178.773	10.02	domics Pos	0.21304	0.99995	1.104	0.14268	0.36326	0.99979	1.0483	0.068047	0.93311	0.99702	0.99737	-0.0038	0.78641	0.99996	1.0271	0.038647	0.6096	0.99701	1.0339	0.048049	0.83637	0.99959	1.0163	0.023305	0.030877	0.19986	1.2781	0.35401	0.59989	0.99984	1.0395	0.055837	0.13639	0.99966	1.1161	0.15851
802.6858	6.23	domics Pos	0.4546	0.99995	1.2669	0.34134	0.53902	0.99979	0.86953	-0.20168	0.45013	0.99046	1.2765	0.35217	0.70816	0.99996	1.0948	0.13067	0.75737	0.99701	1.0781	0.10847	0.2462	0.99959	0.78412	-0.35086	0.034044	0.20254	1.6944	0.76076	0.46521	0.99984	1.2254	0.29325	0.16259	0.99966	1.2433	0.31413
485.985	0.53	domics Pos	0.63572	0.99995	0.95101	-0.07246	0.19291	0.99979	0.87004	-0.20085	0.68384	0.99601	0.95677	-0.06376	0.56632	0.99996	1.0814	0.11295	0.9084	0.99701	0.98954	-0.01518	0.48766	0.99959	1.0896	0.12375	0.015031	0.17068	1.4693	0.55512	0.69407	0.99984	1.0702	0.097899	0.59566	0.99966	0.95039	-0.07341
745.8605	10.05	domics Pos	0.066701	0.99995	1.2169	0.28325	0.073332	0.99979	1.1099	0.15041	0.088036	0.65124	1.1135	0.15508	0.78461	0.99996	1.0245	0.03491	0.21712	0.99598	1.1408	0.19002	0.3749	0.99959	1.1071	0.14677	0.043244	0.21498	1.4344	0.52046	0.50541	0.99984	1.1089	0.14909	0.70111	0.99966	1.0864	0.11961
240.9463	10.14	domics Pos	0.21041	0.99995	0.94388	-0.08332	0.65914	0.99979	0.96881	-0.04571	0.00628	0.17872	0.78166	-0.35538	0.77009	0.99996	1.0449	0.063332	0.11116	0.85684	0.86858	-0.20328	0.64298	0.99959	1.0284	0.040457	0.12365	0.32449	1.3889	0.47395	0.47548	0.99984	0.91828	-0.123	0.79192	0.99966	0.98707	-0.01877
197.8106	12.27	olomics Ne	0.63623	0.9951	1.0145	0.020835	0.24305	0.99975	1.0492	0.069268	0.62034	0.99731	0.98082	-0.02794	0.53264	0.99915	1.0319	0.045357	0.053887	0.58152	1.0773	0.10743	0.12516	0.54162	1.071	0.09895	0.001739	0.041313	1.2441	0.31512	0.27437	0.99481	1.0348	0.049331	0.30818	0.91738	0.96805	-0.04685
534.9747	5.26	olomics Ne	0.91744	0.9951	0.99636	-0.00526	0.13114	0.99975	1.0722	0.10051	0.72679	0.99731	1.0126	0.018046	0.11584	0.99915	1.0708	0.098695	0.030277	0.51873	1.1532	0.20559	0.036632	0.4612	1.1621	0.21677	0.011675	0.062031	1.2383	0.30831	0.066879	0.99481	1.0766	0.10648	0.80323	0.97489	0.98762	-0.01797
553.9663	0.52	domics Pos	0.80922	0.99995	0.98472	-0.02221	0.5091	0.99979	0.95291	-0.06958	0.4764	0.99601	0.94467	-0.08211	0.63042	0.99996	1.0763	0.10613	0.83141	0.99701	0.98501	-0.02178	0.41103	0.99959	1.1169	0.15955	0.026924	0.19234	1.4596	0.5456	0.96241	0.99984	1.0147	0.021034	0.19556	0.99966	1.1132	0.15477
650.8487	0.5	domics Pos	0.9829	0.99995	1.0182	0.026027	0.23107	0.99979	1.142	0.19155	0.84047	0.99702	0.98629	-0.01992	0.74312	0.99996	1.048	0.067589	0.46091	0.99701	1.0839	0.11628	0.3807	0.99959	1.1033	0.14178	0.037994	0.20439	1.2873	0.36438	0.84888	0.99984	1.0249	0.035494	0.21174	0.99966	1.1664	0.22211
151.0421	0.31	olomics Pc	0.69056	0.99964	1.0323	0.045886	0.52009	1	1.0647	0.090437	0.17787	0.99882	1.097	0.13353	0.292	0.99987	1.119	0.16221	0.02703	0.95986	1.1684	0.22457	0.35368	0.99796	1.1167	0.15926	0.13471	0.95284	1.2856	0.3624	0.87397	0.9981	1.0257	0.036635	0.69108	0.99995	0.97495	-0.03661
414.3011	4.19	olomics Pc	0.11721	0.99964	6.2909	2.6533	0.047323	0.88953	4.7752	2.2556	0.10929	0.99882	10.919	3.4487	0.001526	0.99987	11.869	3.5691	0.001509	0.95986	54.073	5.7568	0.001012	0.47502	28.797	4.8478	0.000106	0.09364	41.574	5.3776	0.09281	0.9981	5.5446	2.4711	0.12335	0.99995	4.7618	2.2515
226.9524	0.5	domics Pos	0.49046	0.99995	1.0386	0.054581	0.98211	0.99979	1.003	0.004258	0.057959	0.53253	0.93786	-0.09256	0.66923	0.99996	0.97878	-0.03095	0.33433	0.99701	0.96384	-0.05313	0.77837	0.99959	0.98751	-0.01813	0.025209	0.18884	1.188	0.24856	0.91099	0.99984	0.99953	-0.00068	0.67619	0.99966	1.0329	0.04675
840.5869	5.12	domics Pos	0.52771	0.99995	1.0861	0.11911	0.85245	0.99979	1.0213	0.030431	0.79303	0.99601	0.96712	-0.04823	0.79389	0.99996	1.0648	0.090605	0.96061	0.99701	1.0052	0.007486	0.50558	0.99959	1.1017	0.13972	0.044988	0.21733	1.3378	0.41989	0.59613	0.99984	1.0983	0.1353	0.20083	0.99966	1.2047	0.26869
228.991	12.03	olomics Ne	0.57428	0.9951	1.0402	0.056906	0.77398	0.99975	0.97332	-0.03901	0.073678	0.99731	0.79038	-0.33938	0.44243	0.99915	1.0583	0.081707	0.88869	0.97133	1.0102	0.014688	0.014193	0.45618	0.70105	-0.5124	0.47257	0.58009	1.0638	0.089291	0.082279	0.99481	0.81247	-0.29961	0.017935	0.91738	0.78952	-0.34095
231.9844	3.31	olomics Ne	0.22715	0.9951	1.1862	0.24638	0.056147	0.99975	1.1915	0.25273	0.78006	0.99731	1.0172	0.024602	0.13286	0.99915	1.1509	0.20275	0.14285	0.64314	1.2457	0.31699	0.021109	0.4612	1.321	0.40165	0.001124	0.041313	1.2222	0.28942	0.048678	0.99481	1.2953	0.37334	0.39269	0.92784	1.2274	0.29566
672.5617	6.29	domics Pos	0.73192	0.99995	1.1526	0.20494	0.39514	0.99979	1.2195	0.28626	0.77603	0.99601	0.94444	-0.08246	0.68051	0.99996	1.1048	0.14383	0.17902	0.95949	1.7189	0.78147	0.045348	0.99959	2.1057	1.0743	0.007219	0.13972	3.0336	1.601	0.1467	0.99984	1.7715	0.82498	0.070106	0.99966	1.7582	0.81412
626.8457	10.05	domics Pos	0.14136	0.99995	1.0939	0.12948	0.37836	0.99979	1.0922	0.12725	0.93508	0.99702	1.0033	0.004803	0.39028	0.99996	1.0746	0.10381	0.59952	0.99701	1.0213	0.030346	0.75063	0.99959	0.98578	-0.02067	0.012185	0.16313	1.2988	0.37722	0.65147	0.99984	1.0449	0.063316	0.071842	0.99966	1.1492	0.20057
976.9744	12.03	olomics Ne	0.95158	0.9951	0.99988	-0.00017	0.03249	0.99975	1.4639	0.54978	0.34476	0.99731	1.098	0.13492	0.17581	0.99915	1.1493	0.2008	0.065883	0.5958	1.3645	0.44833	0.013558	0.45618	1.3649	0.44877	0.057331	0.13784	1.2628	0.33665	0.47382	0.99481	1.136	0.18391	0.52739	0.96069	1.2041	0.26799
291.0708	0.6	domics Pos	0.74793	0.99995	1.0843	0.11678	0.87121	0.99979	1.1169	0.15944	0.32252	0.91107	0.80917	-0.30548	0.081372	0.99996	0.70721	-0.49979	0.13624	0.87878	0.74423	-0.42618	0.022642	0.99959	0.68509	-0.54563	0.2481	0.46731	0.81637	-0.2927	0.44673	0.99984	1.2192	0.28593	0.17963	0.99966	1.1872	0.24756
1189.636	6.78	olomics Pc	0.22962	0.99964	1.2731	0.34834	0.41299	1	1.1304	0.17678	0.018652	0.63482	1.5313	0.61474	0.69347	0.99987	1.2409	0.31137	0.56913	0.95986	1.1603	0.21444	0.75082	0.99796	1.133	0.1801	0.12727	0.94474	1.4971	0.58214	0.15109	0.9981	1.6028	0.68055	0.48031	0.99995	1.5304	0.61388

592.4694	3.68	domics Pos	0.87828	0.99995	0.98339	-0.02417	0.37018	0.99979	0.91831	-0.12294	0.59596	0.99601	0.9424	-0.0856	0.72385	0.99996	1.0294	0.0418	0.043639	0.66031	0.7543	-0.40679	0.15083	0.99959	0.86815	-0.20398	0.15342	0.36154	1.3503	0.4333	0.4218	0.99984	0.92397	-0.11408	0.23564	0.99966	1.1424	0.19209
954.3449	0.32	olomics Pc	0.32427	0.99964	0.91054	-0.13521	0.19658	1	1.1105	0.1512	0.24046	0.99882	0.89065	-0.16707	0.26753	0.99987	0.90427	-0.14517	0.55399	0.95986	0.93858	-0.09145	0.83224	0.99796	0.98023	-0.02881	0.011324	0.42006	0.746	-0.42275	0.53959	0.9981	0.94657	-0.07922	0.95057	0.99995	0.99453	-0.00791
377.1706	2.29	domics Pos	0.085476	0.99995	1.2398	0.3101	0.14066	0.99979	1.1626	0.21736	0.67036	0.99601	1.0551	0.077398	0.54998	0.99996	1.2032	0.26682	0.051348	0.70556	1.3134	0.39332	0.68711	0.99959	0.9514	-0.07188	0.046751	0.22017	1.3611	0.44478	0.35494	0.99984	1.1455	0.196	0.7847	0.99966	1.0553	0.077604
278.9872	3.74	olomics Ne	0.3744	0.9951	1.0571	0.080139	0.68412	0.99975	0.9753	-0.03608	0.43132	0.99731	0.95161	-0.07155	0.24697	0.99915	1.0602	0.08437	0.07164	0.5958	1.0937	0.12925	0.065648	0.5223	1.1323	0.17924	0.015715	0.067031	1.2446	0.31569	0.43665	0.99481	1.0473	0.066642	0.4022	0.93948	0.94134	-0.08721
118.1241	11.8	olomics Pc	0.40073	0.99964	0.94114	-0.08751	0.42863	1	0.94445	-0.08246	0.15498	0.99882	0.89372	-0.16211	0.50537	0.99987	0.94529	-0.08118	0.98915	0.99906	0.99829	-0.00246	0.79012	0.99796	0.97755	-0.03276	0.046608	0.74906	1.1942	0.25609	0.95796	0.9981	0.99582	-0.00604	0.92309	0.99995	0.99269	-0.01059
580.292	1.3	domics Pos	0.90198	0.99995	0.9903	-0.01406	0.22198	0.99979	1.1122	0.15342	0.15168	0.77422	0.86434	-0.21033	0.47201	0.99996	1.0976	0.13437	0.48258	0.99701	0.93925	-0.09042	0.79454	0.99959	1.0285	0.040528	0.036224	0.20439	1.5117	0.59615	0.89312	0.99984	0.99076	-0.01339	0.27674	0.99966	1.088	0.12167
326.7684	0.33	olomics Ne	0.52388	0.9951	1.0473	0.066617	0.31303	0.99975	1.1077	0.14762	0.67195	0.99731	1.0457	0.064482	0.25563	0.99915	1.1378	0.18623	0.012226	0.50209	1.2579	0.33107	0.45368	0.78329	1.0703	0.097987	0.098827	0.18716	1.218	0.28453	0.070545	0.99481	1.1755	0.23333	0.51554	0.95528	1.0417	0.058883
277.2169	4.95	olomics Pc	0.17814	0.99964	1.7385	0.7978	0.31028	1	1.7825	0.83387	0.12254	0.99882	2.1859	1.1282	0.047989	0.99987	3.1825	1.6701	0.063693	0.95986	4.5935	2.1996	0.07217	0.99796	2.8031	1.487	0.001881	0.28211	3.8809	1.9564	0.21323	0.9981	2.3872	1.2553	0.083224	0.99995	1.5887	0.66789
141.9853	12.15	olomics Pc	0.67182	0.99964	1.0141	0.020191	0.60672	1	1.0193	0.027599	0.18304	0.99882	0.95383	-0.06819	0.85651	0.99987	0.99512	-0.00706	0.41475	0.95986	1.0227	0.032387	0.95167	0.99796	0.9986	-0.00202	0.022083	0.56957	1.1092	0.14957	0.86205	0.9981	0.99463	-0.00776	0.6798	0.99995	1.0108	0.015486
262.9157	10.15	domics Pos	0.58122	0.99995	1.0473	0.066735	0.2677	0.99979	1.0748	0.10404	0.73283	0.99601	0.97374	-0.03839	0.14353	0.99996	1.1502	0.20184	0.73992	0.99701	1.0208	0.029733	0.53778	0.99959	1.0729	0.10158	0.003396	0.11893	1.3793	0.46392	0.94517	0.99984	1.0238	0.033874	0.19082	0.99966	1.0836	0.11589
203.1544	8.38	olomics Pc	0.2727	0.99964	1.0938	0.12937	0.005908	0.88953	0.7704	-0.37633	0.37412	0.99882	0.9425	-0.08543	0.88267	0.99987	1.0003	0.000484	0.52778	0.95986	1.1957	0.25781	0.44329	0.99796	0.94215	-0.08597	0.64003	0.99768	1.0661	0.092321	0.67905	0.9981	1.0805	0.11172	0.038654	0.99995	0.74278	-0.42899
291.0694	0.35	olomics Pc	0.87678	0.99964	1.0391	0.055325	0.83308	1	1.1111	0.15199	0.3144	0.99882	0.80816	-0.30729	0.028939	0.99987	0.7154	-0.48318	0.16905	0.95986	0.77523	-0.3673	0.07829	0.99796	0.71635	-0.48127	0.042715	0.70447	0.69087	-0.53352	0.42355	0.9981	1.1571	0.21053	0.2073	0.99995	1.2078	0.2724
822.777	0.56	domics Pos	0.82848	0.99995	0.98304	-0.02469	0.91622	0.99979	0.99651	-0.00505	0.13283	0.7364	0.89367	-0.16218	0.5005	0.99996	0.94388	-0.08332	0.035776	0.62141	0.80811	-0.30738	0.53788	0.99959	0.95384	-0.06818	0.46471	0.67729	1.0799	0.11095	0.12618	0.99984	0.84791	-0.23802	0.66797	0.99966	1.0347	0.049263
694.4419	4.52	domics Pos	0.32449	0.99995	1.1723	0.22935	0.66258	0.99979	0.95053	-0.07319	0.23634	0.84537	0.84996	-0.23454	0.84614	0.99996	1.2101	0.27512	0.1364	0.87878	0.71474	-0.48451	0.046895	0.99959	0.78638	-0.3467	0.37594	0.59777	1.6106	0.68764	0.52891	0.99984	1.0829	0.11487	0.84035	0.99966	1.2245	0.29221
762.5996	5.67	domics Pos	0.68503	0.99995	1.0637	0.089037	0.88092	0.99979	0.97759	-0.03271	0.79221	0.99601	0.9673	-0.04796	0.80107	0.99996	0.97079	-0.04277	0.99692	0.99846	1.008	0.011482	0.67737	0.99959	0.94957	-0.07465	0.032777	0.20029	1.3105	0.3901	0.43729	0.99984	1.0863	0.11943	0.068633	0.99966	1.2543	0.32684
158.9653	0.51	domics Pos	0.67507	0.99995	1.0221	0.031599	0.91713	0.99979	0.99805	-0.00281	0.031959	0.38456	0.9324	-0.10098	0.62019	0.99996	0.97282	-0.03976	0.2018	0.97563	0.95329	-0.06901	0.62959	0.99959	0.9766	-0.03417	0.029145	0.19804	1.1798	0.23855	0.78434	0.99984	0.98696	-0.01894	0.75772	0.99966	1.0219	0.031239
552.2739	10.05	olomics Pc	0.65547	0.99964	1.0375	0.053135	0.88031	1	1.0109	0.015594	0.95882	0.99882	1.0108	0.015514	0.12293	0.99987	1.0724	0.10082	0.57152	0.95986	0.97346	-0.03881	0.41949	0.99796	1.0552	0.07747	0.024533	0.57962	1.2006	0.26374	0.30262	0.9981	1.1033	0.14177	0.035208	0.99995	1.2594	0.33274
690.9741	12	olomics Ne	0.6407	0.9951	0.971	-0.04246	0.48632	0.99975	0.94308	-0.08455	0.34926	0.99731	0.91715	-0.12477	0.87977	0.99915	0.99284	-0.01037	0.018271	0.50209	0.83746	-0.2559	0.5037	0.79937	1.0725	0.101	0.010653	0.061656	1.2141	0.27983	0.52471	0.99481	1.0646	0.090255	0.32134	0.91738	0.91544	-0.12747
290.9046	10.16	domics Pos	0.10593	0.99995	0.90078	-0.15075	0.16421	0.99979	0.90957	-0.13675	0.009658	0.21409	0.8577	-0.22146	0.94664	0.99996	1.0276	0.039273	0.048927	0.70135	0.8648	-0.20957	0.41049	0.99959	0.96005	-0.05882	0.31573	0.53721	1.167	0.22284	0.50589	0.99984	0.95221	-0.07065	0.86612	0.99966	1.0187	0.026763
1132.815	11.96	olomics Ne	0.5147	0.9951	0.96503	-0.05136	0.20647	0.99975	1.0475	0.066894	0.67165	0.99731	0.9817	-0.02664	0.52498	0.99915	1.0397	0.056216	0.50909	0.84755	1.041	0.057974	0.5568	0.81988	1.0582	0.081556	0.00337	0.050041	1.2174	0.28386	0.5832	0.99481	0.97076	-0.04282	0.039365	0.91738	0.90214	-0.14858
823.6642	6.23	domics Pos	0.31836	0.99995	1.3625	0.44624	0.66738	0.99979	0.9077	-0.13972	0.33474	0.92155	1.3261	0.40718	0.67241	0.99996	1.1277	0.17341	0.81359	0.99701	1.061	0.085384	0.2827	0.99959	0.79457	-0.33176	0.035786	0.20439	1.7181	0.78086	0.48868	0.99984	1.2452	0.31643	0.18553	0.99966	1.2612	0.33475
200.6368	6.01	olomics Pc	0.002849	0.38271	0.81288	-0.29888	0.035094	0.88953	0.77067	-0.37582	0.16243	0.99882	0.89167	-0.16542	0.49267	0.99987	0.94426	-0.08275	0.163	0.95986	0.89553	-0.15918	0.16032	0.99796	0.81433	-0.29631	0.80133	0.99768	1.0188	0.026898	0.10344	0.9981	0.81935	-0.28745	0.11834	0.99995	0.91427	-0.12931
442.6794	0.57	lomics Neg	0.257	0.99486	1.1785	0.23694	0.27598	0.99506	1.0739	0.10284	0.0803	0.6372	1.236	0.30565	0.038242	0.80547	1.2018	0.26523	0.011096	0.39567	1.2403	0.31072	0.4608	0.89898	1.0712	0.099284	0.28028	0.99905	1.136	0.18392	0.03577	0.96955	1.2334	0.30268	0.068626	0.74722	1.2013	0.26454
531.0101	0.5	domics Pos	0.66988	0.99995	0.91702	-0.12497	0.93256	0.99979	0.9868	-0.01917	0.63768	0.99601	0.91118	-0.13419	0.9223	0.99996	1.0165	0.023566	0.73277	0.99701	1.055	0.077199	0.96693	0.99959	1.033	0.04689	0.019365	0.17969	1.9087	0.9326	0.58126	0.99984	0.89514	-0.15982	0.82342	0.99966	0.94611	-0.07992
112.1101	2.05	domics Pos	0.11087	0.99995	0.85183	-0.23136	0.021884	0.99441	0.77761	-0.36288	0.10391	0.68915	0.85594	-0.22442	0.59066	0.99996	0.94848	-0.0763	0.056929	0.74852	0.79941	-0.323	0.089226	0.99959	0.73428	-0.44561	0.08531	0.27673	1.1871	0.24739	0.098934	0.99984	0.72751	-0.45896	0.10173	0.99966	0.88085	-0.18303
548.6565	0.32	olomics Pc	0.97023	0.99964	1.0098	0.014059	0.9571	1	0.99832	-0.00243	0.85734	0.99882	1.0142	0.020292	0.70109	0.99987	0.97665	-0.03409	0.94763	0.99906	0.99836	-0.00237	0.94933	0.99796	0.99822	-0.00257	0.02224	0.56957	0.87386	-0.19453	0.035601	0.9981	1.083	0.115	0.81577	0.99995	1.0126	0.018087
757.9772	12.04	olomics Ne	0.70155	0.9951	1.0414	0.058525	0.52778	0.99975	1.078	0.1083	0.94671	0.99731	1.0241	0.034369	0.2373	0.99915	1.1057	0.14498	0.37514	0.75206	1.1066	0.14619	0.47862	0.79813	1.0968	0.13324	0.028843	0.092194	1.4739	0.55964	0.61705	0.99481	1.1072	0.14694	0.13849	0.91738	0.78994	-0.34018
928.8474	11.96	olomics Ne	0.91539	0.9951	0.99338	-0.00958	0.63251	0.99975	1.0274	0.039061	0.72746	0.99731	0.97599	-0.03506	0.41843	0.99915	1.0535	0.07519	0.3703	0.75206	1.0535	0.07516	0.43699	0.77401	1.1061	0.14552	0.019693	0.07459	1.223	0.29042	0.66719	0.99481	0.9727	-0.03994	0.11879	0.91738	0.90878	-0.138
298.3106	2.84	domics Pos	0.079108	0.99995	1.2429	0.31377	0.069137	0.99979	1.2337	0.30294	0.63507	0.99601	1.0442	0.062412	0.043591	0.99996	1.3616	0.44532	0.72194	0.99701	1.0336	0.047675	0.091724	0.99959	1.2781	0.35401	0.093663	0.28988	1.3366	0.4186	0.36843	0.99984	1.093	0.12827	0.068337	0.99966	1.2935	0.37125
1096.246	6.26	domics Pos	0.84459	0.99995	0.98693	-0.01898	0.44194	0.99979	0.85129	-0.23229	0.28796	0.89486	0.78551	-0.3483	0.8448	0.99996	1.2787	0.35469	0.048251	0.69757	0.56752	-0.81727	0.265	0.99959	0.80324	-0.31609	0.5544	0.74425	1.6788	0.7474	0.43527	0.99984	0.83618	-0.25812	0.92997	0.99966	1.1768	0.23488
217.8622	12.72	olomics Ne	0.096925	0.9951	1.0624	0.087304	0.38189	0.99975	1.0368	0.052129	0.12421	0.99731	0.96423	-0.05255	0.73821	0.99915	1.0115	0.016451	0.1451	0.64314	1.0735	0.10229	0.26029	0.66013	1.0414	0.058545	0.005532	0.052776	1.2409	0.31137	0.86278	0.99481	0.99505	-0.00717	0.071387	0.91738	0.91709	-0.12486
374.98	4.54	olomics Ne	0.50955	0.9951	1.0291	0.041387	0.26981	0.99975	1.0864	0.11955	0.57237	0.99731	0.97695	-0.03365	0.37593	0.99915	1.0488	0.068692	0.038717	0.53488	1.1038	0.14248	0.12465	0.54162	1.1091	0.14937	0.012194	0.062031	1.1991	0.26198	0.33176	0.99481	1.0509	0.071638	0.41065	0.94239	0.96119	-0.05711
595.2954	1.84	domics Pos	0.76566	0.99995	0.94915	-0.07529	0.51481	0.99979	0.89352	-0.16243	0.048238	0.47775	0.69978	-0.51502	0.76389	0.99996	0.95161	-0.07156	0.36585	0.99701	0.8385	-0.25412	0.45806	0.99959	1.0881	0.12179	0.068601	0.2547	1.2822	0.35868	0.31339	0.99984	0.83659	-0.25741	0.15925	0.99966	1.2362	0.3059
542.9721	12	olomics Ne	0.34197	0.9951	0.88554	-0.17537	0.79696	0.99975	1.0317	0.045019	0.86483	0.99731	1.0318	0.045207	0.87038	0.99915	1.038	0.053856	0.84025	0.96161	0.97723	-0.03323	0.59161	0.83809	1.0567	0.079529	0.045484	0.11862	1.3184	0.39874	0.54448	0.99481	1.0935	0.12898	0.19453	0.91738	0.86837	-0.20363
1165.755	0.52	domics Pos	0.99858	0.99995	1.0116	0.016598	0.81994	0.99979	1.0335	0.047594	0.94341	0.99702	0.99466	-0.00772	0.85518	0.99996	1.0198	0.028262	0.82939	0.99701	0.98541	-0.0212	0.20199	0.99959	0.89264	-0.16385	0.038805	0.20439	1.2785	0.3545	0.67097	0.99984	0.96264	-0.05493	0.97404	0.99966	0.99654	-0.005
606.7913	0.56	domics Pos	0.58815	0.99995	0.9896	-0.01508	0.15638	0.99979	1.0526	0.074024	0.032924	0.38971	0.91839	-0.12282	0.69008	0.99996	0.97109	-0.04232	0.060297	0.76594	0.88082	-0.18308	0.051342	0.99959	0.89884	-0.15386	0.052444	0.23013	0.86082	-0.21621	0.28691	0.99984	1.072	0.10028	0.80415	0.99966	1.0647	0.090496
382.3557	4.45	domics Pos	0.091566	0.99995	1.215	0.28091	0.73059	0.99979	1.0225	0.032093	0.83568	0.99702	0.9657	-0.05035	0.97732	0.99996	1.0114	0.016404	0.54654	0.99701	1.0925	0.12769	0.28146	0.99959	1.1948	0.25673	0.040847	0.20898	1.3512	0.4342	0.28449	0.99984	1.2168	0.28304	0.36375	0.99966	1.1571	0.2105
312.3269	7.87	olomics Pc	0.33399	0.99964	1.1373	0.18566	0.093244	1	1.1881	0.24868	0.36573	0.99882	1.1274	0.17305	0.022482	0.99987	1.3824	0.4672	0.18283	0.95986	1.1594	0.21341	0.053657	0.99796	1.2965	0.37459	0.42864	0.99768	1.1125	0.15386	0.26652	0.9981	1.1871	0.24744	0.036785	0.99995	1.257	0.32993
1033.774	10.03	domics Pos	0.39547	0.99995	1.0563	0.079016	0.003803	0.99441	1.0981	0.13501	0.38263	0.93823	0.98043	-0.02851	0.22517	0.99996	1.1212	0.16504	0.39894	0.99701	0.96536	-0.05087	0.91315	0.99959	1.0078	0.011235	0.063348	0.24673	1.3252	0.40624	0.56856	0.99984	0.95819	-0.06162	0.12056	0.99966	1.0795	0.11034
722.5112	8.87	olomics Ne	0.36628	0.9951	0.91851	-0.12264	0.79406	0.99975	0.98768	-0.01788	0.021657	0.99731	0.7364	-0.44143	0.71703	0.99915	1.0321	0.045557	0.79412	0.94491	0.97502	-0.0365	0.071385	0.5223	1.3137	0.3936	0.04245	0.11482	1.3474	0.4302	0.44786	0.99481	1.1319	0.1787	0.12652	0.91738	1.2095	0.27447
431.9182	10.02	domics Pos	0.77872	0.99995	1.0248	0.035412	0.97269	0.99979	1.0022	0.003223	0.1265	0.72566	0.91637	-0.126	0.65443	0.99996	1.031	0.044012	0.73396	0.99701	0.98019	-0.02887	0.73646	0.99959	0.98249	-0.02548	0.02512	0.18884	1.2669	0.34129	0.84134	0.99984	1.0272	0.038723	0.64421	0.99966	1.0317	0.045039
[truncated: 598,115 more chars]
